# Genomic evidence for local adaptation in the ovoviviparous marine fish *Sebastiscus marmoratus* with a background of population homogeneity

**DOI:** 10.1038/s41598-017-01742-z

**Published:** 2017-05-08

**Authors:** Shengyong Xu, Na Song, Linlin Zhao, Shanshan Cai, Zhiqiang Han, Tianxiang Gao

**Affiliations:** 1 Institute of Evolution & Marine Biodiversity, Ocean University of China, 5th Yushan Road, Qingdao, 266003 P.R. China; 2 The First Institute of Oceanography, State Oceanic Administration, 6th Xianxialing Road, Qingdao, 266061 P.R. China; 3 Fishery College, Zhejiang Ocean University, 1st Haidanan Road, Zhoushan, 316022 P.R. China

**Keywords:** Evolutionary genetics, Genetic markers, Population genetics

## Abstract

Advances in next-generation sequencing techniques have allowed for the generation of genome-wide sequence data, to gain insight into the dynamics influencing genetic structure and the local adaptation of marine fish. Here, using genotyping-by-sequencing (GBS) technique, we identified 31,119 single nucleotide polymorphisms (SNPs) for *Sebastiscus marmoratus* in 59 individuals from three populations in Chinese coastal waters. Based on all SNPs, there was little evidence of genetic differentiation among populations. However, outlier tests revealed 329 SNPs putatively under divergent selection across populations. Structural and phylogenetic topology analyses based on the outliers showed clear genetic differentiation among populations. Gene Ontology (GO) annotation results revealed that most of these outliers are known or hypothesized to be involved in metabolic process. Together with previous work using mitochondrial cytochrome *b* sequences, the present results further suggest that the population structure is strongly influenced by locally adaptive pressure. Overall, adaptive evolution in a heterogeneous environment plays an important role in inducing genetic differentiation among local populations. This study increases understanding of the factors (including gene flow and local adaptation) promoting and constraining population genetic differentiation in marine organisms.

## Introduction

Inferring the degree of genetic differentiation among populations of marine fish species is key to successfully managing fishery resources, allowing the identification of management units, assignment of individuals to geographic regions, and detection of product mislabeling and fraud^1–3^. Many marine fish species are distributed widely across heterogeneous landscapes, and across these ranges, natural selection can promote genetic differentiation and local adaptation. However, the evolution of ecological divergence may be impeded if high rates of migration homogenize the gene pool among populations^4, 5^.

Owing to its distinct geographical features with a series of marginal seas^6^, its wide latitudinal range and its complex geological history^7^, the Northwestern Pacific (NWP) is a good region to investigate the patterns of genetic population structuring in marine fish species. Numerous studies on the phylogeographic patterns of marine fish based on neutral markers have been unable to detect genetic structures over such large geographic distances^8^ (e.g., *Scomberomorus niphonius* ^9^, *Larimichthys polyactis* ^10^, *Trachurus japonicas* ^11^), even for species with weak migration abilities (e.g., *Hexagrammos otakii* ^12^, *Sebastes schlegelii* ^13^). This failure is understandable for fish species with strong migration ability, because the gene flow is unimpeded by ecological homogeneity and a lack of clear dispersal barriers in marine habitats^14, 15^. However, owing to local adaptation and a lack of genetic exchange^16, 17^, this observation makes less intuitive sense for settled or short-distance-migration fish species. Local adaptation plays an important role in promoting genetic divergence between populations, whereby natural selection increases the frequency of traits that enhance the survival or reproductive success of individuals expressing them^18, 19^. Although the marine environment is relatively homogeneous compared with freshwater, numerous studies have revealed that differential thermal tolerance may result in ecological adaptation in marine species^5, 20^. Moreover, with the development of sequencing techniques, a growing number of studies using a high number of genome-wide polymorphic makers suggest that marine populations are not as connected as might be presumed^21, 22^. Hence, these techniques allow for inference of neutral population structure and also provide information on local adaptation or speciation events^19, 22, 23^. Consequently, local adaptation in marine fish species with little intraspecific population structure may commonly arise through ecologically different environments. The low intraspecific genetic structure reported in previous studies might be due to the limitation of markers and/or the short divergence time.

The advent and increasingly widespread use of high-throughput next-generation sequencing, such as the genotyping by sequencing (GBS) technique^24^, has facilitated the identification of such local adaptation through genome-wide scans. For instance, the GBS technique and related approaches have been used to investigate the genetic structure and local adaptation in marine fish species, such as sailfin molly *Poecilia latipinna* ^25^, ide *Leuciscus idus* ^26^, threespine stickleback *Gasterosteus aculeatus* ^27, 28^, small yellow croaker *Larimichthys polyactis* ^22^, and Atlantic mackerel *Scomber scombrus L* ^21^. Using such methods to investigate populations undergoing ecological divergence is particularly useful because these techniques provide good opportunities to detect genomic loci under divergence selection^5^, and such loci are expected to stand out with high *F*_st_ estimates against a background of low genetic divergence^29^. Such genome-wide scans can serve as a useful first pass (especially for non-model organisms) to identify the candidate loci for ecological divergence, which can then be subjected to further functional tests^5^. Given that the factors that promote or hinder local adaptation in marine organisms are still relatively unexplored, genomic studies that examine genetic structure and local adaptation are of great help.

The marbled rockfish, *Sebastiscus marmoratus*, is an excellent marine organism to lend insight into the balance between local adaptation and genetic exchange because it is influenced by several factors that alternately promote and hinder local adaptation. *S. marmoratus* has a wide latitudinal range: it inhabits littoral rocky bottoms along the northwest Pacific coast from Japan to the Philippines^30^. Across this wide distribution, individuals encounter highly heterogeneous environments, thus indicating that natural selection might favour local adaptation. In fact, previous genetic work investigating mitochondrial cytochrome *b* (Cyt *b*) sequences has also shown that the northern and southern populations of this species are locally adapted to thermal stress, with population-specific amino acid substitution (Xu *et al*. under review). In addition, *S. marmoratus* has strong site fidelity and usually appears within narrow home ranges in the daytime and hides in holes or crevices during the night^30^. Therefore, the latitudinal variation and ecological differentiation between the northern and southern rockfish populations used in this study may potentially drive local adaptation. However, population genetic studies of *S. marmoratus* using the mitochondrial control region sequences and the amplified fragment length polymorphism (AFLP) technique^31^ have detected no apparent genetic structure across its distribution area. Compared with the results based on the mitochondrial Cyt *b* sequences, the observed pattern of genetic structure may be derived from either a high level of gene flow in the present or a recent divergence with high levels of gene flow in the past.

This study’s objective was to gain insight into the balance between local adaptation and genetic exchange in geographically distinct *S. marmoratus* populations. We were particularly interested in characterizing the genomic variation across study sites with a background of genetic similarity and identifying genomic regions showing footprints of adaptive selection. Therefore, in this study, we sampled a total of 59* S. marmoratus* individuals from three populations from the northernmost to the southernmost populations across its distribution range in China and then performed genotyping by sequencing (GBS) to examine genome-wide population structure and test for genetic signatures of local adaptation. In particular, we sought to elucidate the extent of the effect of local adaptation on the genetic differentiation of this settled rocky fish with a background of population homogeneity.

## Results

The sequencing produced 68.37 G of raw data (~30 billion raw base pairs) for 59 individuals, with an average of 1.16 G (~515 million base pairs) per individual, which ranged from 0.63 G (~279 million base pairs) to 1.67 G (~743 million base pairs) (see Supplementary Table S1). The sequencing data have been deposited in the NCBI Sequence Read Archive (http://www.ncbi.nlm.nih.gov/Traces/sra) under accession number SRP095927. After quality filtering, a total of 68.37 G of clean data was retained, which presented a nearly 100% effective rate. Overall, our GBS results showed a high phred quality (Q20 ≥ 96.09%, Q30 ≥ 90.46%), a high enzyme catch ratio ranging from 97.1% to 99.5% and a stable GC content ranging from 40.14% to 41.89% (see Supplementary Table S1). After a Mock Reference was generated, quality-filtered reads of each individual were separately realigned to the reference for SNP calling. Alignments revealed a set of 525,170 putative SNPs prior to any quality filtering as implemented in the package SAMtools. Following filtering criteria, with a minimum coverage of 50% individuals, a maximum average read depth of 150 and only biallelic form present, a total of 522,496 SNPs were retained. After removal of loci and sites with quality score < 98 and quality value < 30, 432,387 SNPs were retained. After exclusion of SNPs with a minor allele frequency < 0.1, 124,394 SNPs were retained. After a Hardy-Weinberg equilibrium (HWE) test with a *P* value < 0.05, 31,120 SNPs were retained. After exclusion of putatively mitochondrial SNPs, a total of 31,119 SNPs were retained for downstream analyses across all 59 individuals. Summary statistics for the counts of putative SNP loci and final counts of candidate SNPs after different filtering steps are shown in Table 1.

**Table 1:** Summary statistics for counts of putative SNP loci and final counts of candidate SNPs after different filtering steps.

Filtering step	Count
Total SNPs prior filtering	525,170
Coverage of 50% individuals; average read depth < 150; biallelic loci	522,496
Quality score ≥ 98; quality value ≥ 30	432,387
Minor allele frequency ≥ 0.1	124,394
Hardy-Weinberg equilibrium test (*P* < 0.05)	31,120
Removing putatively mitochondrial SNPs	31,119
Lositan outlier detection in FA-RS comparison (*P* > 0.995)	1005
Lositan outlier detection in FA-ZS comparison (*P* > 0.995)	833
Lositan outlier detection in RS-ZS comparison (*P* > 0.995)	773
Outlier loci detected in at least one dataset	2274
Outlier loci detected in at least two datasets	329
Outlier loci detected in all three datasets	8

### Population genetics statistics

Across all populations, the nucleotide diversity (*π*) for all SNPs was 0.374 (standard error (SE) = 0.118) and ranged from 0.364 to 0.384 within each population (Table 2). The observed heterozygosity (*H*_*o*_) and expected heterozygosity (*H*_*e*_) across all populations were 0.391 (SE = 0.173) and 0.374 (SE = 0.132) respectively (Table 2). Among the three populations the highest *π* and *H*_*e*_ values were detected in the Zhoushan population. The pairwise *F*_st_ values ranged from 0.0037 for the Rushan-Zhoushan comparison to 0.0094 for the Fangchenggang-Rushan comparison, whereas the value for the Fangchenggang-Zhoushan comparison was 0.0088 (Table 2).

**Table 2:** Estimates of basic population genetic parameters in each population using all 31,119 SNPs.

Pop	π	*H*_*o*_	*H*_*e*_		*F*_st_	
RS	0.369 ± 0.179	0.408 ± 0.219	0.374 ± 0.144	*		
ZS	0.384 ± 0.186	0.376 ± 0.171	0.384 ± 0.131	0.0037	*	
FA	0.364 ± 0.176	0.399 ± 0.210	0.368 ± 0.150	0.0094	0.0088	*
All	0.374 ± 0.118	0.391 ± 0.173	0.374 ± 0.132	—	—	—

**Figure 1:**
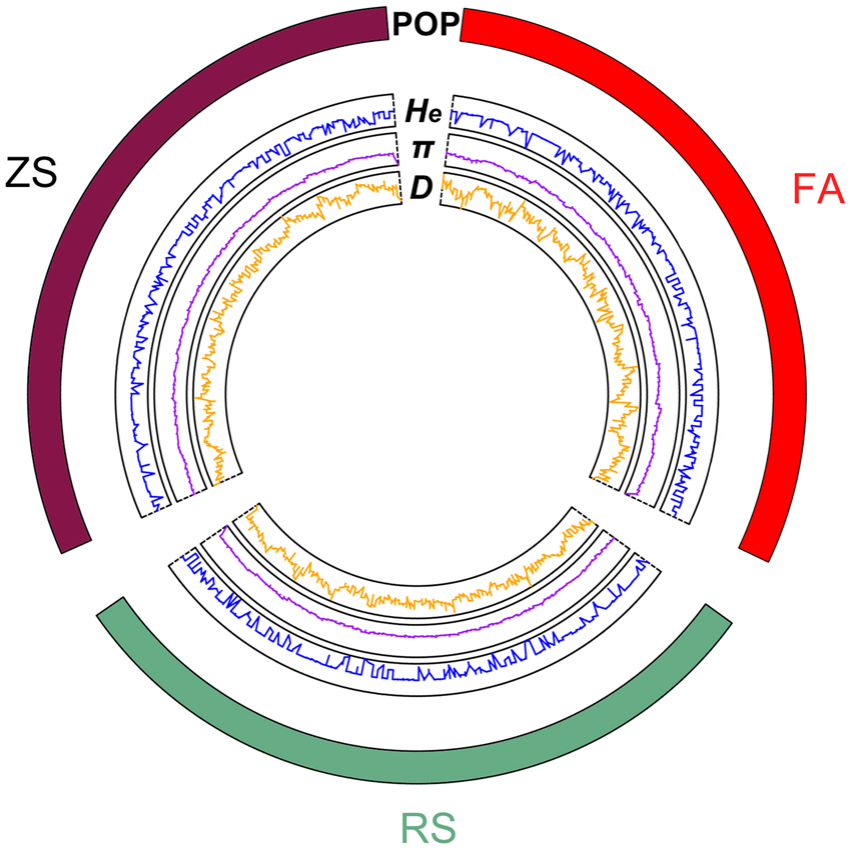
Genomewide distribution of genetic variation and differentiation across three populations using 31,119 SNPs. Populations are represented as dark green (RS), purple (ZS) and red (FA) blocks respectively in a circle. The expected heterozygosity *H*_*e*_ (blue line), nucleotide diversity *π* (purple line) and Tajima’s *D* value (yellow line) are plotted as functions of genomic position with a non-overlapping 100-bp sliding window.

Across all populations, the genome-wide distribution patterns for *H*_*e*_, *π* and Tajima’s *D* were very similar (Fig. 1, see Supplementary Table S2). The range of *H*_*e*_ and *π* values varied slightly across all populations and also within each population, whereas the range of *D* values varied widely within each population, thus suggesting natural selection (Fig. 1, see Supplementary Table S2). The observation of most sites having positive *D* values within each population suggested balancing selection.

### Isolation by distance

For 31,119 putatively neutral SNPs, a Mantel test for a significant relationship between the genetic and geographic distances between sampling sites revealed significant evidence for matrix correlation, with a Z score of −20.345, an r value of 0.986 and a one-sided *P* value of 1.000. Reduced major axis regression estimated an *R* ^2^ of 0.972 for the regression line of genetic vs. geographic distance (see Supplementary Fig. S1).

### Outlier analyses and gene annotation

A total of 2274 SNPs were identified by the Lositan software as putative candidate outliers under selection (*P* > 0.995), of which 1005, 833 and 773 SNPs were identified in the Fangchenggang-Rushan comparison, Fangchenggang-Zhoushan comparison and Zhoushan-Rushan comparison, respectively (Table 1, see Supplementary Fig. S2). Among these SNPs, 329 loci detected in at least two datasets were considered as outlier SNPs for further population genetics and gene annotation analyses. Outlier SNPs were encompassed by a total of 294 contigs.

**Figure 2:**
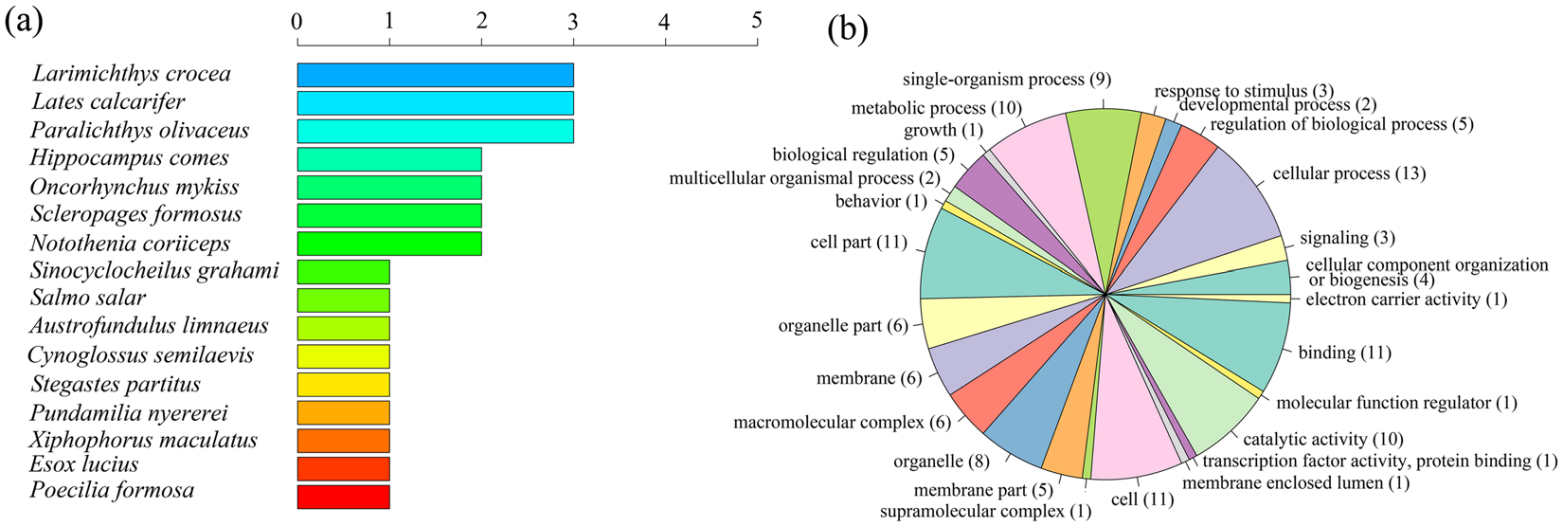
(**a**) Frequency and distribution of matched species of 26 significant BLASTX hits against various bony fish genomes; (**b**) Putative functional categorization and distribution of 21 significant hits according to Blast2GO annotation.

The BLASTX analysis of the contigs containing the outlier SNPs against various bony fish genomes resulted in significant hits (E-value 1E-3) for 16 fish species (Fig. 2a). The BLASTX similarity results showed that 29 of the 294 contigs containing outlier SNPs corresponded to known proteins in the nr database (E-value 1E-3), of which 21 contigs were functionally annotated. The functional categorization of the annotated sequences involved in binding, catalytic activity, cellular process, metabolic process, and single-organism process (Fig. 2b). the classification of biological and molecular functionalities for these hits is listed in Supplementary Table S3. KEGG pathway analysis yielded hits for 6 of the 21 annotated contigs. These hits participated in enzymatic synthesis and belonged to 7 pathways involved in thiamine metabolism, purine metabolism, fatty acid elongation, etc. (see Supplementary Table S4).

### Population genomic analyses

To test the possible effects of local adaptation on inferring population genetic structure, the population genetic structure was analyzed by using two different SNP data sets: all putative neutral SNPs (31,119 SNPs) and candidate outlier SNPs (329 SNPs). When all SNPs were used to construct the NeighborNet topology, three main clusters generally defined by geographic localities were recovered (see Supplementary Fig. S3a). However, the phylogenetic topology showed a shallow structure, and some of the individuals (RS1, FA29, ZS32, ZS35, and ZS37) were grouped incorrectly into other geographic clusters (see Supplementary Fig. S3a). Clusters were better resolved in the topology obtained with outlier SNPs (see Supplementary Fig. S3b) and showed a deeper structure. Except for FA29, which showed a close relationship to individuals in the Rushan population, each individual was grouped into its geographic cluster. In addition, compared with the Rushan and Fangchenggang populations, the clusters representing the Zhoushan population showed a more relaxed structure.

**Figure 3:**
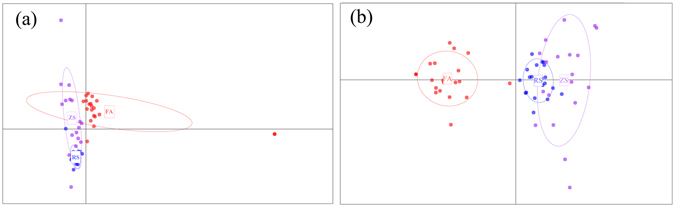
Principal component analysis (PCA) plot of three *S. marmoratus* populations based on all 31,119 SNPs (**a**) and 329 outlier SNPs (**b**).

The PCA recovered the same clusters achieved by the phylogenetic topology based on all SNPs, showing three clusters, with two samples from the Fangchenggang and Zhoushan populations being more distant than other samples with their respective clusters (Fig. 3a). In addition, the results revealed that the Rushan population was closely related to the Zhoushan population. For outlier SNPs, the PCA revealed a hierarchical relationship among the three populations (Fig. 3b). Individuals in the Rushan and Zhoushan populations pooled as one group, whereas those in the Fangchenggang population pooled as another group, showing a closer relationship between the Rushan and Zhoushan population.

**Figure 4:**
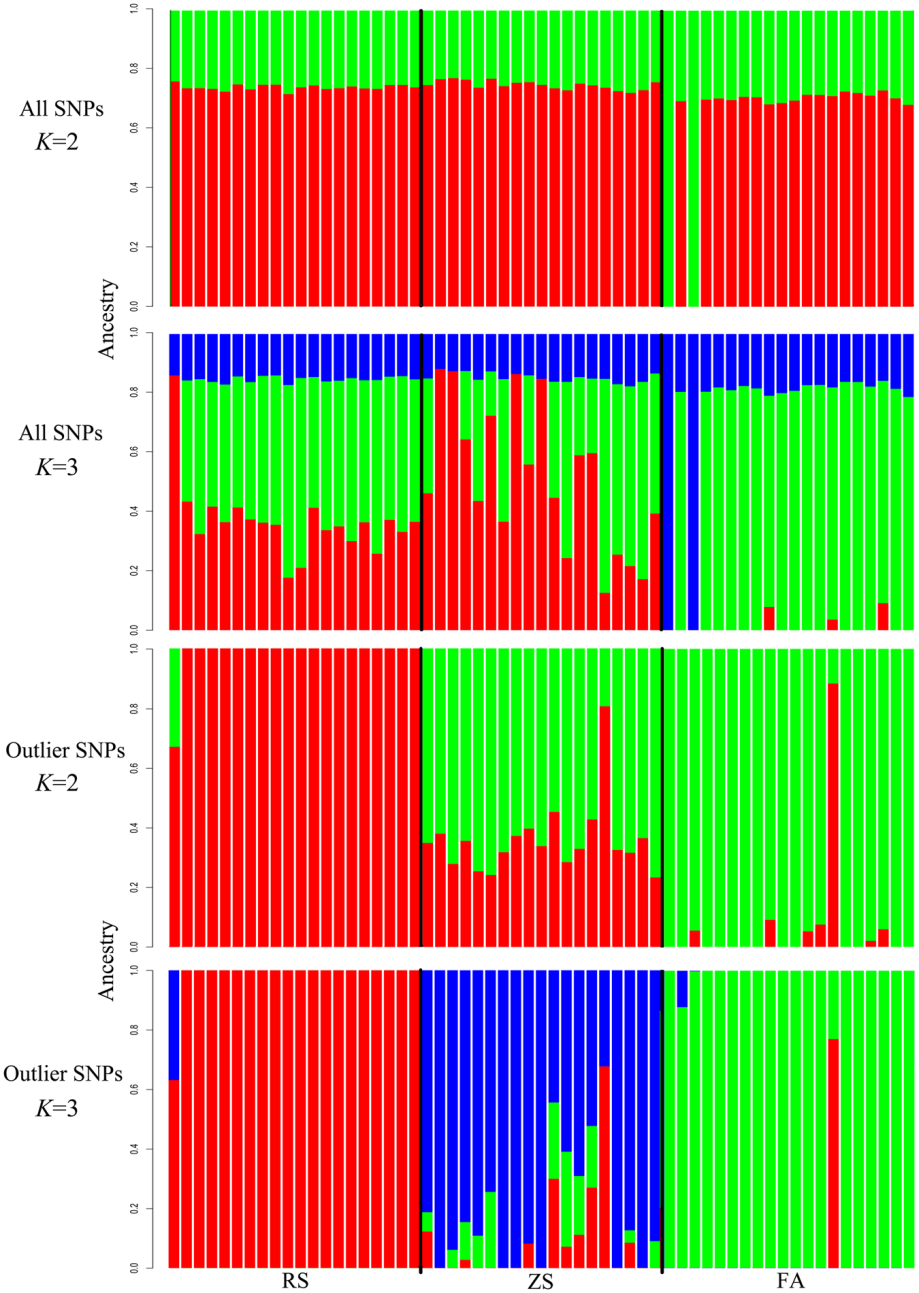
Population Admixture analysis of three *S. marmoratus* populations using all 31,119 SNPs and 329 outlier SNPs. Each bar represents an individual and each color is inferred membership in each of the *K* (2 or 3) potential ancestral populations.

The results of the admixture analyses on all SNPs revealed no consistent evidence for clustering (Fig. 4). When K = 2, the admixture structure showed population homogeneity in all three populations. However, when K = 3, the genetic structure was recovered, showing that the Fangchenggang population formed a separate cluster. Conversely, when outlier SNPs were considered, evident clusters were recovered both for K = 2 and K = 3, indicating that the Rushan and Fangchenggang populations were completely separate. Moreover, gene flow was revealed between the Zhoushan population and the other two populations in the admixture clustering. However, when K = 3, three clusters separated by geographic populations were recovered (Fig. 4).

When all SNPs were used, overall genetic structure inferred by a hierarchical AMOVA resulted in a significant *F*_st_ value of 0.0074 (*P* = 0.000). However, only 0.74% of the variation arose among groups and among populations within groups, and the remaining 99.3% arose within populations (Table 3). The hierarchical AMOVA for the outlier SNPs resulted in a significant *F*_st_ value of 0.1382 (*P* = 0.000), indicating a high level of genetic structure. Additionally, a relatively large proportion of variation (13.81%) occurred among groups and among populations within groups compared with only 0.74% for the all SNPs (Table 3).

**Table 3:** AMOVA results showing the percentage of variation for all 31,119 SNPs and 329 outlier SNPs among groups (population RS&ZS or population FA), among populations within groups, and within populations.

Data set	*F*_st_ value	Percentage of variation		
		Among groups	Among populations within groups	Within populations
All SNPs	0.0074 (*P* = 0.000)	0.43	0.31	99.26
Outlier SNPs	0.1382 (*P* = 0.000)	1.21	12.61	86.19

## Discussion

In the present study, a cost-effective GBS technique was utilized to examine genome-wide population structure and to gain insight into the dynamics influencing regional differentiation among *S. marmoratus* populations. To our knowledge, this might be the first reported large-scale generation of novel SNPs for Scorpaenidae species. Most previous population genetic studies of *S. marmoratus* were based on a handful of microsatellites, mtDNA loci and AFLP markers, which obtained insufficient and inconsistent results^31, 32^. Here, we identified a total of 31,119 SNPs for population genomic analyses, of which 329 outlier SNPs were identified as candidates for selection. Further, we highlighted the potential advantages of the genome-wide SNPs for inferring population divergence and detecting candidate adaptive markers of *S. marmoratus*.

When all SNPs were used, shallow population structure was recovered by multiple analyses, and evident population differentiation was detected on the basis of outlier SNPs. The phylogenetic information from highly differentiated SNPs appeared to be covered by neutral SNPs. The PCA and admixture plotting showed a lack of genetic structure across the three populations (Fig. 3a; Fig. 4); hierarchical AMOVA analysis and splits topology also revealed that the genetic differentiation arose highly within populations (Table 3, see Supplementary Fig. S3a). This background shallow genetic structure might be due to gene flow, lack of drift in large population size or both^5^.

**Figure 5:**
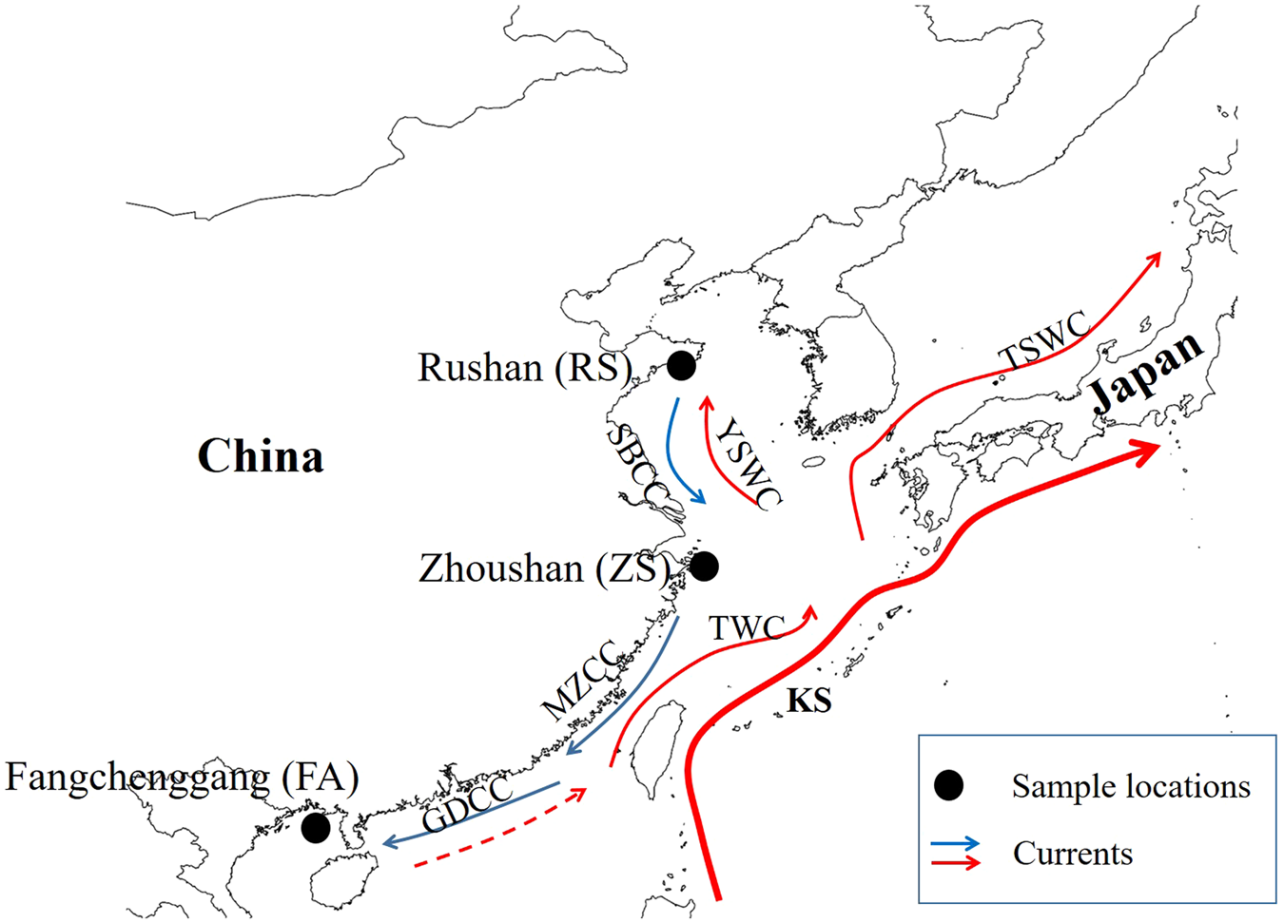
Schematic map showing sample locations of *S. marmoratus* and contemporary main currents of the Northwestern Pacific in winter. KS, Kuroshio current, TWC, Taiwan warm current, TSWC, Tsushima warm current, YSWC, Yellow Sea warm current, SBCC, Subei coastal current, MZCC, Minzhe coastal current, GDCC, Guangdong coastal current. The map was generated using ArcGIS 10.2, made with Natural Earth. The coastline data are available on the web at http://www.naturalearthdata.com/downloads/. The main currents were following the description in Liu^46^.

*S. marmoratus* has strong site fidelity, and only females migrate to deeper sites for parturition^30^. For instance, previous mark-release-recapture studies have shown that the longest distance between the released and captured sites of released drifters was 8 km, and the majority were less than 1 km^33^, thus showing the strong site fidelity and sedentariness of *S. marmoratus*. However, *S. marmoratus* has a pelagic larval duration of nearly 80 days^33^. Under the influence of currents, larval dispersal may be up to 200 km for fish^34^. In addition, night parturition of the female *S. marmoratus* would be advantageous for effective dispersal of larvae, as well as parturition offshore where currents are strong^30^. Night parturition would be advantageous in avoiding predators of larvae because most of the predators are inactive at night in the study area. In addition, during parturition, *S. marmoratus* appears to release eggs offshore where water currents are strong^30^. Larval dispersal would be promoted by the influence of currents (such as the coastal currents shown in Fig. 5). The relatively long pelagic duration and advantageous parturient strategies of *S. marmoratus* might promote larval dispersal across regions. Overall, the site fidelity of *S. marmoratus* after pelagic duration might hinder genetic exchange across regions, whereas the long pelagic duration and currents might promote larval settlement far from natal grounds.

Pelagic larvae might contribute to genetic exchange. In this sense, a hypothesis of stepping stone migration may occur over a few generations^35^, with no direct exchange of migrants occurring across long-distance regions, but instead with genes being exchanged across regions through intermediate sites. Stepping stone migration commonly leads to isolation by distance (IBD)^35^. The present data also supported this stepping stone hypothesis with robust evidence of patterns of IBD (see Supplementary Fig. S1). The stepping stone hypothesis might be a convincing interpretation of genetic similarity across *S. marmoratus* populations.

Large population sizes might be another interpretation of such genetic similarity. Previous studies estimating the population size of *S. marmoratus* have rarely been reported. However, given that *S. marmoratus* is an ovoviviparous species and has efficient parturient strategies^30^, we could speculate that the survival rate is relatively high and that the population size of *S. marmoratus* might be large enough to maintain sufficient mutations. As population size increases, the production of mutations may be exactly balanced by the decreased probability that each mutation will be fixed through genetic drift^36^. Thus, genetic homogeneity will be met. Alternatively, recent population shifts due to glacial cycles might obscure genetic differentiation across regions. During the last glacial maximum (LGM), under the most severe environmental conditions in glacial periods, *S. marmoratus* might have become extinct over large parts of its distribution and have survived in glacial refugium. When favorable conditions returned, population expansions from glacial refugium would be expected in *S. marmoratus*, and genetic similarity would be expected in the recolonized regions^37^ given the relatively young postglacial ecosystems (< 10,000 years)^38^. In the present study, the Zhoushan population showed higher nucleotide diversity and expected heterozygosity than other populations (Table 2). In addition, PCA and topology analyses detected a relaxed pattern of genetic structure in the Zhoushan population (Fig. 3, see Supplementary Fig. S3), which is likely to share high levels of connectivity to other populations^39^. All these results indicated that the glacial refugium of *S. marmoratus* might be located in the basin of East China Sea and the genetic diversity of the ancestral population was expected to be higher than that of the derived population. A higher number of samples from more population localities is needed to explore the demographic history of *S. marmoratus* and to definitively determine the cause of genetic similarity across populations.

Recent studies have shown that high-throughput sequencing technology provides a novel approach for investigating local adaptation in natural populations of marine fishes^15, 22, 40^. In contrast to previous work that found a lack of genetic structure in populations of *S. marmoratus*, on the basis of mitochondrial control region sequences and AFLP markers^31^, the most important finding of this study was the detection of the high degree of population differentiation among three sampling sites by using 329 candidate adaptive SNPs (Figs 3b and 5, see Supplementary Fig. S3b). Given the wide distribution and ecological characters of this species (such as site fidelity), *S. marmoratus* might be susceptible to a heterogeneous local environment. Likewise, despite the strong migration ability to promote gene exchange, outlier SNPs have revealed significant regional differentiation between northern and southern populations in recent genomic studies of the spotted seabass (*Lateolabrax maculatus*)^41^ and the small yellow croaker^22^. In this sense, the high degree of regional differentiation between northern and southern Chinese populations might be common in marine fish species, owing to local adaptation to heterogeneous environment. For instance, with the average annual sea surface temperature ranging from 10.9 °C in the Bohai Sea to 26.5 °C in the South China Sea (data provided by the National Oceanic and Atmospheric Administration), these differences in the thermal environment may result in divergent selection on specific genes^42^. More work is needed to verify the hypothesis of regional differentiation between the northern and southern Chinese populations.

Lositan can create false positives when performing outlier identification process^43^. For further analyses performed well with few false positives, three pairwise datasets were analyzed, and the results were contrasted with a parallel model. Among the three datasets, the number of candidate outliers was the highest in the Fangchenggang-Rushan comparison (1005/2274) whereas it was the lowest in the Rushan-Zhoushan comparison (773/2274). The results were consistent with the pattern of PCA plotting (Fig. 3b), to some extent showing a higher genetic divergence between the Fangchenggang and Rushan populations. While many of the loci were corroborated with at least two approaches, these loci were chosen as outlier SNPs for further analyses, with few false positives. As a result, a total of 329 SNPs encompassed in 294 contigs were chosen as outliers.

The Blast2GO analysis of the contigs containing outliers revealed that 21 out of the 294 contigs were located in known functional genes or genomic regions. In addition, eight of the contigs containing SNPs detected in all three datasets were not annotated. The inefficiency of GO annotation could impede gaining insight into the dynamics of local adaptation. The GO annotated contigs in this study were mainly involved in metabolism, transmembrane transport, cellular processes and catalytic activity (see Supplementary Table S3). Six KEGG pathways involved in metabolic processes were also detected by mapping annotated sequences with corresponding ECs to KEGG pathway database (see Supplementary Table S4). These consistent results suggested that these candidate genes may play important roles in local adaptation. Individuals that inhabit heterogeneous environments (especially thermal differences in the marine environment) along their widely geographical distribution experience spatially divergent selective pressure, which normally results in local adaptation to ecological traits^44^. Considering the environmental similarity of marine waters and the temperature gradients between northern and southern Chinese populations, we hypothesize that the genes annotated by Blast2GO analysis are under divergent selection in response to the differences in thermal stress they encounter.

The analytical approach used herein provided a very conservative estimate of *F*_st_ based on all 31,119 SNPs. However, the considerable level of divergence, as compared with earlier studies based on limited numbers of putatively neutral markers, accords with the contention that at least part of the observed divergence is likely to have been caused by adaptive selection^15^. Low levels of divergence have been examined in previous studies^31^, showing 0.55% and 7.55% of variance among populations when using mitochondrial control region fragments and AFLP techniques, respectively. Here, we estimated that approximately 9.12% of the variance arose among populations based on 329 candidate selective SNPs, whereas only 0.74% of the variance arose among populations based on all SNPs. Approximately 1.1% (329/31,119) of the SNPs represented variation that might be linked to adaptive selection, showing nearly 19 times the variation than when all SNPs were used. Similar results were shown by Wang *et al*.^41^, in which 7.3% (3,122/42,733) of the SNPs demonstrated substantial population differentiation in the spotted seabass. Likewise, 6% (538/13,272) of the SNPs in the study of the small yellow croaker were found to show significant divergence^22^. Much like *S. marmoratus*, earlier studies of small yellow croaker using mitochondrial fragments also failed to find similar levels of differentiation^10^. Hence, the higher number of genetic markers (31,119 SNPs) in our study could be a possible explanation for the lack of correspondence with earlier studies on *S. marmoratus*, in which only a handful of genetic markers were used. A higher number of SNPs may be used as a window to gain relatively comprehensive insights into the dynamics influencing genetic differentiation among *S. marmoratus* populations. In summary, the estimated genetic variation should not be interpreted too literally as a direct measure of gene flow^15^, because the degree of divergence resulting from local adaptation might be underestimated among *S. marmoratus* populations.

From the viewpoint of fishery management, the results in our study revealed a need for further studies and possible refinements in fishery management. Owing to the genetic homogeneity revealed by a handful of genetic markers, previous population studies suggested that *S. marmoratus* populations in Chinese coastal waters should be considered as one fishery management unit^31^. Given the high level of genetic divergence revealed through selective markers within the same management unit, there is a clear mismatch between fishery management units and genetic population structure, which may lead to unsound management of local populations^15, 45^. Similar suggestions were made for the fishery managements of marine fish with genetic backgrounds of population homogeneity (e.g., *L. polyactis* ^10^, *T. japonicas* ^11^, *S. schlegelii* ^13^) across Chinese coastal waters. Much like *S. marmoratus*, a recent study of small yellow croaker using genome-wide SNPs has also found a high level of population divergence within one management unit^22^. Large numbers of genome-wide markers is useful not only for determining traditional genetic structure resulting from gene flow, but also for identifying the hidden genetic structure in populations of marine fish species caused by local adaptation. As a result, given the robust patterns of genetic differentiation, sound management can be implemented for scientific and sustainable development.

## Methods

### Ethics Statement

Ethical approval was not required for this study because no endangered animals were involved. All handling of *S. marmoratus* specimens was conducted in strict accordance with Animal Care Quality Assurance in China.

### Sample collection

The samples were collected in the winter of 2015 from three separate sites: one from the coast of Rushan (RS, 36°43′N, 121°39′E, 20 individuals), Yellow Sea; one from Zhoushan (ZS, 30°03′N, 122°21′E, 19 individuals), East China Sea; and one from Fangchenggang (FA, 21°30′N, 108°21′E, 20 individuals), South China Sea (Fig. 5). The contemporary main currents of the Northwestern Pacific in winter were following the description in Liu^46^. The samples were collected by trawl net or hook fishing in offshore waters at the spawning season, thus ensuring that the samples collected were representative of the local populations. Muscle tissues were preserved in 95% ethanol for DNA isolation by using the standard phenol-chloroform extraction protocol.

### DNA extraction, GBS library preparation and sequencing

DNA was treated with RNase A to produce pure, RNA-free DNA. The GBS libraries were constructed by following a protocol adapted from Elshire *et al*.^24^. Briefly, the DNA was digested with both high-fidelity *NlaIII* and *MseI* restriction enzymes. Three libraries were created by uniquely barcoding each of the individuals from the respective site and then pooling these individually barcoded samples. The barcodes used were six nucleotides in length. The libraries were pooled for multiplexed PCRs, and then the PCR products were purified. The PCR products were sequenced in one lane per library of an Illumina HiSeq2000 platform, using 150-bp paired-end reads, at Novogene in Beijing.

### SNP calling pipeline and quality filtering

The generated GBS-tags were analyzed using the GBS-SNP-CROP pipeline for SNP calling^47^. Paired-end reads were parsed, trimmed and demultiplexed following the three Perl scripts in stage 1, using the bioinformatics tool Trimmomatic 0.36^48^ with default parameters. The individual with the largest numbers of reads was processed to construct a GBS-specific, reduced-representation reference (Mock Reference) to enable reads mapping and facilitate SNP calling in stage 2, using the PEAR 0.9.8^49^ and USEARCH 9.0^50^ software packages with default parameters. The “MockRef_Genome.fasta” file was used for read alignment and the “MockRef_Clusters.fasta” file was used for the downstream gene annotation. After a reliable reference was generated, all quality filtered reads were then sorted according to their unique index sequences and aligned to the reference assembly assembled from paired-end reads by using the bwa-mem algorithm in BWA 0.7.12^51^ with default parameters. After the alignment, SNP calling was performed using a conservative Bayesian approach, as implemented in the package SAMtools 1.3.1^52^. SNP filtering was produced using VCFtools^53^ with the following parameters: (1) the SNP was called in at least 50% of individuals (--max-missing 0.5), (2) the minor allele frequency (MAF) was > 10% (–--maf 0.1), (3) the average read depth was < 150 (–--max-meanDP 150), (4) only two alleles were present (–--min-alleles 2 –--max-alleles 2), (5) loci with quality score < 98 were eliminated (–--minGQ 98), (6) only sites with quality value > 30 were included (–--minQ 30), (7) sites that contained an indel were excluded (–--remove-indels), (8) sites that failed the Hardy-Weinberg equilibrium (HWE) test at *P* < 0.05 were excluded (–--hwe 0.05). Subsequently, contigs consisting of putative SNPs were aligned with the *S. marmoratus* mitochondrial genome (NC_013812) by using Geneious 10.0.6^54^ software, and SNPs contained in aligned contigs were excluded. All datasets were reformatted using PGDSpider 2.0.5.2^55^.

### Population genetics statistics

To characterize the pattern of population differentiation, the program Arlequin 3.5^56^ was used to estimate nucleotide diversity (*π*), observed heterozygosity (*H*_*o*_) and expected heterozygosity (*H*_*e*_) of each population. The fixation index (*F*_st_) for each pairwise comparison was also estimated using Arlequin. In addition, to characterize the genome-wide pattern of genetic variation, the TASSEL v5.2.31^57^ program was used to estimate nucleotide diversity (*π*) and Tajima’s *D* value at each SNP for all individuals, and the Arlequin 3.5 program was used to estimate expected heterozygosity (*H*_*e*_) at each SNP site. The patterns of genomic variation of each population based on all SNPs as reflected in *H*_*e*_, *π* and Tajima’s *D* were visualized using the R package RCircos^58^.

### Isolation by distance

Geographic (shortest straight line) distances between each site were calculated using Baidu Map (http://map.baidu.com). These measures of genetic and geographic distance between each of the three sites were used to test for isolation by distance as implemented in IBDWS (http://ibdws.sdsu.edu/~ibdws/distances.html)^59, 60^. According to Slatkin’s recommendation^61^, the log of both genetic and geographic distance was used as inputs for the Mantel test for matrix correlation between the genetic and geographic distance. Reduced major axis regression using 1000 randomizations was also performed to calculate the intercept and slope of the regression line of genetic distance vs. geographic distance.

### Outlier analyses

The program Lositan^62^ was utilized to detect loci under selection based on the neutral distribution of *F*_st_ values for all loci in relation to *H*_*e*_ (expected heterozygosity). Any locus with *F*_st_ higher or lower than the neutral distribution was considered a candidate that might be under selective pressure^63^. To gain insight into the dynamics influencing adaptive divergence, outlier analyses were implemented on the basis of three pairwise comparisons: (i) individuals in population Rushan and Fangchenggang, (ii) individuals in population Rushan and Zhoushan, and (iii) individuals in population Zhoushan and Fangchenggang. Lositan was first run using all loci under attempted neutral mean *F*_st_, 50,000 simulations, 99.5% confidence interval, infinite alleles mutation model, and false discovery rate of 0.1%, following the procedure described in Antao *et al*.^62^, to decrease the bias in the estimation of the mean neutral *F*_st_ by eliminating extreme loci from the estimation. After the first run, all loci that were outside the confidence interval were removed, and the mean neutral *F*_st_ was recalculated. Only the supposed neutral loci were used in this run under the same parameters as above. The third run used all loci and the newly calculated neutral *F*_st_, with all other parameters maintained. Loci recovered as outliers in the last run were inferred to be under selection. To perform analyses with few false positives, outliers detected on the basis of at least two datasets were chosen for further analyses. The results were shown through a Venn diagram constructed in Venny 2.1.0 (http://bioinfogp.cnb.csic.es/tools/venny/index.html).

### Population genomic analyses

We constructed a splits tree with NeighborNet distance transformation^64^ and equal angle splits transformation^65^ using the SplitsTree v 4.14.4 program^66^ on the basis of (i) all loci and (ii) loci identified as being under selection with the program Lositan. This approach was applied to compare the power of discrimination of individuals within populations of likely non-neutral (or adaptive) markers and putatively neutral markers^67, 68^. To assess the robustness of the topology of the tree, 1,000 bootstrap replicates were performed using the SplitsTree program.

Principal component analyses (PCA) based on the two datasets were implemented using the R package *adegenet* ^69^ to determine whether sampled individuals reflected a history of differentiated populations by outputting individual coordinates along axes of genetic variation within a statistical framework that corresponded to the first two principal components.

To estimate the individual admixture assuming different numbers of clusters, the population structure was investigated by utilizing the program admixture 1.3.0^70^ and analyzing the two datasets, with a maximum-likelihood method. The best value of the coancestry cluster (*K*) was estimated using a corss-validation procedure in the admixture software. The best value of the coancestry cluster exhibited the lowest cross-validation error (CVE) compared with the other cluster values. The CVE values of *K* 1–3 were estimated, and the lowest CVE value was represented by the *K* = 1 (for all SNPs) and *K* = 3 (for outlier SNPs). We increased the coancestry clusters spanning from 1 to 3 and ran the analysis with 10,000 iterations.

We also conducted an analysis of molecular variation (AMOVA) in Arlequin 3.5 to examine the variation within and among groups of genetically similar populations by using two datasets. The hierarchy for this analysis was chosen on the basis of the clustering from the PCA analysis: (i) populations Rushan and Zhoushan, and (ii) population Fangchenggang. The significance of the covariance components associated with the different possible levels of genetic structure was tested using 1000 permutations.

### Gene prediction and functional annotation

The contigs containing outlier SNPs identified using the program Lositan were used as queries in nucleotide searches with BLASTX (E-value 1E-3) against the nr database at the National Center for Biotechnology Information (NCBI) website. In case of multiple hits, the best match was chosen. Then, the functional annotation of these genes were obtained using the Blast2GO software^71^. This software conducts BLAST similarity searches and maps Gene Ontology (GO) for homologous sequences. Blast2GO produces GO annotations as well as corresponding enzyme commission numbers (EC) for sequences with E-value < 1E-6, annotation cut-off > 55 and a GO weight > 5. Subsequently, the annotated sequences with corresponding ECs obtained from Blast2GO were mapped to the Kyoto Encyclopedia of Genes and Genomes (KEGG) metabolic pathway database.

## Electronic supplementary material

Supplementary Information except Table S2

Supplementary Information Table S2
