# Supplementary material for: Genomic evidence for local adaptation in the ovoviviparous marine fish Sebastiscus marmoratus with a background of population homogeneity
Source: Sci Rep. 2017 May 8;7:1562. doi: 10.1038/s41598-017-01742-z (PMC5431535; doi:10.1038/s41598-017-01742-z)

Supplementary information

Genomic evidence for local adaptation in the ovoviviparous marine fish *Sebastiscus marmoratus* with a background of population homogeneity

Shengyong Xu, Na Song, Linlin Zhao, Shanshan Cai, Zhiqiang Han, Tianxiang Gao

Contents:

Supplementary Table S1

Supplementary Table S3

Supplementary Table S4

Supplementary Figure S1

Supplementary Figure S2

Supplementary Figure S3

Supplementary Table S1. Statistics describing the distribution of different properties of each sequenced individual

ID	Raw data /Gb	Raw bases	Clean data/ Gb	Clean bases	Error rate	Q20	Q30	GC content	Enzyme catch ratio (%)
FA1	0.63	279,297,216	0.63	279,297,216	0.03	96.5	91.45	40.81	98.8
FA2	1.15	512,185,536	1.15	512,185,536	0.03	96.74	92.03	40.63	98.8
FA3	1.20	536,178,528	1.20	536,178,240	0.03	96.64	91.75	40.99	98.5
FA5	1.10	488,135,808	1.10	488,135,520	0.03	96.09	90.46	41.28	98.6
FA6	1.04	464,407,200	1.04	464,406,912	0.03	96.78	92.13	41.45	98.7
FA7	1.31	584,929,152	1.31	584,929,152	0.03	96.51	91.52	41.08	98.5
FA8	1.35	602,234,496	1.35	602,234,496	0.03	96.72	91.95	41.48	98.7
FA9	1.34	596,807,136	1.34	596,806,560	0.03	96.7	91.92	41.30	98.1
FA10	1.14	509,468,256	1.14	509,467,968	0.03	96.37	91.14	40.48	98.5
FA11	0.88	390,631,680	0.88	390,631,680	0.03	96.12	90.65	40.83	98.3
FA12	1.41	626,775,840	1.41	626,775,840	0.03	96.41	91.14	41.12	98.1
FA13	1.23	547,136,352	1.23	547,135,488	0.03	96.33	91.00	41.30	98.6
FA15	1.27	565,199,712	1.27	565,199,424	0.03	96.75	92.09	41.37	98.8
FA16	1.40	621,485,568	1.40	621,485,568	0.03	96.65	91.81	41.31	98.8
FA17	1.43	638,371,296	1.43	638,371,296	0.03	96.6	91.71	41.23	98.8
FA18	1.41	628,187,616	1.41	628,187,616	0.03	96.72	92.03	41.49	98.7
FA19	1.45	644,270,688	1.45	644,270,688	0.03	96.72	91.98	41.45	98.8
FA21	1.47	652,987,296	1.47	652,987,296	0.03	96.13	90.47	41.44	98.7
FA22	1.28	570,334,176	1.28	570,334,176	0.03	96.34	91.03	41.28	98.3
FA29	1.16	514,808,640	1.16	514,794,240	0.02	97.12	93.16	41.12	99.2
RS1	1.38	613,854,432	1.38	613,852,992	0.01	97.91	95.15	41.36	98.6
RS2	0.84	372,028,896	0.84	372,016,512	0.02	97.28	93.46	41.21	99.4
RS6	0.73	325,704,384	0.73	325,694,016	0.02	97.28	93.42	41.41	99.4
RS8	0.75	331,696,512	0.75	331,687,584	0.02	96.9	92.53	41.33	99.4
RS9	0.83	366,242,976	0.83	366,230,592	0.01	97.42	93.81	41.57	99.5
RS10	0.77	341,650,080	0.77	341,638,848	0.02	97.25	93.38	41.50	99.3
RS11	0.83	369,481,248	0.83	369,469,728	0.02	97.32	93.56	41.5	99.4
RS12	0.67	295,439,904	0.67	295,433,280	0.02	97.39	93.69	41.5	99.1
RS13	0.81	361,205,568	0.81	361,194,912	0.02	97.07	92.97	40.77	99.3
RS14	0.75	333,597,024	0.75	333,586,368	0.02	96.75	92.37	40.43	99.2
RS15	0.97	428,501,376	0.97	428,491,872	0.02	96.93	92.62	41.28	99.2
RS16	0.80	353,727,360	0.80	353,712,960	0.02	97.41	93.73	41.37	99.5
RS17	0.91	404,602,848	0.91	404,594,784	0.02	96.91	92.63	41.42	99.4
RS18	1.04	465,883,776	1.04	465,871,392	0.02	97.12	93.08	41.71	99.5
RS20	1.03	461,024,064	1.03	461,008,224	0.01	97.25	93.46	41.86	99.5
RS22	0.83	367,039,872	0.83	367,031,232	0.02	97.25	93.42	41.51	99.5
RS23	0.95	420,722,784	0.95	420,710,400	0.02	97.1	93.09	41.43	99.5
RS26	0.97	433,393,344	0.97	433,381,248	0.01	97.25	93.5	41.89	99.5

Supplementary Table S1 (continued).

ID	Raw data/Gb	Raw bases	Clean data/Gb	Clean bases	Error rate	Q20	Q30	GC content	Enzyme catch ratio (%)
RS29	1.03	460,085,184	1.03	460,071,360	0.02	97.28	93.48	41.78	99.5
RS30	0.95	419,628,384	0.95	419,620,032	0.02	96.65	91.98	41.28	99.4
ZS1	1.63	723,848,832	1.63	723,846,528	0.01	97.72	94.84	40.44	98.5
ZS2	1.49	662,437,728	1.49	662,436,864	0.01	97.94	95.23	41.09	98.6
ZS3	1.34	597,074,976	1.34	597,074,400	0.01	97.9	95.15	41.03	98.5
ZS4	1.50	668,546,784	1.50	668,545,920	0.01	97.91	95.23	40.95	98.5
ZS7	1.48	657,551,232	1.48	657,549,792	0.01	97.89	95.22	40.69	98.7
ZS8	1.40	624,568,320	1.40	624,567,168	0.01	97.93	95.24	41.13	98.4
ZS9	1.37	610,747,776	1.37	610,747,488	0.01	97.68	94.73	40.3	98.2
ZS10	1.47	655,331,616	1.47	655,330,464	0.01	97.94	95.18	41.02	98
ZS11	1.33	591,719,328	1.33	591,719,040	0.01	97.78	94.81	41.13	98.5
ZS12	1.38	612,705,888	1.38	612,704,448	0.01	97.81	95.11	40.75	97.8
ZS13	1.45	643,852,224	1.45	643,851,648	0.01	97.69	94.84	40.48	98.3
ZS14	0.96	427,900,320	0.96	427,900,320	0.01	97.67	94.81	40.14	98.3
ZS18	1.54	684,231,840	1.54	684,229,536	0.01	97.79	95.03	40.42	97.1
ZS20	1.30	581,005,152	1.30	581,004,288	0.01	97.79	94.97	40.52	98.3
ZS31	1.10	490,484,448	1.10	490,484,448	0.03	96.39	91.26	40.48	98.7
ZS32	1.67	742,958,784	1.67	742,958,784	0.03	96.56	91.59	41.17	98.7
ZS35	1.36	604,194,048	1.36	604,194,048	0.03	96.42	91.25	41.06	98.7
ZS36	1.27	563,654,304	1.27	563,654,016	0.03	96.67	91.86	40.98	98.8
ZS37	0.84	374,124,960	0.84	374,112,864	0.02	97.12	93.07	41.41	99.2

Supplementary Table S3. Characterization of 21 GO annotations obtained from Blast2GO analysis

SeqName	Description	Length	#Hits	e-Value	sim mean	#GO	GO Names list
Cluster 45549	kinesin KIF11	219	20	1.27E-34	96.55%	19	F:protein kinase binding; C:spindle pole; P:chromosome segregation; C:kinesin complex; P:neurogenesis; F:ATP-dependent microtubule motor activity, plus-end-directed; P:microtubule-based movement; P:regulation of mitotic centrosome separation; F:microtubule binding; P:cell division; C:spindle microtubule; F:ATP binding; C:tubulin complex; C:mitotic spindle; C:cytoplasm; P:angiogenesis; F:protein complex binding; P:mitotic spindle assembly
Cluster 135028	dolichol-phosphate mannosyltransferase subunit 1	229	20	1.41E-13	99.40%	6	C:endoplasmic reticulum membrane; P:GPI anchor biosynthetic process; F:dolichyl-phosphate beta-D-mannosyltransferase activity; P:protein O-linked mannosylation; C:alpha-1,6-mannosyltransferase complex; F:dolichyl-phosphate-mannose-protein mannosyltransferase activity
Cluster 75731	probable flap endonuclease 1 homolog	228	20	7.25E-09	87.60%	5	P:nucleic acid phosphodiester bond hydrolysis; F:DNA binding; P:DNA repair; F:catalytic activity; F:nuclease activity
Cluster 143082	arf-GAP with dual PH domain-containing 1-like	308	20	8.00E-07	95%	2	F:GTPase activator activity; P:positive regulation of GTPase activity
Cluster 127795	ras GTPase-activating IQGAP3	308	20	1.63E-15	74.70%	5	F:protein binding; P:small GTPase mediated signal transduction; F:GTPase activator activity; C:intracellular; P:positive regulation of GTPase activity
Cluster 203354	reverse partial	308	6	4.27E-07	63.67%	2	F:RNA-directed DNA polymerase activity; P:RNA-dependent DNA biosynthetic process
Cluster 259434	next to BRCA1 gene 1 -like	308	6	1.03E-04	77.50%	2	C:membrane; C:integral component of membrane
Cluster 351986	lamina-associated polypeptide isoform beta-like	308	20	3.79E-09	90.05%	2	F:DNA binding; C:integral component of membrane
Cluster 469029	BTB POZ domain-containing 2-like	213	20	3.86E-17	99.40%	6	P:proteasome-mediated ubiquitin-dependent protein catabolic process; F:ubiquitin protein ligase binding; P:regulation of proteolysis; C:P-body; C:SCF ubiquitin ligase complex; P:protein ubiquitination involved in ubiquitin-dependent protein catabolic process

Supplementary Table S3 (continued).

SeqName	Description	Length	#Hits	e-Value	sim mean	#GO	GO Names list
Cluster 269334	calpain-3 isoform X1	308	20	2.03E-17	97.15%	6	F:calcium ion binding; P:proteolysis; C:integral component of membrane; P:sarcomere organization; F:calcium-dependent cysteine-type endopeptidase activity; C:intracellular
Cluster 271608	alpha-mannosidase 2	221	20	8.34E-17	89%	7	F:GTP binding; F:alpha-mannosidase activity; F:zinc ion binding; P:small GTPase mediated signal transduction; P:mannose metabolic process; F:carbohydrate binding; C:intracellular
Cluster 376577	rab s geranylgeranyltransferase component A 1-like	308	1	2.09E-04	100%	9	F:GDP-dissociation inhibitor activity; C:Rab-protein geranylgeranyltransferase complex; F:oxidoreductase activity; P:small GTPase mediated signal transduction; P:regulation of catalytic activity; P:oxidation-reduction process; P:protein geranylgeranylation; F:transferase activity; P:intracellular protein transport
Cluster 295522	myosin-IIIb isoform X2	215	20	2.34E-09	92.95%	6	C:myosin complex; F:ATP binding; F:actin binding; F:protein kinase activity; P:protein phosphorylation; F:motor activity
Cluster 29763	serine threonine- kinase PAK 7-parital	308	20	4.48E-16	85.45%	9	F:ATP binding; P:cytoskeleton organization; P:single-multicellular organism process; P:apoptotic process; P:serine family amino acid metabolic process; F:protein serine/threonine kinase activity; P:protein phosphorylation; P:behavior; P:cell growth
Cluster 306989	acyltransferase 3	231	5	9.11E-07	79.20%	2	F:transferase activity, transferring acyl groups; C:membrane
Cluster 313279	lysosomal thioesterase PPT2-A-like	308	20	9.84E-10	98.70%	5	F:palmitoyl-(protein) hydrolase activity; P:macromolecule depalmitoylation; C:intracellular membrane-bounded organelle; C:integral component of membrane; P:cellular protein modification process
Cluster 419322	nuclear receptor coactivator 2 isoform X1	264	20	1.07E-12	89.45%	9	F:histone acetyltransferase activity; P:acyl-carrier-protein biosynthetic process; P:signal transduction; F:ligand-dependent nuclear receptor transcription coactivator activity; P:regulation of transcription, DNA-templated; F:protein dimerization activity; P:histone acetylation; F:nuclear hormone receptor binding; C:histone acetyltransferase complex
Cluster 454925	cytoplasmic dynein 1 intermediate chain 2-like isoform X4	226	20	8.93E-30	96%	2	C:cytoplasmic dynein complex; P:microtubule-based movement

Supplementary Table S3 (continued).

SeqName	Description	Length	#Hits	e-Value	sim mean	#GO	GO Names list
Cluster 526057	ATP-dependent RNA helicase DDX1	216	20	2.21E-16	88.35%	4	F:nucleic acid binding; F:ATP binding; P:RNA secondary structure unwinding; F:ATP-dependent RNA helicase activity
Cluster 555038	RNA-directed DNA polymerase from transposon X-element	210	6	5.65E-07	81.83%	6	F:RNA-directed DNA polymerase activity; F:oxidoreductase activity, acting on paired donors, with incorporation or reduction of molecular oxygen, 2-oxoglutarate as one donor, and incorporation of one atom each of oxygen into both donors; P:RNA-dependent DNA biosynthetic process; P:oxidation-reduction process; F:methyltransferase activity; P:methylation
Cluster 83662	ceruloplasmin-like	312	20	2.54E-21	62.20%	7	C:nucleus; F:copper ion binding; C:cytoplasm; F:oxidoreductase activity; F:metal ion binding; P:oxidation-reduction process; F:ferroxidase activity

Supplementary Table S4. Characterization of 7 KEGG pathways obtained using Blast2GO

Cluster number	Pathway name	Pathway ID	number of Enzyme in each pathway	Enzyme name
Cluster 45549	Thiamine metabolism, Purine metabolism	map00730, map00230	1, 2	ec:3.6.1.15 - phosphatase, ec:3.6.1.3 - adenylpyrophosphatase, ec:3.6.1.15 - phosphatase
Cluster 271608	Other glycan degradation	map00511	1	ec:3.2.1.24 - alpha-D-mannosidase
Cluster 526057	Thiamine metabolism, Purine metabolism	map00730, map00230	1, 2	ec:3.6.1.15 - phosphatase, ec:3.6.1.3 - adenylpyrophosphatase, ec:3.6.1.15 - phosphatase
Cluster 135028	Other types of O-glycan biosynthesis, N-Glycan biosynthesis, Mannose type O-glycan biosynthesis	map00514, map00510 , map00515	1, 1, 1	ec:2.4.1.109 - mannosyltransferase, ec:2.4.1.83 - beta-D-mannosyltransferase, ec:2.4.1.109 - mannosyltransferase
Cluster 313279	Fatty acid elongation	map00062	1	ec:3.1.2.22 - hydrolase
Cluster 295522	Thiamine metabolism, Purine metabolism	map00730, map00230	1, 2	ec:3.6.1.15 - phosphatase, ec:3.6.1.3 - adenylpyrophosphatase, ec:3.6.1.15 - phosphatase

Supplementary Figure S1. Plotting of isolation by distance plotting the log of geographic distance by the log of genetic distance.

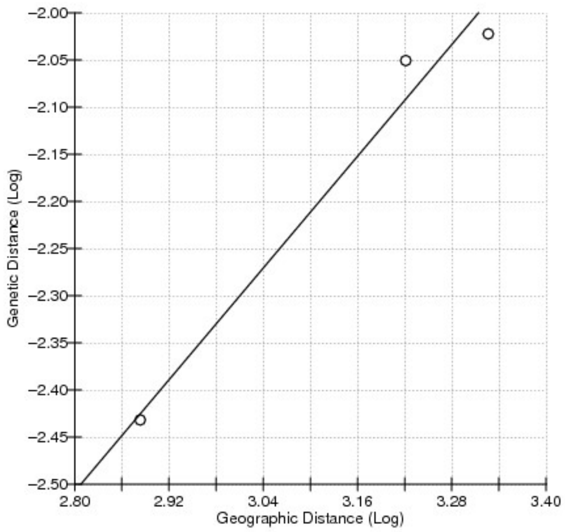

Supplementary Figure S2. Venn diagram of outlier identification based on three pairwise datasets.

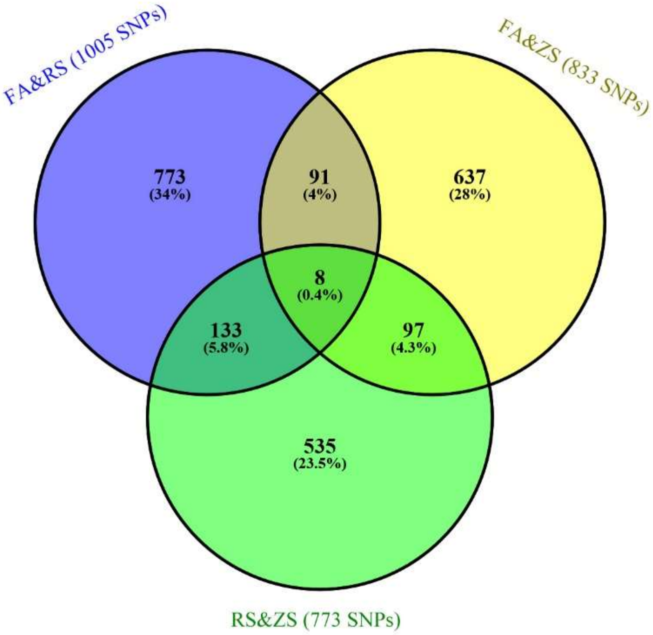

Supplementary Figure S3. NeighborNet topology of *S. marmoratus* individuals among three populations based on all 31,119 SNPs (a) and 329 outlier SNPs (b)

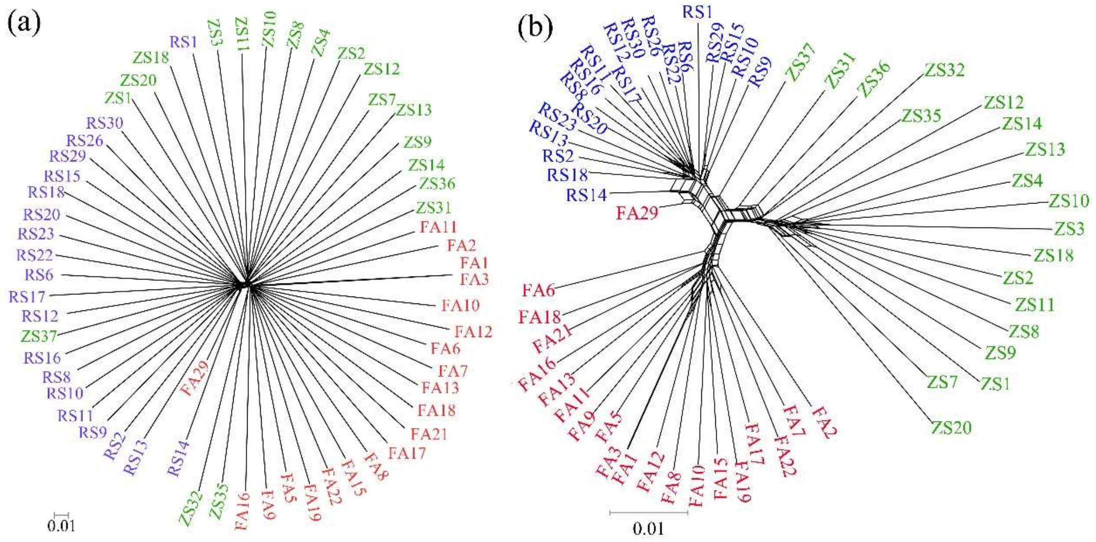

Supplement: Supplementary file 1 — Supplementary Information except Table S2 [file 41598_2017_1742_MOESM1_ESM.pdf]
