# Supplementary material for: Genomic evidence for local adaptation in the ovoviviparous marine fish Sebastiscus marmoratus with a background of population homogeneity
Source: Sci Rep. 2017 May 8;7:1562. doi: 10.1038/s41598-017-01742-z (PMC5431535; doi:10.1038/s41598-017-01742-z)
Supplement: Supplementary file 2 — Supplementary Information Table S2 [file 41598_2017_1742_MOESM2_ESM.pdf]

Supplementary information

Genomic evidence for local adaptation in the ovoviviparous marine fish *Sebastiscus marmoratus* with a background of population homogeneity

Shengyong Xu, Na Song, Linlin Zhao, Shanshan Cai, Zhiqiang Han, Tianxiang Gao

Contents:

Supplementary Table S2

Table S2 Genetic information of each SNP in three S. marmoratus populations									
SNP	Population RS			Population ZS			Population FA		
	Pi	TajimaD	He	Pi	TajimaD	He	Pi	TajimaD	He
0	0.479	1.262	0.481	0.494	1.340	0.501	0.500	1.397	0.501
1	0.500	1.397	0.508	0.488	1.302	0.491	0.450	1.076	0.450
2	0.184	-0.625	0.185	0.342	0.361	0.341	0.358	0.487	0.358
3	0.503	1.413	0.501	0.506	1.415	0.508	0.479	1.262	0.481
4	0.505	1.430	0.512	0.515	1.472	0.514	0.508	1.447	0.508
5	0.484	1.295	0.492	0.488	1.302	0.491	0.513	1.481	0.513
6	0.445	1.043	0.450	0.515	1.472	0.514	0.505	1.430	0.508
7	0.297	0.099	0.296	0.149	-0.882	0.149	0.142	-0.895	0.142
8	0.503	1.413	0.512	0.512	1.453	0.501	0.516	1.498	0.512
9	0.497	1.380	0.508	0.497	1.359	0.491	0.484	1.295	0.481
10	0.497	1.380	0.508	0.485	1.283	0.478	0.484	1.295	0.481
11	0.503	1.413	0.512	0.488	1.302	0.478	0.516	1.498	0.512
12	0.497	1.380	0.508	0.520	1.509	0.512	0.513	1.481	0.512
13	0.497	1.380	0.501	0.506	1.415	0.512	0.482	1.279	0.481
14	0.497	1.380	0.501	0.509	1.434	0.514	0.482	1.279	0.481
15	0.500	1.397	0.512	0.520	1.509	0.512	0.513	1.481	0.508
16	0.500	1.397	0.512	0.518	1.491	0.512	0.487	1.312	0.481
17	0.511	1.464	0.512	0.488	1.302	0.491	0.508	1.447	0.513
18	0.505	1.430	0.512	0.497	1.359	0.501	0.513	1.481	0.508
19	0.503	1.413	0.508	0.515	1.472	0.512	0.505	1.430	0.512
20	0.503	1.413	0.508	0.515	1.472	0.512	0.505	1.430	0.512
21	0.476	1.245	0.481	0.520	1.509	0.514	0.408	0.807	0.409
22	0.226	-0.356	0.224	0.149	-0.882	0.149	0.187	-0.608	0.185
23	0.503	1.413	0.513	0.512	1.453	0.508	0.466	1.178	0.467
24	0.505	1.430	0.513	0.512	1.453	0.508	0.466	1.178	0.467
25	0.513	1.481	0.513	0.512	1.453	0.512	0.463	1.161	0.467
26	0.482	1.279	0.481	0.480	1.246	0.478	0.508	1.447	0.513
27	0.097	-1.181	0.097	0.237	-0.317	0.235	0.226	-0.356	0.224
28	0.487	1.312	0.492	0.512	1.453	0.508	0.505	1.430	0.512
29	0.508	1.447	0.508	0.497	1.359	0.501	0.492	1.346	0.492
30	0.513	1.481	0.513	0.503	1.396	0.508	0.492	1.346	0.492
31	0.466	1.178	0.467	0.509	1.434	0.514	0.511	1.464	0.512
32	0.513	1.481	0.512	0.509	1.434	0.512	0.508	1.447	0.512
33	0.513	1.481	0.512	0.509	1.434	0.512	0.511	1.464	0.513
34	0.505	1.430	0.508	0.497	1.359	0.501	0.500	1.397	0.501
35	0.508	1.447	0.508	0.494	1.340	0.491	0.471	1.211	0.467
36	0.503	1.413	0.508	0.512	1.453	0.514	0.495	1.363	0.492
37	0.503	1.413	0.508	0.512	1.453	0.512	0.495	1.363	0.492
38	0.261	-0.137	0.262	0.199	-0.562	0.193	0.261	-0.137	0.262
39	0.297	0.099	0.296	0.149	-0.882	0.149	0.224	-0.373	0.224
40	0.097	-1.181	0.097	0.272	-0.091	0.273	0.184	-0.625	0.185
41	0.484	1.295	0.481	0.491	1.321	0.491	0.482	1.279	0.481
42	0.500	1.397	0.501	0.503	1.396	0.508	0.503	1.413	0.501
43	0.505	1.430	0.508	0.474	1.208	0.478	0.513	1.481	0.508
44	0.429	0.942	0.431	0.506	1.415	0.512	0.511	1.464	0.512
45	0.495	1.363	0.501	0.444	1.020	0.444	0.513	1.481	0.512
46	0.508	1.447	0.508	0.485	1.283	0.491	0.426	0.925	0.431
47	0.505	1.430	0.508	0.520	1.509	0.514	0.500	1.397	0.501
48	0.479	1.262	0.481	0.462	1.133	0.462	0.500	1.397	0.501
49	0.497	1.380	0.501	0.503	1.396	0.501	0.429	0.942	0.431
50	0.224	-0.373	0.224	0.342	0.361	0.341	0.000	?	0.508
51	0.500	1.397	0.501	0.462	1.133	0.462	0.511	1.464	0.508
52	0.500	1.397	0.501	0.462	1.133	0.462	0.511	1.464	0.512
53	0.508	1.447	0.512	0.482	1.265	0.478	0.511	1.464	0.501
54	0.463	1.161	0.467	0.512	1.453	0.508	0.503	1.413	0.512
55	0.511	1.464	0.513	0.503	1.396	0.501	0.511	1.464	0.501
56	0.463	1.161	0.467	0.515	1.472	0.514	0.503	1.413	0.492
57	0.492	1.346	0.501	0.494	1.340	0.491	0.489	1.329	0.501

58	0.497	1.380	0.508	0.494	1.340	0.491	0.500	1.397	0.501
59	0.489	1.329	0.492	0.512	1.453	0.514	0.500	1.397	0.513
60	0.505	1.430	0.508	0.512	1.453	0.514	0.513	1.481	0.501
61	0.432	0.958	0.431	0.512	1.453	0.512	0.503	1.413	0.508
62	0.450	1.076	0.450	0.506	1.415	0.508	0.508	1.447	0.508
63	0.432	0.958	0.431	0.506	1.415	0.508	0.508	1.447	0.508
64	0.432	0.958	0.431	0.506	1.415	0.508	0.508	1.447	0.508
65	0.432	0.958	0.431	0.506	1.415	0.508	0.508	1.447	0.512
66	0.511	1.464	0.508	0.509	1.434	0.512	0.505	1.430	0.512
67	0.511	1.464	0.508	0.515	1.472	0.514	0.508	1.447	0.513
68	0.463	1.161	0.467	0.488	1.302	0.491	0.513	1.481	0.431
69	0.503	1.413	0.508	0.515	1.472	0.512	0.429	0.942	0.185
70	0.142	−0.895	0.142	0.272	−0.091	0.273	0.184	−0.625	0.508
71	0.511	1.464	0.508	0.488	1.302	0.491	0.505	1.430	0.501
72	0.511	1.464	0.508	0.488	1.302	0.491	0.497	1.380	0.512
73	0.511	1.464	0.513	0.503	1.396	0.501	0.511	1.464	0.097
74	0.329	0.301	0.328	0.149	−0.882	0.149	0.097	−1.181	0.328
75	0.097	−1.181	0.097	0.398	0.718	0.398	0.332	0.318	0.508
76	0.500	1.397	0.512	0.506	1.415	0.501	0.505	1.430	0.328
77	0.097	−1.181	0.097	0.424	0.888	0.422	0.332	0.318	0.492
78	0.492	1.346	0.501	0.465	1.152	0.462	0.495	1.363	0.508
79	0.468	1.194	0.467	0.512	1.453	0.512	0.503	1.413	0.467
80	0.508	1.447	0.508	0.515	1.472	0.512	0.461	1.144	0.513
81	0.484	1.295	0.492	0.515	1.472	0.514	0.513	1.481	0.467
82	0.503	1.413	0.512	0.494	1.340	0.491	0.468	1.194	0.467
83	0.500	1.397	0.512	0.462	1.133	0.462	0.468	1.194	0.328
84	0.000	?	0.050	0.310	0.153	0.309	0.329	0.301	0.385
85	0.050	−1.485	0.050	0.342	0.361	0.341	0.384	0.655	0.224
86	0.000	?	0.050	0.374	0.568	0.371	0.224	−0.373	0.262
87	0.050	−1.485	0.512	0.307	0.135	0.309	0.263	−0.120	0.262
88	0.050	−1.485	0.467	0.275	−0.073	0.273	0.263	−0.120	0.431
89	0.505	1.430	0.512	0.506	1.415	0.508	0.432	0.958	0.512
90	0.466	1.178	0.501	0.488	1.302	0.491	0.511	1.464	0.512
91	0.513	1.481	0.450	0.503	1.396	0.508	0.511	1.464	0.467
92	0.497	1.380	0.513	0.509	1.434	0.508	0.466	1.178	0.431
93	0.453	1.093	0.097	0.506	1.415	0.501	0.426	0.925	0.501
94	0.511	1.464	0.467	0.515	1.472	0.512	0.497	1.380	0.224
95	0.097	−1.181	0.501	0.237	−0.317	0.235	0.226	−0.356	0.513
96	0.466	1.178	0.467	0.515	1.472	0.512	0.513	1.481	0.512
97	0.505	1.430	0.513	0.468	1.170	0.462	0.511	1.464	0.481
98	0.466	1.178	0.262	0.494	1.340	0.491	0.482	1.279	0.513
99	0.511	1.464	0.328	0.459	1.114	0.462	0.511	1.464	0.358
100	0.261	−0.137	0.431	0.345	0.379	0.341	0.361	0.504	0.385
101	0.329	0.301	0.501	0.193	−0.600	0.193	0.387	0.672	0.431
102	0.432	0.958	0.481	0.509	1.434	0.508	0.434	0.975	0.501
103	0.500	1.397	0.481	0.515	1.472	0.512	0.505	1.430	0.467
104	0.476	1.245	0.481	0.485	1.283	0.491	0.466	1.178	0.450
105	0.482	1.279	0.492	0.503	1.396	0.501	0.447	1.060	0.508
106	0.479	1.262	0.513	0.468	1.170	0.462	0.508	1.447	0.262
107	0.000	?	0.185	0.404	0.756	0.398	0.261	−0.137	0.467
108	0.487	1.312	0.508	0.506	1.415	0.508	0.466	1.178	0.492
109	0.511	1.464	0.513	0.512	1.453	0.514	0.492	1.346	0.097
110	0.184	−0.625	0.513	0.272	−0.091	0.273	0.097	−1.181	0.512
111	0.505	1.430	0.513	0.503	1.396	0.512	0.513	1.481	0.450
112	0.511	1.464	0.512	0.494	1.340	0.501	0.450	1.076	0.501
113	0.505	1.430	0.385	0.444	1.020	0.444	0.500	1.397	0.501
114	0.505	1.430	0.508	0.444	1.020	0.444	0.500	1.397	0.409
115	0.503	1.413	0.328	0.491	1.321	0.491	0.405	0.790	0.097
116	0.387	0.672	0.409	0.342	0.361	0.341	0.097	−1.181	0.467
117	0.511	1.464	0.508	0.421	0.869	0.422	0.468	1.194	0.296
118	0.329	0.301	0.450	0.237	−0.317	0.235	0.297	0.099	0.501

119	0.408	0.807	0.185	0.515	1.472	0.514	0.500	1.397	0.501
120	0.505	1.430	0.097	0.506	1.415	0.508	0.503	1.413	0.481
121	0.450	1.076	0.224	0.512	1.453	0.512	0.479	1.262	0.224
122	0.187	-0.608	0.508	0.234	-0.336	0.235	0.224	-0.373	0.262
123	0.097	-1.181	0.385	0.193	-0.600	0.193	0.263	-0.120	0.262
124	0.224	-0.373	0.467	0.193	-0.600	0.193	0.261	-0.137	0.508
125	0.500	1.397	0.450	0.506	1.415	0.508	0.508	1.447	0.508
126	0.384	0.655	0.492	0.485	1.283	0.478	0.508	1.447	0.512
127	0.463	1.161	0.512	0.488	1.302	0.491	0.508	1.447	0.501
128	0.447	1.060	0.224	0.500	1.378	0.501	0.497	1.380	0.512
129	0.487	1.312	0.467	0.491	1.321	0.491	0.513	1.481	0.492
130	0.503	1.413	0.492	0.497	1.359	0.501	0.495	1.363	0.097
131	0.224	-0.373	0.097	0.307	0.135	0.309	0.097	-1.181	0.513
132	0.463	1.161	0.508	0.374	0.568	0.371	0.511	1.464	0.481
133	0.492	1.346	0.224	0.509	1.434	0.514	0.476	1.245	0.262
134	0.100	-1.164	0.467	0.237	-0.317	0.235	0.261	-0.137	0.467
135	0.508	1.447	0.224	0.512	1.453	0.514	0.463	1.161	0.296
136	0.224	-0.373	0.501	0.275	-0.073	0.273	0.297	0.099	0.512
137	0.463	1.161	0.512	0.509	1.434	0.512	0.508	1.447	0.262
138	0.224	-0.373	0.512	0.149	-0.882	0.149	0.261	-0.137	0.450
139	0.497	1.380	0.501	0.512	1.453	0.512	0.447	1.060	0.512
140	0.513	1.481	0.501	0.398	0.718	0.398	0.508	1.447	0.508
141	0.511	1.464	0.492	0.494	1.340	0.491	0.503	1.413	0.508
142	0.497	1.380	0.512	0.477	1.227	0.478	0.508	1.447	0.431
143	0.492	1.346	0.262	0.503	1.396	0.501	0.432	0.958	0.512
144	0.489	1.329	0.185	0.494	1.340	0.501	0.518	1.514	0.492
145	0.500	1.397	0.296	0.515	1.472	0.512	0.492	1.346	0.296
146	0.263	-0.120	0.508	0.234	-0.336	0.235	0.295	0.082	0.358
147	0.184	-0.625	0.431	0.275	-0.073	0.273	0.361	0.504	0.409
148	0.297	0.099	0.481	0.398	0.718	0.398	0.413	0.841	0.501
149	0.508	1.447	0.097	0.494	1.340	0.491	0.503	1.413	0.512
150	0.429	0.942	0.481	0.512	1.453	0.508	0.508	1.447	0.512
151	0.479	1.262	0.513	0.503	1.396	0.508	0.513	1.481	0.296
152	0.097	-1.181	0.508	0.272	-0.091	0.273	0.295	0.082	0.513
153	0.479	1.262	0.492	0.509	1.434	0.508	0.516	1.498	0.508
154	0.518	1.514	0.481	0.509	1.434	0.508	0.505	1.430	0.508
155	0.516	1.498	0.512	0.515	1.472	0.512	0.505	1.430	0.508
156	0.492	1.346	0.513	0.509	1.434	0.508	0.503	1.413	0.508
157	0.482	1.279	0.481	0.509	1.434	0.508	0.503	1.413	0.431
158	0.500	1.397	0.508	0.488	1.302	0.491	0.434	0.975	0.467
159	0.513	1.481	0.467	0.444	1.020	0.444	0.466	1.178	0.481
160	0.479	1.262	0.501	0.442	1.001	0.444	0.479	1.262	0.501
161	0.505	1.430	0.501	0.497	1.359	0.501	0.505	1.430	0.481
162	0.463	1.161	0.513	0.509	1.434	0.512	0.484	1.295	0.512
163	0.492	1.346	0.501	0.480	1.246	0.478	0.511	1.464	0.512
164	0.492	1.346	0.513	0.480	1.246	0.478	0.511	1.464	0.481
165	0.500	1.397	0.481	0.494	1.340	0.491	0.482	1.279	0.513
166	0.492	1.346	0.224	0.480	1.246	0.478	0.511	1.464	0.481
167	0.500	1.397	0.224	0.518	1.491	0.512	0.482	1.279	0.513
168	0.474	1.228	0.467	0.512	1.453	0.508	0.513	1.481	0.185
169	0.226	-0.356	0.508	0.275	-0.073	0.273	0.184	-0.625	0.185
170	0.226	-0.356	0.492	0.237	-0.317	0.235	0.184	-0.625	0.513
171	0.466	1.178	0.512	0.515	1.472	0.514	0.511	1.464	0.508
172	0.505	1.430	0.097	0.480	1.246	0.478	0.508	1.447	0.512
173	0.487	1.312	0.262	0.459	1.114	0.462	0.513	1.481	0.481
174	0.508	1.447	0.508	0.509	1.434	0.508	0.479	1.262	0.262
175	0.097	-1.181	0.512	0.234	-0.336	0.235	0.261	-0.137	0.328
176	0.261	-0.137	0.512	0.275	-0.073	0.273	0.329	0.301	0.512
177	0.503	1.413	0.501	0.518	1.491	0.514	0.513	1.481	0.508
178	0.503	1.413	0.142	0.485	1.283	0.478	0.500	1.397	0.508
179	0.503	1.413	0.097	0.485	1.283	0.478	0.500	1.397	0.467

180	0.503	1.413	0.097	0.497	1.359	0.491	0.461	1.144	0.185
181	0.142	-0.895	0.097	0.234	-0.336	0.235	0.184	-0.625	0.358
182	0.097	-1.181	0.512	0.237	-0.317	0.235	0.358	0.487	0.328
183	0.097	-1.181	0.508	0.152	-0.863	0.149	0.326	0.285	0.358
184	0.097	-1.181	0.508	0.278	-0.054	0.273	0.358	0.487	0.224
185	0.000	?	0.296	0.307	0.135	0.309	0.224	-0.373	0.481
186	0.511	1.464	0.508	0.480	1.246	0.478	0.482	1.279	0.467
187	0.503	1.413	0.512	0.462	1.133	0.462	0.466	1.178	0.501
188	0.505	1.430	0.492	0.444	1.020	0.444	0.500	1.397	0.385
189	0.297	0.099	0.492	0.275	-0.073	0.273	0.387	0.672	0.513
190	0.505	1.430	0.508	0.509	1.434	0.514	0.513	1.481	0.513
191	0.503	1.413	0.508	0.465	1.152	0.462	0.516	1.498	0.501
192	0.487	1.312	0.050	0.512	1.453	0.512	0.500	1.397	0.501
193	0.487	1.312	0.097	0.494	1.340	0.491	0.500	1.397	0.481
194	0.505	1.430	0.328	0.488	1.302	0.491	0.482	1.279	0.512
195	0.508	1.447	0.224	0.509	1.434	0.512	0.508	1.447	0.358
196	0.050	-1.485	0.513	0.149	-0.882	0.149	0.355	0.470	0.467
197	0.097	-1.181	0.481	0.310	0.153	0.309	0.466	1.178	0.142
198	0.329	0.301	0.501	0.275	-0.073	0.273	0.145	-0.878	0.296
199	0.224	-0.373	0.512	0.275	-0.073	0.273	0.297	0.099	0.501
200	0.511	1.464	0.481	0.442	1.001	0.444	0.497	1.380	0.467
201	0.479	1.262	0.508	0.512	1.453	0.512	0.466	1.178	0.501
202	0.503	1.413	0.508	0.444	1.020	0.444	0.500	1.397	0.512
203	0.508	1.447	0.185	0.515	1.472	0.514	0.508	1.447	0.512
204	0.476	1.245	0.508	0.518	1.491	0.514	0.505	1.430	0.508
205	0.505	1.430	0.512	0.518	1.491	0.514	0.503	1.413	0.508
206	0.505	1.430	0.513	0.512	1.453	0.512	0.508	1.447	0.185
207	0.184	-0.625	0.501	0.237	-0.317	0.235	0.187	-0.608	0.512
208	0.503	1.413	0.481	0.494	1.340	0.501	0.513	1.481	0.492
209	0.508	1.447	0.492	0.444	1.020	0.444	0.492	1.346	0.501
210	0.513	1.481	0.512	0.482	1.265	0.478	0.497	1.380	0.508
211	0.500	1.397	0.467	0.482	1.265	0.478	0.505	1.430	0.467
212	0.479	1.262	0.467	0.509	1.434	0.508	0.466	1.178	0.501
213	0.487	1.312	0.501	0.509	1.434	0.512	0.500	1.397	0.501
214	0.511	1.464	0.492	0.512	1.453	0.512	0.497	1.380	0.467
215	0.466	1.178	0.492	0.506	1.415	0.508	0.463	1.161	0.467
216	0.466	1.178	0.508	0.506	1.415	0.508	0.463	1.161	0.512
217	0.500	1.397	0.508	0.480	1.246	0.478	0.505	1.430	0.492
218	0.489	1.329	0.512	0.509	1.434	0.514	0.489	1.329	0.492
219	0.492	1.346	0.512	0.509	1.434	0.508	0.484	1.295	0.501
220	0.505	1.430	0.508	0.491	1.321	0.491	0.503	1.413	0.501
221	0.511	1.464	0.508	0.418	0.850	0.422	0.497	1.380	0.431
222	0.513	1.481	0.185	0.512	1.453	0.512	0.426	0.925	0.492
223	0.508	1.447	0.431	0.512	1.453	0.512	0.487	1.312	0.508
224	0.508	1.447	0.481	0.500	1.378	0.501	0.505	1.430	0.513
225	0.508	1.447	0.501	0.500	1.378	0.501	0.508	1.447	0.296
226	0.187	-0.608	0.508	0.234	-0.336	0.235	0.295	0.082	0.512
227	0.437	0.992	0.508	0.515	1.472	0.508	0.508	1.447	0.501
228	0.482	1.279	0.296	0.474	1.208	0.478	0.505	1.430	0.501
229	0.495	1.363	0.097	0.512	1.453	0.508	0.497	1.380	0.492
230	0.505	1.430	0.512	0.465	1.152	0.462	0.489	1.329	0.512
231	0.505	1.430	0.513	0.512	1.453	0.512	0.513	1.481	0.409
232	0.297	0.099	0.508	0.234	-0.336	0.235	0.411	0.824	0.224
233	0.097	-1.181	0.501	0.237	-0.317	0.235	0.224	-0.373	0.501
234	0.511	1.464	0.501	0.506	1.415	0.512	0.503	1.413	0.501
235	0.511	1.464	0.185	0.497	1.359	0.501	0.503	1.413	0.508
236	0.505	1.430	0.508	0.459	1.114	0.462	0.505	1.430	0.467
237	0.495	1.363	0.513	0.509	1.434	0.512	0.468	1.194	0.450
238	0.495	1.363	0.513	0.509	1.434	0.512	0.450	1.076	0.142
239	0.187	-0.608	0.501	0.237	-0.317	0.235	0.142	-0.895	0.492
240	0.511	1.464	0.262	0.506	1.415	0.508	0.489	1.329	0.492

241	0.516	1.498	0.185	0.509	1.434	0.512	0.489	1.329	0.492
242	0.516	1.498	0.508	0.509	1.434	0.512	0.489	1.329	0.508
243	0.497	1.380	0.512	0.398	0.718	0.398	0.511	1.464	0.262
244	0.261	-0.137	0.508	0.102	-1.184	0.102	0.266	-0.103	0.262
245	0.184	-0.625	0.492	0.102	-1.184	0.102	0.266	-0.103	0.513
246	0.500	1.397	0.512	0.477	1.227	0.478	0.513	1.481	0.508
247	0.503	1.413	0.508	0.480	1.246	0.478	0.511	1.464	0.501
248	0.508	1.447	0.508	0.515	1.472	0.512	0.500	1.397	0.508
249	0.489	1.329	0.492	0.477	1.227	0.478	0.508	1.447	0.508
250	0.505	1.430	0.097	0.512	1.453	0.512	0.511	1.464	0.512
251	0.503	1.413	0.512	0.506	1.415	0.508	0.511	1.464	0.512
252	0.503	1.413	0.501	0.506	1.415	0.508	0.511	1.464	0.385
253	0.484	1.295	0.513	0.518	1.491	0.512	0.382	0.638	0.296
254	0.097	-1.181	0.512	0.310	0.153	0.309	0.300	0.116	0.450
255	0.500	1.397	0.328	0.447	1.039	0.444	0.453	1.093	0.501
256	0.505	1.430	0.262	0.506	1.415	0.508	0.500	1.397	0.512
257	0.505	1.430	0.142	0.465	1.152	0.462	0.518	1.514	0.508
258	0.505	1.430	0.142	0.509	1.434	0.512	0.508	1.447	0.224
259	0.329	0.301	0.513	0.313	0.172	0.309	0.224	-0.373	0.296
260	0.263	-0.120	0.467	0.234	-0.336	0.235	0.297	0.099	0.185
261	0.142	-0.895	0.501	0.368	0.530	0.371	0.184	-0.625	0.142
262	0.142	-0.895	0.513	0.342	0.361	0.341	0.142	-0.895	0.512
263	0.513	1.481	0.467	0.485	1.283	0.491	0.508	1.447	0.450
264	0.466	1.178	0.501	0.480	1.246	0.478	0.455	1.110	0.385
265	0.500	1.397	0.501	0.503	1.396	0.501	0.387	0.672	0.358
266	0.511	1.464	0.512	0.506	1.415	0.508	0.358	0.487	0.501
267	0.468	1.194	0.050	0.456	1.095	0.462	0.500	1.397	0.513
268	0.500	1.397	0.501	0.497	1.359	0.501	0.511	1.464	0.492
269	0.497	1.380	0.050	0.444	1.020	0.444	0.489	1.329	0.467
270	0.511	1.464	0.508	0.503	1.396	0.508	0.471	1.211	0.296
271	0.050	-1.485	0.050	0.196	-0.581	0.193	0.295	0.082	0.431
272	0.492	1.346	0.508	0.503	1.396	0.501	0.434	0.975	0.358
273	0.050	-1.485	0.467	0.193	-0.600	0.193	0.361	0.504	0.492
274	0.497	1.380	0.512	0.506	1.415	0.501	0.489	1.329	0.185
275	0.050	-1.485	0.508	0.310	0.153	0.309	0.187	-0.608	0.513
276	0.505	1.430	0.492	0.506	1.415	0.508	0.511	1.464	0.512
277	0.471	1.211	0.501	0.497	1.359	0.501	0.511	1.464	0.508
278	0.511	1.464	0.224	0.474	1.208	0.478	0.505	1.430	0.481
279	0.508	1.447	0.358	0.456	1.095	0.462	0.479	1.262	0.431
280	0.487	1.312	0.296	0.512	1.453	0.512	0.429	0.942	0.513
281	0.492	1.346	0.296	0.482	1.265	0.478	0.513	1.481	0.224
282	0.226	-0.356	0.296	0.102	-1.184	0.102	0.224	-0.373	0.492
283	0.358	0.487	0.492	0.485	1.283	0.478	0.489	1.329	0.185
284	0.297	0.099	0.262	0.193	-0.600	0.193	0.184	-0.625	0.185
285	0.297	0.099	0.262	0.272	-0.091	0.273	0.184	-0.625	0.097
286	0.297	0.099	0.142	0.193	-0.600	0.193	0.097	-1.181	0.512
287	0.492	1.346	0.262	0.421	0.869	0.422	0.511	1.464	0.224
288	0.261	-0.137	0.431	0.149	-0.882	0.149	0.224	-0.373	0.262
289	0.261	-0.137	0.328	0.149	-0.882	0.149	0.261	-0.137	0.262
290	0.142	-0.895	0.385	0.234	-0.336	0.235	0.263	-0.120	0.385
291	0.263	-0.120	0.513	0.275	-0.073	0.273	0.384	0.655	0.385
292	0.429	0.942	0.185	0.459	1.114	0.462	0.384	0.655	0.142
293	0.326	0.285	0.185	0.234	-0.336	0.235	0.142	-0.895	0.262
294	0.387	0.672	0.358	0.272	-0.091	0.273	0.263	-0.120	0.513
295	0.513	1.481	0.224	0.401	0.737	0.398	0.513	1.481	0.409
296	0.184	-0.625	0.492	0.149	-0.882	0.149	0.413	0.841	0.450
297	0.184	-0.625	0.501	0.149	-0.882	0.149	0.453	1.093	0.224
298	0.358	0.487	0.492	0.199	-0.562	0.193	0.224	-0.373	0.185
299	0.224	-0.373	0.142	0.152	-0.863	0.149	0.184	-0.625	0.501
300	0.495	1.363	0.492	0.518	1.491	0.514	0.497	1.380	0.481
301	0.500	1.397	0.492	0.444	1.020	0.444	0.482	1.279	0.508

302	0.489	1.329	0.512	0.477	1.227	0.478	0.503	1.413	0.185
303	0.142	-0.895	0.097	0.310	0.153	0.309	0.187	-0.608	0.513
304	0.484	1.295	0.097	0.488	1.302	0.491	0.513	1.481	0.513
305	0.484	1.295	0.492	0.488	1.302	0.491	0.513	1.481	0.492
306	0.503	1.413	0.492	0.398	0.718	0.398	0.495	1.363	0.358
307	0.097	-1.181	0.481	0.272	-0.091	0.273	0.361	0.504	0.358
308	0.097	-1.181	0.481	0.272	-0.091	0.273	0.361	0.504	0.450
309	0.495	1.363	0.513	0.482	1.265	0.478	0.445	1.043	0.481
310	0.492	1.346	0.512	0.480	1.246	0.478	0.482	1.279	0.492
311	0.482	1.279	0.512	0.468	1.170	0.462	0.492	1.346	0.481
312	0.482	1.279	0.501	0.506	1.415	0.501	0.479	1.262	0.512
313	0.505	1.430	0.501	0.491	1.321	0.491	0.511	1.464	0.508
314	0.505	1.430	0.142	0.518	1.491	0.512	0.511	1.464	0.508
315	0.505	1.430	0.097	0.520	1.509	0.514	0.511	1.464	0.501
316	0.495	1.363	0.262	0.497	1.359	0.491	0.505	1.430	0.508
317	0.497	1.380	0.512	0.497	1.359	0.491	0.511	1.464	0.262
318	0.142	-0.895	0.513	0.310	0.153	0.309	0.261	-0.137	0.328
319	0.097	-1.181	0.262	0.237	-0.317	0.235	0.326	0.285	0.296
320	0.266	-0.103	0.512	0.339	0.342	0.341	0.295	0.082	0.385
321	0.503	1.413	0.508	0.494	1.340	0.501	0.387	0.672	0.512
322	0.505	1.430	0.409	0.503	1.396	0.501	0.513	1.481	0.224
323	0.263	-0.120	0.512	0.193	-0.600	0.193	0.226	-0.356	0.481
324	0.505	1.430	0.512	0.462	1.133	0.462	0.482	1.279	0.492
325	0.505	1.430	0.508	0.465	1.152	0.462	0.489	1.329	0.508
326	0.408	0.807	0.224	0.518	1.491	0.514	0.503	1.413	0.481
327	0.508	1.447	0.262	0.497	1.359	0.501	0.479	1.262	0.328
328	0.511	1.464	0.262	0.518	1.491	0.512	0.329	0.301	0.492
329	0.505	1.430	0.501	0.482	1.265	0.478	0.487	1.312	0.185
330	0.224	-0.373	0.501	0.234	-0.336	0.235	0.187	-0.608	0.358
331	0.261	-0.137	0.224	0.196	-0.581	0.193	0.361	0.504	0.358
332	0.261	-0.137	0.224	0.196	-0.581	0.193	0.361	0.504	0.492
333	0.500	1.397	0.481	0.491	1.321	0.491	0.484	1.295	0.492
334	0.500	1.397	0.481	0.480	1.246	0.478	0.484	1.295	0.328
335	0.224	-0.373	0.328	0.374	0.568	0.371	0.000	?	0.481
336	0.224	-0.373	0.142	0.374	0.568	0.371	0.329	0.301	0.450
337	0.484	1.295	0.262	0.503	1.396	0.501	0.482	1.279	0.358
338	0.476	1.245	0.296	0.482	1.265	0.478	0.445	1.043	0.185
339	0.326	0.285	0.296	0.339	0.342	0.341	0.358	0.487	0.050
340	0.142	-0.895	0.296	0.275	-0.073	0.273	0.184	-0.625	0.142
341	0.261	-0.137	0.508	0.272	-0.091	0.273	0.050	-1.485	0.097
342	0.295	0.082	0.328	0.368	0.530	0.371	0.142	-0.895	0.097
343	0.295	0.082	0.467	0.368	0.530	0.371	0.097	-1.181	0.481
344	0.295	0.082	0.296	0.368	0.530	0.371	0.097	-1.181	0.185
345	0.503	1.413	0.224	0.488	1.302	0.491	0.479	1.262	0.467
346	0.329	0.301	0.185	0.149	-0.882	0.149	0.184	-0.625	0.185
347	0.468	1.194	0.508	0.497	1.359	0.501	0.463	1.161	0.097
348	0.295	0.082	0.097	0.237	-0.317	0.235	0.184	-0.625	0.185
349	0.224	-0.373	0.142	0.313	0.172	0.309	0.097	-1.181	0.481
350	0.184	-0.625	0.450	0.275	-0.073	0.273	0.184	-0.625	0.358
351	0.505	1.430	0.296	0.491	1.321	0.491	0.474	1.228	0.262
352	0.097	-1.181	0.262	0.105	-1.165	0.102	0.358	0.487	0.262
353	0.142	-0.895	0.501	0.395	0.700	0.398	0.261	-0.137	0.431
354	0.453	1.093	0.262	0.237	-0.317	0.235	0.261	-0.137	0.296
355	0.295	0.082	0.501	0.152	-0.863	0.149	0.429	0.942	0.513
356	0.263	-0.120	0.328	0.339	0.342	0.341	0.295	0.082	0.296
357	0.503	1.413	0.262	0.424	0.888	0.422	0.511	1.464	0.467
358	0.261	-0.137	0.358	0.348	0.398	0.341	0.295	0.082	0.142
359	0.495	1.363	0.224	0.462	1.133	0.462	0.466	1.178	0.409
360	0.329	0.301	0.142	0.053	-1.504	0.053	0.142	-0.895	0.185
361	0.263	-0.120	0.296	0.342	0.361	0.341	0.411	0.824	0.328
362	0.355	0.470	0.492	0.237	-0.317	0.235	0.184	-0.625	0.262

363	0.226	-0.356	0.409	0.345	0.379	0.341	0.329	0.301	0.385
364	0.142	-0.895	0.262	0.310	0.153	0.309	0.263	-0.120	0.492
365	0.297	0.099	0.492	0.272	-0.091	0.273	0.384	0.655	0.467
366	0.492	1.346	0.481	0.515	1.472	0.512	0.492	1.346	0.185
367	0.411	0.824	0.450	0.515	1.472	0.512	0.463	1.161	0.513
368	0.263	-0.120	0.450	0.149	-0.882	0.149	0.184	-0.625	0.512
369	0.495	1.363	0.501	0.462	1.133	0.462	0.505	1.430	0.409
370	0.484	1.295	0.142	0.424	0.888	0.422	0.505	1.430	0.409
371	0.453	1.093	0.513	0.515	1.472	0.514	0.408	0.807	0.467
372	0.453	1.093	0.328	0.500	1.378	0.501	0.413	0.841	0.296
373	0.503	1.413	0.185	0.518	1.491	0.514	0.463	1.161	0.450
374	0.142	-0.895	0.185	0.237	-0.317	0.235	0.297	0.099	0.097
375	0.508	1.447	0.185	0.491	1.321	0.491	0.450	1.076	0.097
376	0.329	0.301	0.296	0.272	-0.091	0.273	0.097	-1.181	0.431
377	0.184	-0.625	0.296	0.278	-0.054	0.273	0.097	-1.181	0.431
378	0.184	-0.625	0.142	0.462	1.133	0.462	0.429	0.942	0.142
379	0.184	-0.625	0.224	0.462	1.133	0.462	0.429	0.942	0.142
380	0.295	0.082	0.481	0.307	0.135	0.309	0.142	-0.895	0.262
381	0.295	0.082	0.481	0.307	0.135	0.309	0.142	-0.895	0.185
382	0.142	-0.895	0.512	0.234	-0.336	0.235	0.263	-0.120	0.513
383	0.224	-0.373	0.296	0.237	-0.317	0.235	0.184	-0.625	0.513
384	0.476	1.245	0.508	0.509	1.434	0.512	0.513	1.481	0.467
385	0.476	1.245	0.508	0.509	1.434	0.514	0.513	1.481	0.185
386	0.508	1.447	0.481	0.480	1.246	0.478	0.463	1.161	0.481
387	0.295	0.082	0.501	0.053	-1.504	0.053	0.184	-0.625	0.481
388	0.505	1.430	0.142	0.488	1.302	0.491	0.482	1.279	0.467
389	0.505	1.430	0.328	0.488	1.302	0.491	0.482	1.279	0.481
390	0.482	1.279	0.450	0.491	1.321	0.491	0.468	1.194	0.142
391	0.492	1.346	0.097	0.444	1.020	0.444	0.482	1.279	0.481
392	0.142	-0.895	0.501	0.313	0.172	0.309	0.142	-0.895	0.142
393	0.329	0.301	0.508	0.307	0.135	0.309	0.000	?	0.467
394	0.450	1.076	0.224	0.506	1.415	0.508	0.474	1.228	0.508
395	0.097	-1.181	0.262	0.313	0.172	0.309	0.142	-0.895	0.185
396	0.497	1.380	0.142	0.515	1.472	0.514	0.461	1.144	0.185
397	0.508	1.447	0.450	0.506	1.415	0.501	0.511	1.464	0.296
398	0.224	-0.373	0.224	0.237	-0.317	0.235	0.184	-0.625	0.492
399	0.261	-0.137	0.262	0.193	-0.600	0.193	0.184	-0.625	0.262
400	0.142	-0.895	0.467	0.193	-0.600	0.193	0.295	0.082	0.358
401	0.447	1.060	0.512	0.459	1.114	0.462	0.497	1.380	0.492
402	0.224	-0.373	0.385	0.149	-0.882	0.149	0.263	-0.120	0.501
403	0.263	-0.120	0.431	0.234	-0.336	0.235	0.355	0.470	0.492
404	0.466	1.178	0.431	0.427	0.907	0.422	0.492	1.346	0.512
405	0.508	1.447	0.467	0.442	1.001	0.444	0.497	1.380	0.512
406	0.382	0.638	0.450	0.515	1.472	0.512	0.492	1.346	0.508
407	0.429	0.942	0.385	0.488	1.302	0.491	0.511	1.464	0.481
408	0.429	0.942	0.501	0.477	1.227	0.478	0.511	1.464	0.262
409	0.468	1.194	0.508	0.500	1.378	0.501	0.508	1.447	0.492
410	0.453	1.093	0.224	0.488	1.302	0.491	0.484	1.295	0.492
411	0.387	0.672	0.467	0.275	-0.073	0.273	0.263	-0.120	0.185
412	0.492	1.346	0.492	0.518	1.491	0.514	0.492	1.346	0.431
413	0.505	1.430	0.513	0.465	1.152	0.462	0.484	1.295	0.508
414	0.226	-0.356	0.501	0.237	-0.317	0.235	0.184	-0.625	0.513
415	0.468	1.194	0.492	0.518	1.491	0.514	0.432	0.958	0.467
416	0.495	1.363	0.142	0.465	1.152	0.462	0.508	1.447	0.501
417	0.511	1.464	0.492	0.512	1.453	0.512	0.513	1.481	0.262
418	0.503	1.413	0.501	0.518	1.491	0.514	0.466	1.178	0.501
419	0.492	1.346	0.409	0.503	1.396	0.501	0.497	1.380	0.501
420	0.142	-0.895	0.224	0.196	-0.581	0.193	0.263	-0.120	0.224
421	0.492	1.346	0.512	0.509	1.434	0.508	0.497	1.380	0.296
422	0.497	1.380	0.508	0.506	1.415	0.508	0.503	1.413	0.481
423	0.413	0.841	0.508	0.371	0.549	0.371	0.224	-0.373	0.431

424	0.224	-0.373	0.185	0.307	0.135	0.309	0.295	0.082	0.512
425	0.513	1.481	0.508	0.491	1.321	0.491	0.482	1.279	0.328
426	0.513	1.481	0.512	0.509	1.434	0.508	0.426	0.925	0.501
427	0.511	1.464	0.512	0.444	1.020	0.444	0.511	1.464	0.512
428	0.184	-0.625	0.508	0.102	-1.184	0.102	0.329	0.301	0.508
429	0.505	1.430	0.512	0.494	1.340	0.501	0.500	1.397	0.467
430	0.503	1.413	0.501	0.465	1.152	0.462	0.511	1.464	0.431
431	0.503	1.413	0.501	0.465	1.152	0.462	0.508	1.447	0.481
432	0.497	1.380	0.512	0.494	1.340	0.491	0.463	1.161	0.501
433	0.511	1.464	0.512	0.506	1.415	0.508	0.426	0.925	0.492
434	0.500	1.397	0.481	0.503	1.396	0.501	0.479	1.262	0.492
435	0.500	1.397	0.512	0.509	1.434	0.508	0.497	1.380	0.512
436	0.511	1.464	0.409	0.509	1.434	0.512	0.492	1.346	0.513
437	0.511	1.464	0.501	0.509	1.434	0.512	0.492	1.346	0.328
438	0.476	1.245	0.501	0.442	1.001	0.444	0.516	1.498	0.328
439	0.511	1.464	0.512	0.444	1.020	0.444	0.518	1.514	0.328
440	0.416	0.857	0.508	0.374	0.568	0.371	0.326	0.285	0.431
441	0.503	1.413	0.508	0.503	1.396	0.501	0.329	0.301	0.492
442	0.503	1.413	0.508	0.503	1.396	0.501	0.329	0.301	0.492
443	0.513	1.481	0.467	0.509	1.434	0.508	0.432	0.958	0.492
444	0.503	1.413	0.358	0.506	1.415	0.514	0.497	1.380	0.467
445	0.503	1.413	0.450	0.506	1.415	0.514	0.497	1.380	0.185
446	0.503	1.413	0.501	0.506	1.415	0.514	0.497	1.380	0.508
447	0.471	1.211	0.508	0.491	1.321	0.491	0.468	1.194	0.512
448	0.363	0.520	0.508	0.234	-0.336	0.235	0.184	-0.625	0.492
449	0.445	1.043	0.050	0.424	0.888	0.422	0.508	1.447	0.501
450	0.500	1.397	0.050	0.427	0.907	0.422	0.511	1.464	0.224
451	0.513	1.481	0.409	0.500	1.378	0.501	0.492	1.346	0.358
452	0.497	1.380	0.512	0.503	1.396	0.501	0.500	1.397	0.513
453	0.050	-1.485	0.508	0.447	1.039	0.444	0.224	-0.373	0.409
454	0.050	-1.485	0.512	0.345	0.379	0.341	0.355	0.470	0.467
455	0.408	0.807	0.385	0.515	1.472	0.512	0.516	1.498	0.512
456	0.511	1.464	0.512	0.442	1.001	0.444	0.411	0.824	0.481
457	0.505	1.430	0.512	0.468	1.170	0.462	0.466	1.178	0.431
458	0.513	1.481	0.512	0.503	1.396	0.501	0.505	1.430	0.431
459	0.382	0.638	0.097	0.506	1.415	0.508	0.482	1.279	0.431
460	0.508	1.447	0.097	0.497	1.359	0.501	0.432	0.958	0.224
461	0.508	1.447	0.450	0.497	1.359	0.501	0.432	0.958	0.224
462	0.508	1.447	0.481	0.503	1.396	0.508	0.432	0.958	0.512
463	0.097	-1.181	0.142	0.371	0.549	0.371	0.224	-0.373	0.512
464	0.097	-1.181	0.450	0.371	0.549	0.371	0.224	-0.373	0.296
465	0.450	1.076	0.224	0.462	1.133	0.462	0.513	1.481	0.467
466	0.482	1.279	0.224	0.515	1.472	0.512	0.513	1.481	0.409
467	0.142	-0.895	0.224	0.310	0.153	0.309	0.300	0.116	0.409
468	0.447	1.060	0.431	0.509	1.434	0.508	0.466	1.178	0.409
469	0.224	-0.373	0.409	0.371	0.549	0.371	0.413	0.841	0.467
470	0.224	-0.373	0.185	0.371	0.549	0.371	0.413	0.841	0.467
471	0.224	-0.373	0.512	0.342	0.361	0.341	0.413	0.841	0.224
472	0.429	0.942	0.185	0.482	1.265	0.478	0.466	1.178	0.508
473	0.405	0.790	0.501	0.482	1.265	0.478	0.466	1.178	0.224
474	0.184	-0.625	0.142	0.149	-0.882	0.149	0.224	-0.373	0.481
475	0.500	1.397	0.512	0.503	1.396	0.501	0.508	1.447	0.358
476	0.184	-0.625	0.050	0.377	0.587	0.371	0.224	-0.373	0.385
477	0.500	1.397	0.513	0.482	1.265	0.478	0.484	1.295	0.097
478	0.142	-0.895	0.512	0.240	-0.299	0.235	0.358	0.487	0.481
479	0.516	1.498	0.328	0.439	0.982	0.444	0.387	0.672	0.450
480	0.050	-1.485	0.262	0.371	0.549	0.371	0.100	-1.164	0.050
481	0.513	1.481	0.501	0.512	1.453	0.512	0.482	1.279	0.142
482	0.511	1.464	0.508	0.512	1.453	0.514	0.450	1.076	0.513
483	0.329	0.301	0.385	0.149	-0.882	0.149	0.050	-1.485	0.467
484	0.261	-0.137	0.508	0.272	-0.091	0.273	0.145	-0.878	0.328

485	0.497	1.380	0.409	0.512	1.453	0.512	0.508	1.447	0.501
486	0.508	1.447	0.513	0.480	1.246	0.478	0.466	1.178	0.508
487	0.387	0.672	0.512	0.377	0.587	0.371	0.329	0.301	0.450
488	0.511	1.464	0.501	0.509	1.434	0.508	0.503	1.413	0.481
489	0.408	0.807	0.431	0.444	1.020	0.444	0.505	1.430	0.467
490	0.500	1.397	0.142	0.494	1.340	0.491	0.450	1.076	0.512
491	0.508	1.447	0.501	0.506	1.415	0.508	0.476	1.245	0.185
492	0.500	1.397	0.513	0.503	1.396	0.508	0.466	1.178	0.492
493	0.434	0.975	0.501	0.520	1.509	0.514	0.508	1.447	0.431
494	0.145	−0.878	0.501	0.237	−0.317	0.235	0.184	−0.625	0.481
495	0.497	1.380	0.097	0.509	1.434	0.512	0.497	1.380	0.481
496	0.511	1.464	0.467	0.512	1.453	0.514	0.434	0.975	0.409
497	0.492	1.346	0.508	0.515	1.472	0.512	0.482	1.279	0.431
498	0.492	1.346	0.050	0.515	1.472	0.512	0.482	1.279	0.508
499	0.097	−1.181	0.501	0.310	0.153	0.309	0.411	0.824	0.224
500	0.466	1.178	0.481	0.494	1.340	0.491	0.429	0.942	0.501
501	0.505	1.430	0.508	0.497	1.359	0.501	0.505	1.430	0.431
502	0.050	−1.485	0.512	0.275	−0.073	0.273	0.226	−0.356	0.467
503	0.497	1.380	0.508	0.506	1.415	0.501	0.497	1.380	0.508
504	0.479	1.262	0.328	0.503	1.396	0.501	0.434	0.975	0.450
505	0.505	1.430	0.501	0.512	1.453	0.512	0.468	1.194	0.262
506	0.511	1.464	0.508	0.491	1.321	0.491	0.511	1.464	0.508
507	0.505	1.430	0.513	0.512	1.453	0.508	0.450	1.076	0.409
508	0.329	0.301	0.224	0.275	−0.073	0.273	0.261	−0.137	0.492
509	0.497	1.380	0.328	0.482	1.265	0.478	0.508	1.447	0.224
510	0.497	1.380	0.262	0.500	1.378	0.501	0.408	0.807	0.262
511	0.508	1.447	0.224	0.459	1.114	0.462	0.492	1.346	0.431
512	0.224	−0.373	0.508	0.193	−0.600	0.193	0.229	−0.339	0.409
513	0.329	0.301	0.508	0.398	0.718	0.398	0.261	−0.137	0.508
514	0.263	−0.120	0.508	0.313	0.172	0.309	0.429	0.942	0.513
515	0.226	−0.356	0.097	0.275	−0.073	0.273	0.408	0.807	0.513
516	0.505	1.430	0.501	0.488	1.302	0.491	0.505	1.430	0.328
517	0.505	1.430	0.262	0.459	1.114	0.462	0.511	1.464	0.450
518	0.505	1.430	0.512	0.477	1.227	0.478	0.511	1.464	0.185
519	0.097	−1.181	0.501	0.272	−0.091	0.273	0.329	0.301	0.492
520	0.492	1.346	0.481	0.518	1.491	0.514	0.453	1.093	0.492
521	0.266	−0.103	0.512	0.193	−0.600	0.193	0.184	−0.625	0.501
522	0.513	1.481	0.513	0.462	1.133	0.462	0.489	1.329	0.431
523	0.500	1.397	0.513	0.462	1.133	0.462	0.489	1.329	0.385
524	0.482	1.279	0.142	0.424	0.888	0.422	0.503	1.413	0.358
525	0.513	1.481	0.512	0.509	1.434	0.501	0.429	0.942	0.185
526	0.511	1.464	0.467	0.520	1.509	0.514	0.387	0.672	0.467
527	0.508	1.447	0.450	0.523	1.528	0.514	0.358	0.487	0.492
528	0.142	−0.895	0.512	0.237	−0.317	0.235	0.184	−0.625	0.513
529	0.511	1.464	0.262	0.488	1.302	0.491	0.463	1.161	0.492
530	0.468	1.194	0.262	0.509	1.434	0.508	0.489	1.329	0.262
531	0.447	1.060	0.481	0.512	1.453	0.508	0.516	1.498	0.296
532	0.513	1.481	0.501	0.494	1.340	0.501	0.489	1.329	0.501
533	0.261	−0.137	0.501	0.193	−0.600	0.193	0.263	−0.120	0.358
534	0.261	−0.137	0.224	0.371	0.549	0.371	0.297	0.099	0.501
535	0.484	1.295	0.224	0.503	1.396	0.501	0.503	1.413	0.185
536	0.500	1.397	0.224	0.497	1.359	0.501	0.358	0.487	0.185
537	0.500	1.397	0.513	0.503	1.396	0.501	0.495	1.363	0.185
538	0.224	−0.373	0.508	0.196	−0.581	0.193	0.184	−0.625	0.481
539	0.224	−0.373	0.501	0.196	−0.581	0.193	0.184	−0.625	0.467
540	0.224	−0.373	0.262	0.196	−0.581	0.193	0.184	−0.625	0.450
541	0.513	1.481	0.450	0.509	1.434	0.508	0.482	1.279	0.185
542	0.505	1.430	0.501	0.488	1.302	0.491	0.463	1.161	0.481
543	0.500	1.397	0.185	0.488	1.302	0.491	0.447	1.060	0.501
544	0.263	−0.120	0.512	0.465	1.152	0.462	0.184	−0.625	0.262
545	0.458	1.127	0.409	0.465	1.152	0.462	0.476	1.245	0.512

546	0.500	1.397	0.097	0.494	1.340	0.501	0.503	1.413	0.508
547	0.184	-0.625	0.185	0.102	-1.184	0.102	0.266	-0.103	0.097
548	0.511	1.464	0.512	0.474	1.208	0.478	0.508	1.447	0.262
549	0.405	0.790	0.512	0.512	1.453	0.512	0.503	1.413	0.450
550	0.097	-1.181	0.512	0.342	0.361	0.341	0.097	-1.181	0.450
551	0.184	-0.625	0.501	0.152	-0.863	0.149	0.263	-0.120	0.450
552	0.513	1.481	0.512	0.497	1.359	0.501	0.445	1.043	0.508
553	0.513	1.481	0.431	0.497	1.359	0.501	0.445	1.043	0.501
554	0.513	1.481	0.142	0.497	1.359	0.501	0.445	1.043	0.501
555	0.503	1.413	0.512	0.494	1.340	0.501	0.508	1.447	0.185
556	0.508	1.447	0.050	0.342	0.361	0.341	0.503	1.413	0.512
557	0.426	0.925	0.501	0.512	1.453	0.514	0.497	1.380	0.185
558	0.142	-0.895	0.328	0.310	0.153	0.309	0.184	-0.625	0.224
559	0.508	1.447	0.467	0.515	1.472	0.512	0.505	1.430	0.409
560	0.050	-1.485	0.467	0.345	0.379	0.341	0.184	-0.625	0.262
561	0.000	?	0.467	0.313	0.172	0.309	0.224	-0.373	0.508
562	0.500	1.397	0.224	0.512	1.453	0.508	0.408	0.807	0.501
563	0.329	0.301	0.431	0.272	-0.091	0.273	0.263	-0.120	0.501
564	0.468	1.194	0.142	0.462	1.133	0.462	0.500	1.397	0.358
565	0.471	1.211	0.501	0.462	1.133	0.462	0.495	1.363	0.467
566	0.471	1.211	0.492	0.462	1.133	0.462	0.495	1.363	0.185
567	0.224	-0.373	0.501	0.345	0.379	0.341	0.361	0.504	0.481
568	0.432	0.958	0.142	0.503	1.396	0.501	0.466	1.178	0.508
569	0.142	-0.895	0.185	0.234	-0.336	0.235	0.184	-0.625	0.481
570	0.500	1.397	0.467	0.503	1.396	0.508	0.482	1.279	0.262
571	0.492	1.346	0.409	0.509	1.434	0.514	0.505	1.430	0.296
572	0.495	1.363	0.508	0.509	1.434	0.508	0.479	1.262	0.262
573	0.142	-0.895	0.492	0.310	0.153	0.309	0.266	-0.103	0.501
574	0.184	-0.625	0.358	0.307	0.135	0.309	0.300	0.116	0.409
575	0.468	1.194	0.467	0.275	-0.073	0.273	0.261	-0.137	0.501
576	0.408	0.807	0.050	0.465	1.152	0.462	0.500	1.397	0.224
577	0.511	1.464	0.501	0.480	1.246	0.478	0.413	0.841	0.467
578	0.492	1.346	0.501	0.459	1.114	0.462	0.495	1.363	0.142
579	0.361	0.504	0.224	0.102	-1.184	0.102	0.226	-0.356	0.501
580	0.461	1.144	0.481	0.512	1.453	0.512	0.463	1.161	0.501
581	0.050	-1.485	0.142	0.342	0.361	0.341	0.142	-0.895	0.142
582	0.503	1.413	0.512	0.447	1.039	0.444	0.503	1.413	0.481
583	0.503	1.413	0.513	0.482	1.265	0.478	0.503	1.413	0.224
584	0.224	-0.373	0.481	0.307	0.135	0.309	0.142	-0.895	0.481
585	0.476	1.245	0.481	0.494	1.340	0.491	0.482	1.279	0.512
586	0.142	-0.895	0.262	0.348	0.398	0.341	0.224	-0.373	0.492
587	0.511	1.464	0.185	0.503	1.396	0.508	0.476	1.245	0.501
588	0.508	1.447	0.185	0.480	1.246	0.478	0.508	1.447	0.185
589	0.484	1.295	0.185	0.515	1.472	0.512	0.497	1.380	0.097
590	0.482	1.279	0.481	0.395	0.700	0.398	0.503	1.413	0.097
591	0.263	-0.120	0.328	0.102	-1.184	0.102	0.187	-0.608	0.097
592	0.184	-0.625	0.328	0.272	-0.091	0.273	0.097	-1.181	0.450
593	0.184	-0.625	0.431	0.272	-0.091	0.273	0.097	-1.181	0.185
594	0.184	-0.625	0.296	0.272	-0.091	0.273	0.097	-1.181	0.185
595	0.479	1.262	0.508	0.503	1.396	0.508	0.447	1.060	0.481
596	0.329	0.301	0.142	0.275	-0.073	0.273	0.184	-0.625	0.262
597	0.329	0.301	0.142	0.275	-0.073	0.273	0.184	-0.625	0.492
598	0.432	0.958	0.513	0.509	1.434	0.501	0.479	1.262	0.262
599	0.297	0.099	0.513	0.278	-0.054	0.273	0.261	-0.137	0.224
600	0.508	1.447	0.513	0.503	1.396	0.508	0.492	1.346	0.492
601	0.145	-0.878	0.513	0.193	-0.600	0.193	0.263	-0.120	0.481
602	0.142	-0.895	0.262	0.237	-0.317	0.235	0.224	-0.373	0.481
603	0.511	1.464	0.492	0.442	1.001	0.444	0.495	1.363	0.481
604	0.513	1.481	0.262	0.339	0.342	0.341	0.484	1.295	0.328
605	0.513	1.481	0.296	0.339	0.342	0.341	0.484	1.295	0.467
606	0.513	1.481	0.224	0.339	0.342	0.341	0.484	1.295	0.358

607	0.266	-0.103	0.185	0.456	1.095	0.462	0.326	0.285	0.185
608	0.492	1.346	0.431	0.421	0.869	0.422	0.461	1.144	0.296
609	0.263	-0.120	0.508	0.196	-0.581	0.193	0.358	0.487	0.328
610	0.295	0.082	0.508	0.275	-0.073	0.273	0.187	-0.608	0.512
611	0.226	-0.356	0.358	0.234	-0.336	0.235	0.297	0.099	0.512
612	0.187	-0.608	0.481	0.234	-0.336	0.235	0.332	0.318	0.512
613	0.429	0.942	0.513	0.398	0.718	0.398	0.508	1.447	0.185
614	0.505	1.430	0.224	0.500	1.378	0.501	0.516	1.498	0.481
615	0.505	1.430	0.450	0.500	1.378	0.501	0.516	1.498	0.508
616	0.358	0.487	0.142	0.374	0.568	0.371	0.187	-0.608	0.409
617	0.474	1.228	0.185	0.480	1.246	0.478	0.479	1.262	0.492
618	0.511	1.464	0.492	0.421	0.869	0.422	0.505	1.430	0.296
619	0.226	-0.356	0.513	0.053	-1.504	0.053	0.411	0.824	0.328
620	0.455	1.110	0.142	0.477	1.227	0.478	0.495	1.363	0.467
621	0.142	-0.895	0.467	0.310	0.153	0.309	0.297	0.099	0.512
622	0.187	-0.608	0.512	0.149	-0.882	0.149	0.329	0.301	0.097
623	0.497	1.380	0.185	0.491	1.321	0.491	0.468	1.194	0.512
624	0.511	1.464	0.097	0.506	1.415	0.508	0.511	1.464	0.513
625	0.142	-0.895	0.481	0.313	0.172	0.309	0.097	-1.181	0.185
626	0.461	1.144	0.481	0.506	1.415	0.508	0.511	1.464	0.296
627	0.508	1.447	0.512	0.477	1.227	0.478	0.511	1.464	0.512
628	0.184	-0.625	0.508	0.193	-0.600	0.193	0.189	-0.592	0.513
629	0.097	-1.181	0.492	0.149	-0.882	0.149	0.300	0.116	0.501
630	0.476	1.245	0.513	0.465	1.152	0.462	0.513	1.481	0.501
631	0.476	1.245	0.097	0.480	1.246	0.478	0.513	1.481	0.508
632	0.500	1.397	0.050	0.491	1.321	0.491	0.505	1.430	0.492
633	0.497	1.380	0.467	0.500	1.378	0.501	0.505	1.430	0.097
634	0.489	1.329	0.508	0.506	1.415	0.508	0.505	1.430	0.097
635	0.511	1.464	0.501	0.515	1.472	0.514	0.492	1.346	0.501
636	0.097	-1.181	0.492	0.374	0.568	0.371	0.097	-1.181	0.501
637	0.050	-1.485	0.296	0.401	0.737	0.398	0.097	-1.181	0.492
638	0.466	1.178	0.224	0.518	1.491	0.514	0.505	1.430	0.508
639	0.505	1.430	0.185	0.477	1.227	0.478	0.500	1.397	0.431
640	0.500	1.397	0.481	0.477	1.227	0.478	0.487	1.312	0.385
641	0.489	1.329	0.050	0.506	1.415	0.508	0.505	1.430	0.385
642	0.297	0.099	0.492	0.310	0.153	0.309	0.432	0.958	0.481
643	0.224	-0.373	0.501	0.234	-0.336	0.235	0.387	0.672	0.224
644	0.184	-0.625	0.508	0.313	0.172	0.309	0.387	0.672	0.513
645	0.482	1.279	0.512	0.515	1.472	0.512	0.482	1.279	0.467
646	0.050	-1.485	0.185	0.275	-0.073	0.273	0.226	-0.356	0.467
647	0.492	1.346	0.358	0.462	1.133	0.462	0.511	1.464	0.481
648	0.500	1.397	0.467	0.485	1.283	0.491	0.466	1.178	0.050
649	0.505	1.430	0.296	0.485	1.283	0.491	0.466	1.178	0.097
650	0.505	1.430	0.185	0.477	1.227	0.478	0.482	1.279	0.409
651	0.184	-0.625	0.450	0.307	0.135	0.309	0.050	-1.485	0.296
652	0.358	0.487	0.512	0.418	0.850	0.422	0.097	-1.181	0.142
653	0.463	1.161	0.409	0.497	1.359	0.501	0.408	0.807	0.431
654	0.297	0.099	0.501	0.193	-0.600	0.193	0.297	0.099	0.492
655	0.184	-0.625	0.501	0.272	-0.091	0.273	0.142	-0.895	0.512
656	0.447	1.060	0.385	0.515	1.472	0.512	0.429	0.942	0.512
657	0.511	1.464	0.409	0.509	1.434	0.512	0.492	1.346	0.450
658	0.411	0.824	0.508	0.500	1.378	0.501	0.508	1.447	0.508
659	0.497	1.380	0.358	0.512	1.453	0.512	0.508	1.447	0.513
660	0.500	1.397	0.450	0.491	1.321	0.491	0.455	1.110	0.512
661	0.387	0.672	0.481	0.462	1.133	0.462	0.508	1.447	0.224
662	0.413	0.841	0.481	0.494	1.340	0.491	0.511	1.464	0.512
663	0.500	1.397	0.481	0.500	1.378	0.501	0.516	1.498	0.492
664	0.358	0.487	0.481	0.237	-0.317	0.235	0.224	-0.373	0.492
665	0.450	1.076	0.224	0.456	1.095	0.462	0.513	1.481	0.492
666	0.479	1.262	0.450	0.488	1.302	0.491	0.489	1.329	0.492
667	0.479	1.262	0.508	0.488	1.302	0.491	0.489	1.329	0.097

668	0.479	1.262	0.501	0.462	1.133	0.462	0.489	1.329	0.512
669	0.479	1.262	0.450	0.477	1.227	0.478	0.489	1.329	0.450
670	0.226	-0.356	0.512	0.342	0.361	0.341	0.097	-1.181	0.513
671	0.453	1.093	0.508	0.462	1.133	0.462	0.503	1.413	0.508
672	0.511	1.464	0.508	0.497	1.359	0.501	0.455	1.110	0.467
673	0.500	1.397	0.492	0.462	1.133	0.462	0.511	1.464	0.492
674	0.447	1.060	0.512	0.503	1.396	0.501	0.511	1.464	0.501
675	0.511	1.464	0.512	0.506	1.415	0.512	0.466	1.178	0.512
676	0.505	1.430	0.508	0.509	1.434	0.512	0.492	1.346	0.492
677	0.503	1.413	0.508	0.506	1.415	0.512	0.503	1.413	0.492
678	0.484	1.295	0.513	0.482	1.265	0.478	0.516	1.498	0.513
679	0.508	1.447	0.508	0.456	1.095	0.462	0.492	1.346	0.508
680	0.508	1.447	0.508	0.456	1.095	0.462	0.492	1.346	0.492
681	0.497	1.380	0.508	0.512	1.453	0.508	0.518	1.514	0.492
682	0.500	1.397	0.097	0.462	1.133	0.462	0.511	1.464	0.492
683	0.511	1.464	0.467	0.509	1.434	0.512	0.492	1.346	0.492
684	0.505	1.430	0.513	0.509	1.434	0.514	0.492	1.346	0.224
685	0.505	1.430	0.513	0.509	1.434	0.514	0.492	1.346	0.501
686	0.505	1.430	0.142	0.509	1.434	0.514	0.492	1.346	0.467
687	0.100	-1.164	0.467	0.275	-0.073	0.273	0.224	-0.373	0.467
688	0.466	1.178	0.501	0.506	1.415	0.508	0.497	1.380	0.262
689	0.508	1.447	0.450	0.512	1.453	0.512	0.466	1.178	0.467
690	0.508	1.447	0.185	0.515	1.472	0.514	0.466	1.178	0.501
691	0.142	-0.895	0.142	0.234	-0.336	0.235	0.266	-0.103	0.508
692	0.468	1.194	0.142	0.515	1.472	0.512	0.466	1.178	0.262
693	0.497	1.380	0.262	0.424	0.888	0.422	0.500	1.397	0.358
694	0.445	1.043	0.097	0.462	1.133	0.462	0.508	1.447	0.358
695	0.187	-0.608	0.097	0.149	-0.882	0.149	0.263	-0.120	0.097
696	0.142	-0.895	0.097	0.152	-0.863	0.149	0.358	0.487	0.185
697	0.142	-0.895	0.296	0.152	-0.863	0.149	0.358	0.487	0.185
698	0.263	-0.120	0.262	0.193	-0.600	0.193	0.097	-1.181	0.185
699	0.097	-1.181	0.508	0.272	-0.091	0.273	0.184	-0.625	0.358
700	0.097	-1.181	0.513	0.272	-0.091	0.273	0.184	-0.625	0.328
701	0.097	-1.181	0.512	0.272	-0.091	0.273	0.184	-0.625	0.501
702	0.295	0.082	0.508	0.240	-0.299	0.235	0.358	0.487	0.492
703	0.261	-0.137	0.142	0.152	-0.863	0.149	0.326	0.285	0.467
704	0.505	1.430	0.224	0.500	1.378	0.501	0.497	1.380	0.409
705	0.508	1.447	0.142	0.520	1.509	0.514	0.489	1.329	0.262
706	0.513	1.481	0.097	0.518	1.491	0.514	0.466	1.178	0.262
707	0.503	1.413	0.185	0.462	1.133	0.462	0.411	0.824	0.328
708	0.145	-0.878	0.142	0.149	-0.882	0.149	0.263	-0.120	0.224
709	0.226	-0.356	0.467	0.237	-0.317	0.235	0.261	-0.137	0.328
710	0.142	-0.895	0.431	0.102	-1.184	0.102	0.332	0.318	0.262
711	0.097	-1.181	0.431	0.237	-0.317	0.235	0.224	-0.373	0.431
712	0.184	-0.625	0.224	0.307	0.135	0.309	0.332	0.318	0.508
713	0.145	-0.878	0.512	0.307	0.135	0.309	0.261	-0.137	0.508
714	0.466	1.178	0.328	0.459	1.114	0.462	0.432	0.958	0.224
715	0.432	0.958	0.296	0.488	1.302	0.491	0.513	1.481	0.481
716	0.432	0.958	0.409	0.488	1.302	0.491	0.513	1.481	0.358
717	0.226	-0.356	0.512	0.193	-0.600	0.193	0.224	-0.373	0.142
718	0.505	1.430	0.328	0.512	1.453	0.512	0.479	1.262	0.142
719	0.329	0.301	0.296	0.307	0.135	0.309	0.355	0.470	0.358
720	0.297	0.099	0.492	0.275	-0.073	0.273	0.142	-0.895	0.097
721	0.411	0.824	0.501	0.401	0.737	0.398	0.142	-0.895	0.185
722	0.508	1.447	0.224	0.462	1.133	0.462	0.361	0.504	0.467
723	0.326	0.285	0.097	0.102	-1.184	0.102	0.097	-1.181	0.512
724	0.295	0.082	0.185	0.149	-0.882	0.149	0.184	-0.625	0.224
725	0.495	1.363	0.431	0.480	1.246	0.478	0.463	1.161	0.328
726	0.497	1.380	0.142	0.477	1.227	0.478	0.508	1.447	0.097
727	0.226	-0.356	0.508	0.234	-0.336	0.235	0.226	-0.356	0.481
728	0.097	-1.181	0.512	0.310	0.153	0.309	0.329	0.301	0.142

729	0.187	-0.608	0.512	0.275	-0.073	0.273	0.097	-1.181	0.501
730	0.432	0.958	0.097	0.512	1.453	0.512	0.479	1.262	0.501
731	0.142	-0.895	0.142	0.374	0.568	0.371	0.142	-0.895	0.501
732	0.505	1.430	0.050	0.512	1.453	0.514	0.497	1.380	0.262
733	0.508	1.447	0.512	0.512	1.453	0.514	0.497	1.380	0.358
734	0.508	1.447	0.050	0.512	1.453	0.514	0.497	1.380	0.185
735	0.097	-1.181	0.262	0.427	0.907	0.422	0.261	-0.137	0.481
736	0.142	-0.895	0.097	0.196	-0.581	0.193	0.361	0.504	0.050
737	0.050	-1.485	0.508	0.313	0.172	0.309	0.184	-0.625	0.050
738	0.503	1.413	0.508	0.509	1.434	0.508	0.479	1.262	0.328
739	0.000	?	0.501	0.424	0.888	0.422	0.050	-1.485	0.185
740	0.050	-1.485	0.050	0.401	0.737	0.398	0.050	-1.485	0.512
741	0.263	-0.120	0.512	0.102	-1.184	0.102	0.329	0.301	0.512
742	0.097	-1.181	0.492	0.275	-0.073	0.273	0.184	-0.625	0.508
743	0.505	1.430	0.142	0.512	1.453	0.514	0.511	1.464	0.328
744	0.505	1.430	0.097	0.512	1.453	0.514	0.511	1.464	0.467
745	0.492	1.346	0.508	0.515	1.472	0.512	0.508	1.447	0.328
746	0.050	-1.485	0.508	0.149	-0.882	0.149	0.329	0.301	0.508
747	0.511	1.464	0.481	0.506	1.415	0.501	0.466	1.178	0.185
748	0.000	?	0.481	0.193	-0.600	0.193	0.326	0.285	0.328
749	0.484	1.295	0.508	0.515	1.472	0.514	0.511	1.464	0.508
750	0.142	-0.895	0.262	0.234	-0.336	0.235	0.187	-0.608	0.508
751	0.100	-1.164	0.508	0.237	-0.317	0.235	0.326	0.285	0.508
752	0.503	1.413	0.508	0.500	1.378	0.501	0.508	1.447	0.508
753	0.503	1.413	0.492	0.500	1.378	0.501	0.508	1.447	0.512
754	0.482	1.279	0.262	0.506	1.415	0.508	0.505	1.430	0.385
755	0.482	1.279	0.185	0.488	1.302	0.491	0.505	1.430	0.296
756	0.508	1.447	0.450	0.444	1.020	0.444	0.508	1.447	0.431
757	0.263	-0.120	0.224	0.307	0.135	0.309	0.389	0.689	0.501
758	0.505	1.430	0.467	0.512	1.453	0.514	0.297	0.099	0.224
759	0.505	1.430	0.501	0.503	1.396	0.508	0.432	0.958	0.358
760	0.492	1.346	0.512	0.491	1.321	0.491	0.497	1.380	0.262
761	0.261	-0.137	0.296	0.374	0.568	0.371	0.226	-0.356	0.185
762	0.184	-0.625	0.262	0.342	0.361	0.341	0.355	0.470	0.512
763	0.455	1.110	0.501	0.307	0.135	0.309	0.263	-0.120	0.492
764	0.224	-0.373	0.142	0.152	-0.863	0.149	0.184	-0.625	0.450
765	0.466	1.178	0.501	0.494	1.340	0.491	0.508	1.447	0.296
766	0.497	1.380	0.262	0.442	1.001	0.444	0.489	1.329	0.224
767	0.513	1.481	0.409	0.459	1.114	0.462	0.447	1.060	0.501
768	0.297	0.099	0.467	0.193	-0.600	0.193	0.295	0.082	0.224
769	0.261	-0.137	0.385	0.149	-0.882	0.149	0.224	-0.373	0.492
770	0.503	1.413	0.492	0.520	1.509	0.514	0.500	1.397	0.185
771	0.142	-0.895	0.185	0.377	0.587	0.371	0.224	-0.373	0.508
772	0.495	1.363	0.512	0.512	1.453	0.512	0.489	1.329	0.501
773	0.261	-0.137	0.512	0.310	0.153	0.309	0.184	-0.625	0.142
774	0.405	0.790	0.467	0.491	1.321	0.491	0.505	1.430	0.508
775	0.471	1.211	0.142	0.503	1.396	0.501	0.503	1.413	0.185
776	0.384	0.655	0.328	0.468	1.170	0.462	0.142	-0.895	0.431
777	0.492	1.346	0.492	0.465	1.152	0.462	0.511	1.464	0.431
778	0.184	-0.625	0.492	0.275	-0.073	0.273	0.187	-0.608	0.501
779	0.508	1.447	0.513	0.488	1.302	0.491	0.426	0.925	0.358
780	0.508	1.447	0.481	0.488	1.302	0.491	0.426	0.925	0.185
781	0.466	1.178	0.224	0.368	0.530	0.371	0.497	1.380	0.450
782	0.142	-0.895	0.262	0.234	-0.336	0.235	0.361	0.504	0.467
783	0.329	0.301	0.185	0.000	?	0.444	0.187	-0.608	0.467
784	0.492	1.346	0.501	0.450	1.057	0.512	0.453	1.093	0.492
785	0.492	1.346	0.501	0.520	1.509	0.462	0.466	1.178	0.328
786	0.508	1.447	0.508	0.462	1.133	0.512	0.466	1.178	0.185
787	0.484	1.295	0.512	0.515	1.472	0.371	0.492	1.346	0.185
788	0.224	-0.373	0.508	0.374	0.568	0.273	0.332	0.318	0.481
789	0.263	-0.120	0.508	0.275	-0.073	0.235	0.187	-0.608	0.481

790	0.187	-0.608	0.508	0.234	-0.336	0.491	0.187	-0.608	0.481
791	0.495	1.363	0.185	0.491	1.321	0.491	0.479	1.262	0.512
792	0.495	1.363	0.508	0.491	1.321	0.491	0.479	1.262	0.431
793	0.503	1.413	0.508	0.491	1.321	0.444	0.479	1.262	0.513
794	0.500	1.397	0.508	0.447	1.039	0.508	0.511	1.464	0.508
795	0.497	1.380	0.512	0.509	1.434	0.501	0.432	0.958	0.142
796	0.513	1.481	0.385	0.500	1.378	0.491	0.505	1.430	0.481
797	0.513	1.481	0.097	0.491	1.321	0.309	0.503	1.413	0.450
798	0.184	-0.625	0.508	0.313	0.172	0.501	0.142	-0.895	0.481
799	0.497	1.380	0.513	0.506	1.415	0.501	0.487	1.312	0.328
800	0.497	1.380	0.513	0.506	1.415	0.501	0.455	1.110	0.501
801	0.497	1.380	0.050	0.506	1.415	0.193	0.487	1.312	0.142
802	0.000	?	0.050	0.193	-0.600	0.444	0.332	0.318	0.224
803	0.503	1.413	0.492	0.442	1.001	0.149	0.503	1.413	0.492
804	0.384	0.655	0.501	0.149	-0.882	0.235	0.142	-0.895	0.467
805	0.097	-1.181	0.512	0.240	-0.299	0.514	0.224	-0.373	0.467
806	0.508	1.447	0.492	0.515	1.472	0.508	0.495	1.363	0.224
807	0.513	1.481	0.450	0.506	1.415	0.508	0.474	1.228	0.224
808	0.513	1.481	0.450	0.506	1.415	0.273	0.474	1.228	0.481
809	0.050	-1.485	0.512	0.278	-0.054	0.309	0.224	-0.373	0.467
810	0.050	-1.485	0.224	0.313	0.172	0.491	0.224	-0.373	0.492
811	0.492	1.346	0.513	0.488	1.302	0.398	0.474	1.228	0.513
812	0.503	1.413	0.512	0.398	0.718	0.491	0.461	1.144	0.512
813	0.505	1.430	0.508	0.494	1.340	0.512	0.489	1.329	0.512
814	0.487	1.312	0.296	0.512	1.453	0.512	0.518	1.514	0.450
815	0.453	1.093	0.450	0.509	1.434	0.508	0.508	1.447	0.224
816	0.453	1.093	0.185	0.503	1.396	0.491	0.508	1.447	0.501
817	0.511	1.464	0.262	0.494	1.340	0.309	0.453	1.093	0.492
818	0.224	-0.373	0.512	0.307	0.135	0.512	0.224	-0.373	0.481
819	0.518	1.514	0.481	0.503	1.396	0.501	0.495	1.363	0.262
820	0.503	1.413	0.501	0.509	1.434	0.514	0.495	1.363	0.467
821	0.497	1.380	0.224	0.518	1.491	0.235	0.482	1.279	0.097
822	0.300	0.116	0.185	0.234	-0.336	0.512	0.263	-0.120	0.142
823	0.450	1.076	0.296	0.515	1.472	0.273	0.466	1.178	0.492
824	0.184	-0.625	0.262	0.275	-0.073	0.149	0.097	-1.181	0.508
825	0.263	-0.120	0.501	0.152	-0.863	0.508	0.142	-0.895	0.513
826	0.511	1.464	0.512	0.506	1.415	0.501	0.492	1.346	0.385
827	0.479	1.262	0.262	0.500	1.378	0.501	0.505	1.430	0.224
828	0.500	1.397	0.224	0.497	1.359	0.149	0.511	1.464	0.142
829	0.226	-0.356	0.050	0.149	-0.882	0.149	0.387	0.672	0.385
830	0.187	-0.608	0.481	0.152	-0.863	0.235	0.224	-0.373	0.492
831	0.295	0.082	0.097	0.234	-0.336	0.149	0.145	-0.878	0.492
832	0.261	-0.137	0.512	0.149	-0.882	0.512	0.384	0.655	0.185
833	0.497	1.380	0.097	0.512	1.453	0.508	0.489	1.329	0.328
834	0.508	1.447	0.508	0.506	1.415	0.149	0.489	1.329	0.385
835	0.263	-0.120	0.492	0.149	-0.882	0.273	0.184	-0.625	0.467
836	0.224	-0.373	0.185	0.275	-0.073	0.235	0.329	0.301	0.328
837	0.050	-1.485	0.450	0.237	-0.317	0.514	0.382	0.638	0.492
838	0.484	1.295	0.358	0.518	1.491	0.273	0.468	1.194	0.358
839	0.097	-1.181	0.262	0.275	-0.073	0.501	0.332	0.318	0.492
840	0.500	1.397	0.508	0.503	1.396	0.273	0.495	1.363	0.508
841	0.097	-1.181	0.513	0.275	-0.073	0.512	0.361	0.504	0.142
842	0.497	1.380	0.501	0.520	1.509	0.491	0.495	1.363	0.512
843	0.495	1.363	0.501	0.485	1.283	0.341	0.505	1.430	0.224
844	0.187	-0.608	0.501	0.342	0.361	0.491	0.142	-0.895	0.512
845	0.453	1.093	0.501	0.491	1.321	0.273	0.513	1.481	0.512
846	0.358	0.487	0.501	0.275	-0.073	0.309	0.224	-0.373	0.513
847	0.261	-0.137	0.467	0.313	0.172	0.398	0.000	?	0.513
848	0.511	1.464	0.481	0.395	0.700	0.398	0.511	1.464	0.513
849	0.516	1.498	0.450	0.395	0.700	0.462	0.511	1.464	0.513
850	0.503	1.413	0.513	0.462	1.133	0.478	0.513	1.481	0.513

851	0.503	1.413	0.185	0.474	1.208	0.478	0.513	1.481	0.508
852	0.503	1.413	0.508	0.477	1.227	0.478	0.513	1.481	0.501
853	0.503	1.413	0.431	0.477	1.227	0.512	0.513	1.481	0.508
854	0.497	1.380	0.431	0.509	1.434	0.508	0.513	1.481	0.513
855	0.466	1.178	0.296	0.509	1.434	0.491	0.505	1.430	0.262
856	0.476	1.245	0.512	0.485	1.283	0.444	0.500	1.397	0.512
857	0.447	1.060	0.481	0.444	1.020	0.501	0.508	1.447	0.512
858	0.511	1.464	0.512	0.497	1.359	0.102	0.516	1.498	0.512
859	0.184	-0.625	0.512	0.102	-1.184	0.501	0.263	-0.120	0.185
860	0.503	1.413	0.508	0.500	1.378	0.508	0.508	1.447	0.513
861	0.429	0.942	0.508	0.506	1.415	0.508	0.511	1.464	0.508
862	0.429	0.942	0.501	0.506	1.415	0.149	0.511	1.464	0.513
863	0.297	0.099	0.224	0.152	-0.863	0.462	0.184	-0.625	0.508
864	0.511	1.464	0.467	0.459	1.114	0.514	0.513	1.481	0.513
865	0.476	1.245	0.467	0.509	1.434	0.462	0.508	1.447	0.513
866	0.511	1.464	0.296	0.459	1.114	0.462	0.513	1.481	0.501
867	0.511	1.464	0.481	0.459	1.114	0.512	0.508	1.447	0.142
868	0.505	1.430	0.431	0.506	1.415	0.512	0.513	1.481	0.431
869	0.505	1.430	0.481	0.506	1.415	0.512	0.513	1.481	0.431
870	0.500	1.397	0.481	0.512	1.453	0.309	0.500	1.397	0.328
871	0.226	-0.356	0.508	0.307	0.135	0.491	0.142	-0.895	0.481
872	0.468	1.194	0.467	0.491	1.321	0.491	0.432	0.958	0.467
873	0.468	1.194	0.508	0.491	1.321	0.341	0.432	0.958	0.481
874	0.300	0.116	0.508	0.342	0.361	0.512	0.326	0.285	0.481
875	0.479	1.262	0.224	0.506	1.415	0.512	0.482	1.279	0.467
876	0.432	0.958	0.185	0.506	1.415	0.478	0.468	1.194	0.501
877	0.479	1.262	0.492	0.482	1.265	0.478	0.484	1.295	0.492
878	0.479	1.262	0.501	0.480	1.246	0.422	0.484	1.295	0.508
879	0.505	1.430	0.142	0.430	0.926	0.501	0.471	1.211	0.185
880	0.463	1.161	0.450	0.500	1.378	0.462	0.497	1.380	0.185
881	0.508	1.447	0.512	0.462	1.133	0.514	0.497	1.380	0.512
882	0.511	1.464	0.492	0.515	1.472	0.193	0.511	1.464	0.512
883	0.224	-0.373	0.513	0.196	-0.581	0.193	0.187	-0.608	0.050
884	0.184	-0.625	0.508	0.196	-0.581	0.501	0.187	-0.608	0.513
885	0.492	1.346	0.501	0.503	1.396	0.422	0.516	1.498	0.508
886	0.497	1.380	0.450	0.424	0.888	0.341	0.516	1.498	0.513
887	0.142	-0.895	0.501	0.342	0.361	0.501	0.050	-1.485	0.508
888	0.450	1.076	0.492	0.500	1.378	0.501	0.511	1.464	0.492
889	0.511	1.464	0.481	0.497	1.359	0.512	0.505	1.430	0.431
890	0.489	1.329	0.501	0.509	1.434	0.501	0.511	1.464	0.513
891	0.511	1.464	0.224	0.497	1.359	0.491	0.505	1.430	0.481
892	0.505	1.430	0.224	0.494	1.340	0.501	0.492	1.346	0.501
893	0.500	1.397	0.467	0.500	1.378	0.462	0.426	0.925	0.492
894	0.447	1.060	0.492	0.462	1.133	0.478	0.513	1.481	0.501
895	0.495	1.363	0.492	0.477	1.227	0.491	0.479	1.262	0.224
896	0.492	1.346	0.328	0.491	1.321	0.478	0.503	1.413	0.097
897	0.476	1.245	0.467	0.477	1.227	0.398	0.495	1.363	0.409
898	0.495	1.363	0.224	0.401	0.737	0.371	0.497	1.380	0.409
899	0.224	-0.373	0.508	0.371	0.549	0.273	0.226	-0.356	0.385
900	0.226	-0.356	0.508	0.275	-0.073	0.444	0.097	-1.181	0.296
901	0.471	1.211	0.501	0.444	1.020	0.501	0.411	0.824	0.512
902	0.489	1.329	0.481	0.497	1.359	0.422	0.408	0.807	0.450
903	0.492	1.346	0.512	0.427	0.907	0.273	0.389	0.689	0.512
904	0.329	0.301	0.512	0.272	-0.091	0.491	0.303	0.133	0.508
905	0.463	1.161	0.512	0.491	1.321	0.309	0.513	1.481	0.508
906	0.224	-0.373	0.512	0.313	0.172	0.444	0.000	?	0.450
907	0.511	1.464	0.097	0.442	1.001	0.478	0.450	1.076	0.409
908	0.497	1.380	0.224	0.480	1.246	0.512	0.511	1.464	0.409
909	0.503	1.413	0.358	0.512	1.453	0.478	0.505	1.430	0.492
910	0.479	1.262	0.328	0.480	1.246	0.491	0.511	1.464	0.262
911	0.511	1.464	0.097	0.491	1.321	0.462	0.450	1.076	0.262

912	0.511	1.464	0.224	0.462	1.133	0.462	0.411	0.824	0.262
913	0.511	1.464	0.224	0.462	1.133	0.462	0.411	0.824	0.262
914	0.503	1.413	0.296	0.465	1.152	0.193	0.492	1.346	0.142
915	0.097	−1.181	0.501	0.193	−0.600	0.309	0.261	−0.137	0.142
916	0.226	−0.356	0.224	0.307	0.135	0.193	0.000	?	0.358
917	0.363	0.520	0.450	0.193	−0.600	0.371	0.261	−0.137	0.481
918	0.329	0.301	0.467	0.371	0.549	0.309	0.261	−0.137	0.224
919	0.097	−1.181	0.481	0.310	0.153	0.193	0.261	−0.137	0.481
920	0.224	−0.373	0.492	0.193	−0.600	0.193	0.142	−0.895	0.492
921	0.224	−0.373	0.467	0.193	−0.600	0.341	0.142	−0.895	0.508
922	0.297	0.099	0.492	0.342	0.361	0.512	0.358	0.487	0.501
923	0.495	1.363	0.185	0.512	1.453	0.149	0.479	1.262	0.450
924	0.224	−0.373	0.185	0.152	−0.863	0.491	0.226	−0.356	0.512
925	0.445	1.043	0.262	0.488	1.302	0.508	0.479	1.262	0.224
926	0.461	1.144	0.481	0.506	1.415	0.514	0.492	1.346	0.224
927	0.479	1.262	0.513	0.509	1.434	0.514	0.508	1.447	0.224
928	0.489	1.329	0.501	0.509	1.434	0.478	0.503	1.413	0.501
929	0.461	1.144	0.501	0.477	1.227	0.491	0.453	1.093	0.431
930	0.487	1.312	0.481	0.494	1.340	0.235	0.513	1.481	0.508
931	0.184	−0.625	0.185	0.237	−0.317	0.235	0.224	−0.373	0.508
932	0.184	−0.625	0.467	0.237	−0.317	0.193	0.224	−0.373	0.450
933	0.261	−0.137	0.467	0.193	−0.600	0.444	0.224	−0.373	0.142
934	0.482	1.279	0.328	0.444	1.020	0.501	0.497	1.380	0.185
935	0.511	1.464	0.513	0.497	1.359	0.512	0.426	0.925	0.185
936	0.500	1.397	0.508	0.512	1.453	0.512	0.508	1.447	0.097
937	0.500	1.397	0.512	0.512	1.453	0.501	0.508	1.447	0.501
938	0.476	1.245	0.508	0.500	1.378	0.309	0.450	1.076	0.508
939	0.184	−0.625	0.512	0.313	0.172	0.273	0.142	−0.895	0.450
940	0.463	1.161	0.512	0.272	−0.091	0.273	0.184	−0.625	0.467
941	0.463	1.161	0.512	0.272	−0.091	0.149	0.184	−0.625	0.512
942	0.326	0.285	0.512	0.149	−0.882	0.422	0.097	−1.181	0.512
943	0.505	1.430	0.142	0.424	0.888	0.512	0.503	1.413	0.512
944	0.503	1.413	0.142	0.509	1.434	0.512	0.508	1.447	0.512
945	0.511	1.464	0.508	0.503	1.396	0.512	0.453	1.093	0.224
946	0.511	1.464	0.224	0.515	1.472	0.491	0.463	1.161	0.409
947	0.508	1.447	0.501	0.485	1.283	0.478	0.511	1.464	0.224
948	0.508	1.447	0.512	0.474	1.208	0.478	0.513	1.481	0.224
949	0.508	1.447	0.513	0.474	1.208	0.478	0.513	1.481	0.492
950	0.500	1.397	0.508	0.482	1.265	0.309	0.511	1.464	0.097
951	0.000	?	0.185	0.310	0.153	0.371	0.224	−0.373	0.492
952	0.000	?	0.513	0.374	0.568	0.235	0.408	0.807	0.467
953	0.142	−0.895	0.185	0.240	−0.299	0.235	0.224	−0.373	0.431
954	0.142	−0.895	0.385	0.240	−0.299	0.512	0.224	−0.373	0.492
955	0.497	1.380	0.508	0.518	1.491	0.235	0.489	1.329	0.224
956	0.224	−0.373	0.142	0.234	−0.336	0.371	0.097	−1.181	0.492
957	0.503	1.413	0.467	0.371	0.549	0.444	0.492	1.346	0.224
958	0.500	1.397	0.142	0.450	1.057	0.512	0.466	1.178	0.508
959	0.505	1.430	0.224	0.515	1.472	0.512	0.429	0.942	0.508
960	0.500	1.397	0.492	0.515	1.472	0.341	0.489	1.329	0.328
961	0.187	−0.608	0.185	0.342	0.361	0.462	0.226	−0.356	0.501
962	0.511	1.464	0.481	0.465	1.152	0.309	0.492	1.346	0.296
963	0.187	−0.608	0.492	0.310	0.153	0.508	0.226	−0.356	0.185
964	0.384	0.655	0.142	0.506	1.415	0.508	0.511	1.464	0.501
965	0.503	1.413	0.513	0.506	1.415	0.309	0.505	1.430	0.262
966	0.145	−0.878	0.481	0.310	0.153	0.508	0.329	0.301	0.481
967	0.466	1.178	0.492	0.506	1.415	0.235	0.503	1.413	0.501
968	0.142	−0.895	0.492	0.237	−0.317	0.149	0.297	0.099	0.050
969	0.224	−0.373	0.185	0.149	−0.882	0.444	0.187	−0.608	0.358
970	0.492	1.346	0.142	0.442	1.001	0.193	0.500	1.397	0.431
971	0.184	−0.625	0.097	0.193	−0.600	0.422	0.263	−0.120	0.492
972	0.476	1.245	0.097	0.418	0.850	0.508	0.482	1.279	0.492

973	0.489	1.329	0.508	0.506	1.415	0.341	0.503	1.413	0.328
974	0.142	-0.895	0.142	0.345	0.379	0.508	0.050	-1.485	0.328
975	0.500	1.397	0.262	0.509	1.434	0.491	0.358	0.487	0.097
976	0.482	1.279	0.501	0.497	1.359	0.462	0.429	0.942	0.097
977	0.487	1.312	0.501	0.459	1.114	0.462	0.495	1.363	0.492
978	0.487	1.312	0.467	0.459	1.114	0.235	0.495	1.363	0.142
979	0.184	-0.625	0.512	0.234	-0.336	0.149	0.329	0.301	0.185
980	0.142	-0.895	0.512	0.149	-0.882	0.341	0.329	0.301	0.450
981	0.097	-1.181	0.262	0.345	0.379	0.341	0.097	-1.181	0.450
982	0.097	-1.181	0.224	0.345	0.379	0.462	0.097	-1.181	0.512
983	0.505	1.430	0.492	0.465	1.152	0.273	0.487	1.312	0.450
984	0.145	-0.878	0.431	0.275	-0.073	0.149	0.142	-0.895	0.409
985	0.263	-0.120	0.224	0.149	-0.882	0.512	0.187	-0.608	0.185
986	0.505	1.430	0.481	0.515	1.472	0.508	0.450	1.076	0.185
987	0.505	1.430	0.409	0.509	1.434	0.462	0.450	1.076	0.492
988	0.463	1.161	0.097	0.462	1.133	0.508	0.508	1.447	0.467
989	0.503	1.413	0.097	0.506	1.415	0.512	0.450	1.076	0.224
990	0.503	1.413	0.385	0.518	1.491	0.149	0.413	0.841	0.492
991	0.263	-0.120	0.385	0.152	-0.863	0.193	0.184	-0.625	0.467
992	0.226	-0.356	0.385	0.196	-0.581	0.462	0.184	-0.625	0.409
993	0.492	1.346	0.224	0.459	1.114	0.444	0.489	1.329	0.409
994	0.429	0.942	0.142	0.444	1.020	0.102	0.463	1.161	0.224
995	0.224	-0.373	0.097	0.102	-1.184	0.478	0.226	-0.356	0.097
996	0.479	1.262	0.508	0.477	1.227	0.501	0.495	1.363	0.050
997	0.411	0.824	0.467	0.497	1.359	0.149	0.461	1.144	0.467
998	0.097	-1.181	0.512	0.149	-0.882	0.149	0.405	0.790	0.508
999	0.097	-1.181	0.328	0.149	-0.882	0.235	0.405	0.790	0.481
1000	0.384	0.655	0.501	0.234	-0.336	0.235	0.000	?	0.097
1001	0.384	0.655	0.501	0.234	-0.336	0.235	0.000	?	0.467
1002	0.384	0.655	0.501	0.234	-0.336	0.149	0.000	?	0.450
1003	0.224	-0.373	0.492	0.152	-0.863	0.398	0.224	-0.373	0.512
1004	0.142	-0.895	0.050	0.398	0.718	0.371	0.097	-1.181	0.512
1005	0.097	-1.181	0.512	0.371	0.549	0.514	0.050	-1.485	0.097
1006	0.511	1.464	0.501	0.509	1.434	0.491	0.463	1.161	0.097
1007	0.468	1.194	0.296	0.488	1.302	0.491	0.505	1.430	0.409
1008	0.511	1.464	0.224	0.494	1.340	0.149	0.474	1.228	0.409
1009	0.332	0.318	0.450	0.149	-0.882	0.508	0.097	-1.181	0.262
1010	0.503	1.413	0.450	0.506	1.415	0.508	0.463	1.161	0.142
1011	0.503	1.413	0.224	0.506	1.415	0.478	0.447	1.060	0.512
1012	0.495	1.363	0.513	0.474	1.208	0.462	0.513	1.481	0.512
1013	0.484	1.295	0.512	0.462	1.133	0.371	0.513	1.481	0.185
1014	0.050	-1.485	0.512	0.374	0.568	0.398	0.097	-1.181	0.508
1015	0.000	?	0.050	0.401	0.737	0.478	0.097	-1.181	0.492
1016	0.508	1.447	0.508	0.480	1.246	0.512	0.405	0.790	0.501
1017	0.503	1.413	0.492	0.506	1.415	0.371	0.408	0.807	0.296
1018	0.300	0.116	0.501	0.368	0.530	0.273	0.263	-0.120	0.492
1019	0.226	-0.356	0.097	0.275	-0.073	0.501	0.142	-0.895	0.481
1020	0.450	1.076	0.431	0.500	1.378	0.501	0.508	1.447	0.481
1021	0.450	1.076	0.097	0.500	1.378	0.149	0.508	1.447	0.296
1022	0.226	-0.356	0.512	0.149	-0.882	0.508	0.184	-0.625	0.050
1023	0.503	1.413	0.431	0.512	1.453	0.512	0.508	1.447	0.185
1024	0.500	1.397	0.513	0.518	1.491	0.508	0.492	1.346	0.513
1025	0.500	1.397	0.328	0.512	1.453	0.193	0.503	1.413	0.512
1026	0.050	-1.485	0.501	0.196	-0.581	0.462	0.297	0.099	0.513
1027	0.497	1.380	0.492	0.465	1.152	0.444	0.495	1.363	0.358
1028	0.495	1.363	0.512	0.442	1.001	0.501	0.476	1.245	0.492
1029	0.503	1.413	0.358	0.500	1.378	0.235	0.474	1.228	0.467
1030	0.097	-1.181	0.385	0.234	-0.336	0.149	0.295	0.082	0.467
1031	0.434	0.975	0.492	0.149	-0.882	0.273	0.050	-1.485	0.296
1032	0.097	-1.181	0.501	0.275	-0.073	0.371	0.184	-0.625	0.512
1033	0.505	1.430	0.262	0.368	0.530	0.478	0.511	1.464	0.481

1034	0.432	0.958	0.262	0.474	1.208	0.422	0.511	1.464	0.481
1035	0.500	1.397	0.492	0.421	0.869	0.371	0.516	1.498	0.185
1036	0.329	0.301	0.481	0.371	0.549	0.512	0.358	0.487	0.328
1037	0.503	1.413	0.467	0.518	1.491	0.512	0.492	1.346	0.501
1038	0.492	1.346	0.185	0.512	1.453	0.491	0.471	1.211	0.467
1039	0.508	1.447	0.492	0.494	1.340	0.149	0.461	1.144	0.481
1040	0.361	0.504	0.512	0.149	-0.882	0.478	0.297	0.099	0.262
1041	0.384	0.655	0.467	0.477	1.227	0.462	0.505	1.430	0.501
1042	0.492	1.346	0.467	0.468	1.170	0.422	0.482	1.279	0.508
1043	0.503	1.413	0.097	0.427	0.907	0.193	0.479	1.262	0.492
1044	0.263	-0.120	0.385	0.193	-0.600	0.102	0.184	-0.625	0.492
1045	0.266	-0.103	0.467	0.102	-1.184	0.462	0.329	0.301	0.224
1046	0.495	1.363	0.481	0.462	1.133	0.501	0.503	1.413	0.185
1047	0.482	1.279	0.492	0.497	1.359	0.501	0.466	1.178	0.467
1048	0.463	1.161	0.262	0.500	1.378	0.273	0.484	1.295	0.513
1049	0.187	-0.608	0.050	0.275	-0.073	0.508	0.263	-0.120	0.508
1050	0.489	1.329	0.296	0.506	1.415	0.508	0.497	1.380	0.409
1051	0.516	1.498	0.492	0.512	1.453	0.508	0.505	1.430	0.142
1052	0.466	1.178	0.508	0.506	1.415	0.512	0.492	1.346	0.142
1053	0.466	1.178	0.185	0.512	1.453	0.273	0.492	1.346	0.492
1054	0.097	-1.181	0.328	0.272	-0.091	0.309	0.224	-0.373	0.385
1055	0.387	0.672	0.328	0.313	0.172	0.444	0.184	-0.625	0.185
1056	0.466	1.178	0.467	0.442	1.001	0.341	0.466	1.178	0.050
1057	0.482	1.279	0.262	0.339	0.342	0.422	0.513	1.481	0.050
1058	0.492	1.346	0.328	0.421	0.869	0.102	0.508	1.447	0.513
1059	0.263	-0.120	0.097	0.102	-1.184	0.341	0.411	0.824	0.097
1060	0.050	-1.485	0.142	0.342	0.361	0.193	0.142	-0.895	0.142
1061	0.295	0.082	0.097	0.193	-0.600	0.491	0.142	-0.895	0.050
1062	0.489	1.329	0.512	0.491	1.321	0.514	0.489	1.329	0.142
1063	0.505	1.430	0.431	0.518	1.491	0.235	0.382	0.638	0.296
1064	0.184	-0.625	0.481	0.237	-0.317	0.235	0.184	-0.625	0.467
1065	0.329	0.301	0.501	0.237	-0.317	0.235	0.050	-1.485	0.501
1066	0.329	0.301	0.501	0.237	-0.317	0.341	0.050	-1.485	0.508
1067	0.466	1.178	0.467	0.345	0.379	0.273	0.505	1.430	0.508
1068	0.263	-0.120	0.481	0.275	-0.073	0.102	0.097	-1.181	0.512
1069	0.329	0.301	0.508	0.102	-1.184	0.371	0.142	-0.895	0.501
1070	0.097	-1.181	0.508	0.371	0.549	0.273	0.050	-1.485	0.481
1071	0.142	-0.895	0.512	0.275	-0.073	0.149	0.142	-0.895	0.501
1072	0.097	-1.181	0.508	0.149	-0.882	0.512	0.300	0.116	0.501
1073	0.505	1.430	0.450	0.515	1.472	0.422	0.466	1.178	0.512
1074	0.429	0.942	0.450	0.424	0.888	0.462	0.500	1.397	0.501
1075	0.476	1.245	0.262	0.462	1.133	0.491	0.508	1.447	0.508
1076	0.495	1.363	0.450	0.491	1.321	0.491	0.508	1.447	0.512
1077	0.495	1.363	0.185	0.491	1.321	0.508	0.513	1.481	0.185
1078	0.461	1.144	0.481	0.509	1.434	0.512	0.500	1.397	0.501
1079	0.482	1.279	0.409	0.515	1.472	0.508	0.482	1.279	0.185
1080	0.508	1.447	0.501	0.512	1.453	0.508	0.503	1.413	0.508
1081	0.508	1.447	0.450	0.512	1.453	0.501	0.503	1.413	0.501
1082	0.513	1.481	0.224	0.503	1.396	0.501	0.511	1.464	0.501
1083	0.508	1.447	0.467	0.506	1.415	0.422	0.503	1.413	0.513
1084	0.453	1.093	0.224	0.427	0.907	0.398	0.503	1.413	0.185
1085	0.450	1.076	0.501	0.398	0.718	0.309	0.511	1.464	0.431
1086	0.263	-0.120	0.262	0.310	0.153	0.512	0.187	-0.608	0.097
1087	0.447	1.060	0.492	0.512	1.453	0.193	0.500	1.397	0.513
1088	0.184	-0.625	0.296	0.193	-0.600	0.514	0.184	-0.625	0.358
1089	0.482	1.279	0.501	0.512	1.453	0.512	0.508	1.447	0.512
1090	0.413	0.841	0.512	0.512	1.453	0.444	0.497	1.380	0.097
1091	0.500	1.397	0.501	0.444	1.020	0.444	0.495	1.363	0.508
1092	0.447	1.060	0.450	0.444	1.020	0.341	0.505	1.430	0.508
1093	0.226	-0.356	0.481	0.345	0.379	0.501	0.184	-0.625	0.508
1094	0.466	1.178	0.492	0.503	1.396	0.371	0.429	0.942	0.508

1095	0.226	-0.356	0.097	0.371	0.549	0.478	0.100	-1.164	0.508
1096	0.505	1.430	0.097	0.480	1.246	0.309	0.511	1.464	0.467
1097	0.263	-0.120	0.097	0.307	0.135	0.462	0.363	0.520	0.409
1098	0.495	1.363	0.097	0.462	1.133	0.309	0.508	1.447	0.185
1099	0.297	0.099	0.492	0.307	0.135	0.422	0.097	-1.181	0.185
1100	0.497	1.380	0.328	0.421	0.869	0.512	0.505	1.430	0.185
1101	0.505	1.430	0.492	0.512	1.453	0.512	0.511	1.464	0.492
1102	0.497	1.380	0.224	0.515	1.472	0.478	0.505	1.430	0.262
1103	0.453	1.093	0.501	0.480	1.246	0.501	0.503	1.413	0.431
1104	0.484	1.295	0.358	0.497	1.359	0.398	0.505	1.430	0.097
1105	0.495	1.363	0.501	0.401	0.737	0.273	0.461	1.144	0.512
1106	0.097	-1.181	0.467	0.272	-0.091	0.341	0.411	0.824	0.508
1107	0.097	-1.181	0.481	0.342	0.361	0.273	0.184	-0.625	0.508
1108	0.097	-1.181	0.262	0.272	-0.091	0.273	0.184	-0.625	0.512
1109	0.097	-1.181	0.185	0.272	-0.091	0.398	0.184	-0.625	0.492
1110	0.492	1.346	0.262	0.398	0.718	0.309	0.497	1.380	0.097
1111	0.332	0.318	0.185	0.310	0.153	0.501	0.263	-0.120	0.296
1112	0.495	1.363	0.142	0.503	1.396	0.235	0.426	0.925	0.262
1113	0.224	-0.373	0.185	0.234	-0.336	0.462	0.097	-1.181	0.262
1114	0.503	1.413	0.508	0.462	1.133	0.478	0.508	1.447	0.262
1115	0.358	0.487	0.358	0.480	1.246	0.444	0.503	1.413	0.296
1116	0.505	1.430	0.358	0.447	1.039	0.444	0.505	1.430	0.501
1117	0.471	1.211	0.097	0.444	1.020	0.508	0.508	1.447	0.050
1118	0.479	1.262	0.097	0.509	1.434	0.273	0.487	1.312	0.050
1119	0.263	-0.120	0.097	0.272	-0.091	0.273	0.097	-1.181	0.262
1120	0.184	-0.625	0.501	0.275	-0.073	0.398	0.300	0.116	0.262
1121	0.261	-0.137	0.512	0.401	0.737	0.273	0.263	-0.120	0.262
1122	0.187	-0.608	0.185	0.275	-0.073	0.309	0.263	-0.120	0.450
1123	0.142	-0.895	0.142	0.307	0.135	0.273	0.261	-0.137	0.508
1124	0.187	-0.608	0.467	0.272	-0.091	0.508	0.297	0.099	0.262
1125	0.500	1.397	0.262	0.506	1.415	0.149	0.503	1.413	0.296
1126	0.358	0.487	0.328	0.149	-0.882	0.235	0.050	-1.485	0.481
1127	0.358	0.487	0.262	0.234	-0.336	0.193	0.050	-1.485	0.097
1128	0.097	-1.181	0.467	0.193	-0.600	0.235	0.261	-0.137	0.185
1129	0.097	-1.181	0.296	0.237	-0.317	0.235	0.261	-0.137	0.097
1130	0.097	-1.181	0.467	0.237	-0.317	0.422	0.261	-0.137	0.501
1131	0.503	1.413	0.224	0.421	0.869	0.491	0.453	1.093	0.185
1132	0.508	1.447	0.358	0.491	1.321	0.235	0.503	1.413	0.296
1133	0.184	-0.625	0.142	0.237	-0.317	0.273	0.263	-0.120	0.492
1134	0.145	-0.878	0.450	0.278	-0.054	0.508	0.295	0.082	0.050
1135	0.466	1.178	0.409	0.506	1.415	0.193	0.484	1.295	0.050
1136	0.261	-0.137	0.185	0.193	-0.600	0.193	0.097	-1.181	0.224
1137	0.329	0.301	0.385	0.193	-0.600	0.235	0.184	-0.625	0.450
1138	0.261	-0.137	0.328	0.234	-0.336	0.478	0.097	-1.181	0.185
1139	0.466	1.178	0.185	0.477	1.227	0.235	0.495	1.363	0.262
1140	0.297	0.099	0.050	0.234	-0.336	0.371	0.184	-0.625	0.142
1141	0.000	?	0.050	0.374	0.568	0.501	0.297	0.099	0.142
1142	0.468	1.194	0.512	0.500	1.378	0.273	0.484	1.295	0.224
1143	0.224	-0.373	0.512	0.272	-0.091	0.235	0.050	-1.485	0.262
1144	0.361	0.504	0.142	0.234	-0.336	0.341	0.050	-1.485	0.097
1145	0.142	-0.895	0.512	0.342	0.361	0.512	0.226	-0.356	0.508
1146	0.453	1.093	0.512	0.509	1.434	0.309	0.450	1.076	0.508
1147	0.413	0.841	0.512	0.307	0.135	0.102	0.184	-0.625	0.224
1148	0.184	-0.625	0.185	0.102	-1.184	0.341	0.261	-0.137	0.492
1149	0.389	0.689	0.050	0.339	0.342	0.273	0.142	-0.895	0.467
1150	0.332	0.318	0.513	0.275	-0.073	0.149	0.142	-0.895	0.481
1151	0.184	-0.625	0.508	0.152	-0.863	0.235	0.226	-0.356	0.097
1152	0.050	-1.485	0.224	0.234	-0.336	0.444	0.263	-0.120	0.142
1153	0.050	-1.485	0.431	0.444	1.020	0.508	0.097	-1.181	0.492
1154	0.511	1.464	0.431	0.512	1.453	0.508	0.505	1.430	0.492
1155	0.511	1.464	0.409	0.512	1.453	0.341	0.505	1.430	0.262

1156	0.142	-0.895	0.185	0.348	0.398	0.501	0.224	-0.373	0.508
1157	0.505	1.430	0.492	0.497	1.359	0.508	0.492	1.346	0.508
1158	0.513	1.481	0.185	0.512	1.453	0.512	0.471	1.211	0.481
1159	0.508	1.447	0.142	0.509	1.434	0.273	0.479	1.262	0.328
1160	0.184	-0.625	0.492	0.275	-0.073	0.422	0.097	-1.181	0.501
1161	0.050	-1.485	0.492	0.421	0.869	0.478	0.142	-0.895	0.296
1162	0.505	1.430	0.142	0.477	1.227	0.491	0.492	1.346	0.296
1163	0.500	1.397	0.492	0.488	1.302	0.102	0.492	1.346	0.481
1164	0.224	-0.373	0.492	0.102	-1.184	0.512	0.263	-0.120	0.512
1165	0.429	0.942	0.262	0.506	1.415	0.514	0.505	1.430	0.097
1166	0.429	0.942	0.185	0.509	1.434	0.512	0.505	1.430	0.450
1167	0.408	0.807	0.185	0.509	1.434	0.235	0.479	1.262	0.450
1168	0.184	-0.625	0.467	0.237	-0.317	0.508	0.332	0.318	0.142
1169	0.492	1.346	0.492	0.512	1.453	0.398	0.495	1.363	0.358
1170	0.187	-0.608	0.224	0.404	0.756	0.235	0.295	0.082	0.328
1171	0.145	-0.878	0.296	0.234	-0.336	0.508	0.295	0.082	0.512
1172	0.487	1.312	0.467	0.509	1.434	0.478	0.479	1.262	0.450
1173	0.484	1.295	0.501	0.482	1.265	0.371	0.513	1.481	0.185
1174	0.142	-0.895	0.185	0.374	0.568	0.508	0.097	-1.181	0.224
1175	0.484	1.295	0.467	0.509	1.434	0.508	0.447	1.060	0.501
1176	0.484	1.295	0.409	0.509	1.434	0.149	0.447	1.060	0.513
1177	0.266	-0.103	0.224	0.149	-0.882	0.053	0.142	-0.895	0.224
1178	0.187	-0.608	0.224	0.053	-1.504	0.053	0.358	0.487	0.481
1179	0.187	-0.608	0.492	0.053	-1.504	0.478	0.329	0.301	0.508
1180	0.466	1.178	0.492	0.474	1.208	0.491	0.511	1.464	0.501
1181	0.487	1.312	0.296	0.485	1.283	0.273	0.453	1.093	0.501
1182	0.226	-0.356	0.508	0.275	-0.073	0.508	0.184	-0.625	0.142
1183	0.297	0.099	0.481	0.000	?	0.478	0.224	-0.373	0.450
1184	0.461	1.144	0.142	0.509	1.434	0.193	0.497	1.380	0.467
1185	0.497	1.380	0.262	0.477	1.227	0.514	0.508	1.447	0.097
1186	0.187	-0.608	0.142	0.193	-0.600	0.508	0.224	-0.373	0.224
1187	0.463	1.161	0.508	0.512	1.453	0.309	0.479	1.262	0.097
1188	0.408	0.807	0.508	0.512	1.453	0.309	0.505	1.430	0.501
1189	0.226	-0.356	0.185	0.310	0.153	0.478	0.000	?	0.481
1190	0.226	-0.356	0.492	0.310	0.153	0.478	0.000	?	0.142
1191	0.489	1.329	0.467	0.480	1.246	0.273	0.495	1.363	0.508
1192	0.489	1.329	0.467	0.480	1.246	0.422	0.495	1.363	0.481
1193	0.295	0.082	0.481	0.278	-0.054	0.491	0.142	-0.895	0.508
1194	0.503	1.413	0.501	0.424	0.888	0.309	0.450	1.076	0.512
1195	0.484	1.295	0.450	0.494	1.340	0.053	0.466	1.178	0.501
1196	0.142	-0.895	0.501	0.307	0.135	0.309	0.097	-1.181	0.492
1197	0.261	-0.137	0.224	0.053	-1.504	0.478	0.224	-0.373	0.450
1198	0.142	-0.895	0.481	0.307	0.135	0.478	0.097	-1.181	0.385
1199	0.508	1.447	0.262	0.480	1.246	0.273	0.500	1.397	0.512
1200	0.508	1.447	0.185	0.480	1.246	0.508	0.479	1.262	0.097
1201	0.184	-0.625	0.296	0.272	-0.091	0.512	0.142	-0.895	0.050
1202	0.492	1.346	0.296	0.509	1.434	0.501	0.503	1.413	0.142
1203	0.466	1.178	0.296	0.515	1.472	0.478	0.474	1.228	0.142
1204	0.466	1.178	0.296	0.503	1.396	0.491	0.505	1.430	0.142
1205	0.479	1.262	0.385	0.477	1.227	0.508	0.508	1.447	0.142
1206	0.500	1.397	0.262	0.491	1.321	0.501	0.497	1.380	0.501
1207	0.453	1.093	0.501	0.509	1.434	0.371	0.492	1.346	0.185
1208	0.505	1.430	0.431	0.497	1.359	0.422	0.450	1.076	0.467
1209	0.224	-0.373	0.508	0.374	0.568	0.235	0.382	0.638	0.501
1210	0.482	1.279	0.508	0.421	0.869	0.341	0.513	1.481	0.508
1211	0.266	-0.103	0.508	0.234	-0.336	0.193	0.097	-1.181	0.481
1212	0.184	-0.625	0.097	0.342	0.361	0.193	0.050	-1.485	0.450
1213	0.297	0.099	0.512	0.193	-0.600	0.193	0.142	-0.895	0.097
1214	0.297	0.099	0.508	0.193	-0.600	0.193	0.142	-0.895	0.385
1215	0.297	0.099	0.296	0.193	-0.600	0.478	0.142	-0.895	0.481
1216	0.297	0.099	0.409	0.193	-0.600	0.273	0.142	-0.895	0.050

1217	0.387	0.672	0.492	0.480	1.246	0.508	0.500	1.397	0.513
1218	0.261	-0.137	0.142	0.272	-0.091	0.508	0.184	-0.625	0.492
1219	0.503	1.413	0.481	0.512	1.453	0.491	0.468	1.194	0.097
1220	0.429	0.942	0.224	0.506	1.415	0.501	0.497	1.380	0.501
1221	0.505	1.430	0.508	0.488	1.302	0.508	0.508	1.447	0.097
1222	0.497	1.380	0.142	0.503	1.396	0.341	0.484	1.295	0.409
1223	0.497	1.380	0.481	0.509	1.434	0.508	0.453	1.093	0.142
1224	0.097	-1.181	0.185	0.345	0.379	0.508	0.097	-1.181	0.467
1225	0.511	1.464	0.492	0.509	1.434	0.235	0.384	0.655	0.385
1226	0.508	1.447	0.431	0.509	1.434	0.508	0.479	1.262	0.508
1227	0.297	0.099	0.501	0.237	-0.317	0.501	0.050	-1.485	0.508
1228	0.405	0.790	0.328	0.506	1.415	0.309	0.511	1.464	0.481
1229	0.487	1.312	0.262	0.506	1.415	0.508	0.487	1.312	0.512
1230	0.142	-0.895	0.142	0.313	0.172	0.273	0.097	-1.181	0.050
1231	0.476	1.245	0.512	0.506	1.415	0.491	0.503	1.413	0.262
1232	0.224	-0.373	0.508	0.275	-0.073	0.273	0.097	-1.181	0.501
1233	0.497	1.380	0.224	0.497	1.359	0.444	0.408	0.807	0.450
1234	0.145	-0.878	0.262	0.272	-0.091	0.398	0.142	-0.895	0.224
1235	0.474	1.228	0.450	0.444	1.020	0.491	0.468	1.194	0.224
1236	0.184	-0.625	0.501	0.404	0.756	0.478	0.384	0.655	0.501
1237	0.484	1.295	0.501	0.491	1.321	0.478	0.513	1.481	0.508
1238	0.429	0.942	0.501	0.480	1.246	0.512	0.513	1.481	0.508
1239	0.505	1.430	0.492	0.477	1.227	0.235	0.482	1.279	0.508
1240	0.326	0.285	0.492	0.512	1.453	0.235	0.511	1.464	0.508
1241	0.263	-0.120	0.501	0.234	-0.336	0.462	0.050	-1.485	0.431
1242	0.142	-0.895	0.501	0.234	-0.336	0.491	0.266	-0.103	0.467
1243	0.513	1.481	0.508	0.465	1.152	0.149	0.505	1.430	0.431
1244	0.497	1.380	0.492	0.491	1.321	0.149	0.450	1.076	0.262
1245	0.224	-0.373	0.097	0.152	-0.863	0.444	0.224	-0.373	0.508
1246	0.261	-0.137	0.097	0.152	-0.863	0.462	0.224	-0.373	0.508
1247	0.450	1.076	0.097	0.444	1.020	0.462	0.495	1.363	0.185
1248	0.492	1.346	0.501	0.462	1.133	0.462	0.508	1.447	0.185
1249	0.492	1.346	0.501	0.462	1.133	0.512	0.508	1.447	0.185
1250	0.492	1.346	0.185	0.462	1.133	0.462	0.508	1.447	0.508
1251	0.487	1.312	0.467	0.520	1.509	0.444	0.508	1.447	0.508
1252	0.489	1.329	0.142	0.462	1.133	0.462	0.426	0.925	0.097
1253	0.497	1.380	0.467	0.444	1.020	0.273	0.466	1.178	0.513
1254	0.497	1.380	0.467	0.465	1.152	0.501	0.426	0.925	0.262
1255	0.000	?	0.481	0.275	-0.073	0.501	0.263	-0.120	0.512
1256	0.497	1.380	0.185	0.506	1.415	0.371	0.511	1.464	0.512
1257	0.492	1.346	0.508	0.497	1.359	0.398	0.503	1.413	0.513
1258	0.097	-1.181	0.512	0.371	0.549	0.371	0.184	-0.625	0.385
1259	0.097	-1.181	0.501	0.398	0.718	0.478	0.184	-0.625	0.508
1260	0.097	-1.181	0.481	0.371	0.549	0.478	0.184	-0.625	0.481
1261	0.500	1.397	0.328	0.474	1.208	0.273	0.505	1.430	0.481
1262	0.500	1.397	0.431	0.474	1.208	0.491	0.505	1.430	0.512
1263	0.184	-0.625	0.296	0.275	-0.073	0.273	0.097	-1.181	0.050
1264	0.466	1.178	0.296	0.488	1.302	0.491	0.513	1.481	0.513
1265	0.145	-0.878	0.262	0.272	-0.091	0.444	0.263	-0.120	0.097
1266	0.461	1.144	0.262	0.494	1.340	0.501	0.513	1.481	0.050
1267	0.461	1.144	0.328	0.442	1.001	0.235	0.516	1.498	0.185
1268	0.482	1.279	0.512	0.503	1.396	0.444	0.505	1.430	0.501
1269	0.184	-0.625	0.467	0.234	-0.336	0.508	0.384	0.655	0.512
1270	0.505	1.430	0.467	0.439	0.982	0.514	0.505	1.430	0.501
1271	0.508	1.447	0.481	0.500	1.378	0.491	0.482	1.279	0.185
1272	0.492	1.346	0.501	0.515	1.472	0.309	0.482	1.279	0.501
1273	0.484	1.295	0.450	0.491	1.321	0.491	0.503	1.413	0.328
1274	0.332	0.318	0.481	0.310	0.153	0.235	0.050	-1.485	0.512
1275	0.434	0.975	0.385	0.488	1.302	0.235	0.503	1.413	0.501
1276	0.297	0.099	0.508	0.237	-0.317	0.235	0.000	?	0.467
1277	0.297	0.099	0.050	0.237	-0.317	0.309	0.000	?	0.508

1278	0.261	-0.137	0.501	0.234	-0.336	0.102	0.097	-1.181	0.262
1279	0.261	-0.137	0.513	0.310	0.153	0.462	0.050	-1.485	0.508
1280	0.329	0.301	0.097	0.102	-1.184	0.514	0.184	-0.625	0.467
1281	0.513	1.481	0.097	0.459	1.114	0.444	0.503	1.413	0.328
1282	0.468	1.194	0.097	0.509	1.434	0.341	0.513	1.481	0.328
1283	0.461	1.144	0.185	0.444	1.020	0.508	0.500	1.397	0.328
1284	0.000	?	0.467	0.342	0.361	0.478	0.184	-0.625	0.296
1285	0.484	1.295	0.097	0.509	1.434	0.491	0.497	1.380	0.513
1286	0.505	1.430	0.492	0.474	1.208	0.501	0.329	0.301	0.328
1287	0.450	1.076	0.501	0.488	1.302	0.478	0.513	1.481	0.450
1288	0.479	1.262	0.512	0.500	1.378	0.491	0.503	1.413	0.450
1289	0.384	0.655	0.512	0.474	1.208	0.309	0.468	1.194	0.492
1290	0.503	1.413	0.481	0.485	1.283	0.512	0.508	1.447	0.508
1291	0.050	-1.485	0.467	0.310	0.153	0.512	0.263	-0.120	0.431
1292	0.497	1.380	0.262	0.515	1.472	0.341	0.503	1.413	0.481
1293	0.503	1.413	0.450	0.518	1.491	0.341	0.466	1.178	0.358
1294	0.097	-1.181	0.467	0.342	0.361	0.341	0.329	0.301	0.467
1295	0.097	-1.181	0.512	0.342	0.361	0.102	0.329	0.301	0.508
1296	0.097	-1.181	0.492	0.342	0.361	0.508	0.329	0.301	0.512
1297	0.184	-0.625	0.512	0.102	-1.184	0.102	0.297	0.099	0.409
1298	0.466	1.178	0.512	0.503	1.396	0.514	0.511	1.464	0.409
1299	0.100	-1.164	0.328	0.102	-1.184	0.514	0.329	0.301	0.501
1300	0.489	1.329	0.262	0.512	1.453	0.512	0.447	1.060	0.296
1301	0.497	1.380	0.512	0.512	1.453	0.514	0.447	1.060	0.142
1302	0.511	1.464	0.262	0.509	1.434	0.512	0.489	1.329	0.492
1303	0.508	1.447	0.262	0.512	1.453	0.491	0.511	1.464	0.262
1304	0.476	1.245	0.262	0.512	1.453	0.193	0.432	0.958	0.142
1305	0.463	1.161	0.501	0.494	1.340	0.512	0.479	1.262	0.142
1306	0.263	-0.120	0.512	0.196	-0.581	0.508	0.358	0.487	0.513
1307	0.453	1.093	0.481	0.512	1.453	0.422	0.466	1.178	0.501
1308	0.468	1.194	0.224	0.509	1.434	0.508	0.505	1.430	0.467
1309	0.516	1.498	0.450	0.424	0.888	0.491	0.513	1.481	0.296
1310	0.495	1.363	0.328	0.509	1.434	0.501	0.405	0.790	0.508
1311	0.513	1.481	0.296	0.488	1.302	0.371	0.408	0.807	0.142
1312	0.505	1.430	0.296	0.500	1.378	0.235	0.503	1.413	0.185
1313	0.329	0.301	0.142	0.374	0.568	0.501	0.295	0.082	0.224
1314	0.263	-0.120	0.224	0.234	-0.336	0.235	0.142	-0.895	0.262
1315	0.508	1.447	0.296	0.497	1.359	0.193	0.489	1.329	0.185
1316	0.266	-0.103	0.492	0.237	-0.317	0.193	0.261	-0.137	0.224
1317	0.263	-0.120	0.508	0.196	-0.581	0.491	0.142	-0.895	0.492
1318	0.263	-0.120	0.097	0.196	-0.581	0.491	0.142	-0.895	0.450
1319	0.492	1.346	0.467	0.494	1.340	0.514	0.511	1.464	0.328
1320	0.516	1.498	0.262	0.494	1.340	0.235	0.500	1.397	0.467
1321	0.482	1.279	0.185	0.512	1.453	0.491	0.463	1.161	0.142
1322	0.224	-0.373	0.185	0.237	-0.317	0.309	0.295	0.082	0.328
1323	0.447	1.060	0.185	0.488	1.302	0.193	0.508	1.447	0.097
1324	0.329	0.301	0.185	0.307	0.135	0.235	0.142	-0.895	0.262
1325	0.297	0.099	0.481	0.193	-0.600	0.149	0.184	-0.625	0.262
1326	0.295	0.082	0.142	0.234	-0.336	0.149	0.226	-0.356	0.492
1327	0.142	-0.895	0.431	0.149	-0.882	0.273	0.263	-0.120	0.262
1328	0.224	-0.373	0.501	0.149	-0.882	0.478	0.184	-0.625	0.512
1329	0.297	0.099	0.481	0.272	-0.091	0.444	0.224	-0.373	0.481
1330	0.489	1.329	0.358	0.485	1.283	0.235	0.489	1.329	0.492
1331	0.508	1.447	0.224	0.439	0.982	0.512	0.447	1.060	0.050
1332	0.097	-1.181	0.481	0.237	-0.317	0.273	0.329	0.301	0.185
1333	0.461	1.144	0.492	0.512	1.453	0.273	0.463	1.161	0.409
1334	0.263	-0.120	0.508	0.272	-0.091	0.273	0.142	-0.895	0.481
1335	0.184	-0.625	0.385	0.272	-0.091	0.341	0.326	0.285	0.492
1336	0.184	-0.625	0.409	0.278	-0.054	0.341	0.097	-1.181	0.262
1337	0.187	-0.608	0.492	0.339	0.342	0.514	0.263	-0.120	0.224
1338	0.187	-0.608	0.467	0.339	0.342	0.235	0.263	-0.120	0.409

1339	0.479	1.262	0.467	0.512	1.453	0.491	0.487	1.312	0.512
1340	0.142	-0.895	0.328	0.237	-0.317	0.478	0.263	-0.120	0.513
1341	0.426	0.925	0.296	0.488	1.302	0.512	0.511	1.464	0.358
1342	0.495	1.363	0.508	0.477	1.227	0.273	0.479	1.262	0.142
1343	0.479	1.262	0.508	0.515	1.472	0.309	0.484	1.295	0.512
1344	0.358	0.487	0.512	0.275	-0.073	0.398	0.050	-1.485	0.512
1345	0.224	-0.373	0.450	0.310	0.153	0.462	0.187	-0.608	0.431
1346	0.476	1.245	0.492	0.398	0.718	0.398	0.411	0.824	0.492
1347	0.484	1.295	0.050	0.465	1.152	0.371	0.484	1.295	0.512
1348	0.497	1.380	0.513	0.401	0.737	0.341	0.492	1.346	0.262
1349	0.382	0.638	0.508	0.368	0.530	0.512	0.261	-0.137	0.508
1350	0.408	0.807	0.185	0.339	0.342	0.478	0.224	-0.373	0.512
1351	0.489	1.329	0.142	0.520	1.509	0.462	0.411	0.824	0.358
1352	0.461	1.144	0.508	0.477	1.227	0.193	0.511	1.464	0.262
1353	0.461	1.144	0.508	0.459	1.114	0.273	0.513	1.481	0.481
1354	0.326	0.285	0.450	0.193	-0.600	0.462	0.361	0.504	0.467
1355	0.295	0.082	0.431	0.275	-0.073	0.462	0.142	-0.895	0.450
1356	0.505	1.430	0.185	0.462	1.133	0.514	0.511	1.464	0.508
1357	0.505	1.430	0.358	0.462	1.133	0.422	0.511	1.464	0.385
1358	0.513	1.481	0.296	0.515	1.472	0.491	0.429	0.942	0.262
1359	0.447	1.060	0.512	0.424	0.888	0.235	0.492	1.346	0.224
1360	0.489	1.329	0.050	0.491	1.321	0.444	0.508	1.447	0.481
1361	0.050	-1.485	0.513	0.237	-0.317	0.508	0.263	-0.120	0.262
1362	0.513	1.481	0.224	0.442	1.001	0.371	0.505	1.430	0.501
1363	0.508	1.447	0.385	0.506	1.415	0.149	0.508	1.447	0.358
1364	0.184	-0.625	0.142	0.374	0.568	0.501	0.361	0.504	0.224
1365	0.142	-0.895	0.185	0.152	-0.863	0.491	0.263	-0.120	0.185
1366	0.503	1.413	0.501	0.500	1.378	0.514	0.479	1.262	0.185
1367	0.508	1.447	0.450	0.488	1.302	0.491	0.468	1.194	0.512
1368	0.447	1.060	0.296	0.512	1.453	0.371	0.447	1.060	0.501
1369	0.434	0.975	0.450	0.491	1.321	0.371	0.505	1.430	0.142
1370	0.184	-0.625	0.512	0.371	0.549	0.273	0.387	0.672	0.481
1371	0.358	0.487	0.467	0.377	0.587	0.514	0.261	-0.137	0.508
1372	0.297	0.099	0.512	0.272	-0.091	0.235	0.226	-0.356	0.492
1373	0.513	1.481	0.481	0.509	1.434	0.512	0.484	1.295	0.492
1374	0.050	-1.485	0.481	0.234	-0.336	0.235	0.266	-0.103	0.512
1375	0.503	1.413	0.481	0.518	1.491	0.235	0.503	1.413	0.431
1376	0.226	-0.356	0.328	0.234	-0.336	0.309	0.361	0.504	0.431
1377	0.382	0.638	0.224	0.234	-0.336	0.235	0.226	-0.356	0.185
1378	0.142	-0.895	0.513	0.307	0.135	0.444	0.184	-0.625	0.224
1379	0.187	-0.608	0.328	0.237	-0.317	0.371	0.184	-0.625	0.508
1380	0.495	1.363	0.512	0.442	1.001	0.149	0.511	1.464	0.328
1381	0.447	1.060	0.224	0.368	0.530	0.422	0.503	1.413	0.501
1382	0.295	0.082	0.262	0.149	-0.882	0.491	0.142	-0.895	0.142
1383	0.450	1.076	0.512	0.421	0.869	0.462	0.484	1.295	0.224
1384	0.513	1.481	0.492	0.491	1.321	0.514	0.508	1.447	0.513
1385	0.468	1.194	0.492	0.462	1.133	0.478	0.492	1.346	0.508
1386	0.511	1.464	0.492	0.515	1.472	0.512	0.492	1.346	0.508
1387	0.476	1.245	0.467	0.480	1.246	0.512	0.508	1.447	0.492
1388	0.476	1.245	0.185	0.512	1.453	0.371	0.432	0.958	0.492
1389	0.476	1.245	0.224	0.512	1.453	0.371	0.432	0.958	0.050
1390	0.329	0.301	0.262	0.374	0.568	0.398	0.184	-0.625	0.185
1391	0.224	-0.373	0.262	0.374	0.568	0.102	0.224	-0.373	0.262
1392	0.513	1.481	0.262	0.398	0.718	0.508	0.508	1.447	0.185
1393	0.329	0.301	0.262	0.102	-1.184	0.235	0.329	0.301	0.185
1394	0.511	1.464	0.508	0.506	1.415	0.273	0.500	1.397	0.185
1395	0.226	-0.356	0.508	0.234	-0.336	0.501	0.142	-0.895	0.481
1396	0.263	-0.120	0.481	0.275	-0.073	0.514	0.226	-0.356	0.481
1397	0.513	1.481	0.492	0.506	1.415	0.501	0.508	1.447	0.467
1398	0.497	1.380	0.492	0.518	1.491	0.491	0.505	1.430	0.467
1399	0.492	1.346	0.224	0.497	1.359	0.491	0.503	1.413	0.467

1400	0.495	1.363	0.296	0.491	1.321	0.341	0.484	1.295	0.328
1401	0.463	1.161	0.431	0.488	1.302	0.193	0.489	1.329	0.185
1402	0.184	-0.625	0.508	0.339	0.342	0.149	0.050	-1.485	0.450
1403	0.226	-0.356	0.185	0.196	-0.581	0.273	0.184	-0.625	0.508
1404	0.263	-0.120	0.492	0.149	-0.882	0.273	0.263	-0.120	0.097
1405	0.263	-0.120	0.512	0.275	-0.073	0.273	0.187	-0.608	0.467
1406	0.263	-0.120	0.450	0.275	-0.073	0.514	0.187	-0.608	0.508
1407	0.263	-0.120	0.431	0.275	-0.073	0.512	0.187	-0.608	0.508
1408	0.513	1.481	0.512	0.518	1.491	0.514	0.479	1.262	0.467
1409	0.505	1.430	0.358	0.512	1.453	0.512	0.474	1.228	0.481
1410	0.482	1.279	0.262	0.515	1.472	0.512	0.463	1.161	0.296
1411	0.492	1.346	0.467	0.512	1.453	0.053	0.463	1.161	0.142
1412	0.492	1.346	0.185	0.512	1.453	0.371	0.463	1.161	0.481
1413	0.224	-0.373	0.481	0.053	-1.504	0.491	0.329	0.301	0.262
1414	0.295	0.082	0.501	0.371	0.549	0.501	0.184	-0.625	0.501
1415	0.432	0.958	0.224	0.491	1.321	0.309	0.447	1.060	0.467
1416	0.503	1.413	0.481	0.500	1.378	0.491	0.505	1.430	0.328
1417	0.184	-0.625	0.481	0.307	0.135	0.508	0.097	-1.181	0.512
1418	0.492	1.346	0.296	0.485	1.283	0.478	0.468	1.194	0.409
1419	0.508	1.447	0.224	0.503	1.396	0.512	0.511	1.464	0.185
1420	0.455	1.110	0.508	0.477	1.227	0.444	0.508	1.447	0.409
1421	0.426	0.925	0.508	0.512	1.453	0.102	0.466	1.178	0.385
1422	0.505	1.430	0.467	0.444	1.020	0.235	0.484	1.295	0.385
1423	0.358	0.487	0.467	0.102	-1.184	0.512	0.300	0.116	0.501
1424	0.261	-0.137	0.501	0.240	-0.299	0.102	0.142	-0.895	0.501
1425	0.466	1.178	0.501	0.518	1.491	0.491	0.484	1.295	0.508
1426	0.187	-0.608	0.358	0.102	-1.184	0.512	0.263	-0.120	0.508
1427	0.476	1.245	0.508	0.491	1.321	0.149	0.500	1.397	0.262
1428	0.503	1.413	0.467	0.512	1.453	0.462	0.463	1.161	0.481
1429	0.226	-0.356	0.512	0.149	-0.882	0.491	0.326	0.285	0.501
1430	0.479	1.262	0.328	0.462	1.133	0.341	0.505	1.430	0.450
1431	0.484	1.295	0.512	0.488	1.302	0.193	0.405	0.790	0.097
1432	0.295	0.082	0.142	0.342	0.361	0.512	0.184	-0.625	0.358
1433	0.226	-0.356	0.358	0.193	-0.600	0.512	0.408	0.807	0.142
1434	0.508	1.447	0.409	0.506	1.415	0.514	0.384	0.655	0.513
1435	0.508	1.447	0.328	0.506	1.415	0.514	0.384	0.655	0.385
1436	0.463	1.161	0.513	0.506	1.415	0.398	0.505	1.430	0.224
1437	0.463	1.161	0.296	0.506	1.415	0.398	0.505	1.430	0.501
1438	0.495	1.363	0.431	0.404	0.756	0.309	0.508	1.447	0.142
1439	0.495	1.363	0.467	0.404	0.756	0.491	0.508	1.447	0.501
1440	0.358	0.487	0.450	0.307	0.135	0.491	0.263	-0.120	0.431
1441	0.508	1.447	0.185	0.488	1.302	0.508	0.479	1.262	0.513
1442	0.468	1.194	0.185	0.482	1.265	0.149	0.497	1.380	0.185
1443	0.505	1.430	0.296	0.509	1.434	0.462	0.447	1.060	0.185
1444	0.329	0.301	0.296	0.152	-0.863	0.309	0.097	-1.181	0.224
1445	0.511	1.464	0.512	0.459	1.114	0.508	0.355	0.470	0.185
1446	0.142	-0.895	0.512	0.313	0.172	0.478	0.142	-0.895	0.467
1447	0.355	0.470	0.224	0.506	1.415	0.341	0.505	1.430	0.481
1448	0.411	0.824	0.185	0.474	1.208	0.491	0.384	0.655	0.185
1449	0.332	0.318	0.501	0.339	0.342	0.193	0.226	-0.356	0.262
1450	0.511	1.464	0.492	0.494	1.340	0.508	0.495	1.363	0.508
1451	0.300	0.116	0.431	0.193	-0.600	0.491	0.142	-0.895	0.358
1452	0.432	0.958	0.296	0.503	1.396	0.462	0.495	1.363	0.450
1453	0.468	1.194	0.097	0.488	1.302	0.309	0.432	0.958	0.185
1454	0.453	1.093	0.097	0.459	1.114	0.235	0.511	1.464	0.328
1455	0.184	-0.625	0.431	0.307	0.135	0.235	0.184	-0.625	0.328
1456	0.184	-0.625	0.492	0.237	-0.317	0.235	0.184	-0.625	0.492
1457	0.300	0.116	0.185	0.234	-0.336	0.371	0.224	-0.373	0.467
1458	0.300	0.116	0.450	0.234	-0.336	0.508	0.184	-0.625	0.296
1459	0.511	1.464	0.050	0.371	0.549	0.149	0.466	1.178	0.501
1460	0.511	1.464	0.481	0.506	1.415	0.149	0.474	1.228	0.224

1461	0.226	-0.356	0.450	0.149	-0.882	0.422	0.184	-0.625	0.492
1462	0.187	-0.608	0.492	0.149	-0.882	0.422	0.261	-0.137	0.501
1463	0.505	1.430	0.492	0.421	0.869	0.514	0.505	1.430	0.508
1464	0.492	1.346	0.185	0.421	0.869	0.193	0.355	0.470	0.358
1465	0.426	0.925	0.097	0.509	1.434	0.273	0.450	1.076	0.097
1466	0.295	0.082	0.492	0.196	-0.581	0.273	0.187	-0.608	0.296
1467	0.097	-1.181	0.224	0.272	-0.091	0.508	0.334	0.335	0.467
1468	0.097	-1.181	0.492	0.272	-0.091	0.512	0.334	0.335	0.262
1469	0.429	0.942	0.142	0.515	1.472	0.398	0.497	1.380	0.481
1470	0.489	1.329	0.328	0.512	1.453	0.501	0.466	1.178	0.142
1471	0.184	-0.625	0.224	0.404	0.756	0.273	0.297	0.099	0.050
1472	0.453	1.093	0.385	0.503	1.396	0.512	0.500	1.397	0.142
1473	0.050	-1.485	0.097	0.272	-0.091	0.514	0.224	-0.373	0.262
1474	0.474	1.228	0.224	0.518	1.491	0.444	0.487	1.312	0.296
1475	0.447	1.060	0.481	0.515	1.472	0.508	0.497	1.380	0.050
1476	0.492	1.346	0.492	0.447	1.039	0.341	0.508	1.447	0.481
1477	0.492	1.346	0.508	0.503	1.396	0.235	0.358	0.487	0.508
1478	0.187	-0.608	0.492	0.342	0.361	0.462	0.097	-1.181	0.501
1479	0.097	-1.181	0.481	0.237	-0.317	0.309	0.297	0.099	0.431
1480	0.489	1.329	0.501	0.462	1.133	0.512	0.466	1.178	0.508
1481	0.226	-0.356	0.512	0.313	0.172	0.309	0.261	-0.137	0.513
1482	0.487	1.312	0.508	0.506	1.415	0.193	0.487	1.312	0.501
1483	0.142	-0.895	0.508	0.307	0.135	0.193	0.145	-0.878	0.512
1484	0.329	0.301	0.508	0.193	-0.600	0.102	0.050	-1.485	0.512
1485	0.226	-0.356	0.508	0.193	-0.600	0.341	0.142	-0.895	0.512
1486	0.387	0.672	0.512	0.102	-1.184	0.309	0.261	-0.137	0.501
1487	0.097	-1.181	0.224	0.345	0.379	0.508	0.297	0.099	0.513
1488	0.224	-0.373	0.185	0.313	0.172	0.341	0.050	-1.485	0.296
1489	0.476	1.245	0.097	0.509	1.434	0.501	0.482	1.279	0.296
1490	0.492	1.346	0.501	0.339	0.342	0.512	0.503	1.413	0.185
1491	0.503	1.413	0.185	0.503	1.396	0.514	0.500	1.397	0.513
1492	0.487	1.312	0.328	0.515	1.472	0.491	0.429	0.942	0.097
1493	0.482	1.279	0.262	0.518	1.491	0.444	0.508	1.447	0.050
1494	0.503	1.413	0.296	0.494	1.340	0.491	0.513	1.481	0.185
1495	0.511	1.464	0.185	0.442	1.001	0.491	0.497	1.380	0.050
1496	0.500	1.397	0.142	0.488	1.302	0.508	0.513	1.481	0.185
1497	0.500	1.397	0.185	0.488	1.302	0.491	0.513	1.481	0.050
1498	0.497	1.380	0.142	0.512	1.453	0.478	0.516	1.498	0.262
1499	0.497	1.380	0.142	0.494	1.340	0.102	0.505	1.430	0.224
1500	0.513	1.481	0.385	0.477	1.227	0.102	0.518	1.514	0.224
1501	0.224	-0.373	0.450	0.102	-1.184	0.422	0.295	0.082	0.328
1502	0.184	-0.625	0.450	0.102	-1.184	0.462	0.295	0.082	0.492
1503	0.097	-1.181	0.328	0.424	0.888	0.273	0.187	-0.608	0.513
1504	0.503	1.413	0.185	0.459	1.114	0.149	0.505	1.430	0.224
1505	0.184	-0.625	0.467	0.272	-0.091	0.309	0.097	-1.181	0.097
1506	0.329	0.301	0.508	0.149	-0.882	0.193	0.050	-1.485	0.467
1507	0.261	-0.137	0.224	0.313	0.172	0.235	0.187	-0.608	0.501
1508	0.300	0.116	0.050	0.193	-0.600	0.341	0.050	-1.485	0.358
1509	0.184	-0.625	0.508	0.237	-0.317	0.422	0.187	-0.608	0.358
1510	0.145	-0.878	0.508	0.342	0.361	0.341	0.050	-1.485	0.467
1511	0.187	-0.608	0.508	0.421	0.869	0.341	0.263	-0.120	0.501
1512	0.142	-0.895	0.512	0.342	0.361	0.508	0.226	-0.356	0.492
1513	0.142	-0.895	0.481	0.342	0.361	0.422	0.226	-0.356	0.501
1514	0.387	0.672	0.050	0.509	1.434	0.512	0.329	0.301	0.492
1515	0.453	1.093	0.050	0.424	0.888	0.149	0.492	1.346	0.262
1516	0.450	1.076	0.512	0.509	1.434	0.273	0.513	1.481	0.385
1517	0.329	0.301	0.481	0.149	-0.882	0.501	0.224	-0.373	0.185
1518	0.184	-0.625	0.481	0.275	-0.073	0.512	0.097	-1.181	0.508
1519	0.468	1.194	0.224	0.503	1.396	0.102	0.466	1.178	0.492
1520	0.508	1.447	0.450	0.512	1.453	0.309	0.500	1.397	0.328
1521	0.224	-0.373	0.450	0.102	-1.184	0.501	0.358	0.487	0.262

1522	0.050	-1.485	0.481	0.310	0.153	0.491	0.361	0.504	0.512
1523	0.508	1.447	0.481	0.506	1.415	0.491	0.463	1.161	0.512
1524	0.508	1.447	0.501	0.497	1.359	0.512	0.497	1.380	0.512
1525	0.508	1.447	0.142	0.497	1.359	0.462	0.487	1.312	0.512
1526	0.513	1.481	0.481	0.509	1.434	0.422	0.505	1.430	0.492
1527	0.482	1.279	0.097	0.462	1.133	0.273	0.492	1.346	0.097
1528	0.050	-1.485	0.097	0.421	0.869	0.371	0.261	-0.137	0.508
1529	0.050	-1.485	0.508	0.275	-0.073	0.478	0.387	0.672	0.358
1530	0.000	?	0.224	0.371	0.549	0.398	0.184	-0.625	0.328
1531	0.511	1.464	0.409	0.474	1.208	0.512	0.503	1.413	0.450
1532	0.476	1.245	0.185	0.401	0.737	0.149	0.497	1.380	0.450
1533	0.482	1.279	0.296	0.515	1.472	0.512	0.329	0.301	0.097
1534	0.224	-0.373	0.142	0.152	-0.863	0.512	0.261	-0.137	0.262
1535	0.447	1.060	0.142	0.509	1.434	0.501	0.513	1.481	0.097
1536	0.447	1.060	0.512	0.509	1.434	0.501	0.513	1.481	0.328
1537	0.479	1.262	0.512	0.497	1.359	0.462	0.508	1.447	0.508
1538	0.479	1.262	0.224	0.497	1.359	0.309	0.508	1.447	0.481
1539	0.500	1.397	0.492	0.462	1.133	0.514	0.492	1.346	0.097
1540	0.142	-0.895	0.492	0.310	0.153	0.273	0.097	-1.181	0.431
1541	0.476	1.245	0.492	0.515	1.472	0.273	0.503	1.413	0.431
1542	0.097	-1.181	0.508	0.275	-0.073	0.514	0.358	0.487	0.431
1543	0.097	-1.181	0.512	0.275	-0.073	0.371	0.329	0.301	0.450
1544	0.500	1.397	0.508	0.515	1.472	0.508	0.450	1.076	0.481
1545	0.226	-0.356	0.296	0.371	0.549	0.273	0.000	?	0.501
1546	0.411	0.824	0.185	0.509	1.434	0.102	0.445	1.043	0.142
1547	0.187	-0.608	0.492	0.275	-0.073	0.309	0.097	-1.181	0.142
1548	0.295	0.082	0.508	0.102	-1.184	0.053	0.263	-0.120	0.501
1549	0.142	-0.895	0.262	0.307	0.135	0.341	0.097	-1.181	0.431
1550	0.142	-0.895	0.262	0.053	-1.504	0.491	0.329	0.301	0.262
1551	0.505	1.430	0.501	0.345	0.379	0.398	0.511	1.464	0.050
1552	0.513	1.481	0.492	0.491	1.321	0.501	0.484	1.295	0.513
1553	0.224	-0.373	0.296	0.401	0.737	0.514	0.097	-1.181	0.512
1554	0.487	1.312	0.328	0.503	1.396	0.514	0.426	0.925	0.050
1555	0.487	1.312	0.513	0.518	1.491	0.512	0.426	0.925	0.050
1556	0.487	1.312	0.262	0.518	1.491	0.462	0.426	0.925	0.501
1557	0.505	1.430	0.508	0.515	1.472	0.501	0.445	1.043	0.262
1558	0.508	1.447	0.508	0.462	1.133	0.235	0.484	1.295	0.501
1559	0.505	1.430	0.328	0.503	1.396	0.273	0.492	1.346	0.512
1560	0.297	0.099	0.481	0.237	-0.317	0.514	0.142	-0.895	0.185
1561	0.184	-0.625	0.296	0.275	-0.073	0.462	0.142	-0.895	0.185
1562	0.492	1.346	0.262	0.515	1.472	0.341	0.505	1.430	0.050
1563	0.508	1.447	0.262	0.456	1.095	0.235	0.429	0.942	0.492
1564	0.261	-0.137	0.450	0.342	0.361	0.508	0.266	-0.103	0.142
1565	0.261	-0.137	0.224	0.237	-0.317	0.501	0.050	-1.485	0.224
1566	0.503	1.413	0.224	0.506	1.415	0.193	0.511	1.464	0.262
1567	0.495	1.363	0.224	0.500	1.378	0.149	0.511	1.464	0.409
1568	0.297	0.099	0.185	0.193	-0.600	0.501	0.050	-1.485	0.262
1569	0.332	0.318	0.492	0.149	-0.882	0.193	0.050	-1.485	0.262
1570	0.508	1.447	0.492	0.497	1.359	0.514	0.503	1.413	0.185
1571	0.263	-0.120	0.262	0.196	-0.581	0.491	0.263	-0.120	0.513
1572	0.508	1.447	0.431	0.518	1.491	0.341	0.492	1.346	0.492
1573	0.508	1.447	0.262	0.491	1.321	0.341	0.505	1.430	0.050
1574	0.000	?	0.296	0.345	0.379	0.341	0.184	-0.625	0.481
1575	0.000	?	0.097	0.345	0.379	0.512	0.184	-0.625	0.142
1576	0.326	0.285	0.508	0.345	0.379	0.309	0.050	-1.485	0.296
1577	0.476	1.245	0.492	0.515	1.472	0.149	0.487	1.312	0.467
1578	0.297	0.099	0.492	0.310	0.153	0.193	0.142	-0.895	0.492
1579	0.263	-0.120	0.450	0.149	-0.882	0.512	0.226	-0.356	0.513
1580	0.263	-0.120	0.224	0.193	-0.600	0.053	0.263	-0.120	0.450
1581	0.450	1.076	0.512	0.509	1.434	0.102	0.408	0.807	0.492
1582	0.226	-0.356	0.508	0.053	-1.504	0.309	0.263	-0.120	0.358

1583	0.226	-0.356	0.481	0.102	-1.184	0.235	0.263	-0.120	0.481
1584	0.224	-0.373	0.296	0.307	0.135	0.444	0.000	?	0.492
1585	0.184	-0.625	0.481	0.234	-0.336	0.491	0.184	-0.625	0.467
1586	0.497	1.380	0.328	0.442	1.001	0.341	0.511	1.464	0.050
1587	0.492	1.346	0.501	0.488	1.302	0.491	0.487	1.312	0.512
1588	0.263	-0.120	0.481	0.342	0.361	0.193	0.050	-1.485	0.097
1589	0.429	0.942	0.385	0.491	1.321	0.193	0.484	1.295	0.467
1590	0.261	-0.137	0.358	0.196	-0.581	0.102	0.145	-0.878	0.501
1591	0.295	0.082	0.501	0.196	-0.581	0.508	0.300	0.116	0.513
1592	0.097	-1.181	0.262	0.102	-1.184	0.512	0.468	1.194	0.050
1593	0.508	1.447	0.262	0.500	1.378	0.512	0.492	1.346	0.450
1594	0.492	1.346	0.097	0.506	1.415	0.514	0.511	1.464	0.142
1595	0.495	1.363	0.501	0.518	1.491	0.102	0.445	1.043	0.050
1596	0.450	1.076	0.492	0.515	1.472	0.491	0.489	1.329	0.262
1597	0.224	-0.373	0.492	0.102	-1.184	0.491	0.358	0.487	0.508
1598	0.513	1.481	0.328	0.491	1.321	0.508	0.482	1.279	0.512
1599	0.508	1.447	0.481	0.491	1.321	0.235	0.492	1.346	0.512
1600	0.484	1.295	0.262	0.503	1.396	0.422	0.463	1.161	0.097
1601	0.297	0.099	0.097	0.234	-0.336	0.273	0.050	-1.485	0.501
1602	0.482	1.279	0.097	0.427	0.907	0.508	0.513	1.481	0.431
1603	0.332	0.318	0.492	0.272	-0.091	0.514	0.097	-1.181	0.385
1604	0.503	1.413	0.481	0.506	1.415	0.422	0.463	1.161	0.358
1605	0.484	1.295	0.142	0.515	1.472	0.235	0.505	1.430	0.492
1606	0.387	0.672	0.262	0.418	0.850	0.478	0.505	1.430	0.492
1607	0.358	0.487	0.467	0.234	-0.336	0.273	0.050	-1.485	0.262
1608	0.497	1.380	0.467	0.477	1.227	0.235	0.447	1.060	0.224
1609	0.263	-0.120	0.501	0.275	-0.073	0.235	0.142	-0.895	0.492
1610	0.263	-0.120	0.328	0.237	-0.317	0.478	0.050	-1.485	0.492
1611	0.097	-1.181	0.097	0.237	-0.317	0.501	0.261	-0.137	0.385
1612	0.492	1.346	0.050	0.482	1.265	0.501	0.505	1.430	0.224
1613	0.497	1.380	0.296	0.500	1.378	0.341	0.508	1.447	0.097
1614	0.497	1.380	0.512	0.500	1.378	0.491	0.508	1.447	0.185
1615	0.329	0.301	0.097	0.342	0.361	0.273	0.097	-1.181	0.050
1616	0.484	1.295	0.097	0.491	1.321	0.193	0.497	1.380	0.467
1617	0.263	-0.120	0.050	0.272	-0.091	0.193	0.434	0.975	0.185
1618	0.097	-1.181	0.224	0.193	-0.600	0.478	0.387	0.672	0.097
1619	0.097	-1.181	0.185	0.193	-0.600	0.462	0.358	0.487	0.142
1620	0.489	1.329	0.481	0.477	1.227	0.309	0.492	1.346	0.224
1621	0.479	1.262	0.481	0.465	1.152	0.149	0.492	1.346	0.328
1622	0.145	-0.878	0.097	0.310	0.153	0.462	0.263	-0.120	0.431
1623	0.263	-0.120	0.185	0.149	-0.882	0.462	0.226	-0.356	0.467
1624	0.468	1.194	0.262	0.462	1.133	0.508	0.489	1.329	0.097
1625	0.468	1.194	0.508	0.462	1.133	0.309	0.489	1.329	0.142
1626	0.497	1.380	0.262	0.503	1.396	0.462	0.389	0.689	0.097
1627	0.329	0.301	0.450	0.310	0.153	0.309	0.224	-0.373	0.501
1628	0.100	-1.164	0.467	0.462	1.133	0.235	0.097	-1.181	0.097
1629	0.050	-1.485	0.224	0.310	0.153	0.501	0.184	-0.625	0.508
1630	0.297	0.099	0.185	0.237	-0.317	0.273	0.050	-1.485	0.501
1631	0.505	1.430	0.185	0.500	1.378	0.398	0.463	1.161	0.097
1632	0.100	-1.164	0.142	0.272	-0.091	0.341	0.184	-0.625	0.262
1633	0.097	-1.181	0.142	0.398	0.718	0.422	0.097	-1.181	0.224
1634	0.050	-1.485	0.097	0.342	0.361	0.235	0.142	-0.895	0.328
1635	0.224	-0.373	0.224	0.421	0.869	0.508	0.224	-0.373	0.097
1636	0.184	-0.625	0.142	0.234	-0.336	0.478	0.332	0.318	0.262
1637	0.479	1.262	0.481	0.503	1.396	0.341	0.429	0.942	0.142
1638	0.487	1.312	0.508	0.477	1.227	0.273	0.461	1.144	0.224
1639	0.097	-1.181	0.409	0.345	0.379	0.193	0.097	-1.181	0.508
1640	0.184	-0.625	0.262	0.275	-0.073	0.462	0.142	-0.895	0.467
1641	0.263	-0.120	0.409	0.193	-0.600	0.462	0.097	-1.181	0.050
1642	0.505	1.430	0.492	0.462	1.133	0.478	0.495	1.363	0.097
1643	0.263	-0.120	0.185	0.465	1.152	0.444	0.097	-1.181	0.481

1644	0.447	1.060	0.512	0.477	1.227	0.341	0.505	1.430	0.501
1645	0.468	1.194	0.513	0.447	1.039	0.102	0.495	1.363	0.185
1646	0.224	-0.373	0.431	0.342	0.361	0.193	0.097	-1.181	0.467
1647	0.187	-0.608	0.328	0.102	-1.184	0.053	0.263	-0.120	0.467
1648	0.184	-0.625	0.224	0.193	-0.600	0.309	0.224	-0.373	0.501
1649	0.142	-0.895	0.296	0.053	-1.504	0.309	0.332	0.318	0.224
1650	0.142	-0.895	0.492	0.313	0.172	0.309	0.097	-1.181	0.224
1651	0.097	-1.181	0.501	0.313	0.172	0.273	0.261	-0.137	0.097
1652	0.226	-0.356	0.501	0.307	0.135	0.462	0.142	-0.895	0.467
1653	0.142	-0.895	0.481	0.272	-0.091	0.514	0.226	-0.356	0.492
1654	0.482	1.279	0.481	0.462	1.133	0.193	0.500	1.397	0.481
1655	0.503	1.413	0.431	0.512	1.453	0.235	0.463	1.161	0.358
1656	0.411	0.824	0.431	0.193	-0.600	0.514	0.050	-1.485	0.358
1657	0.263	-0.120	0.492	0.234	-0.336	0.462	0.097	-1.181	0.492
1658	0.411	0.824	0.328	0.515	1.472	0.309	0.482	1.279	0.481
1659	0.495	1.363	0.328	0.465	1.152	0.444	0.497	1.380	0.492
1660	0.184	-0.625	0.328	0.313	0.172	0.444	0.184	-0.625	0.097
1661	0.508	1.447	0.508	0.447	1.039	0.508	0.466	1.178	0.185
1662	0.511	1.464	0.358	0.447	1.039	0.193	0.466	1.178	0.097
1663	0.426	0.925	0.508	0.512	1.453	0.149	0.497	1.380	0.450
1664	0.329	0.301	0.450	0.193	-0.600	0.149	0.224	-0.373	0.097
1665	0.226	-0.356	0.508	0.152	-0.863	0.491	0.224	-0.373	0.481
1666	0.295	0.082	0.512	0.149	-0.882	0.508	0.097	-1.181	0.385
1667	0.492	1.346	0.328	0.488	1.302	0.508	0.466	1.178	0.512
1668	0.500	1.397	0.142	0.506	1.415	0.501	0.489	1.329	0.512
1669	0.500	1.397	0.358	0.506	1.415	0.501	0.476	1.245	0.224
1670	0.484	1.295	0.296	0.497	1.359	0.508	0.361	0.504	0.262
1671	0.484	1.295	0.296	0.497	1.359	0.508	0.361	0.504	0.097
1672	0.432	0.958	0.328	0.506	1.415	0.478	0.489	1.329	0.050
1673	0.432	0.958	0.142	0.506	1.415	0.149	0.479	1.262	0.050
1674	0.492	1.346	0.142	0.477	1.227	0.149	0.487	1.312	0.262
1675	0.332	0.318	0.224	0.149	-0.882	0.193	0.097	-1.181	0.262
1676	0.329	0.301	0.467	0.149	-0.882	0.491	0.184	-0.625	0.224
1677	0.326	0.285	0.358	0.193	-0.600	0.193	0.097	-1.181	0.142
1678	0.513	1.481	0.185	0.488	1.302	0.491	0.445	1.043	0.501
1679	0.358	0.487	0.512	0.193	-0.600	0.514	0.097	-1.181	0.501
1680	0.511	1.464	0.296	0.482	1.265	0.462	0.479	1.262	0.409
1681	0.447	1.060	0.224	0.520	1.509	0.478	0.387	0.672	0.450
1682	0.505	1.430	0.409	0.462	1.133	0.149	0.513	1.481	0.142
1683	0.508	1.447	0.097	0.480	1.246	0.149	0.513	1.481	0.262
1684	0.332	0.318	0.224	0.149	-0.882	0.341	0.224	-0.373	0.097
1685	0.142	-0.895	0.492	0.149	-0.882	0.341	0.263	-0.120	0.185
1686	0.358	0.487	0.512	0.339	0.342	0.193	0.097	-1.181	0.513
1687	0.295	0.082	0.512	0.342	0.361	0.053	0.050	-1.485	0.508
1688	0.297	0.099	0.050	0.193	-0.600	0.193	0.050	-1.485	0.508
1689	0.332	0.318	0.142	0.053	-1.504	0.193	0.261	-0.137	0.262
1690	0.142	-0.895	0.224	0.193	-0.600	0.193	0.263	-0.120	0.142
1691	0.142	-0.895	0.467	0.193	-0.600	0.444	0.224	-0.373	0.224
1692	0.226	-0.356	0.097	0.193	-0.600	0.444	0.142	-0.895	0.508
1693	0.468	1.194	0.262	0.444	1.020	0.371	0.495	1.363	0.224
1694	0.358	0.487	0.296	0.444	1.020	0.491	0.500	1.397	0.385
1695	0.184	-0.625	0.513	0.368	0.530	0.309	0.411	0.824	0.409
1696	0.513	1.481	0.185	0.500	1.378	0.273	0.450	1.076	0.508
1697	0.297	0.099	0.185	0.310	0.153	0.149	0.142	-0.895	0.097
1698	0.224	-0.373	0.142	0.275	-0.073	0.309	0.261	-0.137	0.224
1699	0.411	0.824	0.508	0.149	-0.882	0.309	0.097	-1.181	0.385
1700	0.097	-1.181	0.501	0.307	0.135	0.422	0.184	-0.625	0.513
1701	0.224	-0.373	0.508	0.313	0.172	0.398	0.000	?	0.512
1702	0.492	1.346	0.501	0.427	0.907	0.371	0.508	1.447	0.467
1703	0.513	1.481	0.185	0.406	0.775	0.371	0.503	1.413	0.492
1704	0.513	1.481	0.185	0.380	0.605	0.309	0.503	1.413	0.142

1705	0.050	-1.485	0.262	0.371	0.549	0.149	0.266	-0.103	0.142
1706	0.142	-0.895	0.224	0.307	0.135	0.512	0.142	-0.895	0.185
1707	0.224	-0.373	0.512	0.149	-0.882	0.309	0.224	-0.373	0.185
1708	0.466	1.178	0.142	0.515	1.472	0.422	0.497	1.380	0.492
1709	0.097	-1.181	0.097	0.307	0.135	0.273	0.224	-0.373	0.224
1710	0.263	-0.120	0.501	0.424	0.888	0.491	0.389	0.689	0.296
1711	0.297	0.099	0.262	0.275	-0.073	0.273	0.411	0.824	0.431
1712	0.500	1.397	0.481	0.494	1.340	0.149	0.513	1.481	0.185
1713	0.187	-0.608	0.409	0.275	-0.073	0.371	0.097	-1.181	0.508
1714	0.187	-0.608	0.296	0.149	-0.882	0.491	0.224	-0.373	0.467
1715	0.142	-0.895	0.431	0.377	0.587	0.341	0.384	0.655	0.142
1716	0.513	1.481	0.185	0.494	1.340	0.508	0.513	1.481	0.513
1717	0.505	1.430	0.358	0.342	0.361	0.444	0.513	1.481	0.262
1718	0.511	1.464	0.296	0.512	1.453	0.273	0.471	1.211	0.185
1719	0.497	1.380	0.492	0.442	1.001	0.235	0.492	1.346	0.050
1720	0.187	-0.608	0.185	0.272	-0.091	0.273	0.142	-0.895	0.508
1721	0.187	-0.608	0.262	0.234	-0.336	0.235	0.142	-0.895	0.097
1722	0.261	-0.137	0.262	0.275	-0.073	0.491	0.187	-0.608	0.142
1723	0.224	-0.373	0.358	0.237	-0.317	0.235	0.187	-0.608	0.185
1724	0.511	1.464	0.185	0.494	1.340	0.273	0.489	1.329	0.097
1725	0.142	-0.895	0.328	0.234	-0.336	0.422	0.224	-0.373	0.097
1726	0.097	-1.181	0.296	0.272	-0.091	0.193	0.297	0.099	0.385
1727	0.503	1.413	0.450	0.424	0.888	0.462	0.426	0.925	0.224
1728	0.261	-0.137	0.409	0.196	-0.581	0.512	0.184	-0.625	0.185
1729	0.479	1.262	0.097	0.462	1.133	0.273	0.511	1.464	0.508
1730	0.411	0.824	0.508	0.512	1.453	0.514	0.463	1.161	0.512
1731	0.295	0.082	0.508	0.275	-0.073	0.309	0.142	-0.895	0.296
1732	0.429	0.942	0.328	0.518	1.491	0.341	0.511	1.464	0.385
1733	0.187	-0.608	0.501	0.310	0.153	0.371	0.263	-0.120	0.385
1734	0.361	0.504	0.185	0.339	0.342	0.341	0.184	-0.625	0.185
1735	0.295	0.082	0.481	0.371	0.549	0.273	0.050	-1.485	0.492
1736	0.495	1.363	0.481	0.342	0.361	0.371	0.503	1.413	0.492
1737	0.187	-0.608	0.492	0.275	-0.073	0.341	0.097	-1.181	0.467
1738	0.263	-0.120	0.501	0.374	0.568	0.193	0.142	-0.895	0.501
1739	0.000	?	0.513	0.342	0.361	0.235	0.184	-0.625	0.512
1740	0.261	-0.137	0.512	0.193	-0.600	0.273	0.097	-1.181	0.481
1741	0.358	0.487	0.481	0.234	-0.336	0.193	0.097	-1.181	0.328
1742	0.184	-0.625	0.224	0.275	-0.073	0.309	0.384	0.655	0.492
1743	0.329	0.301	0.328	0.196	-0.581	0.444	0.224	-0.373	0.262
1744	0.297	0.099	0.142	0.307	0.135	0.512	0.184	-0.625	0.262
1745	0.450	1.076	0.262	0.444	1.020	0.235	0.505	1.430	0.142
1746	0.408	0.807	0.328	0.515	1.472	0.501	0.508	1.447	0.185
1747	0.097	-1.181	0.481	0.237	-0.317	0.501	0.297	0.099	0.262
1748	0.511	1.464	0.450	0.506	1.415	0.273	0.382	0.638	0.385
1749	0.511	1.464	0.097	0.506	1.415	0.501	0.382	0.638	0.185
1750	0.332	0.318	0.512	0.272	-0.091	0.398	0.184	-0.625	0.097
1751	0.497	1.380	0.512	0.506	1.415	0.422	0.484	1.295	0.224
1752	0.184	-0.625	0.512	0.401	0.737	0.508	0.000	?	0.512
1753	0.479	1.262	0.224	0.427	0.907	0.512	0.492	1.346	0.492
1754	0.482	1.279	0.224	0.503	1.396	0.491	0.466	1.178	0.358
1755	0.489	1.329	0.467	0.518	1.491	0.478	0.495	1.363	0.467
1756	0.503	1.413	0.450	0.488	1.302	0.491	0.503	1.413	0.467
1757	0.508	1.447	0.358	0.482	1.265	0.501	0.476	1.245	0.467
1758	0.513	1.481	0.467	0.491	1.321	0.273	0.326	0.285	0.142
1759	0.476	1.245	0.508	0.497	1.359	0.309	0.492	1.346	0.142
1760	0.000	?	0.185	0.275	-0.073	0.193	0.263	-0.120	0.431
1761	0.000	?	0.224	0.310	0.153	0.235	0.263	-0.120	0.431
1762	0.226	-0.356	0.501	0.193	-0.600	0.273	0.142	-0.895	0.328
1763	0.326	0.285	0.512	0.237	-0.317	0.273	0.184	-0.625	0.481
1764	0.142	-0.895	0.097	0.272	-0.091	0.193	0.261	-0.137	0.492
1765	0.261	-0.137	0.097	0.272	-0.091	0.422	0.387	0.672	0.185

1766	0.326	0.285	0.142	0.196	-0.581	0.444	0.184	-0.625	0.508
1767	0.000	?	0.481	0.424	0.888	0.491	0.097	-1.181	0.481
1768	0.000	?	0.224	0.444	1.020	0.422	0.224	-0.373	0.328
1769	0.479	1.262	0.467	0.494	1.340	0.309	0.505	1.430	0.185
1770	0.450	1.076	0.050	0.424	0.888	0.508	0.489	1.329	0.224
1771	0.097	-1.181	0.050	0.310	0.153	0.508	0.358	0.487	0.481
1772	0.508	1.447	0.224	0.509	1.434	0.508	0.466	1.178	0.142
1773	0.508	1.447	0.224	0.509	1.434	0.341	0.466	1.178	0.467
1774	0.508	1.447	0.185	0.509	1.434	0.309	0.466	1.178	0.224
1775	0.226	-0.356	0.358	0.339	0.342	0.514	0.145	-0.878	0.224
1776	0.224	-0.373	0.328	0.310	0.153	0.512	0.145	-0.878	0.224
1777	0.468	1.194	0.185	0.509	1.434	0.149	0.426	0.925	0.224
1778	0.450	1.076	0.508	0.506	1.415	0.512	0.426	0.925	0.262
1779	0.358	0.487	0.262	0.149	-0.882	0.508	0.326	0.285	0.385
1780	0.468	1.194	0.224	0.512	1.453	0.309	0.474	1.228	0.409
1781	0.503	1.413	0.508	0.506	1.415	0.309	0.495	1.363	0.262
1782	0.184	-0.625	0.296	0.307	0.135	0.491	0.184	-0.625	0.512
1783	0.226	-0.356	0.431	0.310	0.153	0.501	0.000	?	0.262
1784	0.497	1.380	0.512	0.485	1.283	0.193	0.513	1.481	0.185
1785	0.511	1.464	0.481	0.494	1.340	0.273	0.479	1.262	0.409
1786	0.097	-1.181	0.501	0.193	-0.600	0.193	0.329	0.301	0.385
1787	0.097	-1.181	0.467	0.275	-0.073	0.514	0.184	-0.625	0.513
1788	0.142	-0.895	0.385	0.193	-0.600	0.235	0.226	-0.356	0.481
1789	0.484	1.295	0.328	0.518	1.491	0.478	0.479	1.262	0.467
1790	0.224	-0.373	0.296	0.234	-0.336	0.309	0.145	-0.878	0.501
1791	0.466	1.178	0.385	0.477	1.227	0.341	0.466	1.178	0.431
1792	0.050	-1.485	0.512	0.310	0.153	0.193	0.226	-0.356	0.296
1793	0.050	-1.485	0.224	0.342	0.361	0.193	0.226	-0.356	0.296
1794	0.224	-0.373	0.262	0.193	-0.600	0.149	0.224	-0.373	0.262
1795	0.224	-0.373	0.224	0.193	-0.600	0.273	0.224	-0.373	0.467
1796	0.184	-0.625	0.512	0.149	-0.882	0.371	0.261	-0.137	0.508
1797	0.361	0.504	0.296	0.275	-0.073	0.193	0.384	0.655	0.296
1798	0.332	0.318	0.467	0.374	0.568	0.444	0.408	0.807	0.050
1799	0.184	-0.625	0.142	0.193	-0.600	0.341	0.263	-0.120	0.142
1800	0.508	1.447	0.097	0.444	1.020	0.193	0.516	1.498	0.431
1801	0.261	-0.137	0.492	0.342	0.361	0.462	0.263	-0.120	0.409
1802	0.224	-0.373	0.142	0.193	-0.600	0.273	0.187	-0.608	0.508
1803	0.508	1.447	0.508	0.465	1.152	0.501	0.413	0.841	0.262
1804	0.295	0.082	0.501	0.272	-0.091	0.398	0.384	0.655	0.296
1805	0.429	0.942	0.512	0.500	1.378	0.508	0.508	1.447	0.481
1806	0.508	1.447	0.262	0.398	0.718	0.491	0.479	1.262	0.224
1807	0.484	1.295	0.508	0.518	1.491	0.491	0.463	1.161	0.431
1808	0.500	1.397	0.481	0.494	1.340	0.149	0.503	1.413	0.492
1809	0.468	1.194	0.450	0.500	1.378	0.149	0.429	0.942	0.492
1810	0.387	0.672	0.262	0.149	-0.882	0.102	0.295	0.082	0.358
1811	0.326	0.285	0.142	0.149	-0.882	0.512	0.295	0.082	0.508
1812	0.295	0.082	0.050	0.102	-1.184	0.371	0.261	-0.137	0.467
1813	0.382	0.638	0.512	0.509	1.434	0.371	0.466	1.178	0.512
1814	0.511	1.464	0.185	0.368	0.530	0.371	0.508	1.447	0.262
1815	0.226	-0.356	0.185	0.371	0.549	0.193	0.295	0.082	0.262
1816	0.261	-0.137	0.142	0.368	0.530	0.444	0.050	-1.485	0.097
1817	0.224	-0.373	0.501	0.196	-0.581	0.235	0.142	-0.895	0.481
1818	0.508	1.447	0.492	0.439	0.982	0.501	0.432	0.958	0.097
1819	0.295	0.082	0.512	0.237	-0.317	0.193	0.411	0.824	0.224
1820	0.466	1.178	0.185	0.506	1.415	0.193	0.508	1.447	0.142
1821	0.142	-0.895	0.262	0.193	-0.600	0.508	0.266	-0.103	0.467
1822	0.097	-1.181	0.224	0.196	-0.581	0.273	0.295	0.082	0.492
1823	0.492	1.346	0.185	0.506	1.415	0.478	0.482	1.279	0.450
1824	0.142	-0.895	0.481	0.272	-0.091	0.508	0.229	-0.339	0.050
1825	0.508	1.447	0.142	0.480	1.246	0.508	0.432	0.958	0.142
1826	0.497	1.380	0.358	0.506	1.415	0.341	0.492	1.346	0.358

1827	0.508	1.447	0.142	0.506	1.415	0.398	0.492	1.346	0.385
1828	0.263	-0.120	0.262	0.339	0.342	0.371	0.361	0.504	0.492
1829	0.511	1.464	0.185	0.395	0.700	0.514	0.505	1.430	0.262
1830	0.479	1.262	0.513	0.368	0.530	0.149	0.468	1.194	0.050
1831	0.447	1.060	0.224	0.512	1.453	0.273	0.505	1.430	0.262
1832	0.261	-0.137	0.501	0.149	-0.882	0.371	0.263	-0.120	0.328
1833	0.142	-0.895	0.492	0.272	-0.091	0.444	0.266	-0.103	0.296
1834	0.050	-1.485	0.467	0.371	0.549	0.273	0.097	-1.181	0.501
1835	0.513	1.481	0.508	0.447	1.039	0.235	0.484	1.295	0.262
1836	0.184	-0.625	0.385	0.275	-0.073	0.341	0.097	-1.181	0.512
1837	0.184	-0.625	0.224	0.237	-0.317	0.512	0.224	-0.373	0.467
1838	0.142	-0.895	0.296	0.342	0.361	0.514	0.142	-0.895	0.492
1839	0.497	1.380	0.142	0.506	1.415	0.491	0.466	1.178	0.501
1840	0.487	1.312	0.142	0.509	1.434	0.309	0.492	1.346	0.328
1841	0.508	1.447	0.185	0.494	1.340	0.235	0.455	1.110	0.185
1842	0.184	-0.625	0.142	0.313	0.172	0.149	0.050	-1.485	0.185
1843	0.261	-0.137	0.508	0.237	-0.317	0.273	0.142	-0.895	0.262
1844	0.226	-0.356	0.142	0.149	-0.882	0.478	0.358	0.487	0.185
1845	0.184	-0.625	0.492	0.272	-0.091	0.309	0.389	0.689	0.328
1846	0.476	1.245	0.185	0.480	1.246	0.235	0.495	1.363	0.296
1847	0.142	-0.895	0.185	0.310	0.153	0.309	0.263	-0.120	0.492
1848	0.358	0.487	0.481	0.237	-0.317	0.273	0.050	-1.485	0.224
1849	0.142	-0.895	0.142	0.313	0.172	0.341	0.261	-0.137	0.450
1850	0.263	-0.120	0.512	0.275	-0.073	0.478	0.326	0.285	0.262
1851	0.187	-0.608	0.467	0.345	0.379	0.309	0.295	0.082	0.224
1852	0.513	1.481	0.512	0.482	1.265	0.491	0.503	1.413	0.431
1853	0.226	-0.356	0.097	0.313	0.172	0.462	0.261	-0.137	0.296
1854	0.503	1.413	0.224	0.497	1.359	0.444	0.511	1.464	0.358
1855	0.495	1.363	0.467	0.465	1.152	0.462	0.461	1.144	0.512
1856	0.466	1.178	0.512	0.450	1.057	0.371	0.487	1.312	0.385
1857	0.511	1.464	0.512	0.459	1.114	0.341	0.500	1.397	0.185
1858	0.387	0.672	0.224	0.374	0.568	0.341	0.329	0.301	0.262
1859	0.224	-0.373	0.262	0.348	0.398	0.149	0.184	-0.625	0.467
1860	0.295	0.082	0.512	0.348	0.398	0.371	0.184	-0.625	0.513
1861	0.142	-0.895	0.296	0.149	-0.882	0.193	0.261	-0.137	0.512
1862	0.142	-0.895	0.481	0.374	0.568	0.193	0.184	-0.625	0.224
1863	0.184	-0.625	0.224	0.196	-0.581	0.444	0.332	0.318	0.513
1864	0.142	-0.895	0.224	0.196	-0.581	0.193	0.297	0.099	0.262
1865	0.503	1.413	0.508	0.444	1.020	0.514	0.495	1.363	0.467
1866	0.142	-0.895	0.492	0.193	-0.600	0.149	0.224	-0.373	0.296
1867	0.484	1.295	0.501	0.509	1.434	0.273	0.453	1.093	0.224
1868	0.187	-0.608	0.508	0.149	-0.882	0.478	0.261	-0.137	0.501
1869	0.184	-0.625	0.185	0.272	-0.091	0.309	0.224	-0.373	0.501
1870	0.476	1.245	0.224	0.477	1.227	0.508	0.429	0.942	0.385
1871	0.145	-0.878	0.409	0.310	0.153	0.422	0.295	0.082	0.508
1872	0.503	1.413	0.328	0.509	1.434	0.478	0.358	0.487	0.097
1873	0.466	1.178	0.296	0.424	0.888	0.273	0.508	1.447	0.328
1874	0.513	1.481	0.501	0.480	1.246	0.102	0.384	0.655	0.513
1875	0.097	-1.181	0.328	0.275	-0.073	0.512	0.184	-0.625	0.385
1876	0.224	-0.373	0.097	0.102	-1.184	0.491	0.261	-0.137	0.185
1877	0.466	1.178	0.224	0.509	1.434	0.491	0.463	1.161	0.481
1878	0.511	1.464	0.185	0.485	1.283	0.309	0.511	1.464	0.097
1879	0.511	1.464	0.501	0.485	1.283	0.398	0.508	1.447	0.262
1880	0.226	-0.356	0.185	0.307	0.135	0.462	0.000	?	0.328
1881	0.261	-0.137	0.224	0.398	0.718	0.149	0.224	-0.373	0.262
1882	0.513	1.481	0.481	0.465	1.152	0.491	0.511	1.464	0.513
1883	0.300	0.116	0.508	0.149	-0.882	0.371	0.261	-0.137	0.185
1884	0.482	1.279	0.492	0.494	1.340	0.102	0.466	1.178	0.097
1885	0.226	-0.356	0.492	0.374	0.568	0.508	0.297	0.099	0.512
1886	0.226	-0.356	0.185	0.102	-1.184	0.478	0.224	-0.373	0.492
1887	0.503	1.413	0.097	0.509	1.434	0.491	0.503	1.413	0.492

1888	0.489	1.329	0.508	0.477	1.227	0.273	0.497	1.380	0.492
1889	0.497	1.380	0.328	0.488	1.302	0.273	0.382	0.638	0.328
1890	0.508	1.447	0.481	0.272	-0.091	0.341	0.500	1.397	0.185
1891	0.184	-0.625	0.450	0.272	-0.091	0.462	0.097	-1.181	0.508
1892	0.224	-0.373	0.358	0.342	0.361	0.193	0.332	0.318	0.097
1893	0.413	0.841	0.512	0.459	1.114	0.273	0.511	1.464	0.513
1894	0.329	0.301	0.481	0.193	-0.600	0.444	0.389	0.689	0.513
1895	0.295	0.082	0.512	0.275	-0.073	0.149	0.184	-0.625	0.050
1896	0.505	1.430	0.508	0.442	1.001	0.235	0.479	1.262	0.501
1897	0.332	0.318	0.501	0.149	-0.882	0.273	0.097	-1.181	0.467
1898	0.097	-1.181	0.328	0.237	-0.317	0.235	0.263	-0.120	0.450
1899	0.224	-0.373	0.467	0.272	-0.091	0.491	0.334	0.335	0.513
1900	0.184	-0.625	0.467	0.237	-0.317	0.235	0.263	-0.120	0.409
1901	0.503	1.413	0.262	0.494	1.340	0.235	0.516	1.498	0.142
1902	0.184	-0.625	0.512	0.237	-0.317	0.462	0.187	-0.608	0.481
1903	0.226	-0.356	0.296	0.237	-0.317	0.512	0.097	-1.181	0.481
1904	0.484	1.295	0.501	0.459	1.114	0.501	0.505	1.430	0.142
1905	0.508	1.447	0.142	0.509	1.434	0.501	0.487	1.312	0.512
1906	0.495	1.363	0.224	0.497	1.359	0.235	0.487	1.312	0.097
1907	0.495	1.363	0.142	0.497	1.359	0.273	0.487	1.312	0.467
1908	0.187	-0.608	0.262	0.237	-0.317	0.491	0.329	0.301	0.431
1909	0.097	-1.181	0.185	0.275	-0.073	0.149	0.184	-0.625	0.142
1910	0.508	1.447	0.296	0.494	1.340	0.398	0.508	1.447	0.185
1911	0.332	0.318	0.224	0.149	-0.882	0.512	0.097	-1.181	0.328
1912	0.482	1.279	0.481	0.401	0.737	0.235	0.503	1.413	0.224
1913	0.447	1.060	0.508	0.512	1.453	0.462	0.511	1.464	0.224
1914	0.358	0.487	0.467	0.234	-0.336	0.501	0.050	-1.485	0.262
1915	0.513	1.481	0.513	0.462	1.133	0.512	0.495	1.363	0.512
1916	0.484	1.295	0.467	0.500	1.378	0.398	0.466	1.178	0.492
1917	0.513	1.481	0.185	0.512	1.453	0.514	0.450	1.076	0.481
1918	0.503	1.413	0.097	0.398	0.718	0.235	0.508	1.447	0.481
1919	0.495	1.363	0.224	0.518	1.491	0.491	0.405	0.790	0.492
1920	0.326	0.285	0.492	0.234	-0.336	0.491	0.142	-0.895	0.296
1921	0.466	1.178	0.512	0.491	1.321	0.193	0.476	1.245	0.097
1922	0.466	1.178	0.296	0.491	1.321	0.508	0.476	1.245	0.296
1923	0.263	-0.120	0.501	0.193	-0.600	0.149	0.142	-0.895	0.481
1924	0.505	1.430	0.512	0.506	1.415	0.512	0.511	1.464	0.450
1925	0.295	0.082	0.185	0.149	-0.882	0.309	0.097	-1.181	0.097
1926	0.497	1.380	0.224	0.509	1.434	0.193	0.463	1.161	0.501
1927	0.142	-0.895	0.450	0.313	0.172	0.273	0.432	0.958	0.508
1928	0.226	-0.356	0.481	0.196	-0.581	0.149	0.142	-0.895	0.097
1929	0.142	-0.895	0.501	0.272	-0.091	0.149	0.184	-0.625	0.262
1930	0.261	-0.137	0.508	0.152	-0.863	0.371	0.332	0.318	0.501
1931	0.184	-0.625	0.358	0.149	-0.882	0.102	0.224	-0.373	0.450
1932	0.297	0.099	0.224	0.377	0.587	0.508	0.224	-0.373	0.450
1933	0.224	-0.373	0.481	0.102	-1.184	0.501	0.261	-0.137	0.385
1934	0.479	1.262	0.409	0.506	1.415	0.501	0.511	1.464	0.409
1935	0.505	1.430	0.296	0.497	1.359	0.491	0.489	1.329	0.097
1936	0.463	1.161	0.185	0.500	1.378	0.501	0.476	1.245	0.492
1937	0.508	1.447	0.185	0.488	1.302	0.102	0.479	1.262	0.409
1938	0.463	1.161	0.262	0.497	1.359	0.341	0.489	1.329	0.262
1939	0.184	-0.625	0.262	0.102	-1.184	0.102	0.297	0.099	0.224
1940	0.097	-1.181	0.185	0.345	0.379	0.462	0.097	-1.181	0.142
1941	0.224	-0.373	0.512	0.102	-1.184	0.514	0.295	0.082	0.224
1942	0.495	1.363	0.501	0.462	1.133	0.235	0.474	1.228	0.224
1943	0.508	1.447	0.512	0.515	1.472	0.491	0.445	1.043	0.185
1944	0.297	0.099	0.501	0.237	-0.317	0.501	0.097	-1.181	0.508
1945	0.500	1.397	0.481	0.488	1.302	0.309	0.503	1.413	0.431
1946	0.511	1.464	0.492	0.500	1.378	0.341	0.508	1.447	0.481
1947	0.187	-0.608	0.224	0.307	0.135	0.462	0.097	-1.181	0.513
1948	0.224	-0.373	0.508	0.342	0.361	0.491	0.261	-0.137	0.450

1949	0.450	1.076	0.431	0.465	1.152	0.462	0.500	1.397	0.450
1950	0.479	1.262	0.450	0.491	1.321	0.501	0.445	1.043	0.097
1951	0.505	1.430	0.450	0.465	1.152	0.491	0.450	1.076	0.512
1952	0.508	1.447	0.409	0.503	1.396	0.341	0.387	0.672	0.512
1953	0.358	0.487	0.409	0.485	1.283	0.444	0.411	0.824	0.450
1954	0.224	-0.373	0.513	0.339	0.342	0.508	0.097	-1.181	0.512
1955	0.482	1.279	0.224	0.447	1.039	0.341	0.489	1.329	0.492
1956	0.408	0.807	0.142	0.506	1.415	0.309	0.405	0.790	0.492
1957	0.297	0.099	0.492	0.342	0.361	0.371	0.263	-0.120	0.508
1958	0.184	-0.625	0.481	0.310	0.153	0.309	0.224	-0.373	0.262
1959	0.184	-0.625	0.481	0.371	0.549	0.341	0.142	-0.895	0.097
1960	0.261	-0.137	0.185	0.307	0.135	0.273	0.226	-0.356	0.508
1961	0.261	-0.137	0.224	0.339	0.342	0.398	0.226	-0.356	0.513
1962	0.184	-0.625	0.224	0.278	-0.054	0.508	0.184	-0.625	0.513
1963	0.511	1.464	0.467	0.398	0.718	0.422	0.500	1.397	0.097
1964	0.497	1.380	0.467	0.503	1.396	0.501	0.429	0.942	0.142
1965	0.511	1.464	0.501	0.418	0.850	0.444	0.476	1.245	0.142
1966	0.495	1.363	0.512	0.497	1.359	0.462	0.513	1.481	0.508
1967	0.482	1.279	0.050	0.444	1.020	0.273	0.447	1.060	0.508
1968	0.489	1.329	0.050	0.462	1.133	0.478	0.447	1.060	0.512
1969	0.224	-0.373	0.467	0.275	-0.073	0.508	0.097	-1.181	0.501
1970	0.508	1.447	0.409	0.480	1.246	0.501	0.500	1.397	0.142
1971	0.432	0.958	0.512	0.509	1.434	0.508	0.511	1.464	0.142
1972	0.450	1.076	0.492	0.500	1.378	0.512	0.450	1.076	0.481
1973	0.453	1.093	0.481	0.509	1.434	0.512	0.511	1.464	0.481
1974	0.408	0.807	0.512	0.512	1.453	0.514	0.489	1.329	0.450
1975	0.408	0.807	0.508	0.512	1.453	0.102	0.489	1.329	0.512
1976	0.511	1.464	0.508	0.520	1.509	0.309	0.511	1.464	0.508
1977	0.224	-0.373	0.512	0.102	-1.184	0.501	0.263	-0.120	0.450
1978	0.142	-0.895	0.513	0.307	0.135	0.512	0.097	-1.181	0.512
1979	0.489	1.329	0.512	0.503	1.396	0.512	0.505	1.430	0.512
1980	0.476	1.245	0.358	0.509	1.434	0.273	0.511	1.464	0.467
1981	0.476	1.245	0.262	0.509	1.434	0.235	0.511	1.464	0.467
1982	0.184	-0.625	0.508	0.275	-0.073	0.235	0.097	-1.181	0.492
1983	0.226	-0.356	0.224	0.237	-0.317	0.512	0.142	-0.895	0.097
1984	0.226	-0.356	0.185	0.237	-0.317	0.508	0.142	-0.895	0.185
1985	0.463	1.161	0.224	0.515	1.472	0.491	0.505	1.430	0.450
1986	0.463	1.161	0.409	0.506	1.415	0.501	0.503	1.413	0.328
1987	0.508	1.447	0.508	0.494	1.340	0.341	0.508	1.447	0.409
1988	0.513	1.481	0.481	0.503	1.396	0.371	0.495	1.363	0.328
1989	0.050	-1.485	0.262	0.342	0.361	0.478	0.142	-0.895	0.513
1990	0.050	-1.485	0.262	0.374	0.568	0.462	0.142	-0.895	0.512
1991	0.466	1.178	0.481	0.474	1.208	0.508	0.482	1.279	0.508
1992	0.411	0.824	0.467	0.459	1.114	0.508	0.482	1.279	0.142
1993	0.511	1.464	0.481	0.506	1.415	0.512	0.453	1.093	0.142
1994	0.487	1.312	0.185	0.509	1.434	0.508	0.516	1.498	0.450
1995	0.476	1.245	0.501	0.512	1.453	0.508	0.511	1.464	0.450
1996	0.511	1.464	0.501	0.506	1.415	0.462	0.445	1.043	0.508
1997	0.505	1.430	0.492	0.506	1.415	0.512	0.511	1.464	0.262
1998	0.505	1.430	0.513	0.465	1.152	0.444	0.516	1.498	0.501
1999	0.511	1.464	0.142	0.512	1.453	0.508	0.463	1.161	0.501
2000	0.511	1.464	0.224	0.442	1.001	0.273	0.461	1.144	0.501
2001	0.508	1.447	0.224	0.509	1.434	0.193	0.489	1.329	0.492
2002	0.358	0.487	0.224	0.272	-0.091	0.514	0.097	-1.181	0.142
2003	0.263	-0.120	0.262	0.193	-0.600	0.235	0.187	-0.608	0.142
2004	0.511	1.464	0.508	0.509	1.434	0.371	0.450	1.076	0.142
2005	0.226	-0.356	0.492	0.237	-0.317	0.193	0.329	0.301	0.142
2006	0.184	-0.625	0.492	0.371	0.549	0.444	0.411	0.824	0.097
2007	0.224	-0.373	0.296	0.196	-0.581	0.501	0.332	0.318	0.450
2008	0.408	0.807	0.224	0.444	1.020	0.501	0.513	1.481	0.501
2009	0.505	1.430	0.296	0.500	1.378	0.235	0.513	1.481	0.481

2010	0.482	1.279	0.296	0.503	1.396	0.235	0.500	1.397	0.097
2011	0.266	−0.103	0.224	0.234	−0.336	0.478	0.142	−0.895	0.097
2012	0.266	−0.103	0.328	0.234	−0.336	0.512	0.142	−0.895	0.142
2013	0.482	1.279	0.262	0.474	1.208	0.462	0.445	1.043	0.050
2014	0.468	1.194	0.450	0.506	1.415	0.149	0.445	1.043	0.097
2015	0.482	1.279	0.450	0.462	1.133	0.462	0.503	1.413	0.467
2016	0.184	−0.625	0.508	0.149	−0.882	0.462	0.266	−0.103	0.467
2017	0.495	1.363	0.262	0.462	1.133	0.508	0.500	1.397	0.508
2018	0.495	1.363	0.328	0.462	1.133	0.501	0.500	1.397	0.224
2019	0.489	1.329	0.450	0.506	1.415	0.341	0.503	1.413	0.142
2020	0.511	1.464	0.185	0.500	1.378	0.235	0.495	1.363	0.513
2021	0.142	−0.895	0.513	0.342	0.361	0.235	0.142	−0.895	0.142
2022	0.224	−0.373	0.358	0.234	−0.336	0.235	0.142	−0.895	0.492
2023	0.224	−0.373	0.224	0.234	−0.336	0.309	0.142	−0.895	0.142
2024	0.224	−0.373	0.262	0.234	−0.336	0.508	0.142	−0.895	0.050
2025	0.263	−0.120	0.492	0.310	0.153	0.444	0.097	−1.181	0.508
2026	0.505	1.430	0.492	0.509	1.434	0.478	0.445	1.043	0.467
2027	0.492	1.346	0.296	0.442	1.001	0.273	0.495	1.363	0.185
2028	0.489	1.329	0.097	0.477	1.227	0.371	0.476	1.245	0.262
2029	0.297	0.099	0.513	0.275	−0.073	0.273	0.097	−1.181	0.492
2030	0.224	−0.373	0.224	0.368	0.530	0.273	0.097	−1.181	0.450
2031	0.297	0.099	0.492	0.272	−0.091	0.235	0.000	?	0.185
2032	0.297	0.099	0.262	0.272	−0.091	0.193	0.000	?	0.450
2033	0.224	−0.373	0.513	0.237	−0.317	0.235	0.142	−0.895	0.450
2034	0.329	0.301	0.492	0.196	−0.581	0.508	0.050	−1.485	0.142
2035	0.261	−0.137	0.262	0.240	−0.299	0.508	0.097	−1.181	0.492
2036	0.450	1.076	0.481	0.509	1.434	0.444	0.461	1.144	0.492
2037	0.450	1.076	0.481	0.509	1.434	0.149	0.461	1.144	0.492
2038	0.508	1.447	0.481	0.439	0.982	0.102	0.505	1.430	0.142
2039	0.261	−0.137	0.328	0.149	−0.882	0.512	0.226	−0.356	0.050
2040	0.329	0.301	0.328	0.102	−1.184	0.235	0.142	−0.895	0.508
2041	0.450	1.076	0.481	0.509	1.434	0.491	0.516	1.498	0.142
2042	0.184	−0.625	0.296	0.237	−0.317	0.193	0.142	−0.895	0.142
2043	0.513	1.481	0.296	0.488	1.302	0.235	0.484	1.295	0.450
2044	0.355	0.470	0.481	0.196	−0.581	0.235	0.000	?	0.328
2045	0.224	−0.373	0.358	0.234	−0.336	0.508	0.142	−0.895	0.262
2046	0.261	−0.137	0.185	0.234	−0.336	0.462	0.050	−1.485	0.050
2047	0.492	1.346	0.358	0.500	1.378	0.235	0.505	1.430	0.262
2048	0.489	1.329	0.262	0.465	1.152	0.341	0.461	1.144	0.097
2049	0.295	0.082	0.328	0.237	−0.317	0.478	0.187	−0.608	0.508
2050	0.097	−1.181	0.481	0.342	0.361	0.371	0.263	−0.120	0.512
2051	0.513	1.481	0.481	0.477	1.227	0.462	0.487	1.312	0.481
2052	0.226	−0.356	0.431	0.371	0.549	0.235	0.000	?	0.262
2053	0.495	1.363	0.262	0.462	1.133	0.501	0.445	1.043	0.492
2054	0.261	−0.137	0.431	0.237	−0.317	0.501	0.184	−0.625	0.224
2055	0.511	1.464	0.097	0.503	1.396	0.309	0.445	1.043	0.508
2056	0.495	1.363	0.481	0.500	1.378	0.508	0.445	1.043	0.097
2057	0.261	−0.137	0.328	0.307	0.135	0.508	0.142	−0.895	0.481
2058	0.482	1.279	0.501	0.509	1.434	0.508	0.489	1.329	0.481
2059	0.482	1.279	0.492	0.509	1.434	0.235	0.489	1.329	0.262
2060	0.482	1.279	0.328	0.509	1.434	0.149	0.489	1.329	0.501
2061	0.329	0.301	0.501	0.234	−0.336	0.398	0.142	−0.895	0.142
2062	0.332	0.318	0.142	0.149	−0.882	0.273	0.050	−1.485	0.142
2063	0.484	1.295	0.296	0.395	0.700	0.235	0.500	1.397	0.050
2064	0.300	0.116	0.262	0.272	−0.091	0.512	0.142	−0.895	0.185
2065	0.300	0.116	0.224	0.234	−0.336	0.235	0.142	−0.895	0.097
2066	0.482	1.279	0.328	0.503	1.396	0.149	0.447	1.060	0.097
2067	0.355	0.470	0.328	0.240	−0.299	0.193	0.332	0.318	0.501
2068	0.187	−0.608	0.501	0.149	−0.882	0.193	0.261	−0.137	0.481
2069	0.361	0.504	0.513	0.193	−0.600	0.273	0.050	−1.485	0.050
2070	0.263	−0.120	0.262	0.196	−0.581	0.462	0.263	−0.120	0.050

2071	0.332	0.318	0.224	0.272	−0.091	0.508	0.097	−1.181	0.097
2072	0.476	1.245	0.185	0.465	1.152	0.462	0.500	1.397	0.142
2073	0.484	1.295	0.262	0.512	1.453	0.102	0.500	1.397	0.142
2074	0.432	0.958	0.262	0.465	1.152	0.501	0.474	1.228	0.481
2075	0.263	−0.120	0.262	0.102	−1.184	0.235	0.261	−0.137	0.501
2076	0.429	0.942	0.492	0.497	1.359	0.501	0.489	1.329	0.481
2077	0.097	−1.181	0.492	0.234	−0.336	0.149	0.226	−0.356	0.050
2078	0.487	1.312	0.512	0.500	1.378	0.501	0.500	1.397	0.296
2079	0.329	0.301	0.296	0.149	−0.882	0.501	0.097	−1.181	0.296
2080	0.505	1.430	0.142	0.494	1.340	0.149	0.474	1.228	0.296
2081	0.495	1.363	0.142	0.500	1.378	0.508	0.474	1.228	0.224
2082	0.329	0.301	0.185	0.149	−0.882	0.273	0.263	−0.120	0.262
2083	0.500	1.397	0.185	0.509	1.434	0.102	0.492	1.346	0.508
2084	0.145	−0.878	0.185	0.275	−0.073	0.273	0.142	−0.895	0.450
2085	0.300	0.116	0.508	0.102	−1.184	0.193	0.142	−0.895	0.185
2086	0.266	−0.103	0.501	0.272	−0.091	0.193	0.050	−1.485	0.050
2087	0.226	−0.356	0.224	0.196	−0.581	0.102	0.184	−0.625	0.142
2088	0.329	0.301	0.328	0.193	−0.600	0.501	0.097	−1.181	0.097
2089	0.332	0.318	0.262	0.102	−1.184	0.501	0.097	−1.181	0.142
2090	0.497	1.380	0.262	0.497	1.359	0.235	0.503	1.413	0.492
2091	0.513	1.481	0.262	0.503	1.396	0.309	0.482	1.279	0.467
2092	0.263	−0.120	0.492	0.234	−0.336	0.309	0.050	−1.485	0.296
2093	0.224	−0.373	0.492	0.313	0.172	0.193	0.000	?	0.492
2094	0.184	−0.625	0.185	0.313	0.172	0.193	0.050	−1.485	0.492
2095	0.263	−0.120	0.501	0.193	−0.600	0.193	0.097	−1.181	0.097
2096	0.261	−0.137	0.450	0.193	−0.600	0.422	0.142	−0.895	0.450
2097	0.261	−0.137	0.224	0.193	−0.600	0.478	0.142	−0.895	0.142
2098	0.487	1.312	0.409	0.424	0.888	0.462	0.476	1.245	0.097
2099	0.484	1.295	0.409	0.477	1.227	0.193	0.503	1.413	0.431
2100	0.511	1.464	0.185	0.462	1.133	0.102	0.474	1.228	0.050
2101	0.295	0.082	0.492	0.193	−0.600	0.102	0.050	−1.485	0.501
2102	0.142	−0.895	0.224	0.102	−1.184	0.193	0.300	0.116	0.142
2103	0.142	−0.895	0.431	0.102	−1.184	0.235	0.300	0.116	0.097
2104	0.187	−0.608	0.262	0.193	−0.600	0.235	0.297	0.099	0.501
2105	0.187	−0.608	0.328	0.234	−0.336	0.422	0.226	−0.356	0.142
2106	0.187	−0.608	0.224	0.234	−0.336	0.491	0.263	−0.120	0.185
2107	0.505	1.430	0.492	0.421	0.869	0.149	0.503	1.413	0.512
2108	0.500	1.397	0.142	0.491	1.321	0.193	0.447	1.060	0.097
2109	0.226	−0.356	0.142	0.149	−0.882	0.149	0.184	−0.625	0.481
2110	0.329	0.301	0.467	0.193	−0.600	0.193	0.050	−1.485	0.142
2111	0.263	−0.120	0.224	0.149	−0.882	0.193	0.142	−0.895	0.142
2112	0.263	−0.120	0.467	0.193	−0.600	0.478	0.097	−1.181	0.097
2113	0.263	−0.120	0.296	0.193	−0.600	0.491	0.142	−0.895	0.508
2114	0.489	1.329	0.262	0.477	1.227	0.102	0.487	1.312	0.142
2115	0.492	1.346	0.296	0.491	1.321	0.462	0.461	1.144	0.142
2116	0.184	−0.625	0.501	0.102	−1.184	0.462	0.295	0.082	0.481
2117	0.500	1.397	0.224	0.456	1.095	0.273	0.489	1.329	0.185
2118	0.450	1.076	0.142	0.459	1.114	0.501	0.487	1.312	0.409
2119	0.224	−0.373	0.467	0.272	−0.091	0.149	0.097	−1.181	0.185
2120	0.413	0.841	0.185	0.494	1.340	0.273	0.445	1.043	0.450
2121	0.408	0.807	0.450	0.149	−0.882	0.491	0.142	−0.895	0.481
2122	0.184	−0.625	0.185	0.275	−0.073	0.273	0.097	−1.181	0.512
2123	0.497	1.380	0.512	0.485	1.283	0.501	0.426	0.925	0.050
2124	0.226	−0.356	0.508	0.275	−0.073	0.149	0.050	−1.485	0.050
2125	0.432	0.958	0.409	0.500	1.378	0.273	0.492	1.346	0.142
2126	0.261	−0.137	0.358	0.149	−0.882	0.309	0.142	−0.895	0.224
2127	0.326	0.285	0.358	0.275	−0.073	0.462	0.097	−1.181	0.050
2128	0.226	−0.356	0.224	0.307	0.135	0.273	0.000	?	0.481
2129	0.492	1.346	0.097	0.456	1.095	0.273	0.497	1.380	0.097
2130	0.142	−0.895	0.328	0.272	−0.091	0.501	0.142	−0.895	0.501
2131	0.142	−0.895	0.512	0.272	−0.091	0.398	0.184	−0.625	0.050

2132	0.466	1.178	0.185	0.506	1.415	0.491	0.508	1.447	0.467
2133	0.226	-0.356	0.431	0.401	0.737	0.193	0.097	-1.181	0.097
2134	0.468	1.194	0.262	0.491	1.321	0.193	0.474	1.228	0.050
2135	0.297	0.099	0.358	0.193	-0.600	0.193	0.142	-0.895	0.097
2136	0.263	-0.120	0.492	0.193	-0.600	0.508	0.142	-0.895	0.481
2137	0.297	0.099	0.224	0.193	-0.600	0.309	0.097	-1.181	0.508
2138	0.500	1.397	0.224	0.509	1.434	0.273	0.503	1.413	0.097
2139	0.224	-0.373	0.185	0.313	0.172	0.501	0.142	-0.895	0.142
2140	0.142	-0.895	0.492	0.278	-0.054	0.235	0.142	-0.895	0.097
2141	0.466	1.178	0.512	0.497	1.359	0.508	0.476	1.245	0.508
2142	0.184	-0.625	0.185	0.234	-0.336	0.193	0.184	-0.625	0.262
2143	0.450	1.076	0.224	0.509	1.434	0.462	0.405	0.790	0.097
2144	0.187	-0.608	0.224	0.193	-0.600	0.478	0.187	-0.608	0.097
2145	0.511	1.464	0.481	0.462	1.133	0.508	0.447	1.060	0.142
2146	0.511	1.464	0.296	0.474	1.208	0.149	0.476	1.245	0.508
2147	0.408	0.807	0.185	0.506	1.415	0.149	0.505	1.430	0.050
2148	0.358	0.487	0.224	0.149	-0.882	0.235	0.050	-1.485	0.481
2149	0.358	0.487	0.185	0.149	-0.882	0.235	0.050	-1.485	0.142
2150	0.226	-0.356	0.481	0.237	-0.317	0.193	0.142	-0.895	0.492
2151	0.097	-1.181	0.262	0.234	-0.336	0.462	0.224	-0.373	0.185
2152	0.329	0.301	0.481	0.193	-0.600	0.341	0.050	-1.485	0.224
2153	0.513	1.481	0.185	0.462	1.133	0.514	0.474	1.228	0.050
2154	0.184	-0.625	0.481	0.342	0.361	0.235	0.097	-1.181	0.097
2155	0.429	0.942	0.296	0.512	1.453	0.235	0.497	1.380	0.097
2156	0.263	-0.120	0.224	0.234	-0.336	0.491	0.050	-1.485	0.050
2157	0.355	0.470	0.262	0.234	-0.336	0.273	0.000	?	0.508
2158	0.489	1.329	0.185	0.491	1.321	0.273	0.461	1.144	0.097
2159	0.224	-0.373	0.262	0.275	-0.073	0.398	0.097	-1.181	0.501
2160	0.224	-0.373	0.296	0.275	-0.073	0.512	0.050	-1.485	0.262
2161	0.184	-0.625	0.262	0.401	0.737	0.398	0.097	-1.181	0.513
2162	0.492	1.346	0.492	0.512	1.453	0.309	0.476	1.245	0.185
2163	0.511	1.464	0.224	0.395	0.700	0.309	0.503	1.413	0.142
2164	0.184	-0.625	0.296	0.310	0.153	0.235	0.097	-1.181	0.512
2165	0.224	-0.373	0.431	0.310	0.153	0.478	0.145	-0.878	0.097
2166	0.224	-0.373	0.296	0.237	-0.317	0.273	0.100	-1.164	0.385
2167	0.482	1.279	0.508	0.474	1.208	0.273	0.503	1.413	0.224
2168	0.297	0.099	0.262	0.000	?	0.273	0.261	-0.137	0.492
2169	0.187	-0.608	0.185	0.272	-0.091	0.508	0.097	-1.181	0.142
2170	0.224	-0.373	0.481	0.272	-0.091	0.273	0.097	-1.181	0.050
2171	0.184	-0.625	0.296	0.275	-0.073	0.512	0.142	-0.895	0.492
2172	0.482	1.279	0.513	0.506	1.415	0.235	0.500	1.397	0.142
2173	0.261	-0.137	0.262	0.275	-0.073	0.501	0.050	-1.485	0.224
2174	0.479	1.262	0.097	0.512	1.453	0.193	0.474	1.228	0.492
2175	0.184	-0.625	0.501	0.237	-0.317	0.149	0.142	-0.895	0.224
2176	0.482	1.279	0.224	0.497	1.359	0.309	0.487	1.312	0.262
2177	0.295	0.082	0.385	0.193	-0.600	0.309	0.187	-0.608	0.358
2178	0.224	-0.373	0.409	0.152	-0.863	0.193	0.224	-0.373	0.358
2179	0.261	-0.137	0.224	0.307	0.135	0.193	0.000	?	0.224
2180	0.184	-0.625	0.481	0.310	0.153	0.273	0.050	-1.485	0.142
2181	0.263	-0.120	0.296	0.193	-0.600	0.478	0.097	-1.181	0.185
2182	0.297	0.099	0.185	0.193	-0.600	0.309	0.097	-1.181	0.185
2183	0.263	-0.120	0.512	0.275	-0.073	0.149	0.050	-1.485	0.224
2184	0.489	1.329	0.142	0.480	1.246	0.508	0.500	1.397	0.328
2185	0.226	-0.356	0.224	0.310	0.153	0.193	0.000	?	0.467
2186	0.295	0.082	0.501	0.149	-0.882	0.462	0.097	-1.181	0.501
2187	0.429	0.942	0.501	0.509	1.434	0.102	0.500	1.397	0.431
2188	0.300	0.116	0.296	0.193	-0.600	0.273	0.263	-0.120	0.262
2189	0.511	1.464	0.224	0.459	1.114	0.512	0.503	1.413	0.185
2190	0.261	-0.137	0.224	0.102	-1.184	0.193	0.184	-0.625	0.467
2191	0.187	-0.608	0.142	0.275	-0.073	0.514	0.142	-0.895	0.481
2192	0.487	1.312	0.142	0.509	1.434	0.273	0.503	1.413	0.467

2193	0.300	0.116	0.262	0.193	−0.600	0.309	0.097	−1.181	0.450
2194	0.516	1.498	0.467	0.509	1.434	0.491	0.387	0.672	0.328
2195	0.261	−0.137	0.481	0.272	−0.091	0.341	0.000	?	0.185
2196	0.097	−1.181	0.467	0.307	0.135	0.102	0.224	−0.373	0.385
2197	0.500	1.397	0.328	0.491	1.321	0.149	0.484	1.295	0.224
2198	0.226	−0.356	0.385	0.342	0.361	0.273	0.142	−0.895	0.224
2199	0.387	0.672	0.492	0.102	−1.184	0.462	0.000	?	0.142
2200	0.411	0.824	0.501	0.149	−0.882	0.149	0.000	?	0.142
2201	0.226	−0.356	0.481	0.275	−0.073	0.235	0.050	−1.485	0.185
2202	0.479	1.262	0.501	0.462	1.133	0.462	0.487	1.312	0.050
2203	0.297	0.099	0.097	0.149	−0.882	0.193	0.145	−0.878	0.296
2204	0.184	−0.625	0.262	0.240	−0.299	0.149	0.224	−0.373	0.296
2205	0.513	1.481	0.050	0.459	1.114	0.501	0.487	1.312	0.481
2206	0.142	−0.895	0.262	0.193	−0.600	0.501	0.226	−0.356	0.262
2207	0.226	−0.356	0.262	0.149	−0.882	0.309	0.263	−0.120	0.142
2208	0.495	1.363	0.431	0.500	1.378	0.371	0.355	0.470	0.262
2209	0.495	1.363	0.224	0.500	1.378	0.371	0.355	0.470	0.185
2210	0.295	0.082	0.185	0.310	0.153	0.273	0.224	−0.373	0.508
2211	0.229	−0.339	0.224	0.371	0.549	0.273	0.142	−0.895	0.097
2212	0.229	−0.339	0.224	0.371	0.549	0.235	0.184	−0.625	0.142
2213	0.142	−0.895	0.262	0.272	−0.091	0.478	0.187	−0.608	0.492
2214	0.145	−0.878	0.508	0.272	−0.091	0.371	0.224	−0.373	0.262
2215	0.263	−0.120	0.358	0.234	−0.336	0.398	0.332	0.318	0.508
2216	0.463	1.161	0.296	0.474	1.208	0.309	0.468	1.194	0.142
2217	0.476	1.245	0.142	0.374	0.568	0.102	0.497	1.380	0.097
2218	0.466	1.178	0.142	0.401	0.737	0.491	0.426	0.925	0.501
2219	0.332	0.318	0.467	0.307	0.135	0.478	0.261	−0.137	0.224
2220	0.387	0.672	0.262	0.102	−1.184	0.491	0.187	−0.608	0.262
2221	0.495	1.363	0.262	0.488	1.302	0.444	0.466	1.178	0.262
2222	0.500	1.397	0.492	0.482	1.265	0.273	0.479	1.262	0.185
2223	0.479	1.262	0.296	0.494	1.340	0.235	0.466	1.178	0.050
2224	0.497	1.380	0.467	0.444	1.020	0.273	0.447	1.060	0.097
2225	0.097	−1.181	0.296	0.272	−0.091	0.102	0.332	0.318	0.513
2226	0.261	−0.137	0.328	0.237	−0.317	0.102	0.187	−0.608	0.481
2227	0.050	−1.485	0.450	0.272	−0.091	0.235	0.387	0.672	0.262
2228	0.263	−0.120	0.185	0.102	−1.184	0.235	0.224	−0.373	0.185
2229	0.263	−0.120	0.185	0.102	−1.184	0.309	0.224	−0.373	0.508
2230	0.432	0.958	0.185	0.234	−0.336	0.273	0.142	−0.895	0.481
2231	0.224	−0.373	0.296	0.234	−0.336	0.193	0.142	−0.895	0.508
2232	0.184	−0.625	0.328	0.310	0.153	0.235	0.187	−0.608	0.513
2233	0.224	−0.373	0.185	0.275	−0.073	0.478	0.050	−1.485	0.481
2234	0.226	−0.356	0.508	0.193	−0.600	0.309	0.295	0.082	0.508
2235	0.263	−0.120	0.481	0.237	−0.317	0.149	0.295	0.082	0.501
2236	0.505	1.430	0.262	0.482	1.265	0.193	0.476	1.245	0.097
2237	0.355	0.470	0.224	0.310	0.153	0.235	0.261	−0.137	0.467
2238	0.295	0.082	0.508	0.152	−0.863	0.478	0.142	−0.895	0.185
2239	0.142	−0.895	0.492	0.196	−0.581	0.273	0.261	−0.137	0.492
2240	0.145	−0.878	0.481	0.237	−0.317	0.149	0.184	−0.625	0.450
2241	0.466	1.178	0.481	0.474	1.208	0.444	0.505	1.430	0.185
2242	0.261	−0.137	0.409	0.275	−0.073	0.102	0.097	−1.181	0.450
2243	0.261	−0.137	0.467	0.149	−0.882	0.422	0.142	−0.895	0.328
2244	0.487	1.312	0.467	0.444	1.020	0.235	0.489	1.329	0.262
2245	0.297	0.099	0.224	0.102	−1.184	0.273	0.261	−0.137	0.467
2246	0.461	1.144	0.431	0.430	0.926	0.501	0.503	1.413	0.328
2247	0.295	0.082	0.262	0.234	−0.336	0.149	0.142	−0.895	0.508
2248	0.332	0.318	0.450	0.272	−0.091	0.102	0.097	−1.181	0.467
2249	0.447	1.060	0.467	0.494	1.340	0.102	0.500	1.397	0.481
2250	0.184	−0.625	0.224	0.149	−0.882	0.273	0.224	−0.373	0.513
2251	0.184	−0.625	0.467	0.102	−1.184	0.149	0.261	−0.137	0.097
2252	0.184	−0.625	0.097	0.102	−1.184	0.309	0.261	−0.137	0.142
2253	0.300	0.116	0.185	0.275	−0.073	0.398	0.184	−0.625	0.481

2254	0.326	0.285	0.501	0.152	-0.863	0.444	0.050	-1.485	0.481
2255	0.187	-0.608	0.358	0.307	0.135	0.235	0.097	-1.181	0.097
2256	0.508	1.447	0.467	0.395	0.700	0.444	0.508	1.447	0.501
2257	0.476	1.245	0.409	0.444	1.020	0.491	0.482	1.279	0.142
2258	0.261	-0.137	0.501	0.000	?	0.478	0.263	-0.120	0.328
2259	0.224	-0.373	0.467	0.237	-0.317	0.478	0.184	-0.625	0.512
2260	0.505	1.430	0.328	0.444	1.020	0.512	0.500	1.397	0.508
2261	0.489	1.329	0.358	0.491	1.321	0.508	0.474	1.228	0.224
2262	0.482	1.279	0.431	0.482	1.265	0.501	0.503	1.413	0.512
2263	0.482	1.279	0.431	0.482	1.265	0.309	0.505	1.430	0.431
2264	0.411	0.824	0.262	0.515	1.472	0.478	0.484	1.295	0.409
2265	0.466	1.178	0.097	0.506	1.415	0.309	0.500	1.397	0.512
2266	0.463	1.161	0.492	0.500	1.378	0.491	0.497	1.380	0.185
2267	0.224	-0.373	0.097	0.307	0.135	0.501	0.097	-1.181	0.224
2268	0.429	0.942	0.050	0.477	1.227	0.309	0.466	1.178	0.481
2269	0.261	-0.137	0.492	0.313	0.172	0.501	0.184	-0.625	0.481
2270	0.450	1.076	0.481	0.494	1.340	0.235	0.487	1.312	0.296
2271	0.463	1.161	0.224	0.500	1.378	0.273	0.450	1.076	0.296
2272	0.224	-0.373	0.467	0.307	0.135	0.508	0.184	-0.625	0.296
2273	0.463	1.161	0.481	0.500	1.378	0.235	0.450	1.076	0.467
2274	0.097	-1.181	0.224	0.237	-0.317	0.422	0.326	0.285	0.501
2275	0.184	-0.625	0.409	0.278	-0.054	0.508	0.263	-0.120	0.262
2276	0.497	1.380	0.185	0.506	1.415	0.444	0.463	1.161	0.224
2277	0.361	0.504	0.262	0.237	-0.317	0.508	0.329	0.301	0.262
2278	0.468	1.194	0.492	0.424	0.888	0.149	0.503	1.413	0.512
2279	0.411	0.824	0.481	0.506	1.415	0.309	0.461	1.144	0.512
2280	0.500	1.397	0.142	0.442	1.001	0.491	0.482	1.279	0.492
2281	0.468	1.194	0.142	0.506	1.415	0.491	0.503	1.413	0.142
2282	0.332	0.318	0.142	0.149	-0.882	0.309	0.097	-1.181	0.142
2283	0.361	0.504	0.501	0.310	0.153	0.341	0.142	-0.895	0.512
2284	0.426	0.925	0.508	0.494	1.340	0.478	0.479	1.262	0.467
2285	0.426	0.925	0.142	0.494	1.340	0.309	0.479	1.262	0.185
2286	0.263	-0.120	0.097	0.307	0.135	0.309	0.000	?	0.328
2287	0.097	-1.181	0.142	0.342	0.361	0.398	0.097	-1.181	0.262
2288	0.489	1.329	0.492	0.480	1.246	0.512	0.500	1.397	0.262
2289	0.097	-1.181	0.481	0.310	0.153	0.235	0.142	-0.895	0.481
2290	0.050	-1.485	0.513	0.310	0.153	0.514	0.329	0.301	0.224
2291	0.487	1.312	0.385	0.404	0.756	0.491	0.511	1.464	0.262
2292	0.482	1.279	0.385	0.515	1.472	0.235	0.508	1.447	0.492
2293	0.226	-0.356	0.467	0.234	-0.336	0.341	0.226	-0.356	0.492
2294	0.468	1.194	0.481	0.509	1.434	0.193	0.508	1.447	0.492
2295	0.476	1.245	0.262	0.491	1.321	0.235	0.432	0.958	0.508
2296	0.224	-0.373	0.142	0.237	-0.317	0.508	0.408	0.807	0.492
2297	0.408	0.807	0.097	0.342	0.361	0.508	0.513	1.481	0.431
2298	0.184	-0.625	0.224	0.193	-0.600	0.149	0.184	-0.625	0.501
2299	0.263	-0.120	0.492	0.234	-0.336	0.149	0.226	-0.356	0.481
2300	0.492	1.346	0.185	0.503	1.396	0.193	0.476	1.245	0.097
2301	0.476	1.245	0.142	0.503	1.396	0.422	0.484	1.295	0.097
2302	0.142	-0.895	0.492	0.149	-0.882	0.422	0.297	0.099	0.050
2303	0.142	-0.895	0.431	0.149	-0.882	0.309	0.297	0.099	0.431
2304	0.142	-0.895	0.501	0.196	-0.581	0.273	0.297	0.099	0.185
2305	0.497	1.380	0.467	0.424	0.888	0.273	0.466	1.178	0.450
2306	0.505	1.430	0.450	0.424	0.888	0.478	0.500	1.397	0.450
2307	0.142	-0.895	0.450	0.310	0.153	0.512	0.263	-0.120	0.508
2308	0.097	-1.181	0.492	0.275	-0.073	0.478	0.224	-0.373	0.508
2309	0.142	-0.895	0.431	0.275	-0.073	0.371	0.261	-0.137	0.467
2310	0.497	1.380	0.142	0.477	1.227	0.371	0.511	1.464	0.501
2311	0.482	1.279	0.142	0.515	1.472	0.491	0.505	1.430	0.508
2312	0.513	1.481	0.224	0.485	1.283	0.508	0.484	1.295	0.296
2313	0.389	0.689	0.501	0.371	0.549	0.235	0.142	-0.895	0.467
2314	0.389	0.689	0.224	0.371	0.549	0.193	0.142	-0.895	0.050

2315	0.463	1.161	0.467	0.494	1.340	0.193	0.503	1.413	0.492
2316	0.479	1.262	0.467	0.506	1.415	0.341	0.466	1.178	0.185
2317	0.261	-0.137	0.467	0.237	-0.317	0.422	0.184	-0.625	0.467
2318	0.142	-0.895	0.450	0.193	-0.600	0.235	0.332	0.318	0.224
2319	0.097	-1.181	0.481	0.193	-0.600	0.235	0.266	-0.103	0.224
2320	0.224	-0.373	0.467	0.345	0.379	0.512	0.261	-0.137	0.501
2321	0.489	1.329	0.508	0.424	0.888	0.508	0.479	1.262	0.501
2322	0.187	-0.608	0.142	0.237	-0.317	0.491	0.224	-0.373	0.481
2323	0.142	-0.895	0.492	0.234	-0.336	0.444	0.261	-0.137	0.142
2324	0.492	1.346	0.185	0.509	1.434	0.462	0.487	1.312	0.224
2325	0.432	0.958	0.501	0.503	1.396	0.491	0.489	1.329	0.296
2326	0.505	1.430	0.142	0.488	1.302	0.444	0.487	1.312	0.224
2327	0.466	1.178	0.508	0.442	1.001	0.444	0.503	1.413	0.262
2328	0.450	1.076	0.296	0.462	1.133	0.309	0.487	1.312	0.508
2329	0.447	1.060	0.328	0.491	1.321	0.309	0.426	0.925	0.508
2330	0.492	1.346	0.467	0.444	1.020	0.309	0.495	1.363	0.224
2331	0.432	0.958	0.492	0.442	1.001	0.462	0.474	1.228	0.450
2332	0.142	-0.895	0.501	0.313	0.172	0.193	0.097	-1.181	0.450
2333	0.142	-0.895	0.296	0.313	0.172	0.462	0.097	-1.181	0.224
2334	0.224	-0.373	0.296	0.313	0.172	0.462	0.050	-1.485	0.492
2335	0.503	1.413	0.097	0.456	1.095	0.491	0.426	0.925	0.050
2336	0.226	-0.356	0.328	0.193	-0.600	0.508	0.184	-0.625	0.097
2337	0.466	1.178	0.328	0.459	1.114	0.512	0.447	1.060	0.450
2338	0.466	1.178	0.492	0.459	1.114	0.512	0.447	1.060	0.142
2339	0.466	1.178	0.492	0.488	1.302	0.508	0.500	1.397	0.508
2340	0.450	1.076	0.224	0.506	1.415	0.235	0.500	1.397	0.481
2341	0.482	1.279	0.508	0.509	1.434	0.478	0.461	1.144	0.224
2342	0.471	1.211	0.492	0.512	1.453	0.309	0.492	1.346	0.508
2343	0.508	1.447	0.262	0.506	1.415	0.462	0.500	1.397	0.450
2344	0.142	-0.895	0.501	0.234	-0.336	0.341	0.300	0.116	0.481
2345	0.489	1.329	0.262	0.480	1.246	0.514	0.461	1.144	0.142
2346	0.187	-0.608	0.224	0.310	0.153	0.053	0.050	-1.485	0.492
2347	0.503	1.413	0.508	0.459	1.114	0.102	0.484	1.295	0.508
2348	0.142	-0.895	0.185	0.342	0.361	0.501	0.184	-0.625	0.492
2349	0.508	1.447	0.508	0.518	1.491	0.501	0.461	1.144	0.328
2350	0.300	0.116	0.501	0.053	-1.504	0.512	0.224	-0.373	0.097
2351	0.332	0.318	0.097	0.102	-1.184	0.193	0.224	-0.373	0.513
2352	0.468	1.194	0.467	0.503	1.396	0.235	0.492	1.346	0.501
2353	0.495	1.363	0.492	0.503	1.396	0.149	0.492	1.346	0.501
2354	0.503	1.413	0.508	0.515	1.472	0.273	0.476	1.245	0.262
2355	0.297	0.099	0.097	0.193	-0.600	0.273	0.142	-0.895	0.358
2356	0.300	0.116	0.450	0.234	-0.336	0.491	0.224	-0.373	0.296
2357	0.097	-1.181	0.467	0.149	-0.882	0.491	0.297	0.099	0.481
2358	0.332	0.318	0.501	0.275	-0.073	0.341	0.224	-0.373	0.501
2359	0.332	0.318	0.185	0.272	-0.091	0.501	0.261	-0.137	0.185
2360	0.489	1.329	0.224	0.491	1.321	0.501	0.508	1.447	0.050
2361	0.489	1.329	0.481	0.491	1.321	0.273	0.508	1.447	0.142
2362	0.226	-0.356	0.481	0.345	0.379	0.501	0.224	-0.373	0.224
2363	0.508	1.447	0.492	0.494	1.340	0.235	0.447	1.060	0.508
2364	0.492	1.346	0.224	0.494	1.340	0.235	0.447	1.060	0.481
2365	0.263	-0.120	0.142	0.275	-0.073	0.508	0.226	-0.356	0.142
2366	0.497	1.380	0.328	0.503	1.396	0.309	0.489	1.329	0.481
2367	0.261	-0.137	0.296	0.234	-0.336	0.444	0.050	-1.485	0.142
2368	0.224	-0.373	0.508	0.234	-0.336	0.462	0.097	-1.181	0.492
2369	0.503	1.413	0.501	0.509	1.434	0.235	0.445	1.043	0.481
2370	0.184	-0.625	0.296	0.313	0.172	0.444	0.142	-0.895	0.142
2371	0.503	1.413	0.328	0.444	1.020	0.478	0.503	1.413	0.501
2372	0.497	1.380	0.262	0.465	1.152	0.422	0.476	1.245	0.492
2373	0.097	-1.181	0.224	0.237	-0.317	0.309	0.226	-0.356	0.097
2374	0.468	1.194	0.481	0.447	1.039	0.491	0.500	1.397	0.492
2375	0.489	1.329	0.508	0.480	1.246	0.462	0.445	1.043	0.431

2376	0.508	1.447	0.185	0.424	0.888	0.462	0.476	1.245	0.097
2377	0.097	-1.181	0.467	0.310	0.153	0.193	0.142	-0.895	0.358
2378	0.450	1.076	0.296	0.494	1.340	0.273	0.492	1.346	0.358
2379	0.468	1.194	0.431	0.462	1.133	0.462	0.503	1.413	0.512
2380	0.500	1.397	0.512	0.462	1.133	0.462	0.489	1.329	0.512
2381	0.184	-0.625	0.296	0.193	-0.600	0.462	0.326	0.285	0.050
2382	0.226	-0.356	0.467	0.272	-0.091	0.273	0.097	-1.181	0.512
2383	0.479	1.262	0.481	0.462	1.133	0.193	0.508	1.447	0.185
2384	0.479	1.262	0.296	0.465	1.152	0.371	0.500	1.397	0.467
2385	0.489	1.329	0.481	0.465	1.152	0.149	0.500	1.397	0.385
2386	0.224	-0.373	0.492	0.275	-0.073	0.501	0.263	-0.120	0.467
2387	0.142	-0.895	0.185	0.196	-0.581	0.508	0.361	0.504	0.513
2388	0.332	0.318	0.358	0.371	0.549	0.053	0.000	?	0.185
2389	0.297	0.099	0.501	0.149	-0.882	0.193	0.297	0.099	0.050
2390	0.513	1.481	0.358	0.506	1.415	0.235	0.476	1.245	0.185
2391	0.495	1.363	0.358	0.512	1.453	0.102	0.495	1.363	0.142
2392	0.295	0.082	0.296	0.053	-1.504	0.462	0.184	-0.625	0.050
2393	0.329	0.301	0.450	0.193	-0.600	0.512	0.050	-1.485	0.501
2394	0.263	-0.120	0.262	0.234	-0.336	0.235	0.142	-0.895	0.185
2395	0.226	-0.356	0.512	0.102	-1.184	0.501	0.226	-0.356	0.097
2396	0.484	1.295	0.512	0.462	1.133	0.149	0.497	1.380	0.142
2397	0.513	1.481	0.508	0.512	1.453	0.478	0.474	1.228	0.050
2398	0.187	-0.608	0.508	0.234	-0.336	0.422	0.142	-0.895	0.467
2399	0.466	1.178	0.262	0.500	1.378	0.193	0.474	1.228	0.224
2400	0.297	0.099	0.224	0.149	-0.882	0.478	0.142	-0.895	0.409
2401	0.432	0.958	0.296	0.477	1.227	0.478	0.484	1.295	0.262
2402	0.513	1.481	0.185	0.418	0.850	0.341	0.474	1.228	0.097
2403	0.300	0.116	0.262	0.193	-0.600	0.462	0.142	-0.895	0.097
2404	0.466	1.178	0.185	0.480	1.246	0.491	0.497	1.380	0.481
2405	0.484	1.295	0.385	0.474	1.208	0.273	0.487	1.312	0.385
2406	0.295	0.082	0.262	0.342	0.361	0.341	0.097	-1.181	0.513
2407	0.479	1.262	0.328	0.468	1.170	0.444	0.495	1.363	0.513
2408	0.492	1.346	0.262	0.497	1.359	0.501	0.429	0.942	0.185
2409	0.184	-0.625	0.262	0.275	-0.073	0.501	0.097	-1.181	0.431
2410	0.358	0.487	0.296	0.345	0.379	0.193	0.355	0.470	0.481
2411	0.508	1.447	0.501	0.442	1.001	0.444	0.358	0.487	0.185
2412	0.361	0.504	0.224	0.500	1.378	0.235	0.513	1.481	0.185
2413	0.361	0.504	0.481	0.500	1.378	0.512	0.513	1.481	0.481
2414	0.297	0.099	0.328	0.193	-0.600	0.478	0.050	-1.485	0.492
2415	0.455	1.110	0.185	0.444	1.020	0.508	0.508	1.447	0.224
2416	0.261	-0.137	0.328	0.237	-0.317	0.444	0.184	-0.625	0.513
2417	0.511	1.464	0.328	0.509	1.434	0.371	0.461	1.144	0.050
2418	0.511	1.464	0.492	0.474	1.208	0.273	0.382	0.638	0.467
2419	0.508	1.447	0.513	0.506	1.415	0.102	0.466	1.178	0.050
2420	0.508	1.447	0.512	0.439	0.982	0.341	0.511	1.464	0.097
2421	0.261	-0.137	0.512	0.368	0.530	0.193	0.184	-0.625	0.142
2422	0.224	-0.373	0.050	0.272	-0.091	0.309	0.050	-1.485	0.492
2423	0.297	0.099	0.501	0.102	-1.184	0.462	0.184	-0.625	0.185
2424	0.184	-0.625	0.492	0.342	0.361	0.102	0.000	?	0.224
2425	0.261	-0.137	0.328	0.193	-0.600	0.273	0.142	-0.895	0.508
2426	0.187	-0.608	0.358	0.310	0.153	0.273	0.050	-1.485	0.142
2427	0.387	0.672	0.508	0.459	1.114	0.149	0.500	1.397	0.508
2428	0.263	-0.120	0.508	0.102	-1.184	0.235	0.184	-0.625	0.512
2429	0.326	0.285	0.185	0.275	-0.073	0.478	0.097	-1.181	0.492
2430	0.261	-0.137	0.467	0.275	-0.073	0.149	0.000	?	0.501
2431	0.266	-0.103	0.224	0.149	-0.882	0.478	0.142	-0.895	0.481
2432	0.297	0.099	0.385	0.234	-0.336	0.102	0.050	-1.485	0.508
2433	0.505	1.430	0.224	0.474	1.208	0.371	0.463	1.161	0.142
2434	0.224	-0.373	0.328	0.149	-0.882	0.102	0.224	-0.373	0.185
2435	0.482	1.279	0.262	0.471	1.189	0.102	0.408	0.807	0.185
2436	0.334	0.335	0.492	0.102	-1.184	0.444	0.261	-0.137	0.050

2437	0.184	-0.625	0.224	0.371	0.549	0.462	0.000	?	0.097
2438	0.329	0.301	0.358	0.102	-1.184	0.444	0.097	-1.181	0.224
2439	0.329	0.301	0.467	0.102	-1.184	0.444	0.097	-1.181	0.409
2440	0.492	1.346	0.328	0.442	1.001	0.309	0.482	1.279	0.097
2441	0.511	1.464	0.492	0.462	1.133	0.462	0.382	0.638	0.450
2442	0.511	1.464	0.467	0.442	1.001	0.491	0.505	1.430	0.142
2443	0.511	1.464	0.492	0.442	1.001	0.235	0.505	1.430	0.142
2444	0.050	-1.485	0.467	0.313	0.172	0.102	0.184	-0.625	0.142
2445	0.500	1.397	0.508	0.465	1.152	0.491	0.426	0.925	0.185
2446	0.489	1.329	0.467	0.491	1.321	0.491	0.479	1.262	0.097
2447	0.329	0.301	0.185	0.234	-0.336	0.193	0.184	-0.625	0.492
2448	0.358	0.487	0.262	0.102	-1.184	0.422	0.184	-0.625	0.224
2449	0.505	1.430	0.262	0.494	1.340	0.273	0.474	1.228	0.097
2450	0.505	1.430	0.296	0.494	1.340	0.491	0.484	1.295	0.097
2451	0.184	-0.625	0.262	0.193	-0.600	0.273	0.226	-0.356	0.097
2452	0.466	1.178	0.185	0.421	0.869	0.102	0.505	1.430	0.097
2453	0.224	-0.373	0.508	0.275	-0.073	0.193	0.050	-1.485	0.450
2454	0.384	0.655	0.328	0.485	1.283	0.508	0.463	1.161	0.142
2455	0.224	-0.373	0.501	0.275	-0.073	0.235	0.050	-1.485	0.481
2456	0.326	0.285	0.328	0.102	-1.184	0.102	0.097	-1.181	0.492
2457	0.263	-0.120	0.328	0.196	-0.581	0.478	0.142	-0.895	0.142
2458	0.484	1.295	0.328	0.512	1.453	0.235	0.497	1.380	0.142
2459	0.224	-0.373	0.296	0.237	-0.317	0.501	0.184	-0.625	0.142
2460	0.358	0.487	0.296	0.102	-1.184	0.422	0.224	-0.373	0.142
2461	0.461	1.144	0.296	0.477	1.227	0.491	0.505	1.430	0.262
2462	0.332	0.318	0.492	0.237	-0.317	0.512	0.142	-0.895	0.481
2463	0.495	1.363	0.385	0.503	1.396	0.462	0.503	1.413	0.501
2464	0.468	1.194	0.296	0.424	0.888	0.462	0.505	1.430	0.142
2465	0.492	1.346	0.296	0.488	1.302	0.273	0.487	1.312	0.142
2466	0.463	1.161	0.296	0.512	1.453	0.309	0.500	1.397	0.328
2467	0.505	1.430	0.328	0.462	1.133	0.235	0.479	1.262	0.450
2468	0.463	1.161	0.481	0.459	1.114	0.193	0.505	1.430	0.481
2469	0.184	-0.625	0.224	0.272	-0.091	0.235	0.142	-0.895	0.185
2470	0.261	-0.137	0.450	0.307	0.135	0.273	0.184	-0.625	0.296
2471	0.261	-0.137	0.492	0.234	-0.336	0.501	0.184	-0.625	0.224
2472	0.297	0.099	0.185	0.196	-0.581	0.422	0.050	-1.485	0.328
2473	0.261	-0.137	0.224	0.237	-0.317	0.501	0.097	-1.181	0.185
2474	0.187	-0.608	0.224	0.275	-0.073	0.273	0.224	-0.373	0.185
2475	0.505	1.430	0.224	0.497	1.359	0.273	0.405	0.790	0.467
2476	0.332	0.318	0.262	0.421	0.869	0.273	0.097	-1.181	0.501
2477	0.503	1.413	0.467	0.500	1.378	0.309	0.445	1.043	0.224
2478	0.329	0.301	0.409	0.275	-0.073	0.193	0.142	-0.895	0.508
2479	0.329	0.301	0.262	0.275	-0.073	0.273	0.142	-0.895	0.296
2480	0.329	0.301	0.328	0.275	-0.073	0.478	0.142	-0.895	0.224
2481	0.300	0.116	0.224	0.310	0.153	0.053	0.184	-0.625	0.296
2482	0.300	0.116	0.512	0.193	-0.600	0.235	0.097	-1.181	0.512
2483	0.297	0.099	0.492	0.272	-0.091	0.235	0.000	?	0.501
2484	0.492	1.346	0.185	0.474	1.208	0.235	0.487	1.312	0.492
2485	0.384	0.655	0.097	0.053	-1.504	0.102	0.226	-0.356	0.097
2486	0.297	0.099	0.224	0.234	-0.336	0.444	0.097	-1.181	0.262
2487	0.297	0.099	0.097	0.234	-0.336	0.341	0.097	-1.181	0.481
2488	0.297	0.099	0.328	0.234	-0.336	0.462	0.097	-1.181	0.467
2489	0.326	0.285	0.328	0.102	-1.184	0.422	0.097	-1.181	0.492
2490	0.482	1.279	0.492	0.444	1.020	0.235	0.445	1.043	0.142
2491	0.224	-0.373	0.450	0.342	0.361	0.235	0.142	-0.895	0.142
2492	0.450	1.076	0.296	0.459	1.114	0.235	0.476	1.245	0.097
2493	0.492	1.346	0.431	0.421	0.869	0.235	0.489	1.329	0.097
2494	0.184	-0.625	0.097	0.234	-0.336	0.273	0.142	-0.895	0.481
2495	0.226	-0.356	0.224	0.234	-0.336	0.491	0.142	-0.895	0.512
2496	0.226	-0.356	0.385	0.234	-0.336	0.501	0.142	-0.895	0.492
2497	0.226	-0.356	0.431	0.234	-0.336	0.309	0.142	-0.895	0.492

2498	0.263	−0.120	0.481	0.275	−0.073	0.193	0.263	−0.120	0.492
2499	0.471	1.211	0.467	0.488	1.302	0.371	0.476	1.245	0.512
2500	0.411	0.824	0.328	0.500	1.378	0.491	0.497	1.380	0.513
2501	0.263	−0.120	0.185	0.307	0.135	0.508	0.145	−0.878	0.262
2502	0.332	0.318	0.501	0.193	−0.600	0.235	0.142	−0.895	0.492
2503	0.224	−0.373	0.481	0.371	0.549	0.235	0.326	0.285	0.501
2504	0.511	1.464	0.431	0.488	1.302	0.149	0.447	1.060	0.296
2505	0.489	1.329	0.224	0.503	1.396	0.102	0.479	1.262	0.224
2506	0.187	−0.608	0.296	0.234	−0.336	0.149	0.187	−0.608	0.185
2507	0.097	−1.181	0.296	0.234	−0.336	0.149	0.295	0.082	0.512
2508	0.226	−0.356	0.296	0.149	−0.882	0.491	0.226	−0.356	0.512
2509	0.097	−1.181	0.296	0.102	−1.184	0.491	0.326	0.285	0.450
2510	0.329	0.301	0.450	0.149	−0.882	0.053	0.184	−0.625	0.450
2511	0.329	0.301	0.508	0.149	−0.882	0.501	0.184	−0.625	0.481
2512	0.489	1.329	0.508	0.491	1.321	0.193	0.463	1.161	0.508
2513	0.447	1.060	0.508	0.494	1.340	0.235	0.497	1.380	0.512
2514	0.297	0.099	0.512	0.053	−1.504	0.102	0.224	−0.373	0.467
2515	0.429	0.942	0.501	0.503	1.396	0.491	0.497	1.380	0.409
2516	0.097	−1.181	0.501	0.193	−0.600	0.462	0.297	0.099	0.262
2517	0.226	−0.356	0.328	0.234	−0.336	0.501	0.224	−0.373	0.262
2518	0.387	0.672	0.492	0.102	−1.184	0.273	0.295	0.082	0.512
2519	0.432	0.958	0.492	0.485	1.283	0.149	0.505	1.430	0.409
2520	0.476	1.245	0.142	0.459	1.114	0.491	0.503	1.413	0.501
2521	0.466	1.178	0.185	0.500	1.378	0.478	0.487	1.312	0.328
2522	0.329	0.301	0.358	0.278	−0.054	0.491	0.097	−1.181	0.328
2523	0.187	−0.608	0.508	0.149	−0.882	0.309	0.261	−0.137	0.481
2524	0.503	1.413	0.431	0.485	1.283	0.102	0.479	1.262	0.513
2525	0.484	1.295	0.512	0.480	1.246	0.102	0.468	1.194	0.513
2526	0.429	0.942	0.501	0.491	1.321	0.235	0.492	1.346	0.492
2527	0.224	−0.373	0.501	0.310	0.153	0.149	0.000	?	0.501
2528	0.295	0.082	0.450	0.102	−1.184	0.512	0.142	−0.895	0.501
2529	0.295	0.082	0.450	0.102	−1.184	0.422	0.142	−0.895	0.501
2530	0.295	0.082	0.467	0.234	−0.336	0.501	0.097	−1.181	0.328
2531	0.297	0.099	0.513	0.149	−0.882	0.501	0.097	−1.181	0.185
2532	0.445	1.043	0.262	0.515	1.472	0.478	0.479	1.262	0.492
2533	0.511	1.464	0.142	0.421	0.869	0.444	0.500	1.397	0.328
2534	0.511	1.464	0.450	0.500	1.378	0.422	0.484	1.295	0.142
2535	0.511	1.464	0.097	0.500	1.378	0.193	0.484	1.295	0.467
2536	0.508	1.447	0.409	0.477	1.227	0.462	0.487	1.312	0.492
2537	0.503	1.413	0.409	0.439	0.982	0.444	0.516	1.498	0.224
2538	0.503	1.413	0.142	0.418	0.850	0.193	0.516	1.498	0.409
2539	0.326	0.285	0.481	0.196	−0.581	0.235	0.263	−0.120	0.358
2540	0.487	1.312	0.492	0.462	1.133	0.235	0.495	1.363	0.097
2541	0.487	1.312	0.492	0.442	1.001	0.398	0.503	1.413	0.142
2542	0.142	−0.895	0.508	0.193	−0.600	0.478	0.295	0.082	0.512
2543	0.184	−0.625	0.467	0.234	−0.336	0.491	0.224	−0.373	0.262
2544	0.358	0.487	0.467	0.240	−0.299	0.398	0.184	−0.625	0.450
2545	0.505	1.430	0.467	0.398	0.718	0.514	0.505	1.430	0.450
2546	0.429	0.942	0.185	0.480	1.246	0.462	0.508	1.447	0.431
2547	0.508	1.447	0.224	0.488	1.302	0.462	0.447	1.060	0.450
2548	0.508	1.447	0.513	0.398	0.718	0.512	0.447	1.060	0.512
2549	0.503	1.413	0.296	0.515	1.472	0.444	0.482	1.279	0.512
2550	0.450	1.076	0.224	0.459	1.114	0.149	0.503	1.413	0.328
2551	0.450	1.076	0.501	0.459	1.114	0.193	0.505	1.430	0.296
2552	0.461	1.144	0.508	0.506	1.415	0.512	0.468	1.194	0.142
2553	0.505	1.430	0.296	0.442	1.001	0.235	0.411	0.824	0.450
2554	0.263	−0.120	0.262	0.149	−0.882	0.508	0.263	−0.120	0.450
2555	0.142	−0.895	0.508	0.193	−0.600	0.053	0.261	−0.137	0.481
2556	0.445	1.043	0.262	0.509	1.434	0.193	0.513	1.481	0.142
2557	0.097	−1.181	0.142	0.234	−0.336	0.501	0.411	0.824	0.262
2558	0.408	0.807	0.508	0.503	1.396	0.444	0.497	1.380	0.508

2559	0.408	0.807	0.358	0.053	-1.504	0.444	0.326	0.285	0.467
2560	0.145	-0.878	0.512	0.193	-0.600	0.422	0.326	0.285	0.508
2561	0.479	1.262	0.512	0.500	1.378	0.501	0.479	1.262	0.262
2562	0.492	1.346	0.492	0.444	1.020	0.501	0.508	1.447	0.185
2563	0.492	1.346	0.508	0.444	1.020	0.501	0.508	1.447	0.185
2564	0.505	1.430	0.481	0.421	0.869	0.235	0.489	1.329	0.450
2565	0.466	1.178	0.501	0.500	1.378	0.235	0.495	1.363	0.501
2566	0.466	1.178	0.262	0.500	1.378	0.444	0.495	1.363	0.431
2567	0.466	1.178	0.142	0.500	1.378	0.235	0.495	1.363	0.512
2568	0.184	-0.625	0.224	0.234	-0.336	0.193	0.332	0.318	0.224
2569	0.226	-0.356	0.467	0.234	-0.336	0.512	0.187	-0.608	0.142
2570	0.511	1.464	0.508	0.447	1.039	0.398	0.489	1.329	0.185
2571	0.297	0.099	0.508	0.234	-0.336	0.149	0.326	0.285	0.296
2572	0.224	-0.373	0.224	0.193	-0.600	0.273	0.142	-0.895	0.328
2573	0.500	1.397	0.185	0.518	1.491	0.478	0.466	1.178	0.296
2574	0.503	1.413	0.450	0.398	0.718	0.273	0.495	1.363	0.508
2575	0.295	0.082	0.512	0.149	-0.882	0.273	0.224	-0.373	0.481
2576	0.263	-0.120	0.492	0.272	-0.091	0.444	0.411	0.824	0.262
2577	0.500	1.397	0.262	0.477	1.227	0.235	0.355	0.470	0.328
2578	0.261	-0.137	0.224	0.272	-0.091	0.462	0.097	-1.181	0.501
2579	0.142	-0.895	0.224	0.275	-0.073	0.462	0.142	-0.895	0.328
2580	0.508	1.447	0.492	0.442	1.001	0.462	0.513	1.481	0.450
2581	0.361	0.504	0.492	0.234	-0.336	0.478	0.263	-0.120	0.409
2582	0.508	1.447	0.467	0.462	1.133	0.501	0.445	1.043	0.328
2583	0.508	1.447	0.409	0.462	1.133	0.478	0.445	1.043	0.385
2584	0.495	1.363	0.224	0.462	1.133	0.371	0.432	0.958	0.385
2585	0.511	1.464	0.224	0.477	1.227	0.273	0.445	1.043	0.142
2586	0.479	1.262	0.185	0.503	1.396	0.193	0.508	1.447	0.481
2587	0.497	1.380	0.224	0.474	1.208	0.491	0.511	1.464	0.467
2588	0.261	-0.137	0.185	0.368	0.530	0.501	0.326	0.285	0.050
2589	0.142	-0.895	0.409	0.275	-0.073	0.478	0.300	0.116	0.467
2590	0.224	-0.373	0.481	0.196	-0.581	0.193	0.142	-0.895	0.262
2591	0.468	1.194	0.450	0.482	1.265	0.309	0.447	1.060	0.328
2592	0.505	1.430	0.142	0.494	1.340	0.462	0.450	1.076	0.262
2593	0.505	1.430	0.224	0.474	1.208	0.444	0.479	1.262	0.097
2594	0.224	-0.373	0.431	0.196	-0.581	0.462	0.142	-0.895	0.501
2595	0.184	-0.625	0.224	0.313	0.172	0.102	0.263	-0.120	0.501
2596	0.447	1.060	0.501	0.459	1.114	0.235	0.503	1.413	0.185
2597	0.516	1.498	0.409	0.442	1.001	0.235	0.474	1.228	0.467
2598	0.489	1.329	0.185	0.462	1.133	0.508	0.505	1.430	0.501
2599	0.266	-0.103	0.358	0.102	-1.184	0.512	0.261	-0.137	0.512
2600	0.224	-0.373	0.358	0.237	-0.317	0.478	0.184	-0.625	0.508
2601	0.224	-0.373	0.262	0.237	-0.317	0.491	0.184	-0.625	0.481
2602	0.487	1.312	0.492	0.506	1.415	0.193	0.450	1.076	0.512
2603	0.487	1.312	0.492	0.509	1.434	0.309	0.500	1.397	0.508
2604	0.471	1.211	0.224	0.480	1.246	0.193	0.434	0.975	0.097
2605	0.413	0.841	0.501	0.488	1.302	0.273	0.511	1.464	0.501
2606	0.226	-0.356	0.262	0.193	-0.600	0.149	0.226	-0.356	0.481
2607	0.229	-0.339	0.262	0.307	0.135	0.309	0.142	-0.895	0.450
2608	0.184	-0.625	0.262	0.196	-0.581	0.491	0.187	-0.608	0.050
2609	0.226	-0.356	0.224	0.272	-0.091	0.491	0.297	0.099	0.296
2610	0.184	-0.625	0.508	0.149	-0.882	0.193	0.332	0.318	0.185
2611	0.411	0.824	0.492	0.313	0.172	0.235	0.295	0.082	0.512
2612	0.479	1.262	0.185	0.491	1.321	0.512	0.505	1.430	0.513
2613	0.447	1.060	0.501	0.491	1.321	0.193	0.479	1.262	0.513
2614	0.142	-0.895	0.501	0.196	-0.581	0.422	0.263	-0.120	0.481
2615	0.226	-0.356	0.467	0.234	-0.336	0.491	0.332	0.318	0.481
2616	0.432	0.958	0.492	0.506	1.415	0.444	0.500	1.397	0.467
2617	0.224	-0.373	0.467	0.193	-0.600	0.341	0.329	0.301	0.185
2618	0.500	1.397	0.508	0.421	0.869	0.341	0.447	1.060	0.185
2619	0.411	0.824	0.512	0.491	1.321	0.193	0.405	0.790	0.185

2620	0.184	−0.625	0.328	0.444	1.020	0.462	0.326	0.285	0.450
2621	0.361	0.504	0.492	0.342	0.361	0.462	0.384	0.655	0.142
2622	0.361	0.504	0.481	0.342	0.361	0.309	0.384	0.655	0.142
2623	0.261	−0.137	0.501	0.193	−0.600	0.398	0.145	−0.878	0.508
2624	0.492	1.346	0.262	0.465	1.152	0.102	0.479	1.262	0.142
2625	0.495	1.363	0.050	0.462	1.133	0.341	0.466	1.178	0.142
2626	0.224	−0.373	0.262	0.313	0.172	0.273	0.050	−1.485	0.142
2627	0.503	1.413	0.501	0.401	0.737	0.341	0.463	1.161	0.501
2628	0.261	−0.137	0.508	0.102	−1.184	0.512	0.261	−0.137	0.358
2629	0.261	−0.137	0.501	0.339	0.342	0.512	0.332	0.318	0.409
2630	0.261	−0.137	0.467	0.278	−0.054	0.193	0.261	−0.137	0.467
2631	0.224	−0.373	0.409	0.345	0.379	0.512	0.097	−1.181	0.224
2632	0.511	1.464	0.508	0.503	1.396	0.501	0.500	1.397	0.142
2633	0.495	1.363	0.262	0.503	1.396	0.501	0.500	1.397	0.508
2634	0.187	−0.608	0.050	0.196	−0.581	0.462	0.184	−0.625	0.492
2635	0.503	1.413	0.358	0.515	1.472	0.478	0.463	1.161	0.492
2636	0.503	1.413	0.450	0.500	1.378	0.462	0.497	1.380	0.328
2637	0.466	1.178	0.142	0.500	1.378	0.444	0.508	1.447	0.142
2638	0.492	1.346	0.224	0.459	1.114	0.193	0.500	1.397	0.481
2639	0.463	1.161	0.431	0.480	1.246	0.491	0.476	1.245	0.385
2640	0.503	1.413	0.097	0.465	1.152	0.491	0.508	1.447	0.328
2641	0.508	1.447	0.097	0.444	1.020	0.508	0.505	1.430	0.508
2642	0.332	0.318	0.097	0.193	−0.600	0.235	0.097	−1.181	0.508
2643	0.489	1.329	0.508	0.494	1.340	0.341	0.503	1.413	0.224
2644	0.479	1.262	0.385	0.488	1.302	0.102	0.476	1.245	0.224
2645	0.497	1.380	0.513	0.506	1.415	0.491	0.447	1.060	0.450
2646	0.261	−0.137	0.512	0.237	−0.317	0.491	0.050	−1.485	0.481
2647	0.050	−1.485	0.142	0.345	0.379	0.491	0.295	0.082	0.492
2648	0.263	−0.120	0.224	0.102	−1.184	0.512	0.184	−0.625	0.501
2649	0.492	1.346	0.185	0.491	1.321	0.512	0.511	1.464	0.185
2650	0.505	1.430	0.492	0.488	1.302	0.478	0.513	1.481	0.185
2651	0.500	1.397	0.450	0.491	1.321	0.371	0.508	1.447	0.142
2652	0.463	1.161	0.481	0.512	1.453	0.341	0.482	1.279	0.467
2653	0.405	0.790	0.050	0.512	1.453	0.235	0.482	1.279	0.142
2654	0.505	1.430	0.185	0.480	1.246	0.512	0.466	1.178	0.409
2655	0.261	−0.137	0.508	0.374	0.568	0.273	0.184	−0.625	0.185
2656	0.050	−1.485	0.508	0.339	0.342	0.235	0.187	−0.608	0.409
2657	0.358	0.487	0.431	0.240	−0.299	0.478	0.184	−0.625	0.050
2658	0.445	1.043	0.508	0.506	1.415	0.309	0.450	1.076	0.508
2659	0.142	−0.895	0.492	0.272	−0.091	0.309	0.142	−0.895	0.508
2660	0.224	−0.373	0.224	0.234	−0.336	0.341	0.142	−0.895	0.492
2661	0.429	0.942	0.224	0.477	1.227	0.462	0.508	1.447	0.492
2662	0.097	−1.181	0.501	0.307	0.135	0.235	0.142	−0.895	0.262
2663	0.097	−1.181	0.513	0.307	0.135	0.512	0.142	−0.895	0.224
2664	0.097	−1.181	0.501	0.339	0.342	0.491	0.142	−0.895	0.097
2665	0.503	1.413	0.467	0.471	1.189	0.273	0.508	1.447	0.097
2666	0.387	0.672	0.262	0.237	−0.317	0.235	0.358	0.487	0.097
2667	0.516	1.498	0.296	0.509	1.434	0.371	0.408	0.807	0.431
2668	0.511	1.464	0.328	0.488	1.302	0.501	0.463	1.161	0.508
2669	0.145	−0.878	0.492	0.275	−0.073	0.514	0.224	−0.373	0.262
2670	0.226	−0.356	0.224	0.237	−0.317	0.512	0.142	−0.895	0.450
2671	0.184	−0.625	0.050	0.374	0.568	0.235	0.000	?	0.450
2672	0.489	1.329	0.262	0.494	1.340	0.371	0.508	1.447	0.296
2673	0.445	1.043	0.512	0.520	1.509	0.501	0.495	1.363	0.185
2674	0.479	1.262	0.296	0.512	1.453	0.478	0.492	1.346	0.224
2675	0.050	−1.485	0.481	0.234	−0.336	0.371	0.326	0.285	0.467
2676	0.184	−0.625	0.481	0.374	0.568	0.478	0.142	−0.895	0.513
2677	0.503	1.413	0.467	0.503	1.396	0.508	0.484	1.295	0.450
2678	0.513	1.481	0.467	0.477	1.227	0.371	0.387	0.672	0.185
2679	0.437	0.992	0.328	0.374	0.568	0.371	0.326	0.285	0.512
2680	0.505	1.430	0.262	0.477	1.227	0.501	0.505	1.430	0.050

2681	0.492	1.346	0.262	0.506	1.415	0.501	0.505	1.430	0.492
2682	0.226	-0.356	0.224	0.371	0.549	0.508	0.224	-0.373	0.450
2683	0.226	-0.356	0.224	0.371	0.549	0.491	0.224	-0.373	0.501
2684	0.500	1.397	0.512	0.497	1.359	0.193	0.447	1.060	0.142
2685	0.511	1.464	0.492	0.497	1.359	0.235	0.479	1.262	0.142
2686	0.500	1.397	0.224	0.506	1.415	0.309	0.487	1.312	0.097
2687	0.466	1.178	0.512	0.494	1.340	0.422	0.503	1.413	0.481
2688	0.261	-0.137	0.508	0.193	-0.600	0.273	0.184	-0.625	0.513
2689	0.300	0.116	0.296	0.234	-0.336	0.193	0.184	-0.625	0.050
2690	0.329	0.301	0.385	0.310	0.153	0.273	0.142	-0.895	0.262
2691	0.495	1.363	0.296	0.424	0.888	0.491	0.468	1.194	0.508
2692	0.224	-0.373	0.450	0.275	-0.073	0.273	0.142	-0.895	0.296
2693	0.050	-1.485	0.481	0.196	-0.581	0.501	0.405	0.790	0.142
2694	0.261	-0.137	0.512	0.272	-0.091	0.501	0.184	-0.625	0.185
2695	0.505	1.430	0.385	0.491	1.321	0.462	0.408	0.807	0.501
2696	0.295	0.082	0.431	0.272	-0.091	0.462	0.050	-1.485	0.409
2697	0.479	1.262	0.224	0.500	1.378	0.273	0.505	1.430	0.224
2698	0.479	1.262	0.512	0.500	1.378	0.273	0.505	1.430	0.481
2699	0.466	1.178	0.492	0.462	1.133	0.371	0.492	1.346	0.481
2700	0.466	1.178	0.513	0.462	1.133	0.235	0.492	1.346	0.501
2701	0.332	0.318	0.358	0.275	-0.073	0.235	0.261	-0.137	0.513
2702	0.263	-0.120	0.358	0.275	-0.073	0.501	0.224	-0.373	0.185
2703	0.263	-0.120	0.358	0.371	0.549	0.508	0.097	-1.181	0.385
2704	0.224	-0.373	0.501	0.234	-0.336	0.309	0.097	-1.181	0.185
2705	0.224	-0.373	0.481	0.234	-0.336	0.371	0.097	-1.181	0.358
2706	0.508	1.447	0.185	0.497	1.359	0.398	0.429	0.942	0.501
2707	0.492	1.346	0.296	0.506	1.415	0.273	0.505	1.430	0.097
2708	0.226	-0.356	0.481	0.307	0.135	0.309	0.261	-0.137	0.262
2709	0.508	1.447	0.296	0.368	0.530	0.193	0.447	1.060	0.481
2710	0.505	1.430	0.296	0.395	0.700	0.512	0.447	1.060	0.450
2711	0.297	0.099	0.296	0.272	-0.091	0.501	0.300	0.116	0.328
2712	0.387	0.672	0.481	0.313	0.172	0.462	0.184	-0.625	0.185
2713	0.297	0.099	0.508	0.193	-0.600	0.273	0.224	-0.373	0.358
2714	0.453	1.093	0.142	0.515	1.472	0.491	0.468	1.194	0.050
2715	0.476	1.245	0.450	0.500	1.378	0.309	0.516	1.498	0.185
2716	0.511	1.464	0.508	0.468	1.170	0.478	0.450	1.076	0.224
2717	0.387	0.672	0.501	0.272	-0.091	0.478	0.184	-0.625	0.450
2718	0.429	0.942	0.385	0.494	1.340	0.444	0.505	1.430	0.467
2719	0.224	-0.373	0.224	0.310	0.153	0.273	0.050	-1.485	0.262
2720	0.508	1.447	0.467	0.480	1.246	0.273	0.484	1.295	0.296
2721	0.489	1.329	0.224	0.477	1.227	0.235	0.450	1.076	0.450
2722	0.513	1.481	0.142	0.442	1.001	0.508	0.495	1.363	0.481
2723	0.361	0.504	0.481	0.272	-0.091	0.501	0.145	-0.878	0.450
2724	0.361	0.504	0.050	0.272	-0.091	0.309	0.145	-0.878	0.513
2725	0.363	0.520	0.097	0.234	-0.336	0.149	0.097	-1.181	0.431
2726	0.500	1.397	0.450	0.509	1.434	0.501	0.482	1.279	0.501
2727	0.482	1.279	0.467	0.503	1.396	0.235	0.511	1.464	0.328
2728	0.184	-0.625	0.142	0.313	0.172	0.102	0.050	-1.485	0.501
2729	0.297	0.099	0.262	0.149	-0.882	0.149	0.261	-0.137	0.296
2730	0.482	1.279	0.262	0.503	1.396	0.462	0.505	1.430	0.328
2731	0.297	0.099	0.185	0.234	-0.336	0.508	0.295	0.082	0.224
2732	0.295	0.082	0.296	0.102	-1.184	0.273	0.142	-0.895	0.224
2733	0.295	0.082	0.142	0.152	-0.863	0.512	0.184	-0.625	0.501
2734	0.482	1.279	0.481	0.465	1.152	0.491	0.505	1.430	0.050
2735	0.505	1.430	0.512	0.506	1.415	0.501	0.408	0.807	0.481
2736	0.142	-0.895	0.185	0.272	-0.091	0.501	0.224	-0.373	0.508
2737	0.447	1.060	0.142	0.506	1.415	0.309	0.482	1.279	0.224
2738	0.508	1.447	0.492	0.488	1.302	0.491	0.482	1.279	0.481
2739	0.505	1.430	0.467	0.503	1.396	0.341	0.497	1.380	0.050
2740	0.382	0.638	0.512	0.494	1.340	0.309	0.511	1.464	0.481
2741	0.224	-0.373	0.481	0.310	0.153	0.444	0.184	-0.625	0.142

2742	0.463	1.161	0.513	0.494	1.340	0.371	0.387	0.672	0.296
2743	0.224	-0.373	0.450	0.342	0.361	0.341	0.184	-0.625	0.142
2744	0.142	-0.895	0.224	0.307	0.135	0.508	0.363	0.520	0.097
2745	0.476	1.245	0.501	0.444	1.020	0.508	0.500	1.397	0.185
2746	0.050	-1.485	0.142	0.374	0.568	0.193	0.097	-1.181	0.185
2747	0.097	-1.181	0.328	0.342	0.361	0.102	0.261	-0.137	0.508
2748	0.453	1.093	0.224	0.509	1.434	0.273	0.482	1.279	0.358
2749	0.468	1.194	0.224	0.515	1.472	0.309	0.450	1.076	0.467
2750	0.142	-0.895	0.450	0.193	-0.600	0.341	0.326	0.285	0.262
2751	0.261	-0.137	0.358	0.102	-1.184	0.193	0.184	-0.625	0.142
2752	0.261	-0.137	0.481	0.275	-0.073	0.508	0.358	0.487	0.358
2753	0.184	-0.625	0.467	0.307	0.135	0.422	0.050	-1.485	0.450
2754	0.300	0.116	0.142	0.342	0.361	0.149	0.184	-0.625	0.097
2755	0.142	-0.895	0.467	0.196	-0.581	0.149	0.226	-0.356	0.450
2756	0.482	1.279	0.328	0.509	1.434	0.478	0.450	1.076	0.450
2757	0.511	1.464	0.508	0.421	0.869	0.478	0.466	1.178	0.296
2758	0.187	-0.608	0.262	0.149	-0.882	0.491	0.263	-0.120	0.467
2759	0.145	-0.878	0.224	0.149	-0.882	0.512	0.295	0.082	0.481
2760	0.489	1.329	0.296	0.480	1.246	0.501	0.447	1.060	0.467
2761	0.466	1.178	0.262	0.480	1.246	0.501	0.482	1.279	0.508
2762	0.513	1.481	0.142	0.488	1.302	0.235	0.447	1.060	0.492
2763	0.484	1.295	0.328	0.512	1.453	0.462	0.511	1.464	0.262
2764	0.508	1.447	0.467	0.500	1.378	0.371	0.426	0.925	0.224
2765	0.447	1.060	0.050	0.503	1.396	0.149	0.495	1.363	0.296
2766	0.226	-0.356	0.501	0.234	-0.336	0.102	0.329	0.301	0.467
2767	0.500	1.397	0.142	0.465	1.152	0.102	0.503	1.413	0.224
2768	0.142	-0.895	0.185	0.371	0.549	0.462	0.297	0.099	0.296
2769	0.329	0.301	0.296	0.149	-0.882	0.341	0.326	0.285	0.224
2770	0.224	-0.373	0.450	0.102	-1.184	0.462	0.224	-0.373	0.501
2771	0.224	-0.373	0.358	0.102	-1.184	0.501	0.224	-0.373	0.501
2772	0.450	1.076	0.512	0.456	1.095	0.235	0.500	1.397	0.296
2773	0.358	0.487	0.512	0.342	0.361	0.512	0.050	-1.485	0.508
2774	0.479	1.262	0.224	0.462	1.133	0.193	0.476	1.245	0.508
2775	0.463	1.161	0.481	0.497	1.359	0.508	0.508	1.447	0.450
2776	0.142	-0.895	0.481	0.234	-0.336	0.149	0.224	-0.373	0.467
2777	0.463	1.161	0.409	0.509	1.434	0.102	0.479	1.262	0.481
2778	0.332	0.318	0.450	0.193	-0.600	0.309	0.050	-1.485	0.501
2779	0.505	1.430	0.501	0.512	1.453	0.309	0.484	1.295	0.409
2780	0.263	-0.120	0.224	0.149	-0.882	0.235	0.142	-0.895	0.142
2781	0.224	-0.373	0.185	0.105	-1.165	0.309	0.297	0.099	0.097
2782	0.295	0.082	0.142	0.310	0.153	0.478	0.142	-0.895	0.328
2783	0.261	-0.137	0.513	0.310	0.153	0.235	0.097	-1.181	0.262
2784	0.142	-0.895	0.224	0.237	-0.317	0.501	0.187	-0.608	0.512
2785	0.329	0.301	0.508	0.310	0.153	0.235	0.187	-0.608	0.450
2786	0.466	1.178	0.224	0.480	1.246	0.309	0.500	1.397	0.450
2787	0.050	-1.485	0.513	0.237	-0.317	0.341	0.358	0.487	0.501
2788	0.505	1.430	0.513	0.503	1.396	0.491	0.468	1.194	0.050
2789	0.142	-0.895	0.050	0.234	-0.336	0.193	0.263	-0.120	0.481
2790	0.187	-0.608	0.501	0.310	0.153	0.478	0.142	-0.895	0.409
2791	0.297	0.099	0.492	0.339	0.342	0.478	0.361	0.504	0.358
2792	0.450	1.076	0.431	0.491	1.321	0.149	0.447	1.060	0.185
2793	0.358	0.487	0.492	0.196	-0.581	0.462	0.097	-1.181	0.508
2794	0.511	1.464	0.501	0.482	1.265	0.398	0.450	1.076	0.450
2795	0.511	1.464	0.492	0.482	1.265	0.508	0.450	1.076	0.262
2796	0.226	-0.356	0.492	0.149	-0.882	0.508	0.295	0.082	0.262
2797	0.476	1.245	0.262	0.468	1.170	0.478	0.471	1.211	0.501
2798	0.476	1.245	0.296	0.404	0.756	0.309	0.484	1.295	0.467
2799	0.408	0.807	0.185	0.509	1.434	0.235	0.461	1.144	0.513
2800	0.453	1.093	0.142	0.509	1.434	0.235	0.505	1.430	0.481
2801	0.503	1.413	0.467	0.474	1.208	0.478	0.489	1.329	0.328
2802	0.224	-0.373	0.513	0.310	0.153	0.149	0.261	-0.137	0.481

2803	0.184	-0.625	0.513	0.237	-0.317	0.508	0.224	-0.373	0.467
2804	0.142	-0.895	0.481	0.237	-0.317	0.193	0.297	0.099	0.492
2805	0.516	1.498	0.185	0.477	1.227	0.508	0.468	1.194	0.262
2806	0.226	-0.356	0.513	0.149	-0.882	0.508	0.224	-0.373	0.262
2807	0.508	1.447	0.467	0.515	1.472	0.193	0.295	0.082	0.142
2808	0.226	-0.356	0.296	0.193	-0.600	0.501	0.224	-0.373	0.185
2809	0.516	1.498	0.224	0.509	1.434	0.478	0.500	1.397	0.492
2810	0.516	1.498	0.492	0.509	1.434	0.478	0.500	1.397	0.481
2811	0.050	-1.485	0.481	0.196	-0.581	0.501	0.297	0.099	0.185
2812	0.497	1.380	0.450	0.503	1.396	0.462	0.503	1.413	0.431
2813	0.489	1.329	0.097	0.480	1.246	0.501	0.503	1.413	0.512
2814	0.429	0.942	0.450	0.480	1.246	0.491	0.447	1.060	0.450
2815	0.492	1.346	0.142	0.506	1.415	0.149	0.468	1.194	0.296
2816	0.505	1.430	0.450	0.465	1.152	0.235	0.482	1.279	0.501
2817	0.489	1.329	0.450	0.500	1.378	0.235	0.503	1.413	0.358
2818	0.492	1.346	0.385	0.491	1.321	0.149	0.405	0.790	0.481
2819	0.263	-0.120	0.097	0.152	-0.863	0.462	0.142	-0.895	0.409
2820	0.297	0.099	0.501	0.237	-0.317	0.508	0.097	-1.181	0.409
2821	0.187	-0.608	0.492	0.234	-0.336	0.508	0.332	0.318	0.467
2822	0.145	-0.878	0.513	0.149	-0.882	0.462	0.263	-0.120	0.358
2823	0.468	1.194	0.097	0.468	1.170	0.309	0.513	1.481	0.142
2824	0.518	1.514	0.142	0.503	1.396	0.512	0.447	1.060	0.142
2825	0.518	1.514	0.262	0.503	1.396	0.422	0.447	1.060	0.501
2826	0.479	1.262	0.142	0.459	1.114	0.444	0.497	1.380	0.224
2827	0.187	-0.608	0.512	0.310	0.153	0.235	0.050	-1.485	0.513
2828	0.508	1.447	0.481	0.509	1.434	0.444	0.479	1.262	0.296
2829	0.468	1.194	0.262	0.421	0.869	0.462	0.413	0.841	0.185
2830	0.297	0.099	0.512	0.442	1.001	0.193	0.361	0.504	0.450
2831	0.224	-0.373	0.450	0.234	-0.336	0.193	0.187	-0.608	0.262
2832	0.495	1.363	0.508	0.444	1.020	0.501	0.505	1.430	0.409
2833	0.482	1.279	0.296	0.468	1.170	0.398	0.447	1.060	0.450
2834	0.450	1.076	0.492	0.196	-0.581	0.508	0.261	-0.137	0.450
2835	0.097	-1.181	0.508	0.196	-0.581	0.512	0.261	-0.137	0.467
2836	0.447	1.060	0.508	0.503	1.396	0.478	0.495	1.363	0.512
2837	0.142	-0.895	0.224	0.401	0.737	0.149	0.000	?	0.492
2838	0.447	1.060	0.224	0.506	1.415	0.501	0.463	1.161	0.358
2839	0.450	1.076	0.513	0.512	1.453	0.478	0.511	1.464	0.481
2840	0.384	0.655	0.097	0.480	1.246	0.478	0.482	1.279	0.185
2841	0.097	-1.181	0.224	0.149	-0.882	0.193	0.329	0.301	0.481
2842	0.500	1.397	0.224	0.497	1.359	0.193	0.476	1.245	0.508
2843	0.492	1.346	0.492	0.477	1.227	0.309	0.466	1.178	0.481
2844	0.513	1.481	0.262	0.471	1.189	0.235	0.492	1.346	0.481
2845	0.097	-1.181	0.512	0.193	-0.600	0.444	0.263	-0.120	0.501
2846	0.142	-0.895	0.224	0.193	-0.600	0.512	0.263	-0.120	0.050
2847	0.261	-0.137	0.262	0.313	0.172	0.149	0.142	-0.895	0.262
2848	0.142	-0.895	0.508	0.234	-0.336	0.512	0.184	-0.625	0.492
2849	0.508	1.447	0.385	0.447	1.039	0.444	0.487	1.312	0.185
2850	0.479	1.262	0.481	0.512	1.453	0.444	0.482	1.279	0.512
2851	0.261	-0.137	0.481	0.149	-0.882	0.235	0.184	-0.625	0.142
2852	0.513	1.481	0.481	0.515	1.472	0.462	0.426	0.925	0.492
2853	0.447	1.060	0.492	0.444	1.020	0.508	0.505	1.430	0.328
2854	0.505	1.430	0.385	0.444	1.020	0.491	0.447	1.060	0.328
2855	0.297	0.099	0.467	0.237	-0.317	0.341	0.297	0.099	0.492
2856	0.492	1.346	0.142	0.471	1.189	0.341	0.500	1.397	0.296
2857	0.508	1.447	0.467	0.509	1.434	0.341	0.358	0.487	0.512
2858	0.508	1.447	0.262	0.491	1.321	0.149	0.482	1.279	0.328
2859	0.224	-0.373	0.508	0.342	0.361	0.193	0.411	0.824	0.262
2860	0.224	-0.373	0.492	0.342	0.361	0.193	0.411	0.824	0.512
2861	0.511	1.464	0.513	0.339	0.342	0.371	0.463	1.161	0.508
2862	0.097	-1.181	0.512	0.152	-0.863	0.235	0.355	0.470	0.508
2863	0.224	-0.373	0.431	0.193	-0.600	0.508	0.142	-0.895	0.508

2864	0.224	-0.373	0.262	0.193	-0.600	0.371	0.142	-0.895	0.262
2865	0.492	1.346	0.185	0.368	0.530	0.341	0.497	1.380	0.296
2866	0.263	-0.120	0.467	0.237	-0.317	0.491	0.224	-0.373	0.262
2867	0.511	1.464	0.358	0.512	1.453	0.273	0.508	1.447	0.508
2868	0.224	-0.373	0.512	0.371	0.549	0.508	0.295	0.082	0.296
2869	0.261	-0.137	0.142	0.342	0.361	0.508	0.184	-0.625	0.492
2870	0.508	1.447	0.508	0.491	1.321	0.508	0.447	1.060	0.185
2871	0.389	0.689	0.224	0.275	-0.073	0.491	0.261	-0.137	0.492
2872	0.476	1.245	0.262	0.503	1.396	0.422	0.408	0.807	0.224
2873	0.476	1.245	0.481	0.503	1.396	0.508	0.450	1.076	0.262
2874	0.476	1.245	0.262	0.503	1.396	0.053	0.450	1.076	0.450
2875	0.487	1.312	0.467	0.485	1.283	0.398	0.466	1.178	0.512
2876	0.387	0.672	0.262	0.424	0.888	0.235	0.513	1.481	0.508
2877	0.471	1.211	0.224	0.506	1.415	0.512	0.497	1.380	0.262
2878	0.142	-0.895	0.508	0.053	-1.504	0.491	0.361	0.504	0.512
2879	0.474	1.228	0.501	0.398	0.718	0.501	0.479	1.262	0.512
2880	0.261	-0.137	0.492	0.234	-0.336	0.501	0.184	-0.625	0.512
2881	0.505	1.430	0.508	0.509	1.434	0.508	0.476	1.245	0.508
2882	0.487	1.312	0.358	0.494	1.340	0.235	0.503	1.413	0.328
2883	0.516	1.498	0.262	0.497	1.359	0.149	0.482	1.279	0.501
2884	0.513	1.481	0.262	0.497	1.359	0.512	0.482	1.279	0.492
2885	0.429	0.942	0.467	0.503	1.396	0.149	0.505	1.430	0.328
2886	0.261	-0.137	0.185	0.234	-0.336	0.491	0.050	-1.485	0.512
2887	0.184	-0.625	0.512	0.152	-0.863	0.273	0.263	-0.120	0.508
2888	0.468	1.194	0.142	0.512	1.453	0.501	0.492	1.346	0.501
2889	0.358	0.487	0.501	0.149	-0.882	0.053	0.184	-0.625	0.467
2890	0.508	1.447	0.050	0.485	1.283	0.053	0.511	1.464	0.501
2891	0.142	-0.895	0.262	0.275	-0.073	0.478	0.145	-0.878	0.513
2892	0.508	1.447	0.224	0.497	1.359	0.235	0.489	1.329	0.501
2893	0.224	-0.373	0.512	0.053	-1.504	0.491	0.329	0.301	0.262
2894	0.261	-0.137	0.431	0.053	-1.504	0.102	0.329	0.301	0.296
2895	0.476	1.245	0.501	0.474	1.208	0.193	0.495	1.363	0.467
2896	0.261	-0.137	0.296	0.234	-0.336	0.478	0.303	0.133	0.481
2897	0.463	1.161	0.508	0.488	1.302	0.508	0.513	1.481	0.513
2898	0.263	-0.120	0.508	0.102	-1.184	0.501	0.326	0.285	0.513
2899	0.224	-0.373	0.508	0.196	-0.581	0.491	0.263	-0.120	0.431
2900	0.508	1.447	0.450	0.480	1.246	0.193	0.511	1.464	0.508
2901	0.503	1.413	0.142	0.506	1.415	0.273	0.505	1.430	0.409
2902	0.495	1.363	0.481	0.497	1.359	0.235	0.505	1.430	0.450
2903	0.508	1.447	0.467	0.491	1.321	0.462	0.505	1.430	0.450
2904	0.361	0.504	0.296	0.196	-0.581	0.341	0.261	-0.137	0.501
2905	0.263	-0.120	0.481	0.275	-0.073	0.462	0.295	0.082	0.481
2906	0.263	-0.120	0.481	0.234	-0.336	0.273	0.261	-0.137	0.512
2907	0.466	1.178	0.512	0.462	1.133	0.444	0.508	1.447	0.508
2908	0.187	-0.608	0.481	0.342	0.361	0.273	0.295	0.082	0.385
2909	0.516	1.498	0.481	0.465	1.152	0.273	0.492	1.346	0.185
2910	0.142	-0.895	0.508	0.272	-0.091	0.371	0.187	-0.608	0.512
2911	0.503	1.413	0.492	0.447	1.039	0.491	0.492	1.346	0.501
2912	0.050	-1.485	0.142	0.272	-0.091	0.508	0.226	-0.356	0.296
2913	0.263	-0.120	0.431	0.275	-0.073	0.491	0.261	-0.137	0.142
2914	0.224	-0.373	0.467	0.374	0.568	0.235	0.000	?	0.450
2915	0.508	1.447	0.450	0.491	1.321	0.512	0.447	1.060	0.492
2916	0.432	0.958	0.501	0.503	1.396	0.508	0.508	1.447	0.501
2917	0.503	1.413	0.501	0.491	1.321	0.508	0.511	1.464	0.508
2918	0.297	0.099	0.513	0.234	-0.336	0.501	0.266	-0.103	0.450
2919	0.508	1.447	0.492	0.509	1.434	0.193	0.516	1.498	0.185
2920	0.505	1.430	0.512	0.503	1.396	0.508	0.511	1.464	0.513
2921	0.505	1.430	0.512	0.503	1.396	0.508	0.511	1.464	0.513
2922	0.450	1.076	0.512	0.500	1.378	0.273	0.513	1.481	0.501
2923	0.142	-0.895	0.450	0.193	-0.600	0.444	0.326	0.285	0.501
2924	0.476	1.245	0.481	0.512	1.453	0.444	0.503	1.413	0.513

2925	0.463	1.161	0.513	0.512	1.453	0.491	0.492	1.346	0.262
2926	0.297	0.099	0.513	0.275	-0.073	0.508	0.332	0.318	0.262
2927	0.479	1.262	0.328	0.442	1.001	0.508	0.505	1.430	0.501
2928	0.479	1.262	0.224	0.442	1.001	0.478	0.503	1.413	0.431
2929	0.513	1.481	0.481	0.491	1.321	0.508	0.497	1.380	0.492
2930	0.479	1.262	0.508	0.512	1.453	0.193	0.461	1.144	0.501
2931	0.474	1.228	0.185	0.509	1.434	0.273	0.500	1.397	0.492
2932	0.508	1.447	0.224	0.477	1.227	0.508	0.511	1.464	0.492
2933	0.489	1.329	0.512	0.506	1.415	0.501	0.500	1.397	0.512
2934	0.142	-0.895	0.492	0.193	-0.600	0.501	0.261	-0.137	0.450
2935	0.437	0.992	0.409	0.272	-0.091	0.501	0.297	0.099	0.296
2936	0.463	1.161	0.501	0.512	1.453	0.512	0.468	1.194	0.501
2937	0.450	1.076	0.513	0.500	1.378	0.512	0.484	1.295	0.262
2938	0.495	1.363	0.296	0.497	1.359	0.512	0.513	1.481	0.481
2939	0.495	1.363	0.467	0.497	1.359	0.478	0.513	1.481	0.481
2940	0.508	1.447	0.467	0.515	1.472	0.478	0.426	0.925	0.501
2941	0.497	1.380	0.492	0.515	1.472	0.508	0.511	1.464	0.492
2942	0.511	1.464	0.492	0.509	1.434	0.491	0.405	0.790	0.224
2943	0.505	1.430	0.492	0.474	1.208	0.422	0.453	1.093	0.512
2944	0.505	1.430	0.185	0.474	1.208	0.398	0.453	1.093	0.296
2945	0.447	1.060	0.185	0.509	1.434	0.193	0.508	1.447	0.224
2946	0.479	1.262	0.481	0.485	1.283	0.149	0.476	1.245	0.501
2947	0.518	1.514	0.512	0.418	0.850	0.514	0.511	1.464	0.450
2948	0.518	1.514	0.467	0.395	0.700	0.501	0.508	1.447	0.481
2949	0.332	0.318	0.492	0.193	-0.600	0.235	0.382	0.638	0.508
2950	0.224	-0.373	0.501	0.149	-0.882	0.193	0.184	-0.625	0.501
2951	0.476	1.245	0.501	0.512	1.453	0.491	0.508	1.447	0.142
2952	0.508	1.447	0.431	0.497	1.359	0.478	0.495	1.363	0.508
2953	0.187	-0.608	0.501	0.240	-0.299	0.514	0.295	0.082	0.097
2954	0.226	-0.356	0.142	0.193	-0.600	0.478	0.142	-0.895	0.508
2955	0.505	1.430	0.431	0.491	1.321	0.491	0.445	1.043	0.185
2956	0.492	1.346	0.050	0.480	1.246	0.273	0.492	1.346	0.431
2957	0.411	0.824	0.508	0.515	1.472	0.501	0.497	1.380	0.224
2958	0.497	1.380	0.508	0.482	1.265	0.501	0.508	1.447	0.492
2959	0.511	1.464	0.508	0.488	1.302	0.501	0.445	1.043	0.512
2960	0.297	0.099	0.508	0.275	-0.073	0.501	0.187	-0.608	0.328
2961	0.466	1.178	0.142	0.497	1.359	0.444	0.513	1.481	0.431
2962	0.466	1.178	0.467	0.497	1.359	0.273	0.513	1.481	0.431
2963	0.489	1.329	0.262	0.497	1.359	0.273	0.503	1.413	0.508
2964	0.489	1.329	0.185	0.497	1.359	0.478	0.503	1.413	0.185
2965	0.487	1.312	0.467	0.444	1.020	0.508	0.511	1.464	0.508
2966	0.184	-0.625	0.409	0.275	-0.073	0.478	0.263	-0.120	0.097
2967	0.184	-0.625	0.467	0.275	-0.073	0.491	0.263	-0.120	0.467
2968	0.479	1.262	0.450	0.480	1.246	0.491	0.497	1.380	0.467
2969	0.508	1.447	0.450	0.503	1.396	0.491	0.432	0.958	0.513
2970	0.468	1.194	0.296	0.477	1.227	0.422	0.492	1.346	0.508
2971	0.487	1.312	0.450	0.488	1.302	0.508	0.500	1.397	0.512
2972	0.497	1.380	0.328	0.488	1.302	0.235	0.492	1.346	0.409
2973	0.497	1.380	0.481	0.488	1.302	0.444	0.492	1.346	0.512
2974	0.432	0.958	0.296	0.424	0.888	0.309	0.511	1.464	0.508
2975	0.500	1.397	0.501	0.506	1.415	0.512	0.450	1.076	0.467
2976	0.142	-0.895	0.224	0.234	-0.336	0.512	0.295	0.082	0.492
2977	0.432	0.958	0.501	0.447	1.039	0.514	0.500	1.397	0.513
2978	0.050	-1.485	0.508	0.310	0.153	0.514	0.261	-0.137	0.513
2979	0.505	1.430	0.185	0.515	1.472	0.422	0.484	1.295	0.501
2980	0.505	1.430	0.097	0.515	1.472	0.309	0.484	1.295	0.501
2981	0.508	1.447	0.492	0.518	1.491	0.273	0.497	1.380	0.385
2982	0.508	1.447	0.492	0.518	1.491	0.273	0.489	1.329	0.513
2983	0.142	-0.895	0.450	0.418	0.850	0.508	0.224	-0.373	0.513
2984	0.468	1.194	0.262	0.310	0.153	0.514	0.511	1.464	0.492
2985	0.263	-0.120	0.492	0.278	-0.054	0.514	0.295	0.082	0.385

2986	0.187	-0.608	0.142	0.275	-0.073	0.508	0.224	-0.373	0.185
2987	0.466	1.178	0.409	0.503	1.396	0.508	0.500	1.397	0.467
2988	0.408	0.807	0.409	0.518	1.491	0.235	0.445	1.043	0.492
2989	0.466	1.178	0.492	0.515	1.472	0.491	0.476	1.245	0.513
2990	0.450	1.076	0.481	0.512	1.453	0.149	0.503	1.413	0.481
2991	0.450	1.076	0.501	0.512	1.453	0.478	0.497	1.380	0.492
2992	0.297	0.099	0.262	0.234	-0.336	0.149	0.142	-0.895	0.501
2993	0.447	1.060	0.481	0.488	1.302	0.512	0.508	1.447	0.501
2994	0.329	0.301	0.492	0.152	-0.863	0.149	0.097	-1.181	0.501
2995	0.484	1.295	0.513	0.474	1.208	0.512	0.500	1.397	0.328
2996	0.300	0.116	0.501	0.149	-0.882	0.491	0.184	-0.625	0.501
2997	0.503	1.413	0.467	0.509	1.434	0.398	0.426	0.925	0.512
2998	0.229	-0.339	0.501	0.149	-0.882	0.149	0.224	-0.373	0.431
2999	0.497	1.380	0.481	0.509	1.434	0.508	0.492	1.346	0.431
3000	0.505	1.430	0.501	0.488	1.302	0.508	0.513	1.481	0.385
3001	0.184	-0.625	0.512	0.401	0.737	0.508	0.000	?	0.513
3002	0.097	-1.181	0.481	0.149	-0.882	0.149	0.332	0.318	0.328
3003	0.492	1.346	0.481	0.503	1.396	0.462	0.426	0.925	0.501
3004	0.492	1.346	0.501	0.503	1.396	0.341	0.426	0.925	0.097
3005	0.450	1.076	0.142	0.503	1.396	0.501	0.505	1.430	0.296
3006	0.261	-0.137	0.224	0.149	-0.882	0.501	0.184	-0.625	0.501
3007	0.492	1.346	0.481	0.459	1.114	0.508	0.503	1.413	0.262
3008	0.142	-0.895	0.501	0.345	0.379	0.501	0.097	-1.181	0.481
3009	0.411	0.824	0.481	0.503	1.396	0.462	0.468	1.194	0.185
3010	0.411	0.824	0.492	0.503	1.396	0.273	0.468	1.194	0.508
3011	0.492	1.346	0.492	0.506	1.415	0.478	0.508	1.447	0.512
3012	0.476	1.245	0.501	0.506	1.415	0.501	0.503	1.413	0.296
3013	0.497	1.380	0.501	0.465	1.152	0.501	0.508	1.447	0.358
3014	0.261	-0.137	0.513	0.275	-0.073	0.462	0.408	0.807	0.501
3015	0.479	1.262	0.512	0.480	1.246	0.501	0.505	1.430	0.262
3016	0.495	1.363	0.501	0.500	1.378	0.501	0.505	1.430	0.501
3017	0.508	1.447	0.492	0.497	1.359	0.462	0.468	1.194	0.296
3018	0.500	1.397	0.508	0.468	1.170	0.462	0.489	1.329	0.508
3019	0.466	1.178	0.492	0.494	1.340	0.478	0.513	1.481	0.467
3020	0.500	1.397	0.224	0.500	1.378	0.512	0.511	1.464	0.512
3021	0.482	1.279	0.481	0.462	1.133	0.512	0.497	1.380	0.512
3022	0.505	1.430	0.492	0.459	1.114	0.478	0.497	1.380	0.512
3023	0.503	1.413	0.492	0.477	1.227	0.149	0.384	0.655	0.296
3024	0.474	1.228	0.262	0.509	1.434	0.193	0.513	1.481	0.358
3025	0.474	1.228	0.262	0.509	1.434	0.514	0.513	1.481	0.492
3026	0.495	1.363	0.508	0.480	1.246	0.462	0.497	1.380	0.467
3027	0.142	-0.895	0.097	0.152	-0.863	0.508	0.384	0.655	0.097
3028	0.224	-0.373	0.508	0.196	-0.581	0.478	0.184	-0.625	0.431
3029	0.482	1.279	0.296	0.512	1.453	0.491	0.461	1.144	0.501
3030	0.505	1.430	0.409	0.459	1.114	0.514	0.484	1.295	0.185
3031	0.484	1.295	0.508	0.506	1.415	0.514	0.505	1.430	0.492
3032	0.492	1.346	0.142	0.477	1.227	0.514	0.479	1.262	0.097
3033	0.492	1.346	0.185	0.488	1.302	0.512	0.489	1.329	0.142
3034	0.492	1.346	0.513	0.518	1.491	0.444	0.497	1.380	0.262
3035	0.492	1.346	0.262	0.518	1.491	0.512	0.497	1.380	0.467
3036	0.508	1.447	0.513	0.512	1.453	0.501	0.500	1.397	0.296
3037	0.508	1.447	0.097	0.509	1.434	0.508	0.326	0.285	0.492
3038	0.495	1.363	0.467	0.447	1.039	0.309	0.500	1.397	0.508
3039	0.492	1.346	0.501	0.506	1.415	0.501	0.511	1.464	0.508
3040	0.500	1.397	0.481	0.500	1.378	0.501	0.429	0.942	0.097
3041	0.489	1.329	0.512	0.509	1.434	0.478	0.429	0.942	0.224
3042	0.226	-0.356	0.512	0.310	0.153	0.235	0.382	0.638	0.328
3043	0.484	1.295	0.050	0.506	1.415	0.341	0.513	1.481	0.450
3044	0.489	1.329	0.050	0.506	1.415	0.444	0.326	0.285	0.481
3045	0.495	1.363	0.508	0.480	1.246	0.193	0.495	1.363	0.501
3046	0.263	-0.120	0.512	0.234	-0.336	0.478	0.097	-1.181	0.512

3047	0.263	-0.120	0.262	0.342	0.361	0.235	0.300	0.116	0.513
3048	0.508	1.447	0.050	0.447	1.039	0.512	0.497	1.380	0.492
3049	0.097	-1.181	0.512	0.196	-0.581	0.478	0.263	-0.120	0.513
3050	0.505	1.430	0.224	0.477	1.227	0.149	0.482	1.279	0.142
3051	0.300	0.116	0.492	0.237	-0.317	0.235	0.184	-0.625	0.512
3052	0.408	0.807	0.262	0.512	1.453	0.462	0.508	1.447	0.501
3053	0.511	1.464	0.224	0.480	1.246	0.193	0.511	1.464	0.501
3054	0.142	-0.895	0.262	0.149	-0.882	0.444	0.295	0.082	0.508
3055	0.184	-0.625	0.512	0.234	-0.336	0.149	0.358	0.487	0.501
3056	0.513	1.481	0.296	0.459	1.114	0.508	0.495	1.363	0.097
3057	0.263	-0.120	0.501	0.193	-0.600	0.501	0.261	-0.137	0.050
3058	0.513	1.481	0.508	0.444	1.020	0.512	0.492	1.346	0.467
3059	0.097	-1.181	0.508	0.149	-0.882	0.501	0.300	0.116	0.508
3060	0.466	1.178	0.262	0.506	1.415	0.501	0.503	1.413	0.431
3061	0.503	1.413	0.262	0.497	1.359	0.273	0.463	1.161	0.508
3062	0.479	1.262	0.050	0.515	1.472	0.341	0.511	1.464	0.508
3063	0.511	1.464	0.501	0.503	1.396	0.422	0.511	1.464	0.501
3064	0.511	1.464	0.513	0.503	1.396	0.508	0.511	1.464	0.467
3065	0.050	-1.485	0.501	0.272	-0.091	0.235	0.297	0.099	0.450
3066	0.050	-1.485	0.450	0.342	0.361	0.102	0.361	0.504	0.097
3067	0.497	1.380	0.512	0.424	0.888	0.508	0.492	1.346	0.492
3068	0.511	1.464	0.481	0.506	1.415	0.444	0.461	1.144	0.492
3069	0.261	-0.137	0.501	0.240	-0.299	0.508	0.097	-1.181	0.431
3070	0.050	-1.485	0.185	0.102	-1.184	0.273	0.429	0.942	0.492
3071	0.513	1.481	0.501	0.503	1.396	0.309	0.500	1.397	0.467
3072	0.224	-0.373	0.501	0.447	1.039	0.193	0.184	-0.625	0.512
3073	0.487	1.312	0.508	0.512	1.453	0.508	0.489	1.329	0.262
3074	0.263	-0.120	0.501	0.275	-0.073	0.462	0.097	-1.181	0.262
3075	0.226	-0.356	0.508	0.310	0.153	0.512	0.142	-0.895	0.385
3076	0.261	-0.137	0.328	0.199	-0.562	0.508	0.263	-0.120	0.409
3077	0.508	1.447	0.224	0.509	1.434	0.508	0.468	1.194	0.481
3078	0.297	0.099	0.501	0.462	1.133	0.193	0.295	0.082	0.508
3079	0.500	1.397	0.492	0.509	1.434	0.273	0.487	1.312	0.385
3080	0.503	1.413	0.481	0.503	1.396	0.193	0.508	1.447	0.431
3081	0.503	1.413	0.512	0.503	1.396	0.371	0.508	1.447	0.481
3082	0.263	-0.120	0.513	0.193	-0.600	0.462	0.097	-1.181	0.358
3083	0.261	-0.137	0.501	0.272	-0.091	0.508	0.224	-0.373	0.185
3084	0.050	-1.485	0.492	0.193	-0.600	0.512	0.329	0.301	0.512
3085	0.500	1.397	0.492	0.371	0.549	0.444	0.447	1.060	0.501
3086	0.508	1.447	0.262	0.462	1.133	0.514	0.482	1.279	0.512
3087	0.495	1.363	0.501	0.509	1.434	0.478	0.497	1.380	0.450
3088	0.447	1.060	0.501	0.509	1.434	0.341	0.511	1.464	0.492
3089	0.508	1.447	0.481	0.444	1.020	0.501	0.516	1.498	0.512
3090	0.476	1.245	0.508	0.515	1.472	0.501	0.492	1.346	0.508
3091	0.492	1.346	0.508	0.474	1.208	0.501	0.518	1.514	0.262
3092	0.184	-0.625	0.501	0.339	0.342	0.478	0.142	-0.895	0.097
3093	0.500	1.397	0.185	0.503	1.396	0.501	0.505	1.430	0.097
3094	0.503	1.413	0.142	0.503	1.396	0.193	0.495	1.363	0.508
3095	0.508	1.447	0.512	0.503	1.396	0.341	0.495	1.363	0.508
3096	0.503	1.413	0.512	0.477	1.227	0.512	0.503	1.413	0.501
3097	0.508	1.447	0.513	0.503	1.396	0.422	0.495	1.363	0.508
3098	0.329	0.301	0.508	0.193	-0.600	0.512	0.097	-1.181	0.409
3099	0.224	-0.373	0.328	0.345	0.379	0.491	0.050	-1.485	0.512
3100	0.497	1.380	0.508	0.512	1.453	0.478	0.463	1.161	0.512
3101	0.492	1.346	0.513	0.418	0.850	0.491	0.505	1.430	0.097
3102	0.482	1.279	0.050	0.512	1.453	0.512	0.432	0.958	0.224
3103	0.513	1.481	0.097	0.494	1.340	0.508	0.511	1.464	0.224
3104	0.513	1.481	0.508	0.480	1.246	0.235	0.511	1.464	0.512
3105	0.500	1.397	0.409	0.491	1.321	0.491	0.500	1.397	0.224
3106	0.487	1.312	0.385	0.509	1.434	0.491	0.466	1.178	0.512
3107	0.492	1.346	0.508	0.503	1.396	0.514	0.447	1.060	0.508

3108	0.266	−0.103	0.467	0.234	−0.336	0.444	0.097	−1.181	0.501
3109	0.500	1.397	0.481	0.491	1.321	0.444	0.484	1.295	0.512
3110	0.500	1.397	0.501	0.491	1.321	0.512	0.484	1.295	0.481
3111	0.482	1.279	0.097	0.515	1.472	0.235	0.429	0.942	0.467
3112	0.511	1.464	0.097	0.447	1.039	0.149	0.492	1.346	0.508
3113	0.511	1.464	0.050	0.447	1.039	0.512	0.468	1.194	0.409
3114	0.495	1.363	0.508	0.509	1.434	0.478	0.516	1.498	0.409
3115	0.184	−0.625	0.508	0.234	−0.336	0.508	0.261	−0.137	0.513
3116	0.142	−0.895	0.492	0.149	−0.882	0.512	0.261	−0.137	0.224
3117	0.500	1.397	0.467	0.512	1.453	0.371	0.387	0.672	0.262
3118	0.500	1.397	0.467	0.477	1.227	0.512	0.411	0.824	0.501
3119	0.511	1.464	0.508	0.509	1.434	0.512	0.476	1.245	0.467
3120	0.505	1.430	0.508	0.509	1.434	0.309	0.505	1.430	0.512
3121	0.329	0.301	0.296	0.371	0.549	0.341	0.387	0.672	0.431
3122	0.505	1.430	0.185	0.509	1.434	0.462	0.429	0.942	0.450
3123	0.508	1.447	0.185	0.515	1.472	0.512	0.479	1.262	0.512
3124	0.050	−1.485	0.385	0.307	0.135	0.491	0.358	0.487	0.328
3125	0.097	−1.181	0.050	0.345	0.379	0.371	0.184	−0.625	0.492
3126	0.503	1.413	0.512	0.465	1.152	0.501	0.505	1.430	0.224
3127	0.405	0.790	0.512	0.515	1.472	0.478	0.497	1.380	0.492
3128	0.384	0.655	0.512	0.494	1.340	0.512	0.508	1.447	0.262
3129	0.508	1.447	0.512	0.371	0.549	0.193	0.445	1.043	0.142
3130	0.468	1.194	0.492	0.503	1.396	0.341	0.497	1.380	0.508
3131	0.474	1.228	0.508	0.474	1.208	0.371	0.521	1.531	0.050
3132	0.492	1.346	0.508	0.509	1.434	0.444	0.511	1.464	0.467
3133	0.097	−1.181	0.050	0.196	−0.581	0.444	0.261	−0.137	0.467
3134	0.097	−1.181	0.050	0.342	0.361	0.422	0.097	−1.181	0.467
3135	0.050	−1.485	0.501	0.371	0.549	0.398	0.097	−1.181	0.328
3136	0.505	1.430	0.050	0.442	1.001	0.508	0.505	1.430	0.262
3137	0.505	1.430	0.492	0.442	1.001	0.478	0.505	1.430	0.467
3138	0.484	1.295	0.481	0.424	0.888	0.501	0.500	1.397	0.328
3139	0.461	1.144	0.501	0.398	0.718	0.149	0.503	1.413	0.328
3140	0.461	1.144	0.508	0.509	1.434	0.193	0.405	0.790	0.513
3141	0.497	1.380	0.508	0.480	1.246	0.149	0.516	1.498	0.262
3142	0.497	1.380	0.050	0.506	1.415	0.462	0.516	1.498	0.358
3143	0.297	0.099	0.492	0.149	−0.882	0.371	0.097	−1.181	0.358
3144	0.187	−0.608	0.097	0.196	−0.581	0.478	0.224	−0.373	0.508
3145	0.187	−0.608	0.513	0.149	−0.882	0.501	0.224	−0.373	0.492
3146	0.387	0.672	0.224	0.462	1.133	0.478	0.503	1.413	0.513
3147	0.050	−1.485	0.501	0.371	0.549	0.491	0.226	−0.356	0.512
3148	0.503	1.413	0.185	0.482	1.265	0.462	0.511	1.464	0.358
3149	0.503	1.413	0.512	0.506	1.415	0.508	0.508	1.447	0.358
3150	0.503	1.413	0.512	0.482	1.265	0.508	0.500	1.397	0.262
3151	0.503	1.413	0.512	0.494	1.340	0.053	0.513	1.481	0.262
3152	0.487	1.312	0.050	0.462	1.133	0.053	0.484	1.295	0.431
3153	0.505	1.430	0.050	0.506	1.415	0.508	0.468	1.194	0.508
3154	0.500	1.397	0.513	0.503	1.396	0.309	0.511	1.464	0.185
3155	0.050	−1.485	0.501	0.053	−1.504	0.235	0.411	0.824	0.185
3156	0.050	−1.485	0.050	0.053	−1.504	0.501	0.411	0.824	0.296
3157	0.495	1.363	0.185	0.509	1.434	0.514	0.511	1.464	0.224
3158	0.000	?	0.185	0.310	0.153	0.422	0.226	−0.356	0.512
3159	0.050	−1.485	0.481	0.234	−0.336	0.193	0.263	−0.120	0.512
3160	0.487	1.312	0.512	0.497	1.359	0.508	0.500	1.397	0.501
3161	0.479	1.262	0.492	0.509	1.434	0.512	0.463	1.161	0.409
3162	0.500	1.397	0.492	0.418	0.850	0.273	0.511	1.464	0.450
3163	0.000	?	0.097	0.193	−0.600	0.508	0.432	0.958	0.492
3164	0.497	1.380	0.296	0.512	1.453	0.309	0.447	1.060	0.501
3165	0.503	1.413	0.262	0.518	1.491	0.491	0.513	1.481	0.450
3166	0.050	−1.485	0.501	0.272	−0.091	0.273	0.329	0.301	0.185
3167	0.484	1.295	0.513	0.512	1.453	0.371	0.495	1.363	0.224
3168	0.097	−1.181	0.097	0.310	0.153	0.512	0.224	−0.373	0.481

3169	0.503	1.413	0.097	0.491	1.321	0.309	0.492	1.346	0.097
3170	0.226	-0.356	0.097	0.275	-0.073	0.514	0.263	-0.120	0.513
3171	0.000	?	0.185	0.371	0.549	0.508	0.142	-0.895	0.328
3172	0.497	1.380	0.508	0.512	1.453	0.462	0.505	1.430	0.224
3173	0.187	-0.608	0.508	0.307	0.135	0.149	0.050	-1.485	0.431
3174	0.503	1.413	0.508	0.515	1.472	0.235	0.468	1.194	0.481
3175	0.503	1.413	0.501	0.509	1.434	0.514	0.468	1.194	0.185
3176	0.503	1.413	0.513	0.465	1.152	0.193	0.471	1.211	0.224
3177	0.050	-1.485	0.492	0.149	-0.882	0.235	0.329	0.301	0.185
3178	0.050	-1.485	0.409	0.237	-0.317	0.478	0.261	-0.137	0.492
3179	0.500	1.397	0.050	0.518	1.491	0.235	0.468	1.194	0.224
3180	0.000	?	0.097	0.196	-0.581	0.235	0.329	0.301	0.431
3181	0.000	?	0.467	0.234	-0.336	0.193	0.332	0.318	0.481
3182	0.495	1.363	0.142	0.474	1.208	0.462	0.513	1.481	0.142
3183	0.050	-1.485	0.508	0.237	-0.317	0.462	0.263	-0.120	0.513
3184	0.187	-0.608	0.142	0.237	-0.317	0.444	0.358	0.487	0.512
3185	0.187	-0.608	0.097	0.196	-0.581	0.512	0.358	0.487	0.450
3186	0.479	1.262	0.512	0.462	1.133	0.309	0.505	1.430	0.508
3187	0.511	1.464	0.097	0.465	1.152	0.273	0.492	1.346	0.358
3188	0.492	1.346	0.262	0.447	1.039	0.273	0.516	1.498	0.513
3189	0.484	1.295	0.050	0.518	1.491	0.235	0.508	1.447	0.512
3190	0.000	?	0.512	0.310	0.153	0.462	0.355	0.470	0.501
3191	0.097	-1.181	0.508	0.275	-0.073	0.478	0.355	0.470	0.492
3192	0.300	0.116	0.508	0.272	-0.091	0.309	0.261	-0.137	0.492
3193	0.266	-0.103	0.097	0.234	-0.336	0.309	0.261	-0.137	0.513
3194	0.495	1.363	0.508	0.462	1.133	0.371	0.426	0.925	0.508
3195	0.505	1.430	0.492	0.474	1.208	0.273	0.511	1.464	0.513
3196	0.100	-1.164	0.513	0.310	0.153	0.508	0.184	-0.625	0.224
3197	0.100	-1.164	0.508	0.310	0.153	0.508	0.184	-0.625	0.262
3198	0.100	-1.164	0.512	0.371	0.549	0.491	0.297	0.099	0.409
3199	0.187	-0.608	0.508	0.272	-0.091	0.273	0.224	-0.373	0.501
3200	0.497	1.380	0.501	0.509	1.434	0.491	0.516	1.498	0.467
3201	0.497	1.380	0.508	0.509	1.434	0.491	0.516	1.498	0.481
3202	0.497	1.380	0.508	0.491	1.321	0.491	0.503	1.413	0.512
3203	0.000	?	0.481	0.272	-0.091	0.478	0.411	0.824	0.508
3204	0.495	1.363	0.481	0.488	1.302	0.341	0.450	1.076	0.508
3205	0.503	1.413	0.508	0.497	1.359	0.273	0.489	1.329	0.481
3206	0.487	1.312	0.513	0.494	1.340	0.508	0.500	1.397	0.481
3207	0.408	0.807	0.512	0.474	1.208	0.341	0.445	1.043	0.097
3208	0.050	-1.485	0.513	0.342	0.361	0.501	0.184	-0.625	0.492
3209	0.100	-1.164	0.513	0.272	-0.091	0.309	0.224	-0.373	0.512
3210	0.463	1.161	0.492	0.506	1.415	0.235	0.479	1.262	0.492
3211	0.142	-0.895	0.501	0.342	0.361	0.193	0.097	-1.181	0.512
3212	0.497	1.380	0.508	0.506	1.415	0.398	0.511	1.464	0.508
3213	0.145	-0.878	0.513	0.313	0.172	0.309	0.326	0.285	0.492
3214	0.097	-1.181	0.513	0.237	-0.317	0.235	0.224	-0.373	0.492
3215	0.000	?	0.450	0.193	-0.600	0.444	0.434	0.975	0.467
3216	0.500	1.397	0.513	0.401	0.737	0.462	0.479	1.262	0.450
3217	0.097	-1.181	0.501	0.307	0.135	0.422	0.184	-0.625	0.450
3218	0.263	-0.120	0.481	0.237	-0.317	0.501	0.226	-0.356	0.512
3219	0.050	-1.485	0.481	0.447	1.039	0.491	0.184	-0.625	0.512
3220	0.505	1.430	0.481	0.465	1.152	0.309	0.492	1.346	0.508
3221	0.000	?	0.501	0.424	0.888	0.444	0.224	-0.373	0.513
3222	0.497	1.380	0.501	0.500	1.378	0.512	0.432	0.958	0.409
3223	0.497	1.380	0.501	0.494	1.340	0.491	0.479	1.262	0.513
3224	0.100	-1.164	0.467	0.307	0.135	0.501	0.142	-0.895	0.501
3225	0.505	1.430	0.492	0.442	1.001	0.193	0.513	1.481	0.142
3226	0.484	1.295	0.501	0.506	1.415	0.444	0.516	1.498	0.513
3227	0.503	1.413	0.501	0.494	1.340	0.501	0.447	1.060	0.262
3228	0.497	1.380	0.431	0.503	1.396	0.512	0.508	1.447	0.296
3229	0.000	?	0.385	0.193	-0.600	0.512	0.361	0.504	0.481

3230	0.505	1.430	0.097	0.442	1.001	0.512	0.511	1.464	0.508
3231	0.500	1.397	0.501	0.503	1.396	0.512	0.511	1.464	0.508
3232	0.497	1.380	0.512	0.512	1.453	0.444	0.503	1.413	0.296
3233	0.503	1.413	0.512	0.512	1.453	0.512	0.489	1.329	0.224
3234	0.503	1.413	0.262	0.512	1.453	0.309	0.489	1.329	0.512
3235	0.476	1.245	0.224	0.512	1.453	0.341	0.513	1.481	0.409
3236	0.482	1.279	0.512	0.444	1.020	0.462	0.511	1.464	0.050
3237	0.500	1.397	0.513	0.518	1.491	0.508	0.516	1.498	0.513
3238	0.000	?	0.512	0.313	0.172	0.491	0.224	-0.373	0.501
3239	0.000	?	0.185	0.345	0.379	0.491	0.261	-0.137	0.450
3240	0.513	1.481	0.224	0.465	1.152	0.491	0.411	0.824	0.501
3241	0.503	1.413	0.501	0.512	1.453	0.501	0.503	1.413	0.513
3242	0.503	1.413	0.508	0.497	1.359	0.501	0.463	1.161	0.513
3243	0.503	1.413	0.224	0.497	1.359	0.501	0.476	1.245	0.501
3244	0.487	1.312	0.450	0.491	1.321	0.501	0.511	1.464	0.224
3245	0.500	1.397	0.513	0.500	1.378	0.398	0.505	1.430	0.481
3246	0.505	1.430	0.508	0.500	1.378	0.508	0.505	1.430	0.467
3247	0.503	1.413	0.501	0.500	1.378	0.478	0.482	1.279	0.492
3248	0.503	1.413	0.513	0.500	1.378	0.501	0.482	1.279	0.492
3249	0.450	1.076	0.481	0.401	0.737	0.501	0.097	-1.181	0.508
3250	0.503	1.413	0.501	0.509	1.434	0.501	0.495	1.363	0.224
3251	0.492	1.346	0.185	0.477	1.227	0.444	0.513	1.481	0.501
3252	0.479	1.262	0.508	0.503	1.396	0.444	0.495	1.363	0.262
3253	0.479	1.262	0.492	0.503	1.396	0.462	0.511	1.464	0.328
3254	0.479	1.262	0.508	0.503	1.396	0.514	0.508	1.447	0.097
3255	0.497	1.380	0.481	0.442	1.001	0.514	0.492	1.346	0.450
3256	0.497	1.380	0.492	0.442	1.001	0.491	0.492	1.346	0.358
3257	0.500	1.397	0.224	0.459	1.114	0.491	0.463	1.161	0.358
3258	0.461	1.144	0.508	0.512	1.453	0.491	0.455	1.110	0.508
3259	0.489	1.329	0.185	0.518	1.491	0.501	0.445	1.043	0.450
3260	0.497	1.380	0.262	0.485	1.283	0.422	0.513	1.481	0.492
3261	0.497	1.380	0.224	0.485	1.283	0.491	0.513	1.481	0.492
3262	0.432	0.958	0.512	0.488	1.302	0.462	0.511	1.464	0.224
3263	0.384	0.655	0.224	0.497	1.359	0.371	0.511	1.464	0.492
3264	0.097	-1.181	0.262	0.421	0.869	0.508	0.413	0.841	0.508
3265	0.495	1.363	0.512	0.494	1.340	0.235	0.513	1.481	0.450
3266	0.500	1.397	0.409	0.468	1.170	0.273	0.508	1.447	0.512
3267	0.000	?	0.508	0.374	0.568	0.512	0.142	-0.895	0.512
3268	0.513	1.481	0.512	0.509	1.434	0.508	0.513	1.481	0.409
3269	0.261	-0.137	0.185	0.234	-0.336	0.508	0.261	-0.137	0.224
3270	0.224	-0.373	0.467	0.275	-0.073	0.193	0.295	0.082	0.467
3271	0.500	1.397	0.512	0.515	1.472	0.273	0.482	1.279	0.508
3272	0.511	1.464	0.513	0.503	1.396	0.491	0.508	1.447	0.492
3273	0.511	1.464	0.481	0.503	1.396	0.501	0.508	1.447	0.508
3274	0.187	-0.608	0.481	0.193	-0.600	0.398	0.297	0.099	0.508
3275	0.226	-0.356	0.097	0.272	-0.091	0.508	0.226	-0.356	0.492
3276	0.492	1.346	0.481	0.491	1.321	0.512	0.511	1.464	0.467
3277	0.508	1.447	0.481	0.500	1.378	0.462	0.413	0.841	0.501
3278	0.226	-0.356	0.492	0.401	0.737	0.491	0.050	-1.485	0.467
3279	0.450	1.076	0.508	0.509	1.434	0.462	0.516	1.498	0.097
3280	0.508	1.447	0.492	0.506	1.415	0.508	0.503	1.413	0.501
3281	0.505	1.430	0.492	0.465	1.152	0.462	0.453	1.093	0.512
3282	0.500	1.397	0.513	0.494	1.340	0.149	0.505	1.430	0.481
3283	0.508	1.447	0.501	0.462	1.133	0.501	0.508	1.447	0.328
3284	0.484	1.295	0.501	0.509	1.434	0.508	0.516	1.498	0.431
3285	0.503	1.413	0.296	0.462	1.133	0.512	0.497	1.380	0.492
3286	0.184	-0.625	0.481	0.149	-0.882	0.491	0.224	-0.373	0.512
3287	0.505	1.430	0.492	0.494	1.340	0.514	0.482	1.279	0.508
3288	0.489	1.329	0.513	0.506	1.415	0.102	0.468	1.194	0.481
3289	0.508	1.447	0.142	0.506	1.415	0.491	0.495	1.363	0.185
3290	0.482	1.279	0.513	0.494	1.340	0.149	0.492	1.346	0.512

3291	0.484	1.295	0.512	0.515	1.472	0.273	0.505	1.430	0.512
3292	0.229	-0.339	0.501	0.102	-1.184	0.235	0.224	-0.373	0.501
3293	0.505	1.430	0.501	0.494	1.340	0.462	0.500	1.397	0.501
3294	0.184	-0.625	0.512	0.149	-0.882	0.235	0.261	-0.137	0.224
3295	0.261	-0.137	0.224	0.275	-0.073	0.398	0.329	0.301	0.224
3296	0.226	-0.356	0.501	0.234	-0.336	0.462	0.097	-1.181	0.431
3297	0.511	1.464	0.492	0.462	1.133	0.235	0.447	1.060	0.450
3298	0.224	-0.373	0.512	0.234	-0.336	0.478	0.358	0.487	0.513
3299	0.261	-0.137	0.492	0.404	0.756	0.514	0.358	0.487	0.481
3300	0.513	1.481	0.142	0.468	1.170	0.273	0.505	1.430	0.142
3301	0.413	0.841	0.142	0.237	-0.317	0.491	0.447	1.060	0.508
3302	0.503	1.413	0.467	0.477	1.227	0.512	0.489	1.329	0.481
3303	0.508	1.447	0.501	0.515	1.472	0.512	0.489	1.329	0.409
3304	0.187	-0.608	0.481	0.272	-0.091	0.478	0.226	-0.356	0.185
3305	0.461	1.144	0.450	0.491	1.321	0.478	0.489	1.329	0.492
3306	0.505	1.430	0.224	0.509	1.434	0.398	0.508	1.447	0.224
3307	0.503	1.413	0.501	0.518	1.491	0.309	0.455	1.110	0.097
3308	0.479	1.262	0.513	0.480	1.246	0.508	0.511	1.464	0.481
3309	0.479	1.262	0.097	0.480	1.246	0.491	0.511	1.464	0.467
3310	0.097	-1.181	0.142	0.404	0.756	0.514	0.408	0.807	0.450
3311	0.000	?	0.481	0.310	0.153	0.512	0.224	-0.373	0.501
3312	0.476	1.245	0.224	0.503	1.396	0.462	0.466	1.178	0.512
3313	0.474	1.228	0.142	0.491	1.321	0.508	0.505	1.430	0.508
3314	0.492	1.346	0.512	0.515	1.472	0.508	0.492	1.346	0.512
3315	0.508	1.447	0.512	0.515	1.472	0.398	0.503	1.413	0.431
3316	0.492	1.346	0.512	0.465	1.152	0.501	0.511	1.464	0.492
3317	0.484	1.295	0.513	0.506	1.415	0.235	0.492	1.346	0.224
3318	0.505	1.430	0.431	0.509	1.434	0.508	0.468	1.194	0.492
3319	0.492	1.346	0.467	0.404	0.756	0.501	0.508	1.447	0.501
3320	0.492	1.346	0.492	0.506	1.415	0.491	0.468	1.194	0.142
3321	0.295	0.082	0.492	0.234	-0.336	0.149	0.097	-1.181	0.467
3322	0.479	1.262	0.501	0.509	1.434	0.478	0.503	1.413	0.467
3323	0.489	1.329	0.185	0.503	1.396	0.512	0.503	1.413	0.185
3324	0.513	1.481	0.512	0.485	1.283	0.491	0.479	1.262	0.385
3325	0.142	-0.895	0.492	0.149	-0.882	0.491	0.326	0.285	0.431
3326	0.511	1.464	0.224	0.477	1.227	0.478	0.434	0.975	0.513
3327	0.508	1.447	0.512	0.512	1.453	0.149	0.487	1.312	0.513
3328	0.500	1.397	0.512	0.485	1.283	0.512	0.508	1.447	0.409
3329	0.500	1.397	0.262	0.485	1.283	0.501	0.505	1.430	0.512
3330	0.508	1.447	0.492	0.474	1.208	0.422	0.479	1.262	0.492
3331	0.226	-0.356	0.224	0.149	-0.882	0.501	0.187	-0.608	0.492
3332	0.497	1.380	0.481	0.506	1.415	0.341	0.513	1.481	0.185
3333	0.484	1.295	0.481	0.494	1.340	0.341	0.516	1.498	0.512
3334	0.503	1.413	0.512	0.424	0.888	0.462	0.500	1.397	0.431
3335	0.484	1.295	0.467	0.497	1.359	0.512	0.503	1.413	0.467
3336	0.142	-0.895	0.513	0.342	0.361	0.508	0.224	-0.373	0.467
3337	0.142	-0.895	0.512	0.342	0.361	0.491	0.224	-0.373	0.224
3338	0.466	1.178	0.224	0.462	1.133	0.193	0.434	0.975	0.224
3339	0.497	1.380	0.508	0.509	1.434	0.491	0.447	1.060	0.097
3340	0.476	1.245	0.513	0.506	1.415	0.514	0.511	1.464	0.512
3341	0.447	1.060	0.481	0.488	1.302	0.193	0.484	1.295	0.142
3342	0.224	-0.373	0.501	0.196	-0.581	0.235	0.142	-0.895	0.296
3343	0.503	1.413	0.358	0.488	1.302	0.512	0.511	1.464	0.409
3344	0.505	1.430	0.358	0.520	1.509	0.102	0.487	1.312	0.481
3345	0.097	-1.181	0.262	0.193	-0.600	0.309	0.411	0.824	0.508
3346	0.142	-0.895	0.467	0.237	-0.317	0.508	0.187	-0.608	0.431
3347	0.482	1.279	0.262	0.506	1.415	0.508	0.487	1.312	0.385
3348	0.224	-0.373	0.262	0.102	-1.184	0.512	0.226	-0.356	0.431
3349	0.142	-0.895	0.328	0.307	0.135	0.508	0.097	-1.181	0.431
3350	0.508	1.447	0.508	0.506	1.415	0.512	0.482	1.279	0.492
3351	0.508	1.447	0.142	0.506	1.415	0.478	0.468	1.194	0.508

3352	0.508	1.447	0.501	0.509	1.434	0.478	0.450	1.076	0.513
3353	0.513	1.481	0.097	0.503	1.396	0.512	0.497	1.380	0.224
3354	0.426	0.925	0.050	0.512	1.453	0.512	0.508	1.447	0.492
3355	0.468	1.194	0.050	0.477	1.227	0.371	0.513	1.481	0.385
3356	0.489	1.329	0.050	0.477	1.227	0.341	0.508	1.447	0.262
3357	0.492	1.346	0.409	0.512	1.453	0.512	0.426	0.925	0.450
3358	0.500	1.397	0.481	0.509	1.434	0.309	0.489	1.329	0.097
3359	0.187	-0.608	0.492	0.368	0.530	0.478	0.224	-0.373	0.481
3360	0.505	1.430	0.050	0.339	0.342	0.514	0.492	1.346	0.262
3361	0.487	1.312	0.492	0.509	1.434	0.149	0.500	1.397	0.492
3362	0.224	-0.373	0.050	0.307	0.135	0.512	0.142	-0.895	0.492
3363	0.500	1.397	0.224	0.482	1.265	0.398	0.471	1.211	0.508
3364	0.500	1.397	0.431	0.520	1.509	0.398	0.466	1.178	0.508
3365	0.266	-0.103	0.262	0.149	-0.882	0.512	0.184	-0.625	0.512
3366	0.487	1.312	0.481	0.512	1.453	0.501	0.389	0.689	0.508
3367	0.224	-0.373	0.097	0.000	?	0.462	0.434	0.975	0.513
3368	0.479	1.262	0.450	0.398	0.718	0.491	0.516	1.498	0.142
3369	0.479	1.262	0.481	0.398	0.718	0.235	0.516	1.498	0.185
3370	0.503	1.413	0.501	0.518	1.491	0.422	0.411	0.824	0.492
3371	0.463	1.161	0.513	0.503	1.396	0.508	0.516	1.498	0.097
3372	0.513	1.481	0.513	0.462	1.133	0.508	0.497	1.380	0.224
3373	0.508	1.447	0.492	0.491	1.321	0.514	0.487	1.312	0.512
3374	0.226	-0.356	0.513	0.237	-0.317	0.341	0.184	-0.625	0.358
3375	0.500	1.397	0.296	0.427	0.907	0.341	0.513	1.481	0.262
3376	0.503	1.413	0.501	0.512	1.453	0.235	0.434	0.975	0.262
3377	0.479	1.262	0.512	0.506	1.415	0.512	0.461	1.144	0.262
3378	0.500	1.397	0.358	0.512	1.453	0.235	0.461	1.144	0.467
3379	0.358	0.487	0.142	0.345	0.379	0.193	0.226	-0.356	0.513
3380	0.358	0.487	0.481	0.345	0.379	0.235	0.226	-0.356	0.508
3381	0.266	-0.103	0.262	0.234	-0.336	0.491	0.097	-1.181	0.512
3382	0.463	1.161	0.185	0.515	1.472	0.371	0.511	1.464	0.409
3383	0.266	-0.103	0.142	0.234	-0.336	0.491	0.142	-0.895	0.262
3384	0.263	-0.120	0.185	0.196	-0.581	0.149	0.297	0.099	0.492
3385	0.329	0.301	0.512	0.237	-0.317	0.149	0.408	0.807	0.492
3386	0.505	1.430	0.508	0.485	1.283	0.102	0.482	1.279	0.467
3387	0.142	-0.895	0.512	0.368	0.530	0.149	0.000	?	0.467
3388	0.503	1.413	0.450	0.491	1.321	0.512	0.511	1.464	0.467
3389	0.097	-1.181	0.512	0.152	-0.863	0.512	0.429	0.942	0.431
3390	0.050	-1.485	0.512	0.152	-0.863	0.512	0.382	0.638	0.431
3391	0.050	-1.485	0.492	0.105	-1.165	0.273	0.429	0.942	0.467
3392	0.050	-1.485	0.512	0.152	-0.863	0.501	0.429	0.942	0.501
3393	0.408	0.807	0.508	0.512	1.453	0.053	0.492	1.346	0.450
3394	0.476	1.245	0.508	0.518	1.491	0.235	0.508	1.447	0.224
3395	0.489	1.329	0.513	0.518	1.491	0.422	0.516	1.498	0.481
3396	0.050	-1.485	0.513	0.275	-0.073	0.309	0.226	-0.356	0.481
3397	0.484	1.295	0.501	0.506	1.415	0.371	0.489	1.329	0.224
3398	0.050	-1.485	0.512	0.053	-1.504	0.193	0.387	0.672	0.467
3399	0.226	-0.356	0.492	0.234	-0.336	0.491	0.266	-0.103	0.508
3400	0.432	0.958	0.142	0.424	0.888	0.508	0.445	1.043	0.512
3401	0.261	-0.137	0.508	0.307	0.135	0.462	0.097	-1.181	0.501
3402	0.479	1.262	0.508	0.374	0.568	0.444	0.482	1.279	0.481
3403	0.097	-1.181	0.142	0.193	-0.600	0.478	0.263	-0.120	0.328
3404	0.447	1.060	0.501	0.491	1.321	0.478	0.497	1.380	0.492
3405	0.476	1.245	0.501	0.509	1.434	0.491	0.495	1.363	0.450
3406	0.495	1.363	0.508	0.462	1.133	0.193	0.505	1.430	0.481
3407	0.508	1.447	0.501	0.447	1.039	0.512	0.500	1.397	0.492
3408	0.511	1.464	0.508	0.477	1.227	0.512	0.511	1.464	0.492
3409	0.489	1.329	0.262	0.477	1.227	0.309	0.508	1.447	0.492
3410	0.511	1.464	0.492	0.488	1.302	0.371	0.511	1.464	0.328
3411	0.295	0.082	0.467	0.193	-0.600	0.478	0.142	-0.895	0.501
3412	0.503	1.413	0.481	0.515	1.472	0.341	0.184	-0.625	0.385

3413	0.508	1.447	0.501	0.518	1.491	0.235	0.492	1.346	0.492
3414	0.358	0.487	0.492	0.310	0.153	0.235	0.097	-1.181	0.296
3415	0.142	-0.895	0.512	0.371	0.549	0.193	0.224	-0.373	0.501
3416	0.482	1.279	0.224	0.477	1.227	0.508	0.508	1.447	0.508
3417	0.263	-0.120	0.501	0.339	0.342	0.478	0.358	0.487	0.512
3418	0.184	-0.625	0.296	0.234	-0.336	0.444	0.261	-0.137	0.512
3419	0.142	-0.895	0.492	0.234	-0.336	0.508	0.261	-0.137	0.513
3420	0.184	-0.625	0.512	0.193	-0.600	0.491	0.261	-0.137	0.512
3421	0.500	1.397	0.467	0.509	1.434	0.273	0.468	1.194	0.492
3422	0.505	1.430	0.513	0.482	1.265	0.422	0.513	1.481	0.467
3423	0.500	1.397	0.501	0.444	1.020	0.508	0.508	1.447	0.492
3424	0.453	1.093	0.501	0.512	1.453	0.514	0.511	1.464	0.467
3425	0.500	1.397	0.501	0.497	1.359	0.491	0.408	0.807	0.492
3426	0.000	?	0.513	0.275	-0.073	0.491	0.263	-0.120	0.185
3427	0.508	1.447	0.512	0.424	0.888	0.491	0.487	1.312	0.224
3428	0.487	1.312	0.512	0.509	1.434	0.491	0.489	1.329	0.185
3429	0.511	1.464	0.512	0.509	1.434	0.512	0.463	1.161	0.185
3430	0.505	1.430	0.512	0.491	1.321	0.478	0.463	1.161	0.296
3431	0.505	1.430	0.512	0.491	1.321	0.512	0.463	1.161	0.513
3432	0.513	1.481	0.142	0.494	1.340	0.273	0.434	0.975	0.513
3433	0.513	1.481	0.224	0.494	1.340	0.514	0.434	0.975	0.513
3434	0.500	1.397	0.262	0.515	1.472	0.514	0.468	1.194	0.358
3435	0.511	1.464	0.097	0.482	1.265	0.193	0.503	1.413	0.501
3436	0.495	1.363	0.501	0.509	1.434	0.491	0.447	1.060	0.262
3437	0.142	-0.895	0.501	0.275	-0.073	0.508	0.226	-0.356	0.358
3438	0.508	1.447	0.501	0.518	1.491	0.508	0.479	1.262	0.296
3439	0.508	1.447	0.097	0.518	1.491	0.491	0.479	1.262	0.185
3440	0.142	-0.895	0.512	0.196	-0.581	0.501	0.226	-0.356	0.513
3441	0.500	1.397	0.097	0.488	1.302	0.309	0.468	1.194	0.512
3442	0.495	1.363	0.097	0.509	1.434	0.501	0.513	1.481	0.512
3443	0.511	1.464	0.097	0.509	1.434	0.501	0.511	1.464	0.512
3444	0.503	1.413	0.513	0.491	1.321	0.512	0.495	1.363	0.328
3445	0.505	1.430	0.513	0.500	1.378	0.478	0.479	1.262	0.328
3446	0.266	-0.103	0.513	0.310	0.153	0.478	0.329	0.301	0.097
3447	0.492	1.346	0.513	0.500	1.378	0.508	0.495	1.363	0.097
3448	0.466	1.178	0.050	0.500	1.378	0.149	0.450	1.076	0.501
3449	0.479	1.262	0.481	0.509	1.434	0.512	0.482	1.279	0.450
3450	0.500	1.397	0.501	0.477	1.227	0.273	0.487	1.312	0.512
3451	0.489	1.329	0.508	0.482	1.265	0.512	0.489	1.329	0.467
3452	0.511	1.464	0.501	0.503	1.396	0.512	0.495	1.363	0.296
3453	0.226	-0.356	0.185	0.149	-0.882	0.501	0.332	0.318	0.296
3454	0.495	1.363	0.328	0.512	1.453	0.508	0.500	1.397	0.508
3455	0.297	0.099	0.492	0.275	-0.073	0.512	0.384	0.655	0.512
3456	0.495	1.363	0.492	0.515	1.472	0.512	0.492	1.346	0.512
3457	0.508	1.447	0.512	0.512	1.453	0.512	0.295	0.082	0.508
3458	0.468	1.194	0.512	0.494	1.340	0.491	0.497	1.380	0.492
3459	0.511	1.464	0.508	0.506	1.415	0.462	0.503	1.413	0.512
3460	0.500	1.397	0.512	0.509	1.434	0.444	0.508	1.447	0.508
3461	0.500	1.397	0.513	0.509	1.434	0.512	0.508	1.447	0.328
3462	0.500	1.397	0.097	0.509	1.434	0.508	0.508	1.447	0.492
3463	0.503	1.413	0.467	0.494	1.340	0.508	0.513	1.481	0.512
3464	0.503	1.413	0.409	0.468	1.170	0.235	0.492	1.346	0.467
3465	0.503	1.413	0.492	0.450	1.057	0.309	0.468	1.194	0.450
3466	0.503	1.413	0.492	0.518	1.491	0.273	0.495	1.363	0.185
3467	0.500	1.397	0.328	0.515	1.472	0.273	0.463	1.161	0.409
3468	0.500	1.397	0.185	0.515	1.472	0.341	0.492	1.346	0.185
3469	0.142	-0.895	0.409	0.234	-0.336	0.444	0.184	-0.625	0.262
3470	0.000	?	0.328	0.310	0.153	0.444	0.224	-0.373	0.262
3471	0.226	-0.356	0.224	0.275	-0.073	0.444	0.184	-0.625	0.385
3472	0.263	-0.120	0.097	0.275	-0.073	0.235	0.184	-0.625	0.501
3473	0.097	-1.181	0.501	0.348	0.398	0.514	0.295	0.082	0.501

3474	0.497	1.380	0.501	0.439	0.982	0.341	0.513	1.481	0.467
3475	0.497	1.380	0.262	0.439	0.982	0.309	0.513	1.481	0.185
3476	0.497	1.380	0.296	0.439	0.982	0.444	0.513	1.481	0.512
3477	0.097	-1.181	0.501	0.237	-0.317	0.444	0.358	0.487	0.512
3478	0.500	1.397	0.501	0.515	1.472	0.478	0.503	1.413	0.185
3479	0.100	-1.164	0.296	0.342	0.361	0.501	0.263	-0.120	0.224
3480	0.000	?	0.185	0.307	0.135	0.491	0.358	0.487	0.467
3481	0.097	-1.181	0.501	0.447	1.039	0.491	0.295	0.082	0.328
3482	0.097	-1.181	0.097	0.447	1.039	0.309	0.184	-0.625	0.185
3483	0.513	1.481	0.185	0.477	1.227	0.193	0.513	1.481	0.492
3484	0.500	1.397	0.501	0.503	1.396	0.398	0.513	1.481	0.481
3485	0.500	1.397	0.501	0.494	1.340	0.398	0.513	1.481	0.492
3486	0.500	1.397	0.481	0.494	1.340	0.508	0.513	1.481	0.508
3487	0.050	-1.485	0.508	0.307	0.135	0.512	0.329	0.301	0.508
3488	0.000	?	0.508	0.196	-0.581	0.478	0.326	0.285	0.185
3489	0.000	?	0.262	0.401	0.737	0.512	0.097	-1.181	0.431
3490	0.000	?	0.385	0.401	0.737	0.273	0.097	-1.181	0.224
3491	0.474	1.228	0.224	0.509	1.434	0.341	0.503	1.413	0.508
3492	0.492	1.346	0.508	0.512	1.453	0.514	0.453	1.093	0.508
3493	0.508	1.447	0.508	0.480	1.246	0.491	0.511	1.464	0.508
3494	0.500	1.397	0.508	0.515	1.472	0.501	0.468	1.194	0.185
3495	0.184	-0.625	0.185	0.275	-0.073	0.514	0.297	0.099	0.513
3496	0.329	0.301	0.512	0.345	0.379	0.512	0.297	0.099	0.512
3497	0.489	1.329	0.512	0.512	1.453	0.491	0.505	1.430	0.508
3498	0.489	1.329	0.508	0.491	1.321	0.491	0.505	1.430	0.467
3499	0.511	1.464	0.431	0.500	1.378	0.102	0.513	1.481	0.467
3500	0.505	1.430	0.467	0.515	1.472	0.444	0.505	1.430	0.185
3501	0.505	1.430	0.358	0.515	1.472	0.444	0.487	1.312	0.501
3502	0.505	1.430	0.431	0.488	1.302	0.491	0.511	1.464	0.501
3503	0.508	1.447	0.431	0.488	1.302	0.478	0.508	1.447	0.296
3504	0.097	-1.181	0.097	0.102	-1.184	0.309	0.326	0.285	0.512
3505	0.474	1.228	0.467	0.447	1.039	0.398	0.489	1.329	0.513
3506	0.411	0.824	0.492	0.444	1.020	0.371	0.505	1.430	0.501
3507	0.495	1.363	0.512	0.491	1.321	0.102	0.463	1.161	0.501
3508	0.492	1.346	0.512	0.477	1.227	0.102	0.453	1.093	0.512
3509	0.326	0.285	0.508	0.307	0.135	0.235	0.187	-0.608	0.492
3510	0.184	-0.625	0.512	0.401	0.737	0.478	0.413	0.841	0.481
3511	0.408	0.807	0.467	0.371	0.549	0.478	0.187	-0.608	0.508
3512	0.329	0.301	0.508	0.102	-1.184	0.273	0.261	-0.137	0.450
3513	0.226	-0.356	0.431	0.102	-1.184	0.193	0.261	-0.137	0.512
3514	0.097	-1.181	0.512	0.234	-0.336	0.478	0.389	0.689	0.296
3515	0.492	1.346	0.224	0.482	1.265	0.478	0.497	1.380	0.262
3516	0.492	1.346	0.262	0.482	1.265	0.235	0.497	1.380	0.097
3517	0.263	-0.120	0.262	0.275	-0.073	0.149	0.468	1.194	0.450
3518	0.295	0.082	0.513	0.193	-0.600	0.512	0.187	-0.608	0.328
3519	0.508	1.447	0.097	0.477	1.227	0.309	0.513	1.481	0.450
3520	0.508	1.447	0.512	0.477	1.227	0.309	0.513	1.481	0.492
3521	0.295	0.082	0.508	0.237	-0.317	0.501	0.187	-0.608	0.492
3522	0.187	-0.608	0.508	0.149	-0.882	0.491	0.224	-0.373	0.328
3523	0.500	1.397	0.501	0.509	1.434	0.462	0.468	1.194	0.513
3524	0.097	-1.181	0.492	0.310	0.153	0.491	0.329	0.301	0.508
3525	0.184	-0.625	0.492	0.307	0.135	0.491	0.184	-0.625	0.512
3526	0.497	1.380	0.185	0.509	1.434	0.398	0.495	1.363	0.262
3527	0.497	1.380	0.467	0.497	1.359	0.478	0.482	1.279	0.512
3528	0.484	1.295	0.467	0.462	1.133	0.341	0.489	1.329	0.512
3529	0.503	1.413	0.467	0.485	1.283	0.501	0.511	1.464	0.512
3530	0.503	1.413	0.512	0.485	1.283	0.501	0.511	1.464	0.513
3531	0.261	-0.137	0.185	0.404	0.756	0.501	0.184	-0.625	0.142
3532	0.382	0.638	0.431	0.477	1.227	0.235	0.426	0.925	0.467
3533	0.224	-0.373	0.431	0.342	0.361	0.512	0.224	-0.373	0.467
3534	0.505	1.430	0.409	0.500	1.378	0.512	0.505	1.430	0.467

3535	0.505	1.430	0.224	0.500	1.378	0.501	0.505	1.430	0.262
3536	0.505	1.430	0.450	0.500	1.378	0.501	0.505	1.430	0.501
3537	0.184	-0.625	0.501	0.234	-0.336	0.491	0.184	-0.625	0.501
3538	0.508	1.447	0.508	0.512	1.453	0.235	0.511	1.464	0.501
3539	0.508	1.447	0.501	0.512	1.453	0.512	0.508	1.447	0.450
3540	0.513	1.481	0.481	0.503	1.396	0.512	0.511	1.464	0.409
3541	0.434	0.975	0.501	0.506	1.415	0.149	0.466	1.178	0.512
3542	0.468	1.194	0.501	0.488	1.302	0.444	0.463	1.161	0.512
3543	0.355	0.470	0.481	0.237	-0.317	0.478	0.187	-0.608	0.492
3544	0.426	0.925	0.512	0.518	1.491	0.491	0.508	1.447	0.262
3545	0.426	0.925	0.512	0.518	1.491	0.491	0.508	1.447	0.431
3546	0.097	-1.181	0.262	0.149	-0.882	0.508	0.300	0.116	0.431
3547	0.471	1.211	0.050	0.444	1.020	0.508	0.513	1.481	0.224
3548	0.492	1.346	0.513	0.480	1.246	0.508	0.508	1.447	0.385
3549	0.513	1.481	0.467	0.491	1.321	0.491	0.497	1.380	0.508
3550	0.513	1.481	0.050	0.491	1.321	0.508	0.497	1.380	0.512
3551	0.505	1.430	0.492	0.509	1.434	0.491	0.511	1.464	0.296
3552	0.511	1.464	0.512	0.512	1.453	0.444	0.487	1.312	0.513
3553	0.463	1.161	0.512	0.506	1.415	0.273	0.479	1.262	0.431
3554	0.508	1.447	0.508	0.488	1.302	0.193	0.500	1.397	0.431
3555	0.434	0.975	0.508	0.512	1.453	0.508	0.447	1.060	0.512
3556	0.511	1.464	0.512	0.488	1.302	0.235	0.505	1.430	0.450
3557	0.226	-0.356	0.142	0.447	1.039	0.514	0.295	0.082	0.481
3558	0.263	-0.120	0.224	0.275	-0.073	0.508	0.261	-0.137	0.224
3559	0.266	-0.103	0.328	0.193	-0.600	0.508	0.097	-1.181	0.185
3560	0.513	1.481	0.224	0.512	1.453	0.371	0.453	1.093	0.224
3561	0.097	-1.181	0.224	0.237	-0.317	0.478	0.329	0.301	0.224
3562	0.503	1.413	0.142	0.512	1.453	0.444	0.450	1.076	0.185
3563	0.497	1.380	0.501	0.506	1.415	0.491	0.495	1.363	0.142
3564	0.497	1.380	0.467	0.506	1.415	0.235	0.495	1.363	0.512
3565	0.000	?	0.385	0.371	0.549	0.478	0.326	0.285	0.512
3566	0.497	1.380	0.097	0.477	1.227	0.501	0.511	1.464	0.296
3567	0.489	1.329	0.492	0.450	1.057	0.508	0.505	1.430	0.508
3568	0.492	1.346	0.328	0.500	1.378	0.512	0.508	1.447	0.512
3569	0.187	-0.608	0.492	0.234	-0.336	0.309	0.263	-0.120	0.501
3570	0.463	1.161	0.512	0.477	1.227	0.491	0.508	1.447	0.328
3571	0.468	1.194	0.050	0.503	1.396	0.491	0.505	1.430	0.481
3572	0.468	1.194	0.450	0.509	1.434	0.501	0.505	1.430	0.409
3573	0.513	1.481	0.501	0.515	1.472	0.235	0.516	1.498	0.262
3574	0.184	-0.625	0.501	0.313	0.172	0.491	0.142	-0.895	0.185
3575	0.432	0.958	0.501	0.488	1.302	0.514	0.466	1.178	0.513
3576	0.432	0.958	0.508	0.488	1.302	0.501	0.466	1.178	0.513
3577	0.411	0.824	0.508	0.497	1.359	0.462	0.466	1.178	0.501
3578	0.224	-0.373	0.185	0.237	-0.317	0.501	0.263	-0.120	0.501
3579	0.453	1.093	0.508	0.491	1.321	0.462	0.500	1.397	0.481
3580	0.497	1.380	0.224	0.512	1.453	0.491	0.503	1.413	0.142
3581	0.503	1.413	0.328	0.503	1.396	0.491	0.503	1.413	0.512
3582	0.500	1.397	0.262	0.465	1.152	0.273	0.450	1.076	0.097
3583	0.479	1.262	0.508	0.503	1.396	0.462	0.411	0.824	0.328
3584	0.495	1.363	0.508	0.465	1.152	0.462	0.508	1.447	0.296
3585	0.503	1.413	0.431	0.494	1.340	0.053	0.508	1.447	0.467
3586	0.482	1.279	0.358	0.497	1.359	0.149	0.489	1.329	0.481
3587	0.000	?	0.224	0.278	-0.054	0.422	0.261	-0.137	0.492
3588	0.513	1.481	0.296	0.468	1.170	0.422	0.426	0.925	0.262
3589	0.513	1.481	0.501	0.468	1.170	0.235	0.426	0.925	0.296
3590	0.266	-0.103	0.508	0.053	-1.504	0.514	0.224	-0.373	0.224
3591	0.050	-1.485	0.224	0.149	-0.882	0.491	0.387	0.672	0.501
3592	0.508	1.447	0.142	0.421	0.869	0.491	0.505	1.430	0.492
3593	0.468	1.194	0.450	0.421	0.869	0.514	0.518	1.514	0.224
3594	0.050	-1.485	0.431	0.234	-0.336	0.444	0.295	0.082	0.097
3595	0.495	1.363	0.512	0.515	1.472	0.478	0.516	1.498	0.501

3596	0.513	1.481	0.492	0.491	1.321	0.273	0.432	0.958	0.512
3597	0.513	1.481	0.492	0.491	1.321	0.235	0.432	0.958	0.492
3598	0.508	1.447	0.508	0.515	1.472	0.341	0.513	1.481	0.512
3599	0.505	1.430	0.262	0.444	1.020	0.149	0.445	1.043	0.512
3600	0.508	1.447	0.185	0.480	1.246	0.149	0.476	1.245	0.481
3601	0.142	-0.895	0.508	0.275	-0.073	0.341	0.224	-0.373	0.296
3602	0.224	-0.373	0.185	0.234	-0.336	0.512	0.184	-0.625	0.296
3603	0.326	0.285	0.409	0.345	0.379	0.491	0.224	-0.373	0.501
3604	0.224	-0.373	0.512	0.149	-0.882	0.273	0.224	-0.373	0.296
3605	0.224	-0.373	0.481	0.149	-0.882	0.273	0.184	-0.625	0.512
3606	0.142	-0.895	0.513	0.339	0.342	0.462	0.142	-0.895	0.508
3607	0.500	1.397	0.513	0.509	1.434	0.193	0.508	1.447	0.508
3608	0.468	1.194	0.262	0.491	1.321	0.462	0.505	1.430	0.501
3609	0.389	0.689	0.512	0.272	-0.091	0.491	0.000	?	0.501
3610	0.100	-1.164	0.481	0.272	-0.091	0.149	0.297	0.099	0.142
3611	0.489	1.329	0.501	0.462	1.133	0.273	0.500	1.397	0.492
3612	0.329	0.301	0.501	0.196	-0.581	0.149	0.000	?	0.492
3613	0.489	1.329	0.467	0.459	1.114	0.309	0.511	1.464	0.508
3614	0.508	1.447	0.508	0.488	1.302	0.398	0.497	1.380	0.492
3615	0.050	-1.485	0.481	0.149	-0.882	0.501	0.326	0.285	0.508
3616	0.445	1.043	0.508	0.278	-0.054	0.501	0.474	1.228	0.262
3617	0.000	?	0.185	0.149	-0.882	0.512	0.405	0.790	0.492
3618	0.000	?	0.508	0.307	0.135	0.444	0.261	-0.137	0.481
3619	0.000	?	0.296	0.395	0.700	0.512	0.184	-0.625	0.492
3620	0.495	1.363	0.481	0.503	1.396	0.235	0.513	1.481	0.050
3621	0.495	1.363	0.508	0.503	1.396	0.478	0.513	1.481	0.508
3622	0.492	1.346	0.492	0.515	1.472	0.273	0.503	1.413	0.142
3623	0.500	1.397	0.142	0.447	1.039	0.235	0.505	1.430	0.508
3624	0.503	1.413	0.185	0.512	1.453	0.273	0.484	1.295	0.328
3625	0.187	-0.608	0.185	0.237	-0.317	0.444	0.142	-0.895	0.492
3626	0.503	1.413	0.501	0.480	1.246	0.491	0.508	1.447	0.142
3627	0.224	-0.373	0.512	0.275	-0.073	0.491	0.097	-1.181	0.185
3628	0.326	0.285	0.481	0.234	-0.336	0.371	0.329	0.301	0.224
3629	0.261	-0.137	0.467	0.272	-0.091	0.149	0.297	0.099	0.431
3630	0.511	1.464	0.481	0.447	1.039	0.422	0.466	1.178	0.481
3631	0.508	1.447	0.508	0.497	1.359	0.508	0.482	1.279	0.508
3632	0.429	0.942	0.185	0.494	1.340	0.462	0.487	1.312	0.481
3633	0.358	0.487	0.513	0.371	0.549	0.149	0.261	-0.137	0.492
3634	0.226	-0.356	0.513	0.149	-0.882	0.309	0.295	0.082	0.508
3635	0.295	0.082	0.512	0.421	0.869	0.512	0.224	-0.373	0.328
3636	0.497	1.380	0.508	0.509	1.434	0.491	0.500	1.397	0.492
3637	0.497	1.380	0.513	0.465	1.152	0.478	0.492	1.346	0.492
3638	0.224	-0.373	0.512	0.149	-0.882	0.512	0.224	-0.373	0.492
3639	0.142	-0.895	0.224	0.310	0.153	0.512	0.097	-1.181	0.492
3640	0.445	1.043	0.262	0.515	1.472	0.422	0.503	1.413	0.481
3641	0.432	0.958	0.409	0.491	1.321	0.398	0.511	1.464	0.481
3642	0.500	1.397	0.501	0.482	1.265	0.422	0.497	1.380	0.142
3643	0.484	1.295	0.508	0.520	1.509	0.422	0.516	1.498	0.185
3644	0.484	1.295	0.296	0.520	1.509	0.422	0.516	1.498	0.467
3645	0.511	1.464	0.431	0.421	0.869	0.491	0.479	1.262	0.508
3646	0.266	-0.103	0.142	0.395	0.700	0.422	0.295	0.082	0.450
3647	0.187	-0.608	0.501	0.421	0.869	0.491	0.295	0.082	0.409
3648	0.511	1.464	0.492	0.418	0.850	0.508	0.500	1.397	0.512
3649	0.187	-0.608	0.097	0.421	0.869	0.508	0.295	0.082	0.097
3650	0.411	0.824	0.501	0.494	1.340	0.149	0.513	1.481	0.431
3651	0.508	1.447	0.385	0.424	0.888	0.512	0.503	1.413	0.481
3652	0.484	1.295	0.050	0.494	1.340	0.462	0.503	1.413	0.224
3653	0.511	1.464	0.296	0.506	1.415	0.501	0.505	1.430	0.508
3654	0.505	1.430	0.508	0.512	1.453	0.501	0.497	1.380	0.501
3655	0.263	-0.120	0.481	0.149	-0.882	0.501	0.142	-0.895	0.224
3656	0.508	1.447	0.050	0.515	1.472	0.273	0.487	1.312	0.224

3657	0.476	1.245	0.050	0.462	1.133	0.501	0.489	1.329	0.431
3658	0.497	1.380	0.328	0.500	1.378	0.491	0.503	1.413	0.508
3659	0.503	1.413	0.481	0.506	1.415	0.508	0.495	1.363	0.185
3660	0.466	1.178	0.185	0.500	1.378	0.341	0.511	1.464	0.185
3661	0.000	?	0.512	0.275	-0.073	0.491	0.261	-0.137	0.050
3662	0.500	1.397	0.492	0.506	1.415	0.193	0.492	1.346	0.501
3663	0.484	1.295	0.492	0.494	1.340	0.508	0.484	1.295	0.142
3664	0.508	1.447	0.224	0.509	1.434	0.514	0.497	1.380	0.450
3665	0.184	-0.625	0.185	0.342	0.361	0.508	0.050	-1.485	0.513
3666	0.503	1.413	0.142	0.491	1.321	0.309	0.508	1.447	0.501
3667	0.297	0.099	0.512	0.193	-0.600	0.341	0.145	-0.878	0.224
3668	0.476	1.245	0.501	0.509	1.434	0.235	0.505	1.430	0.185
3669	0.497	1.380	0.501	0.515	1.472	0.508	0.326	0.285	0.142
3670	0.497	1.380	0.385	0.512	1.453	0.512	0.487	1.312	0.492
3671	0.142	-0.895	0.385	0.313	0.172	0.444	0.142	-0.895	0.481
3672	0.187	-0.608	0.358	0.342	0.361	0.514	0.184	-0.625	0.492
3673	0.187	-0.608	0.481	0.237	-0.317	0.514	0.224	-0.373	0.328
3674	0.503	1.413	0.481	0.509	1.434	0.444	0.434	0.975	0.296
3675	0.516	1.498	0.492	0.509	1.434	0.102	0.479	1.262	0.358
3676	0.484	1.295	0.501	0.439	0.982	0.508	0.505	1.430	0.508
3677	0.474	1.228	0.467	0.512	1.453	0.508	0.476	1.245	0.513
3678	0.482	1.279	0.512	0.518	1.491	0.508	0.489	1.329	0.508
3679	0.503	1.413	0.512	0.442	1.001	0.491	0.508	1.447	0.492
3680	0.184	-0.625	0.508	0.102	-1.184	0.508	0.326	0.285	0.492
3681	0.516	1.498	0.224	0.512	1.453	0.501	0.492	1.346	0.481
3682	0.516	1.498	0.224	0.512	1.453	0.235	0.492	1.346	0.481
3683	0.500	1.397	0.508	0.512	1.453	0.398	0.495	1.363	0.501
3684	0.497	1.380	0.262	0.497	1.359	0.478	0.497	1.380	0.409
3685	0.500	1.397	0.508	0.512	1.453	0.478	0.487	1.312	0.409
3686	0.500	1.397	0.512	0.506	1.415	0.478	0.487	1.312	0.450
3687	0.224	-0.373	0.185	0.237	-0.317	0.341	0.145	-0.878	0.224
3688	0.261	-0.137	0.501	0.398	0.718	0.398	0.184	-0.625	0.492
3689	0.408	0.807	0.142	0.477	1.227	0.309	0.468	1.194	0.508
3690	0.500	1.397	0.467	0.480	1.246	0.501	0.505	1.430	0.328
3691	0.500	1.397	0.492	0.477	1.227	0.491	0.453	1.093	0.467
3692	0.297	0.099	0.508	0.342	0.361	0.235	0.413	0.841	0.050
3693	0.437	0.992	0.513	0.404	0.756	0.501	0.508	1.447	0.501
3694	0.142	-0.895	0.467	0.310	0.153	0.491	0.097	-1.181	0.512
3695	0.503	1.413	0.467	0.503	1.396	0.273	0.426	0.925	0.512
3696	0.495	1.363	0.512	0.491	1.321	0.309	0.476	1.245	0.492
3697	0.097	-1.181	0.501	0.237	-0.317	0.491	0.226	-0.356	0.512
3698	0.492	1.346	0.097	0.503	1.396	0.478	0.508	1.447	0.512
3699	0.382	0.638	0.097	0.491	1.321	0.309	0.497	1.380	0.512
3700	0.050	-1.485	0.512	0.272	-0.091	0.309	0.226	-0.356	0.358
3701	0.295	0.082	0.513	0.313	0.172	0.309	0.226	-0.356	0.508
3702	0.508	1.447	0.492	0.494	1.340	0.508	0.432	0.958	0.358
3703	0.482	1.279	0.508	0.480	1.246	0.273	0.513	1.481	0.358
3704	0.050	-1.485	0.508	0.310	0.153	0.491	0.187	-0.608	0.358
3705	0.050	-1.485	0.508	0.310	0.153	0.508	0.187	-0.608	0.481
3706	0.326	0.285	0.296	0.307	0.135	0.444	0.050	-1.485	0.481
3707	0.479	1.262	0.501	0.506	1.415	0.235	0.497	1.380	0.481
3708	0.184	-0.625	0.481	0.272	-0.091	0.273	0.142	-0.895	0.431
3709	0.508	1.447	0.481	0.488	1.302	0.273	0.447	1.060	0.492
3710	0.492	1.346	0.185	0.506	1.415	0.444	0.511	1.464	0.492
3711	0.484	1.295	0.512	0.442	1.001	0.444	0.503	1.413	0.050
3712	0.224	-0.373	0.492	0.237	-0.317	0.508	0.224	-0.373	0.501
3713	0.184	-0.625	0.142	0.275	-0.073	0.149	0.184	-0.625	0.512
3714	0.142	-0.895	0.097	0.275	-0.073	0.193	0.142	-0.895	0.501
3715	0.508	1.447	0.142	0.447	1.039	0.193	0.489	1.329	0.142
3716	0.497	1.380	0.512	0.442	1.001	0.512	0.476	1.245	0.481
3717	0.503	1.413	0.501	0.509	1.434	0.508	0.495	1.363	0.512

3718	0.384	0.655	0.481	0.149	-0.882	0.514	0.332	0.318	0.358
3719	0.384	0.655	0.512	0.196	-0.581	0.444	0.297	0.099	0.385
3720	0.358	0.487	0.508	0.196	-0.581	0.444	0.361	0.504	0.385
3721	0.479	1.262	0.508	0.512	1.453	0.501	0.511	1.464	0.492
3722	0.476	1.245	0.508	0.500	1.378	0.501	0.516	1.498	0.467
3723	0.495	1.363	0.492	0.515	1.472	0.501	0.511	1.464	0.513
3724	0.503	1.413	0.501	0.444	1.020	0.193	0.492	1.346	0.512
3725	0.471	1.211	0.492	0.447	1.039	0.235	0.489	1.329	0.467
3726	0.503	1.413	0.492	0.497	1.359	0.508	0.484	1.295	0.501
3727	0.503	1.413	0.508	0.497	1.359	0.053	0.484	1.295	0.501
3728	0.503	1.413	0.431	0.497	1.359	0.514	0.500	1.397	0.450
3729	0.226	-0.356	0.467	0.196	-0.581	0.501	0.408	0.807	0.512
3730	0.226	-0.356	0.431	0.240	-0.299	0.102	0.408	0.807	0.512
3731	0.511	1.464	0.431	0.506	1.415	0.508	0.450	1.076	0.431
3732	0.263	-0.120	0.431	0.053	-1.504	0.341	0.224	-0.373	0.513
3733	0.497	1.380	0.328	0.512	1.453	0.444	0.495	1.363	0.508
3734	0.500	1.397	0.508	0.500	1.378	0.512	0.511	1.464	0.513
3735	0.187	-0.608	0.467	0.102	-1.184	0.508	0.329	0.301	0.512
3736	0.503	1.413	0.450	0.503	1.396	0.462	0.466	1.178	0.512
3737	0.145	-0.878	0.513	0.342	0.361	0.508	0.050	-1.485	0.512
3738	0.466	1.178	0.501	0.447	1.039	0.508	0.503	1.413	0.097
3739	0.492	1.346	0.501	0.518	1.491	0.371	0.513	1.481	0.481
3740	0.513	1.481	0.501	0.509	1.434	0.273	0.513	1.481	0.481
3741	0.516	1.498	0.142	0.465	1.152	0.491	0.492	1.346	0.481
3742	0.466	1.178	0.513	0.509	1.434	0.149	0.511	1.464	0.481
3743	0.466	1.178	0.409	0.509	1.434	0.273	0.511	1.464	0.501
3744	0.500	1.397	0.385	0.371	0.549	0.149	0.513	1.481	0.328
3745	0.000	?	0.492	0.275	-0.073	0.508	0.355	0.470	0.450
3746	0.492	1.346	0.224	0.491	1.321	0.462	0.508	1.447	0.450
3747	0.097	-1.181	0.431	0.149	-0.882	0.512	0.358	0.487	0.224
3748	0.000	?	0.328	0.275	-0.073	0.444	0.355	0.470	0.481
3749	0.097	-1.181	0.142	0.149	-0.882	0.444	0.358	0.487	0.501
3750	0.511	1.464	0.501	0.512	1.453	0.508	0.482	1.279	0.492
3751	0.513	1.481	0.513	0.465	1.152	0.193	0.482	1.279	0.481
3752	0.492	1.346	0.296	0.512	1.453	0.444	0.479	1.262	0.224
3753	0.497	1.380	0.328	0.447	1.039	0.512	0.432	0.958	0.513
3754	0.497	1.380	0.501	0.447	1.039	0.512	0.497	1.380	0.185
3755	0.500	1.397	0.224	0.512	1.453	0.235	0.487	1.312	0.296
3756	0.297	0.099	0.513	0.196	-0.581	0.462	0.050	-1.485	0.512
3757	0.503	1.413	0.450	0.439	0.982	0.514	0.495	1.363	0.481
3758	0.474	1.228	0.450	0.512	1.453	0.102	0.513	1.481	0.296
3759	0.482	1.279	0.224	0.512	1.453	0.102	0.500	1.397	0.097
3760	0.184	-0.625	0.262	0.234	-0.336	0.102	0.142	-0.895	0.481
3761	0.513	1.481	0.328	0.465	1.152	0.478	0.479	1.262	0.142
3762	0.495	1.363	0.501	0.515	1.472	0.508	0.513	1.481	0.467
3763	0.142	-0.895	0.492	0.102	-1.184	0.508	0.358	0.487	0.508
3764	0.097	-1.181	0.492	0.102	-1.184	0.508	0.387	0.672	0.467
3765	0.142	-0.895	0.296	0.102	-1.184	0.514	0.387	0.672	0.296
3766	0.503	1.413	0.185	0.480	1.246	0.462	0.500	1.397	0.296
3767	0.505	1.430	0.262	0.512	1.453	0.501	0.466	1.178	0.501
3768	0.482	1.279	0.501	0.509	1.434	0.512	0.513	1.481	0.512
3769	0.508	1.447	0.512	0.506	1.415	0.514	0.508	1.447	0.513
3770	0.503	1.413	0.358	0.512	1.453	0.491	0.463	1.161	0.508
3771	0.503	1.413	0.262	0.468	1.170	0.512	0.505	1.430	0.512
3772	0.505	1.430	0.050	0.497	1.359	0.508	0.500	1.397	0.224
3773	0.492	1.346	0.481	0.506	1.415	0.512	0.450	1.076	0.097
3774	0.497	1.380	0.296	0.515	1.472	0.508	0.511	1.464	0.481
3775	0.489	1.329	0.262	0.491	1.321	0.491	0.511	1.464	0.481
3776	0.495	1.363	0.224	0.515	1.472	0.491	0.429	0.942	0.296
3777	0.508	1.447	0.501	0.503	1.396	0.508	0.516	1.498	0.409
3778	0.429	0.942	0.512	0.506	1.415	0.273	0.508	1.447	0.185

3779	0.466	1.178	0.142	0.503	1.396	0.491	0.513	1.481	0.481
3780	0.426	0.925	0.142	0.485	1.283	0.462	0.513	1.481	0.262
3781	0.426	0.925	0.224	0.485	1.283	0.422	0.513	1.481	0.431
3782	0.426	0.925	0.508	0.503	1.396	0.462	0.513	1.481	0.185
3783	0.332	0.318	0.481	0.275	-0.073	0.512	0.097	-1.181	0.385
3784	0.497	1.380	0.501	0.497	1.359	0.193	0.482	1.279	0.467
3785	0.468	1.194	0.492	0.468	1.170	0.444	0.482	1.279	0.185
3786	0.453	1.093	0.508	0.424	0.888	0.444	0.482	1.279	0.185
3787	0.500	1.397	0.481	0.468	1.170	0.341	0.487	1.312	0.262
3788	0.500	1.397	0.358	0.512	1.453	0.478	0.495	1.363	0.508
3789	0.000	?	0.467	0.196	-0.581	0.514	0.326	0.285	0.501
3790	0.503	1.413	0.508	0.442	1.001	0.462	0.445	1.043	0.513
3791	0.503	1.413	0.492	0.442	1.001	0.398	0.445	1.043	0.501
3792	0.142	-0.895	0.450	0.342	0.361	0.102	0.224	-0.373	0.467
3793	0.518	1.514	0.224	0.477	1.227	0.398	0.479	1.262	0.512
3794	0.411	0.824	0.224	0.515	1.472	0.235	0.497	1.380	0.296
3795	0.387	0.672	0.224	0.459	1.114	0.341	0.487	1.312	0.431
3796	0.495	1.363	0.142	0.404	0.756	0.478	0.476	1.245	0.358
3797	0.224	-0.373	0.492	0.102	-1.184	0.501	0.224	-0.373	0.409
3798	0.432	0.958	0.501	0.398	0.718	0.273	0.516	1.498	0.450
3799	0.329	0.301	0.512	0.234	-0.336	0.341	0.184	-0.625	0.224
3800	0.142	-0.895	0.512	0.339	0.342	0.444	0.295	0.082	0.097
3801	0.497	1.380	0.467	0.482	1.265	0.193	0.505	1.430	0.097
3802	0.505	1.430	0.358	0.500	1.378	0.478	0.479	1.262	0.224
3803	0.300	0.116	0.296	0.275	-0.073	0.508	0.295	0.082	0.513
3804	0.329	0.301	0.431	0.339	0.342	0.491	0.097	-1.181	0.501
3805	0.497	1.380	0.431	0.444	1.020	0.273	0.476	1.245	0.409
3806	0.224	-0.373	0.492	0.193	-0.600	0.309	0.142	-0.895	0.501
3807	0.508	1.447	0.501	0.477	1.227	0.478	0.463	1.161	0.492
3808	0.450	1.076	0.501	0.506	1.415	0.462	0.503	1.413	0.328
3809	0.450	1.076	0.501	0.491	1.321	0.462	0.463	1.161	0.328
3810	0.226	-0.356	0.481	0.272	-0.091	0.501	0.297	0.099	0.185
3811	0.263	-0.120	0.050	0.307	0.135	0.478	0.297	0.099	0.185
3812	0.329	0.301	0.050	0.474	1.208	0.341	0.497	1.380	0.512
3813	0.497	1.380	0.050	0.462	1.133	0.273	0.508	1.447	0.409
3814	0.489	1.329	0.512	0.465	1.152	0.514	0.508	1.447	0.450
3815	0.495	1.363	0.508	0.503	1.396	0.514	0.505	1.430	0.481
3816	0.295	0.082	0.262	0.482	1.265	0.273	0.508	1.447	0.450
3817	0.187	-0.608	0.224	0.342	0.361	0.273	0.224	-0.373	0.385
3818	0.263	-0.120	0.512	0.272	-0.091	0.309	0.097	-1.181	0.385
3819	0.497	1.380	0.492	0.512	1.453	0.508	0.482	1.279	0.385
3820	0.508	1.447	0.185	0.512	1.453	0.371	0.482	1.279	0.481
3821	0.361	0.504	0.262	0.272	-0.091	0.235	0.297	0.099	0.501
3822	0.261	-0.137	0.492	0.275	-0.073	0.273	0.413	0.841	0.142
3823	0.050	-1.485	0.492	0.313	0.172	0.478	0.184	-0.625	0.224
3824	0.484	1.295	0.508	0.512	1.453	0.501	0.476	1.245	0.512
3825	0.295	0.082	0.050	0.374	0.568	0.235	0.261	-0.137	0.492
3826	0.263	-0.120	0.450	0.237	-0.317	0.235	0.429	0.942	0.262
3827	0.224	-0.373	0.467	0.275	-0.073	0.193	0.184	-0.625	0.224
3828	0.503	1.413	0.508	0.480	1.246	0.491	0.384	0.655	0.385
3829	0.513	1.481	0.501	0.497	1.359	0.444	0.463	1.161	0.512
3830	0.142	-0.895	0.409	0.237	-0.317	0.462	0.187	-0.608	0.501
3831	0.142	-0.895	0.409	0.237	-0.317	0.508	0.187	-0.608	0.262
3832	0.224	-0.373	0.224	0.196	-0.581	0.501	0.263	-0.120	0.185
3833	0.508	1.447	0.501	0.491	1.321	0.422	0.508	1.447	0.262
3834	0.482	1.279	0.409	0.444	1.020	0.273	0.503	1.413	0.501
3835	0.497	1.380	0.467	0.465	1.152	0.501	0.516	1.498	0.481
3836	0.489	1.329	0.501	0.509	1.434	0.478	0.495	1.363	0.431
3837	0.503	1.413	0.501	0.503	1.396	0.501	0.466	1.178	0.467
3838	0.476	1.245	0.492	0.421	0.869	0.422	0.511	1.464	0.501
3839	0.358	0.487	0.467	0.275	-0.073	0.193	0.297	0.099	0.097

3840	0.463	1.161	0.142	0.500	1.378	0.309	0.426	0.925	0.262
3841	0.508	1.447	0.142	0.477	1.227	0.235	0.355	0.470	0.431
3842	0.495	1.363	0.296	0.503	1.396	0.273	0.405	0.790	0.224
3843	0.450	1.076	0.296	0.418	0.850	0.514	0.453	1.093	0.512
3844	0.224	-0.373	0.501	0.193	-0.600	0.508	0.224	-0.373	0.501
3845	0.224	-0.373	0.467	0.310	0.153	0.514	0.097	-1.181	0.450
3846	0.226	-0.356	0.467	0.237	-0.317	0.501	0.097	-1.181	0.450
3847	0.142	-0.895	0.481	0.278	-0.054	0.444	0.224	-0.373	0.431
3848	0.492	1.346	0.467	0.512	1.453	0.273	0.511	1.464	0.142
3849	0.500	1.397	0.513	0.506	1.415	0.273	0.500	1.397	0.097
3850	0.511	1.464	0.492	0.512	1.453	0.273	0.408	0.807	0.262
3851	0.508	1.447	0.501	0.500	1.378	0.273	0.503	1.413	0.185
3852	0.466	1.178	0.508	0.444	1.020	0.398	0.489	1.329	0.513
3853	0.358	0.487	0.224	0.278	-0.054	0.508	0.329	0.301	0.481
3854	0.297	0.099	0.467	0.278	-0.054	0.508	0.329	0.301	0.501
3855	0.432	0.958	0.481	0.272	-0.091	0.501	0.184	-0.625	0.450
3856	0.432	0.958	0.467	0.272	-0.091	0.512	0.184	-0.625	0.508
3857	0.492	1.346	0.501	0.395	0.700	0.149	0.508	1.447	0.431
3858	0.500	1.397	0.185	0.506	1.415	0.149	0.408	0.807	0.492
3859	0.500	1.397	0.185	0.506	1.415	0.053	0.450	1.076	0.358
3860	0.500	1.397	0.328	0.500	1.378	0.491	0.482	1.279	0.508
3861	0.482	1.279	0.501	0.509	1.434	0.514	0.445	1.043	0.050
3862	0.050	-1.485	0.513	0.149	-0.882	0.371	0.387	0.672	0.450
3863	0.050	-1.485	0.513	0.149	-0.882	0.102	0.387	0.672	0.467
3864	0.050	-1.485	0.513	0.053	-1.504	0.478	0.387	0.672	0.512
3865	0.508	1.447	0.512	0.494	1.340	0.462	0.482	1.279	0.501
3866	0.511	1.464	0.508	0.506	1.415	0.102	0.503	1.413	0.224
3867	0.261	-0.137	0.431	0.374	0.568	0.398	0.142	-0.895	0.224
3868	0.224	-0.373	0.142	0.102	-1.184	0.508	0.226	-0.356	0.224
3869	0.508	1.447	0.431	0.477	1.227	0.462	0.511	1.464	0.513
3870	0.489	1.329	0.097	0.459	1.114	0.512	0.495	1.363	0.481
3871	0.184	-0.625	0.501	0.102	-1.184	0.398	0.263	-0.120	0.409
3872	0.261	-0.137	0.512	0.401	0.737	0.341	0.226	-0.356	0.409
3873	0.487	1.312	0.262	0.506	1.415	0.309	0.384	0.655	0.513
3874	0.489	1.329	0.450	0.462	1.133	0.512	0.516	1.498	0.513
3875	0.505	1.430	0.481	0.512	1.453	0.501	0.497	1.380	0.481
3876	0.000	?	0.513	0.401	0.737	0.512	0.261	-0.137	0.296
3877	0.050	-1.485	0.508	0.339	0.342	0.501	0.184	-0.625	0.512
3878	0.000	?	0.050	0.313	0.172	0.512	0.261	-0.137	0.262
3879	0.450	1.076	0.050	0.509	1.434	0.235	0.500	1.397	0.501
3880	0.471	1.211	0.142	0.503	1.396	0.491	0.482	1.279	0.481
3881	0.505	1.430	0.513	0.509	1.434	0.444	0.426	0.925	0.296
3882	0.497	1.380	0.512	0.500	1.378	0.273	0.468	1.194	0.501
3883	0.411	0.824	0.467	0.518	1.491	0.512	0.497	1.380	0.409
3884	0.408	0.807	0.501	0.234	-0.336	0.514	0.097	-1.181	0.513
3885	0.224	-0.373	0.501	0.497	1.359	0.491	0.261	-0.137	0.513
3886	0.505	1.430	0.492	0.447	1.039	0.501	0.429	0.942	0.185
3887	0.416	0.857	0.501	0.272	-0.091	0.512	0.224	-0.373	0.224
3888	0.461	1.144	0.492	0.512	1.453	0.309	0.508	1.447	0.431
3889	0.497	1.380	0.512	0.515	1.472	0.309	0.500	1.397	0.508
3890	0.503	1.413	0.501	0.497	1.359	0.235	0.445	1.043	0.512
3891	0.495	1.363	0.467	0.500	1.378	0.235	0.450	1.076	0.450
3892	0.468	1.194	0.262	0.515	1.472	0.462	0.432	0.958	0.512
3893	0.142	-0.895	0.481	0.313	0.172	0.444	0.142	-0.895	0.512
3894	0.145	-0.878	0.481	0.310	0.153	0.478	0.097	-1.181	0.492
3895	0.300	0.116	0.492	0.234	-0.336	0.514	0.263	-0.120	0.385
3896	0.300	0.116	0.481	0.234	-0.336	0.514	0.187	-0.608	0.501
3897	0.503	1.413	0.224	0.465	1.152	0.512	0.513	1.481	0.467
3898	0.466	1.178	0.492	0.447	1.039	0.462	0.482	1.279	0.358
3899	0.466	1.178	0.450	0.474	1.208	0.514	0.497	1.380	0.513
3900	0.479	1.262	0.097	0.515	1.472	0.508	0.447	1.060	0.224

3901	0.468	1.194	0.467	0.512	1.453	0.273	0.508	1.447	0.512
3902	0.513	1.481	0.467	0.515	1.472	0.478	0.437	0.992	0.512
3903	0.495	1.363	0.467	0.465	1.152	0.491	0.495	1.363	0.512
3904	0.495	1.363	0.097	0.515	1.472	0.508	0.355	0.470	0.467
3905	0.505	1.430	0.050	0.506	1.415	0.501	0.511	1.464	0.358
3906	0.226	-0.356	0.097	0.275	-0.073	0.235	0.050	-1.485	0.431
3907	0.463	1.161	0.050	0.474	1.208	0.235	0.450	1.076	0.501
3908	0.479	1.262	0.050	0.485	1.283	0.273	0.466	1.178	0.358
3909	0.471	1.211	0.328	0.509	1.434	0.501	0.516	1.498	0.501
3910	0.503	1.413	0.296	0.500	1.378	0.508	0.500	1.397	0.508
3911	0.184	-0.625	0.224	0.237	-0.317	0.508	0.224	-0.373	0.492
3912	0.184	-0.625	0.262	0.234	-0.336	0.508	0.226	-0.356	0.262
3913	0.329	0.301	0.501	0.272	-0.091	0.491	0.224	-0.373	0.262
3914	0.492	1.346	0.481	0.500	1.378	0.508	0.513	1.481	0.262
3915	0.513	1.481	0.296	0.506	1.415	0.508	0.487	1.312	0.262
3916	0.513	1.481	0.513	0.506	1.415	0.193	0.413	0.841	0.296
3917	0.513	1.481	0.513	0.506	1.415	0.491	0.413	0.841	0.296
3918	0.513	1.481	0.185	0.488	1.302	0.341	0.508	1.447	0.262
3919	0.503	1.413	0.409	0.509	1.434	0.508	0.513	1.481	0.296
3920	0.426	0.925	0.385	0.506	1.415	0.512	0.479	1.262	0.142
3921	0.142	-0.895	0.142	0.193	-0.600	0.193	0.300	0.116	0.328
3922	0.426	0.925	0.450	0.488	1.302	0.478	0.513	1.481	0.512
3923	0.097	-1.181	0.501	0.342	0.361	0.512	0.266	-0.103	0.328
3924	0.497	1.380	0.450	0.506	1.415	0.512	0.500	1.397	0.481
3925	0.511	1.464	0.508	0.512	1.453	0.512	0.476	1.245	0.450
3926	0.263	-0.120	0.492	0.193	-0.600	0.341	0.297	0.099	0.142
3927	0.450	1.076	0.512	0.474	1.208	0.273	0.500	1.397	0.513
3928	0.482	1.279	0.450	0.512	1.453	0.193	0.413	0.841	0.513
3929	0.505	1.430	0.501	0.509	1.434	0.462	0.516	1.498	0.328
3930	0.500	1.397	0.262	0.509	1.434	0.478	0.516	1.498	0.508
3931	0.050	-1.485	0.508	0.342	0.361	0.444	0.184	-0.625	0.512
3932	0.050	-1.485	0.512	0.275	-0.073	0.512	0.226	-0.356	0.508
3933	0.142	-0.895	0.262	0.193	-0.600	0.501	0.434	0.975	0.481
3934	0.508	1.447	0.262	0.462	1.133	0.462	0.513	1.481	0.481
3935	0.511	1.464	0.185	0.480	1.246	0.508	0.508	1.447	0.481
3936	0.468	1.194	0.385	0.444	1.020	0.512	0.450	1.076	0.508
3937	0.492	1.346	0.492	0.512	1.453	0.501	0.513	1.481	0.492
3938	0.492	1.346	0.296	0.503	1.396	0.501	0.516	1.498	0.328
3939	0.489	1.329	0.296	0.459	1.114	0.462	0.489	1.329	0.512
3940	0.497	1.380	0.450	0.509	1.434	0.193	0.384	0.655	0.513
3941	0.492	1.346	0.467	0.506	1.415	0.422	0.500	1.397	0.262
3942	0.513	1.481	0.409	0.506	1.415	0.422	0.466	1.178	0.262
3943	0.505	1.430	0.224	0.497	1.359	0.422	0.358	0.487	0.262
3944	0.468	1.194	0.501	0.462	1.133	0.462	0.513	1.481	0.262
3945	0.261	-0.137	0.296	0.196	-0.581	0.193	0.224	-0.373	0.481
3946	0.474	1.228	0.328	0.421	0.869	0.462	0.511	1.464	0.142
3947	0.474	1.228	0.409	0.421	0.869	0.501	0.511	1.464	0.142
3948	0.487	1.312	0.481	0.421	0.869	0.371	0.511	1.464	0.450
3949	0.482	1.279	0.508	0.459	1.114	0.508	0.463	1.161	0.513
3950	0.226	-0.356	0.512	0.193	-0.600	0.478	0.358	0.487	0.492
3951	0.492	1.346	0.467	0.459	1.114	0.491	0.429	0.942	0.296
3952	0.450	1.076	0.296	0.497	1.359	0.235	0.495	1.363	0.409
3953	0.097	-1.181	0.431	0.374	0.568	0.235	0.358	0.487	0.185
3954	0.468	1.194	0.467	0.509	1.434	0.235	0.503	1.413	0.296
3955	0.463	1.161	0.450	0.474	1.208	0.235	0.508	1.447	0.296
3956	0.466	1.178	0.508	0.482	1.265	0.235	0.492	1.346	0.481
3957	0.097	-1.181	0.512	0.234	-0.336	0.102	0.261	-0.137	0.501
3958	0.050	-1.485	0.513	0.234	-0.336	0.102	0.266	-0.103	0.512
3959	0.097	-1.181	0.512	0.234	-0.336	0.235	0.266	-0.103	0.431
3960	0.050	-1.485	0.512	0.234	-0.336	0.235	0.266	-0.103	0.224
3961	0.050	-1.485	0.142	0.237	-0.317	0.501	0.295	0.082	0.385

3962	0.332	0.318	0.508	0.105	-1.165	0.501	0.295	0.082	0.508
3963	0.297	0.099	0.450	0.102	-1.184	0.273	0.261	-0.137	0.513
3964	0.224	-0.373	0.185	0.237	-0.317	0.491	0.297	0.099	0.508
3965	0.263	-0.120	0.508	0.234	-0.336	0.501	0.142	-0.895	0.508
3966	0.500	1.397	0.481	0.494	1.340	0.235	0.329	0.301	0.513
3967	0.476	1.245	0.512	0.503	1.396	0.501	0.511	1.464	0.481
3968	0.297	0.099	0.513	0.272	-0.091	0.501	0.329	0.301	0.492
3969	0.516	1.498	0.050	0.488	1.302	0.053	0.474	1.228	0.328
3970	0.511	1.464	0.467	0.500	1.378	0.501	0.447	1.060	0.431
3971	0.184	-0.625	0.050	0.237	-0.317	0.491	0.145	-0.878	0.481
3972	0.405	0.790	0.262	0.506	1.415	0.491	0.511	1.464	0.224
3973	0.382	0.638	0.262	0.506	1.415	0.478	0.511	1.464	0.508
3974	0.142	-0.895	0.224	0.053	-1.504	0.508	0.329	0.301	0.467
3975	0.445	1.043	0.262	0.503	1.396	0.508	0.511	1.464	0.431
3976	0.500	1.397	0.224	0.488	1.302	0.309	0.505	1.430	0.512
3977	0.450	1.076	0.481	0.488	1.302	0.371	0.503	1.413	0.328
3978	0.508	1.447	0.501	0.474	1.208	0.102	0.476	1.245	0.481
3979	0.487	1.312	0.481	0.506	1.415	0.398	0.479	1.262	0.328
3980	0.505	1.430	0.512	0.506	1.415	0.501	0.479	1.262	0.328
3981	0.450	1.076	0.050	0.307	0.135	0.235	0.508	1.447	0.262
3982	0.497	1.380	0.050	0.371	0.549	0.193	0.492	1.346	0.431
3983	0.263	-0.120	0.508	0.102	-1.184	0.149	0.329	0.301	0.385
3984	0.503	1.413	0.508	0.404	0.756	0.309	0.508	1.447	0.097
3985	0.508	1.447	0.501	0.503	1.396	0.371	0.508	1.447	0.097
3986	0.263	-0.120	0.512	0.237	-0.317	0.193	0.261	-0.137	0.467
3987	0.263	-0.120	0.481	0.196	-0.581	0.193	0.263	-0.120	0.508
3988	0.187	-0.608	0.467	0.149	-0.882	0.512	0.263	-0.120	0.450
3989	0.384	0.655	0.296	0.310	0.153	0.462	0.263	-0.120	0.501
3990	0.492	1.346	0.492	0.371	0.549	0.508	0.482	1.279	0.185
3991	0.300	0.116	0.501	0.193	-0.600	0.341	0.142	-0.895	0.185
3992	0.300	0.116	0.050	0.193	-0.600	0.514	0.142	-0.895	0.512
3993	0.445	1.043	0.481	0.509	1.434	0.193	0.447	1.060	0.501
3994	0.468	1.194	0.262	0.465	1.152	0.193	0.511	1.464	0.508
3995	0.408	0.807	0.431	0.509	1.434	0.273	0.495	1.363	0.450
3996	0.224	-0.373	0.512	0.342	0.361	0.478	0.297	0.099	0.513
3997	0.500	1.397	0.262	0.515	1.472	0.501	0.408	0.807	0.508
3998	0.295	0.082	0.224	0.193	-0.600	0.371	0.184	-0.625	0.142
3999	0.329	0.301	0.450	0.193	-0.600	0.193	0.300	0.116	0.512
4000	0.408	0.807	0.481	0.272	-0.091	0.102	0.300	0.116	0.492
4001	0.474	1.228	0.385	0.482	1.265	0.309	0.482	1.279	0.224
4002	0.508	1.447	0.097	0.494	1.340	0.512	0.503	1.413	0.512
4003	0.511	1.464	0.385	0.368	0.530	0.501	0.516	1.498	0.296
4004	0.463	1.161	0.501	0.193	-0.600	0.491	0.426	0.925	0.492
4005	0.295	0.082	0.513	0.102	-1.184	0.491	0.224	-0.373	0.512
4006	0.426	0.925	0.501	0.310	0.153	0.444	0.382	0.638	0.097
4007	0.463	1.161	0.512	0.512	1.453	0.512	0.508	1.447	0.185
4008	0.450	1.076	0.512	0.497	1.359	0.508	0.511	1.464	0.467
4009	0.497	1.380	0.512	0.494	1.340	0.371	0.508	1.447	0.481
4010	0.500	1.397	0.492	0.494	1.340	0.491	0.508	1.447	0.512
4011	0.503	1.413	0.467	0.444	1.020	0.462	0.513	1.481	0.358
4012	0.500	1.397	0.501	0.518	1.491	0.341	0.479	1.262	0.512
4013	0.500	1.397	0.512	0.512	1.453	0.501	0.495	1.363	0.508
4014	0.142	-0.895	0.481	0.374	0.568	0.508	0.329	0.301	0.358
4015	0.497	1.380	0.450	0.494	1.340	0.478	0.429	0.942	0.501
4016	0.447	1.060	0.185	0.459	1.114	0.422	0.479	1.262	0.508
4017	0.184	-0.625	0.262	0.345	0.379	0.341	0.226	-0.356	0.512
4018	0.505	1.430	0.185	0.497	1.359	0.508	0.508	1.447	0.512
4019	0.474	1.228	0.508	0.512	1.453	0.193	0.461	1.144	0.501
4020	0.500	1.397	0.481	0.485	1.283	0.371	0.434	0.975	0.513
4021	0.503	1.413	0.481	0.433	0.944	0.422	0.511	1.464	0.492
4022	0.050	-1.485	0.142	0.345	0.379	0.371	0.326	0.285	0.481

4023	0.466	1.178	0.050	0.506	1.415	0.398	0.479	1.262	0.508
4024	0.000	?	0.185	0.193	-0.600	0.235	0.326	0.285	0.467
4025	0.050	-1.485	0.050	0.368	0.530	0.273	0.326	0.285	0.262
4026	0.261	-0.137	0.492	0.427	0.907	0.512	0.261	-0.137	0.142
4027	0.263	-0.120	0.508	0.374	0.568	0.371	0.434	0.975	0.262
4028	0.224	-0.373	0.508	0.401	0.737	0.512	0.387	0.672	0.508
4029	0.263	-0.120	0.142	0.234	-0.336	0.491	0.097	-1.181	0.492
4030	0.224	-0.373	0.224	0.272	-0.091	0.309	0.097	-1.181	0.450
4031	0.474	1.228	0.224	0.512	1.453	0.309	0.466	1.178	0.358
4032	0.492	1.346	0.296	0.371	0.549	0.371	0.505	1.430	0.328
4033	0.482	1.279	0.224	0.518	1.491	0.371	0.447	1.060	0.224
4034	0.508	1.447	0.481	0.488	1.302	0.508	0.500	1.397	0.296
4035	0.050	-1.485	0.050	0.310	0.153	0.508	0.184	-0.625	0.508
4036	0.050	-1.485	0.467	0.310	0.153	0.478	0.184	-0.625	0.512
4037	0.500	1.397	0.450	0.374	0.568	0.501	0.508	1.447	0.512
4038	0.500	1.397	0.492	0.374	0.568	0.193	0.497	1.380	0.328
4039	0.495	1.363	0.262	0.506	1.415	0.501	0.508	1.447	0.097
4040	0.500	1.397	0.512	0.512	1.453	0.491	0.455	1.110	0.142
4041	0.484	1.295	0.501	0.482	1.265	0.371	0.513	1.481	0.409
4042	0.466	1.178	0.097	0.500	1.378	0.508	0.508	1.447	0.224
4043	0.295	0.082	0.262	0.193	-0.600	0.273	0.142	-0.895	0.467
4044	0.484	1.295	0.262	0.512	1.453	0.444	0.513	1.481	0.224
4045	0.492	1.346	0.481	0.500	1.378	0.491	0.492	1.346	0.508
4046	0.050	-1.485	0.481	0.374	0.568	0.273	0.224	-0.373	0.501
4047	0.474	1.228	0.467	0.515	1.472	0.149	0.516	1.498	0.467
4048	0.263	-0.120	0.185	0.272	-0.091	0.501	0.297	0.099	0.328
4049	0.429	0.942	0.385	0.450	1.057	0.514	0.492	1.346	0.512
4050	0.508	1.447	0.467	0.488	1.302	0.491	0.508	1.447	0.492
4051	0.261	-0.137	0.262	0.275	-0.073	0.102	0.100	-1.164	0.409
4052	0.226	-0.356	0.142	0.149	-0.882	0.491	0.187	-0.608	0.224
4053	0.447	1.060	0.512	0.494	1.340	0.501	0.471	1.211	0.142
4054	0.482	1.279	0.492	0.506	1.415	0.478	0.484	1.295	0.501
4055	0.387	0.672	0.467	0.497	1.359	0.462	0.511	1.464	0.467
4056	0.097	-1.181	0.467	0.102	-1.184	0.444	0.358	0.487	0.467
4057	0.387	0.672	0.492	0.488	1.302	0.444	0.513	1.481	0.185
4058	0.500	1.397	0.512	0.500	1.378	0.444	0.511	1.464	0.467
4059	0.511	1.464	0.467	0.474	1.208	0.462	0.358	0.487	0.512
4060	0.497	1.380	0.512	0.456	1.095	0.491	0.503	1.413	0.185
4061	0.508	1.447	0.501	0.442	1.001	0.462	0.505	1.430	0.224
4062	0.508	1.447	0.492	0.442	1.001	0.508	0.511	1.464	0.431
4063	0.508	1.447	0.512	0.442	1.001	0.491	0.511	1.464	0.508
4064	0.487	1.312	0.501	0.462	1.133	0.371	0.500	1.397	0.508
4065	0.471	1.211	0.513	0.494	1.340	0.149	0.516	1.498	0.508
4066	0.500	1.397	0.492	0.468	1.170	0.371	0.495	1.363	0.501
4067	0.511	1.464	0.296	0.509	1.434	0.273	0.479	1.262	0.508
4068	0.474	1.228	0.512	0.491	1.321	0.462	0.508	1.447	0.501
4069	0.450	1.076	0.185	0.371	0.549	0.501	0.474	1.228	0.431
4070	0.187	-0.608	0.185	0.149	-0.882	0.512	0.263	-0.120	0.501
4071	0.261	-0.137	0.508	0.371	0.549	0.102	0.142	-0.895	0.501
4072	0.184	-0.625	0.328	0.275	-0.073	0.398	0.266	-0.103	0.492
4073	0.505	1.430	0.097	0.462	1.133	0.149	0.508	1.447	0.492
4074	0.476	1.245	0.296	0.500	1.378	0.235	0.495	1.363	0.501
4075	0.479	1.262	0.097	0.515	1.472	0.501	0.447	1.060	0.508
4076	0.142	-0.895	0.501	0.102	-1.184	0.512	0.355	0.470	0.328
4077	0.050	-1.485	0.431	0.404	0.756	0.512	0.326	0.285	0.501
4078	0.187	-0.608	0.501	0.149	-0.882	0.102	0.226	-0.356	0.224
4079	0.050	-1.485	0.481	0.237	-0.317	0.273	0.295	0.082	0.224
4080	0.489	1.329	0.142	0.497	1.359	0.235	0.511	1.464	0.501
4081	0.500	1.397	0.185	0.520	1.509	0.193	0.511	1.464	0.296
4082	0.500	1.397	0.224	0.520	1.509	0.273	0.511	1.464	0.296
4083	0.142	-0.895	0.508	0.102	-1.184	0.501	0.326	0.285	0.296

4084	0.224	−0.373	0.508	0.272	−0.091	0.273	0.097	−1.181	0.409
4085	0.226	−0.356	0.224	0.234	−0.336	0.512	0.142	−0.895	0.492
4086	0.297	0.099	0.508	0.193	−0.600	0.514	0.411	0.824	0.501
4087	0.226	−0.356	0.050	0.275	−0.073	0.444	0.226	−0.356	0.358
4088	0.482	1.279	0.296	0.506	1.415	0.193	0.466	1.178	0.481
4089	0.050	−1.485	0.328	0.278	−0.054	0.501	0.224	−0.373	0.328
4090	0.463	1.161	0.262	0.509	1.434	0.462	0.508	1.447	0.262
4091	0.450	1.076	0.431	0.515	1.472	0.273	0.500	1.397	0.262
4092	0.492	1.346	0.450	0.447	1.039	0.273	0.468	1.194	0.481
4093	0.261	−0.137	0.450	0.193	−0.600	0.235	0.329	0.301	0.481
4094	0.511	1.464	0.185	0.500	1.378	0.491	0.505	1.430	0.224
4095	0.497	1.380	0.142	0.465	1.152	0.491	0.484	1.295	0.508
4096	0.097	−1.181	0.467	0.275	−0.073	0.491	0.411	0.824	0.262
4097	0.261	−0.137	0.224	0.275	−0.073	0.273	0.224	−0.373	0.185
4098	0.261	−0.137	0.508	0.237	−0.317	0.501	0.142	−0.895	0.142
4099	0.479	1.262	0.481	0.491	1.321	0.491	0.495	1.363	0.142
4100	0.479	1.262	0.492	0.491	1.321	0.444	0.461	1.144	0.508
4101	0.466	1.178	0.224	0.491	1.321	0.193	0.461	1.144	0.501
4102	0.187	−0.608	0.450	0.275	−0.073	0.508	0.184	−0.625	0.508
4103	0.387	0.672	0.358	0.509	1.434	0.422	0.466	1.178	0.296
4104	0.471	1.211	0.328	0.497	1.359	0.444	0.508	1.447	0.185
4105	0.263	−0.120	0.142	0.447	1.039	0.444	0.184	−0.625	0.513
4106	0.142	−0.895	0.262	0.193	−0.600	0.491	0.224	−0.373	0.224
4107	0.505	1.430	0.328	0.503	1.396	0.398	0.432	0.958	0.431
4108	0.492	1.346	0.501	0.424	0.888	0.491	0.503	1.413	0.501
4109	0.466	1.178	0.512	0.444	1.020	0.371	0.503	1.413	0.431
4110	0.466	1.178	0.513	0.444	1.020	0.501	0.503	1.413	0.224
4111	0.495	1.363	0.142	0.494	1.340	0.508	0.505	1.430	0.512
4112	0.516	1.498	0.481	0.401	0.737	0.444	0.503	1.413	0.512
4113	0.466	1.178	0.513	0.494	1.340	0.491	0.497	1.380	0.385
4114	0.511	1.464	0.508	0.368	0.530	0.422	0.426	0.925	0.328
4115	0.495	1.363	0.185	0.500	1.378	0.478	0.505	1.430	0.262
4116	0.484	1.295	0.492	0.506	1.415	0.102	0.505	1.430	0.328
4117	0.516	1.498	0.501	0.450	1.057	0.491	0.489	1.329	0.431
4118	0.495	1.363	0.492	0.491	1.321	0.149	0.495	1.363	0.501
4119	0.516	1.498	0.450	0.418	0.850	0.149	0.497	1.380	0.492
4120	0.495	1.363	0.142	0.480	1.246	0.514	0.503	1.413	0.328
4121	0.297	0.099	0.467	0.102	−1.184	0.478	0.332	0.318	0.501
4122	0.508	1.447	0.501	0.488	1.302	0.341	0.500	1.397	0.481
4123	0.184	−0.625	0.512	0.149	−0.882	0.422	0.229	−0.339	0.501
4124	0.184	−0.625	0.512	0.149	−0.882	0.193	0.229	−0.339	0.224
4125	0.511	1.464	0.296	0.509	1.434	0.514	0.500	1.397	0.513
4126	0.329	0.301	0.262	0.480	1.246	0.491	0.297	0.099	0.513
4127	0.097	−1.181	0.492	0.342	0.361	0.514	0.295	0.082	0.513
4128	0.295	0.082	0.358	0.427	0.907	0.478	0.295	0.082	0.512
4129	0.097	−1.181	0.513	0.196	−0.581	0.371	0.411	0.824	0.262
4130	0.503	1.413	0.512	0.512	1.453	0.273	0.489	1.329	0.501
4131	0.434	0.975	0.512	0.488	1.302	0.149	0.500	1.397	0.467
4132	0.503	1.413	0.358	0.515	1.472	0.501	0.355	0.470	0.501
4133	0.479	1.262	0.512	0.480	1.246	0.501	0.482	1.279	0.492
4134	0.142	−0.895	0.224	0.374	0.568	0.273	0.329	0.301	0.224
4135	0.184	−0.625	0.185	0.275	−0.073	0.501	0.263	−0.120	0.050
4136	0.229	−0.339	0.185	0.149	−0.882	0.309	0.261	−0.137	0.481
4137	0.505	1.430	0.224	0.500	1.378	0.149	0.476	1.245	0.224
4138	0.505	1.430	0.097	0.500	1.378	0.149	0.476	1.245	0.501
4139	0.224	−0.373	0.513	0.275	−0.073	0.193	0.224	−0.373	0.508
4140	0.505	1.430	0.262	0.500	1.378	0.512	0.505	1.430	0.512
4141	0.050	−1.485	0.262	0.310	0.153	0.514	0.261	−0.137	0.296
4142	0.297	0.099	0.262	0.149	−0.882	0.514	0.187	−0.608	0.501
4143	0.332	0.318	0.224	0.149	−0.882	0.309	0.142	−0.895	0.224
4144	0.263	−0.120	0.492	0.196	−0.581	0.341	0.142	−0.895	0.185

4145	0.426	0.925	0.185	0.512	1.453	0.491	0.505	1.430	0.224
4146	0.447	1.060	0.513	0.512	1.453	0.462	0.497	1.380	0.097
4147	0.447	1.060	0.296	0.512	1.453	0.508	0.505	1.430	0.185
4148	0.187	-0.608	0.409	0.307	0.135	0.444	0.297	0.099	0.450
4149	0.142	-0.895	0.224	0.342	0.361	0.501	0.187	-0.608	0.185
4150	0.463	1.161	0.097	0.491	1.321	0.193	0.511	1.464	0.185
4151	0.224	-0.373	0.508	0.462	1.133	0.422	0.224	-0.373	0.142
4152	0.508	1.447	0.358	0.503	1.396	0.491	0.429	0.942	0.097
4153	0.482	1.279	0.481	0.439	0.982	0.309	0.500	1.397	0.467
4154	0.497	1.380	0.431	0.506	1.415	0.309	0.429	0.942	0.296
4155	0.224	-0.373	0.512	0.196	-0.581	0.371	0.226	-0.356	0.467
4156	0.450	1.076	0.431	0.424	0.888	0.309	0.516	1.498	0.142
4157	0.355	0.470	0.508	0.488	1.302	0.478	0.513	1.481	0.501
4158	0.326	0.285	0.262	0.310	0.153	0.491	0.387	0.672	0.224
4159	0.145	-0.878	0.296	0.310	0.153	0.491	0.329	0.301	0.262
4160	0.266	-0.103	0.508	0.371	0.549	0.102	0.261	-0.137	0.501
4161	0.332	0.318	0.431	0.307	0.135	0.501	0.332	0.318	0.492
4162	0.503	1.413	0.296	0.480	1.246	0.444	0.429	0.942	0.501
4163	0.511	1.464	0.409	0.494	1.340	0.508	0.500	1.397	0.450
4164	0.511	1.464	0.508	0.485	1.283	0.235	0.489	1.329	0.481
4165	0.142	-0.895	0.481	0.102	-1.184	0.514	0.329	0.301	0.450
4166	0.479	1.262	0.328	0.503	1.396	0.514	0.503	1.413	0.492
4167	0.513	1.481	0.142	0.442	1.001	0.514	0.482	1.279	0.142
4168	0.497	1.380	0.385	0.509	1.434	0.508	0.503	1.413	0.097
4169	0.184	-0.625	0.328	0.237	-0.317	0.149	0.226	-0.356	0.467
4170	0.489	1.329	0.385	0.506	1.415	0.501	0.513	1.481	0.409
4171	0.500	1.397	0.358	0.506	1.415	0.514	0.513	1.481	0.050
4172	0.489	1.329	0.358	0.506	1.415	0.444	0.513	1.481	0.513
4173	0.445	1.043	0.385	0.506	1.415	0.422	0.508	1.447	0.512
4174	0.142	-0.895	0.385	0.152	-0.863	0.341	0.263	-0.120	0.431
4175	0.466	1.178	0.142	0.503	1.396	0.235	0.505	1.430	0.142
4176	0.500	1.397	0.142	0.515	1.472	0.491	0.466	1.178	0.142
4177	0.505	1.430	0.508	0.444	1.020	0.149	0.497	1.380	0.262
4178	0.516	1.498	0.501	0.427	0.907	0.462	0.492	1.346	0.512
4179	0.297	0.099	0.185	0.342	0.361	0.508	0.226	-0.356	0.450
4180	0.263	-0.120	0.492	0.234	-0.336	0.444	0.050	-1.485	0.296
4181	0.489	1.329	0.501	0.494	1.340	0.235	0.476	1.245	0.296
4182	0.361	0.504	0.467	0.152	-0.863	0.508	0.224	-0.373	0.467
4183	0.505	1.430	0.501	0.462	1.133	0.149	0.500	1.397	0.467
4184	0.503	1.413	0.508	0.509	1.434	0.273	0.508	1.447	0.296
4185	0.516	1.498	0.481	0.442	1.001	0.273	0.505	1.430	0.097
4186	0.363	0.520	0.431	0.237	-0.317	0.273	0.295	0.082	0.508
4187	0.505	1.430	0.431	0.509	1.434	0.273	0.497	1.380	0.492
4188	0.224	-0.373	0.431	0.149	-0.882	0.444	0.226	-0.356	0.328
4189	0.184	-0.625	0.142	0.272	-0.091	0.273	0.187	-0.608	0.481
4190	0.184	-0.625	0.501	0.272	-0.091	0.273	0.226	-0.356	0.512
4191	0.224	-0.373	0.492	0.272	-0.091	0.235	0.097	-1.181	0.481
4192	0.097	-1.181	0.492	0.275	-0.073	0.371	0.184	-0.625	0.513
4193	0.516	1.498	0.097	0.444	1.020	0.444	0.447	1.060	0.513
4194	0.263	-0.120	0.097	0.275	-0.073	0.053	0.184	-0.625	0.431
4195	0.263	-0.120	0.050	0.275	-0.073	0.422	0.184	-0.625	0.431
4196	0.261	-0.137	0.097	0.234	-0.336	0.102	0.142	-0.895	0.431
4197	0.224	-0.373	0.512	0.377	0.587	0.508	0.097	-1.181	0.431
4198	0.492	1.346	0.481	0.442	1.001	0.149	0.463	1.161	0.185
4199	0.184	-0.625	0.262	0.053	-1.504	0.309	0.297	0.099	0.508
4200	0.516	1.498	0.481	0.421	0.869	0.371	0.463	1.161	0.508
4201	0.297	0.099	0.508	0.102	-1.184	0.512	0.142	-0.895	0.501
4202	0.413	0.841	0.512	0.503	1.396	0.491	0.495	1.363	0.296
4203	0.226	-0.356	0.224	0.149	-0.882	0.512	0.226	-0.356	0.262
4204	0.097	-1.181	0.224	0.307	0.135	0.371	0.261	-0.137	0.185
4205	0.508	1.447	0.097	0.368	0.530	0.512	0.497	1.380	0.513

4206	0.355	0.470	0.185	0.515	1.472	0.422	0.492	1.346	0.328
4207	0.482	1.279	0.142	0.488	1.302	0.149	0.495	1.363	0.328
4208	0.426	0.925	0.481	0.512	1.453	0.398	0.447	1.060	0.431
4209	0.511	1.464	0.481	0.371	0.549	0.398	0.476	1.245	0.501
4210	0.426	0.925	0.142	0.515	1.472	0.512	0.447	1.060	0.501
4211	0.508	1.447	0.501	0.418	0.850	0.193	0.487	1.312	0.262
4212	0.263	-0.120	0.508	0.149	-0.882	0.422	0.145	-0.878	0.296
4213	0.295	0.082	0.492	0.398	0.718	0.508	0.097	-1.181	0.142
4214	0.508	1.447	0.501	0.395	0.700	0.491	0.463	1.161	0.296
4215	0.432	0.958	0.492	0.509	1.434	0.273	0.408	0.807	0.385
4216	0.295	0.082	0.296	0.193	-0.600	0.341	0.050	-1.485	0.492
4217	0.411	0.824	0.501	0.424	0.888	0.341	0.516	1.498	0.508
4218	0.508	1.447	0.512	0.512	1.453	0.501	0.508	1.447	0.142
4219	0.479	1.262	0.467	0.488	1.302	0.512	0.432	0.958	0.467
4220	0.326	0.285	0.508	0.272	-0.091	0.398	0.142	-0.895	0.508
4221	0.142	-0.895	0.513	0.339	0.342	0.193	0.142	-0.895	0.512
4222	0.389	0.689	0.185	0.342	0.361	0.501	0.261	-0.137	0.508
4223	0.332	0.318	0.467	0.500	1.378	0.501	0.513	1.481	0.501
4224	0.387	0.672	0.224	0.515	1.472	0.235	0.450	1.076	0.358
4225	0.361	0.504	0.492	0.401	0.737	0.309	0.297	0.099	0.492
4226	0.358	0.487	0.512	0.193	-0.600	0.491	0.297	0.099	0.492
4227	0.384	0.655	0.512	0.503	1.396	0.462	0.463	1.161	0.450
4228	0.384	0.655	0.512	0.503	1.396	0.149	0.463	1.161	0.508
4229	0.142	-0.895	0.501	0.240	-0.299	0.444	0.295	0.082	0.512
4230	0.142	-0.895	0.508	0.310	0.153	0.508	0.097	-1.181	0.328
4231	0.508	1.447	0.492	0.488	1.302	0.512	0.508	1.447	0.328
4232	0.503	1.413	0.512	0.471	1.189	0.508	0.487	1.312	0.262
4233	0.184	-0.625	0.142	0.149	-0.882	0.508	0.332	0.318	0.481
4234	0.492	1.346	0.508	0.442	1.001	0.508	0.479	1.262	0.501
4235	0.500	1.397	0.262	0.506	1.415	0.514	0.508	1.447	0.492
4236	0.466	1.178	0.508	0.515	1.472	0.514	0.482	1.279	0.492
4237	0.503	1.413	0.450	0.503	1.396	0.514	0.511	1.464	0.512
4238	0.508	1.447	0.097	0.503	1.396	0.398	0.511	1.464	0.512
4239	0.474	1.228	0.097	0.506	1.415	0.491	0.429	0.942	0.431
4240	0.426	0.925	0.508	0.515	1.472	0.508	0.429	0.942	0.467
4241	0.426	0.925	0.508	0.515	1.472	0.491	0.429	0.942	0.328
4242	0.426	0.925	0.262	0.515	1.472	0.309	0.429	0.942	0.142
4243	0.142	-0.895	0.262	0.401	0.737	0.273	0.187	-0.608	0.481
4244	0.497	1.380	0.508	0.491	1.321	0.341	0.505	1.430	0.262
4245	0.487	1.312	0.142	0.509	1.434	0.398	0.505	1.430	0.492
4246	0.489	1.329	0.097	0.494	1.340	0.478	0.500	1.397	0.450
4247	0.097	-1.181	0.224	0.310	0.153	0.501	0.297	0.099	0.142
4248	0.097	-1.181	0.467	0.272	-0.091	0.398	0.263	-0.120	0.328
4249	0.050	-1.485	0.467	0.342	0.361	0.462	0.184	-0.625	0.358
4250	0.097	-1.181	0.450	0.398	0.718	0.508	0.000	?	0.450
4251	0.503	1.413	0.492	0.480	1.246	0.508	0.511	1.464	0.450
4252	0.479	1.262	0.431	0.506	1.415	0.235	0.329	0.301	0.328
4253	0.261	-0.137	0.467	0.404	0.756	0.193	0.329	0.301	0.358
4254	0.482	1.279	0.296	0.468	1.170	0.341	0.432	0.958	0.512
4255	0.505	1.430	0.296	0.503	1.396	0.193	0.500	1.397	0.185
4256	0.508	1.447	0.385	0.512	1.453	0.309	0.505	1.430	0.328
4257	0.226	-0.356	0.296	0.234	-0.336	0.501	0.263	-0.120	0.224
4258	0.226	-0.356	0.508	0.193	-0.600	0.462	0.297	0.099	0.467
4259	0.097	-1.181	0.501	0.342	0.361	0.309	0.145	-0.878	0.431
4260	0.187	-0.608	0.501	0.193	-0.600	0.512	0.297	0.099	0.450
4261	0.142	-0.895	0.501	0.307	0.135	0.512	0.389	0.689	0.467
4262	0.476	1.245	0.501	0.500	1.378	0.512	0.489	1.329	0.467
4263	0.479	1.262	0.501	0.468	1.170	0.512	0.511	1.464	0.450
4264	0.142	-0.895	0.467	0.313	0.172	0.462	0.142	-0.895	0.097
4265	0.497	1.380	0.185	0.509	1.434	0.273	0.466	1.178	0.097
4266	0.503	1.413	0.481	0.515	1.472	0.501	0.505	1.430	0.097

4267	0.489	1.329	0.142	0.512	1.453	0.508	0.508	1.447	0.050
4268	0.497	1.380	0.492	0.512	1.453	0.462	0.505	1.430	0.501
4269	0.489	1.329	0.501	0.462	1.133	0.501	0.495	1.363	0.501
4270	0.295	0.082	0.492	0.278	−0.054	0.501	0.355	0.470	0.501
4271	0.497	1.380	0.224	0.491	1.321	0.341	0.497	1.380	0.501
4272	0.508	1.447	0.467	0.503	1.396	0.508	0.492	1.346	0.501
4273	0.463	1.161	0.050	0.459	1.114	0.462	0.453	1.093	0.450
4274	0.500	1.397	0.050	0.500	1.378	0.444	0.508	1.447	0.481
4275	0.513	1.481	0.142	0.506	1.415	0.514	0.511	1.464	0.262
4276	0.187	−0.608	0.050	0.339	0.342	0.508	0.329	0.301	0.508
4277	0.466	1.178	0.492	0.512	1.453	0.501	0.332	0.318	0.450
4278	0.224	−0.373	0.142	0.465	1.152	0.508	0.263	−0.120	0.512
4279	0.489	1.329	0.501	0.447	1.039	0.478	0.487	1.312	0.492
4280	0.505	1.430	0.224	0.512	1.453	0.478	0.503	1.413	0.481
4281	0.500	1.397	0.501	0.512	1.453	0.501	0.495	1.363	0.142
4282	0.503	1.413	0.050	0.500	1.378	0.235	0.492	1.346	0.513
4283	0.497	1.380	0.262	0.509	1.434	0.341	0.505	1.430	0.385
4284	0.505	1.430	0.262	0.474	1.208	0.462	0.516	1.498	0.385
4285	0.497	1.380	0.501	0.474	1.208	0.193	0.434	0.975	0.358
4286	0.516	1.498	0.501	0.497	1.359	0.501	0.471	1.211	0.296
4287	0.000	?	0.431	0.237	−0.317	0.478	0.329	0.301	0.431
4288	0.142	−0.895	0.513	0.339	0.342	0.341	0.142	−0.895	0.262
4289	0.511	1.464	0.097	0.459	1.114	0.371	0.482	1.279	0.492
4290	0.263	−0.120	0.508	0.196	−0.581	0.273	0.263	−0.120	0.185
4291	0.508	1.447	0.492	0.500	1.378	0.478	0.489	1.329	0.467
4292	0.445	1.043	0.224	0.480	1.246	0.478	0.455	1.110	0.185
4293	0.097	−1.181	0.185	0.345	0.379	0.149	0.142	−0.895	0.050
4294	0.000	?	0.185	0.368	0.530	0.273	0.332	0.318	0.050
4295	0.097	−1.181	0.501	0.275	−0.073	0.398	0.358	0.487	0.467
4296	0.508	1.447	0.513	0.480	1.246	0.341	0.445	1.043	0.492
4297	0.508	1.447	0.512	0.480	1.246	0.193	0.445	1.043	0.492
4298	0.263	−0.120	0.508	0.149	−0.882	0.102	0.332	0.318	0.512
4299	0.261	−0.137	0.492	0.272	−0.091	0.508	0.361	0.504	0.409
4300	0.500	1.397	0.409	0.395	0.700	0.512	0.516	1.498	0.512
4301	0.142	−0.895	0.185	0.345	0.379	0.512	0.184	−0.625	0.508
4302	0.097	−1.181	0.262	0.196	−0.581	0.491	0.329	0.301	0.097
4303	0.226	−0.356	0.481	0.102	−1.184	0.491	0.224	−0.373	0.142
4304	0.474	1.228	0.467	0.506	1.415	0.501	0.461	1.144	0.262
4305	0.474	1.228	0.467	0.509	1.434	0.273	0.426	0.925	0.512
4306	0.455	1.110	0.501	0.509	1.434	0.273	0.445	1.043	0.492
4307	0.489	1.329	0.501	0.494	1.340	0.102	0.471	1.211	0.501
4308	0.426	0.925	0.508	0.494	1.340	0.273	0.471	1.211	0.501
4309	0.466	1.178	0.492	0.500	1.378	0.501	0.450	1.076	0.508
4310	0.297	0.099	0.050	0.275	−0.073	0.501	0.097	−1.181	0.508
4311	0.297	0.099	0.492	0.275	−0.073	0.501	0.097	−1.181	0.262
4312	0.387	0.672	0.512	0.102	−1.184	0.501	0.097	−1.181	0.224
4313	0.297	0.099	0.512	0.272	−0.091	0.501	0.050	−1.485	0.481
4314	0.497	1.380	0.512	0.506	1.415	0.501	0.500	1.397	0.467
4315	0.492	1.346	0.513	0.506	1.415	0.462	0.500	1.397	0.513
4316	0.492	1.346	0.050	0.506	1.415	0.235	0.500	1.397	0.450
4317	0.492	1.346	0.481	0.506	1.415	0.501	0.500	1.397	0.492
4318	0.492	1.346	0.224	0.506	1.415	0.193	0.500	1.397	0.481
4319	0.495	1.363	0.512	0.503	1.396	0.508	0.445	1.043	0.512
4320	0.466	1.178	0.050	0.462	1.133	0.444	0.476	1.245	0.358
4321	0.184	−0.625	0.501	0.234	−0.336	0.501	0.261	−0.137	0.492
4322	0.479	1.262	0.492	0.506	1.415	0.193	0.503	1.413	0.431
4323	0.142	−0.895	0.224	0.193	−0.600	0.491	0.453	1.093	0.492
4324	0.487	1.312	0.142	0.512	1.453	0.309	0.508	1.447	0.450
4325	0.497	1.380	0.185	0.444	1.020	0.309	0.489	1.329	0.512
4326	0.484	1.295	0.467	0.500	1.378	0.235	0.482	1.279	0.185
4327	0.224	−0.373	0.501	0.193	−0.600	0.341	0.142	−0.895	0.224

4328	0.466	1.178	0.224	0.491	1.321	0.508	0.505	1.430	0.050
4329	0.050	-1.485	0.501	0.307	0.135	0.193	0.384	0.655	0.467
4330	0.050	-1.485	0.512	0.307	0.135	0.491	0.384	0.655	0.185
4331	0.142	-0.895	0.492	0.234	-0.336	0.273	0.358	0.487	0.492
4332	0.050	-1.485	0.501	0.342	0.361	0.501	0.297	0.099	0.224
4333	0.487	1.312	0.513	0.515	1.472	0.398	0.434	0.975	0.501
4334	0.145	-0.878	0.185	0.196	-0.581	0.273	0.261	-0.137	0.492
4335	0.495	1.363	0.224	0.491	1.321	0.309	0.495	1.363	0.097
4336	0.226	-0.356	0.224	0.275	-0.073	0.462	0.184	-0.625	0.097
4337	0.495	1.363	0.409	0.500	1.378	0.508	0.466	1.178	0.142
4338	0.050	-1.485	0.450	0.398	0.718	0.478	0.187	-0.608	0.512
4339	0.263	-0.120	0.481	0.272	-0.091	0.508	0.050	-1.485	0.467
4340	0.263	-0.120	0.467	0.307	0.135	0.371	0.050	-1.485	0.328
4341	0.500	1.397	0.512	0.459	1.114	0.371	0.463	1.161	0.508
4342	0.500	1.397	0.224	0.506	1.415	0.462	0.487	1.312	0.508
4343	0.434	0.975	0.501	0.480	1.246	0.235	0.492	1.346	0.501
4344	0.503	1.413	0.185	0.512	1.453	0.309	0.511	1.464	0.508
4345	0.097	-1.181	0.431	0.371	0.549	0.102	0.411	0.824	0.513
4346	0.508	1.447	0.296	0.374	0.568	0.508	0.516	1.498	0.296
4347	0.492	1.346	0.185	0.468	1.170	0.462	0.503	1.413	0.358
4348	0.226	-0.356	0.409	0.234	-0.336	0.491	0.097	-1.181	0.358
4349	0.184	-0.625	0.501	0.307	0.135	0.514	0.142	-0.895	0.492
4350	0.184	-0.625	0.142	0.102	-1.184	0.508	0.261	-0.137	0.492
4351	0.500	1.397	0.409	0.509	1.434	0.501	0.505	1.430	0.508
4352	0.508	1.447	0.142	0.465	1.152	0.102	0.487	1.312	0.492
4353	0.511	1.464	0.508	0.494	1.340	0.235	0.492	1.346	0.513
4354	0.508	1.447	0.492	0.518	1.491	0.462	0.492	1.346	0.185
4355	0.489	1.329	0.501	0.509	1.434	0.512	0.500	1.397	0.481
4356	0.405	0.790	0.142	0.500	1.378	0.371	0.505	1.430	0.262
4357	0.184	-0.625	0.296	0.102	-1.184	0.514	0.261	-0.137	0.513
4358	0.261	-0.137	0.185	0.240	-0.299	0.491	0.224	-0.373	0.142
4359	0.484	1.295	0.224	0.462	1.133	0.508	0.479	1.262	0.328
4360	0.466	1.178	0.224	0.509	1.434	0.512	0.461	1.144	0.512
4361	0.461	1.144	0.185	0.371	0.549	0.273	0.511	1.464	0.512
4362	0.500	1.397	0.501	0.515	1.472	0.478	0.447	1.060	0.296
4363	0.500	1.397	0.328	0.491	1.321	0.508	0.495	1.363	0.508
4364	0.508	1.447	0.224	0.509	1.434	0.422	0.479	1.262	0.185
4365	0.489	1.329	0.224	0.512	1.453	0.514	0.511	1.464	0.431
4366	0.050	-1.485	0.512	0.275	-0.073	0.501	0.358	0.487	0.450
4367	0.489	1.329	0.512	0.482	1.265	0.371	0.487	1.312	0.501
4368	0.500	1.397	0.142	0.512	1.453	0.371	0.429	0.942	0.262
4369	0.503	1.413	0.385	0.427	0.907	0.444	0.487	1.312	0.097
4370	0.500	1.397	0.358	0.518	1.491	0.501	0.447	1.060	0.262
4371	0.500	1.397	0.328	0.503	1.396	0.149	0.516	1.498	0.142
4372	0.000	?	0.142	0.371	0.549	0.444	0.184	-0.625	0.328
4373	0.000	?	0.467	0.371	0.549	0.398	0.226	-0.356	0.328
4374	0.050	-1.485	0.296	0.444	1.020	0.444	0.050	-1.485	0.467
4375	0.482	1.279	0.097	0.497	1.359	0.422	0.461	1.144	0.185
4376	0.226	-0.356	0.185	0.149	-0.882	0.235	0.184	-0.625	0.328
4377	0.503	1.413	0.501	0.447	1.039	0.309	0.495	1.363	0.224
4378	0.050	-1.485	0.508	0.401	0.737	0.235	0.224	-0.373	0.431
4379	0.500	1.397	0.450	0.442	1.001	0.512	0.500	1.397	0.358
4380	0.487	1.312	0.513	0.421	0.869	0.478	0.489	1.329	0.224
4381	0.226	-0.356	0.142	0.234	-0.336	0.193	0.097	-1.181	0.501
4382	0.142	-0.895	0.501	0.310	0.153	0.508	0.097	-1.181	0.385
4383	0.184	-0.625	0.409	0.234	-0.336	0.491	0.142	-0.895	0.224
4384	0.461	1.144	0.385	0.518	1.491	0.422	0.511	1.464	0.262
4385	0.500	1.397	0.296	0.474	1.208	0.478	0.466	1.178	0.501
4386	0.226	-0.356	0.431	0.193	-0.600	0.508	0.329	0.301	0.262
4387	0.500	1.397	0.481	0.509	1.434	0.273	0.503	1.413	0.431
4388	0.508	1.447	0.481	0.488	1.302	0.149	0.505	1.430	0.358

4389	0.495	1.363	0.409	0.424	0.888	0.149	0.503	1.413	0.501
4390	0.497	1.380	0.185	0.474	1.208	0.501	0.508	1.447	0.481
4391	0.508	1.447	0.224	0.509	1.434	0.512	0.513	1.481	0.450
4392	0.187	-0.608	0.513	0.272	-0.091	0.422	0.295	0.082	0.431
4393	0.224	-0.373	0.409	0.149	-0.882	0.444	0.361	0.504	0.262
4394	0.224	-0.373	0.328	0.149	-0.882	0.422	0.361	0.504	0.492
4395	0.411	0.824	0.328	0.500	1.378	0.235	0.492	1.346	0.492
4396	0.453	1.093	0.501	0.512	1.453	0.508	0.492	1.346	0.501
4397	0.479	1.262	0.262	0.424	0.888	0.235	0.505	1.430	0.224
4398	0.466	1.178	0.328	0.447	1.039	0.444	0.492	1.346	0.508
4399	0.513	1.481	0.262	0.427	0.907	0.273	0.516	1.498	0.501
4400	0.224	-0.373	0.262	0.237	-0.317	0.193	0.187	-0.608	0.501
4401	0.500	1.397	0.262	0.509	1.434	0.422	0.482	1.279	0.358
4402	0.187	-0.608	0.409	0.237	-0.317	0.478	0.261	-0.137	0.262
4403	0.432	0.958	0.385	0.450	1.057	0.309	0.513	1.481	0.185
4404	0.300	0.116	0.142	0.275	-0.073	0.501	0.142	-0.895	0.481
4405	0.184	-0.625	0.512	0.193	-0.600	0.235	0.329	0.301	0.358
4406	0.405	0.790	0.467	0.421	0.869	0.508	0.513	1.481	0.142
4407	0.505	1.430	0.450	0.477	1.227	0.514	0.513	1.481	0.050
4408	0.142	-0.895	0.450	0.310	0.153	0.508	0.300	0.116	0.492
4409	0.405	0.790	0.328	0.500	1.378	0.193	0.503	1.413	0.050
4410	0.142	-0.895	0.450	0.234	-0.336	0.149	0.184	-0.625	0.097
4411	0.503	1.413	0.481	0.506	1.415	0.398	0.429	0.942	0.097
4412	0.492	1.346	0.481	0.509	1.434	0.341	0.447	1.060	0.097
4413	0.497	1.380	0.224	0.509	1.434	0.193	0.495	1.363	0.185
4414	0.145	-0.878	0.224	0.193	-0.600	0.508	0.261	-0.137	0.492
4415	0.300	0.116	0.142	0.149	-0.882	0.193	0.097	-1.181	0.501
4416	0.184	-0.625	0.185	0.395	0.700	0.273	0.263	-0.120	0.142
4417	0.224	-0.373	0.508	0.339	0.342	0.341	0.142	-0.895	0.481
4418	0.224	-0.373	0.296	0.000	?	0.478	0.329	0.301	0.513
4419	0.184	-0.625	0.262	0.196	-0.581	0.478	0.329	0.301	0.508
4420	0.495	1.363	0.296	0.512	1.453	0.309	0.466	1.178	0.508
4421	0.332	0.318	0.296	0.193	-0.600	0.444	0.187	-0.608	0.097
4422	0.224	-0.373	0.296	0.272	-0.091	0.514	0.329	0.301	0.492
4423	0.224	-0.373	0.262	0.342	0.361	0.444	0.224	-0.373	0.467
4424	0.513	1.481	0.450	0.480	1.246	0.309	0.429	0.942	0.467
4425	0.511	1.464	0.467	0.480	1.246	0.508	0.358	0.487	0.185
4426	0.145	-0.878	0.467	0.310	0.153	0.398	0.224	-0.373	0.185
4427	0.384	0.655	0.224	0.442	1.001	0.235	0.503	1.413	0.097
4428	0.358	0.487	0.224	0.515	1.472	0.235	0.382	0.638	0.450
4429	0.329	0.301	0.492	0.444	1.020	0.508	0.226	-0.356	0.097
4430	0.142	-0.895	0.409	0.307	0.135	0.444	0.263	-0.120	0.262
4431	0.463	1.161	0.262	0.503	1.396	0.491	0.500	1.397	0.050
4432	0.297	0.099	0.467	0.398	0.718	0.512	0.261	-0.137	0.050
4433	0.097	-1.181	0.262	0.237	-0.317	0.341	0.429	0.942	0.050
4434	0.184	-0.625	0.142	0.234	-0.336	0.462	0.358	0.487	0.050
4435	0.500	1.397	0.185	0.512	1.453	0.508	0.500	1.397	0.508
4436	0.503	1.413	0.262	0.439	0.982	0.501	0.482	1.279	0.501
4437	0.450	1.076	0.262	0.491	1.321	0.102	0.447	1.060	0.501
4438	0.511	1.464	0.262	0.512	1.453	0.508	0.429	0.942	0.142
4439	0.142	-0.895	0.385	0.345	0.379	0.508	0.261	-0.137	0.142
4440	0.508	1.447	0.142	0.462	1.133	0.491	0.489	1.329	0.501
4441	0.408	0.807	0.492	0.509	1.434	0.149	0.489	1.329	0.512
4442	0.384	0.655	0.492	0.500	1.378	0.309	0.497	1.380	0.142
4443	0.295	0.082	0.185	0.102	-1.184	0.149	0.224	-0.373	0.492
4444	0.432	0.958	0.501	0.518	1.491	0.462	0.505	1.430	0.142
4445	0.482	1.279	0.224	0.509	1.434	0.235	0.497	1.380	0.185
4446	0.479	1.262	0.481	0.491	1.321	0.398	0.505	1.430	0.296
4447	0.411	0.824	0.508	0.149	-0.882	0.149	0.355	0.470	0.050
4448	0.184	-0.625	0.501	0.313	0.172	0.478	0.261	-0.137	0.050
4449	0.224	-0.373	0.224	0.149	-0.882	0.273	0.184	-0.625	0.050

4450	0.513	1.481	0.492	0.462	1.133	0.273	0.487	1.312	0.097
4451	0.411	0.824	0.492	0.240	−0.299	0.309	0.358	0.487	0.142
4452	0.326	0.285	0.481	0.404	0.756	0.193	0.142	−0.895	0.481
4453	0.329	0.301	0.481	0.149	−0.882	0.193	0.050	−1.485	0.481
4454	0.503	1.413	0.450	0.477	1.227	0.514	0.487	1.312	0.262
4455	0.263	−0.120	0.224	0.275	−0.073	0.508	0.050	−1.485	0.481
4456	0.329	0.301	0.450	0.275	−0.073	0.273	0.097	−1.181	0.142
4457	0.263	−0.120	0.296	0.310	0.153	0.501	0.097	−1.181	0.513
4458	0.263	−0.120	0.501	0.196	−0.581	0.512	0.097	−1.181	0.508
4459	0.266	−0.103	0.097	0.193	−0.600	0.512	0.184	−0.625	0.481
4460	0.405	0.790	0.358	0.515	1.472	0.512	0.484	1.295	0.185
4461	0.382	0.638	0.481	0.509	1.434	0.235	0.492	1.346	0.481
4462	0.145	−0.878	0.358	0.272	−0.091	0.491	0.142	−0.895	0.508
4463	0.513	1.481	0.501	0.500	1.378	0.512	0.479	1.262	0.501
4464	0.463	1.161	0.328	0.512	1.453	0.512	0.511	1.464	0.501
4465	0.447	1.060	0.262	0.509	1.434	0.273	0.505	1.430	0.467
4466	0.447	1.060	0.296	0.509	1.434	0.309	0.505	1.430	0.097
4467	0.326	0.285	0.142	0.234	−0.336	0.341	0.097	−1.181	0.467
4468	0.453	1.093	0.142	0.488	1.302	0.371	0.484	1.295	0.142
4469	0.482	1.279	0.501	0.512	1.453	0.508	0.461	1.144	0.508
4470	0.482	1.279	0.224	0.512	1.453	0.273	0.461	1.144	0.296
4471	0.224	−0.373	0.481	0.275	−0.073	0.193	0.184	−0.625	0.142
4472	0.226	−0.356	0.450	0.310	0.153	0.273	0.187	−0.608	0.512
4473	0.142	−0.895	0.467	0.342	0.361	0.273	0.100	−1.164	0.185
4474	0.184	−0.625	0.450	0.371	0.549	0.273	0.000	?	0.467
4475	0.500	1.397	0.492	0.512	1.453	0.235	0.453	1.093	0.097
4476	0.295	0.082	0.328	0.275	−0.073	0.422	0.097	−1.181	0.262
4477	0.261	−0.137	0.142	0.193	−0.600	0.462	0.261	−0.137	0.262
4478	0.295	0.082	0.185	0.275	−0.073	0.462	0.050	−1.485	0.050
4479	0.295	0.082	0.185	0.275	−0.073	0.193	0.050	−1.485	0.142
4480	0.295	0.082	0.467	0.275	−0.073	0.193	0.050	−1.485	0.481
4481	0.263	−0.120	0.185	0.234	−0.336	0.462	0.050	−1.485	0.296
4482	0.450	1.076	0.492	0.421	0.869	0.501	0.500	1.397	0.467
4483	0.471	1.211	0.262	0.465	1.152	0.193	0.495	1.363	0.512
4484	0.471	1.211	0.097	0.465	1.152	0.501	0.495	1.363	0.431
4485	0.226	−0.356	0.328	0.196	−0.581	0.193	0.142	−0.895	0.508
4486	0.226	−0.356	0.508	0.196	−0.581	0.235	0.142	−0.895	0.512
4487	0.495	1.363	0.385	0.465	1.152	0.309	0.492	1.346	0.142
4488	0.408	0.807	0.492	0.500	1.378	0.273	0.503	1.413	0.224
4489	0.263	−0.120	0.481	0.193	−0.600	0.273	0.142	−0.895	0.185
4490	0.468	1.194	0.512	0.500	1.378	0.273	0.484	1.295	0.097
4491	0.263	−0.120	0.513	0.193	−0.600	0.193	0.145	−0.878	0.450
4492	0.142	−0.895	0.097	0.237	−0.317	0.341	0.187	−0.608	0.185
4493	0.187	−0.608	0.097	0.310	0.153	0.478	0.297	0.099	0.385
4494	0.261	−0.137	0.431	0.272	−0.091	0.501	0.050	−1.485	0.358
4495	0.261	−0.137	0.431	0.272	−0.091	0.102	0.050	−1.485	0.262
4496	0.261	−0.137	0.467	0.272	−0.091	0.501	0.050	−1.485	0.328
4497	0.387	0.672	0.512	0.196	−0.581	0.273	0.097	−1.181	0.512
4498	0.142	−0.895	0.492	0.339	0.342	0.462	0.142	−0.895	0.409
4499	0.492	1.346	0.450	0.474	1.208	0.462	0.479	1.262	0.508
4500	0.492	1.346	0.492	0.503	1.396	0.508	0.482	1.279	0.512
4501	0.184	−0.625	0.492	0.102	−1.184	0.149	0.263	−0.120	0.409
4502	0.500	1.397	0.467	0.494	1.340	0.501	0.479	1.262	0.409
4503	0.226	−0.356	0.467	0.272	−0.091	0.444	0.142	−0.895	0.224
4504	0.484	1.295	0.492	0.459	1.114	0.512	0.503	1.413	0.262
4505	0.508	1.447	0.501	0.465	1.152	0.512	0.500	1.397	0.512
4506	0.492	1.346	0.142	0.509	1.434	0.512	0.482	1.279	0.501
4507	0.226	−0.356	0.224	0.149	−0.882	0.273	0.184	−0.625	0.467
4508	0.492	1.346	0.224	0.500	1.378	0.508	0.474	1.228	0.501
4509	0.489	1.329	0.501	0.439	0.982	0.193	0.505	1.430	0.431
4510	0.479	1.262	0.501	0.512	1.453	0.491	0.495	1.363	0.508

4511	0.479	1.262	0.097	0.512	1.453	0.193	0.495	1.363	0.501
4512	0.453	1.093	0.501	0.515	1.472	0.491	0.461	1.144	0.512
4513	0.224	-0.373	0.142	0.275	-0.073	0.273	0.097	-1.181	0.492
4514	0.447	1.060	0.328	0.506	1.415	0.512	0.461	1.144	0.492
4515	0.295	0.082	0.185	0.193	-0.600	0.193	0.142	-0.895	0.512
4516	0.500	1.397	0.224	0.491	1.321	0.235	0.505	1.430	0.450
4517	0.097	-1.181	0.185	0.193	-0.600	0.309	0.295	0.082	0.224
4518	0.358	0.487	0.409	0.000	?	0.398	0.142	-0.895	0.097
4519	0.482	1.279	0.481	0.488	1.302	0.273	0.505	1.430	0.185
4520	0.358	0.487	0.481	0.272	-0.091	0.422	0.187	-0.608	0.508
4521	0.500	1.397	0.501	0.512	1.453	0.341	0.461	1.144	0.508
4522	0.329	0.301	0.224	0.193	-0.600	0.491	0.097	-1.181	0.328
4523	0.261	-0.137	0.467	0.234	-0.336	0.501	0.263	-0.120	0.467
4524	0.297	0.099	0.224	0.310	0.153	0.491	0.263	-0.120	0.224
4525	0.142	-0.895	0.224	0.401	0.737	0.422	0.050	-1.485	0.431
4526	0.142	-0.895	0.224	0.275	-0.073	0.508	0.145	-0.878	0.224
4527	0.503	1.413	0.224	0.421	0.869	0.193	0.474	1.228	0.385
4528	0.224	-0.373	0.467	0.342	0.361	0.193	0.300	0.116	0.296
4529	0.476	1.245	0.142	0.491	1.321	0.193	0.466	1.178	0.097
4530	0.450	1.076	0.185	0.497	1.359	0.309	0.505	1.430	0.492
4531	0.466	1.178	0.328	0.488	1.302	0.501	0.426	0.925	0.501
4532	0.450	1.076	0.296	0.424	0.888	0.371	0.500	1.397	0.481
4533	0.492	1.346	0.467	0.512	1.453	0.501	0.508	1.447	0.262
4534	0.329	0.301	0.467	0.193	-0.600	0.398	0.142	-0.895	0.467
4535	0.142	-0.895	0.142	0.193	-0.600	0.235	0.224	-0.373	0.224
4536	0.184	-0.625	0.492	0.193	-0.600	0.273	0.184	-0.625	0.097
4537	0.187	-0.608	0.224	0.310	0.153	0.462	0.097	-1.181	0.328
4538	0.463	1.161	0.097	0.500	1.378	0.193	0.447	1.060	0.328
4539	0.184	-0.625	0.492	0.377	0.587	0.508	0.184	-0.625	0.513
4540	0.495	1.363	0.358	0.503	1.396	0.508	0.387	0.672	0.296
4541	0.263	-0.120	0.512	0.401	0.737	0.501	0.355	0.470	0.142
4542	0.097	-1.181	0.262	0.234	-0.336	0.501	0.261	-0.137	0.097
4543	0.329	0.301	0.508	0.275	-0.073	0.309	0.329	0.301	0.185
4544	0.511	1.464	0.512	0.465	1.152	0.273	0.513	1.481	0.501
4545	0.387	0.672	0.142	0.196	-0.581	0.508	0.408	0.807	0.501
4546	0.497	1.380	0.224	0.509	1.434	0.508	0.508	1.447	0.185
4547	0.484	1.295	0.450	0.500	1.378	0.512	0.511	1.464	0.481
4548	0.508	1.447	0.467	0.497	1.359	0.398	0.405	0.790	0.142
4549	0.511	1.464	0.224	0.497	1.359	0.512	0.405	0.790	0.185
4550	0.097	-1.181	0.481	0.313	0.172	0.508	0.224	-0.373	0.431
4551	0.097	-1.181	0.328	0.275	-0.073	0.491	0.261	-0.137	0.224
4552	0.426	0.925	0.512	0.515	1.472	0.508	0.505	1.430	0.385
4553	0.429	0.942	0.467	0.506	1.415	0.508	0.497	1.380	0.262
4554	0.461	1.144	0.508	0.515	1.472	0.508	0.461	1.144	0.512
4555	0.511	1.464	0.185	0.398	0.718	0.501	0.503	1.413	0.358
4556	0.489	1.329	0.185	0.512	1.453	0.514	0.429	0.942	0.185
4557	0.447	1.060	0.501	0.503	1.396	0.193	0.505	1.430	0.185
4558	0.489	1.329	0.467	0.485	1.283	0.235	0.500	1.397	0.492
4559	0.492	1.346	0.450	0.506	1.415	0.149	0.508	1.447	0.481
4560	0.463	1.161	0.512	0.509	1.434	0.508	0.487	1.312	0.224
4561	0.463	1.161	0.296	0.509	1.434	0.508	0.487	1.312	0.501
4562	0.492	1.346	0.185	0.500	1.378	0.149	0.516	1.498	0.050
4563	0.495	1.363	0.328	0.515	1.472	0.491	0.447	1.060	0.501
4564	0.142	-0.895	0.481	0.196	-0.581	0.273	0.224	-0.373	0.481
4565	0.224	-0.373	0.481	0.234	-0.336	0.341	0.097	-1.181	0.512
4566	0.224	-0.373	0.328	0.149	-0.882	0.273	0.184	-0.625	0.185
4567	0.495	1.363	0.467	0.509	1.434	0.273	0.505	1.430	0.185
4568	0.495	1.363	0.492	0.509	1.434	0.273	0.505	1.430	0.431
4569	0.097	-1.181	0.508	0.149	-0.882	0.235	0.329	0.301	0.501
4570	0.500	1.397	0.185	0.485	1.283	0.514	0.463	1.161	0.481
4571	0.145	-0.878	0.224	0.272	-0.091	0.514	0.224	-0.373	0.481

4572	0.329	0.301	0.262	0.342	0.361	0.508	0.434	0.975	0.185
4573	0.184	-0.625	0.450	0.275	-0.073	0.193	0.224	-0.373	0.224
4574	0.226	-0.356	0.142	0.272	-0.091	0.444	0.387	0.672	0.224
4575	0.187	-0.608	0.142	0.272	-0.091	0.149	0.300	0.116	0.467
4576	0.411	0.824	0.224	0.234	-0.336	0.341	0.097	-1.181	0.467
4577	0.482	1.279	0.481	0.512	1.453	0.341	0.484	1.295	0.358
4578	0.482	1.279	0.185	0.512	1.453	0.341	0.492	1.346	0.512
4579	0.505	1.430	0.492	0.509	1.434	0.398	0.482	1.279	0.467
4580	0.224	-0.373	0.358	0.193	-0.600	0.193	0.261	-0.137	0.431
4581	0.466	1.178	0.097	0.442	1.001	0.273	0.466	1.178	0.142
4582	0.224	-0.373	0.050	0.149	-0.882	0.102	0.224	-0.373	0.328
4583	0.224	-0.373	0.262	0.345	0.379	0.398	0.097	-1.181	0.224
4584	0.224	-0.373	0.481	0.339	0.342	0.462	0.329	0.301	0.296
4585	0.224	-0.373	0.508	0.339	0.342	0.462	0.329	0.301	0.185
4586	0.468	1.194	0.262	0.395	0.700	0.235	0.513	1.481	0.224
4587	0.142	-0.895	0.296	0.193	-0.600	0.501	0.297	0.099	0.467
4588	0.184	-0.625	0.262	0.272	-0.091	0.371	0.142	-0.895	0.262
4589	0.329	0.301	0.450	0.102	-1.184	0.273	0.097	-1.181	0.513
4590	0.297	0.099	0.501	0.401	0.737	0.422	0.187	-0.608	0.142
4591	0.463	1.161	0.481	0.465	1.152	0.193	0.497	1.380	0.262
4592	0.463	1.161	0.512	0.465	1.152	0.508	0.497	1.380	0.224
4593	0.145	-0.878	0.262	0.237	-0.317	0.053	0.184	-0.625	0.142
4594	0.489	1.329	0.358	0.506	1.415	0.371	0.479	1.262	0.492
4595	0.226	-0.356	0.512	0.374	0.568	0.508	0.142	-0.895	0.450
4596	0.097	-1.181	0.224	0.278	-0.054	0.235	0.184	-0.625	0.185
4597	0.487	1.312	0.508	0.424	0.888	0.273	0.429	0.942	0.185
4598	0.361	0.504	0.467	0.193	-0.600	0.491	0.224	-0.373	0.358
4599	0.511	1.464	0.224	0.506	1.415	0.398	0.384	0.655	0.492
4600	0.263	-0.120	0.501	0.053	-1.504	0.149	0.261	-0.137	0.450
4601	0.511	1.464	0.467	0.368	0.530	0.514	0.513	1.481	0.501
4602	0.505	1.430	0.224	0.506	1.415	0.149	0.355	0.470	0.450
4603	0.142	-0.895	0.481	0.240	-0.299	0.478	0.184	-0.625	0.224
4604	0.226	-0.356	0.224	0.272	-0.091	0.444	0.187	-0.608	0.224
4605	0.445	1.043	0.492	0.494	1.340	0.444	0.487	1.312	0.409
4606	0.466	1.178	0.409	0.395	0.700	0.193	0.479	1.262	0.050
4607	0.224	-0.373	0.185	0.149	-0.882	0.193	0.226	-0.356	0.481
4608	0.482	1.279	0.512	0.512	1.453	0.514	0.495	1.363	0.492
4609	0.329	0.301	0.501	0.149	-0.882	0.508	0.050	-1.485	0.328
4610	0.516	1.498	0.508	0.477	1.227	0.508	0.497	1.380	0.513
4611	0.468	1.194	0.501	0.442	1.001	0.398	0.479	1.262	0.481
4612	0.513	1.481	0.508	0.447	1.039	0.235	0.511	1.464	0.224
4613	0.184	-0.625	0.296	0.193	-0.600	0.273	0.187	-0.608	0.512
4614	0.184	-0.625	0.262	0.196	-0.581	0.193	0.187	-0.608	0.224
4615	0.503	1.413	0.097	0.512	1.453	0.501	0.429	0.942	0.409
4616	0.466	1.178	0.262	0.503	1.396	0.501	0.500	1.397	0.508
4617	0.447	1.060	0.185	0.503	1.396	0.309	0.482	1.279	0.185
4618	0.508	1.447	0.262	0.401	0.737	0.444	0.479	1.262	0.467
4619	0.297	0.099	0.262	0.234	-0.336	0.462	0.184	-0.625	0.450
4620	0.184	-0.625	0.481	0.275	-0.073	0.491	0.224	-0.373	0.508
4621	0.332	0.318	0.512	0.193	-0.600	0.235	0.224	-0.373	0.501
4622	0.479	1.262	0.385	0.503	1.396	0.309	0.471	1.211	0.512
4623	0.479	1.262	0.224	0.503	1.396	0.398	0.471	1.211	0.185
4624	0.329	0.301	0.492	0.313	0.172	0.309	0.358	0.487	0.328
4625	0.463	1.161	0.296	0.442	1.001	0.235	0.518	1.514	0.296
4626	0.495	1.363	0.492	0.468	1.170	0.273	0.466	1.178	0.142
4627	0.513	1.481	0.328	0.494	1.340	0.149	0.432	0.958	0.224
4628	0.184	-0.625	0.296	0.237	-0.317	0.512	0.142	-0.895	0.185
4629	0.224	-0.373	0.467	0.313	0.172	0.102	0.000	?	0.262
4630	0.263	-0.120	0.142	0.395	0.700	0.501	0.329	0.301	0.512
4631	0.455	1.110	0.224	0.307	0.135	0.341	0.224	-0.373	0.450
4632	0.142	-0.895	0.481	0.234	-0.336	0.341	0.295	0.082	0.501

4633	0.145	−0.878	0.328	0.272	−0.091	0.309	0.184	−0.625	0.328
4634	0.226	−0.356	0.431	0.149	−0.882	0.149	0.224	−0.373	0.512
4635	0.482	1.279	0.467	0.509	1.434	0.491	0.466	1.178	0.296
4636	0.184	−0.625	0.481	0.102	−1.184	0.422	0.261	−0.137	0.450
4637	0.495	1.363	0.224	0.500	1.378	0.235	0.516	1.498	0.224
4638	0.358	0.487	0.492	0.342	0.361	0.193	0.142	−0.895	0.328
4639	0.100	−1.164	0.296	0.342	0.361	0.462	0.261	−0.137	0.467
4640	0.050	−1.485	0.512	0.307	0.135	0.444	0.224	−0.373	0.262
4641	0.261	−0.137	0.467	0.152	−0.863	0.501	0.145	−0.878	0.358
4642	0.484	1.295	0.501	0.491	1.321	0.508	0.492	1.346	0.492
4643	0.513	1.481	0.185	0.424	0.888	0.422	0.447	1.060	0.185
4644	0.263	−0.120	0.262	0.237	−0.317	0.053	0.184	−0.625	0.512
4645	0.297	0.099	0.262	0.196	−0.581	0.102	0.184	−0.625	0.512
4646	0.266	−0.103	0.328	0.465	1.152	0.462	0.358	0.487	0.512
4647	0.447	1.060	0.467	0.442	1.001	0.341	0.487	1.312	0.185
4648	0.503	1.413	0.185	0.503	1.396	0.512	0.453	1.093	0.431
4649	0.482	1.279	0.501	0.506	1.415	0.462	0.505	1.430	0.185
4650	0.511	1.464	0.328	0.424	0.888	0.273	0.453	1.093	0.467
4651	0.266	−0.103	0.385	0.053	−1.504	0.512	0.224	−0.373	0.481
4652	0.361	0.504	0.431	0.102	−1.184	0.491	0.224	−0.373	0.492
4653	0.511	1.464	0.262	0.462	1.133	0.309	0.405	0.790	0.224
4654	0.224	−0.373	0.262	0.345	0.379	0.478	0.050	−1.485	0.097
4655	0.508	1.447	0.296	0.506	1.415	0.149	0.476	1.245	0.097
4656	0.471	1.211	0.450	0.462	1.133	0.491	0.489	1.329	0.358
4657	0.224	−0.373	0.224	0.272	−0.091	0.512	0.332	0.318	0.450
4658	0.505	1.430	0.492	0.509	1.434	0.341	0.508	1.447	0.224
4659	0.463	1.161	0.262	0.491	1.321	0.512	0.479	1.262	0.512
4660	0.224	−0.373	0.262	0.310	0.153	0.512	0.226	−0.356	0.185
4661	0.479	1.262	0.296	0.477	1.227	0.478	0.513	1.481	0.512
4662	0.226	−0.356	0.492	0.149	−0.882	0.422	0.226	−0.356	0.481
4663	0.495	1.363	0.501	0.491	1.321	0.491	0.408	0.807	0.224
4664	0.413	0.841	0.262	0.518	1.491	0.371	0.508	1.447	0.262
4665	0.187	−0.608	0.262	0.342	0.361	0.273	0.184	−0.625	0.409
4666	0.516	1.498	0.224	0.509	1.434	0.235	0.468	1.194	0.358
4667	0.495	1.363	0.467	0.506	1.415	0.273	0.453	1.093	0.296
4668	0.511	1.464	0.501	0.477	1.227	0.149	0.505	1.430	0.492
4669	0.500	1.397	0.409	0.421	0.869	0.273	0.500	1.397	0.097
4670	0.505	1.430	0.328	0.497	1.359	0.341	0.516	1.498	0.050
4671	0.300	0.116	0.097	0.371	0.549	0.398	0.184	−0.625	0.142
4672	0.261	−0.137	0.224	0.272	−0.091	0.444	0.334	0.335	0.501
4673	0.097	−1.181	0.358	0.234	−0.336	0.478	0.295	0.082	0.513
4674	0.263	−0.120	0.262	0.272	−0.091	0.053	0.142	−0.895	0.224
4675	0.187	−0.608	0.185	0.149	−0.882	0.501	0.226	−0.356	0.224
4676	0.261	−0.137	0.513	0.272	−0.091	0.341	0.187	−0.608	0.185
4677	0.261	−0.137	0.185	0.342	0.361	0.478	0.263	−0.120	0.501
4678	0.482	1.279	0.409	0.401	0.737	0.235	0.511	1.464	0.492
4679	0.513	1.481	0.501	0.444	1.020	0.273	0.447	1.060	0.296
4680	0.384	0.655	0.328	0.480	1.246	0.508	0.503	1.413	0.142
4681	0.224	−0.373	0.385	0.053	−1.504	0.273	0.332	0.318	0.262
4682	0.495	1.363	0.224	0.503	1.396	0.341	0.508	1.447	0.358
4683	0.295	0.082	0.508	0.342	0.361	0.508	0.300	0.116	0.358
4684	0.489	1.329	0.467	0.477	1.227	0.501	0.450	1.076	0.262
4685	0.329	0.301	0.262	0.237	−0.317	0.273	0.226	−0.356	0.450
4686	0.300	0.116	0.142	0.275	−0.073	0.462	0.326	0.285	0.481
4687	0.463	1.161	0.262	0.509	1.434	0.193	0.466	1.178	0.224
4688	0.142	−0.895	0.262	0.272	−0.091	0.478	0.266	−0.103	0.512
4689	0.226	−0.356	0.501	0.339	0.342	0.102	0.363	0.520	0.512
4690	0.479	1.262	0.296	0.509	1.434	0.478	0.487	1.312	0.296
4691	0.332	0.318	0.328	0.000	?	0.501	0.184	−0.625	0.492
4692	0.432	0.958	0.142	0.503	1.396	0.462	0.511	1.464	0.262
4693	0.466	1.178	0.050	0.275	−0.073	0.193	0.513	1.481	0.508

4694	0.479	1.262	0.050	0.465	1.152	0.193	0.513	1.481	0.512
4695	0.226	-0.356	0.097	0.193	-0.600	0.193	0.184	-0.625	0.296
4696	0.492	1.346	0.050	0.474	1.208	0.235	0.432	0.958	0.224
4697	0.297	0.099	0.097	0.102	-1.184	0.478	0.187	-0.608	0.262
4698	0.513	1.481	0.492	0.474	1.208	0.149	0.468	1.194	0.431
4699	0.463	1.161	0.328	0.500	1.378	0.491	0.476	1.245	0.467
4700	0.497	1.380	0.501	0.456	1.095	0.102	0.492	1.346	0.358
4701	0.184	-0.625	0.501	0.193	-0.600	0.512	0.224	-0.373	0.185
4702	0.263	-0.120	0.262	0.193	-0.600	0.444	0.097	-1.181	0.224
4703	0.263	-0.120	0.097	0.193	-0.600	0.235	0.097	-1.181	0.431
4704	0.332	0.318	0.097	0.234	-0.336	0.462	0.355	0.470	0.296
4705	0.463	1.161	0.508	0.480	1.246	0.309	0.450	1.076	0.262
4706	0.184	-0.625	0.512	0.149	-0.882	0.508	0.224	-0.373	0.358
4707	0.500	1.397	0.512	0.491	1.321	0.273	0.511	1.464	0.050
4708	0.329	0.301	0.513	0.102	-1.184	0.491	0.184	-0.625	0.512
4709	0.384	0.655	0.513	0.512	1.453	0.193	0.505	1.430	0.262
4710	0.434	0.975	0.508	0.439	0.982	0.273	0.479	1.262	0.512
4711	0.261	-0.137	0.385	0.234	-0.336	0.309	0.224	-0.373	0.501
4712	0.261	-0.137	0.492	0.465	1.152	0.478	0.261	-0.137	0.224
4713	0.297	0.099	0.492	0.313	0.172	0.491	0.411	0.824	0.296
4714	0.450	1.076	0.501	0.509	1.434	0.341	0.361	0.504	0.262
4715	0.226	-0.356	0.185	0.278	-0.054	0.341	0.295	0.082	0.481
4716	0.492	1.346	0.512	0.491	1.321	0.309	0.495	1.363	0.501
4717	0.261	-0.137	0.296	0.193	-0.600	0.512	0.097	-1.181	0.512
4718	0.261	-0.137	0.409	0.275	-0.073	0.501	0.050	-1.485	0.512
4719	0.297	0.099	0.409	0.307	0.135	0.273	0.142	-0.895	0.512
4720	0.495	1.363	0.501	0.482	1.265	0.371	0.497	1.380	0.508
4721	0.500	1.397	0.501	0.488	1.302	0.193	0.511	1.464	0.450
4722	0.263	-0.120	0.508	0.345	0.379	0.371	0.224	-0.373	0.512
4723	0.263	-0.120	0.512	0.345	0.379	0.149	0.224	-0.373	0.513
4724	0.226	-0.356	0.467	0.313	0.172	0.102	0.184	-0.625	0.492
4725	0.463	1.161	0.513	0.512	1.453	0.235	0.497	1.380	0.097
4726	0.495	1.363	0.358	0.503	1.396	0.514	0.489	1.329	0.467
4727	0.411	0.824	0.328	0.272	-0.091	0.309	0.297	0.099	0.296
4728	0.329	0.301	0.481	0.371	0.549	0.501	0.142	-0.895	0.492
4729	0.097	-1.181	0.185	0.196	-0.581	0.478	0.261	-0.137	0.492
4730	0.224	-0.373	0.512	0.374	0.568	0.341	0.358	0.487	0.409
4731	0.361	0.504	0.512	0.149	-0.882	0.501	0.355	0.470	0.492
4732	0.263	-0.120	0.508	0.102	-1.184	0.193	0.261	-0.137	0.508
4733	0.184	-0.625	0.296	0.237	-0.317	0.491	0.453	1.093	0.296
4734	0.513	1.481	0.431	0.518	1.491	0.514	0.476	1.245	0.508
4735	0.184	-0.625	0.501	0.313	0.172	0.235	0.226	-0.356	0.508
4736	0.408	0.807	0.185	0.500	1.378	0.193	0.505	1.430	0.492
4737	0.500	1.397	0.185	0.482	1.265	0.235	0.516	1.498	0.185
4738	0.326	0.285	0.450	0.342	0.361	0.341	0.297	0.099	0.185
4739	0.382	0.638	0.467	0.500	1.378	0.514	0.497	1.380	0.501
4740	0.226	-0.356	0.467	0.193	-0.600	0.235	0.261	-0.137	0.185
4741	0.503	1.413	0.450	0.491	1.321	0.149	0.508	1.447	0.501
4742	0.468	1.194	0.508	0.512	1.453	0.273	0.516	1.498	0.481
4743	0.263	-0.120	0.050	0.234	-0.336	0.193	0.297	0.099	0.512
4744	0.142	-0.895	0.050	0.193	-0.600	0.235	0.224	-0.373	0.097
4745	0.263	-0.120	0.050	0.237	-0.317	0.371	0.263	-0.120	0.513
4746	0.263	-0.120	0.050	0.345	0.379	0.422	0.432	0.958	0.513
4747	0.497	1.380	0.097	0.512	1.453	0.371	0.463	1.161	0.262
4748	0.297	0.099	0.501	0.234	-0.336	0.444	0.358	0.487	0.262
4749	0.329	0.301	0.296	0.149	-0.882	0.309	0.184	-0.625	0.508
4750	0.142	-0.895	0.501	0.275	-0.073	0.341	0.224	-0.373	0.481
4751	0.050	-1.485	0.097	0.193	-0.600	0.514	0.434	0.975	0.501
4752	0.050	-1.485	0.050	0.237	-0.317	0.149	0.295	0.082	0.481
4753	0.097	-1.181	0.050	0.374	0.568	0.149	0.261	-0.137	0.409
4754	0.050	-1.485	0.512	0.421	0.869	0.193	0.363	0.520	0.185

4755	0.097	-1.181	0.262	0.371	0.549	0.501	0.050	-1.485	0.262
4756	0.492	1.346	0.512	0.447	1.039	0.444	0.503	1.413	0.296
4757	0.332	0.318	0.050	0.310	0.153	0.491	0.261	-0.137	0.296
4758	0.503	1.413	0.328	0.342	0.361	0.491	0.505	1.430	0.358
4759	0.503	1.413	0.481	0.515	1.472	0.491	0.500	1.397	0.262
4760	0.261	-0.137	0.492	0.149	-0.882	0.512	0.226	-0.356	0.262
4761	0.097	-1.181	0.501	0.152	-0.863	0.514	0.295	0.082	0.185
4762	0.097	-1.181	0.431	0.196	-0.581	0.508	0.261	-0.137	0.450
4763	0.505	1.430	0.185	0.497	1.359	0.462	0.484	1.295	0.050
4764	0.511	1.464	0.185	0.439	0.982	0.508	0.505	1.430	0.501
4765	0.511	1.464	0.513	0.491	1.321	0.309	0.505	1.430	0.224
4766	0.513	1.481	0.492	0.491	1.321	0.491	0.505	1.430	0.224
4767	0.513	1.481	0.431	0.491	1.321	0.102	0.505	1.430	0.450
4768	0.505	1.430	0.467	0.512	1.453	0.478	0.503	1.413	0.142
4769	0.387	0.672	0.512	0.520	1.509	0.462	0.447	1.060	0.501
4770	0.484	1.295	0.512	0.509	1.434	0.053	0.511	1.464	0.185
4771	0.492	1.346	0.467	0.465	1.152	0.422	0.505	1.430	0.224
4772	0.497	1.380	0.492	0.506	1.415	0.398	0.489	1.329	0.513
4773	0.184	-0.625	0.467	0.310	0.153	0.491	0.097	-1.181	0.142
4774	0.505	1.430	0.224	0.485	1.283	0.501	0.471	1.211	0.185
4775	0.300	0.116	0.450	0.105	-1.165	0.512	0.295	0.082	0.328
4776	0.411	0.824	0.512	0.477	1.227	0.491	0.492	1.346	0.385
4777	0.411	0.824	0.467	0.459	1.114	0.149	0.492	1.346	0.508
4778	0.000	?	0.512	0.053	-1.504	0.149	0.408	0.807	0.508
4779	0.492	1.346	0.142	0.418	0.850	0.508	0.495	1.363	0.513
4780	0.492	1.346	0.262	0.395	0.700	0.193	0.511	1.464	0.296
4781	0.497	1.380	0.492	0.491	1.321	0.462	0.295	0.082	0.385
4782	0.508	1.447	0.467	0.494	1.340	0.478	0.508	1.447	0.508
4783	0.463	1.161	0.050	0.506	1.415	0.508	0.511	1.464	0.501
4784	0.513	1.481	0.262	0.482	1.265	0.235	0.495	1.363	0.481
4785	0.358	0.487	0.501	0.149	-0.882	0.508	0.184	-0.625	0.492
4786	0.329	0.301	0.501	0.149	-0.882	0.422	0.184	-0.625	0.508
4787	0.484	1.295	0.481	0.503	1.396	0.149	0.497	1.380	0.385
4788	0.184	-0.625	0.481	0.196	-0.581	0.149	0.184	-0.625	0.492
4789	0.500	1.397	0.385	0.465	1.152	0.508	0.503	1.413	0.492
4790	0.500	1.397	0.450	0.482	1.265	0.491	0.482	1.279	0.492
4791	0.497	1.380	0.262	0.515	1.472	0.514	0.518	1.514	0.097
4792	0.295	0.082	0.296	0.234	-0.336	0.514	0.097	-1.181	0.512
4793	0.432	0.958	0.296	0.509	1.434	0.501	0.508	1.447	0.481
4794	0.497	1.380	0.492	0.421	0.869	0.309	0.511	1.464	0.513
4795	0.184	-0.625	0.492	0.152	-0.863	0.371	0.263	-0.120	0.467
4796	0.184	-0.625	0.296	0.152	-0.863	0.273	0.263	-0.120	0.328
4797	0.445	1.043	0.185	0.512	1.453	0.273	0.503	1.413	0.097
4798	0.461	1.144	0.501	0.497	1.359	0.235	0.476	1.245	0.512
4799	0.463	1.161	0.492	0.512	1.453	0.371	0.500	1.397	0.508
4800	0.447	1.060	0.431	0.512	1.453	0.371	0.482	1.279	0.097
4801	0.497	1.380	0.431	0.500	1.378	0.371	0.413	0.841	0.185
4802	0.050	-1.485	0.481	0.310	0.153	0.514	0.184	-0.625	0.492
4803	0.050	-1.485	0.328	0.374	0.568	0.193	0.261	-0.137	0.492
4804	0.000	?	0.508	0.272	-0.091	0.444	0.297	0.099	0.358
4805	0.000	?	0.467	0.272	-0.091	0.273	0.297	0.099	0.481
4806	0.000	?	0.492	0.234	-0.336	0.341	0.361	0.504	0.450
4807	0.050	-1.485	0.512	0.371	0.549	0.371	0.263	-0.120	0.492
4808	0.050	-1.485	0.142	0.371	0.549	0.341	0.263	-0.120	0.296
4809	0.097	-1.181	0.512	0.371	0.549	0.508	0.184	-0.625	0.050
4810	0.503	1.413	0.501	0.506	1.415	0.102	0.450	1.076	0.185
4811	0.300	0.116	0.512	0.193	-0.600	0.398	0.050	-1.485	0.508
4812	0.492	1.346	0.185	0.444	1.020	0.462	0.500	1.397	0.501
4813	0.097	-1.181	0.262	0.272	-0.091	0.398	0.224	-0.373	0.097
4814	0.000	?	0.431	0.345	0.379	0.341	0.224	-0.373	0.328
4815	0.050	-1.485	0.431	0.371	0.549	0.053	0.450	1.076	0.492

4816	0.050	-1.485	0.224	0.342	0.361	0.514	0.142	-0.895	0.409
4817	0.503	1.413	0.358	0.506	1.415	0.478	0.503	1.413	0.492
4818	0.261	-0.137	0.481	0.102	-1.184	0.514	0.187	-0.608	0.513
4819	0.000	?	0.501	0.398	0.718	0.508	0.226	-0.356	0.508
4820	0.503	1.413	0.097	0.465	1.152	0.193	0.511	1.464	0.097
4821	0.000	?	0.097	0.401	0.737	0.193	0.142	-0.895	0.481
4822	0.050	-1.485	0.492	0.339	0.342	0.462	0.187	-0.608	0.481
4823	0.332	0.318	0.262	0.053	-1.504	0.508	0.329	0.301	0.467
4824	0.474	1.228	0.508	0.515	1.472	0.491	0.384	0.655	0.508
4825	0.484	1.295	0.328	0.482	1.265	0.491	0.511	1.464	0.097
4826	0.500	1.397	0.481	0.512	1.453	0.462	0.503	1.413	0.508
4827	0.426	0.925	0.481	0.500	1.378	0.508	0.516	1.498	0.450
4828	0.184	-0.625	0.513	0.193	-0.600	0.508	0.297	0.099	0.385
4829	0.184	-0.625	0.492	0.193	-0.600	0.501	0.389	0.689	0.097
4830	0.503	1.413	0.512	0.471	1.189	0.501	0.500	1.397	0.185
4831	0.492	1.346	0.481	0.509	1.434	0.273	0.495	1.363	0.492
4832	0.432	0.958	0.481	0.497	1.359	0.512	0.482	1.279	0.492
4833	0.466	1.178	0.508	0.494	1.340	0.508	0.492	1.346	0.142
4834	0.511	1.464	0.513	0.468	1.170	0.508	0.513	1.481	0.224
4835	0.505	1.430	0.185	0.515	1.472	0.501	0.387	0.672	0.501
4836	0.468	1.194	0.508	0.512	1.453	0.193	0.492	1.346	0.513
4837	0.495	1.363	0.508	0.503	1.396	0.193	0.492	1.346	0.328
4838	0.463	1.161	0.224	0.497	1.359	0.491	0.492	1.346	0.328
4839	0.224	-0.373	0.328	0.278	-0.054	0.501	0.097	-1.181	0.467
4840	0.447	1.060	0.467	0.515	1.472	0.371	0.508	1.447	0.262
4841	0.511	1.464	0.262	0.506	1.415	0.235	0.476	1.245	0.481
4842	0.463	1.161	0.358	0.506	1.415	0.462	0.508	1.447	0.224
4843	0.508	1.447	0.481	0.503	1.396	0.462	0.461	1.144	0.481
4844	0.142	-0.895	0.450	0.193	-0.600	0.514	0.329	0.301	0.492
4845	0.263	-0.120	0.262	0.193	-0.600	0.478	0.097	-1.181	0.450
4846	0.489	1.329	0.409	0.491	1.321	0.508	0.511	1.464	0.358
4847	0.471	1.211	0.296	0.497	1.359	0.508	0.500	1.397	0.508
4848	0.050	-1.485	0.097	0.371	0.549	0.398	0.100	-1.164	0.481
4849	0.261	-0.137	0.492	0.234	-0.336	0.235	0.187	-0.608	0.467
4850	0.500	1.397	0.508	0.462	1.133	0.193	0.487	1.312	0.512
4851	0.500	1.397	0.450	0.462	1.133	0.501	0.487	1.312	0.467
4852	0.479	1.262	0.450	0.509	1.434	0.422	0.358	0.487	0.142
4853	0.479	1.262	0.262	0.480	1.246	0.193	0.474	1.228	0.508
4854	0.382	0.638	0.513	0.506	1.415	0.371	0.453	1.093	0.508
4855	0.445	1.043	0.508	0.506	1.415	0.508	0.492	1.346	0.097
4856	0.261	-0.137	0.501	0.404	0.756	0.501	0.295	0.082	0.501
4857	0.300	0.116	0.492	0.234	-0.336	0.514	0.050	-1.485	0.450
4858	0.300	0.116	0.262	0.193	-0.600	0.512	0.184	-0.625	0.328
4859	0.489	1.329	0.385	0.497	1.359	0.491	0.505	1.430	0.328
4860	0.489	1.329	0.262	0.421	0.869	0.273	0.505	1.430	0.450
4861	0.297	0.099	0.501	0.193	-0.600	0.491	0.097	-1.181	0.481
4862	0.184	-0.625	0.481	0.374	0.568	0.478	0.332	0.318	0.185
4863	0.500	1.397	0.262	0.506	1.415	0.491	0.495	1.363	0.467
4864	0.497	1.380	0.508	0.500	1.378	0.444	0.408	0.807	0.185
4865	0.434	0.975	0.492	0.512	1.453	0.309	0.495	1.363	0.185
4866	0.434	0.975	0.142	0.512	1.453	0.422	0.516	1.498	0.467
4867	0.476	1.245	0.501	0.491	1.321	0.501	0.505	1.430	0.481
4868	0.329	0.301	0.501	0.272	-0.091	0.508	0.097	-1.181	0.512
4869	0.513	1.481	0.508	0.491	1.321	0.309	0.479	1.262	0.512
4870	0.468	1.194	0.492	0.480	1.246	0.149	0.476	1.245	0.142
4871	0.487	1.312	0.481	0.488	1.302	0.508	0.466	1.178	0.450
4872	0.508	1.447	0.185	0.444	1.020	0.508	0.503	1.413	0.513
4873	0.145	-0.878	0.513	0.307	0.135	0.273	0.097	-1.181	0.431
4874	0.508	1.447	0.492	0.424	0.888	0.149	0.503	1.413	0.431
4875	0.497	1.380	0.467	0.500	1.378	0.491	0.447	1.060	0.409
4876	0.508	1.447	0.224	0.509	1.434	0.508	0.384	0.655	0.512

4877	0.187	-0.608	0.512	0.310	0.153	0.102	0.097	-1.181	0.262
4878	0.263	-0.120	0.481	0.149	-0.882	0.102	0.187	-0.608	0.492
4879	0.429	0.942	0.492	0.506	1.415	0.508	0.492	1.346	0.492
4880	0.429	0.942	0.185	0.506	1.415	0.235	0.492	1.346	0.142
4881	0.226	-0.356	0.513	0.275	-0.073	0.444	0.142	-0.895	0.431
4882	0.363	0.520	0.512	0.149	-0.882	0.273	0.224	-0.373	0.501
4883	0.482	1.279	0.512	0.485	1.283	0.512	0.495	1.363	0.050
4884	0.503	1.413	0.409	0.503	1.396	0.508	0.511	1.464	0.224
4885	0.097	-1.181	0.050	0.102	-1.184	0.444	0.329	0.301	0.508
4886	0.097	-1.181	0.481	0.102	-1.184	0.514	0.329	0.301	0.431
4887	0.489	1.329	0.142	0.506	1.415	0.462	0.466	1.178	0.501
4888	0.263	-0.120	0.501	0.234	-0.336	0.514	0.263	-0.120	0.501
4889	0.505	1.430	0.501	0.439	0.982	0.514	0.482	1.279	0.492
4890	0.329	0.301	0.185	0.275	-0.073	0.491	0.224	-0.373	0.224
4891	0.479	1.262	0.185	0.512	1.453	0.514	0.474	1.228	0.431
4892	0.482	1.279	0.508	0.506	1.415	0.235	0.484	1.295	0.508
4893	0.516	1.498	0.142	0.444	1.020	0.508	0.453	1.093	0.513
4894	0.495	1.363	0.142	0.518	1.491	0.501	0.358	0.487	0.262
4895	0.508	1.447	0.513	0.456	1.095	0.398	0.511	1.464	0.492
4896	0.474	1.228	0.224	0.518	1.491	0.508	0.482	1.279	0.492
4897	0.474	1.228	0.224	0.518	1.491	0.508	0.468	1.194	0.501
4898	0.511	1.464	0.224	0.488	1.302	0.309	0.511	1.464	0.142
4899	0.513	1.481	0.512	0.512	1.453	0.309	0.463	1.161	0.467
4900	0.184	-0.625	0.512	0.240	-0.299	0.508	0.142	-0.895	0.450
4901	0.500	1.397	0.512	0.503	1.396	0.491	0.511	1.464	0.450
4902	0.500	1.397	0.512	0.497	1.359	0.193	0.511	1.464	0.501
4903	0.224	-0.373	0.501	0.401	0.737	0.478	0.097	-1.181	0.185
4904	0.326	0.285	0.185	0.506	1.415	0.273	0.495	1.363	0.508
4905	0.468	1.194	0.097	0.512	1.453	0.273	0.445	1.043	0.185
4906	0.261	-0.137	0.450	0.307	0.135	0.501	0.329	0.301	0.512
4907	0.358	0.487	0.501	0.307	0.135	0.462	0.326	0.285	0.450
4908	0.482	1.279	0.224	0.512	1.453	0.491	0.450	1.076	0.142
4909	0.450	1.076	0.501	0.488	1.302	0.491	0.479	1.262	0.142
4910	0.263	-0.120	0.501	0.193	-0.600	0.398	0.184	-0.625	0.467
4911	0.408	0.807	0.185	0.477	1.227	0.508	0.466	1.178	0.185
4912	0.297	0.099	0.142	0.272	-0.091	0.444	0.184	-0.625	0.185
4913	0.097	-1.181	0.508	0.275	-0.073	0.501	0.184	-0.625	0.501
4914	0.492	1.346	0.409	0.503	1.396	0.501	0.468	1.194	0.328
4915	0.511	1.464	0.296	0.462	1.133	0.102	0.479	1.262	0.328
4916	0.450	1.076	0.512	0.491	1.321	0.491	0.511	1.464	0.328
4917	0.450	1.076	0.224	0.491	1.321	0.398	0.511	1.464	0.481
4918	0.261	-0.137	0.224	0.398	0.718	0.508	0.142	-0.895	0.481
4919	0.516	1.498	0.050	0.512	1.453	0.444	0.450	1.076	0.481
4920	0.503	1.413	0.050	0.447	1.039	0.235	0.511	1.464	0.481
4921	0.497	1.380	0.050	0.500	1.378	0.508	0.432	0.958	0.508
4922	0.489	1.329	0.050	0.500	1.378	0.478	0.432	0.958	0.262
4923	0.263	-0.120	0.050	0.102	-1.184	0.462	0.411	0.824	0.097
4924	0.384	0.655	0.262	0.494	1.340	0.341	0.505	1.430	0.492
4925	0.266	-0.103	0.481	0.401	0.737	0.501	0.261	-0.137	0.492
4926	0.503	1.413	0.492	0.509	1.434	0.501	0.495	1.363	0.097
4927	0.484	1.295	0.508	0.444	1.020	0.501	0.487	1.312	0.501
4928	0.263	-0.120	0.508	0.234	-0.336	0.462	0.145	-0.878	0.501
4929	0.503	1.413	0.481	0.509	1.434	0.508	0.426	0.925	0.050
4930	0.487	1.312	0.481	0.480	1.246	0.273	0.500	1.397	0.142
4931	0.142	-0.895	0.450	0.465	1.152	0.508	0.050	-1.485	0.508
4932	0.000	?	0.185	0.342	0.361	0.512	0.226	-0.356	0.185
4933	0.500	1.397	0.508	0.500	1.378	0.512	0.503	1.413	0.358
4934	0.492	1.346	0.481	0.503	1.396	0.273	0.434	0.975	0.492
4935	0.500	1.397	0.358	0.500	1.378	0.462	0.503	1.413	0.142
4936	0.484	1.295	0.142	0.465	1.152	0.508	0.503	1.413	0.185
4937	0.479	1.262	0.328	0.503	1.396	0.514	0.489	1.329	0.185

4938	0.184	-0.625	0.385	0.275	-0.073	0.235	0.226	-0.356	0.185
4939	0.511	1.464	0.385	0.509	1.434	0.514	0.426	0.925	0.185
4940	0.492	1.346	0.409	0.509	1.434	0.462	0.505	1.430	0.142
4941	0.466	1.178	0.050	0.506	1.415	0.478	0.516	1.498	0.508
4942	0.224	-0.373	0.097	0.272	-0.091	0.444	0.263	-0.120	0.501
4943	0.511	1.464	0.097	0.462	1.133	0.422	0.487	1.312	0.481
4944	0.479	1.262	0.142	0.509	1.434	0.491	0.492	1.346	0.481
4945	0.489	1.329	0.050	0.518	1.491	0.235	0.497	1.380	0.501
4946	0.184	-0.625	0.431	0.237	-0.317	0.444	0.145	-0.878	0.501
4947	0.513	1.481	0.431	0.512	1.453	0.491	0.463	1.161	0.492
4948	0.513	1.481	0.501	0.459	1.114	0.235	0.447	1.060	0.185
4949	0.516	1.498	0.050	0.474	1.208	0.235	0.447	1.060	0.492
4950	0.413	0.841	0.050	0.444	1.020	0.512	0.495	1.363	0.492
4951	0.050	-1.485	0.142	0.427	0.907	0.235	0.184	-0.625	0.097
4952	0.487	1.312	0.185	0.494	1.340	0.235	0.508	1.447	0.296
4953	0.142	-0.895	0.328	0.234	-0.336	0.462	0.184	-0.625	0.513
4954	0.500	1.397	0.224	0.442	1.001	0.371	0.505	1.430	0.513
4955	0.500	1.397	0.224	0.497	1.359	0.371	0.447	1.060	0.513
4956	0.184	-0.625	0.224	0.237	-0.317	0.371	0.145	-0.878	0.513
4957	0.184	-0.625	0.358	0.237	-0.317	0.508	0.145	-0.878	0.409
4958	0.511	1.464	0.097	0.512	1.453	0.508	0.463	1.161	0.431
4959	0.145	-0.878	0.467	0.234	-0.336	0.508	0.184	-0.625	0.409
4960	0.145	-0.878	0.467	0.234	-0.336	0.508	0.184	-0.625	0.358
4961	0.516	1.498	0.512	0.459	1.114	0.462	0.497	1.380	0.409
4962	0.226	-0.356	0.512	0.374	0.568	0.149	0.329	0.301	0.481
4963	0.226	-0.356	0.409	0.374	0.568	0.341	0.329	0.301	0.467
4964	0.226	-0.356	0.185	0.374	0.568	0.501	0.329	0.301	0.492
4965	0.511	1.464	0.385	0.506	1.415	0.478	0.476	1.245	0.262
4966	0.511	1.464	0.185	0.506	1.415	0.235	0.476	1.245	0.097
4967	0.511	1.464	0.097	0.506	1.415	0.444	0.476	1.245	0.185
4968	0.511	1.464	0.224	0.506	1.415	0.444	0.476	1.245	0.224
4969	0.500	1.397	0.224	0.465	1.152	0.309	0.505	1.430	0.385
4970	0.184	-0.625	0.296	0.149	-0.882	0.273	0.261	-0.137	0.097
4971	0.097	-1.181	0.508	0.339	0.342	0.422	0.100	-1.164	0.097
4972	0.447	1.060	0.508	0.500	1.378	0.193	0.495	1.363	0.050
4973	0.500	1.397	0.142	0.480	1.246	0.273	0.487	1.312	0.296
4974	0.226	-0.356	0.358	0.237	-0.317	0.501	0.097	-1.181	0.185
4975	0.500	1.397	0.296	0.439	0.982	0.309	0.497	1.380	0.431
4976	0.500	1.397	0.262	0.439	0.982	0.309	0.497	1.380	0.409
4977	0.187	-0.608	0.467	0.310	0.153	0.478	0.050	-1.485	0.508
4978	0.142	-0.895	0.185	0.272	-0.091	0.462	0.145	-0.878	0.508
4979	0.508	1.447	0.142	0.421	0.869	0.462	0.500	1.397	0.097
4980	0.411	0.824	0.385	0.193	-0.600	0.462	0.184	-0.625	0.185
4981	0.297	0.099	0.262	0.275	-0.073	0.478	0.361	0.504	0.050
4982	0.511	1.464	0.328	0.506	1.415	0.235	0.495	1.363	0.385
4983	0.226	-0.356	0.185	0.307	0.135	0.512	0.000	?	0.409
4984	0.226	-0.356	0.450	0.307	0.135	0.514	0.000	?	0.328
4985	0.050	-1.485	0.431	0.477	1.227	0.512	0.142	-0.895	0.142
4986	0.050	-1.485	0.431	0.462	1.133	0.512	0.184	-0.625	0.142
4987	0.050	-1.485	0.450	0.462	1.133	0.462	0.184	-0.625	0.097
4988	0.050	-1.485	0.508	0.462	1.133	0.462	0.184	-0.625	0.501
4989	0.050	-1.485	0.185	0.480	1.246	0.508	0.184	-0.625	0.501
4990	0.261	-0.137	0.185	0.234	-0.336	0.193	0.145	-0.878	0.296
4991	0.482	1.279	0.492	0.512	1.453	0.514	0.497	1.380	0.050
4992	0.484	1.295	0.492	0.518	1.491	0.514	0.497	1.380	0.185
4993	0.508	1.447	0.492	0.506	1.415	0.149	0.484	1.295	0.185
4994	0.508	1.447	0.492	0.506	1.415	0.235	0.484	1.295	0.501
4995	0.479	1.262	0.492	0.456	1.095	0.512	0.497	1.380	0.262
4996	0.479	1.262	0.492	0.456	1.095	0.508	0.497	1.380	0.328
4997	0.453	1.093	0.467	0.503	1.396	0.508	0.484	1.295	0.296
4998	0.184	-0.625	0.296	0.193	-0.600	0.501	0.184	-0.625	0.097

4999	0.500	1.397	0.262	0.515	1.472	0.235	0.492	1.346	0.142
5000	0.487	1.312	0.513	0.518	1.491	0.273	0.492	1.346	0.097
5001	0.358	0.487	0.481	0.149	-0.882	0.193	0.097	-1.181	0.097
5002	0.142	-0.895	0.481	0.234	-0.336	0.341	0.300	0.116	0.492
5003	0.329	0.301	0.512	0.515	1.472	0.273	0.513	1.481	0.492
5004	0.387	0.672	0.224	0.509	1.434	0.508	0.516	1.498	0.467
5005	0.387	0.672	0.513	0.509	1.434	0.508	0.516	1.498	0.481
5006	0.413	0.841	0.513	0.503	1.396	0.512	0.511	1.464	0.467
5007	0.050	-1.485	0.513	0.234	-0.336	0.398	0.408	0.807	0.224
5008	0.097	-1.181	0.492	0.272	-0.091	0.398	0.432	0.958	0.224
5009	0.097	-1.181	0.513	0.193	-0.600	0.273	0.413	0.841	0.513
5010	0.142	-0.895	0.512	0.342	0.361	0.149	0.358	0.487	0.513
5011	0.050	-1.485	0.508	0.275	-0.073	0.273	0.411	0.824	0.513
5012	0.432	0.958	0.185	0.503	1.396	0.309	0.484	1.295	0.508
5013	0.432	0.958	0.492	0.503	1.396	0.309	0.468	1.194	0.513
5014	0.492	1.346	0.492	0.512	1.453	0.273	0.492	1.346	0.513
5015	0.050	-1.485	0.262	0.398	0.718	0.273	0.263	-0.120	0.512
5016	0.050	-1.485	0.385	0.401	0.737	0.273	0.097	-1.181	0.262
5017	0.142	-0.895	0.185	0.272	-0.091	0.422	0.184	-0.625	0.050
5018	0.184	-0.625	0.492	0.149	-0.882	0.422	0.224	-0.373	0.513
5019	0.326	0.285	0.224	0.275	-0.073	0.444	0.384	0.655	0.508
5020	0.226	-0.356	0.296	0.307	0.135	0.444	0.097	-1.181	0.508
5021	0.226	-0.356	0.501	0.307	0.135	0.193	0.097	-1.181	0.512
5022	0.226	-0.356	0.512	0.272	-0.091	0.193	0.050	-1.485	0.296
5023	0.358	0.487	0.492	0.275	-0.073	0.309	0.300	0.116	0.467
5024	0.097	-1.181	0.492	0.275	-0.073	0.273	0.184	-0.625	0.481
5025	0.466	1.178	0.450	0.421	0.869	0.273	0.429	0.942	0.467
5026	0.466	1.178	0.492	0.421	0.869	0.398	0.408	0.807	0.481
5027	0.516	1.498	0.492	0.442	1.001	0.341	0.503	1.413	0.467
5028	0.516	1.498	0.185	0.442	1.001	0.193	0.503	1.413	0.481
5029	0.411	0.824	0.185	0.193	-0.600	0.309	0.097	-1.181	0.492
5030	0.187	-0.608	0.185	0.193	-0.600	0.444	0.187	-0.608	0.262
5031	0.382	0.638	0.409	0.307	0.135	0.444	0.050	-1.485	0.467
5032	0.000	?	0.492	0.275	-0.073	0.102	0.382	0.638	0.492
5033	0.187	-0.608	0.508	0.275	-0.073	0.235	0.411	0.824	0.385
5034	0.097	-1.181	0.492	0.401	0.737	0.235	0.329	0.301	0.450
5035	0.224	-0.373	0.508	0.339	0.342	0.235	0.142	-0.895	0.050
5036	0.229	-0.339	0.492	0.193	-0.600	0.398	0.142	-0.895	0.431
5037	0.300	0.116	0.185	0.307	0.135	0.102	0.097	-1.181	0.142
5038	0.505	1.430	0.385	0.442	1.001	0.193	0.500	1.397	0.097
5039	0.505	1.430	0.262	0.442	1.001	0.273	0.500	1.397	0.508
5040	0.142	-0.895	0.224	0.102	-1.184	0.309	0.300	0.116	0.481
5041	0.358	0.487	0.142	0.234	-0.336	0.309	0.050	-1.485	0.513
5042	0.297	0.099	0.224	0.237	-0.317	0.193	0.184	-0.625	0.501
5043	0.261	-0.137	0.481	0.237	-0.317	0.309	0.184	-0.625	0.450
5044	0.468	1.194	0.508	0.401	0.737	0.501	0.500	1.397	0.513
5045	0.187	-0.608	0.450	0.102	-1.184	0.501	0.261	-0.137	0.492
5046	0.142	-0.895	0.262	0.193	-0.600	0.508	0.332	0.318	0.142
5047	0.000	?	0.224	0.275	-0.073	0.508	0.297	0.099	0.224
5048	0.382	0.638	0.142	0.307	0.135	0.398	0.097	-1.181	0.262
5049	0.261	-0.137	0.142	0.310	0.153	0.273	0.142	-0.895	0.492
5050	0.326	0.285	0.224	0.193	-0.600	0.235	0.097	-1.181	0.492
5051	0.184	-0.625	0.512	0.310	0.153	0.508	0.097	-1.181	0.508
5052	0.447	1.060	0.501	0.509	1.434	0.508	0.489	1.329	0.508
5053	0.429	0.942	0.224	0.509	1.434	0.508	0.489	1.329	0.508
5054	0.429	0.942	0.467	0.515	1.472	0.508	0.466	1.178	0.492
5055	0.447	1.060	0.508	0.515	1.472	0.508	0.479	1.262	0.097
5056	0.505	1.430	0.492	0.398	0.718	0.508	0.461	1.144	0.512
5057	0.184	-0.625	0.512	0.272	-0.091	0.508	0.224	-0.373	0.185
5058	0.184	-0.625	0.224	0.240	-0.299	0.371	0.224	-0.373	0.142
5059	0.492	1.346	0.467	0.500	1.378	0.235	0.513	1.481	0.142

5060	0.492	1.346	0.492	0.500	1.378	0.512	0.513	1.481	0.224
5061	0.492	1.346	0.513	0.500	1.378	0.501	0.513	1.481	0.450
5062	0.492	1.346	0.508	0.500	1.378	0.501	0.505	1.430	0.492
5063	0.492	1.346	0.508	0.500	1.378	0.398	0.513	1.481	0.467
5064	0.492	1.346	0.224	0.500	1.378	0.371	0.513	1.481	0.097
5065	0.468	1.194	0.409	0.497	1.359	0.462	0.511	1.464	0.142
5066	0.297	0.099	0.501	0.368	0.530	0.462	0.261	-0.137	0.296
5067	0.261	-0.137	0.224	0.237	-0.317	0.462	0.050	-1.485	0.224
5068	0.516	1.498	0.224	0.515	1.472	0.462	0.511	1.464	0.097
5069	0.482	1.279	0.224	0.503	1.396	0.444	0.505	1.430	0.492
5070	0.482	1.279	0.481	0.503	1.396	0.398	0.505	1.430	0.513
5071	0.500	1.397	0.185	0.401	0.737	0.508	0.513	1.481	0.262
5072	0.224	-0.373	0.385	0.374	0.568	0.102	0.295	0.082	0.492
5073	0.505	1.430	0.385	0.468	1.170	0.478	0.463	1.161	0.512
5074	0.505	1.430	0.262	0.468	1.170	0.508	0.479	1.262	0.492
5075	0.505	1.430	0.262	0.468	1.170	0.422	0.463	1.161	0.492
5076	0.484	1.295	0.513	0.465	1.152	0.512	0.479	1.262	0.142
5077	0.505	1.430	0.262	0.447	1.039	0.341	0.463	1.161	0.467
5078	0.505	1.430	0.481	0.404	0.756	0.514	0.476	1.245	0.492
5079	0.505	1.430	0.450	0.509	1.434	0.341	0.484	1.295	0.409
5080	0.187	-0.608	0.467	0.102	-1.184	0.235	0.263	-0.120	0.492
5081	0.497	1.380	0.512	0.474	1.208	0.508	0.468	1.194	0.492
5082	0.492	1.346	0.512	0.506	1.415	0.512	0.492	1.346	0.328
5083	0.263	-0.120	0.328	0.424	0.888	0.508	0.387	0.672	0.262
5084	0.384	0.655	0.492	0.512	1.453	0.462	0.445	1.043	0.467
5085	0.184	-0.625	0.492	0.345	0.379	0.478	0.050	-1.485	0.185
5086	0.489	1.329	0.185	0.515	1.472	0.508	0.426	0.925	0.185
5087	0.224	-0.373	0.501	0.348	0.398	0.462	0.142	-0.895	0.185
5088	0.297	0.099	0.481	0.234	-0.336	0.309	0.100	-1.164	0.492
5089	0.497	1.380	0.513	0.503	1.396	0.149	0.508	1.447	0.142
5090	0.503	1.413	0.501	0.518	1.491	0.149	0.476	1.245	0.508
5091	0.487	1.312	0.409	0.512	1.453	0.491	0.511	1.464	0.508
5092	0.484	1.295	0.224	0.462	1.133	0.508	0.500	1.397	0.431
5093	0.447	1.060	0.296	0.480	1.246	0.501	0.447	1.060	0.385
5094	0.492	1.346	0.512	0.509	1.434	0.501	0.513	1.481	0.431
5095	0.492	1.346	0.467	0.462	1.133	0.491	0.487	1.312	0.513
5096	0.184	-0.625	0.501	0.313	0.172	0.491	0.142	-0.895	0.512
5097	0.187	-0.608	0.050	0.149	-0.882	0.309	0.226	-0.356	0.513
5098	0.187	-0.608	0.513	0.149	-0.882	0.508	0.263	-0.120	0.409
5099	0.405	0.790	0.224	0.488	1.302	0.102	0.487	1.312	0.481
5100	0.495	1.363	0.358	0.509	1.434	0.235	0.492	1.346	0.097
5101	0.505	1.430	0.358	0.497	1.359	0.309	0.508	1.447	0.492
5102	0.489	1.329	0.512	0.503	1.396	0.149	0.511	1.464	0.492
5103	0.503	1.413	0.501	0.491	1.321	0.491	0.503	1.413	0.097
5104	0.489	1.329	0.513	0.491	1.321	0.508	0.492	1.346	0.467
5105	0.184	-0.625	0.142	0.307	0.135	0.501	0.097	-1.181	0.467
5106	0.384	0.655	0.142	0.509	1.434	0.235	0.508	1.447	0.501
5107	0.261	-0.137	0.508	0.102	-1.184	0.235	0.184	-0.625	0.501
5108	0.224	-0.373	0.501	0.234	-0.336	0.273	0.145	-0.878	0.481
5109	0.142	-0.895	0.224	0.313	0.172	0.193	0.142	-0.895	0.142
5110	0.224	-0.373	0.431	0.152	-0.863	0.341	0.226	-0.356	0.050
5111	0.484	1.295	0.492	0.491	1.321	0.422	0.447	1.060	0.501
5112	0.500	1.397	0.512	0.503	1.396	0.501	0.495	1.363	0.481
5113	0.453	1.093	0.450	0.500	1.378	0.309	0.463	1.161	0.385
5114	0.266	-0.103	0.508	0.234	-0.336	0.422	0.097	-1.181	0.409
5115	0.224	-0.373	0.142	0.234	-0.336	0.398	0.142	-0.895	0.481
5116	0.142	-0.895	0.512	0.278	-0.054	0.491	0.297	0.099	0.224
5117	0.142	-0.895	0.508	0.196	-0.581	0.491	0.226	-0.356	0.142
5118	0.224	-0.373	0.050	0.342	0.361	0.341	0.097	-1.181	0.142
5119	0.511	1.464	0.501	0.424	0.888	0.422	0.489	1.329	0.431
5120	0.500	1.397	0.501	0.500	1.378	0.508	0.516	1.498	0.508

5121	0.224	-0.373	0.097	0.310	0.153	0.512	0.266	-0.103	0.501
5122	0.463	1.161	0.492	0.424	0.888	0.512	0.497	1.380	0.262
5123	0.503	1.413	0.492	0.398	0.718	0.508	0.513	1.481	0.262
5124	0.489	1.329	0.142	0.485	1.283	0.309	0.495	1.363	0.481
5125	0.511	1.464	0.492	0.485	1.283	0.371	0.495	1.363	0.501
5126	0.226	-0.356	0.492	0.342	0.361	0.422	0.142	-0.895	0.296
5127	0.471	1.211	0.097	0.424	0.888	0.193	0.466	1.178	0.492
5128	0.492	1.346	0.481	0.512	1.453	0.149	0.492	1.346	0.481
5129	0.508	1.447	0.050	0.512	1.453	0.149	0.405	0.790	0.481
5130	0.500	1.397	0.508	0.506	1.415	0.462	0.497	1.380	0.501
5131	0.500	1.397	0.050	0.503	1.396	0.235	0.497	1.380	0.492
5132	0.226	-0.356	0.050	0.310	0.153	0.512	0.326	0.285	0.097
5133	0.408	0.807	0.512	0.368	0.530	0.512	0.261	-0.137	0.467
5134	0.500	1.397	0.513	0.418	0.850	0.273	0.463	1.161	0.513
5135	0.226	-0.356	0.513	0.193	-0.600	0.193	0.187	-0.608	0.431
5136	0.226	-0.356	0.512	0.149	-0.882	0.444	0.187	-0.608	0.481
5137	0.226	-0.356	0.501	0.149	-0.882	0.193	0.187	-0.608	0.431
5138	0.482	1.279	0.512	0.459	1.114	0.512	0.489	1.329	0.262
5139	0.187	-0.608	0.097	0.237	-0.317	0.514	0.142	-0.895	0.450
5140	0.389	0.689	0.097	0.512	1.453	0.514	0.500	1.397	0.431
5141	0.389	0.689	0.481	0.512	1.453	0.512	0.500	1.397	0.328
5142	0.261	-0.137	0.492	0.275	-0.073	0.478	0.000	?	0.328
5143	0.263	-0.120	0.328	0.196	-0.581	0.273	0.429	0.942	0.328
5144	0.518	1.514	0.508	0.444	1.020	0.508	0.384	0.655	0.328
5145	0.263	-0.120	0.512	0.196	-0.581	0.508	0.429	0.942	0.224
5146	0.476	1.245	0.512	0.512	1.453	0.273	0.513	1.481	0.492
5147	0.450	1.076	0.513	0.515	1.472	0.422	0.511	1.464	0.492
5148	0.463	1.161	0.492	0.515	1.472	0.501	0.511	1.464	0.050
5149	0.500	1.397	0.142	0.520	1.509	0.478	0.411	0.824	0.481
5150	0.500	1.397	0.501	0.480	1.246	0.508	0.482	1.279	0.385
5151	0.329	0.301	0.501	0.272	-0.091	0.508	0.097	-1.181	0.501
5152	0.489	1.329	0.512	0.506	1.415	0.341	0.492	1.346	0.142
5153	0.489	1.329	0.508	0.506	1.415	0.193	0.492	1.346	0.262
5154	0.184	-0.625	0.262	0.272	-0.091	0.444	0.100	-1.164	0.467
5155	0.503	1.413	0.097	0.421	0.869	0.508	0.461	1.144	0.512
5156	0.482	1.279	0.513	0.500	1.378	0.514	0.461	1.144	0.512
5157	0.513	1.481	0.513	0.477	1.227	0.102	0.492	1.346	0.513
5158	0.503	1.413	0.513	0.509	1.434	0.501	0.492	1.346	0.512
5159	0.411	0.824	0.508	0.506	1.415	0.273	0.474	1.228	0.508
5160	0.224	-0.373	0.097	0.339	0.342	0.235	0.142	-0.895	0.142
5161	0.297	0.099	0.512	0.196	-0.581	0.235	0.050	-1.485	0.185
5162	0.513	1.481	0.508	0.442	1.001	0.501	0.500	1.397	0.501
5163	0.461	1.144	0.508	0.509	1.434	0.462	0.476	1.245	0.481
5164	0.497	1.380	0.097	0.506	1.415	0.462	0.384	0.655	0.050
5165	0.050	-1.485	0.508	0.102	-1.184	0.273	0.408	0.807	0.513
5166	0.513	1.481	0.492	0.503	1.396	0.273	0.482	1.279	0.513
5167	0.226	-0.356	0.492	0.278	-0.054	0.491	0.224	-0.373	0.467
5168	0.358	0.487	0.512	0.237	-0.317	0.444	0.142	-0.895	0.481
5169	0.358	0.487	0.512	0.237	-0.317	0.235	0.142	-0.895	0.481
5170	0.511	1.464	0.508	0.500	1.378	0.491	0.429	0.942	0.185
5171	0.503	1.413	0.501	0.459	1.114	0.478	0.503	1.413	0.512
5172	0.516	1.498	0.097	0.465	1.152	0.501	0.495	1.363	0.512
5173	0.142	-0.895	0.450	0.275	-0.073	0.514	0.263	-0.120	0.513
5174	0.142	-0.895	0.501	0.275	-0.073	0.508	0.263	-0.120	0.512
5175	0.508	1.447	0.508	0.491	1.321	0.371	0.476	1.245	0.185
5176	0.503	1.413	0.142	0.444	1.020	0.462	0.500	1.397	0.224
5177	0.224	-0.373	0.513	0.234	-0.336	0.444	0.297	0.099	0.508
5178	0.429	0.942	0.501	0.494	1.340	0.235	0.489	1.329	0.492
5179	0.495	1.363	0.501	0.477	1.227	0.462	0.476	1.245	0.492
5180	0.511	1.464	0.050	0.500	1.378	0.501	0.476	1.245	0.501
5181	0.453	1.093	0.097	0.512	1.453	0.193	0.495	1.363	0.296

5182	0.497	1.380	0.097	0.515	1.472	0.491	0.487	1.312	0.512
5183	0.142	-0.895	0.512	0.371	0.549	0.491	0.097	-1.181	0.185
5184	0.500	1.397	0.431	0.468	1.170	0.235	0.463	1.161	0.513
5185	0.500	1.397	0.262	0.444	1.020	0.235	0.511	1.464	0.513
5186	0.050	-1.485	0.467	0.234	-0.336	0.235	0.429	0.942	0.142
5187	0.492	1.346	0.512	0.465	1.152	0.235	0.479	1.262	0.481
5188	0.495	1.363	0.185	0.500	1.378	0.273	0.432	0.958	0.409
5189	0.097	-1.181	0.262	0.193	-0.600	0.462	0.263	-0.120	0.409
5190	0.484	1.295	0.185	0.488	1.302	0.462	0.450	1.076	0.512
5191	0.484	1.295	0.481	0.488	1.302	0.371	0.429	0.942	0.450
5192	0.000	?	0.296	0.234	-0.336	0.512	0.329	0.301	0.512
5193	0.000	?	0.492	0.234	-0.336	0.235	0.329	0.301	0.467
5194	0.000	?	0.185	0.234	-0.336	0.478	0.329	0.301	0.328
5195	0.000	?	0.185	0.234	-0.336	0.422	0.329	0.301	0.512
5196	0.142	-0.895	0.185	0.278	-0.054	0.371	0.224	-0.373	0.492
5197	0.495	1.363	0.185	0.459	1.114	0.462	0.487	1.312	0.512
5198	0.495	1.363	0.142	0.459	1.114	0.491	0.487	1.312	0.185
5199	0.097	-1.181	0.481	0.374	0.568	0.501	0.050	-1.485	0.501
5200	0.474	1.228	0.224	0.512	1.453	0.398	0.482	1.279	0.409
5201	0.050	-1.485	0.185	0.237	-0.317	0.398	0.387	0.672	0.409
5202	0.500	1.397	0.481	0.480	1.246	0.371	0.500	1.397	0.328
5203	0.050	-1.485	0.185	0.424	0.888	0.309	0.142	-0.895	0.296
5204	0.050	-1.485	0.262	0.371	0.549	0.309	0.263	-0.120	0.185
5205	0.500	1.397	0.224	0.468	1.170	0.508	0.466	1.178	0.501
5206	0.513	1.481	0.450	0.491	1.321	0.462	0.505	1.430	0.501
5207	0.513	1.481	0.358	0.500	1.378	0.149	0.505	1.430	0.185
5208	0.500	1.397	0.358	0.404	0.756	0.478	0.511	1.464	0.492
5209	0.500	1.397	0.296	0.398	0.718	0.462	0.518	1.514	0.512
5210	0.505	1.430	0.467	0.371	0.549	0.508	0.511	1.464	0.185
5211	0.097	-1.181	0.224	0.313	0.172	0.512	0.142	-0.895	0.097
5212	0.097	-1.181	0.513	0.313	0.172	0.491	0.184	-0.625	0.097
5213	0.479	1.262	0.467	0.512	1.453	0.235	0.500	1.397	0.508
5214	0.497	1.380	0.262	0.468	1.170	0.462	0.479	1.262	0.512
5215	0.329	0.301	0.508	0.149	-0.882	0.462	0.050	-1.485	0.501
5216	0.508	1.447	0.481	0.480	1.246	0.491	0.513	1.481	0.050
5217	0.511	1.464	0.296	0.465	1.152	0.491	0.513	1.481	0.050
5218	0.511	1.464	0.328	0.503	1.396	0.102	0.466	1.178	0.050
5219	0.513	1.481	0.296	0.509	1.434	0.235	0.479	1.262	0.224
5220	0.489	1.329	0.262	0.488	1.302	0.478	0.479	1.262	0.262
5221	0.142	-0.895	0.224	0.237	-0.317	0.462	0.184	-0.625	0.501
5222	0.495	1.363	0.224	0.462	1.133	0.462	0.508	1.447	0.050
5223	0.495	1.363	0.224	0.462	1.133	0.462	0.508	1.447	0.050
5224	0.511	1.464	0.262	0.491	1.321	0.398	0.513	1.481	0.501
5225	0.505	1.430	0.431	0.491	1.321	0.514	0.511	1.464	0.185
5226	0.266	-0.103	0.358	0.102	-1.184	0.341	0.184	-0.625	0.142
5227	0.097	-1.181	0.358	0.237	-0.317	0.491	0.226	-0.356	0.050
5228	0.513	1.481	0.224	0.477	1.227	0.491	0.511	1.464	0.512
5229	0.513	1.481	0.224	0.462	1.133	0.371	0.495	1.363	0.142
5230	0.513	1.481	0.467	0.462	1.133	0.444	0.495	1.363	0.050
5231	0.497	1.380	0.501	0.462	1.133	0.501	0.503	1.413	0.508
5232	0.097	-1.181	0.224	0.398	0.718	0.508	0.295	0.082	0.142
5233	0.500	1.397	0.409	0.520	1.509	0.422	0.511	1.464	0.501
5234	0.000	?	0.296	0.339	0.342	0.508	0.184	-0.625	0.492
5235	0.497	1.380	0.224	0.494	1.340	0.508	0.513	1.481	0.097
5236	0.497	1.380	0.262	0.494	1.340	0.514	0.513	1.481	0.508
5237	0.097	-1.181	0.358	0.371	0.549	0.235	0.145	-0.878	0.501
5238	0.497	1.380	0.501	0.447	1.039	0.462	0.484	1.295	0.097
5239	0.484	1.295	0.467	0.509	1.434	0.478	0.408	0.807	0.142
5240	0.484	1.295	0.431	0.518	1.491	0.422	0.408	0.807	0.142
5241	0.503	1.413	0.385	0.424	0.888	0.371	0.511	1.464	0.050
5242	0.503	1.413	0.481	0.512	1.453	0.422	0.445	1.043	0.142

5243	0.500	1.397	0.409	0.512	1.453	0.508	0.511	1.464	0.142
5244	0.495	1.363	0.224	0.518	1.491	0.508	0.463	1.161	0.142
5245	0.097	-1.181	0.501	0.237	-0.317	0.444	0.329	0.301	0.501
5246	0.445	1.043	0.224	0.462	1.133	0.422	0.511	1.464	0.492
5247	0.500	1.397	0.262	0.480	1.246	0.273	0.495	1.363	0.050
5248	0.500	1.397	0.296	0.427	0.907	0.491	0.508	1.447	0.097
5249	0.142	-0.895	0.224	0.377	0.587	0.512	0.184	-0.625	0.262
5250	0.516	1.498	0.142	0.418	0.850	0.235	0.492	1.346	0.512
5251	0.495	1.363	0.431	0.503	1.396	0.491	0.408	0.807	0.450
5252	0.495	1.363	0.097	0.503	1.396	0.501	0.408	0.807	0.097
5253	0.050	-1.485	0.050	0.444	1.020	0.193	0.329	0.301	0.050
5254	0.097	-1.181	0.467	0.424	0.888	0.273	0.297	0.099	0.050
5255	0.100	-1.164	0.385	0.275	-0.073	0.273	0.184	-0.625	0.142
5256	0.511	1.464	0.262	0.485	1.283	0.512	0.497	1.380	0.097
5257	0.429	0.942	0.508	0.512	1.453	0.508	0.495	1.363	0.097
5258	0.261	-0.137	0.481	0.237	-0.317	0.462	0.184	-0.625	0.481
5259	0.474	1.228	0.097	0.491	1.321	0.309	0.484	1.295	0.513
5260	0.516	1.498	0.513	0.503	1.396	0.309	0.503	1.413	0.508
5261	0.184	-0.625	0.185	0.193	-0.600	0.309	0.184	-0.625	0.097
5262	0.261	-0.137	0.142	0.275	-0.073	0.235	0.097	-1.181	0.450
5263	0.187	-0.608	0.262	0.275	-0.073	0.149	0.097	-1.181	0.501
5264	0.479	1.262	0.481	0.512	1.453	0.478	0.500	1.397	0.142
5265	0.295	0.082	0.467	0.512	1.453	0.273	0.505	1.430	0.492
5266	0.492	1.346	0.224	0.462	1.133	0.309	0.492	1.346	0.185
5267	0.184	-0.625	0.224	0.310	0.153	0.512	0.050	-1.485	0.142
5268	0.184	-0.625	0.224	0.310	0.153	0.193	0.050	-1.485	0.142
5269	0.184	-0.625	0.467	0.310	0.153	0.149	0.050	-1.485	0.050
5270	0.187	-0.608	0.224	0.234	-0.336	0.273	0.224	-0.373	0.467
5271	0.145	-0.878	0.492	0.149	-0.882	0.462	0.261	-0.137	0.224
5272	0.482	1.279	0.481	0.477	1.227	0.235	0.497	1.380	0.262
5273	0.224	-0.373	0.185	0.272	-0.091	0.149	0.050	-1.485	0.512
5274	0.187	-0.608	0.431	0.307	0.135	0.235	0.050	-1.485	0.097
5275	0.479	1.262	0.358	0.515	1.472	0.491	0.492	1.346	0.050
5276	0.187	-0.608	0.262	0.196	-0.581	0.193	0.184	-0.625	0.512
5277	0.263	-0.120	0.508	0.149	-0.882	0.422	0.142	-0.895	0.467
5278	0.226	-0.356	0.262	0.272	-0.091	0.514	0.050	-1.485	0.185
5279	0.450	1.076	0.492	0.462	1.133	0.273	0.505	1.430	0.492
5280	0.361	0.504	0.431	0.234	-0.336	0.491	0.142	-0.895	0.097
5281	0.361	0.504	0.185	0.149	-0.882	0.398	0.050	-1.485	0.224
5282	0.297	0.099	0.492	0.237	-0.317	0.149	0.000	?	0.050
5283	0.466	1.178	0.481	0.494	1.340	0.149	0.497	1.380	0.492
5284	0.224	-0.373	0.481	0.196	-0.581	0.149	0.142	-0.895	0.508
5285	0.508	1.447	0.481	0.427	0.907	0.235	0.492	1.346	0.185
5286	0.466	1.178	0.296	0.515	1.472	0.235	0.484	1.295	0.142
5287	0.261	-0.137	0.224	0.272	-0.091	0.309	0.097	-1.181	0.097
5288	0.513	1.481	0.185	0.485	1.283	0.235	0.497	1.380	0.467
5289	0.482	1.279	0.328	0.395	0.700	0.371	0.500	1.397	0.097
5290	0.297	0.099	0.296	0.149	-0.882	0.512	0.097	-1.181	0.508
5291	0.326	0.285	0.296	0.149	-0.882	0.491	0.142	-0.895	0.492
5292	0.295	0.082	0.296	0.149	-0.882	0.193	0.142	-0.895	0.185
5293	0.263	-0.120	0.262	0.237	-0.317	0.235	0.050	-1.485	0.142
5294	0.224	-0.373	0.358	0.234	-0.336	0.149	0.142	-0.895	0.142
5295	0.224	-0.373	0.508	0.310	0.153	0.491	0.142	-0.895	0.050
5296	0.224	-0.373	0.224	0.234	-0.336	0.512	0.142	-0.895	0.450
5297	0.263	-0.120	0.328	0.371	0.549	0.235	0.000	?	0.142
5298	0.429	0.942	0.328	0.512	1.453	0.193	0.495	1.363	0.492
5299	0.358	0.487	0.296	0.491	1.321	0.193	0.484	1.295	0.492
5300	0.358	0.487	0.450	0.193	-0.600	0.193	0.050	-1.485	0.050
5301	0.226	-0.356	0.142	0.234	-0.336	0.193	0.097	-1.181	0.492
5302	0.224	-0.373	0.512	0.152	-0.863	0.149	0.263	-0.120	0.501
5303	0.466	1.178	0.513	0.491	1.321	0.478	0.503	1.413	0.492

5304	0.503	1.413	0.328	0.512	1.453	0.444	0.445	1.043	0.501
5305	0.224	-0.373	0.512	0.234	-0.336	0.501	0.097	-1.181	0.224
5306	0.405	0.790	0.512	0.193	-0.600	0.235	0.050	-1.485	0.142
5307	0.297	0.099	0.481	0.196	-0.581	0.491	0.050	-1.485	0.142
5308	0.224	-0.373	0.492	0.196	-0.581	0.491	0.142	-0.895	0.097
5309	0.263	-0.120	0.224	0.193	-0.600	0.309	0.097	-1.181	0.185
5310	0.355	0.470	0.296	0.149	-0.882	0.514	0.097	-1.181	0.097
5311	0.497	1.380	0.508	0.477	1.227	0.309	0.479	1.262	0.097
5312	0.468	1.194	0.481	0.444	1.020	0.235	0.503	1.413	0.097
5313	0.429	0.942	0.501	0.503	1.396	0.235	0.497	1.380	0.224
5314	0.387	0.672	0.262	0.234	-0.336	0.235	0.097	-1.181	0.467
5315	0.482	1.279	0.097	0.491	1.321	0.341	0.445	1.043	0.224
5316	0.408	0.807	0.296	0.494	1.340	0.501	0.495	1.363	0.142
5317	0.224	-0.373	0.262	0.310	0.153	0.235	0.142	-0.895	0.142
5318	0.497	1.380	0.328	0.515	1.472	0.341	0.487	1.312	0.142
5319	0.224	-0.373	0.508	0.310	0.153	0.491	0.000	?	0.508
5320	0.261	-0.137	0.097	0.237	-0.317	0.309	0.184	-0.625	0.185
5321	0.295	0.082	0.513	0.234	-0.336	0.273	0.145	-0.878	0.508
5322	0.224	-0.373	0.508	0.234	-0.336	0.491	0.142	-0.895	0.501
5323	0.142	-0.895	0.224	0.345	0.379	0.462	0.050	-1.485	0.097
5324	0.429	0.942	0.224	0.497	1.359	0.273	0.463	1.161	0.467
5325	0.097	-1.181	0.512	0.237	-0.317	0.491	0.224	-0.373	0.431
5326	0.050	-1.485	0.501	0.342	0.361	0.273	0.263	-0.120	0.467
5327	0.468	1.194	0.185	0.491	1.321	0.235	0.505	1.430	0.450
5328	0.384	0.655	0.481	0.313	0.172	0.273	0.097	-1.181	0.050
5329	0.261	-0.137	0.450	0.275	-0.073	0.444	0.050	-1.485	0.050
5330	0.503	1.413	0.492	0.494	1.340	0.444	0.505	1.430	0.481
5331	0.476	1.245	0.385	0.465	1.152	0.149	0.461	1.144	0.492
5332	0.097	-1.181	0.508	0.272	-0.091	0.193	0.184	-0.625	0.467
5333	0.511	1.464	0.224	0.488	1.302	0.235	0.489	1.329	0.328
5334	0.184	-0.625	0.513	0.278	-0.054	0.501	0.097	-1.181	0.328
5335	0.142	-0.895	0.431	0.237	-0.317	0.235	0.224	-0.373	0.296
5336	0.261	-0.137	0.501	0.275	-0.073	0.444	0.050	-1.485	0.224
5337	0.482	1.279	0.224	0.442	1.001	0.462	0.489	1.329	0.142
5338	0.463	1.161	0.431	0.444	1.020	0.309	0.508	1.447	0.409
5339	0.226	-0.356	0.492	0.149	-0.882	0.149	0.184	-0.625	0.185
5340	0.226	-0.356	0.262	0.196	-0.581	0.193	0.142	-0.895	0.450
5341	0.224	-0.373	0.450	0.234	-0.336	0.273	0.097	-1.181	0.501
5342	0.466	1.178	0.050	0.497	1.359	0.444	0.461	1.144	0.097
5343	0.226	-0.356	0.224	0.237	-0.317	0.371	0.097	-1.181	0.328
5344	0.489	1.329	0.296	0.439	0.982	0.462	0.505	1.430	0.450
5345	0.479	1.262	0.492	0.462	1.133	0.422	0.487	1.312	0.512
5346	0.184	-0.625	0.467	0.307	0.135	0.309	0.184	-0.625	0.328
5347	0.429	0.942	0.481	0.149	-0.882	0.514	0.142	-0.895	0.512
5348	0.358	0.487	0.142	0.196	-0.581	0.478	0.142	-0.895	0.501
5349	0.261	-0.137	0.097	0.272	-0.091	0.444	0.050	-1.485	0.467
5350	0.511	1.464	0.492	0.447	1.039	0.501	0.445	1.043	0.224
5351	0.261	-0.137	0.409	0.374	0.568	0.102	0.142	-0.895	0.481
5352	0.492	1.346	0.142	0.462	1.133	0.193	0.484	1.295	0.142
5353	0.434	0.975	0.508	0.424	0.888	0.371	0.484	1.295	0.450
5354	0.184	-0.625	0.358	0.313	0.172	0.235	0.050	-1.485	0.467
5355	0.492	1.346	0.142	0.512	1.453	0.193	0.487	1.312	0.481
5356	0.484	1.295	0.409	0.471	1.189	0.149	0.495	1.363	0.328
5357	0.484	1.295	0.431	0.439	0.982	0.149	0.487	1.312	0.409
5358	0.479	1.262	0.431	0.500	1.378	0.235	0.495	1.363	0.512
5359	0.295	0.082	0.512	0.102	-1.184	0.193	0.229	-0.339	0.185
5360	0.224	-0.373	0.481	0.196	-0.581	0.512	0.145	-0.878	0.501
5361	0.184	-0.625	0.224	0.371	0.549	0.235	0.142	-0.895	0.224
5362	0.329	0.301	0.185	0.234	-0.336	0.235	0.097	-1.181	0.142
5363	0.295	0.082	0.185	0.193	-0.600	0.235	0.187	-0.608	0.296
5364	0.297	0.099	0.224	0.149	-0.882	0.341	0.097	-1.181	0.467

5365	0.297	0.099	0.296	0.149	-0.882	0.491	0.097	-1.181	0.296
5366	0.263	-0.120	0.224	0.234	-0.336	0.273	0.097	-1.181	0.512
5367	0.363	0.520	0.508	0.193	-0.600	0.444	0.224	-0.373	0.328
5368	0.513	1.481	0.508	0.512	1.453	0.444	0.463	1.161	0.262
5369	0.224	-0.373	0.142	0.234	-0.336	0.149	0.224	-0.373	0.467
5370	0.329	0.301	0.513	0.234	-0.336	0.478	0.142	-0.895	0.185
5371	0.329	0.301	0.358	0.234	-0.336	0.501	0.142	-0.895	0.296
5372	0.295	0.082	0.185	0.345	0.379	0.501	0.142	-0.895	0.481
5373	0.447	1.060	0.467	0.500	1.378	0.491	0.500	1.397	0.409
5374	0.142	-0.895	0.142	0.278	-0.054	0.273	0.184	-0.625	0.262
5375	0.516	1.498	0.142	0.444	1.020	0.193	0.500	1.397	0.508
5376	0.516	1.498	0.262	0.444	1.020	0.422	0.495	1.363	0.508
5377	0.332	0.318	0.508	0.149	-0.882	0.398	0.097	-1.181	0.512
5378	0.505	1.430	0.508	0.480	1.246	0.508	0.463	1.161	0.431
5379	0.505	1.430	0.185	0.503	1.396	0.444	0.429	0.942	0.501
5380	0.484	1.295	0.481	0.500	1.378	0.235	0.461	1.144	0.185
5381	0.495	1.363	0.358	0.485	1.283	0.053	0.445	1.043	0.097
5382	0.226	-0.356	0.501	0.272	-0.091	0.193	0.050	-1.485	0.224
5383	0.295	0.082	0.508	0.193	-0.600	0.491	0.050	-1.485	0.185
5384	0.511	1.464	0.262	0.418	0.850	0.273	0.474	1.228	0.142
5385	0.476	1.245	0.142	0.398	0.718	0.462	0.489	1.329	0.097
5386	0.503	1.413	0.501	0.509	1.434	0.512	0.461	1.144	0.450
5387	0.261	-0.137	0.262	0.447	1.039	0.273	0.326	0.285	0.512
5388	0.097	-1.181	0.296	0.234	-0.336	0.371	0.329	0.301	0.142
5389	0.297	0.099	0.296	0.000	?	0.514	0.295	0.082	0.501
5390	0.263	-0.120	0.508	0.053	-1.504	0.422	0.226	-0.356	0.097
5391	0.332	0.318	0.508	0.193	-0.600	0.273	0.142	-0.895	0.224
5392	0.505	1.430	0.492	0.488	1.302	0.491	0.405	0.790	0.467
5393	0.097	-1.181	0.508	0.275	-0.073	0.512	0.184	-0.625	0.328
5394	0.511	1.464	0.296	0.459	1.114	0.422	0.447	1.060	0.385
5395	0.503	1.413	0.512	0.512	1.453	0.371	0.500	1.397	0.450
5396	0.224	-0.373	0.512	0.278	-0.054	0.514	0.097	-1.181	0.481
5397	0.224	-0.373	0.328	0.371	0.549	0.309	0.326	0.285	0.097
5398	0.511	1.464	0.512	0.512	1.453	0.491	0.447	1.060	0.512
5399	0.503	1.413	0.262	0.421	0.869	0.508	0.511	1.464	0.262
5400	0.184	-0.625	0.097	0.275	-0.073	0.371	0.329	0.301	0.431
5401	0.484	1.295	0.492	0.491	1.321	0.193	0.511	1.464	0.508
5402	0.450	1.076	0.467	0.518	1.491	0.508	0.503	1.413	0.097
5403	0.495	1.363	0.492	0.424	0.888	0.491	0.466	1.178	0.501
5404	0.387	0.672	0.185	0.371	0.549	0.102	0.224	-0.373	0.050
5405	0.508	1.447	0.185	0.515	1.472	0.508	0.484	1.295	0.050
5406	0.224	-0.373	0.050	0.310	0.153	0.273	0.142	-0.895	0.050
5407	0.508	1.447	0.224	0.491	1.321	0.193	0.447	1.060	0.501
5408	0.432	0.958	0.481	0.515	1.472	0.273	0.466	1.178	0.513
5409	0.503	1.413	0.296	0.368	0.530	0.478	0.482	1.279	0.481
5410	0.224	-0.373	0.185	0.193	-0.600	0.501	0.329	0.301	0.467
5411	0.429	0.942	0.142	0.509	1.434	0.508	0.411	0.824	0.142
5412	0.495	1.363	0.185	0.494	1.340	0.053	0.508	1.447	0.467
5413	0.261	-0.137	0.296	0.102	-1.184	0.193	0.184	-0.625	0.481
5414	0.447	1.060	0.481	0.512	1.453	0.512	0.503	1.413	0.501
5415	0.050	-1.485	0.450	0.275	-0.073	0.149	0.226	-0.356	0.097
5416	0.224	-0.373	0.512	0.193	-0.600	0.235	0.142	-0.895	0.492
5417	0.297	0.099	0.385	0.275	-0.073	0.478	0.297	0.099	0.185
5418	0.487	1.312	0.296	0.477	1.227	0.193	0.471	1.211	0.185
5419	0.471	1.211	0.481	0.500	1.378	0.193	0.297	0.099	0.185
5420	0.476	1.245	0.224	0.518	1.491	0.491	0.516	1.498	0.508
5421	0.142	-0.895	0.185	0.053	-1.504	0.491	0.332	0.318	0.296
5422	0.097	-1.181	0.508	0.193	-0.600	0.512	0.261	-0.137	0.467
5423	0.487	1.312	0.508	0.515	1.472	0.398	0.468	1.194	0.467
5424	0.411	0.824	0.501	0.152	-0.863	0.512	0.184	-0.625	0.050
5425	0.145	-0.878	0.142	0.234	-0.336	0.149	0.295	0.082	0.262

5426	0.503	1.413	0.262	0.480	1.246	0.309	0.476	1.245	0.262
5427	0.358	0.487	0.501	0.193	-0.600	0.193	0.411	0.824	0.142
5428	0.142	-0.895	0.467	0.196	-0.581	0.235	0.261	-0.137	0.501
5429	0.408	0.807	0.450	0.488	1.302	0.273	0.508	1.447	0.097
5430	0.429	0.942	0.296	0.491	1.321	0.235	0.511	1.464	0.050
5431	0.429	0.942	0.224	0.512	1.453	0.508	0.513	1.481	0.142
5432	0.516	1.498	0.262	0.398	0.718	0.444	0.432	0.958	0.185
5433	0.479	1.262	0.185	0.512	1.453	0.273	0.495	1.363	0.142
5434	0.226	-0.356	0.262	0.152	-0.863	0.444	0.184	-0.625	0.492
5435	0.184	-0.625	0.185	0.310	0.153	0.235	0.100	-1.164	0.508
5436	0.184	-0.625	0.185	0.193	-0.600	0.193	0.224	-0.373	0.450
5437	0.224	-0.373	0.492	0.234	-0.336	0.422	0.184	-0.625	0.050
5438	0.295	0.082	0.450	0.272	-0.091	0.371	0.142	-0.895	0.142
5439	0.224	-0.373	0.358	0.234	-0.336	0.422	0.097	-1.181	0.492
5440	0.508	1.447	0.358	0.512	1.453	0.371	0.453	1.093	0.142
5441	0.505	1.430	0.492	0.442	1.001	0.501	0.508	1.447	0.097
5442	0.142	-0.895	0.501	0.272	-0.091	0.501	0.142	-0.895	0.501
5443	0.516	1.498	0.512	0.444	1.020	0.309	0.505	1.430	0.467
5444	0.361	0.504	0.508	0.234	-0.336	0.491	0.097	-1.181	0.508
5445	0.184	-0.625	0.492	0.193	-0.600	0.149	0.224	-0.373	0.224
5446	0.471	1.211	0.508	0.427	0.907	0.512	0.463	1.161	0.097
5447	0.142	-0.895	0.512	0.374	0.568	0.462	0.000	?	0.512
5448	0.142	-0.895	0.492	0.424	0.888	0.309	0.329	0.301	0.501
5449	0.263	-0.120	0.512	0.371	0.549	0.341	0.387	0.672	0.467
5450	0.511	1.464	0.450	0.506	1.415	0.514	0.450	1.076	0.050
5451	0.511	1.464	0.385	0.506	1.415	0.235	0.479	1.262	0.050
5452	0.184	-0.625	0.501	0.307	0.135	0.235	0.097	-1.181	0.050
5453	0.484	1.295	0.185	0.485	1.283	0.193	0.511	1.464	0.224
5454	0.361	0.504	0.097	0.149	-0.882	0.501	0.261	-0.137	0.142
5455	0.497	1.380	0.508	0.512	1.453	0.478	0.432	0.958	0.097
5456	0.505	1.430	0.185	0.459	1.114	0.491	0.508	1.447	0.097
5457	0.261	-0.137	0.142	0.310	0.153	0.491	0.000	?	0.508
5458	0.142	-0.895	0.050	0.339	0.342	0.102	0.100	-1.164	0.501
5459	0.503	1.413	0.513	0.506	1.415	0.149	0.500	1.397	0.142
5460	0.263	-0.120	0.513	0.234	-0.336	0.508	0.050	-1.485	0.481
5461	0.297	0.099	0.185	0.234	-0.336	0.478	0.050	-1.485	0.492
5462	0.295	0.082	0.097	0.196	-0.581	0.102	0.050	-1.485	0.508
5463	0.508	1.447	0.492	0.500	1.378	0.462	0.492	1.346	0.513
5464	0.511	1.464	0.508	0.474	1.208	0.149	0.505	1.430	0.481
5465	0.495	1.363	0.492	0.485	1.283	0.273	0.474	1.228	0.296
5466	0.497	1.380	0.508	0.497	1.359	0.371	0.466	1.178	0.492
5467	0.297	0.099	0.224	0.102	-1.184	0.514	0.142	-0.895	0.481
5468	0.000	?	0.224	0.149	-0.882	0.235	0.468	1.194	0.492
5469	0.503	1.413	0.481	0.509	1.434	0.512	0.479	1.262	0.512
5470	0.508	1.447	0.142	0.477	1.227	0.501	0.500	1.397	0.450
5471	0.326	0.285	0.508	0.102	-1.184	0.309	0.097	-1.181	0.508
5472	0.511	1.464	0.492	0.459	1.114	0.309	0.489	1.329	0.224
5473	0.261	-0.137	0.142	0.149	-0.882	0.309	0.187	-0.608	0.185
5474	0.100	-1.164	0.492	0.272	-0.091	0.193	0.184	-0.625	0.492
5475	0.000	?	0.467	0.374	0.568	0.514	0.184	-0.625	0.262
5476	0.484	1.295	0.431	0.520	1.509	0.193	0.505	1.430	0.262
5477	0.000	?	0.097	0.234	-0.336	0.341	0.300	0.116	0.262
5478	0.463	1.161	0.492	0.503	1.396	0.273	0.468	1.194	0.492
5479	0.492	1.346	0.492	0.500	1.378	0.193	0.461	1.144	0.492
5480	0.187	-0.608	0.501	0.307	0.135	0.102	0.050	-1.485	0.224
5481	0.184	-0.625	0.501	0.307	0.135	0.491	0.263	-0.120	0.262
5482	0.050	-1.485	0.512	0.310	0.153	0.478	0.261	-0.137	0.450
5483	0.226	-0.356	0.097	0.196	-0.581	0.491	0.142	-0.895	0.409
5484	0.474	1.228	0.481	0.515	1.472	0.053	0.500	1.397	0.513
5485	0.297	0.099	0.508	0.193	-0.600	0.193	0.097	-1.181	0.512
5486	0.184	-0.625	0.481	0.342	0.361	0.501	0.050	-1.485	0.185

5487	0.142	-0.895	0.501	0.275	-0.073	0.235	0.142	-0.895	0.185
5488	0.184	-0.625	0.508	0.193	-0.600	0.273	0.184	-0.625	0.501
5489	0.295	0.082	0.358	0.102	-1.184	0.491	0.142	-0.895	0.296
5490	0.482	1.279	0.492	0.491	1.321	0.501	0.484	1.295	0.501
5491	0.447	1.060	0.512	0.480	1.246	0.512	0.500	1.397	0.492
5492	0.511	1.464	0.328	0.485	1.283	0.309	0.447	1.060	0.185
5493	0.382	0.638	0.513	0.053	-1.504	0.193	0.050	-1.485	0.492
5494	0.297	0.099	0.296	0.193	-0.600	0.422	0.145	-0.878	0.513
5495	0.482	1.279	0.296	0.497	1.359	0.491	0.487	1.312	0.501
5496	0.224	-0.373	0.185	0.234	-0.336	0.508	0.142	-0.895	0.262
5497	0.187	-0.608	0.185	0.272	-0.091	0.193	0.097	-1.181	0.467
5498	0.505	1.430	0.385	0.488	1.302	0.273	0.497	1.380	0.492
5499	0.508	1.447	0.508	0.497	1.359	0.309	0.461	1.144	0.508
5500	0.505	1.430	0.508	0.506	1.415	0.149	0.500	1.397	0.492
5501	0.142	-0.895	0.512	0.307	0.135	0.193	0.229	-0.339	0.481
5502	0.261	-0.137	0.097	0.196	-0.581	0.273	0.097	-1.181	0.185
5503	0.505	1.430	0.492	0.418	0.850	0.273	0.503	1.413	0.481
5504	0.468	1.194	0.492	0.485	1.283	0.422	0.495	1.363	0.450
5505	0.453	1.093	0.224	0.506	1.415	0.462	0.461	1.144	0.431
5506	0.297	0.099	0.296	0.196	-0.581	0.193	0.050	-1.485	0.481
5507	0.226	-0.356	0.296	0.275	-0.073	0.193	0.050	-1.485	0.508
5508	0.261	-0.137	0.501	0.310	0.153	0.444	0.050	-1.485	0.513
5509	0.187	-0.608	0.492	0.149	-0.882	0.508	0.224	-0.373	0.501
5510	0.263	-0.120	0.512	0.193	-0.600	0.501	0.142	-0.895	0.097
5511	0.184	-0.625	0.262	0.272	-0.091	0.491	0.097	-1.181	0.450
5512	0.184	-0.625	0.508	0.272	-0.091	0.478	0.097	-1.181	0.097
5513	0.495	1.363	0.492	0.418	0.850	0.371	0.500	1.397	0.142
5514	0.450	1.076	0.262	0.462	1.133	0.514	0.495	1.363	0.224
5515	0.358	0.487	0.262	0.196	-0.581	0.491	0.142	-0.895	0.185
5516	0.361	0.504	0.492	0.193	-0.600	0.501	0.000	?	0.097
5517	0.492	1.346	0.492	0.442	1.001	0.478	0.476	1.245	0.492
5518	0.497	1.380	0.358	0.506	1.415	0.501	0.489	1.329	0.501
5519	0.500	1.397	0.492	0.503	1.396	0.273	0.508	1.447	0.512
5520	0.508	1.447	0.501	0.488	1.302	0.444	0.513	1.481	0.296
5521	0.489	1.329	0.512	0.477	1.227	0.149	0.482	1.279	0.481
5522	0.000	?	0.328	0.371	0.549	0.341	0.295	0.082	0.512
5523	0.497	1.380	0.492	0.512	1.453	0.514	0.495	1.363	0.097
5524	0.503	1.413	0.142	0.497	1.359	0.149	0.479	1.262	0.262
5525	0.484	1.295	0.142	0.506	1.415	0.398	0.489	1.329	0.185
5526	0.511	1.464	0.501	0.477	1.227	0.235	0.505	1.430	0.185
5527	0.447	1.060	0.501	0.497	1.359	0.444	0.447	1.060	0.467
5528	0.389	0.689	0.450	0.272	-0.091	0.444	0.000	?	0.501
5529	0.492	1.346	0.467	0.447	1.039	0.235	0.508	1.447	0.450
5530	0.187	-0.608	0.508	0.149	-0.882	0.273	0.224	-0.373	0.097
5531	0.097	-1.181	0.501	0.345	0.379	0.478	0.184	-0.625	0.431
5532	0.508	1.447	0.097	0.512	1.453	0.514	0.495	1.363	0.513
5533	0.184	-0.625	0.328	0.149	-0.882	0.462	0.261	-0.137	0.296
5534	0.142	-0.895	0.492	0.401	0.737	0.478	0.263	-0.120	0.296
5535	0.050	-1.485	0.431	0.234	-0.336	0.149	0.263	-0.120	0.501
5536	0.513	1.481	0.431	0.442	1.001	0.235	0.487	1.312	0.512
5537	0.513	1.481	0.185	0.442	1.001	0.341	0.487	1.312	0.512
5538	0.187	-0.608	0.492	0.234	-0.336	0.273	0.224	-0.373	0.512
5539	0.097	-1.181	0.492	0.275	-0.073	0.512	0.263	-0.120	0.409
5540	0.489	1.329	0.501	0.477	1.227	0.514	0.453	1.093	0.050
5541	0.500	1.397	0.501	0.518	1.491	0.273	0.405	0.790	0.492
5542	0.492	1.346	0.508	0.459	1.114	0.341	0.511	1.464	0.097
5543	0.508	1.447	0.492	0.471	1.189	0.501	0.513	1.481	0.097
5544	0.226	-0.356	0.492	0.149	-0.882	0.508	0.184	-0.625	0.501
5545	0.229	-0.339	0.492	0.234	-0.336	0.341	0.184	-0.625	0.512
5546	0.479	1.262	0.296	0.345	0.379	0.501	0.497	1.380	0.492
5547	0.142	-0.895	0.142	0.275	-0.073	0.514	0.300	0.116	0.492

5548	0.503	1.413	0.492	0.512	1.453	0.478	0.503	1.413	0.409
5549	0.492	1.346	0.142	0.509	1.434	0.508	0.487	1.312	0.512
5550	0.142	-0.895	0.467	0.272	-0.091	0.512	0.184	-0.625	0.224
5551	0.489	1.329	0.142	0.342	0.361	0.398	0.487	1.312	0.050
5552	0.463	1.161	0.501	0.500	1.378	0.491	0.516	1.498	0.508
5553	0.426	0.925	0.481	0.512	1.453	0.512	0.495	1.363	0.142
5554	0.097	-1.181	0.512	0.345	0.379	0.462	0.263	-0.120	0.142
5555	0.492	1.346	0.431	0.500	1.378	0.341	0.463	1.161	0.224
5556	0.489	1.329	0.450	0.509	1.434	0.501	0.489	1.329	0.450
5557	0.505	1.430	0.450	0.477	1.227	0.193	0.503	1.413	0.450
5558	0.497	1.380	0.185	0.506	1.415	0.514	0.492	1.346	0.512
5559	0.505	1.430	0.224	0.518	1.491	0.501	0.479	1.262	0.512
5560	0.097	-1.181	0.185	0.401	0.737	0.235	0.184	-0.625	0.512
5561	0.474	1.228	0.185	0.491	1.321	0.501	0.479	1.262	0.450
5562	0.505	1.430	0.296	0.515	1.472	0.309	0.445	1.043	0.512
5563	0.482	1.279	0.501	0.468	1.170	0.102	0.429	0.942	0.481
5564	0.497	1.380	0.224	0.339	0.342	0.422	0.474	1.228	0.185
5565	0.508	1.447	0.296	0.503	1.396	0.371	0.508	1.447	0.409
5566	0.358	0.487	0.508	0.196	-0.581	0.371	0.000	?	0.492
5567	0.492	1.346	0.296	0.512	1.453	0.514	0.513	1.481	0.358
5568	0.508	1.447	0.512	0.503	1.396	0.514	0.495	1.363	0.508
5569	0.326	0.285	0.385	0.237	-0.317	0.501	0.097	-1.181	0.262
5570	0.511	1.464	0.481	0.500	1.378	0.371	0.450	1.076	0.501
5571	0.300	0.116	0.050	0.310	0.153	0.514	0.097	-1.181	0.512
5572	0.295	0.082	0.050	0.102	-1.184	0.491	0.142	-0.895	0.409
5573	0.184	-0.625	0.050	0.424	0.888	0.371	0.224	-0.373	0.501
5574	0.184	-0.625	0.431	0.374	0.568	0.273	0.184	-0.625	0.450
5575	0.384	0.655	0.481	0.377	0.587	0.273	0.097	-1.181	0.467
5576	0.508	1.447	0.481	0.512	1.453	0.273	0.489	1.329	0.296
5577	0.508	1.447	0.050	0.512	1.453	0.462	0.497	1.380	0.142
5578	0.505	1.430	0.501	0.506	1.415	0.491	0.511	1.464	0.097
5579	0.097	-1.181	0.358	0.374	0.568	0.512	0.297	0.099	0.142
5580	0.492	1.346	0.385	0.509	1.434	0.235	0.482	1.279	0.224
5581	0.495	1.363	0.358	0.488	1.302	0.512	0.511	1.464	0.492
5582	0.226	-0.356	0.508	0.371	0.549	0.444	0.097	-1.181	0.296
5583	0.000	?	0.185	0.275	-0.073	0.053	0.261	-0.137	0.142
5584	0.295	0.082	0.296	0.272	-0.091	0.053	0.184	-0.625	0.431
5585	0.295	0.082	0.508	0.272	-0.091	0.478	0.184	-0.625	0.185
5586	0.505	1.430	0.328	0.462	1.133	0.491	0.466	1.178	0.450
5587	0.484	1.295	0.467	0.494	1.340	0.193	0.500	1.397	0.385
5588	0.508	1.447	0.185	0.503	1.396	0.512	0.453	1.093	0.450
5589	0.266	-0.103	0.358	0.234	-0.336	0.508	0.097	-1.181	0.262
5590	0.505	1.430	0.358	0.520	1.509	0.514	0.432	0.958	0.185
5591	0.492	1.346	0.508	0.439	0.982	0.193	0.508	1.447	0.185
5592	0.263	-0.120	0.508	0.053	-1.504	0.508	0.297	0.099	0.385
5593	0.263	-0.120	0.508	0.053	-1.504	0.398	0.297	0.099	0.431
5594	0.489	1.329	0.481	0.471	1.189	0.341	0.500	1.397	0.512
5595	0.489	1.329	0.481	0.491	1.321	0.508	0.505	1.430	0.296
5596	0.358	0.487	0.262	0.193	-0.600	0.508	0.000	?	0.450
5597	0.487	1.312	0.296	0.512	1.453	0.512	0.511	1.464	0.050
5598	0.505	1.430	0.467	0.509	1.434	0.508	0.513	1.481	0.097
5599	0.503	1.413	0.501	0.512	1.453	0.512	0.408	0.807	0.142
5600	0.329	0.301	0.296	0.196	-0.581	0.514	0.050	-1.485	0.450
5601	0.489	1.329	0.501	0.506	1.415	0.273	0.495	1.363	0.328
5602	0.142	-0.895	0.501	0.401	0.737	0.235	0.097	-1.181	0.185
5603	0.142	-0.895	0.185	0.342	0.361	0.422	0.097	-1.181	0.508
5604	0.500	1.397	0.508	0.503	1.396	0.149	0.497	1.380	0.050
5605	0.495	1.363	0.512	0.506	1.415	0.149	0.511	1.464	0.492
5606	0.450	1.076	0.512	0.512	1.453	0.341	0.484	1.295	0.185
5607	0.468	1.194	0.508	0.506	1.415	0.478	0.495	1.363	0.142
5608	0.505	1.430	0.512	0.506	1.415	0.501	0.405	0.790	0.142

5609	0.500	1.397	0.481	0.509	1.434	0.491	0.508	1.447	0.481
5610	0.097	-1.181	0.481	0.278	-0.054	0.491	0.224	-0.373	0.467
5611	0.329	0.301	0.492	0.234	-0.336	0.462	0.050	-1.485	0.481
5612	0.487	1.312	0.185	0.424	0.888	0.501	0.503	1.413	0.409
5613	0.432	0.958	0.262	0.149	-0.882	0.508	0.142	-0.895	0.508
5614	0.432	0.958	0.481	0.149	-0.882	0.501	0.142	-0.895	0.142
5615	0.184	-0.625	0.431	0.348	0.398	0.341	0.224	-0.373	0.097
5616	0.492	1.346	0.409	0.482	1.265	0.235	0.450	1.076	0.501
5617	0.492	1.346	0.501	0.506	1.415	0.478	0.450	1.076	0.501
5618	0.497	1.380	0.513	0.482	1.265	0.102	0.513	1.481	0.185
5619	0.497	1.380	0.508	0.482	1.265	0.508	0.513	1.481	0.467
5620	0.505	1.430	0.508	0.456	1.095	0.235	0.513	1.481	0.467
5621	0.492	1.346	0.481	0.500	1.378	0.501	0.445	1.043	0.224
5622	0.495	1.363	0.513	0.512	1.453	0.478	0.511	1.464	0.512
5623	0.487	1.312	0.501	0.503	1.396	0.501	0.479	1.262	0.450
5624	0.295	0.082	0.142	0.339	0.342	0.512	0.184	-0.625	0.508
5625	0.142	-0.895	0.142	0.237	-0.317	0.512	0.408	0.807	0.508
5626	0.495	1.363	0.385	0.474	1.208	0.508	0.489	1.329	0.508
5627	0.142	-0.895	0.409	0.102	-1.184	0.193	0.355	0.470	0.508
5628	0.461	1.144	0.409	0.506	1.415	0.193	0.505	1.430	0.513
5629	0.142	-0.895	0.358	0.240	-0.299	0.273	0.261	-0.137	0.450
5630	0.500	1.397	0.358	0.503	1.396	0.273	0.505	1.430	0.185
5631	0.479	1.262	0.097	0.477	1.227	0.053	0.505	1.430	0.142
5632	0.508	1.447	0.262	0.497	1.359	0.514	0.408	0.807	0.431
5633	0.432	0.958	0.481	0.515	1.472	0.102	0.503	1.413	0.185
5634	0.447	1.060	0.508	0.518	1.491	0.341	0.450	1.076	0.512
5635	0.447	1.060	0.097	0.515	1.472	0.478	0.466	1.178	0.501
5636	0.187	-0.608	0.492	0.193	-0.600	0.341	0.295	0.082	0.450
5637	0.224	-0.373	0.262	0.193	-0.600	0.508	0.142	-0.895	0.512
5638	0.184	-0.625	0.513	0.278	-0.054	0.273	0.097	-1.181	0.512
5639	0.187	-0.608	0.224	0.272	-0.091	0.478	0.142	-0.895	0.481
5640	0.295	0.082	0.508	0.053	-1.504	0.341	0.224	-0.373	0.467
5641	0.497	1.380	0.450	0.512	1.453	0.309	0.492	1.346	0.492
5642	0.226	-0.356	0.224	0.102	-1.184	0.309	0.297	0.099	0.097
5643	0.297	0.099	0.508	0.345	0.379	0.501	0.142	-0.895	0.185
5644	0.505	1.430	0.481	0.477	1.227	0.501	0.429	0.942	0.467
5645	0.297	0.099	0.142	0.345	0.379	0.501	0.184	-0.625	0.481
5646	0.508	1.447	0.508	0.506	1.415	0.193	0.445	1.043	0.467
5647	0.389	0.689	0.492	0.275	-0.073	0.512	0.384	0.655	0.097
5648	0.482	1.279	0.492	0.477	1.227	0.235	0.447	1.060	0.097
5649	0.050	-1.485	0.512	0.339	0.342	0.149	0.261	-0.137	0.224
5650	0.050	-1.485	0.142	0.307	0.135	0.102	0.184	-0.625	0.050
5651	0.050	-1.485	0.142	0.307	0.135	0.514	0.184	-0.625	0.508
5652	0.429	0.942	0.450	0.497	1.359	0.102	0.384	0.655	0.508
5653	0.479	1.262	0.224	0.500	1.378	0.102	0.429	0.942	0.185
5654	0.479	1.262	0.224	0.500	1.378	0.501	0.508	1.447	0.409
5655	0.050	-1.485	0.501	0.193	-0.600	0.235	0.297	0.099	0.097
5656	0.500	1.397	0.501	0.512	1.453	0.512	0.445	1.043	0.501
5657	0.361	0.504	0.481	0.234	-0.336	0.273	0.050	-1.485	0.185
5658	0.387	0.672	0.224	0.149	-0.882	0.309	0.097	-1.181	0.431
5659	0.361	0.504	0.142	0.102	-1.184	0.309	0.142	-0.895	0.512
5660	0.505	1.430	0.296	0.509	1.434	0.501	0.447	1.060	0.142
5661	0.184	-0.625	0.224	0.102	-1.184	0.501	0.326	0.285	0.513
5662	0.297	0.099	0.492	0.105	-1.165	0.491	0.184	-0.625	0.450
5663	0.511	1.464	0.185	0.506	1.415	0.478	0.505	1.430	0.431
5664	0.329	0.301	0.142	0.234	-0.336	0.462	0.050	-1.485	0.512
5665	0.468	1.194	0.492	0.509	1.434	0.149	0.487	1.312	0.513
5666	0.187	-0.608	0.513	0.272	-0.091	0.193	0.184	-0.625	0.467
5667	0.358	0.487	0.492	0.307	0.135	0.444	0.142	-0.895	0.296
5668	0.358	0.487	0.513	0.307	0.135	0.422	0.142	-0.895	0.296
5669	0.503	1.413	0.224	0.500	1.378	0.053	0.479	1.262	0.512

5670	0.503	1.413	0.185	0.500	1.378	0.512	0.466	1.178	0.296
5671	0.503	1.413	0.512	0.488	1.302	0.512	0.479	1.262	0.262
5672	0.487	1.312	0.296	0.471	1.189	0.149	0.405	0.790	0.513
5673	0.479	1.262	0.431	0.462	1.133	0.478	0.503	1.413	0.508
5674	0.263	−0.120	0.296	0.149	−0.882	0.512	0.142	−0.895	0.508
5675	0.295	0.082	0.513	0.193	−0.600	0.512	0.097	−1.181	0.296
5676	0.471	1.211	0.358	0.447	1.039	0.512	0.497	1.380	0.296
5677	0.500	1.397	0.358	0.424	0.888	0.512	0.495	1.363	0.385
5678	0.300	0.116	0.481	0.053	−1.504	0.501	0.184	−0.625	0.296
5679	0.503	1.413	0.501	0.509	1.434	0.341	0.468	1.194	0.431
5680	0.503	1.413	0.492	0.509	1.434	0.512	0.468	1.194	0.296
5681	0.184	−0.625	0.431	0.149	−0.882	0.371	0.224	−0.373	0.185
5682	0.508	1.447	0.508	0.477	1.227	0.273	0.508	1.447	0.431
5683	0.513	1.481	0.501	0.509	1.434	0.501	0.450	1.076	0.450
5684	0.508	1.447	0.501	0.512	1.453	0.422	0.505	1.430	0.492
5685	0.503	1.413	0.508	0.512	1.453	0.508	0.505	1.430	0.467
5686	0.508	1.447	0.512	0.512	1.453	0.512	0.505	1.430	0.142
5687	0.476	1.245	0.512	0.500	1.378	0.512	0.505	1.430	0.142
5688	0.479	1.262	0.431	0.339	0.342	0.478	0.516	1.498	0.492
5689	0.489	1.329	0.431	0.515	1.472	0.478	0.450	1.076	0.385
5690	0.184	−0.625	0.508	0.377	0.587	0.491	0.184	−0.625	0.501
5691	0.261	−0.137	0.508	0.275	−0.073	0.508	0.142	−0.895	0.385
5692	0.479	1.262	0.481	0.503	1.396	0.501	0.432	0.958	0.492
5693	0.432	0.958	0.450	0.424	0.888	0.398	0.184	−0.625	0.508
5694	0.408	0.807	0.512	0.512	1.453	0.235	0.518	1.514	0.450
5695	0.495	1.363	0.492	0.509	1.434	0.508	0.505	1.430	0.467
5696	0.511	1.464	0.501	0.512	1.453	0.514	0.447	1.060	0.508
5697	0.505	1.430	0.467	0.477	1.227	0.508	0.511	1.464	0.501
5698	0.505	1.430	0.097	0.477	1.227	0.053	0.511	1.464	0.501
5699	0.476	1.245	0.481	0.488	1.302	0.053	0.479	1.262	0.492
5700	0.511	1.464	0.262	0.506	1.415	0.398	0.466	1.178	0.512
5701	0.500	1.397	0.492	0.497	1.359	0.235	0.495	1.363	0.501
5702	0.145	−0.878	0.296	0.398	0.718	0.514	0.097	−1.181	0.512
5703	0.145	−0.878	0.431	0.234	−0.336	0.462	0.184	−0.625	0.492
5704	0.387	0.672	0.512	0.509	1.434	0.273	0.471	1.211	0.492
5705	0.411	0.824	0.508	0.512	1.453	0.478	0.484	1.295	0.501
5706	0.411	0.824	0.467	0.509	1.434	0.193	0.471	1.211	0.492
5707	0.361	0.504	0.097	0.053	−1.504	0.422	0.097	−1.181	0.409
5708	0.361	0.504	0.508	0.053	−1.504	0.235	0.097	−1.181	0.512
5709	0.097	−1.181	0.467	0.401	0.737	0.422	0.224	−0.373	0.481
5710	0.263	−0.120	0.508	0.234	−0.336	0.508	0.050	−1.485	0.512
5711	0.476	1.245	0.492	0.512	1.453	0.193	0.511	1.464	0.501
5712	0.500	1.397	0.508	0.465	1.152	0.462	0.505	1.430	0.467
5713	0.097	−1.181	0.513	0.272	−0.091	0.462	0.187	−0.608	0.431
5714	0.489	1.329	0.513	0.477	1.227	0.371	0.411	0.824	0.508
5715	0.266	−0.103	0.224	0.193	−0.600	0.444	0.097	−1.181	0.185
5716	0.508	1.447	0.512	0.424	0.888	0.462	0.500	1.397	0.450
5717	0.226	−0.356	0.508	0.234	−0.336	0.462	0.184	−0.625	0.142
5718	0.505	1.430	0.481	0.424	0.888	0.508	0.434	0.975	0.512
5719	0.447	1.060	0.296	0.512	1.453	0.102	0.516	1.498	0.492
5720	0.226	−0.356	0.512	0.193	−0.600	0.102	0.142	−0.895	0.450
5721	0.503	1.413	0.185	0.462	1.133	0.462	0.508	1.447	0.409
5722	0.484	1.295	0.296	0.456	1.095	0.422	0.447	1.060	0.224
5723	0.142	−0.895	0.512	0.374	0.568	0.235	0.000	?	0.492
5724	0.500	1.397	0.512	0.442	1.001	0.512	0.429	0.942	0.512
5725	0.495	1.363	0.501	0.465	1.152	0.512	0.511	1.464	0.492
5726	0.495	1.363	0.224	0.465	1.152	0.478	0.511	1.464	0.492
5727	0.513	1.481	0.262	0.500	1.378	0.309	0.466	1.178	0.512
5728	0.142	−0.895	0.296	0.105	−1.165	0.193	0.297	0.099	0.501
5729	0.142	−0.895	0.501	0.105	−1.165	0.398	0.297	0.099	0.501
5730	0.445	1.043	0.328	0.462	1.133	0.149	0.513	1.481	0.224

5731	0.224	-0.373	0.328	0.427	0.907	0.508	0.297	0.099	0.431
5732	0.226	-0.356	0.501	0.234	-0.336	0.309	0.266	-0.103	0.467
5733	0.497	1.380	0.431	0.509	1.434	0.235	0.511	1.464	0.492
5734	0.497	1.380	0.409	0.509	1.434	0.514	0.505	1.430	0.296
5735	0.479	1.262	0.142	0.477	1.227	0.508	0.503	1.413	0.492
5736	0.224	-0.373	0.501	0.310	0.153	0.512	0.295	0.082	0.185
5737	0.142	-0.895	0.328	0.193	-0.600	0.512	0.295	0.082	0.097
5738	0.297	0.099	0.142	0.404	0.756	0.273	0.384	0.655	0.508
5739	0.224	-0.373	0.097	0.152	-0.863	0.235	0.297	0.099	0.508
5740	0.487	1.312	0.328	0.509	1.434	0.478	0.429	0.942	0.501
5741	0.187	-0.608	0.328	0.307	0.135	0.273	0.300	0.116	0.185
5742	0.142	-0.895	0.492	0.234	-0.336	0.501	0.187	-0.608	0.185
5743	0.495	1.363	0.508	0.512	1.453	0.235	0.429	0.942	0.185
5744	0.508	1.447	0.508	0.512	1.453	0.444	0.445	1.043	0.481
5745	0.497	1.380	0.097	0.515	1.472	0.235	0.495	1.363	0.097
5746	0.508	1.447	0.097	0.509	1.434	0.235	0.466	1.178	0.097
5747	0.229	-0.339	0.492	0.272	-0.091	0.508	0.142	-0.895	0.501
5748	0.184	-0.625	0.501	0.237	-0.317	0.491	0.145	-0.878	0.501
5749	0.516	1.498	0.185	0.474	1.208	0.478	0.495	1.363	0.508
5750	0.295	0.082	0.262	0.272	-0.091	0.514	0.382	0.638	0.224
5751	0.432	0.958	0.508	0.500	1.378	0.491	0.500	1.397	0.481
5752	0.295	0.082	0.501	0.234	-0.336	0.478	0.382	0.638	0.097
5753	0.513	1.481	0.467	0.442	1.001	0.501	0.487	1.312	0.224
5754	0.358	0.487	0.467	0.234	-0.336	0.478	0.000	?	0.262
5755	0.358	0.487	0.513	0.234	-0.336	0.444	0.000	?	0.050
5756	0.479	1.262	0.513	0.512	1.453	0.462	0.511	1.464	0.050
5757	0.505	1.430	0.501	0.488	1.302	0.491	0.447	1.060	0.492
5758	0.489	1.329	0.513	0.477	1.227	0.478	0.471	1.211	0.481
5759	0.432	0.958	0.185	0.512	1.453	0.512	0.508	1.447	0.508
5760	0.505	1.430	0.142	0.488	1.302	0.501	0.500	1.397	0.385
5761	0.500	1.397	0.508	0.477	1.227	0.512	0.500	1.397	0.185
5762	0.503	1.413	0.467	0.503	1.396	0.462	0.489	1.329	0.513
5763	0.511	1.464	0.481	0.480	1.246	0.491	0.508	1.447	0.513
5764	0.508	1.447	0.296	0.439	0.982	0.512	0.500	1.397	0.262
5765	0.508	1.447	0.262	0.456	1.095	0.501	0.511	1.464	0.296
5766	0.426	0.925	0.142	0.485	1.283	0.444	0.492	1.346	0.328
5767	0.426	0.925	0.492	0.474	1.208	0.149	0.492	1.346	0.501
5768	0.503	1.413	0.097	0.509	1.434	0.444	0.505	1.430	0.508
5769	0.505	1.430	0.097	0.506	1.415	0.462	0.487	1.312	0.508
5770	0.482	1.279	0.224	0.509	1.434	0.491	0.405	0.790	0.501
5771	0.447	1.060	0.262	0.456	1.095	0.371	0.516	1.498	0.501
5772	0.511	1.464	0.512	0.494	1.340	0.491	0.484	1.295	0.467
5773	0.492	1.346	0.262	0.509	1.434	0.512	0.513	1.481	0.385
5774	0.500	1.397	0.296	0.500	1.378	0.512	0.495	1.363	0.097
5775	0.468	1.194	0.513	0.442	1.001	0.512	0.463	1.161	0.262
5776	0.097	-1.181	0.492	0.149	-0.882	0.341	0.432	0.958	0.508
5777	0.482	1.279	0.501	0.447	1.039	0.514	0.513	1.481	0.358
5778	0.263	-0.120	0.513	0.459	1.114	0.478	0.184	-0.625	0.467
5779	0.492	1.346	0.467	0.494	1.340	0.501	0.450	1.076	0.185
5780	0.300	0.116	0.467	0.371	0.549	0.508	0.142	-0.895	0.296
5781	0.432	0.958	0.185	0.488	1.302	0.508	0.518	1.514	0.262
5782	0.516	1.498	0.185	0.512	1.453	0.422	0.492	1.346	0.492
5783	0.508	1.447	0.185	0.515	1.472	0.422	0.445	1.043	0.185
5784	0.463	1.161	0.097	0.515	1.472	0.102	0.408	0.807	0.185
5785	0.100	-1.164	0.142	0.342	0.361	0.478	0.224	-0.373	0.142
5786	0.505	1.430	0.097	0.509	1.434	0.478	0.489	1.329	0.224
5787	0.461	1.144	0.512	0.482	1.265	0.512	0.511	1.464	0.481
5788	0.513	1.481	0.501	0.506	1.415	0.193	0.492	1.346	0.224
5789	0.489	1.329	0.185	0.506	1.415	0.462	0.489	1.329	0.224
5790	0.508	1.447	0.224	0.503	1.396	0.193	0.511	1.464	0.481
5791	0.513	1.481	0.358	0.421	0.869	0.193	0.500	1.397	0.481

5792	0.513	1.481	0.224	0.421	0.869	0.508	0.500	1.397	0.501
5793	0.226	-0.356	0.296	0.102	-1.184	0.508	0.226	-0.356	0.492
5794	0.508	1.447	0.501	0.474	1.208	0.508	0.434	0.975	0.508
5795	0.500	1.397	0.385	0.477	1.227	0.235	0.466	1.178	0.385
5796	0.479	1.262	0.097	0.518	1.491	0.193	0.497	1.380	0.050
5797	0.297	0.099	0.050	0.193	-0.600	0.235	0.300	0.116	0.050
5798	0.513	1.481	0.262	0.462	1.133	0.478	0.495	1.363	0.050
5799	0.184	-0.625	0.142	0.196	-0.581	0.235	0.184	-0.625	0.097
5800	0.297	0.099	0.262	0.196	-0.581	0.102	0.097	-1.181	0.097
5801	0.513	1.481	0.492	0.503	1.396	0.444	0.505	1.430	0.097
5802	0.513	1.481	0.328	0.503	1.396	0.508	0.505	1.430	0.492
5803	0.505	1.430	0.501	0.503	1.396	0.508	0.500	1.397	0.513
5804	0.224	-0.373	0.481	0.234	-0.336	0.193	0.184	-0.625	0.224
5805	0.261	-0.137	0.481	0.193	-0.600	0.462	0.187	-0.608	0.262
5806	0.297	0.099	0.224	0.234	-0.336	0.149	0.184	-0.625	0.385
5807	0.505	1.430	0.328	0.480	1.246	0.235	0.479	1.262	0.224
5808	0.329	0.301	0.224	0.237	-0.317	0.193	0.097	-1.181	0.450
5809	0.329	0.301	0.224	0.102	-1.184	0.235	0.097	-1.181	0.501
5810	0.500	1.397	0.481	0.442	1.001	0.235	0.495	1.363	0.450
5811	0.429	0.942	0.450	0.506	1.415	0.478	0.497	1.380	0.142
5812	0.408	0.807	0.450	0.503	1.396	0.491	0.503	1.413	0.142
5813	0.142	-0.895	0.097	0.193	-0.600	0.512	0.224	-0.373	0.142
5814	0.503	1.413	0.385	0.462	1.133	0.053	0.479	1.262	0.224
5815	0.332	0.318	0.185	0.149	-0.882	0.273	0.097	-1.181	0.185
5816	0.142	-0.895	0.450	0.237	-0.317	0.508	0.224	-0.373	0.481
5817	0.097	-1.181	0.467	0.193	-0.600	0.508	0.263	-0.120	0.296
5818	0.329	0.301	0.481	0.237	-0.317	0.193	0.050	-1.485	0.467
5819	0.329	0.301	0.512	0.237	-0.317	0.398	0.050	-1.485	0.513
5820	0.487	1.312	0.142	0.477	1.227	0.514	0.489	1.329	0.513
5821	0.508	1.447	0.097	0.488	1.302	0.478	0.479	1.262	0.050
5822	0.508	1.447	0.508	0.512	1.453	0.478	0.503	1.413	0.050
5823	0.097	-1.181	0.513	0.053	-1.504	0.478	0.387	0.672	0.328
5824	0.097	-1.181	0.501	0.275	-0.073	0.508	0.187	-0.608	0.328
5825	0.492	1.346	0.328	0.506	1.415	0.508	0.513	1.481	0.467
5826	0.500	1.397	0.481	0.506	1.415	0.478	0.513	1.481	0.481
5827	0.184	-0.625	0.492	0.196	-0.581	0.512	0.263	-0.120	0.513
5828	0.263	-0.120	0.262	0.401	0.737	0.309	0.297	0.099	0.185
5829	0.505	1.430	0.501	0.509	1.434	0.149	0.329	0.301	0.050
5830	0.497	1.380	0.467	0.474	1.208	0.491	0.503	1.413	0.224
5831	0.466	1.178	0.508	0.477	1.227	0.508	0.508	1.447	0.501
5832	0.466	1.178	0.467	0.477	1.227	0.501	0.505	1.430	0.409
5833	0.511	1.464	0.467	0.506	1.415	0.235	0.500	1.397	0.492
5834	0.511	1.464	0.467	0.506	1.415	0.149	0.500	1.397	0.501
5835	0.497	1.380	0.508	0.477	1.227	0.149	0.466	1.178	0.224
5836	0.511	1.464	0.450	0.503	1.396	0.491	0.387	0.672	0.224
5837	0.184	-0.625	0.501	0.313	0.172	0.309	0.097	-1.181	0.512
5838	0.142	-0.895	0.328	0.149	-0.882	0.309	0.261	-0.137	0.431
5839	0.505	1.430	0.358	0.488	1.302	0.273	0.508	1.447	0.508
5840	0.466	1.178	0.097	0.512	1.453	0.371	0.358	0.487	0.097
5841	0.479	1.262	0.185	0.503	1.396	0.501	0.466	1.178	0.450
5842	0.297	0.099	0.467	0.237	-0.317	0.309	0.184	-0.625	0.385
5843	0.263	-0.120	0.512	0.149	-0.882	0.398	0.297	0.099	0.296
5844	0.142	-0.895	0.513	0.149	-0.882	0.462	0.263	-0.120	0.431
5845	0.487	1.312	0.512	0.494	1.340	0.444	0.489	1.329	0.492
5846	0.097	-1.181	0.513	0.313	0.172	0.501	0.184	-0.625	0.481
5847	0.097	-1.181	0.224	0.313	0.172	0.398	0.184	-0.625	0.492
5848	0.226	-0.356	0.142	0.275	-0.073	0.398	0.142	-0.895	0.501
5849	0.263	-0.120	0.450	0.368	0.530	0.478	0.224	-0.373	0.467
5850	0.508	1.447	0.385	0.497	1.359	0.309	0.482	1.279	0.501
5851	0.266	-0.103	0.385	0.310	0.153	0.309	0.224	-0.373	0.185
5852	0.297	0.099	0.142	0.401	0.737	0.309	0.224	-0.373	0.481

5853	0.511	1.464	0.185	0.462	1.133	0.341	0.474	1.228	0.097
5854	0.492	1.346	0.512	0.447	1.039	0.371	0.482	1.279	0.097
5855	0.497	1.380	0.142	0.500	1.378	0.371	0.500	1.397	0.262
5856	0.516	1.498	0.262	0.398	0.718	0.501	0.492	1.346	0.262
5857	0.466	1.178	0.262	0.401	0.737	0.478	0.511	1.464	0.481
5858	0.468	1.194	0.142	0.480	1.246	0.273	0.387	0.672	0.508
5859	0.187	-0.608	0.142	0.310	0.153	0.149	0.050	-1.485	0.508
5860	0.187	-0.608	0.467	0.310	0.153	0.371	0.050	-1.485	0.508
5861	0.187	-0.608	0.481	0.310	0.153	0.193	0.050	-1.485	0.508
5862	0.097	-1.181	0.431	0.339	0.342	0.341	0.097	-1.181	0.224
5863	0.142	-0.895	0.450	0.368	0.530	0.478	0.097	-1.181	0.224
5864	0.097	-1.181	0.481	0.368	0.530	0.491	0.097	-1.181	0.513
5865	0.511	1.464	0.328	0.494	1.340	0.398	0.489	1.329	0.328
5866	0.500	1.397	0.328	0.474	1.208	0.371	0.508	1.447	0.328
5867	0.187	-0.608	0.097	0.275	-0.073	0.149	0.224	-0.373	0.296
5868	0.226	-0.356	0.142	0.149	-0.882	0.193	0.261	-0.137	0.185
5869	0.361	0.504	0.224	0.371	0.549	0.149	0.384	0.655	0.501
5870	0.226	-0.356	0.224	0.193	-0.600	0.491	0.224	-0.373	0.097
5871	0.295	0.082	0.224	0.339	0.342	0.371	0.447	1.060	0.097
5872	0.500	1.397	0.385	0.477	1.227	0.501	0.500	1.397	0.097
5873	0.382	0.638	0.492	0.488	1.302	0.508	0.447	1.060	0.224
5874	0.097	-1.181	0.409	0.401	0.737	0.501	0.142	-0.895	0.224
5875	0.050	-1.485	0.492	0.368	0.530	0.273	0.142	-0.895	0.481
5876	0.261	-0.137	0.185	0.149	-0.882	0.309	0.142	-0.895	0.431
5877	0.142	-0.895	0.508	0.193	-0.600	0.149	0.224	-0.373	0.513
5878	0.263	-0.120	0.512	0.149	-0.882	0.149	0.184	-0.625	0.512
5879	0.495	1.363	0.224	0.491	1.321	0.514	0.479	1.262	0.492
5880	0.329	0.301	0.385	0.371	0.549	0.514	0.295	0.082	0.409
5881	0.497	1.380	0.385	0.497	1.359	0.478	0.466	1.178	0.185
5882	0.479	1.262	0.481	0.509	1.434	0.273	0.513	1.481	0.328
5883	0.479	1.262	0.508	0.503	1.396	0.309	0.513	1.481	0.224
5884	0.226	-0.356	0.508	0.272	-0.091	0.341	0.050	-1.485	0.296
5885	0.326	0.285	0.185	0.307	0.135	0.341	0.050	-1.485	0.296
5886	0.226	-0.356	0.512	0.149	-0.882	0.491	0.326	0.285	0.296
5887	0.226	-0.356	0.513	0.149	-0.882	0.491	0.326	0.285	0.296
5888	0.479	1.262	0.481	0.515	1.472	0.462	0.468	1.194	0.481
5889	0.447	1.060	0.224	0.515	1.472	0.193	0.484	1.295	0.501
5890	0.450	1.076	0.481	0.477	1.227	0.235	0.513	1.481	0.501
5891	0.097	-1.181	0.481	0.272	-0.091	0.491	0.184	-0.625	0.185
5892	0.387	0.672	0.508	0.310	0.153	0.501	0.050	-1.485	0.508
5893	0.184	-0.625	0.512	0.345	0.379	0.462	0.226	-0.356	0.492
5894	0.450	1.076	0.328	0.342	0.361	0.273	0.500	1.397	0.185
5895	0.466	1.178	0.224	0.497	1.359	0.478	0.413	0.841	0.296
5896	0.484	1.295	0.508	0.494	1.340	0.508	0.492	1.346	0.296
5897	0.516	1.498	0.501	0.459	1.114	0.508	0.500	1.397	0.467
5898	0.142	-0.895	0.501	0.193	-0.600	0.514	0.224	-0.373	0.467
5899	0.097	-1.181	0.431	0.234	-0.336	0.501	0.224	-0.373	0.512
5900	0.503	1.413	0.296	0.491	1.321	0.478	0.508	1.447	0.224
5901	0.516	1.498	0.481	0.503	1.396	0.512	0.426	0.925	0.358
5902	0.500	1.397	0.481	0.462	1.133	0.478	0.503	1.413	0.385
5903	0.329	0.301	0.481	0.272	-0.091	0.508	0.097	-1.181	0.467
5904	0.482	1.279	0.262	0.480	1.246	0.102	0.447	1.060	0.481
5905	0.492	1.346	0.501	0.509	1.434	0.508	0.382	0.638	0.512
5906	0.263	-0.120	0.431	0.000	?	0.193	0.297	0.099	0.142
5907	0.500	1.397	0.409	0.506	1.415	0.149	0.429	0.942	0.431
5908	0.466	1.178	0.513	0.515	1.472	0.193	0.487	1.312	0.512
5909	0.511	1.464	0.481	0.503	1.396	0.309	0.482	1.279	0.513
5910	0.463	1.161	0.481	0.480	1.246	0.512	0.492	1.346	0.467
5911	0.468	1.194	0.501	0.509	1.434	0.478	0.495	1.363	0.142
5912	0.468	1.194	0.512	0.477	1.227	0.501	0.463	1.161	0.358
5913	0.505	1.430	0.492	0.509	1.434	0.478	0.505	1.430	0.431

5914	0.453	1.093	0.450	0.102	-1.184	0.478	0.184	-0.625	0.467
5915	0.497	1.380	0.513	0.506	1.415	0.341	0.482	1.279	0.450
5916	0.329	0.301	0.481	0.196	-0.581	0.193	0.097	-1.181	0.385
5917	0.361	0.504	0.328	0.149	-0.882	0.512	0.097	-1.181	0.328
5918	0.097	-1.181	0.296	0.196	-0.581	0.273	0.261	-0.137	0.431
5919	0.187	-0.608	0.358	0.313	0.172	0.273	0.261	-0.137	0.450
5920	0.461	1.144	0.296	0.512	1.453	0.273	0.479	1.262	0.450
5921	0.511	1.464	0.508	0.477	1.227	0.193	0.503	1.413	0.142
5922	0.513	1.481	0.224	0.500	1.378	0.444	0.503	1.413	0.409
5923	0.511	1.464	0.467	0.477	1.227	0.309	0.503	1.413	0.431
5924	0.513	1.481	0.481	0.477	1.227	0.273	0.503	1.413	0.450
5925	0.224	-0.373	0.450	0.342	0.361	0.273	0.226	-0.356	0.508
5926	0.142	-0.895	0.450	0.193	-0.600	0.273	0.226	-0.356	0.431
5927	0.450	1.076	0.481	0.512	1.453	0.309	0.518	1.514	0.431
5928	0.389	0.689	0.501	0.272	-0.091	0.462	0.329	0.301	0.513
5929	0.389	0.689	0.142	0.272	-0.091	0.491	0.329	0.301	0.450
5930	0.142	-0.895	0.142	0.275	-0.073	0.462	0.295	0.082	0.431
5931	0.184	-0.625	0.224	0.193	-0.600	0.491	0.184	-0.625	0.481
5932	0.513	1.481	0.224	0.444	1.020	0.422	0.497	1.380	0.492
5933	0.142	-0.895	0.224	0.313	0.172	0.309	0.097	-1.181	0.513
5934	0.261	-0.137	0.224	0.272	-0.091	0.193	0.097	-1.181	0.097
5935	0.261	-0.137	0.262	0.272	-0.091	0.235	0.097	-1.181	0.097
5936	0.142	-0.895	0.492	0.272	-0.091	0.193	0.226	-0.356	0.185
5937	0.142	-0.895	0.409	0.307	0.135	0.149	0.226	-0.356	0.501
5938	0.468	1.194	0.467	0.459	1.114	0.149	0.479	1.262	0.358
5939	0.484	1.295	0.481	0.488	1.302	0.149	0.432	0.958	0.450
5940	0.429	0.942	0.467	0.465	1.152	0.309	0.516	1.498	0.481
5941	0.445	1.043	0.450	0.491	1.321	0.514	0.516	1.498	0.501
5942	0.484	1.295	0.513	0.424	0.888	0.444	0.495	1.363	0.501
5943	0.329	0.301	0.328	0.307	0.135	0.508	0.408	0.807	0.512
5944	0.326	0.285	0.508	0.196	-0.581	0.341	0.184	-0.625	0.450
5945	0.100	-1.164	0.467	0.234	-0.336	0.462	0.329	0.301	0.185
5946	0.142	-0.895	0.492	0.193	-0.600	0.478	0.224	-0.373	0.142
5947	0.226	-0.356	0.328	0.149	-0.882	0.149	0.297	0.099	0.185
5948	0.226	-0.356	0.262	0.149	-0.882	0.371	0.297	0.099	0.328
5949	0.226	-0.356	0.481	0.149	-0.882	0.371	0.297	0.099	0.328
5950	0.387	0.672	0.492	0.310	0.153	0.501	0.297	0.099	0.142
5951	0.495	1.363	0.185	0.520	1.509	0.501	0.482	1.279	0.409
5952	0.411	0.824	0.450	0.444	1.020	0.491	0.503	1.413	0.501
5953	0.492	1.346	0.450	0.509	1.434	0.149	0.497	1.380	0.508
5954	0.184	-0.625	0.185	0.345	0.379	0.341	0.184	-0.625	0.508
5955	0.508	1.447	0.512	0.462	1.133	0.235	0.500	1.397	0.508
5956	0.513	1.481	0.224	0.477	1.227	0.501	0.484	1.295	0.513
5957	0.229	-0.339	0.450	0.149	-0.882	0.501	0.184	-0.625	0.501
5958	0.387	0.672	0.409	0.374	0.568	0.478	0.297	0.099	0.467
5959	0.387	0.672	0.409	0.374	0.568	0.193	0.297	0.099	0.450
5960	0.482	1.279	0.409	0.503	1.396	0.508	0.468	1.194	0.481
5961	0.511	1.464	0.508	0.503	1.396	0.478	0.468	1.194	0.512
5962	0.503	1.413	0.097	0.491	1.321	0.508	0.508	1.447	0.467
5963	0.187	-0.608	0.492	0.149	-0.882	0.512	0.224	-0.373	0.409
5964	0.000	?	0.481	0.342	0.361	0.309	0.358	0.487	0.142
5965	0.000	?	0.050	0.234	-0.336	0.371	0.384	0.655	0.512
5966	0.508	1.447	0.508	0.497	1.359	0.514	0.466	1.178	0.409
5967	0.511	1.464	0.142	0.497	1.359	0.478	0.479	1.262	0.431
5968	0.479	1.262	0.492	0.477	1.227	0.462	0.511	1.464	0.508
5969	0.226	-0.356	0.512	0.193	-0.600	0.478	0.145	-0.878	0.508
5970	0.484	1.295	0.508	0.512	1.453	0.235	0.429	0.942	0.185
5971	0.482	1.279	0.481	0.480	1.246	0.501	0.513	1.481	0.467
5972	0.508	1.447	0.450	0.512	1.453	0.508	0.516	1.498	0.224
5973	0.511	1.464	0.492	0.518	1.491	0.508	0.468	1.194	0.409
5974	0.329	0.301	0.492	0.307	0.135	0.273	0.142	-0.895	0.467

5975	0.226	-0.356	0.492	0.371	0.549	0.508	0.361	0.504	0.467
5976	0.505	1.430	0.142	0.515	1.472	0.512	0.434	0.975	0.467
5977	0.500	1.397	0.262	0.477	1.227	0.508	0.468	1.194	0.467
5978	0.497	1.380	0.508	0.459	1.114	0.512	0.447	1.060	0.185
5979	0.429	0.942	0.481	0.477	1.227	0.501	0.387	0.672	0.467
5980	0.297	0.099	0.296	0.234	-0.336	0.508	0.326	0.285	0.508
5981	0.482	1.279	0.296	0.497	1.359	0.478	0.432	0.958	0.296
5982	0.479	1.262	0.296	0.506	1.415	0.501	0.450	1.076	0.512
5983	0.479	1.262	0.262	0.506	1.415	0.512	0.450	1.076	0.185
5984	0.261	-0.137	0.142	0.272	-0.091	0.462	0.142	-0.895	0.450
5985	0.503	1.413	0.467	0.509	1.434	0.501	0.411	0.824	0.450
5986	0.429	0.942	0.097	0.512	1.453	0.478	0.429	0.942	0.467
5987	0.405	0.790	0.097	0.509	1.434	0.102	0.447	1.060	0.508
5988	0.513	1.481	0.185	0.512	1.453	0.273	0.511	1.464	0.450
5989	0.479	1.262	0.185	0.500	1.378	0.235	0.429	0.942	0.467
5990	0.479	1.262	0.492	0.509	1.434	0.273	0.429	0.942	0.512
5991	0.503	1.413	0.262	0.480	1.246	0.491	0.513	1.481	0.481
5992	0.511	1.464	0.513	0.503	1.396	0.371	0.447	1.060	0.262
5993	0.492	1.346	0.467	0.515	1.472	0.508	0.429	0.942	0.358
5994	0.447	1.060	0.224	0.459	1.114	0.462	0.479	1.262	0.385
5995	0.508	1.447	0.431	0.503	1.396	0.512	0.489	1.329	0.501
5996	0.482	1.279	0.358	0.477	1.227	0.512	0.511	1.464	0.262
5997	0.329	0.301	0.328	0.102	-1.184	0.462	0.097	-1.181	0.262
5998	0.297	0.099	0.328	0.272	-0.091	0.501	0.097	-1.181	0.262
5999	0.361	0.504	0.492	0.234	-0.336	0.422	0.184	-0.625	0.262
6000	0.300	0.116	0.328	0.272	-0.091	0.273	0.000	?	0.185
6001	0.508	1.447	0.513	0.491	1.321	0.273	0.503	1.413	0.431
6002	0.224	-0.373	0.142	0.371	0.549	0.235	0.361	0.504	0.262
6003	0.463	1.161	0.512	0.512	1.453	0.371	0.447	1.060	0.224
6004	0.476	1.245	0.296	0.462	1.133	0.508	0.482	1.279	0.328
6005	0.447	1.060	0.296	0.509	1.434	0.514	0.505	1.430	0.328
6006	0.447	1.060	0.185	0.509	1.434	0.514	0.505	1.430	0.467
6007	0.479	1.262	0.050	0.465	1.152	0.462	0.505	1.430	0.262
6008	0.497	1.380	0.492	0.500	1.378	0.508	0.450	1.076	0.481
6009	0.142	-0.895	0.450	0.421	0.869	0.478	0.184	-0.625	0.492
6010	0.142	-0.895	0.328	0.275	-0.073	0.514	0.145	-0.878	0.262
6011	0.226	-0.356	0.224	0.275	-0.073	0.273	0.184	-0.625	0.409
6012	0.226	-0.356	0.224	0.000	?	0.422	0.329	0.301	0.296
6013	0.226	-0.356	0.097	0.000	?	0.491	0.329	0.301	0.185
6014	0.224	-0.373	0.450	0.234	-0.336	0.422	0.145	-0.878	0.185
6015	0.263	-0.120	0.224	0.371	0.549	0.102	0.413	0.841	0.508
6016	0.492	1.346	0.409	0.509	1.434	0.309	0.500	1.397	0.224
6017	0.408	0.807	0.492	0.518	1.491	0.462	0.505	1.430	0.508
6018	0.466	1.178	0.142	0.518	1.491	0.512	0.505	1.430	0.328
6019	0.479	1.262	0.481	0.462	1.133	0.149	0.503	1.413	0.501
6020	0.466	1.178	0.409	0.509	1.434	0.491	0.511	1.464	0.224
6021	0.447	1.060	0.492	0.485	1.283	0.491	0.505	1.430	0.224
6022	0.513	1.481	0.501	0.512	1.453	0.193	0.463	1.161	0.185
6023	0.329	0.301	0.142	0.278	-0.054	0.444	0.450	1.076	0.262
6024	0.503	1.413	0.385	0.421	0.869	0.273	0.476	1.245	0.481
6025	0.463	1.161	0.358	0.491	1.321	0.508	0.505	1.430	0.508
6026	0.497	1.380	0.512	0.427	0.907	0.512	0.461	1.144	0.385
6027	0.329	0.301	0.508	0.102	-1.184	0.512	0.408	0.807	0.185
6028	0.261	-0.137	0.262	0.307	0.135	0.512	0.142	-0.895	0.097
6029	0.482	1.279	0.142	0.465	1.152	0.478	0.513	1.481	0.262
6030	0.492	1.346	0.481	0.512	1.453	0.341	0.413	0.841	0.481
6031	0.184	-0.625	0.296	0.149	-0.882	0.491	0.434	0.975	0.142
6032	0.447	1.060	0.262	0.494	1.340	0.501	0.508	1.447	0.262
6033	0.447	1.060	0.224	0.494	1.340	0.193	0.508	1.447	0.450
6034	0.187	-0.608	0.328	0.196	-0.581	0.501	0.184	-0.625	0.224
6035	0.505	1.430	0.481	0.444	1.020	0.309	0.466	1.178	0.512

6036	0.226	-0.356	0.224	0.272	-0.091	0.444	0.224	-0.373	0.450
6037	0.445	1.043	0.050	0.506	1.415	0.478	0.411	0.824	0.501
6038	0.405	0.790	0.481	0.509	1.434	0.444	0.466	1.178	0.481
6039	0.405	0.790	0.224	0.509	1.434	0.371	0.466	1.178	0.185
6040	0.405	0.790	0.224	0.509	1.434	0.462	0.466	1.178	0.262
6041	0.505	1.430	0.262	0.480	1.246	0.491	0.461	1.144	0.142
6042	0.097	-1.181	0.097	0.342	0.361	0.422	0.184	-0.625	0.481
6043	0.487	1.312	0.142	0.488	1.302	0.371	0.468	1.194	0.450
6044	0.479	1.262	0.450	0.497	1.359	0.149	0.508	1.447	0.262
6045	0.050	-1.485	0.508	0.196	-0.581	0.235	0.295	0.082	0.097
6046	0.508	1.447	0.185	0.494	1.340	0.444	0.511	1.464	0.467
6047	0.142	-0.895	0.142	0.310	0.153	0.444	0.184	-0.625	0.224
6048	0.492	1.346	0.501	0.447	1.039	0.273	0.450	1.076	0.328
6049	0.513	1.481	0.501	0.482	1.265	0.273	0.445	1.043	0.224
6050	0.511	1.464	0.450	0.447	1.039	0.273	0.466	1.178	0.224
6051	0.482	1.279	0.224	0.368	0.530	0.235	0.513	1.481	0.467
6052	0.453	1.093	0.185	0.462	1.133	0.309	0.450	1.076	0.262
6053	0.484	1.295	0.385	0.491	1.321	0.491	0.466	1.178	0.262
6054	0.492	1.346	0.185	0.421	0.869	0.235	0.511	1.464	0.492
6055	0.492	1.346	0.513	0.371	0.549	0.309	0.482	1.279	0.224
6056	0.142	-0.895	0.185	0.149	-0.882	0.149	0.266	-0.103	0.185
6057	0.263	-0.120	0.492	0.234	-0.336	0.149	0.361	0.504	0.185
6058	0.505	1.430	0.492	0.439	0.982	0.491	0.384	0.655	0.296
6059	0.484	1.295	0.508	0.444	1.020	0.273	0.500	1.397	0.262
6060	0.297	0.099	0.224	0.272	-0.091	0.508	0.263	-0.120	0.508
6061	0.297	0.099	0.185	0.272	-0.091	0.491	0.263	-0.120	0.501
6062	0.297	0.099	0.185	0.272	-0.091	0.273	0.263	-0.120	0.050
6063	0.261	-0.137	0.481	0.237	-0.317	0.478	0.261	-0.137	0.142
6064	0.142	-0.895	0.492	0.310	0.153	0.341	0.187	-0.608	0.501
6065	0.468	1.194	0.224	0.494	1.340	0.235	0.432	0.958	0.508
6066	0.097	-1.181	0.492	0.234	-0.336	0.235	0.261	-0.137	0.481
6067	0.097	-1.181	0.409	0.310	0.153	0.422	0.226	-0.356	0.142
6068	0.184	-0.625	0.512	0.149	-0.882	0.102	0.326	0.285	0.185
6069	0.184	-0.625	0.508	0.149	-0.882	0.444	0.326	0.285	0.431
6070	0.495	1.363	0.328	0.491	1.321	0.149	0.468	1.194	0.262
6071	0.261	-0.137	0.262	0.275	-0.073	0.478	0.261	-0.137	0.501
6072	0.516	1.498	0.481	0.515	1.472	0.309	0.479	1.262	0.262
6073	0.466	1.178	0.431	0.491	1.321	0.309	0.489	1.329	0.431
6074	0.224	-0.373	0.142	0.278	-0.054	0.235	0.263	-0.120	0.431
6075	0.429	0.942	0.328	0.477	1.227	0.235	0.411	0.824	0.512
6076	0.358	0.487	0.508	0.342	0.361	0.478	0.300	0.116	0.224
6077	0.329	0.301	0.296	0.234	-0.336	0.398	0.184	-0.625	0.142
6078	0.329	0.301	0.508	0.234	-0.336	0.193	0.184	-0.625	0.224
6079	0.492	1.346	0.512	0.424	0.888	0.235	0.511	1.464	0.492
6080	0.329	0.301	0.492	0.102	-1.184	0.273	0.226	-0.356	0.481
6081	0.511	1.464	0.512	0.439	0.982	0.273	0.511	1.464	0.142
6082	0.142	-0.895	0.512	0.149	-0.882	0.444	0.329	0.301	0.508
6083	0.511	1.464	0.224	0.477	1.227	0.309	0.508	1.447	0.508
6084	0.297	0.099	0.262	0.307	0.135	0.371	0.224	-0.373	0.492
6085	0.295	0.082	0.508	0.313	0.172	0.462	0.224	-0.373	0.467
6086	0.187	-0.608	0.481	0.234	-0.336	0.273	0.187	-0.608	0.185
6087	0.050	-1.485	0.501	0.237	-0.317	0.478	0.263	-0.120	0.262
6088	0.497	1.380	0.492	0.480	1.246	0.508	0.479	1.262	0.467
6089	0.453	1.093	0.224	0.398	0.718	0.444	0.505	1.430	0.431
6090	0.329	0.301	0.409	0.196	-0.581	0.478	0.387	0.672	0.050
6091	0.224	-0.373	0.358	0.237	-0.317	0.235	0.184	-0.625	0.224
6092	0.224	-0.373	0.296	0.275	-0.073	0.371	0.097	-1.181	0.481
6093	0.097	-1.181	0.185	0.275	-0.073	0.273	0.261	-0.137	0.296
6094	0.450	1.076	0.142	0.444	1.020	0.491	0.476	1.245	0.467
6095	0.226	-0.356	0.450	0.310	0.153	0.478	0.142	-0.895	0.450
6096	0.413	0.841	0.508	0.371	0.549	0.273	0.263	-0.120	0.513

6097	0.503	1.413	0.450	0.459	1.114	0.309	0.450	1.076	0.467
6098	0.142	-0.895	0.481	0.275	-0.073	0.478	0.226	-0.356	0.467
6099	0.476	1.245	0.492	0.474	1.208	0.235	0.511	1.464	0.224
6100	0.405	0.790	0.262	0.506	1.415	0.235	0.445	1.043	0.097
6101	0.484	1.295	0.224	0.444	1.020	0.398	0.500	1.397	0.431
6102	0.497	1.380	0.224	0.480	1.246	0.193	0.474	1.228	0.481
6103	0.145	-0.878	0.224	0.237	-0.317	0.512	0.184	-0.625	0.512
6104	0.387	0.672	0.358	0.371	0.549	0.235	0.263	-0.120	0.467
6105	0.355	0.470	0.431	0.275	-0.073	0.235	0.142	-0.895	0.185
6106	0.513	1.481	0.385	0.491	1.321	0.422	0.484	1.295	0.512
6107	0.508	1.447	0.296	0.480	1.246	0.235	0.453	1.093	0.142
6108	0.261	-0.137	0.512	0.272	-0.091	0.273	0.263	-0.120	0.142
6109	0.142	-0.895	0.142	0.313	0.172	0.309	0.097	-1.181	0.262
6110	0.474	1.228	0.224	0.474	1.208	0.149	0.471	1.211	0.296
6111	0.297	0.099	0.328	0.237	-0.317	0.444	0.226	-0.356	0.501
6112	0.261	-0.137	0.508	0.234	-0.336	0.422	0.329	0.301	0.450
6113	0.224	-0.373	0.142	0.404	0.756	0.478	0.224	-0.373	0.492
6114	0.329	0.301	0.467	0.193	-0.600	0.309	0.224	-0.373	0.501
6115	0.482	1.279	0.097	0.515	1.472	0.309	0.468	1.194	0.467
6116	0.224	-0.373	0.467	0.234	-0.336	0.309	0.266	-0.103	0.142
6117	0.050	-1.485	0.328	0.237	-0.317	0.491	0.261	-0.137	0.185
6118	0.479	1.262	0.224	0.418	0.850	0.508	0.492	1.346	0.185
6119	0.224	-0.373	0.224	0.237	-0.317	0.193	0.224	-0.373	0.296
6120	0.224	-0.373	0.328	0.275	-0.073	0.235	0.184	-0.625	0.185
6121	0.263	-0.120	0.142	0.313	0.172	0.512	0.184	-0.625	0.508
6122	0.097	-1.181	0.296	0.152	-0.863	0.102	0.297	0.099	0.492
6123	0.142	-0.895	0.050	0.447	1.039	0.398	0.263	-0.120	0.296
6124	0.445	1.043	0.450	0.424	0.888	0.371	0.505	1.430	0.508
6125	0.511	1.464	0.492	0.477	1.227	0.508	0.497	1.380	0.262
6126	0.184	-0.625	0.142	0.310	0.153	0.508	0.050	-1.485	0.262
6127	0.142	-0.895	0.481	0.307	0.135	0.508	0.142	-0.895	0.358
6128	0.500	1.397	0.508	0.310	0.153	0.102	0.497	1.380	0.450
6129	0.500	1.397	0.224	0.497	1.359	0.371	0.503	1.413	0.358
6130	0.453	1.093	0.431	0.506	1.415	0.149	0.484	1.295	0.492
6131	0.226	-0.356	0.467	0.196	-0.581	0.444	0.142	-0.895	0.296
6132	0.184	-0.625	0.481	0.234	-0.336	0.444	0.184	-0.625	0.508
6133	0.382	0.638	0.185	0.512	1.453	0.235	0.429	0.942	0.050
6134	0.184	-0.625	0.142	0.102	-1.184	0.444	0.263	-0.120	0.224
6135	0.508	1.447	0.296	0.398	0.718	0.508	0.497	1.380	0.296
6136	0.184	-0.625	0.508	0.368	0.530	0.501	0.261	-0.137	0.097
6137	0.489	1.329	0.224	0.506	1.415	0.444	0.432	0.958	0.358
6138	0.489	1.329	0.224	0.506	1.415	0.193	0.432	0.958	0.097
6139	0.508	1.447	0.481	0.506	1.415	0.102	0.513	1.481	0.224
6140	0.224	-0.373	0.481	0.102	-1.184	0.444	0.226	-0.356	0.501
6141	0.184	-0.625	0.185	0.374	0.568	0.501	0.142	-0.895	0.508
6142	0.187	-0.608	0.492	0.149	-0.882	0.371	0.224	-0.373	0.185
6143	0.482	1.279	0.512	0.444	1.020	0.193	0.495	1.363	0.501
6144	0.495	1.363	0.185	0.444	1.020	0.462	0.484	1.295	0.501
6145	0.224	-0.373	0.508	0.234	-0.336	0.309	0.142	-0.895	0.097
6146	0.489	1.329	0.501	0.444	1.020	0.462	0.511	1.464	0.481
6147	0.413	0.841	0.492	0.509	1.434	0.491	0.505	1.430	0.512
6148	0.508	1.447	0.492	0.497	1.359	0.501	0.489	1.329	0.450
6149	0.508	1.447	0.385	0.442	1.001	0.462	0.463	1.161	0.185
6150	0.332	0.318	0.328	0.196	-0.581	0.462	0.184	-0.625	0.224
6151	0.263	-0.120	0.296	0.102	-1.184	0.193	0.263	-0.120	0.050
6152	0.484	1.295	0.501	0.447	1.039	0.193	0.466	1.178	0.492
6153	0.432	0.958	0.431	0.500	1.378	0.512	0.429	0.942	0.224
6154	0.142	-0.895	0.431	0.374	0.568	0.491	0.050	-1.485	0.185
6155	0.329	0.301	0.142	0.193	-0.600	0.512	0.226	-0.356	0.467
6156	0.505	1.430	0.185	0.465	1.152	0.444	0.474	1.228	0.492
6157	0.297	0.099	0.508	0.310	0.153	0.235	0.295	0.082	0.185

6158	0.505	1.430	0.481	0.465	1.152	0.508	0.466	1.178	0.467
6159	0.511	1.464	0.450	0.491	1.321	0.235	0.445	1.043	0.409
6160	0.487	1.312	0.262	0.503	1.396	0.193	0.508	1.447	0.224
6161	0.511	1.464	0.296	0.465	1.152	0.235	0.461	1.144	0.450
6162	0.511	1.464	0.262	0.465	1.152	0.193	0.461	1.144	0.450
6163	0.226	−0.356	0.450	0.196	−0.581	0.462	0.224	−0.373	0.481
6164	0.263	−0.120	0.328	0.193	−0.600	0.508	0.097	−1.181	0.358
6165	0.503	1.413	0.508	0.512	1.453	0.501	0.429	0.942	0.467
6166	0.482	1.279	0.142	0.497	1.359	0.501	0.474	1.228	0.185
6167	0.500	1.397	0.467	0.515	1.472	0.462	0.511	1.464	0.492
6168	0.489	1.329	0.501	0.450	1.057	0.235	0.466	1.178	0.481
6169	0.226	−0.356	0.512	0.234	−0.336	0.235	0.184	−0.625	0.481
6170	0.413	0.841	0.296	0.506	1.415	0.235	0.508	1.447	0.224
6171	0.361	0.504	0.450	0.237	−0.317	0.371	0.142	−0.895	0.262
6172	0.295	0.082	0.431	0.199	−0.562	0.273	0.142	−0.895	0.431
6173	0.184	−0.625	0.512	0.234	−0.336	0.462	0.263	−0.120	0.508
6174	0.142	−0.895	0.492	0.193	−0.600	0.462	0.297	0.099	0.481
6175	0.445	1.043	0.492	0.465	1.152	0.273	0.497	1.380	0.185
6176	0.508	1.447	0.501	0.512	1.453	0.444	0.447	1.060	0.142
6177	0.445	1.043	0.185	0.500	1.378	0.193	0.492	1.346	0.097
6178	0.474	1.228	0.501	0.500	1.378	0.102	0.500	1.397	0.512
6179	0.489	1.329	0.512	0.465	1.152	0.273	0.463	1.161	0.262
6180	0.263	−0.120	0.513	0.234	−0.336	0.501	0.142	−0.895	0.385
6181	0.224	−0.373	0.492	0.234	−0.336	0.149	0.187	−0.608	0.142
6182	0.224	−0.373	0.508	0.234	−0.336	0.478	0.187	−0.608	0.481
6183	0.224	−0.373	0.097	0.371	0.549	0.235	0.300	0.116	0.467
6184	0.355	0.470	0.142	0.275	−0.073	0.462	0.184	−0.625	0.501
6185	0.432	0.958	0.501	0.465	1.152	0.235	0.503	1.413	0.328
6186	0.384	0.655	0.328	0.459	1.114	0.273	0.492	1.346	0.513
6187	0.295	0.082	0.512	0.272	−0.091	0.273	0.297	0.099	0.467
6188	0.511	1.464	0.512	0.439	0.982	0.149	0.505	1.430	0.467
6189	0.142	−0.895	0.224	0.193	−0.600	0.149	0.261	−0.137	0.512
6190	0.226	−0.356	0.224	0.102	−1.184	0.193	0.261	−0.137	0.450
6191	0.326	0.285	0.185	0.275	−0.073	0.273	0.361	0.504	0.467
6192	0.511	1.464	0.185	0.506	1.415	0.422	0.447	1.060	0.142
6193	0.142	−0.895	0.358	0.149	−0.882	0.501	0.361	0.504	0.358
6194	0.463	1.161	0.501	0.477	1.227	0.309	0.489	1.329	0.431
6195	0.097	−1.181	0.262	0.237	−0.317	0.501	0.295	0.082	0.431
6196	0.468	1.194	0.097	0.462	1.133	0.491	0.500	1.397	0.481
6197	0.329	0.301	0.492	0.234	−0.336	0.235	0.050	−1.485	0.481
6198	0.224	−0.373	0.224	0.272	−0.091	0.501	0.224	−0.373	0.296
6199	0.224	−0.373	0.224	0.272	−0.091	0.462	0.297	0.099	0.296
6200	0.329	0.301	0.481	0.149	−0.882	0.514	0.097	−1.181	0.467
6201	0.142	−0.895	0.501	0.149	−0.882	0.193	0.358	0.487	0.224
6202	0.297	0.099	0.431	0.193	−0.600	0.193	0.097	−1.181	0.481
6203	0.050	−1.485	0.224	0.275	−0.073	0.193	0.224	−0.373	0.450
6204	0.447	1.060	0.224	0.427	0.907	0.512	0.497	1.380	0.358
6205	0.492	1.346	0.481	0.503	1.396	0.309	0.503	1.413	0.358
6206	0.142	−0.895	0.481	0.310	0.153	0.193	0.184	−0.625	0.262
6207	0.479	1.262	0.328	0.503	1.396	0.491	0.497	1.380	0.431
6208	0.508	1.447	0.328	0.494	1.340	0.491	0.500	1.397	0.185
6209	0.224	−0.373	0.224	0.234	−0.336	0.422	0.097	−1.181	0.512
6210	0.429	0.942	0.328	0.503	1.396	0.478	0.476	1.245	0.142
6211	0.466	1.178	0.328	0.462	1.133	0.514	0.503	1.413	0.358
6212	0.479	1.262	0.185	0.512	1.453	0.149	0.445	1.043	0.501
6213	0.184	−0.625	0.508	0.193	−0.600	0.491	0.187	−0.608	0.142
6214	0.142	−0.895	0.508	0.196	−0.581	0.491	0.224	−0.373	0.296
6215	0.297	0.099	0.185	0.196	−0.581	0.398	0.050	−1.485	0.501
6216	0.511	1.464	0.481	0.518	1.491	0.508	0.495	1.363	0.467
6217	0.224	−0.373	0.185	0.310	0.153	0.514	0.224	−0.373	0.501
6218	0.224	−0.373	0.224	0.193	−0.600	0.193	0.184	−0.625	0.224

6219	0.479	1.262	0.050	0.485	1.283	0.149	0.466	1.178	0.224
6220	0.476	1.245	0.296	0.488	1.302	0.462	0.489	1.329	0.481
6221	0.187	-0.608	0.481	0.424	0.888	0.508	0.184	-0.625	0.481
6222	0.495	1.363	0.224	0.477	1.227	0.508	0.461	1.144	0.142
6223	0.511	1.464	0.358	0.506	1.415	0.309	0.405	0.790	0.185
6224	0.187	-0.608	0.224	0.152	-0.863	0.444	0.224	-0.373	0.185
6225	0.508	1.447	0.358	0.494	1.340	0.422	0.450	1.076	0.224
6226	0.500	1.397	0.224	0.488	1.302	0.501	0.445	1.043	0.328
6227	0.495	1.363	0.431	0.401	0.737	0.444	0.476	1.245	0.224
6228	0.492	1.346	0.328	0.503	1.396	0.149	0.358	0.487	0.508
6229	0.384	0.655	0.185	0.509	1.434	0.149	0.468	1.194	0.508
6230	0.332	0.318	0.328	0.193	-0.600	0.193	0.000	?	0.296
6231	0.300	0.116	0.142	0.149	-0.882	0.478	0.184	-0.625	0.481
6232	0.505	1.430	0.224	0.462	1.133	0.235	0.489	1.329	0.328
6233	0.429	0.942	0.097	0.509	1.434	0.512	0.482	1.279	0.296
6234	0.429	0.942	0.481	0.509	1.434	0.309	0.482	1.279	0.142
6235	0.142	-0.895	0.481	0.307	0.135	0.462	0.224	-0.373	0.050
6236	0.187	-0.608	0.431	0.442	1.001	0.501	0.266	-0.103	0.508
6237	0.511	1.464	0.328	0.427	0.907	0.508	0.426	0.925	0.050
6238	0.482	1.279	0.481	0.500	1.378	0.273	0.505	1.430	0.097
6239	0.455	1.110	0.467	0.444	1.020	0.508	0.476	1.245	0.142
6240	0.261	-0.137	0.224	0.149	-0.882	0.501	0.184	-0.625	0.142
6241	0.297	0.099	0.224	0.149	-0.882	0.462	0.142	-0.895	0.481
6242	0.263	-0.120	0.512	0.193	-0.600	0.508	0.097	-1.181	0.224
6243	0.445	1.043	0.050	0.474	1.208	0.508	0.511	1.464	0.185
6244	0.329	0.301	0.262	0.234	-0.336	0.508	0.263	-0.120	0.142
6245	0.503	1.413	0.481	0.512	1.453	0.273	0.382	0.638	0.296
6246	0.142	-0.895	0.262	0.313	0.172	0.478	0.142	-0.895	0.358
6247	0.468	1.194	0.492	0.462	1.133	0.444	0.474	1.228	0.296
6248	0.497	1.380	0.492	0.500	1.378	0.422	0.463	1.161	0.450
6249	0.511	1.464	0.224	0.506	1.415	0.398	0.497	1.380	0.492
6250	0.300	0.116	0.142	0.272	-0.091	0.508	0.329	0.301	0.501
6251	0.455	1.110	0.431	0.506	1.415	0.149	0.516	1.498	0.501
6252	0.429	0.942	0.508	0.500	1.378	0.149	0.466	1.178	0.481
6253	0.508	1.447	0.508	0.462	1.133	0.478	0.466	1.178	0.501
6254	0.492	1.346	0.185	0.503	1.396	0.371	0.513	1.481	0.296
6255	0.492	1.346	0.185	0.506	1.415	0.491	0.445	1.043	0.097
6256	0.500	1.397	0.142	0.506	1.415	0.491	0.461	1.144	0.501
6257	0.184	-0.625	0.409	0.278	-0.054	0.422	0.142	-0.895	0.262
6258	0.500	1.397	0.142	0.480	1.246	0.371	0.358	0.487	0.185
6259	0.511	1.464	0.492	0.444	1.020	0.235	0.429	0.942	0.508
6260	0.513	1.481	0.262	0.424	0.888	0.193	0.429	0.942	0.050
6261	0.495	1.363	0.185	0.398	0.718	0.235	0.482	1.279	0.431
6262	0.508	1.447	0.296	0.509	1.434	0.462	0.482	1.279	0.467
6263	0.097	-1.181	0.492	0.149	-0.882	0.235	0.295	0.082	0.142
6264	0.142	-0.895	0.492	0.149	-0.882	0.235	0.295	0.082	0.185
6265	0.500	1.397	0.492	0.480	1.246	0.478	0.463	1.161	0.185
6266	0.332	0.318	0.492	0.371	0.549	0.341	0.224	-0.373	0.467
6267	0.511	1.464	0.142	0.491	1.321	0.273	0.482	1.279	0.467
6268	0.508	1.447	0.262	0.488	1.302	0.478	0.447	1.060	0.185
6269	0.224	-0.373	0.262	0.424	0.888	0.508	0.358	0.487	0.185
6270	0.224	-0.373	0.262	0.374	0.568	0.512	0.358	0.487	0.142
6271	0.184	-0.625	0.262	0.234	-0.336	0.149	0.261	-0.137	0.508
6272	0.184	-0.625	0.224	0.196	-0.581	0.149	0.432	0.958	0.185
6273	0.355	0.470	0.450	0.237	-0.317	0.512	0.184	-0.625	0.513
6274	0.500	1.397	0.224	0.465	1.152	0.512	0.505	1.430	0.185
6275	0.261	-0.137	0.513	0.234	-0.336	0.273	0.142	-0.895	0.224
6276	0.097	-1.181	0.508	0.237	-0.317	0.273	0.358	0.487	0.097
6277	0.495	1.363	0.185	0.477	1.227	0.371	0.497	1.380	0.450
6278	0.224	-0.373	0.296	0.342	0.361	0.309	0.142	-0.895	0.492
6279	0.224	-0.373	0.296	0.272	-0.091	0.193	0.295	0.082	0.492

6280	0.476	1.245	0.467	0.477	1.227	0.149	0.497	1.380	0.513
6281	0.497	1.380	0.450	0.509	1.434	0.478	0.463	1.161	0.142
6282	0.434	0.975	0.450	0.509	1.434	0.491	0.508	1.447	0.185
6283	0.224	−0.373	0.358	0.149	−0.882	0.341	0.224	−0.373	0.050
6284	0.224	−0.373	0.328	0.149	−0.882	0.422	0.224	−0.373	0.142
6285	0.476	1.245	0.262	0.509	1.434	0.273	0.479	1.262	0.296
6286	0.476	1.245	0.501	0.509	1.434	0.273	0.479	1.262	0.450
6287	0.329	0.301	0.467	0.278	−0.054	0.341	0.142	−0.895	0.050
6288	0.329	0.301	0.481	0.278	−0.054	0.341	0.184	−0.625	0.501
6289	0.226	−0.356	0.450	0.371	0.549	0.462	0.184	−0.625	0.508
6290	0.332	0.318	0.224	0.307	0.135	0.309	0.226	−0.356	0.185
6291	0.329	0.301	0.224	0.196	−0.581	0.149	0.329	0.301	0.185
6292	0.187	−0.608	0.262	0.149	−0.882	0.273	0.224	−0.373	0.185
6293	0.505	1.430	0.224	0.477	1.227	0.149	0.503	1.413	0.508
6294	0.505	1.430	0.467	0.488	1.302	0.371	0.503	1.413	0.431
6295	0.187	−0.608	0.512	0.342	0.361	0.491	0.295	0.082	0.431
6296	0.482	1.279	0.467	0.424	0.888	0.235	0.474	1.228	0.185
6297	0.187	−0.608	0.262	0.275	−0.073	0.309	0.329	0.301	0.185
6298	0.224	−0.373	0.358	0.275	−0.073	0.193	0.295	0.082	0.501
6299	0.050	−1.485	0.481	0.345	0.379	0.102	0.142	−0.895	0.481
6300	0.295	0.082	0.296	0.342	0.361	0.193	0.050	−1.485	0.501
6301	0.482	1.279	0.142	0.462	1.133	0.149	0.508	1.447	0.492
6302	0.226	−0.356	0.385	0.310	0.153	0.514	0.050	−1.485	0.185
6303	0.355	0.470	0.296	0.149	−0.882	0.491	0.097	−1.181	0.224
6304	0.224	−0.373	0.224	0.272	−0.091	0.512	0.142	−0.895	0.224
6305	0.361	0.504	0.467	0.149	−0.882	0.508	0.142	−0.895	0.142
6306	0.226	−0.356	0.262	0.371	0.549	0.462	0.000	?	0.501
6307	0.432	0.958	0.431	0.491	1.321	0.462	0.476	1.245	0.481
6308	0.329	0.301	0.185	0.234	−0.336	0.309	0.226	−0.356	0.501
6309	0.187	−0.608	0.481	0.310	0.153	0.235	0.184	−0.625	0.097
6310	0.329	0.301	0.185	0.193	−0.600	0.508	0.142	−0.895	0.185
6311	0.142	−0.895	0.262	0.102	−1.184	0.235	0.297	0.099	0.481
6312	0.224	−0.373	0.185	0.193	−0.600	0.149	0.361	0.504	0.142
6313	0.097	−1.181	0.328	0.149	−0.882	0.478	0.297	0.099	0.185
6314	0.476	1.245	0.262	0.512	1.453	0.309	0.450	1.076	0.142
6315	0.476	1.245	0.224	0.491	1.321	0.478	0.489	1.329	0.142
6316	0.432	0.958	0.142	0.509	1.434	0.501	0.497	1.380	0.467
6317	0.332	0.318	0.467	0.503	1.396	0.235	0.497	1.380	0.224
6318	0.484	1.295	0.185	0.459	1.114	0.273	0.476	1.245	0.481
6319	0.463	1.161	0.385	0.462	1.133	0.193	0.503	1.413	0.224
6320	0.224	−0.373	0.467	0.310	0.153	0.491	0.297	0.099	0.492
6321	0.226	−0.356	0.467	0.234	−0.336	0.478	0.097	−1.181	0.097
6322	0.511	1.464	0.450	0.509	1.434	0.193	0.495	1.363	0.328
6323	0.050	−1.485	0.450	0.234	−0.336	0.193	0.261	−0.137	0.224
6324	0.263	−0.120	0.224	0.149	−0.882	0.398	0.187	−0.608	0.142
6325	0.482	1.279	0.508	0.477	1.227	0.508	0.500	1.397	0.142
6326	0.261	−0.137	0.513	0.307	0.135	0.235	0.050	−1.485	0.142
6327	0.489	1.329	0.224	0.485	1.283	0.398	0.429	0.942	0.262
6328	0.492	1.346	0.492	0.506	1.415	0.102	0.463	1.161	0.481
6329	0.224	−0.373	0.431	0.237	−0.317	0.193	0.142	−0.895	0.185
6330	0.145	−0.878	0.467	0.272	−0.091	0.235	0.184	−0.625	0.501
6331	0.429	0.942	0.358	0.193	−0.600	0.478	0.187	−0.608	0.512
6332	0.503	1.413	0.185	0.494	1.340	0.501	0.461	1.144	0.512
6333	0.505	1.430	0.512	0.477	1.227	0.444	0.468	1.194	0.467
6334	0.187	−0.608	0.097	0.193	−0.600	0.462	0.184	−0.625	0.512
6335	0.187	−0.608	0.358	0.193	−0.600	0.398	0.184	−0.625	0.050
6336	0.142	−0.895	0.467	0.401	0.737	0.273	0.145	−0.878	0.481
6337	0.411	0.824	0.262	0.509	1.434	0.309	0.503	1.413	0.492
6338	0.142	−0.895	0.185	0.234	−0.336	0.273	0.187	−0.608	0.185
6339	0.487	1.312	0.467	0.401	0.737	0.273	0.505	1.430	0.501
6340	0.261	−0.137	0.328	0.102	−1.184	0.273	0.184	−0.625	0.501

6341	0.184	-0.625	0.185	0.193	-0.600	0.512	0.226	-0.356	0.508
6342	0.295	0.082	0.467	0.234	-0.336	0.309	0.097	-1.181	0.508
6343	0.495	1.363	0.492	0.477	1.227	0.478	0.445	1.043	0.262
6344	0.492	1.346	0.508	0.500	1.378	0.398	0.492	1.346	0.512
6345	0.495	1.363	0.512	0.442	1.001	0.273	0.492	1.346	0.097
6346	0.495	1.363	0.296	0.459	1.114	0.235	0.513	1.481	0.050
6347	0.142	-0.895	0.328	0.401	0.737	0.235	0.000	?	0.512
6348	0.261	-0.137	0.185	0.275	-0.073	0.462	0.142	-0.895	0.050
6349	0.266	-0.103	0.224	0.310	0.153	0.371	0.184	-0.625	0.224
6350	0.263	-0.120	0.224	0.272	-0.091	0.371	0.050	-1.485	0.467
6351	0.266	-0.103	0.262	0.272	-0.091	0.149	0.142	-0.895	0.142
6352	0.224	-0.373	0.224	0.275	-0.073	0.149	0.297	0.099	0.185
6353	0.450	1.076	0.224	0.512	1.453	0.149	0.445	1.043	0.508
6354	0.224	-0.373	0.262	0.310	0.153	0.398	0.050	-1.485	0.467
6355	0.508	1.447	0.467	0.480	1.246	0.478	0.495	1.363	0.492
6356	0.503	1.413	0.328	0.398	0.718	0.512	0.503	1.413	0.481
6357	0.187	-0.608	0.385	0.272	-0.091	0.512	0.184	-0.625	0.050
6358	0.297	0.099	0.508	0.234	-0.336	0.273	0.184	-0.625	0.142
6359	0.297	0.099	0.296	0.234	-0.336	0.309	0.184	-0.625	0.224
6360	0.463	1.161	0.262	0.465	1.152	0.193	0.503	1.413	0.097
6361	0.450	1.076	0.142	0.371	0.549	0.235	0.426	0.925	0.050
6362	0.450	1.076	0.142	0.371	0.549	0.491	0.426	0.925	0.142
6363	0.361	0.504	0.296	0.149	-0.882	0.512	0.000	?	0.097
6364	0.326	0.285	0.224	0.149	-0.882	0.491	0.184	-0.625	0.097
6365	0.261	-0.137	0.262	0.149	-0.882	0.235	0.184	-0.625	0.185
6366	0.500	1.397	0.185	0.398	0.718	0.444	0.497	1.380	0.508
6367	0.463	1.161	0.501	0.477	1.227	0.444	0.479	1.262	0.142
6368	0.474	1.228	0.512	0.512	1.453	0.235	0.500	1.397	0.050
6369	0.447	1.060	0.501	0.512	1.453	0.309	0.487	1.312	0.501
6370	0.224	-0.373	0.358	0.272	-0.091	0.309	0.184	-0.625	0.050
6371	0.224	-0.373	0.409	0.313	0.172	0.235	0.226	-0.356	0.050
6372	0.263	-0.120	0.358	0.193	-0.600	0.273	0.224	-0.373	0.142
6373	0.224	-0.373	0.328	0.237	-0.317	0.478	0.142	-0.895	0.050
6374	0.468	1.194	0.296	0.488	1.302	0.102	0.495	1.363	0.142
6375	0.511	1.464	0.224	0.515	1.472	0.512	0.476	1.245	0.097
6376	0.468	1.194	0.185	0.491	1.321	0.273	0.495	1.363	0.142
6377	0.261	-0.137	0.185	0.234	-0.336	0.478	0.097	-1.181	0.142
6378	0.361	0.504	0.142	0.442	1.001	0.273	0.184	-0.625	0.501
6379	0.482	1.279	0.185	0.444	1.020	0.371	0.474	1.228	0.492
6380	0.295	0.082	0.512	0.234	-0.336	0.149	0.000	?	0.501
6381	0.142	-0.895	0.512	0.307	0.135	0.149	0.142	-0.895	0.097
6382	0.387	0.672	0.185	0.310	0.153	0.193	0.184	-0.625	0.492
6383	0.297	0.099	0.328	0.237	-0.317	0.193	0.142	-0.895	0.097
6384	0.226	-0.356	0.481	0.275	-0.073	0.193	0.142	-0.895	0.224
6385	0.461	1.144	0.512	0.480	1.246	0.444	0.463	1.161	0.142
6386	0.266	-0.103	0.262	0.102	-1.184	0.193	0.224	-0.373	0.097
6387	0.432	0.958	0.262	0.512	1.453	0.514	0.476	1.245	0.142
6388	0.184	-0.625	0.296	0.275	-0.073	0.478	0.224	-0.373	0.050
6389	0.482	1.279	0.492	0.474	1.208	0.478	0.489	1.329	0.097
6390	0.184	-0.625	0.450	0.275	-0.073	0.512	0.097	-1.181	0.512
6391	0.263	-0.120	0.450	0.374	0.568	0.398	0.326	0.285	0.512
6392	0.184	-0.625	0.501	0.149	-0.882	0.341	0.224	-0.373	0.185
6393	0.329	0.301	0.481	0.149	-0.882	0.422	0.142	-0.895	0.050
6394	0.263	-0.120	0.296	0.196	-0.581	0.508	0.142	-0.895	0.508
6395	0.226	-0.356	0.185	0.196	-0.581	0.273	0.142	-0.895	0.508
6396	0.142	-0.895	0.185	0.193	-0.600	0.478	0.263	-0.120	0.224
6397	0.463	1.161	0.512	0.444	1.020	0.514	0.476	1.245	0.224
6398	0.184	-0.625	0.513	0.196	-0.581	0.478	0.187	-0.608	0.097
6399	0.384	0.655	0.224	0.515	1.472	0.462	0.495	1.363	0.512
6400	0.463	1.161	0.185	0.480	1.246	0.102	0.508	1.447	0.492
6401	0.463	1.161	0.512	0.480	1.246	0.422	0.508	1.447	0.481

6402	0.447	1.060	0.431	0.515	1.472	0.371	0.461	1.144	0.501
6403	0.453	1.093	0.431	0.398	0.718	0.102	0.508	1.447	0.501
6404	0.226	−0.356	0.224	0.342	0.361	0.512	0.050	−1.485	0.358
6405	0.508	1.447	0.296	0.421	0.869	0.273	0.476	1.245	0.358
6406	0.516	1.498	0.508	0.503	1.396	0.149	0.495	1.363	0.358
6407	0.224	−0.373	0.328	0.275	−0.073	0.501	0.187	−0.608	0.492
6408	0.495	1.363	0.328	0.474	1.208	0.235	0.497	1.380	0.467
6409	0.432	0.958	0.328	0.512	1.453	0.193	0.500	1.397	0.142
6410	0.466	1.178	0.142	0.480	1.246	0.444	0.500	1.397	0.262
6411	0.366	0.537	0.185	0.456	1.095	0.501	0.500	1.397	0.492
6412	0.184	−0.625	0.262	0.102	−1.184	0.501	0.261	−0.137	0.508
6413	0.511	1.464	0.481	0.424	0.888	0.444	0.503	1.413	0.512
6414	0.100	−1.164	0.481	0.371	0.549	0.193	0.097	−1.181	0.097
6415	0.361	0.504	0.224	0.102	−1.184	0.273	0.050	−1.485	0.185
6416	0.468	1.194	0.492	0.509	1.434	0.341	0.505	1.430	0.450
6417	0.263	−0.120	0.481	0.275	−0.073	0.309	0.050	−1.485	0.097
6418	0.184	−0.625	0.358	0.149	−0.882	0.273	0.224	−0.373	0.097
6419	0.463	1.161	0.185	0.500	1.378	0.341	0.466	1.178	0.097
6420	0.326	0.285	0.224	0.237	−0.317	0.341	0.142	−0.895	0.097
6421	0.184	−0.625	0.224	0.193	−0.600	0.273	0.184	−0.625	0.142
6422	0.468	1.194	0.501	0.444	1.020	0.193	0.500	1.397	0.492
6423	0.489	1.329	0.467	0.497	1.359	0.478	0.463	1.161	0.501
6424	0.505	1.430	0.262	0.500	1.378	0.235	0.489	1.329	0.097
6425	0.508	1.447	0.501	0.442	1.001	0.193	0.476	1.245	0.513
6426	0.297	0.099	0.501	0.196	−0.581	0.422	0.050	−1.485	0.513
6427	0.329	0.301	0.513	0.278	−0.054	0.235	0.142	−0.895	0.296
6428	0.187	−0.608	0.513	0.339	0.342	0.398	0.224	−0.373	0.142
6429	0.226	−0.356	0.512	0.307	0.135	0.273	0.097	−1.181	0.185
6430	0.224	−0.373	0.224	0.272	−0.091	0.341	0.050	−1.485	0.142
6431	0.263	−0.120	0.508	0.342	0.361	0.273	0.142	−0.895	0.513
6432	0.226	−0.356	0.512	0.342	0.361	0.235	0.097	−1.181	0.501
6433	0.224	−0.373	0.467	0.275	−0.073	0.193	0.097	−1.181	0.262
6434	0.261	−0.137	0.296	0.193	−0.600	0.235	0.184	−0.625	0.512
6435	0.463	1.161	0.508	0.480	1.246	0.478	0.503	1.413	0.492
6436	0.326	0.285	0.097	0.240	−0.299	0.508	0.142	−0.895	0.492
6437	0.387	0.672	0.050	0.193	−0.600	0.501	0.050	−1.485	0.492
6438	0.505	1.430	0.512	0.421	0.869	0.193	0.497	1.380	0.501
6439	0.297	0.099	0.050	0.237	−0.317	0.501	0.050	−1.485	0.262
6440	0.261	−0.137	0.512	0.404	0.756	0.235	0.050	−1.485	0.508
6441	0.142	−0.895	0.512	0.275	−0.073	0.341	0.142	−0.895	0.512
6442	0.142	−0.895	0.185	0.342	0.361	0.193	0.050	−1.485	0.512
6443	0.297	0.099	0.050	0.275	−0.073	0.235	0.142	−0.895	0.097
6444	0.226	−0.356	0.508	0.237	−0.317	0.341	0.097	−1.181	0.481
6445	0.263	−0.120	0.296	0.196	−0.581	0.309	0.142	−0.895	0.296
6446	0.184	−0.625	0.224	0.237	−0.317	0.341	0.142	−0.895	0.296
6447	0.503	1.413	0.512	0.480	1.246	0.273	0.495	1.363	0.431
6448	0.511	1.464	0.508	0.506	1.415	0.508	0.487	1.312	0.262
6449	0.495	1.363	0.513	0.500	1.378	0.508	0.500	1.397	0.450
6450	0.358	0.487	0.501	0.196	−0.581	0.341	0.097	−1.181	0.501
6451	0.405	0.790	0.508	0.503	1.396	0.273	0.487	1.312	0.431
6452	0.358	0.487	0.512	0.240	−0.299	0.508	0.097	−1.181	0.409
6453	0.329	0.301	0.501	0.342	0.361	0.508	0.000	?	0.508
6454	0.297	0.099	0.513	0.196	−0.581	0.422	0.226	−0.356	0.296
6455	0.226	−0.356	0.513	0.234	−0.336	0.422	0.142	−0.895	0.224
6456	0.184	−0.625	0.501	0.345	0.379	0.235	0.097	−1.181	0.492
6457	0.184	−0.625	0.481	0.313	0.172	0.508	0.142	−0.895	0.508
6458	0.142	−0.895	0.492	0.345	0.379	0.514	0.050	−1.485	0.508
6459	0.184	−0.625	0.501	0.275	−0.073	0.478	0.097	−1.181	0.467
6460	0.508	1.447	0.481	0.509	1.434	0.478	0.508	1.447	0.467
6461	0.508	1.447	0.450	0.509	1.434	0.508	0.508	1.447	0.508
6462	0.184	−0.625	0.450	0.342	0.361	0.102	0.184	−0.625	0.508

6463	0.326	0.285	0.097	0.272	-0.091	0.273	0.050	-1.485	0.450
6464	0.479	1.262	0.508	0.503	1.396	0.273	0.511	1.464	0.512
6465	0.511	1.464	0.467	0.503	1.396	0.462	0.511	1.464	0.492
6466	0.263	-0.120	0.467	0.424	0.888	0.398	0.224	-0.373	0.481
6467	0.263	-0.120	0.467	0.424	0.888	0.273	0.224	-0.373	0.492
6468	0.297	0.099	0.508	0.234	-0.336	0.102	0.097	-1.181	0.492
6469	0.492	1.346	0.467	0.506	1.415	0.501	0.505	1.430	0.501
6470	0.445	1.043	0.512	0.512	1.453	0.501	0.492	1.346	0.512
6471	0.450	1.076	0.512	0.477	1.227	0.508	0.476	1.245	0.512
6472	0.497	1.380	0.467	0.477	1.227	0.235	0.503	1.413	0.358
6473	0.479	1.262	0.513	0.503	1.396	0.102	0.503	1.413	0.142
6474	0.295	0.082	0.501	0.105	-1.165	0.508	0.358	0.487	0.481
6475	0.184	-0.625	0.512	0.275	-0.073	0.102	0.361	0.504	0.450
6476	0.184	-0.625	0.512	0.275	-0.073	0.102	0.361	0.504	0.467
6477	0.511	1.464	0.512	0.462	1.133	0.102	0.489	1.329	0.467
6478	0.508	1.447	0.513	0.404	0.756	0.341	0.468	1.194	0.481
6479	0.224	-0.373	0.450	0.272	-0.091	0.235	0.142	-0.895	0.508
6480	0.184	-0.625	0.508	0.102	-1.184	0.273	0.263	-0.120	0.467
6481	0.513	1.481	0.097	0.500	1.378	0.491	0.492	1.346	0.492
6482	0.426	0.925	0.513	0.500	1.378	0.491	0.503	1.413	0.501
6483	0.429	0.942	0.508	0.509	1.434	0.235	0.508	1.447	0.481
6484	0.226	-0.356	0.481	0.234	-0.336	0.491	0.097	-1.181	0.492
6485	0.297	0.099	0.512	0.102	-1.184	0.478	0.187	-0.608	0.501
6486	0.505	1.430	0.097	0.506	1.415	0.053	0.453	1.093	0.512
6487	0.332	0.318	0.508	0.102	-1.184	0.341	0.097	-1.181	0.512
6488	0.332	0.318	0.501	0.102	-1.184	0.341	0.097	-1.181	0.467
6489	0.332	0.318	0.501	0.102	-1.184	0.193	0.097	-1.181	0.481
6490	0.142	-0.895	0.501	0.345	0.379	0.501	0.097	-1.181	0.501
6491	0.184	-0.625	0.508	0.237	-0.317	0.514	0.142	-0.895	0.358
6492	0.261	-0.137	0.508	0.272	-0.091	0.053	0.000	?	0.512
6493	0.479	1.262	0.508	0.491	1.321	0.514	0.492	1.346	0.467
6494	0.479	1.262	0.508	0.491	1.321	0.491	0.500	1.397	0.513
6495	0.224	-0.373	0.508	0.234	-0.336	0.512	0.100	-1.164	0.501
6496	0.489	1.329	0.508	0.488	1.302	0.512	0.508	1.447	0.224
6497	0.479	1.262	0.508	0.477	1.227	0.512	0.508	1.447	0.501
6498	0.358	0.487	0.492	0.053	-1.504	0.235	0.297	0.099	0.512
6499	0.184	-0.625	0.481	0.345	0.379	0.514	0.142	-0.895	0.431
6500	0.226	-0.356	0.224	0.345	0.379	0.508	0.184	-0.625	0.385
6501	0.226	-0.356	0.501	0.196	-0.581	0.501	0.142	-0.895	0.481
6502	0.497	1.380	0.492	0.497	1.359	0.309	0.513	1.481	0.501
6503	0.461	1.144	0.513	0.518	1.491	0.508	0.503	1.413	0.508
6504	0.261	-0.137	0.513	0.053	-1.504	0.371	0.266	-0.103	0.508
6505	0.503	1.413	0.492	0.518	1.491	0.273	0.516	1.498	0.508
6506	0.503	1.413	0.492	0.488	1.302	0.501	0.495	1.363	0.508
6507	0.508	1.447	0.501	0.512	1.453	0.235	0.495	1.363	0.508
6508	0.508	1.447	0.501	0.512	1.453	0.512	0.495	1.363	0.512
6509	0.508	1.447	0.501	0.512	1.453	0.444	0.503	1.413	0.512
6510	0.224	-0.373	0.185	0.237	-0.317	0.341	0.263	-0.120	0.185
6511	0.508	1.447	0.508	0.509	1.434	0.309	0.503	1.413	0.492
6512	0.513	1.481	0.224	0.503	1.396	0.491	0.508	1.447	0.481
6513	0.463	1.161	0.224	0.503	1.396	0.149	0.513	1.481	0.508
6514	0.297	0.099	0.512	0.307	0.135	0.309	0.100	-1.164	0.513
6515	0.505	1.430	0.450	0.506	1.415	0.512	0.479	1.262	0.501
6516	0.097	-1.181	0.508	0.374	0.568	0.501	0.297	0.099	0.501
6517	0.050	-1.485	0.512	0.275	-0.073	0.478	0.297	0.099	0.512
6518	0.503	1.413	0.501	0.503	1.396	0.512	0.429	0.942	0.512
6519	0.050	-1.485	0.508	0.237	-0.317	0.512	0.263	-0.120	0.097
6520	0.511	1.464	0.501	0.512	1.453	0.478	0.450	1.076	0.501
6521	0.511	1.464	0.492	0.444	1.020	0.462	0.497	1.380	0.185
6522	0.187	-0.608	0.481	0.342	0.361	0.514	0.432	0.958	0.508
6523	0.050	-1.485	0.512	0.307	0.135	0.501	0.405	0.790	0.296

6524	0.505	1.430	0.142	0.497	1.359	0.514	0.511	1.464	0.224
6525	0.297	0.099	0.142	0.149	-0.882	0.514	0.300	0.116	0.513
6526	0.226	-0.356	0.512	0.310	0.153	0.512	0.226	-0.356	0.513
6527	0.508	1.447	0.508	0.515	1.472	0.491	0.484	1.295	0.467
6528	0.505	1.430	0.512	0.500	1.378	0.422	0.503	1.413	0.501
6529	0.511	1.464	0.501	0.477	1.227	0.512	0.503	1.413	0.501
6530	0.497	1.380	0.512	0.518	1.491	0.512	0.466	1.178	0.501
6531	0.505	1.430	0.467	0.518	1.491	0.193	0.466	1.178	0.501
6532	0.508	1.447	0.467	0.482	1.265	0.309	0.508	1.447	0.450
6533	0.497	1.380	0.467	0.468	1.170	0.501	0.508	1.447	0.450
6534	0.503	1.413	0.467	0.518	1.491	0.491	0.447	1.060	0.501
6535	0.511	1.464	0.467	0.497	1.359	0.491	0.508	1.447	0.142
6536	0.497	1.380	0.142	0.515	1.472	0.491	0.489	1.329	0.142
6537	0.479	1.262	0.501	0.515	1.472	0.491	0.479	1.262	0.508
6538	0.489	1.329	0.481	0.512	1.453	0.501	0.489	1.329	0.450
6539	0.503	1.413	0.450	0.494	1.340	0.508	0.495	1.363	0.501
6540	0.479	1.262	0.467	0.424	0.888	0.444	0.505	1.430	0.481
6541	0.445	1.043	0.481	0.515	1.472	0.512	0.518	1.514	0.492
6542	0.445	1.043	0.467	0.515	1.472	0.514	0.518	1.514	0.501
6543	0.000	?	0.492	0.193	-0.600	0.512	0.361	0.504	0.501
6544	0.100	-1.164	0.501	0.307	0.135	0.462	0.142	-0.895	0.501
6545	0.503	1.413	0.501	0.497	1.359	0.512	0.482	1.279	0.512
6546	0.463	1.161	0.431	0.494	1.340	0.512	0.450	1.076	0.501
6547	0.463	1.161	0.501	0.494	1.340	0.508	0.466	1.178	0.224
6548	0.463	1.161	0.501	0.494	1.340	0.422	0.466	1.178	0.508
6549	0.503	1.413	0.481	0.488	1.302	0.491	0.479	1.262	0.467
6550	0.463	1.161	0.508	0.500	1.378	0.444	0.508	1.447	0.513
6551	0.508	1.447	0.512	0.503	1.396	0.462	0.468	1.194	0.508
6552	0.508	1.447	0.512	0.447	1.039	0.478	0.484	1.295	0.492
6553	0.468	1.194	0.501	0.512	1.453	0.514	0.503	1.413	0.508
6554	0.511	1.464	0.501	0.515	1.472	0.508	0.476	1.245	0.501
6555	0.495	1.363	0.501	0.518	1.491	0.235	0.489	1.329	0.508
6556	0.505	1.430	0.296	0.462	1.133	0.508	0.503	1.413	0.142
6557	0.503	1.413	0.385	0.515	1.472	0.508	0.511	1.464	0.492
6558	0.503	1.413	0.501	0.515	1.472	0.512	0.511	1.464	0.501
6559	0.511	1.464	0.492	0.506	1.415	0.512	0.461	1.144	0.467
6560	0.447	1.060	0.358	0.421	0.869	0.512	0.479	1.262	0.513
6561	0.508	1.447	0.512	0.485	1.283	0.462	0.497	1.380	0.513
6562	0.097	-1.181	0.513	0.442	1.001	0.514	0.358	0.487	0.512
6563	0.513	1.481	0.224	0.462	1.133	0.508	0.508	1.447	0.481
6564	0.508	1.447	0.513	0.477	1.227	0.514	0.471	1.211	0.450
6565	0.479	1.262	0.513	0.509	1.434	0.514	0.513	1.481	0.467
6566	0.513	1.481	0.467	0.506	1.415	0.514	0.497	1.380	0.508
6567	0.097	-1.181	0.450	0.240	-0.299	0.478	0.224	-0.373	0.508
6568	0.497	1.380	0.513	0.509	1.434	0.478	0.503	1.413	0.262
6569	0.503	1.413	0.508	0.509	1.434	0.235	0.508	1.447	0.328
6570	0.500	1.397	0.513	0.518	1.491	0.512	0.429	0.942	0.512
6571	0.500	1.397	0.492	0.518	1.491	0.512	0.382	0.638	0.481
6572	0.505	1.430	0.508	0.515	1.472	0.512	0.484	1.295	0.296
6573	0.508	1.447	0.501	0.465	1.152	0.512	0.503	1.413	0.481
6574	0.505	1.430	0.185	0.515	1.472	0.512	0.505	1.430	0.492
6575	0.505	1.430	0.492	0.509	1.434	0.512	0.505	1.430	0.097
6576	0.505	1.430	0.492	0.515	1.472	0.512	0.505	1.430	0.467
6577	0.505	1.430	0.492	0.515	1.472	0.341	0.505	1.430	0.467
6578	0.505	1.430	0.513	0.515	1.472	0.398	0.505	1.430	0.508
6579	0.489	1.329	0.492	0.477	1.227	0.478	0.508	1.447	0.450
6580	0.479	1.262	0.492	0.477	1.227	0.193	0.508	1.447	0.508
6581	0.226	-0.356	0.512	0.237	-0.317	0.508	0.184	-0.625	0.508
6582	0.503	1.413	0.508	0.509	1.434	0.235	0.492	1.346	0.492
6583	0.492	1.346	0.262	0.509	1.434	0.273	0.479	1.262	0.513
6584	0.511	1.464	0.513	0.512	1.453	0.478	0.503	1.413	0.508

6585	0.511	1.464	0.501	0.512	1.453	0.512	0.508	1.447	0.358
6586	0.492	1.346	0.512	0.509	1.434	0.491	0.500	1.397	0.385
6587	0.492	1.346	0.512	0.509	1.434	0.491	0.500	1.397	0.512
6588	0.503	1.413	0.501	0.509	1.434	0.478	0.513	1.481	0.358
6589	0.500	1.397	0.508	0.342	0.361	0.478	0.513	1.481	0.358
6590	0.000	?	0.501	0.401	0.737	0.478	0.097	-1.181	0.501
6591	0.503	1.413	0.492	0.482	1.265	0.508	0.497	1.380	0.512
6592	0.187	-0.608	0.431	0.196	-0.581	0.501	0.184	-0.625	0.512
6593	0.511	1.464	0.501	0.512	1.453	0.491	0.505	1.430	0.450
6594	0.226	-0.356	0.501	0.237	-0.317	0.309	0.297	0.099	0.467
6595	0.226	-0.356	0.512	0.275	-0.073	0.309	0.226	-0.356	0.050
6596	0.511	1.464	0.501	0.482	1.265	0.512	0.503	1.413	0.513
6597	0.445	1.043	0.185	0.509	1.434	0.491	0.511	1.464	0.481
6598	0.513	1.481	0.481	0.491	1.321	0.422	0.466	1.178	0.296
6599	0.511	1.464	0.431	0.491	1.321	0.508	0.503	1.413	0.450
6600	0.500	1.397	0.450	0.480	1.246	0.462	0.492	1.346	0.492
6601	0.505	1.430	0.450	0.480	1.246	0.512	0.492	1.346	0.492
6602	0.500	1.397	0.467	0.480	1.246	0.512	0.492	1.346	0.508
6603	0.487	1.312	0.501	0.512	1.453	0.512	0.450	1.076	0.508
6604	0.482	1.279	0.492	0.506	1.415	0.514	0.453	1.093	0.467
6605	0.511	1.464	0.467	0.485	1.283	0.514	0.500	1.397	0.512
6606	0.142	-0.895	0.467	0.313	0.172	0.193	0.142	-0.895	0.492
6607	0.142	-0.895	0.512	0.313	0.172	0.508	0.142	-0.895	0.450
6608	0.511	1.464	0.224	0.509	1.434	0.501	0.503	1.413	0.385
6609	0.503	1.413	0.481	0.494	1.340	0.398	0.455	1.110	0.467
6610	0.511	1.464	0.262	0.424	0.888	0.462	0.500	1.397	0.409
6611	0.500	1.397	0.492	0.509	1.434	0.512	0.479	1.262	0.481
6612	0.511	1.464	0.501	0.468	1.170	0.512	0.484	1.295	0.492
6613	0.466	1.178	0.508	0.512	1.453	0.508	0.500	1.397	0.508
6614	0.466	1.178	0.508	0.512	1.453	0.501	0.500	1.397	0.512
6615	0.466	1.178	0.431	0.512	1.453	0.422	0.500	1.397	0.512
6616	0.463	1.161	0.492	0.515	1.472	0.508	0.516	1.498	0.501
6617	0.463	1.161	0.296	0.515	1.472	0.512	0.505	1.430	0.481
6618	0.145	-0.878	0.050	0.196	-0.581	0.512	0.224	-0.373	0.512
6619	0.497	1.380	0.467	0.506	1.415	0.501	0.508	1.447	0.501
6620	0.474	1.228	0.481	0.500	1.378	0.478	0.466	1.178	0.467
6621	0.453	1.093	0.508	0.398	0.718	0.478	0.511	1.464	0.296
6622	0.468	1.194	0.512	0.465	1.152	0.512	0.508	1.447	0.492
6623	0.482	1.279	0.508	0.509	1.434	0.501	0.492	1.346	0.142
6624	0.471	1.211	0.185	0.509	1.434	0.501	0.503	1.413	0.492
6625	0.492	1.346	0.050	0.503	1.396	0.508	0.497	1.380	0.492
6626	0.500	1.397	0.513	0.497	1.359	0.508	0.503	1.413	0.508
6627	0.000	?	0.501	0.418	0.850	0.235	0.142	-0.895	0.492
6628	0.492	1.346	0.508	0.503	1.396	0.273	0.497	1.380	0.512
6629	0.426	0.925	0.358	0.518	1.491	0.491	0.500	1.397	0.512
6630	0.497	1.380	0.501	0.512	1.453	0.501	0.461	1.144	0.185
6631	0.497	1.380	0.501	0.500	1.378	0.235	0.508	1.447	0.409
6632	0.479	1.262	0.501	0.477	1.227	0.491	0.513	1.481	0.481
6633	0.508	1.447	0.501	0.480	1.246	0.491	0.505	1.430	0.481
6634	0.508	1.447	0.501	0.509	1.434	0.371	0.479	1.262	0.508
6635	0.508	1.447	0.492	0.497	1.359	0.508	0.447	1.060	0.467
6636	0.497	1.380	0.512	0.500	1.378	0.508	0.463	1.161	0.492
6637	0.497	1.380	0.185	0.506	1.415	0.444	0.505	1.430	0.185
6638	0.497	1.380	0.185	0.506	1.415	0.512	0.505	1.430	0.385
6639	0.297	0.099	0.513	0.237	-0.317	0.444	0.263	-0.120	0.501
6640	0.387	0.672	0.513	0.275	-0.073	0.514	0.329	0.301	0.481
6641	0.495	1.363	0.513	0.485	1.283	0.512	0.513	1.481	0.467
6642	0.489	1.329	0.512	0.503	1.396	0.501	0.476	1.245	0.224
6643	0.358	0.487	0.492	0.234	-0.336	0.444	0.295	0.082	0.409
6644	0.508	1.447	0.262	0.491	1.321	0.512	0.479	1.262	0.450
6645	0.511	1.464	0.512	0.485	1.283	0.102	0.489	1.329	0.450

6646	0.226	−0.356	0.481	0.374	0.568	0.422	0.097	−1.181	0.481
6647	0.503	1.413	0.097	0.512	1.453	0.514	0.466	1.178	0.481
6648	0.503	1.413	0.358	0.512	1.453	0.514	0.466	1.178	0.501
6649	0.471	1.211	0.050	0.444	1.020	0.444	0.508	1.447	0.501
6650	0.453	1.093	0.409	0.512	1.453	0.508	0.450	1.076	0.262
6651	0.513	1.481	0.409	0.444	1.020	0.508	0.508	1.447	0.262
6652	0.503	1.413	0.431	0.515	1.472	0.514	0.503	1.413	0.512
6653	0.511	1.464	0.481	0.512	1.453	0.512	0.489	1.329	0.508
6654	0.492	1.346	0.467	0.497	1.359	0.235	0.513	1.481	0.513
6655	0.505	1.430	0.492	0.442	1.001	0.491	0.503	1.413	0.431
6656	0.500	1.397	0.492	0.515	1.472	0.508	0.355	0.470	0.512
6657	0.184	−0.625	0.467	0.102	−1.184	0.309	0.387	0.672	0.185
6658	0.489	1.329	0.501	0.421	0.869	0.514	0.508	1.447	0.513
6659	0.492	1.346	0.142	0.518	1.491	0.491	0.355	0.470	0.467
6660	0.492	1.346	0.185	0.518	1.491	0.478	0.355	0.470	0.296
6661	0.503	1.413	0.358	0.447	1.039	0.491	0.503	1.413	0.097
6662	0.492	1.346	0.431	0.503	1.396	0.514	0.511	1.464	0.328
6663	0.492	1.346	0.185	0.503	1.396	0.491	0.511	1.464	0.512
6664	0.505	1.430	0.224	0.515	1.472	0.478	0.453	1.093	0.512
6665	0.505	1.430	0.224	0.515	1.472	0.508	0.471	1.211	0.512
6666	0.263	−0.120	0.467	0.237	−0.317	0.444	0.050	−1.485	0.501
6667	0.503	1.413	0.508	0.488	1.302	0.491	0.516	1.498	0.513
6668	0.492	1.346	0.142	0.506	1.415	0.508	0.484	1.295	0.492
6669	0.000	?	0.185	0.310	0.153	0.309	0.297	0.099	0.513
6670	0.500	1.397	0.501	0.515	1.472	0.444	0.455	1.110	0.481
6671	0.500	1.397	0.501	0.494	1.340	0.491	0.492	1.346	0.409
6672	0.500	1.397	0.481	0.471	1.189	0.462	0.492	1.346	0.262
6673	0.505	1.430	0.513	0.485	1.283	0.514	0.508	1.447	0.296
6674	0.500	1.397	0.508	0.506	1.415	0.491	0.508	1.447	0.142
6675	0.497	1.380	0.097	0.491	1.321	0.508	0.468	1.194	0.492
6676	0.432	0.958	0.097	0.482	1.265	0.491	0.511	1.464	0.296
6677	0.505	1.430	0.513	0.509	1.434	0.462	0.489	1.329	0.296
6678	0.503	1.413	0.513	0.442	1.001	0.462	0.450	1.076	0.385
6679	0.511	1.464	0.492	0.488	1.302	0.462	0.384	0.655	0.512
6680	0.503	1.413	0.512	0.503	1.396	0.149	0.466	1.178	0.467
6681	0.184	−0.625	0.501	0.307	0.135	0.514	0.411	0.824	0.224
6682	0.484	1.295	0.508	0.447	1.039	0.235	0.479	1.262	0.262
6683	0.429	0.942	0.508	0.494	1.340	0.444	0.487	1.312	0.508
6684	0.447	1.060	0.512	0.465	1.152	0.508	0.505	1.430	0.508
6685	0.450	1.076	0.508	0.512	1.453	0.512	0.505	1.430	0.508
6686	0.463	1.161	0.450	0.482	1.265	0.512	0.513	1.481	0.501
6687	0.500	1.397	0.097	0.509	1.434	0.514	0.497	1.380	0.501
6688	0.484	1.295	0.097	0.494	1.340	0.514	0.482	1.279	0.050
6689	0.468	1.194	0.450	0.459	1.114	0.149	0.508	1.447	0.050
6690	0.468	1.194	0.481	0.459	1.114	0.193	0.497	1.380	0.492
6691	0.508	1.447	0.481	0.462	1.133	0.508	0.461	1.144	0.492
6692	0.224	−0.373	0.492	0.152	−0.863	0.508	0.295	0.082	0.467
6693	0.476	1.245	0.185	0.518	1.491	0.491	0.495	1.363	0.512
6694	0.263	−0.120	0.508	0.237	−0.317	0.508	0.142	−0.895	0.508
6695	0.489	1.329	0.409	0.447	1.039	0.508	0.492	1.346	0.512
6696	0.505	1.430	0.467	0.506	1.415	0.235	0.495	1.363	0.512
6697	0.505	1.430	0.481	0.509	1.434	0.235	0.508	1.447	0.501
6698	0.505	1.430	0.450	0.509	1.434	0.501	0.489	1.329	0.409
6699	0.432	0.958	0.185	0.515	1.472	0.514	0.505	1.430	0.512
6700	0.495	1.363	0.513	0.512	1.453	0.514	0.505	1.430	0.385
6701	0.297	0.099	0.481	0.149	−0.882	0.309	0.187	−0.608	0.385
6702	0.050	−1.485	0.185	0.196	−0.581	0.512	0.411	0.824	0.492
6703	0.468	1.194	0.492	0.506	1.415	0.512	0.476	1.245	0.409
6704	0.482	1.279	0.513	0.506	1.415	0.512	0.476	1.245	0.508
6705	0.513	1.481	0.513	0.491	1.321	0.501	0.505	1.430	0.492
6706	0.513	1.481	0.262	0.506	1.415	0.501	0.466	1.178	0.262

6707	0.505	1.430	0.481	0.506	1.415	0.512	0.492	1.346	0.501
6708	0.187	-0.608	0.185	0.234	-0.336	0.501	0.184	-0.625	0.512
6709	0.050	-1.485	0.185	0.234	-0.336	0.102	0.389	0.689	0.513
6710	0.511	1.464	0.492	0.494	1.340	0.102	0.500	1.397	0.431
6711	0.500	1.397	0.185	0.512	1.453	0.501	0.479	1.262	0.512
6712	0.508	1.447	0.185	0.509	1.434	0.508	0.461	1.144	0.224
6713	0.358	0.487	0.481	0.307	0.135	0.508	0.224	-0.373	0.467
6714	0.497	1.380	0.492	0.512	1.453	0.478	0.413	0.841	0.467
6715	0.497	1.380	0.512	0.512	1.453	0.478	0.453	1.093	0.296
6716	0.497	1.380	0.050	0.512	1.453	0.193	0.453	1.093	0.501
6717	0.500	1.397	0.050	0.503	1.396	0.491	0.476	1.245	0.409
6718	0.500	1.397	0.097	0.503	1.396	0.462	0.476	1.245	0.508
6719	0.489	1.329	0.512	0.509	1.434	0.273	0.497	1.380	0.097
6720	0.511	1.464	0.508	0.494	1.340	0.102	0.500	1.397	0.501
6721	0.184	-0.625	0.492	0.102	-1.184	0.193	0.263	-0.120	0.185
6722	0.184	-0.625	0.492	0.102	-1.184	0.462	0.263	-0.120	0.185
6723	0.518	1.514	0.501	0.500	1.378	0.462	0.513	1.481	0.508
6724	0.511	1.464	0.501	0.503	1.396	0.462	0.505	1.430	0.142
6725	0.511	1.464	0.501	0.503	1.396	0.444	0.511	1.464	0.224
6726	0.513	1.481	0.467	0.482	1.265	0.512	0.429	0.942	0.481
6727	0.495	1.363	0.385	0.482	1.265	0.444	0.513	1.481	0.512
6728	0.263	-0.120	0.385	0.196	-0.581	0.422	0.184	-0.625	0.467
6729	0.511	1.464	0.508	0.491	1.321	0.508	0.508	1.447	0.358
6730	0.482	1.279	0.467	0.462	1.133	0.501	0.468	1.194	0.142
6731	0.097	-1.181	0.467	0.275	-0.073	0.193	0.297	0.099	0.142
6732	0.361	0.504	0.467	0.102	-1.184	0.149	0.097	-1.181	0.262
6733	0.050	-1.485	0.501	0.193	-0.600	0.309	0.326	0.285	0.513
6734	0.408	0.807	0.501	0.462	1.133	0.491	0.513	1.481	0.501
6735	0.408	0.807	0.501	0.462	1.133	0.193	0.513	1.481	0.492
6736	0.429	0.942	0.512	0.462	1.133	0.371	0.513	1.481	0.492
6737	0.479	1.262	0.185	0.444	1.020	0.102	0.500	1.397	0.508
6738	0.468	1.194	0.262	0.512	1.453	0.478	0.505	1.430	0.508
6739	0.492	1.346	0.262	0.442	1.001	0.514	0.492	1.346	0.481
6740	0.489	1.329	0.492	0.418	0.850	0.371	0.516	1.498	0.481
6741	0.463	1.161	0.501	0.500	1.378	0.149	0.482	1.279	0.358
6742	0.497	1.380	0.512	0.494	1.340	0.514	0.411	0.824	0.328
6743	0.142	-0.895	0.508	0.196	-0.581	0.514	0.263	-0.120	0.513
6744	0.187	-0.608	0.185	0.152	-0.863	0.512	0.295	0.082	0.508
6745	0.358	0.487	0.513	0.310	0.153	0.491	0.142	-0.895	0.508
6746	0.429	0.942	0.513	0.488	1.302	0.444	0.495	1.363	0.450
6747	0.187	-0.608	0.492	0.193	-0.600	0.462	0.295	0.082	0.492
6748	0.224	-0.373	0.513	0.371	0.549	0.462	0.295	0.082	0.492
6749	0.224	-0.373	0.142	0.102	-1.184	0.478	0.387	0.672	0.492
6750	0.468	1.194	0.467	0.480	1.246	0.478	0.516	1.498	0.508
6751	0.505	1.430	0.467	0.512	1.453	0.491	0.468	1.194	0.296
6752	0.142	-0.895	0.481	0.377	0.587	0.491	0.224	-0.373	0.224
6753	0.187	-0.608	0.492	0.152	-0.863	0.508	0.261	-0.137	0.262
6754	0.497	1.380	0.501	0.509	1.434	0.508	0.508	1.447	0.262
6755	0.497	1.380	0.450	0.509	1.434	0.508	0.508	1.447	0.481
6756	0.479	1.262	0.481	0.506	1.415	0.501	0.508	1.447	0.492
6757	0.505	1.430	0.501	0.491	1.321	0.508	0.503	1.413	0.501
6758	0.503	1.413	0.142	0.442	1.001	0.508	0.497	1.380	0.501
6759	0.097	-1.181	0.512	0.456	1.095	0.273	0.050	-1.485	0.328
6760	0.097	-1.181	0.508	0.456	1.095	0.273	0.050	-1.485	0.501
6761	0.513	1.481	0.492	0.477	1.227	0.501	0.492	1.346	0.501
6762	0.513	1.481	0.262	0.477	1.227	0.512	0.492	1.346	0.467
6763	0.484	1.295	0.508	0.491	1.321	0.508	0.466	1.178	0.508
6764	0.511	1.464	0.508	0.494	1.340	0.512	0.511	1.464	0.097
6765	0.497	1.380	0.512	0.509	1.434	0.273	0.511	1.464	0.501
6766	0.503	1.413	0.513	0.509	1.434	0.508	0.513	1.481	0.501
6767	0.503	1.413	0.481	0.509	1.434	0.501	0.513	1.481	0.508

6768	0.503	1.413	0.492	0.503	1.396	0.508	0.500	1.397	0.512
6769	0.497	1.380	0.513	0.509	1.434	0.508	0.408	0.807	0.508
6770	0.445	1.043	0.492	0.506	1.415	0.444	0.513	1.481	0.481
6771	0.097	-1.181	0.513	0.278	-0.054	0.235	0.382	0.638	0.467
6772	0.097	-1.181	0.492	0.278	-0.054	0.508	0.382	0.638	0.501
6773	0.447	1.060	0.050	0.503	1.396	0.514	0.492	1.346	0.296
6774	0.482	1.279	0.481	0.518	1.491	0.149	0.411	0.824	0.512
6775	0.484	1.295	0.501	0.512	1.453	0.501	0.511	1.464	0.450
6776	0.489	1.329	0.508	0.506	1.415	0.514	0.492	1.346	0.508
6777	0.184	-0.625	0.501	0.275	-0.073	0.478	0.263	-0.120	0.224
6778	0.503	1.413	0.296	0.509	1.434	0.341	0.497	1.380	0.513
6779	0.408	0.807	0.185	0.506	1.415	0.501	0.513	1.481	0.513
6780	0.466	1.178	0.185	0.509	1.434	0.309	0.513	1.481	0.492
6781	0.482	1.279	0.296	0.512	1.453	0.309	0.429	0.942	0.508
6782	0.447	1.060	0.050	0.447	1.039	0.491	0.505	1.430	0.431
6783	0.187	-0.608	0.512	0.237	-0.317	0.235	0.224	-0.373	0.431
6784	0.511	1.464	0.142	0.503	1.396	0.149	0.466	1.178	0.492
6785	0.482	1.279	0.467	0.509	1.434	0.444	0.461	1.144	0.467
6786	0.187	-0.608	0.501	0.149	-0.882	0.514	0.297	0.099	0.467
6787	0.495	1.363	0.467	0.500	1.378	0.501	0.505	1.430	0.431
6788	0.508	1.447	0.097	0.515	1.472	0.371	0.411	0.824	0.224
6789	0.516	1.498	0.185	0.480	1.246	0.371	0.508	1.447	0.512
6790	0.261	-0.137	0.431	0.345	0.379	0.341	0.097	-1.181	0.501
6791	0.484	1.295	0.224	0.503	1.396	0.341	0.495	1.363	0.467
6792	0.184	-0.625	0.508	0.310	0.153	0.478	0.184	-0.625	0.492
6793	0.184	-0.625	0.512	0.310	0.153	0.501	0.184	-0.625	0.296
6794	0.495	1.363	0.512	0.491	1.321	0.508	0.503	1.413	0.097
6795	0.184	-0.625	0.501	0.234	-0.336	0.514	0.142	-0.895	0.224
6796	0.184	-0.625	0.262	0.149	-0.882	0.514	0.224	-0.373	0.097
6797	0.479	1.262	0.492	0.439	0.982	0.512	0.479	1.262	0.296
6798	0.489	1.329	0.492	0.506	1.415	0.508	0.516	1.498	0.512
6799	0.505	1.430	0.097	0.509	1.434	0.371	0.474	1.228	0.262
6800	0.050	-1.485	0.097	0.374	0.568	0.398	0.358	0.487	0.512
6801	0.050	-1.485	0.097	0.374	0.568	0.398	0.142	-0.895	0.513
6802	0.097	-1.181	0.508	0.342	0.361	0.444	0.142	-0.895	0.501
6803	0.000	?	0.050	0.345	0.379	0.512	0.261	-0.137	0.185
6804	0.505	1.430	0.050	0.480	1.246	0.512	0.513	1.481	0.328
6805	0.500	1.397	0.508	0.503	1.396	0.491	0.500	1.397	0.492
6806	0.489	1.329	0.224	0.509	1.434	0.478	0.487	1.312	0.224
6807	0.489	1.329	0.097	0.518	1.491	0.478	0.495	1.363	0.501
6808	0.497	1.380	0.431	0.518	1.491	0.478	0.508	1.447	0.450
6809	0.503	1.413	0.513	0.509	1.434	0.512	0.500	1.397	0.450
6810	0.495	1.363	0.513	0.509	1.434	0.341	0.479	1.262	0.512
6811	0.468	1.194	0.512	0.371	0.549	0.371	0.479	1.262	0.224
6812	0.387	0.672	0.481	0.401	0.737	0.273	0.358	0.487	0.512
6813	0.387	0.672	0.512	0.401	0.737	0.309	0.329	0.301	0.501
6814	0.511	1.464	0.501	0.444	1.020	0.512	0.516	1.498	0.296
6815	0.463	1.161	0.508	0.515	1.472	0.512	0.500	1.397	0.296
6816	0.463	1.161	0.512	0.515	1.472	0.491	0.500	1.397	0.224
6817	0.463	1.161	0.296	0.491	1.321	0.501	0.447	1.060	0.431
6818	0.495	1.363	0.508	0.477	1.227	0.273	0.492	1.346	0.296
6819	0.495	1.363	0.512	0.477	1.227	0.444	0.492	1.346	0.431
6820	0.495	1.363	0.492	0.477	1.227	0.444	0.492	1.346	0.512
6821	0.505	1.430	0.296	0.509	1.434	0.501	0.508	1.447	0.097
6822	0.184	-0.625	0.513	0.342	0.361	0.501	0.300	0.116	0.224
6823	0.261	-0.137	0.501	0.374	0.568	0.341	0.226	-0.356	0.501
6824	0.261	-0.137	0.512	0.275	-0.073	0.462	0.266	-0.103	0.512
6825	0.000	?	0.513	0.313	0.172	0.462	0.261	-0.137	0.512
6826	0.487	1.312	0.481	0.512	1.453	0.444	0.479	1.262	0.508
6827	0.492	1.346	0.513	0.512	1.453	0.444	0.492	1.346	0.501
6828	0.508	1.447	0.508	0.491	1.321	0.422	0.500	1.397	0.508

6829	0.503	1.413	0.467	0.503	1.396	0.478	0.500	1.397	0.385
6830	0.184	-0.625	0.467	0.275	-0.073	0.508	0.332	0.318	0.512
6831	0.516	1.498	0.512	0.444	1.020	0.491	0.497	1.380	0.508
6832	0.516	1.498	0.467	0.444	1.020	0.193	0.497	1.380	0.224
6833	0.495	1.363	0.185	0.494	1.340	0.422	0.461	1.144	0.492
6834	0.508	1.447	0.431	0.497	1.359	0.501	0.511	1.464	0.492
6835	0.142	-0.895	0.431	0.342	0.361	0.422	0.097	-1.181	0.431
6836	0.468	1.194	0.050	0.465	1.152	0.273	0.500	1.397	0.450
6837	0.468	1.194	0.097	0.465	1.152	0.478	0.500	1.397	0.450
6838	0.479	1.262	0.185	0.444	1.020	0.478	0.508	1.447	0.431
6839	0.489	1.329	0.501	0.444	1.020	0.462	0.513	1.481	0.508
6840	0.505	1.430	0.050	0.418	0.850	0.422	0.516	1.498	0.508
6841	0.453	1.093	0.431	0.482	1.265	0.491	0.476	1.245	0.481
6842	0.484	1.295	0.224	0.509	1.434	0.491	0.468	1.194	0.501
6843	0.508	1.447	0.224	0.488	1.302	0.444	0.503	1.413	0.508
6844	0.142	-0.895	0.492	0.196	-0.581	0.462	0.295	0.082	0.450
6845	0.511	1.464	0.508	0.427	0.907	0.462	0.511	1.464	0.508
6846	0.511	1.464	0.501	0.503	1.396	0.491	0.447	1.060	0.450
6847	0.495	1.363	0.501	0.424	0.888	0.273	0.511	1.464	0.508
6848	0.263	-0.120	0.501	0.275	-0.073	0.501	0.226	-0.356	0.501
6849	0.505	1.430	0.050	0.474	1.208	0.501	0.511	1.464	0.481
6850	0.505	1.430	0.050	0.474	1.208	0.444	0.511	1.464	0.224
6851	0.513	1.481	0.185	0.468	1.170	0.478	0.492	1.346	0.224
6852	0.511	1.464	0.513	0.427	0.907	0.149	0.513	1.481	0.185
6853	0.479	1.262	0.492	0.494	1.340	0.273	0.437	0.992	0.513
6854	0.489	1.329	0.185	0.494	1.340	0.309	0.437	0.992	0.409
6855	0.503	1.413	0.508	0.447	1.039	0.371	0.489	1.329	0.512
6856	0.492	1.346	0.431	0.465	1.152	0.235	0.466	1.178	0.142
6857	0.513	1.481	0.492	0.459	1.114	0.478	0.463	1.161	0.142
6858	0.495	1.363	0.492	0.488	1.302	0.193	0.426	0.925	0.450
6859	0.050	-1.485	0.262	0.275	-0.073	0.501	0.224	-0.373	0.501
6860	0.479	1.262	0.262	0.500	1.378	0.462	0.511	1.464	0.508
6861	0.500	1.397	0.481	0.500	1.378	0.478	0.497	1.380	0.508
6862	0.503	1.413	0.467	0.450	1.057	0.273	0.466	1.178	0.513
6863	0.497	1.380	0.431	0.482	1.265	0.235	0.492	1.346	0.328
6864	0.297	0.099	0.385	0.152	-0.863	0.422	0.295	0.082	0.296
6865	0.184	-0.625	0.512	0.275	-0.073	0.149	0.097	-1.181	0.328
6866	0.184	-0.625	0.512	0.310	0.153	0.491	0.229	-0.339	0.328
6867	0.297	0.099	0.262	0.374	0.568	0.508	0.097	-1.181	0.512
6868	0.050	-1.485	0.450	0.237	-0.317	0.508	0.297	0.099	0.508
6869	0.500	1.397	0.185	0.480	1.246	0.491	0.513	1.481	0.224
6870	0.142	-0.895	0.513	0.196	-0.581	0.149	0.263	-0.120	0.467
6871	0.468	1.194	0.508	0.500	1.378	0.508	0.513	1.481	0.508
6872	0.503	1.413	0.050	0.468	1.170	0.478	0.508	1.447	0.481
6873	0.468	1.194	0.050	0.474	1.208	0.309	0.505	1.430	0.481
6874	0.097	-1.181	0.050	0.272	-0.091	0.371	0.184	-0.625	0.224
6875	0.184	-0.625	0.492	0.234	-0.336	0.273	0.326	0.285	0.224
6876	0.432	0.958	0.512	0.418	0.850	0.508	0.489	1.329	0.467
6877	0.224	-0.373	0.512	0.149	-0.882	0.273	0.224	-0.373	0.501
6878	0.503	1.413	0.224	0.488	1.302	0.341	0.505	1.430	0.492
6879	0.505	1.430	0.224	0.506	1.415	0.491	0.447	1.060	0.492
6880	0.505	1.430	0.508	0.506	1.415	0.235	0.447	1.060	0.431
6881	0.500	1.397	0.185	0.491	1.321	0.235	0.518	1.514	0.501
6882	0.261	-0.137	0.185	0.149	-0.882	0.501	0.224	-0.373	0.385
6883	0.489	1.329	0.467	0.509	1.434	0.478	0.508	1.447	0.492
6884	0.487	1.312	0.467	0.485	1.283	0.478	0.495	1.363	0.097
6885	0.100	-1.164	0.481	0.310	0.153	0.398	0.297	0.099	0.481
6886	0.100	-1.164	0.508	0.371	0.549	0.501	0.297	0.099	0.512
6887	0.097	-1.181	0.512	0.275	-0.073	0.478	0.224	-0.373	0.328
6888	0.500	1.397	0.501	0.506	1.415	0.501	0.432	0.958	0.328
6889	0.050	-1.485	0.481	0.272	-0.091	0.422	0.295	0.082	0.262

6890	0.050	-1.485	0.097	0.342	0.361	0.512	0.434	0.975	0.492
6891	0.500	1.397	0.262	0.494	1.340	0.149	0.513	1.481	0.512
6892	0.226	-0.356	0.450	0.237	-0.317	0.444	0.097	-1.181	0.508
6893	0.097	-1.181	0.385	0.237	-0.317	0.462	0.224	-0.373	0.185
6894	0.432	0.958	0.385	0.500	1.378	0.478	0.497	1.380	0.185
6895	0.508	1.447	0.142	0.477	1.227	0.371	0.508	1.447	0.513
6896	0.508	1.447	0.142	0.477	1.227	0.501	0.508	1.447	0.328
6897	0.513	1.481	0.508	0.401	0.737	0.462	0.505	1.430	0.328
6898	0.476	1.245	0.385	0.500	1.378	0.462	0.497	1.380	0.492
6899	0.505	1.430	0.185	0.477	1.227	0.478	0.505	1.430	0.492
6900	0.495	1.363	0.492	0.500	1.378	0.514	0.384	0.655	0.501
6901	0.497	1.380	0.185	0.421	0.869	0.512	0.516	1.498	0.501
6902	0.500	1.397	0.481	0.512	1.453	0.508	0.511	1.464	0.450
6903	0.297	0.099	0.224	0.152	-0.863	0.491	0.224	-0.373	0.450
6904	0.511	1.464	0.508	0.447	1.039	0.478	0.495	1.363	0.501
6905	0.513	1.481	0.185	0.465	1.152	0.508	0.495	1.363	0.262
6906	0.495	1.363	0.512	0.480	1.246	0.462	0.432	0.958	0.097
6907	0.295	0.082	0.328	0.374	0.568	0.371	0.453	1.093	0.481
6908	0.511	1.464	0.224	0.503	1.396	0.422	0.447	1.060	0.185
6909	0.495	1.363	0.431	0.462	1.133	0.491	0.429	0.942	0.185
6910	0.508	1.447	0.512	0.465	1.152	0.273	0.500	1.397	0.224
6911	0.511	1.464	0.296	0.482	1.265	0.235	0.500	1.397	0.224
6912	0.479	1.262	0.492	0.512	1.453	0.273	0.479	1.262	0.385
6913	0.511	1.464	0.492	0.509	1.434	0.444	0.500	1.397	0.450
6914	0.497	1.380	0.224	0.512	1.453	0.193	0.505	1.430	0.142
6915	0.463	1.161	0.185	0.491	1.321	0.462	0.450	1.076	0.501
6916	0.463	1.161	0.185	0.474	1.208	0.309	0.505	1.430	0.142
6917	0.511	1.464	0.262	0.503	1.396	0.273	0.447	1.060	0.512
6918	0.463	1.161	0.492	0.459	1.114	0.501	0.505	1.430	0.185
6919	0.184	-0.625	0.501	0.374	0.568	0.462	0.000	?	0.431
6920	0.429	0.942	0.508	0.421	0.869	0.508	0.497	1.380	0.262
6921	0.426	0.925	0.512	0.491	1.321	0.508	0.479	1.262	0.492
6922	0.050	-1.485	0.481	0.275	-0.073	0.478	0.226	-0.356	0.050
6923	0.097	-1.181	0.508	0.234	-0.336	0.309	0.226	-0.356	0.142
6924	0.184	-0.625	0.296	0.272	-0.091	0.309	0.184	-0.625	0.501
6925	0.497	1.380	0.296	0.442	1.001	0.309	0.521	1.531	0.512
6926	0.050	-1.485	0.501	0.196	-0.581	0.102	0.411	0.824	0.224
6927	0.429	0.942	0.508	0.465	1.152	0.508	0.511	1.464	0.409
6928	0.226	-0.356	0.501	0.307	0.135	0.398	0.142	-0.895	0.385
6929	0.226	-0.356	0.512	0.272	-0.091	0.341	0.142	-0.895	0.328
6930	0.487	1.312	0.050	0.500	1.378	0.462	0.445	1.043	0.224
6931	0.508	1.447	0.508	0.465	1.152	0.512	0.505	1.430	0.328
6932	0.492	1.346	0.262	0.512	1.453	0.508	0.505	1.430	0.262
6933	0.492	1.346	0.492	0.512	1.453	0.508	0.505	1.430	0.492
6934	0.503	1.413	0.512	0.477	1.227	0.193	0.513	1.481	0.508
6935	0.050	-1.485	0.508	0.310	0.153	0.193	0.329	0.301	0.492
6936	0.000	?	0.501	0.310	0.153	0.491	0.295	0.082	0.508
6937	0.050	-1.485	0.512	0.310	0.153	0.491	0.329	0.301	0.467
6938	0.187	-0.608	0.492	0.102	-1.184	0.501	0.329	0.301	0.467
6939	0.511	1.464	0.501	0.503	1.396	0.501	0.511	1.464	0.431
6940	0.489	1.329	0.450	0.398	0.718	0.462	0.503	1.413	0.467
6941	0.184	-0.625	0.512	0.345	0.379	0.462	0.226	-0.356	0.508
6942	0.505	1.430	0.492	0.471	1.189	0.053	0.461	1.144	0.508
6943	0.429	0.942	0.224	0.518	1.491	0.491	0.503	1.413	0.508
6944	0.492	1.346	0.492	0.500	1.378	0.309	0.482	1.279	0.501
6945	0.492	1.346	0.501	0.500	1.378	0.501	0.479	1.262	0.185
6946	0.261	-0.137	0.296	0.193	-0.600	0.444	0.226	-0.356	0.512
6947	0.261	-0.137	0.224	0.193	-0.600	0.273	0.226	-0.356	0.185
6948	0.484	1.295	0.097	0.488	1.302	0.273	0.461	1.144	0.501
6949	0.466	1.178	0.262	0.485	1.283	0.309	0.500	1.397	0.467
6950	0.429	0.942	0.262	0.494	1.340	0.512	0.495	1.363	0.492

6951	0.382	0.638	0.513	0.494	1.340	0.491	0.495	1.363	0.467
6952	0.505	1.430	0.481	0.459	1.114	0.478	0.432	0.958	0.508
6953	0.500	1.397	0.501	0.465	1.152	0.193	0.503	1.413	0.481
6954	0.261	-0.137	0.512	0.053	-1.504	0.193	0.389	0.689	0.513
6955	0.447	1.060	0.097	0.485	1.283	0.508	0.492	1.346	0.512
6956	0.184	-0.625	0.142	0.310	0.153	0.491	0.100	-1.164	0.508
6957	0.503	1.413	0.492	0.500	1.378	0.491	0.482	1.279	0.409
6958	0.508	1.447	0.481	0.444	1.020	0.341	0.513	1.481	0.328
6959	0.050	-1.485	0.501	0.272	-0.091	0.512	0.332	0.318	0.358
6960	0.050	-1.485	0.481	0.272	-0.091	0.491	0.332	0.318	0.513
6961	0.050	-1.485	0.481	0.310	0.153	0.508	0.263	-0.120	0.513
6962	0.487	1.312	0.501	0.518	1.491	0.512	0.492	1.346	0.224
6963	0.500	1.397	0.262	0.497	1.359	0.273	0.518	1.514	0.358
6964	0.500	1.397	0.185	0.482	1.265	0.235	0.513	1.481	0.296
6965	0.224	-0.373	0.328	0.193	-0.600	0.508	0.187	-0.608	0.328
6966	0.224	-0.373	0.296	0.193	-0.600	0.149	0.187	-0.608	0.328
6967	0.505	1.430	0.224	0.503	1.396	0.149	0.511	1.464	0.492
6968	0.184	-0.625	0.492	0.000	?	0.273	0.332	0.318	0.481
6969	0.184	-0.625	0.501	0.000	?	0.273	0.329	0.301	0.450
6970	0.468	1.194	0.492	0.488	1.302	0.501	0.492	1.346	0.481
6971	0.468	1.194	0.512	0.488	1.302	0.235	0.492	1.346	0.097
6972	0.474	1.228	0.512	0.342	0.361	0.273	0.500	1.397	0.296
6973	0.505	1.430	0.492	0.515	1.472	0.508	0.503	1.413	0.481
6974	0.511	1.464	0.512	0.485	1.283	0.371	0.447	1.060	0.501
6975	0.500	1.397	0.513	0.503	1.396	0.501	0.447	1.060	0.481
6976	0.476	1.245	0.050	0.512	1.453	0.149	0.500	1.397	0.508
6977	0.097	-1.181	0.512	0.275	-0.073	0.444	0.263	-0.120	0.508
6978	0.263	-0.120	0.508	0.234	-0.336	0.235	0.097	-1.181	0.450
6979	0.453	1.093	0.512	0.509	1.434	0.508	0.479	1.262	0.224
6980	0.384	0.655	0.508	0.152	-0.863	0.149	0.184	-0.625	0.142
6981	0.384	0.655	0.185	0.152	-0.863	0.235	0.184	-0.625	0.358
6982	0.142	-0.895	0.224	0.275	-0.073	0.512	0.224	-0.373	0.224
6983	0.142	-0.895	0.050	0.275	-0.073	0.462	0.224	-0.373	0.185
6984	0.508	1.447	0.512	0.503	1.396	0.102	0.382	0.638	0.501
6985	0.387	0.672	0.142	0.234	-0.336	0.514	0.455	1.110	0.450
6986	0.184	-0.625	0.508	0.275	-0.073	0.514	0.142	-0.895	0.508
6987	0.489	1.329	0.409	0.509	1.434	0.149	0.503	1.413	0.512
6988	0.184	-0.625	0.224	0.371	0.549	0.273	0.142	-0.895	0.512
6989	0.476	1.245	0.431	0.503	1.396	0.102	0.511	1.464	0.481
6990	0.224	-0.373	0.450	0.152	-0.863	0.309	0.184	-0.625	0.501
6991	0.508	1.447	0.224	0.444	1.020	0.508	0.432	0.958	0.481
6992	0.184	-0.625	0.481	0.240	-0.299	0.422	0.261	-0.137	0.185
6993	0.508	1.447	0.512	0.515	1.472	0.422	0.489	1.329	0.481
6994	0.332	0.318	0.142	0.149	-0.882	0.462	0.050	-1.485	0.512
6995	0.224	-0.373	0.142	0.237	-0.317	0.501	0.142	-0.895	0.508
6996	0.429	0.942	0.508	0.515	1.472	0.444	0.495	1.363	0.508
6997	0.511	1.464	0.328	0.456	1.095	0.235	0.511	1.464	0.097
6998	0.297	0.099	0.508	0.102	-1.184	0.235	0.226	-0.356	0.262
6999	0.495	1.363	0.296	0.518	1.491	0.501	0.408	0.807	0.431
7000	0.495	1.363	0.492	0.518	1.491	0.491	0.384	0.655	0.431
7001	0.224	-0.373	0.492	0.152	-0.863	0.491	0.329	0.301	0.358
7002	0.184	-0.625	0.224	0.272	-0.091	0.462	0.226	-0.356	0.431
7003	0.184	-0.625	0.512	0.102	-1.184	0.309	0.329	0.301	0.512
7004	0.263	-0.120	0.513	0.307	0.135	0.491	0.263	-0.120	0.431
7005	0.495	1.363	0.492	0.509	1.434	0.149	0.492	1.346	0.512
7006	0.500	1.397	0.262	0.424	0.888	0.512	0.505	1.430	0.467
7007	0.505	1.430	0.501	0.424	0.888	0.512	0.489	1.329	0.185
7008	0.508	1.447	0.224	0.465	1.152	0.508	0.505	1.430	0.501
7009	0.476	1.245	0.508	0.503	1.396	0.508	0.466	1.178	0.467
7010	0.505	1.430	0.409	0.444	1.020	0.508	0.466	1.178	0.385
7011	0.297	0.099	0.513	0.237	-0.317	0.478	0.429	0.942	0.262

7012	0.297	0.099	0.512	0.237	-0.317	0.341	0.468	1.194	0.481
7013	0.497	1.380	0.481	0.500	1.378	0.462	0.513	1.481	0.296
7014	0.505	1.430	0.481	0.491	1.321	0.512	0.511	1.464	0.501
7015	0.497	1.380	0.512	0.491	1.321	0.444	0.513	1.481	0.142
7016	0.508	1.447	0.097	0.465	1.152	0.102	0.495	1.363	0.513
7017	0.050	-1.485	0.142	0.307	0.135	0.149	0.184	-0.625	0.513
7018	0.503	1.413	0.513	0.494	1.340	0.491	0.508	1.447	0.262
7019	0.263	-0.120	0.501	0.149	-0.882	0.501	0.184	-0.625	0.481
7020	0.492	1.346	0.508	0.515	1.472	0.371	0.500	1.397	0.512
7021	0.500	1.397	0.050	0.518	1.491	0.273	0.463	1.161	0.481
7022	0.505	1.430	0.224	0.503	1.396	0.341	0.489	1.329	0.224
7023	0.500	1.397	0.492	0.503	1.396	0.341	0.463	1.161	0.501
7024	0.508	1.447	0.492	0.506	1.415	0.491	0.508	1.447	0.224
7025	0.492	1.346	0.492	0.482	1.265	0.508	0.482	1.279	0.512
7026	0.503	1.413	0.097	0.345	0.379	0.508	0.513	1.481	0.262
7027	0.450	1.076	0.097	0.471	1.189	0.512	0.508	1.447	0.512
7028	0.505	1.430	0.097	0.518	1.491	0.422	0.500	1.397	0.501
7029	0.495	1.363	0.097	0.444	1.020	0.149	0.413	0.841	0.481
7030	0.226	-0.356	0.224	0.102	-1.184	0.501	0.329	0.301	0.481
7031	0.000	?	0.513	0.152	-0.863	0.501	0.358	0.487	0.481
7032	0.492	1.346	0.142	0.494	1.340	0.462	0.516	1.498	0.512
7033	0.500	1.397	0.431	0.500	1.378	0.462	0.508	1.447	0.262
7034	0.300	0.116	0.050	0.374	0.568	0.462	0.224	-0.373	0.224
7035	0.224	-0.373	0.050	0.000	?	0.508	0.358	0.487	0.481
7036	0.097	-1.181	0.050	0.275	-0.073	0.235	0.297	0.099	0.508
7037	0.263	-0.120	0.501	0.342	0.361	0.341	0.332	0.318	0.492
7038	0.263	-0.120	0.431	0.342	0.361	0.235	0.332	0.318	0.328
7039	0.511	1.464	0.185	0.488	1.302	0.398	0.489	1.329	0.262
7040	0.482	1.279	0.262	0.506	1.415	0.309	0.479	1.262	0.431
7041	0.503	1.413	0.501	0.509	1.434	0.508	0.447	1.060	0.409
7042	0.511	1.464	0.262	0.512	1.453	0.444	0.476	1.245	0.409
7043	0.097	-1.181	0.385	0.421	0.869	0.444	0.100	-1.164	0.385
7044	0.142	-0.895	0.385	0.149	-0.882	0.462	0.295	0.082	0.431
7045	0.489	1.329	0.501	0.503	1.396	0.508	0.476	1.245	0.385
7046	0.474	1.228	0.492	0.503	1.396	0.508	0.497	1.380	0.431
7047	0.497	1.380	0.481	0.468	1.170	0.491	0.482	1.279	0.050
7048	0.482	1.279	0.431	0.456	1.095	0.491	0.511	1.464	0.512
7049	0.482	1.279	0.185	0.456	1.095	0.398	0.511	1.464	0.296
7050	0.500	1.397	0.296	0.509	1.434	0.514	0.447	1.060	0.431
7051	0.263	-0.120	0.328	0.237	-0.317	0.501	0.226	-0.356	0.385
7052	0.184	-0.625	0.328	0.339	0.342	0.491	0.142	-0.895	0.358
7053	0.329	0.301	0.501	0.237	-0.317	0.462	0.358	0.487	0.358
7054	0.295	0.082	0.262	0.398	0.718	0.273	0.224	-0.373	0.481
7055	0.226	-0.356	0.262	0.310	0.153	0.102	0.187	-0.608	0.450
7056	0.492	1.346	0.512	0.509	1.434	0.193	0.500	1.397	0.097
7057	0.497	1.380	0.142	0.439	0.982	0.508	0.453	1.093	0.097
7058	0.484	1.295	0.512	0.447	1.039	0.341	0.508	1.447	0.512
7059	0.508	1.447	0.512	0.459	1.114	0.501	0.516	1.498	0.050
7060	0.503	1.413	0.512	0.509	1.434	0.501	0.511	1.464	0.385
7061	0.487	1.312	0.501	0.509	1.434	0.193	0.479	1.262	0.385
7062	0.500	1.397	0.262	0.494	1.340	0.444	0.500	1.397	0.481
7063	0.500	1.397	0.262	0.497	1.359	0.398	0.482	1.279	0.492
7064	0.050	-1.485	0.513	0.398	0.718	0.309	0.184	-0.625	0.492
7065	0.511	1.464	0.385	0.512	1.453	0.508	0.474	1.228	0.467
7066	0.497	1.380	0.481	0.503	1.396	0.512	0.511	1.464	0.142
7067	0.500	1.397	0.409	0.494	1.340	0.053	0.508	1.447	0.262
7068	0.503	1.413	0.358	0.459	1.114	0.149	0.508	1.447	0.296
7069	0.184	-0.625	0.385	0.272	-0.091	0.462	0.097	-1.181	0.296
7070	0.224	-0.373	0.385	0.102	-1.184	0.309	0.263	-0.120	0.508
7071	0.050	-1.485	0.185	0.193	-0.600	0.512	0.434	0.975	0.097
7072	0.503	1.413	0.185	0.512	1.453	0.193	0.434	0.975	0.385

7073	0.142	-0.895	0.467	0.342	0.361	0.444	0.361	0.504	0.097
7074	0.497	1.380	0.097	0.497	1.359	0.444	0.434	0.975	0.481
7075	0.408	0.807	0.262	0.497	1.359	0.273	0.508	1.447	0.296
7076	0.224	-0.373	0.185	0.196	-0.581	0.422	0.432	0.958	0.450
7077	0.429	0.942	0.513	0.442	1.001	0.478	0.508	1.447	0.481
7078	0.450	1.076	0.501	0.404	0.756	0.508	0.471	1.211	0.481
7079	0.229	-0.339	0.508	0.310	0.153	0.053	0.184	-0.625	0.467
7080	0.479	1.262	0.492	0.503	1.396	0.512	0.500	1.397	0.262
7081	0.500	1.397	0.508	0.515	1.472	0.193	0.466	1.178	0.097
7082	0.142	-0.895	0.508	0.053	-1.504	0.491	0.384	0.655	0.467
7083	0.142	-0.895	0.050	0.149	-0.882	0.309	0.263	-0.120	0.385
7084	0.508	1.447	0.358	0.468	1.170	0.508	0.479	1.262	0.450
7085	0.329	0.301	0.431	0.310	0.153	0.478	0.297	0.099	0.385
7086	0.505	1.430	0.328	0.515	1.472	0.512	0.505	1.430	0.296
7087	0.297	0.099	0.513	0.196	-0.581	0.501	0.142	-0.895	0.385
7088	0.489	1.329	0.450	0.444	1.020	0.501	0.518	1.514	0.385
7089	0.489	1.329	0.097	0.444	1.020	0.491	0.516	1.498	0.262
7090	0.224	-0.373	0.492	0.275	-0.073	0.193	0.261	-0.137	0.296
7091	0.505	1.430	0.492	0.430	0.926	0.273	0.482	1.279	0.512
7092	0.508	1.447	0.501	0.482	1.265	0.462	0.513	1.481	0.224
7093	0.492	1.346	0.097	0.503	1.396	0.512	0.482	1.279	0.050
7094	0.263	-0.120	0.501	0.053	-1.504	0.462	0.224	-0.373	0.185
7095	0.503	1.413	0.097	0.515	1.472	0.149	0.500	1.397	0.492
7096	0.224	-0.373	0.501	0.193	-0.600	0.273	0.226	-0.356	0.385
7097	0.508	1.447	0.224	0.491	1.321	0.508	0.511	1.464	0.512
7098	0.000	?	0.508	0.307	0.135	0.512	0.261	-0.137	0.481
7099	0.405	0.790	0.224	0.506	1.415	0.508	0.511	1.464	0.512
7100	0.508	1.447	0.224	0.480	1.246	0.102	0.495	1.363	0.508
7101	0.508	1.447	0.142	0.515	1.472	0.149	0.479	1.262	0.409
7102	0.476	1.245	0.224	0.506	1.415	0.102	0.476	1.245	0.262
7103	0.476	1.245	0.224	0.506	1.415	0.193	0.476	1.245	0.512
7104	0.500	1.397	0.501	0.491	1.321	0.422	0.516	1.498	0.185
7105	0.097	-1.181	0.467	0.196	-0.581	0.491	0.261	-0.137	0.481
7106	0.142	-0.895	0.262	0.275	-0.073	0.371	0.224	-0.373	0.142
7107	0.513	1.481	0.328	0.468	1.170	0.491	0.482	1.279	0.501
7108	0.492	1.346	0.296	0.515	1.472	0.341	0.505	1.430	0.328
7109	0.505	1.430	0.481	0.462	1.133	0.341	0.495	1.363	0.467
7110	0.050	-1.485	0.185	0.149	-0.882	0.273	0.326	0.285	0.450
7111	0.226	-0.356	0.358	0.275	-0.073	0.462	0.261	-0.137	0.431
7112	0.487	1.312	0.492	0.512	1.453	0.508	0.432	0.958	0.097
7113	0.487	1.312	0.512	0.515	1.472	0.273	0.411	0.824	0.501
7114	0.489	1.329	0.513	0.512	1.453	0.235	0.411	0.824	0.185
7115	0.097	-1.181	0.050	0.102	-1.184	0.491	0.387	0.672	0.492
7116	0.097	-1.181	0.513	0.149	-0.882	0.235	0.432	0.958	0.185
7117	0.097	-1.181	0.050	0.102	-1.184	0.193	0.384	0.655	0.409
7118	0.097	-1.181	0.097	0.193	-0.600	0.193	0.432	0.958	0.358
7119	0.224	-0.373	0.508	0.427	0.907	0.398	0.050	-1.485	0.142
7120	0.513	1.481	0.512	0.497	1.359	0.501	0.511	1.464	0.142
7121	0.142	-0.895	0.050	0.374	0.568	0.444	0.297	0.099	0.296
7122	0.429	0.942	0.097	0.491	1.321	0.508	0.432	0.958	0.097
7123	0.050	-1.485	0.481	0.345	0.379	0.235	0.384	0.655	0.224
7124	0.050	-1.485	0.512	0.345	0.379	0.235	0.358	0.487	0.501
7125	0.050	-1.485	0.142	0.278	-0.054	0.444	0.358	0.487	0.328
7126	0.500	1.397	0.358	0.462	1.133	0.444	0.482	1.279	0.224
7127	0.429	0.942	0.328	0.512	1.453	0.514	0.445	1.043	0.050
7128	0.184	-0.625	0.224	0.272	-0.091	0.341	0.097	-1.181	0.185
7129	0.266	-0.103	0.481	0.234	-0.336	0.193	0.097	-1.181	0.467
7130	0.497	1.380	0.185	0.488	1.302	0.193	0.508	1.447	0.431
7131	0.266	-0.103	0.501	0.234	-0.336	0.501	0.050	-1.485	0.409
7132	0.387	0.672	0.512	0.193	-0.600	0.102	0.387	0.672	0.508
7133	0.387	0.672	0.513	0.193	-0.600	0.491	0.387	0.672	0.501

7134	0.505	1.430	0.262	0.395	0.700	0.501	0.479	1.262	0.481
7135	0.487	1.312	0.262	0.500	1.378	0.501	0.497	1.380	0.185
7136	0.484	1.295	0.492	0.444	1.020	0.478	0.489	1.329	0.409
7137	0.432	0.958	0.492	0.509	1.434	0.309	0.461	1.144	0.224
7138	0.184	-0.625	0.492	0.234	-0.336	0.398	0.142	-0.895	0.296
7139	0.297	0.099	0.358	0.240	-0.299	0.508	0.261	-0.137	0.481
7140	0.329	0.301	0.409	0.447	1.039	0.341	0.297	0.099	0.492
7141	0.329	0.301	0.467	0.447	1.039	0.514	0.297	0.099	0.185
7142	0.503	1.413	0.142	0.518	1.491	0.309	0.497	1.380	0.097
7143	0.263	-0.120	0.142	0.342	0.361	0.193	0.097	-1.181	0.467
7144	0.000	?	0.296	0.193	-0.600	0.309	0.387	0.672	0.467
7145	0.261	-0.137	0.450	0.193	-0.600	0.309	0.097	-1.181	0.409
7146	0.500	1.397	0.185	0.503	1.396	0.102	0.482	1.279	0.262
7147	0.142	-0.895	0.185	0.102	-1.184	0.102	0.300	0.116	0.224
7148	0.503	1.413	0.224	0.494	1.340	0.508	0.453	1.093	0.385
7149	0.503	1.413	0.431	0.506	1.415	0.371	0.482	1.279	0.492
7150	0.503	1.413	0.513	0.506	1.415	0.371	0.484	1.295	0.097
7151	0.503	1.413	0.513	0.480	1.246	0.273	0.466	1.178	0.512
7152	0.263	-0.120	0.358	0.310	0.153	0.444	0.263	-0.120	0.508
7153	0.263	-0.120	0.358	0.401	0.737	0.512	0.097	-1.181	0.513
7154	0.500	1.397	0.185	0.512	1.453	0.501	0.466	1.178	0.097
7155	0.387	0.672	0.185	0.339	0.342	0.508	0.382	0.638	0.050
7156	0.482	1.279	0.262	0.515	1.472	0.512	0.447	1.060	0.512
7157	0.413	0.841	0.409	0.307	0.135	0.514	0.382	0.638	0.512
7158	0.358	0.487	0.450	0.193	-0.600	0.273	0.295	0.082	0.512
7159	0.387	0.672	0.481	0.307	0.135	0.273	0.382	0.638	0.185
7160	0.387	0.672	0.492	0.307	0.135	0.508	0.382	0.638	0.467
7161	0.187	-0.608	0.431	0.102	-1.184	0.341	0.261	-0.137	0.492
7162	0.187	-0.608	0.142	0.102	-1.184	0.508	0.295	0.082	0.142
7163	0.463	1.161	0.467	0.506	1.415	0.371	0.511	1.464	0.142
7164	0.097	-1.181	0.409	0.374	0.568	0.501	0.226	-0.356	0.262
7165	0.261	-0.137	0.050	0.374	0.568	0.193	0.050	-1.485	0.501
7166	0.184	-0.625	0.097	0.272	-0.091	0.491	0.184	-0.625	0.328
7167	0.500	1.397	0.467	0.447	1.039	0.508	0.495	1.363	0.142
7168	0.500	1.397	0.492	0.509	1.434	0.491	0.387	0.672	0.450
7169	0.500	1.397	0.513	0.500	1.378	0.341	0.511	1.464	0.492
7170	0.484	1.295	0.512	0.506	1.415	0.501	0.484	1.295	0.481
7171	0.508	1.447	0.512	0.509	1.434	0.371	0.508	1.447	0.492
7172	0.497	1.380	0.512	0.518	1.491	0.508	0.505	1.430	0.185
7173	0.050	-1.485	0.508	0.275	-0.073	0.422	0.411	0.824	0.185
7174	0.361	0.504	0.481	0.278	-0.054	0.235	0.261	-0.137	0.262
7175	0.434	0.975	0.467	0.509	1.434	0.501	0.508	1.447	0.262
7176	0.329	0.301	0.501	0.345	0.379	0.193	0.184	-0.625	0.358
7177	0.518	1.514	0.296	0.509	1.434	0.235	0.479	1.262	0.262
7178	0.000	?	0.224	0.374	0.568	0.149	0.142	-0.895	0.512
7179	0.450	1.076	0.262	0.497	1.359	0.273	0.505	1.430	0.501
7180	0.097	-1.181	0.481	0.193	-0.600	0.193	0.332	0.318	0.508
7181	0.484	1.295	0.262	0.491	1.321	0.491	0.468	1.194	0.450
7182	0.484	1.295	0.185	0.512	1.453	0.478	0.450	1.076	0.142
7183	0.500	1.397	0.185	0.494	1.340	0.341	0.432	0.958	0.481
7184	0.097	-1.181	0.508	0.345	0.379	0.341	0.097	-1.181	0.481
7185	0.495	1.363	0.481	0.503	1.396	0.341	0.503	1.413	0.296
7186	0.097	-1.181	0.224	0.374	0.568	0.491	0.184	-0.625	0.185
7187	0.495	1.363	0.508	0.509	1.434	0.309	0.489	1.329	0.508
7188	0.000	?	0.467	0.424	0.888	0.341	0.184	-0.625	0.513
7189	0.226	-0.356	0.508	0.237	-0.317	0.501	0.411	0.824	0.481
7190	0.500	1.397	0.467	0.503	1.396	0.508	0.358	0.487	0.450
7191	0.224	-0.373	0.262	0.193	-0.600	0.512	0.145	-0.878	0.467
7192	0.224	-0.373	0.481	0.234	-0.336	0.444	0.145	-0.878	0.501
7193	0.142	-0.895	0.262	0.149	-0.882	0.508	0.300	0.116	0.450
7194	0.224	-0.373	0.450	0.275	-0.073	0.444	0.100	-1.164	0.512

7195	0.224	-0.373	0.450	0.196	-0.581	0.193	0.226	-0.356	0.508
7196	0.503	1.413	0.142	0.494	1.340	0.512	0.503	1.413	0.481
7197	0.466	1.178	0.492	0.482	1.265	0.512	0.326	0.285	0.050
7198	0.261	-0.137	0.492	0.342	0.361	0.309	0.224	-0.373	0.097
7199	0.326	0.285	0.142	0.345	0.379	0.341	0.050	-1.485	0.328
7200	0.295	0.082	0.481	0.342	0.361	0.462	0.184	-0.625	0.262
7201	0.484	1.295	0.481	0.494	1.340	0.508	0.468	1.194	0.508
7202	0.187	-0.608	0.481	0.310	0.153	0.149	0.432	0.958	0.358
7203	0.358	0.487	0.262	0.342	0.361	0.273	0.411	0.824	0.142
7204	0.487	1.312	0.328	0.506	1.415	0.273	0.505	1.430	0.142
7205	0.503	1.413	0.296	0.518	1.491	0.053	0.497	1.380	0.508
7206	0.503	1.413	0.050	0.518	1.491	0.478	0.479	1.262	0.492
7207	0.050	-1.485	0.512	0.447	1.039	0.398	0.184	-0.625	0.409
7208	0.503	1.413	0.185	0.509	1.434	0.478	0.408	0.807	0.467
7209	0.050	-1.485	0.185	0.444	1.020	0.501	0.224	-0.373	0.508
7210	0.097	-1.181	0.185	0.193	-0.600	0.501	0.295	0.082	0.508
7211	0.505	1.430	0.512	0.512	1.453	0.309	0.482	1.279	0.501
7212	0.500	1.397	0.508	0.515	1.472	0.235	0.495	1.363	0.097
7213	0.050	-1.485	0.467	0.310	0.153	0.512	0.184	-0.625	0.512
7214	0.097	-1.181	0.450	0.345	0.379	0.491	0.097	-1.181	0.262
7215	0.476	1.245	0.481	0.465	1.152	0.501	0.461	1.144	0.492
7216	0.511	1.464	0.481	0.506	1.415	0.193	0.461	1.144	0.492
7217	0.142	-0.895	0.097	0.152	-0.863	0.478	0.411	0.824	0.185
7218	0.361	0.504	0.467	0.272	-0.091	0.422	0.261	-0.137	0.508
7219	0.329	0.301	0.492	0.275	-0.073	0.273	0.224	-0.373	0.508
7220	0.224	-0.373	0.262	0.053	-1.504	0.273	0.389	0.689	0.385
7221	0.482	1.279	0.142	0.474	1.208	0.341	0.492	1.346	0.409
7222	0.184	-0.625	0.481	0.404	0.756	0.309	0.097	-1.181	0.409
7223	0.500	1.397	0.501	0.477	1.227	0.341	0.505	1.430	0.501
7224	0.505	1.430	0.512	0.506	1.415	0.341	0.508	1.447	0.185
7225	0.505	1.430	0.409	0.506	1.415	0.193	0.508	1.447	0.185
7226	0.266	-0.103	0.142	0.307	0.135	0.422	0.097	-1.181	0.142
7227	0.266	-0.103	0.142	0.234	-0.336	0.512	0.050	-1.485	0.409
7228	0.492	1.346	0.296	0.515	1.472	0.491	0.513	1.481	0.328
7229	0.495	1.363	0.328	0.491	1.321	0.273	0.513	1.481	0.224
7230	0.495	1.363	0.262	0.500	1.378	0.273	0.513	1.481	0.224
7231	0.363	0.520	0.224	0.193	-0.600	0.398	0.184	-0.625	0.409
7232	0.411	0.824	0.185	0.480	1.246	0.398	0.461	1.144	0.512
7233	0.468	1.194	0.450	0.421	0.869	0.273	0.487	1.312	0.492
7234	0.142	-0.895	0.224	0.275	-0.073	0.235	0.142	-0.895	0.512
7235	0.142	-0.895	0.508	0.275	-0.073	0.398	0.142	-0.895	0.409
7236	0.295	0.082	0.501	0.339	0.342	0.444	0.263	-0.120	0.450
7237	0.447	1.060	0.508	0.310	0.153	0.501	0.500	1.397	0.185
7238	0.187	-0.608	0.185	0.342	0.361	0.508	0.329	0.301	0.492
7239	0.187	-0.608	0.492	0.342	0.361	0.422	0.142	-0.895	0.481
7240	0.226	-0.356	0.508	0.193	-0.600	0.508	0.447	1.060	0.185
7241	0.426	0.925	0.185	0.421	0.869	0.501	0.495	1.363	0.224
7242	0.513	1.481	0.501	0.509	1.434	0.341	0.479	1.262	0.481
7243	0.508	1.447	0.512	0.485	1.283	0.309	0.492	1.346	0.481
7244	0.361	0.504	0.262	0.275	-0.073	0.491	0.184	-0.625	0.467
7245	0.361	0.504	0.481	0.275	-0.073	0.512	0.184	-0.625	0.512
7246	0.184	-0.625	0.467	0.401	0.737	0.501	0.263	-0.120	0.409
7247	0.184	-0.625	0.512	0.401	0.737	0.444	0.263	-0.120	0.385
7248	0.263	-0.120	0.481	0.272	-0.091	0.491	0.358	0.487	0.262
7249	0.405	0.790	0.512	0.234	-0.336	0.508	0.261	-0.137	0.385
7250	0.447	1.060	0.142	0.398	0.718	0.444	0.513	1.481	0.262
7251	0.476	1.245	0.431	0.442	1.001	0.491	0.503	1.413	0.262
7252	0.495	1.363	0.508	0.497	1.359	0.491	0.505	1.430	0.224
7253	0.434	0.975	0.508	0.512	1.453	0.501	0.450	1.076	0.508
7254	0.142	-0.895	0.513	0.421	0.869	0.341	0.142	-0.895	0.142
7255	0.466	1.178	0.492	0.506	1.415	0.398	0.479	1.262	0.513

7256	0.408	0.807	0.142	0.506	1.415	0.235	0.474	1.228	0.501
7257	0.050	−1.485	0.142	0.339	0.342	0.193	0.295	0.082	0.501
7258	0.097	−1.181	0.358	0.307	0.135	0.478	0.184	−0.625	0.328
7259	0.463	1.161	0.512	0.494	1.340	0.444	0.505	1.430	0.481
7260	0.489	1.329	0.501	0.518	1.491	0.309	0.511	1.464	0.501
7261	0.508	1.447	0.467	0.509	1.434	0.235	0.482	1.279	0.142
7262	0.508	1.447	0.512	0.444	1.020	0.422	0.453	1.093	0.501
7263	0.500	1.397	0.431	0.494	1.340	0.501	0.466	1.178	0.512
7264	0.513	1.481	0.508	0.506	1.415	0.371	0.497	1.380	0.185
7265	0.508	1.447	0.508	0.444	1.020	0.512	0.453	1.093	0.467
7266	0.479	1.262	0.481	0.488	1.302	0.508	0.511	1.464	0.508
7267	0.463	1.161	0.185	0.488	1.302	0.462	0.508	1.447	0.492
7268	0.500	1.397	0.296	0.503	1.396	0.422	0.476	1.245	0.492
7269	0.295	0.082	0.358	0.342	0.361	0.235	0.050	−1.485	0.501
7270	0.000	?	0.097	0.401	0.737	0.462	0.097	−1.181	0.185
7271	0.224	−0.373	0.501	0.237	−0.317	0.273	0.332	0.318	0.358
7272	0.261	−0.137	0.358	0.193	−0.600	0.422	0.263	−0.120	0.501
7273	0.482	1.279	0.513	0.480	1.246	0.422	0.503	1.413	0.501
7274	0.263	−0.120	0.450	0.444	1.020	0.235	0.355	0.470	0.467
7275	0.184	−0.625	0.467	0.307	0.135	0.309	0.142	−0.895	0.492
7276	0.184	−0.625	0.492	0.234	−0.336	0.309	0.142	−0.895	0.142
7277	0.508	1.447	0.492	0.424	0.888	0.398	0.513	1.481	0.185
7278	0.479	1.262	0.513	0.500	1.378	0.514	0.489	1.329	0.097
7279	0.226	−0.356	0.450	0.374	0.568	0.514	0.408	0.807	0.508
7280	0.503	1.413	0.185	0.509	1.434	0.462	0.468	1.194	0.492
7281	0.461	1.144	0.492	0.509	1.434	0.273	0.511	1.464	0.481
7282	0.503	1.413	0.481	0.459	1.114	0.235	0.511	1.464	0.513
7283	0.466	1.178	0.224	0.424	0.888	0.149	0.497	1.380	0.467
7284	0.266	−0.103	0.185	0.234	−0.336	0.462	0.097	−1.181	0.467
7285	0.482	1.279	0.262	0.462	1.133	0.444	0.503	1.413	0.492
7286	0.261	−0.137	0.185	0.275	−0.073	0.309	0.263	−0.120	0.513
7287	0.455	1.110	0.513	0.424	0.888	0.235	0.492	1.346	0.328
7288	0.455	1.110	0.508	0.424	0.888	0.235	0.492	1.346	0.296
7289	0.142	−0.895	0.481	0.237	−0.317	0.478	0.184	−0.625	0.097
7290	0.487	1.312	0.501	0.307	0.135	0.501	0.505	1.430	0.296
7291	0.487	1.312	0.513	0.307	0.135	0.514	0.505	1.430	0.450
7292	0.142	−0.895	0.508	0.398	0.718	0.514	0.382	0.638	0.508
7293	0.482	1.279	0.142	0.520	1.509	0.514	0.408	0.807	0.328
7294	0.482	1.279	0.512	0.520	1.509	0.514	0.408	0.807	0.512
7295	0.482	1.279	0.508	0.462	1.133	0.273	0.500	1.397	0.508
7296	0.261	−0.137	0.512	0.275	−0.073	0.512	0.000	?	0.501
7297	0.329	0.301	0.512	0.237	−0.317	0.444	0.184	−0.625	0.501
7298	0.297	0.099	0.481	0.149	−0.882	0.149	0.184	−0.625	0.492
7299	0.050	−1.485	0.467	0.462	1.133	0.193	0.142	−0.895	0.450
7300	0.511	1.464	0.513	0.442	1.001	0.512	0.408	0.807	0.097
7301	0.184	−0.625	0.450	0.307	0.135	0.512	0.326	0.285	0.501
7302	0.184	−0.625	0.224	0.234	−0.336	0.462	0.226	−0.356	0.508
7303	0.184	−0.625	0.481	0.234	−0.336	0.514	0.226	−0.356	0.328
7304	0.511	1.464	0.512	0.477	1.227	0.273	0.408	0.807	0.224
7305	0.503	1.413	0.492	0.497	1.359	0.309	0.511	1.464	0.142
7306	0.468	1.194	0.467	0.512	1.453	0.235	0.487	1.312	0.224
7307	0.450	1.076	0.409	0.512	1.453	0.371	0.511	1.464	0.431
7308	0.479	1.262	0.467	0.512	1.453	0.193	0.408	0.807	0.467
7309	0.479	1.262	0.296	0.512	1.453	0.235	0.450	1.076	0.467
7310	0.097	−1.181	0.409	0.275	−0.073	0.149	0.187	−0.608	0.492
7311	0.466	1.178	0.501	0.512	1.453	0.462	0.492	1.346	0.508
7312	0.489	1.329	0.492	0.444	1.020	0.235	0.482	1.279	0.450
7313	0.266	−0.103	0.508	0.149	−0.882	0.501	0.184	−0.625	0.224
7314	0.142	−0.895	0.481	0.193	−0.600	0.491	0.224	−0.373	0.481
7315	0.476	1.245	0.185	0.515	1.472	0.491	0.479	1.262	0.467
7316	0.503	1.413	0.185	0.518	1.491	0.102	0.476	1.245	0.481

7317	0.516	1.498	0.142	0.462	1.133	0.508	0.466	1.178	0.481
7318	0.411	0.824	0.050	0.515	1.472	0.491	0.508	1.447	0.481
7319	0.142	-0.895	0.050	0.272	-0.091	0.273	0.411	0.824	0.508
7320	0.142	-0.895	0.508	0.310	0.153	0.478	0.384	0.655	0.450
7321	0.297	0.099	0.513	0.240	-0.299	0.478	0.261	-0.137	0.512
7322	0.329	0.301	0.097	0.374	0.568	0.149	0.384	0.655	0.185
7323	0.261	-0.137	0.097	0.193	-0.600	0.462	0.261	-0.137	0.467
7324	0.224	-0.373	0.224	0.234	-0.336	0.422	0.263	-0.120	0.501
7325	0.184	-0.625	0.296	0.149	-0.882	0.462	0.224	-0.373	0.512
7326	0.445	1.043	0.296	0.462	1.133	0.508	0.503	1.413	0.501
7327	0.224	-0.373	0.296	0.237	-0.317	0.398	0.142	-0.895	0.492
7328	0.500	1.397	0.262	0.503	1.396	0.235	0.516	1.498	0.492
7329	0.505	1.430	0.501	0.488	1.302	0.235	0.497	1.380	0.097
7330	0.511	1.464	0.481	0.488	1.302	0.444	0.497	1.380	0.142
7331	0.184	-0.625	0.328	0.102	-1.184	0.501	0.329	0.301	0.481
7332	0.489	1.329	0.512	0.509	1.434	0.512	0.476	1.245	0.513
7333	0.503	1.413	0.508	0.491	1.321	0.462	0.500	1.397	0.481
7334	0.187	-0.608	0.492	0.272	-0.091	0.371	0.145	-0.878	0.508
7335	0.495	1.363	0.508	0.480	1.246	0.235	0.500	1.397	0.224
7336	0.505	1.430	0.481	0.480	1.246	0.444	0.511	1.464	0.296
7337	0.266	-0.103	0.142	0.149	-0.882	0.478	0.184	-0.625	0.296
7338	0.484	1.295	0.050	0.462	1.133	0.501	0.466	1.178	0.328
7339	0.463	1.161	0.450	0.424	0.888	0.478	0.508	1.447	0.328
7340	0.500	1.397	0.142	0.465	1.152	0.371	0.495	1.363	0.492
7341	0.479	1.262	0.409	0.506	1.415	0.422	0.492	1.346	0.513
7342	0.511	1.464	0.224	0.398	0.718	0.478	0.497	1.380	0.185
7343	0.145	-0.878	0.450	0.237	-0.317	0.478	0.184	-0.625	0.142
7344	0.437	0.992	0.492	0.234	-0.336	0.501	0.355	0.470	0.224
7345	0.500	1.397	0.262	0.447	1.039	0.235	0.503	1.413	0.097
7346	0.497	1.380	0.142	0.506	1.415	0.422	0.503	1.413	0.097
7347	0.503	1.413	0.492	0.515	1.472	0.193	0.466	1.178	0.097
7348	0.492	1.346	0.481	0.465	1.152	0.193	0.492	1.346	0.185
7349	0.142	-0.895	0.328	0.374	0.568	0.508	0.142	-0.895	0.467
7350	0.142	-0.895	0.450	0.237	-0.317	0.501	0.184	-0.625	0.467
7351	0.361	0.504	0.508	0.444	1.020	0.512	0.097	-1.181	0.224
7352	0.511	1.464	0.508	0.474	1.208	0.508	0.503	1.413	0.481
7353	0.495	1.363	0.492	0.503	1.396	0.508	0.489	1.329	0.501
7354	0.468	1.194	0.501	0.474	1.208	0.512	0.476	1.245	0.508
7355	0.500	1.397	0.501	0.374	0.568	0.444	0.513	1.481	0.481
7356	0.429	0.942	0.501	0.424	0.888	0.478	0.468	1.194	0.508
7357	0.505	1.430	0.501	0.480	1.246	0.501	0.463	1.161	0.262
7358	0.505	1.430	0.431	0.480	1.246	0.273	0.487	1.312	0.224
7359	0.479	1.262	0.142	0.497	1.359	0.462	0.511	1.464	0.481
7360	0.187	-0.608	0.185	0.234	-0.336	0.514	0.326	0.285	0.262
7361	0.295	0.082	0.512	0.424	0.888	0.273	0.300	0.116	0.512
7362	0.358	0.487	0.508	0.193	-0.600	0.149	0.097	-1.181	0.185
7363	0.097	-1.181	0.328	0.193	-0.600	0.235	0.300	0.116	0.513
7364	0.505	1.430	0.467	0.506	1.415	0.235	0.447	1.060	0.431
7365	0.355	0.470	0.467	0.494	1.340	0.508	0.508	1.447	0.097
7366	0.511	1.464	0.185	0.509	1.434	0.514	0.326	0.285	0.142
7367	0.447	1.060	0.142	0.503	1.396	0.422	0.511	1.464	0.097
7368	0.468	1.194	0.409	0.512	1.453	0.491	0.511	1.464	0.512
7369	0.497	1.380	0.508	0.512	1.453	0.491	0.503	1.413	0.481
7370	0.495	1.363	0.512	0.444	1.020	0.491	0.495	1.363	0.262
7371	0.508	1.447	0.512	0.480	1.246	0.193	0.489	1.329	0.492
7372	0.450	1.076	0.512	0.500	1.378	0.508	0.450	1.076	0.512
7373	0.184	-0.625	0.450	0.275	-0.073	0.512	0.097	-1.181	0.512
7374	0.495	1.363	0.481	0.462	1.133	0.491	0.500	1.397	0.513
7375	0.482	1.279	0.512	0.518	1.491	0.491	0.508	1.447	0.492
7376	0.224	-0.373	0.481	0.278	-0.054	0.512	0.326	0.285	0.508
7377	0.184	-0.625	0.296	0.152	-0.863	0.501	0.224	-0.373	0.513

7378	0.263	-0.120	0.467	0.234	-0.336	0.501	0.142	-0.895	0.512
7379	0.187	-0.608	0.467	0.234	-0.336	0.444	0.226	-0.356	0.512
7380	0.511	1.464	0.409	0.512	1.453	0.371	0.429	0.942	0.262
7381	0.503	1.413	0.358	0.509	1.434	0.462	0.466	1.178	0.296
7382	0.487	1.312	0.050	0.421	0.869	0.501	0.463	1.161	0.513
7383	0.500	1.397	0.185	0.491	1.321	0.512	0.489	1.329	0.513
7384	0.513	1.481	0.296	0.491	1.321	0.478	0.508	1.447	0.358
7385	0.505	1.430	0.512	0.491	1.321	0.512	0.447	1.060	0.512
7386	0.142	-0.895	0.296	0.193	-0.600	0.512	0.224	-0.373	0.512
7387	0.508	1.447	0.224	0.509	1.434	0.235	0.479	1.262	0.358
7388	0.500	1.397	0.450	0.512	1.453	0.193	0.466	1.178	0.328
7389	0.513	1.481	0.142	0.488	1.302	0.491	0.479	1.262	0.513
7390	0.513	1.481	0.513	0.488	1.302	0.462	0.479	1.262	0.508
7391	0.479	1.262	0.501	0.509	1.434	0.512	0.479	1.262	0.508
7392	0.466	1.178	0.508	0.500	1.378	0.501	0.500	1.397	0.508
7393	0.513	1.481	0.142	0.503	1.396	0.193	0.450	1.076	0.501
7394	0.450	1.076	0.467	0.444	1.020	0.149	0.508	1.447	0.501
7395	0.226	-0.356	0.262	0.371	0.549	0.149	0.184	-0.625	0.513
7396	0.479	1.262	0.431	0.459	1.114	0.398	0.466	1.178	0.508
7397	0.500	1.397	0.328	0.500	1.378	0.341	0.505	1.430	0.450
7398	0.484	1.295	0.142	0.509	1.434	0.462	0.513	1.481	0.185
7399	0.466	1.178	0.501	0.477	1.227	0.422	0.497	1.380	0.481
7400	0.408	0.807	0.501	0.509	1.434	0.398	0.489	1.329	0.508
7401	0.466	1.178	0.481	0.509	1.434	0.309	0.489	1.329	0.492
7402	0.297	0.099	0.512	0.234	-0.336	0.102	0.097	-1.181	0.097
7403	0.411	0.824	0.501	0.193	-0.600	0.149	0.142	-0.895	0.262
7404	0.503	1.413	0.501	0.488	1.302	0.149	0.479	1.262	0.185
7405	0.495	1.363	0.508	0.459	1.114	0.149	0.511	1.464	0.224
7406	0.508	1.447	0.512	0.512	1.453	0.149	0.479	1.262	0.409
7407	0.482	1.279	0.097	0.500	1.378	0.508	0.511	1.464	0.224
7408	0.184	-0.625	0.050	0.193	-0.600	0.478	0.224	-0.373	0.185
7409	0.184	-0.625	0.481	0.149	-0.882	0.371	0.295	0.082	0.501
7410	0.142	-0.895	0.185	0.149	-0.882	0.491	0.295	0.082	0.296
7411	0.050	-1.485	0.501	0.398	0.718	0.514	0.329	0.301	0.513
7412	0.050	-1.485	0.450	0.345	0.379	0.501	0.329	0.301	0.492
7413	0.497	1.380	0.262	0.459	1.114	0.444	0.497	1.380	0.501
7414	0.513	1.481	0.358	0.421	0.869	0.422	0.508	1.447	0.185
7415	0.097	-1.181	0.185	0.401	0.737	0.309	0.184	-0.625	0.501
7416	0.097	-1.181	0.328	0.307	0.135	0.309	0.142	-0.895	0.185
7417	0.224	-0.373	0.492	0.102	-1.184	0.514	0.224	-0.373	0.409
7418	0.300	0.116	0.508	0.149	-0.882	0.193	0.097	-1.181	0.296
7419	0.300	0.116	0.508	0.149	-0.882	0.501	0.097	-1.181	0.142
7420	0.300	0.116	0.450	0.149	-0.882	0.149	0.097	-1.181	0.431
7421	0.263	-0.120	0.450	0.149	-0.882	0.514	0.184	-0.625	0.501
7422	0.503	1.413	0.450	0.503	1.396	0.501	0.463	1.161	0.501
7423	0.479	1.262	0.501	0.474	1.208	0.398	0.468	1.194	0.431
7424	0.329	0.301	0.296	0.374	0.568	0.235	0.226	-0.356	0.501
7425	0.508	1.447	0.142	0.488	1.302	0.371	0.482	1.279	0.513
7426	0.513	1.481	0.501	0.518	1.491	0.462	0.500	1.397	0.492
7427	0.495	1.363	0.142	0.503	1.396	0.478	0.508	1.447	0.450
7428	0.508	1.447	0.467	0.442	1.001	0.371	0.474	1.228	0.409
7429	0.482	1.279	0.501	0.424	0.888	0.512	0.513	1.481	0.358
7430	0.142	-0.895	0.508	0.313	0.172	0.462	0.261	-0.137	0.512
7431	0.050	-1.485	0.481	0.307	0.135	0.462	0.226	-0.356	0.142
7432	0.447	1.060	0.142	0.512	1.453	0.462	0.479	1.262	0.492
7433	0.142	-0.895	0.501	0.193	-0.600	0.501	0.263	-0.120	0.492
7434	0.411	0.824	0.508	0.500	1.378	0.508	0.508	1.447	0.328
7435	0.226	-0.356	0.450	0.149	-0.882	0.508	0.187	-0.608	0.409
7436	0.450	1.076	0.492	0.512	1.453	0.478	0.516	1.498	0.185
7437	0.492	1.346	0.224	0.503	1.396	0.444	0.432	0.958	0.262
7438	0.000	?	0.224	0.398	0.718	0.235	0.097	-1.181	0.492

7439	0.261	−0.137	0.431	0.240	−0.299	0.149	0.142	−0.895	0.481
7440	0.142	−0.895	0.513	0.371	0.549	0.462	0.097	−1.181	0.513
7441	0.487	1.312	0.142	0.465	1.152	0.444	0.508	1.447	0.185
7442	0.476	1.245	0.097	0.480	1.246	0.309	0.476	1.245	0.185
7443	0.329	0.301	0.097	0.377	0.587	0.512	0.261	−0.137	0.185
7444	0.450	1.076	0.512	0.509	1.434	0.512	0.492	1.346	0.508
7445	0.508	1.447	0.467	0.459	1.114	0.273	0.513	1.481	0.097
7446	0.508	1.447	0.492	0.459	1.114	0.341	0.513	1.481	0.296
7447	0.492	1.346	0.501	0.459	1.114	0.512	0.516	1.498	0.508
7448	0.497	1.380	0.185	0.497	1.359	0.508	0.492	1.346	0.185
7449	0.495	1.363	0.185	0.506	1.415	0.491	0.508	1.447	0.492
7450	0.497	1.380	0.224	0.506	1.415	0.491	0.516	1.498	0.431
7451	0.497	1.380	0.224	0.482	1.265	0.508	0.508	1.447	0.431
7452	0.426	0.925	0.328	0.447	1.039	0.422	0.516	1.498	0.512
7453	0.142	−0.895	0.185	0.234	−0.336	0.512	0.266	−0.103	0.262
7454	0.184	−0.625	0.224	0.149	−0.882	0.478	0.295	0.082	0.512
7455	0.505	1.430	0.450	0.459	1.114	0.478	0.513	1.481	0.492
7456	0.503	1.413	0.224	0.442	1.001	0.371	0.513	1.481	0.492
7457	0.332	0.318	0.262	0.307	0.135	0.508	0.361	0.504	0.492
7458	0.463	1.161	0.512	0.515	1.472	0.462	0.516	1.498	0.050
7459	0.463	1.161	0.467	0.515	1.472	0.512	0.516	1.498	0.050
7460	0.184	−0.625	0.492	0.275	−0.073	0.309	0.355	0.470	0.492
7461	0.142	−0.895	0.467	0.345	0.379	0.309	0.329	0.301	0.358
7462	0.405	0.790	0.467	0.509	1.434	0.193	0.513	1.481	0.296
7463	0.500	1.397	0.467	0.503	1.396	0.193	0.511	1.464	0.328
7464	0.505	1.430	0.467	0.494	1.340	0.462	0.505	1.430	0.328
7465	0.505	1.430	0.450	0.494	1.340	0.193	0.505	1.430	0.450
7466	0.508	1.447	0.097	0.506	1.415	0.193	0.497	1.380	0.481
7467	0.450	1.076	0.050	0.421	0.869	0.508	0.500	1.397	0.501
7468	0.479	1.262	0.050	0.509	1.434	0.273	0.511	1.464	0.501
7469	0.516	1.498	0.050	0.482	1.265	0.444	0.500	1.397	0.328
7470	0.482	1.279	0.097	0.477	1.227	0.491	0.453	1.093	0.097
7471	0.297	0.099	0.512	0.371	0.549	0.491	0.187	−0.608	0.328
7472	0.466	1.178	0.142	0.509	1.434	0.235	0.479	1.262	0.142
7473	0.463	1.161	0.481	0.462	1.133	0.514	0.503	1.413	0.142
7474	0.411	0.824	0.142	0.518	1.491	0.341	0.484	1.295	0.224
7475	0.358	0.487	0.296	0.313	0.172	0.491	0.097	−1.181	0.431
7476	0.050	−1.485	0.296	0.307	0.135	0.193	0.263	−0.120	0.050
7477	0.184	−0.625	0.262	0.193	−0.600	0.309	0.184	−0.625	0.142
7478	0.295	0.082	0.142	0.193	−0.600	0.508	0.226	−0.356	0.512
7479	0.505	1.430	0.409	0.462	1.133	0.478	0.408	0.807	0.501
7480	0.295	0.082	0.224	0.193	−0.600	0.478	0.226	−0.356	0.508
7481	0.224	−0.373	0.492	0.193	−0.600	0.478	0.184	−0.625	0.467
7482	0.447	1.060	0.185	0.509	1.434	0.512	0.495	1.363	0.492
7483	0.142	−0.895	0.481	0.275	−0.073	0.508	0.297	0.099	0.492
7484	0.513	1.481	0.185	0.447	1.039	0.501	0.511	1.464	0.492
7485	0.492	1.346	0.224	0.491	1.321	0.478	0.492	1.346	0.492
7486	0.497	1.380	0.262	0.491	1.321	0.371	0.503	1.413	0.296
7487	0.145	−0.878	0.481	0.234	−0.336	0.309	0.184	−0.625	0.224
7488	0.466	1.178	0.481	0.512	1.453	0.491	0.495	1.363	0.262
7489	0.266	−0.103	0.185	0.342	0.361	0.235	0.184	−0.625	0.224
7490	0.429	0.942	0.492	0.485	1.283	0.422	0.413	0.841	0.185
7491	0.332	0.318	0.185	0.193	−0.600	0.444	0.297	0.099	0.512
7492	0.142	−0.895	0.492	0.313	0.172	0.341	0.142	−0.895	0.185
7493	0.503	1.413	0.185	0.509	1.434	0.193	0.429	0.942	0.481
7494	0.497	1.380	0.385	0.477	1.227	0.193	0.500	1.397	0.224
7495	0.476	1.245	0.450	0.477	1.227	0.235	0.500	1.397	0.142
7496	0.508	1.447	0.185	0.474	1.208	0.478	0.429	0.942	0.142
7497	0.500	1.397	0.501	0.509	1.434	0.512	0.497	1.380	0.185
7498	0.500	1.397	0.512	0.506	1.415	0.491	0.508	1.447	0.224
7499	0.508	1.447	0.501	0.500	1.378	0.273	0.489	1.329	0.508

7500	0.511	1.464	0.501	0.477	1.227	0.273	0.447	1.060	0.358
7501	0.097	-1.181	0.097	0.371	0.549	0.273	0.408	0.807	0.508
7502	0.050	-1.485	0.296	0.313	0.172	0.501	0.355	0.470	0.409
7503	0.476	1.245	0.481	0.491	1.321	0.422	0.511	1.464	0.450
7504	0.184	-0.625	0.142	0.237	-0.317	0.309	0.142	-0.895	0.409
7505	0.503	1.413	0.492	0.424	0.888	0.398	0.487	1.312	0.142
7506	0.450	1.076	0.262	0.444	1.020	0.341	0.492	1.346	0.050
7507	0.261	-0.137	0.431	0.339	0.342	0.478	0.326	0.285	0.501
7508	0.361	0.504	0.501	0.193	-0.600	0.508	0.411	0.824	0.501
7509	0.184	-0.625	0.513	0.196	-0.581	0.491	0.184	-0.625	0.142
7510	0.329	0.301	0.513	0.240	-0.299	0.462	0.261	-0.137	0.492
7511	0.495	1.363	0.050	0.477	1.227	0.309	0.492	1.346	0.262
7512	0.505	1.430	0.262	0.512	1.453	0.508	0.484	1.295	0.508
7513	0.508	1.447	0.358	0.488	1.302	0.501	0.513	1.481	0.050
7514	0.453	1.093	0.467	0.272	-0.091	0.501	0.187	-0.608	0.450
7515	0.453	1.093	0.431	0.272	-0.091	0.501	0.187	-0.608	0.431
7516	0.453	1.093	0.501	0.272	-0.091	0.309	0.187	-0.608	0.185
7517	0.497	1.380	0.467	0.503	1.396	0.309	0.508	1.447	0.467
7518	0.297	0.099	0.185	0.424	0.888	0.478	0.097	-1.181	0.385
7519	0.145	-0.878	0.508	0.307	0.135	0.462	0.297	0.099	0.512
7520	0.500	1.397	0.262	0.401	0.737	0.273	0.505	1.430	0.358
7521	0.142	-0.895	0.512	0.345	0.379	0.193	0.184	-0.625	0.512
7522	0.466	1.178	0.508	0.485	1.283	0.193	0.489	1.329	0.185
7523	0.503	1.413	0.512	0.509	1.434	0.478	0.429	0.942	0.328
7524	0.508	1.447	0.512	0.491	1.321	0.491	0.429	0.942	0.492
7525	0.476	1.245	0.513	0.462	1.133	0.491	0.513	1.481	0.224
7526	0.142	-0.895	0.262	0.313	0.172	0.478	0.263	-0.120	0.385
7527	0.503	1.413	0.512	0.506	1.415	0.273	0.505	1.430	0.142
7528	0.505	1.430	0.508	0.494	1.340	0.273	0.492	1.346	0.508
7529	0.453	1.093	0.262	0.506	1.415	0.273	0.487	1.312	0.450
7530	0.497	1.380	0.385	0.503	1.396	0.235	0.489	1.329	0.501
7531	0.226	-0.356	0.481	0.310	0.153	0.193	0.050	-1.485	0.501
7532	0.226	-0.356	0.358	0.310	0.153	0.398	0.050	-1.485	0.262
7533	0.429	0.942	0.224	0.480	1.246	0.235	0.497	1.380	0.050
7534	0.511	1.464	0.492	0.462	1.133	0.514	0.363	0.520	0.296
7535	0.142	-0.895	0.508	0.275	-0.073	0.371	0.297	0.099	0.513
7536	0.097	-1.181	0.050	0.193	-0.600	0.149	0.332	0.318	0.508
7537	0.097	-1.181	0.142	0.193	-0.600	0.444	0.332	0.318	0.481
7538	0.513	1.481	0.501	0.485	1.283	0.444	0.447	1.060	0.501
7539	0.468	1.194	0.358	0.497	1.359	0.512	0.479	1.262	0.185
7540	0.489	1.329	0.262	0.494	1.340	0.491	0.505	1.430	0.512
7541	0.500	1.397	0.492	0.482	1.265	0.491	0.505	1.430	0.185
7542	0.187	-0.608	0.501	0.272	-0.091	0.462	0.326	0.285	0.467
7543	0.187	-0.608	0.050	0.275	-0.073	0.462	0.097	-1.181	0.481
7544	0.226	-0.356	0.508	0.272	-0.091	0.514	0.326	0.285	0.431
7545	0.224	-0.373	0.513	0.234	-0.336	0.149	0.142	-0.895	0.467
7546	0.326	0.285	0.481	0.193	-0.600	0.309	0.000	?	0.467
7547	0.184	-0.625	0.050	0.398	0.718	0.309	0.142	-0.895	0.262
7548	0.226	-0.356	0.512	0.237	-0.317	0.309	0.224	-0.373	0.501
7549	0.450	1.076	0.431	0.520	1.509	0.309	0.429	0.942	0.501
7550	0.224	-0.373	0.328	0.374	0.568	0.512	0.050	-1.485	0.224
7551	0.263	-0.120	0.508	0.149	-0.882	0.235	0.142	-0.895	0.224
7552	0.513	1.481	0.512	0.444	1.020	0.512	0.516	1.498	0.358
7553	0.468	1.194	0.450	0.447	1.039	0.193	0.500	1.397	0.508
7554	0.492	1.346	0.512	0.512	1.453	0.102	0.513	1.481	0.431
7555	0.466	1.178	0.450	0.491	1.321	0.149	0.463	1.161	0.513
7556	0.463	1.161	0.492	0.491	1.321	0.149	0.492	1.346	0.450
7557	0.463	1.161	0.492	0.462	1.133	0.235	0.492	1.346	0.142
7558	0.463	1.161	0.492	0.462	1.133	0.462	0.492	1.346	0.262
7559	0.445	1.043	0.513	0.509	1.434	0.193	0.492	1.346	0.481
7560	0.097	-1.181	0.513	0.152	-0.863	0.501	0.297	0.099	0.097

7561	0.050	-1.485	0.513	0.310	0.153	0.309	0.226	-0.356	0.142
7562	0.050	-1.485	0.513	0.310	0.153	0.478	0.263	-0.120	0.492
7563	0.050	-1.485	0.050	0.310	0.153	0.235	0.226	-0.356	0.262
7564	0.097	-1.181	0.512	0.307	0.135	0.273	0.187	-0.608	0.450
7565	0.505	1.430	0.508	0.512	1.453	0.273	0.511	1.464	0.328
7566	0.142	-0.895	0.492	0.237	-0.317	0.501	0.187	-0.608	0.492
7567	0.476	1.245	0.050	0.512	1.453	0.501	0.479	1.262	0.513
7568	0.145	-0.878	0.481	0.193	-0.600	0.273	0.224	-0.373	0.492
7569	0.297	0.099	0.142	0.102	-1.184	0.501	0.142	-0.895	0.262
7570	0.297	0.099	0.512	0.152	-0.863	0.149	0.142	-0.895	0.328
7571	0.263	-0.120	0.328	0.152	-0.863	0.508	0.184	-0.625	0.450
7572	0.142	-0.895	0.501	0.237	-0.317	0.444	0.224	-0.373	0.328
7573	0.413	0.841	0.224	0.465	1.152	0.508	0.505	1.430	0.296
7574	0.226	-0.356	0.508	0.193	-0.600	0.508	0.358	0.487	0.501
7575	0.492	1.346	0.481	0.503	1.396	0.193	0.497	1.380	0.492
7576	0.187	-0.608	0.501	0.310	0.153	0.491	0.411	0.824	0.450
7577	0.476	1.245	0.508	0.474	1.208	0.512	0.450	1.076	0.512
7578	0.187	-0.608	0.513	0.234	-0.336	0.512	0.411	0.824	0.450
7579	0.224	-0.373	0.508	0.272	-0.091	0.371	0.142	-0.895	0.409
7580	0.263	-0.120	0.262	0.272	-0.091	0.512	0.050	-1.485	0.409
7581	0.484	1.295	0.262	0.500	1.378	0.273	0.492	1.346	0.409
7582	0.484	1.295	0.262	0.500	1.378	0.053	0.492	1.346	0.450
7583	0.184	-0.625	0.450	0.272	-0.091	0.422	0.142	-0.895	0.467
7584	0.492	1.346	0.262	0.497	1.359	0.235	0.492	1.346	0.450
7585	0.184	-0.625	0.097	0.152	-0.863	0.501	0.263	-0.120	0.450
7586	0.492	1.346	0.097	0.503	1.396	0.309	0.508	1.447	0.097
7587	0.187	-0.608	0.467	0.444	1.020	0.444	0.050	-1.485	0.512
7588	0.384	0.655	0.512	0.509	1.434	0.478	0.447	1.060	0.512
7589	0.450	1.076	0.513	0.506	1.415	0.462	0.426	0.925	0.512
7590	0.184	-0.625	0.492	0.196	-0.581	0.462	0.187	-0.608	0.262
7591	0.495	1.363	0.501	0.491	1.321	0.309	0.463	1.161	0.508
7592	0.505	1.430	0.481	0.509	1.434	0.235	0.384	0.655	0.097
7593	0.492	1.346	0.492	0.515	1.472	0.341	0.513	1.481	0.431
7594	0.000	?	0.224	0.374	0.568	0.508	0.355	0.470	0.492
7595	0.492	1.346	0.467	0.515	1.472	0.491	0.513	1.481	0.097
7596	0.097	-1.181	0.512	0.275	-0.073	0.491	0.184	-0.625	0.501
7597	0.300	0.116	0.512	0.053	-1.504	0.478	0.326	0.285	0.512
7598	0.479	1.262	0.481	0.418	0.850	0.273	0.492	1.346	0.450
7599	0.142	-0.895	0.492	0.237	-0.317	0.309	0.224	-0.373	0.450
7600	0.487	1.312	0.508	0.500	1.378	0.273	0.384	0.655	0.512
7601	0.261	-0.137	0.512	0.307	0.135	0.508	0.142	-0.895	0.492
7602	0.429	0.942	0.492	0.447	1.039	0.501	0.505	1.430	0.050
7603	0.500	1.397	0.431	0.477	1.227	0.462	0.453	1.093	0.050
7604	0.503	1.413	0.508	0.465	1.152	0.478	0.505	1.430	0.050
7605	0.503	1.413	0.508	0.465	1.152	0.444	0.505	1.430	0.513
7606	0.050	-1.485	0.512	0.310	0.153	0.149	0.261	-0.137	0.097
7607	0.263	-0.120	0.467	0.237	-0.317	0.508	0.050	-1.485	0.385
7608	0.358	0.487	0.467	0.342	0.361	0.398	0.295	0.082	0.385
7609	0.468	1.194	0.097	0.506	1.415	0.053	0.505	1.430	0.450
7610	0.432	0.958	0.185	0.488	1.302	0.444	0.500	1.397	0.481
7611	0.500	1.397	0.492	0.494	1.340	0.501	0.474	1.228	0.431
7612	0.466	1.178	0.431	0.474	1.208	0.478	0.503	1.413	0.512
7613	0.184	-0.625	0.467	0.275	-0.073	0.149	0.187	-0.608	0.513
7614	0.508	1.447	0.185	0.310	0.153	0.491	0.513	1.481	0.492
7615	0.263	-0.120	0.185	0.275	-0.073	0.444	0.187	-0.608	0.512
7616	0.508	1.447	0.481	0.509	1.434	0.371	0.466	1.178	0.142
7617	0.505	1.430	0.501	0.503	1.396	0.371	0.476	1.245	0.508
7618	0.508	1.447	0.450	0.462	1.133	0.462	0.426	0.925	0.501
7619	0.511	1.464	0.262	0.480	1.246	0.149	0.466	1.178	0.481
7620	0.513	1.481	0.262	0.442	1.001	0.309	0.466	1.178	0.501
7621	0.261	-0.137	0.296	0.149	-0.882	0.398	0.261	-0.137	0.512

7622	0.500	1.397	0.185	0.509	1.434	0.273	0.503	1.413	0.501
7623	0.500	1.397	0.185	0.398	0.718	0.508	0.503	1.413	0.481
7624	0.261	-0.137	0.467	0.053	-1.504	0.193	0.226	-0.356	0.409
7625	0.387	0.672	0.409	0.439	0.982	0.508	0.224	-0.373	0.224
7626	0.479	1.262	0.296	0.494	1.340	0.462	0.363	0.520	0.224
7627	0.355	0.470	0.224	0.482	1.265	0.491	0.508	1.447	0.224
7628	0.226	-0.356	0.185	0.149	-0.882	0.309	0.432	0.958	0.501
7629	0.489	1.329	0.097	0.485	1.283	0.193	0.511	1.464	0.431
7630	0.500	1.397	0.262	0.447	1.039	0.398	0.445	1.043	0.501
7631	0.050	-1.485	0.512	0.374	0.568	0.444	0.142	-0.895	0.512
7632	0.142	-0.895	0.185	0.371	0.549	0.309	0.261	-0.137	0.467
7633	0.497	1.380	0.185	0.459	1.114	0.514	0.476	1.245	0.467
7634	0.355	0.470	0.142	0.149	-0.882	0.508	0.097	-1.181	0.431
7635	0.266	-0.103	0.492	0.307	0.135	0.512	0.142	-0.895	0.328
7636	0.489	1.329	0.262	0.401	0.737	0.462	0.492	1.346	0.185
7637	0.000	?	0.508	0.272	-0.091	0.508	0.263	-0.120	0.512
7638	0.492	1.346	0.224	0.509	1.434	0.491	0.447	1.060	0.050
7639	0.050	-1.485	0.501	0.193	-0.600	0.491	0.332	0.318	0.467
7640	0.497	1.380	0.492	0.512	1.453	0.508	0.492	1.346	0.185
7641	0.500	1.397	0.262	0.465	1.152	0.512	0.513	1.481	0.142
7642	0.482	1.279	0.185	0.491	1.321	0.512	0.484	1.295	0.513
7643	0.000	?	0.262	0.313	0.172	0.512	0.261	-0.137	0.450
7644	0.050	-1.485	0.508	0.193	-0.600	0.508	0.329	0.301	0.501
7645	0.505	1.430	0.508	0.401	0.737	0.371	0.453	1.093	0.097
7646	0.429	0.942	0.142	0.447	1.039	0.491	0.329	0.301	0.097
7647	0.329	0.301	0.481	0.310	0.153	0.501	0.295	0.082	0.185
7648	0.505	1.430	0.513	0.515	1.472	0.508	0.497	1.380	0.262
7649	0.513	1.481	0.492	0.509	1.434	0.273	0.495	1.363	0.328
7650	0.447	1.060	0.224	0.509	1.434	0.462	0.453	1.093	0.492
7651	0.503	1.413	0.224	0.459	1.114	0.309	0.516	1.498	0.097
7652	0.453	1.093	0.296	0.509	1.434	0.501	0.453	1.093	0.296
7653	0.492	1.346	0.328	0.494	1.340	0.371	0.405	0.790	0.296
7654	0.492	1.346	0.431	0.494	1.340	0.444	0.405	0.790	0.224
7655	0.492	1.346	0.262	0.512	1.453	0.235	0.405	0.790	0.142
7656	0.503	1.413	0.328	0.515	1.472	0.508	0.447	1.060	0.142
7657	0.503	1.413	0.492	0.520	1.509	0.508	0.463	1.161	0.481
7658	0.503	1.413	0.513	0.520	1.509	0.462	0.447	1.060	0.142
7659	0.503	1.413	0.513	0.515	1.472	0.444	0.447	1.060	0.142
7660	0.050	-1.485	0.512	0.374	0.568	0.478	0.097	-1.181	0.142
7661	0.508	1.447	0.409	0.488	1.302	0.478	0.508	1.447	0.508
7662	0.503	1.413	0.142	0.500	1.378	0.309	0.508	1.447	0.050
7663	0.489	1.329	0.050	0.503	1.396	0.309	0.511	1.464	0.501
7664	0.050	-1.485	0.508	0.272	-0.091	0.309	0.261	-0.137	0.185
7665	0.479	1.262	0.481	0.459	1.114	0.508	0.505	1.430	0.467
7666	0.142	-0.895	0.262	0.310	0.153	0.193	0.097	-1.181	0.512
7667	0.513	1.481	0.467	0.500	1.378	0.053	0.426	0.925	0.050
7668	0.332	0.318	0.450	0.371	0.549	0.053	0.000	?	0.142
7669	0.503	1.413	0.296	0.442	1.001	0.501	0.492	1.346	0.492
7670	0.224	-0.373	0.501	0.237	-0.317	0.512	0.100	-1.164	0.492
7671	0.505	1.430	0.513	0.503	1.396	0.508	0.500	1.397	0.185
7672	0.479	1.262	0.296	0.503	1.396	0.478	0.511	1.464	0.513
7673	0.497	1.380	0.508	0.462	1.133	0.478	0.450	1.076	0.358
7674	0.503	1.413	0.467	0.444	1.020	0.491	0.450	1.076	0.467
7675	0.500	1.397	0.481	0.482	1.265	0.491	0.518	1.514	0.050
7676	0.503	1.413	0.501	0.477	1.227	0.193	0.495	1.363	0.050
7677	0.261	-0.137	0.508	0.307	0.135	0.491	0.050	-1.485	0.512
7678	0.261	-0.137	0.467	0.307	0.135	0.501	0.050	-1.485	0.328
7679	0.261	-0.137	0.508	0.307	0.135	0.491	0.050	-1.485	0.050
7680	0.447	1.060	0.385	0.509	1.434	0.444	0.511	1.464	0.467
7681	0.261	-0.137	0.262	0.193	-0.600	0.514	0.097	-1.181	0.513
7682	0.097	-1.181	0.328	0.053	-1.504	0.501	0.387	0.672	0.513

7683	0.097	-1.181	0.501	0.053	-1.504	0.478	0.387	0.672	0.513
7684	0.461	1.144	0.512	0.503	1.396	0.501	0.450	1.076	0.481
7685	0.508	1.447	0.185	0.509	1.434	0.341	0.479	1.262	0.224
7686	0.508	1.447	0.481	0.515	1.472	0.371	0.432	0.958	0.224
7687	0.489	1.329	0.501	0.482	1.265	0.341	0.511	1.464	0.262
7688	0.497	1.380	0.492	0.482	1.265	0.512	0.513	1.481	0.467
7689	0.479	1.262	0.224	0.497	1.359	0.478	0.492	1.346	0.508
7690	0.489	1.329	0.481	0.488	1.302	0.501	0.511	1.464	0.185
7691	0.224	-0.373	0.185	0.193	-0.600	0.491	0.142	-0.895	0.492
7692	0.468	1.194	0.224	0.488	1.302	0.512	0.500	1.397	0.481
7693	0.511	1.464	0.185	0.497	1.359	0.512	0.497	1.380	0.142
7694	0.511	1.464	0.467	0.488	1.302	0.193	0.479	1.262	0.512
7695	0.484	1.295	0.512	0.439	0.982	0.309	0.503	1.413	0.467
7696	0.487	1.312	0.224	0.518	1.491	0.235	0.508	1.447	0.467
7697	0.505	1.430	0.224	0.500	1.378	0.371	0.500	1.397	0.409
7698	0.513	1.481	0.224	0.477	1.227	0.102	0.482	1.279	0.508
7699	0.492	1.346	0.262	0.503	1.396	0.512	0.411	0.824	0.508
7700	0.000	?	0.501	0.342	0.361	0.371	0.224	-0.373	0.513
7701	0.000	?	0.224	0.371	0.549	0.235	0.224	-0.373	0.513
7702	0.000	?	0.224	0.342	0.361	0.491	0.224	-0.373	0.512
7703	0.426	0.925	0.492	0.512	1.453	0.462	0.503	1.413	0.481
7704	0.508	1.447	0.296	0.477	1.227	0.444	0.426	0.925	0.142
7705	0.503	1.413	0.296	0.497	1.359	0.235	0.503	1.413	0.508
7706	0.508	1.447	0.512	0.491	1.321	0.235	0.511	1.464	0.262
7707	0.466	1.178	0.450	0.512	1.453	0.193	0.461	1.144	0.501
7708	0.466	1.178	0.450	0.512	1.453	0.149	0.461	1.144	0.467
7709	0.100	-1.164	0.508	0.193	-0.600	0.273	0.426	0.925	0.224
7710	0.000	?	0.501	0.310	0.153	0.462	0.329	0.301	0.512
7711	0.184	-0.625	0.262	0.234	-0.336	0.273	0.184	-0.625	0.467
7712	0.495	1.363	0.481	0.374	0.568	0.102	0.505	1.430	0.492
7713	0.432	0.958	0.512	0.102	-1.184	0.193	0.050	-1.485	0.358
7714	0.466	1.178	0.492	0.509	1.434	0.149	0.461	1.144	0.508
7715	0.184	-0.625	0.224	0.374	0.568	0.398	0.184	-0.625	0.224
7716	0.184	-0.625	0.512	0.237	-0.317	0.149	0.142	-0.895	0.328
7717	0.479	1.262	0.481	0.500	1.378	0.501	0.511	1.464	0.142
7718	0.497	1.380	0.467	0.471	1.189	0.273	0.447	1.060	0.431
7719	0.450	1.076	0.142	0.444	1.020	0.273	0.492	1.346	0.385
7720	0.263	-0.120	0.450	0.237	-0.317	0.273	0.097	-1.181	0.262
7721	0.263	-0.120	0.328	0.234	-0.336	0.501	0.097	-1.181	0.262
7722	0.295	0.082	0.508	0.196	-0.581	0.273	0.184	-0.625	0.262
7723	0.184	-0.625	0.224	0.149	-0.882	0.478	0.261	-0.137	0.185
7724	0.187	-0.608	0.262	0.275	-0.073	0.309	0.329	0.301	0.481
7725	0.463	1.161	0.224	0.465	1.152	0.508	0.492	1.346	0.328
7726	0.408	0.807	0.508	0.272	-0.091	0.398	0.097	-1.181	0.328
7727	0.295	0.082	0.328	0.102	-1.184	0.235	0.297	0.099	0.467
7728	0.224	-0.373	0.508	0.196	-0.581	0.341	0.297	0.099	0.185
7729	0.187	-0.608	0.262	0.149	-0.882	0.149	0.224	-0.373	0.050
7730	0.097	-1.181	0.097	0.398	0.718	0.508	0.142	-0.895	0.431
7731	0.261	-0.137	0.512	0.152	-0.863	0.508	0.145	-0.878	0.501
7732	0.505	1.430	0.512	0.500	1.378	0.235	0.479	1.262	0.501
7733	0.184	-0.625	0.513	0.275	-0.073	0.501	0.142	-0.895	0.492
7734	0.184	-0.625	0.492	0.275	-0.073	0.444	0.142	-0.895	0.501
7735	0.142	-0.895	0.512	0.275	-0.073	0.491	0.142	-0.895	0.097
7736	0.489	1.329	0.467	0.500	1.378	0.273	0.503	1.413	0.481
7737	0.261	-0.137	0.501	0.272	-0.091	0.273	0.050	-1.485	0.512
7738	0.511	1.464	0.513	0.480	1.246	0.235	0.495	1.363	0.508
7739	0.226	-0.356	0.185	0.307	0.135	0.193	0.187	-0.608	0.224
7740	0.497	1.380	0.492	0.509	1.434	0.514	0.463	1.161	0.481
7741	0.489	1.329	0.512	0.401	0.737	0.341	0.505	1.430	0.512
7742	0.263	-0.120	0.481	0.234	-0.336	0.149	0.050	-1.485	0.409
7743	0.187	-0.608	0.512	0.342	0.361	0.491	0.000	?	0.409

7744	0.261	-0.137	0.450	0.152	-0.863	0.491	0.142	-0.895	0.501
7745	0.508	1.447	0.262	0.506	1.415	0.491	0.484	1.295	0.224
7746	0.508	1.447	0.501	0.506	1.415	0.491	0.484	1.295	0.501
7747	0.145	-0.878	0.492	0.234	-0.336	0.501	0.187	-0.608	0.358
7748	0.482	1.279	0.450	0.497	1.359	0.341	0.505	1.430	0.358
7749	0.513	1.481	0.224	0.442	1.001	0.309	0.358	0.487	0.296
7750	0.492	1.346	0.481	0.491	1.321	0.398	0.461	1.144	0.508
7751	0.226	-0.356	0.492	0.272	-0.091	0.462	0.050	-1.485	0.296
7752	0.226	-0.356	0.467	0.272	-0.091	0.422	0.050	-1.485	0.508
7753	0.295	0.082	0.185	0.240	-0.299	0.341	0.000	?	0.262
7754	0.329	0.301	0.492	0.196	-0.581	0.478	0.000	?	0.328
7755	0.434	0.975	0.450	0.512	1.453	0.512	0.500	1.397	0.501
7756	0.263	-0.120	0.328	0.342	0.361	0.102	0.329	0.301	0.512
7757	0.329	0.301	0.296	0.152	-0.863	0.512	0.050	-1.485	0.512
7758	0.487	1.312	0.296	0.491	1.321	0.508	0.463	1.161	0.512
7759	0.513	1.481	0.224	0.497	1.359	0.273	0.513	1.481	0.481
7760	0.513	1.481	0.481	0.497	1.359	0.478	0.513	1.481	0.492
7761	0.511	1.464	0.185	0.497	1.359	0.478	0.513	1.481	0.501
7762	0.413	0.841	0.142	0.506	1.415	0.501	0.479	1.262	0.492
7763	0.142	-0.895	0.224	0.345	0.379	0.422	0.226	-0.356	0.409
7764	0.000	?	0.431	0.307	0.135	0.422	0.226	-0.356	0.481
7765	0.050	-1.485	0.431	0.401	0.737	0.462	0.261	-0.137	0.481
7766	0.508	1.447	0.097	0.459	1.114	0.512	0.461	1.144	0.508
7767	0.479	1.262	0.501	0.421	0.869	0.102	0.505	1.430	0.481
7768	0.261	-0.137	0.467	0.342	0.361	0.512	0.187	-0.608	0.513
7769	0.466	1.178	0.467	0.477	1.227	0.235	0.487	1.312	0.385
7770	0.447	1.060	0.450	0.506	1.415	0.478	0.479	1.262	0.508
7771	0.297	0.099	0.501	0.102	-1.184	0.501	0.142	-0.895	0.512
7772	0.497	1.380	0.508	0.515	1.472	0.309	0.505	1.430	0.501
7773	0.513	1.481	0.513	0.506	1.415	0.491	0.466	1.178	0.185
7774	0.297	0.099	0.501	0.272	-0.091	0.491	0.471	1.211	0.513
7775	0.508	1.447	0.450	0.480	1.246	0.478	0.411	0.824	0.508
7776	0.466	1.178	0.467	0.482	1.265	0.273	0.508	1.447	0.508
7777	0.479	1.262	0.431	0.500	1.378	0.512	0.508	1.447	0.224
7778	0.497	1.380	0.513	0.418	0.850	0.149	0.513	1.481	0.492
7779	0.503	1.413	0.508	0.418	0.850	0.235	0.513	1.481	0.512
7780	0.463	1.161	0.185	0.459	1.114	0.235	0.513	1.481	0.142
7781	0.511	1.464	0.467	0.515	1.472	0.508	0.476	1.245	0.097
7782	0.387	0.672	0.224	0.102	-1.184	0.514	0.142	-0.895	0.097
7783	0.263	-0.120	0.224	0.515	1.472	0.193	0.508	1.447	0.185
7784	0.326	0.285	0.467	0.237	-0.317	0.193	0.261	-0.137	0.512
7785	0.503	1.413	0.467	0.480	1.246	0.193	0.503	1.413	0.142
7786	0.513	1.481	0.450	0.503	1.396	0.273	0.466	1.178	0.142
7787	0.184	-0.625	0.262	0.313	0.172	0.512	0.224	-0.373	0.142
7788	0.476	1.245	0.501	0.494	1.340	0.053	0.518	1.514	0.512
7789	0.497	1.380	0.224	0.497	1.359	0.149	0.466	1.178	0.512
7790	0.492	1.346	0.328	0.477	1.227	0.444	0.487	1.312	0.185
7791	0.226	-0.356	0.296	0.275	-0.073	0.422	0.358	0.487	0.508
7792	0.482	1.279	0.185	0.515	1.472	0.235	0.503	1.413	0.501
7793	0.184	-0.625	0.492	0.149	-0.882	0.501	0.224	-0.373	0.501
7794	0.224	-0.373	0.492	0.234	-0.336	0.512	0.326	0.285	0.513
7795	0.184	-0.625	0.501	0.234	-0.336	0.512	0.142	-0.895	0.492
7796	0.463	1.161	0.501	0.512	1.453	0.491	0.437	0.992	0.492
7797	0.513	1.481	0.481	0.518	1.491	0.478	0.384	0.655	0.467
7798	0.226	-0.356	0.492	0.193	-0.600	0.341	0.263	-0.120	0.385
7799	0.226	-0.356	0.296	0.193	-0.600	0.508	0.263	-0.120	0.481
7800	0.226	-0.356	0.513	0.193	-0.600	0.478	0.263	-0.120	0.481
7801	0.261	-0.137	0.385	0.275	-0.073	0.491	0.184	-0.625	0.481
7802	0.495	1.363	0.512	0.509	1.434	0.235	0.482	1.279	0.508
7803	0.226	-0.356	0.224	0.053	-1.504	0.478	0.329	0.301	0.501
7804	0.226	-0.356	0.224	0.149	-0.882	0.478	0.329	0.301	0.097

7805	0.489	1.329	0.450	0.447	1.039	0.491	0.468	1.194	0.409
7806	0.297	0.099	0.501	0.427	0.907	0.273	0.184	-0.625	0.185
7807	0.297	0.099	0.409	0.234	-0.336	0.444	0.050	-1.485	0.262
7808	0.511	1.464	0.467	0.500	1.378	0.193	0.432	0.958	0.512
7809	0.445	1.043	0.450	0.515	1.472	0.508	0.497	1.380	0.513
7810	0.445	1.043	0.409	0.515	1.472	0.398	0.497	1.380	0.492
7811	0.511	1.464	0.481	0.497	1.359	0.235	0.487	1.312	0.185
7812	0.505	1.430	0.097	0.477	1.227	0.193	0.503	1.413	0.501
7813	0.263	-0.120	0.097	0.345	0.379	0.422	0.097	-1.181	0.224
7814	0.476	1.245	0.385	0.512	1.453	0.235	0.476	1.245	0.296
7815	0.513	1.481	0.409	0.471	1.189	0.491	0.511	1.464	0.185
7816	0.489	1.329	0.467	0.491	1.321	0.273	0.508	1.447	0.185
7817	0.226	-0.356	0.358	0.234	-0.336	0.149	0.224	-0.373	0.492
7818	0.511	1.464	0.328	0.480	1.246	0.501	0.484	1.295	0.492
7819	0.474	1.228	0.097	0.480	1.246	0.491	0.508	1.447	0.512
7820	0.466	1.178	0.142	0.497	1.359	0.478	0.413	0.841	0.512
7821	0.142	-0.895	0.501	0.272	-0.091	0.478	0.411	0.824	0.501
7822	0.450	1.076	0.224	0.444	1.020	0.508	0.503	1.413	0.512
7823	0.332	0.318	0.508	0.196	-0.581	0.478	0.224	-0.373	0.224
7824	0.505	1.430	0.501	0.506	1.415	0.462	0.500	1.397	0.513
7825	0.224	-0.373	0.185	0.401	0.737	0.512	0.358	0.487	0.385
7826	0.261	-0.137	0.185	0.234	-0.336	0.053	0.358	0.487	0.508
7827	0.224	-0.373	0.467	0.193	-0.600	0.501	0.297	0.099	0.185
7828	0.511	1.464	0.512	0.424	0.888	0.491	0.505	1.430	0.185
7829	0.326	0.285	0.262	0.234	-0.336	0.501	0.295	0.082	0.492
7830	0.505	1.430	0.185	0.491	1.321	0.501	0.505	1.430	0.492
7831	0.263	-0.120	0.358	0.275	-0.073	0.491	0.263	-0.120	0.508
7832	0.100	-1.164	0.467	0.149	-0.882	0.371	0.329	0.301	0.512
7833	0.508	1.447	0.467	0.503	1.396	0.501	0.497	1.380	0.512
7834	0.505	1.430	0.512	0.494	1.340	0.491	0.508	1.447	0.262
7835	0.513	1.481	0.512	0.482	1.265	0.508	0.511	1.464	0.409
7836	0.492	1.346	0.224	0.482	1.265	0.309	0.511	1.464	0.409
7837	0.511	1.464	0.328	0.506	1.415	0.462	0.484	1.295	0.409
7838	0.463	1.161	0.262	0.474	1.208	0.512	0.497	1.380	0.262
7839	0.497	1.380	0.262	0.459	1.114	0.512	0.508	1.447	0.358
7840	0.518	1.514	0.467	0.512	1.453	0.149	0.484	1.295	0.492
7841	0.184	-0.625	0.467	0.053	-1.504	0.398	0.413	0.841	0.185
7842	0.497	1.380	0.467	0.497	1.359	0.462	0.482	1.279	0.262
7843	0.513	1.481	0.467	0.494	1.340	0.309	0.479	1.262	0.358
7844	0.479	1.262	0.142	0.500	1.378	0.235	0.505	1.430	0.097
7845	0.508	1.447	0.142	0.497	1.359	0.235	0.482	1.279	0.508
7846	0.447	1.060	0.467	0.491	1.321	0.193	0.511	1.464	0.328
7847	0.263	-0.120	0.467	0.377	0.587	0.508	0.384	0.655	0.481
7848	0.503	1.413	0.262	0.503	1.396	0.398	0.505	1.430	0.467
7849	0.487	1.312	0.224	0.494	1.340	0.273	0.516	1.498	0.185
7850	0.450	1.076	0.512	0.512	1.453	0.193	0.508	1.447	0.262
7851	0.224	-0.373	0.512	0.310	0.153	0.491	0.187	-0.608	0.492
7852	0.479	1.262	0.508	0.465	1.152	0.491	0.511	1.464	0.481
7853	0.492	1.346	0.224	0.515	1.472	0.273	0.508	1.447	0.050
7854	0.466	1.178	0.501	0.515	1.472	0.501	0.508	1.447	0.358
7855	0.187	-0.608	0.481	0.149	-0.882	0.371	0.226	-0.356	0.508
7856	0.495	1.363	0.450	0.401	0.737	0.398	0.484	1.295	0.508
7857	0.453	1.093	0.262	0.462	1.133	0.398	0.508	1.447	0.512
7858	0.326	0.285	0.358	0.313	0.172	0.501	0.142	-0.895	0.512
7859	0.297	0.099	0.409	0.234	-0.336	0.501	0.097	-1.181	0.097
7860	0.297	0.099	0.224	0.234	-0.336	0.514	0.097	-1.181	0.185
7861	0.224	-0.373	0.385	0.193	-0.600	0.514	0.184	-0.625	0.328
7862	0.476	1.245	0.142	0.506	1.415	0.491	0.511	1.464	0.328
7863	0.184	-0.625	0.142	0.401	0.737	0.514	0.142	-0.895	0.501
7864	0.145	-0.878	0.142	0.275	-0.073	0.491	0.142	-0.895	0.501
7865	0.224	-0.373	0.431	0.196	-0.581	0.491	0.142	-0.895	0.501

7866	0.434	0.975	0.097	0.491	1.321	0.508	0.511	1.464	0.512
7867	0.434	0.975	0.224	0.491	1.321	0.341	0.511	1.464	0.185
7868	0.097	-1.181	0.385	0.278	-0.054	0.478	0.184	-0.625	0.185
7869	0.500	1.397	0.296	0.497	1.359	0.193	0.508	1.447	0.467
7870	0.466	1.178	0.492	0.368	0.530	0.193	0.497	1.380	0.467
7871	0.466	1.178	0.097	0.395	0.700	0.341	0.497	1.380	0.185
7872	0.447	1.060	0.185	0.404	0.756	0.398	0.513	1.481	0.296
7873	0.505	1.430	0.513	0.497	1.359	0.512	0.484	1.295	0.501
7874	0.513	1.481	0.492	0.497	1.359	0.102	0.484	1.295	0.513
7875	0.503	1.413	0.296	0.518	1.491	0.508	0.466	1.178	0.385
7876	0.492	1.346	0.097	0.518	1.491	0.102	0.384	0.655	0.142
7877	0.447	1.060	0.296	0.488	1.302	0.102	0.479	1.262	0.508
7878	0.466	1.178	0.409	0.512	1.453	0.235	0.479	1.262	0.512
7879	0.429	0.942	0.262	0.491	1.321	0.193	0.479	1.262	0.492
7880	0.503	1.413	0.481	0.494	1.340	0.371	0.505	1.430	0.501
7881	0.497	1.380	0.492	0.512	1.453	0.371	0.500	1.397	0.050
7882	0.187	-0.608	0.492	0.342	0.361	0.444	0.097	-1.181	0.296
7883	0.463	1.161	0.224	0.480	1.246	0.444	0.413	0.841	0.142
7884	0.224	-0.373	0.492	0.193	-0.600	0.444	0.184	-0.625	0.142
7885	0.226	-0.356	0.224	0.193	-0.600	0.478	0.261	-0.137	0.328
7886	0.466	1.178	0.450	0.342	0.361	0.193	0.511	1.464	0.296
7887	0.463	1.161	0.185	0.401	0.737	0.444	0.508	1.447	0.296
7888	0.450	1.076	0.224	0.509	1.434	0.508	0.500	1.397	0.467
7889	0.263	-0.120	0.431	0.102	-1.184	0.398	0.184	-0.625	0.328
7890	0.497	1.380	0.224	0.509	1.434	0.341	0.500	1.397	0.142
7891	0.224	-0.373	0.224	0.102	-1.184	0.273	0.226	-0.356	0.467
7892	0.326	0.285	0.492	0.102	-1.184	0.501	0.300	0.116	0.097
7893	0.297	0.099	0.481	0.237	-0.317	0.501	0.187	-0.608	0.358
7894	0.184	-0.625	0.385	0.196	-0.581	0.508	0.187	-0.608	0.296
7895	0.487	1.312	0.097	0.371	0.549	0.512	0.492	1.346	0.185
7896	0.487	1.312	0.501	0.371	0.549	0.512	0.492	1.346	0.450
7897	0.497	1.380	0.224	0.439	0.982	0.341	0.513	1.481	0.512
7898	0.497	1.380	0.296	0.439	0.982	0.491	0.513	1.481	0.262
7899	0.482	1.279	0.185	0.442	1.001	0.235	0.497	1.380	0.409
7900	0.495	1.363	0.508	0.477	1.227	0.235	0.508	1.447	0.262
7901	0.297	0.099	0.262	0.193	-0.600	0.341	0.226	-0.356	0.050
7902	0.511	1.464	0.508	0.447	1.039	0.371	0.516	1.498	0.097
7903	0.384	0.655	0.385	0.509	1.434	0.422	0.387	0.672	0.492
7904	0.503	1.413	0.513	0.398	0.718	0.273	0.511	1.464	0.481
7905	0.224	-0.373	0.450	0.342	0.361	0.149	0.184	-0.625	0.501
7906	0.224	-0.373	0.467	0.275	-0.073	0.149	0.184	-0.625	0.262
7907	0.453	1.093	0.467	0.500	1.378	0.371	0.487	1.312	0.431
7908	0.505	1.430	0.501	0.500	1.378	0.508	0.487	1.312	0.142
7909	0.408	0.807	0.467	0.509	1.434	0.102	0.503	1.413	0.492
7910	0.468	1.194	0.481	0.512	1.453	0.462	0.503	1.413	0.262
7911	0.450	1.076	0.262	0.512	1.453	0.491	0.503	1.413	0.142
7912	0.411	0.824	0.450	0.339	0.342	0.193	0.261	-0.137	0.492
7913	0.479	1.262	0.501	0.491	1.321	0.149	0.408	0.807	0.142
7914	0.097	-1.181	0.385	0.237	-0.317	0.478	0.411	0.824	0.296
7915	0.097	-1.181	0.450	0.237	-0.317	0.512	0.411	0.824	0.512
7916	0.384	0.655	0.467	0.342	0.361	0.273	0.261	-0.137	0.431
7917	0.408	0.807	0.224	0.371	0.549	0.273	0.358	0.487	0.512
7918	0.471	1.211	0.185	0.424	0.888	0.309	0.492	1.346	0.328
7919	0.361	0.504	0.512	0.275	-0.073	0.514	0.184	-0.625	0.508
7920	0.332	0.318	0.358	0.149	-0.882	0.514	0.263	-0.120	0.097
7921	0.097	-1.181	0.508	0.149	-0.882	0.491	0.355	0.470	0.097
7922	0.142	-0.895	0.450	0.374	0.568	0.491	0.097	-1.181	0.142
7923	0.500	1.397	0.501	0.509	1.434	0.235	0.508	1.447	0.450
7924	0.224	-0.373	0.512	0.102	-1.184	0.309	0.329	0.301	0.142
7925	0.508	1.447	0.097	0.456	1.095	0.193	0.476	1.245	0.467
7926	0.497	1.380	0.097	0.488	1.302	0.193	0.463	1.161	0.050

7927	0.187	-0.608	0.224	0.196	-0.581	0.514	0.184	-0.625	0.492
7928	0.184	-0.625	0.481	0.149	-0.882	0.514	0.261	-0.137	0.492
7929	0.468	1.194	0.409	0.474	1.208	0.462	0.487	1.312	0.492
7930	0.513	1.481	0.508	0.509	1.434	0.491	0.476	1.245	0.431
7931	0.263	-0.120	0.492	0.275	-0.073	0.235	0.050	-1.485	0.512
7932	0.184	-0.625	0.481	0.275	-0.073	0.273	0.358	0.487	0.467
7933	0.361	0.504	0.492	0.310	0.153	0.501	0.000	?	0.481
7934	0.463	1.161	0.185	0.518	1.491	0.501	0.503	1.413	0.142
7935	0.463	1.161	0.224	0.518	1.491	0.235	0.503	1.413	0.512
7936	0.503	1.413	0.142	0.494	1.340	0.102	0.508	1.447	0.467
7937	0.503	1.413	0.512	0.494	1.340	0.501	0.508	1.447	0.492
7938	0.224	-0.373	0.409	0.234	-0.336	0.444	0.097	-1.181	0.492
7939	0.332	0.318	0.097	0.310	0.153	0.512	0.184	-0.625	0.512
7940	0.263	-0.120	0.492	0.193	-0.600	0.193	0.326	0.285	0.296
7941	0.263	-0.120	0.296	0.193	-0.600	0.491	0.326	0.285	0.262
7942	0.463	1.161	0.508	0.518	1.491	0.512	0.505	1.430	0.481
7943	0.463	1.161	0.224	0.518	1.491	0.371	0.505	1.430	0.050
7944	0.468	1.194	0.385	0.459	1.114	0.508	0.497	1.380	0.508
7945	0.466	1.178	0.501	0.488	1.302	0.149	0.505	1.430	0.501
7946	0.142	-0.895	0.481	0.234	-0.336	0.273	0.184	-0.625	0.409
7947	0.142	-0.895	0.296	0.275	-0.073	0.273	0.184	-0.625	0.450
7948	0.466	1.178	0.262	0.503	1.396	0.309	0.471	1.211	0.224
7949	0.466	1.178	0.481	0.503	1.396	0.273	0.471	1.211	0.224
7950	0.263	-0.120	0.328	0.237	-0.317	0.341	0.184	-0.625	0.262
7951	0.224	-0.373	0.481	0.102	-1.184	0.341	0.295	0.082	0.481
7952	0.508	1.447	0.296	0.500	1.378	0.514	0.497	1.380	0.492
7953	0.511	1.464	0.481	0.447	1.039	0.273	0.508	1.447	0.501
7954	0.505	1.430	0.508	0.515	1.472	0.193	0.382	0.638	0.492
7955	0.226	-0.356	0.501	0.193	-0.600	0.491	0.142	-0.895	0.508
7956	0.497	1.380	0.262	0.497	1.359	0.149	0.511	1.464	0.508
7957	0.476	1.245	0.296	0.520	1.509	0.512	0.516	1.498	0.262
7958	0.455	1.110	0.296	0.371	0.549	0.341	0.492	1.346	0.385
7959	0.261	-0.137	0.224	0.509	1.434	0.235	0.503	1.413	0.296
7960	0.358	0.487	0.262	0.149	-0.882	0.514	0.050	-1.485	0.450
7961	0.416	0.857	0.508	0.272	-0.091	0.501	0.295	0.082	0.492
7962	0.226	-0.356	0.481	0.272	-0.091	0.273	0.142	-0.895	0.185
7963	0.387	0.672	0.224	0.310	0.153	0.053	0.142	-0.895	0.409
7964	0.145	-0.878	0.328	0.275	-0.073	0.371	0.329	0.301	0.328
7965	0.142	-0.895	0.385	0.345	0.379	0.273	0.295	0.082	0.481
7966	0.142	-0.895	0.512	0.345	0.379	0.235	0.295	0.082	0.050
7967	0.429	0.942	0.501	0.515	1.472	0.478	0.466	1.178	0.501
7968	0.097	-1.181	0.097	0.275	-0.073	0.512	0.329	0.301	0.492
7969	0.224	-0.373	0.508	0.193	-0.600	0.491	0.142	-0.895	0.512
7970	0.387	0.672	0.492	0.488	1.302	0.398	0.461	1.144	0.185
7971	0.295	0.082	0.409	0.149	-0.882	0.501	0.097	-1.181	0.097
7972	0.495	1.363	0.262	0.509	1.434	0.235	0.358	0.487	0.450
7973	0.097	-1.181	0.512	0.345	0.379	0.508	0.297	0.099	0.513
7974	0.184	-0.625	0.224	0.234	-0.336	0.149	0.184	-0.625	0.508
7975	0.508	1.447	0.512	0.515	1.472	0.398	0.450	1.076	0.050
7976	0.492	1.346	0.512	0.506	1.415	0.478	0.511	1.464	0.450
7977	0.295	0.082	0.508	0.275	-0.073	0.193	0.261	-0.137	0.492
7978	0.097	-1.181	0.142	0.053	-1.504	0.053	0.411	0.824	0.467
7979	0.297	0.099	0.097	0.371	0.549	0.491	0.263	-0.120	0.142
7980	0.411	0.824	0.097	0.275	-0.073	0.512	0.050	-1.485	0.224
7981	0.263	-0.120	0.050	0.237	-0.317	0.462	0.097	-1.181	0.142
7982	0.482	1.279	0.224	0.480	1.246	0.102	0.495	1.363	0.224
7983	0.492	1.346	0.328	0.509	1.434	0.444	0.479	1.262	0.262
7984	0.495	1.363	0.409	0.485	1.283	0.235	0.500	1.397	0.501
7985	0.224	-0.373	0.224	0.401	0.737	0.149	0.263	-0.120	0.385
7986	0.495	1.363	0.358	0.500	1.378	0.309	0.429	0.942	0.097
7987	0.224	-0.373	0.492	0.234	-0.336	0.512	0.142	-0.895	0.501

7988	0.447	1.060	0.481	0.506	1.415	0.309	0.487	1.312	0.050
7989	0.184	-0.625	0.492	0.149	-0.882	0.444	0.263	-0.120	0.492
7990	0.226	-0.356	0.467	0.401	0.737	0.273	0.142	-0.895	0.467
7991	0.432	0.958	0.481	0.480	1.246	0.478	0.492	1.346	0.296
7992	0.226	-0.356	0.328	0.193	-0.600	0.512	0.142	-0.895	0.467
7993	0.224	-0.373	0.481	0.053	-1.504	0.514	0.297	0.099	0.481
7994	0.489	1.329	0.224	0.488	1.302	0.512	0.508	1.447	0.508
7995	0.482	1.279	0.512	0.515	1.472	0.478	0.432	0.958	0.142
7996	0.387	0.672	0.050	0.462	1.133	0.422	0.516	1.498	0.508
7997	0.097	-1.181	0.508	0.102	-1.184	0.491	0.326	0.285	0.097
7998	0.500	1.397	0.513	0.444	1.020	0.273	0.500	1.397	0.512
7999	0.224	-0.373	0.513	0.237	-0.317	0.501	0.097	-1.181	0.501
8000	0.295	0.082	0.513	0.149	-0.882	0.462	0.097	-1.181	0.501
8001	0.184	-0.625	0.224	0.307	0.135	0.501	0.142	-0.895	0.409
8002	0.508	1.447	0.262	0.512	1.453	0.478	0.447	1.060	0.262
8003	0.261	-0.137	0.262	0.310	0.153	0.491	0.142	-0.895	0.262
8004	0.508	1.447	0.481	0.442	1.001	0.193	0.461	1.144	0.431
8005	0.384	0.655	0.328	0.278	-0.054	0.309	0.050	-1.485	0.050
8006	0.513	1.481	0.450	0.477	1.227	0.371	0.489	1.329	0.097
8007	0.453	1.093	0.450	0.509	1.434	0.102	0.487	1.312	0.224
8008	0.471	1.211	0.224	0.512	1.453	0.478	0.489	1.329	0.224
8009	0.466	1.178	0.262	0.506	1.415	0.491	0.434	0.975	0.508
8010	0.503	1.413	0.467	0.474	1.208	0.478	0.511	1.464	0.481
8011	0.468	1.194	0.492	0.421	0.869	0.501	0.461	1.144	0.508
8012	0.482	1.279	0.508	0.488	1.302	0.273	0.476	1.245	0.431
8013	0.261	-0.137	0.481	0.272	-0.091	0.273	0.142	-0.895	0.467
8014	0.450	1.076	0.262	0.497	1.359	0.422	0.511	1.464	0.385
8015	0.500	1.397	0.224	0.462	1.133	0.422	0.463	1.161	0.262
8016	0.384	0.655	0.224	0.497	1.359	0.444	0.489	1.329	0.467
8017	0.453	1.093	0.296	0.477	1.227	0.501	0.489	1.329	0.142
8018	0.471	1.211	0.481	0.491	1.321	0.501	0.508	1.447	0.508
8019	0.224	-0.373	0.224	0.193	-0.600	0.478	0.295	0.082	0.262
8020	0.184	-0.625	0.450	0.307	0.135	0.478	0.263	-0.120	0.467
8021	0.511	1.464	0.450	0.368	0.530	0.149	0.476	1.245	0.467
8022	0.361	0.504	0.328	0.102	-1.184	0.149	0.050	-1.485	0.467
8023	0.511	1.464	0.512	0.477	1.227	0.149	0.505	1.430	0.467
8024	0.450	1.076	0.501	0.485	1.283	0.444	0.497	1.380	0.097
8025	0.500	1.397	0.296	0.474	1.208	0.491	0.408	0.807	0.142
8026	0.508	1.447	0.501	0.500	1.378	0.273	0.447	1.060	0.142
8027	0.097	-1.181	0.481	0.275	-0.073	0.478	0.226	-0.356	0.467
8028	0.097	-1.181	0.513	0.275	-0.073	0.193	0.226	-0.356	0.262
8029	0.224	-0.373	0.481	0.424	0.888	0.462	0.261	-0.137	0.481
8030	0.482	1.279	0.097	0.421	0.869	0.309	0.476	1.245	0.501
8031	0.411	0.824	0.262	0.442	1.001	0.491	0.495	1.363	0.142
8032	0.505	1.430	0.224	0.500	1.378	0.512	0.500	1.397	0.142
8033	0.489	1.329	0.142	0.500	1.378	0.508	0.489	1.329	0.508
8034	0.474	1.228	0.142	0.477	1.227	0.273	0.508	1.447	0.450
8035	0.487	1.312	0.501	0.477	1.227	0.309	0.508	1.447	0.385
8036	0.184	-0.625	0.185	0.149	-0.882	0.478	0.261	-0.137	0.508
8037	0.226	-0.356	0.431	0.149	-0.882	0.501	0.387	0.672	0.142
8038	0.142	-0.895	0.492	0.149	-0.882	0.491	0.295	0.082	0.097
8039	0.503	1.413	0.296	0.444	1.020	0.273	0.450	1.076	0.358
8040	0.405	0.790	0.097	0.491	1.321	0.444	0.489	1.329	0.358
8041	0.097	-1.181	0.097	0.278	-0.054	0.444	0.184	-0.625	0.512
8042	0.487	1.312	0.512	0.477	1.227	0.444	0.408	0.807	0.296
8043	0.295	0.082	0.097	0.193	-0.600	0.235	0.326	0.285	0.512
8044	0.511	1.464	0.262	0.462	1.133	0.273	0.474	1.228	0.512
8045	0.224	-0.373	0.481	0.310	0.153	0.462	0.050	-1.485	0.142
8046	0.387	0.672	0.467	0.491	1.321	0.149	0.497	1.380	0.481
8047	0.505	1.430	0.492	0.506	1.415	0.149	0.489	1.329	0.481
8048	0.484	1.295	0.481	0.503	1.396	0.422	0.505	1.430	0.224

8049	0.295	0.082	0.512	0.272	-0.091	0.478	0.187	-0.608	0.481
8050	0.263	-0.120	0.512	0.307	0.135	0.235	0.097	-1.181	0.450
8051	0.479	1.262	0.450	0.480	1.246	0.491	0.447	1.060	0.467
8052	0.326	0.285	0.513	0.506	1.415	0.235	0.513	1.481	0.501
8053	0.479	1.262	0.450	0.491	1.321	0.444	0.511	1.464	0.262
8054	0.295	0.082	0.492	0.272	-0.091	0.508	0.050	-1.485	0.097
8055	0.482	1.279	0.431	0.442	1.001	0.309	0.450	1.076	0.142
8056	0.505	1.430	0.142	0.442	1.001	0.462	0.492	1.346	0.296
8057	0.497	1.380	0.358	0.442	1.001	0.512	0.466	1.178	0.296
8058	0.261	-0.137	0.262	0.234	-0.336	0.491	0.142	-0.895	0.450
8059	0.297	0.099	0.492	0.272	-0.091	0.235	0.224	-0.373	0.185
8060	0.297	0.099	0.262	0.462	1.133	0.478	0.142	-0.895	0.501
8061	0.226	-0.356	0.050	0.149	-0.882	0.273	0.224	-0.373	0.508
8062	0.263	-0.120	0.050	0.152	-0.863	0.478	0.261	-0.137	0.142
8063	0.505	1.430	0.513	0.421	0.869	0.462	0.500	1.397	0.328
8064	0.476	1.245	0.450	0.477	1.227	0.501	0.384	0.655	0.328
8065	0.224	-0.373	0.492	0.237	-0.317	0.273	0.097	-1.181	0.467
8066	0.326	0.285	0.097	0.494	1.340	0.462	0.500	1.397	0.358
8067	0.387	0.672	0.492	0.237	-0.317	0.462	0.050	-1.485	0.050
8068	0.516	1.498	0.481	0.444	1.020	0.235	0.484	1.295	0.513
8069	0.500	1.397	0.492	0.506	1.415	0.371	0.463	1.161	0.513
8070	0.097	-1.181	0.142	0.307	0.135	0.149	0.300	0.116	0.431
8071	0.508	1.447	0.501	0.459	1.114	0.273	0.463	1.161	0.501
8072	0.492	1.346	0.050	0.509	1.434	0.235	0.476	1.245	0.492
8073	0.411	0.824	0.501	0.488	1.302	0.462	0.500	1.397	0.513
8074	0.261	-0.137	0.050	0.240	-0.299	0.398	0.142	-0.895	0.501
8075	0.511	1.464	0.409	0.480	1.246	0.478	0.505	1.430	0.450
8076	0.226	-0.356	0.409	0.272	-0.091	0.491	0.097	-1.181	0.492
8077	0.511	1.464	0.492	0.480	1.246	0.508	0.511	1.464	0.508
8078	0.513	1.481	0.508	0.459	1.114	0.478	0.495	1.363	0.501
8079	0.511	1.464	0.224	0.494	1.340	0.273	0.495	1.363	0.050
8080	0.142	-0.895	0.185	0.275	-0.073	0.512	0.405	0.790	0.050
8081	0.097	-1.181	0.501	0.462	1.133	0.193	0.261	-0.137	0.185
8082	0.097	-1.181	0.501	0.462	1.133	0.501	0.261	-0.137	0.450
8083	0.050	-1.485	0.501	0.237	-0.317	0.235	0.432	0.958	0.328
8084	0.224	-0.373	0.492	0.371	0.549	0.514	0.050	-1.485	0.185
8085	0.329	0.301	0.296	0.152	-0.863	0.508	0.097	-1.181	0.142
8086	0.411	0.824	0.097	0.275	-0.073	0.501	0.224	-0.373	0.492
8087	0.224	-0.373	0.508	0.234	-0.336	0.508	0.226	-0.356	0.512
8088	0.358	0.487	0.481	0.462	1.133	0.235	0.508	1.447	0.492
8089	0.487	1.312	0.508	0.398	0.718	0.193	0.479	1.262	0.142
8090	0.476	1.245	0.492	0.474	1.208	0.235	0.508	1.447	0.512
8091	0.495	1.363	0.097	0.485	1.283	0.491	0.426	0.925	0.409
8092	0.466	1.178	0.097	0.506	1.415	0.235	0.463	1.161	0.501
8093	0.476	1.245	0.185	0.485	1.283	0.491	0.384	0.655	0.185
8094	0.326	0.285	0.431	0.275	-0.073	0.508	0.261	-0.137	0.385
8095	0.484	1.295	0.481	0.512	1.453	0.193	0.463	1.161	0.262
8096	0.226	-0.356	0.358	0.196	-0.581	0.149	0.142	-0.895	0.513
8097	0.511	1.464	0.358	0.503	1.396	0.508	0.513	1.481	0.050
8098	0.050	-1.485	0.492	0.237	-0.317	0.501	0.261	-0.137	0.492
8099	0.497	1.380	0.512	0.515	1.472	0.501	0.468	1.194	0.492
8100	0.508	1.447	0.513	0.506	1.415	0.501	0.466	1.178	0.492
8101	0.508	1.447	0.501	0.500	1.378	0.193	0.466	1.178	0.501
8102	0.508	1.447	0.050	0.506	1.415	0.273	0.466	1.178	0.385
8103	0.226	-0.356	0.050	0.234	-0.336	0.341	0.097	-1.181	0.097
8104	0.261	-0.137	0.050	0.193	-0.600	0.235	0.142	-0.895	0.512
8105	0.261	-0.137	0.492	0.237	-0.317	0.478	0.142	-0.895	0.481
8106	0.474	1.228	0.492	0.491	1.321	0.053	0.466	1.178	0.481
8107	0.329	0.301	0.512	0.234	-0.336	0.371	0.261	-0.137	0.481
8108	0.450	1.076	0.501	0.491	1.321	0.371	0.476	1.245	0.409
8109	0.445	1.043	0.501	0.506	1.415	0.309	0.497	1.380	0.409

8110	0.224	−0.373	0.431	0.193	−0.600	0.514	0.142	−0.895	0.467
8111	0.261	−0.137	0.431	0.149	−0.882	0.508	0.142	−0.895	0.508
8112	0.466	1.178	0.296	0.503	1.396	0.149	0.511	1.464	0.512
8113	0.492	1.346	0.296	0.503	1.396	0.512	0.453	1.093	0.492
8114	0.508	1.447	0.224	0.500	1.378	0.478	0.384	0.655	0.142
8115	0.482	1.279	0.508	0.503	1.396	0.501	0.500	1.397	0.185
8116	0.266	−0.103	0.409	0.193	−0.600	0.501	0.142	−0.895	0.142
8117	0.224	−0.373	0.512	0.272	−0.091	0.309	0.097	−1.181	0.467
8118	0.224	−0.373	0.450	0.345	0.379	0.341	0.361	0.504	0.512
8119	0.297	0.099	0.467	0.237	−0.317	0.273	0.361	0.504	0.185
8120	0.482	1.279	0.481	0.482	1.265	0.102	0.511	1.464	0.185
8121	0.224	−0.373	0.481	0.053	−1.504	0.102	0.295	0.082	0.512
8122	0.453	1.093	0.492	0.374	0.568	0.512	0.511	1.464	0.512
8123	0.453	1.093	0.492	0.374	0.568	0.193	0.511	1.464	0.492
8124	0.329	0.301	0.431	0.307	0.135	0.501	0.142	−0.895	0.431
8125	0.503	1.413	0.501	0.512	1.453	0.501	0.482	1.279	0.185
8126	0.492	1.346	0.097	0.506	1.415	0.193	0.482	1.279	0.185
8127	0.297	0.099	0.513	0.149	−0.882	0.422	0.226	−0.356	0.185
8128	0.503	1.413	0.224	0.512	1.453	0.422	0.487	1.312	0.501
8129	0.484	1.295	0.481	0.477	1.227	0.444	0.445	1.043	0.508
8130	0.503	1.413	0.097	0.503	1.396	0.398	0.466	1.178	0.492
8131	0.487	1.312	0.508	0.497	1.359	0.341	0.495	1.363	0.501
8132	0.097	−1.181	0.508	0.310	0.153	0.501	0.261	−0.137	0.501
8133	0.263	−0.120	0.512	0.345	0.379	0.422	0.097	−1.181	0.512
8134	0.224	−0.373	0.508	0.275	−0.073	0.478	0.142	−0.895	0.513
8135	0.142	−0.895	0.513	0.105	−1.165	0.501	0.297	0.099	0.358
8136	0.142	−0.895	0.508	0.105	−1.165	0.491	0.297	0.099	0.185
8137	0.503	1.413	0.296	0.506	1.415	0.491	0.445	1.043	0.097
8138	0.184	−0.625	0.508	0.193	−0.600	0.341	0.184	−0.625	0.450
8139	0.432	0.958	0.185	0.503	1.396	0.512	0.505	1.430	0.481
8140	0.489	1.329	0.142	0.500	1.378	0.478	0.505	1.430	0.409
8141	0.295	0.082	0.512	0.193	−0.600	0.462	0.145	−0.878	0.492
8142	0.097	−1.181	0.142	0.424	0.888	0.501	0.332	0.318	0.409
8143	0.097	−1.181	0.513	0.424	0.888	0.371	0.332	0.318	0.492
8144	0.503	1.413	0.501	0.444	1.020	0.235	0.466	1.178	0.512
8145	0.097	−1.181	0.508	0.401	0.737	0.149	0.361	0.504	0.508
8146	0.261	−0.137	0.513	0.342	0.361	0.478	0.050	−1.485	0.492
8147	0.479	1.262	0.358	0.506	1.415	0.235	0.513	1.481	0.513
8148	0.463	1.161	0.358	0.421	0.869	0.341	0.511	1.464	0.450
8149	0.492	1.346	0.492	0.480	1.246	0.371	0.429	0.942	0.097
8150	0.476	1.245	0.501	0.500	1.378	0.508	0.503	1.413	0.492
8151	0.513	1.481	0.512	0.488	1.302	0.501	0.489	1.329	0.296
8152	0.513	1.481	0.097	0.491	1.321	0.512	0.508	1.447	0.431
8153	0.453	1.093	0.508	0.345	0.379	0.309	0.500	1.397	0.224
8154	0.513	1.481	0.501	0.515	1.472	0.501	0.453	1.093	0.467
8155	0.450	1.076	0.501	0.480	1.246	0.491	0.495	1.363	0.467
8156	0.489	1.329	0.501	0.465	1.152	0.508	0.511	1.464	0.512
8157	0.432	0.958	0.508	0.500	1.378	0.235	0.492	1.346	0.450
8158	0.142	−0.895	0.508	0.371	0.549	0.508	0.050	−1.485	0.450
8159	0.358	0.487	0.142	0.237	−0.317	0.371	0.050	−1.485	0.501
8160	0.261	−0.137	0.467	0.149	−0.882	0.491	0.184	−0.625	0.224
8161	0.497	1.380	0.142	0.477	1.227	0.398	0.450	1.076	0.481
8162	0.263	−0.120	0.501	0.234	−0.336	0.491	0.329	0.301	0.097
8163	0.050	−1.485	0.450	0.342	0.361	0.491	0.184	−0.625	0.097
8164	0.050	−1.485	0.450	0.371	0.549	0.491	0.142	−0.895	0.512
8165	0.508	1.447	0.262	0.506	1.415	0.508	0.489	1.329	0.142
8166	0.447	1.060	0.512	0.500	1.378	0.273	0.505	1.430	0.481
8167	0.492	1.346	0.508	0.512	1.453	0.309	0.492	1.346	0.431
8168	0.097	−1.181	0.224	0.307	0.135	0.478	0.142	−0.895	0.450
8169	0.484	1.295	0.492	0.503	1.396	0.512	0.508	1.447	0.450
8170	0.474	1.228	0.224	0.485	1.283	0.514	0.411	0.824	0.296

8171	0.487	1.312	0.508	0.506	1.415	0.501	0.500	1.397	0.296
8172	0.142	-0.895	0.296	0.234	-0.336	0.149	0.184	-0.625	0.450
8173	0.492	1.346	0.508	0.512	1.453	0.235	0.384	0.655	0.450
8174	0.050	-1.485	0.508	0.374	0.568	0.491	0.263	-0.120	0.501
8175	0.495	1.363	0.508	0.491	1.321	0.512	0.511	1.464	0.224
8176	0.050	-1.485	0.142	0.401	0.737	0.514	0.050	-1.485	0.467
8177	0.411	0.824	0.296	0.488	1.302	0.444	0.492	1.346	0.481
8178	0.411	0.824	0.224	0.485	1.283	0.341	0.492	1.346	0.501
8179	0.484	1.295	0.513	0.488	1.302	0.273	0.495	1.363	0.501
8180	0.500	1.397	0.512	0.512	1.453	0.309	0.500	1.397	0.512
8181	0.224	-0.373	0.512	0.275	-0.073	0.508	0.389	0.689	0.512
8182	0.187	-0.608	0.185	0.310	0.153	0.422	0.097	-1.181	0.224
8183	0.500	1.397	0.142	0.477	1.227	0.309	0.508	1.447	0.513
8184	0.497	1.380	0.142	0.509	1.434	0.309	0.482	1.279	0.262
8185	0.497	1.380	0.097	0.512	1.453	0.508	0.482	1.279	0.481
8186	0.487	1.312	0.328	0.500	1.378	0.478	0.482	1.279	0.513
8187	0.295	0.082	0.262	0.149	-0.882	0.512	0.408	0.807	0.508
8188	0.097	-1.181	0.481	0.237	-0.317	0.462	0.411	0.824	0.224
8189	0.505	1.430	0.508	0.494	1.340	0.398	0.466	1.178	0.409
8190	0.476	1.245	0.501	0.518	1.491	0.398	0.500	1.397	0.481
8191	0.508	1.447	0.097	0.518	1.491	0.398	0.508	1.447	0.224
8192	0.492	1.346	0.262	0.447	1.039	0.508	0.489	1.329	0.385
8193	0.097	-1.181	0.224	0.342	0.361	0.508	0.142	-0.895	0.296
8194	0.097	-1.181	0.262	0.275	-0.073	0.514	0.184	-0.625	0.450
8195	0.184	-0.625	0.296	0.310	0.153	0.512	0.142	-0.895	0.142
8196	0.432	0.958	0.501	0.506	1.415	0.512	0.463	1.161	0.467
8197	0.479	1.262	0.431	0.421	0.869	0.508	0.505	1.430	0.513
8198	0.358	0.487	0.512	0.310	0.153	0.501	0.184	-0.625	0.467
8199	0.358	0.487	0.508	0.310	0.153	0.102	0.184	-0.625	0.097
8200	0.489	1.329	0.508	0.509	1.434	0.341	0.516	1.498	0.050
8201	0.513	1.481	0.508	0.482	1.265	0.273	0.516	1.498	0.224
8202	0.511	1.464	0.513	0.512	1.453	0.514	0.487	1.312	0.492
8203	0.500	1.397	0.328	0.462	1.133	0.491	0.429	0.942	0.501
8204	0.050	-1.485	0.481	0.401	0.737	0.514	0.184	-0.625	0.450
8205	0.050	-1.485	0.450	0.401	0.737	0.501	0.184	-0.625	0.262
8206	0.050	-1.485	0.409	0.401	0.737	0.512	0.184	-0.625	0.262
8207	0.497	1.380	0.512	0.509	1.434	0.512	0.492	1.346	0.142
8208	0.489	1.329	0.481	0.506	1.415	0.508	0.508	1.447	0.262
8209	0.505	1.430	0.185	0.512	1.453	0.508	0.492	1.346	0.097
8210	0.503	1.413	0.512	0.509	1.434	0.478	0.500	1.397	0.224
8211	0.503	1.413	0.262	0.509	1.434	0.462	0.500	1.397	0.508
8212	0.432	0.958	0.450	0.509	1.434	0.514	0.513	1.481	0.492
8213	0.432	0.958	0.296	0.503	1.396	0.341	0.513	1.481	0.328
8214	0.300	0.116	0.492	0.102	-1.184	0.501	0.355	0.470	0.262
8215	0.297	0.099	0.224	0.339	0.342	0.193	0.184	-0.625	0.185
8216	0.224	-0.373	0.481	0.272	-0.091	0.512	0.097	-1.181	0.224
8217	0.505	1.430	0.508	0.512	1.453	0.309	0.447	1.060	0.142
8218	0.411	0.824	0.142	0.491	1.321	0.501	0.482	1.279	0.328
8219	0.511	1.464	0.097	0.512	1.453	0.501	0.405	0.790	0.501
8220	0.445	1.043	0.492	0.497	1.359	0.508	0.492	1.346	0.501
8221	0.463	1.161	0.481	0.509	1.434	0.508	0.408	0.807	0.492
8222	0.482	1.279	0.513	0.512	1.453	0.512	0.495	1.363	0.512
8223	0.474	1.228	0.097	0.512	1.453	0.512	0.508	1.447	0.512
8224	0.487	1.312	0.513	0.506	1.415	0.309	0.505	1.430	0.512
8225	0.492	1.346	0.431	0.474	1.208	0.508	0.487	1.312	0.481
8226	0.432	0.958	0.409	0.468	1.170	0.273	0.513	1.481	0.142
8227	0.500	1.397	0.296	0.518	1.491	0.341	0.450	1.076	0.508
8228	0.097	-1.181	0.492	0.345	0.379	0.462	0.097	-1.181	0.512
8229	0.503	1.413	0.513	0.503	1.396	0.273	0.492	1.346	0.467
8230	0.229	-0.339	0.296	0.196	-0.581	0.514	0.295	0.082	0.512
8231	0.479	1.262	0.296	0.512	1.453	0.462	0.426	0.925	0.501

8232	0.097	-1.181	0.296	0.310	0.153	0.491	0.226	-0.356	0.262
8233	0.500	1.397	0.450	0.497	1.359	0.491	0.468	1.194	0.481
8234	0.500	1.397	0.450	0.497	1.359	0.309	0.468	1.194	0.097
8235	0.511	1.464	0.224	0.503	1.396	0.309	0.508	1.447	0.508
8236	0.505	1.430	0.224	0.506	1.415	0.422	0.450	1.076	0.050
8237	0.511	1.464	0.224	0.509	1.434	0.501	0.450	1.076	0.512
8238	0.500	1.397	0.450	0.512	1.453	0.512	0.505	1.430	0.358
8239	0.295	0.082	0.385	0.310	0.153	0.235	0.224	-0.373	0.409
8240	0.505	1.430	0.385	0.509	1.434	0.508	0.476	1.245	0.508
8241	0.184	-0.625	0.409	0.278	-0.054	0.512	0.097	-1.181	0.358
8242	0.142	-0.895	0.492	0.342	0.361	0.491	0.097	-1.181	0.185
8243	0.508	1.447	0.508	0.459	1.114	0.462	0.513	1.481	0.512
8244	0.142	-0.895	0.508	0.275	-0.073	0.478	0.142	-0.895	0.492
8245	0.505	1.430	0.467	0.515	1.472	0.491	0.482	1.279	0.492
8246	0.497	1.380	0.512	0.465	1.152	0.235	0.432	0.958	0.262
8247	0.503	1.413	0.358	0.491	1.321	0.462	0.450	1.076	0.508
8248	0.508	1.447	0.185	0.491	1.321	0.149	0.450	1.076	0.512
8249	0.358	0.487	0.296	0.310	0.153	0.508	0.300	0.116	0.512
8250	0.358	0.487	0.467	0.310	0.153	0.512	0.300	0.116	0.142
8251	0.492	1.346	0.508	0.424	0.888	0.422	0.453	1.093	0.501
8252	0.500	1.397	0.185	0.497	1.359	0.193	0.447	1.060	0.450
8253	0.508	1.447	0.328	0.506	1.415	0.501	0.503	1.413	0.409
8254	0.097	-1.181	0.409	0.237	-0.317	0.501	0.226	-0.356	0.409
8255	0.505	1.430	0.224	0.509	1.434	0.193	0.466	1.178	0.431
8256	0.500	1.397	0.224	0.515	1.472	0.491	0.484	1.295	0.512
8257	0.505	1.430	0.097	0.488	1.302	0.273	0.500	1.397	0.512
8258	0.505	1.430	0.512	0.465	1.152	0.508	0.500	1.397	0.409
8259	0.513	1.481	0.508	0.480	1.246	0.235	0.511	1.464	0.262
8260	0.513	1.481	0.512	0.488	1.302	0.501	0.511	1.464	0.262
8261	0.142	-0.895	0.492	0.234	-0.336	0.501	0.224	-0.373	0.296
8262	0.471	1.211	0.508	0.462	1.133	0.508	0.508	1.447	0.512
8263	0.142	-0.895	0.296	0.152	-0.863	0.309	0.263	-0.120	0.492
8264	0.503	1.413	0.450	0.503	1.396	0.341	0.479	1.262	0.409
8265	0.447	1.060	0.262	0.509	1.434	0.149	0.513	1.481	0.501
8266	0.450	1.076	0.513	0.424	0.888	0.491	0.508	1.447	0.481
8267	0.261	-0.137	0.142	0.193	-0.600	0.501	0.226	-0.356	0.481
8268	0.511	1.464	0.512	0.500	1.378	0.501	0.411	0.824	0.512
8269	0.508	1.447	0.492	0.500	1.378	0.309	0.476	1.245	0.409
8270	0.226	-0.356	0.481	0.193	-0.600	0.193	0.226	-0.356	0.492
8271	0.495	1.363	0.492	0.491	1.321	0.273	0.384	0.655	0.142
8272	0.224	-0.373	0.492	0.275	-0.073	0.235	0.297	0.099	0.328
8273	0.513	1.481	0.492	0.509	1.434	0.149	0.450	1.076	0.492
8274	0.300	0.116	0.512	0.234	-0.336	0.235	0.142	-0.895	0.431
8275	0.516	1.498	0.512	0.503	1.396	0.491	0.466	1.178	0.097
8276	0.508	1.447	0.481	0.500	1.378	0.422	0.513	1.481	0.481
8277	0.505	1.430	0.501	0.509	1.434	0.514	0.461	1.144	0.512
8278	0.142	-0.895	0.467	0.313	0.172	0.193	0.097	-1.181	0.142
8279	0.297	0.099	0.501	0.345	0.379	0.235	0.050	-1.485	0.296
8280	0.224	-0.373	0.097	0.149	-0.882	0.309	0.224	-0.373	0.142
8281	0.505	1.430	0.512	0.491	1.321	0.149	0.492	1.346	0.508
8282	0.505	1.430	0.450	0.503	1.396	0.193	0.500	1.397	0.450
8283	0.511	1.464	0.512	0.500	1.378	0.512	0.447	1.060	0.409
8284	0.184	-0.625	0.481	0.310	0.153	0.462	0.266	-0.103	0.508
8285	0.142	-0.895	0.328	0.193	-0.600	0.512	0.266	-0.103	0.492
8286	0.145	-0.878	0.328	0.272	-0.091	0.491	0.142	-0.895	0.185
8287	0.097	-1.181	0.508	0.234	-0.336	0.491	0.263	-0.120	0.508
8288	0.329	0.301	0.501	0.149	-0.882	0.491	0.100	-1.164	0.185
8289	0.263	-0.120	0.481	0.234	-0.336	0.444	0.224	-0.373	0.481
8290	0.476	1.245	0.508	0.491	1.321	0.235	0.511	1.464	0.262
8291	0.500	1.397	0.481	0.421	0.869	0.512	0.489	1.329	0.508
8292	0.497	1.380	0.185	0.515	1.472	0.462	0.326	0.285	0.508

8293	0.097	-1.181	0.508	0.193	-0.600	0.491	0.261	-0.137	0.501
8294	0.261	-0.137	0.097	0.234	-0.336	0.462	0.184	-0.625	0.513
8295	0.226	-0.356	0.492	0.310	0.153	0.491	0.224	-0.373	0.513
8296	0.261	-0.137	0.296	0.149	-0.882	0.235	0.142	-0.895	0.513
8297	0.295	0.082	0.450	0.196	-0.581	0.508	0.326	0.285	0.501
8298	0.497	1.380	0.513	0.509	1.434	0.235	0.500	1.397	0.450
8299	0.429	0.942	0.050	0.465	1.152	0.478	0.497	1.380	0.512
8300	0.511	1.464	0.513	0.515	1.472	0.235	0.489	1.329	0.481
8301	0.508	1.447	0.328	0.491	1.321	0.422	0.505	1.430	0.501
8302	0.508	1.447	0.328	0.491	1.321	0.371	0.505	1.430	0.492
8303	0.508	1.447	0.431	0.491	1.321	0.512	0.505	1.430	0.142
8304	0.503	1.413	0.358	0.444	1.020	0.508	0.482	1.279	0.358
8305	0.329	0.301	0.481	0.240	-0.299	0.273	0.142	-0.895	0.492
8306	0.484	1.295	0.512	0.512	1.453	0.273	0.503	1.413	0.358
8307	0.450	1.076	0.512	0.468	1.170	0.462	0.503	1.413	0.481
8308	0.408	0.807	0.512	0.494	1.340	0.491	0.461	1.144	0.097
8309	0.505	1.430	0.501	0.468	1.170	0.501	0.505	1.430	0.097
8310	0.474	1.228	0.185	0.494	1.340	0.309	0.495	1.363	0.481
8311	0.187	-0.608	0.501	0.237	-0.317	0.444	0.261	-0.137	0.501
8312	0.500	1.397	0.508	0.509	1.434	0.491	0.484	1.295	0.508
8313	0.261	-0.137	0.508	0.234	-0.336	0.462	0.097	-1.181	0.492
8314	0.450	1.076	0.512	0.482	1.265	0.398	0.505	1.430	0.431
8315	0.295	0.082	0.050	0.237	-0.317	0.309	0.050	-1.485	0.328
8316	0.492	1.346	0.050	0.421	0.869	0.512	0.513	1.481	0.450
8317	0.226	-0.356	0.513	0.374	0.568	0.309	0.358	0.487	0.142
8318	0.482	1.279	0.512	0.520	1.509	0.371	0.408	0.807	0.508
8319	0.503	1.413	0.050	0.506	1.415	0.273	0.505	1.430	0.097
8320	0.142	-0.895	0.512	0.272	-0.091	0.462	0.361	0.504	0.409
8321	0.097	-1.181	0.481	0.272	-0.091	0.462	0.184	-0.625	0.492
8322	0.495	1.363	0.481	0.462	1.133	0.309	0.511	1.464	0.142
8323	0.482	1.279	0.467	0.488	1.302	0.309	0.489	1.329	0.512
8324	0.511	1.464	0.097	0.506	1.415	0.273	0.487	1.312	0.142
8325	0.097	-1.181	0.508	0.313	0.172	0.309	0.261	-0.137	0.142
8326	0.513	1.481	0.097	0.447	1.039	0.514	0.511	1.464	0.328
8327	0.432	0.958	0.512	0.491	1.321	0.512	0.516	1.498	0.224
8328	0.405	0.790	0.501	0.462	1.133	0.508	0.513	1.481	0.508
8329	0.297	0.099	0.481	0.401	0.737	0.491	0.142	-0.895	0.492
8330	0.500	1.397	0.185	0.307	0.135	0.512	0.497	1.380	0.501
8331	0.511	1.464	0.467	0.509	1.434	0.501	0.445	1.043	0.508
8332	0.295	0.082	0.481	0.310	0.153	0.512	0.413	0.841	0.481
8333	0.295	0.082	0.050	0.374	0.568	0.508	0.413	0.841	0.185
8334	0.295	0.082	0.050	0.275	-0.073	0.491	0.434	0.975	0.481
8335	0.453	1.093	0.050	0.462	1.133	0.235	0.513	1.481	0.513
8336	0.453	1.093	0.508	0.462	1.133	0.371	0.513	1.481	0.512
8337	0.226	-0.356	0.467	0.307	0.135	0.478	0.413	0.841	0.508
8338	0.226	-0.356	0.262	0.307	0.135	0.508	0.263	-0.120	0.185
8339	0.226	-0.356	0.467	0.272	-0.091	0.273	0.263	-0.120	0.224
8340	0.450	1.076	0.492	0.310	0.153	0.193	0.300	0.116	0.512
8341	0.387	0.672	0.185	0.515	1.472	0.512	0.511	1.464	0.512
8342	0.384	0.655	0.467	0.512	1.453	0.235	0.489	1.329	0.185
8343	0.411	0.824	0.512	0.509	1.434	0.102	0.408	0.807	0.513
8344	0.492	1.346	0.508	0.488	1.302	0.309	0.503	1.413	0.508
8345	0.505	1.430	0.224	0.512	1.453	0.478	0.482	1.279	0.512
8346	0.505	1.430	0.224	0.497	1.359	0.512	0.476	1.245	0.492
8347	0.463	1.161	0.328	0.509	1.434	0.512	0.508	1.447	0.185
8348	0.513	1.481	0.262	0.512	1.453	0.478	0.405	0.790	0.513
8349	0.361	0.504	0.492	0.491	1.321	0.444	0.487	1.312	0.224
8350	0.184	-0.625	0.142	0.237	-0.317	0.235	0.145	-0.878	0.481
8351	0.297	0.099	0.185	0.371	0.549	0.514	0.329	0.301	0.513
8352	0.466	1.178	0.262	0.480	1.246	0.309	0.495	1.363	0.501
8353	0.508	1.447	0.501	0.503	1.396	0.501	0.426	0.925	0.224

8354	0.184	−0.625	0.512	0.275	−0.073	0.149	0.097	−1.181	0.512
8355	0.326	0.285	0.501	0.193	−0.600	0.478	0.479	1.262	0.508
8356	0.411	0.824	0.501	0.509	1.434	0.512	0.511	1.464	0.431
8357	0.224	−0.373	0.262	0.237	−0.317	0.514	0.142	−0.895	0.409
8358	0.224	−0.373	0.492	0.102	−1.184	0.478	0.297	0.099	0.409
8359	0.097	−1.181	0.501	0.307	0.135	0.478	0.145	−0.878	0.513
8360	0.508	1.447	0.512	0.477	1.227	0.478	0.505	1.430	0.492
8361	0.503	1.413	0.481	0.512	1.453	0.514	0.450	1.076	0.185
8362	0.508	1.447	0.492	0.512	1.453	0.478	0.411	0.824	0.508
8363	0.492	1.346	0.185	0.480	1.246	0.422	0.508	1.447	0.508
8364	0.508	1.447	0.142	0.447	1.039	0.508	0.489	1.329	0.450
8365	0.297	0.099	0.501	0.237	−0.317	0.478	0.187	−0.608	0.450
8366	0.453	1.093	0.512	0.520	1.509	0.462	0.505	1.430	0.431
8367	0.263	−0.120	0.467	0.310	0.153	0.341	0.184	−0.625	0.508
8368	0.516	1.498	0.296	0.506	1.415	0.508	0.482	1.279	0.185
8369	0.142	−0.895	0.501	0.149	−0.882	0.422	0.266	−0.103	0.185
8370	0.505	1.430	0.467	0.474	1.208	0.501	0.511	1.464	0.050
8371	0.489	1.329	0.185	0.515	1.472	0.491	0.511	1.464	0.224
8372	0.479	1.262	0.508	0.518	1.491	0.149	0.500	1.397	0.492
8373	0.489	1.329	0.512	0.477	1.227	0.193	0.508	1.447	0.328
8374	0.489	1.329	0.508	0.477	1.227	0.512	0.508	1.447	0.262
8375	0.489	1.329	0.513	0.477	1.227	0.512	0.508	1.447	0.097
8376	0.511	1.464	0.492	0.515	1.472	0.512	0.503	1.413	0.508
8377	0.511	1.464	0.224	0.471	1.189	0.422	0.453	1.093	0.501
8378	0.482	1.279	0.224	0.424	0.888	0.491	0.516	1.498	0.508
8379	0.497	1.380	0.050	0.509	1.434	0.341	0.474	1.228	0.508
8380	0.463	1.161	0.481	0.477	1.227	0.478	0.497	1.380	0.224
8381	0.495	1.363	0.508	0.462	1.133	0.309	0.489	1.329	0.508
8382	0.097	−1.181	0.142	0.342	0.361	0.444	0.142	−0.895	0.501
8383	0.505	1.430	0.512	0.512	1.453	0.309	0.358	0.487	0.501
8384	0.447	1.060	0.450	0.424	0.888	0.501	0.489	1.329	0.409
8385	0.508	1.447	0.185	0.503	1.396	0.501	0.355	0.470	0.467
8386	0.476	1.245	0.142	0.494	1.340	0.341	0.474	1.228	0.224
8387	0.332	0.318	0.508	0.149	−0.882	0.341	0.097	−1.181	0.142
8388	0.329	0.301	0.501	0.193	−0.600	0.273	0.097	−1.181	0.513
8389	0.503	1.413	0.492	0.512	1.453	0.273	0.482	1.279	0.512
8390	0.497	1.380	0.185	0.512	1.453	0.309	0.503	1.413	0.467
8391	0.479	1.262	0.431	0.509	1.434	0.273	0.508	1.447	0.097
8392	0.511	1.464	0.508	0.418	0.850	0.478	0.495	1.363	0.492
8393	0.484	1.295	0.224	0.491	1.321	0.478	0.429	0.942	0.508
8394	0.184	−0.625	0.512	0.345	0.379	0.508	0.329	0.301	0.142
8395	0.505	1.430	0.512	0.477	1.227	0.491	0.447	1.060	0.481
8396	0.097	−1.181	0.512	0.307	0.135	0.491	0.142	−0.895	0.501
8397	0.489	1.329	0.512	0.444	1.020	0.309	0.503	1.413	0.508
8398	0.297	0.099	0.501	0.310	0.153	0.478	0.097	−1.181	0.450
8399	0.450	1.076	0.097	0.494	1.340	0.478	0.408	0.807	0.431
8400	0.505	1.430	0.185	0.503	1.396	0.478	0.489	1.329	0.296
8401	0.050	−1.485	0.050	0.345	0.379	0.508	0.142	−0.895	0.328
8402	0.505	1.430	0.050	0.339	0.342	0.341	0.511	1.464	0.097
8403	0.326	0.285	0.050	0.272	−0.091	0.341	0.142	−0.895	0.512
8404	0.326	0.285	0.050	0.272	−0.091	0.462	0.142	−0.895	0.513
8405	0.432	0.958	0.467	0.307	0.135	0.478	0.329	0.301	0.262
8406	0.358	0.487	0.467	0.272	−0.091	0.309	0.226	−0.356	0.512
8407	0.482	1.279	0.467	0.480	1.246	0.501	0.511	1.464	0.431
8408	0.500	1.397	0.467	0.482	1.265	0.512	0.495	1.363	0.185
8409	0.500	1.397	0.328	0.512	1.453	0.512	0.505	1.430	0.142
8410	0.505	1.430	0.050	0.494	1.340	0.462	0.505	1.430	0.501
8411	0.503	1.413	0.224	0.485	1.283	0.273	0.476	1.245	0.512
8412	0.184	−0.625	0.431	0.307	0.135	0.444	0.184	−0.625	0.508
8413	0.503	1.413	0.450	0.480	1.246	0.235	0.479	1.262	0.262
8414	0.497	1.380	0.296	0.480	1.246	0.512	0.513	1.481	0.481

8415	0.497	1.380	0.481	0.480	1.246	0.508	0.511	1.464	0.431
8416	0.503	1.413	0.481	0.512	1.453	0.462	0.513	1.481	0.224
8417	0.050	-1.485	0.508	0.342	0.361	0.149	0.184	-0.625	0.481
8418	0.050	-1.485	0.409	0.342	0.361	0.398	0.224	-0.373	0.481
8419	0.500	1.397	0.431	0.471	1.189	0.422	0.511	1.464	0.481
8420	0.500	1.397	0.467	0.482	1.265	0.309	0.513	1.481	0.508
8421	0.050	-1.485	0.262	0.310	0.153	0.273	0.187	-0.608	0.450
8422	0.503	1.413	0.492	0.506	1.415	0.273	0.511	1.464	0.050
8423	0.479	1.262	0.512	0.515	1.472	0.508	0.511	1.464	0.050
8424	0.479	1.262	0.508	0.515	1.472	0.512	0.513	1.481	0.050
8425	0.461	1.144	0.262	0.468	1.170	0.102	0.497	1.380	0.296
8426	0.097	-1.181	0.508	0.272	-0.091	0.398	0.184	-0.625	0.224
8427	0.505	1.430	0.501	0.444	1.020	0.462	0.508	1.447	0.224
8428	0.097	-1.181	0.508	0.237	-0.317	0.102	0.226	-0.356	0.224
8429	0.500	1.397	0.508	0.518	1.491	0.491	0.482	1.279	0.450
8430	0.495	1.363	0.142	0.512	1.453	0.512	0.516	1.498	0.467
8431	0.474	1.228	0.097	0.468	1.170	0.491	0.503	1.413	0.508
8432	0.184	-0.625	0.050	0.152	-0.863	0.193	0.226	-0.356	0.508
8433	0.461	1.144	0.492	0.401	0.737	0.193	0.508	1.447	0.262
8434	0.474	1.228	0.492	0.427	0.907	0.149	0.505	1.430	0.296
8435	0.050	-1.485	0.492	0.307	0.135	0.235	0.434	0.975	0.296
8436	0.050	-1.485	0.492	0.272	-0.091	0.512	0.411	0.824	0.508
8437	0.050	-1.485	0.512	0.272	-0.091	0.273	0.411	0.824	0.501
8438	0.497	1.380	0.492	0.509	1.434	0.102	0.513	1.481	0.224
8439	0.463	1.161	0.262	0.518	1.491	0.273	0.495	1.363	0.492
8440	0.261	-0.137	0.224	0.102	-1.184	0.512	0.184	-0.625	0.492
8441	0.466	1.178	0.224	0.398	0.718	0.491	0.508	1.447	0.508
8442	0.489	1.329	0.508	0.462	1.133	0.512	0.508	1.447	0.492
8443	0.184	-0.625	0.508	0.102	-1.184	0.512	0.447	1.060	0.492
8444	0.466	1.178	0.481	0.488	1.302	0.193	0.450	1.076	0.508
8445	0.508	1.447	0.481	0.512	1.453	0.508	0.429	0.942	0.185
8446	0.508	1.447	0.512	0.491	1.321	0.478	0.497	1.380	0.450
8447	0.226	-0.356	0.512	0.193	-0.600	0.491	0.184	-0.625	0.501
8448	0.226	-0.356	0.508	0.193	-0.600	0.512	0.184	-0.625	0.508
8449	0.329	0.301	0.481	0.152	-0.863	0.478	0.050	-1.485	0.296
8450	0.263	-0.120	0.492	0.234	-0.336	0.422	0.226	-0.356	0.481
8451	0.489	1.329	0.142	0.512	1.453	0.273	0.492	1.346	0.501
8452	0.142	-0.895	0.431	0.275	-0.073	0.514	0.332	0.318	0.501
8453	0.184	-0.625	0.512	0.102	-1.184	0.462	0.263	-0.120	0.512
8454	0.263	-0.120	0.467	0.272	-0.091	0.508	0.097	-1.181	0.185
8455	0.500	1.397	0.142	0.509	1.434	0.193	0.503	1.413	0.185
8456	0.511	1.464	0.501	0.488	1.302	0.512	0.497	1.380	0.224
8457	0.497	1.380	0.296	0.506	1.415	0.508	0.508	1.447	0.097
8458	0.497	1.380	0.450	0.506	1.415	0.273	0.508	1.447	0.501
8459	0.261	-0.137	0.142	0.193	-0.600	0.508	0.224	-0.373	0.501
8460	0.489	1.329	0.492	0.509	1.434	0.514	0.503	1.413	0.501
8461	0.497	1.380	0.296	0.474	1.208	0.444	0.500	1.397	0.501
8462	0.505	1.430	0.508	0.488	1.302	0.508	0.500	1.397	0.467
8463	0.479	1.262	0.501	0.506	1.415	0.501	0.408	0.807	0.501
8464	0.487	1.312	0.050	0.480	1.246	0.102	0.466	1.178	0.142
8465	0.184	-0.625	0.142	0.421	0.869	0.102	0.224	-0.373	0.328
8466	0.145	-0.878	0.185	0.272	-0.091	0.398	0.142	-0.895	0.097
8467	0.497	1.380	0.512	0.512	1.453	0.462	0.513	1.481	0.097
8468	0.508	1.447	0.492	0.462	1.133	0.501	0.511	1.464	0.501
8469	0.466	1.178	0.508	0.506	1.415	0.235	0.461	1.144	0.492
8470	0.295	0.082	0.512	0.193	-0.600	0.462	0.097	-1.181	0.450
8471	0.500	1.397	0.296	0.512	1.453	0.501	0.487	1.312	0.467
8472	0.471	1.211	0.296	0.509	1.434	0.193	0.500	1.397	0.512
8473	0.184	-0.625	0.513	0.272	-0.091	0.341	0.142	-0.895	0.512
8474	0.508	1.447	0.050	0.506	1.415	0.501	0.476	1.245	0.358
8475	0.505	1.430	0.501	0.512	1.453	0.462	0.500	1.397	0.501

8476	0.500	1.397	0.508	0.444	1.020	0.462	0.511	1.464	0.492
8477	0.516	1.498	0.508	0.503	1.396	0.149	0.453	1.093	0.467
8478	0.495	1.363	0.508	0.497	1.359	0.478	0.434	0.975	0.185
8479	0.224	-0.373	0.097	0.102	-1.184	0.512	0.297	0.099	0.450
8480	0.224	-0.373	0.508	0.102	-1.184	0.102	0.329	0.301	0.467
8481	0.050	-1.485	0.328	0.395	0.700	0.512	0.097	-1.181	0.508
8482	0.484	1.295	0.050	0.459	1.114	0.512	0.505	1.430	0.262
8483	0.503	1.413	0.512	0.503	1.396	0.514	0.511	1.464	0.481
8484	0.142	-0.895	0.358	0.234	-0.336	0.478	0.263	-0.120	0.142
8485	0.505	1.430	0.501	0.459	1.114	0.512	0.513	1.481	0.512
8486	0.450	1.076	0.467	0.500	1.378	0.462	0.432	0.958	0.296
8487	0.187	-0.608	0.185	0.193	-0.600	0.371	0.184	-0.625	0.431
8488	0.142	-0.895	0.185	0.342	0.361	0.371	0.142	-0.895	0.097
8489	0.503	1.413	0.262	0.503	1.396	0.193	0.500	1.397	0.501
8490	0.497	1.380	0.492	0.465	1.152	0.341	0.505	1.430	0.512
8491	0.492	1.346	0.262	0.462	1.133	0.341	0.500	1.397	0.328
8492	0.184	-0.625	0.224	0.149	-0.882	0.309	0.261	-0.137	0.328
8493	0.429	0.942	0.512	0.474	1.208	0.491	0.479	1.262	0.262
8494	0.497	1.380	0.262	0.515	1.472	0.491	0.429	0.942	0.450
8495	0.226	-0.356	0.296	0.102	-1.184	0.512	0.226	-0.356	0.501
8496	0.513	1.481	0.296	0.512	1.453	0.512	0.476	1.245	0.450
8497	0.513	1.481	0.185	0.512	1.453	0.273	0.476	1.245	0.512
8498	0.513	1.481	0.142	0.512	1.453	0.235	0.476	1.245	0.185
8499	0.508	1.447	0.142	0.477	1.227	0.235	0.505	1.430	0.185
8500	0.495	1.363	0.431	0.515	1.472	0.491	0.450	1.076	0.431
8501	0.000	?	0.224	0.462	1.133	0.491	0.050	-1.485	0.296
8502	0.097	-1.181	0.508	0.374	0.568	0.235	0.050	-1.485	0.501
8503	0.184	-0.625	0.450	0.374	0.568	0.508	0.050	-1.485	0.501
8504	0.050	-1.485	0.512	0.193	-0.600	0.508	0.295	0.082	0.501
8505	0.050	-1.485	0.296	0.342	0.361	0.508	0.224	-0.373	0.501
8506	0.050	-1.485	0.358	0.342	0.361	0.501	0.224	-0.373	0.262
8507	0.050	-1.485	0.481	0.307	0.135	0.512	0.224	-0.373	0.508
8508	0.461	1.144	0.142	0.488	1.302	0.508	0.453	1.093	0.262
8509	0.461	1.144	0.050	0.488	1.302	0.149	0.471	1.211	0.185
8510	0.463	1.161	0.262	0.509	1.434	0.491	0.505	1.430	0.512
8511	0.463	1.161	0.492	0.509	1.434	0.512	0.505	1.430	0.513
8512	0.326	0.285	0.262	0.272	-0.091	0.508	0.263	-0.120	0.501
8513	0.050	-1.485	0.224	0.234	-0.336	0.193	0.297	0.099	0.467
8514	0.224	-0.373	0.262	0.234	-0.336	0.478	0.303	0.133	0.262
8515	0.426	0.925	0.409	0.491	1.321	0.501	0.505	1.430	0.224
8516	0.445	1.043	0.185	0.491	1.321	0.462	0.500	1.397	0.142
8517	0.297	0.099	0.492	0.234	-0.336	0.512	0.226	-0.356	0.508
8518	0.482	1.279	0.142	0.503	1.396	0.309	0.487	1.312	0.050
8519	0.482	1.279	0.142	0.503	1.396	0.273	0.487	1.312	0.097
8520	0.505	1.430	0.492	0.506	1.415	0.371	0.508	1.447	0.492
8521	0.405	0.790	0.481	0.503	1.396	0.398	0.489	1.329	0.097
8522	0.426	0.925	0.501	0.515	1.472	0.444	0.489	1.329	0.050
8523	0.468	1.194	0.328	0.509	1.434	0.444	0.503	1.413	0.050
8524	0.263	-0.120	0.296	0.149	-0.882	0.444	0.187	-0.608	0.142
8525	0.489	1.329	0.450	0.488	1.302	0.444	0.447	1.060	0.097
8526	0.505	1.430	0.262	0.512	1.453	0.508	0.503	1.413	0.262
8527	0.505	1.430	0.450	0.509	1.434	0.462	0.505	1.430	0.450
8528	0.263	-0.120	0.142	0.196	-0.581	0.193	0.297	0.099	0.142
8529	0.503	1.413	0.296	0.477	1.227	0.235	0.476	1.245	0.492
8530	0.500	1.397	0.467	0.500	1.378	0.273	0.497	1.380	0.481
8531	0.503	1.413	0.501	0.462	1.133	0.273	0.500	1.397	0.492
8532	0.500	1.397	0.050	0.512	1.453	0.512	0.511	1.464	0.097
8533	0.142	-0.895	0.508	0.313	0.172	0.514	0.184	-0.625	0.097
8534	0.097	-1.181	0.142	0.278	-0.054	0.508	0.184	-0.625	0.492
8535	0.050	-1.485	0.492	0.368	0.530	0.508	0.224	-0.373	0.142
8536	0.000	?	0.513	0.398	0.718	0.462	0.097	-1.181	0.185

8537	0.484	1.295	0.050	0.444	1.020	0.462	0.497	1.380	0.097
8538	0.484	1.295	0.512	0.444	1.020	0.398	0.497	1.380	0.501
8539	0.484	1.295	0.508	0.444	1.020	0.514	0.497	1.380	0.050
8540	0.484	1.295	0.492	0.444	1.020	0.508	0.497	1.380	0.142
8541	0.500	1.397	0.501	0.515	1.472	0.341	0.466	1.178	0.050
8542	0.487	1.312	0.501	0.468	1.170	0.273	0.503	1.413	0.513
8543	0.261	-0.137	0.328	0.196	-0.581	0.422	0.142	-0.895	0.185
8544	0.000	?	0.328	0.237	-0.317	0.501	0.326	0.285	0.508
8545	0.226	-0.356	0.328	0.275	-0.073	0.491	0.097	-1.181	0.097
8546	0.226	-0.356	0.224	0.275	-0.073	0.235	0.097	-1.181	0.097
8547	0.497	1.380	0.142	0.518	1.491	0.501	0.497	1.380	0.481
8548	0.497	1.380	0.512	0.520	1.509	0.102	0.489	1.329	0.492
8549	0.476	1.245	0.508	0.506	1.415	0.478	0.450	1.076	0.481
8550	0.476	1.245	0.512	0.506	1.415	0.235	0.466	1.178	0.097
8551	0.505	1.430	0.501	0.462	1.133	0.491	0.508	1.447	0.050
8552	0.505	1.430	0.501	0.462	1.133	0.149	0.508	1.447	0.513
8553	0.000	?	0.512	0.401	0.737	0.341	0.358	0.487	0.185
8554	0.497	1.380	0.512	0.520	1.509	0.508	0.505	1.430	0.508
8555	0.476	1.245	0.512	0.506	1.415	0.193	0.495	1.363	0.185
8556	0.487	1.312	0.512	0.342	0.361	0.422	0.466	1.178	0.508
8557	0.142	-0.895	0.508	0.272	-0.091	0.193	0.187	-0.608	0.501
8558	0.426	0.925	0.501	0.424	0.888	0.371	0.445	1.043	0.328
8559	0.508	1.447	0.501	0.497	1.359	0.508	0.466	1.178	0.296
8560	0.463	1.161	0.481	0.488	1.302	0.478	0.508	1.447	0.481
8561	0.142	-0.895	0.508	0.234	-0.336	0.512	0.263	-0.120	0.185
8562	0.497	1.380	0.224	0.494	1.340	0.102	0.482	1.279	0.508
8563	0.300	0.116	0.185	0.102	-1.184	0.102	0.142	-0.895	0.513
8564	0.447	1.060	0.185	0.477	1.227	0.501	0.508	1.447	0.097
8565	0.142	-0.895	0.185	0.234	-0.336	0.273	0.300	0.116	0.481
8566	0.484	1.295	0.431	0.491	1.321	0.514	0.426	0.925	0.508
8567	0.300	0.116	0.467	0.149	-0.882	0.508	0.097	-1.181	0.513
8568	0.508	1.447	0.481	0.342	0.361	0.478	0.500	1.397	0.492
8569	0.497	1.380	0.501	0.509	1.434	0.491	0.513	1.481	0.508
8570	0.050	-1.485	0.328	0.193	-0.600	0.193	0.329	0.301	0.050
8571	0.142	-0.895	0.492	0.424	0.888	0.491	0.332	0.318	0.050
8572	0.184	-0.625	0.492	0.196	-0.581	0.309	0.263	-0.120	0.050
8573	0.508	1.447	0.185	0.371	0.549	0.371	0.453	1.093	0.097
8574	0.487	1.312	0.142	0.509	1.434	0.491	0.505	1.430	0.262
8575	0.500	1.397	0.142	0.480	1.246	0.512	0.450	1.076	0.431
8576	0.508	1.447	0.501	0.506	1.415	0.512	0.513	1.481	0.481
8577	0.297	0.099	0.262	0.102	-1.184	0.501	0.187	-0.608	0.467
8578	0.297	0.099	0.358	0.102	-1.184	0.193	0.187	-0.608	0.481
8579	0.516	1.498	0.501	0.500	1.378	0.193	0.432	0.958	0.481
8580	0.050	-1.485	0.467	0.272	-0.091	0.193	0.297	0.099	0.513
8581	0.497	1.380	0.512	0.512	1.453	0.501	0.497	1.380	0.513
8582	0.503	1.413	0.385	0.509	1.434	0.235	0.497	1.380	0.513
8583	0.503	1.413	0.450	0.477	1.227	0.235	0.497	1.380	0.513
8584	0.503	1.413	0.467	0.491	1.321	0.501	0.497	1.380	0.508
8585	0.097	-1.181	0.358	0.193	-0.600	0.235	0.261	-0.137	0.142
8586	0.500	1.397	0.501	0.491	1.321	0.193	0.505	1.430	0.481
8587	0.329	0.301	0.512	0.313	0.172	0.273	0.261	-0.137	0.467
8588	0.050	-1.485	0.097	0.368	0.530	0.235	0.184	-0.625	0.481
8589	0.505	1.430	0.296	0.491	1.321	0.309	0.513	1.481	0.501
8590	0.355	0.470	0.492	0.512	1.453	0.309	0.505	1.430	0.296
8591	0.497	1.380	0.097	0.509	1.434	0.501	0.500	1.397	0.097
8592	0.468	1.194	0.262	0.497	1.359	0.193	0.463	1.161	0.142
8593	0.184	-0.625	0.296	0.193	-0.600	0.444	0.261	-0.137	0.358
8594	0.184	-0.625	0.385	0.193	-0.600	0.508	0.224	-0.373	0.481
8595	0.263	-0.120	0.513	0.196	-0.581	0.478	0.142	-0.895	0.492
8596	0.495	1.363	0.481	0.497	1.359	0.193	0.500	1.397	0.501
8597	0.263	-0.120	0.185	0.237	-0.317	0.235	0.050	-1.485	0.508

8598	0.226	-0.356	0.296	0.234	-0.336	0.491	0.097	-1.181	0.185
8599	0.511	1.464	0.224	0.500	1.378	0.273	0.484	1.295	0.481
8600	0.261	-0.137	0.262	0.234	-0.336	0.371	0.097	-1.181	0.513
8601	0.297	0.099	0.296	0.193	-0.600	0.341	0.050	-1.485	0.185
8602	0.297	0.099	0.492	0.275	-0.073	0.478	0.050	-1.485	0.262
8603	0.184	-0.625	0.501	0.237	-0.317	0.371	0.142	-0.895	0.358
8604	0.142	-0.895	0.492	0.310	0.153	0.235	0.097	-1.181	0.513
8605	0.142	-0.895	0.508	0.310	0.153	0.273	0.261	-0.137	0.328
8606	0.432	0.958	0.508	0.503	1.396	0.501	0.445	1.043	0.262
8607	0.226	-0.356	0.224	0.196	-0.581	0.193	0.142	-0.895	0.409
8608	0.508	1.447	0.492	0.442	1.001	0.444	0.487	1.312	0.501
8609	0.455	1.110	0.142	0.509	1.434	0.309	0.476	1.245	0.467
8610	0.518	1.514	0.481	0.480	1.246	0.309	0.484	1.295	0.508
8611	0.295	0.082	0.481	0.196	-0.581	0.501	0.097	-1.181	0.501
8612	0.361	0.504	0.512	0.234	-0.336	0.478	0.097	-1.181	0.501
8613	0.482	1.279	0.328	0.491	1.321	0.478	0.484	1.295	0.296
8614	0.142	-0.895	0.450	0.278	-0.054	0.102	0.142	-0.895	0.467
8615	0.050	-1.485	0.385	0.371	0.549	0.273	0.184	-0.625	0.508
8616	0.261	-0.137	0.385	0.342	0.361	0.478	0.097	-1.181	0.224
8617	0.497	1.380	0.467	0.480	1.246	0.273	0.492	1.346	0.050
8618	0.261	-0.137	0.492	0.371	0.549	0.491	0.050	-1.485	0.481
8619	0.226	-0.356	0.501	0.234	-0.336	0.273	0.142	-0.895	0.328
8620	0.261	-0.137	0.512	0.275	-0.073	0.273	0.050	-1.485	0.185
8621	0.413	0.841	0.185	0.503	1.396	0.508	0.508	1.447	0.097
8622	0.187	-0.608	0.492	0.193	-0.600	0.462	0.184	-0.625	0.224
8623	0.492	1.346	0.224	0.442	1.001	0.309	0.500	1.397	0.450
8624	0.142	-0.895	0.142	0.310	0.153	0.235	0.097	-1.181	0.508
8625	0.142	-0.895	0.409	0.310	0.153	0.514	0.097	-1.181	0.358
8626	0.487	1.312	0.185	0.503	1.396	0.273	0.479	1.262	0.097
8627	0.476	1.245	0.224	0.477	1.227	0.462	0.489	1.329	0.050
8628	0.495	1.363	0.409	0.477	1.227	0.422	0.479	1.262	0.385
8629	0.329	0.301	0.385	0.102	-1.184	0.371	0.097	-1.181	0.296
8630	0.297	0.099	0.467	0.272	-0.091	0.444	0.050	-1.485	0.513
8631	0.453	1.093	0.492	0.477	1.227	0.512	0.503	1.413	0.409
8632	0.261	-0.137	0.450	0.275	-0.073	0.501	0.184	-0.625	0.508
8633	0.450	1.076	0.328	0.494	1.340	0.512	0.497	1.380	0.501
8634	0.145	-0.878	0.431	0.275	-0.073	0.501	0.184	-0.625	0.492
8635	0.297	0.099	0.513	0.272	-0.091	0.273	0.000	?	0.385
8636	0.466	1.178	0.501	0.509	1.434	0.273	0.500	1.397	0.385
8637	0.492	1.346	0.262	0.468	1.170	0.273	0.500	1.397	0.296
8638	0.000	?	0.142	0.310	0.153	0.309	0.329	0.301	0.467
8639	0.050	-1.485	0.501	0.234	-0.336	0.309	0.297	0.099	0.467
8640	0.505	1.430	0.501	0.512	1.453	0.491	0.479	1.262	0.467
8641	0.142	-0.895	0.185	0.275	-0.073	0.478	0.184	-0.625	0.328
8642	0.489	1.329	0.508	0.456	1.095	0.478	0.505	1.430	0.450
8643	0.500	1.397	0.431	0.424	0.888	0.462	0.516	1.498	0.262
8644	0.050	-1.485	0.467	0.371	0.549	0.514	0.097	-1.181	0.262
8645	0.503	1.413	0.185	0.447	1.039	0.501	0.476	1.245	0.501
8646	0.500	1.397	0.450	0.509	1.434	0.478	0.511	1.464	0.512
8647	0.484	1.295	0.262	0.497	1.359	0.478	0.516	1.498	0.492
8648	0.492	1.346	0.512	0.509	1.434	0.478	0.495	1.363	0.481
8649	0.492	1.346	0.512	0.497	1.359	0.491	0.511	1.464	0.501
8650	0.326	0.285	0.501	0.272	-0.091	0.371	0.050	-1.485	0.185
8651	0.326	0.285	0.097	0.272	-0.091	0.512	0.050	-1.485	0.185
8652	0.326	0.285	0.328	0.272	-0.091	0.514	0.050	-1.485	0.409
8653	0.224	-0.373	0.492	0.313	0.172	0.491	0.097	-1.181	0.185
8654	0.142	-0.895	0.185	0.313	0.172	0.309	0.263	-0.120	0.262
8655	0.503	1.413	0.492	0.494	1.340	0.341	0.432	0.958	0.185
8656	0.497	1.380	0.508	0.485	1.283	0.273	0.479	1.262	0.185
8657	0.503	1.413	0.508	0.482	1.265	0.235	0.466	1.178	0.501
8658	0.492	1.346	0.296	0.465	1.152	0.053	0.479	1.262	0.508

8659	0.492	1.346	0.185	0.520	1.509	0.478	0.476	1.245	0.513
8660	0.500	1.397	0.142	0.506	1.415	0.491	0.513	1.481	0.142
8661	0.500	1.397	0.224	0.482	1.265	0.422	0.513	1.481	0.512
8662	0.500	1.397	0.513	0.482	1.265	0.444	0.513	1.481	0.492
8663	0.500	1.397	0.358	0.482	1.265	0.149	0.513	1.481	0.450
8664	0.505	1.430	0.492	0.494	1.340	0.491	0.508	1.447	0.328
8665	0.000	?	0.185	0.374	0.568	0.462	0.142	-0.895	0.296
8666	0.495	1.363	0.492	0.515	1.472	0.193	0.482	1.279	0.481
8667	0.495	1.363	0.512	0.515	1.472	0.341	0.468	1.194	0.481
8668	0.474	1.228	0.512	0.494	1.340	0.235	0.482	1.279	0.296
8669	0.497	1.380	0.467	0.310	0.153	0.501	0.500	1.397	0.501
8670	0.226	-0.356	0.224	0.342	0.361	0.193	0.295	0.082	0.513
8671	0.184	-0.625	0.050	0.278	-0.054	0.149	0.097	-1.181	0.512
8672	0.184	-0.625	0.185	0.237	-0.317	0.478	0.142	-0.895	0.050
8673	0.184	-0.625	0.185	0.053	-1.504	0.371	0.361	0.504	0.513
8674	0.434	0.975	0.512	0.477	1.227	0.501	0.476	1.245	0.050
8675	0.466	1.178	0.185	0.485	1.283	0.501	0.495	1.363	0.513
8676	0.482	1.279	0.512	0.421	0.869	0.508	0.500	1.397	0.492
8677	0.497	1.380	0.481	0.447	1.039	0.508	0.500	1.397	0.450
8678	0.326	0.285	0.508	0.149	-0.882	0.273	0.184	-0.625	0.097
8679	0.492	1.346	0.262	0.491	1.321	0.512	0.482	1.279	0.262
8680	0.492	1.346	0.385	0.462	1.133	0.309	0.513	1.481	0.467
8681	0.187	-0.608	0.328	0.193	-0.600	0.273	0.187	-0.608	0.262
8682	0.142	-0.895	0.501	0.342	0.361	0.193	0.261	-0.137	0.501
8683	0.142	-0.895	0.450	0.237	-0.317	0.444	0.355	0.470	0.481
8684	0.500	1.397	0.050	0.506	1.415	0.309	0.516	1.498	0.481
8685	0.263	-0.120	0.450	0.193	-0.600	0.309	0.332	0.318	0.097
8686	0.363	0.520	0.262	0.149	-0.882	0.235	0.261	-0.137	0.224
8687	0.503	1.413	0.262	0.474	1.208	0.341	0.405	0.790	0.224
8688	0.463	1.161	0.185	0.374	0.568	0.508	0.500	1.397	0.262
8689	0.508	1.447	0.358	0.503	1.396	0.514	0.468	1.194	0.481
8690	0.384	0.655	0.492	0.500	1.378	0.398	0.511	1.464	0.262
8691	0.445	1.043	0.513	0.506	1.415	0.149	0.503	1.413	0.431
8692	0.463	1.161	0.185	0.506	1.415	0.273	0.503	1.413	0.224
8693	0.358	0.487	0.512	0.272	-0.091	0.309	0.295	0.082	0.501
8694	0.500	1.397	0.508	0.512	1.453	0.422	0.466	1.178	0.481
8695	0.511	1.464	0.508	0.313	0.172	0.478	0.511	1.464	0.481
8696	0.097	-1.181	0.501	0.278	-0.054	0.512	0.224	-0.373	0.512
8697	0.295	0.082	0.501	0.193	-0.600	0.512	0.050	-1.485	0.185
8698	0.492	1.346	0.296	0.447	1.039	0.462	0.479	1.262	0.262
8699	0.097	-1.181	0.296	0.310	0.153	0.491	0.332	0.318	0.328
8700	0.263	-0.120	0.467	0.310	0.153	0.273	0.184	-0.625	0.328
8701	0.297	0.099	0.467	0.237	-0.317	0.501	0.097	-1.181	0.492
8702	0.387	0.672	0.262	0.342	0.361	0.273	0.226	-0.356	0.185
8703	0.513	1.481	0.492	0.506	1.415	0.508	0.445	1.043	0.508
8704	0.476	1.245	0.492	0.515	1.472	0.514	0.503	1.413	0.501
8705	0.184	-0.625	0.481	0.401	0.737	0.398	0.361	0.504	0.481
8706	0.297	0.099	0.224	0.149	-0.882	0.235	0.097	-1.181	0.358
8707	0.226	-0.356	0.467	0.272	-0.091	0.514	0.050	-1.485	0.501
8708	0.263	-0.120	0.262	0.313	0.172	0.309	0.384	0.655	0.262
8709	0.297	0.099	0.467	0.427	0.907	0.309	0.297	0.099	0.492
8710	0.495	1.363	0.481	0.480	1.246	0.508	0.513	1.481	0.513
8711	0.500	1.397	0.328	0.512	1.453	0.501	0.411	0.824	0.328
8712	0.492	1.346	0.513	0.509	1.434	0.491	0.503	1.413	0.512
8713	0.505	1.430	0.358	0.462	1.133	0.444	0.500	1.397	0.296
8714	0.503	1.413	0.481	0.494	1.340	0.341	0.495	1.363	0.097
8715	0.224	-0.373	0.512	0.272	-0.091	0.462	0.387	0.672	0.262
8716	0.492	1.346	0.296	0.503	1.396	0.149	0.382	0.638	0.262
8717	0.142	-0.895	0.467	0.278	-0.054	0.235	0.297	0.099	0.450
8718	0.476	1.245	0.262	0.506	1.415	0.501	0.466	1.178	0.501
8719	0.479	1.262	0.224	0.512	1.453	0.309	0.463	1.161	0.328

8720	0.513	1.481	0.328	0.395	0.700	0.235	0.471	1.211	0.409
8721	0.329	0.301	0.467	0.237	−0.317	0.235	0.326	0.285	0.431
8722	0.455	1.110	0.185	0.515	1.472	0.235	0.450	1.076	0.431
8723	0.387	0.672	0.185	0.310	0.153	0.462	0.261	−0.137	0.450
8724	0.387	0.672	0.501	0.310	0.153	0.462	0.261	−0.137	0.450
8725	0.468	1.194	0.409	0.512	1.453	0.462	0.503	1.413	0.142
8726	0.495	1.363	0.512	0.500	1.378	0.149	0.516	1.498	0.224
8727	0.500	1.397	0.467	0.491	1.321	0.501	0.489	1.329	0.481
8728	0.513	1.481	0.467	0.444	1.020	0.512	0.476	1.245	0.508
8729	0.184	−0.625	0.481	0.342	0.361	0.512	0.000	?	0.224
8730	0.487	1.312	0.481	0.468	1.170	0.235	0.495	1.363	0.492
8731	0.224	−0.373	0.501	0.152	−0.863	0.193	0.184	−0.625	0.492
8732	0.142	−0.895	0.224	0.237	−0.317	0.491	0.184	−0.625	0.467
8733	0.405	0.790	0.508	0.500	1.378	0.491	0.408	0.807	0.142
8734	0.184	−0.625	0.142	0.313	0.172	0.053	0.184	−0.625	0.481
8735	0.226	−0.356	0.224	0.234	−0.336	0.309	0.263	−0.120	0.358
8736	0.408	0.807	0.262	0.237	−0.317	0.501	0.184	−0.625	0.508
8737	0.382	0.638	0.431	0.237	−0.317	0.514	0.184	−0.625	0.492
8738	0.463	1.161	0.431	0.465	1.152	0.309	0.505	1.430	0.142
8739	0.489	1.329	0.513	0.462	1.133	0.462	0.511	1.464	0.501
8740	0.447	1.060	0.296	0.462	1.133	0.235	0.518	1.514	0.501
8741	0.329	0.301	0.358	0.149	−0.882	0.501	0.145	−0.878	0.450
8742	0.432	0.958	0.431	0.497	1.359	0.491	0.511	1.464	0.501
8743	0.513	1.481	0.224	0.509	1.434	0.491	0.487	1.312	0.185
8744	0.500	1.397	0.185	0.512	1.453	0.398	0.447	1.060	0.513
8745	0.266	−0.103	0.142	0.234	−0.336	0.273	0.326	0.285	0.142
8746	0.142	−0.895	0.450	0.196	−0.581	0.512	0.297	0.099	0.262
8747	0.497	1.380	0.481	0.491	1.321	0.102	0.482	1.279	0.142
8748	0.497	1.380	0.097	0.491	1.321	0.422	0.482	1.279	0.467
8749	0.187	−0.608	0.492	0.053	−1.504	0.491	0.297	0.099	0.185
8750	0.508	1.447	0.481	0.310	0.153	0.501	0.500	1.397	0.185
8751	0.432	0.958	0.481	0.506	1.415	0.273	0.516	1.498	0.508
8752	0.463	1.161	0.185	0.515	1.472	0.193	0.511	1.464	0.450
8753	0.187	−0.608	0.328	0.310	0.153	0.193	0.050	−1.485	0.431
8754	0.450	1.076	0.097	0.465	1.152	0.149	0.503	1.413	0.512
8755	0.261	−0.137	0.501	0.237	−0.317	0.444	0.050	−1.485	0.481
8756	0.513	1.481	0.501	0.494	1.340	0.235	0.516	1.498	0.492
8757	0.513	1.481	0.097	0.485	1.283	0.514	0.495	1.363	0.492
8758	0.500	1.397	0.513	0.485	1.283	0.193	0.450	1.076	0.481
8759	0.097	−1.181	0.262	0.395	0.700	0.514	0.097	−1.181	0.328
8760	0.326	0.285	0.185	0.275	−0.073	0.501	0.263	−0.120	0.501
8761	0.487	1.312	0.142	0.509	1.434	0.491	0.468	1.194	0.224
8762	0.184	−0.625	0.142	0.102	−1.184	0.478	0.261	−0.137	0.328
8763	0.492	1.346	0.185	0.427	0.907	0.149	0.495	1.363	0.262
8764	0.505	1.430	0.450	0.494	1.340	0.309	0.474	1.228	0.431
8765	0.505	1.430	0.097	0.506	1.415	0.102	0.474	1.228	0.431
8766	0.297	0.099	0.501	0.272	−0.091	0.102	0.097	−1.181	0.409
8767	0.184	−0.625	0.501	0.193	−0.600	0.501	0.224	−0.373	0.097
8768	0.142	−0.895	0.185	0.193	−0.600	0.341	0.224	−0.373	0.224
8769	0.224	−0.373	0.185	0.149	−0.882	0.462	0.261	−0.137	0.481
8770	0.513	1.481	0.142	0.447	1.039	0.491	0.479	1.262	0.185
8771	0.358	0.487	0.262	0.234	−0.336	0.508	0.263	−0.120	0.224
8772	0.489	1.329	0.450	0.515	1.472	0.273	0.429	0.942	0.224
8773	0.184	−0.625	0.385	0.196	−0.581	0.508	0.226	−0.356	0.385
8774	0.495	1.363	0.450	0.518	1.491	0.273	0.503	1.413	0.492
8775	0.516	1.498	0.224	0.503	1.396	0.514	0.482	1.279	0.224
8776	0.516	1.498	0.508	0.494	1.340	0.491	0.482	1.279	0.492
8777	0.466	1.178	0.508	0.474	1.208	0.309	0.508	1.447	0.492
8778	0.226	−0.356	0.224	0.149	−0.882	0.341	0.184	−0.625	0.481
8779	0.050	−1.485	0.262	0.310	0.153	0.193	0.263	−0.120	0.142
8780	0.184	−0.625	0.262	0.105	−1.165	0.193	0.329	0.301	0.358

8781	0.184	-0.625	0.492	0.105	-1.165	0.235	0.329	0.301	0.328
8782	0.508	1.447	0.409	0.497	1.359	0.422	0.492	1.346	0.492
8783	0.184	-0.625	0.513	0.342	0.361	0.508	0.184	-0.625	0.512
8784	0.511	1.464	0.450	0.462	1.133	0.462	0.511	1.464	0.142
8785	0.482	1.279	0.481	0.491	1.321	0.371	0.500	1.397	0.513
8786	0.508	1.447	0.481	0.506	1.415	0.508	0.479	1.262	0.185
8787	0.263	-0.120	0.481	0.275	-0.073	0.508	0.361	0.504	0.097
8788	0.387	0.672	0.481	0.512	1.453	0.501	0.500	1.397	0.142
8789	0.332	0.318	0.142	0.272	-0.091	0.491	0.263	-0.120	0.185
8790	0.500	1.397	0.492	0.515	1.472	0.491	0.492	1.346	0.224
8791	0.447	1.060	0.467	0.494	1.340	0.309	0.511	1.464	0.409
8792	0.050	-1.485	0.450	0.313	0.172	0.149	0.329	0.301	0.296
8793	0.445	1.043	0.450	0.339	0.342	0.514	0.508	1.447	0.508
8794	0.263	-0.120	0.450	0.193	-0.600	0.514	0.300	0.116	0.492
8795	0.263	-0.120	0.512	0.196	-0.581	0.273	0.097	-1.181	0.185
8796	0.184	-0.625	0.492	0.237	-0.317	0.501	0.261	-0.137	0.185
8797	0.363	0.520	0.224	0.421	0.869	0.501	0.261	-0.137	0.224
8798	0.489	1.329	0.513	0.512	1.453	0.508	0.447	1.060	0.097
8799	0.511	1.464	0.296	0.459	1.114	0.193	0.500	1.397	0.431
8800	0.184	-0.625	0.501	0.371	0.549	0.478	0.332	0.318	0.224
8801	0.505	1.430	0.492	0.512	1.453	0.149	0.405	0.790	0.481
8802	0.503	1.413	0.358	0.515	1.472	0.422	0.429	0.942	0.097
8803	0.503	1.413	0.431	0.506	1.415	0.501	0.429	0.942	0.492
8804	0.497	1.380	0.508	0.494	1.340	0.309	0.445	1.043	0.512
8805	0.497	1.380	0.385	0.494	1.340	0.491	0.445	1.043	0.097
8806	0.300	0.116	0.508	0.310	0.153	0.508	0.142	-0.895	0.097
8807	0.297	0.099	0.467	0.149	-0.882	0.508	0.224	-0.373	0.097
8808	0.468	1.194	0.296	0.515	1.472	0.491	0.474	1.228	0.467
8809	0.468	1.194	0.467	0.512	1.453	0.053	0.505	1.430	0.508
8810	0.266	-0.103	0.481	0.275	-0.073	0.501	0.224	-0.373	0.450
8811	0.492	1.346	0.508	0.500	1.378	0.149	0.489	1.329	0.481
8812	0.492	1.346	0.481	0.500	1.378	0.235	0.489	1.329	0.481
8813	0.479	1.262	0.481	0.506	1.415	0.273	0.466	1.178	0.481
8814	0.226	-0.356	0.492	0.193	-0.600	0.478	0.145	-0.878	0.481
8815	0.463	1.161	0.224	0.477	1.227	0.193	0.479	1.262	0.481
8816	0.263	-0.120	0.185	0.149	-0.882	0.193	0.361	0.504	0.185
8817	0.466	1.178	0.450	0.421	0.869	0.462	0.508	1.447	0.481
8818	0.476	1.245	0.296	0.500	1.378	0.478	0.492	1.346	0.492
8819	0.332	0.318	0.262	0.307	0.135	0.508	0.142	-0.895	0.481
8820	0.513	1.481	0.512	0.485	1.283	0.462	0.503	1.413	0.481
8821	0.358	0.487	0.262	0.512	1.453	0.491	0.503	1.413	0.492
8822	0.482	1.279	0.262	0.509	1.434	0.444	0.450	1.076	0.481
8823	0.511	1.464	0.512	0.488	1.302	0.444	0.495	1.363	0.481
8824	0.297	0.099	0.224	0.053	-1.504	0.422	0.184	-0.625	0.262
8825	0.466	1.178	0.409	0.500	1.378	0.102	0.505	1.430	0.409
8826	0.263	-0.120	0.481	0.149	-0.882	0.501	0.142	-0.895	0.097
8827	0.224	-0.373	0.409	0.234	-0.336	0.341	0.261	-0.137	0.481
8828	0.326	0.285	0.385	0.275	-0.073	0.235	0.142	-0.895	0.481
8829	0.466	1.178	0.512	0.480	1.246	0.053	0.461	1.144	0.142
8830	0.184	-0.625	0.508	0.196	-0.581	0.514	0.184	-0.625	0.501
8831	0.184	-0.625	0.501	0.196	-0.581	0.514	0.184	-0.625	0.467
8832	0.503	1.413	0.431	0.456	1.095	0.491	0.503	1.413	0.142
8833	0.405	0.790	0.296	0.477	1.227	0.149	0.447	1.060	0.501
8834	0.513	1.481	0.185	0.506	1.415	0.193	0.426	0.925	0.467
8835	0.463	1.161	0.185	0.459	1.114	0.478	0.508	1.447	0.185
8836	0.466	1.178	0.224	0.491	1.321	0.235	0.476	1.245	0.492
8837	0.476	1.245	0.512	0.444	1.020	0.193	0.492	1.346	0.512
8838	0.476	1.245	0.501	0.444	1.020	0.193	0.492	1.346	0.450
8839	0.497	1.380	0.262	0.421	0.869	0.501	0.479	1.262	0.467
8840	0.226	-0.356	0.492	0.102	-1.184	0.508	0.329	0.301	0.467
8841	0.505	1.430	0.492	0.500	1.378	0.273	0.497	1.380	0.492

8842	0.142	-0.895	0.450	0.342	0.361	0.508	0.226	-0.356	0.296
8843	0.224	-0.373	0.185	0.234	-0.336	0.508	0.329	0.301	0.296
8844	0.263	-0.120	0.185	0.053	-1.504	0.491	0.263	-0.120	0.501
8845	0.429	0.942	0.512	0.518	1.491	0.235	0.426	0.925	0.097
8846	0.429	0.942	0.224	0.518	1.491	0.309	0.426	0.925	0.431
8847	0.513	1.481	0.224	0.494	1.340	0.273	0.408	0.807	0.467
8848	0.295	0.082	0.224	0.152	-0.863	0.462	0.097	-1.181	0.097
8849	0.361	0.504	0.328	0.193	-0.600	0.444	0.224	-0.373	0.097
8850	0.432	0.958	0.185	0.480	1.246	0.341	0.479	1.262	0.492
8851	0.224	-0.373	0.492	0.237	-0.317	0.444	0.184	-0.625	0.224
8852	0.187	-0.608	0.508	0.193	-0.600	0.149	0.224	-0.373	0.385
8853	0.142	-0.895	0.142	0.196	-0.581	0.309	0.224	-0.373	0.431
8854	0.450	1.076	0.142	0.500	1.378	0.273	0.387	0.672	0.450
8855	0.482	1.279	0.492	0.503	1.396	0.273	0.489	1.329	0.492
8856	0.097	-1.181	0.512	0.272	-0.091	0.398	0.224	-0.373	0.431
8857	0.492	1.346	0.513	0.506	1.415	0.478	0.489	1.329	0.508
8858	0.479	1.262	0.050	0.506	1.415	0.193	0.489	1.329	0.492
8859	0.479	1.262	0.492	0.491	1.321	0.462	0.479	1.262	0.481
8860	0.184	-0.625	0.224	0.234	-0.336	0.444	0.142	-0.895	0.142
8861	0.326	0.285	0.385	0.310	0.153	0.273	0.358	0.487	0.185
8862	0.097	-1.181	0.224	0.275	-0.073	0.273	0.332	0.318	0.185
8863	0.503	1.413	0.185	0.459	1.114	0.371	0.487	1.312	0.296
8864	0.503	1.413	0.508	0.442	1.001	0.371	0.511	1.464	0.508
8865	0.097	-1.181	0.185	0.342	0.361	0.508	0.142	-0.895	0.492
8866	0.511	1.464	0.501	0.442	1.001	0.371	0.511	1.464	0.328
8867	0.261	-0.137	0.501	0.149	-0.882	0.508	0.184	-0.625	0.501
8868	0.184	-0.625	0.492	0.310	0.153	0.273	0.097	-1.181	0.501
8869	0.142	-0.895	0.142	0.278	-0.054	0.478	0.142	-0.895	0.513
8870	0.145	-0.878	0.142	0.275	-0.073	0.491	0.184	-0.625	0.185
8871	0.184	-0.625	0.296	0.401	0.737	0.341	0.224	-0.373	0.296
8872	0.450	1.076	0.513	0.477	1.227	0.341	0.405	0.790	0.501
8873	0.097	-1.181	0.185	0.193	-0.600	0.235	0.297	0.099	0.097
8874	0.495	1.363	0.262	0.459	1.114	0.444	0.511	1.464	0.409
8875	0.503	1.413	0.224	0.444	1.020	0.491	0.487	1.312	0.185
8876	0.184	-0.625	0.481	0.272	-0.091	0.478	0.184	-0.625	0.328
8877	0.184	-0.625	0.185	0.272	-0.091	0.512	0.184	-0.625	0.328
8878	0.145	-0.878	0.224	0.374	0.568	0.491	0.224	-0.373	0.501
8879	0.261	-0.137	0.501	0.374	0.568	0.501	0.097	-1.181	0.492
8880	0.450	1.076	0.492	0.509	1.434	0.501	0.426	0.925	0.224
8881	0.387	0.672	0.185	0.374	0.568	0.501	0.224	-0.373	0.224
8882	0.450	1.076	0.262	0.509	1.434	0.273	0.474	1.228	0.492
8883	0.226	-0.356	0.481	0.272	-0.091	0.478	0.097	-1.181	0.385
8884	0.505	1.430	0.185	0.482	1.265	0.512	0.484	1.295	0.467
8885	0.505	1.430	0.097	0.491	1.321	0.501	0.505	1.430	0.224
8886	0.224	-0.373	0.097	0.342	0.361	0.501	0.100	-1.164	0.492
8887	0.261	-0.137	0.142	0.339	0.342	0.491	0.097	-1.181	0.097
8888	0.263	-0.120	0.431	0.234	-0.336	0.444	0.097	-1.181	0.185
8889	0.487	1.312	0.097	0.444	1.020	0.444	0.463	1.161	0.097
8890	0.413	0.841	0.508	0.491	1.321	0.273	0.505	1.430	0.296
8891	0.508	1.447	0.492	0.477	1.227	0.478	0.447	1.060	0.481
8892	0.450	1.076	0.385	0.512	1.453	0.193	0.476	1.245	0.142
8893	0.479	1.262	0.358	0.491	1.321	0.514	0.482	1.279	0.481
8894	0.476	1.245	0.492	0.497	1.359	0.514	0.479	1.262	0.512
8895	0.476	1.245	0.481	0.497	1.359	0.149	0.479	1.262	0.501
8896	0.476	1.245	0.492	0.497	1.359	0.501	0.479	1.262	0.296
8897	0.145	-0.878	0.481	0.272	-0.091	0.501	0.187	-0.608	0.296
8898	0.489	1.329	0.224	0.477	1.227	0.193	0.479	1.262	0.185
8899	0.463	1.161	0.224	0.509	1.434	0.478	0.489	1.329	0.513
8900	0.445	1.043	0.481	0.500	1.378	0.508	0.479	1.262	0.262
8901	0.445	1.043	0.481	0.500	1.378	0.193	0.479	1.262	0.296
8902	0.445	1.043	0.142	0.491	1.321	0.491	0.489	1.329	0.185

8903	0.516	1.498	0.431	0.444	1.020	0.501	0.474	1.228	0.467
8904	0.495	1.363	0.409	0.444	1.020	0.512	0.474	1.228	0.097
8905	0.224	-0.373	0.501	0.272	-0.091	0.491	0.263	-0.120	0.224
8906	0.508	1.447	0.328	0.474	1.208	0.491	0.408	0.807	0.481
8907	0.297	0.099	0.481	0.193	-0.600	0.508	0.097	-1.181	0.450
8908	0.497	1.380	0.450	0.509	1.434	0.193	0.479	1.262	0.224
8909	0.489	1.329	0.185	0.509	1.434	0.053	0.479	1.262	0.050
8910	0.358	0.487	0.501	0.149	-0.882	0.444	0.142	-0.895	0.501
8911	0.432	0.958	0.492	0.503	1.396	0.273	0.495	1.363	0.262
8912	0.505	1.430	0.492	0.503	1.396	0.309	0.461	1.144	0.185
8913	0.384	0.655	0.492	0.193	-0.600	0.491	0.145	-0.878	0.185
8914	0.508	1.447	0.262	0.477	1.227	0.193	0.500	1.397	0.185
8915	0.466	1.178	0.501	0.509	1.434	0.193	0.463	1.161	0.501
8916	0.297	0.099	0.385	0.193	-0.600	0.444	0.184	-0.625	0.358
8917	0.463	1.161	0.358	0.494	1.340	0.149	0.489	1.329	0.481
8918	0.482	1.279	0.467	0.497	1.359	0.235	0.508	1.447	0.492
8919	0.508	1.447	0.431	0.515	1.472	0.478	0.447	1.060	0.224
8920	0.482	1.279	0.508	0.488	1.302	0.501	0.468	1.194	0.409
8921	0.482	1.279	0.508	0.488	1.302	0.491	0.468	1.194	0.481
8922	0.492	1.346	0.512	0.506	1.415	0.512	0.495	1.363	0.513
8923	0.224	-0.373	0.501	0.193	-0.600	0.462	0.295	0.082	0.512
8924	0.189	-0.592	0.467	0.053	-1.504	0.512	0.295	0.082	0.481
8925	0.450	1.076	0.508	0.444	1.020	0.508	0.503	1.413	0.097
8926	0.295	0.082	0.450	0.275	-0.073	0.149	0.097	-1.181	0.142
8927	0.263	-0.120	0.481	0.313	0.172	0.193	0.429	0.942	0.481
8928	0.511	1.464	0.450	0.497	1.359	0.193	0.471	1.211	0.492
8929	0.261	-0.137	0.512	0.193	-0.600	0.193	0.097	-1.181	0.328
8930	0.261	-0.137	0.512	0.193	-0.600	0.273	0.097	-1.181	0.467
8931	0.508	1.447	0.185	0.442	1.001	0.508	0.495	1.363	0.512
8932	0.224	-0.373	0.508	0.149	-0.882	0.422	0.224	-0.373	0.467
8933	0.413	0.841	0.492	0.234	-0.336	0.462	0.387	0.672	0.262
8934	0.476	1.245	0.513	0.480	1.246	0.501	0.426	0.925	0.450
8935	0.408	0.807	0.185	0.503	1.396	0.491	0.445	1.043	0.492
8936	0.382	0.638	0.481	0.494	1.340	0.193	0.489	1.329	0.224
8937	0.511	1.464	0.409	0.515	1.472	0.193	0.429	0.942	0.467
8938	0.505	1.430	0.501	0.465	1.152	0.512	0.508	1.447	0.492
8939	0.495	1.363	0.501	0.515	1.472	0.235	0.487	1.312	0.492
8940	0.429	0.942	0.481	0.509	1.434	0.235	0.482	1.279	0.450
8941	0.295	0.082	0.492	0.149	-0.882	0.193	0.142	-0.895	0.358
8942	0.187	-0.608	0.492	0.193	-0.600	0.149	0.187	-0.608	0.508
8943	0.187	-0.608	0.508	0.193	-0.600	0.341	0.187	-0.608	0.224
8944	0.229	-0.339	0.185	0.193	-0.600	0.501	0.295	0.082	0.097
8945	0.511	1.464	0.142	0.275	-0.073	0.462	0.511	1.464	0.492
8946	0.497	1.380	0.142	0.512	1.453	0.273	0.495	1.363	0.481
8947	0.263	-0.120	0.492	0.424	0.888	0.273	0.329	0.301	0.409
8948	0.489	1.329	0.512	0.462	1.133	0.514	0.503	1.413	0.492
8949	0.492	1.346	0.409	0.503	1.396	0.491	0.500	1.397	0.501
8950	0.450	1.076	0.409	0.491	1.321	0.508	0.513	1.481	0.501
8951	0.184	-0.625	0.185	0.193	-0.600	0.422	0.184	-0.625	0.492
8952	0.187	-0.608	0.501	0.193	-0.600	0.512	0.295	0.082	0.467
8953	0.511	1.464	0.185	0.518	1.491	0.273	0.503	1.413	0.481
8954	0.224	-0.373	0.450	0.237	-0.317	0.371	0.100	-1.164	0.481
8955	0.224	-0.373	0.481	0.237	-0.317	0.273	0.408	0.807	0.512
8956	0.226	-0.356	0.224	0.193	-0.600	0.102	0.184	-0.625	0.492
8957	0.326	0.285	0.409	0.152	-0.863	0.508	0.332	0.318	0.492
8958	0.184	-0.625	0.050	0.345	0.379	0.235	0.329	0.301	0.224
8959	0.487	1.312	0.296	0.506	1.415	0.478	0.500	1.397	0.512
8960	0.503	1.413	0.224	0.462	1.133	0.462	0.492	1.346	0.512
8961	0.142	-0.895	0.224	0.278	-0.054	0.491	0.224	-0.373	0.512
8962	0.142	-0.895	0.385	0.278	-0.054	0.309	0.224	-0.373	0.224
8963	0.492	1.346	0.492	0.512	1.453	0.309	0.484	1.295	0.450

8964	0.505	1.430	0.185	0.491	1.321	0.398	0.382	0.638	0.501
8965	0.511	1.464	0.450	0.506	1.415	0.398	0.461	1.144	0.501
8966	0.050	-1.485	0.262	0.424	0.888	0.149	0.224	-0.373	0.501
8967	0.487	1.312	0.513	0.509	1.434	0.309	0.492	1.346	0.508
8968	0.224	-0.373	0.513	0.275	-0.073	0.235	0.097	-1.181	0.431
8969	0.382	0.638	0.508	0.371	0.549	0.512	0.187	-0.608	0.508
8970	0.224	-0.373	0.467	0.275	-0.073	0.273	0.097	-1.181	0.508
8971	0.184	-0.625	0.185	0.102	-1.184	0.273	0.295	0.082	0.262
8972	0.513	1.481	0.185	0.512	1.453	0.478	0.482	1.279	0.328
8973	0.184	-0.625	0.467	0.234	-0.336	0.478	0.145	-0.878	0.328
8974	0.497	1.380	0.262	0.474	1.208	0.235	0.479	1.262	0.492
8975	0.500	1.397	0.296	0.462	1.133	0.235	0.508	1.447	0.501
8976	0.489	1.329	0.467	0.497	1.359	0.508	0.505	1.430	0.513
8977	0.142	-0.895	0.508	0.310	0.153	0.193	0.297	0.099	0.512
8978	0.142	-0.895	0.328	0.310	0.153	0.341	0.297	0.099	0.185
8979	0.300	0.116	0.224	0.398	0.718	0.341	0.187	-0.608	0.431
8980	0.516	1.498	0.185	0.401	0.737	0.341	0.516	1.498	0.224
8981	0.187	-0.608	0.328	0.149	-0.882	0.444	0.261	-0.137	0.467
8982	0.263	-0.120	0.224	0.310	0.153	0.193	0.297	0.099	0.492
8983	0.226	-0.356	0.501	0.234	-0.336	0.462	0.184	-0.625	0.262
8984	0.479	1.262	0.185	0.509	1.434	0.478	0.466	1.178	0.481
8985	0.184	-0.625	0.512	0.272	-0.091	0.341	0.097	-1.181	0.431
8986	0.224	-0.373	0.262	0.272	-0.091	0.273	0.226	-0.356	0.262
8987	0.497	1.380	0.358	0.477	1.227	0.508	0.482	1.279	0.185
8988	0.487	1.312	0.185	0.480	1.246	0.478	0.447	1.060	0.185
8989	0.184	-0.625	0.501	0.237	-0.317	0.478	0.224	-0.373	0.142
8990	0.261	-0.137	0.224	0.240	-0.299	0.341	0.050	-1.485	0.508
8991	0.482	1.279	0.050	0.506	1.415	0.273	0.503	1.413	0.142
8992	0.184	-0.625	0.050	0.193	-0.600	0.235	0.261	-0.137	0.501
8993	0.097	-1.181	0.513	0.342	0.361	0.501	0.187	-0.608	0.296
8994	0.097	-1.181	0.224	0.342	0.361	0.444	0.187	-0.608	0.450
8995	0.142	-0.895	0.492	0.342	0.361	0.102	0.184	-0.625	0.450
8996	0.429	0.942	0.501	0.444	1.020	0.462	0.500	1.397	0.431
8997	0.097	-1.181	0.296	0.193	-0.600	0.512	0.358	0.487	0.508
8998	0.505	1.430	0.508	0.462	1.133	0.512	0.484	1.295	0.262
8999	0.497	1.380	0.501	0.480	1.246	0.398	0.484	1.295	0.262
9000	0.384	0.655	0.501	0.339	0.342	0.512	0.226	-0.356	0.492
9001	0.361	0.504	0.262	0.272	-0.091	0.508	0.411	0.824	0.142
9002	0.489	1.329	0.431	0.509	1.434	0.193	0.474	1.228	0.097
9003	0.484	1.295	0.508	0.480	1.246	0.491	0.508	1.447	0.492
9004	0.495	1.363	0.224	0.480	1.246	0.491	0.508	1.447	0.512
9005	0.479	1.262	0.224	0.339	0.342	0.491	0.476	1.245	0.185
9006	0.224	-0.373	0.224	0.278	-0.054	0.491	0.097	-1.181	0.224
9007	0.226	-0.356	0.262	0.234	-0.336	0.309	0.142	-0.895	0.224
9008	0.479	1.262	0.185	0.497	1.359	0.398	0.476	1.245	0.224
9009	0.476	1.245	0.481	0.447	1.039	0.149	0.495	1.363	0.508
9010	0.142	-0.895	0.185	0.102	-1.184	0.309	0.329	0.301	0.097
9011	0.434	0.975	0.185	0.465	1.152	0.508	0.468	1.194	0.512
9012	0.411	0.824	0.262	0.515	1.472	0.491	0.508	1.447	0.097
9013	0.495	1.363	0.467	0.523	1.528	0.512	0.466	1.178	0.185
9014	0.329	0.301	0.296	0.401	0.737	0.514	0.261	-0.137	0.224
9015	0.476	1.245	0.224	0.518	1.491	0.512	0.453	1.093	0.467
9016	0.447	1.060	0.492	0.509	1.434	0.508	0.487	1.312	0.262
9017	0.184	-0.625	0.492	0.193	-0.600	0.491	0.224	-0.373	0.358
9018	0.503	1.413	0.481	0.494	1.340	0.514	0.463	1.161	0.358
9019	0.495	1.363	0.481	0.494	1.340	0.478	0.489	1.329	0.481
9020	0.495	1.363	0.185	0.494	1.340	0.501	0.489	1.329	0.185
9021	0.495	1.363	0.262	0.494	1.340	0.462	0.450	1.076	0.385
9022	0.261	-0.137	0.501	0.310	0.153	0.478	0.358	0.487	0.508
9023	0.500	1.397	0.501	0.395	0.700	0.478	0.508	1.447	0.097
9024	0.387	0.672	0.481	0.152	-0.863	0.235	0.224	-0.373	0.513

9025	0.358	0.487	0.224	0.310	0.153	0.444	0.097	-1.181	0.385
9026	0.468	1.194	0.512	0.512	1.453	0.501	0.489	1.329	0.385
9027	0.426	0.925	0.409	0.491	1.321	0.512	0.479	1.262	0.185
9028	0.500	1.397	0.481	0.506	1.415	0.149	0.413	0.841	0.492
9029	0.505	1.430	0.097	0.515	1.472	0.501	0.489	1.329	0.513
9030	0.508	1.447	0.450	0.512	1.453	0.501	0.497	1.380	0.097
9031	0.503	1.413	0.501	0.506	1.415	0.491	0.497	1.380	0.097
9032	0.463	1.161	0.328	0.488	1.302	0.462	0.492	1.346	0.097
9033	0.505	1.430	0.262	0.512	1.453	0.491	0.466	1.178	0.358
9034	0.445	1.043	0.185	0.480	1.246	0.512	0.484	1.295	0.097
9035	0.479	1.262	0.385	0.500	1.378	0.514	0.479	1.262	0.508
9036	0.447	1.060	0.328	0.462	1.133	0.508	0.513	1.481	0.328
9037	0.511	1.464	0.431	0.477	1.227	0.193	0.497	1.380	0.224
9038	0.511	1.464	0.508	0.477	1.227	0.193	0.497	1.380	0.097
9039	0.184	-0.625	0.431	0.234	-0.336	0.193	0.224	-0.373	0.467
9040	0.508	1.447	0.296	0.447	1.039	0.512	0.511	1.464	0.097
9041	0.489	1.329	0.296	0.500	1.378	0.508	0.508	1.447	0.097
9042	0.513	1.481	0.185	0.509	1.434	0.508	0.508	1.447	0.467
9043	0.184	-0.625	0.431	0.149	-0.882	0.508	0.226	-0.356	0.450
9044	0.479	1.262	0.224	0.503	1.396	0.273	0.453	1.093	0.481
9045	0.408	0.807	0.431	0.506	1.415	0.512	0.505	1.430	0.508
9046	0.500	1.397	0.409	0.494	1.340	0.193	0.500	1.397	0.262
9047	0.500	1.397	0.224	0.465	1.152	0.501	0.503	1.413	0.050
9048	0.479	1.262	0.185	0.494	1.340	0.444	0.508	1.447	0.450
9049	0.489	1.329	0.512	0.512	1.453	0.371	0.429	0.942	0.409
9050	0.489	1.329	0.296	0.512	1.453	0.508	0.503	1.413	0.467
9051	0.505	1.430	0.262	0.506	1.415	0.193	0.503	1.413	0.185
9052	0.184	-0.625	0.501	0.193	-0.600	0.149	0.263	-0.120	0.409
9053	0.142	-0.895	0.508	0.193	-0.600	0.193	0.326	0.285	0.492
9054	0.142	-0.895	0.262	0.193	-0.600	0.149	0.326	0.285	0.467
9055	0.489	1.329	0.501	0.512	1.453	0.422	0.489	1.329	0.358
9056	0.505	1.430	0.467	0.509	1.434	0.501	0.500	1.397	0.512
9057	0.408	0.807	0.431	0.506	1.415	0.235	0.513	1.481	0.409
9058	0.408	0.807	0.262	0.506	1.415	0.501	0.511	1.464	0.358
9059	0.184	-0.625	0.513	0.275	-0.073	0.149	0.184	-0.625	0.296
9060	0.503	1.413	0.262	0.512	1.453	0.501	0.434	0.975	0.142
9061	0.187	-0.608	0.224	0.193	-0.600	0.501	0.226	-0.356	0.512
9062	0.455	1.110	0.050	0.497	1.359	0.478	0.474	1.228	0.050
9063	0.487	1.312	0.097	0.444	1.020	0.398	0.487	1.312	0.492
9064	0.226	-0.356	0.224	0.374	0.568	0.309	0.261	-0.137	0.512
9065	0.411	0.824	0.142	0.512	1.453	0.341	0.479	1.262	0.481
9066	0.050	-1.485	0.492	0.196	-0.581	0.514	0.432	0.958	0.097
9067	0.295	0.082	0.513	0.149	-0.882	0.149	0.263	-0.120	0.142
9068	0.224	-0.373	0.262	0.193	-0.600	0.193	0.184	-0.625	0.185
9069	0.224	-0.373	0.512	0.149	-0.882	0.478	0.184	-0.625	0.097
9070	0.387	0.672	0.512	0.424	0.888	0.462	0.142	-0.895	0.097
9071	0.492	1.346	0.431	0.497	1.359	0.193	0.503	1.413	0.508
9072	0.184	-0.625	0.501	0.237	-0.317	0.341	0.142	-0.895	0.492
9073	0.447	1.060	0.142	0.500	1.378	0.193	0.497	1.380	0.142
9074	0.263	-0.120	0.142	0.152	-0.863	0.149	0.297	0.099	0.185
9075	0.516	1.498	0.262	0.503	1.396	0.235	0.450	1.076	0.492
9076	0.516	1.498	0.481	0.503	1.396	0.501	0.450	1.076	0.097
9077	0.500	1.397	0.185	0.480	1.246	0.309	0.429	0.942	0.262
9078	0.463	1.161	0.262	0.398	0.718	0.422	0.511	1.464	0.467
9079	0.184	-0.625	0.262	0.310	0.153	0.193	0.263	-0.120	0.501
9080	0.184	-0.625	0.512	0.342	0.361	0.149	0.261	-0.137	0.097
9081	0.466	1.178	0.185	0.509	1.434	0.235	0.492	1.346	0.431
9082	0.263	-0.120	0.185	0.149	-0.882	0.478	0.142	-0.895	0.481
9083	0.295	0.082	0.328	0.193	-0.600	0.398	0.097	-1.181	0.450
9084	0.468	1.194	0.328	0.477	1.227	0.398	0.487	1.312	0.262
9085	0.508	1.447	0.185	0.462	1.133	0.341	0.505	1.430	0.492

9086	0.326	0.285	0.492	0.193	-0.600	0.462	0.000	?	0.097
9087	0.224	-0.373	0.142	0.345	0.379	0.193	0.184	-0.625	0.296
9088	0.184	-0.625	0.262	0.193	-0.600	0.514	0.224	-0.373	0.385
9089	0.332	0.318	0.262	0.152	-0.863	0.371	0.224	-0.373	0.262
9090	0.226	-0.356	0.262	0.237	-0.317	0.149	0.224	-0.373	0.185
9091	0.500	1.397	0.358	0.500	1.378	0.444	0.503	1.413	0.142
9092	0.187	-0.608	0.142	0.310	0.153	0.478	0.097	-1.181	0.450
9093	0.508	1.447	0.501	0.421	0.869	0.444	0.505	1.430	0.450
9094	0.263	-0.120	0.492	0.193	-0.600	0.235	0.097	-1.181	0.501
9095	0.363	0.520	0.185	0.149	-0.882	0.491	0.184	-0.625	0.501
9096	0.184	-0.625	0.328	0.237	-0.317	0.444	0.224	-0.373	0.508
9097	0.500	1.397	0.224	0.477	1.227	0.273	0.461	1.144	0.431
9098	0.224	-0.373	0.492	0.401	0.737	0.235	0.263	-0.120	0.185
9099	0.050	-1.485	0.185	0.401	0.737	0.309	0.355	0.470	0.185
9100	0.050	-1.485	0.224	0.342	0.361	0.371	0.355	0.470	0.097
9101	0.508	1.447	0.467	0.462	1.133	0.273	0.476	1.245	0.492
9102	0.224	-0.373	0.262	0.196	-0.581	0.478	0.187	-0.608	0.224
9103	0.489	1.329	0.224	0.509	1.434	0.149	0.387	0.672	0.142
9104	0.505	1.430	0.185	0.374	0.568	0.149	0.503	1.413	0.097
9105	0.295	0.082	0.481	0.152	-0.863	0.193	0.097	-1.181	0.492
9106	0.505	1.430	0.492	0.442	1.001	0.478	0.508	1.447	0.142
9107	0.503	1.413	0.224	0.477	1.227	0.149	0.384	0.655	0.224
9108	0.503	1.413	0.508	0.442	1.001	0.309	0.384	0.655	0.097
9109	0.261	-0.137	0.467	0.237	-0.317	0.508	0.184	-0.625	0.142
9110	0.429	0.942	0.501	0.491	1.321	0.491	0.489	1.329	0.224
9111	0.500	1.397	0.185	0.444	1.020	0.491	0.511	1.464	0.492
9112	0.224	-0.373	0.492	0.272	-0.091	0.478	0.097	-1.181	0.097
9113	0.224	-0.373	0.492	0.234	-0.336	0.371	0.097	-1.181	0.142
9114	0.224	-0.373	0.328	0.310	0.153	0.273	0.097	-1.181	0.142
9115	0.261	-0.137	0.185	0.371	0.549	0.512	0.363	0.520	0.142
9116	0.184	-0.625	0.296	0.275	-0.073	0.462	0.097	-1.181	0.050
9117	0.482	1.279	0.185	0.480	1.246	0.398	0.511	1.464	0.224
9118	0.187	-0.608	0.262	0.149	-0.882	0.398	0.329	0.301	0.512
9119	0.187	-0.608	0.481	0.149	-0.882	0.512	0.226	-0.356	0.492
9120	0.261	-0.137	0.508	0.196	-0.581	0.478	0.097	-1.181	0.185
9121	0.466	1.178	0.224	0.477	1.227	0.478	0.463	1.161	0.142
9122	0.297	0.099	0.409	0.149	-0.882	0.102	0.097	-1.181	0.296
9123	0.226	-0.356	0.409	0.310	0.153	0.462	0.097	-1.181	0.508
9124	0.489	1.329	0.185	0.512	1.453	0.512	0.463	1.161	0.142
9125	0.489	1.329	0.262	0.485	1.283	0.273	0.447	1.060	0.224
9126	0.479	1.262	0.481	0.491	1.321	0.149	0.476	1.245	0.508
9127	0.482	1.279	0.508	0.474	1.208	0.235	0.503	1.413	0.185
9128	0.187	-0.608	0.501	0.371	0.549	0.478	0.261	-0.137	0.185
9129	0.261	-0.137	0.450	0.275	-0.073	0.149	0.050	-1.485	0.262
9130	0.503	1.413	0.409	0.512	1.453	0.462	0.445	1.043	0.501
9131	0.505	1.430	0.512	0.465	1.152	0.491	0.405	0.790	0.492
9132	0.484	1.295	0.185	0.401	0.737	0.508	0.463	1.161	0.142
9133	0.224	-0.373	0.224	0.401	0.737	0.309	0.187	-0.608	0.450
9134	0.511	1.464	0.501	0.515	1.472	0.235	0.408	0.807	0.431
9135	0.411	0.824	0.501	0.477	1.227	0.273	0.484	1.295	0.358
9136	0.479	1.262	0.385	0.477	1.227	0.193	0.466	1.178	0.050
9137	0.097	-1.181	0.501	0.102	-1.184	0.235	0.358	0.487	0.512
9138	0.447	1.060	0.142	0.465	1.152	0.512	0.511	1.464	0.431
9139	0.495	1.363	0.224	0.515	1.472	0.501	0.405	0.790	0.097
9140	0.329	0.301	0.501	0.272	-0.091	0.235	0.358	0.487	0.224
9141	0.261	-0.137	0.501	0.152	-0.863	0.193	0.295	0.082	0.296
9142	0.184	-0.625	0.501	0.234	-0.336	0.422	0.142	-0.895	0.050
9143	0.384	0.655	0.097	0.477	1.227	0.149	0.505	1.430	0.481
9144	0.332	0.318	0.296	0.149	-0.882	0.149	0.050	-1.485	0.492
9145	0.432	0.958	0.224	0.459	1.114	0.422	0.487	1.312	0.142
9146	0.508	1.447	0.224	0.491	1.321	0.462	0.503	1.413	0.224

9147	0.432	0.958	0.328	0.506	1.415	0.193	0.479	1.262	0.508
9148	0.295	0.082	0.450	0.307	0.135	0.512	0.097	-1.181	0.185
9149	0.297	0.099	0.296	0.234	-0.336	0.478	0.142	-0.895	0.224
9150	0.184	-0.625	0.262	0.272	-0.091	0.422	0.184	-0.625	0.492
9151	0.432	0.958	0.358	0.196	-0.581	0.273	0.097	-1.181	0.467
9152	0.224	-0.373	0.097	0.234	-0.336	0.491	0.097	-1.181	0.492
9153	0.432	0.958	0.467	0.512	1.453	0.235	0.500	1.397	0.508
9154	0.411	0.824	0.481	0.497	1.359	0.309	0.487	1.312	0.097
9155	0.224	-0.373	0.450	0.234	-0.336	0.193	0.142	-0.895	0.409
9156	0.187	-0.608	0.185	0.193	-0.600	0.053	0.184	-0.625	0.296
9157	0.511	1.464	0.142	0.418	0.850	0.235	0.487	1.312	0.296
9158	0.300	0.116	0.224	0.149	-0.882	0.341	0.097	-1.181	0.450
9159	0.261	-0.137	0.481	0.149	-0.882	0.309	0.261	-0.137	0.512
9160	0.500	1.397	0.492	0.424	0.888	0.444	0.466	1.178	0.481
9161	0.513	1.481	0.501	0.456	1.095	0.444	0.492	1.346	0.450
9162	0.263	-0.120	0.224	0.196	-0.581	0.273	0.097	-1.181	0.142
9163	0.500	1.397	0.224	0.509	1.434	0.491	0.426	0.925	0.185
9164	0.466	1.178	0.262	0.477	1.227	0.491	0.476	1.245	0.409
9165	0.432	0.958	0.512	0.424	0.888	0.491	0.445	1.043	0.409
9166	0.261	-0.137	0.185	0.275	-0.073	0.501	0.266	-0.103	0.409
9167	0.511	1.464	0.262	0.488	1.302	0.235	0.487	1.312	0.224
9168	0.263	-0.120	0.481	0.234	-0.336	0.371	0.097	-1.181	0.050
9169	0.226	-0.356	0.450	0.310	0.153	0.273	0.000	?	0.097
9170	0.050	-1.485	0.296	0.193	-0.600	0.491	0.300	0.116	0.097
9171	0.097	-1.181	0.224	0.053	-1.504	0.193	0.384	0.655	0.224
9172	0.224	-0.373	0.385	0.234	-0.336	0.235	0.263	-0.120	0.450
9173	0.000	?	0.328	0.345	0.379	0.193	0.184	-0.625	0.097
9174	0.142	-0.895	0.467	0.313	0.172	0.501	0.142	-0.895	0.097
9175	0.489	1.329	0.262	0.442	1.001	0.273	0.447	1.060	0.097
9176	0.508	1.447	0.328	0.442	1.001	0.309	0.447	1.060	0.385
9177	0.263	-0.120	0.224	0.272	-0.091	0.309	0.000	?	0.492
9178	0.511	1.464	0.185	0.485	1.283	0.309	0.497	1.380	0.492
9179	0.511	1.464	0.431	0.485	1.283	0.309	0.497	1.380	0.512
9180	0.432	0.958	0.224	0.491	1.321	0.512	0.503	1.413	0.185
9181	0.500	1.397	0.224	0.500	1.378	0.309	0.429	0.942	0.224
9182	0.142	-0.895	0.450	0.237	-0.317	0.273	0.184	-0.625	0.262
9183	0.142	-0.895	0.296	0.374	0.568	0.273	0.184	-0.625	0.431
9184	0.261	-0.137	0.296	0.275	-0.073	0.273	0.100	-1.164	0.508
9185	0.484	1.295	0.262	0.494	1.340	0.102	0.489	1.329	0.492
9186	0.187	-0.608	0.097	0.196	-0.581	0.273	0.224	-0.373	0.050
9187	0.263	-0.120	0.097	0.234	-0.336	0.462	0.142	-0.895	0.142
9188	0.263	-0.120	0.450	0.196	-0.581	0.462	0.097	-1.181	0.185
9189	0.505	1.430	0.508	0.497	1.359	0.341	0.492	1.346	0.467
9190	0.184	-0.625	0.431	0.272	-0.091	0.102	0.142	-0.895	0.097
9191	0.184	-0.625	0.224	0.310	0.153	0.273	0.224	-0.373	0.097
9192	0.329	0.301	0.512	0.313	0.172	0.512	0.097	-1.181	0.481
9193	0.329	0.301	0.358	0.313	0.172	0.235	0.142	-0.895	0.492
9194	0.184	-0.625	0.262	0.310	0.153	0.102	0.224	-0.373	0.185
9195	0.495	1.363	0.492	0.512	1.453	0.501	0.487	1.312	0.050
9196	0.145	-0.878	0.492	0.310	0.153	0.309	0.097	-1.181	0.508
9197	0.263	-0.120	0.224	0.272	-0.091	0.309	0.142	-0.895	0.097
9198	0.263	-0.120	0.467	0.272	-0.091	0.149	0.142	-0.895	0.513
9199	0.263	-0.120	0.492	0.272	-0.091	0.512	0.142	-0.895	0.050
9200	0.358	0.487	0.501	0.102	-1.184	0.512	0.050	-1.485	0.050
9201	0.142	-0.895	0.185	0.272	-0.091	0.235	0.226	-0.356	0.050
9202	0.500	1.397	0.328	0.462	1.133	0.422	0.505	1.430	0.050
9203	0.497	1.380	0.385	0.465	1.152	0.512	0.487	1.312	0.508
9204	0.184	-0.625	0.431	0.348	0.398	0.478	0.184	-0.625	0.142
9205	0.332	0.318	0.328	0.102	-1.184	0.309	0.142	-0.895	0.097
9206	0.226	-0.356	0.481	0.275	-0.073	0.508	0.297	0.099	0.481
9207	0.492	1.346	0.224	0.518	1.491	0.508	0.497	1.380	0.050

9208	0.184	-0.625	0.262	0.237	-0.317	0.102	0.142	-0.895	0.142
9209	0.226	-0.356	0.296	0.102	-1.184	0.149	0.226	-0.356	0.050
9210	0.466	1.178	0.450	0.500	1.378	0.235	0.500	1.397	0.142
9211	0.266	-0.103	0.262	0.310	0.153	0.235	0.184	-0.625	0.142
9212	0.226	-0.356	0.481	0.310	0.153	0.309	0.187	-0.608	0.450
9213	0.184	-0.625	0.224	0.149	-0.882	0.508	0.261	-0.137	0.481
9214	0.482	1.279	0.224	0.512	1.453	0.462	0.500	1.397	0.513
9215	0.492	1.346	0.224	0.512	1.453	0.309	0.492	1.346	0.142
9216	0.224	-0.373	0.296	0.237	-0.317	0.193	0.142	-0.895	0.508
9217	0.511	1.464	0.224	0.418	0.850	0.478	0.455	1.110	0.050
9218	0.468	1.194	0.450	0.512	1.453	0.235	0.429	0.942	0.142
9219	0.505	1.430	0.142	0.477	1.227	0.102	0.358	0.487	0.512
9220	0.184	-0.625	0.097	0.307	0.135	0.508	0.050	-1.485	0.512
9221	0.492	1.346	0.492	0.512	1.453	0.512	0.505	1.430	0.050
9222	0.489	1.329	0.492	0.506	1.415	0.512	0.426	0.925	0.467
9223	0.329	0.301	0.296	0.102	-1.184	0.462	0.097	-1.181	0.467
9224	0.184	-0.625	0.296	0.149	-0.882	0.341	0.224	-0.373	0.501
9225	0.297	0.099	0.492	0.234	-0.336	0.491	0.000	?	0.097
9226	0.184	-0.625	0.262	0.234	-0.336	0.273	0.295	0.082	0.097
9227	0.261	-0.137	0.513	0.307	0.135	0.149	0.050	-1.485	0.512
9228	0.484	1.295	0.262	0.506	1.415	0.478	0.474	1.228	0.142
9229	0.505	1.430	0.224	0.465	1.152	0.491	0.487	1.312	0.492
9230	0.224	-0.373	0.513	0.310	0.153	0.508	0.145	-0.878	0.185
9231	0.411	0.824	0.142	0.193	-0.600	0.491	0.226	-0.356	0.512
9232	0.405	0.790	0.481	0.477	1.227	0.273	0.508	1.447	0.142
9233	0.187	-0.608	0.262	0.234	-0.336	0.273	0.187	-0.608	0.450
9234	0.261	-0.137	0.501	0.102	-1.184	0.491	0.226	-0.356	0.185
9235	0.482	1.279	0.409	0.509	1.434	0.491	0.484	1.295	0.481
9236	0.505	1.430	0.409	0.509	1.434	0.491	0.466	1.178	0.224
9237	0.500	1.397	0.492	0.515	1.472	0.309	0.484	1.295	0.097
9238	0.447	1.060	0.142	0.465	1.152	0.193	0.503	1.413	0.097
9239	0.408	0.807	0.185	0.345	0.379	0.341	0.097	-1.181	0.185
9240	0.511	1.464	0.512	0.488	1.302	0.341	0.405	0.790	0.050
9241	0.184	-0.625	0.185	0.272	-0.091	0.193	0.297	0.099	0.512
9242	0.224	-0.373	0.501	0.149	-0.882	0.478	0.295	0.082	0.142
9243	0.500	1.397	0.467	0.474	1.208	0.193	0.447	1.060	0.142
9244	0.500	1.397	0.185	0.488	1.302	0.273	0.505	1.430	0.501
9245	0.382	0.638	0.142	0.506	1.415	0.309	0.476	1.245	0.492
9246	0.497	1.380	0.142	0.488	1.302	0.235	0.447	1.060	0.185
9247	0.142	-0.895	0.492	0.272	-0.091	0.514	0.142	-0.895	0.142
9248	0.226	-0.356	0.224	0.272	-0.091	0.514	0.184	-0.625	0.450
9249	0.497	1.380	0.467	0.491	1.321	0.501	0.405	0.790	0.224
9250	0.497	1.380	0.492	0.491	1.321	0.193	0.405	0.790	0.508
9251	0.497	1.380	0.097	0.491	1.321	0.273	0.405	0.790	0.142
9252	0.097	-1.181	0.142	0.310	0.153	0.273	0.226	-0.356	0.262
9253	0.295	0.082	0.481	0.196	-0.581	0.478	0.050	-1.485	0.481
9254	0.226	-0.356	0.481	0.342	0.361	0.491	0.097	-1.181	0.097
9255	0.226	-0.356	0.501	0.342	0.361	0.508	0.097	-1.181	0.481
9256	0.332	0.318	0.501	0.193	-0.600	0.309	0.226	-0.356	0.185
9257	0.450	1.076	0.296	0.480	1.246	0.235	0.445	1.043	0.431
9258	0.297	0.099	0.296	0.193	-0.600	0.149	0.097	-1.181	0.508
9259	0.263	-0.120	0.481	0.272	-0.091	0.444	0.097	-1.181	0.508
9260	0.358	0.487	0.296	0.310	0.153	0.341	0.097	-1.181	0.501
9261	0.097	-1.181	0.185	0.234	-0.336	0.235	0.382	0.638	0.097
9262	0.463	1.161	0.262	0.515	1.472	0.462	0.487	1.312	0.050
9263	0.476	1.245	0.512	0.515	1.472	0.508	0.487	1.312	0.492
9264	0.450	1.076	0.512	0.500	1.378	0.193	0.503	1.413	0.185
9265	0.184	-0.625	0.501	0.193	-0.600	0.273	0.184	-0.625	0.409
9266	0.142	-0.895	0.508	0.272	-0.091	0.444	0.224	-0.373	0.508
9267	0.226	-0.356	0.097	0.272	-0.091	0.102	0.261	-0.137	0.185
9268	0.479	1.262	0.431	0.477	1.227	0.514	0.426	0.925	0.296

9269	0.495	1.363	0.050	0.491	1.321	0.273	0.500	1.397	0.358
9270	0.500	1.397	0.185	0.509	1.434	0.193	0.484	1.295	0.481
9271	0.226	-0.356	0.328	0.310	0.153	0.309	0.050	-1.485	0.097
9272	0.224	-0.373	0.296	0.234	-0.336	0.341	0.145	-0.878	0.467
9273	0.261	-0.137	0.358	0.149	-0.882	0.478	0.184	-0.625	0.467
9274	0.513	1.481	0.481	0.442	1.001	0.235	0.461	1.144	0.262
9275	0.184	-0.625	0.262	0.342	0.361	0.273	0.097	-1.181	0.142
9276	0.263	-0.120	0.431	0.237	-0.317	0.512	0.097	-1.181	0.508
9277	0.479	1.262	0.262	0.462	1.133	0.193	0.476	1.245	0.492
9278	0.447	1.060	0.501	0.506	1.415	0.273	0.487	1.312	0.467
9279	0.295	0.082	0.513	0.193	-0.600	0.235	0.184	-0.625	0.467
9280	0.226	-0.356	0.513	0.275	-0.073	0.341	0.050	-1.485	0.050
9281	0.389	0.689	0.501	0.442	1.001	0.341	0.497	1.380	0.492
9282	0.329	0.301	0.450	0.102	-1.184	0.514	0.097	-1.181	0.097
9283	0.471	1.211	0.224	0.515	1.472	0.491	0.505	1.430	0.262
9284	0.263	-0.120	0.185	0.275	-0.073	0.462	0.050	-1.485	0.050
9285	0.332	0.318	0.512	0.193	-0.600	0.193	0.050	-1.485	0.431
9286	0.226	-0.356	0.328	0.310	0.153	0.462	0.050	-1.485	0.492
9287	0.184	-0.625	0.296	0.342	0.361	0.102	0.050	-1.485	0.467
9288	0.429	0.942	0.501	0.480	1.246	0.235	0.500	1.397	0.467
9289	0.226	-0.356	0.262	0.237	-0.317	0.398	0.142	-0.895	0.358
9290	0.224	-0.373	0.224	0.275	-0.073	0.398	0.097	-1.181	0.513
9291	0.450	1.076	0.185	0.512	1.453	0.273	0.474	1.228	0.296
9292	0.297	0.099	0.185	0.193	-0.600	0.508	0.050	-1.485	0.050
9293	0.295	0.082	0.481	0.275	-0.073	0.462	0.142	-0.895	0.097
9294	0.263	-0.120	0.142	0.237	-0.317	0.501	0.050	-1.485	0.142
9295	0.097	-1.181	0.481	0.345	0.379	0.341	0.142	-0.895	0.262
9296	0.097	-1.181	0.142	0.345	0.379	0.235	0.142	-0.895	0.481
9297	0.447	1.060	0.512	0.518	1.491	0.235	0.445	1.043	0.097
9298	0.511	1.464	0.185	0.491	1.321	0.444	0.474	1.228	0.501
9299	0.432	0.958	0.328	0.462	1.133	0.053	0.503	1.413	0.050
9300	0.224	-0.373	0.481	0.193	-0.600	0.462	0.142	-0.895	0.512
9301	0.511	1.464	0.467	0.462	1.133	0.149	0.500	1.397	0.492
9302	0.358	0.487	0.142	0.102	-1.184	0.514	0.050	-1.485	0.492
9303	0.263	-0.120	0.224	0.237	-0.317	0.102	0.142	-0.895	0.512
9304	0.492	1.346	0.501	0.398	0.718	0.491	0.503	1.413	0.508
9305	0.492	1.346	0.431	0.398	0.718	0.235	0.503	1.413	0.097
9306	0.224	-0.373	0.262	0.275	-0.073	0.501	0.050	-1.485	0.050
9307	0.471	1.211	0.508	0.503	1.396	0.102	0.466	1.178	0.481
9308	0.492	1.346	0.224	0.462	1.133	0.273	0.463	1.161	0.050
9309	0.508	1.447	0.224	0.491	1.321	0.273	0.495	1.363	0.142
9310	0.187	-0.608	0.328	0.339	0.342	0.149	0.097	-1.181	0.501
9311	0.326	0.285	0.492	0.234	-0.336	0.273	0.000	?	0.262
9312	0.382	0.638	0.142	0.240	-0.299	0.501	0.097	-1.181	0.142
9313	0.432	0.958	0.142	0.442	1.001	0.273	0.505	1.430	0.142
9314	0.332	0.318	0.296	0.053	-1.504	0.309	0.142	-0.895	0.467
9315	0.479	1.262	0.481	0.462	1.133	0.508	0.487	1.312	0.224
9316	0.224	-0.373	0.185	0.149	-0.882	0.444	0.184	-0.625	0.492
9317	0.263	-0.120	0.296	0.515	1.472	0.309	0.505	1.430	0.185
9318	0.297	0.099	0.262	0.105	-1.165	0.309	0.142	-0.895	0.481
9319	0.450	1.076	0.508	0.488	1.302	0.491	0.447	1.060	0.050
9320	0.263	-0.120	0.508	0.237	-0.317	0.309	0.184	-0.625	0.050
9321	0.484	1.295	0.508	0.500	1.378	0.444	0.474	1.228	0.508
9322	0.226	-0.356	0.185	0.102	-1.184	0.193	0.224	-0.373	0.501
9323	0.224	-0.373	0.224	0.275	-0.073	0.235	0.100	-1.164	0.185
9324	0.224	-0.373	0.385	0.275	-0.073	0.422	0.100	-1.164	0.224
9325	0.297	0.099	0.142	0.149	-0.882	0.309	0.187	-0.608	0.450
9326	0.226	-0.356	0.142	0.275	-0.073	0.508	0.050	-1.485	0.513
9327	0.450	1.076	0.296	0.500	1.378	0.309	0.503	1.413	0.142
9328	0.142	-0.895	0.450	0.275	-0.073	0.422	0.142	-0.895	0.467
9329	0.097	-1.181	0.262	0.313	0.172	0.512	0.142	-0.895	0.142

9330	0.497	1.380	0.450	0.500	1.378	0.512	0.495	1.363	0.142
9331	0.487	1.312	0.296	0.447	1.039	0.508	0.489	1.329	0.050
9332	0.297	0.099	0.296	0.313	0.172	0.309	0.184	-0.625	0.481
9333	0.297	0.099	0.481	0.313	0.172	0.341	0.142	-0.895	0.142
9334	0.492	1.346	0.508	0.488	1.302	0.512	0.445	1.043	0.097
9335	0.266	-0.103	0.501	0.310	0.153	0.309	0.224	-0.373	0.097
9336	0.513	1.481	0.224	0.450	1.057	0.491	0.505	1.430	0.492
9337	0.263	-0.120	0.224	0.193	-0.600	0.512	0.142	-0.895	0.142
9338	0.224	-0.373	0.501	0.237	-0.317	0.235	0.266	-0.103	0.142
9339	0.508	1.447	0.508	0.427	0.907	0.273	0.476	1.245	0.097
9340	0.142	-0.895	0.224	0.307	0.135	0.102	0.097	-1.181	0.492
9341	0.476	1.245	0.224	0.509	1.434	0.501	0.476	1.245	0.492
9342	0.263	-0.120	0.467	0.310	0.153	0.309	0.184	-0.625	0.508
9343	0.503	1.413	0.467	0.421	0.869	0.508	0.426	0.925	0.097
9344	0.408	0.807	0.481	0.512	1.453	0.512	0.500	1.397	0.050
9345	0.408	0.807	0.224	0.512	1.453	0.193	0.500	1.397	0.262
9346	0.492	1.346	0.501	0.509	1.434	0.341	0.495	1.363	0.185
9347	0.142	-0.895	0.481	0.313	0.172	0.462	0.097	-1.181	0.262
9348	0.184	-0.625	0.185	0.339	0.342	0.478	0.050	-1.485	0.224
9349	0.516	1.498	0.431	0.512	1.453	0.462	0.484	1.295	0.450
9350	0.187	-0.608	0.467	0.310	0.153	0.462	0.184	-0.625	0.142
9351	0.500	1.397	0.224	0.494	1.340	0.235	0.405	0.790	0.431
9352	0.468	1.194	0.409	0.512	1.453	0.273	0.503	1.413	0.358
9353	0.187	-0.608	0.501	0.237	-0.317	0.508	0.184	-0.625	0.262
9354	0.145	-0.878	0.492	0.272	-0.091	0.193	0.300	0.116	0.481
9355	0.142	-0.895	0.142	0.102	-1.184	0.341	0.361	0.504	0.450
9356	0.492	1.346	0.501	0.500	1.378	0.273	0.474	1.228	0.492
9357	0.224	-0.373	0.224	0.310	0.153	0.491	0.097	-1.181	0.328
9358	0.463	1.161	0.492	0.506	1.415	0.491	0.463	1.161	0.185
9359	0.489	1.329	0.508	0.506	1.415	0.508	0.468	1.194	0.501
9360	0.097	-1.181	0.185	0.193	-0.600	0.512	0.263	-0.120	0.492
9361	0.142	-0.895	0.142	0.342	0.361	0.193	0.142	-0.895	0.097
9362	0.482	1.279	0.224	0.462	1.133	0.478	0.503	1.413	0.097
9363	0.484	1.295	0.450	0.482	1.265	0.193	0.487	1.312	0.508
9364	0.503	1.413	0.450	0.465	1.152	0.309	0.461	1.144	0.501
9365	0.503	1.413	0.501	0.465	1.152	0.193	0.461	1.144	0.450
9366	0.297	0.099	0.224	0.237	-0.317	0.102	0.050	-1.485	0.262
9367	0.297	0.099	0.142	0.272	-0.091	0.341	0.000	?	0.481
9368	0.482	1.279	0.224	0.512	1.453	0.508	0.487	1.312	0.508
9369	0.295	0.082	0.501	0.193	-0.600	0.235	0.097	-1.181	0.358
9370	0.184	-0.625	0.224	0.345	0.379	0.478	0.263	-0.120	0.512
9371	0.263	-0.120	0.501	0.272	-0.091	0.235	0.050	-1.485	0.508
9372	0.508	1.447	0.508	0.488	1.302	0.508	0.426	0.925	0.185
9373	0.511	1.464	0.185	0.488	1.302	0.514	0.487	1.312	0.431
9374	0.505	1.430	0.097	0.506	1.415	0.514	0.468	1.194	0.492
9375	0.511	1.464	0.142	0.509	1.434	0.508	0.468	1.194	0.481
9376	0.097	-1.181	0.296	0.193	-0.600	0.501	0.355	0.470	0.296
9377	0.434	0.975	0.262	0.480	1.246	0.235	0.503	1.413	0.328
9378	0.050	-1.485	0.492	0.193	-0.600	0.341	0.295	0.082	0.142
9379	0.184	-0.625	0.224	0.310	0.153	0.422	0.050	-1.485	0.501
9380	0.329	0.301	0.142	0.193	-0.600	0.193	0.097	-1.181	0.358
9381	0.300	0.116	0.097	0.102	-1.184	0.102	0.142	-0.895	0.185
9382	0.358	0.487	0.050	0.339	0.342	0.422	0.261	-0.137	0.358
9383	0.474	1.228	0.224	0.509	1.434	0.273	0.482	1.279	0.224
9384	0.263	-0.120	0.097	0.237	-0.317	0.053	0.097	-1.181	0.431
9385	0.429	0.942	0.431	0.477	1.227	0.235	0.497	1.380	0.431
9386	0.263	-0.120	0.358	0.234	-0.336	0.235	0.050	-1.485	0.501
9387	0.503	1.413	0.262	0.509	1.434	0.514	0.503	1.413	0.142
9388	0.516	1.498	0.328	0.512	1.453	0.193	0.484	1.295	0.185
9389	0.516	1.498	0.450	0.512	1.453	0.514	0.484	1.295	0.185
9390	0.503	1.413	0.224	0.509	1.434	0.309	0.503	1.413	0.358

9391	0.450	1.076	0.262	0.506	1.415	0.478	0.497	1.380	0.296
9392	0.224	-0.373	0.450	0.237	-0.317	0.309	0.097	-1.181	0.431
9393	0.187	-0.608	0.481	0.339	0.342	0.193	0.050	-1.485	0.467
9394	0.511	1.464	0.142	0.421	0.869	0.512	0.476	1.245	0.050
9395	0.332	0.318	0.492	0.193	-0.600	0.512	0.050	-1.485	0.385
9396	0.295	0.082	0.492	0.102	-1.184	0.273	0.142	-0.895	0.142
9397	0.503	1.413	0.224	0.421	0.869	0.102	0.492	1.346	0.185
9398	0.263	-0.120	0.224	0.272	-0.091	0.478	0.000	?	0.142
9399	0.226	-0.356	0.262	0.053	-1.504	0.478	0.263	-0.120	0.450
9400	0.184	-0.625	0.262	0.237	-0.317	0.149	0.142	-0.895	0.185
9401	0.184	-0.625	0.512	0.237	-0.317	0.514	0.142	-0.895	0.296
9402	0.479	1.262	0.431	0.512	1.453	0.273	0.461	1.144	0.185
9403	0.145	-0.878	0.224	0.193	-0.600	0.193	0.224	-0.373	0.296
9404	0.484	1.295	0.508	0.509	1.434	0.193	0.484	1.295	0.358
9405	0.145	-0.878	0.481	0.310	0.153	0.501	0.184	-0.625	0.385
9406	0.511	1.464	0.296	0.482	1.265	0.371	0.476	1.245	0.513
9407	0.184	-0.625	0.296	0.310	0.153	0.309	0.050	-1.485	0.481
9408	0.329	0.301	0.262	0.193	-0.600	0.149	0.050	-1.485	0.224
9409	0.482	1.279	0.185	0.512	1.453	0.491	0.497	1.380	0.224
9410	0.468	1.194	0.224	0.503	1.396	0.235	0.497	1.380	0.512
9411	0.142	-0.895	0.296	0.275	-0.073	0.102	0.184	-0.625	0.097
9412	0.226	-0.356	0.224	0.102	-1.184	0.193	0.224	-0.373	0.097
9413	0.500	1.397	0.224	0.477	1.227	0.478	0.445	1.043	0.431
9414	0.429	0.942	0.481	0.480	1.246	0.478	0.503	1.413	0.409
9415	0.261	-0.137	0.467	0.152	-0.863	0.491	0.142	-0.895	0.224
9416	0.505	1.430	0.224	0.512	1.453	0.341	0.461	1.144	0.481
9417	0.226	-0.356	0.224	0.272	-0.091	0.273	0.142	-0.895	0.501
9418	0.224	-0.373	0.097	0.193	-0.600	0.273	0.142	-0.895	0.142
9419	0.329	0.301	0.185	0.193	-0.600	0.235	0.050	-1.485	0.097
9420	0.487	1.312	0.512	0.506	1.415	0.341	0.474	1.228	0.409
9421	0.142	-0.895	0.224	0.371	0.549	0.273	0.142	-0.895	0.296
9422	0.142	-0.895	0.224	0.313	0.172	0.478	0.097	-1.181	0.467
9423	0.297	0.099	0.385	0.149	-0.882	0.235	0.097	-1.181	0.512
9424	0.479	1.262	0.296	0.491	1.321	0.501	0.484	1.295	0.512
9425	0.184	-0.625	0.358	0.237	-0.317	0.273	0.145	-0.878	0.501
9426	0.295	0.082	0.224	0.102	-1.184	0.193	0.142	-0.895	0.508
9427	0.261	-0.137	0.262	0.196	-0.581	0.398	0.097	-1.181	0.224
9428	0.508	1.447	0.358	0.477	1.227	0.341	0.484	1.295	0.185
9429	0.508	1.447	0.142	0.477	1.227	0.398	0.484	1.295	0.142
9430	0.505	1.430	0.142	0.488	1.302	0.273	0.505	1.430	0.296
9431	0.184	-0.625	0.512	0.342	0.361	0.309	0.097	-1.181	0.262
9432	0.224	-0.373	0.185	0.278	-0.054	0.512	0.050	-1.485	0.142
9433	0.382	0.638	0.185	0.272	-0.091	0.491	0.261	-0.137	0.224
9434	0.142	-0.895	0.358	0.234	-0.336	0.309	0.187	-0.608	0.142
9435	0.142	-0.895	0.142	0.342	0.361	0.309	0.266	-0.103	0.492
9436	0.295	0.082	0.450	0.275	-0.073	0.501	0.226	-0.356	0.508
9437	0.450	1.076	0.409	0.480	1.246	0.491	0.450	1.076	0.142
9438	0.261	-0.137	0.513	0.234	-0.336	0.444	0.142	-0.895	0.185
9439	0.447	1.060	0.224	0.503	1.396	0.235	0.426	0.925	0.142
9440	0.295	0.082	0.185	0.278	-0.054	0.398	0.358	0.487	0.296
9441	0.300	0.116	0.512	0.196	-0.581	0.462	0.261	-0.137	0.513
9442	0.484	1.295	0.142	0.395	0.700	0.309	0.479	1.262	0.097
9443	0.511	1.464	0.185	0.339	0.342	0.491	0.447	1.060	0.224
9444	0.497	1.380	0.296	0.398	0.718	0.491	0.492	1.346	0.358
9445	0.224	-0.373	0.501	0.272	-0.091	0.193	0.329	0.301	0.262
9446	0.224	-0.373	0.512	0.307	0.135	0.508	0.184	-0.625	0.296
9447	0.497	1.380	0.512	0.512	1.453	0.462	0.500	1.397	0.142
9448	0.505	1.430	0.481	0.491	1.321	0.501	0.487	1.312	0.142
9449	0.226	-0.356	0.492	0.310	0.153	0.149	0.097	-1.181	0.296
9450	0.226	-0.356	0.508	0.310	0.153	0.512	0.097	-1.181	0.296
9451	0.468	1.194	0.492	0.500	1.378	0.491	0.505	1.430	0.185

9452	0.463	1.161	0.492	0.485	1.283	0.491	0.500	1.397	0.358
9453	0.476	1.245	0.501	0.442	1.001	0.514	0.450	1.076	0.050
9454	0.224	-0.373	0.142	0.234	-0.336	0.193	0.263	-0.120	0.050
9455	0.503	1.413	0.501	0.401	0.737	0.341	0.474	1.228	0.142
9456	0.482	1.279	0.512	0.462	1.133	0.102	0.503	1.413	0.431
9457	0.184	-0.625	0.409	0.307	0.135	0.478	0.361	0.504	0.508
9458	0.429	0.942	0.185	0.491	1.321	0.478	0.505	1.430	0.512
9459	0.463	1.161	0.513	0.494	1.340	0.501	0.503	1.413	0.262
9460	0.224	-0.373	0.481	0.193	-0.600	0.235	0.184	-0.625	0.296
9461	0.405	0.790	0.501	0.506	1.415	0.235	0.426	0.925	0.501
9462	0.495	1.363	0.185	0.462	1.133	0.309	0.492	1.346	0.328
9463	0.487	1.312	0.512	0.503	1.396	0.491	0.479	1.262	0.097
9464	0.142	-0.895	0.512	0.149	-0.882	0.102	0.300	0.116	0.050
9465	0.500	1.397	0.328	0.515	1.472	0.512	0.329	0.301	0.467
9466	0.224	-0.373	0.513	0.494	1.340	0.462	0.142	-0.895	0.501
9467	0.489	1.329	0.508	0.491	1.321	0.309	0.500	1.397	0.492
9468	0.508	1.447	0.508	0.512	1.453	0.273	0.355	0.470	0.492
9469	0.184	-0.625	0.224	0.196	-0.581	0.341	0.187	-0.608	0.501
9470	0.145	-0.878	0.512	0.339	0.342	0.235	0.358	0.487	0.508
9471	0.224	-0.373	0.512	0.105	-1.165	0.149	0.224	-0.373	0.513
9472	0.447	1.060	0.512	0.477	1.227	0.462	0.432	0.958	0.508
9473	0.447	1.060	0.512	0.477	1.227	0.309	0.432	0.958	0.501
9474	0.500	1.397	0.512	0.497	1.359	0.102	0.500	1.397	0.224
9475	0.224	-0.373	0.481	0.234	-0.336	0.309	0.142	-0.895	0.492
9476	0.142	-0.895	0.481	0.237	-0.317	0.273	0.184	-0.625	0.508
9477	0.226	-0.356	0.481	0.313	0.172	0.102	0.184	-0.625	0.409
9478	0.497	1.380	0.501	0.488	1.302	0.102	0.355	0.470	0.185
9479	0.224	-0.373	0.481	0.105	-1.165	0.444	0.295	0.082	0.467
9480	0.492	1.346	0.262	0.512	1.453	0.514	0.432	0.958	0.508
9481	0.508	1.447	0.512	0.462	1.133	0.235	0.461	1.144	0.481
9482	0.187	-0.608	0.481	0.310	0.153	0.309	0.050	-1.485	0.142
9483	0.097	-1.181	0.508	0.275	-0.073	0.478	0.387	0.672	0.508
9484	0.142	-0.895	0.508	0.345	0.379	0.235	0.142	-0.895	0.508
9485	0.300	0.116	0.512	0.234	-0.336	0.235	0.184	-0.625	0.450
9486	0.263	-0.120	0.467	0.149	-0.882	0.491	0.142	-0.895	0.385
9487	0.495	1.363	0.492	0.459	1.114	0.462	0.447	1.060	0.467
9488	0.224	-0.373	0.185	0.310	0.153	0.371	0.184	-0.625	0.467
9489	0.142	-0.895	0.501	0.102	-1.184	0.501	0.297	0.099	0.328
9490	0.097	-1.181	0.050	0.310	0.153	0.491	0.184	-0.625	0.508
9491	0.050	-1.485	0.142	0.275	-0.073	0.309	0.297	0.099	0.501
9492	0.226	-0.356	0.185	0.102	-1.184	0.341	0.361	0.504	0.512
9493	0.097	-1.181	0.185	0.102	-1.184	0.309	0.387	0.672	0.492
9494	0.429	0.942	0.492	0.444	1.020	0.235	0.511	1.464	0.508
9495	0.358	0.487	0.512	0.515	1.472	0.512	0.476	1.245	0.467
9496	0.263	-0.120	0.512	0.234	-0.336	0.462	0.224	-0.373	0.467
9497	0.329	0.301	0.513	0.310	0.153	0.514	0.224	-0.373	0.467
9498	0.450	1.076	0.224	0.474	1.208	0.512	0.511	1.464	0.501
9499	0.224	-0.373	0.224	0.234	-0.336	0.514	0.097	-1.181	0.512
9500	0.261	-0.137	0.513	0.234	-0.336	0.341	0.097	-1.181	0.296
9501	0.447	1.060	0.513	0.491	1.321	0.149	0.432	0.958	0.512
9502	0.479	1.262	0.508	0.462	1.133	0.149	0.408	0.807	0.508
9503	0.142	-0.895	0.097	0.374	0.568	0.371	0.224	-0.373	0.501
9504	0.492	1.346	0.501	0.500	1.378	0.149	0.484	1.295	0.501
9505	0.489	1.329	0.185	0.494	1.340	0.102	0.497	1.380	0.501
9506	0.224	-0.373	0.450	0.307	0.135	0.102	0.142	-0.895	0.467
9507	0.226	-0.356	0.409	0.342	0.361	0.193	0.097	-1.181	0.467
9508	0.261	-0.137	0.508	0.310	0.153	0.514	0.408	0.807	0.431
9509	0.261	-0.137	0.512	0.237	-0.317	0.501	0.295	0.082	0.450
9510	0.508	1.447	0.501	0.509	1.434	0.273	0.463	1.161	0.185
9511	0.434	0.975	0.185	0.462	1.133	0.341	0.513	1.481	0.185
9512	0.224	-0.373	0.512	0.515	1.472	0.309	0.513	1.481	0.185

9513	0.511	1.464	0.450	0.512	1.453	0.371	0.497	1.380	0.224
9514	0.482	1.279	0.481	0.520	1.509	0.444	0.505	1.430	0.467
9515	0.297	0.099	0.224	0.345	0.379	0.273	0.224	-0.373	0.385
9516	0.297	0.099	0.508	0.149	-0.882	0.341	0.187	-0.608	0.481
9517	0.261	-0.137	0.142	0.149	-0.882	0.309	0.142	-0.895	0.508
9518	0.184	-0.625	0.501	0.377	0.587	0.193	0.295	0.082	0.097
9519	0.224	-0.373	0.142	0.149	-0.882	0.149	0.263	-0.120	0.097
9520	0.295	0.082	0.492	0.102	-1.184	0.193	0.142	-0.895	0.450
9521	0.224	-0.373	0.492	0.102	-1.184	0.273	0.224	-0.373	0.467
9522	0.224	-0.373	0.492	0.193	-0.600	0.193	0.142	-0.895	0.512
9523	0.482	1.279	0.508	0.512	1.453	0.102	0.487	1.312	0.185
9524	0.463	1.161	0.467	0.503	1.396	0.235	0.500	1.397	0.481
9525	0.226	-0.356	0.185	0.275	-0.073	0.512	0.142	-0.895	0.296
9526	0.226	-0.356	0.508	0.345	0.379	0.444	0.184	-0.625	0.513
9527	0.097	-1.181	0.501	0.313	0.172	0.462	0.142	-0.895	0.508
9528	0.184	-0.625	0.185	0.371	0.549	0.235	0.295	0.082	0.513
9529	0.511	1.464	0.467	0.444	1.020	0.398	0.508	1.447	0.512
9530	0.226	-0.356	0.481	0.275	-0.073	0.512	0.097	-1.181	0.508
9531	0.229	-0.339	0.508	0.342	0.361	0.508	0.224	-0.373	0.328
9532	0.384	0.655	0.481	0.307	0.135	0.491	0.361	0.504	0.481
9533	0.295	0.082	0.185	0.193	-0.600	0.102	0.261	-0.137	0.501
9534	0.361	0.504	0.142	0.149	-0.882	0.102	0.297	0.099	0.508
9535	0.224	-0.373	0.142	0.193	-0.600	0.462	0.142	-0.895	0.224
9536	0.266	-0.103	0.501	0.272	-0.091	0.371	0.142	-0.895	0.508
9537	0.358	0.487	0.262	0.193	-0.600	0.273	0.300	0.116	0.262
9538	0.142	-0.895	0.501	0.102	-1.184	0.341	0.295	0.082	0.512
9539	0.142	-0.895	0.224	0.237	-0.317	0.508	0.187	-0.608	0.224
9540	0.511	1.464	0.296	0.518	1.491	0.512	0.358	0.487	0.467
9541	0.184	-0.625	0.508	0.444	1.020	0.512	0.050	-1.485	0.512
9542	0.184	-0.625	0.097	0.462	1.133	0.512	0.050	-1.485	0.501
9543	0.358	0.487	0.050	0.234	-0.336	0.514	0.142	-0.895	0.508
9544	0.145	-0.878	0.513	0.395	0.700	0.478	0.000	?	0.492
9545	0.447	1.060	0.508	0.509	1.434	0.501	0.426	0.925	0.328
9546	0.405	0.790	0.508	0.506	1.415	0.501	0.503	1.413	0.492
9547	0.513	1.481	0.501	0.491	1.321	0.512	0.508	1.447	0.512
9548	0.224	-0.373	0.512	0.102	-1.184	0.309	0.261	-0.137	0.050
9549	0.184	-0.625	0.492	0.102	-1.184	0.512	0.295	0.082	0.508
9550	0.503	1.413	0.501	0.462	1.133	0.508	0.503	1.413	0.492
9551	0.142	-0.895	0.512	0.371	0.549	0.193	0.326	0.285	0.513
9552	0.184	-0.625	0.512	0.272	-0.091	0.273	0.097	-1.181	0.508
9553	0.295	0.082	0.512	0.339	0.342	0.508	0.050	-1.485	0.185
9554	0.500	1.397	0.508	0.506	1.415	0.444	0.461	1.144	0.185
9555	0.511	1.464	0.508	0.512	1.453	0.478	0.503	1.413	0.185
9556	0.508	1.447	0.142	0.512	1.453	0.235	0.492	1.346	0.513
9557	0.479	1.262	0.142	0.509	1.434	0.422	0.489	1.329	0.409
9558	0.487	1.312	0.142	0.518	1.491	0.512	0.500	1.397	0.467
9559	0.505	1.430	0.513	0.482	1.265	0.462	0.508	1.447	0.097
9560	0.495	1.363	0.501	0.503	1.396	0.501	0.518	1.514	0.142
9561	0.487	1.312	0.508	0.503	1.396	0.512	0.513	1.481	0.508
9562	0.497	1.380	0.492	0.509	1.434	0.508	0.503	1.413	0.185
9563	0.142	-0.895	0.492	0.310	0.153	0.371	0.226	-0.356	0.185
9564	0.492	1.346	0.224	0.512	1.453	0.508	0.497	1.380	0.481
9565	0.511	1.464	0.262	0.506	1.415	0.508	0.503	1.413	0.512
9566	0.408	0.807	0.508	0.193	-0.600	0.508	0.413	0.841	0.513
9567	0.184	-0.625	0.508	0.275	-0.073	0.512	0.187	-0.608	0.409
9568	0.513	1.481	0.481	0.515	1.472	0.422	0.466	1.178	0.508
9569	0.479	1.262	0.481	0.447	1.039	0.491	0.511	1.464	0.501
9570	0.500	1.397	0.492	0.474	1.208	0.491	0.482	1.279	0.409
9571	0.184	-0.625	0.492	0.234	-0.336	0.491	0.142	-0.895	0.409
9572	0.508	1.447	0.097	0.427	0.907	0.508	0.508	1.447	0.409
9573	0.508	1.447	0.508	0.520	1.509	0.491	0.508	1.447	0.508

9574	0.329	0.301	0.508	0.465	1.152	0.149	0.450	1.076	0.508
9575	0.511	1.464	0.508	0.506	1.415	0.514	0.384	0.655	0.508
9576	0.508	1.447	0.508	0.509	1.434	0.501	0.463	1.161	0.296
9577	0.508	1.447	0.501	0.506	1.415	0.508	0.463	1.161	0.296
9578	0.224	-0.373	0.431	0.371	0.549	0.512	0.334	0.335	0.296
9579	0.511	1.464	0.467	0.509	1.434	0.478	0.505	1.430	0.501
9580	0.511	1.464	0.501	0.509	1.434	0.501	0.503	1.413	0.481
9581	0.505	1.430	0.513	0.512	1.453	0.508	0.513	1.481	0.508
9582	0.511	1.464	0.508	0.509	1.434	0.273	0.489	1.329	0.501
9583	0.513	1.481	0.328	0.421	0.869	0.508	0.511	1.464	0.513
9584	0.479	1.262	0.481	0.485	1.283	0.309	0.463	1.161	0.185
9585	0.479	1.262	0.431	0.485	1.283	0.273	0.463	1.161	0.050
9586	0.479	1.262	0.492	0.485	1.283	0.235	0.463	1.161	0.513
9587	0.503	1.413	0.409	0.500	1.378	0.341	0.500	1.397	0.501
9588	0.474	1.228	0.385	0.488	1.302	0.508	0.518	1.514	0.508
9589	0.261	-0.137	0.467	0.149	-0.882	0.512	0.295	0.082	0.512
9590	0.500	1.397	0.512	0.515	1.472	0.508	0.513	1.481	0.501
9591	0.476	1.245	0.512	0.500	1.378	0.514	0.505	1.430	0.501
9592	0.500	1.397	0.481	0.506	1.415	0.235	0.505	1.430	0.328
9593	0.500	1.397	0.481	0.509	1.434	0.235	0.508	1.447	0.512
9594	0.503	1.413	0.481	0.477	1.227	0.398	0.505	1.430	0.512
9595	0.468	1.194	0.224	0.497	1.359	0.422	0.463	1.161	0.512
9596	0.484	1.295	0.450	0.509	1.434	0.512	0.466	1.178	0.512
9597	0.184	-0.625	0.450	0.275	-0.073	0.309	0.432	0.958	0.512
9598	0.503	1.413	0.492	0.503	1.396	0.501	0.447	1.060	0.512
9599	0.050	-1.485	0.501	0.310	0.153	0.309	0.187	-0.608	0.481
9600	0.142	-0.895	0.492	0.275	-0.073	0.491	0.184	-0.625	0.508
9601	0.184	-0.625	0.467	0.237	-0.317	0.398	0.184	-0.625	0.492
9602	0.187	-0.608	0.262	0.342	0.361	0.478	0.224	-0.373	0.508
9603	0.489	1.329	0.492	0.503	1.396	0.462	0.463	1.161	0.501
9604	0.505	1.430	0.224	0.512	1.453	0.462	0.384	0.655	0.508
9605	0.503	1.413	0.296	0.506	1.415	0.193	0.482	1.279	0.513
9606	0.503	1.413	0.224	0.515	1.472	0.514	0.508	1.447	0.481
9607	0.224	-0.373	0.262	0.240	-0.299	0.508	0.097	-1.181	0.467
9608	0.224	-0.373	0.296	0.240	-0.299	0.422	0.097	-1.181	0.481
9609	0.511	1.464	0.512	0.398	0.718	0.273	0.450	1.076	0.508
9610	0.511	1.464	0.142	0.424	0.888	0.478	0.466	1.178	0.508
9611	0.511	1.464	0.512	0.506	1.415	0.235	0.508	1.447	0.508
9612	0.097	-1.181	0.512	0.313	0.172	0.462	0.184	-0.625	0.501
9613	0.500	1.397	0.512	0.503	1.396	0.235	0.484	1.295	0.492
9614	0.184	-0.625	0.328	0.310	0.153	0.512	0.300	0.116	0.492
9615	0.450	1.076	0.185	0.488	1.302	0.462	0.513	1.481	0.385
9616	0.408	0.807	0.328	0.395	0.700	0.512	0.508	1.447	0.508
9617	0.505	1.430	0.050	0.477	1.227	0.462	0.508	1.447	0.450
9618	0.508	1.447	0.050	0.465	1.152	0.512	0.505	1.430	0.467
9619	0.500	1.397	0.467	0.465	1.152	0.235	0.503	1.413	0.467
9620	0.187	-0.608	0.097	0.193	-0.600	0.478	0.332	0.318	0.481
9621	0.505	1.430	0.097	0.515	1.472	0.422	0.479	1.262	0.501
9622	0.447	1.060	0.512	0.509	1.434	0.309	0.503	1.413	0.262
9623	0.482	1.279	0.050	0.427	0.907	0.514	0.508	1.447	0.358
9624	0.224	-0.373	0.050	0.278	-0.054	0.508	0.226	-0.356	0.224
9625	0.500	1.397	0.050	0.474	1.208	0.501	0.511	1.464	0.328
9626	0.142	-0.895	0.508	0.237	-0.317	0.508	0.263	-0.120	0.224
9627	0.495	1.363	0.508	0.462	1.133	0.193	0.513	1.481	0.358
9628	0.142	-0.895	0.512	0.234	-0.336	0.309	0.224	-0.373	0.358
9629	0.484	1.295	0.512	0.512	1.453	0.309	0.474	1.228	0.492
9630	0.484	1.295	0.512	0.462	1.133	0.491	0.511	1.464	0.097
9631	0.489	1.329	0.481	0.509	1.434	0.309	0.500	1.397	0.501
9632	0.513	1.481	0.262	0.465	1.152	0.491	0.503	1.413	0.481
9633	0.468	1.194	0.431	0.515	1.472	0.235	0.492	1.346	0.467
9634	0.184	-0.625	0.481	0.237	-0.317	0.273	0.329	0.301	0.224

9635	0.503	1.413	0.492	0.480	1.246	0.508	0.495	1.363	0.296
9636	0.497	1.380	0.501	0.421	0.869	0.273	0.516	1.498	0.185
9637	0.184	-0.625	0.467	0.313	0.172	0.422	0.050	-1.485	0.296
9638	0.461	1.144	0.431	0.515	1.472	0.444	0.511	1.464	0.296
9639	0.484	1.295	0.508	0.509	1.434	0.478	0.497	1.380	0.508
9640	0.497	1.380	0.328	0.506	1.415	0.491	0.511	1.464	0.142
9641	0.474	1.228	0.501	0.509	1.434	0.508	0.508	1.447	0.097
9642	0.184	-0.625	0.492	0.193	-0.600	0.512	0.184	-0.625	0.385
9643	0.142	-0.895	0.512	0.307	0.135	0.444	0.184	-0.625	0.262
9644	0.142	-0.895	0.492	0.307	0.135	0.512	0.184	-0.625	0.262
9645	0.500	1.397	0.492	0.491	1.321	0.514	0.511	1.464	0.262
9646	0.266	-0.103	0.512	0.310	0.153	0.508	0.408	0.807	0.328
9647	0.500	1.397	0.481	0.491	1.321	0.422	0.468	1.194	0.512
9648	0.224	-0.373	0.501	0.240	-0.299	0.422	0.097	-1.181	0.512
9649	0.295	0.082	0.501	0.278	-0.054	0.462	0.142	-0.895	0.512
9650	0.505	1.430	0.142	0.512	1.453	0.371	0.505	1.430	0.512
9651	0.097	-1.181	0.508	0.275	-0.073	0.371	0.187	-0.608	0.492
9652	0.050	-1.485	0.501	0.427	0.907	0.371	0.184	-0.625	0.501
9653	0.505	1.430	0.481	0.447	1.039	0.398	0.482	1.279	0.328
9654	0.497	1.380	0.481	0.480	1.246	0.512	0.513	1.481	0.450
9655	0.497	1.380	0.481	0.488	1.302	0.501	0.518	1.514	0.508
9656	0.497	1.380	0.508	0.506	1.415	0.514	0.405	0.790	0.492
9657	0.508	1.447	0.142	0.509	1.434	0.512	0.511	1.464	0.492
9658	0.495	1.363	0.224	0.439	0.982	0.149	0.503	1.413	0.512
9659	0.500	1.397	0.501	0.515	1.472	0.309	0.408	0.807	0.508
9660	0.511	1.464	0.142	0.515	1.472	0.491	0.408	0.807	0.512
9661	0.500	1.397	0.050	0.509	1.434	0.398	0.413	0.841	0.224
9662	0.511	1.464	0.501	0.424	0.888	0.462	0.505	1.430	0.450
9663	0.508	1.447	0.467	0.424	0.888	0.462	0.505	1.430	0.467
9664	0.508	1.447	0.185	0.462	1.133	0.491	0.505	1.430	0.481
9665	0.142	-0.895	0.097	0.374	0.568	0.491	0.295	0.082	0.450
9666	0.142	-0.895	0.262	0.374	0.568	0.273	0.295	0.082	0.481
9667	0.142	-0.895	0.512	0.374	0.568	0.478	0.295	0.082	0.467
9668	0.508	1.447	0.050	0.395	0.700	0.478	0.497	1.380	0.481
9669	0.500	1.397	0.508	0.518	1.491	0.478	0.476	1.245	0.185
9670	0.503	1.413	0.508	0.497	1.359	0.478	0.508	1.447	0.492
9671	0.487	1.312	0.492	0.518	1.491	0.508	0.503	1.413	0.481
9672	0.489	1.329	0.467	0.515	1.472	0.512	0.511	1.464	0.296
9673	0.224	-0.373	0.467	0.149	-0.882	0.512	0.184	-0.625	0.467
9674	0.261	-0.137	0.481	0.313	0.172	0.508	0.050	-1.485	0.467
9675	0.505	1.430	0.467	0.491	1.321	0.491	0.511	1.464	0.467
9676	0.505	1.430	0.467	0.395	0.700	0.508	0.500	1.397	0.512
9677	0.484	1.295	0.512	0.468	1.170	0.371	0.508	1.447	0.467
9678	0.484	1.295	0.262	0.468	1.170	0.512	0.511	1.464	0.481
9679	0.495	1.363	0.481	0.494	1.340	0.501	0.503	1.413	0.358
9680	0.495	1.363	0.467	0.494	1.340	0.501	0.503	1.413	0.185
9681	0.097	-1.181	0.224	0.275	-0.073	0.462	0.329	0.301	0.508
9682	0.497	1.380	0.142	0.480	1.246	0.444	0.513	1.481	0.296
9683	0.497	1.380	0.328	0.480	1.246	0.491	0.513	1.481	0.262
9684	0.497	1.380	0.050	0.480	1.246	0.501	0.513	1.481	0.508
9685	0.497	1.380	0.050	0.480	1.246	0.501	0.513	1.481	0.512
9686	0.495	1.363	0.513	0.512	1.453	0.514	0.508	1.447	0.185
9687	0.429	0.942	0.513	0.515	1.472	0.514	0.511	1.464	0.450
9688	0.463	1.161	0.262	0.509	1.434	0.514	0.482	1.279	0.224
9689	0.505	1.430	0.262	0.506	1.415	0.309	0.508	1.447	0.512
9690	0.513	1.481	0.450	0.488	1.302	0.508	0.487	1.312	0.142
9691	0.508	1.447	0.224	0.506	1.415	0.501	0.508	1.447	0.467
9692	0.332	0.318	0.185	0.374	0.568	0.478	0.497	1.380	0.467
9693	0.479	1.262	0.467	0.506	1.415	0.501	0.505	1.430	0.501
9694	0.429	0.942	0.050	0.497	1.359	0.512	0.516	1.498	0.508
9695	0.487	1.312	0.467	0.500	1.378	0.508	0.476	1.245	0.481

9696	0.413	0.841	0.467	0.456	1.095	0.273	0.463	1.161	0.492
9697	0.389	0.689	0.492	0.439	0.982	0.512	0.479	1.262	0.492
9698	0.463	1.161	0.328	0.485	1.283	0.235	0.505	1.430	0.481
9699	0.505	1.430	0.467	0.497	1.359	0.273	0.508	1.447	0.328
9700	0.505	1.430	0.481	0.497	1.359	0.193	0.508	1.447	0.262
9701	0.474	1.228	0.512	0.512	1.453	0.341	0.505	1.430	0.431
9702	0.474	1.228	0.512	0.512	1.453	0.371	0.497	1.380	0.512
9703	0.474	1.228	0.508	0.512	1.453	0.491	0.497	1.380	0.358
9704	0.226	−0.356	0.508	0.310	0.153	0.398	0.382	0.638	0.328
9705	0.453	1.093	0.097	0.503	1.396	0.462	0.505	1.430	0.224
9706	0.450	1.076	0.097	0.500	1.378	0.512	0.450	1.076	0.262
9707	0.492	1.346	0.512	0.477	1.227	0.512	0.466	1.178	0.501
9708	0.500	1.397	0.501	0.500	1.378	0.102	0.461	1.144	0.501
9709	0.495	1.363	0.185	0.512	1.453	0.235	0.476	1.245	0.185
9710	0.466	1.178	0.185	0.509	1.434	0.309	0.500	1.397	0.262
9711	0.261	−0.137	0.508	0.272	−0.091	0.371	0.266	−0.103	0.508
9712	0.492	1.346	0.512	0.512	1.453	0.371	0.358	0.487	0.224
9713	0.226	−0.356	0.501	0.237	−0.317	0.514	0.224	−0.373	0.224
9714	0.295	0.082	0.512	0.275	−0.073	0.371	0.332	0.318	0.513
9715	0.224	−0.373	0.224	0.193	−0.600	0.371	0.224	−0.373	0.501
9716	0.261	−0.137	0.328	0.345	0.379	0.491	0.361	0.504	0.467
9717	0.297	0.099	0.512	0.374	0.568	0.371	0.361	0.504	0.501
9718	0.508	1.447	0.262	0.488	1.302	0.341	0.492	1.346	0.050
9719	0.142	−0.895	0.492	0.398	0.718	0.398	0.097	−1.181	0.501
9720	0.503	1.413	0.501	0.468	1.170	0.273	0.505	1.430	0.501
9721	0.505	1.430	0.508	0.509	1.434	0.508	0.482	1.279	0.492
9722	0.505	1.430	0.224	0.509	1.434	0.501	0.466	1.178	0.492
9723	0.326	0.285	0.224	0.102	−1.184	0.508	0.224	−0.373	0.481
9724	0.187	−0.608	0.508	0.237	−0.317	0.508	0.297	0.099	0.467
9725	0.326	0.285	0.262	0.310	0.153	0.478	0.184	−0.625	0.358
9726	0.050	−1.485	0.467	0.374	0.568	0.422	0.295	0.082	0.328
9727	0.050	−1.485	0.481	0.374	0.568	0.149	0.295	0.082	0.481
9728	0.463	1.161	0.050	0.518	1.491	0.462	0.503	1.413	0.467
9729	0.097	−1.181	0.142	0.374	0.568	0.501	0.142	−0.895	0.385
9730	0.097	−1.181	0.492	0.371	0.549	0.371	0.097	−1.181	0.385
9731	0.503	1.413	0.431	0.494	1.340	0.501	0.384	0.655	0.450
9732	0.050	−1.485	0.501	0.368	0.530	0.478	0.261	−0.137	0.467
9733	0.050	−1.485	0.508	0.342	0.361	0.491	0.261	−0.137	0.512
9734	0.050	−1.485	0.262	0.398	0.718	0.512	0.263	−0.120	0.492
9735	0.000	?	0.450	0.275	−0.073	0.309	0.326	0.285	0.185
9736	0.503	1.413	0.142	0.509	1.434	0.501	0.508	1.447	0.097
9737	0.503	1.413	0.450	0.500	1.378	0.398	0.508	1.447	0.508
9738	0.505	1.430	0.142	0.509	1.434	0.508	0.508	1.447	0.097
9739	0.505	1.430	0.501	0.509	1.434	0.508	0.508	1.447	0.513
9740	0.500	1.397	0.142	0.482	1.265	0.508	0.489	1.329	0.450
9741	0.474	1.228	0.492	0.421	0.869	0.444	0.503	1.413	0.450
9742	0.261	−0.137	0.492	0.149	−0.882	0.512	0.329	0.301	0.224
9743	0.429	0.942	0.512	0.462	1.133	0.371	0.453	1.093	0.296
9744	0.474	1.228	0.513	0.503	1.396	0.478	0.505	1.430	0.450
9745	0.484	1.295	0.512	0.377	0.587	0.422	0.497	1.380	0.409
9746	0.495	1.363	0.513	0.503	1.396	0.102	0.492	1.346	0.512
9747	0.461	1.144	0.467	0.482	1.265	0.514	0.511	1.464	0.409
9748	0.426	0.925	0.185	0.494	1.340	0.512	0.511	1.464	0.224
9749	0.503	1.413	0.262	0.509	1.434	0.491	0.511	1.464	0.224
9750	0.329	0.301	0.513	0.310	0.153	0.491	0.224	−0.373	0.501
9751	0.500	1.397	0.513	0.503	1.396	0.491	0.453	1.093	0.508
9752	0.487	1.312	0.508	0.401	0.737	0.491	0.468	1.194	0.508
9753	0.508	1.447	0.185	0.506	1.415	0.273	0.482	1.279	0.467
9754	0.487	1.312	0.450	0.506	1.415	0.193	0.447	1.060	0.224
9755	0.487	1.312	0.501	0.506	1.415	0.512	0.479	1.262	0.467
9756	0.503	1.413	0.508	0.447	1.039	0.102	0.468	1.194	0.097

9757	0.474	1.228	0.501	0.512	1.453	0.235	0.479	1.262	0.467
9758	0.000	?	0.512	0.374	0.568	0.422	0.184	-0.625	0.142
9759	0.503	1.413	0.508	0.474	1.208	0.508	0.492	1.346	0.508
9760	0.503	1.413	0.508	0.430	0.926	0.273	0.479	1.262	0.185
9761	0.145	-0.878	0.050	0.102	-1.184	0.273	0.297	0.099	0.481
9762	0.500	1.397	0.050	0.515	1.472	0.149	0.463	1.161	0.481
9763	0.500	1.397	0.512	0.509	1.434	0.514	0.466	1.178	0.481
9764	0.479	1.262	0.512	0.485	1.283	0.341	0.463	1.161	0.481
9765	0.479	1.262	0.492	0.485	1.283	0.501	0.511	1.464	0.358
9766	0.479	1.262	0.512	0.485	1.283	0.501	0.463	1.161	0.481
9767	0.497	1.380	0.467	0.494	1.340	0.512	0.487	1.312	0.513
9768	0.145	-0.878	0.492	0.272	-0.091	0.512	0.361	0.504	0.262
9769	0.226	-0.356	0.501	0.196	-0.581	0.508	0.184	-0.625	0.262
9770	0.503	1.413	0.501	0.512	1.453	0.462	0.503	1.413	0.492
9771	0.142	-0.895	0.262	0.102	-1.184	0.508	0.297	0.099	0.492
9772	0.050	-1.485	0.097	0.234	-0.336	0.491	0.261	-0.137	0.481
9773	0.503	1.413	0.501	0.421	0.869	0.478	0.505	1.430	0.450
9774	0.466	1.178	0.508	0.506	1.415	0.341	0.511	1.464	0.501
9775	0.184	-0.625	0.431	0.278	-0.054	0.491	0.184	-0.625	0.467
9776	0.100	-1.164	0.185	0.275	-0.073	0.478	0.450	1.076	0.512
9777	0.263	-0.120	0.492	0.149	-0.882	0.053	0.226	-0.356	0.385
9778	0.511	1.464	0.512	0.515	1.472	0.149	0.508	1.447	0.508
9779	0.050	-1.485	0.224	0.339	0.342	0.193	0.142	-0.895	0.508
9780	0.505	1.430	0.142	0.500	1.378	0.371	0.468	1.194	0.508
9781	0.505	1.430	0.142	0.500	1.378	0.273	0.468	1.194	0.185
9782	0.492	1.346	0.492	0.512	1.453	0.512	0.505	1.430	0.185
9783	0.463	1.161	0.512	0.509	1.434	0.514	0.505	1.430	0.409
9784	0.466	1.178	0.512	0.506	1.415	0.235	0.479	1.262	0.385
9785	0.479	1.262	0.501	0.459	1.114	0.309	0.492	1.346	0.492
9786	0.463	1.161	0.501	0.503	1.396	0.462	0.497	1.380	0.492
9787	0.466	1.178	0.450	0.491	1.321	0.341	0.479	1.262	0.481
9788	0.516	1.498	0.481	0.474	1.208	0.193	0.326	0.285	0.450
9789	0.261	-0.137	0.501	0.342	0.361	0.512	0.263	-0.120	0.501
9790	0.482	1.279	0.050	0.494	1.340	0.422	0.432	0.958	0.501
9791	0.461	1.144	0.142	0.477	1.227	0.462	0.511	1.464	0.142
9792	0.224	-0.373	0.097	0.053	-1.504	0.501	0.361	0.504	0.262
9793	0.142	-0.895	0.481	0.149	-0.882	0.508	0.329	0.301	0.513
9794	0.326	0.285	0.513	0.193	-0.600	0.149	0.000	?	0.508
9795	0.050	-1.485	0.481	0.371	0.549	0.508	0.226	-0.356	0.513
9796	0.050	-1.485	0.481	0.272	-0.091	0.514	0.263	-0.120	0.097
9797	0.511	1.464	0.512	0.512	1.453	0.508	0.495	1.363	0.508
9798	0.511	1.464	0.492	0.515	1.472	0.508	0.495	1.363	0.512
9799	0.261	-0.137	0.481	0.234	-0.336	0.501	0.184	-0.625	0.262
9800	0.261	-0.137	0.501	0.310	0.153	0.514	0.263	-0.120	0.262
9801	0.447	1.060	0.512	0.465	1.152	0.102	0.505	1.430	0.501
9802	0.224	-0.373	0.513	0.345	0.379	0.102	0.226	-0.356	0.481
9803	0.184	-0.625	0.513	0.196	-0.581	0.508	0.226	-0.356	0.481
9804	0.466	1.178	0.508	0.518	1.491	0.512	0.511	1.464	0.481
9805	0.050	-1.485	0.501	0.421	0.869	0.309	0.000	?	0.512
9806	0.463	1.161	0.501	0.459	1.114	0.273	0.500	1.397	0.508
9807	0.468	1.194	0.481	0.494	1.340	0.462	0.466	1.178	0.467
9808	0.489	1.329	0.467	0.503	1.396	0.512	0.500	1.397	0.508
9809	0.332	0.318	0.467	0.149	-0.882	0.478	0.050	-1.485	0.185
9810	0.468	1.194	0.512	0.509	1.434	0.478	0.497	1.380	0.262
9811	0.482	1.279	0.512	0.512	1.453	0.193	0.497	1.380	0.262
9812	0.511	1.464	0.512	0.506	1.415	0.273	0.487	1.312	0.185
9813	0.511	1.464	0.185	0.506	1.415	0.444	0.487	1.312	0.501
9814	0.508	1.447	0.512	0.500	1.378	0.193	0.476	1.245	0.512
9815	0.503	1.413	0.501	0.518	1.491	0.462	0.466	1.178	0.501
9816	0.097	-1.181	0.450	0.102	-1.184	0.514	0.355	0.470	0.501
9817	0.097	-1.181	0.142	0.102	-1.184	0.501	0.326	0.285	0.450

9818	0.508	1.447	0.142	0.503	1.396	0.149	0.479	1.262	0.431
9819	0.500	1.397	0.385	0.506	1.415	0.193	0.468	1.194	0.492
9820	0.187	-0.608	0.224	0.310	0.153	0.508	0.384	0.655	0.450
9821	0.187	-0.608	0.508	0.275	-0.073	0.309	0.384	0.655	0.513
9822	0.505	1.430	0.512	0.462	1.133	0.501	0.445	1.043	0.409
9823	0.511	1.464	0.224	0.518	1.491	0.514	0.466	1.178	0.431
9824	0.500	1.397	0.508	0.474	1.208	0.341	0.508	1.447	0.512
9825	0.513	1.481	0.050	0.474	1.208	0.235	0.497	1.380	0.467
9826	0.224	-0.373	0.508	0.193	-0.600	0.478	0.184	-0.625	0.450
9827	0.332	0.318	0.467	0.275	-0.073	0.508	0.097	-1.181	0.512
9828	0.516	1.498	0.501	0.442	1.001	0.444	0.497	1.380	0.492
9829	0.263	-0.120	0.513	0.196	-0.581	0.444	0.097	-1.181	0.508
9830	0.489	1.329	0.262	0.462	1.133	0.149	0.513	1.481	0.508
9831	0.503	1.413	0.450	0.518	1.491	0.508	0.445	1.043	0.513
9832	0.513	1.481	0.512	0.497	1.359	0.341	0.445	1.043	0.508
9833	0.226	-0.356	0.512	0.149	-0.882	0.508	0.226	-0.356	0.262
9834	0.226	-0.356	0.501	0.193	-0.600	0.273	0.300	0.116	0.142
9835	0.500	1.397	0.050	0.506	1.415	0.508	0.453	1.093	0.262
9836	0.263	-0.120	0.467	0.310	0.153	0.273	0.411	0.824	0.481
9837	0.466	1.178	0.501	0.500	1.378	0.398	0.511	1.464	0.450
9838	0.482	1.279	0.508	0.520	1.509	0.398	0.408	0.807	0.450
9839	0.050	-1.485	0.097	0.342	0.361	0.491	0.226	-0.356	0.185
9840	0.142	-0.895	0.097	0.240	-0.299	0.512	0.224	-0.373	0.097
9841	0.489	1.329	0.097	0.477	1.227	0.508	0.497	1.380	0.431
9842	0.429	0.942	0.508	0.503	1.396	0.508	0.503	1.413	0.097
9843	0.503	1.413	0.185	0.444	1.020	0.501	0.503	1.413	0.481
9844	0.513	1.481	0.224	0.447	1.039	0.102	0.463	1.161	0.450
9845	0.261	-0.137	0.508	0.149	-0.882	0.235	0.226	-0.356	0.050
9846	0.453	1.093	0.501	0.506	1.415	0.512	0.463	1.161	0.513
9847	0.142	-0.895	0.501	0.345	0.379	0.512	0.097	-1.181	0.262
9848	0.445	1.043	0.512	0.509	1.434	0.512	0.463	1.161	0.409
9849	0.142	-0.895	0.508	0.278	-0.054	0.235	0.142	-0.895	0.512
9850	0.505	1.430	0.224	0.509	1.434	0.444	0.505	1.430	0.508
9851	0.142	-0.895	0.328	0.275	-0.073	0.501	0.187	-0.608	0.492
9852	0.495	1.363	0.508	0.398	0.718	0.491	0.474	1.228	0.262
9853	0.495	1.363	0.409	0.398	0.718	0.512	0.474	1.228	0.508
9854	0.503	1.413	0.501	0.491	1.321	0.478	0.479	1.262	0.481
9855	0.505	1.430	0.508	0.512	1.453	0.501	0.479	1.262	0.481
9856	0.505	1.430	0.492	0.509	1.434	0.501	0.358	0.487	0.492
9857	0.511	1.464	0.050	0.509	1.434	0.371	0.484	1.295	0.262
9858	0.466	1.178	0.508	0.503	1.396	0.422	0.511	1.464	0.512
9859	0.187	-0.608	0.224	0.102	-1.184	0.478	0.263	-0.120	0.501
9860	0.263	-0.120	0.142	0.234	-0.336	0.478	0.266	-0.103	0.508
9861	0.511	1.464	0.508	0.509	1.434	0.462	0.492	1.346	0.224
9862	0.511	1.464	0.050	0.509	1.434	0.462	0.492	1.346	0.224
9863	0.505	1.430	0.492	0.509	1.434	0.501	0.479	1.262	0.262
9864	0.184	-0.625	0.513	0.237	-0.317	0.478	0.447	1.060	0.512
9865	0.453	1.093	0.513	0.442	1.001	0.478	0.497	1.380	0.385
9866	0.500	1.397	0.142	0.497	1.359	0.478	0.466	1.178	0.296
9867	0.503	1.413	0.142	0.488	1.302	0.235	0.511	1.464	0.481
9868	0.497	1.380	0.296	0.515	1.472	0.193	0.384	0.655	0.467
9869	0.511	1.464	0.262	0.477	1.227	0.512	0.503	1.413	0.467
9870	0.500	1.397	0.142	0.500	1.378	0.422	0.511	1.464	0.501
9871	0.500	1.397	0.262	0.500	1.378	0.478	0.511	1.464	0.508
9872	0.050	-1.485	0.450	0.368	0.530	0.309	0.184	-0.625	0.050
9873	0.050	-1.485	0.501	0.424	0.888	0.491	0.187	-0.608	0.050
9874	0.508	1.447	0.501	0.480	1.246	0.514	0.405	0.790	0.492
9875	0.508	1.447	0.492	0.480	1.246	0.309	0.382	0.638	0.262
9876	0.489	1.329	0.492	0.459	1.114	0.149	0.487	1.312	0.224
9877	0.500	1.397	0.224	0.468	1.170	0.149	0.489	1.329	0.262
9878	0.468	1.194	0.185	0.503	1.396	0.462	0.482	1.279	0.501

9879	0.495	1.363	0.481	0.482	1.265	0.501	0.453	1.093	0.512
9880	0.503	1.413	0.508	0.477	1.227	0.501	0.505	1.430	0.492
9881	0.503	1.413	0.296	0.477	1.227	0.501	0.505	1.430	0.262
9882	0.263	-0.120	0.501	0.234	-0.336	0.478	0.142	-0.895	0.501
9883	0.097	-1.181	0.481	0.193	-0.600	0.444	0.266	-0.103	0.262
9884	0.492	1.346	0.224	0.515	1.472	0.501	0.513	1.481	0.296
9885	0.497	1.380	0.481	0.421	0.869	0.478	0.513	1.481	0.450
9886	0.429	0.942	0.512	0.477	1.227	0.341	0.513	1.481	0.262
9887	0.187	-0.608	0.328	0.307	0.135	0.398	0.097	-1.181	0.224
9888	0.492	1.346	0.467	0.497	1.359	0.422	0.508	1.447	0.512
9889	0.505	1.430	0.262	0.515	1.472	0.341	0.508	1.447	0.450
9890	0.224	-0.373	0.512	0.313	0.172	0.501	0.000	?	0.492
9891	0.142	-0.895	0.501	0.149	-0.882	0.462	0.261	-0.137	0.224
9892	0.142	-0.895	0.492	0.149	-0.882	0.478	0.261	-0.137	0.224
9893	0.484	1.295	0.385	0.462	1.133	0.478	0.503	1.413	0.097
9894	0.516	1.498	0.385	0.497	1.359	0.512	0.479	1.262	0.097
9895	0.516	1.498	0.185	0.497	1.359	0.508	0.479	1.262	0.224
9896	0.500	1.397	0.262	0.503	1.396	0.491	0.482	1.279	0.050
9897	0.500	1.397	0.262	0.471	1.189	0.512	0.511	1.464	0.501
9898	0.450	1.076	0.262	0.444	1.020	0.478	0.511	1.464	0.481
9899	0.482	1.279	0.431	0.503	1.396	0.508	0.463	1.161	0.513
9900	0.503	1.413	0.142	0.480	1.246	0.508	0.508	1.447	0.508
9901	0.050	-1.485	0.142	0.345	0.379	0.501	0.184	-0.625	0.508
9902	0.000	?	0.450	0.398	0.718	0.491	0.263	-0.120	0.142
9903	0.145	-0.878	0.513	0.421	0.869	0.512	0.263	-0.120	0.296
9904	0.097	-1.181	0.481	0.345	0.379	0.422	0.184	-0.625	0.467
9905	0.479	1.262	0.481	0.494	1.340	0.508	0.500	1.397	0.501
9906	0.511	1.464	0.512	0.459	1.114	0.491	0.508	1.447	0.097
9907	0.479	1.262	0.512	0.480	1.246	0.462	0.497	1.380	0.508
9908	0.479	1.262	0.513	0.480	1.246	0.512	0.497	1.380	0.450
9909	0.508	1.447	0.512	0.515	1.472	0.444	0.447	1.060	0.358
9910	0.489	1.329	0.513	0.512	1.453	0.102	0.429	0.942	0.492
9911	0.479	1.262	0.512	0.488	1.302	0.371	0.489	1.329	0.385
9912	0.500	1.397	0.224	0.512	1.453	0.371	0.447	1.060	0.358
9913	0.513	1.481	0.385	0.471	1.189	0.491	0.508	1.447	0.481
9914	0.503	1.413	0.512	0.509	1.434	0.512	0.408	0.807	0.328
9915	0.503	1.413	0.481	0.509	1.434	0.341	0.429	0.942	0.508
9916	0.497	1.380	0.481	0.500	1.378	0.309	0.516	1.498	0.512
9917	0.492	1.346	0.467	0.494	1.340	0.422	0.463	1.161	0.501
9918	0.492	1.346	0.097	0.515	1.472	0.501	0.447	1.060	0.512
9919	0.474	1.228	0.481	0.421	0.869	0.422	0.511	1.464	0.512
9920	0.463	1.161	0.492	0.509	1.434	0.478	0.492	1.346	0.262
9921	0.468	1.194	0.142	0.488	1.302	0.491	0.505	1.430	0.224
9922	0.511	1.464	0.492	0.459	1.114	0.309	0.503	1.413	0.328
9923	0.513	1.481	0.512	0.509	1.434	0.491	0.513	1.481	0.050
9924	0.513	1.481	0.142	0.447	1.039	0.309	0.503	1.413	0.513
9925	0.184	-0.625	0.224	0.102	-1.184	0.508	0.263	-0.120	0.142
9926	0.000	?	0.296	0.371	0.549	0.491	0.142	-0.895	0.296
9927	0.000	?	0.328	0.371	0.549	0.501	0.261	-0.137	0.467
9928	0.511	1.464	0.481	0.488	1.302	0.501	0.482	1.279	0.501
9929	0.500	1.397	0.467	0.512	1.453	0.149	0.450	1.076	0.492
9930	0.450	1.076	0.142	0.339	0.342	0.508	0.453	1.093	0.492
9931	0.142	-0.895	0.262	0.310	0.153	0.478	0.187	-0.608	0.481
9932	0.142	-0.895	0.142	0.424	0.888	0.512	0.097	-1.181	0.450
9933	0.384	0.655	0.224	0.500	1.378	0.501	0.432	0.958	0.508
9934	0.224	-0.373	0.409	0.424	0.888	0.273	0.097	-1.181	0.481
9935	0.503	1.413	0.185	0.480	1.246	0.462	0.476	1.245	0.501
9936	0.505	1.430	0.501	0.494	1.340	0.512	0.447	1.060	0.512
9937	0.224	-0.373	0.185	0.313	0.172	0.491	0.050	-1.485	0.224
9938	0.505	1.430	0.142	0.491	1.321	0.235	0.513	1.481	0.262
9939	0.050	-1.485	0.328	0.313	0.172	0.235	0.261	-0.137	0.512

9940	0.503	1.413	0.481	0.503	1.396	0.273	0.408	0.807	0.512
9941	0.466	1.178	0.262	0.491	1.321	0.501	0.516	1.498	0.512
9942	0.500	1.397	0.185	0.500	1.378	0.193	0.503	1.413	0.450
9943	0.500	1.397	0.185	0.503	1.396	0.149	0.495	1.363	0.262
9944	0.261	-0.137	0.492	0.149	-0.882	0.512	0.261	-0.137	0.492
9945	0.447	1.060	0.492	0.500	1.378	0.462	0.508	1.447	0.450
9946	0.500	1.397	0.296	0.480	1.246	0.512	0.482	1.279	0.409
9947	0.500	1.397	0.262	0.518	1.491	0.398	0.482	1.279	0.481
9948	0.492	1.346	0.481	0.497	1.359	0.512	0.500	1.397	0.501
9949	0.050	-1.485	0.501	0.275	-0.073	0.309	0.263	-0.120	0.142
9950	0.463	1.161	0.512	0.459	1.114	0.193	0.516	1.498	0.296
9951	0.492	1.346	0.501	0.518	1.491	0.508	0.500	1.397	0.262
9952	0.505	1.430	0.501	0.491	1.321	0.273	0.511	1.464	0.296
9953	0.097	-1.181	0.467	0.240	-0.299	0.309	0.224	-0.373	0.467
9954	0.097	-1.181	0.185	0.240	-0.299	0.273	0.224	-0.373	0.467
9955	0.097	-1.181	0.224	0.278	-0.054	0.508	0.261	-0.137	0.262
9956	0.497	1.380	0.431	0.506	1.415	0.508	0.508	1.447	0.185
9957	0.184	-0.625	0.358	0.196	-0.581	0.501	0.387	0.672	0.385
9958	0.224	-0.373	0.097	0.152	-0.863	0.235	0.297	0.099	0.185
9959	0.511	1.464	0.481	0.506	1.415	0.491	0.476	1.245	0.050
9960	0.492	1.346	0.508	0.462	1.133	0.193	0.466	1.178	0.224
9961	0.492	1.346	0.097	0.515	1.472	0.193	0.466	1.178	0.492
9962	0.503	1.413	0.501	0.398	0.718	0.512	0.503	1.413	0.097
9963	0.497	1.380	0.467	0.512	1.453	0.398	0.511	1.464	0.224
9964	0.224	-0.373	0.508	0.307	0.135	0.273	0.050	-1.485	0.185
9965	0.329	0.301	0.450	0.193	-0.600	0.514	0.050	-1.485	0.450
9966	0.508	1.447	0.513	0.509	1.434	0.512	0.487	1.312	0.142
9967	0.411	0.824	0.185	0.272	-0.091	0.501	0.261	-0.137	0.262
9968	0.000	?	0.142	0.310	0.153	0.371	0.226	-0.356	0.224
9969	0.000	?	0.142	0.272	-0.091	0.371	0.263	-0.120	0.501
9970	0.495	1.363	0.492	0.506	1.415	0.235	0.500	1.397	0.481
9971	0.503	1.413	0.097	0.509	1.434	0.235	0.511	1.464	0.185
9972	0.487	1.312	0.492	0.503	1.396	0.309	0.489	1.329	0.142
9973	0.050	-1.485	0.512	0.237	-0.317	0.273	0.263	-0.120	0.501
9974	0.505	1.430	0.508	0.491	1.321	0.501	0.500	1.397	0.431
9975	0.226	-0.356	0.508	0.193	-0.600	0.508	0.263	-0.120	0.450
9976	0.145	-0.878	0.508	0.193	-0.600	0.512	0.297	0.099	0.512
9977	0.505	1.430	0.501	0.509	1.434	0.491	0.450	1.076	0.501
9978	0.000	?	0.481	0.398	0.718	0.491	0.261	-0.137	0.481
9979	0.050	-1.485	0.296	0.272	-0.091	0.273	0.226	-0.356	0.296
9980	0.489	1.329	0.142	0.512	1.453	0.193	0.511	1.464	0.142
9981	0.503	1.413	0.481	0.515	1.472	0.512	0.447	1.060	0.296
9982	0.503	1.413	0.492	0.509	1.434	0.462	0.492	1.346	0.409
9983	0.142	-0.895	0.296	0.371	0.549	0.273	0.224	-0.373	0.328
9984	0.142	-0.895	0.296	0.371	0.549	0.501	0.224	-0.373	0.450
9985	0.297	0.099	0.512	0.234	-0.336	0.501	0.097	-1.181	0.513
9986	0.263	-0.120	0.185	0.234	-0.336	0.309	0.097	-1.181	0.142
9987	0.142	-0.895	0.328	0.307	0.135	0.512	0.224	-0.373	0.467
9988	0.261	-0.137	0.185	0.272	-0.091	0.514	0.050	-1.485	0.501
9989	0.450	1.076	0.431	0.503	1.396	0.235	0.503	1.413	0.450
9990	0.500	1.397	0.185	0.506	1.415	0.444	0.479	1.262	0.481
9991	0.495	1.363	0.185	0.515	1.472	0.235	0.511	1.464	0.467
9992	0.492	1.346	0.328	0.494	1.340	0.508	0.516	1.498	0.296
9993	0.492	1.346	0.185	0.494	1.340	0.422	0.516	1.498	0.296
9994	0.226	-0.356	0.296	0.275	-0.073	0.501	0.142	-0.895	0.328
9995	0.184	-0.625	0.142	0.196	-0.581	0.512	0.297	0.099	0.508
9996	0.482	1.279	0.296	0.512	1.453	0.512	0.463	1.161	0.185
9997	0.513	1.481	0.050	0.462	1.133	0.273	0.500	1.397	0.501
9998	0.297	0.099	0.185	0.275	-0.073	0.102	0.097	-1.181	0.501
9999	0.497	1.380	0.185	0.500	1.378	0.235	0.503	1.413	0.501
10000	0.474	1.228	0.385	0.497	1.359	0.235	0.453	1.093	0.513

10001	0.224	-0.373	0.508	0.313	0.172	0.444	0.361	0.504	0.501
10002	0.474	1.228	0.142	0.509	1.434	0.273	0.495	1.363	0.508
10003	0.500	1.397	0.185	0.518	1.491	0.102	0.384	0.655	0.262
10004	0.329	0.301	0.431	0.237	-0.317	0.501	0.361	0.504	0.501
10005	0.466	1.178	0.512	0.442	1.001	0.508	0.479	1.262	0.262
10006	0.263	-0.120	0.185	0.234	-0.336	0.514	0.332	0.318	0.296
10007	0.511	1.464	0.481	0.506	1.415	0.514	0.511	1.464	0.501
10008	0.500	1.397	0.508	0.427	0.907	0.478	0.513	1.481	0.409
10009	0.489	1.329	0.501	0.497	1.359	0.478	0.497	1.380	0.224
10010	0.384	0.655	0.501	0.512	1.453	0.501	0.513	1.481	0.296
10011	0.384	0.655	0.224	0.512	1.453	0.501	0.513	1.481	0.431
10012	0.184	-0.625	0.328	0.272	-0.091	0.508	0.263	-0.120	0.185
10013	0.263	-0.120	0.508	0.102	-1.184	0.444	0.226	-0.356	0.328
10014	0.261	-0.137	0.492	0.234	-0.336	0.341	0.332	0.318	0.224
10015	0.263	-0.120	0.262	0.237	-0.317	0.371	0.050	-1.485	0.512
10016	0.429	0.942	0.450	0.444	1.020	0.444	0.516	1.498	0.224
10017	0.142	-0.895	0.508	0.272	-0.091	0.514	0.142	-0.895	0.224
10018	0.142	-0.895	0.512	0.102	-1.184	0.514	0.295	0.082	0.142
10019	0.450	1.076	0.508	0.500	1.378	0.512	0.463	1.161	0.262
10020	0.513	1.481	0.501	0.506	1.415	0.235	0.495	1.363	0.358
10021	0.482	1.279	0.492	0.518	1.491	0.512	0.495	1.363	0.262
10022	0.482	1.279	0.501	0.518	1.491	0.508	0.495	1.363	0.296
10023	0.516	1.498	0.512	0.482	1.265	0.235	0.479	1.262	0.328
10024	0.516	1.498	0.185	0.482	1.265	0.491	0.447	1.060	0.409
10025	0.508	1.447	0.513	0.500	1.378	0.512	0.505	1.430	0.385
10026	0.511	1.464	0.385	0.500	1.378	0.341	0.484	1.295	0.142
10027	0.511	1.464	0.185	0.509	1.434	0.273	0.505	1.430	0.501
10028	0.508	1.447	0.481	0.447	1.039	0.309	0.518	1.514	0.262
10029	0.226	-0.356	0.296	0.339	0.342	0.235	0.226	-0.356	0.097
10030	0.389	0.689	0.513	0.368	0.530	0.444	0.261	-0.137	0.513
10031	0.511	1.464	0.512	0.447	1.039	0.444	0.516	1.498	0.492
10032	0.476	1.245	0.512	0.512	1.453	0.235	0.508	1.447	0.097
10033	0.476	1.245	0.296	0.512	1.453	0.444	0.508	1.447	0.512
10034	0.468	1.194	0.512	0.518	1.491	0.149	0.453	1.093	0.481
10035	0.097	-1.181	0.512	0.237	-0.317	0.341	0.263	-0.120	0.501
10036	0.484	1.295	0.512	0.509	1.434	0.149	0.489	1.329	0.501
10037	0.484	1.295	0.508	0.509	1.434	0.193	0.450	1.076	0.185
10038	0.142	-0.895	0.328	0.237	-0.317	0.501	0.411	0.824	0.142
10039	0.484	1.295	0.501	0.494	1.340	0.273	0.482	1.279	0.450
10040	0.508	1.447	0.508	0.509	1.434	0.235	0.500	1.397	0.431
10041	0.145	-0.878	0.481	0.342	0.361	0.149	0.142	-0.895	0.296
10042	0.226	-0.356	0.097	0.272	-0.091	0.462	0.295	0.082	0.512
10043	0.297	0.099	0.513	0.310	0.153	0.235	0.261	-0.137	0.450
10044	0.329	0.301	0.513	0.234	-0.336	0.102	0.295	0.082	0.467
10045	0.476	1.245	0.142	0.442	1.001	0.273	0.466	1.178	0.481
10046	0.463	1.161	0.142	0.442	1.001	0.491	0.466	1.178	0.492
10047	0.142	-0.895	0.050	0.234	-0.336	0.491	0.261	-0.137	0.512
10048	0.263	-0.120	0.142	0.442	1.001	0.102	0.184	-0.625	0.481
10049	0.142	-0.895	0.142	0.149	-0.882	0.309	0.384	0.655	0.481
10050	0.229	-0.339	0.142	0.342	0.361	0.491	0.184	-0.625	0.262
10051	0.408	0.807	0.262	0.149	-0.882	0.478	0.050	-1.485	0.501
10052	0.187	-0.608	0.296	0.193	-0.600	0.514	0.226	-0.356	0.513
10053	0.500	1.397	0.481	0.500	1.378	0.514	0.487	1.312	0.262
10054	0.184	-0.625	0.224	0.278	-0.054	0.478	0.097	-1.181	0.512
10055	0.142	-0.895	0.050	0.237	-0.317	0.444	0.224	-0.373	0.097
10056	0.329	0.301	0.296	0.152	-0.863	0.341	0.184	-0.625	0.492
10057	0.482	1.279	0.492	0.462	1.133	0.235	0.450	1.076	0.513
10058	0.261	-0.137	0.224	0.234	-0.336	0.398	0.142	-0.895	0.513
10059	0.187	-0.608	0.467	0.102	-1.184	0.341	0.261	-0.137	0.050
10060	0.187	-0.608	0.481	0.275	-0.073	0.149	0.224	-0.373	0.328
10061	0.495	1.363	0.512	0.491	1.321	0.508	0.497	1.380	0.409

10062	0.495	1.363	0.481	0.491	1.321	0.398	0.479	1.262	0.431
10063	0.300	0.116	0.050	0.102	-1.184	0.309	0.184	-0.625	0.467
10064	0.261	-0.137	0.224	0.307	0.135	0.491	0.142	-0.895	0.185
10065	0.476	1.245	0.185	0.488	1.302	0.508	0.497	1.380	0.409
10066	0.503	1.413	0.467	0.480	1.246	0.462	0.434	0.975	0.409
10067	0.508	1.447	0.467	0.506	1.415	0.501	0.453	1.093	0.481
10068	0.497	1.380	0.481	0.509	1.434	0.422	0.511	1.464	0.262
10069	0.503	1.413	0.481	0.474	1.208	0.193	0.500	1.397	0.501
10070	0.471	1.211	0.224	0.442	1.001	0.235	0.476	1.245	0.501
10071	0.187	-0.608	0.142	0.342	0.361	0.273	0.297	0.099	0.224
10072	0.226	-0.356	0.512	0.234	-0.336	0.508	0.142	-0.895	0.224
10073	0.429	0.942	0.501	0.398	0.718	0.273	0.300	0.116	0.224
10074	0.358	0.487	0.501	0.342	0.361	0.514	0.413	0.841	0.296
10075	0.097	-1.181	0.512	0.149	-0.882	0.478	0.329	0.301	0.262
10076	0.474	1.228	0.185	0.509	1.434	0.514	0.450	1.076	0.142
10077	0.505	1.430	0.296	0.398	0.718	0.478	0.511	1.464	0.142
10078	0.097	-1.181	0.296	0.310	0.153	0.501	0.142	-0.895	0.142
10079	0.497	1.380	0.328	0.488	1.302	0.512	0.471	1.211	0.492
10080	0.466	1.178	0.328	0.512	1.453	0.341	0.495	1.363	0.142
10081	0.508	1.447	0.481	0.462	1.133	0.508	0.453	1.093	0.224
10082	0.447	1.060	0.431	0.503	1.396	0.235	0.484	1.295	0.185
10083	0.513	1.481	0.224	0.424	0.888	0.193	0.466	1.178	0.492
10084	0.187	-0.608	0.224	0.193	-0.600	0.478	0.297	0.099	0.185
10085	0.145	-0.878	0.450	0.234	-0.336	0.491	0.297	0.099	0.512
10086	0.145	-0.878	0.501	0.272	-0.091	0.102	0.332	0.318	0.512
10087	0.484	1.295	0.513	0.512	1.453	0.102	0.503	1.413	0.481
10088	0.100	-1.164	0.450	0.275	-0.073	0.501	0.184	-0.625	0.492
10089	0.489	1.329	0.481	0.518	1.491	0.235	0.500	1.397	0.097
10090	0.511	1.464	0.481	0.480	1.246	0.341	0.492	1.346	0.296
10091	0.497	1.380	0.513	0.520	1.509	0.341	0.500	1.397	0.224
10092	0.497	1.380	0.512	0.480	1.246	0.462	0.513	1.481	0.467
10093	0.497	1.380	0.467	0.509	1.434	0.149	0.500	1.397	0.450
10094	0.492	1.346	0.185	0.518	1.491	0.149	0.511	1.464	0.481
10095	0.000	?	0.508	0.339	0.342	0.273	0.261	-0.137	0.481
10096	0.476	1.245	0.431	0.506	1.415	0.102	0.500	1.397	0.328
10097	0.297	0.099	0.185	0.237	-0.317	0.371	0.261	-0.137	0.185
10098	0.142	-0.895	0.508	0.196	-0.581	0.193	0.297	0.099	0.409
10099	0.479	1.262	0.492	0.482	1.265	0.398	0.500	1.397	0.450
10100	0.495	1.363	0.501	0.494	1.340	0.235	0.408	0.807	0.492
10101	0.297	0.099	0.501	0.102	-1.184	0.273	0.224	-0.373	0.467
10102	0.297	0.099	0.501	0.102	-1.184	0.102	0.297	0.099	0.328
10103	0.511	1.464	0.492	0.503	1.396	0.398	0.429	0.942	0.050
10104	0.184	-0.625	0.508	0.237	-0.317	0.508	0.184	-0.625	0.185
10105	0.329	0.301	0.508	0.339	0.342	0.309	0.332	0.318	0.224
10106	0.184	-0.625	0.492	0.345	0.379	0.341	0.226	-0.356	0.262
10107	0.432	0.958	0.142	0.459	1.114	0.508	0.511	1.464	0.481
10108	0.184	-0.625	0.050	0.149	-0.882	0.444	0.226	-0.356	0.492
10109	0.184	-0.625	0.431	0.149	-0.882	0.273	0.226	-0.356	0.296
10110	0.329	0.301	0.328	0.275	-0.073	0.508	0.145	-0.878	0.296
10111	0.184	-0.625	0.142	0.102	-1.184	0.514	0.263	-0.120	0.492
10112	0.295	0.082	0.501	0.374	0.568	0.512	0.361	0.504	0.513
10113	0.145	-0.878	0.501	0.193	-0.600	0.512	0.263	-0.120	0.512
10114	0.297	0.099	0.492	0.401	0.737	0.398	0.295	0.082	0.467
10115	0.050	-1.485	0.224	0.237	-0.317	0.149	0.326	0.285	0.492
10116	0.187	-0.608	0.262	0.275	-0.073	0.512	0.408	0.807	0.492
10117	0.184	-0.625	0.262	0.102	-1.184	0.501	0.384	0.655	0.467
10118	0.387	0.672	0.050	0.398	0.718	0.235	0.145	-0.878	0.492
10119	0.508	1.447	0.512	0.509	1.434	0.491	0.505	1.430	0.501
10120	0.142	-0.895	0.492	0.313	0.172	0.501	0.263	-0.120	0.296
10121	0.184	-0.625	0.501	0.345	0.379	0.462	0.097	-1.181	0.481
10122	0.429	0.942	0.185	0.509	1.434	0.512	0.513	1.481	0.481

10123	0.513	1.481	0.508	0.439	0.982	0.273	0.489	1.329	0.185
10124	0.184	-0.625	0.262	0.278	-0.054	0.508	0.097	-1.181	0.450
10125	0.476	1.245	0.142	0.509	1.434	0.512	0.508	1.447	0.501
10126	0.505	1.430	0.296	0.512	1.453	0.512	0.482	1.279	0.512
10127	0.497	1.380	0.467	0.520	1.509	0.309	0.505	1.430	0.512
10128	0.497	1.380	0.467	0.520	1.509	0.512	0.505	1.430	0.513
10129	0.226	-0.356	0.508	0.401	0.737	0.514	0.184	-0.625	0.508
10130	0.332	0.318	0.224	0.149	-0.882	0.341	0.142	-0.895	0.508
10131	0.500	1.397	0.224	0.509	1.434	0.478	0.450	1.076	0.512
10132	0.489	1.329	0.508	0.500	1.378	0.193	0.426	0.925	0.481
10133	0.261	-0.137	0.501	0.234	-0.336	0.478	0.297	0.099	0.185
10134	0.447	1.060	0.262	0.488	1.302	0.422	0.513	1.481	0.224
10135	0.505	1.430	0.501	0.500	1.378	0.422	0.450	1.076	0.512
10136	0.503	1.413	0.508	0.462	1.133	0.341	0.468	1.194	0.224
10137	0.503	1.413	0.097	0.512	1.453	0.508	0.479	1.262	0.358
10138	0.497	1.380	0.358	0.272	-0.091	0.508	0.489	1.329	0.450
10139	0.489	1.329	0.185	0.512	1.453	0.508	0.511	1.464	0.450
10140	0.503	1.413	0.467	0.512	1.453	0.512	0.479	1.262	0.481
10141	0.513	1.481	0.508	0.512	1.453	0.309	0.479	1.262	0.224
10142	0.187	-0.608	0.481	0.307	0.135	0.501	0.263	-0.120	0.409
10143	0.511	1.464	0.385	0.512	1.453	0.501	0.497	1.380	0.328
10144	0.387	0.672	0.385	0.518	1.491	0.478	0.508	1.447	0.262
10145	0.184	-0.625	0.142	0.342	0.361	0.193	0.263	-0.120	0.512
10146	0.479	1.262	0.492	0.485	1.283	0.491	0.508	1.447	0.467
10147	0.295	0.082	0.431	0.193	-0.600	0.491	0.097	-1.181	0.481
10148	0.511	1.464	0.328	0.474	1.208	0.309	0.492	1.346	0.142
10149	0.511	1.464	0.296	0.418	0.850	0.309	0.513	1.481	0.492
10150	0.511	1.464	0.431	0.418	0.850	0.341	0.513	1.481	0.142
10151	0.297	0.099	0.481	0.339	0.342	0.273	0.050	-1.485	0.224
10152	0.511	1.464	0.492	0.506	1.415	0.235	0.326	0.285	0.142
10153	0.511	1.464	0.262	0.506	1.415	0.273	0.405	0.790	0.467
10154	0.511	1.464	0.501	0.515	1.472	0.149	0.426	0.925	0.481
10155	0.508	1.447	0.501	0.512	1.453	0.102	0.468	1.194	0.450
10156	0.329	0.301	0.224	0.307	0.135	0.491	0.184	-0.625	0.328
10157	0.503	1.413	0.481	0.500	1.378	0.371	0.408	0.807	0.185
10158	0.508	1.447	0.224	0.500	1.378	0.398	0.408	0.807	0.431
10159	0.476	1.245	0.296	0.480	1.246	0.235	0.476	1.245	0.450
10160	0.097	-1.181	0.431	0.193	-0.600	0.508	0.261	-0.137	0.142
10161	0.508	1.447	0.431	0.491	1.321	0.193	0.497	1.380	0.481
10162	0.508	1.447	0.450	0.491	1.321	0.514	0.497	1.380	0.481
10163	0.142	-0.895	0.262	0.310	0.153	0.514	0.224	-0.373	0.385
10164	0.142	-0.895	0.508	0.313	0.172	0.462	0.224	-0.373	0.050
10165	0.050	-1.485	0.508	0.342	0.361	0.508	0.224	-0.373	0.224
10166	0.142	-0.895	0.262	0.278	-0.054	0.371	0.295	0.082	0.501
10167	0.142	-0.895	0.450	0.240	-0.299	0.193	0.261	-0.137	0.508
10168	0.145	-0.878	0.328	0.275	-0.073	0.149	0.142	-0.895	0.501
10169	0.263	-0.120	0.262	0.149	-0.882	0.512	0.142	-0.895	0.501
10170	0.297	0.099	0.097	0.102	-1.184	0.512	0.142	-0.895	0.501
10171	0.479	1.262	0.296	0.491	1.321	0.501	0.484	1.295	0.185
10172	0.226	-0.356	0.481	0.371	0.549	0.501	0.142	-0.895	0.450
10173	0.050	-1.485	0.492	0.398	0.718	0.341	0.224	-0.373	0.481
10174	0.300	0.116	0.501	0.234	-0.336	0.235	0.184	-0.625	0.097
10175	0.489	1.329	0.501	0.506	1.415	0.462	0.487	1.312	0.097
10176	0.226	-0.356	0.097	0.193	-0.600	0.491	0.184	-0.625	0.097
10177	0.463	1.161	0.501	0.515	1.472	0.512	0.511	1.464	0.492
10178	0.476	1.245	0.513	0.515	1.472	0.478	0.511	1.464	0.492
10179	0.508	1.447	0.481	0.465	1.152	0.102	0.474	1.228	0.185
10180	0.482	1.279	0.513	0.506	1.415	0.235	0.487	1.312	0.508
10181	0.050	-1.485	0.185	0.368	0.530	0.053	0.097	-1.181	0.450
10182	0.224	-0.373	0.501	0.196	-0.581	0.371	0.297	0.099	0.358
10183	0.187	-0.608	0.467	0.149	-0.882	0.273	0.226	-0.356	0.501

10184	0.463	1.161	0.512	0.509	1.434	0.508	0.463	1.161	0.185
10185	0.466	1.178	0.481	0.509	1.434	0.491	0.447	1.060	0.262
10186	0.482	1.279	0.262	0.497	1.359	0.273	0.476	1.245	0.481
10187	0.482	1.279	0.224	0.497	1.359	0.273	0.476	1.245	0.481
10188	0.224	-0.373	0.262	0.345	0.379	0.501	0.326	0.285	0.492
10189	0.142	-0.895	0.431	0.237	-0.317	0.512	0.187	-0.608	0.262
10190	0.516	1.498	0.481	0.468	1.170	0.462	0.408	0.807	0.296
10191	0.495	1.363	0.492	0.494	1.340	0.514	0.455	1.110	0.296
10192	0.495	1.363	0.481	0.515	1.472	0.462	0.492	1.346	0.142
10193	0.503	1.413	0.262	0.480	1.246	0.462	0.471	1.211	0.450
10194	0.187	-0.608	0.431	0.102	-1.184	0.491	0.329	0.301	0.328
10195	0.295	0.082	0.481	0.237	-0.317	0.508	0.050	-1.485	0.142
10196	0.297	0.099	0.385	0.053	-1.504	0.512	0.187	-0.608	0.262
10197	0.326	0.285	0.450	0.377	0.587	0.193	0.226	-0.356	0.142
10198	0.329	0.301	0.224	0.272	-0.091	0.444	0.261	-0.137	0.492
10199	0.476	1.245	0.296	0.503	1.396	0.508	0.482	1.279	0.508
10200	0.434	0.975	0.512	0.488	1.302	0.235	0.487	1.312	0.512
10201	0.224	-0.373	0.409	0.275	-0.073	0.508	0.300	0.116	0.501
10202	0.224	-0.373	0.508	0.275	-0.073	0.508	0.300	0.116	0.185
10203	0.450	1.076	0.501	0.497	1.359	0.514	0.487	1.312	0.501
10204	0.495	1.363	0.296	0.515	1.472	0.514	0.513	1.481	0.508
10205	0.508	1.447	0.296	0.465	1.152	0.514	0.511	1.464	0.508
10206	0.453	1.093	0.481	0.509	1.434	0.478	0.461	1.144	0.450
10207	0.482	1.279	0.492	0.459	1.114	0.491	0.489	1.329	0.224
10208	0.482	1.279	0.385	0.459	1.114	0.508	0.489	1.329	0.467
10209	0.516	1.498	0.501	0.494	1.340	0.514	0.466	1.178	0.501
10210	0.505	1.430	0.224	0.506	1.415	0.235	0.495	1.363	0.508
10211	0.466	1.178	0.501	0.518	1.491	0.273	0.503	1.413	0.467
10212	0.184	-0.625	0.262	0.196	-0.581	0.371	0.297	0.099	0.097
10213	0.505	1.430	0.450	0.447	1.039	0.309	0.476	1.245	0.050
10214	0.432	0.958	0.492	0.509	1.434	0.193	0.476	1.245	0.185
10215	0.184	-0.625	0.185	0.237	-0.317	0.512	0.187	-0.608	0.501
10216	0.500	1.397	0.185	0.512	1.453	0.512	0.450	1.076	0.450
10217	0.484	1.295	0.501	0.512	1.453	0.508	0.505	1.430	0.492
10218	0.503	1.413	0.185	0.509	1.434	0.341	0.511	1.464	0.513
10219	0.503	1.413	0.508	0.509	1.434	0.235	0.511	1.464	0.097
10220	0.503	1.413	0.358	0.509	1.434	0.102	0.511	1.464	0.492
10221	0.489	1.329	0.409	0.482	1.265	0.371	0.508	1.447	0.512
10222	0.500	1.397	0.185	0.488	1.302	0.501	0.508	1.447	0.185
10223	0.508	1.447	0.142	0.506	1.415	0.478	0.513	1.481	0.508
10224	0.495	1.363	0.262	0.515	1.472	0.462	0.476	1.245	0.224
10225	0.142	-0.895	0.185	0.237	-0.317	0.235	0.184	-0.625	0.185
10226	0.050	-1.485	0.185	0.272	-0.091	0.508	0.224	-0.373	0.409
10227	0.429	0.942	0.185	0.377	0.587	0.309	0.511	1.464	0.501
10228	0.332	0.318	0.492	0.307	0.135	0.193	0.226	-0.356	0.501
10229	0.142	-0.895	0.185	0.193	-0.600	0.193	0.358	0.487	0.431
10230	0.505	1.430	0.501	0.515	1.472	0.512	0.453	1.093	0.262
10231	0.495	1.363	0.501	0.512	1.453	0.478	0.453	1.093	0.262
10232	0.487	1.312	0.481	0.506	1.415	0.462	0.484	1.295	0.409
10233	0.224	-0.373	0.467	0.342	0.361	0.149	0.224	-0.373	0.492
10234	0.263	-0.120	0.385	0.234	-0.336	0.149	0.411	0.824	0.224
10235	0.261	-0.137	0.224	0.102	-1.184	0.422	0.329	0.301	0.185
10236	0.050	-1.485	0.224	0.374	0.568	0.444	0.263	-0.120	0.385
10237	0.508	1.447	0.409	0.497	1.359	0.193	0.513	1.481	0.142
10238	0.492	1.346	0.508	0.477	1.227	0.501	0.463	1.161	0.501
10239	0.505	1.430	0.142	0.468	1.170	0.501	0.482	1.279	0.296
10240	0.187	-0.608	0.481	0.234	-0.336	0.235	0.145	-0.878	0.492
10241	0.505	1.430	0.224	0.503	1.396	0.149	0.489	1.329	0.512
10242	0.263	-0.120	0.224	0.313	0.172	0.149	0.142	-0.895	0.185
10243	0.142	-0.895	0.512	0.193	-0.600	0.341	0.224	-0.373	0.142
10244	0.297	0.099	0.508	0.193	-0.600	0.491	0.145	-0.878	0.467

10245	0.463	1.161	0.328	0.515	1.472	0.512	0.463	1.161	0.385
10246	0.468	1.194	0.296	0.482	1.265	0.491	0.479	1.262	0.501
10247	0.511	1.464	0.358	0.465	1.152	0.491	0.447	1.060	0.185
10248	0.224	-0.373	0.296	0.149	-0.882	0.235	0.329	0.301	0.224
10249	0.224	-0.373	0.262	0.149	-0.882	0.444	0.184	-0.625	0.224
10250	0.511	1.464	0.467	0.418	0.850	0.462	0.432	0.958	0.409
10251	0.505	1.430	0.262	0.439	0.982	0.371	0.450	1.076	0.328
10252	0.261	-0.137	0.492	0.193	-0.600	0.341	0.142	-0.895	0.142
10253	0.503	1.413	0.185	0.503	1.396	0.309	0.479	1.262	0.142
10254	0.511	1.464	0.142	0.503	1.396	0.514	0.476	1.245	0.097
10255	0.097	-1.181	0.142	0.240	-0.299	0.444	0.384	0.655	0.431
10256	0.358	0.487	0.142	0.149	-0.882	0.309	0.050	-1.485	0.224
10257	0.184	-0.625	0.512	0.149	-0.882	0.491	0.224	-0.373	0.492
10258	0.468	1.194	0.467	0.339	0.342	0.491	0.500	1.397	0.481
10259	0.505	1.430	0.050	0.494	1.340	0.235	0.508	1.447	0.481
10260	0.482	1.279	0.512	0.512	1.453	0.491	0.497	1.380	0.501
10261	0.382	0.638	0.185	0.494	1.340	0.193	0.495	1.363	0.492
10262	0.382	0.638	0.097	0.494	1.340	0.149	0.495	1.363	0.385
10263	0.142	-0.895	0.185	0.234	-0.336	0.508	0.184	-0.625	0.385
10264	0.489	1.329	0.450	0.444	1.020	0.508	0.447	1.060	0.492
10265	0.429	0.942	0.224	0.462	1.133	0.491	0.476	1.245	0.467
10266	0.326	0.285	0.501	0.368	0.530	0.149	0.097	-1.181	0.224
10267	0.295	0.082	0.512	0.339	0.342	0.491	0.097	-1.181	0.467
10268	0.426	0.925	0.224	0.307	0.135	0.501	0.097	-1.181	0.097
10269	0.484	1.295	0.481	0.512	1.453	0.341	0.495	1.363	0.262
10270	0.495	1.363	0.512	0.439	0.982	0.341	0.492	1.346	0.492
10271	0.261	-0.137	0.492	0.307	0.135	0.235	0.187	-0.608	0.501
10272	0.503	1.413	0.467	0.491	1.321	0.193	0.508	1.447	0.431
10273	0.500	1.397	0.050	0.494	1.340	0.235	0.447	1.060	0.431
10274	0.226	-0.356	0.224	0.237	-0.317	0.193	0.358	0.487	0.142
10275	0.479	1.262	0.385	0.491	1.321	0.512	0.495	1.363	0.097
10276	0.224	-0.373	0.224	0.196	-0.581	0.508	0.184	-0.625	0.142
10277	0.300	0.116	0.142	0.149	-0.882	0.478	0.261	-0.137	0.358
10278	0.429	0.942	0.142	0.506	1.415	0.514	0.482	1.279	0.185
10279	0.429	0.942	0.328	0.506	1.415	0.273	0.482	1.279	0.508
10280	0.450	1.076	0.224	0.494	1.340	0.309	0.487	1.312	0.358
10281	0.261	-0.137	0.224	0.149	-0.882	0.371	0.263	-0.120	0.358
10282	0.503	1.413	0.501	0.494	1.340	0.508	0.295	0.082	0.358
10283	0.503	1.413	0.097	0.503	1.396	0.501	0.295	0.082	0.185
10284	0.261	-0.137	0.467	0.345	0.379	0.193	0.142	-0.895	0.185
10285	0.450	1.076	0.501	0.345	0.379	0.422	0.447	1.060	0.185
10286	0.329	0.301	0.409	0.237	-0.317	0.501	0.329	0.301	0.409
10287	0.263	-0.120	0.224	0.196	-0.581	0.514	0.142	-0.895	0.467
10288	0.097	-1.181	0.328	0.237	-0.317	0.478	0.263	-0.120	0.328
10289	0.297	0.099	0.142	0.196	-0.581	0.341	0.142	-0.895	0.296
10290	0.484	1.295	0.142	0.506	1.415	0.309	0.489	1.329	0.224
10291	0.489	1.329	0.508	0.506	1.415	0.273	0.503	1.413	0.481
10292	0.500	1.397	0.508	0.474	1.208	0.462	0.508	1.447	0.224
10293	0.500	1.397	0.508	0.509	1.434	0.491	0.500	1.397	0.262
10294	0.097	-1.181	0.262	0.272	-0.091	0.501	0.184	-0.625	0.328
10295	0.505	1.430	0.328	0.307	0.135	0.444	0.495	1.363	0.512
10296	0.516	1.498	0.481	0.371	0.549	0.193	0.500	1.397	0.262
10297	0.479	1.262	0.481	0.512	1.453	0.444	0.505	1.430	0.481
10298	0.511	1.464	0.296	0.500	1.378	0.462	0.445	1.043	0.450
10299	0.184	-0.625	0.050	0.196	-0.581	0.102	0.224	-0.373	0.142
10300	0.497	1.380	0.050	0.421	0.869	0.491	0.463	1.161	0.512
10301	0.466	1.178	0.262	0.506	1.415	0.273	0.497	1.380	0.513
10302	0.500	1.397	0.431	0.518	1.491	0.102	0.508	1.447	0.512
10303	0.479	1.262	0.142	0.477	1.227	0.462	0.463	1.161	0.450
10304	0.263	-0.120	0.262	0.342	0.361	0.462	0.097	-1.181	0.224
10305	0.229	-0.339	0.512	0.307	0.135	0.341	0.050	-1.485	0.142

10306	0.263	-0.120	0.097	0.275	-0.073	0.508	0.184	-0.625	0.501
10307	0.434	0.975	0.097	0.462	1.133	0.053	0.500	1.397	0.296
10308	0.484	1.295	0.467	0.494	1.340	0.102	0.447	1.060	0.262
10309	0.489	1.329	0.097	0.503	1.396	0.512	0.492	1.346	0.409
10310	0.476	1.245	0.224	0.444	1.020	0.444	0.513	1.481	0.262
10311	0.266	-0.103	0.185	0.193	-0.600	0.309	0.097	-1.181	0.431
10312	0.426	0.925	0.481	0.442	1.001	0.235	0.495	1.363	0.296
10313	0.479	1.262	0.142	0.465	1.152	0.398	0.511	1.464	0.431
10314	0.389	0.689	0.409	0.102	-1.184	0.235	0.184	-0.625	0.224
10315	0.453	1.093	0.409	0.485	1.283	0.478	0.503	1.413	0.492
10316	0.224	-0.373	0.409	0.278	-0.054	0.309	0.224	-0.373	0.450
10317	0.297	0.099	0.185	0.102	-1.184	0.398	0.184	-0.625	0.262
10318	0.511	1.464	0.512	0.462	1.133	0.508	0.408	0.807	0.296
10319	0.413	0.841	0.512	0.465	1.152	0.341	0.497	1.380	0.142
10320	0.508	1.447	0.224	0.342	0.361	0.309	0.497	1.380	0.262
10321	0.500	1.397	0.224	0.509	1.434	0.501	0.429	0.942	0.224
10322	0.300	0.116	0.508	0.053	-1.504	0.309	0.261	-0.137	0.513
10323	0.300	0.116	0.512	0.102	-1.184	0.491	0.261	-0.137	0.513
10324	0.479	1.262	0.508	0.509	1.434	0.149	0.408	0.807	0.513
10325	0.495	1.363	0.513	0.444	1.020	0.149	0.495	1.363	0.224
10326	0.000	?	0.097	0.310	0.153	0.273	0.226	-0.356	0.328
10327	0.384	0.655	0.097	0.237	-0.317	0.341	0.187	-0.608	0.513
10328	0.495	1.363	0.328	0.398	0.718	0.193	0.384	0.655	0.467
10329	0.224	-0.373	0.262	0.237	-0.317	0.341	0.142	-0.895	0.097
10330	0.505	1.430	0.501	0.480	1.246	0.341	0.500	1.397	0.296
10331	0.263	-0.120	0.513	0.310	0.153	0.273	0.297	0.099	0.185
10332	0.453	1.093	0.492	0.398	0.718	0.491	0.492	1.346	0.262
10333	0.489	1.329	0.262	0.512	1.453	0.273	0.508	1.447	0.501
10334	0.184	-0.625	0.501	0.345	0.379	0.514	0.187	-0.608	0.224
10335	0.184	-0.625	0.512	0.310	0.153	0.514	0.145	-0.878	0.296
10336	0.505	1.430	0.467	0.500	1.378	0.491	0.463	1.161	0.467
10337	0.184	-0.625	0.450	0.310	0.153	0.462	0.389	0.689	0.224
10338	0.505	1.430	0.467	0.494	1.340	0.491	0.505	1.430	0.185
10339	0.358	0.487	0.142	0.149	-0.882	0.341	0.187	-0.608	0.450
10340	0.408	0.807	0.185	0.149	-0.882	0.341	0.226	-0.356	0.185
10341	0.187	-0.608	0.224	0.275	-0.073	0.501	0.224	-0.373	0.224
10342	0.142	-0.895	0.224	0.342	0.361	0.478	0.411	0.824	0.224
10343	0.268	-0.086	0.097	0.193	-0.600	0.235	0.326	0.285	0.296
10344	0.187	-0.608	0.513	0.342	0.361	0.512	0.142	-0.895	0.492
10345	0.187	-0.608	0.467	0.342	0.361	0.273	0.142	-0.895	0.142
10346	0.187	-0.608	0.262	0.272	-0.091	0.235	0.097	-1.181	0.224
10347	0.495	1.363	0.481	0.488	1.302	0.501	0.426	0.925	0.185
10348	0.184	-0.625	0.467	0.275	-0.073	0.491	0.224	-0.373	0.492
10349	0.495	1.363	0.492	0.518	1.491	0.149	0.489	1.329	0.262
10350	0.495	1.363	0.142	0.518	1.491	0.053	0.479	1.262	0.492
10351	0.474	1.228	0.508	0.494	1.340	0.235	0.479	1.262	0.512
10352	0.463	1.161	0.508	0.462	1.133	0.235	0.503	1.413	0.328
10353	0.382	0.638	0.450	0.491	1.321	0.273	0.492	1.346	0.328
10354	0.224	-0.373	0.409	0.342	0.361	0.491	0.384	0.655	0.508
10355	0.224	-0.373	0.097	0.342	0.361	0.149	0.384	0.655	0.513
10356	0.405	0.790	0.142	0.500	1.378	0.508	0.489	1.329	0.512
10357	0.503	1.413	0.481	0.480	1.246	0.273	0.461	1.144	0.431
10358	0.142	-0.895	0.512	0.237	-0.317	0.309	0.224	-0.373	0.097
10359	0.479	1.262	0.501	0.512	1.453	0.309	0.466	1.178	0.142
10360	0.226	-0.356	0.358	0.272	-0.091	0.341	0.097	-1.181	0.385
10361	0.226	-0.356	0.467	0.234	-0.336	0.341	0.261	-0.137	0.328
10362	0.500	1.397	0.492	0.503	1.396	0.309	0.495	1.363	0.481
10363	0.503	1.413	0.513	0.491	1.321	0.491	0.500	1.397	0.481
10364	0.326	0.285	0.512	0.149	-0.882	0.512	0.429	0.942	0.481
10365	0.295	0.082	0.296	0.053	-1.504	0.309	0.429	0.942	0.185
10366	0.355	0.470	0.262	0.234	-0.336	0.341	0.142	-0.895	0.492

10367	0.297	0.099	0.508	0.237	-0.317	0.309	0.097	-1.181	0.467
10368	0.261	-0.137	0.512	0.272	-0.091	0.478	0.142	-0.895	0.467
10369	0.468	1.194	0.512	0.494	1.340	0.235	0.355	0.470	0.467
10370	0.261	-0.137	0.262	0.149	-0.882	0.193	0.184	-0.625	0.492
10371	0.492	1.346	0.185	0.515	1.472	0.053	0.505	1.430	0.358
10372	0.184	-0.625	0.185	0.272	-0.091	0.508	0.355	0.470	0.296
10373	0.000	?	0.512	0.313	0.172	0.422	0.358	0.487	0.142
10374	0.000	?	0.508	0.313	0.172	0.512	0.358	0.487	0.142
10375	0.142	-0.895	0.142	0.342	0.361	0.462	0.184	-0.625	0.409
10376	0.142	-0.895	0.508	0.342	0.361	0.193	0.184	-0.625	0.409
10377	0.142	-0.895	0.492	0.310	0.153	0.491	0.184	-0.625	0.492
10378	0.511	1.464	0.467	0.494	1.340	0.444	0.411	0.824	0.262
10379	0.461	1.144	0.492	0.518	1.491	0.501	0.463	1.161	0.492
10380	0.000	?	0.185	0.310	0.153	0.491	0.329	0.301	0.492
10381	0.000	?	0.481	0.345	0.379	0.398	0.295	0.082	0.481
10382	0.050	-1.485	0.467	0.310	0.153	0.273	0.226	-0.356	0.358
10383	0.511	1.464	0.185	0.480	1.246	0.491	0.482	1.279	0.409
10384	0.184	-0.625	0.492	0.234	-0.336	0.235	0.224	-0.373	0.431
10385	0.097	-1.181	0.450	0.193	-0.600	0.309	0.261	-0.137	0.512
10386	0.184	-0.625	0.508	0.053	-1.504	0.193	0.329	0.301	0.501
10387	0.445	1.043	0.512	0.512	1.453	0.193	0.508	1.447	0.328
10388	0.224	-0.373	0.513	0.424	0.888	0.235	0.261	-0.137	0.328
10389	0.503	1.413	0.262	0.515	1.472	0.193	0.482	1.279	0.492
10390	0.508	1.447	0.262	0.465	1.152	0.422	0.447	1.060	0.481
10391	0.224	-0.373	0.185	0.193	-0.600	0.371	0.142	-0.895	0.481
10392	0.487	1.312	0.185	0.488	1.302	0.508	0.505	1.430	0.097
10393	0.500	1.397	0.097	0.450	1.057	0.512	0.518	1.514	0.492
10394	0.492	1.346	0.328	0.506	1.415	0.149	0.511	1.464	0.513
10395	0.463	1.161	0.501	0.491	1.321	0.149	0.447	1.060	0.481
10396	0.050	-1.485	0.097	0.398	0.718	0.444	0.224	-0.373	0.492
10397	0.224	-0.373	0.481	0.275	-0.073	0.193	0.142	-0.895	0.185
10398	0.382	0.638	0.185	0.491	1.321	0.193	0.500	1.397	0.185
10399	0.226	-0.356	0.050	0.234	-0.336	0.491	0.300	0.116	0.492
10400	0.142	-0.895	0.501	0.313	0.172	0.491	0.263	-0.120	0.512
10401	0.142	-0.895	0.185	0.193	-0.600	0.491	0.411	0.824	0.492
10402	0.326	0.285	0.328	0.196	-0.581	0.149	0.266	-0.103	0.097
10403	0.224	-0.373	0.296	0.237	-0.317	0.444	0.432	0.958	0.358
10404	0.224	-0.373	0.501	0.196	-0.581	0.422	0.300	0.116	0.385
10405	0.500	1.397	0.224	0.421	0.869	0.514	0.426	0.925	0.431
10406	0.097	-1.181	0.358	0.371	0.549	0.149	0.226	-0.356	0.481
10407	0.463	1.161	0.512	0.503	1.396	0.341	0.492	1.346	0.142
10408	0.500	1.397	0.501	0.518	1.491	0.309	0.455	1.110	0.501
10409	0.413	0.841	0.050	0.149	-0.882	0.102	0.261	-0.137	0.481
10410	0.224	-0.373	0.097	0.149	-0.882	0.371	0.295	0.082	0.492
10411	0.332	0.318	0.467	0.442	1.001	0.309	0.142	-0.895	0.512
10412	0.142	-0.895	0.492	0.193	-0.600	0.273	0.261	-0.137	0.224
10413	0.142	-0.895	0.142	0.193	-0.600	0.478	0.224	-0.373	0.508
10414	0.508	1.447	0.142	0.491	1.321	0.341	0.513	1.481	0.492
10415	0.508	1.447	0.185	0.491	1.321	0.273	0.513	1.481	0.185
10416	0.508	1.447	0.512	0.491	1.321	0.444	0.513	1.481	0.467
10417	0.261	-0.137	0.492	0.149	-0.882	0.444	0.226	-0.356	0.508
10418	0.332	0.318	0.481	0.447	1.039	0.398	0.326	0.285	0.501
10419	0.476	1.245	0.481	0.421	0.869	0.309	0.511	1.464	0.431
10420	0.474	1.228	0.097	0.515	1.472	0.149	0.466	1.178	0.450
10421	0.300	0.116	0.224	0.149	-0.882	0.508	0.097	-1.181	0.262
10422	0.050	-1.485	0.262	0.339	0.342	0.273	0.297	0.099	0.262
10423	0.050	-1.485	0.262	0.307	0.135	0.341	0.187	-0.608	0.224
10424	0.263	-0.120	0.185	0.102	-1.184	0.341	0.261	-0.137	0.142
10425	0.432	0.958	0.185	0.374	0.568	0.512	0.500	1.397	0.296
10426	0.142	-0.895	0.508	0.307	0.135	0.235	0.226	-0.356	0.328
10427	0.263	-0.120	0.508	0.272	-0.091	0.514	0.295	0.082	0.481

10428	0.500	1.397	0.185	0.480	1.246	0.444	0.468	1.194	0.185
10429	0.097	-1.181	0.492	0.342	0.361	0.273	0.224	-0.373	0.512
10430	0.097	-1.181	0.262	0.272	-0.091	0.273	0.184	-0.625	0.409
10431	0.463	1.161	0.501	0.447	1.039	0.491	0.450	1.076	0.185
10432	0.100	-1.164	0.358	0.442	1.001	0.371	0.184	-0.625	0.467
10433	0.000	?	0.224	0.401	0.737	0.514	0.224	-0.373	0.142
10434	0.226	-0.356	0.481	0.313	0.172	0.444	0.224	-0.373	0.097
10435	0.184	-0.625	0.262	0.149	-0.882	0.371	0.297	0.099	0.513
10436	0.479	1.262	0.450	0.512	1.453	0.444	0.492	1.346	0.050
10437	0.142	-0.895	0.185	0.278	-0.054	0.193	0.142	-0.895	0.224
10438	0.411	0.824	0.492	0.339	0.342	0.341	0.224	-0.373	0.097
10439	0.411	0.824	0.467	0.339	0.342	0.508	0.184	-0.625	0.467
10440	0.411	0.824	0.492	0.506	1.415	0.512	0.500	1.397	0.481
10441	0.187	-0.608	0.450	0.234	-0.336	0.508	0.263	-0.120	0.185
10442	0.513	1.481	0.481	0.518	1.491	0.422	0.495	1.363	0.224
10443	0.511	1.464	0.513	0.447	1.039	0.501	0.511	1.464	0.358
10444	0.224	-0.373	0.097	0.272	-0.091	0.444	0.332	0.318	0.501
10445	0.224	-0.373	0.409	0.272	-0.091	0.501	0.332	0.318	0.142
10446	0.503	1.413	0.385	0.488	1.302	0.462	0.508	1.447	0.358
10447	0.503	1.413	0.262	0.377	0.587	0.462	0.516	1.498	0.224
10448	0.511	1.464	0.262	0.518	1.491	0.193	0.516	1.498	0.481
10449	0.513	1.481	0.224	0.444	1.020	0.341	0.432	0.958	0.492
10450	0.097	-1.181	0.262	0.374	0.568	0.309	0.097	-1.181	0.512
10451	0.097	-1.181	0.512	0.447	1.039	0.398	0.142	-0.895	0.513
10452	0.332	0.318	0.512	0.193	-0.600	0.309	0.384	0.655	0.262
10453	0.263	-0.120	0.492	0.342	0.361	0.478	0.329	0.301	0.142
10454	0.505	1.430	0.492	0.503	1.396	0.512	0.479	1.262	0.142
10455	0.518	1.514	0.450	0.509	1.434	0.149	0.479	1.262	0.296
10456	0.495	1.363	0.450	0.503	1.396	0.309	0.482	1.279	0.262
10457	0.261	-0.137	0.185	0.430	0.926	0.462	0.184	-0.625	0.328
10458	0.497	1.380	0.513	0.500	1.378	0.491	0.487	1.312	0.431
10459	0.503	1.413	0.185	0.442	1.001	0.193	0.468	1.194	0.467
10460	0.468	1.194	0.481	0.497	1.359	0.501	0.463	1.161	0.224
10461	0.450	1.076	0.492	0.462	1.133	0.501	0.471	1.211	0.501
10462	0.468	1.194	0.508	0.465	1.152	0.501	0.497	1.380	0.262
10463	0.142	-0.895	0.512	0.193	-0.600	0.478	0.363	0.520	0.508
10464	0.187	-0.608	0.513	0.342	0.361	0.273	0.297	0.099	0.185
10465	0.224	-0.373	0.501	0.310	0.153	0.273	0.145	-0.878	0.224
10466	0.224	-0.373	0.142	0.398	0.718	0.512	0.142	-0.895	0.450
10467	0.097	-1.181	0.512	0.307	0.135	0.512	0.413	0.841	0.296
10468	0.503	1.413	0.492	0.477	1.227	0.512	0.411	0.824	0.508
10469	0.466	1.178	0.508	0.520	1.509	0.193	0.487	1.312	0.224
10470	0.261	-0.137	0.328	0.152	-0.863	0.501	0.263	-0.120	0.501
10471	0.484	1.295	0.508	0.310	0.153	0.512	0.489	1.329	0.467
10472	0.463	1.161	0.224	0.468	1.170	0.444	0.489	1.329	0.492
10473	0.487	1.312	0.492	0.497	1.359	0.508	0.479	1.262	0.481
10474	0.142	-0.895	0.501	0.196	-0.581	0.149	0.355	0.470	0.481
10475	0.505	1.430	0.481	0.497	1.359	0.235	0.405	0.790	0.492
10476	0.505	1.430	0.481	0.497	1.359	0.508	0.426	0.925	0.224
10477	0.453	1.093	0.492	0.497	1.359	0.501	0.508	1.447	0.431
10478	0.413	0.841	0.262	0.477	1.227	0.501	0.497	1.380	0.492
10479	0.097	-1.181	0.492	0.278	-0.054	0.273	0.329	0.301	0.385
10480	0.142	-0.895	0.224	0.272	-0.091	0.341	0.326	0.285	0.385
10481	0.479	1.262	0.224	0.512	1.453	0.309	0.489	1.329	0.050
10482	0.511	1.464	0.224	0.509	1.434	0.478	0.479	1.262	0.050
10483	0.500	1.397	0.224	0.509	1.434	0.508	0.479	1.262	0.450
10484	0.358	0.487	0.262	0.193	-0.600	0.273	0.100	-1.164	0.450
10485	0.466	1.178	0.481	0.497	1.359	0.501	0.489	1.329	0.508
10486	0.489	1.329	0.481	0.509	1.434	0.508	0.513	1.481	0.508
10487	0.513	1.481	0.385	0.442	1.001	0.491	0.479	1.262	0.467
10488	0.508	1.447	0.296	0.503	1.396	0.371	0.495	1.363	0.450

10489	0.297	0.099	0.467	0.149	−0.882	0.273	0.184	−0.625	0.185
10490	0.263	−0.120	0.296	0.234	−0.336	0.501	0.184	−0.625	0.450
10491	0.505	1.430	0.481	0.512	1.453	0.501	0.495	1.363	0.224
10492	0.505	1.430	0.481	0.497	1.359	0.309	0.513	1.481	0.481
10493	0.508	1.447	0.501	0.503	1.396	0.422	0.495	1.363	0.501
10494	0.261	−0.137	0.501	0.272	−0.091	0.371	0.100	−1.164	0.467
10495	0.187	−0.608	0.501	0.345	0.379	0.501	0.358	0.487	0.508
10496	0.187	−0.608	0.185	0.313	0.172	0.422	0.384	0.655	0.467
10497	0.505	1.430	0.358	0.477	1.227	0.501	0.432	0.958	0.512
10498	0.508	1.447	0.185	0.509	1.434	0.149	0.476	1.245	0.262
10499	0.142	−0.895	0.185	0.272	−0.091	0.149	0.142	−0.895	0.508
10500	0.505	1.430	0.501	0.500	1.378	0.193	0.500	1.397	0.328
10501	0.489	1.329	0.508	0.506	1.415	0.235	0.476	1.245	0.450
10502	0.466	1.178	0.224	0.494	1.340	0.398	0.489	1.329	0.097
10503	0.489	1.329	0.450	0.371	0.549	0.273	0.505	1.430	0.481
10504	0.184	−0.625	0.296	0.272	−0.091	0.491	0.226	−0.356	0.262
10505	0.479	1.262	0.481	0.497	1.359	0.341	0.505	1.430	0.513
10506	0.468	1.194	0.224	0.506	1.415	0.341	0.495	1.363	0.492
10507	0.184	−0.625	0.224	0.310	0.153	0.149	0.184	−0.625	0.512
10508	0.487	1.312	0.262	0.421	0.869	0.371	0.466	1.178	0.450
10509	0.445	1.043	0.262	0.371	0.549	0.508	0.505	1.430	0.501
10510	0.497	1.380	0.097	0.503	1.396	0.371	0.503	1.413	0.185
10511	0.513	1.481	0.492	0.424	0.888	0.235	0.429	0.942	0.501
10512	0.511	1.464	0.385	0.503	1.396	0.398	0.453	1.093	0.050
10513	0.261	−0.137	0.467	0.149	−0.882	0.341	0.261	−0.137	0.050
10514	0.261	−0.137	0.481	0.149	−0.882	0.341	0.261	−0.137	0.050
10515	0.184	−0.625	0.296	0.193	−0.600	0.341	0.226	−0.356	0.050
10516	0.184	−0.625	0.097	0.237	−0.317	0.398	0.142	−0.895	0.142
10517	0.097	−1.181	0.358	0.401	0.737	0.501	0.297	0.099	0.385
10518	0.332	0.318	0.501	0.272	−0.091	0.512	0.332	0.318	0.385
10519	0.505	1.430	0.467	0.497	1.359	0.371	0.479	1.262	0.142
10520	0.097	−1.181	0.512	0.345	0.379	0.235	0.184	−0.625	0.185
10521	0.482	1.279	0.508	0.342	0.361	0.491	0.508	1.447	0.481
10522	0.187	−0.608	0.508	0.152	−0.863	0.462	0.408	0.807	0.185
10523	0.050	−1.485	0.508	0.371	0.549	0.273	0.187	−0.608	0.501
10524	0.497	1.380	0.050	0.518	1.491	0.193	0.471	1.211	0.481
10525	0.184	−0.625	0.512	0.371	0.549	0.371	0.000	?	0.467
10526	0.332	0.318	0.481	0.234	−0.336	0.512	0.145	−0.878	0.467
10527	0.297	0.099	0.481	0.398	0.718	0.444	0.097	−1.181	0.508
10528	0.500	1.397	0.501	0.342	0.361	0.514	0.505	1.430	0.097
10529	0.226	−0.356	0.501	0.339	0.342	0.512	0.050	−1.485	0.262
10530	0.000	?	0.185	0.342	0.361	0.193	0.224	−0.373	0.262
10531	0.358	0.487	0.185	0.398	0.718	0.193	0.097	−1.181	0.409
10532	0.508	1.447	0.185	0.503	1.396	0.193	0.466	1.178	0.481
10533	0.500	1.397	0.450	0.518	1.491	0.235	0.482	1.279	0.481
10534	0.050	−1.485	0.501	0.374	0.568	0.273	0.184	−0.625	0.224
10535	0.097	−1.181	0.296	0.237	−0.317	0.235	0.224	−0.373	0.409
10536	0.468	1.194	0.185	0.485	1.283	0.462	0.361	0.504	0.262
10537	0.492	1.346	0.512	0.462	1.133	0.501	0.500	1.397	0.501
10538	0.142	−0.895	0.142	0.275	−0.073	0.273	0.142	−0.895	0.097
10539	0.145	−0.878	0.185	0.196	−0.581	0.491	0.358	0.487	0.185
10540	0.184	−0.625	0.512	0.374	0.568	0.149	0.226	−0.356	0.262
10541	0.511	1.464	0.450	0.512	1.453	0.508	0.476	1.245	0.431
10542	0.487	1.312	0.262	0.444	1.020	0.273	0.495	1.363	0.262
10543	0.476	1.245	0.358	0.512	1.453	0.149	0.508	1.447	0.492
10544	0.476	1.245	0.185	0.509	1.434	0.501	0.516	1.498	0.262
10545	0.097	−1.181	0.508	0.193	−0.600	0.235	0.263	−0.120	0.501
10546	0.224	−0.373	0.492	0.196	−0.581	0.422	0.142	−0.895	0.508
10547	0.263	−0.120	0.501	0.193	−0.600	0.309	0.145	−0.878	0.358
10548	0.263	−0.120	0.409	0.234	−0.336	0.512	0.295	0.082	0.262
10549	0.187	−0.608	0.508	0.278	−0.054	0.501	0.261	−0.137	0.296

10550	0.187	-0.608	0.142	0.237	-0.317	0.508	0.326	0.285	0.431
10551	0.505	1.430	0.501	0.459	1.114	0.508	0.429	0.942	0.450
10552	0.508	1.447	0.450	0.506	1.415	0.512	0.471	1.211	0.492
10553	0.184	-0.625	0.467	0.272	-0.091	0.501	0.224	-0.373	0.431
10554	0.492	1.346	0.481	0.488	1.302	0.235	0.497	1.380	0.481
10555	0.261	-0.137	0.481	0.152	-0.863	0.491	0.261	-0.137	0.409
10556	0.503	1.413	0.501	0.506	1.415	0.422	0.503	1.413	0.224
10557	0.361	0.504	0.501	0.275	-0.073	0.273	0.184	-0.625	0.512
10558	0.224	-0.373	0.512	0.149	-0.882	0.273	0.224	-0.373	0.492
10559	0.482	1.279	0.513	0.500	1.378	0.273	0.447	1.060	0.467
10560	0.261	-0.137	0.501	0.237	-0.317	0.273	0.297	0.099	0.501
10561	0.445	1.043	0.501	0.421	0.869	0.512	0.513	1.481	0.501
10562	0.184	-0.625	0.508	0.313	0.172	0.512	0.226	-0.356	0.358
10563	0.484	1.295	0.501	0.512	1.453	0.514	0.503	1.413	0.142
10564	0.471	1.211	0.508	0.503	1.396	0.514	0.463	1.161	0.185
10565	0.492	1.346	0.512	0.512	1.453	0.501	0.489	1.329	0.492
10566	0.455	1.110	0.409	0.509	1.434	0.501	0.476	1.245	0.512
10567	0.482	1.279	0.508	0.512	1.453	0.422	0.484	1.295	0.262
10568	0.511	1.464	0.185	0.497	1.359	0.491	0.489	1.329	0.296
10569	0.097	-1.181	0.512	0.234	-0.336	0.149	0.224	-0.373	0.467
10570	0.405	0.790	0.501	0.497	1.359	0.508	0.432	0.958	0.097
10571	0.387	0.672	0.508	0.427	0.907	0.501	0.495	1.363	0.097
10572	0.266	-0.103	0.501	0.275	-0.073	0.512	0.382	0.638	0.431
10573	0.266	-0.103	0.501	0.275	-0.073	0.512	0.382	0.638	0.467
10574	0.224	-0.373	0.385	0.272	-0.091	0.508	0.050	-1.485	0.142
10575	0.263	-0.120	0.501	0.272	-0.091	0.478	0.050	-1.485	0.409
10576	0.508	1.447	0.224	0.512	1.453	0.309	0.445	1.043	0.050
10577	0.508	1.447	0.224	0.512	1.453	0.501	0.445	1.043	0.450
10578	0.492	1.346	0.142	0.512	1.453	0.512	0.503	1.413	0.481
10579	0.492	1.346	0.501	0.512	1.453	0.478	0.503	1.413	0.385
10580	0.455	1.110	0.513	0.494	1.340	0.235	0.466	1.178	0.467
10581	0.455	1.110	0.512	0.494	1.340	0.491	0.447	1.060	0.409
10582	0.187	-0.608	0.262	0.421	0.869	0.193	0.187	-0.608	0.328
10583	0.513	1.481	0.262	0.485	1.283	0.491	0.450	1.076	0.513
10584	0.184	-0.625	0.262	0.149	-0.882	0.462	0.224	-0.373	0.450
10585	0.479	1.262	0.501	0.509	1.434	0.512	0.476	1.245	0.492
10586	0.492	1.346	0.501	0.500	1.378	0.478	0.497	1.380	0.358
10587	0.505	1.430	0.296	0.512	1.453	0.501	0.466	1.178	0.409
10588	0.511	1.464	0.467	0.512	1.453	0.235	0.511	1.464	0.512
10589	0.503	1.413	0.262	0.509	1.434	0.514	0.466	1.178	0.512
10590	0.500	1.397	0.431	0.477	1.227	0.309	0.508	1.447	0.508
10591	0.142	-0.895	0.296	0.313	0.172	0.273	0.263	-0.120	0.501
10592	0.505	1.430	0.467	0.503	1.396	0.273	0.505	1.430	0.512
10593	0.489	1.329	0.512	0.506	1.415	0.273	0.329	0.301	0.512
10594	0.503	1.413	0.512	0.480	1.246	0.149	0.445	1.043	0.513
10595	0.329	0.301	0.513	0.237	-0.317	0.512	0.097	-1.181	0.513
10596	0.505	1.430	0.501	0.491	1.321	0.512	0.474	1.228	0.467
10597	0.224	-0.373	0.512	0.193	-0.600	0.149	0.261	-0.137	0.467
10598	0.492	1.346	0.512	0.491	1.321	0.273	0.511	1.464	0.409
10599	0.495	1.363	0.467	0.465	1.152	0.478	0.489	1.329	0.512
10600	0.479	1.262	0.097	0.512	1.453	0.273	0.518	1.514	0.328
10601	0.484	1.295	0.431	0.477	1.227	0.462	0.445	1.043	0.431
10602	0.492	1.346	0.501	0.497	1.359	0.478	0.497	1.380	0.467
10603	0.263	-0.120	0.501	0.234	-0.336	0.422	0.187	-0.608	0.431
10604	0.489	1.329	0.512	0.512	1.453	0.422	0.497	1.380	0.385
10605	0.226	-0.356	0.492	0.310	0.153	0.462	0.050	-1.485	0.385
10606	0.226	-0.356	0.385	0.275	-0.073	0.273	0.050	-1.485	0.513
10607	0.226	-0.356	0.050	0.275	-0.073	0.273	0.050	-1.485	0.508
10608	0.226	-0.356	0.501	0.275	-0.073	0.235	0.050	-1.485	0.296
10609	0.263	-0.120	0.492	0.149	-0.882	0.273	0.142	-0.895	0.296
10610	0.476	1.245	0.512	0.515	1.472	0.514	0.384	0.655	0.296

10611	0.476	1.245	0.467	0.515	1.472	0.514	0.384	0.655	0.508
10612	0.389	0.689	0.185	0.149	-0.882	0.149	0.142	-0.895	0.481
10613	0.297	0.099	0.224	0.275	-0.073	0.501	0.184	-0.625	0.450
10614	0.466	1.178	0.185	0.477	1.227	0.102	0.476	1.245	0.262
10615	0.297	0.099	0.224	0.275	-0.073	0.478	0.184	-0.625	0.185
10616	0.479	1.262	0.467	0.462	1.133	0.273	0.497	1.380	0.481
10617	0.479	1.262	0.513	0.482	1.265	0.193	0.482	1.279	0.501
10618	0.495	1.363	0.508	0.427	0.907	0.193	0.471	1.211	0.296
10619	0.495	1.363	0.450	0.427	0.907	0.273	0.471	1.211	0.467
10620	0.492	1.346	0.185	0.465	1.152	0.341	0.513	1.481	0.262
10621	0.184	-0.625	0.508	0.275	-0.073	0.508	0.097	-1.181	0.492
10622	0.358	0.487	0.492	0.278	-0.054	0.398	0.261	-0.137	0.296
10623	0.184	-0.625	0.358	0.237	-0.317	0.491	0.261	-0.137	0.501
10624	0.184	-0.625	0.050	0.272	-0.091	0.514	0.411	0.824	0.513
10625	0.500	1.397	0.481	0.509	1.434	0.273	0.476	1.245	0.513
10626	0.508	1.447	0.097	0.509	1.434	0.273	0.476	1.245	0.501
10627	0.224	-0.373	0.097	0.149	-0.882	0.273	0.224	-0.373	0.492
10628	0.450	1.076	0.224	0.503	1.396	0.444	0.411	0.824	0.450
10629	0.300	0.116	0.185	0.102	-1.184	0.371	0.261	-0.137	0.501
10630	0.479	1.262	0.508	0.477	1.227	0.501	0.495	1.363	0.467
10631	0.224	-0.373	0.467	0.272	-0.091	0.508	0.097	-1.181	0.358
10632	0.224	-0.373	0.508	0.193	-0.600	0.512	0.184	-0.625	0.501
10633	0.261	-0.137	0.492	0.193	-0.600	0.508	0.261	-0.137	0.501
10634	0.263	-0.120	0.467	0.275	-0.073	0.398	0.432	0.958	0.492
10635	0.097	-1.181	0.296	0.339	0.342	0.462	0.263	-0.120	0.492
10636	0.489	1.329	0.224	0.509	1.434	0.508	0.489	1.329	0.501
10637	0.384	0.655	0.467	0.401	0.737	0.501	0.261	-0.137	0.508
10638	0.463	1.161	0.508	0.488	1.302	0.512	0.497	1.380	0.142
10639	0.479	1.262	0.296	0.509	1.434	0.512	0.508	1.447	0.481
10640	0.297	0.099	0.501	0.275	-0.073	0.309	0.358	0.487	0.508
10641	0.097	-1.181	0.328	0.272	-0.091	0.309	0.263	-0.120	0.431
10642	0.358	0.487	0.224	0.272	-0.091	0.193	0.297	0.099	0.513
10643	0.505	1.430	0.296	0.444	1.020	0.478	0.429	0.942	0.431
10644	0.468	1.194	0.296	0.374	0.568	0.371	0.453	1.093	0.185
10645	0.511	1.464	0.224	0.500	1.378	0.273	0.492	1.346	0.296
10646	0.508	1.447	0.492	0.506	1.415	0.273	0.429	0.942	0.296
10647	0.508	1.447	0.328	0.512	1.453	0.398	0.482	1.279	0.492
10648	0.505	1.430	0.224	0.509	1.434	0.309	0.408	0.807	0.385
10649	0.050	-1.485	0.296	0.401	0.737	0.309	0.224	-0.373	0.508
10650	0.505	1.430	0.296	0.462	1.133	0.508	0.516	1.498	0.481
10651	0.479	1.262	0.224	0.509	1.434	0.491	0.495	1.363	0.296
10652	0.479	1.262	0.481	0.503	1.396	0.193	0.466	1.178	0.431
10653	0.500	1.397	0.492	0.512	1.453	0.309	0.503	1.413	0.431
10654	0.500	1.397	0.142	0.512	1.453	0.341	0.503	1.413	0.501
10655	0.184	-0.625	0.142	0.313	0.172	0.514	0.358	0.487	0.224
10656	0.184	-0.625	0.467	0.310	0.153	0.478	0.142	-0.895	0.508
10657	0.184	-0.625	0.467	0.196	-0.581	0.422	0.184	-0.625	0.185
10658	0.453	1.093	0.450	0.480	1.246	0.462	0.489	1.329	0.296
10659	0.503	1.413	0.224	0.371	0.549	0.508	0.508	1.447	0.185
10660	0.297	0.099	0.224	0.275	-0.073	0.235	0.263	-0.120	0.262
10661	0.187	-0.608	0.501	0.272	-0.091	0.512	0.297	0.099	0.050
10662	0.511	1.464	0.328	0.398	0.718	0.422	0.466	1.178	0.481
10663	0.142	-0.895	0.328	0.307	0.135	0.462	0.097	-1.181	0.512
10664	0.187	-0.608	0.409	0.307	0.135	0.512	0.097	-1.181	0.492
10665	0.513	1.481	0.097	0.509	1.434	0.512	0.426	0.925	0.512
10666	0.450	1.076	0.097	0.494	1.340	0.501	0.466	1.178	0.481
10667	0.261	-0.137	0.328	0.196	-0.581	0.501	0.142	-0.895	0.185
10668	0.358	0.487	0.492	0.313	0.172	0.514	0.413	0.841	0.262
10669	0.184	-0.625	0.224	0.345	0.379	0.478	0.050	-1.485	0.450
10670	0.503	1.413	0.296	0.512	1.453	0.508	0.450	1.076	0.467
10671	0.489	1.329	0.296	0.477	1.227	0.508	0.476	1.245	0.050

10672	0.500	1.397	0.296	0.421	0.869	0.491	0.384	0.655	0.481
10673	0.408	0.807	0.262	0.456	1.095	0.508	0.466	1.178	0.262
10674	0.508	1.447	0.492	0.503	1.396	0.512	0.411	0.824	0.097
10675	0.145	-0.878	0.492	0.234	-0.336	0.491	0.329	0.301	0.142
10676	0.497	1.380	0.513	0.509	1.434	0.501	0.511	1.464	0.185
10677	0.447	1.060	0.513	0.424	0.888	0.444	0.445	1.043	0.142
10678	0.466	1.178	0.492	0.465	1.152	0.193	0.492	1.346	0.467
10679	0.476	1.245	0.513	0.518	1.491	0.514	0.358	0.487	0.097
10680	0.476	1.245	0.385	0.518	1.491	0.512	0.411	0.824	0.224
10681	0.500	1.397	0.512	0.503	1.396	0.514	0.513	1.481	0.097
10682	0.500	1.397	0.512	0.503	1.396	0.514	0.513	1.481	0.097
10683	0.511	1.464	0.224	0.518	1.491	0.512	0.511	1.464	0.328
10684	0.508	1.447	0.262	0.482	1.265	0.514	0.492	1.346	0.467
10685	0.497	1.380	0.492	0.512	1.453	0.512	0.503	1.413	0.501
10686	0.497	1.380	0.185	0.512	1.453	0.371	0.503	1.413	0.224
10687	0.503	1.413	0.262	0.494	1.340	0.341	0.513	1.481	0.224
10688	0.497	1.380	0.328	0.506	1.415	0.193	0.511	1.464	0.409
10689	0.503	1.413	0.508	0.512	1.453	0.512	0.468	1.194	0.450
10690	0.508	1.447	0.431	0.491	1.321	0.491	0.468	1.194	0.450
10691	0.408	0.807	0.492	0.509	1.434	0.508	0.411	0.824	0.296
10692	0.508	1.447	0.467	0.442	1.001	0.309	0.513	1.481	0.328
10693	0.184	-0.625	0.512	0.193	-0.600	0.341	0.334	0.335	0.508
10694	0.500	1.397	0.512	0.518	1.491	0.273	0.432	0.958	0.097
10695	0.495	1.363	0.050	0.515	1.472	0.462	0.461	1.144	0.097
10696	0.500	1.397	0.262	0.512	1.453	0.478	0.434	0.975	0.492
10697	0.497	1.380	0.492	0.512	1.453	0.149	0.387	0.672	0.097
10698	0.497	1.380	0.142	0.512	1.453	0.444	0.387	0.672	0.224
10699	0.382	0.638	0.508	0.512	1.453	0.102	0.508	1.447	0.185
10700	0.500	1.397	0.481	0.509	1.434	0.508	0.503	1.413	0.492
10701	0.226	-0.356	0.262	0.377	0.587	0.102	0.295	0.082	0.224
10702	0.226	-0.356	0.262	0.345	0.379	0.491	0.295	0.082	0.142
10703	0.145	-0.878	0.296	0.193	-0.600	0.462	0.295	0.082	0.296
10704	0.492	1.346	0.328	0.515	1.472	0.462	0.505	1.430	0.296
10705	0.511	1.464	0.185	0.491	1.321	0.508	0.479	1.262	0.224
10706	0.511	1.464	0.185	0.509	1.434	0.508	0.450	1.076	0.467
10707	0.261	-0.137	0.142	0.307	0.135	0.501	0.000	?	0.450
10708	0.261	-0.137	0.097	0.345	0.379	0.478	0.263	-0.120	0.481
10709	0.261	-0.137	0.481	0.272	-0.091	0.512	0.184	-0.625	0.481
10710	0.495	1.363	0.467	0.465	1.152	0.193	0.479	1.262	0.467
10711	0.500	1.397	0.262	0.477	1.227	0.501	0.500	1.397	0.481
10712	0.295	0.082	0.328	0.149	-0.882	0.514	0.295	0.082	0.142
10713	0.466	1.178	0.358	0.442	1.001	0.514	0.463	1.161	0.481
10714	0.261	-0.137	0.431	0.102	-1.184	0.508	0.261	-0.137	0.262
10715	0.434	0.975	0.385	0.503	1.396	0.444	0.484	1.295	0.450
10716	0.297	0.099	0.224	0.102	-1.184	0.478	0.300	0.116	0.262
10717	0.463	1.161	0.512	0.488	1.302	0.341	0.497	1.380	0.185
10718	0.505	1.430	0.512	0.468	1.170	0.512	0.508	1.447	0.481
10719	0.505	1.430	0.262	0.468	1.170	0.444	0.508	1.447	0.224
10720	0.503	1.413	0.512	0.512	1.453	0.501	0.503	1.413	0.296
10721	0.497	1.380	0.508	0.515	1.472	0.444	0.492	1.346	0.262
10722	0.511	1.464	0.467	0.497	1.359	0.273	0.450	1.076	0.450
10723	0.516	1.498	0.467	0.477	1.227	0.149	0.497	1.380	0.501
10724	0.468	1.194	0.185	0.515	1.472	0.341	0.466	1.178	0.501
10725	0.097	-1.181	0.492	0.193	-0.600	0.053	0.358	0.487	0.409
10726	0.429	0.942	0.185	0.500	1.378	0.501	0.495	1.363	0.513
10727	0.495	1.363	0.097	0.512	1.453	0.491	0.500	1.397	0.492
10728	0.495	1.363	0.501	0.512	1.453	0.501	0.492	1.346	0.142
10729	0.508	1.447	0.508	0.506	1.415	0.501	0.489	1.329	0.050
10730	0.495	1.363	0.508	0.447	1.039	0.341	0.500	1.397	0.142
10731	0.382	0.638	0.481	0.477	1.227	0.508	0.505	1.430	0.431
10732	0.050	-1.485	0.512	0.339	0.342	0.508	0.142	-0.895	0.224

10733	0.497	1.380	0.512	0.512	1.453	0.444	0.482	1.279	0.481
10734	0.487	1.312	0.512	0.444	1.020	0.273	0.505	1.430	0.481
10735	0.508	1.447	0.492	0.491	1.321	0.512	0.434	0.975	0.050
10736	0.463	1.161	0.508	0.442	1.001	0.273	0.508	1.447	0.050
10737	0.184	-0.625	0.262	0.272	-0.091	0.235	0.432	0.958	0.185
10738	0.224	-0.373	0.501	0.152	-0.863	0.398	0.184	-0.625	0.224
10739	0.187	-0.608	0.450	0.342	0.361	0.193	0.295	0.082	0.385
10740	0.226	-0.356	0.450	0.053	-1.504	0.341	0.295	0.082	0.385
10741	0.463	1.161	0.185	0.500	1.378	0.501	0.489	1.329	0.185
10742	0.511	1.464	0.513	0.497	1.359	0.478	0.384	0.655	0.450
10743	0.505	1.430	0.185	0.503	1.396	0.444	0.503	1.413	0.508
10744	0.453	1.093	0.185	0.503	1.396	0.371	0.482	1.279	0.481
10745	0.187	-0.608	0.296	0.339	0.342	0.508	0.300	0.116	0.328
10746	0.508	1.447	0.262	0.509	1.434	0.273	0.429	0.942	0.185
10747	0.492	1.346	0.296	0.509	1.434	0.193	0.429	0.942	0.185
10748	0.363	0.520	0.501	0.447	1.039	0.478	0.495	1.363	0.501
10749	0.050	-1.485	0.467	0.275	-0.073	0.491	0.226	-0.356	0.513
10750	0.479	1.262	0.501	0.515	1.472	0.273	0.508	1.447	0.097
10751	0.100	-1.164	0.262	0.275	-0.073	0.478	0.184	-0.625	0.409
10752	0.000	?	0.450	0.234	-0.336	0.193	0.295	0.082	0.450
10753	0.100	-1.164	0.481	0.401	0.737	0.235	0.184	-0.625	0.224
10754	0.226	-0.356	0.481	0.193	-0.600	0.149	0.263	-0.120	0.450
10755	0.184	-0.625	0.467	0.345	0.379	0.193	0.050	-1.485	0.492
10756	0.508	1.447	0.358	0.500	1.378	0.193	0.482	1.279	0.409
10757	0.466	1.178	0.450	0.477	1.227	0.508	0.508	1.447	0.385
10758	0.511	1.464	0.467	0.442	1.001	0.235	0.492	1.346	0.224
10759	0.487	1.312	0.185	0.371	0.549	0.102	0.513	1.481	0.492
10760	0.471	1.211	0.262	0.503	1.396	0.309	0.476	1.245	0.050
10761	0.295	0.082	0.467	0.278	-0.054	0.309	0.184	-0.625	0.224
10762	0.226	-0.356	0.512	0.196	-0.581	0.235	0.261	-0.137	0.481
10763	0.468	1.194	0.512	0.477	1.227	0.422	0.447	1.060	0.431
10764	0.511	1.464	0.481	0.494	1.340	0.444	0.468	1.194	0.450
10765	0.297	0.099	0.262	0.272	-0.091	0.309	0.050	-1.485	0.513
10766	0.503	1.413	0.142	0.474	1.208	0.309	0.474	1.228	0.467
10767	0.326	0.285	0.492	0.193	-0.600	0.478	0.263	-0.120	0.328
10768	0.224	-0.373	0.262	0.234	-0.336	0.422	0.097	-1.181	0.328
10769	0.295	0.082	0.262	0.149	-0.882	0.491	0.142	-0.895	0.508
10770	0.300	0.116	0.481	0.193	-0.600	0.193	0.184	-0.625	0.492
10771	0.226	-0.356	0.512	0.193	-0.600	0.193	0.142	-0.895	0.142
10772	0.489	1.329	0.481	0.506	1.415	0.462	0.461	1.144	0.501
10773	0.332	0.318	0.185	0.234	-0.336	0.193	0.097	-1.181	0.512
10774	0.229	-0.339	0.508	0.102	-1.184	0.193	0.224	-0.373	0.508
10775	0.297	0.099	0.142	0.307	0.135	0.491	0.097	-1.181	0.296
10776	0.297	0.099	0.142	0.307	0.135	0.371	0.097	-1.181	0.512
10777	0.224	-0.373	0.185	0.234	-0.336	0.235	0.326	0.285	0.142
10778	0.479	1.262	0.508	0.427	0.907	0.235	0.466	1.178	0.097
10779	0.489	1.329	0.501	0.444	1.020	0.478	0.503	1.413	0.097
10780	0.145	-0.878	0.501	0.307	0.135	0.273	0.224	-0.373	0.142
10781	0.145	-0.878	0.358	0.307	0.135	0.309	0.224	-0.373	0.481
10782	0.466	1.178	0.224	0.480	1.246	0.462	0.411	0.824	0.431
10783	0.466	1.178	0.262	0.421	0.869	0.462	0.453	1.093	0.508
10784	0.447	1.060	0.481	0.494	1.340	0.193	0.450	1.076	0.050
10785	0.224	-0.373	0.262	0.196	-0.581	0.444	0.295	0.082	0.492
10786	0.226	-0.356	0.492	0.193	-0.600	0.444	0.332	0.318	0.508
10787	0.505	1.430	0.512	0.462	1.133	0.444	0.500	1.397	0.508
10788	0.326	0.285	0.050	0.193	-0.600	0.444	0.097	-1.181	0.508
10789	0.326	0.285	0.431	0.193	-0.600	0.462	0.097	-1.181	0.142
10790	0.411	0.824	0.512	0.488	1.302	0.462	0.484	1.295	0.450
10791	0.097	-1.181	0.512	0.371	0.549	0.309	0.097	-1.181	0.508
10792	0.097	-1.181	0.224	0.234	-0.336	0.501	0.224	-0.373	0.262
10793	0.332	0.318	0.508	0.237	-0.317	0.341	0.184	-0.625	0.296

10794	0.489	1.329	0.467	0.480	1.246	0.478	0.489	1.329	0.492
10795	0.224	-0.373	0.513	0.272	-0.091	0.341	0.224	-0.373	0.431
10796	0.297	0.099	0.512	0.310	0.153	0.341	0.142	-0.895	0.409
10797	0.297	0.099	0.512	0.465	1.152	0.512	0.295	0.082	0.501
10798	0.297	0.099	0.450	0.465	1.152	0.193	0.295	0.082	0.097
10799	0.263	-0.120	0.512	0.193	-0.600	0.235	0.224	-0.373	0.185
10800	0.489	1.329	0.492	0.444	1.020	0.309	0.468	1.194	0.492
10801	0.489	1.329	0.185	0.444	1.020	0.478	0.453	1.093	0.097
10802	0.511	1.464	0.142	0.444	1.020	0.444	0.479	1.262	0.097
10803	0.511	1.464	0.492	0.444	1.020	0.462	0.479	1.262	0.492
10804	0.492	1.346	0.467	0.465	1.152	0.462	0.471	1.211	0.450
10805	0.513	1.481	0.467	0.465	1.152	0.508	0.479	1.262	0.450
10806	0.384	0.655	0.492	0.310	0.153	0.462	0.142	-0.895	0.296
10807	0.500	1.397	0.450	0.500	1.378	0.341	0.489	1.329	0.512
10808	0.000	?	0.262	0.342	0.361	0.444	0.261	-0.137	0.185
10809	0.500	1.397	0.467	0.477	1.227	0.309	0.458	1.127	0.262
10810	0.224	-0.373	0.492	0.342	0.361	0.478	0.261	-0.137	0.296
10811	0.261	-0.137	0.296	0.342	0.361	0.273	0.184	-0.625	0.512
10812	0.492	1.346	0.501	0.512	1.453	0.501	0.479	1.262	0.481
10813	0.184	-0.625	0.501	0.193	-0.600	0.508	0.226	-0.356	0.481
10814	0.261	-0.137	0.358	0.234	-0.336	0.273	0.295	0.082	0.142
10815	0.332	0.318	0.501	0.310	0.153	0.273	0.261	-0.137	0.492
10816	0.513	1.481	0.224	0.477	1.227	0.193	0.445	1.043	0.328
10817	0.434	0.975	0.501	0.447	1.039	0.149	0.500	1.397	0.481
10818	0.497	1.380	0.097	0.465	1.152	0.444	0.500	1.397	0.409
10819	0.468	1.194	0.385	0.465	1.152	0.444	0.408	0.807	0.501
10820	0.513	1.481	0.185	0.512	1.453	0.273	0.516	1.498	0.481
10821	0.508	1.447	0.328	0.462	1.133	0.341	0.489	1.329	0.358
10822	0.050	-1.485	0.328	0.339	0.342	0.478	0.142	-0.895	0.508
10823	0.000	?	0.328	0.444	1.020	0.491	0.050	-1.485	0.431
10824	0.261	-0.137	0.512	0.310	0.153	0.149	0.142	-0.895	0.450
10825	0.489	1.329	0.508	0.477	1.227	0.193	0.429	0.942	0.185
10826	0.142	-0.895	0.224	0.275	-0.073	0.235	0.224	-0.373	0.431
10827	0.503	1.413	0.492	0.506	1.415	0.462	0.474	1.228	0.501
10828	0.484	1.295	0.262	0.506	1.415	0.444	0.474	1.228	0.450
10829	0.261	-0.137	0.512	0.272	-0.091	0.235	0.050	-1.485	0.409
10830	0.261	-0.137	0.224	0.272	-0.091	0.508	0.050	-1.485	0.431
10831	0.295	0.082	0.501	0.193	-0.600	0.371	0.187	-0.608	0.501
10832	0.326	0.285	0.185	0.149	-0.882	0.193	0.226	-0.356	0.508
10833	0.184	-0.625	0.385	0.447	1.039	0.444	0.384	0.655	0.492
10834	0.184	-0.625	0.450	0.447	1.039	0.501	0.384	0.655	0.296
10835	0.142	-0.895	0.296	0.275	-0.073	0.512	0.184	-0.625	0.185
10836	0.097	-1.181	0.492	0.345	0.379	0.512	0.450	1.076	0.508
10837	0.479	1.262	0.492	0.471	1.189	0.235	0.508	1.447	0.501
10838	0.463	1.161	0.224	0.494	1.340	0.462	0.482	1.279	0.501
10839	0.261	-0.137	0.185	0.149	-0.882	0.398	0.332	0.318	0.501
10840	0.329	0.301	0.501	0.193	-0.600	0.235	0.187	-0.608	0.512
10841	0.358	0.487	0.492	0.234	-0.336	0.491	0.184	-0.625	0.296
10842	0.429	0.942	0.501	0.462	1.133	0.512	0.503	1.413	0.492
10843	0.382	0.638	0.501	0.444	1.020	0.444	0.513	1.481	0.492
10844	0.226	-0.356	0.224	0.234	-0.336	0.444	0.097	-1.181	0.050
10845	0.500	1.397	0.185	0.515	1.472	0.422	0.405	0.790	0.481
10846	0.508	1.447	0.385	0.368	0.530	0.491	0.447	1.060	0.467
10847	0.263	-0.120	0.142	0.193	-0.600	0.491	0.226	-0.356	0.501
10848	0.508	1.447	0.185	0.442	1.001	0.478	0.447	1.060	0.431
10849	0.505	1.430	0.431	0.500	1.378	0.462	0.497	1.380	0.328
10850	0.466	1.178	0.450	0.518	1.491	0.193	0.411	0.824	0.512
10851	0.466	1.178	0.224	0.515	1.472	0.501	0.384	0.655	0.409
10852	0.187	-0.608	0.512	0.234	-0.336	0.501	0.224	-0.373	0.142
10853	0.492	1.346	0.262	0.462	1.133	0.501	0.484	1.295	0.224
10854	0.184	-0.625	0.296	0.398	0.718	0.309	0.050	-1.485	0.050

10855	0.097	-1.181	0.508	0.234	-0.336	0.491	0.224	-0.373	0.050
10856	0.500	1.397	0.508	0.494	1.340	0.309	0.479	1.262	0.050
10857	0.497	1.380	0.050	0.512	1.453	0.341	0.432	0.958	0.467
10858	0.505	1.430	0.097	0.442	1.001	0.235	0.447	1.060	0.508
10859	0.482	1.279	0.262	0.439	0.982	0.149	0.508	1.447	0.224
10860	0.500	1.397	0.508	0.424	0.888	0.273	0.468	1.194	0.513
10861	0.503	1.413	0.224	0.491	1.321	0.512	0.326	0.285	0.185
10862	0.503	1.413	0.512	0.491	1.321	0.508	0.326	0.285	0.328
10863	0.497	1.380	0.097	0.477	1.227	0.444	0.497	1.380	0.262
10864	0.513	1.481	0.467	0.462	1.133	0.235	0.484	1.295	0.409
10865	0.261	-0.137	0.185	0.196	-0.581	0.422	0.142	-0.895	0.224
10866	0.500	1.397	0.512	0.500	1.378	0.508	0.497	1.380	0.262
10867	0.447	1.060	0.501	0.500	1.378	0.508	0.505	1.430	0.467
10868	0.447	1.060	0.450	0.500	1.378	0.462	0.503	1.413	0.097
10869	0.184	-0.625	0.224	0.313	0.172	0.235	0.297	0.099	0.501
10870	0.508	1.447	0.467	0.494	1.340	0.508	0.511	1.464	0.501
10871	0.184	-0.625	0.224	0.313	0.172	0.462	0.142	-0.895	0.097
10872	0.184	-0.625	0.224	0.345	0.379	0.102	0.097	-1.181	0.142
10873	0.297	0.099	0.142	0.237	-0.317	0.193	0.097	-1.181	0.431
10874	0.263	-0.120	0.481	0.149	-0.882	0.478	0.145	-0.878	0.492
10875	0.297	0.099	0.358	0.275	-0.073	0.508	0.000	?	0.508
10876	0.503	1.413	0.492	0.512	1.453	0.491	0.476	1.245	0.512
10877	0.466	1.178	0.492	0.512	1.453	0.501	0.426	0.925	0.097
10878	0.492	1.346	0.508	0.447	1.039	0.341	0.508	1.447	0.431
10879	0.266	-0.103	0.431	0.234	-0.336	0.235	0.050	-1.485	0.467
10880	0.453	1.093	0.431	0.424	0.888	0.478	0.492	1.346	0.185
10881	0.479	1.262	0.142	0.509	1.434	0.341	0.503	1.413	0.262
10882	0.479	1.262	0.508	0.509	1.434	0.341	0.503	1.413	0.512
10883	0.466	1.178	0.501	0.468	1.170	0.512	0.503	1.413	0.513
10884	0.361	0.504	0.467	0.237	-0.317	0.512	0.142	-0.895	0.224
10885	0.447	1.060	0.262	0.509	1.434	0.512	0.450	1.076	0.501
10886	0.466	1.178	0.431	0.465	1.152	0.053	0.497	1.380	0.185
10887	0.184	-0.625	0.262	0.102	-1.184	0.501	0.261	-0.137	0.385
10888	0.263	-0.120	0.185	0.193	-0.600	0.235	0.297	0.099	0.467
10889	0.461	1.144	0.501	0.480	1.246	0.193	0.492	1.346	0.492
10890	0.503	1.413	0.142	0.509	1.434	0.235	0.432	0.958	0.262
10891	0.516	1.498	0.450	0.491	1.321	0.491	0.405	0.790	0.409
10892	0.476	1.245	0.328	0.497	1.359	0.398	0.500	1.397	0.142
10893	0.263	-0.120	0.512	0.342	0.361	0.398	0.097	-1.181	0.508
10894	0.142	-0.895	0.508	0.237	-0.317	0.102	0.184	-0.625	0.142
10895	0.484	1.295	0.224	0.477	1.227	0.193	0.492	1.346	0.450
10896	0.263	-0.120	0.185	0.345	0.379	0.235	0.097	-1.181	0.262
10897	0.263	-0.120	0.501	0.345	0.379	0.462	0.097	-1.181	0.501
10898	0.482	1.279	0.508	0.512	1.453	0.193	0.484	1.295	0.262
10899	0.513	1.481	0.481	0.515	1.472	0.422	0.450	1.076	0.501
10900	0.479	1.262	0.513	0.512	1.453	0.478	0.450	1.076	0.501
10901	0.187	-0.608	0.185	0.053	-1.504	0.309	0.295	0.082	0.450
10902	0.508	1.447	0.224	0.500	1.378	0.508	0.508	1.447	0.185
10903	0.142	-0.895	0.185	0.237	-0.317	0.478	0.184	-0.625	0.512
10904	0.142	-0.895	0.097	0.196	-0.581	0.478	0.263	-0.120	0.262
10905	0.187	-0.608	0.097	0.237	-0.317	0.149	0.297	0.099	0.262
10906	0.508	1.447	0.050	0.494	1.340	0.491	0.516	1.498	0.328
10907	0.505	1.430	0.328	0.401	0.737	0.491	0.476	1.245	0.492
10908	0.505	1.430	0.431	0.401	0.737	0.514	0.476	1.245	0.050
10909	0.358	0.487	0.224	0.102	-1.184	0.501	0.142	-0.895	0.450
10910	0.226	-0.356	0.262	0.193	-0.600	0.501	0.495	1.363	0.450
10911	0.263	-0.120	0.508	0.237	-0.317	0.501	0.329	0.301	0.450
10912	0.482	1.279	0.512	0.456	1.095	0.491	0.479	1.262	0.481
10913	0.266	-0.103	0.142	0.193	-0.600	0.508	0.411	0.824	0.492
10914	0.489	1.329	0.328	0.421	0.869	0.235	0.500	1.397	0.296
10915	0.511	1.464	0.142	0.477	1.227	0.273	0.476	1.245	0.481

10916	0.050	-1.485	0.481	0.310	0.153	0.501	0.361	0.504	0.501
10917	0.426	0.925	0.224	0.503	1.396	0.512	0.505	1.430	0.492
10918	0.511	1.464	0.262	0.474	1.208	0.501	0.426	0.925	0.385
10919	0.511	1.464	0.224	0.471	1.189	0.444	0.447	1.060	0.450
10920	0.226	-0.356	0.097	0.149	-0.882	0.501	0.184	-0.625	0.097
10921	0.503	1.413	0.431	0.491	1.321	0.341	0.426	0.925	0.296
10922	0.466	1.178	0.262	0.494	1.340	0.501	0.500	1.397	0.481
10923	0.508	1.447	0.508	0.515	1.472	0.491	0.447	1.060	0.328
10924	0.511	1.464	0.481	0.500	1.378	0.273	0.408	0.807	0.358
10925	0.513	1.481	0.492	0.503	1.396	0.462	0.434	0.975	0.185
10926	0.453	1.093	0.481	0.503	1.396	0.462	0.495	1.363	0.508
10927	0.508	1.447	0.262	0.488	1.302	0.478	0.513	1.481	0.481
10928	0.492	1.346	0.050	0.509	1.434	0.444	0.489	1.329	0.142
10929	0.184	-0.625	0.296	0.237	-0.317	0.149	0.297	0.099	0.050
10930	0.142	-0.895	0.467	0.275	-0.073	0.491	0.184	-0.625	0.492
10931	0.492	1.346	0.296	0.500	1.378	0.193	0.505	1.430	0.501
10932	0.461	1.144	0.097	0.509	1.434	0.273	0.503	1.413	0.513
10933	0.461	1.144	0.481	0.497	1.359	0.341	0.503	1.413	0.431
10934	0.487	1.312	0.142	0.444	1.020	0.273	0.500	1.397	0.224
10935	0.450	1.076	0.142	0.497	1.359	0.273	0.505	1.430	0.185
10936	0.263	-0.120	0.142	0.339	0.342	0.273	0.295	0.082	0.097
10937	0.463	1.161	0.185	0.497	1.359	0.512	0.489	1.329	0.262
10938	0.492	1.346	0.328	0.494	1.340	0.512	0.484	1.295	0.224
10939	0.295	0.082	0.501	0.272	-0.091	0.149	0.050	-1.485	0.262
10940	0.497	1.380	0.501	0.465	1.152	0.512	0.479	1.262	0.328
10941	0.497	1.380	0.492	0.465	1.152	0.341	0.466	1.178	0.142
10942	0.355	0.470	0.358	0.480	1.246	0.501	0.500	1.397	0.097
10943	0.497	1.380	0.492	0.447	1.039	0.235	0.429	0.942	0.481
10944	0.224	-0.373	0.508	0.149	-0.882	0.501	0.329	0.301	0.185
10945	0.492	1.346	0.431	0.491	1.321	0.309	0.511	1.464	0.262
10946	0.097	-1.181	0.431	0.193	-0.600	0.235	0.411	0.824	0.501
10947	0.382	0.638	0.142	0.278	-0.054	0.478	0.142	-0.895	0.492
10948	0.187	-0.608	0.501	0.339	0.342	0.193	0.226	-0.356	0.262
10949	0.326	0.285	0.513	0.278	-0.054	0.512	0.050	-1.485	0.050
10950	0.326	0.285	0.512	0.278	-0.054	0.512	0.050	-1.485	0.185
10951	0.326	0.285	0.296	0.278	-0.054	0.371	0.050	-1.485	0.492
10952	0.503	1.413	0.431	0.515	1.472	0.341	0.463	1.161	0.142
10953	0.500	1.397	0.508	0.515	1.472	0.512	0.505	1.430	0.142
10954	0.226	-0.356	0.142	0.149	-0.882	0.508	0.226	-0.356	0.296
10955	0.489	1.329	0.513	0.518	1.491	0.478	0.513	1.481	0.097
10956	0.263	-0.120	0.185	0.342	0.361	0.478	0.184	-0.625	0.501
10957	0.511	1.464	0.513	0.506	1.415	0.273	0.326	0.285	0.185
10958	0.224	-0.373	0.296	0.234	-0.336	0.149	0.261	-0.137	0.467
10959	0.500	1.397	0.467	0.494	1.340	0.512	0.408	0.807	0.467
10960	0.184	-0.625	0.050	0.307	0.135	0.309	0.224	-0.373	0.467
10961	0.389	0.689	0.262	0.234	-0.336	0.235	0.261	-0.137	0.508
10962	0.450	1.076	0.481	0.474	1.208	0.491	0.463	1.161	0.097
10963	0.295	0.082	0.512	0.193	-0.600	0.491	0.097	-1.181	0.385
10964	0.489	1.329	0.481	0.506	1.415	0.341	0.500	1.397	0.142
10965	0.489	1.329	0.481	0.506	1.415	0.478	0.500	1.397	0.512
10966	0.224	-0.373	0.512	0.374	0.568	0.149	0.097	-1.181	0.050
10967	0.187	-0.608	0.512	0.342	0.361	0.309	0.142	-0.895	0.142
10968	0.497	1.380	0.513	0.512	1.453	0.501	0.429	0.942	0.501
10969	0.487	1.312	0.358	0.506	1.415	0.491	0.492	1.346	0.185
10970	0.505	1.430	0.512	0.480	1.246	0.341	0.503	1.413	0.185
10971	0.508	1.447	0.262	0.480	1.246	0.398	0.508	1.447	0.185
10972	0.224	-0.373	0.262	0.275	-0.073	0.193	0.097	-1.181	0.296
10973	0.184	-0.625	0.501	0.149	-0.882	0.478	0.432	0.958	0.142
10974	0.384	0.655	0.097	0.515	1.472	0.193	0.463	1.161	0.512
10975	0.142	-0.895	0.097	0.310	0.153	0.512	0.187	-0.608	0.513
10976	0.184	-0.625	0.185	0.234	-0.336	0.273	0.263	-0.120	0.508

10977	0.429	0.942	0.450	0.494	1.340	0.462	0.513	1.481	0.097
10978	0.450	1.076	0.142	0.494	1.340	0.149	0.516	1.498	0.501
10979	0.226	-0.356	0.450	0.342	0.361	0.422	0.226	-0.356	0.450
10980	0.513	1.481	0.481	0.471	1.189	0.508	0.497	1.380	0.501
10981	0.263	-0.120	0.508	0.149	-0.882	0.508	0.184	-0.625	0.501
10982	0.295	0.082	0.501	0.310	0.153	0.193	0.384	0.655	0.224
10983	0.505	1.430	0.501	0.500	1.378	0.422	0.461	1.144	0.467
10984	0.503	1.413	0.185	0.491	1.321	0.149	0.489	1.329	0.492
10985	0.050	-1.485	0.501	0.345	0.379	0.149	0.261	-0.137	0.450
10986	0.097	-1.181	0.431	0.395	0.700	0.235	0.411	0.824	0.185
10987	0.261	-0.137	0.142	0.193	-0.600	0.491	0.142	-0.895	0.513
10988	0.505	1.430	0.185	0.482	1.265	0.149	0.511	1.464	0.501
10989	0.224	-0.373	0.142	0.193	-0.600	0.501	0.142	-0.895	0.097
10990	0.508	1.447	0.501	0.512	1.453	0.508	0.450	1.076	0.431
10991	0.097	-1.181	0.508	0.275	-0.073	0.444	0.263	-0.120	0.262
10992	0.466	1.178	0.224	0.465	1.152	0.491	0.495	1.363	0.431
10993	0.184	-0.625	0.224	0.149	-0.882	0.478	0.263	-0.120	0.185
10994	0.513	1.481	0.431	0.427	0.907	0.102	0.503	1.413	0.481
10995	0.505	1.430	0.450	0.509	1.434	0.444	0.505	1.430	0.097
10996	0.447	1.060	0.501	0.503	1.396	0.491	0.445	1.043	0.142
10997	0.229	-0.339	0.512	0.193	-0.600	0.491	0.184	-0.625	0.512
10998	0.466	1.178	0.050	0.421	0.869	0.371	0.505	1.430	0.467
10999	0.224	-0.373	0.262	0.152	-0.863	0.478	0.261	-0.137	0.481
11000	0.224	-0.373	0.142	0.152	-0.863	0.273	0.261	-0.137	0.512
11001	0.142	-0.895	0.224	0.234	-0.336	0.309	0.326	0.285	0.513
11002	0.484	1.295	0.262	0.491	1.321	0.514	0.492	1.346	0.450
11003	0.361	0.504	0.262	0.149	-0.882	0.273	0.050	-1.485	0.513
11004	0.489	1.329	0.512	0.506	1.415	0.512	0.447	1.060	0.262
11005	0.487	1.312	0.512	0.506	1.415	0.235	0.450	1.076	0.492
11006	0.505	1.430	0.492	0.447	1.039	0.501	0.453	1.093	0.409
11007	0.429	0.942	0.097	0.491	1.321	0.422	0.482	1.279	0.262
11008	0.429	0.942	0.296	0.477	1.227	0.309	0.495	1.363	0.467
11009	0.142	-0.895	0.409	0.102	-1.184	0.341	0.295	0.082	0.224
11010	0.505	1.430	0.481	0.450	1.057	0.478	0.476	1.245	0.224
11011	0.495	1.363	0.328	0.485	1.283	0.514	0.505	1.430	0.262
11012	0.468	1.194	0.481	0.485	1.283	0.501	0.489	1.329	0.501
11013	0.263	-0.120	0.328	0.371	0.549	0.512	0.387	0.672	0.328
11014	0.434	0.975	0.358	0.477	1.227	0.341	0.450	1.076	0.501
11015	0.263	-0.120	0.224	0.272	-0.091	0.341	0.097	-1.181	0.481
11016	0.184	-0.625	0.450	0.307	0.135	0.371	0.303	0.133	0.508
11017	0.497	1.380	0.512	0.512	1.453	0.398	0.479	1.262	0.501
11018	0.142	-0.895	0.512	0.275	-0.073	0.371	0.329	0.301	0.501
11019	0.447	1.060	0.501	0.515	1.472	0.398	0.358	0.487	0.224
11020	0.329	0.301	0.508	0.237	-0.317	0.273	0.184	-0.625	0.492
11021	0.508	1.447	0.358	0.497	1.359	0.371	0.511	1.464	0.512
11022	0.508	1.447	0.142	0.421	0.869	0.193	0.479	1.262	0.262
11023	0.226	-0.356	0.492	0.310	0.153	0.512	0.142	-0.895	0.262
11024	0.184	-0.625	0.508	0.342	0.361	0.273	0.050	-1.485	0.224
11025	0.505	1.430	0.450	0.474	1.208	0.273	0.487	1.312	0.492
11026	0.497	1.380	0.508	0.515	1.472	0.491	0.503	1.413	0.467
11027	0.482	1.279	0.508	0.497	1.359	0.491	0.505	1.430	0.262
11028	0.505	1.430	0.508	0.512	1.453	0.309	0.434	0.975	0.097
11029	0.187	-0.608	0.467	0.342	0.361	0.193	0.226	-0.356	0.508
11030	0.224	-0.373	0.501	0.339	0.342	0.235	0.187	-0.608	0.431
11031	0.187	-0.608	0.142	0.371	0.549	0.508	0.097	-1.181	0.508
11032	0.100	-1.164	0.501	0.398	0.718	0.193	0.263	-0.120	0.501
11033	0.000	?	0.513	0.368	0.530	0.235	0.224	-0.373	0.296
11034	0.100	-1.164	0.450	0.398	0.718	0.149	0.263	-0.120	0.142
11035	0.050	-1.485	0.481	0.278	-0.054	0.341	0.326	0.285	0.328
11036	0.000	?	0.492	0.371	0.549	0.422	0.142	-0.895	0.296
11037	0.329	0.301	0.185	0.193	-0.600	0.193	0.097	-1.181	0.262

11038	0.432	0.958	0.262	0.512	1.453	0.478	0.476	1.245	0.262
11039	0.224	-0.373	0.262	0.275	-0.073	0.508	0.184	-0.625	0.328
11040	0.261	-0.137	0.296	0.278	-0.054	0.508	0.261	-0.137	0.512
11041	0.497	1.380	0.481	0.491	1.321	0.491	0.503	1.413	0.467
11042	0.511	1.464	0.328	0.494	1.340	0.193	0.487	1.312	0.508
11043	0.145	-0.878	0.501	0.307	0.135	0.102	0.261	-0.137	0.296
11044	0.332	0.318	0.224	0.193	-0.600	0.273	0.050	-1.485	0.224
11045	0.142	-0.895	0.492	0.234	-0.336	0.462	0.184	-0.625	0.492
11046	0.479	1.262	0.224	0.509	1.434	0.193	0.487	1.312	0.501
11047	0.224	-0.373	0.296	0.193	-0.600	0.309	0.142	-0.895	0.142
11048	0.261	-0.137	0.450	0.234	-0.336	0.501	0.142	-0.895	0.467
11049	0.224	-0.373	0.224	0.149	-0.882	0.235	0.295	0.082	0.185
11050	0.097	-1.181	0.501	0.339	0.342	0.235	0.097	-1.181	0.185
11051	0.432	0.958	0.508	0.424	0.888	0.235	0.497	1.380	0.142
11052	0.261	-0.137	0.501	0.193	-0.600	0.235	0.187	-0.608	0.501
11053	0.508	1.447	0.508	0.480	1.246	0.341	0.468	1.194	0.481
11054	0.479	1.262	0.508	0.509	1.434	0.501	0.468	1.194	0.481
11055	0.489	1.329	0.508	0.509	1.434	0.478	0.468	1.194	0.508
11056	0.476	1.245	0.492	0.494	1.340	0.501	0.500	1.397	0.467
11057	0.263	-0.120	0.262	0.196	-0.581	0.102	0.097	-1.181	0.142
11058	0.050	-1.485	0.501	0.102	-1.184	0.501	0.387	0.672	0.224
11059	0.297	0.099	0.513	0.275	-0.073	0.508	0.142	-0.895	0.512
11060	0.468	1.194	0.050	0.462	1.133	0.501	0.500	1.397	0.513
11061	0.297	0.099	0.501	0.193	-0.600	0.508	0.050	-1.485	0.431
11062	0.097	-1.181	0.492	0.307	0.135	0.273	0.142	-0.895	0.467
11063	0.479	1.262	0.508	0.500	1.378	0.478	0.495	1.363	0.467
11064	0.142	-0.895	0.481	0.234	-0.336	0.491	0.184	-0.625	0.450
11065	0.142	-0.895	0.224	0.234	-0.336	0.501	0.184	-0.625	0.409
11066	0.142	-0.895	0.501	0.234	-0.336	0.053	0.184	-0.625	0.328
11067	0.184	-0.625	0.512	0.240	-0.299	0.491	0.297	0.099	0.513
11068	0.332	0.318	0.481	0.342	0.361	0.501	0.142	-0.895	0.224
11069	0.495	1.363	0.450	0.503	1.396	0.309	0.508	1.447	0.450
11070	0.495	1.363	0.481	0.480	1.246	0.514	0.511	1.464	0.450
11071	0.487	1.312	0.501	0.500	1.378	0.149	0.508	1.447	0.501
11072	0.361	0.504	0.262	0.102	-1.184	0.514	0.097	-1.181	0.492
11073	0.484	1.295	0.512	0.500	1.378	0.193	0.503	1.413	0.467
11074	0.503	1.413	0.097	0.506	1.415	0.501	0.453	1.093	0.296
11075	0.434	0.975	0.513	0.500	1.378	0.371	0.497	1.380	0.185
11076	0.432	0.958	0.492	0.506	1.415	0.273	0.500	1.397	0.185
11077	0.142	-0.895	0.508	0.275	-0.073	0.491	0.226	-0.356	0.296
11078	0.492	1.346	0.513	0.477	1.227	0.501	0.466	1.178	0.492
11079	0.503	1.413	0.512	0.497	1.359	0.341	0.487	1.312	0.262
11080	0.508	1.447	0.492	0.503	1.396	0.514	0.453	1.093	0.492
11081	0.295	0.082	0.481	0.053	-1.504	0.491	0.184	-0.625	0.097
11082	0.432	0.958	0.142	0.488	1.302	0.508	0.511	1.464	0.492
11083	0.503	1.413	0.450	0.500	1.378	0.491	0.497	1.380	0.262
11084	0.142	-0.895	0.450	0.310	0.153	0.309	0.100	-1.164	0.224
11085	0.516	1.498	0.450	0.512	1.453	0.491	0.434	0.975	0.512
11086	0.184	-0.625	0.512	0.152	-0.863	0.273	0.263	-0.120	0.142
11087	0.516	1.498	0.492	0.512	1.453	0.193	0.434	0.975	0.501
11088	0.297	0.099	0.512	0.193	-0.600	0.478	0.184	-0.625	0.501
11089	0.471	1.211	0.328	0.491	1.321	0.273	0.479	1.262	0.481
11090	0.050	-1.485	0.185	0.368	0.530	0.235	0.097	-1.181	0.501
11091	0.261	-0.137	0.512	0.275	-0.073	0.102	0.145	-0.878	0.501
11092	0.484	1.295	0.501	0.491	1.321	0.512	0.511	1.464	0.501
11093	0.511	1.464	0.512	0.500	1.378	0.235	0.461	1.144	0.512
11094	0.479	1.262	0.512	0.339	0.342	0.512	0.476	1.245	0.385
11095	0.474	1.228	0.508	0.518	1.491	0.512	0.518	1.514	0.467
11096	0.513	1.481	0.512	0.497	1.359	0.462	0.511	1.464	0.481
11097	0.511	1.464	0.508	0.515	1.472	0.462	0.450	1.076	0.385
11098	0.513	1.481	0.224	0.497	1.359	0.462	0.511	1.464	0.467

11099	0.355	0.470	0.185	0.310	0.153	0.273	0.266	-0.103	0.501
11100	0.508	1.447	0.328	0.488	1.302	0.491	0.492	1.346	0.467
11101	0.263	-0.120	0.492	0.275	-0.073	0.508	0.411	0.824	0.512
11102	0.263	-0.120	0.492	0.193	-0.600	0.235	0.266	-0.103	0.097
11103	0.503	1.413	0.185	0.474	1.208	0.235	0.463	1.161	0.513
11104	0.097	-1.181	0.481	0.272	-0.091	0.273	0.224	-0.373	0.508
11105	0.097	-1.181	0.481	0.234	-0.336	0.514	0.224	-0.373	0.512
11106	0.184	-0.625	0.512	0.105	-1.165	0.512	0.263	-0.120	0.512
11107	0.450	1.076	0.296	0.515	1.472	0.193	0.503	1.413	0.512
11108	0.145	-0.878	0.513	0.234	-0.336	0.273	0.332	0.318	0.467
11109	0.445	1.043	0.481	0.515	1.472	0.462	0.497	1.380	0.097
11110	0.482	1.279	0.050	0.509	1.434	0.508	0.479	1.262	0.481
11111	0.511	1.464	0.512	0.459	1.114	0.501	0.508	1.447	0.224
11112	0.503	1.413	0.512	0.459	1.114	0.491	0.500	1.397	0.467
11113	0.503	1.413	0.358	0.459	1.114	0.193	0.500	1.397	0.513
11114	0.187	-0.608	0.481	0.272	-0.091	0.309	0.226	-0.356	0.467
11115	0.500	1.397	0.513	0.494	1.340	0.102	0.495	1.363	0.512
11116	0.426	0.925	0.481	0.509	1.434	0.053	0.505	1.430	0.513
11117	0.142	-0.895	0.450	0.234	-0.336	0.273	0.261	-0.137	0.501
11118	0.184	-0.625	0.185	0.234	-0.336	0.309	0.261	-0.137	0.450
11119	0.142	-0.895	0.262	0.272	-0.091	0.193	0.224	-0.373	0.224
11120	0.500	1.397	0.262	0.509	1.434	0.462	0.497	1.380	0.492
11121	0.508	1.447	0.492	0.509	1.434	0.508	0.468	1.194	0.513
11122	0.224	-0.373	0.508	0.193	-0.600	0.491	0.261	-0.137	0.512
11123	0.224	-0.373	0.512	0.278	-0.054	0.149	0.097	-1.181	0.501
11124	0.429	0.942	0.508	0.465	1.152	0.102	0.500	1.397	0.508
11125	0.445	1.043	0.358	0.509	1.434	0.462	0.426	0.925	0.501
11126	0.495	1.363	0.467	0.494	1.340	0.514	0.511	1.464	0.050
11127	0.511	1.464	0.409	0.488	1.302	0.149	0.505	1.430	0.185
11128	0.050	-1.485	0.409	0.193	-0.600	0.512	0.295	0.082	0.501
11129	0.261	-0.137	0.328	0.313	0.172	0.193	0.142	-0.895	0.508
11130	0.145	-0.878	0.185	0.102	-1.184	0.309	0.329	0.301	0.409
11131	0.226	-0.356	0.492	0.053	-1.504	0.371	0.295	0.082	0.431
11132	0.263	-0.120	0.097	0.275	-0.073	0.478	0.263	-0.120	0.431
11133	0.263	-0.120	0.262	0.310	0.153	0.512	0.263	-0.120	0.450
11134	0.000	?	0.185	0.193	-0.600	0.512	0.329	0.301	0.512
11135	0.505	1.430	0.492	0.459	1.114	0.444	0.511	1.464	0.262
11136	0.503	1.413	0.492	0.512	1.453	0.491	0.463	1.161	0.224
11137	0.489	1.329	0.409	0.491	1.321	0.371	0.511	1.464	0.431
11138	0.097	-1.181	0.492	0.149	-0.882	0.193	0.295	0.082	0.467
11139	0.297	0.099	0.262	0.102	-1.184	0.501	0.224	-0.373	0.467
11140	0.405	0.790	0.358	0.462	1.133	0.444	0.489	1.329	0.142
11141	0.479	1.262	0.296	0.509	1.434	0.508	0.497	1.380	0.512
11142	0.329	0.301	0.296	0.149	-0.882	0.501	0.142	-0.895	0.431
11143	0.484	1.295	0.508	0.512	1.453	0.501	0.463	1.161	0.508
11144	0.329	0.301	0.142	0.193	-0.600	0.501	0.184	-0.625	0.142
11145	0.361	0.504	0.262	0.310	0.153	0.462	0.184	-0.625	0.501
11146	0.224	-0.373	0.262	0.374	0.568	0.235	0.142	-0.895	0.501
11147	0.447	1.060	0.328	0.477	1.227	0.398	0.505	1.430	0.224
11148	0.513	1.481	0.467	0.509	1.434	0.371	0.484	1.295	0.512
11149	0.513	1.481	0.262	0.509	1.434	0.512	0.484	1.295	0.508
11150	0.503	1.413	0.296	0.442	1.001	0.514	0.500	1.397	0.409
11151	0.503	1.413	0.513	0.488	1.302	0.501	0.466	1.178	0.508
11152	0.361	0.504	0.385	0.371	0.549	0.398	0.142	-0.895	0.492
11153	0.142	-0.895	0.501	0.193	-0.600	0.512	0.226	-0.356	0.508
11154	0.489	1.329	0.492	0.500	1.378	0.053	0.513	1.481	0.512
11155	0.511	1.464	0.467	0.444	1.020	0.102	0.511	1.464	0.328
11156	0.445	1.043	0.512	0.503	1.396	0.102	0.432	0.958	0.224
11157	0.503	1.413	0.492	0.497	1.359	0.053	0.468	1.194	0.224
11158	0.503	1.413	0.142	0.497	1.359	0.491	0.468	1.194	0.512
11159	0.503	1.413	0.296	0.497	1.359	0.273	0.450	1.076	0.501

11160	0.468	1.194	0.296	0.465	1.152	0.462	0.411	0.824	0.450
11161	0.000	?	0.296	0.237	-0.317	0.235	0.329	0.301	0.467
11162	0.495	1.363	0.296	0.401	0.737	0.478	0.505	1.430	0.097
11163	0.142	-0.895	0.501	0.371	0.549	0.273	0.226	-0.356	0.467
11164	0.497	1.380	0.492	0.509	1.434	0.273	0.447	1.060	0.512
11165	0.513	1.481	0.467	0.515	1.472	0.512	0.453	1.093	0.492
11166	0.447	1.060	0.185	0.500	1.378	0.235	0.503	1.413	0.262
11167	0.476	1.245	0.492	0.401	0.737	0.514	0.497	1.380	0.185
11168	0.489	1.329	0.185	0.509	1.434	0.501	0.463	1.161	0.501
11169	0.184	-0.625	0.501	0.053	-1.504	0.514	0.295	0.082	0.262
11170	0.263	-0.120	0.358	0.102	-1.184	0.514	0.184	-0.625	0.262
11171	0.263	-0.120	0.450	0.102	-1.184	0.514	0.184	-0.625	0.185
11172	0.297	0.099	0.097	0.053	-1.504	0.514	0.297	0.099	0.508
11173	0.482	1.279	0.185	0.488	1.302	0.478	0.495	1.363	0.501
11174	0.329	0.301	0.508	0.272	-0.091	0.235	0.261	-0.137	0.224
11175	0.497	1.380	0.224	0.462	1.133	0.514	0.487	1.312	0.450
11176	0.224	-0.373	0.481	0.234	-0.336	0.462	0.097	-1.181	0.296
11177	0.489	1.329	0.508	0.480	1.246	0.193	0.484	1.295	0.328
11178	0.226	-0.356	0.328	0.272	-0.091	0.512	0.261	-0.137	0.224
11179	0.297	0.099	0.512	0.275	-0.073	0.508	0.226	-0.356	0.512
11180	0.450	1.076	0.142	0.515	1.472	0.514	0.500	1.397	0.296
11181	0.224	-0.373	0.501	0.234	-0.336	0.462	0.145	-0.878	0.385
11182	0.495	1.363	0.492	0.515	1.472	0.273	0.500	1.397	0.385
11183	0.505	1.430	0.224	0.497	1.359	0.491	0.497	1.380	0.508
11184	0.497	1.380	0.467	0.509	1.434	0.478	0.479	1.262	0.142
11185	0.505	1.430	0.481	0.509	1.434	0.512	0.500	1.397	0.262
11186	0.505	1.430	0.508	0.509	1.434	0.512	0.500	1.397	0.513
11187	0.505	1.430	0.508	0.509	1.434	0.512	0.500	1.397	0.185
11188	0.489	1.329	0.481	0.480	1.246	0.508	0.511	1.464	0.501
11189	0.263	-0.120	0.450	0.234	-0.336	0.235	0.387	0.672	0.508
11190	0.497	1.380	0.501	0.515	1.472	0.501	0.463	1.161	0.508
11191	0.508	1.447	0.508	0.462	1.133	0.235	0.482	1.279	0.450
11192	0.050	-1.485	0.224	0.193	-0.600	0.501	0.387	0.672	0.508
11193	0.495	1.363	0.385	0.515	1.472	0.501	0.461	1.144	0.296
11194	0.489	1.329	0.512	0.503	1.396	0.501	0.500	1.397	0.262
11195	0.505	1.430	0.512	0.509	1.434	0.462	0.466	1.178	0.262
11196	0.487	1.312	0.508	0.465	1.152	0.462	0.508	1.447	0.262
11197	0.226	-0.356	0.492	0.272	-0.091	0.491	0.097	-1.181	0.262
11198	0.492	1.346	0.328	0.497	1.359	0.508	0.508	1.447	0.508
11199	0.513	1.481	0.142	0.480	1.246	0.193	0.500	1.397	0.409
11200	0.479	1.262	0.481	0.509	1.434	0.491	0.508	1.447	0.512
11201	0.447	1.060	0.450	0.509	1.434	0.501	0.508	1.447	0.262
11202	0.479	1.262	0.224	0.509	1.434	0.501	0.508	1.447	0.492
11203	0.500	1.397	0.262	0.509	1.434	0.512	0.468	1.194	0.481
11204	0.261	-0.137	0.296	0.234	-0.336	0.501	0.097	-1.181	0.142
11205	0.511	1.464	0.481	0.500	1.378	0.462	0.476	1.245	0.467
11206	0.097	-1.181	0.508	0.237	-0.317	0.149	0.226	-0.356	0.224
11207	0.508	1.447	0.501	0.506	1.415	0.193	0.471	1.211	0.224
11208	0.492	1.346	0.481	0.500	1.378	0.462	0.518	1.514	0.467
11209	0.505	1.430	0.508	0.506	1.415	0.462	0.466	1.178	0.050
11210	0.513	1.481	0.501	0.459	1.114	0.512	0.513	1.481	0.450
11211	0.503	1.413	0.512	0.462	1.133	0.512	0.513	1.481	0.481
11212	0.489	1.329	0.512	0.494	1.340	0.512	0.500	1.397	0.050
11213	0.482	1.279	0.097	0.509	1.434	0.512	0.447	1.060	0.481
11214	0.142	-0.895	0.142	0.193	-0.600	0.491	0.224	-0.373	0.224
11215	0.450	1.076	0.097	0.488	1.302	0.273	0.489	1.329	0.508
11216	0.453	1.093	0.050	0.506	1.415	0.273	0.511	1.464	0.501
11217	0.453	1.093	0.512	0.506	1.415	0.273	0.508	1.447	0.262
11218	0.511	1.464	0.501	0.509	1.434	0.491	0.503	1.413	0.512
11219	0.492	1.346	0.512	0.500	1.378	0.478	0.508	1.447	0.358
11220	0.511	1.464	0.501	0.456	1.095	0.235	0.500	1.397	0.467

11221	0.329	0.301	0.385	0.152	-0.863	0.371	0.050	-1.485	0.467
11222	0.184	-0.625	0.481	0.196	-0.581	0.478	0.187	-0.608	0.512
11223	0.511	1.464	0.050	0.462	1.133	0.491	0.495	1.363	0.508
11224	0.500	1.397	0.097	0.462	1.133	0.193	0.503	1.413	0.513
11225	0.508	1.447	0.512	0.512	1.453	0.478	0.408	0.807	0.508
11226	0.508	1.447	0.185	0.512	1.453	0.501	0.429	0.942	0.142
11227	0.503	1.413	0.481	0.512	1.453	0.309	0.429	0.942	0.501
11228	0.508	1.447	0.142	0.512	1.453	0.491	0.450	1.076	0.492
11229	0.508	1.447	0.467	0.488	1.302	0.491	0.511	1.464	0.492
11230	0.224	-0.373	0.508	0.275	-0.073	0.341	0.263	-0.120	0.431
11231	0.184	-0.625	0.501	0.275	-0.073	0.512	0.226	-0.356	0.508
11232	0.329	0.301	0.481	0.275	-0.073	0.508	0.434	0.975	0.050
11233	0.487	1.312	0.492	0.494	1.340	0.514	0.463	1.161	0.050
11234	0.487	1.312	0.508	0.480	1.246	0.514	0.463	1.161	0.492
11235	0.184	-0.625	0.513	0.234	-0.336	0.235	0.142	-0.895	0.481
11236	0.479	1.262	0.296	0.371	0.549	0.149	0.511	1.464	0.050
11237	0.479	1.262	0.097	0.482	1.265	0.149	0.429	0.942	0.328
11238	0.516	1.498	0.097	0.482	1.265	0.462	0.505	1.430	0.328
11239	0.297	0.099	0.050	0.193	-0.600	0.462	0.142	-0.895	0.467
11240	0.513	1.481	0.481	0.480	1.246	0.444	0.495	1.363	0.501
11241	0.484	1.295	0.481	0.503	1.396	0.371	0.495	1.363	0.450
11242	0.050	-1.485	0.492	0.307	0.135	0.309	0.226	-0.356	0.508
11243	0.511	1.464	0.508	0.488	1.302	0.444	0.508	1.447	0.501
11244	0.511	1.464	0.224	0.488	1.302	0.514	0.505	1.430	0.467
11245	0.361	0.504	0.492	0.339	0.342	0.478	0.413	0.841	0.501
11246	0.482	1.279	0.328	0.515	1.472	0.309	0.511	1.464	0.481
11247	0.516	1.498	0.328	0.509	1.434	0.273	0.492	1.346	0.224
11248	0.482	1.279	0.508	0.515	1.472	0.512	0.511	1.464	0.296
11249	0.453	1.093	0.481	0.515	1.472	0.235	0.513	1.481	0.185
11250	0.184	-0.625	0.501	0.237	-0.317	0.341	0.332	0.318	0.409
11251	0.266	-0.103	0.512	0.149	-0.882	0.193	0.224	-0.373	0.431
11252	0.266	-0.103	0.508	0.149	-0.882	0.512	0.224	-0.373	0.512
11253	0.492	1.346	0.512	0.465	1.152	0.508	0.508	1.447	0.508
11254	0.508	1.447	0.508	0.462	1.133	0.309	0.495	1.363	0.508
11255	0.513	1.481	0.508	0.447	1.039	0.491	0.447	1.060	0.508
11256	0.511	1.464	0.513	0.377	0.587	0.371	0.466	1.178	0.050
11257	0.358	0.487	0.513	0.310	0.153	0.309	0.097	-1.181	0.508
11258	0.471	1.211	0.513	0.442	1.001	0.273	0.466	1.178	0.262
11259	0.411	0.824	0.492	0.512	1.453	0.273	0.503	1.413	0.224
11260	0.408	0.807	0.501	0.480	1.246	0.512	0.495	1.363	0.508
11261	0.329	0.301	0.450	0.310	0.153	0.273	0.261	-0.137	0.185
11262	0.187	-0.608	0.142	0.272	-0.091	0.309	0.187	-0.608	0.409
11263	0.489	1.329	0.142	0.512	1.453	0.309	0.500	1.397	0.097
11264	0.097	-1.181	0.481	0.234	-0.336	0.193	0.263	-0.120	0.501
11265	0.263	-0.120	0.508	0.345	0.379	0.512	0.261	-0.137	0.501
11266	0.184	-0.625	0.508	0.193	-0.600	0.149	0.184	-0.625	0.450
11267	0.497	1.380	0.492	0.515	1.472	0.273	0.503	1.413	0.467
11268	0.492	1.346	0.512	0.506	1.415	0.444	0.495	1.363	0.481
11269	0.411	0.824	0.467	0.310	0.153	0.102	0.226	-0.356	0.508
11270	0.492	1.346	0.262	0.491	1.321	0.462	0.447	1.060	0.481
11271	0.263	-0.120	0.050	0.371	0.549	0.462	0.295	0.082	0.224
11272	0.361	0.504	0.224	0.310	0.153	0.512	0.329	0.301	0.050
11273	0.297	0.099	0.097	0.275	-0.073	0.501	0.000	?	0.142
11274	0.300	0.116	0.508	0.275	-0.073	0.462	0.224	-0.373	0.142
11275	0.505	1.430	0.185	0.512	1.453	0.193	0.508	1.447	0.224
11276	0.142	-0.895	0.512	0.275	-0.073	0.273	0.300	0.116	0.512
11277	0.266	-0.103	0.050	0.310	0.153	0.273	0.384	0.655	0.512
11278	0.266	-0.103	0.142	0.310	0.153	0.273	0.384	0.655	0.512
11279	0.329	0.301	0.185	0.196	-0.581	0.273	0.000	?	0.467
11280	0.471	1.211	0.508	0.509	1.434	0.491	0.505	1.430	0.385
11281	0.261	-0.137	0.513	0.149	-0.882	0.514	0.142	-0.895	0.492

11282	0.295	0.082	0.481	0.272	-0.091	0.444	0.263	-0.120	0.050
11283	0.511	1.464	0.050	0.442	1.001	0.149	0.513	1.481	0.097
11284	0.387	0.672	0.508	0.102	-1.184	0.478	0.184	-0.625	0.467
11285	0.497	1.380	0.512	0.465	1.152	0.341	0.500	1.397	0.512
11286	0.497	1.380	0.512	0.465	1.152	0.512	0.508	1.447	0.508
11287	0.466	1.178	0.512	0.515	1.472	0.193	0.508	1.447	0.492
11288	0.503	1.413	0.512	0.500	1.378	0.478	0.450	1.076	0.481
11289	0.487	1.312	0.262	0.459	1.114	0.273	0.508	1.447	0.450
11290	0.145	-0.878	0.481	0.196	-0.581	0.422	0.295	0.082	0.501
11291	0.300	0.116	0.501	0.275	-0.073	0.512	0.261	-0.137	0.492
11292	0.300	0.116	0.501	0.275	-0.073	0.273	0.261	-0.137	0.512
11293	0.300	0.116	0.501	0.275	-0.073	0.462	0.261	-0.137	0.508
11294	0.300	0.116	0.513	0.275	-0.073	0.491	0.261	-0.137	0.508
11295	0.500	1.397	0.224	0.491	1.321	0.235	0.513	1.481	0.481
11296	0.489	1.329	0.431	0.512	1.453	0.491	0.411	0.824	0.467
11297	0.471	1.211	0.501	0.444	1.020	0.193	0.505	1.430	0.508
11298	0.184	-0.625	0.501	0.152	-0.863	0.309	0.263	-0.120	0.097
11299	0.492	1.346	0.097	0.477	1.227	0.491	0.487	1.312	0.262
11300	0.187	-0.608	0.142	0.339	0.342	0.193	0.000	?	0.501
11301	0.497	1.380	0.481	0.518	1.491	0.422	0.479	1.262	0.512
11302	0.361	0.504	0.481	0.193	-0.600	0.491	0.142	-0.895	0.512
11303	0.447	1.060	0.224	0.480	1.246	0.478	0.461	1.144	0.481
11304	0.097	-1.181	0.262	0.275	-0.073	0.478	0.224	-0.373	0.481
11305	0.184	-0.625	0.262	0.427	0.907	0.501	0.224	-0.373	0.508
11306	0.508	1.447	0.492	0.515	1.472	0.462	0.466	1.178	0.142
11307	0.224	-0.373	0.512	0.272	-0.091	0.491	0.050	-1.485	0.097
11308	0.479	1.262	0.508	0.459	1.114	0.514	0.453	1.093	0.262
11309	0.505	1.430	0.262	0.485	1.283	0.193	0.479	1.262	0.185
11310	0.332	0.318	0.142	0.234	-0.336	0.512	0.050	-1.485	0.142
11311	0.508	1.447	0.501	0.488	1.302	0.512	0.482	1.279	0.296
11312	0.142	-0.895	0.512	0.193	-0.600	0.512	0.226	-0.356	0.512
11313	0.497	1.380	0.467	0.307	0.135	0.512	0.508	1.447	0.328
11314	0.492	1.346	0.296	0.488	1.302	0.444	0.495	1.363	0.508
11315	0.224	-0.373	0.492	0.196	-0.581	0.149	0.263	-0.120	0.097
11316	0.463	1.161	0.467	0.421	0.869	0.341	0.505	1.430	0.328
11317	0.482	1.279	0.467	0.494	1.340	0.478	0.358	0.487	0.296
11318	0.508	1.447	0.467	0.480	1.246	0.501	0.468	1.194	0.450
11319	0.508	1.447	0.450	0.480	1.246	0.273	0.468	1.194	0.513
11320	0.482	1.279	0.501	0.500	1.378	0.371	0.511	1.464	0.513
11321	0.450	1.076	0.492	0.465	1.152	0.398	0.503	1.413	0.296
11322	0.503	1.413	0.512	0.491	1.321	0.501	0.516	1.498	0.262
11323	0.505	1.430	0.097	0.509	1.434	0.478	0.505	1.430	0.224
11324	0.224	-0.373	0.050	0.193	-0.600	0.512	0.142	-0.895	0.508
11325	0.387	0.672	0.142	0.515	1.472	0.478	0.503	1.413	0.409
11326	0.513	1.481	0.409	0.500	1.378	0.512	0.492	1.346	0.501
11327	0.513	1.481	0.142	0.500	1.378	0.478	0.492	1.346	0.296
11328	0.503	1.413	0.185	0.512	1.453	0.462	0.429	0.942	0.467
11329	0.489	1.329	0.224	0.444	1.020	0.491	0.511	1.464	0.296
11330	0.332	0.318	0.296	0.149	-0.882	0.235	0.050	-1.485	0.508
11331	0.142	-0.895	0.508	0.345	0.379	0.149	0.050	-1.485	0.050
11332	0.482	1.279	0.508	0.480	1.246	0.398	0.484	1.295	0.328
11333	0.450	1.076	0.296	0.500	1.378	0.398	0.474	1.228	0.492
11334	0.224	-0.373	0.501	0.278	-0.054	0.341	0.050	-1.485	0.512
11335	0.261	-0.137	0.508	0.374	0.568	0.398	0.332	0.318	0.512
11336	0.295	0.082	0.185	0.401	0.737	0.501	0.332	0.318	0.512
11337	0.484	1.295	0.097	0.497	1.359	0.501	0.461	1.144	0.481
11338	0.497	1.380	0.450	0.480	1.246	0.491	0.505	1.430	0.142
11339	0.503	1.413	0.492	0.506	1.415	0.478	0.445	1.043	0.508
11340	0.482	1.279	0.513	0.477	1.227	0.235	0.505	1.430	0.501
11341	0.505	1.430	0.513	0.506	1.415	0.371	0.500	1.397	0.508
11342	0.495	1.363	0.513	0.474	1.208	0.341	0.468	1.194	0.185

11343	0.503	1.413	0.501	0.462	1.133	0.273	0.500	1.397	0.262
11344	0.505	1.430	0.142	0.494	1.340	0.501	0.484	1.295	0.513
11345	0.097	-1.181	0.296	0.234	-0.336	0.193	0.224	-0.373	0.512
11346	0.142	-0.895	0.513	0.152	-0.863	0.491	0.297	0.099	0.296
11347	0.000	?	0.513	0.401	0.737	0.309	0.184	-0.625	0.185
11348	0.097	-1.181	0.481	0.401	0.737	0.491	0.000	?	0.296
11349	0.000	?	0.328	0.342	0.361	0.512	0.411	0.824	0.508
11350	0.050	-1.485	0.296	0.398	0.718	0.462	0.434	0.975	0.512
11351	0.513	1.481	0.296	0.506	1.415	0.508	0.505	1.430	0.481
11352	0.503	1.413	0.481	0.506	1.415	0.508	0.503	1.413	0.262
11353	0.513	1.481	0.467	0.494	1.340	0.462	0.503	1.413	0.097
11354	0.503	1.413	0.481	0.482	1.265	0.508	0.503	1.413	0.450
11355	0.387	0.672	0.481	0.234	-0.336	0.309	0.050	-1.485	0.481
11356	0.474	1.228	0.142	0.374	0.568	0.309	0.505	1.430	0.501
11357	0.050	-1.485	0.224	0.345	0.379	0.371	0.261	-0.137	0.185
11358	0.097	-1.181	0.508	0.275	-0.073	0.371	0.224	-0.373	0.492
11359	0.508	1.447	0.097	0.497	1.359	0.273	0.505	1.430	0.481
11360	0.184	-0.625	0.508	0.193	-0.600	0.491	0.184	-0.625	0.481
11361	0.476	1.245	0.508	0.491	1.321	0.491	0.408	0.807	0.501
11362	0.145	-0.878	0.501	0.310	0.153	0.462	0.097	-1.181	0.409
11363	0.466	1.178	0.481	0.491	1.321	0.514	0.495	1.363	0.492
11364	0.511	1.464	0.492	0.512	1.453	0.149	0.492	1.346	0.492
11365	0.503	1.413	0.508	0.465	1.152	0.491	0.445	1.043	0.481
11366	0.476	1.245	0.512	0.509	1.434	0.149	0.463	1.161	0.185
11367	0.489	1.329	0.501	0.509	1.434	0.102	0.476	1.245	0.185
11368	0.505	1.430	0.501	0.459	1.114	0.462	0.508	1.447	0.296
11369	0.503	1.413	0.142	0.512	1.453	0.444	0.479	1.262	0.492
11370	0.000	?	0.097	0.310	0.153	0.508	0.224	-0.373	0.262
11371	0.297	0.099	0.508	0.307	0.135	0.512	0.050	-1.485	0.358
11372	0.097	-1.181	0.467	0.371	0.549	0.512	0.142	-0.895	0.050
11373	0.097	-1.181	0.492	0.371	0.549	0.514	0.142	-0.895	0.097
11374	0.050	-1.485	0.431	0.272	-0.091	0.512	0.224	-0.373	0.513
11375	0.479	1.262	0.050	0.488	1.302	0.501	0.508	1.447	0.501
11376	0.479	1.262	0.224	0.488	1.302	0.501	0.508	1.447	0.142
11377	0.489	1.329	0.224	0.462	1.133	0.501	0.508	1.447	0.467
11378	0.497	1.380	0.097	0.520	1.509	0.478	0.461	1.144	0.481
11379	0.226	-0.356	0.142	0.149	-0.882	0.478	0.387	0.672	0.328
11380	0.489	1.329	0.508	0.494	1.340	0.501	0.495	1.363	0.385
11381	0.329	0.301	0.501	0.149	-0.882	0.514	0.050	-1.485	0.508
11382	0.329	0.301	0.501	0.102	-1.184	0.309	0.097	-1.181	0.481
11383	0.508	1.447	0.450	0.459	1.114	0.193	0.461	1.144	0.501
11384	0.482	1.279	0.224	0.442	1.001	0.508	0.503	1.413	0.508
11385	0.497	1.380	0.513	0.506	1.415	0.501	0.505	1.430	0.508
11386	0.505	1.430	0.513	0.515	1.472	0.501	0.487	1.312	0.512
11387	0.500	1.397	0.185	0.509	1.434	0.514	0.482	1.279	0.409
11388	0.505	1.430	0.492	0.512	1.453	0.508	0.447	1.060	0.097
11389	0.500	1.397	0.050	0.515	1.472	0.371	0.500	1.397	0.492
11390	0.500	1.397	0.481	0.503	1.396	0.149	0.489	1.329	0.492
11391	0.500	1.397	0.501	0.506	1.415	0.422	0.513	1.481	0.492
11392	0.500	1.397	0.185	0.506	1.415	0.422	0.511	1.464	0.097
11393	0.500	1.397	0.431	0.482	1.265	0.149	0.511	1.464	0.262
11394	0.489	1.329	0.467	0.480	1.246	0.341	0.482	1.279	0.296
11395	0.497	1.380	0.492	0.497	1.359	0.273	0.466	1.178	0.508
11396	0.447	1.060	0.185	0.509	1.434	0.491	0.511	1.464	0.481
11397	0.142	-0.895	0.328	0.313	0.172	0.102	0.097	-1.181	0.492
11398	0.142	-0.895	0.501	0.196	-0.581	0.462	0.263	-0.120	0.492
11399	0.476	1.245	0.296	0.506	1.415	0.371	0.500	1.397	0.431
11400	0.500	1.397	0.492	0.506	1.415	0.273	0.505	1.430	0.185
11401	0.500	1.397	0.358	0.506	1.415	0.235	0.505	1.430	0.467
11402	0.484	1.295	0.467	0.518	1.491	0.501	0.482	1.279	0.097
11403	0.503	1.413	0.431	0.512	1.453	0.514	0.482	1.279	0.512

11404	0.463	1.161	0.262	0.371	0.549	0.508	0.511	1.464	0.501
11405	0.263	-0.120	0.262	0.149	-0.882	0.371	0.142	-0.895	0.481
11406	0.000	?	0.481	0.424	0.888	0.398	0.097	-1.181	0.513
11407	0.050	-1.485	0.097	0.421	0.869	0.309	0.263	-0.120	0.501
11408	0.226	-0.356	0.508	0.149	-0.882	0.422	0.187	-0.608	0.481
11409	0.097	-1.181	0.450	0.345	0.379	0.462	0.142	-0.895	0.481
11410	0.000	?	0.296	0.272	-0.091	0.491	0.297	0.099	0.481
11411	0.497	1.380	0.481	0.491	1.321	0.341	0.513	1.481	0.467
11412	0.184	-0.625	0.481	0.102	-1.184	0.491	0.332	0.318	0.224
11413	0.503	1.413	0.224	0.465	1.152	0.371	0.511	1.464	0.409
11414	0.050	-1.485	0.328	0.368	0.530	0.462	0.097	-1.181	0.409
11415	0.142	-0.895	0.296	0.272	-0.091	0.422	0.329	0.301	0.409
11416	0.187	-0.608	0.358	0.234	-0.336	0.149	0.295	0.082	0.508
11417	0.497	1.380	0.296	0.503	1.396	0.462	0.447	1.060	0.501
11418	0.503	1.413	0.501	0.518	1.491	0.514	0.511	1.464	0.224
11419	0.474	1.228	0.409	0.512	1.453	0.514	0.516	1.498	0.142
11420	0.050	-1.485	0.409	0.377	0.587	0.514	0.295	0.082	0.050
11421	0.000	?	0.467	0.401	0.737	0.478	0.263	-0.120	0.262
11422	0.000	?	0.296	0.307	0.135	0.193	0.224	-0.373	0.224
11423	0.503	1.413	0.508	0.424	0.888	0.501	0.508	1.447	0.450
11424	0.505	1.430	0.450	0.465	1.152	0.478	0.411	0.824	0.492
11425	0.500	1.397	0.431	0.497	1.359	0.508	0.497	1.380	0.501
11426	0.000	?	0.492	0.345	0.379	0.273	0.295	0.082	0.224
11427	0.500	1.397	0.492	0.494	1.340	0.341	0.466	1.178	0.262
11428	0.000	?	0.492	0.374	0.568	0.501	0.297	0.099	0.512
11429	0.500	1.397	0.185	0.468	1.170	0.462	0.505	1.430	0.431
11430	0.000	?	0.492	0.421	0.869	0.053	0.050	-1.485	0.142
11431	0.261	-0.137	0.508	0.149	-0.882	0.235	0.326	0.285	0.501
11432	0.474	1.228	0.328	0.465	1.152	0.193	0.489	1.329	0.385
11433	0.497	1.380	0.431	0.509	1.434	0.508	0.511	1.464	0.296
11434	0.497	1.380	0.409	0.509	1.434	0.501	0.511	1.464	0.508
11435	0.497	1.380	0.431	0.509	1.434	0.501	0.511	1.464	0.512
11436	0.508	1.447	0.185	0.477	1.227	0.193	0.482	1.279	0.050
11437	0.224	-0.373	0.492	0.193	-0.600	0.309	0.145	-0.878	0.508
11438	0.429	0.942	0.224	0.503	1.396	0.491	0.500	1.397	0.508
11439	0.497	1.380	0.185	0.474	1.208	0.514	0.503	1.413	0.501
11440	0.492	1.346	0.508	0.512	1.453	0.514	0.505	1.430	0.142
11441	0.097	-1.181	0.492	0.275	-0.073	0.102	0.187	-0.608	0.450
11442	0.142	-0.895	0.481	0.342	0.361	0.478	0.261	-0.137	0.050
11443	0.476	1.245	0.224	0.500	1.378	0.508	0.508	1.447	0.508
11444	0.476	1.245	0.142	0.459	1.114	0.508	0.511	1.464	0.481
11445	0.226	-0.356	0.508	0.053	-1.504	0.422	0.297	0.099	0.508
11446	0.266	-0.103	0.142	0.234	-0.336	0.512	0.184	-0.625	0.097
11447	0.263	-0.120	0.185	0.196	-0.581	0.514	0.297	0.099	0.097
11448	0.492	1.346	0.512	0.506	1.415	0.512	0.500	1.397	0.492
11449	0.511	1.464	0.185	0.497	1.359	0.478	0.511	1.464	0.142
11450	0.513	1.481	0.224	0.500	1.378	0.273	0.482	1.279	0.501
11451	0.263	-0.120	0.262	0.193	-0.600	0.309	0.261	-0.137	0.512
11452	0.142	-0.895	0.296	0.307	0.135	0.149	0.097	-1.181	0.097
11453	0.497	1.380	0.296	0.488	1.302	0.512	0.450	1.076	0.513
11454	0.508	1.447	0.358	0.518	1.491	0.149	0.474	1.228	0.492
11455	0.463	1.161	0.262	0.509	1.434	0.149	0.503	1.413	0.050
11456	0.295	0.082	0.508	0.102	-1.184	0.273	0.184	-0.625	0.097
11457	0.489	1.329	0.508	0.477	1.227	0.273	0.489	1.329	0.481
11458	0.461	1.144	0.467	0.512	1.453	0.478	0.474	1.228	0.097
11459	0.461	1.144	0.492	0.512	1.453	0.491	0.474	1.228	0.492
11460	0.471	1.211	0.224	0.424	0.888	0.273	0.500	1.397	0.512
11461	0.453	1.093	0.508	0.515	1.472	0.508	0.405	0.790	0.492
11462	0.495	1.363	0.358	0.515	1.472	0.501	0.495	1.363	0.492
11463	0.487	1.312	0.467	0.515	1.472	0.149	0.495	1.363	0.097
11464	0.513	1.481	0.262	0.480	1.246	0.398	0.482	1.279	0.501

11465	0.097	-1.181	0.492	0.275	-0.073	0.478	0.187	-0.608	0.512
11466	0.050	-1.485	0.492	0.307	0.135	0.501	0.184	-0.625	0.501
11467	0.142	-0.895	0.512	0.149	-0.882	0.462	0.300	0.116	0.492
11468	0.405	0.790	0.481	0.512	1.453	0.462	0.497	1.380	0.492
11469	0.142	-0.895	0.328	0.149	-0.882	0.462	0.263	-0.120	0.492
11470	0.184	-0.625	0.142	0.149	-0.882	0.501	0.361	0.504	0.185
11471	0.226	-0.356	0.467	0.275	-0.073	0.149	0.050	-1.485	0.492
11472	0.297	0.099	0.097	0.275	-0.073	0.149	0.097	-1.181	0.512
11473	0.500	1.397	0.481	0.477	1.227	0.422	0.516	1.498	0.050
11474	0.503	1.413	0.142	0.494	1.340	0.422	0.497	1.380	0.508
11475	0.297	0.099	0.185	0.272	-0.091	0.491	0.142	-0.895	0.492
11476	0.503	1.413	0.508	0.509	1.434	0.193	0.468	1.194	0.492
11477	0.508	1.447	0.185	0.500	1.378	0.273	0.484	1.295	0.097
11478	0.184	-0.625	0.185	0.149	-0.882	0.273	0.329	0.301	0.492
11479	0.097	-1.181	0.481	0.401	0.737	0.462	0.384	0.655	0.097
11480	0.447	1.060	0.262	0.480	1.246	0.478	0.500	1.397	0.097
11481	0.489	1.329	0.450	0.503	1.396	0.491	0.474	1.228	0.508
11482	0.511	1.464	0.492	0.459	1.114	0.491	0.500	1.397	0.450
11483	0.511	1.464	0.185	0.459	1.114	0.273	0.508	1.447	0.501
11484	0.511	1.464	0.142	0.459	1.114	0.149	0.508	1.447	0.097
11485	0.497	1.380	0.185	0.500	1.378	0.512	0.508	1.447	0.097
11486	0.142	-0.895	0.508	0.152	-0.863	0.341	0.411	0.824	0.409
11487	0.297	0.099	0.508	0.149	-0.882	0.491	0.097	-1.181	0.224
11488	0.508	1.447	0.508	0.421	0.869	0.512	0.489	1.329	0.185
11489	0.508	1.447	0.328	0.421	0.869	0.512	0.489	1.329	0.492
11490	0.476	1.245	0.481	0.491	1.321	0.501	0.489	1.329	0.097
11491	0.326	0.285	0.224	0.193	-0.600	0.512	0.097	-1.181	0.185
11492	0.297	0.099	0.513	0.272	-0.091	0.491	0.261	-0.137	0.185
11493	0.297	0.099	0.508	0.272	-0.091	0.491	0.295	0.082	0.097
11494	0.484	1.295	0.508	0.468	1.170	0.491	0.505	1.430	0.097
11495	0.463	1.161	0.224	0.480	1.246	0.512	0.479	1.262	0.185
11496	0.476	1.245	0.262	0.491	1.321	0.273	0.489	1.329	0.185
11497	0.476	1.245	0.512	0.491	1.321	0.235	0.489	1.329	0.450
11498	0.142	-0.895	0.224	0.278	-0.054	0.491	0.432	0.958	0.481
11499	0.226	-0.356	0.512	0.149	-0.882	0.508	0.184	-0.625	0.513
11500	0.497	1.380	0.385	0.518	1.491	0.508	0.463	1.161	0.481
11501	0.097	-1.181	0.328	0.345	0.379	0.491	0.097	-1.181	0.097
11502	0.497	1.380	0.185	0.491	1.321	0.273	0.513	1.481	0.450
11503	0.497	1.380	0.262	0.515	1.472	0.273	0.503	1.413	0.097
11504	0.492	1.346	0.142	0.515	1.472	0.273	0.482	1.279	0.467
11505	0.476	1.245	0.142	0.497	1.359	0.235	0.511	1.464	0.262
11506	0.484	1.295	0.262	0.509	1.434	0.235	0.503	1.413	0.513
11507	0.500	1.397	0.481	0.485	1.283	0.398	0.484	1.295	0.513
11508	0.503	1.413	0.501	0.488	1.302	0.444	0.484	1.295	0.481
11509	0.495	1.363	0.185	0.485	1.283	0.478	0.484	1.295	0.467
11510	0.492	1.346	0.512	0.512	1.453	0.102	0.471	1.211	0.296
11511	0.142	-0.895	0.224	0.272	-0.091	0.193	0.226	-0.356	0.097
11512	0.097	-1.181	0.224	0.234	-0.336	0.514	0.413	0.841	0.508
11513	0.500	1.397	0.328	0.497	1.359	0.512	0.408	0.807	0.296
11514	0.466	1.178	0.467	0.503	1.396	0.235	0.411	0.824	0.501
11515	0.484	1.295	0.508	0.512	1.453	0.508	0.503	1.413	0.224
11516	0.429	0.942	0.185	0.494	1.340	0.235	0.500	1.397	0.262
11517	0.050	-1.485	0.262	0.272	-0.091	0.235	0.224	-0.373	0.508
11518	0.224	-0.373	0.185	0.278	-0.054	0.508	0.142	-0.895	0.097
11519	0.226	-0.356	0.142	0.275	-0.073	0.491	0.050	-1.485	0.185
11520	0.097	-1.181	0.296	0.237	-0.317	0.309	0.263	-0.120	0.467
11521	0.142	-0.895	0.508	0.237	-0.317	0.422	0.226	-0.356	0.097
11522	0.497	1.380	0.224	0.398	0.718	0.422	0.450	1.076	0.492
11523	0.495	1.363	0.512	0.442	1.001	0.501	0.495	1.363	0.450
11524	0.495	1.363	0.501	0.480	1.246	0.273	0.503	1.413	0.097
11525	0.453	1.093	0.296	0.102	-1.184	0.193	0.224	-0.373	0.224

11526	0.224	-0.373	0.358	0.193	-0.600	0.512	0.261	-0.137	0.142
11527	0.503	1.413	0.512	0.518	1.491	0.235	0.511	1.464	0.481
11528	0.503	1.413	0.385	0.515	1.472	0.341	0.434	0.975	0.481
11529	0.184	-0.625	0.328	0.234	-0.336	0.193	0.142	-0.895	0.492
11530	0.484	1.295	0.185	0.512	1.453	0.514	0.503	1.413	0.097
11531	0.000	?	0.492	0.234	-0.336	0.491	0.387	0.672	0.358
11532	0.050	-1.485	0.224	0.234	-0.336	0.235	0.297	0.099	0.296
11533	0.482	1.279	0.050	0.506	1.415	0.235	0.500	1.397	0.512
11534	0.505	1.430	0.262	0.488	1.302	0.501	0.503	1.413	0.467
11535	0.184	-0.625	0.450	0.307	0.135	0.309	0.050	-1.485	0.450
11536	0.432	0.958	0.142	0.424	0.888	0.512	0.500	1.397	0.296
11537	0.466	1.178	0.142	0.427	0.907	0.491	0.500	1.397	0.450
11538	0.492	1.346	0.513	0.500	1.378	0.149	0.495	1.363	0.142
11539	0.184	-0.625	0.262	0.275	-0.073	0.508	0.145	-0.878	0.492
11540	0.326	0.285	0.224	0.193	-0.600	0.501	0.000	?	0.512
11541	0.503	1.413	0.142	0.509	1.434	0.273	0.445	1.043	0.097
11542	0.297	0.099	0.512	0.234	-0.336	0.273	0.050	-1.485	0.296
11543	0.492	1.346	0.450	0.342	0.361	0.235	0.497	1.380	0.142
11544	0.358	0.487	0.185	0.196	-0.581	0.508	0.000	?	0.224
11545	0.468	1.194	0.431	0.509	1.434	0.193	0.474	1.228	0.224
11546	0.429	0.942	0.097	0.491	1.321	0.398	0.500	1.397	0.050
11547	0.266	-0.103	0.358	0.234	-0.336	0.501	0.097	-1.181	0.501
11548	0.263	-0.120	0.501	0.237	-0.317	0.508	0.097	-1.181	0.512
11549	0.482	1.279	0.296	0.503	1.396	0.444	0.487	1.312	0.262
11550	0.097	-1.181	0.296	0.310	0.153	0.273	0.145	-0.878	0.409
11551	0.508	1.447	0.450	0.515	1.472	0.491	0.492	1.346	0.142
11552	0.455	1.110	0.512	0.494	1.340	0.501	0.503	1.413	0.097
11553	0.295	0.082	0.513	0.149	-0.882	0.512	0.097	-1.181	0.262
11554	0.479	1.262	0.097	0.515	1.472	0.514	0.508	1.447	0.508
11555	0.484	1.295	0.262	0.500	1.378	0.514	0.484	1.295	0.492
11556	0.224	-0.373	0.262	0.275	-0.073	0.512	0.050	-1.485	0.097
11557	0.329	0.301	0.262	0.272	-0.091	0.193	0.000	?	0.328
11558	0.297	0.099	0.262	0.237	-0.317	0.512	0.097	-1.181	0.224
11559	0.358	0.487	0.328	0.509	1.434	0.462	0.474	1.228	0.185
11560	0.297	0.099	0.492	0.193	-0.600	0.149	0.097	-1.181	0.328
11561	0.500	1.397	0.358	0.398	0.718	0.512	0.487	1.312	0.501
11562	0.411	0.824	0.224	0.503	1.396	0.514	0.508	1.447	0.097
11563	0.411	0.824	0.224	0.509	1.434	0.512	0.487	1.312	0.481
11564	0.468	1.194	0.224	0.444	1.020	0.273	0.487	1.312	0.492
11565	0.295	0.082	0.224	0.275	-0.073	0.501	0.097	-1.181	0.262
11566	0.505	1.430	0.262	0.491	1.321	0.309	0.495	1.363	0.296
11567	0.455	1.110	0.501	0.500	1.378	0.273	0.503	1.413	0.481
11568	0.429	0.942	0.262	0.509	1.434	0.444	0.497	1.380	0.513
11569	0.495	1.363	0.501	0.512	1.453	0.422	0.489	1.329	0.142
11570	0.495	1.363	0.262	0.512	1.453	0.462	0.489	1.329	0.224
11571	0.495	1.363	0.224	0.509	1.434	0.235	0.489	1.329	0.508
11572	0.184	-0.625	0.409	0.196	-0.581	0.341	0.184	-0.625	0.097
11573	0.492	1.346	0.224	0.512	1.453	0.514	0.489	1.329	0.224
11574	0.508	1.447	0.224	0.462	1.133	0.235	0.503	1.413	0.224
11575	0.332	0.318	0.262	0.149	-0.882	0.235	0.050	-1.485	0.501
11576	0.429	0.942	0.097	0.515	1.472	0.501	0.500	1.397	0.262
11577	0.411	0.824	0.467	0.512	1.453	0.341	0.484	1.295	0.262
11578	0.429	0.942	0.328	0.512	1.453	0.149	0.484	1.295	0.450
11579	0.187	-0.608	0.262	0.275	-0.073	0.371	0.097	-1.181	0.185
11580	0.492	1.346	0.512	0.506	1.415	0.235	0.484	1.295	0.224
11581	0.224	-0.373	0.185	0.310	0.153	0.341	0.097	-1.181	0.224
11582	0.184	-0.625	0.501	0.278	-0.054	0.235	0.097	-1.181	0.512
11583	0.508	1.447	0.492	0.444	1.020	0.235	0.500	1.397	0.512
11584	0.492	1.346	0.050	0.421	0.869	0.491	0.445	1.043	0.328
11585	0.479	1.262	0.492	0.462	1.133	0.508	0.495	1.363	0.481
11586	0.226	-0.356	0.224	0.234	-0.336	0.491	0.097	-1.181	0.296

11587	0.142	-0.895	0.097	0.342	0.361	0.398	0.100	-1.164	0.296
11588	0.508	1.447	0.501	0.518	1.491	0.235	0.405	0.790	0.492
11589	0.142	-0.895	0.512	0.237	-0.317	0.501	0.226	-0.356	0.185
11590	0.187	-0.608	0.508	0.234	-0.336	0.444	0.184	-0.625	0.185
11591	0.508	1.447	0.450	0.497	1.359	0.491	0.489	1.329	0.492
11592	0.184	-0.625	0.501	0.345	0.379	0.149	0.097	-1.181	0.481
11593	0.226	-0.356	0.513	0.149	-0.882	0.491	0.187	-0.608	0.481
11594	0.263	-0.120	0.492	0.371	0.549	0.491	0.187	-0.608	0.185
11595	0.297	0.099	0.185	0.234	-0.336	0.491	0.097	-1.181	0.050
11596	0.295	0.082	0.185	0.345	0.379	0.508	0.097	-1.181	0.142
11597	0.358	0.487	0.512	0.237	-0.317	0.273	0.184	-0.625	0.142
11598	0.263	-0.120	0.513	0.237	-0.317	0.309	0.184	-0.625	0.050
11599	0.505	1.430	0.512	0.488	1.302	0.508	0.447	1.060	0.050
11600	0.505	1.430	0.481	0.500	1.378	0.273	0.482	1.279	0.450
11601	0.461	1.144	0.501	0.491	1.321	0.491	0.511	1.464	0.185
11602	0.492	1.346	0.481	0.395	0.700	0.273	0.476	1.245	0.185
11603	0.226	-0.356	0.512	0.234	-0.336	0.273	0.097	-1.181	0.142
11604	0.508	1.447	0.142	0.500	1.378	0.462	0.445	1.043	0.142
11605	0.358	0.487	0.224	0.442	1.001	0.309	0.097	-1.181	0.097
11606	0.471	1.211	0.450	0.494	1.340	0.193	0.463	1.161	0.097
11607	0.263	-0.120	0.467	0.149	-0.882	0.512	0.261	-0.137	0.431
11608	0.497	1.380	0.467	0.488	1.302	0.193	0.516	1.498	0.142
11609	0.497	1.380	0.467	0.488	1.302	0.444	0.516	1.498	0.501
11610	0.513	1.481	0.492	0.488	1.302	0.508	0.482	1.279	0.097
11611	0.479	1.262	0.224	0.509	1.434	0.273	0.461	1.144	0.097
11612	0.329	0.301	0.185	0.275	-0.073	0.273	0.297	0.099	0.508
11613	0.142	-0.895	0.492	0.307	0.135	0.371	0.097	-1.181	0.097
11614	0.463	1.161	0.224	0.509	1.434	0.491	0.511	1.464	0.097
11615	0.097	-1.181	0.358	0.272	-0.091	0.508	0.300	0.116	0.097
11616	0.476	1.245	0.224	0.488	1.302	0.508	0.503	1.413	0.097
11617	0.142	-0.895	0.296	0.272	-0.091	0.371	0.224	-0.373	0.512
11618	0.184	-0.625	0.142	0.278	-0.054	0.491	0.261	-0.137	0.185
11619	0.505	1.430	0.501	0.459	1.114	0.193	0.508	1.447	0.185
11620	0.184	-0.625	0.508	0.310	0.153	0.501	0.097	-1.181	0.512
11621	0.187	-0.608	0.467	0.193	-0.600	0.371	0.184	-0.625	0.142
11622	0.482	1.279	0.431	0.512	1.453	0.371	0.461	1.144	0.501
11623	0.263	-0.120	0.450	0.193	-0.600	0.309	0.097	-1.181	0.512
11624	0.450	1.076	0.481	0.442	1.001	0.273	0.492	1.346	0.097
11625	0.487	1.312	0.262	0.509	1.434	0.501	0.445	1.043	0.501
11626	0.184	-0.625	0.508	0.275	-0.073	0.193	0.097	-1.181	0.142
11627	0.142	-0.895	0.224	0.275	-0.073	0.371	0.224	-0.373	0.142
11628	0.184	-0.625	0.224	0.371	0.549	0.478	0.142	-0.895	0.501
11629	0.505	1.430	0.358	0.494	1.340	0.341	0.482	1.279	0.385
11630	0.503	1.413	0.358	0.509	1.434	0.309	0.482	1.279	0.501
11631	0.503	1.413	0.481	0.509	1.434	0.193	0.492	1.346	0.513
11632	0.329	0.301	0.481	0.374	0.568	0.341	0.097	-1.181	0.513
11633	0.479	1.262	0.224	0.491	1.321	0.341	0.358	0.487	0.481
11634	0.224	-0.373	0.328	0.193	-0.600	0.309	0.295	0.082	0.450
11635	0.513	1.481	0.142	0.500	1.378	0.512	0.511	1.464	0.328
11636	0.508	1.447	0.142	0.368	0.530	0.371	0.461	1.144	0.328
11637	0.508	1.447	0.185	0.368	0.530	0.273	0.445	1.043	0.481
11638	0.224	-0.373	0.501	0.310	0.153	0.478	0.000	?	0.481
11639	0.263	-0.120	0.450	0.275	-0.073	0.193	0.297	0.099	0.501
11640	0.508	1.447	0.492	0.500	1.378	0.341	0.447	1.060	0.508
11641	0.224	-0.373	0.508	0.193	-0.600	0.371	0.142	-0.895	0.450
11642	0.511	1.464	0.501	0.371	0.549	0.462	0.487	1.312	0.501
11643	0.387	0.672	0.501	0.477	1.227	0.478	0.511	1.464	0.501
11644	0.326	0.285	0.467	0.342	0.361	0.273	0.097	-1.181	0.262
11645	0.187	-0.608	0.296	0.310	0.153	0.371	0.295	0.082	0.262
11646	0.263	-0.120	0.431	0.193	-0.600	0.273	0.142	-0.895	0.512
11647	0.142	-0.895	0.512	0.342	0.361	0.273	0.226	-0.356	0.501

11648	0.142	−0.895	0.185	0.342	0.361	0.273	0.226	−0.356	0.501
11649	0.261	−0.137	0.501	0.313	0.172	0.501	0.050	−1.485	0.508
11650	0.474	1.228	0.508	0.518	1.491	0.341	0.495	1.363	0.481
11651	0.495	1.363	0.492	0.374	0.568	0.478	0.505	1.430	0.142
11652	0.187	−0.608	0.492	0.275	−0.073	0.398	0.261	−0.137	0.185
11653	0.511	1.464	0.185	0.480	1.246	0.309	0.405	0.790	0.512
11654	0.224	−0.373	0.185	0.193	−0.600	0.341	0.142	−0.895	0.097
11655	0.224	−0.373	0.224	0.345	0.379	0.444	0.097	−1.181	0.050
11656	0.326	0.285	0.467	0.374	0.568	0.462	0.263	−0.120	0.185
11657	0.466	1.178	0.328	0.465	1.152	0.309	0.503	1.413	0.185
11658	0.508	1.447	0.296	0.474	1.208	0.273	0.487	1.312	0.185
11659	0.184	−0.625	0.512	0.272	−0.091	0.501	0.097	−1.181	0.450
11660	0.263	−0.120	0.501	0.371	0.549	0.309	0.326	0.285	0.501
11661	0.187	−0.608	0.262	0.275	−0.073	0.273	0.224	−0.373	0.512
11662	0.142	−0.895	0.185	0.275	−0.073	0.149	0.184	−0.625	0.492
11663	0.295	0.082	0.358	0.272	−0.091	0.444	0.329	0.301	0.481
11664	0.511	1.464	0.097	0.497	1.359	0.193	0.497	1.380	0.467
11665	0.224	−0.373	0.097	0.339	0.342	0.193	0.097	−1.181	0.050
11666	0.511	1.464	0.431	0.480	1.246	0.462	0.479	1.262	0.508
11667	0.500	1.397	0.481	0.398	0.718	0.273	0.489	1.329	0.142
11668	0.295	0.082	0.481	0.313	0.172	0.273	0.261	−0.137	0.097
11669	0.358	0.487	0.481	0.342	0.361	0.341	0.297	0.099	0.050
11670	0.513	1.481	0.508	0.442	1.001	0.462	0.474	1.228	0.050
11671	0.382	0.638	0.467	0.462	1.133	0.462	0.511	1.464	0.508
11672	0.329	0.301	0.481	0.307	0.135	0.235	0.142	−0.895	0.512
11673	0.184	−0.625	0.481	0.272	−0.091	0.508	0.226	−0.356	0.097
11674	0.492	1.346	0.224	0.500	1.378	0.193	0.508	1.447	0.142
11675	0.226	−0.356	0.050	0.307	0.135	0.235	0.097	−1.181	0.050
11676	0.050	−1.485	0.492	0.278	−0.054	0.501	0.224	−0.373	0.185
11677	0.261	−0.137	0.492	0.149	−0.882	0.309	0.226	−0.356	0.142
11678	0.453	1.093	0.358	0.442	1.001	0.273	0.497	1.380	0.492
11679	0.142	−0.895	0.481	0.193	−0.600	0.508	0.266	−0.103	0.492
11680	0.142	−0.895	0.224	0.193	−0.600	0.422	0.261	−0.137	0.508
11681	0.511	1.464	0.224	0.459	1.114	0.422	0.447	1.060	0.450
11682	0.261	−0.137	0.467	0.275	−0.073	0.341	0.184	−0.625	0.508
11683	0.226	−0.356	0.501	0.275	−0.073	0.309	0.226	−0.356	0.450
11684	0.142	−0.895	0.224	0.345	0.379	0.193	0.226	−0.356	0.508
11685	0.513	1.481	0.358	0.459	1.114	0.193	0.511	1.464	0.185
11686	0.447	1.060	0.492	0.459	1.114	0.273	0.508	1.447	0.481
11687	0.184	−0.625	0.512	0.237	−0.317	0.149	0.332	0.318	0.481
11688	0.426	0.925	0.409	0.506	1.415	0.508	0.479	1.262	0.142
11689	0.097	−1.181	0.262	0.196	−0.581	0.149	0.297	0.099	0.512
11690	0.361	0.504	0.481	0.234	−0.336	0.149	0.297	0.099	0.450
11691	0.497	1.380	0.512	0.503	1.396	0.193	0.487	1.312	0.481
11692	0.295	0.082	0.328	0.310	0.153	0.193	0.187	−0.608	0.492
11693	0.297	0.099	0.492	0.272	−0.091	0.235	0.184	−0.625	0.097
11694	0.447	1.060	0.328	0.503	1.396	0.235	0.489	1.329	0.097
11695	0.511	1.464	0.328	0.418	0.850	0.462	0.479	1.262	0.097
11696	0.511	1.464	0.328	0.418	0.850	0.149	0.479	1.262	0.501
11697	0.097	−1.181	0.328	0.345	0.379	0.491	0.184	−0.625	0.142
11698	0.266	−0.103	0.501	0.307	0.135	0.235	0.050	−1.485	0.050
11699	0.263	−0.120	0.185	0.196	−0.581	0.235	0.142	−0.895	0.492
11700	0.263	−0.120	0.508	0.196	−0.581	0.501	0.142	−0.895	0.481
11701	0.261	−0.137	0.467	0.272	−0.091	0.235	0.050	−1.485	0.097
11702	0.329	0.301	0.262	0.149	−0.882	0.309	0.050	−1.485	0.142
11703	0.495	1.363	0.262	0.509	1.434	0.193	0.447	1.060	0.097
11704	0.361	0.504	0.142	0.149	−0.882	0.341	0.184	−0.625	0.097
11705	0.224	−0.373	0.142	0.149	−0.882	0.508	0.184	−0.625	0.142
11706	0.226	−0.356	0.224	0.193	−0.600	0.341	0.142	−0.895	0.467
11707	0.226	−0.356	0.481	0.193	−0.600	0.309	0.142	−0.895	0.481
11708	0.226	−0.356	0.508	0.234	−0.336	0.478	0.100	−1.164	0.481

11709	0.261	-0.137	0.050	0.237	-0.317	0.273	0.097	-1.181	0.508
11710	0.505	1.430	0.262	0.459	1.114	0.501	0.426	0.925	0.467
11711	0.261	-0.137	0.142	0.149	-0.882	0.398	0.142	-0.895	0.508
11712	0.500	1.397	0.262	0.491	1.321	0.371	0.495	1.363	0.481
11713	0.263	-0.120	0.262	0.234	-0.336	0.491	0.097	-1.181	0.492
11714	0.224	-0.373	0.481	0.237	-0.317	0.235	0.097	-1.181	0.050
11715	0.408	0.807	0.508	0.503	1.396	0.371	0.500	1.397	0.296
11716	0.224	-0.373	0.328	0.237	-0.317	0.478	0.097	-1.181	0.467
11717	0.224	-0.373	0.097	0.313	0.172	0.512	0.097	-1.181	0.409
11718	0.263	-0.120	0.224	0.193	-0.600	0.501	0.097	-1.181	0.142
11719	0.097	-1.181	0.450	0.342	0.361	0.422	0.097	-1.181	0.492
11720	0.463	1.161	0.481	0.509	1.434	0.512	0.505	1.430	0.142
11721	0.329	0.301	0.296	0.342	0.361	0.501	0.187	-0.608	0.142
11722	0.263	-0.120	0.431	0.310	0.153	0.508	0.187	-0.608	0.450
11723	0.508	1.447	0.467	0.477	1.227	0.235	0.513	1.481	0.481
11724	0.187	-0.608	0.508	0.272	-0.091	0.235	0.145	-0.878	0.050
11725	0.505	1.430	0.450	0.500	1.378	0.512	0.495	1.363	0.501
11726	0.495	1.363	0.467	0.401	0.737	0.512	0.511	1.464	0.481
11727	0.050	-1.485	0.385	0.374	0.568	0.478	0.097	-1.181	0.508
11728	0.489	1.329	0.385	0.494	1.340	0.514	0.495	1.363	0.501
11729	0.226	-0.356	0.296	0.234	-0.336	0.501	0.142	-0.895	0.385
11730	0.097	-1.181	0.512	0.374	0.568	0.462	0.142	-0.895	0.185
11731	0.497	1.380	0.467	0.477	1.227	0.462	0.497	1.380	0.481
11732	0.513	1.481	0.467	0.512	1.453	0.149	0.384	0.655	0.385
11733	0.505	1.430	0.467	0.494	1.340	0.149	0.500	1.397	0.385
11734	0.447	1.060	0.185	0.424	0.888	0.512	0.508	1.447	0.409
11735	0.497	1.380	0.358	0.512	1.453	0.478	0.508	1.447	0.385
11736	0.516	1.498	0.262	0.500	1.378	0.478	0.476	1.245	0.513
11737	0.489	1.329	0.142	0.503	1.396	0.478	0.447	1.060	0.185
11738	0.184	-0.625	0.512	0.234	-0.336	0.512	0.334	0.335	0.431
11739	0.184	-0.625	0.501	0.234	-0.336	0.193	0.334	0.335	0.512
11740	0.505	1.430	0.296	0.512	1.453	0.193	0.479	1.262	0.262
11741	0.508	1.447	0.512	0.512	1.453	0.422	0.479	1.262	0.262
11742	0.508	1.447	0.328	0.480	1.246	0.235	0.503	1.413	0.224
11743	0.479	1.262	0.050	0.512	1.453	0.235	0.508	1.447	0.224
11744	0.497	1.380	0.513	0.500	1.378	0.371	0.445	1.043	0.142
11745	0.482	1.279	0.224	0.462	1.133	0.309	0.497	1.380	0.508
11746	0.511	1.464	0.142	0.462	1.133	0.273	0.497	1.380	0.385
11747	0.145	-0.878	0.492	0.149	-0.882	0.478	0.263	-0.120	0.409
11748	0.226	-0.356	0.450	0.149	-0.882	0.398	0.263	-0.120	0.185
11749	0.447	1.060	0.328	0.512	1.453	0.514	0.505	1.430	0.224
11750	0.466	1.178	0.296	0.480	1.246	0.508	0.492	1.346	0.185
11751	0.466	1.178	0.296	0.480	1.246	0.491	0.492	1.346	0.097
11752	0.468	1.194	0.262	0.480	1.246	0.501	0.500	1.397	0.450
11753	0.492	1.346	0.450	0.512	1.453	0.235	0.474	1.228	0.467
11754	0.224	-0.373	0.328	0.193	-0.600	0.478	0.142	-0.895	0.142
11755	0.187	-0.608	0.508	0.193	-0.600	0.193	0.187	-0.608	0.296
11756	0.495	1.363	0.358	0.421	0.869	0.235	0.503	1.413	0.262
11757	0.224	-0.373	0.385	0.234	-0.336	0.102	0.097	-1.181	0.501
11758	0.361	0.504	0.224	0.234	-0.336	0.102	0.050	-1.485	0.409
11759	0.224	-0.373	0.450	0.371	0.549	0.478	0.187	-0.608	0.142
11760	0.295	0.082	0.501	0.307	0.135	0.508	0.184	-0.625	0.508
11761	0.142	-0.895	0.508	0.272	-0.091	0.235	0.187	-0.608	0.513
11762	0.500	1.397	0.481	0.477	1.227	0.341	0.447	1.060	0.450
11763	0.511	1.464	0.481	0.395	0.700	0.371	0.497	1.380	0.467
11764	0.468	1.194	0.185	0.512	1.453	0.273	0.505	1.430	0.492
11765	0.434	0.975	0.185	0.509	1.434	0.273	0.484	1.295	0.050
11766	0.447	1.060	0.431	0.491	1.321	0.444	0.476	1.245	0.142
11767	0.482	1.279	0.185	0.497	1.359	0.514	0.463	1.161	0.224
11768	0.263	-0.120	0.328	0.234	-0.336	0.508	0.050	-1.485	0.467
11769	0.511	1.464	0.508	0.474	1.208	0.462	0.500	1.397	0.512

11770	0.224	−0.373	0.431	0.196	−0.581	0.501	0.142	−0.895	0.501
11771	0.226	−0.356	0.385	0.237	−0.317	0.491	0.097	−1.181	0.501
11772	0.361	0.504	0.296	0.102	−1.184	0.478	0.050	−1.485	0.097
11773	0.361	0.504	0.450	0.102	−1.184	0.235	0.050	−1.485	0.224
11774	0.479	1.262	0.481	0.477	1.227	0.491	0.503	1.413	0.296
11775	0.479	1.262	0.296	0.506	1.415	0.478	0.505	1.430	0.224
11776	0.224	−0.373	0.358	0.234	−0.336	0.309	0.097	−1.181	0.492
11777	0.329	0.301	0.358	0.342	0.361	0.478	0.142	−0.895	0.481
11778	0.142	−0.895	0.358	0.371	0.549	0.508	0.050	−1.485	0.050
11779	0.142	−0.895	0.467	0.272	−0.091	0.512	0.184	−0.625	0.467
11780	0.184	−0.625	0.224	0.275	−0.073	0.501	0.142	−0.895	0.097
11781	0.500	1.397	0.224	0.439	0.982	0.309	0.489	1.329	0.097
11782	0.450	1.076	0.262	0.512	1.453	0.309	0.487	1.312	0.512
11783	0.495	1.363	0.185	0.509	1.434	0.309	0.497	1.380	0.358
11784	0.505	1.430	0.450	0.462	1.133	0.478	0.445	1.043	0.481
11785	0.503	1.413	0.450	0.503	1.396	0.149	0.503	1.413	0.512
11786	0.500	1.397	0.492	0.488	1.302	0.193	0.447	1.060	0.142
11787	0.468	1.194	0.467	0.477	1.227	0.478	0.508	1.447	0.097
11788	0.295	0.082	0.328	0.237	−0.317	0.512	0.184	−0.625	0.097
11789	0.429	0.942	0.262	0.491	1.321	0.193	0.482	1.279	0.508
11790	0.511	1.464	0.262	0.477	1.227	0.273	0.474	1.228	0.097
11791	0.187	−0.608	0.097	0.310	0.153	0.149	0.142	−0.895	0.481
11792	0.500	1.397	0.262	0.482	1.265	0.341	0.505	1.430	0.481
11793	0.505	1.430	0.142	0.509	1.434	0.309	0.450	1.076	0.185
11794	0.492	1.346	0.185	0.509	1.434	0.501	0.474	1.228	0.097
11795	0.489	1.329	0.492	0.506	1.415	0.491	0.484	1.295	0.450
11796	0.184	−0.625	0.262	0.313	0.172	0.478	0.097	−1.181	0.501
11797	0.184	−0.625	0.501	0.307	0.135	0.508	0.097	−1.181	0.492
11798	0.224	−0.373	0.097	0.307	0.135	0.444	0.097	−1.181	0.501
11799	0.463	1.161	0.512	0.474	1.208	0.501	0.500	1.397	0.492
11800	0.329	0.301	0.492	0.149	−0.882	0.478	0.142	−0.895	0.097
11801	0.295	0.082	0.492	0.193	−0.600	0.444	0.050	−1.485	0.185
11802	0.513	1.481	0.185	0.480	1.246	0.273	0.484	1.295	0.508
11803	0.503	1.413	0.185	0.509	1.434	0.193	0.476	1.245	0.142
11804	0.261	−0.137	0.224	0.193	−0.600	0.444	0.097	−1.181	0.450
11805	0.184	−0.625	0.262	0.272	−0.091	0.512	0.142	−0.895	0.513
11806	0.358	0.487	0.492	0.149	−0.882	0.309	0.097	−1.181	0.492
11807	0.097	−1.181	0.224	0.345	0.379	0.514	0.097	−1.181	0.508
11808	0.097	−1.181	0.501	0.310	0.153	0.309	0.142	−0.895	0.097
11809	0.429	0.942	0.501	0.497	1.359	0.309	0.463	1.161	0.501
11810	0.479	1.262	0.185	0.491	1.321	0.462	0.476	1.245	0.358
11811	0.482	1.279	0.328	0.477	1.227	0.501	0.476	1.245	0.142
11812	0.484	1.295	0.467	0.509	1.434	0.309	0.500	1.397	0.097
11813	0.508	1.447	0.492	0.447	1.039	0.149	0.461	1.144	0.097
11814	0.463	1.161	0.296	0.497	1.359	0.491	0.505	1.430	0.142
11815	0.482	1.279	0.262	0.474	1.208	0.491	0.476	1.245	0.481
11816	0.476	1.245	0.358	0.444	1.020	0.444	0.489	1.329	0.224
11817	0.226	−0.356	0.431	0.275	−0.073	0.273	0.050	−1.485	0.142
11818	0.050	−1.485	0.508	0.193	−0.600	0.491	0.295	0.082	0.185
11819	0.492	1.346	0.296	0.444	1.020	0.508	0.466	1.178	0.185
11820	0.492	1.346	0.296	0.512	1.453	0.309	0.408	0.807	0.501
11821	0.358	0.487	0.185	0.307	0.135	0.462	0.142	−0.895	0.467
11822	0.479	1.262	0.224	0.515	1.472	0.273	0.489	1.329	0.450
11823	0.226	−0.356	0.481	0.307	0.135	0.273	0.142	−0.895	0.501
11824	0.224	−0.373	0.262	0.313	0.172	0.273	0.142	−0.895	0.224
11825	0.466	1.178	0.296	0.468	1.170	0.273	0.445	1.043	0.050
11826	0.505	1.430	0.224	0.503	1.396	0.444	0.476	1.245	0.097
11827	0.226	−0.356	0.409	0.307	0.135	0.273	0.050	−1.485	0.262
11828	0.358	0.487	0.097	0.149	−0.882	0.512	0.000	?	0.142
11829	0.495	1.363	0.328	0.488	1.302	0.501	0.500	1.397	0.296
11830	0.513	1.481	0.185	0.488	1.302	0.102	0.474	1.228	0.185

11831	0.413	0.841	0.492	0.447	1.039	0.102	0.505	1.430	0.492
11832	0.263	-0.120	0.262	0.272	-0.091	0.235	0.000	?	0.296
11833	0.482	1.279	0.431	0.488	1.302	0.235	0.495	1.363	0.492
11834	0.505	1.430	0.409	0.512	1.453	0.273	0.384	0.655	0.224
11835	0.329	0.301	0.224	0.310	0.153	0.478	0.184	-0.625	0.481
11836	0.489	1.329	0.450	0.465	1.152	0.491	0.484	1.295	0.508
11837	0.329	0.301	0.385	0.272	-0.091	0.053	0.387	0.672	0.450
11838	0.329	0.301	0.492	0.272	-0.091	0.371	0.387	0.672	0.328
11839	0.329	0.301	0.512	0.272	-0.091	0.193	0.413	0.841	0.328
11840	0.329	0.301	0.142	0.272	-0.091	0.193	0.389	0.689	0.097
11841	0.503	1.413	0.467	0.442	1.001	0.193	0.508	1.447	0.097
11842	0.184	-0.625	0.185	0.272	-0.091	0.501	0.184	-0.625	0.492
11843	0.505	1.430	0.385	0.506	1.415	0.444	0.429	0.942	0.358
11844	0.466	1.178	0.513	0.500	1.378	0.149	0.511	1.464	0.492
11845	0.263	-0.120	0.513	0.102	-1.184	0.273	0.261	-0.137	0.492
11846	0.263	-0.120	0.512	0.102	-1.184	0.193	0.261	-0.137	0.185
11847	0.142	-0.895	0.097	0.234	-0.336	0.478	0.226	-0.356	0.097
11848	0.142	-0.895	0.262	0.234	-0.336	0.478	0.224	-0.373	0.513
11849	0.224	-0.373	0.328	0.278	-0.054	0.102	0.142	-0.895	0.450
11850	0.482	1.279	0.097	0.477	1.227	0.491	0.505	1.430	0.224
11851	0.508	1.447	0.050	0.497	1.359	0.508	0.384	0.655	0.224
11852	0.050	-1.485	0.358	0.053	-1.504	0.508	0.405	0.790	0.142
11853	0.261	-0.137	0.185	0.374	0.568	0.512	0.184	-0.625	0.467
11854	0.142	-0.895	0.513	0.193	-0.600	0.512	0.224	-0.373	0.481
11855	0.261	-0.137	0.262	0.193	-0.600	0.235	0.187	-0.608	0.097
11856	0.263	-0.120	0.513	0.193	-0.600	0.398	0.097	-1.181	0.097
11857	0.479	1.262	0.513	0.500	1.378	0.273	0.447	1.060	0.142
11858	0.508	1.447	0.097	0.439	0.982	0.491	0.463	1.161	0.142
11859	0.329	0.301	0.492	0.149	-0.882	0.491	0.142	-0.895	0.385
11860	0.097	-1.181	0.142	0.272	-0.091	0.491	0.295	0.082	0.097
11861	0.224	-0.373	0.142	0.193	-0.600	0.478	0.263	-0.120	0.185
11862	0.450	1.076	0.512	0.474	1.208	0.341	0.500	1.397	0.262
11863	0.482	1.279	0.508	0.477	1.227	0.149	0.405	0.790	0.142
11864	0.297	0.099	0.512	0.102	-1.184	0.149	0.142	-0.895	0.224
11865	0.434	0.975	0.262	0.491	1.321	0.235	0.505	1.430	0.097
11866	0.468	1.194	0.262	0.503	1.396	0.398	0.508	1.447	0.224
11867	0.511	1.464	0.501	0.503	1.396	0.462	0.445	1.043	0.481
11868	0.450	1.076	0.481	0.512	1.453	0.309	0.466	1.178	0.097
11869	0.468	1.194	0.409	0.512	1.453	0.508	0.492	1.346	0.508
11870	0.384	0.655	0.409	0.237	-0.317	0.235	0.050	-1.485	0.513
11871	0.384	0.655	0.501	0.401	0.737	0.398	0.142	-0.895	0.185
11872	0.297	0.099	0.501	0.272	-0.091	0.444	0.224	-0.373	0.481
11873	0.511	1.464	0.481	0.488	1.302	0.341	0.463	1.161	0.142
11874	0.466	1.178	0.142	0.491	1.321	0.235	0.505	1.430	0.467
11875	0.466	1.178	0.296	0.491	1.321	0.501	0.497	1.380	0.501
11876	0.463	1.161	0.508	0.477	1.227	0.478	0.497	1.380	0.262
11877	0.184	-0.625	0.224	0.342	0.361	0.235	0.097	-1.181	0.467
11878	0.361	0.504	0.262	0.152	-0.863	0.149	0.224	-0.373	0.097
11879	0.263	-0.120	0.508	0.149	-0.882	0.149	0.300	0.116	0.142
11880	0.142	-0.895	0.409	0.240	-0.299	0.273	0.224	-0.373	0.450
11881	0.513	1.481	0.262	0.395	0.700	0.422	0.487	1.312	0.450
11882	0.503	1.413	0.185	0.465	1.152	0.193	0.482	1.279	0.481
11883	0.297	0.099	0.224	0.310	0.153	0.462	0.050	-1.485	0.185
11884	0.511	1.464	0.358	0.506	1.415	0.491	0.461	1.144	0.185
11885	0.326	0.285	0.296	0.234	-0.336	0.149	0.100	-1.164	0.142
11886	0.050	-1.485	0.296	0.401	0.737	0.235	0.097	-1.181	0.185
11887	0.513	1.481	0.185	0.442	1.001	0.512	0.503	1.413	0.262
11888	0.224	-0.373	0.409	0.345	0.379	0.444	0.000	?	0.512
11889	0.142	-0.895	0.481	0.234	-0.336	0.508	0.358	0.487	0.185
11890	0.487	1.312	0.501	0.503	1.396	0.512	0.476	1.245	0.450
11891	0.445	1.043	0.512	0.480	1.246	0.491	0.508	1.447	0.358

11892	0.329	0.301	0.508	0.237	-0.317	0.273	0.142	-0.895	0.481
11893	0.297	0.099	0.431	0.149	-0.882	0.273	0.097	-1.181	0.481
11894	0.297	0.099	0.467	0.149	-0.882	0.491	0.097	-1.181	0.185
11895	0.263	-0.120	0.467	0.275	-0.073	0.309	0.000	?	0.358
11896	0.447	1.060	0.512	0.427	0.907	0.053	0.505	1.430	0.142
11897	0.332	0.318	0.185	0.193	-0.600	0.371	0.097	-1.181	0.142
11898	0.508	1.447	0.224	0.459	1.114	0.501	0.476	1.245	0.450
11899	0.361	0.504	0.431	0.485	1.283	0.514	0.474	1.228	0.450
11900	0.384	0.655	0.431	0.152	-0.863	0.273	0.184	-0.625	0.431
11901	0.226	-0.356	0.481	0.234	-0.336	0.512	0.097	-1.181	0.262
11902	0.447	1.060	0.512	0.518	1.491	0.491	0.450	1.076	0.224
11903	0.503	1.413	0.481	0.444	1.020	0.102	0.503	1.413	0.450
11904	0.505	1.430	0.512	0.503	1.396	0.309	0.489	1.329	0.512
11905	0.479	1.262	0.431	0.515	1.472	0.309	0.497	1.380	0.431
11906	0.482	1.279	0.513	0.488	1.302	0.309	0.484	1.295	0.450
11907	0.184	-0.625	0.513	0.272	-0.091	0.462	0.097	-1.181	0.481
11908	0.184	-0.625	0.385	0.272	-0.091	0.102	0.184	-0.625	0.481
11909	0.429	0.942	0.328	0.488	1.302	0.193	0.505	1.430	0.508
11910	0.187	-0.608	0.467	0.310	0.153	0.235	0.142	-0.895	0.097
11911	0.332	0.318	0.467	0.053	-1.504	0.309	0.450	1.076	0.050
11912	0.511	1.464	0.431	0.368	0.530	0.501	0.511	1.464	0.492
11913	0.429	0.942	0.431	0.500	1.378	0.491	0.487	1.312	0.097
11914	0.384	0.655	0.481	0.512	1.453	0.508	0.505	1.430	0.142
11915	0.295	0.082	0.481	0.272	-0.091	0.422	0.097	-1.181	0.481
11916	0.450	1.076	0.262	0.512	1.453	0.341	0.495	1.363	0.450
11917	0.484	1.295	0.512	0.491	1.321	0.235	0.358	0.487	0.050
11918	0.295	0.082	0.262	0.102	-1.184	0.193	0.142	-0.895	0.097
11919	0.358	0.487	0.142	0.307	0.135	0.235	0.097	-1.181	0.142
11920	0.358	0.487	0.142	0.307	0.135	0.371	0.097	-1.181	0.467
11921	0.358	0.487	0.262	0.307	0.135	0.193	0.142	-0.895	0.097
11922	0.463	1.161	0.262	0.462	1.133	0.193	0.479	1.262	0.097
11923	0.224	-0.373	0.508	0.105	-1.165	0.422	0.224	-0.373	0.185
11924	0.224	-0.373	0.501	0.193	-0.600	0.149	0.142	-0.895	0.050
11925	0.263	-0.120	0.262	0.234	-0.336	0.491	0.184	-0.625	0.501
11926	0.187	-0.608	0.508	0.307	0.135	0.235	0.184	-0.625	0.492
11927	0.445	1.043	0.224	0.500	1.378	0.512	0.497	1.380	0.501
11928	0.447	1.060	0.481	0.491	1.321	0.478	0.463	1.161	0.508
11929	0.489	1.329	0.450	0.509	1.434	0.512	0.447	1.060	0.481
11930	0.463	1.161	0.492	0.421	0.869	0.149	0.500	1.397	0.508
11931	0.334	0.335	0.224	0.342	0.361	0.149	0.224	-0.373	0.501
11932	0.263	-0.120	0.492	0.237	-0.317	0.309	0.050	-1.485	0.501
11933	0.263	-0.120	0.481	0.193	-0.600	0.309	0.097	-1.181	0.224
11934	0.097	-1.181	0.481	0.234	-0.336	0.462	0.261	-0.137	0.185
11935	0.263	-0.120	0.224	0.374	0.568	0.102	0.142	-0.895	0.501
11936	0.142	-0.895	0.224	0.196	-0.581	0.422	0.297	0.099	0.501
11937	0.184	-0.625	0.492	0.193	-0.600	0.422	0.184	-0.625	0.501
11938	0.495	1.363	0.481	0.421	0.869	0.193	0.492	1.346	0.508
11939	0.263	-0.120	0.296	0.149	-0.882	0.102	0.300	0.116	0.501
11940	0.492	1.346	0.296	0.497	1.359	0.478	0.487	1.312	0.481
11941	0.097	-1.181	0.409	0.240	-0.299	0.491	0.224	-0.373	0.492
11942	0.505	1.430	0.262	0.509	1.434	0.235	0.484	1.295	0.501
11943	0.487	1.312	0.142	0.482	1.265	0.149	0.500	1.397	0.501
11944	0.492	1.346	0.467	0.509	1.434	0.371	0.453	1.093	0.450
11945	0.184	-0.625	0.224	0.149	-0.882	0.508	0.332	0.318	0.185
11946	0.184	-0.625	0.508	0.149	-0.882	0.514	0.332	0.318	0.512
11947	0.224	-0.373	0.508	0.313	0.172	0.193	0.097	-1.181	0.501
11948	0.261	-0.137	0.508	0.313	0.172	0.193	0.097	-1.181	0.492
11949	0.492	1.346	0.224	0.465	1.152	0.341	0.492	1.346	0.450
11950	0.224	-0.373	0.431	0.102	-1.184	0.341	0.358	0.487	0.467
11951	0.497	1.380	0.450	0.421	0.869	0.508	0.492	1.346	0.450
11952	0.497	1.380	0.450	0.421	0.869	0.273	0.492	1.346	0.097

11953	0.187	-0.608	0.492	0.196	-0.581	0.149	0.184	-0.625	0.450
11954	0.332	0.318	0.512	0.102	-1.184	0.273	0.097	-1.181	0.262
11955	0.463	1.161	0.224	0.471	1.189	0.371	0.513	1.481	0.142
11956	0.492	1.346	0.409	0.488	1.302	0.273	0.447	1.060	0.142
11957	0.295	0.082	0.492	0.234	-0.336	0.341	0.226	-0.356	0.050
11958	0.261	-0.137	0.467	0.149	-0.882	0.235	0.226	-0.356	0.050
11959	0.355	0.470	0.185	0.371	0.549	0.508	0.142	-0.895	0.467
11960	0.432	0.958	0.492	0.506	1.415	0.193	0.466	1.178	0.501
11961	0.503	1.413	0.409	0.515	1.472	0.512	0.476	1.245	0.097
11962	0.300	0.116	0.224	0.193	-0.600	0.462	0.097	-1.181	0.501
11963	0.300	0.116	0.358	0.193	-0.600	0.235	0.097	-1.181	0.185
11964	0.184	-0.625	0.358	0.342	0.361	0.514	0.142	-0.895	0.467
11965	0.224	-0.373	0.358	0.342	0.361	0.102	0.142	-0.895	0.492
11966	0.479	1.262	0.492	0.503	1.396	0.491	0.382	0.638	0.501
11967	0.261	-0.137	0.492	0.278	-0.054	0.478	0.097	-1.181	0.296
11968	0.300	0.116	0.450	0.149	-0.882	0.309	0.184	-0.625	0.508
11969	0.224	-0.373	0.262	0.275	-0.073	0.462	0.261	-0.137	0.508
11970	0.408	0.807	0.385	0.374	0.568	0.273	0.142	-0.895	0.481
11971	0.097	-1.181	0.050	0.278	-0.054	0.371	0.224	-0.373	0.185
11972	0.326	0.285	0.467	0.342	0.361	0.501	0.097	-1.181	0.328
11973	0.184	-0.625	0.224	0.237	-0.317	0.501	0.224	-0.373	0.481
11974	0.495	1.363	0.513	0.506	1.415	0.478	0.482	1.279	0.492
11975	0.261	-0.137	0.185	0.193	-0.600	0.273	0.097	-1.181	0.097
11976	0.426	0.925	0.508	0.509	1.434	0.193	0.505	1.430	0.513
11977	0.405	0.790	0.224	0.456	1.095	0.235	0.513	1.481	0.185
11978	0.226	-0.356	0.262	0.234	-0.336	0.309	0.184	-0.625	0.224
11979	0.450	1.076	0.492	0.518	1.491	0.235	0.482	1.279	0.512
11980	0.387	0.672	0.508	0.102	-1.184	0.478	0.142	-0.895	0.050
11981	0.492	1.346	0.185	0.494	1.340	0.193	0.468	1.194	0.512
11982	0.511	1.464	0.328	0.482	1.265	0.512	0.503	1.413	0.512
11983	0.142	-0.895	0.296	0.310	0.153	0.053	0.261	-0.137	0.512
11984	0.463	1.161	0.481	0.462	1.133	0.508	0.466	1.178	0.224
11985	0.187	-0.608	0.262	0.272	-0.091	0.501	0.097	-1.181	0.501
11986	0.389	0.689	0.224	0.371	0.549	0.273	0.142	-0.895	0.501
11987	0.508	1.447	0.296	0.497	1.359	0.508	0.453	1.093	0.501
11988	0.508	1.447	0.142	0.497	1.359	0.273	0.453	1.093	0.512
11989	0.513	1.481	0.224	0.477	1.227	0.273	0.476	1.245	0.481
11990	0.097	-1.181	0.262	0.275	-0.073	0.491	0.184	-0.625	0.142
11991	0.261	-0.137	0.409	0.193	-0.600	0.491	0.184	-0.625	0.492
11992	0.326	0.285	0.185	0.234	-0.336	0.508	0.142	-0.895	0.481
11993	0.097	-1.181	0.358	0.313	0.172	0.193	0.184	-0.625	0.501
11994	0.050	-1.485	0.492	0.234	-0.336	0.193	0.263	-0.120	0.097
11995	0.355	0.470	0.224	0.480	1.246	0.512	0.508	1.447	0.467
11996	0.184	-0.625	0.385	0.193	-0.600	0.422	0.187	-0.608	0.508
11997	0.513	1.481	0.328	0.509	1.434	0.491	0.447	1.060	0.050
11998	0.266	-0.103	0.431	0.053	-1.504	0.462	0.358	0.487	0.224
11999	0.508	1.447	0.142	0.506	1.415	0.478	0.479	1.262	0.224
12000	0.508	1.447	0.481	0.500	1.378	0.462	0.479	1.262	0.224
12001	0.097	-1.181	0.142	0.272	-0.091	0.478	0.187	-0.608	0.492
12002	0.492	1.346	0.492	0.503	1.396	0.341	0.355	0.470	0.467
12003	0.142	-0.895	0.224	0.275	-0.073	0.341	0.142	-0.895	0.492
12004	0.142	-0.895	0.481	0.275	-0.073	0.462	0.142	-0.895	0.296
12005	0.511	1.464	0.492	0.491	1.321	0.371	0.447	1.060	0.296
12006	0.505	1.430	0.492	0.491	1.321	0.341	0.445	1.043	0.481
12007	0.511	1.464	0.508	0.509	1.434	0.422	0.429	0.942	0.097
12008	0.263	-0.120	0.512	0.196	-0.581	0.501	0.261	-0.137	0.431
12009	0.261	-0.137	0.224	0.193	-0.600	0.422	0.224	-0.373	0.481
12010	0.503	1.413	0.296	0.509	1.434	0.309	0.445	1.043	0.328
12011	0.484	1.295	0.328	0.424	0.888	0.193	0.511	1.464	0.097
12012	0.411	0.824	0.296	0.488	1.302	0.501	0.432	0.958	0.512
12013	0.411	0.824	0.328	0.459	1.114	0.193	0.450	1.076	0.481

12014	0.503	1.413	0.328	0.477	1.227	0.193	0.476	1.245	0.142
12015	0.500	1.397	0.185	0.459	1.114	0.193	0.476	1.245	0.185
12016	0.484	1.295	0.262	0.482	1.265	0.193	0.508	1.447	0.185
12017	0.142	−0.895	0.492	0.345	0.379	0.514	0.097	−1.181	0.492
12018	0.297	0.099	0.296	0.345	0.379	0.501	0.050	−1.485	0.142
12019	0.511	1.464	0.262	0.462	1.133	0.422	0.489	1.329	0.224
12020	0.224	−0.373	0.358	0.371	0.549	0.491	0.097	−1.181	0.262
12021	0.263	−0.120	0.185	0.342	0.361	0.514	0.142	−0.895	0.262
12022	0.508	1.447	0.450	0.421	0.869	0.491	0.476	1.245	0.097
12023	0.411	0.824	0.262	0.497	1.359	0.501	0.445	1.043	0.508
12024	0.263	−0.120	0.508	0.424	0.888	0.444	0.050	−1.485	0.097
12025	0.187	−0.608	0.492	0.310	0.153	0.273	0.097	−1.181	0.262
12026	0.226	−0.356	0.492	0.193	−0.600	0.309	0.142	−0.895	0.501
12027	0.355	0.470	0.296	0.503	1.396	0.462	0.461	1.144	0.185
12028	0.295	0.082	0.492	0.196	−0.581	0.491	0.097	−1.181	0.481
12029	0.295	0.082	0.492	0.196	−0.581	0.422	0.097	−1.181	0.142
12030	0.187	−0.608	0.142	0.193	−0.600	0.462	0.184	−0.625	0.508
12031	0.413	0.841	0.358	0.193	−0.600	0.422	0.050	−1.485	0.142
12032	0.482	1.279	0.358	0.515	1.472	0.478	0.495	1.363	0.508
12033	0.503	1.413	0.431	0.500	1.378	0.514	0.489	1.329	0.142
12034	0.511	1.464	0.224	0.421	0.869	0.501	0.500	1.397	0.450
12035	0.505	1.430	0.262	0.491	1.321	0.501	0.500	1.397	0.142
12036	0.426	0.925	0.508	0.509	1.434	0.508	0.479	1.262	0.467
12037	0.466	1.178	0.358	0.494	1.340	0.053	0.505	1.430	0.492
12038	0.463	1.161	0.492	0.503	1.396	0.444	0.495	1.363	0.492
12039	0.511	1.464	0.262	0.444	1.020	0.398	0.492	1.346	0.513
12040	0.187	−0.608	0.513	0.272	−0.091	0.501	0.224	−0.373	0.512
12041	0.226	−0.356	0.513	0.310	0.153	0.512	0.184	−0.625	0.097
12042	0.432	0.958	0.508	0.462	1.133	0.462	0.495	1.363	0.185
12043	0.432	0.958	0.508	0.491	1.321	0.462	0.495	1.363	0.185
12044	0.484	1.295	0.296	0.421	0.869	0.273	0.495	1.363	0.050
12045	0.516	1.498	0.262	0.459	1.114	0.478	0.500	1.397	0.050
12046	0.484	1.295	0.262	0.421	0.869	0.193	0.495	1.363	0.296
12047	0.516	1.498	0.296	0.477	1.227	0.309	0.474	1.228	0.050
12048	0.432	0.958	0.481	0.512	1.453	0.309	0.484	1.295	0.467
12049	0.513	1.481	0.481	0.497	1.359	0.309	0.495	1.363	0.142
12050	0.513	1.481	0.431	0.497	1.359	0.309	0.495	1.363	0.185
12051	0.384	0.655	0.409	0.509	1.434	0.512	0.447	1.060	0.185
12052	0.332	0.318	0.409	0.053	−1.504	0.508	0.184	−0.625	0.097
12053	0.468	1.194	0.492	0.444	1.020	0.235	0.503	1.413	0.501
12054	0.468	1.194	0.296	0.398	0.718	0.491	0.492	1.346	0.097
12055	0.432	0.958	0.296	0.500	1.378	0.149	0.487	1.312	0.492
12056	0.434	0.975	0.467	0.509	1.434	0.514	0.445	1.043	0.431
12057	0.476	1.245	0.450	0.462	1.133	0.491	0.463	1.161	0.467
12058	0.482	1.279	0.296	0.465	1.152	0.512	0.450	1.076	0.431
12059	0.263	−0.120	0.142	0.272	−0.091	0.193	0.097	−1.181	0.512
12060	0.511	1.464	0.328	0.482	1.265	0.422	0.447	1.060	0.512
12061	0.263	−0.120	0.513	0.193	−0.600	0.508	0.263	−0.120	0.328
12062	0.142	−0.895	0.492	0.310	0.153	0.508	0.142	−0.895	0.142
12063	0.142	−0.895	0.481	0.310	0.153	0.149	0.142	−0.895	0.142
12064	0.263	−0.120	0.508	0.310	0.153	0.149	0.050	−1.485	0.431
12065	0.263	−0.120	0.385	0.310	0.153	0.501	0.050	−1.485	0.185
12066	0.505	1.430	0.385	0.512	1.453	0.501	0.461	1.144	0.142
12067	0.503	1.413	0.467	0.506	1.415	0.371	0.492	1.346	0.481
12068	0.261	−0.137	0.450	0.234	−0.336	0.193	0.097	−1.181	0.262
12069	0.508	1.447	0.224	0.488	1.302	0.422	0.500	1.397	0.450
12070	0.229	−0.339	0.501	0.149	−0.882	0.341	0.184	−0.625	0.358
12071	0.479	1.262	0.512	0.515	1.472	0.422	0.463	1.161	0.467
12072	0.450	1.076	0.512	0.488	1.302	0.491	0.487	1.312	0.467
12073	0.495	1.363	0.467	0.512	1.453	0.273	0.495	1.363	0.450
12074	0.226	−0.356	0.492	0.196	−0.581	0.478	0.297	0.099	0.450

12075	0.497	1.380	0.185	0.421	0.869	0.478	0.497	1.380	0.050
12076	0.479	1.262	0.185	0.506	1.415	0.478	0.503	1.413	0.050
12077	0.479	1.262	0.481	0.506	1.415	0.193	0.476	1.245	0.262
12078	0.229	−0.339	0.296	0.149	−0.882	0.491	0.184	−0.625	0.262
12079	0.226	−0.356	0.142	0.149	−0.882	0.491	0.329	0.301	0.501
12080	0.489	1.329	0.501	0.500	1.378	0.491	0.476	1.245	0.492
12081	0.479	1.262	0.050	0.500	1.378	0.491	0.487	1.312	0.492
12082	0.295	0.082	0.050	0.374	0.568	0.501	0.000	?	0.508
12083	0.297	0.099	0.296	0.196	−0.581	0.273	0.097	−1.181	0.508
12084	0.408	0.807	0.508	0.421	0.869	0.501	0.505	1.430	0.492
12085	0.261	−0.137	0.142	0.342	0.361	0.514	0.184	−0.625	0.050
12086	0.145	−0.878	0.508	0.421	0.869	0.508	0.226	−0.356	0.481
12087	0.466	1.178	0.262	0.491	1.321	0.273	0.508	1.447	0.481
12088	0.224	−0.373	0.262	0.272	−0.091	0.462	0.050	−1.485	0.097
12089	0.508	1.447	0.262	0.477	1.227	0.462	0.503	1.413	0.097
12090	0.508	1.447	0.492	0.477	1.227	0.273	0.503	1.413	0.185
12091	0.508	1.447	0.097	0.477	1.227	0.309	0.503	1.413	0.450
12092	0.226	−0.356	0.409	0.193	−0.600	0.309	0.226	−0.356	0.512
12093	0.429	0.942	0.513	0.491	1.321	0.309	0.495	1.363	0.262
12094	0.450	1.076	0.328	0.491	1.321	0.422	0.495	1.363	0.492
12095	0.450	1.076	0.467	0.491	1.321	0.501	0.495	1.363	0.296
12096	0.495	1.363	0.467	0.488	1.302	0.501	0.505	1.430	0.385
12097	0.511	1.464	0.467	0.500	1.378	0.193	0.476	1.245	0.512
12098	0.224	−0.373	0.512	0.272	−0.091	0.193	0.142	−0.895	0.467
12099	0.411	0.824	0.262	0.506	1.415	0.508	0.495	1.363	0.097
12100	0.492	1.346	0.262	0.512	1.453	0.309	0.476	1.245	0.492
12101	0.466	1.178	0.450	0.506	1.415	0.514	0.495	1.363	0.512
12102	0.184	−0.625	0.142	0.275	−0.073	0.341	0.097	−1.181	0.512
12103	0.489	1.329	0.296	0.465	1.152	0.501	0.461	1.144	0.492
12104	0.411	0.824	0.358	0.456	1.095	0.273	0.503	1.413	0.431
12105	0.224	−0.373	0.467	0.275	−0.073	0.193	0.050	−1.485	0.224
12106	0.358	0.487	0.142	0.310	0.153	0.501	0.224	−0.373	0.262
12107	0.358	0.487	0.450	0.310	0.153	0.508	0.224	−0.373	0.508
12108	0.358	0.487	0.097	0.310	0.153	0.273	0.224	−0.373	0.142
12109	0.495	1.363	0.492	0.421	0.869	0.102	0.487	1.312	0.262
12110	0.492	1.346	0.467	0.494	1.340	0.053	0.463	1.161	0.467
12111	0.450	1.076	0.224	0.500	1.378	0.491	0.484	1.295	0.296
12112	0.261	−0.137	0.262	0.000	?	0.149	0.295	0.082	0.296
12113	0.384	0.655	0.450	0.193	−0.600	0.193	0.000	?	0.142
12114	0.050	−1.485	0.185	0.193	−0.600	0.341	0.295	0.082	0.481
12115	0.461	1.144	0.224	0.506	1.415	0.193	0.482	1.279	0.358
12116	0.224	−0.373	0.481	0.313	0.172	0.053	0.097	−1.181	0.481
12117	0.505	1.430	0.224	0.512	1.453	0.193	0.429	0.942	0.097
12118	0.184	−0.625	0.185	0.342	0.361	0.501	0.000	?	0.097
12119	0.513	1.481	0.481	0.500	1.378	0.273	0.476	1.245	0.097
12120	0.224	−0.373	0.097	0.275	−0.073	0.235	0.332	0.318	0.508
12121	0.261	−0.137	0.224	0.196	−0.581	0.491	0.097	−1.181	0.224
12122	0.489	1.329	0.512	0.500	1.378	0.235	0.508	1.447	0.262
12123	0.503	1.413	0.262	0.509	1.434	0.514	0.476	1.245	0.501
12124	0.184	−0.625	0.097	0.275	−0.073	0.149	0.145	−0.878	0.262
12125	0.332	0.318	0.185	0.102	−1.184	0.462	0.184	−0.625	0.508
12126	0.300	0.116	0.450	0.053	−1.504	0.273	0.184	−0.625	0.512
12127	0.476	1.245	0.185	0.488	1.302	0.398	0.489	1.329	0.513
12128	0.261	−0.137	0.492	0.149	−0.882	0.273	0.142	−0.895	0.431
12129	0.224	−0.373	0.450	0.193	−0.600	0.501	0.226	−0.356	0.385
12130	0.300	0.116	0.142	0.339	0.342	0.341	0.000	?	0.185
12131	0.142	−0.895	0.513	0.193	−0.600	0.478	0.263	−0.120	0.508
12132	0.229	−0.339	0.224	0.053	−1.504	0.444	0.261	−0.137	0.262
12133	0.261	−0.137	0.142	0.196	−0.581	0.444	0.097	−1.181	0.328
12134	0.411	0.824	0.450	0.497	1.359	0.501	0.500	1.397	0.328
12135	0.187	−0.608	0.512	0.272	−0.091	0.422	0.097	−1.181	0.467

12136	0.358	0.487	0.450	0.237	-0.317	0.273	0.261	-0.137	0.262
12137	0.492	1.346	0.508	0.491	1.321	0.193	0.500	1.397	0.508
12138	0.226	-0.356	0.512	0.237	-0.317	0.193	0.184	-0.625	0.142
12139	0.384	0.655	0.328	0.520	1.509	0.235	0.474	1.228	0.501
12140	0.332	0.318	0.492	0.149	-0.882	0.235	0.142	-0.895	0.492
12141	0.432	0.958	0.512	0.462	1.133	0.235	0.503	1.413	0.097
12142	0.142	-0.895	0.185	0.275	-0.073	0.149	0.142	-0.895	0.224
12143	0.482	1.279	0.508	0.398	0.718	0.273	0.503	1.413	0.513
12144	0.142	-0.895	0.097	0.275	-0.073	0.478	0.142	-0.895	0.097
12145	0.489	1.329	0.185	0.500	1.378	0.149	0.447	1.060	0.224
12146	0.224	-0.373	0.508	0.348	0.398	0.149	0.142	-0.895	0.508
12147	0.479	1.262	0.185	0.477	1.227	0.309	0.463	1.161	0.358
12148	0.495	1.363	0.501	0.439	0.982	0.341	0.487	1.312	0.262
12149	0.495	1.363	0.262	0.439	0.982	0.491	0.487	1.312	0.492
12150	0.511	1.464	0.224	0.497	1.359	0.193	0.513	1.481	0.262
12151	0.505	1.430	0.513	0.421	0.869	0.462	0.508	1.447	0.224
12152	0.224	-0.373	0.409	0.278	-0.054	0.501	0.097	-1.181	0.481
12153	0.297	0.099	0.409	0.196	-0.581	0.512	0.184	-0.625	0.185
12154	0.332	0.318	0.481	0.196	-0.581	0.149	0.184	-0.625	0.358
12155	0.297	0.099	0.513	0.234	-0.336	0.273	0.000	?	0.296
12156	0.329	0.301	0.512	0.234	-0.336	0.309	0.050	-1.485	0.481
12157	0.329	0.301	0.142	0.234	-0.336	0.102	0.050	-1.485	0.262
12158	0.184	-0.625	0.142	0.149	-0.882	0.149	0.297	0.099	0.450
12159	0.263	-0.120	0.142	0.275	-0.073	0.149	0.050	-1.485	0.513
12160	0.492	1.346	0.492	0.477	1.227	0.478	0.461	1.144	0.262
12161	0.295	0.082	0.492	0.149	-0.882	0.235	0.142	-0.895	0.450
12162	0.263	-0.120	0.508	0.152	-0.863	0.235	0.184	-0.625	0.097
12163	0.355	0.470	0.185	0.310	0.153	0.491	0.184	-0.625	0.185
12164	0.184	-0.625	0.262	0.339	0.342	0.341	0.097	-1.181	0.467
12165	0.450	1.076	0.508	0.491	1.321	0.512	0.495	1.363	0.467
12166	0.263	-0.120	0.097	0.196	-0.581	0.193	0.097	-1.181	0.467
12167	0.511	1.464	0.409	0.459	1.114	0.371	0.484	1.295	0.508
12168	0.489	1.329	0.501	0.503	1.396	0.444	0.426	0.925	0.467
12169	0.492	1.346	0.481	0.506	1.415	0.508	0.463	1.161	0.328
12170	0.300	0.116	0.185	0.149	-0.882	0.508	0.432	0.958	0.501
12171	0.495	1.363	0.224	0.272	-0.091	0.273	0.513	1.481	0.492
12172	0.495	1.363	0.508	0.307	0.135	0.273	0.513	1.481	0.142
12173	0.142	-0.895	0.296	0.102	-1.184	0.149	0.329	0.301	0.431
12174	0.358	0.487	0.296	0.149	-0.882	0.149	0.142	-0.895	0.385
12175	0.358	0.487	0.142	0.149	-0.882	0.491	0.142	-0.895	0.328
12176	0.434	0.975	0.224	0.477	1.227	0.478	0.426	0.925	0.512
12177	0.226	-0.356	0.409	0.237	-0.317	0.478	0.184	-0.625	0.262
12178	0.263	-0.120	0.409	0.234	-0.336	0.462	0.142	-0.895	0.492
12179	0.508	1.447	0.409	0.488	1.302	0.444	0.479	1.262	0.385
12180	0.358	0.487	0.409	0.339	0.342	0.478	0.263	-0.120	0.385
12181	0.489	1.329	0.492	0.512	1.453	0.235	0.450	1.076	0.501
12182	0.263	-0.120	0.492	0.193	-0.600	0.309	0.358	0.487	0.409
12183	0.511	1.464	0.185	0.368	0.530	0.514	0.466	1.178	0.409
12184	0.511	1.464	0.224	0.442	1.001	0.508	0.466	1.178	0.467
12185	0.503	1.413	0.097	0.506	1.415	0.273	0.447	1.060	0.431
12186	0.503	1.413	0.097	0.506	1.415	0.309	0.447	1.060	0.409
12187	0.297	0.099	0.142	0.275	-0.073	0.102	0.050	-1.485	0.050
12188	0.263	-0.120	0.450	0.275	-0.073	0.491	0.050	-1.485	0.050
12189	0.261	-0.137	0.296	0.152	-0.863	0.512	0.263	-0.120	0.185
12190	0.297	0.099	0.185	0.152	-0.863	0.514	0.263	-0.120	0.501
12191	0.479	1.262	0.185	0.488	1.302	0.398	0.497	1.380	0.508
12192	0.482	1.279	0.501	0.474	1.208	0.149	0.487	1.312	0.467
12193	0.434	0.975	0.501	0.474	1.208	0.235	0.489	1.329	0.185
12194	0.411	0.824	0.508	0.459	1.114	0.514	0.503	1.413	0.097
12195	0.411	0.824	0.262	0.442	1.001	0.512	0.503	1.413	0.450
12196	0.487	1.312	0.262	0.477	1.227	0.235	0.492	1.346	0.328

12197	0.295	0.082	0.262	0.234	-0.336	0.508	0.000	?	0.508
12198	0.295	0.082	0.262	0.307	0.135	0.478	0.050	-1.485	0.501
12199	0.466	1.178	0.467	0.512	1.453	0.478	0.476	1.245	0.492
12200	0.450	1.076	0.142	0.509	1.434	0.491	0.476	1.245	0.224
12201	0.295	0.082	0.224	0.272	-0.091	0.501	0.097	-1.181	0.185
12202	0.142	-0.895	0.224	0.307	0.135	0.149	0.097	-1.181	0.492
12203	0.329	0.301	0.224	0.102	-1.184	0.149	0.187	-0.608	0.262
12204	0.511	1.464	0.185	0.488	1.302	0.501	0.450	1.076	0.262
12205	0.489	1.329	0.185	0.515	1.472	0.102	0.505	1.430	0.224
12206	0.482	1.279	0.467	0.515	1.472	0.149	0.263	-0.120	0.224
12207	0.508	1.447	0.481	0.401	0.737	0.422	0.495	1.363	0.501
12208	0.384	0.655	0.450	0.152	-0.863	0.235	0.295	0.082	0.508
12209	0.384	0.655	0.431	0.237	-0.317	0.235	0.387	0.672	0.508
12210	0.463	1.161	0.508	0.512	1.453	0.102	0.508	1.447	0.508
12211	0.447	1.060	0.224	0.512	1.453	0.478	0.468	1.194	0.481
12212	0.226	-0.356	0.224	0.237	-0.317	0.273	0.097	-1.181	0.492
12213	0.497	1.380	0.467	0.506	1.415	0.478	0.492	1.346	0.328
12214	0.508	1.447	0.501	0.474	1.208	0.235	0.511	1.464	0.328
12215	0.508	1.447	0.492	0.474	1.208	0.273	0.511	1.464	0.328
12216	0.463	1.161	0.501	0.488	1.302	0.273	0.492	1.346	0.328
12217	0.487	1.312	0.431	0.500	1.378	0.508	0.429	0.942	0.296
12218	0.184	-0.625	0.508	0.149	-0.882	0.235	0.224	-0.373	0.450
12219	0.184	-0.625	0.508	0.149	-0.882	0.309	0.263	-0.120	0.262
12220	0.482	1.279	0.501	0.500	1.378	0.491	0.505	1.430	0.296
12221	0.295	0.082	0.262	0.102	-1.184	0.193	0.142	-0.895	0.262
12222	0.142	-0.895	0.050	0.149	-0.882	0.501	0.261	-0.137	0.450
12223	0.497	1.380	0.492	0.424	0.888	0.501	0.466	1.178	0.501
12224	0.050	-1.485	0.450	0.234	-0.336	0.478	0.295	0.082	0.467
12225	0.050	-1.485	0.512	0.234	-0.336	0.508	0.295	0.082	0.142
12226	0.295	0.082	0.467	0.102	-1.184	0.341	0.142	-0.895	0.142
12227	0.508	1.447	0.492	0.482	1.265	0.273	0.482	1.279	0.142
12228	0.142	-0.895	0.492	0.275	-0.073	0.478	0.361	0.504	0.142
12229	0.508	1.447	0.512	0.482	1.265	0.341	0.482	1.279	0.513
12230	0.263	-0.120	0.508	0.237	-0.317	0.341	0.097	-1.181	0.262
12231	0.263	-0.120	0.224	0.275	-0.073	0.398	0.097	-1.181	0.185
12232	0.263	-0.120	0.501	0.275	-0.073	0.491	0.097	-1.181	0.185
12233	0.492	1.346	0.512	0.512	1.453	0.149	0.511	1.464	0.185
12234	0.097	-1.181	0.501	0.237	-0.317	0.462	0.226	-0.356	0.185
12235	0.411	0.824	0.262	0.310	0.153	0.341	0.261	-0.137	0.185
12236	0.511	1.464	0.508	0.491	1.321	0.491	0.503	1.413	0.508
12237	0.332	0.318	0.224	0.196	-0.581	0.398	0.261	-0.137	0.501
12238	0.471	1.211	0.512	0.500	1.378	0.235	0.497	1.380	0.450
12239	0.471	1.211	0.501	0.500	1.378	0.422	0.503	1.413	0.481
12240	0.471	1.211	0.467	0.480	1.246	0.422	0.503	1.413	0.508
12241	0.511	1.464	0.467	0.512	1.453	0.273	0.432	0.958	0.142
12242	0.263	-0.120	0.467	0.339	0.342	0.341	0.384	0.655	0.142
12243	0.261	-0.137	0.513	0.275	-0.073	0.371	0.187	-0.608	0.481
12244	0.445	1.043	0.224	0.474	1.208	0.341	0.508	1.447	0.492
12245	0.142	-0.895	0.512	0.345	0.379	0.273	0.261	-0.137	0.467
12246	0.300	0.116	0.513	0.342	0.361	0.501	0.329	0.301	0.450
12247	0.358	0.487	0.296	0.398	0.718	0.193	0.332	0.318	0.512
12248	0.466	1.178	0.492	0.491	1.321	0.193	0.466	1.178	0.508
12249	0.142	-0.895	0.142	0.152	-0.863	0.512	0.261	-0.137	0.508
12250	0.445	1.043	0.185	0.462	1.133	0.444	0.508	1.447	0.513
12251	0.097	-1.181	0.224	0.342	0.361	0.102	0.142	-0.895	0.097
12252	0.492	1.346	0.450	0.488	1.302	0.235	0.495	1.363	0.296
12253	0.466	1.178	0.224	0.398	0.718	0.478	0.489	1.329	0.508
12254	0.226	-0.356	0.050	0.234	-0.336	0.102	0.097	-1.181	0.431
12255	0.261	-0.137	0.185	0.424	0.888	0.444	0.226	-0.356	0.492
12256	0.447	1.060	0.501	0.424	0.888	0.491	0.505	1.430	0.492
12257	0.187	-0.608	0.328	0.275	-0.073	0.273	0.097	-1.181	0.450

12258	0.224	−0.373	0.142	0.342	0.361	0.508	0.226	−0.356	0.450
12259	0.476	1.245	0.492	0.374	0.568	0.309	0.505	1.430	0.508
12260	0.224	−0.373	0.501	0.345	0.379	0.235	0.358	0.487	0.512
12261	0.184	−0.625	0.508	0.275	−0.073	0.508	0.261	−0.137	0.185
12262	0.476	1.245	0.508	0.509	1.434	0.512	0.492	1.346	0.512
12263	0.097	−1.181	0.492	0.196	−0.581	0.508	0.263	−0.120	0.481
12264	0.224	−0.373	0.512	0.193	−0.600	0.508	0.226	−0.356	0.492
12265	0.516	1.498	0.508	0.512	1.453	0.508	0.484	1.295	0.492
12266	0.263	−0.120	0.450	0.447	1.039	0.149	0.184	−0.625	0.142
12267	0.097	−1.181	0.296	0.102	−1.184	0.512	0.358	0.487	0.481
12268	0.184	−0.625	0.224	0.237	−0.317	0.491	0.295	0.082	0.467
12269	0.453	1.093	0.097	0.477	1.227	0.235	0.479	1.262	0.501
12270	0.184	−0.625	0.450	0.102	−1.184	0.478	0.263	−0.120	0.513
12271	0.492	1.346	0.450	0.444	1.020	0.309	0.453	1.093	0.513
12272	0.453	1.093	0.224	0.494	1.340	0.398	0.516	1.498	0.450
12273	0.142	−0.895	0.501	0.272	−0.091	0.478	0.263	−0.120	0.185
12274	0.511	1.464	0.467	0.506	1.415	0.422	0.445	1.043	0.450
12275	0.224	−0.373	0.224	0.313	0.172	0.512	0.097	−1.181	0.431
12276	0.142	−0.895	0.481	0.234	−0.336	0.273	0.184	−0.625	0.508
12277	0.445	1.043	0.481	0.506	1.415	0.273	0.466	1.178	0.224
12278	0.516	1.498	0.481	0.515	1.472	0.501	0.463	1.161	0.296
12279	0.453	1.093	0.481	0.506	1.415	0.478	0.471	1.211	0.097
12280	0.508	1.447	0.501	0.509	1.434	0.491	0.500	1.397	0.508
12281	0.508	1.447	0.501	0.506	1.415	0.341	0.463	1.161	0.142
12282	0.329	0.301	0.508	0.149	−0.882	0.462	0.329	0.301	0.328
12283	0.495	1.363	0.492	0.518	1.491	0.512	0.497	1.380	0.185
12284	0.505	1.430	0.492	0.491	1.321	0.341	0.489	1.329	0.358
12285	0.184	−0.625	0.512	0.237	−0.317	0.341	0.142	−0.895	0.296
12286	0.511	1.464	0.224	0.480	1.246	0.235	0.432	0.958	0.431
12287	0.097	−1.181	0.224	0.310	0.153	0.422	0.384	0.655	0.450
12288	0.184	−0.625	0.262	0.398	0.718	0.444	0.329	0.301	0.501
12289	0.508	1.447	0.328	0.474	1.208	0.512	0.511	1.464	0.501
12290	0.187	−0.608	0.431	0.421	0.869	0.341	0.261	−0.137	0.481
12291	0.503	1.413	0.262	0.518	1.491	0.273	0.492	1.346	0.492
12292	0.263	−0.120	0.467	0.272	−0.091	0.514	0.384	0.655	0.467
12293	0.224	−0.373	0.508	0.272	−0.091	0.273	0.384	0.655	0.481
12294	0.508	1.447	0.481	0.503	1.396	0.444	0.497	1.380	0.142
12295	0.408	0.807	0.512	0.480	1.246	0.398	0.405	0.790	0.385
12296	0.408	0.807	0.142	0.491	1.321	0.501	0.405	0.790	0.385
12297	0.476	1.245	0.467	0.348	0.398	0.193	0.466	1.178	0.492
12298	0.511	1.464	0.185	0.462	1.133	0.235	0.429	0.942	0.508
12299	0.505	1.430	0.097	0.518	1.491	0.478	0.408	0.807	0.097
12300	0.145	−0.878	0.185	0.342	0.361	0.341	0.050	−1.485	0.513
12301	0.145	−0.878	0.224	0.342	0.361	0.398	0.050	−1.485	0.492
12302	0.142	−0.895	0.224	0.234	−0.336	0.235	0.184	−0.625	0.224
12303	0.492	1.346	0.512	0.424	0.888	0.235	0.503	1.413	0.481
12304	0.492	1.346	0.185	0.444	1.020	0.478	0.508	1.447	0.492
12305	0.508	1.447	0.501	0.515	1.472	0.478	0.466	1.178	0.467
12306	0.184	−0.625	0.512	0.345	0.379	0.478	0.187	−0.608	0.467
12307	0.261	−0.137	0.224	0.275	−0.073	0.478	0.100	−1.164	0.512
12308	0.508	1.447	0.185	0.515	1.472	0.478	0.447	1.060	0.501
12309	0.097	−1.181	0.224	0.272	−0.091	0.508	0.329	0.301	0.492
12310	0.411	0.824	0.328	0.444	1.020	0.273	0.513	1.481	0.481
12311	0.503	1.413	0.142	0.395	0.700	0.273	0.503	1.413	0.508
12312	0.479	1.262	0.224	0.503	1.396	0.149	0.492	1.346	0.409
12313	0.187	−0.608	0.513	0.196	−0.581	0.149	0.224	−0.373	0.142
12314	0.224	−0.373	0.224	0.237	−0.317	0.273	0.184	−0.625	0.185
12315	0.505	1.430	0.481	0.477	1.227	0.478	0.487	1.312	0.262
12316	0.295	0.082	0.508	0.342	0.361	0.341	0.263	−0.120	0.262
12317	0.295	0.082	0.508	0.401	0.737	0.102	0.261	−0.137	0.512
12318	0.142	−0.895	0.224	0.240	−0.299	0.102	0.224	−0.373	0.224

12319	0.226	-0.356	0.492	0.237	-0.317	0.501	0.224	-0.373	0.512
12320	0.405	0.790	0.328	0.471	1.189	0.501	0.503	1.413	0.450
12321	0.405	0.790	0.224	0.471	1.189	0.501	0.511	1.464	0.450
12322	0.405	0.790	0.224	0.471	1.189	0.309	0.511	1.464	0.481
12323	0.405	0.790	0.185	0.471	1.189	0.193	0.511	1.464	0.224
12324	0.487	1.312	0.296	0.477	1.227	0.193	0.482	1.279	0.512
12325	0.487	1.312	0.142	0.509	1.434	0.193	0.492	1.346	0.185
12326	0.184	-0.625	0.467	0.272	-0.091	0.501	0.326	0.285	0.224
12327	0.224	-0.373	0.296	0.272	-0.091	0.193	0.326	0.285	0.185
12328	0.097	-1.181	0.224	0.149	-0.882	0.149	0.326	0.285	0.296
12329	0.097	-1.181	0.142	0.149	-0.882	0.149	0.326	0.285	0.185
12330	0.142	-0.895	0.142	0.272	-0.091	0.149	0.297	0.099	0.431
12331	0.453	1.093	0.501	0.477	1.227	0.341	0.450	1.076	0.358
12332	0.297	0.099	0.262	0.339	0.342	0.341	0.263	-0.120	0.512
12333	0.184	-0.625	0.501	0.102	-1.184	0.341	0.295	0.082	0.492
12334	0.184	-0.625	0.492	0.102	-1.184	0.508	0.261	-0.137	0.224
12335	0.497	1.380	0.296	0.500	1.378	0.501	0.450	1.076	0.262
12336	0.497	1.380	0.492	0.500	1.378	0.514	0.500	1.397	0.296
12337	0.505	1.430	0.328	0.500	1.378	0.422	0.466	1.178	0.142
12338	0.263	-0.120	0.450	0.307	0.135	0.309	0.142	-0.895	0.224
12339	0.266	-0.103	0.481	0.193	-0.600	0.309	0.142	-0.895	0.385
12340	0.266	-0.103	0.501	0.193	-0.600	0.501	0.142	-0.895	0.409
12341	0.266	-0.103	0.262	0.193	-0.600	0.462	0.142	-0.895	0.224
12342	0.466	1.178	0.508	0.497	1.359	0.512	0.508	1.447	0.512
12343	0.142	-0.895	0.501	0.196	-0.581	0.462	0.263	-0.120	0.501
12344	0.226	-0.356	0.262	0.152	-0.863	0.501	0.184	-0.625	0.508
12345	0.226	-0.356	0.097	0.152	-0.863	0.491	0.184	-0.625	0.185
12346	0.226	-0.356	0.262	0.152	-0.863	0.501	0.184	-0.625	0.467
12347	0.187	-0.608	0.481	0.342	0.361	0.508	0.187	-0.608	0.358
12348	0.187	-0.608	0.492	0.342	0.361	0.235	0.187	-0.608	0.262
12349	0.463	1.161	0.501	0.339	0.342	0.273	0.508	1.447	0.142
12350	0.482	1.279	0.224	0.503	1.396	0.501	0.503	1.413	0.142
12351	0.450	1.076	0.328	0.497	1.359	0.514	0.453	1.093	0.224
12352	0.426	0.925	0.328	0.512	1.453	0.341	0.479	1.262	0.142
12353	0.505	1.430	0.508	0.421	0.869	0.444	0.508	1.447	0.501
12354	0.226	-0.356	0.450	0.307	0.135	0.491	0.142	-0.895	0.142
12355	0.226	-0.356	0.512	0.307	0.135	0.491	0.142	-0.895	0.296
12356	0.471	1.211	0.513	0.500	1.378	0.462	0.474	1.228	0.224
12357	0.500	1.397	0.512	0.462	1.133	0.501	0.487	1.312	0.224
12358	0.489	1.329	0.296	0.518	1.491	0.193	0.471	1.211	0.501
12359	0.497	1.380	0.296	0.459	1.114	0.514	0.450	1.076	0.224
12360	0.426	0.925	0.501	0.494	1.340	0.512	0.513	1.481	0.450
12361	0.508	1.447	0.385	0.488	1.302	0.491	0.511	1.464	0.508
12362	0.508	1.447	0.142	0.497	1.359	0.273	0.511	1.464	0.185
12363	0.500	1.397	0.224	0.503	1.396	0.478	0.516	1.498	0.508
12364	0.261	-0.137	0.481	0.234	-0.336	0.235	0.097	-1.181	0.097
12365	0.050	-1.485	0.512	0.272	-0.091	0.422	0.297	0.099	0.467
12366	0.492	1.346	0.501	0.503	1.396	0.491	0.511	1.464	0.508
12367	0.447	1.060	0.142	0.512	1.453	0.501	0.434	0.975	0.431
12368	0.513	1.481	0.450	0.339	0.342	0.462	0.489	1.329	0.142
12369	0.468	1.194	0.492	0.439	0.982	0.462	0.487	1.312	0.512
12370	0.489	1.329	0.450	0.491	1.321	0.501	0.445	1.043	0.358
12371	0.489	1.329	0.467	0.491	1.321	0.149	0.445	1.043	0.142
12372	0.511	1.464	0.328	0.456	1.095	0.512	0.511	1.464	0.385
12373	0.511	1.464	0.224	0.497	1.359	0.508	0.505	1.430	0.296
12374	0.229	-0.339	0.328	0.193	-0.600	0.341	0.184	-0.625	0.492
12375	0.492	1.346	0.385	0.515	1.472	0.422	0.511	1.464	0.508
12376	0.505	1.430	0.513	0.515	1.472	0.273	0.479	1.262	0.512
12377	0.500	1.397	0.224	0.488	1.302	0.053	0.487	1.312	0.358
12378	0.261	-0.137	0.142	0.278	-0.054	0.235	0.000	?	0.185
12379	0.505	1.430	0.224	0.477	1.227	0.512	0.497	1.380	0.185

12380	0.226	-0.356	0.224	0.237	-0.317	0.235	0.142	-0.895	0.512
12381	0.505	1.430	0.501	0.421	0.869	0.149	0.476	1.245	0.467
12382	0.495	1.363	0.185	0.491	1.321	0.193	0.463	1.161	0.467
12383	0.466	1.178	0.481	0.494	1.340	0.508	0.497	1.380	0.431
12384	0.471	1.211	0.501	0.465	1.152	0.193	0.511	1.464	0.385
12385	0.471	1.211	0.358	0.465	1.152	0.102	0.511	1.464	0.358
12386	0.511	1.464	0.262	0.497	1.359	0.501	0.453	1.093	0.097
12387	0.224	-0.373	0.262	0.149	-0.882	0.512	0.184	-0.625	0.481
12388	0.511	1.464	0.385	0.506	1.415	0.478	0.450	1.076	0.513
12389	0.511	1.464	0.097	0.503	1.396	0.478	0.432	0.958	0.385
12390	0.297	0.099	0.481	0.342	0.361	0.512	0.000	?	0.296
12391	0.495	1.363	0.328	0.421	0.869	0.512	0.497	1.380	0.512
12392	0.142	-0.895	0.431	0.275	-0.073	0.491	0.224	-0.373	0.501
12393	0.184	-0.625	0.501	0.053	-1.504	0.508	0.297	0.099	0.501
12394	0.224	-0.373	0.501	0.234	-0.336	0.273	0.097	-1.181	0.296
12395	0.450	1.076	0.224	0.515	1.472	0.309	0.500	1.397	0.501
12396	0.224	-0.373	0.224	0.237	-0.317	0.235	0.142	-0.895	0.385
12397	0.050	-1.485	0.224	0.149	-0.882	0.491	0.329	0.301	0.501
12398	0.184	-0.625	0.492	0.193	-0.600	0.491	0.184	-0.625	0.508
12399	0.497	1.380	0.224	0.512	1.453	0.235	0.355	0.470	0.050
12400	0.329	0.301	0.385	0.193	-0.600	0.514	0.000	?	0.142
12401	0.142	-0.895	0.296	0.102	-1.184	0.491	0.297	0.099	0.097
12402	0.495	1.363	0.050	0.500	1.378	0.193	0.432	0.958	0.492
12403	0.500	1.397	0.142	0.509	1.434	0.501	0.447	1.060	0.409
12404	0.508	1.447	0.142	0.474	1.208	0.508	0.500	1.397	0.050
12405	0.508	1.447	0.508	0.474	1.208	0.478	0.500	1.397	0.050
12406	0.492	1.346	0.097	0.512	1.453	0.462	0.479	1.262	0.142
12407	0.508	1.447	0.481	0.509	1.434	0.478	0.489	1.329	0.050
12408	0.503	1.413	0.481	0.494	1.340	0.478	0.461	1.144	0.467
12409	0.447	1.060	0.481	0.509	1.434	0.508	0.482	1.279	0.508
12410	0.297	0.099	0.224	0.275	-0.073	0.491	0.142	-0.895	0.501
12411	0.224	-0.373	0.481	0.310	0.153	0.371	0.384	0.655	0.185
12412	0.097	-1.181	0.512	0.237	-0.317	0.462	0.387	0.672	0.050
12413	0.455	1.110	0.142	0.494	1.340	0.193	0.489	1.329	0.142
12414	0.453	1.093	0.467	0.494	1.340	0.193	0.505	1.430	0.185
12415	0.224	-0.373	0.142	0.237	-0.317	0.102	0.097	-1.181	0.262
12416	0.503	1.413	0.142	0.512	1.453	0.309	0.508	1.447	0.492
12417	0.468	1.194	0.142	0.497	1.359	0.478	0.492	1.346	0.097
12418	0.226	-0.356	0.185	0.193	-0.600	0.235	0.226	-0.356	0.512
12419	0.482	1.279	0.508	0.500	1.378	0.512	0.479	1.262	0.512
12420	0.479	1.262	0.467	0.509	1.434	0.512	0.489	1.329	0.409
12421	0.476	1.245	0.501	0.477	1.227	0.512	0.463	1.161	0.296
12422	0.476	1.245	0.501	0.462	1.133	0.514	0.463	1.161	0.296
12423	0.503	1.413	0.431	0.482	1.265	0.235	0.516	1.498	0.097
12424	0.500	1.397	0.513	0.480	1.246	0.501	0.508	1.447	0.481
12425	0.505	1.430	0.409	0.506	1.415	0.235	0.489	1.329	0.097
12426	0.492	1.346	0.508	0.485	1.283	0.341	0.479	1.262	0.050
12427	0.492	1.346	0.481	0.368	0.530	0.193	0.513	1.481	0.050
12428	0.511	1.464	0.501	0.462	1.133	0.309	0.413	0.841	0.262
12429	0.224	-0.373	0.492	0.196	-0.581	0.341	0.142	-0.895	0.142
12430	0.224	-0.373	0.508	0.196	-0.581	0.514	0.184	-0.625	0.185
12431	0.261	-0.137	0.501	0.105	-1.165	0.309	0.263	-0.120	0.492
12432	0.332	0.318	0.481	0.313	0.172	0.491	0.261	-0.137	0.185
12433	0.429	0.942	0.481	0.482	1.265	0.514	0.513	1.481	0.508
12434	0.263	-0.120	0.450	0.240	-0.299	0.341	0.224	-0.373	0.512
12435	0.466	1.178	0.467	0.509	1.434	0.309	0.513	1.481	0.501
12436	0.505	1.430	0.097	0.512	1.453	0.341	0.445	1.043	0.328
12437	0.479	1.262	0.481	0.509	1.434	0.309	0.445	1.043	0.481
12438	0.513	1.481	0.512	0.518	1.491	0.273	0.484	1.295	0.450
12439	0.142	-0.895	0.492	0.237	-0.317	0.371	0.226	-0.356	0.224
12440	0.466	1.178	0.050	0.500	1.378	0.501	0.511	1.464	0.508

12441	0.187	-0.608	0.513	0.234	-0.336	0.273	0.184	-0.625	0.328
12442	0.097	-1.181	0.508	0.339	0.342	0.501	0.224	-0.373	0.328
12443	0.184	-0.625	0.262	0.193	-0.600	0.501	0.184	-0.625	0.328
12444	0.224	-0.373	0.450	0.313	0.172	0.478	0.295	0.082	0.296
12445	0.224	-0.373	0.492	0.345	0.379	0.193	0.184	-0.625	0.501
12446	0.508	1.447	0.508	0.515	1.472	0.462	0.432	0.958	0.508
12447	0.184	-0.625	0.185	0.310	0.153	0.193	0.361	0.504	0.513
12448	0.500	1.397	0.501	0.491	1.321	0.149	0.511	1.464	0.513
12449	0.508	1.447	0.224	0.512	1.453	0.193	0.489	1.329	0.481
12450	0.224	-0.373	0.224	0.342	0.361	0.235	0.224	-0.373	0.492
12451	0.184	-0.625	0.508	0.310	0.153	0.193	0.261	-0.137	0.508
12452	0.224	-0.373	0.512	0.342	0.361	0.273	0.297	0.099	0.513
12453	0.332	0.318	0.492	0.310	0.153	0.478	0.142	-0.895	0.508
12454	0.142	-0.895	0.501	0.272	-0.091	0.193	0.226	-0.356	0.512
12455	0.224	-0.373	0.142	0.371	0.549	0.235	0.384	0.655	0.501
12456	0.516	1.498	0.508	0.500	1.378	0.193	0.411	0.824	0.492
12457	0.224	-0.373	0.492	0.272	-0.091	0.193	0.226	-0.356	0.467
12458	0.476	1.245	0.492	0.500	1.378	0.491	0.513	1.481	0.513
12459	0.500	1.397	0.508	0.500	1.378	0.235	0.500	1.397	0.508
12460	0.508	1.447	0.508	0.480	1.246	0.514	0.516	1.498	0.508
12461	0.224	-0.373	0.409	0.193	-0.600	0.508	0.187	-0.608	0.481
12462	0.492	1.346	0.481	0.465	1.152	0.102	0.474	1.228	0.328
12463	0.332	0.318	0.512	0.196	-0.581	0.508	0.358	0.487	0.512
12464	0.226	-0.356	0.513	0.149	-0.882	0.235	0.261	-0.137	0.409
12465	0.224	-0.373	0.142	0.193	-0.600	0.508	0.142	-0.895	0.492
12466	0.184	-0.625	0.097	0.234	-0.336	0.512	0.142	-0.895	0.224
12467	0.295	0.082	0.512	0.196	-0.581	0.512	0.224	-0.373	0.467
12468	0.142	-0.895	0.501	0.275	-0.073	0.309	0.145	-0.878	0.492
12469	0.466	1.178	0.501	0.480	1.246	0.422	0.497	1.380	0.185
12470	0.297	0.099	0.513	0.193	-0.600	0.501	0.145	-0.878	0.492
12471	0.226	-0.356	0.481	0.237	-0.317	0.149	0.297	0.099	0.501
12472	0.145	-0.878	0.492	0.193	-0.600	0.102	0.224	-0.373	0.296
12473	0.145	-0.878	0.508	0.193	-0.600	0.341	0.224	-0.373	0.508
12474	0.500	1.397	0.508	0.494	1.340	0.512	0.503	1.413	0.296
12475	0.261	-0.137	0.508	0.234	-0.336	0.491	0.226	-0.356	0.501
12476	0.500	1.397	0.262	0.515	1.472	0.508	0.447	1.060	0.224
12477	0.489	1.329	0.492	0.509	1.434	0.371	0.505	1.430	0.142
12478	0.300	0.116	0.501	0.102	-1.184	0.371	0.184	-0.625	0.481
12479	0.489	1.329	0.508	0.500	1.378	0.371	0.508	1.447	0.481
12480	0.329	0.301	0.508	0.234	-0.336	0.462	0.097	-1.181	0.481
12481	0.453	1.093	0.508	0.509	1.434	0.491	0.466	1.178	0.481
12482	0.482	1.279	0.481	0.515	1.472	0.508	0.508	1.447	0.224
12483	0.511	1.464	0.467	0.515	1.472	0.508	0.426	0.925	0.508
12484	0.263	-0.120	0.142	0.310	0.153	0.501	0.142	-0.895	0.508
12485	0.508	1.447	0.512	0.430	0.926	0.273	0.511	1.464	0.501
12486	0.500	1.397	0.492	0.506	1.415	0.149	0.358	0.487	0.492
12487	0.263	-0.120	0.492	0.152	-0.863	0.508	0.142	-0.895	0.508
12488	0.097	-1.181	0.050	0.105	-1.165	0.478	0.384	0.655	0.513
12489	0.261	-0.137	0.513	0.345	0.379	0.235	0.300	0.116	0.501
12490	0.482	1.279	0.467	0.512	1.453	0.149	0.489	1.329	0.512
12491	0.487	1.312	0.492	0.488	1.302	0.512	0.508	1.447	0.513
12492	0.495	1.363	0.508	0.503	1.396	0.508	0.513	1.481	0.409
12493	0.226	-0.356	0.501	0.368	0.530	0.501	0.355	0.470	0.296
12494	0.332	0.318	0.481	0.371	0.549	0.273	0.184	-0.625	0.328
12495	0.332	0.318	0.508	0.371	0.549	0.512	0.184	-0.625	0.450
12496	0.508	1.447	0.508	0.465	1.152	0.491	0.513	1.481	0.492
12497	0.447	1.060	0.467	0.494	1.340	0.512	0.474	1.228	0.501
12498	0.508	1.447	0.512	0.503	1.396	0.462	0.471	1.211	0.492
12499	0.516	1.498	0.512	0.503	1.396	0.149	0.426	0.925	0.492
12500	0.516	1.498	0.508	0.497	1.359	0.235	0.382	0.638	0.467
12501	0.295	0.082	0.492	0.272	-0.091	0.102	0.355	0.470	0.450

12502	0.300	0.116	0.492	0.149	-0.882	0.478	0.097	-1.181	0.508
12503	0.505	1.430	0.481	0.500	1.378	0.512	0.476	1.245	0.492
12504	0.387	0.672	0.512	0.477	1.227	0.273	0.513	1.481	0.142
12505	0.142	-0.895	0.481	0.237	-0.317	0.341	0.387	0.672	0.508
12506	0.226	-0.356	0.512	0.152	-0.863	0.193	0.295	0.082	0.501
12507	0.479	1.262	0.467	0.512	1.453	0.273	0.516	1.498	0.508
12508	0.508	1.447	0.512	0.509	1.434	0.514	0.503	1.413	0.512
12509	0.497	1.380	0.224	0.500	1.378	0.341	0.503	1.413	0.512
12510	0.142	-0.895	0.508	0.275	-0.073	0.444	0.300	0.116	0.481
12511	0.450	1.076	0.513	0.515	1.472	0.478	0.503	1.413	0.467
12512	0.492	1.346	0.512	0.491	1.321	0.149	0.384	0.655	0.185
12513	0.450	1.076	0.224	0.515	1.472	0.235	0.503	1.413	0.481
12514	0.466	1.178	0.508	0.462	1.133	0.193	0.500	1.397	0.467
12515	0.332	0.318	0.508	0.149	-0.882	0.193	0.050	-1.485	0.467
12516	0.226	-0.356	0.501	0.237	-0.317	0.309	0.142	-0.895	0.262
12517	0.326	0.285	0.501	0.102	-1.184	0.512	0.097	-1.181	0.142
12518	0.387	0.672	0.262	0.482	1.265	0.102	0.492	1.346	0.501
12519	0.511	1.464	0.501	0.515	1.472	0.444	0.408	0.807	0.508
12520	0.226	-0.356	0.513	0.272	-0.091	0.478	0.050	-1.485	0.508
12521	0.142	-0.895	0.508	0.345	0.379	0.462	0.050	-1.485	0.512
12522	0.226	-0.356	0.450	0.193	-0.600	0.053	0.142	-0.895	0.409
12523	0.224	-0.373	0.142	0.275	-0.073	0.053	0.050	-1.485	0.501
12524	0.503	1.413	0.481	0.509	1.434	0.309	0.461	1.144	0.050
12525	0.184	-0.625	0.481	0.345	0.379	0.444	0.000	?	0.501
12526	0.482	1.279	0.481	0.442	1.001	0.273	0.500	1.397	0.501
12527	0.503	1.413	0.481	0.474	1.208	0.193	0.495	1.363	0.513
12528	0.361	0.504	0.512	0.149	-0.882	0.193	0.184	-0.625	0.467
12529	0.261	-0.137	0.501	0.234	-0.336	0.398	0.050	-1.485	0.512
12530	0.263	-0.120	0.501	0.193	-0.600	0.273	0.142	-0.895	0.467
12531	0.384	0.655	0.508	0.193	-0.600	0.273	0.184	-0.625	0.512
12532	0.097	-1.181	0.508	0.310	0.153	0.512	0.263	-0.120	0.508
12533	0.482	1.279	0.492	0.512	1.453	0.309	0.484	1.295	0.508
12534	0.329	0.301	0.492	0.102	-1.184	0.491	0.097	-1.181	0.512
12535	0.426	0.925	0.467	0.444	1.020	0.462	0.508	1.447	0.508
12536	0.495	1.363	0.512	0.480	1.246	0.514	0.508	1.447	0.513
12537	0.500	1.397	0.501	0.459	1.114	0.235	0.405	0.790	0.512
12538	0.224	-0.373	0.492	0.053	-1.504	0.422	0.300	0.116	0.262
12539	0.224	-0.373	0.508	0.053	-1.504	0.508	0.300	0.116	0.492
12540	0.226	-0.356	0.385	0.310	0.153	0.193	0.097	-1.181	0.097
12541	0.489	1.329	0.358	0.444	1.020	0.512	0.487	1.312	0.501
12542	0.224	-0.373	0.358	0.278	-0.054	0.235	0.097	-1.181	0.492
12543	0.382	0.638	0.385	0.193	-0.600	0.235	0.050	-1.485	0.492
12544	0.297	0.099	0.142	0.193	-0.600	0.235	0.050	-1.485	0.224
12545	0.050	-1.485	0.262	0.401	0.737	0.193	0.263	-0.120	0.508
12546	0.142	-0.895	0.481	0.275	-0.073	0.512	0.145	-0.878	0.508
12547	0.142	-0.895	0.481	0.275	-0.073	0.478	0.187	-0.608	0.508
12548	0.508	1.447	0.512	0.518	1.491	0.462	0.492	1.346	0.409
12549	0.100	-1.164	0.481	0.310	0.153	0.501	0.184	-0.625	0.328
12550	0.479	1.262	0.224	0.491	1.321	0.491	0.508	1.447	0.492
12551	0.482	1.279	0.224	0.465	1.152	0.508	0.511	1.464	0.481
12552	0.474	1.228	0.328	0.512	1.453	0.501	0.508	1.447	0.492
12553	0.226	-0.356	0.508	0.234	-0.336	0.501	0.329	0.301	0.508
12554	0.476	1.245	0.508	0.424	0.888	0.514	0.476	1.245	0.142
12555	0.511	1.464	0.296	0.506	1.415	0.512	0.447	1.060	0.501
12556	0.142	-0.895	0.508	0.193	-0.600	0.514	0.224	-0.373	0.508
12557	0.471	1.211	0.501	0.512	1.453	0.478	0.505	1.430	0.512
12558	0.142	-0.895	0.358	0.237	-0.317	0.512	0.332	0.318	0.513
12559	0.142	-0.895	0.409	0.237	-0.317	0.478	0.332	0.318	0.501
12560	0.142	-0.895	0.492	0.237	-0.317	0.501	0.332	0.318	0.492
12561	0.184	-0.625	0.512	0.193	-0.600	0.478	0.300	0.116	0.512
12562	0.503	1.413	0.467	0.506	1.415	0.491	0.505	1.430	0.450

12563	0.471	1.211	0.508	0.480	1.246	0.102	0.511	1.464	0.481
12564	0.495	1.363	0.512	0.459	1.114	0.501	0.513	1.481	0.501
12565	0.495	1.363	0.512	0.500	1.378	0.491	0.513	1.481	0.501
12566	0.426	0.925	0.513	0.485	1.283	0.514	0.479	1.262	0.508
12567	0.511	1.464	0.501	0.503	1.396	0.309	0.489	1.329	0.501
12568	0.408	0.807	0.450	0.503	1.396	0.508	0.511	1.464	0.513
12569	0.505	1.430	0.492	0.503	1.396	0.512	0.508	1.447	0.513
12570	0.479	1.262	0.512	0.515	1.472	0.102	0.503	1.413	0.512
12571	0.500	1.397	0.097	0.515	1.472	0.508	0.513	1.481	0.142
12572	0.492	1.346	0.512	0.515	1.472	0.478	0.503	1.413	0.185
12573	0.503	1.413	0.508	0.482	1.265	0.273	0.487	1.312	0.185
12574	0.495	1.363	0.501	0.518	1.491	0.508	0.461	1.144	0.185
12575	0.484	1.295	0.262	0.482	1.265	0.053	0.508	1.447	0.262
12576	0.484	1.295	0.508	0.503	1.396	0.512	0.503	1.413	0.224
12577	0.447	1.060	0.513	0.474	1.208	0.149	0.508	1.447	0.512
12578	0.468	1.194	0.501	0.488	1.302	0.193	0.476	1.245	0.512
12579	0.097	-1.181	0.467	0.102	-1.184	0.422	0.329	0.301	0.512
12580	0.476	1.245	0.467	0.503	1.396	0.398	0.508	1.447	0.513
12581	0.508	1.447	0.097	0.491	1.321	0.398	0.411	0.824	0.296
12582	0.489	1.329	0.262	0.515	1.472	0.512	0.489	1.329	0.296
12583	0.050	-1.485	0.512	0.307	0.135	0.193	0.224	-0.373	0.385
12584	0.516	1.498	0.508	0.506	1.415	0.508	0.466	1.178	0.450
12585	0.508	1.447	0.508	0.515	1.472	0.501	0.497	1.380	0.450
12586	0.266	-0.103	0.501	0.102	-1.184	0.512	0.184	-0.625	0.508
12587	0.450	1.076	0.467	0.512	1.453	0.444	0.487	1.312	0.508
12588	0.497	1.380	0.492	0.480	1.246	0.462	0.495	1.363	0.050
12589	0.000	?	0.097	0.272	-0.091	0.512	0.297	0.099	0.358
12590	0.503	1.413	0.050	0.500	1.378	0.514	0.511	1.464	0.467
12591	0.187	-0.608	0.142	0.053	-1.504	0.491	0.297	0.099	0.467
12592	0.492	1.346	0.501	0.518	1.491	0.491	0.503	1.413	0.431
12593	0.229	-0.339	0.185	0.149	-0.882	0.235	0.224	-0.373	0.508
12594	0.224	-0.373	0.481	0.193	-0.600	0.341	0.142	-0.895	0.450
12595	0.505	1.430	0.508	0.418	0.850	0.309	0.479	1.262	0.385
12596	0.508	1.447	0.508	0.395	0.700	0.491	0.479	1.262	0.513
12597	0.489	1.329	0.501	0.395	0.700	0.512	0.479	1.262	0.492
12598	0.497	1.380	0.501	0.509	1.434	0.462	0.479	1.262	0.431
12599	0.142	-0.895	0.512	0.196	-0.581	0.491	0.226	-0.356	0.513
12600	0.503	1.413	0.512	0.506	1.415	0.491	0.505	1.430	0.467
12601	0.489	1.329	0.501	0.503	1.396	0.478	0.511	1.464	0.262
12602	0.492	1.346	0.142	0.515	1.472	0.512	0.497	1.380	0.409
12603	0.503	1.413	0.328	0.442	1.001	0.512	0.489	1.329	0.492
12604	0.505	1.430	0.467	0.465	1.152	0.462	0.508	1.447	0.467
12605	0.408	0.807	0.450	0.515	1.472	0.149	0.513	1.481	0.224
12606	0.476	1.245	0.513	0.509	1.434	0.462	0.500	1.397	0.467
12607	0.511	1.464	0.512	0.488	1.302	0.491	0.513	1.481	0.431
12608	0.505	1.430	0.512	0.491	1.321	0.491	0.513	1.481	0.501
12609	0.142	-0.895	0.492	0.237	-0.317	0.491	0.411	0.824	0.492
12610	0.097	-1.181	0.492	0.345	0.379	0.491	0.295	0.082	0.481
12611	0.000	?	0.296	0.310	0.153	0.491	0.326	0.285	0.224
12612	0.500	1.397	0.467	0.491	1.321	0.512	0.453	1.093	0.224
12613	0.492	1.346	0.501	0.509	1.434	0.422	0.495	1.363	0.512
12614	0.495	1.363	0.508	0.462	1.133	0.512	0.500	1.397	0.467
12615	0.513	1.481	0.224	0.494	1.340	0.462	0.495	1.363	0.512
12616	0.476	1.245	0.512	0.488	1.302	0.462	0.489	1.329	0.508
12617	0.489	1.329	0.431	0.477	1.227	0.422	0.463	1.161	0.501
12618	0.503	1.413	0.142	0.518	1.491	0.341	0.450	1.076	0.513
12619	0.511	1.464	0.185	0.515	1.472	0.514	0.508	1.447	0.262
12620	0.505	1.430	0.492	0.465	1.152	0.508	0.492	1.346	0.358
12621	0.261	-0.137	0.481	0.149	-0.882	0.508	0.145	-0.878	0.224
12622	0.484	1.295	0.492	0.462	1.133	0.478	0.516	1.498	0.431
12623	0.500	1.397	0.481	0.485	1.283	0.512	0.497	1.380	0.224

12624	0.505	1.430	0.481	0.485	1.283	0.462	0.505	1.430	0.450
12625	0.505	1.430	0.508	0.485	1.283	0.422	0.511	1.464	0.501
12626	0.505	1.430	0.185	0.485	1.283	0.478	0.513	1.481	0.501
12627	0.479	1.262	0.513	0.488	1.302	0.478	0.476	1.245	0.501
12628	0.466	1.178	0.513	0.512	1.453	0.508	0.463	1.161	0.501
12629	0.145	-0.878	0.508	0.424	0.888	0.512	0.184	-0.625	0.513
12630	0.513	1.481	0.097	0.515	1.472	0.491	0.482	1.279	0.512
12631	0.492	1.346	0.050	0.465	1.152	0.491	0.466	1.178	0.508
12632	0.492	1.346	0.296	0.465	1.152	0.508	0.466	1.178	0.142
12633	0.000	?	0.296	0.427	0.907	0.514	0.261	-0.137	0.262
12634	0.050	-1.485	0.097	0.345	0.379	0.514	0.142	-0.895	0.508
12635	0.511	1.464	0.508	0.509	1.434	0.501	0.500	1.397	0.508
12636	0.466	1.178	0.508	0.518	1.491	0.514	0.508	1.447	0.467
12637	0.492	1.346	0.097	0.518	1.491	0.478	0.508	1.447	0.431
12638	0.497	1.380	0.508	0.482	1.265	0.501	0.513	1.481	0.501
12639	0.492	1.346	0.097	0.515	1.472	0.273	0.408	0.807	0.501
12640	0.474	1.228	0.512	0.459	1.114	0.371	0.503	1.413	0.501
12641	0.000	?	0.512	0.424	0.888	0.235	0.050	-1.485	0.296
12642	0.500	1.397	0.512	0.477	1.227	0.462	0.503	1.413	0.481
12643	0.500	1.397	0.512	0.477	1.227	0.514	0.503	1.413	0.358
12644	0.461	1.144	0.467	0.506	1.415	0.514	0.516	1.498	0.513
12645	0.508	1.447	0.513	0.506	1.415	0.149	0.466	1.178	0.481
12646	0.508	1.447	0.492	0.488	1.302	0.462	0.508	1.447	0.512
12647	0.500	1.397	0.512	0.488	1.302	0.462	0.466	1.178	0.296
12648	0.495	1.363	0.492	0.512	1.453	0.422	0.516	1.498	0.296
12649	0.495	1.363	0.409	0.518	1.491	0.478	0.511	1.464	0.185
12650	0.482	1.279	0.492	0.518	1.491	0.149	0.511	1.464	0.508
12651	0.511	1.464	0.296	0.500	1.378	0.512	0.516	1.498	0.508
12652	0.482	1.279	0.481	0.518	1.491	0.501	0.511	1.464	0.508
12653	0.508	1.447	0.501	0.477	1.227	0.508	0.513	1.481	0.508
12654	0.466	1.178	0.185	0.500	1.378	0.512	0.508	1.447	0.508
12655	0.000	?	0.492	0.278	-0.054	0.309	0.261	-0.137	0.492
12656	0.505	1.430	0.097	0.371	0.549	0.501	0.487	1.312	0.224
12657	0.226	-0.356	0.097	0.237	-0.317	0.462	0.097	-1.181	0.467
12658	0.508	1.447	0.501	0.465	1.152	0.501	0.500	1.397	0.492
12659	0.508	1.447	0.492	0.509	1.434	0.501	0.495	1.363	0.512
12660	0.505	1.430	0.097	0.509	1.434	0.512	0.495	1.363	0.262
12661	0.226	-0.356	0.501	0.149	-0.882	0.501	0.224	-0.373	0.358
12662	0.500	1.397	0.508	0.468	1.170	0.491	0.511	1.464	0.296
12663	0.500	1.397	0.097	0.468	1.170	0.512	0.511	1.464	0.296
12664	0.495	1.363	0.185	0.421	0.869	0.512	0.511	1.464	0.358
12665	0.492	1.346	0.512	0.480	1.246	0.501	0.408	0.807	0.508
12666	0.263	-0.120	0.142	0.152	-0.863	0.501	0.329	0.301	0.431
12667	0.492	1.346	0.431	0.518	1.491	0.501	0.497	1.380	0.328
12668	0.513	1.481	0.512	0.503	1.396	0.514	0.476	1.245	0.513
12669	0.508	1.447	0.142	0.509	1.434	0.514	0.487	1.312	0.262
12670	0.447	1.060	0.513	0.509	1.434	0.514	0.511	1.464	0.501
12671	0.142	-0.895	0.050	0.310	0.153	0.491	0.142	-0.895	0.492
12672	0.479	1.262	0.224	0.500	1.378	0.193	0.500	1.397	0.508
12673	0.474	1.228	0.262	0.462	1.133	0.235	0.511	1.464	0.508
12674	0.474	1.228	0.185	0.506	1.415	0.235	0.513	1.481	0.512
12675	0.474	1.228	0.508	0.506	1.415	0.235	0.513	1.481	0.508
12676	0.503	1.413	0.296	0.515	1.472	0.149	0.503	1.413	0.508
12677	0.492	1.346	0.512	0.503	1.396	0.102	0.489	1.329	0.501
12678	0.495	1.363	0.501	0.497	1.359	0.501	0.511	1.464	0.501
12679	0.503	1.413	0.492	0.512	1.453	0.491	0.445	1.043	0.501
12680	0.505	1.430	0.492	0.509	1.434	0.444	0.479	1.262	0.501
12681	0.487	1.312	0.492	0.500	1.378	0.512	0.497	1.380	0.358
12682	0.492	1.346	0.508	0.503	1.396	0.341	0.497	1.380	0.492
12683	0.466	1.178	0.508	0.497	1.359	0.371	0.508	1.447	0.481
12684	0.505	1.430	0.481	0.515	1.472	0.371	0.497	1.380	0.262

12685	0.500	1.397	0.296	0.515	1.472	0.462	0.505	1.430	0.492
12686	0.492	1.346	0.296	0.515	1.472	0.462	0.505	1.430	0.224
12687	0.508	1.447	0.224	0.494	1.340	0.273	0.511	1.464	0.358
12688	0.387	0.672	0.185	0.193	−0.600	0.398	0.142	−0.895	0.512
12689	0.358	0.487	0.296	0.234	−0.336	0.341	0.184	−0.625	0.512
12690	0.358	0.487	0.492	0.234	−0.336	0.341	0.184	−0.625	0.296
12691	0.387	0.672	0.492	0.234	−0.336	0.508	0.184	−0.625	0.501
12692	0.142	−0.895	0.513	0.149	−0.882	0.501	0.261	−0.137	0.492
12693	0.261	−0.137	0.501	0.102	−1.184	0.462	0.224	−0.373	0.224
12694	0.482	1.279	0.512	0.503	1.396	0.508	0.511	1.464	0.185
12695	0.482	1.279	0.224	0.494	1.340	0.501	0.511	1.464	0.512
12696	0.511	1.464	0.512	0.442	1.001	0.508	0.505	1.430	0.185
12697	0.479	1.262	0.142	0.512	1.453	0.478	0.508	1.447	0.508
12698	0.226	−0.356	0.508	0.339	0.342	0.508	0.295	0.082	0.513
12699	0.226	−0.356	0.512	0.368	0.530	0.501	0.295	0.082	0.296
12700	0.332	0.318	0.185	0.368	0.530	0.514	0.384	0.655	0.501
12701	0.511	1.464	0.185	0.459	1.114	0.478	0.450	1.076	0.142
12702	0.511	1.464	0.513	0.459	1.114	0.512	0.450	1.076	0.050
12703	0.297	0.099	0.467	0.275	−0.073	0.235	0.000	?	0.224
12704	0.503	1.413	0.142	0.398	0.718	0.491	0.508	1.447	0.185
12705	0.497	1.380	0.501	0.339	0.342	0.341	0.508	1.447	0.512
12706	0.355	0.470	0.508	0.345	0.379	0.508	0.050	−1.485	0.262
12707	0.408	0.807	0.513	0.512	1.453	0.053	0.361	0.504	0.296
12708	0.489	1.329	0.450	0.500	1.378	0.508	0.463	1.161	0.481
12709	0.505	1.430	0.508	0.459	1.114	0.491	0.466	1.178	0.431
12710	0.466	1.178	0.501	0.506	1.415	0.512	0.429	0.942	0.431
12711	0.497	1.380	0.508	0.506	1.415	0.501	0.508	1.447	0.431
12712	0.500	1.397	0.512	0.512	1.453	0.491	0.450	1.076	0.512
12713	0.500	1.397	0.512	0.485	1.283	0.273	0.384	0.655	0.513
12714	0.511	1.464	0.513	0.506	1.415	0.235	0.511	1.464	0.513
12715	0.497	1.380	0.501	0.497	1.359	0.508	0.489	1.329	0.262
12716	0.453	1.093	0.513	0.509	1.434	0.501	0.429	0.942	0.185
12717	0.492	1.346	0.142	0.480	1.246	0.508	0.511	1.464	0.097
12718	0.505	1.430	0.296	0.515	1.472	0.512	0.463	1.161	0.050
12719	0.097	−1.181	0.508	0.234	−0.336	0.512	0.263	−0.120	0.142
12720	0.500	1.397	0.512	0.491	1.321	0.512	0.408	0.807	0.501
12721	0.497	1.380	0.512	0.342	0.361	0.309	0.489	1.329	0.508
12722	0.500	1.397	0.508	0.509	1.434	0.309	0.468	1.194	0.467
12723	0.263	−0.120	0.385	0.053	−1.504	0.398	0.226	−0.356	0.467
12724	0.503	1.413	0.513	0.509	1.434	0.512	0.468	1.194	0.508
12725	0.508	1.447	0.513	0.491	1.321	0.193	0.432	0.958	0.358
12726	0.500	1.397	0.142	0.509	1.434	0.478	0.503	1.413	0.467
12727	0.468	1.194	0.431	0.494	1.340	0.512	0.495	1.363	0.262
12728	0.463	1.161	0.262	0.488	1.302	0.508	0.479	1.262	0.409
12729	0.100	−1.164	0.512	0.272	−0.091	0.508	0.224	−0.373	0.450
12730	0.261	−0.137	0.142	0.237	−0.317	0.508	0.224	−0.373	0.385
12731	0.511	1.464	0.050	0.509	1.434	0.491	0.505	1.430	0.185
12732	0.511	1.464	0.450	0.494	1.340	0.501	0.468	1.194	0.409
12733	0.511	1.464	0.501	0.500	1.378	0.508	0.513	1.481	0.501
12734	0.505	1.430	0.508	0.506	1.415	0.273	0.505	1.430	0.328
12735	0.466	1.178	0.481	0.509	1.434	0.053	0.497	1.380	0.467
12736	0.489	1.329	0.501	0.509	1.434	0.491	0.511	1.464	0.467
12737	0.100	−1.164	0.492	0.307	0.135	0.491	0.261	−0.137	0.508
12738	0.050	−1.485	0.501	0.310	0.153	0.508	0.361	0.504	0.501
12739	0.142	−0.895	0.492	0.401	0.737	0.508	0.224	−0.373	0.467
12740	0.500	1.397	0.512	0.509	1.434	0.512	0.429	0.942	0.492
12741	0.184	−0.625	0.262	0.196	−0.581	0.491	0.224	−0.373	0.467
12742	0.484	1.295	0.142	0.477	1.227	0.491	0.450	1.076	0.512
12743	0.503	1.413	0.185	0.520	1.509	0.309	0.503	1.413	0.385
12744	0.508	1.447	0.097	0.503	1.396	0.501	0.505	1.430	0.450
12745	0.495	1.363	0.185	0.503	1.396	0.508	0.503	1.413	0.450

12746	0.495	1.363	0.512	0.503	1.396	0.462	0.503	1.413	0.492
12747	0.508	1.447	0.512	0.488	1.302	0.371	0.513	1.481	0.185
12748	0.511	1.464	0.296	0.500	1.378	0.501	0.511	1.464	0.358
12749	0.497	1.380	0.501	0.509	1.434	0.508	0.508	1.447	0.467
12750	0.142	-0.895	0.512	0.275	-0.073	0.102	0.142	-0.895	0.431
12751	0.326	0.285	0.262	0.053	-1.504	0.102	0.266	-0.103	0.431
12752	0.466	1.178	0.409	0.488	1.302	0.341	0.505	1.430	0.481
12753	0.450	1.076	0.512	0.488	1.302	0.508	0.505	1.430	0.328
12754	0.511	1.464	0.142	0.509	1.434	0.508	0.471	1.211	0.481
12755	0.508	1.447	0.512	0.509	1.434	0.508	0.434	0.975	0.501
12756	0.508	1.447	0.501	0.512	1.453	0.508	0.508	1.447	0.501
12757	0.487	1.312	0.512	0.488	1.302	0.508	0.503	1.413	0.097
12758	0.487	1.312	0.513	0.488	1.302	0.501	0.503	1.413	0.512
12759	0.297	0.099	0.508	0.310	0.153	0.149	0.300	0.116	0.358
12760	0.471	1.211	0.508	0.500	1.378	0.422	0.484	1.295	0.508
12761	0.508	1.447	0.501	0.509	1.434	0.491	0.358	0.487	0.385
12762	0.503	1.413	0.501	0.462	1.133	0.309	0.513	1.481	0.142
12763	0.224	-0.373	0.358	0.374	0.568	0.273	0.000	?	0.467
12764	0.511	1.464	0.492	0.500	1.378	0.235	0.476	1.245	0.501
12765	0.426	0.925	0.481	0.503	1.396	0.235	0.511	1.464	0.508
12766	0.142	-0.895	0.501	0.102	-1.184	0.193	0.300	0.116	0.513
12767	0.184	-0.625	0.185	0.102	-1.184	0.149	0.300	0.116	0.501
12768	0.000	?	0.185	0.342	0.361	0.478	0.187	-0.608	0.501
12769	0.489	1.329	0.358	0.506	1.415	0.508	0.505	1.430	0.513
12770	0.476	1.245	0.358	0.506	1.415	0.235	0.505	1.430	0.512
12771	0.489	1.329	0.501	0.506	1.415	0.508	0.505	1.430	0.492
12772	0.476	1.245	0.431	0.506	1.415	0.273	0.505	1.430	0.328
12773	0.476	1.245	0.142	0.506	1.415	0.491	0.505	1.430	0.185
12774	0.500	1.397	0.481	0.497	1.359	0.512	0.495	1.363	0.262
12775	0.184	-0.625	0.513	0.149	-0.882	0.501	0.226	-0.356	0.296
12776	0.513	1.481	0.492	0.421	0.869	0.501	0.463	1.161	0.097
12777	0.508	1.447	0.508	0.488	1.302	0.501	0.489	1.329	0.513
12778	0.513	1.481	0.262	0.307	0.135	0.512	0.508	1.447	0.513
12779	0.097	-1.181	0.450	0.275	-0.073	0.491	0.261	-0.137	0.185
12780	0.050	-1.485	0.296	0.234	-0.336	0.508	0.361	0.504	0.512
12781	0.297	0.099	0.328	0.234	-0.336	0.501	0.297	0.099	0.501
12782	0.297	0.099	0.481	0.193	-0.600	0.491	0.297	0.099	0.050
12783	0.097	-1.181	0.481	0.149	-0.882	0.444	0.358	0.487	0.508
12784	0.503	1.413	0.224	0.480	1.246	0.309	0.505	1.430	0.431
12785	0.497	1.380	0.185	0.512	1.453	0.444	0.429	0.942	0.050
12786	0.097	-1.181	0.224	0.234	-0.336	0.462	0.332	0.318	0.508
12787	0.497	1.380	0.467	0.509	1.434	0.235	0.518	1.514	0.481
12788	0.097	-1.181	0.450	0.275	-0.073	0.478	0.263	-0.120	0.513
12789	0.500	1.397	0.262	0.488	1.302	0.273	0.505	1.430	0.512
12790	0.508	1.447	0.512	0.512	1.453	0.371	0.492	1.346	0.431
12791	0.505	1.430	0.185	0.491	1.321	0.491	0.513	1.481	0.431
12792	0.505	1.430	0.508	0.491	1.321	0.501	0.513	1.481	0.492
12793	0.463	1.161	0.185	0.509	1.434	0.193	0.516	1.498	0.501
12794	0.513	1.481	0.328	0.512	1.453	0.514	0.503	1.413	0.142
12795	0.484	1.295	0.262	0.491	1.321	0.491	0.508	1.447	0.492
12796	0.508	1.447	0.513	0.506	1.415	0.235	0.500	1.397	0.450
12797	0.489	1.329	0.142	0.500	1.378	0.235	0.500	1.397	0.431
12798	0.411	0.824	0.492	0.488	1.302	0.491	0.505	1.430	0.358
12799	0.492	1.346	0.508	0.444	1.020	0.235	0.503	1.413	0.358
12800	0.300	0.116	0.501	0.310	0.153	0.478	0.358	0.487	0.508
12801	0.476	1.245	0.481	0.450	1.057	0.462	0.489	1.329	0.508
12802	0.497	1.380	0.481	0.462	1.133	0.273	0.476	1.245	0.512
12803	0.184	-0.625	0.296	0.234	-0.336	0.462	0.263	-0.120	0.450
12804	0.492	1.346	0.512	0.474	1.208	0.341	0.492	1.346	0.262
12805	0.097	-1.181	0.513	0.275	-0.073	0.273	0.224	-0.373	0.512
12806	0.097	-1.181	0.296	0.371	0.549	0.273	0.355	0.470	0.481

12807	0.497	1.380	0.513	0.488	1.302	0.193	0.508	1.447	0.508
12808	0.487	1.312	0.501	0.500	1.378	0.508	0.508	1.447	0.512
12809	0.097	-1.181	0.508	0.193	-0.600	0.273	0.295	0.082	0.385
12810	0.500	1.397	0.431	0.512	1.453	0.478	0.503	1.413	0.409
12811	0.503	1.413	0.224	0.488	1.302	0.508	0.492	1.346	0.385
12812	0.097	-1.181	0.467	0.237	-0.317	0.512	0.224	-0.373	0.142
12813	0.184	-0.625	0.467	0.234	-0.336	0.512	0.184	-0.625	0.409
12814	0.508	1.447	0.508	0.488	1.302	0.512	0.508	1.447	0.409
12815	0.142	-0.895	0.501	0.234	-0.336	0.478	0.184	-0.625	0.224
12816	0.434	0.975	0.142	0.477	1.227	0.491	0.508	1.447	0.224
12817	0.513	1.481	0.512	0.462	1.133	0.512	0.511	1.464	0.142
12818	0.142	-0.895	0.501	0.275	-0.073	0.235	0.297	0.099	0.492
12819	0.513	1.481	0.262	0.456	1.095	0.193	0.503	1.413	0.467
12820	0.050	-1.485	0.185	0.342	0.361	0.309	0.142	-0.895	0.185
12821	0.226	-0.356	0.431	0.272	-0.091	0.309	0.050	-1.485	0.508
12822	0.261	-0.137	0.262	0.275	-0.073	0.102	0.226	-0.356	0.224
12823	0.184	-0.625	0.481	0.199	-0.562	0.478	0.184	-0.625	0.512
12824	0.511	1.464	0.501	0.506	1.415	0.398	0.505	1.430	0.262
12825	0.297	0.099	0.097	0.278	-0.054	0.501	0.261	-0.137	0.050
12826	0.500	1.397	0.409	0.477	1.227	0.512	0.295	0.082	0.262
12827	0.500	1.397	0.185	0.500	1.378	0.491	0.479	1.262	0.501
12828	0.489	1.329	0.501	0.512	1.453	0.102	0.434	0.975	0.185
12829	0.489	1.329	0.512	0.512	1.453	0.501	0.434	0.975	0.508
12830	0.489	1.329	0.328	0.512	1.453	0.398	0.434	0.975	0.512
12831	0.505	1.430	0.431	0.480	1.246	0.508	0.508	1.447	0.508
12832	0.505	1.430	0.492	0.491	1.321	0.508	0.511	1.464	0.481
12833	0.484	1.295	0.512	0.515	1.472	0.102	0.511	1.464	0.481
12834	0.297	0.099	0.450	0.234	-0.336	0.235	0.263	-0.120	0.185
12835	0.300	0.116	0.224	0.193	-0.600	0.512	0.184	-0.625	0.467
12836	0.224	-0.373	0.358	0.310	0.153	0.501	0.100	-1.164	0.467
12837	0.184	-0.625	0.431	0.310	0.153	0.273	0.050	-1.485	0.409
12838	0.297	0.099	0.508	0.102	-1.184	0.508	0.145	-0.878	0.224
12839	0.487	1.312	0.262	0.482	1.265	0.491	0.495	1.363	0.512
12840	0.487	1.312	0.450	0.401	0.737	0.398	0.500	1.397	0.450
12841	0.503	1.413	0.328	0.500	1.378	0.462	0.471	1.211	0.512
12842	0.492	1.346	0.185	0.512	1.453	0.422	0.474	1.228	0.467
12843	0.503	1.413	0.492	0.491	1.321	0.512	0.516	1.498	0.431
12844	0.224	-0.373	0.224	0.105	-1.165	0.512	0.361	0.504	0.492
12845	0.508	1.447	0.431	0.500	1.378	0.422	0.461	1.144	0.492
12846	0.142	-0.895	0.492	0.404	0.756	0.512	0.261	-0.137	0.508
12847	0.497	1.380	0.492	0.515	1.472	0.514	0.405	0.790	0.508
12848	0.500	1.397	0.450	0.515	1.472	0.501	0.447	1.060	0.328
12849	0.184	-0.625	0.513	0.102	-1.184	0.398	0.387	0.672	0.513
12850	0.184	-0.625	0.513	0.234	-0.336	0.309	0.184	-0.625	0.409
12851	0.513	1.481	0.492	0.512	1.453	0.235	0.408	0.807	0.185
12852	0.463	1.161	0.513	0.500	1.378	0.478	0.503	1.413	0.262
12853	0.142	-0.895	0.512	0.278	-0.054	0.512	0.329	0.301	0.512
12854	0.492	1.346	0.481	0.515	1.472	0.512	0.468	1.194	0.185
12855	0.500	1.397	0.501	0.494	1.340	0.491	0.466	1.178	0.409
12856	0.516	1.498	0.513	0.395	0.700	0.193	0.511	1.464	0.501
12857	0.450	1.076	0.508	0.462	1.133	0.514	0.497	1.380	0.508
12858	0.508	1.447	0.513	0.421	0.869	0.462	0.468	1.194	0.224
12859	0.500	1.397	0.185	0.512	1.453	0.478	0.492	1.346	0.262
12860	0.505	1.430	0.467	0.512	1.453	0.309	0.466	1.178	0.185
12861	0.500	1.397	0.512	0.427	0.907	0.508	0.511	1.464	0.185
12862	0.500	1.397	0.512	0.509	1.434	0.149	0.389	0.689	0.501
12863	0.500	1.397	0.512	0.512	1.453	0.444	0.455	1.110	0.450
12864	0.497	1.380	0.296	0.503	1.396	0.371	0.453	1.093	0.050
12865	0.513	1.481	0.492	0.395	0.700	0.341	0.489	1.329	0.508
12866	0.142	-0.895	0.328	0.310	0.153	0.512	0.184	-0.625	0.385
12867	0.300	0.116	0.409	0.234	-0.336	0.514	0.355	0.470	0.467

12868	0.497	1.380	0.512	0.480	1.246	0.514	0.466	1.178	0.512
12869	0.513	1.481	0.508	0.512	1.453	0.514	0.432	0.958	0.097
12870	0.513	1.481	0.513	0.512	1.453	0.491	0.432	0.958	0.185
12871	0.503	1.413	0.097	0.491	1.321	0.508	0.479	1.262	0.450
12872	0.000	?	0.431	0.193	-0.600	0.462	0.332	0.318	0.450
12873	0.384	0.655	0.481	0.512	1.453	0.422	0.482	1.279	0.185
12874	0.516	1.498	0.185	0.456	1.095	0.512	0.505	1.430	0.481
12875	0.513	1.481	0.512	0.471	1.189	0.371	0.503	1.413	0.262
12876	0.142	-0.895	0.512	0.310	0.153	0.273	0.097	-1.181	0.262
12877	0.429	0.942	0.185	0.509	1.434	0.102	0.508	1.447	0.508
12878	0.266	-0.103	0.296	0.149	-0.882	0.235	0.355	0.470	0.185
12879	0.511	1.464	0.431	0.447	1.039	0.273	0.508	1.447	0.142
12880	0.142	-0.895	0.481	0.368	0.530	0.478	0.389	0.689	0.508
12881	0.050	-1.485	0.508	0.339	0.342	0.478	0.142	-0.895	0.508
12882	0.445	1.043	0.328	0.509	1.434	0.193	0.466	1.178	0.501
12883	0.492	1.346	0.262	0.523	1.528	0.512	0.503	1.413	0.508
12884	0.505	1.430	0.185	0.512	1.453	0.444	0.503	1.413	0.508
12885	0.479	1.262	0.513	0.518	1.491	0.273	0.518	1.514	0.512
12886	0.497	1.380	0.508	0.494	1.340	0.462	0.508	1.447	0.492
12887	0.492	1.346	0.450	0.515	1.472	0.501	0.500	1.397	0.501
12888	0.497	1.380	0.358	0.465	1.152	0.398	0.513	1.481	0.513
12889	0.487	1.312	0.050	0.421	0.869	0.501	0.513	1.481	0.501
12890	0.511	1.464	0.142	0.515	1.472	0.462	0.495	1.363	0.508
12891	0.261	-0.137	0.513	0.371	0.549	0.491	0.329	0.301	0.492
12892	0.145	-0.878	0.512	0.275	-0.073	0.478	0.184	-0.625	0.513
12893	0.187	-0.608	0.508	0.105	-1.165	0.501	0.261	-0.137	0.185
12894	0.097	-1.181	0.512	0.234	-0.336	0.501	0.297	0.099	0.450
12895	0.187	-0.608	0.512	0.275	-0.073	0.512	0.097	-1.181	0.385
12896	0.508	1.447	0.185	0.471	1.189	0.512	0.516	1.498	0.358
12897	0.508	1.447	0.262	0.471	1.189	0.235	0.516	1.498	0.385
12898	0.300	0.116	0.185	0.193	-0.600	0.508	0.184	-0.625	0.142
12899	0.500	1.397	0.467	0.520	1.509	0.508	0.508	1.447	0.512
12900	0.511	1.464	0.358	0.447	1.039	0.491	0.503	1.413	0.296
12901	0.263	-0.120	0.508	0.272	-0.091	0.193	0.050	-1.485	0.431
12902	0.408	0.807	0.512	0.462	1.133	0.193	0.503	1.413	0.467
12903	0.500	1.397	0.512	0.506	1.415	0.514	0.429	0.942	0.512
12904	0.142	-0.895	0.512	0.401	0.737	0.508	0.050	-1.485	0.467
12905	0.513	1.481	0.513	0.503	1.396	0.491	0.511	1.464	0.296
12906	0.497	1.380	0.431	0.468	1.170	0.514	0.482	1.279	0.513
12907	0.508	1.447	0.224	0.497	1.359	0.235	0.513	1.481	0.512
12908	0.511	1.464	0.512	0.485	1.283	0.444	0.513	1.481	0.097
12909	0.505	1.430	0.492	0.494	1.340	0.462	0.429	0.942	0.431
12910	0.505	1.430	0.050	0.494	1.340	0.491	0.429	0.942	0.512
12911	0.503	1.413	0.512	0.509	1.434	0.501	0.492	1.346	0.328
12912	0.503	1.413	0.492	0.509	1.434	0.309	0.500	1.397	0.224
12913	0.358	0.487	0.296	0.234	-0.336	0.508	0.142	-0.895	0.512
12914	0.495	1.363	0.385	0.506	1.415	0.341	0.487	1.312	0.501
12915	0.479	1.262	0.296	0.509	1.434	0.149	0.450	1.076	0.512
12916	0.503	1.413	0.481	0.488	1.302	0.508	0.434	0.975	0.050
12917	0.184	-0.625	0.050	0.193	-0.600	0.508	0.363	0.520	0.185
12918	0.184	-0.625	0.142	0.193	-0.600	0.149	0.363	0.520	0.224
12919	0.358	0.487	0.467	0.520	1.509	0.235	0.505	1.430	0.481
12920	0.358	0.487	0.467	0.515	1.472	0.193	0.505	1.430	0.385
12921	0.497	1.380	0.481	0.491	1.321	0.462	0.513	1.481	0.501
12922	0.429	0.942	0.185	0.518	1.491	0.512	0.453	1.093	0.224
12923	0.142	-0.895	0.501	0.237	-0.317	0.149	0.263	-0.120	0.262
12924	0.476	1.245	0.508	0.442	1.001	0.501	0.513	1.481	0.481
12925	0.503	1.413	0.358	0.465	1.152	0.235	0.479	1.262	0.481
12926	0.489	1.329	0.508	0.488	1.302	0.478	0.511	1.464	0.501
12927	0.508	1.447	0.328	0.503	1.396	0.422	0.511	1.464	0.492
12928	0.261	-0.137	0.328	0.310	0.153	0.235	0.387	0.672	0.512

12929	0.453	1.093	0.328	0.509	1.434	0.235	0.411	0.824	0.385
12930	0.295	0.082	0.296	0.345	0.379	0.501	0.389	0.689	0.262
12931	0.329	0.301	0.224	0.149	-0.882	0.235	0.142	-0.895	0.224
12932	0.484	1.295	0.224	0.506	1.415	0.371	0.408	0.807	0.501
12933	0.484	1.295	0.142	0.506	1.415	0.501	0.408	0.807	0.142
12934	0.224	-0.373	0.512	0.149	-0.882	0.422	0.226	-0.356	0.508
12935	0.184	-0.625	0.501	0.234	-0.336	0.512	0.226	-0.356	0.450
12936	0.226	-0.356	0.142	0.193	-0.600	0.514	0.142	-0.895	0.409
12937	0.468	1.194	0.467	0.465	1.152	0.102	0.487	1.312	0.481
12938	0.450	1.076	0.142	0.518	1.491	0.512	0.461	1.144	0.409
12939	0.261	-0.137	0.142	0.149	-0.882	0.512	0.184	-0.625	0.492
12940	0.503	1.413	0.512	0.503	1.396	0.149	0.505	1.430	0.224
12941	0.187	-0.608	0.296	0.237	-0.317	0.235	0.224	-0.373	0.224
12942	0.508	1.447	0.512	0.480	1.246	0.398	0.505	1.430	0.508
12943	0.187	-0.608	0.512	0.424	0.888	0.501	0.261	-0.137	0.512
12944	0.326	0.285	0.501	0.237	-0.317	0.422	0.050	-1.485	0.328
12945	0.261	-0.137	0.501	0.237	-0.317	0.501	0.261	-0.137	0.296
12946	0.513	1.481	0.358	0.497	1.359	0.235	0.497	1.380	0.467
12947	0.142	-0.895	0.501	0.234	-0.336	0.444	0.184	-0.625	0.492
12948	0.489	1.329	0.409	0.374	0.568	0.444	0.503	1.413	0.097
12949	0.508	1.447	0.142	0.503	1.396	0.501	0.513	1.481	0.097
12950	0.503	1.413	0.097	0.421	0.869	0.501	0.505	1.430	0.097
12951	0.479	1.262	0.142	0.515	1.472	0.053	0.479	1.262	0.492
12952	0.479	1.262	0.481	0.518	1.491	0.398	0.479	1.262	0.328
12953	0.295	0.082	0.501	0.102	-1.184	0.478	0.184	-0.625	0.185
12954	0.505	1.430	0.501	0.512	1.453	0.371	0.466	1.178	0.508
12955	0.508	1.447	0.467	0.512	1.453	0.422	0.466	1.178	0.501
12956	0.000	?	0.262	0.149	-0.882	0.478	0.408	0.807	0.508
12957	0.300	0.116	0.262	0.234	-0.336	0.102	0.226	-0.356	0.185
12958	0.511	1.464	0.409	0.395	0.700	0.102	0.511	1.464	0.508
12959	0.495	1.363	0.224	0.500	1.378	0.501	0.453	1.093	0.492
12960	0.505	1.430	0.508	0.421	0.869	0.478	0.511	1.464	0.512
12961	0.426	0.925	0.501	0.500	1.378	0.273	0.468	1.194	0.492
12962	0.226	-0.356	0.142	0.234	-0.336	0.273	0.432	0.958	0.050
12963	0.471	1.211	0.224	0.439	0.982	0.371	0.489	1.329	0.050
12964	0.471	1.211	0.050	0.439	0.982	0.193	0.489	1.329	0.050
12965	0.508	1.447	0.097	0.497	1.359	0.371	0.511	1.464	0.097
12966	0.500	1.397	0.513	0.494	1.340	0.514	0.508	1.447	0.097
12967	0.142	-0.895	0.185	0.053	-1.504	0.193	0.326	0.285	0.050
12968	0.511	1.464	0.409	0.401	0.737	0.444	0.518	1.514	0.409
12969	0.495	1.363	0.431	0.482	1.265	0.444	0.408	0.807	0.513
12970	0.261	-0.137	0.328	0.371	0.549	0.512	0.184	-0.625	0.467
12971	0.184	-0.625	0.467	0.427	0.907	0.512	0.261	-0.137	0.097
12972	0.429	0.942	0.467	0.477	1.227	0.309	0.505	1.430	0.409
12973	0.266	-0.103	0.224	0.102	-1.184	0.501	0.184	-0.625	0.296
12974	0.000	?	0.358	0.102	-1.184	0.444	0.411	0.824	0.296
12975	0.487	1.312	0.431	0.503	1.396	0.235	0.497	1.380	0.385
12976	0.492	1.346	0.296	0.477	1.227	0.508	0.511	1.464	0.097
12977	0.097	-1.181	0.492	0.275	-0.073	0.309	0.226	-0.356	0.467
12978	0.000	?	0.512	0.278	-0.054	0.193	0.261	-0.137	0.467
12979	0.408	0.807	0.142	0.368	0.530	0.444	0.187	-0.608	0.467
12980	0.187	-0.608	0.513	0.196	-0.581	0.149	0.184	-0.625	0.492
12981	0.503	1.413	0.224	0.371	0.549	0.273	0.500	1.397	0.513
12982	0.511	1.464	0.492	0.515	1.472	0.478	0.450	1.076	0.501
12983	0.329	0.301	0.467	0.193	-0.600	0.398	0.050	-1.485	0.492
12984	0.434	0.975	0.296	0.447	1.039	0.462	0.505	1.430	0.185
12985	0.492	1.346	0.492	0.447	1.039	0.444	0.382	0.638	0.385
12986	0.511	1.464	0.050	0.512	1.453	0.444	0.466	1.178	0.358
12987	0.450	1.076	0.481	0.506	1.415	0.462	0.511	1.464	0.481
12988	0.226	-0.356	0.508	0.310	0.153	0.462	0.097	-1.181	0.508
12989	0.358	0.487	0.224	0.000	?	0.512	0.184	-0.625	0.501

12990	0.432	0.958	0.142	0.503	1.396	0.514	0.450	1.076	0.431
12991	0.513	1.481	0.492	0.444	1.020	0.501	0.453	1.093	0.224
12992	0.261	−0.137	0.097	0.234	−0.336	0.491	0.184	−0.625	0.358
12993	0.445	1.043	0.512	0.506	1.415	0.491	0.484	1.295	0.513
12994	0.326	0.285	0.467	0.307	0.135	0.501	0.263	−0.120	0.142
12995	0.184	−0.625	0.224	0.193	−0.600	0.235	0.261	−0.137	0.481
12996	0.495	1.363	0.097	0.442	1.001	0.462	0.503	1.413	0.481
12997	0.226	−0.356	0.513	0.149	−0.882	0.501	0.184	−0.625	0.224
12998	0.437	0.992	0.224	0.272	−0.091	0.501	0.142	−0.895	0.142
12999	0.495	1.363	0.296	0.480	1.246	0.501	0.505	1.430	0.262
13000	0.487	1.312	0.431	0.401	0.737	0.193	0.508	1.447	0.262
13001	0.447	1.060	0.467	0.465	1.152	0.512	0.503	1.413	0.492
13002	0.511	1.464	0.492	0.442	1.001	0.309	0.505	1.430	0.328
13003	0.511	1.464	0.508	0.442	1.001	0.444	0.505	1.430	0.513
13004	0.487	1.312	0.508	0.468	1.170	0.501	0.513	1.481	0.508
13005	0.511	1.464	0.501	0.465	1.152	0.508	0.495	1.363	0.358
13006	0.505	1.430	0.492	0.509	1.434	0.501	0.505	1.430	0.492
13007	0.479	1.262	0.481	0.512	1.453	0.235	0.516	1.498	0.492
13008	0.497	1.380	0.492	0.500	1.378	0.501	0.497	1.380	0.224
13009	0.516	1.498	0.467	0.488	1.302	0.508	0.505	1.430	0.358
13010	0.511	1.464	0.296	0.488	1.302	0.273	0.489	1.329	0.508
13011	0.508	1.447	0.224	0.497	1.359	0.478	0.513	1.481	0.508
13012	0.184	−0.625	0.481	0.234	−0.336	0.444	0.184	−0.625	0.262
13013	0.474	1.228	0.481	0.468	1.170	0.341	0.445	1.043	0.385
13014	0.505	1.430	0.296	0.500	1.378	0.235	0.382	0.638	0.512
13015	0.505	1.430	0.385	0.500	1.378	0.444	0.355	0.470	0.296
13016	0.505	1.430	0.508	0.500	1.378	0.514	0.382	0.638	0.508
13017	0.295	0.082	0.224	0.193	−0.600	0.444	0.142	−0.895	0.050
13018	0.487	1.312	0.097	0.509	1.434	0.235	0.511	1.464	0.508
13019	0.326	0.285	0.185	0.310	0.153	0.235	0.295	0.082	0.481
13020	0.405	0.790	0.224	0.444	1.020	0.193	0.432	0.958	0.262
13021	0.516	1.498	0.467	0.503	1.396	0.501	0.468	1.194	0.513
13022	0.508	1.447	0.508	0.512	1.453	0.512	0.511	1.464	0.296
13023	0.508	1.447	0.492	0.500	1.378	0.462	0.466	1.178	0.481
13024	0.097	−1.181	0.501	0.234	−0.336	0.273	0.297	0.099	0.262
13025	0.429	0.942	0.481	0.500	1.378	0.273	0.516	1.498	0.513
13026	0.479	1.262	0.481	0.509	1.434	0.149	0.516	1.498	0.185
13027	0.184	−0.625	0.296	0.275	−0.073	0.491	0.100	−1.164	0.097
13028	0.508	1.447	0.512	0.482	1.265	0.422	0.432	0.958	0.508
13029	0.500	1.397	0.481	0.447	1.039	0.508	0.513	1.481	0.328
13030	0.184	−0.625	0.508	0.339	0.342	0.422	0.326	0.285	0.501
13031	0.300	0.116	0.481	0.237	−0.317	0.422	0.224	−0.373	0.508
13032	0.429	0.942	0.508	0.447	1.039	0.053	0.513	1.481	0.142
13033	0.482	1.279	0.512	0.518	1.491	0.193	0.495	1.363	0.328
13034	0.511	1.464	0.385	0.439	0.982	0.235	0.505	1.430	0.492
13035	0.326	0.285	0.508	0.234	−0.336	0.478	0.050	−1.485	0.097
13036	0.266	−0.103	0.328	0.234	−0.336	0.235	0.184	−0.625	0.142
13037	0.187	−0.608	0.501	0.193	−0.600	0.501	0.224	−0.373	0.409
13038	0.513	1.481	0.501	0.500	1.378	0.491	0.482	1.279	0.467
13039	0.505	1.430	0.097	0.509	1.434	0.512	0.387	0.672	0.512
13040	0.450	1.076	0.481	0.462	1.133	0.508	0.500	1.397	0.512
13041	0.358	0.487	0.513	0.275	−0.073	0.501	0.000	?	0.513
13042	0.050	−1.485	0.492	0.275	−0.073	0.508	0.224	−0.373	0.501
13043	0.142	−0.895	0.492	0.149	−0.882	0.102	0.261	−0.137	0.481
13044	0.511	1.464	0.296	0.491	1.321	0.341	0.476	1.245	0.492
13045	0.511	1.464	0.508	0.424	0.888	0.478	0.479	1.262	0.481
13046	0.505	1.430	0.262	0.506	1.415	0.478	0.497	1.380	0.481
13047	0.505	1.430	0.450	0.427	0.907	0.341	0.492	1.346	0.262
13048	0.505	1.430	0.512	0.427	0.907	0.273	0.513	1.481	0.097
13049	0.184	−0.625	0.492	0.053	−1.504	0.501	0.384	0.655	0.512
13050	0.261	−0.137	0.492	0.196	−0.581	0.501	0.261	−0.137	0.512

13051	0.184	-0.625	0.431	0.234	-0.336	0.309	0.226	-0.356	0.097
13052	0.461	1.144	0.296	0.477	1.227	0.371	0.497	1.380	0.512
13053	0.363	0.520	0.224	0.234	-0.336	0.341	0.142	-0.895	0.431
13054	0.508	1.447	0.450	0.500	1.378	0.501	0.505	1.430	0.185
13055	0.508	1.447	0.185	0.491	1.321	0.462	0.450	1.076	0.296
13056	0.511	1.464	0.262	0.509	1.434	0.273	0.408	0.807	0.142
13057	0.511	1.464	0.481	0.503	1.396	0.512	0.476	1.245	0.262
13058	0.511	1.464	0.409	0.500	1.378	0.512	0.405	0.790	0.097
13059	0.429	0.942	0.481	0.512	1.453	0.501	0.495	1.363	0.501
13060	0.224	-0.373	0.481	0.102	-1.184	0.235	0.224	-0.373	0.450
13061	0.000	?	0.492	0.339	0.342	0.508	0.224	-0.373	0.450
13062	0.500	1.397	0.481	0.482	1.265	0.478	0.508	1.447	0.492
13063	0.489	1.329	0.492	0.482	1.265	0.501	0.505	1.430	0.513
13064	0.000	?	0.501	0.339	0.342	0.444	0.332	0.318	0.481
13065	0.050	-1.485	0.513	0.272	-0.091	0.341	0.300	0.116	0.328
13066	0.511	1.464	0.467	0.497	1.359	0.309	0.461	1.144	0.508
13067	0.492	1.346	0.492	0.500	1.378	0.309	0.492	1.346	0.508
13068	0.297	0.099	0.224	0.313	0.172	0.193	0.097	-1.181	0.492
13069	0.387	0.672	0.492	0.374	0.568	0.273	0.097	-1.181	0.508
13070	0.297	0.099	0.185	0.345	0.379	0.273	0.097	-1.181	0.450
13071	0.479	1.262	0.492	0.503	1.396	0.309	0.492	1.346	0.467
13072	0.050	-1.485	0.492	0.462	1.133	0.491	0.329	0.301	0.224
13073	0.145	-0.878	0.481	0.272	-0.091	0.501	0.184	-0.625	0.513
13074	0.466	1.178	0.513	0.503	1.396	0.422	0.508	1.447	0.185
13075	0.466	1.178	0.501	0.503	1.396	0.478	0.503	1.413	0.492
13076	0.476	1.245	0.492	0.497	1.359	0.102	0.508	1.447	0.492
13077	0.187	-0.608	0.492	0.234	-0.336	0.102	0.187	-0.608	0.142
13078	0.497	1.380	0.492	0.509	1.434	0.512	0.508	1.447	0.508
13079	0.497	1.380	0.512	0.477	1.227	0.149	0.495	1.363	0.501
13080	0.355	0.470	0.467	0.500	1.378	0.508	0.511	1.464	0.508
13081	0.497	1.380	0.467	0.450	1.057	0.508	0.495	1.363	0.508
13082	0.332	0.318	0.501	0.342	0.361	0.508	0.050	-1.485	0.481
13083	0.332	0.318	0.501	0.307	0.135	0.371	0.050	-1.485	0.358
13084	0.332	0.318	0.512	0.307	0.135	0.512	0.050	-1.485	0.385
13085	0.295	0.082	0.328	0.193	-0.600	0.508	0.097	-1.181	0.450
13086	0.224	-0.373	0.185	0.272	-0.091	0.512	0.097	-1.181	0.492
13087	0.224	-0.373	0.358	0.272	-0.091	0.273	0.050	-1.485	0.481
13088	0.142	-0.895	0.185	0.310	0.153	0.053	0.413	0.841	0.481
13089	0.503	1.413	0.296	0.488	1.302	0.371	0.516	1.498	0.508
13090	0.495	1.363	0.431	0.500	1.378	0.512	0.466	1.178	0.262
13091	0.142	-0.895	0.481	0.421	0.869	0.512	0.097	-1.181	0.358
13092	0.468	1.194	0.513	0.482	1.265	0.462	0.411	0.824	0.508
13093	0.142	-0.895	0.501	0.105	-1.165	0.512	0.297	0.099	0.296
13094	0.142	-0.895	0.185	0.105	-1.165	0.235	0.297	0.099	0.142
13095	0.503	1.413	0.450	0.515	1.472	0.273	0.382	0.638	0.492
13096	0.300	0.116	0.450	0.149	-0.882	0.478	0.097	-1.181	0.492
13097	0.513	1.481	0.512	0.503	1.396	0.309	0.461	1.144	0.508
13098	0.513	1.481	0.492	0.503	1.396	0.512	0.461	1.144	0.513
13099	0.503	1.413	0.508	0.503	1.396	0.501	0.461	1.144	0.512
13100	0.503	1.413	0.481	0.371	0.549	0.235	0.492	1.346	0.501
13101	0.355	0.470	0.512	0.509	1.434	0.193	0.511	1.464	0.512
13102	0.495	1.363	0.501	0.509	1.434	0.341	0.497	1.380	0.481
13103	0.411	0.824	0.508	0.515	1.472	0.398	0.492	1.346	0.467
13104	0.142	-0.895	0.512	0.275	-0.073	0.478	0.187	-0.608	0.467
13105	0.097	-1.181	0.508	0.053	-1.504	0.273	0.387	0.672	0.481
13106	0.142	-0.895	0.512	0.371	0.549	0.462	0.363	0.520	0.296
13107	0.476	1.245	0.262	0.512	1.453	0.501	0.479	1.262	0.512
13108	0.497	1.380	0.262	0.515	1.472	0.193	0.508	1.447	0.050
13109	0.497	1.380	0.185	0.462	1.133	0.478	0.503	1.413	0.513
13110	0.463	1.161	0.450	0.512	1.453	0.478	0.429	0.942	0.512
13111	0.263	-0.120	0.450	0.237	-0.317	0.193	0.226	-0.356	0.508

13112	0.261	-0.137	0.492	0.272	-0.091	0.235	0.363	0.520	0.431
13113	0.405	0.790	0.492	0.482	1.265	0.491	0.511	1.464	0.492
13114	0.224	-0.373	0.296	0.307	0.135	0.398	0.142	-0.895	0.508
13115	0.503	1.413	0.492	0.512	1.453	0.341	0.479	1.262	0.508
13116	0.497	1.380	0.508	0.503	1.396	0.512	0.476	1.245	0.501
13117	0.145	-0.878	0.513	0.234	-0.336	0.501	0.224	-0.373	0.481
13118	0.226	-0.356	0.296	0.193	-0.600	0.398	0.142	-0.895	0.513
13119	0.050	-1.485	0.142	0.339	0.342	0.512	0.261	-0.137	0.513
13120	0.097	-1.181	0.431	0.401	0.737	0.341	0.261	-0.137	0.481
13121	0.503	1.413	0.450	0.482	1.265	0.501	0.489	1.329	0.450
13122	0.184	-0.625	0.467	0.275	-0.073	0.462	0.332	0.318	0.467
13123	0.411	0.824	0.492	0.468	1.170	0.149	0.513	1.481	0.185
13124	0.432	0.958	0.450	0.500	1.378	0.398	0.508	1.447	0.050
13125	0.332	0.318	0.224	0.193	-0.600	0.273	0.358	0.487	0.185
13126	0.466	1.178	0.296	0.480	1.246	0.491	0.492	1.346	0.050
13127	0.466	1.178	0.409	0.480	1.246	0.273	0.492	1.346	0.508
13128	0.226	-0.356	0.385	0.193	-0.600	0.508	0.224	-0.373	0.481
13129	0.000	?	0.492	0.234	-0.336	0.273	0.361	0.504	0.492
13130	0.355	0.470	0.513	0.485	1.283	0.341	0.508	1.447	0.501
13131	0.432	0.958	0.185	0.398	0.718	0.501	0.511	1.464	0.358
13132	0.297	0.099	0.512	0.342	0.361	0.149	0.266	-0.103	0.492
13133	0.492	1.346	0.512	0.509	1.434	0.512	0.387	0.672	0.492
13134	0.511	1.464	0.501	0.500	1.378	0.478	0.518	1.514	0.512
13135	0.142	-0.895	0.512	0.401	0.737	0.193	0.297	0.099	0.450
13136	0.500	1.397	0.512	0.518	1.491	0.309	0.511	1.464	0.512
13137	0.224	-0.373	0.513	0.342	0.361	0.462	0.050	-1.485	0.501
13138	0.495	1.363	0.512	0.503	1.396	0.235	0.508	1.447	0.481
13139	0.471	1.211	0.467	0.462	1.133	0.273	0.479	1.262	0.492
13140	0.297	0.099	0.142	0.149	-0.882	0.508	0.263	-0.120	0.501
13141	0.492	1.346	0.328	0.398	0.718	0.491	0.511	1.464	0.501
13142	0.050	-1.485	0.328	0.272	-0.091	0.478	0.300	0.116	0.501
13143	0.476	1.245	0.262	0.494	1.340	0.462	0.476	1.245	0.492
13144	0.000	?	0.450	0.275	-0.073	0.462	0.263	-0.120	0.262
13145	0.508	1.447	0.513	0.500	1.378	0.491	0.511	1.464	0.296
13146	0.224	-0.373	0.513	0.275	-0.073	0.501	0.187	-0.608	0.431
13147	0.142	-0.895	0.508	0.345	0.379	0.501	0.097	-1.181	0.501
13148	0.489	1.329	0.501	0.494	1.340	0.512	0.511	1.464	0.501
13149	0.097	-1.181	0.501	0.152	-0.863	0.508	0.329	0.301	0.467
13150	0.513	1.481	0.050	0.509	1.434	0.149	0.495	1.363	0.467
13151	0.466	1.178	0.513	0.480	1.246	0.309	0.500	1.397	0.097
13152	0.226	-0.356	0.508	0.196	-0.581	0.512	0.142	-0.895	0.513
13153	0.097	-1.181	0.224	0.310	0.153	0.512	0.329	0.301	0.492
13154	0.513	1.481	0.492	0.462	1.133	0.149	0.495	1.363	0.501
13155	0.224	-0.373	0.481	0.237	-0.317	0.491	0.097	-1.181	0.224
13156	0.295	0.082	0.501	0.275	-0.073	0.501	0.142	-0.895	0.358
13157	0.426	0.925	0.097	0.512	1.453	0.193	0.405	0.790	0.512
13158	0.471	1.211	0.508	0.491	1.321	0.422	0.466	1.178	0.492
13159	0.487	1.312	0.385	0.477	1.227	0.444	0.508	1.447	0.508
13160	0.505	1.430	0.508	0.462	1.133	0.193	0.508	1.447	0.224
13161	0.503	1.413	0.481	0.468	1.170	0.235	0.513	1.481	0.501
13162	0.497	1.380	0.142	0.497	1.359	0.444	0.500	1.397	0.512
13163	0.487	1.312	0.262	0.500	1.378	0.491	0.479	1.262	0.296
13164	0.476	1.245	0.328	0.500	1.378	0.462	0.489	1.329	0.097
13165	0.489	1.329	0.358	0.509	1.434	0.478	0.479	1.262	0.512
13166	0.466	1.178	0.296	0.506	1.415	0.512	0.476	1.245	0.296
13167	0.297	0.099	0.296	0.152	-0.863	0.444	0.261	-0.137	0.512
13168	0.224	-0.373	0.296	0.310	0.153	0.235	0.097	-1.181	0.467
13169	0.482	1.279	0.450	0.509	1.434	0.508	0.511	1.464	0.185
13170	0.482	1.279	0.467	0.509	1.434	0.514	0.511	1.464	0.513
13171	0.295	0.082	0.508	0.152	-0.863	0.501	0.100	-1.164	0.513
13172	0.387	0.672	0.142	0.491	1.321	0.508	0.508	1.447	0.512

13173	0.497	1.380	0.492	0.503	1.396	0.512	0.429	0.942	0.467
13174	0.224	-0.373	0.328	0.199	-0.562	0.491	0.184	-0.625	0.467
13175	0.100	-1.164	0.450	0.421	0.869	0.273	0.295	0.082	0.467
13176	0.000	?	0.328	0.439	0.982	0.508	0.142	-0.895	0.501
13177	0.184	-0.625	0.501	0.193	-0.600	0.309	0.263	-0.120	0.512
13178	0.226	-0.356	0.492	0.234	-0.336	0.501	0.097	-1.181	0.385
13179	0.461	1.144	0.262	0.444	1.020	0.512	0.503	1.413	0.385
13180	0.500	1.397	0.450	0.491	1.321	0.309	0.447	1.060	0.050
13181	0.487	1.312	0.512	0.465	1.152	0.508	0.450	1.076	0.142
13182	0.495	1.363	0.431	0.485	1.283	0.512	0.492	1.346	0.501
13183	0.476	1.245	0.185	0.509	1.434	0.462	0.511	1.464	0.481
13184	0.479	1.262	0.508	0.444	1.020	0.462	0.482	1.279	0.492
13185	0.297	0.099	0.224	0.237	-0.317	0.273	0.329	0.301	0.501
13186	0.500	1.397	0.224	0.512	1.453	0.512	0.508	1.447	0.467
13187	0.479	1.262	0.185	0.512	1.453	0.273	0.505	1.430	0.481
13188	0.505	1.430	0.508	0.500	1.378	0.501	0.495	1.363	0.296
13189	0.479	1.262	0.492	0.506	1.415	0.501	0.508	1.447	0.501
13190	0.508	1.447	0.508	0.518	1.491	0.444	0.450	1.076	0.385
13191	0.508	1.447	0.262	0.488	1.302	0.478	0.463	1.161	0.262
13192	0.389	0.689	0.409	0.275	-0.073	0.444	0.224	-0.373	0.501
13193	0.508	1.447	0.142	0.506	1.415	0.193	0.511	1.464	0.512
13194	0.329	0.301	0.513	0.310	0.153	0.371	0.187	-0.608	0.513
13195	0.503	1.413	0.492	0.497	1.359	0.478	0.489	1.329	0.296
13196	0.503	1.413	0.385	0.509	1.434	0.341	0.489	1.329	0.508
13197	0.097	-1.181	0.142	0.313	0.172	0.273	0.142	-0.895	0.142
13198	0.484	1.295	0.328	0.509	1.434	0.478	0.511	1.464	0.431
13199	0.511	1.464	0.431	0.509	1.434	0.508	0.500	1.397	0.512
13200	0.487	1.312	0.501	0.462	1.133	0.508	0.508	1.447	0.296
13201	0.487	1.312	0.492	0.462	1.133	0.501	0.508	1.447	0.328
13202	0.297	0.099	0.224	0.272	-0.091	0.514	0.484	1.295	0.262
13203	0.511	1.464	0.224	0.515	1.472	0.512	0.355	0.470	0.508
13204	0.263	-0.120	0.142	0.278	-0.054	0.514	0.384	0.655	0.185
13205	0.450	1.076	0.431	0.500	1.378	0.501	0.445	1.043	0.185
13206	0.505	1.430	0.512	0.506	1.415	0.462	0.489	1.329	0.185
13207	0.495	1.363	0.224	0.444	1.020	0.462	0.482	1.279	0.512
13208	0.495	1.363	0.501	0.480	1.246	0.508	0.482	1.279	0.512
13209	0.432	0.958	0.492	0.444	1.020	0.273	0.511	1.464	0.467
13210	0.297	0.099	0.296	0.193	-0.600	0.514	0.266	-0.103	0.224
13211	0.224	-0.373	0.481	0.374	0.568	0.341	0.361	0.504	0.501
13212	0.450	1.076	0.501	0.477	1.227	0.508	0.505	1.430	0.097
13213	0.184	-0.625	0.492	0.342	0.361	0.508	0.295	0.082	0.501
13214	0.263	-0.120	0.142	0.275	-0.073	0.514	0.142	-0.895	0.185
13215	0.476	1.245	0.328	0.477	1.227	0.491	0.495	1.363	0.467
13216	0.408	0.807	0.385	0.509	1.434	0.508	0.484	1.295	0.450
13217	0.476	1.245	0.513	0.509	1.434	0.514	0.503	1.413	0.142
13218	0.484	1.295	0.513	0.497	1.359	0.514	0.508	1.447	0.467
13219	0.495	1.363	0.513	0.515	1.472	0.512	0.508	1.447	0.513
13220	0.479	1.262	0.358	0.509	1.434	0.444	0.500	1.397	0.512
13221	0.492	1.346	0.431	0.512	1.453	0.501	0.513	1.481	0.142
13222	0.492	1.346	0.224	0.500	1.378	0.501	0.487	1.312	0.513
13223	0.505	1.430	0.512	0.465	1.152	0.491	0.463	1.161	0.262
13224	0.463	1.161	0.328	0.465	1.152	0.491	0.466	1.178	0.262
13225	0.489	1.329	0.328	0.512	1.453	0.508	0.482	1.279	0.185
13226	0.224	-0.373	0.481	0.275	-0.073	0.341	0.295	0.082	0.512
13227	0.487	1.312	0.492	0.515	1.472	0.309	0.513	1.481	0.492
13228	0.187	-0.608	0.513	0.342	0.361	0.341	0.050	-1.485	0.328
13229	0.484	1.295	0.492	0.515	1.472	0.309	0.518	1.514	0.450
13230	0.484	1.295	0.492	0.515	1.472	0.273	0.516	1.498	0.431
13231	0.474	1.228	0.467	0.520	1.509	0.508	0.513	1.481	0.450
13232	0.511	1.464	0.142	0.497	1.359	0.501	0.429	0.942	0.358
13233	0.500	1.397	0.296	0.515	1.472	0.514	0.492	1.346	0.513

13234	0.495	1.363	0.296	0.515	1.472	0.501	0.503	1.413	0.501
13235	0.495	1.363	0.296	0.515	1.472	0.398	0.503	1.413	0.467
13236	0.489	1.329	0.185	0.512	1.453	0.512	0.497	1.380	0.296
13237	0.513	1.481	0.224	0.442	1.001	0.508	0.476	1.245	0.385
13238	0.461	1.144	0.501	0.500	1.378	0.508	0.513	1.481	0.512
13239	0.461	1.144	0.501	0.500	1.378	0.512	0.513	1.481	0.492
13240	0.503	1.413	0.224	0.494	1.340	0.514	0.487	1.312	0.501
13241	0.503	1.413	0.501	0.494	1.340	0.478	0.455	1.110	0.142
13242	0.513	1.481	0.142	0.509	1.434	0.478	0.463	1.161	0.185
13243	0.332	0.318	0.224	0.342	0.361	0.501	0.184	-0.625	0.296
13244	0.184	-0.625	0.508	0.313	0.172	0.514	0.050	-1.485	0.512
13245	0.361	0.504	0.296	0.342	0.361	0.514	0.184	-0.625	0.501
13246	0.184	-0.625	0.296	0.313	0.172	0.514	0.050	-1.485	0.512
13247	0.300	0.116	0.492	0.272	-0.091	0.491	0.000	?	0.512
13248	0.429	0.942	0.224	0.509	1.434	0.235	0.503	1.413	0.296
13249	0.476	1.245	0.185	0.500	1.378	0.235	0.487	1.312	0.492
13250	0.511	1.464	0.224	0.512	1.453	0.235	0.492	1.346	0.450
13251	0.495	1.363	0.142	0.503	1.396	0.491	0.500	1.397	0.450
13252	0.187	-0.608	0.513	0.401	0.737	0.491	0.355	0.470	0.142
13253	0.445	1.043	0.185	0.515	1.472	0.514	0.492	1.346	0.409
13254	0.445	1.043	0.467	0.509	1.434	0.514	0.489	1.329	0.185
13255	0.511	1.464	0.224	0.509	1.434	0.149	0.508	1.447	0.508
13256	0.495	1.363	0.409	0.512	1.453	0.491	0.447	1.060	0.512
13257	0.508	1.447	0.501	0.520	1.509	0.501	0.508	1.447	0.508
13258	0.482	1.279	0.501	0.485	1.283	0.478	0.500	1.397	0.512
13259	0.508	1.447	0.508	0.477	1.227	0.309	0.476	1.245	0.450
13260	0.505	1.430	0.467	0.503	1.396	0.235	0.492	1.346	0.097
13261	0.511	1.464	0.492	0.515	1.472	0.512	0.503	1.413	0.512
13262	0.516	1.498	0.492	0.515	1.472	0.501	0.503	1.413	0.142
13263	0.511	1.464	0.224	0.515	1.472	0.501	0.503	1.413	0.142
13264	0.516	1.498	0.467	0.494	1.340	0.309	0.492	1.346	0.409
13265	0.266	-0.103	0.492	0.234	-0.336	0.512	0.263	-0.120	0.492
13266	0.266	-0.103	0.501	0.234	-0.336	0.398	0.297	0.099	0.501
13267	0.184	-0.625	0.385	0.237	-0.317	0.193	0.429	0.942	0.512
13268	0.450	1.076	0.097	0.497	1.359	0.309	0.505	1.430	0.512
13269	0.450	1.076	0.262	0.497	1.359	0.508	0.505	1.430	0.450
13270	0.489	1.329	0.409	0.512	1.453	0.371	0.466	1.178	0.296
13271	0.489	1.329	0.431	0.512	1.453	0.444	0.466	1.178	0.262
13272	0.295	0.082	0.224	0.149	-0.882	0.422	0.097	-1.181	0.328
13273	0.489	1.329	0.501	0.485	1.283	0.309	0.511	1.464	0.185
13274	0.505	1.430	0.501	0.497	1.359	0.501	0.489	1.329	0.185
13275	0.511	1.464	0.492	0.477	1.227	0.501	0.500	1.397	0.050
13276	0.300	0.116	0.385	0.310	0.153	0.508	0.224	-0.373	0.481
13277	0.142	-0.895	0.492	0.234	-0.336	0.512	0.355	0.470	0.492
13278	0.429	0.942	0.185	0.512	1.453	0.508	0.511	1.464	0.050
13279	0.450	1.076	0.185	0.503	1.396	0.491	0.489	1.329	0.431
13280	0.461	1.144	0.467	0.500	1.378	0.478	0.513	1.481	0.358
13281	0.000	?	0.224	0.313	0.172	0.514	0.224	-0.373	0.358
13282	0.489	1.329	0.185	0.515	1.472	0.501	0.492	1.346	0.508
13283	0.447	1.060	0.467	0.401	0.737	0.371	0.503	1.413	0.050
13284	0.226	-0.356	0.385	0.193	-0.600	0.193	0.297	0.099	0.224
13285	0.300	0.116	0.513	0.307	0.135	0.193	0.097	-1.181	0.385
13286	0.408	0.807	0.501	0.506	1.415	0.501	0.511	1.464	0.050
13287	0.387	0.672	0.296	0.374	0.568	0.512	0.297	0.099	0.262
13288	0.492	1.346	0.097	0.444	1.020	0.444	0.513	1.481	0.185
13289	0.511	1.464	0.508	0.421	0.869	0.512	0.461	1.144	0.224
13290	0.184	-0.625	0.296	0.310	0.153	0.491	0.184	-0.625	0.481
13291	0.503	1.413	0.467	0.503	1.396	0.491	0.516	1.498	0.185
13292	0.503	1.413	0.467	0.500	1.378	0.422	0.516	1.498	0.467
13293	0.505	1.430	0.142	0.512	1.453	0.444	0.513	1.481	0.185
13294	0.500	1.397	0.262	0.518	1.491	0.501	0.468	1.194	0.512

13295	0.505	1.430	0.512	0.506	1.415	0.235	0.466	1.178	0.501
13296	0.513	1.481	0.224	0.488	1.302	0.422	0.461	1.144	0.508
13297	0.505	1.430	0.431	0.477	1.227	0.309	0.505	1.430	0.467
13298	0.463	1.161	0.142	0.512	1.453	0.508	0.513	1.481	0.508
13299	0.142	-0.895	0.262	0.500	1.378	0.235	0.384	0.655	0.508
13300	0.326	0.285	0.142	0.371	0.549	0.422	0.384	0.655	0.431
13301	0.326	0.285	0.513	0.193	-0.600	0.193	0.050	-1.485	0.097
13302	0.261	-0.137	0.467	0.193	-0.600	0.512	0.142	-0.895	0.492
13303	0.453	1.093	0.328	0.506	1.415	0.341	0.500	1.397	0.501
13304	0.503	1.413	0.409	0.518	1.491	0.371	0.479	1.262	0.481
13305	0.503	1.413	0.385	0.450	1.057	0.193	0.492	1.346	0.262
13306	0.497	1.380	0.224	0.518	1.491	0.371	0.503	1.413	0.262
13307	0.492	1.346	0.467	0.497	1.359	0.508	0.461	1.144	0.224
13308	0.492	1.346	0.296	0.497	1.359	0.235	0.476	1.245	0.328
13309	0.050	-1.485	0.262	0.424	0.888	0.235	0.295	0.082	0.481
13310	0.516	1.498	0.512	0.439	0.982	0.235	0.503	1.413	0.224
13311	0.511	1.464	0.512	0.497	1.359	0.462	0.384	0.655	0.508
13312	0.224	-0.373	0.513	0.234	-0.336	0.478	0.263	-0.120	0.508
13313	0.492	1.346	0.513	0.424	0.888	0.422	0.508	1.447	0.508
13314	0.482	1.279	0.512	0.310	0.153	0.273	0.516	1.498	0.513
13315	0.497	1.380	0.508	0.503	1.396	0.462	0.513	1.481	0.508
13316	0.097	-1.181	0.512	0.234	-0.336	0.193	0.295	0.082	0.409
13317	0.505	1.430	0.142	0.421	0.869	0.491	0.508	1.447	0.409
13318	0.387	0.672	0.481	0.193	-0.600	0.273	0.145	-0.878	0.513
13319	0.505	1.430	0.513	0.518	1.491	0.462	0.429	0.942	0.224
13320	0.484	1.295	0.467	0.342	0.361	0.491	0.505	1.430	0.224
13321	0.142	-0.895	0.224	0.374	0.568	0.273	0.297	0.099	0.328
13322	0.261	-0.137	0.501	0.193	-0.600	0.501	0.329	0.301	0.328
13323	0.332	0.318	0.513	0.374	0.568	0.512	0.261	-0.137	0.513
13324	0.355	0.470	0.513	0.509	1.434	0.478	0.508	1.447	0.512
13325	0.300	0.116	0.097	0.234	-0.336	0.273	0.184	-0.625	0.513
13326	0.300	0.116	0.513	0.234	-0.336	0.478	0.184	-0.625	0.409
13327	0.300	0.116	0.142	0.234	-0.336	0.053	0.184	-0.625	0.262
13328	0.450	1.076	0.224	0.468	1.170	0.053	0.513	1.481	0.224
13329	0.466	1.178	0.467	0.482	1.265	0.309	0.513	1.481	0.467
13330	0.505	1.430	0.224	0.427	0.907	0.462	0.468	1.194	0.328
13331	0.142	-0.895	0.508	0.275	-0.073	0.508	0.226	-0.356	0.501
13332	0.489	1.329	0.262	0.462	1.133	0.193	0.495	1.363	0.501
13333	0.329	0.301	0.431	0.193	-0.600	0.508	0.097	-1.181	0.224
13334	0.450	1.076	0.185	0.491	1.321	0.053	0.500	1.397	0.050
13335	0.332	0.318	0.512	0.275	-0.073	0.273	0.184	-0.625	0.508
13336	0.500	1.397	0.508	0.462	1.133	0.149	0.466	1.178	0.358
13337	0.492	1.346	0.501	0.485	1.283	0.422	0.447	1.060	0.512
13338	0.263	-0.120	0.185	0.275	-0.073	0.512	0.142	-0.895	0.296
13339	0.450	1.076	0.501	0.500	1.378	0.501	0.468	1.194	0.185
13340	0.513	1.481	0.501	0.509	1.434	0.341	0.508	1.447	0.262
13341	0.429	0.942	0.481	0.482	1.265	0.422	0.511	1.464	0.467
13342	0.184	-0.625	0.185	0.275	-0.073	0.501	0.142	-0.895	0.492
13343	0.508	1.447	0.431	0.485	1.283	0.508	0.511	1.464	0.050
13344	0.224	-0.373	0.501	0.053	-1.504	0.508	0.263	-0.120	0.050
13345	0.224	-0.373	0.481	0.053	-1.504	0.309	0.263	-0.120	0.142
13346	0.184	-0.625	0.501	0.310	0.153	0.309	0.184	-0.625	0.501
13347	0.500	1.397	0.513	0.459	1.114	0.102	0.513	1.481	0.097
13348	0.484	1.295	0.508	0.512	1.453	0.508	0.495	1.363	0.142
13349	0.000	?	0.481	0.196	-0.581	0.514	0.326	0.285	0.450
13350	0.508	1.447	0.358	0.506	1.415	0.309	0.450	1.076	0.450
13351	0.261	-0.137	0.296	0.053	-1.504	0.514	0.429	0.942	0.513
13352	0.408	0.807	0.512	0.275	-0.073	0.422	0.453	1.093	0.508
13353	0.142	-0.895	0.492	0.149	-0.882	0.309	0.358	0.487	0.481
13354	0.505	1.430	0.467	0.427	0.907	0.501	0.511	1.464	0.467
13355	0.495	1.363	0.296	0.509	1.434	0.478	0.495	1.363	0.513

13356	0.384	0.655	0.508	0.497	1.359	0.478	0.466	1.178	0.224
13357	0.142	-0.895	0.224	0.342	0.361	0.309	0.297	0.099	0.512
13358	0.329	0.301	0.224	0.421	0.869	0.309	0.384	0.655	0.481
13359	0.429	0.942	0.492	0.503	1.396	0.273	0.511	1.464	0.492
13360	0.500	1.397	0.262	0.509	1.434	0.478	0.487	1.312	0.097
13361	0.489	1.329	0.097	0.509	1.434	0.462	0.495	1.363	0.508
13362	0.224	-0.373	0.501	0.310	0.153	0.478	0.142	-0.895	0.501
13363	0.224	-0.373	0.185	0.310	0.153	0.514	0.184	-0.625	0.501
13364	0.142	-0.895	0.185	0.102	-1.184	0.514	0.297	0.099	0.142
13365	0.432	0.958	0.262	0.509	1.434	0.309	0.500	1.397	0.501
13366	0.508	1.447	0.501	0.509	1.434	0.491	0.503	1.413	0.224
13367	0.224	-0.373	0.467	0.313	0.172	0.102	0.000	?	0.296
13368	0.497	1.380	0.224	0.515	1.472	0.102	0.511	1.464	0.481
13369	0.492	1.346	0.262	0.421	0.869	0.501	0.516	1.498	0.328
13370	0.295	0.082	0.508	0.313	0.172	0.508	0.295	0.082	0.358
13371	0.484	1.295	0.481	0.503	1.396	0.341	0.492	1.346	0.296
13372	0.500	1.397	0.492	0.474	1.208	0.478	0.447	1.060	0.512
13373	0.492	1.346	0.450	0.474	1.208	0.478	0.447	1.060	0.185
13374	0.142	-0.895	0.358	0.313	0.172	0.444	0.142	-0.895	0.481
13375	0.326	0.285	0.185	0.307	0.135	0.398	0.408	0.807	0.481
13376	0.387	0.672	0.142	0.272	-0.091	0.193	0.184	-0.625	0.450
13377	0.508	1.447	0.185	0.477	1.227	0.273	0.505	1.430	0.262
13378	0.508	1.447	0.224	0.462	1.133	0.273	0.508	1.447	0.501
13379	0.508	1.447	0.185	0.477	1.227	0.341	0.505	1.430	0.508
13380	0.355	0.470	0.512	0.512	1.453	0.341	0.505	1.430	0.450
13381	0.429	0.942	0.501	0.509	1.434	0.512	0.453	1.093	0.328
13382	0.226	-0.356	0.185	0.307	0.135	0.398	0.097	-1.181	0.385
13383	0.508	1.447	0.224	0.494	1.340	0.273	0.518	1.514	0.492
13384	0.326	0.285	0.508	0.102	-1.184	0.462	0.142	-0.895	0.385
13385	0.326	0.285	0.481	0.102	-1.184	0.235	0.142	-0.895	0.501
13386	0.479	1.262	0.224	0.500	1.378	0.273	0.408	0.807	0.501
13387	0.495	1.363	0.409	0.506	1.415	0.508	0.489	1.329	0.431
13388	0.511	1.464	0.501	0.339	0.342	0.193	0.497	1.380	0.508
13389	0.489	1.329	0.142	0.474	1.208	0.398	0.511	1.464	0.050
13390	0.489	1.329	0.492	0.474	1.208	0.478	0.511	1.464	0.050
13391	0.466	1.178	0.224	0.447	1.039	0.273	0.455	1.110	0.512
13392	0.142	-0.895	0.513	0.398	0.718	0.273	0.297	0.099	0.508
13393	0.295	0.082	0.224	0.193	-0.600	0.235	0.261	-0.137	0.512
13394	0.300	0.116	0.142	0.272	-0.091	0.193	0.329	0.301	0.508
13395	0.295	0.082	0.409	0.272	-0.091	0.508	0.184	-0.625	0.097
13396	0.187	-0.608	0.142	0.339	0.342	0.478	0.184	-0.625	0.050
13397	0.224	-0.373	0.481	0.342	0.361	0.508	0.050	-1.485	0.481
13398	0.500	1.397	0.450	0.512	1.453	0.149	0.482	1.279	0.224
13399	0.500	1.397	0.467	0.398	0.718	0.512	0.497	1.380	0.409
13400	0.224	-0.373	0.450	0.275	-0.073	0.501	0.050	-1.485	0.385
13401	0.497	1.380	0.142	0.465	1.152	0.501	0.429	0.942	0.142
13402	0.142	-0.895	0.512	0.234	-0.336	0.508	0.355	0.470	0.185
13403	0.226	-0.356	0.508	0.272	-0.091	0.508	0.355	0.470	0.513
13404	0.508	1.447	0.512	0.506	1.415	0.508	0.505	1.430	0.492
13405	0.300	0.116	0.512	0.193	-0.600	0.462	0.050	-1.485	0.508
13406	0.297	0.099	0.185	0.401	0.737	0.235	0.224	-0.373	0.185
13407	0.495	1.363	0.467	0.482	1.265	0.491	0.384	0.655	0.185
13408	0.224	-0.373	0.224	0.272	-0.091	0.462	0.050	-1.485	0.501
13409	0.184	-0.625	0.185	0.278	-0.054	0.514	0.263	-0.120	0.512
13410	0.224	-0.373	0.467	0.240	-0.299	0.193	0.184	-0.625	0.385
13411	0.142	-0.895	0.467	0.196	-0.581	0.193	0.226	-0.356	0.492
13412	0.513	1.481	0.481	0.506	1.415	0.273	0.476	1.245	0.328
13413	0.184	-0.625	0.508	0.482	1.265	0.309	0.184	-0.625	0.185
13414	0.463	1.161	0.512	0.512	1.453	0.491	0.466	1.178	0.224
13415	0.224	-0.373	0.358	0.149	-0.882	0.235	0.184	-0.625	0.224
13416	0.408	0.807	0.328	0.515	1.472	0.478	0.505	1.430	0.224

13417	0.492	1.346	0.501	0.503	1.396	0.478	0.500	1.397	0.224
13418	0.492	1.346	0.492	0.503	1.396	0.478	0.508	1.447	0.450
13419	0.500	1.397	0.328	0.509	1.434	0.512	0.468	1.194	0.481
13420	0.463	1.161	0.501	0.506	1.415	0.478	0.505	1.430	0.185
13421	0.492	1.346	0.508	0.506	1.415	0.341	0.508	1.447	0.142
13422	0.492	1.346	0.481	0.465	1.152	0.341	0.432	0.958	0.467
13423	0.226	-0.356	0.501	0.234	-0.336	0.491	0.097	-1.181	0.481
13424	0.468	1.194	0.492	0.491	1.321	0.235	0.497	1.380	0.328
13425	0.497	1.380	0.508	0.462	1.133	0.235	0.500	1.397	0.508
13426	0.500	1.397	0.513	0.512	1.453	0.371	0.484	1.295	0.481
13427	0.387	0.672	0.501	0.193	-0.600	0.371	0.261	-0.137	0.224
13428	0.097	-1.181	0.501	0.193	-0.600	0.512	0.261	-0.137	0.508
13429	0.263	-0.120	0.501	0.275	-0.073	0.508	0.224	-0.373	0.142
13430	0.408	0.807	0.492	0.310	0.153	0.512	0.329	0.301	0.328
13431	0.429	0.942	0.512	0.491	1.321	0.478	0.487	1.312	0.328
13432	0.224	-0.373	0.501	0.237	-0.317	0.193	0.224	-0.373	0.467
13433	0.503	1.413	0.501	0.477	1.227	0.398	0.500	1.397	0.358
13434	0.503	1.413	0.467	0.477	1.227	0.512	0.500	1.397	0.262
13435	0.492	1.346	0.450	0.477	1.227	0.309	0.500	1.397	0.508
13436	0.384	0.655	0.512	0.512	1.453	0.478	0.503	1.413	0.185
13437	0.492	1.346	0.508	0.477	1.227	0.478	0.500	1.397	0.450
13438	0.184	-0.625	0.492	0.345	0.379	0.235	0.411	0.824	0.501
13439	0.184	-0.625	0.508	0.345	0.379	0.462	0.411	0.824	0.409
13440	0.466	1.178	0.501	0.497	1.359	0.512	0.511	1.464	0.508
13441	0.224	-0.373	0.050	0.234	-0.336	0.149	0.224	-0.373	0.328
13442	0.184	-0.625	0.097	0.234	-0.336	0.462	0.224	-0.373	0.492
13443	0.000	?	0.097	0.371	0.549	0.235	0.326	0.285	0.492
13444	0.000	?	0.492	0.371	0.549	0.309	0.326	0.285	0.450
13445	0.461	1.144	0.050	0.509	1.434	0.309	0.516	1.498	0.481
13446	0.384	0.655	0.185	0.506	1.415	0.514	0.505	1.430	0.262
13447	0.505	1.430	0.512	0.512	1.453	0.422	0.513	1.481	0.262
13448	0.503	1.413	0.450	0.477	1.227	0.235	0.411	0.824	0.262
13449	0.300	0.116	0.501	0.196	-0.581	0.149	0.261	-0.137	0.262
13450	0.097	-1.181	0.467	0.398	0.718	0.193	0.224	-0.373	0.492
13451	0.497	1.380	0.296	0.512	1.453	0.193	0.468	1.194	0.492
13452	0.297	0.099	0.431	0.307	0.135	0.491	0.326	0.285	0.512
13453	0.466	1.178	0.431	0.477	1.227	0.341	0.505	1.430	0.450
13454	0.466	1.178	0.385	0.477	1.227	0.341	0.505	1.430	0.501
13455	0.142	-0.895	0.328	0.234	-0.336	0.478	0.226	-0.356	0.224
13456	0.266	-0.103	0.467	0.459	1.114	0.478	0.050	-1.485	0.262
13457	0.511	1.464	0.508	0.512	1.453	0.478	0.508	1.447	0.481
13458	0.224	-0.373	0.224	0.149	-0.882	0.444	0.361	0.504	0.512
13459	0.432	0.958	0.262	0.465	1.152	0.514	0.513	1.481	0.492
13460	0.142	-0.895	0.185	0.237	-0.317	0.514	0.297	0.099	0.481
13461	0.261	-0.137	0.512	0.310	0.153	0.444	0.184	-0.625	0.492
13462	0.142	-0.895	0.481	0.313	0.172	0.193	0.261	-0.137	0.512
13463	0.511	1.464	0.481	0.515	1.472	0.491	0.461	1.144	0.508
13464	0.463	1.161	0.467	0.421	0.869	0.462	0.492	1.346	0.501
13465	0.329	0.301	0.467	0.237	-0.317	0.514	0.050	-1.485	0.513
13466	0.408	0.807	0.481	0.149	-0.882	0.235	0.050	-1.485	0.508
13467	0.384	0.655	0.512	0.193	-0.600	0.512	0.000	?	0.431
13468	0.226	-0.356	0.481	0.196	-0.581	0.491	0.142	-0.895	0.431
13469	0.471	1.211	0.513	0.488	1.302	0.491	0.500	1.397	0.431
13470	0.295	0.082	0.513	0.342	0.361	0.341	0.097	-1.181	0.431
13471	0.261	-0.137	0.501	0.345	0.379	0.508	0.142	-0.895	0.513
13472	0.508	1.447	0.142	0.474	1.208	0.341	0.447	1.060	0.481
13473	0.508	1.447	0.097	0.474	1.208	0.309	0.447	1.060	0.450
13474	0.508	1.447	0.508	0.474	1.208	0.501	0.513	1.481	0.501
13475	0.508	1.447	0.508	0.439	0.982	0.235	0.508	1.447	0.512
13476	0.508	1.447	0.050	0.512	1.453	0.462	0.479	1.262	0.431
13477	0.503	1.413	0.450	0.512	1.453	0.273	0.466	1.178	0.481

13478	0.505	1.430	0.142	0.442	1.001	0.478	0.513	1.481	0.508
13479	0.142	-0.895	0.492	0.199	-0.562	0.193	0.224	-0.373	0.467
13480	0.476	1.245	0.467	0.491	1.321	0.491	0.518	1.514	0.492
13481	0.518	1.514	0.467	0.459	1.114	0.501	0.474	1.228	0.185
13482	0.474	1.228	0.224	0.509	1.434	0.478	0.487	1.312	0.224
13483	0.224	-0.373	0.224	0.234	-0.336	0.235	0.097	-1.181	0.262
13484	0.497	1.380	0.262	0.509	1.434	0.501	0.505	1.430	0.508
13485	0.516	1.498	0.492	0.491	1.321	0.501	0.505	1.430	0.050
13486	0.516	1.498	0.508	0.491	1.321	0.508	0.505	1.430	0.142
13487	0.097	-1.181	0.296	0.342	0.361	0.149	0.142	-0.895	0.185
13488	0.503	1.413	0.492	0.512	1.453	0.309	0.505	1.430	0.501
13489	0.142	-0.895	0.481	0.342	0.361	0.508	0.226	-0.356	0.512
13490	0.224	-0.373	0.185	0.310	0.153	0.508	0.295	0.082	0.501
13491	0.463	1.161	0.185	0.503	1.396	0.512	0.484	1.295	0.508
13492	0.226	-0.356	0.431	0.234	-0.336	0.501	0.329	0.301	0.050
13493	0.497	1.380	0.431	0.465	1.152	0.514	0.358	0.487	0.492
13494	0.261	-0.137	0.481	0.275	-0.073	0.491	0.295	0.082	0.431
13495	0.429	0.942	0.224	0.482	1.265	0.193	0.513	1.481	0.097
13496	0.187	-0.608	0.513	0.193	-0.600	0.193	0.187	-0.608	0.262
13497	0.511	1.464	0.185	0.488	1.302	0.462	0.474	1.228	0.508
13498	0.508	1.447	0.262	0.497	1.359	0.491	0.474	1.228	0.492
13499	0.500	1.397	0.450	0.471	1.189	0.491	0.450	1.076	0.097
13500	0.184	-0.625	0.385	0.237	-0.317	0.235	0.263	-0.120	0.097
13501	0.503	1.413	0.450	0.494	1.340	0.501	0.497	1.380	0.481
13502	0.503	1.413	0.481	0.494	1.340	0.235	0.503	1.413	0.512
13503	0.482	1.279	0.513	0.506	1.415	0.341	0.450	1.076	0.512
13504	0.184	-0.625	0.097	0.149	-0.882	0.462	0.332	0.318	0.508
13505	0.432	0.958	0.097	0.310	0.153	0.235	0.389	0.689	0.508
13506	0.503	1.413	0.512	0.509	1.434	0.102	0.489	1.329	0.450
13507	0.482	1.279	0.508	0.509	1.434	0.491	0.382	0.638	0.512
13508	0.503	1.413	0.262	0.512	1.453	0.273	0.503	1.413	0.492
13509	0.511	1.464	0.262	0.503	1.396	0.235	0.495	1.363	0.492
13510	0.497	1.380	0.492	0.520	1.509	0.512	0.434	0.975	0.450
13511	0.482	1.279	0.492	0.488	1.302	0.491	0.503	1.413	0.513
13512	0.358	0.487	0.467	0.193	-0.600	0.444	0.050	-1.485	0.358
13513	0.297	0.099	0.513	0.196	-0.581	0.235	0.050	-1.485	0.097
13514	0.511	1.464	0.508	0.462	1.133	0.102	0.505	1.430	0.467
13515	0.495	1.363	0.481	0.485	1.283	0.491	0.505	1.430	0.467
13516	0.468	1.194	0.050	0.485	1.283	0.501	0.508	1.447	0.224
13517	0.295	0.082	0.450	0.234	-0.336	0.514	0.000	?	0.467
13518	0.508	1.447	0.262	0.500	1.378	0.514	0.503	1.413	0.431
13519	0.226	-0.356	0.501	0.237	-0.317	0.273	0.097	-1.181	0.501
13520	0.224	-0.373	0.501	0.345	0.379	0.273	0.050	-1.485	0.492
13521	0.489	1.329	0.501	0.465	1.152	0.235	0.479	1.262	0.512
13522	0.261	-0.137	0.262	0.234	-0.336	0.235	0.224	-0.373	0.185
13523	0.097	-1.181	0.296	0.102	-1.184	0.273	0.405	0.790	0.185
13524	0.497	1.380	0.501	0.491	1.321	0.149	0.382	0.638	0.050
13525	0.187	-0.608	0.296	0.275	-0.073	0.508	0.142	-0.895	0.450
13526	0.184	-0.625	0.296	0.237	-0.317	0.491	0.184	-0.625	0.513
13527	0.261	-0.137	0.501	0.512	1.453	0.193	0.513	1.481	0.224
13528	0.505	1.430	0.296	0.491	1.321	0.193	0.492	1.346	0.512
13529	0.463	1.161	0.296	0.447	1.039	0.501	0.508	1.447	0.512
13530	0.224	-0.373	0.492	0.237	-0.317	0.491	0.184	-0.625	0.328
13531	0.263	-0.120	0.467	0.102	-1.184	0.149	0.187	-0.608	0.328
13532	0.500	1.397	0.262	0.491	1.321	0.398	0.505	1.430	0.512
13533	0.476	1.245	0.224	0.497	1.359	0.512	0.511	1.464	0.512
13534	0.495	1.363	0.467	0.512	1.453	0.193	0.382	0.638	0.450
13535	0.450	1.076	0.481	0.512	1.453	0.444	0.492	1.346	0.262
13536	0.358	0.487	0.501	0.275	-0.073	0.193	0.329	0.301	0.492
13537	0.184	-0.625	0.512	0.275	-0.073	0.273	0.184	-0.625	0.185
13538	0.142	-0.895	0.513	0.234	-0.336	0.371	0.224	-0.373	0.097

13539	0.184	-0.625	0.409	0.234	-0.336	0.508	0.224	-0.373	0.481
13540	0.224	-0.373	0.050	0.275	-0.073	0.309	0.224	-0.373	0.512
13541	0.184	-0.625	0.185	0.149	-0.882	0.309	0.224	-0.373	0.512
13542	0.513	1.481	0.262	0.509	1.434	0.512	0.445	1.043	0.492
13543	0.497	1.380	0.296	0.488	1.302	0.309	0.479	1.262	0.492
13544	0.184	-0.625	0.467	0.193	-0.600	0.501	0.184	-0.625	0.142
13545	0.224	-0.373	0.262	0.196	-0.581	0.514	0.142	-0.895	0.224
13546	0.505	1.430	0.450	0.500	1.378	0.422	0.461	1.144	0.431
13547	0.476	1.245	0.508	0.491	1.321	0.462	0.476	1.245	0.508
13548	0.224	-0.373	0.328	0.149	-0.882	0.102	0.329	0.301	0.185
13549	0.411	0.824	0.513	0.398	0.718	0.422	0.508	1.447	0.296
13550	0.500	1.397	0.409	0.515	1.472	0.422	0.487	1.312	0.512
13551	0.142	-0.895	0.328	0.196	-0.581	0.501	0.224	-0.373	0.467
13552	0.489	1.329	0.262	0.442	1.001	0.512	0.505	1.430	0.512
13553	0.224	-0.373	0.262	0.193	-0.600	0.149	0.142	-0.895	0.467
13554	0.000	?	0.481	0.275	-0.073	0.508	0.329	0.301	0.467
13555	0.000	?	0.508	0.371	0.549	0.149	0.329	0.301	0.481
13556	0.505	1.430	0.481	0.509	1.434	0.102	0.468	1.194	0.262
13557	0.226	-0.356	0.481	0.310	0.153	0.508	0.355	0.470	0.492
13558	0.142	-0.895	0.224	0.313	0.172	0.508	0.263	-0.120	0.142
13559	0.405	0.790	0.501	0.515	1.472	0.491	0.503	1.413	0.513
13560	0.142	-0.895	0.050	0.307	0.135	0.512	0.184	-0.625	0.513
13561	0.479	1.262	0.050	0.506	1.415	0.491	0.447	1.060	0.513
13562	0.450	1.076	0.224	0.515	1.472	0.149	0.500	1.397	0.097
13563	0.468	1.194	0.512	0.421	0.869	0.149	0.405	0.790	0.097
13564	0.450	1.076	0.224	0.459	1.114	0.491	0.503	1.413	0.431
13565	0.142	-0.895	0.185	0.102	-1.184	0.512	0.329	0.301	0.097
13566	0.513	1.481	0.050	0.421	0.869	0.422	0.495	1.363	0.097
13567	0.511	1.464	0.508	0.421	0.869	0.512	0.495	1.363	0.431
13568	0.508	1.447	0.508	0.497	1.359	0.478	0.447	1.060	0.185
13569	0.508	1.447	0.262	0.509	1.434	0.478	0.479	1.262	0.142
13570	0.187	-0.608	0.512	0.149	-0.882	0.514	0.261	-0.137	0.501
13571	0.471	1.211	0.508	0.503	1.396	0.514	0.263	-0.120	0.481
13572	0.226	-0.356	0.262	0.149	-0.882	0.512	0.263	-0.120	0.185
13573	0.187	-0.608	0.467	0.102	-1.184	0.462	0.261	-0.137	0.185
13574	0.463	1.161	0.296	0.509	1.434	0.514	0.495	1.363	0.492
13575	0.463	1.161	0.296	0.509	1.434	0.514	0.495	1.363	0.492
13576	0.479	1.262	0.262	0.491	1.321	0.514	0.505	1.430	0.492
13577	0.505	1.430	0.358	0.512	1.453	0.514	0.445	1.043	0.431
13578	0.500	1.397	0.224	0.494	1.340	0.444	0.500	1.397	0.450
13579	0.363	0.520	0.512	0.149	-0.882	0.491	0.224	-0.373	0.492
13580	0.332	0.318	0.481	0.149	-0.882	0.512	0.263	-0.120	0.358
13581	0.497	1.380	0.481	0.491	1.321	0.512	0.482	1.279	0.142
13582	0.497	1.380	0.492	0.512	1.453	0.462	0.511	1.464	0.097
13583	0.329	0.301	0.262	0.421	0.869	0.462	0.489	1.329	0.224
13584	0.497	1.380	0.142	0.512	1.453	0.462	0.479	1.262	0.481
13585	0.503	1.413	0.512	0.477	1.227	0.462	0.489	1.329	0.142
13586	0.476	1.245	0.328	0.485	1.283	0.478	0.508	1.447	0.501
13587	0.497	1.380	0.431	0.515	1.472	0.512	0.511	1.464	0.508
13588	0.492	1.346	0.467	0.523	1.528	0.398	0.500	1.397	0.185
13589	0.497	1.380	0.508	0.509	1.434	0.341	0.518	1.514	0.481
13590	0.508	1.447	0.467	0.459	1.114	0.371	0.505	1.430	0.481
13591	0.497	1.380	0.097	0.515	1.472	0.508	0.434	0.975	0.050
13592	0.497	1.380	0.481	0.515	1.472	0.478	0.434	0.975	0.185
13593	0.497	1.380	0.296	0.515	1.472	0.398	0.434	0.975	0.185
13594	0.487	1.312	0.185	0.515	1.472	0.193	0.434	0.975	0.450
13595	0.505	1.430	0.224	0.444	1.020	0.512	0.516	1.498	0.508
13596	0.497	1.380	0.328	0.491	1.321	0.491	0.484	1.295	0.512
13597	0.497	1.380	0.185	0.512	1.453	0.508	0.453	1.093	0.512
13598	0.463	1.161	0.262	0.515	1.472	0.444	0.508	1.447	0.296
13599	0.447	1.060	0.492	0.468	1.170	0.235	0.518	1.514	0.513

13600	0.511	1.464	0.296	0.465	1.152	0.512	0.429	0.942	0.185
13601	0.505	1.430	0.481	0.462	1.133	0.512	0.487	1.312	0.296
13602	0.489	1.329	0.296	0.462	1.133	0.102	0.513	1.481	0.262
13603	0.508	1.447	0.185	0.477	1.227	0.422	0.471	1.211	0.296
13604	0.497	1.380	0.501	0.512	1.453	0.462	0.487	1.312	0.142
13605	0.050	-1.485	0.467	0.401	0.737	0.478	0.184	-0.625	0.185
13606	0.097	-1.181	0.501	0.339	0.342	0.235	0.226	-0.356	0.296
13607	0.097	-1.181	0.508	0.374	0.568	0.309	0.261	-0.137	0.431
13608	0.489	1.329	0.296	0.512	1.453	0.273	0.503	1.413	0.481
13609	0.050	-1.485	0.481	0.477	1.227	0.422	0.050	-1.485	0.050
13610	0.000	?	0.185	0.401	0.737	0.422	0.142	-0.895	0.467
13611	0.184	-0.625	0.513	0.196	-0.581	0.491	0.187	-0.608	0.481
13612	0.513	1.481	0.450	0.509	1.434	0.501	0.497	1.380	0.262
13613	0.453	1.093	0.508	0.491	1.321	0.501	0.503	1.413	0.513
13614	0.495	1.363	0.431	0.512	1.453	0.491	0.503	1.413	0.262
13615	0.463	1.161	0.097	0.447	1.039	0.478	0.508	1.447	0.262
13616	0.297	0.099	0.492	0.234	-0.336	0.491	0.050	-1.485	0.185
13617	0.429	0.942	0.450	0.512	1.453	0.462	0.489	1.329	0.097
13618	0.426	0.925	0.185	0.512	1.453	0.508	0.429	0.942	0.185
13619	0.387	0.672	0.481	0.102	-1.184	0.444	0.097	-1.181	0.450
13620	0.332	0.318	0.097	0.427	0.907	0.341	0.261	-0.137	0.512
13621	0.466	1.178	0.185	0.468	1.170	0.341	0.505	1.430	0.512
13622	0.508	1.447	0.097	0.482	1.265	0.514	0.492	1.346	0.492
13623	0.226	-0.356	0.185	0.234	-0.336	0.508	0.097	-1.181	0.185
13624	0.261	-0.137	0.492	0.310	0.153	0.273	0.000	?	0.481
13625	0.184	-0.625	0.358	0.275	-0.073	0.462	0.097	-1.181	0.224
13626	0.508	1.447	0.501	0.421	0.869	0.193	0.479	1.262	0.501
13627	0.479	1.262	0.492	0.418	0.850	0.422	0.511	1.464	0.481
13628	0.479	1.262	0.492	0.485	1.283	0.512	0.511	1.464	0.431
13629	0.466	1.178	0.467	0.497	1.359	0.514	0.505	1.430	0.450
13630	0.466	1.178	0.262	0.497	1.359	0.193	0.505	1.430	0.262
13631	0.482	1.279	0.296	0.491	1.321	0.193	0.450	1.076	0.431
13632	0.508	1.447	0.185	0.474	1.208	0.341	0.516	1.498	0.224
13633	0.476	1.245	0.431	0.497	1.359	0.501	0.492	1.346	0.262
13634	0.513	1.481	0.492	0.459	1.114	0.422	0.487	1.312	0.262
13635	0.513	1.481	0.185	0.509	1.434	0.371	0.450	1.076	0.512
13636	0.503	1.413	0.508	0.442	1.001	0.462	0.513	1.481	0.358
13637	0.142	-0.895	0.097	0.342	0.361	0.512	0.361	0.504	0.262
13638	0.097	-1.181	0.501	0.345	0.379	0.235	0.097	-1.181	0.481
13639	0.497	1.380	0.492	0.515	1.472	0.235	0.466	1.178	0.224
13640	0.503	1.413	0.097	0.515	1.472	0.501	0.468	1.194	0.492
13641	0.050	-1.485	0.185	0.278	-0.054	0.501	0.224	-0.373	0.262
13642	0.447	1.060	0.224	0.462	1.133	0.501	0.461	1.144	0.431
13643	0.142	-0.895	0.185	0.196	-0.581	0.193	0.432	0.958	0.512
13644	0.489	1.329	0.142	0.424	0.888	0.422	0.497	1.380	0.481
13645	0.463	1.161	0.512	0.515	1.472	0.309	0.492	1.346	0.492
13646	0.463	1.161	0.492	0.509	1.434	0.273	0.511	1.464	0.467
13647	0.224	-0.373	0.492	0.193	-0.600	0.478	0.184	-0.625	0.328
13648	0.224	-0.373	0.492	0.193	-0.600	0.371	0.184	-0.625	0.512
13649	0.263	-0.120	0.492	0.342	0.361	0.398	0.050	-1.485	0.328
13650	0.495	1.363	0.492	0.494	1.340	0.491	0.447	1.060	0.481
13651	0.505	1.430	0.512	0.421	0.869	0.508	0.508	1.447	0.492
13652	0.297	0.099	0.450	0.374	0.568	0.309	0.226	-0.356	0.481
13653	0.489	1.329	0.050	0.462	1.133	0.235	0.508	1.447	0.467
13654	0.482	1.279	0.224	0.512	1.453	0.514	0.511	1.464	0.409
13655	0.187	-0.608	0.358	0.234	-0.336	0.491	0.329	0.301	0.512
13656	0.187	-0.608	0.492	0.234	-0.336	0.193	0.329	0.301	0.501
13657	0.432	0.958	0.492	0.497	1.359	0.149	0.508	1.447	0.328
13658	0.432	0.958	0.501	0.497	1.359	0.444	0.508	1.447	0.508
13659	0.479	1.262	0.467	0.497	1.359	0.512	0.450	1.076	0.142
13660	0.224	-0.373	0.512	0.193	-0.600	0.491	0.263	-0.120	0.224

13661	0.511	1.464	0.185	0.421	0.869	0.508	0.487	1.312	0.328
13662	0.184	−0.625	0.508	0.310	0.153	0.501	0.184	−0.625	0.296
13663	0.263	−0.120	0.501	0.275	−0.073	0.462	0.097	−1.181	0.501
13664	0.447	1.060	0.296	0.480	1.246	0.309	0.479	1.262	0.224
13665	0.384	0.655	0.296	0.374	0.568	0.444	0.511	1.464	0.512
13666	0.450	1.076	0.224	0.401	0.737	0.149	0.511	1.464	0.481
13667	0.474	1.228	0.224	0.491	1.321	0.508	0.489	1.329	0.501
13668	0.503	1.413	0.185	0.509	1.434	0.508	0.495	1.363	0.501
13669	0.097	−1.181	0.508	0.310	0.153	0.508	0.145	−0.878	0.224
13670	0.097	−1.181	0.328	0.234	−0.336	0.193	0.229	−0.339	0.050
13671	0.508	1.447	0.450	0.509	1.434	0.273	0.429	0.942	0.224
13672	0.503	1.413	0.450	0.485	1.283	0.514	0.508	1.447	0.508
13673	0.263	−0.120	0.467	0.193	−0.600	0.273	0.187	−0.608	0.513
13674	0.263	−0.120	0.501	0.149	−0.882	0.273	0.297	0.099	0.142
13675	0.489	1.329	0.328	0.444	1.020	0.512	0.513	1.481	0.508
13676	0.492	1.346	0.492	0.509	1.434	0.053	0.461	1.144	0.358
13677	0.463	1.161	0.513	0.488	1.302	0.193	0.508	1.447	0.431
13678	0.505	1.430	0.501	0.509	1.434	0.462	0.468	1.194	0.492
13679	0.500	1.397	0.262	0.503	1.396	0.514	0.468	1.194	0.224
13680	0.474	1.228	0.296	0.462	1.133	0.309	0.482	1.279	0.262
13681	0.050	−1.485	0.296	0.310	0.153	0.235	0.261	−0.137	0.262
13682	0.453	1.093	0.224	0.444	1.020	0.501	0.492	1.346	0.262
13683	0.263	−0.120	0.224	0.149	−0.882	0.422	0.142	−0.895	0.262
13684	0.492	1.346	0.224	0.512	1.453	0.501	0.513	1.481	0.409
13685	0.492	1.346	0.508	0.512	1.453	0.512	0.513	1.481	0.512
13686	0.492	1.346	0.467	0.512	1.453	0.514	0.513	1.481	0.492
13687	0.263	−0.120	0.142	0.196	−0.581	0.514	0.097	−1.181	0.512
13688	0.297	0.099	0.508	0.275	−0.073	0.149	0.097	−1.181	0.508
13689	0.500	1.397	0.450	0.506	1.415	0.341	0.429	0.942	0.467
13690	0.297	0.099	0.508	0.275	−0.073	0.193	0.097	−1.181	0.508
13691	0.297	0.099	0.431	0.275	−0.073	0.501	0.097	−1.181	0.512
13692	0.500	1.397	0.097	0.506	1.415	0.193	0.429	0.942	0.142
13693	0.297	0.099	0.296	0.053	−1.504	0.501	0.187	−0.608	0.050
13694	0.295	0.082	0.224	0.193	−0.600	0.514	0.142	−0.895	0.409
13695	0.495	1.363	0.224	0.462	1.133	0.371	0.497	1.380	0.492
13696	0.463	1.161	0.501	0.515	1.472	0.462	0.476	1.245	0.492
13697	0.261	−0.137	0.492	0.310	0.153	0.508	0.184	−0.625	0.512
13698	0.224	−0.373	0.512	0.234	−0.336	0.193	0.184	−0.625	0.513
13699	0.463	1.161	0.224	0.500	1.378	0.149	0.487	1.312	0.450
13700	0.484	1.295	0.481	0.424	0.888	0.149	0.487	1.312	0.185
13701	0.495	1.363	0.450	0.500	1.378	0.508	0.492	1.346	0.492
13702	0.508	1.447	0.431	0.515	1.472	0.478	0.429	0.942	0.508
13703	0.513	1.481	0.185	0.518	1.491	0.501	0.455	1.110	0.097
13704	0.411	0.824	0.296	0.515	1.472	0.501	0.497	1.380	0.097
13705	0.050	−1.485	0.431	0.149	−0.882	0.193	0.361	0.504	0.185
13706	0.184	−0.625	0.262	0.339	0.342	0.512	0.142	−0.895	0.185
13707	0.263	−0.120	0.467	0.193	−0.600	0.371	0.097	−1.181	0.385
13708	0.300	0.116	0.409	0.000	?	0.235	0.224	−0.373	0.467
13709	0.468	1.194	0.513	0.497	1.359	0.371	0.476	1.245	0.296
13710	0.261	−0.137	0.492	0.196	−0.581	0.491	0.142	−0.895	0.467
13711	0.450	1.076	0.450	0.494	1.340	0.193	0.497	1.380	0.467
13712	0.508	1.447	0.450	0.512	1.453	0.309	0.503	1.413	0.467
13713	0.329	0.301	0.450	0.374	0.568	0.309	0.184	−0.625	0.501
13714	0.500	1.397	0.492	0.462	1.133	0.501	0.484	1.295	0.142
13715	0.413	0.841	0.097	0.509	1.434	0.444	0.474	1.228	0.508
13716	0.329	0.301	0.097	0.193	−0.600	0.235	0.050	−1.485	0.508
13717	0.261	−0.137	0.513	0.149	−0.882	0.501	0.184	−0.625	0.492
13718	0.261	−0.137	0.512	0.149	−0.882	0.512	0.184	−0.625	0.185
13719	0.479	1.262	0.142	0.506	1.415	0.273	0.453	1.093	0.050
13720	0.508	1.447	0.492	0.477	1.227	0.501	0.513	1.481	0.097
13721	0.476	1.245	0.501	0.506	1.415	0.193	0.513	1.481	0.142

13722	0.476	1.245	0.450	0.506	1.415	0.193	0.513	1.481	0.142
13723	0.224	-0.373	0.450	0.193	-0.600	0.193	0.300	0.116	0.262
13724	0.492	1.346	0.409	0.512	1.453	0.235	0.518	1.514	0.481
13725	0.050	-1.485	0.142	0.368	0.530	0.149	0.184	-0.625	0.481
13726	0.050	-1.485	0.296	0.234	-0.336	0.512	0.297	0.099	0.262
13727	0.229	-0.339	0.328	0.371	0.549	0.478	0.261	-0.137	0.508
13728	0.508	1.447	0.262	0.488	1.302	0.478	0.295	0.082	0.467
13729	0.224	-0.373	0.512	0.196	-0.581	0.514	0.142	-0.895	0.409
13730	0.184	-0.625	0.296	0.307	0.135	0.273	0.184	-0.625	0.508
13731	0.050	-1.485	0.512	0.310	0.153	0.235	0.297	0.099	0.185
13732	0.497	1.380	0.097	0.506	1.415	0.491	0.426	0.925	0.224
13733	0.497	1.380	0.296	0.447	1.039	0.309	0.479	1.262	0.142
13734	0.263	-0.120	0.142	0.237	-0.317	0.444	0.050	-1.485	0.262
13735	0.511	1.464	0.328	0.500	1.378	0.462	0.463	1.161	0.492
13736	0.513	1.481	0.097	0.509	1.434	0.501	0.474	1.228	0.513
13737	0.263	-0.120	0.224	0.275	-0.073	0.501	0.263	-0.120	0.508
13738	0.468	1.194	0.492	0.500	1.378	0.235	0.505	1.430	0.450
13739	0.297	0.099	0.450	0.196	-0.581	0.462	0.261	-0.137	0.492
13740	0.297	0.099	0.492	0.196	-0.581	0.273	0.261	-0.137	0.501
13741	0.261	-0.137	0.185	0.196	-0.581	0.102	0.184	-0.625	0.501
13742	0.361	0.504	0.431	0.234	-0.336	0.102	0.097	-1.181	0.142
13743	0.224	-0.373	0.142	0.149	-0.882	0.478	0.187	-0.608	0.185
13744	0.505	1.430	0.492	0.512	1.453	0.193	0.447	1.060	0.501
13745	0.482	1.279	0.512	0.480	1.246	0.235	0.505	1.430	0.142
13746	0.482	1.279	0.224	0.480	1.246	0.512	0.505	1.430	0.501
13747	0.492	1.346	0.097	0.515	1.472	0.193	0.489	1.329	0.481
13748	0.266	-0.103	0.262	0.272	-0.091	0.508	0.000	?	0.501
13749	0.142	-0.895	0.296	0.234	-0.336	0.193	0.184	-0.625	0.512
13750	0.508	1.447	0.296	0.488	1.302	0.341	0.476	1.245	0.508
13751	0.329	0.301	0.501	0.310	0.153	0.501	0.224	-0.373	0.508
13752	0.429	0.942	0.492	0.447	1.039	0.508	0.495	1.363	0.508
13753	0.466	1.178	0.492	0.462	1.133	0.478	0.474	1.228	0.512
13754	0.505	1.430	0.467	0.503	1.396	0.501	0.434	0.975	0.185
13755	0.466	1.178	0.492	0.503	1.396	0.235	0.447	1.060	0.185
13756	0.097	-1.181	0.142	0.237	-0.317	0.444	0.261	-0.137	0.409
13757	0.482	1.279	0.492	0.465	1.152	0.235	0.432	0.958	0.409
13758	0.295	0.082	0.467	0.275	-0.073	0.491	0.229	-0.339	0.097
13759	0.184	-0.625	0.492	0.102	-1.184	0.512	0.263	-0.120	0.512
13760	0.224	-0.373	0.492	0.102	-1.184	0.444	0.263	-0.120	0.492
13761	0.326	0.285	0.501	0.482	1.265	0.512	0.513	1.481	0.508
13762	0.184	-0.625	0.501	0.193	-0.600	0.309	0.358	0.487	0.467
13763	0.263	-0.120	0.513	0.237	-0.317	0.508	0.263	-0.120	0.467
13764	0.489	1.329	0.296	0.512	1.453	0.501	0.482	1.279	0.185
13765	0.297	0.099	0.296	0.196	-0.581	0.309	0.224	-0.373	0.142
13766	0.482	1.279	0.501	0.515	1.472	0.512	0.489	1.329	0.097
13767	0.300	0.116	0.513	0.193	-0.600	0.371	0.261	-0.137	0.097
13768	0.184	-0.625	0.513	0.345	0.379	0.273	0.432	0.958	0.409
13769	0.505	1.430	0.050	0.503	1.396	0.149	0.508	1.447	0.142
13770	0.471	1.211	0.409	0.506	1.415	0.149	0.479	1.262	0.512
13771	0.500	1.397	0.185	0.482	1.265	0.444	0.492	1.346	0.142
13772	0.508	1.447	0.501	0.506	1.415	0.193	0.463	1.161	0.097
13773	0.300	0.116	0.296	0.237	-0.317	0.512	0.326	0.285	0.224
13774	0.479	1.262	0.224	0.442	1.001	0.491	0.516	1.498	0.142
13775	0.184	-0.625	0.492	0.234	-0.336	0.462	0.326	0.285	0.142
13776	0.513	1.481	0.450	0.485	1.283	0.491	0.476	1.245	0.097
13777	0.447	1.060	0.097	0.512	1.453	0.235	0.487	1.312	0.508
13778	0.508	1.447	0.262	0.444	1.020	0.193	0.479	1.262	0.508
13779	0.429	0.942	0.481	0.509	1.434	0.309	0.463	1.161	0.501
13780	0.097	-1.181	0.224	0.307	0.135	0.462	0.413	0.841	0.185
13781	0.489	1.329	0.224	0.512	1.453	0.491	0.513	1.481	0.508
13782	0.447	1.060	0.508	0.503	1.396	0.273	0.497	1.380	0.262

13783	0.184	-0.625	0.501	0.307	0.135	0.501	0.326	0.285	0.467
13784	0.479	1.262	0.467	0.515	1.472	0.235	0.505	1.430	0.467
13785	0.097	-1.181	0.142	0.374	0.568	0.491	0.142	-0.895	0.262
13786	0.184	-0.625	0.142	0.275	-0.073	0.512	0.224	-0.373	0.097
13787	0.097	-1.181	0.467	0.149	-0.882	0.235	0.326	0.285	0.142
13788	0.184	-0.625	0.501	0.152	-0.863	0.193	0.295	0.082	0.142
13789	0.492	1.346	0.262	0.442	1.001	0.193	0.503	1.413	0.097
13790	0.361	0.504	0.224	0.196	-0.581	0.309	0.224	-0.373	0.467
13791	0.500	1.397	0.508	0.515	1.472	0.235	0.508	1.447	0.513
13792	0.495	1.363	0.224	0.494	1.340	0.508	0.479	1.262	0.513
13793	0.489	1.329	0.409	0.462	1.133	0.501	0.497	1.380	0.467
13794	0.466	1.178	0.224	0.488	1.302	0.444	0.495	1.363	0.431
13795	0.266	-0.103	0.513	0.234	-0.336	0.501	0.224	-0.373	0.328
13796	0.295	0.082	0.513	0.196	-0.581	0.478	0.050	-1.485	0.450
13797	0.187	-0.608	0.224	0.313	0.172	0.462	0.224	-0.373	0.492
13798	0.432	0.958	0.328	0.462	1.133	0.512	0.503	1.413	0.513
13799	0.484	1.295	0.296	0.494	1.340	0.491	0.521	1.531	0.513
13800	0.184	-0.625	0.508	0.278	-0.054	0.398	0.142	-0.895	0.508
13801	0.505	1.430	0.481	0.497	1.359	0.273	0.511	1.464	0.512
13802	0.097	-1.181	0.492	0.234	-0.336	0.149	0.358	0.487	0.467
13803	0.495	1.363	0.481	0.488	1.302	0.501	0.429	0.942	0.185
13804	0.489	1.329	0.492	0.506	1.415	0.501	0.492	1.346	0.097
13805	0.097	-1.181	0.481	0.237	-0.317	0.478	0.224	-0.373	0.513
13806	0.184	-0.625	0.431	0.193	-0.600	0.462	0.263	-0.120	0.262
13807	0.224	-0.373	0.513	0.193	-0.600	0.491	0.263	-0.120	0.262
13808	0.184	-0.625	0.508	0.310	0.153	0.235	0.263	-0.120	0.385
13809	0.142	-0.895	0.224	0.234	-0.336	0.514	0.263	-0.120	0.481
13810	0.505	1.430	0.467	0.509	1.434	0.478	0.411	0.824	0.409
13811	0.487	1.312	0.467	0.500	1.378	0.149	0.513	1.481	0.512
13812	0.492	1.346	0.050	0.442	1.001	0.235	0.489	1.329	0.224
13813	0.492	1.346	0.262	0.497	1.359	0.149	0.505	1.430	0.097
13814	0.492	1.346	0.224	0.474	1.208	0.149	0.511	1.464	0.467
13815	0.492	1.346	0.224	0.465	1.152	0.309	0.466	1.178	0.512
13816	0.511	1.464	0.224	0.509	1.434	0.478	0.508	1.447	0.224
13817	0.450	1.076	0.296	0.491	1.321	0.273	0.508	1.447	0.142
13818	0.050	-1.485	0.508	0.398	0.718	0.462	0.145	-0.878	0.508
13819	0.226	-0.356	0.262	0.275	-0.073	0.478	0.050	-1.485	0.224
13820	0.358	0.487	0.262	0.149	-0.882	0.478	0.413	0.841	0.224
13821	0.492	1.346	0.262	0.500	1.378	0.512	0.484	1.295	0.508
13822	0.492	1.346	0.185	0.500	1.378	0.273	0.484	1.295	0.492
13823	0.500	1.397	0.142	0.477	1.227	0.422	0.505	1.430	0.450
13824	0.463	1.161	0.508	0.465	1.152	0.462	0.513	1.481	0.050
13825	0.511	1.464	0.185	0.488	1.302	0.501	0.450	1.076	0.050
13826	0.187	-0.608	0.481	0.237	-0.317	0.102	0.184	-0.625	0.492
13827	0.503	1.413	0.050	0.515	1.472	0.273	0.487	1.312	0.450
13828	0.495	1.363	0.467	0.480	1.246	0.149	0.503	1.413	0.142
13829	0.297	0.099	0.501	0.149	-0.882	0.235	0.097	-1.181	0.142
13830	0.295	0.082	0.492	0.234	-0.336	0.235	0.097	-1.181	0.492
13831	0.226	-0.356	0.508	0.149	-0.882	0.309	0.187	-0.608	0.185
13832	0.226	-0.356	0.467	0.149	-0.882	0.512	0.187	-0.608	0.508
13833	0.184	-0.625	0.467	0.310	0.153	0.462	0.382	0.638	0.142
13834	0.505	1.430	0.185	0.474	1.208	0.235	0.471	1.211	0.431
13835	0.332	0.318	0.512	0.275	-0.073	0.514	0.297	0.099	0.431
13836	0.450	1.076	0.328	0.459	1.114	0.501	0.466	1.178	0.142
13837	0.450	1.076	0.467	0.477	1.227	0.508	0.466	1.178	0.224
13838	0.466	1.178	0.185	0.477	1.227	0.491	0.466	1.178	0.185
13839	0.495	1.363	0.409	0.512	1.453	0.341	0.500	1.397	0.450
13840	0.329	0.301	0.501	0.275	-0.073	0.309	0.142	-0.895	0.508
13841	0.489	1.329	0.450	0.421	0.869	0.235	0.505	1.430	0.467
13842	0.503	1.413	0.224	0.468	1.170	0.235	0.508	1.447	0.385
13843	0.508	1.447	0.050	0.506	1.415	0.444	0.489	1.329	0.492

13844	0.266	-0.103	0.262	0.102	-1.184	0.514	0.184	-0.625	0.450
13845	0.297	0.099	0.296	0.272	-0.091	0.508	0.050	-1.485	0.501
13846	0.297	0.099	0.512	0.149	-0.882	0.309	0.097	-1.181	0.508
13847	0.226	-0.356	0.481	0.234	-0.336	0.478	0.142	-0.895	0.501
13848	0.229	-0.339	0.224	0.234	-0.336	0.491	0.142	-0.895	0.224
13849	0.226	-0.356	0.142	0.313	0.172	0.491	0.261	-0.137	0.467
13850	0.503	1.413	0.492	0.512	1.453	0.235	0.476	1.245	0.481
13851	0.466	1.178	0.512	0.462	1.133	0.102	0.474	1.228	0.296
13852	0.142	-0.895	0.512	0.237	-0.317	0.491	0.261	-0.137	0.142
13853	0.505	1.430	0.409	0.509	1.434	0.149	0.505	1.430	0.142
13854	0.453	1.093	0.050	0.503	1.396	0.512	0.466	1.178	0.142
13855	0.500	1.397	0.508	0.506	1.415	0.508	0.408	0.807	0.185
13856	0.429	0.942	0.328	0.491	1.321	0.478	0.508	1.447	0.224
13857	0.097	-1.181	0.450	0.342	0.361	0.478	0.184	-0.625	0.431
13858	0.297	0.099	0.142	0.310	0.153	0.501	0.224	-0.373	0.224
13859	0.224	-0.373	0.050	0.237	-0.317	0.491	0.145	-0.878	0.224
13860	0.224	-0.373	0.450	0.234	-0.336	0.514	0.261	-0.137	0.224
13861	0.500	1.397	0.512	0.439	0.982	0.462	0.489	1.329	0.262
13862	0.487	1.312	0.513	0.509	1.434	0.398	0.516	1.498	0.358
13863	0.511	1.464	0.513	0.506	1.415	0.422	0.508	1.447	0.513
13864	0.226	-0.356	0.501	0.310	0.153	0.514	0.450	1.076	0.224
13865	0.482	1.279	0.224	0.477	1.227	0.514	0.497	1.380	0.501
13866	0.450	1.076	0.185	0.491	1.321	0.309	0.503	1.413	0.142
13867	0.432	0.958	0.467	0.491	1.321	0.462	0.503	1.413	0.492
13868	0.184	-0.625	0.467	0.237	-0.317	0.462	0.142	-0.895	0.481
13869	0.300	0.116	0.262	0.102	-1.184	0.478	0.184	-0.625	0.481
13870	0.426	0.925	0.185	0.488	1.302	0.478	0.500	1.397	0.501
13871	0.263	-0.120	0.513	0.152	-0.863	0.512	0.142	-0.895	0.450
13872	0.461	1.144	0.508	0.512	1.453	0.235	0.497	1.380	0.450
13873	0.408	0.807	0.512	0.509	1.434	0.193	0.484	1.295	0.224
13874	0.511	1.464	0.501	0.477	1.227	0.149	0.497	1.380	0.431
13875	0.489	1.329	0.224	0.477	1.227	0.193	0.508	1.447	0.050
13876	0.450	1.076	0.328	0.503	1.396	0.514	0.508	1.447	0.512
13877	0.450	1.076	0.185	0.491	1.321	0.149	0.508	1.447	0.142
13878	0.450	1.076	0.185	0.515	1.472	0.508	0.508	1.447	0.050
13879	0.487	1.312	0.492	0.459	1.114	0.341	0.513	1.481	0.385
13880	0.097	-1.181	0.050	0.395	0.700	0.309	0.187	-0.608	0.508
13881	0.097	-1.181	0.492	0.418	0.850	0.235	0.187	-0.608	0.142
13882	0.511	1.464	0.492	0.512	1.453	0.193	0.411	0.824	0.185
13883	0.511	1.464	0.385	0.512	1.453	0.309	0.411	0.824	0.050
13884	0.142	-0.895	0.501	0.310	0.153	0.235	0.100	-1.164	0.224
13885	0.492	1.346	0.097	0.462	1.133	0.501	0.508	1.447	0.501
13886	0.500	1.397	0.467	0.462	1.133	0.514	0.487	1.312	0.508
13887	0.453	1.093	0.513	0.474	1.208	0.422	0.500	1.397	0.224
13888	0.450	1.076	0.097	0.474	1.208	0.273	0.463	1.161	0.097
13889	0.405	0.790	0.501	0.509	1.434	0.491	0.463	1.161	0.492
13890	0.142	-0.895	0.501	0.237	-0.317	0.193	0.187	-0.608	0.492
13891	0.297	0.099	0.296	0.196	-0.581	0.508	0.142	-0.895	0.492
13892	0.332	0.318	0.512	0.149	-0.882	0.512	0.097	-1.181	0.481
13893	0.266	-0.103	0.508	0.193	-0.600	0.102	0.097	-1.181	0.296
13894	0.503	1.413	0.296	0.515	1.472	0.371	0.408	0.807	0.501
13895	0.300	0.116	0.481	0.149	-0.882	0.309	0.142	-0.895	0.328
13896	0.505	1.430	0.481	0.506	1.415	0.309	0.511	1.464	0.512
13897	0.097	-1.181	0.431	0.339	0.342	0.273	0.142	-0.895	0.185
13898	0.297	0.099	0.224	0.313	0.172	0.491	0.097	-1.181	0.262
13899	0.145	-0.878	0.409	0.234	-0.336	0.422	0.224	-0.373	0.513
13900	0.329	0.301	0.467	0.193	-0.600	0.444	0.145	-0.878	0.512
13901	0.097	-1.181	0.142	0.310	0.153	0.508	0.142	-0.895	0.450
13902	0.224	-0.373	0.450	0.234	-0.336	0.512	0.097	-1.181	0.450
13903	0.489	1.329	0.492	0.503	1.396	0.149	0.505	1.430	0.481
13904	0.447	1.060	0.501	0.518	1.491	0.514	0.503	1.413	0.097

13905	0.489	1.329	0.142	0.424	0.888	0.508	0.495	1.363	0.262
13906	0.184	-0.625	0.296	0.275	-0.073	0.478	0.184	-0.625	0.409
13907	0.426	0.925	0.492	0.491	1.321	0.478	0.503	1.413	0.409
13908	0.142	-0.895	0.508	0.196	-0.581	0.491	0.263	-0.120	0.050
13909	0.489	1.329	0.262	0.509	1.434	0.478	0.468	1.194	0.185
13910	0.500	1.397	0.467	0.515	1.472	0.512	0.466	1.178	0.492
13911	0.229	-0.339	0.492	0.102	-1.184	0.235	0.261	-0.137	0.450
13912	0.097	-1.181	0.492	0.371	0.549	0.235	0.097	-1.181	0.431
13913	0.261	-0.137	0.513	0.307	0.135	0.478	0.142	-0.895	0.492
13914	0.295	0.082	0.328	0.307	0.135	0.501	0.142	-0.895	0.409
13915	0.295	0.082	0.262	0.272	-0.091	0.501	0.097	-1.181	0.262
13916	0.495	1.363	0.296	0.491	1.321	0.235	0.463	1.161	0.142
13917	0.489	1.329	0.296	0.424	0.888	0.462	0.516	1.498	0.142
13918	0.489	1.329	0.097	0.447	1.039	0.149	0.516	1.498	0.492
13919	0.466	1.178	0.513	0.509	1.434	0.478	0.461	1.144	0.097
13920	0.492	1.346	0.512	0.509	1.434	0.235	0.429	0.942	0.492
13921	0.145	-0.878	0.262	0.149	-0.882	0.235	0.326	0.285	0.492
13922	0.489	1.329	0.142	0.515	1.472	0.514	0.447	1.060	0.296
13923	0.466	1.178	0.450	0.506	1.415	0.491	0.489	1.329	0.513
13924	0.487	1.312	0.513	0.482	1.265	0.273	0.511	1.464	0.296
13925	0.487	1.312	0.262	0.482	1.265	0.149	0.511	1.464	0.513
13926	0.495	1.363	0.328	0.494	1.340	0.501	0.505	1.430	0.450
13927	0.495	1.363	0.481	0.482	1.265	0.149	0.508	1.447	0.185
13928	0.503	1.413	0.185	0.512	1.453	0.149	0.468	1.194	0.513
13929	0.300	0.116	0.224	0.234	-0.336	0.508	0.187	-0.608	0.492
13930	0.300	0.116	0.492	0.234	-0.336	0.508	0.097	-1.181	0.296
13931	0.503	1.413	0.512	0.477	1.227	0.478	0.511	1.464	0.481
13932	0.513	1.481	0.513	0.500	1.378	0.341	0.261	-0.137	0.481
13933	0.513	1.481	0.296	0.500	1.378	0.341	0.261	-0.137	0.142
13934	0.050	-1.485	0.481	0.240	-0.299	0.444	0.382	0.638	0.501
13935	0.408	0.807	0.450	0.465	1.152	0.514	0.484	1.295	0.513
13936	0.187	-0.608	0.358	0.149	-0.882	0.193	0.411	0.824	0.508
13937	0.500	1.397	0.262	0.474	1.208	0.193	0.513	1.481	0.142
13938	0.297	0.099	0.492	0.237	-0.317	0.462	0.226	-0.356	0.328
13939	0.224	-0.373	0.262	0.237	-0.317	0.149	0.097	-1.181	0.508
13940	0.492	1.346	0.262	0.515	1.472	0.501	0.466	1.178	0.224
13941	0.453	1.093	0.262	0.485	1.283	0.273	0.516	1.498	0.501
13942	0.097	-1.181	0.508	0.275	-0.073	0.501	0.226	-0.356	0.467
13943	0.261	-0.137	0.185	0.152	-0.863	0.501	0.142	-0.895	0.481
13944	0.476	1.245	0.185	0.506	1.415	0.193	0.503	1.413	0.142
13945	0.226	-0.356	0.501	0.149	-0.882	0.235	0.226	-0.356	0.224
13946	0.226	-0.356	0.358	0.149	-0.882	0.235	0.226	-0.356	0.492
13947	0.511	1.464	0.358	0.512	1.453	0.514	0.503	1.413	0.513
13948	0.505	1.430	0.513	0.515	1.472	0.501	0.487	1.312	0.431
13949	0.468	1.194	0.296	0.482	1.265	0.444	0.447	1.060	0.481
13950	0.142	-0.895	0.097	0.345	0.379	0.514	0.050	-1.485	0.481
13951	0.142	-0.895	0.508	0.345	0.379	0.512	0.050	-1.485	0.481
13952	0.468	1.194	0.450	0.442	1.001	0.501	0.484	1.295	0.409
13953	0.500	1.397	0.358	0.512	1.453	0.491	0.450	1.076	0.513
13954	0.261	-0.137	0.467	0.196	-0.581	0.462	0.142	-0.895	0.358
13955	0.224	-0.373	0.508	0.196	-0.581	0.444	0.142	-0.895	0.224
13956	0.508	1.447	0.481	0.462	1.133	0.398	0.489	1.329	0.224
13957	0.224	-0.373	0.492	0.152	-0.863	0.512	0.184	-0.625	0.185
13958	0.405	0.790	0.450	0.497	1.359	0.491	0.505	1.430	0.450
13959	0.226	-0.356	0.481	0.272	-0.091	0.193	0.142	-0.895	0.501
13960	0.511	1.464	0.224	0.500	1.378	0.193	0.426	0.925	0.185
13961	0.511	1.464	0.450	0.500	1.378	0.193	0.426	0.925	0.224
13962	0.224	-0.373	0.050	0.196	-0.581	0.193	0.142	-0.895	0.450
13963	0.326	0.285	0.050	0.234	-0.336	0.235	0.224	-0.373	0.431
13964	0.295	0.082	0.185	0.234	-0.336	0.309	0.184	-0.625	0.097
13965	0.511	1.464	0.296	0.512	1.453	0.478	0.450	1.076	0.142

13966	0.476	1.245	0.508	0.503	1.396	0.193	0.503	1.413	0.431
13967	0.492	1.346	0.142	0.439	0.982	0.193	0.466	1.178	0.296
13968	0.482	1.279	0.513	0.515	1.472	0.193	0.382	0.638	0.262
13969	0.495	1.363	0.431	0.515	1.472	0.193	0.487	1.312	0.508
13970	0.482	1.279	0.050	0.503	1.396	0.273	0.445	1.043	0.467
13971	0.432	0.958	0.185	0.494	1.340	0.462	0.492	1.346	0.508
13972	0.513	1.481	0.224	0.468	1.170	0.149	0.500	1.397	0.142
13973	0.508	1.447	0.142	0.447	1.039	0.514	0.495	1.363	0.450
13974	0.224	-0.373	0.224	0.401	0.737	0.341	0.226	-0.356	0.512
13975	0.466	1.178	0.501	0.518	1.491	0.478	0.466	1.178	0.409
13976	0.466	1.178	0.296	0.497	1.359	0.462	0.479	1.262	0.431
13977	0.050	-1.485	0.508	0.193	-0.600	0.478	0.300	0.116	0.467
13978	0.261	-0.137	0.097	0.196	-0.581	0.462	0.142	-0.895	0.185
13979	0.224	-0.373	0.296	0.196	-0.581	0.514	0.142	-0.895	0.185
13980	0.224	-0.373	0.512	0.196	-0.581	0.514	0.142	-0.895	0.142
13981	0.226	-0.356	0.328	0.234	-0.336	0.193	0.184	-0.625	0.492
13982	0.297	0.099	0.296	0.313	0.172	0.508	0.224	-0.373	0.431
13983	0.508	1.447	0.262	0.482	1.265	0.309	0.432	0.958	0.450
13984	0.261	-0.137	0.296	0.196	-0.581	0.501	0.224	-0.373	0.450
13985	0.261	-0.137	0.501	0.196	-0.581	0.235	0.224	-0.373	0.185
13986	0.261	-0.137	0.296	0.196	-0.581	0.273	0.224	-0.373	0.185
13987	0.184	-0.625	0.385	0.193	-0.600	0.478	0.263	-0.120	0.492
13988	0.142	-0.895	0.492	0.275	-0.073	0.508	0.361	0.504	0.328
13989	0.497	1.380	0.450	0.462	1.133	0.193	0.513	1.481	0.262
13990	0.184	-0.625	0.512	0.149	-0.882	0.309	0.224	-0.373	0.492
13991	0.482	1.279	0.512	0.520	1.509	0.273	0.503	1.413	0.513
13992	0.050	-1.485	0.142	0.345	0.379	0.193	0.142	-0.895	0.097
13993	0.463	1.161	0.512	0.482	1.265	0.462	0.497	1.380	0.481
13994	0.503	1.413	0.481	0.465	1.152	0.514	0.474	1.228	0.492
13995	0.492	1.346	0.467	0.480	1.246	0.149	0.474	1.228	0.512
13996	0.508	1.447	0.296	0.465	1.152	0.422	0.492	1.346	0.512
13997	0.466	1.178	0.512	0.515	1.472	0.501	0.445	1.043	0.328
13998	0.466	1.178	0.142	0.515	1.472	0.508	0.445	1.043	0.508
13999	0.187	-0.608	0.385	0.193	-0.600	0.512	0.224	-0.373	0.097
14000	0.505	1.430	0.508	0.509	1.434	0.478	0.426	0.925	0.450
14001	0.329	0.301	0.142	0.310	0.153	0.235	0.050	-1.485	0.185
14002	0.463	1.161	0.492	0.503	1.396	0.491	0.513	1.481	0.262
14003	0.184	-0.625	0.262	0.237	-0.317	0.235	0.142	-0.895	0.296
14004	0.408	0.807	0.481	0.275	-0.073	0.501	0.050	-1.485	0.262
14005	0.500	1.397	0.512	0.474	1.208	0.309	0.384	0.655	0.501
14006	0.450	1.076	0.224	0.509	1.434	0.235	0.508	1.447	0.224
14007	0.226	-0.356	0.467	0.193	-0.600	0.491	0.142	-0.895	0.450
14008	0.050	-1.485	0.185	0.310	0.153	0.491	0.184	-0.625	0.508
14009	0.263	-0.120	0.224	0.275	-0.073	0.501	0.050	-1.485	0.385
14010	0.297	0.099	0.224	0.196	-0.581	0.508	0.224	-0.373	0.185
14011	0.511	1.464	0.224	0.462	1.133	0.398	0.497	1.380	0.224
14012	0.482	1.279	0.224	0.515	1.472	0.273	0.508	1.447	0.185
14013	0.224	-0.373	0.450	0.149	-0.882	0.193	0.224	-0.373	0.224
14014	0.142	-0.895	0.050	0.424	0.888	0.512	0.097	-1.181	0.481
14015	0.487	1.312	0.050	0.497	1.359	0.512	0.492	1.346	0.262
14016	0.508	1.447	0.513	0.506	1.415	0.235	0.489	1.329	0.508
14017	0.508	1.447	0.467	0.509	1.434	0.193	0.489	1.329	0.050
14018	0.405	0.790	0.508	0.474	1.208	0.512	0.476	1.245	0.050
14019	0.050	-1.485	0.450	0.234	-0.336	0.508	0.300	0.116	0.296
14020	0.497	1.380	0.501	0.494	1.340	0.462	0.505	1.430	0.467
14021	0.334	0.335	0.508	0.234	-0.336	0.491	0.329	0.301	0.224
14022	0.450	1.076	0.508	0.500	1.378	0.341	0.511	1.464	0.097
14023	0.145	-0.878	0.385	0.310	0.153	0.398	0.184	-0.625	0.097
14024	0.050	-1.485	0.481	0.234	-0.336	0.235	0.263	-0.120	0.142
14025	0.453	1.093	0.224	0.491	1.321	0.235	0.513	1.481	0.385
14026	0.505	1.430	0.262	0.485	1.283	0.508	0.513	1.481	0.185

14027	0.511	1.464	0.328	0.500	1.378	0.398	0.450	1.076	0.050
14028	0.511	1.464	0.467	0.506	1.415	0.508	0.450	1.076	0.508
14029	0.497	1.380	0.224	0.395	0.700	0.508	0.479	1.262	0.501
14030	0.224	−0.373	0.508	0.275	−0.073	0.193	0.100	−1.164	0.450
14031	0.184	−0.625	0.358	0.196	−0.581	0.508	0.261	−0.137	0.481
14032	0.466	1.178	0.262	0.509	1.434	0.193	0.405	0.790	0.142
14033	0.466	1.178	0.296	0.509	1.434	0.478	0.405	0.790	0.512
14034	0.261	−0.137	0.262	0.234	−0.336	0.501	0.050	−1.485	0.513
14035	0.187	−0.608	0.492	0.193	−0.600	0.309	0.184	−0.625	0.513
14036	0.511	1.464	0.481	0.512	1.453	0.514	0.489	1.329	0.050
14037	0.505	1.430	0.224	0.509	1.434	0.508	0.447	1.060	0.512
14038	0.511	1.464	0.501	0.465	1.152	0.102	0.429	0.942	0.185
14039	0.492	1.346	0.508	0.491	1.321	0.508	0.492	1.346	0.431
14040	0.226	−0.356	0.512	0.339	0.342	0.514	0.411	0.824	0.512
14041	0.332	0.318	0.508	0.398	0.718	0.309	0.261	−0.137	0.185
14042	0.187	−0.608	0.185	0.234	−0.336	0.512	0.142	−0.895	0.513
14043	0.187	−0.608	0.492	0.234	−0.336	0.508	0.142	−0.895	0.142
14044	0.487	1.312	0.097	0.509	1.434	0.501	0.489	1.329	0.508
14045	0.050	−1.485	0.185	0.401	0.737	0.235	0.097	−1.181	0.508
14046	0.487	1.312	0.492	0.509	1.434	0.149	0.489	1.329	0.501
14047	0.487	1.312	0.508	0.509	1.434	0.491	0.489	1.329	0.262
14048	0.387	0.672	0.508	0.193	−0.600	0.309	0.297	0.099	0.142
14049	0.497	1.380	0.050	0.509	1.434	0.491	0.508	1.447	0.097
14050	0.097	−1.181	0.097	0.196	−0.581	0.501	0.297	0.099	0.097
14051	0.463	1.161	0.513	0.480	1.246	0.508	0.505	1.430	0.097
14052	0.511	1.464	0.185	0.494	1.340	0.273	0.447	1.060	0.492
14053	0.097	−1.181	0.467	0.310	0.153	0.309	0.184	−0.625	0.224
14054	0.503	1.413	0.492	0.515	1.472	0.491	0.511	1.464	0.409
14055	0.500	1.397	0.492	0.506	1.415	0.478	0.489	1.329	0.508
14056	0.300	0.116	0.492	0.102	−1.184	0.193	0.297	0.099	0.501
14057	0.508	1.447	0.501	0.503	1.396	0.512	0.479	1.262	0.501
14058	0.505	1.430	0.492	0.512	1.453	0.512	0.479	1.262	0.501
14059	0.297	0.099	0.142	0.313	0.172	0.512	0.142	−0.895	0.492
14060	0.484	1.295	0.467	0.518	1.491	0.508	0.503	1.413	0.501
14061	0.482	1.279	0.508	0.500	1.378	0.341	0.511	1.464	0.467
14062	0.429	0.942	0.512	0.497	1.359	0.235	0.503	1.413	0.467
14063	0.224	−0.373	0.296	0.234	−0.336	0.371	0.142	−0.895	0.492
14064	0.413	0.841	0.409	0.149	−0.882	0.422	0.329	0.301	0.296
14065	0.463	1.161	0.481	0.491	1.321	0.273	0.503	1.413	0.050
14066	0.142	−0.895	0.492	0.310	0.153	0.501	0.226	−0.356	0.142
14067	0.447	1.060	0.501	0.488	1.302	0.512	0.497	1.380	0.467
14068	0.489	1.329	0.512	0.497	1.359	0.102	0.466	1.178	0.296
14069	0.503	1.413	0.512	0.506	1.415	0.273	0.476	1.245	0.262
14070	0.142	−0.895	0.501	0.272	−0.091	0.512	0.142	−0.895	0.185
14071	0.297	0.099	0.450	0.310	0.153	0.491	0.224	−0.373	0.185
14072	0.492	1.346	0.492	0.488	1.302	0.341	0.492	1.346	0.050
14073	0.505	1.430	0.142	0.474	1.208	0.235	0.511	1.464	0.142
14074	0.263	−0.120	0.512	0.196	−0.581	0.501	0.432	0.958	0.492
14075	0.466	1.178	0.512	0.512	1.453	0.193	0.479	1.262	0.467
14076	0.492	1.346	0.512	0.512	1.453	0.149	0.479	1.262	0.142
14077	0.492	1.346	0.467	0.512	1.453	0.478	0.479	1.262	0.512
14078	0.511	1.464	0.501	0.503	1.396	0.478	0.416	0.857	0.501
14079	0.326	0.285	0.501	0.339	0.342	0.501	0.516	1.498	0.512
14080	0.263	−0.120	0.512	0.237	−0.317	0.273	0.358	0.487	0.501
14081	0.297	0.099	0.262	0.371	0.549	0.478	0.224	−0.373	0.328
14082	0.297	0.099	0.142	0.424	0.888	0.501	0.224	−0.373	0.481
14083	0.097	−1.181	0.185	0.278	−0.054	0.193	0.184	−0.625	0.296
14084	0.505	1.430	0.385	0.503	1.396	0.193	0.447	1.060	0.050
14085	0.505	1.430	0.512	0.515	1.472	0.501	0.503	1.413	0.467
14086	0.266	−0.103	0.513	0.102	−1.184	0.193	0.184	−0.625	0.450
14087	0.142	−0.895	0.296	0.272	−0.091	0.193	0.226	−0.356	0.450

14088	0.450	1.076	0.501	0.512	1.453	0.149	0.450	1.076	0.296
14089	0.508	1.447	0.262	0.491	1.321	0.444	0.429	0.942	0.358
14090	0.261	-0.137	0.224	0.348	0.398	0.235	0.097	-1.181	0.450
14091	0.329	0.301	0.450	0.237	-0.317	0.053	0.145	-0.878	0.385
14092	0.484	1.295	0.512	0.497	1.359	0.512	0.434	0.975	0.513
14093	0.184	-0.625	0.467	0.196	-0.581	0.235	0.297	0.099	0.513
14094	0.226	-0.356	0.512	0.149	-0.882	0.235	0.263	-0.120	0.513
14095	0.492	1.346	0.224	0.480	1.246	0.444	0.503	1.413	0.513
14096	0.511	1.464	0.513	0.480	1.246	0.309	0.461	1.144	0.513
14097	0.511	1.464	0.431	0.500	1.378	0.273	0.503	1.413	0.501
14098	0.300	0.116	0.262	0.275	-0.073	0.491	0.142	-0.895	0.142
14099	0.479	1.262	0.492	0.480	1.246	0.444	0.450	1.076	0.492
14100	0.453	1.093	0.481	0.500	1.378	0.193	0.511	1.464	0.508
14101	0.358	0.487	0.467	0.193	-0.600	0.501	0.411	0.824	0.501
14102	0.261	-0.137	0.467	0.196	-0.581	0.235	0.432	0.958	0.185
14103	0.497	1.380	0.185	0.503	1.396	0.444	0.466	1.178	0.481
14104	0.263	-0.120	0.512	0.193	-0.600	0.462	0.184	-0.625	0.467
14105	0.263	-0.120	0.501	0.193	-0.600	0.514	0.184	-0.625	0.450
14106	0.263	-0.120	0.512	0.149	-0.882	0.508	0.142	-0.895	0.481
14107	0.503	1.413	0.481	0.447	1.039	0.235	0.492	1.346	0.513
14108	0.187	-0.608	0.262	0.237	-0.317	0.501	0.429	0.942	0.513
14109	0.187	-0.608	0.262	0.053	-1.504	0.309	0.447	1.060	0.467
14110	0.505	1.430	0.358	0.515	1.472	0.235	0.453	1.093	0.512
14111	0.358	0.487	0.185	0.240	-0.299	0.341	0.184	-0.625	0.467
14112	0.358	0.487	0.431	0.240	-0.299	0.371	0.184	-0.625	0.328
14113	0.513	1.481	0.262	0.444	1.020	0.501	0.495	1.363	0.431
14114	0.297	0.099	0.481	0.310	0.153	0.371	0.332	0.318	0.492
14115	0.097	-1.181	0.492	0.272	-0.091	0.491	0.263	-0.120	0.492
14116	0.505	1.430	0.492	0.485	1.283	0.491	0.492	1.346	0.513
14117	0.450	1.076	0.185	0.442	1.001	0.309	0.511	1.464	0.508
14118	0.361	0.504	0.262	0.193	-0.600	0.309	0.097	-1.181	0.508
14119	0.463	1.161	0.492	0.497	1.359	0.102	0.482	1.279	0.501
14120	0.505	1.430	0.501	0.234	-0.336	0.235	0.489	1.329	0.097
14121	0.479	1.262	0.512	0.447	1.039	0.341	0.511	1.464	0.185
14122	0.492	1.346	0.224	0.468	1.170	0.444	0.511	1.464	0.262
14123	0.450	1.076	0.224	0.512	1.453	0.235	0.326	0.285	0.185
14124	0.479	1.262	0.224	0.509	1.434	0.491	0.508	1.447	0.492
14125	0.224	-0.373	0.513	0.234	-0.336	0.371	0.097	-1.181	0.409
14126	0.453	1.093	0.450	0.503	1.396	0.273	0.447	1.060	0.050
14127	0.050	-1.485	0.492	0.310	0.153	0.309	0.184	-0.625	0.450
14128	0.050	-1.485	0.481	0.234	-0.336	0.444	0.261	-0.137	0.185
14129	0.184	-0.625	0.492	0.345	0.379	0.273	0.295	0.082	0.328
14130	0.297	0.099	0.492	0.371	0.549	0.149	0.261	-0.137	0.481
14131	0.500	1.397	0.512	0.500	1.378	0.193	0.500	1.397	0.512
14132	0.142	-0.895	0.481	0.371	0.549	0.193	0.224	-0.373	0.481
14133	0.508	1.447	0.508	0.494	1.340	0.462	0.453	1.093	0.508
14134	0.426	0.925	0.512	0.491	1.321	0.102	0.511	1.464	0.185
14135	0.050	-1.485	0.501	0.310	0.153	0.273	0.387	0.672	0.481
14136	0.187	-0.608	0.050	0.310	0.153	0.462	0.184	-0.625	0.512
14137	0.224	-0.373	0.492	0.105	-1.165	0.512	0.224	-0.373	0.097
14138	0.142	-0.895	0.492	0.234	-0.336	0.508	0.184	-0.625	0.492
14139	0.226	-0.356	0.508	0.342	0.361	0.508	0.226	-0.356	0.501
14140	0.492	1.346	0.185	0.444	1.020	0.273	0.482	1.279	0.492
14141	0.300	0.116	0.481	0.237	-0.317	0.501	0.261	-0.137	0.501
14142	0.497	1.380	0.481	0.491	1.321	0.462	0.508	1.447	0.224
14143	0.097	-1.181	0.492	0.374	0.568	0.462	0.050	-1.485	0.450
14144	0.295	0.082	0.508	0.275	-0.073	0.193	0.050	-1.485	0.508
14145	0.000	?	0.142	0.310	0.153	0.462	0.297	0.099	0.481
14146	0.503	1.413	0.492	0.450	1.057	0.235	0.466	1.178	0.501
14147	0.329	0.301	0.185	0.275	-0.073	0.309	0.224	-0.373	0.097
14148	0.297	0.099	0.512	0.149	-0.882	0.371	0.097	-1.181	0.097

14149	0.261	−0.137	0.481	0.193	−0.600	0.273	0.097	−1.181	0.481
14150	0.297	0.099	0.481	0.193	−0.600	0.491	0.142	−0.895	0.262
14151	0.500	1.397	0.185	0.465	1.152	0.193	0.382	0.638	0.508
14152	0.295	0.082	0.508	0.102	−1.184	0.501	0.184	−0.625	0.185
14153	0.384	0.655	0.097	0.275	−0.073	0.462	0.050	−1.485	0.492
14154	0.495	1.363	0.185	0.462	1.133	0.341	0.500	1.397	0.481
14155	0.447	1.060	0.492	0.515	1.472	0.501	0.497	1.380	0.481
14156	0.500	1.397	0.513	0.512	1.453	0.193	0.447	1.060	0.296
14157	0.500	1.397	0.385	0.512	1.453	0.273	0.479	1.262	0.262
14158	0.142	−0.895	0.512	0.275	−0.073	0.235	0.142	−0.895	0.513
14159	0.511	1.464	0.262	0.500	1.378	0.235	0.508	1.447	0.481
14160	0.482	1.279	0.262	0.465	1.152	0.235	0.505	1.430	0.501
14161	0.468	1.194	0.467	0.465	1.152	0.491	0.505	1.430	0.097
14162	0.297	0.099	0.492	0.193	−0.600	0.341	0.050	−1.485	0.224
14163	0.508	1.447	0.467	0.462	1.133	0.235	0.508	1.447	0.142
14164	0.142	−0.895	0.481	0.237	−0.317	0.491	0.184	−0.625	0.431
14165	0.384	0.655	0.224	0.310	0.153	0.422	0.426	0.925	0.513
14166	0.505	1.430	0.492	0.371	0.549	0.462	0.511	1.464	0.450
14167	0.142	−0.895	0.328	0.272	−0.091	0.422	0.184	−0.625	0.492
14168	0.492	1.346	0.513	0.485	1.283	0.512	0.508	1.447	0.512
14169	0.263	−0.120	0.142	0.193	−0.600	0.512	0.142	−0.895	0.492
14170	0.482	1.279	0.492	0.500	1.378	0.478	0.497	1.380	0.508
14171	0.511	1.464	0.492	0.465	1.152	0.501	0.497	1.380	0.508
14172	0.224	−0.373	0.262	0.342	0.361	0.422	0.000	?	0.508
14173	0.471	1.211	0.050	0.500	1.378	0.235	0.497	1.380	0.467
14174	0.187	−0.608	0.224	0.196	−0.581	0.309	0.261	−0.137	0.508
14175	0.224	−0.373	0.508	0.272	−0.091	0.444	0.142	−0.895	0.328
14176	0.226	−0.356	0.512	0.237	−0.317	0.508	0.097	−1.181	0.501
14177	0.226	−0.356	0.050	0.237	−0.317	0.149	0.097	−1.181	0.512
14178	0.226	−0.356	0.050	0.237	−0.317	0.512	0.097	−1.181	0.467
14179	0.447	1.060	0.431	0.497	1.359	0.273	0.487	1.312	0.358
14180	0.050	−1.485	0.142	0.345	0.379	0.149	0.224	−0.373	0.358
14181	0.050	−1.485	0.508	0.237	−0.317	0.193	0.411	0.824	0.508
14182	0.503	1.413	0.512	0.485	1.283	0.273	0.513	1.481	0.508
14183	0.468	1.194	0.296	0.421	0.869	0.422	0.492	1.346	0.385
14184	0.511	1.464	0.501	0.462	1.133	0.512	0.492	1.346	0.296
14185	0.450	1.076	0.185	0.424	0.888	0.341	0.492	1.346	0.467
14186	0.497	1.380	0.481	0.506	1.415	0.508	0.492	1.346	0.296
14187	0.497	1.380	0.142	0.518	1.491	0.444	0.500	1.397	0.492
14188	0.497	1.380	0.431	0.482	1.265	0.491	0.466	1.178	0.501
14189	0.384	0.655	0.512	0.500	1.378	0.462	0.461	1.144	0.508
14190	0.484	1.295	0.224	0.421	0.869	0.193	0.484	1.295	0.224
14191	0.226	−0.356	0.512	0.234	−0.336	0.491	0.297	0.099	0.431
14192	0.263	−0.120	0.358	0.310	0.153	0.193	0.050	−1.485	0.385
14193	0.326	0.285	0.385	0.447	1.039	0.341	0.142	−0.895	0.142
14194	0.466	1.178	0.481	0.506	1.415	0.512	0.468	1.194	0.481
14195	0.224	−0.373	0.185	0.149	−0.882	0.422	0.295	0.082	0.508
14196	0.511	1.464	0.492	0.509	1.434	0.478	0.263	−0.120	0.142
14197	0.358	0.487	0.450	0.275	−0.073	0.235	0.184	−0.625	0.467
14198	0.263	−0.120	0.097	0.149	−0.882	0.273	0.184	−0.625	0.224
14199	0.297	0.099	0.513	0.193	−0.600	0.444	0.050	−1.485	0.224
14200	0.263	−0.120	0.492	0.275	−0.073	0.235	0.142	−0.895	0.512
14201	0.489	1.329	0.142	0.424	0.888	0.462	0.489	1.329	0.481
14202	0.479	1.262	0.501	0.506	1.415	0.398	0.466	1.178	0.481
14203	0.226	−0.356	0.512	0.342	0.361	0.398	0.142	−0.895	0.492
14204	0.500	1.397	0.097	0.509	1.434	0.422	0.503	1.413	0.097
14205	0.505	1.430	0.508	0.444	1.020	0.491	0.503	1.413	0.409
14206	0.508	1.447	0.508	0.494	1.340	0.512	0.511	1.464	0.224
14207	0.505	1.430	0.262	0.462	1.133	0.273	0.503	1.413	0.501
14208	0.187	−0.608	0.513	0.196	−0.581	0.501	0.326	0.285	0.185
14209	0.492	1.346	0.467	0.500	1.378	0.444	0.479	1.262	0.501

14210	0.097	-1.181	0.492	0.196	-0.581	0.512	0.297	0.099	0.508
14211	0.184	-0.625	0.262	0.342	0.361	0.235	0.050	-1.485	0.097
14212	0.489	1.329	0.262	0.512	1.453	0.422	0.463	1.161	0.262
14213	0.505	1.430	0.481	0.421	0.869	0.501	0.447	1.060	0.142
14214	0.505	1.430	0.358	0.477	1.227	0.501	0.447	1.060	0.508
14215	0.050	-1.485	0.358	0.234	-0.336	0.512	0.297	0.099	0.409
14216	0.100	-1.164	0.450	0.272	-0.091	0.501	0.358	0.487	0.185
14217	0.503	1.413	0.508	0.447	1.039	0.501	0.447	1.060	0.185
14218	0.184	-0.625	0.492	0.234	-0.336	0.501	0.384	0.655	0.450
14219	0.471	1.211	0.501	0.462	1.133	0.508	0.503	1.413	0.328
14220	0.492	1.346	0.050	0.398	0.718	0.491	0.511	1.464	0.450
14221	0.492	1.346	0.050	0.398	0.718	0.273	0.511	1.464	0.508
14222	0.492	1.346	0.050	0.421	0.869	0.491	0.511	1.464	0.328
14223	0.503	1.413	0.050	0.491	1.321	0.501	0.511	1.464	0.512
14224	0.492	1.346	0.501	0.515	1.472	0.501	0.492	1.346	0.262
14225	0.142	-0.895	0.050	0.275	-0.073	0.512	0.142	-0.895	0.501
14226	0.468	1.194	0.142	0.503	1.396	0.501	0.492	1.346	0.262
14227	0.505	1.430	0.050	0.442	1.001	0.491	0.503	1.413	0.450
14228	0.511	1.464	0.142	0.512	1.453	0.478	0.500	1.397	0.512
14229	0.297	0.099	0.512	0.234	-0.336	0.235	0.184	-0.625	0.185
14230	0.411	0.824	0.501	0.427	0.907	0.235	0.479	1.262	0.492
14231	0.476	1.245	0.097	0.500	1.378	0.102	0.468	1.194	0.450
14232	0.489	1.329	0.481	0.509	1.434	0.235	0.445	1.043	0.450
14233	0.497	1.380	0.501	0.520	1.509	0.444	0.476	1.245	0.431
14234	0.513	1.481	0.501	0.500	1.378	0.478	0.503	1.413	0.224
14235	0.513	1.481	0.467	0.500	1.378	0.235	0.503	1.413	0.385
14236	0.503	1.413	0.508	0.497	1.359	0.501	0.468	1.194	0.512
14237	0.447	1.060	0.508	0.506	1.415	0.102	0.508	1.447	0.385
14238	0.492	1.346	0.512	0.494	1.340	0.273	0.471	1.211	0.513
14239	0.142	-0.895	0.097	0.278	-0.054	0.512	0.329	0.301	0.481
14240	0.508	1.447	0.050	0.494	1.340	0.444	0.426	0.925	0.050
14241	0.508	1.447	0.508	0.494	1.340	0.512	0.492	1.346	0.513
14242	0.508	1.447	0.508	0.494	1.340	0.422	0.492	1.346	0.513
14243	0.463	1.161	0.508	0.506	1.415	0.235	0.513	1.481	0.050
14244	0.497	1.380	0.328	0.494	1.340	0.512	0.508	1.447	0.513
14245	0.497	1.380	0.492	0.485	1.283	0.501	0.508	1.447	0.513
14246	0.511	1.464	0.501	0.474	1.208	0.341	0.497	1.380	0.224
14247	0.261	-0.137	0.431	0.234	-0.336	0.478	0.097	-1.181	0.450
14248	0.142	-0.895	0.185	0.234	-0.336	0.478	0.184	-0.625	0.481
14249	0.184	-0.625	0.328	0.102	-1.184	0.462	0.263	-0.120	0.508
14250	0.384	0.655	0.050	0.237	-0.317	0.462	0.184	-0.625	0.185
14251	0.505	1.430	0.450	0.444	1.020	0.398	0.495	1.363	0.185
14252	0.513	1.481	0.050	0.482	1.265	0.512	0.411	0.824	0.481
14253	0.295	0.082	0.097	0.234	-0.336	0.462	0.050	-1.485	0.050
14254	0.508	1.447	0.185	0.494	1.340	0.512	0.447	1.060	0.513
14255	0.261	-0.137	0.512	0.102	-1.184	0.478	0.184	-0.625	0.508
14256	0.224	-0.373	0.513	0.275	-0.073	0.235	0.326	0.285	0.512
14257	0.447	1.060	0.513	0.509	1.434	0.273	0.479	1.262	0.492
14258	0.511	1.464	0.508	0.444	1.020	0.501	0.511	1.464	0.224
14259	0.466	1.178	0.050	0.518	1.491	0.193	0.479	1.262	0.224
14260	0.511	1.464	0.185	0.424	0.888	0.501	0.513	1.481	0.224
14261	0.224	-0.373	0.185	0.237	-0.317	0.102	0.184	-0.625	0.050
14262	0.511	1.464	0.492	0.506	1.415	0.501	0.482	1.279	0.508
14263	0.432	0.958	0.481	0.503	1.396	0.478	0.505	1.430	0.142
14264	0.263	-0.120	0.513	0.345	0.379	0.398	0.097	-1.181	0.142
14265	0.492	1.346	0.513	0.474	1.208	0.341	0.497	1.380	0.097
14266	0.479	1.262	0.050	0.480	1.246	0.235	0.495	1.363	0.142
14267	0.466	1.178	0.492	0.462	1.133	0.514	0.489	1.329	0.262
14268	0.466	1.178	0.050	0.462	1.133	0.514	0.497	1.380	0.481
14269	0.184	-0.625	0.512	0.404	0.756	0.478	0.224	-0.373	0.501
14270	0.511	1.464	0.512	0.512	1.453	0.235	0.447	1.060	0.142

14271	0.500	1.397	0.508	0.459	1.114	0.235	0.503	1.413	0.450
14272	0.508	1.447	0.501	0.512	1.453	0.193	0.482	1.279	0.450
14273	0.476	1.245	0.512	0.477	1.227	0.501	0.497	1.380	0.467
14274	0.263	−0.120	0.512	0.234	−0.336	0.478	0.097	−1.181	0.358
14275	0.263	−0.120	0.512	0.275	−0.073	0.501	0.097	−1.181	0.492
14276	0.358	0.487	0.358	0.512	1.453	0.478	0.479	1.262	0.512
14277	0.184	−0.625	0.481	0.196	−0.581	0.444	0.261	−0.137	0.512
14278	0.434	0.975	0.097	0.500	1.378	0.478	0.503	1.413	0.142
14279	0.261	−0.137	0.513	0.105	−1.165	0.478	0.184	−0.625	0.224
14280	0.476	1.245	0.508	0.497	1.359	0.478	0.489	1.329	0.512
14281	0.489	1.329	0.296	0.474	1.208	0.508	0.479	1.262	0.467
14282	0.489	1.329	0.224	0.401	0.737	0.501	0.479	1.262	0.467
14283	0.184	−0.625	0.508	0.342	0.361	0.422	0.297	0.099	0.467
14284	0.263	−0.120	0.481	0.237	−0.317	0.235	0.263	−0.120	0.431
14285	0.489	1.329	0.512	0.509	1.434	0.501	0.511	1.464	0.409
14286	0.500	1.397	0.513	0.509	1.434	0.508	0.476	1.245	0.467
14287	0.511	1.464	0.097	0.480	1.246	0.501	0.497	1.380	0.185
14288	0.226	−0.356	0.097	0.237	−0.317	0.053	0.097	−1.181	0.097
14289	0.226	−0.356	0.097	0.237	−0.317	0.508	0.224	−0.373	0.224
14290	0.224	−0.373	0.513	0.193	−0.600	0.478	0.142	−0.895	0.512
14291	0.511	1.464	0.508	0.503	1.396	0.478	0.434	0.975	0.296
14292	0.447	1.060	0.512	0.482	1.265	0.462	0.518	1.514	0.328
14293	0.489	1.329	0.512	0.500	1.378	0.102	0.450	1.076	0.185
14294	0.476	1.245	0.512	0.482	1.265	0.501	0.497	1.380	0.513
14295	0.492	1.346	0.512	0.442	1.001	0.273	0.505	1.430	0.501
14296	0.489	1.329	0.501	0.477	1.227	0.508	0.492	1.346	0.224
14297	0.511	1.464	0.481	0.477	1.227	0.514	0.508	1.447	0.508
14298	0.474	1.228	0.328	0.477	1.227	0.512	0.508	1.447	0.481
14299	0.508	1.447	0.512	0.509	1.434	0.273	0.508	1.447	0.296
14300	0.511	1.464	0.296	0.500	1.378	0.491	0.463	1.161	0.385
14301	0.503	1.413	0.492	0.421	0.869	0.235	0.508	1.447	0.385
14302	0.050	−1.485	0.481	0.234	−0.336	0.309	0.332	0.318	0.467
14303	0.489	1.329	0.050	0.494	1.340	0.514	0.503	1.413	0.508
14304	0.489	1.329	0.185	0.506	1.415	0.462	0.508	1.447	0.328
14305	0.508	1.447	0.508	0.500	1.378	0.371	0.466	1.178	0.467
14306	0.184	−0.625	0.508	0.053	−1.504	0.512	0.361	0.504	0.450
14307	0.479	1.262	0.508	0.506	1.415	0.235	0.358	0.487	0.185
14308	0.476	1.245	0.508	0.477	1.227	0.235	0.508	1.447	0.512
14309	0.487	1.312	0.262	0.477	1.227	0.508	0.508	1.447	0.050
14310	0.511	1.464	0.481	0.462	1.133	0.478	0.382	0.638	0.385
14311	0.142	−0.895	0.492	0.105	−1.165	0.512	0.295	0.082	0.385
14312	0.495	1.363	0.492	0.494	1.340	0.512	0.466	1.178	0.501
14313	0.187	−0.608	0.481	0.272	−0.091	0.273	0.300	0.116	0.501
14314	0.511	1.464	0.481	0.509	1.434	0.422	0.487	1.312	0.512
14315	0.479	1.262	0.508	0.512	1.453	0.273	0.495	1.363	0.385
14316	0.482	1.279	0.431	0.515	1.472	0.491	0.497	1.380	0.385
14317	0.184	−0.625	0.467	0.275	−0.073	0.235	0.224	−0.373	0.385
14318	0.503	1.413	0.467	0.494	1.340	0.501	0.432	0.958	0.467
14319	0.097	−1.181	0.508	0.237	−0.317	0.444	0.387	0.672	0.050
14320	0.184	−0.625	0.508	0.313	0.172	0.235	0.142	−0.895	0.328
14321	0.495	1.363	0.501	0.515	1.472	0.235	0.482	1.279	0.492
14322	0.513	1.481	0.512	0.459	1.114	0.193	0.503	1.413	0.142
14323	0.387	0.672	0.508	0.374	0.568	0.444	0.142	−0.895	0.467
14324	0.505	1.430	0.508	0.509	1.434	0.512	0.466	1.178	0.409
14325	0.263	−0.120	0.513	0.237	−0.317	0.371	0.226	−0.356	0.097
14326	0.263	−0.120	0.512	0.237	−0.317	0.309	0.226	−0.356	0.050
14327	0.463	1.161	0.512	0.506	1.415	0.508	0.513	1.481	0.492
14328	0.489	1.329	0.358	0.477	1.227	0.422	0.482	1.279	0.512
14329	0.468	1.194	0.328	0.512	1.453	0.491	0.482	1.279	0.492
14330	0.482	1.279	0.501	0.512	1.453	0.444	0.489	1.329	0.501
14331	0.226	−0.356	0.492	0.272	−0.091	0.273	0.097	−1.181	0.328

14332	0.495	1.363	0.224	0.424	0.888	0.478	0.408	0.807	0.328
14333	0.329	0.301	0.431	0.275	-0.073	0.149	0.224	-0.373	0.328
14334	0.516	1.498	0.508	0.494	1.340	0.512	0.503	1.413	0.492
14335	0.142	-0.895	0.481	0.237	-0.317	0.309	0.184	-0.625	0.492
14336	0.492	1.346	0.467	0.494	1.340	0.478	0.500	1.397	0.501
14337	0.489	1.329	0.328	0.442	1.001	0.478	0.513	1.481	0.512
14338	0.261	-0.137	0.224	0.237	-0.317	0.149	0.097	-1.181	0.508
14339	0.050	-1.485	0.481	0.237	-0.317	0.371	0.263	-0.120	0.512
14340	0.224	-0.373	0.481	0.193	-0.600	0.235	0.142	-0.895	0.501
14341	0.508	1.447	0.512	0.447	1.039	0.193	0.513	1.481	0.492
14342	0.511	1.464	0.296	0.509	1.434	0.508	0.405	0.790	0.262
14343	0.050	-1.485	0.508	0.371	0.549	0.309	0.184	-0.625	0.501
14344	0.050	-1.485	0.185	0.310	0.153	0.478	0.184	-0.625	0.142
14345	0.429	0.942	0.508	0.506	1.415	0.422	0.447	1.060	0.492
14346	0.142	-0.895	0.508	0.421	0.869	0.053	0.326	0.285	0.501
14347	0.508	1.447	0.508	0.494	1.340	0.462	0.447	1.060	0.050
14348	0.508	1.447	0.481	0.444	1.020	0.491	0.503	1.413	0.097
14349	0.300	0.116	0.492	0.272	-0.091	0.422	0.332	0.318	0.481
14350	0.495	1.363	0.185	0.480	1.246	0.371	0.508	1.447	0.467
14351	0.184	-0.625	0.481	0.149	-0.882	0.371	0.266	-0.103	0.450
14352	0.482	1.279	0.431	0.512	1.453	0.398	0.497	1.380	0.450
14353	0.142	-0.895	0.467	0.310	0.153	0.491	0.261	-0.137	0.097
14354	0.432	0.958	0.513	0.488	1.302	0.491	0.447	1.060	0.512
14355	0.513	1.481	0.185	0.488	1.302	0.149	0.511	1.464	0.508
14356	0.226	-0.356	0.467	0.149	-0.882	0.444	0.184	-0.625	0.513
14357	0.513	1.481	0.224	0.374	0.568	0.444	0.495	1.363	0.450
14358	0.361	0.504	0.262	0.234	-0.336	0.478	0.455	1.110	0.431
14359	0.387	0.672	0.296	0.193	-0.600	0.235	0.455	1.110	0.481
14360	0.479	1.262	0.409	0.509	1.434	0.102	0.426	0.925	0.501
14361	0.184	-0.625	0.358	0.310	0.153	0.501	0.224	-0.373	0.501
14362	0.489	1.329	0.050	0.482	1.265	0.149	0.384	0.655	0.501
14363	0.450	1.076	0.481	0.421	0.869	0.193	0.505	1.430	0.481
14364	0.097	-1.181	0.328	0.053	-1.504	0.491	0.384	0.655	0.481
14365	0.508	1.447	0.512	0.462	1.133	0.462	0.513	1.481	0.481
14366	0.489	1.329	0.501	0.488	1.302	0.512	0.476	1.245	0.409
14367	0.142	-0.895	0.501	0.424	0.888	0.462	0.050	-1.485	0.409
14368	0.497	1.380	0.512	0.371	0.549	0.273	0.511	1.464	0.409
14369	0.508	1.447	0.508	0.371	0.549	0.273	0.511	1.464	0.409
14370	0.097	-1.181	0.492	0.401	0.737	0.273	0.050	-1.485	0.409
14371	0.505	1.430	0.481	0.485	1.283	0.398	0.516	1.498	0.409
14372	0.505	1.430	0.185	0.485	1.283	0.501	0.516	1.498	0.224
14373	0.263	-0.120	0.508	0.149	-0.882	0.398	0.224	-0.373	0.185
14374	0.511	1.464	0.508	0.442	1.001	0.273	0.450	1.076	0.385
14375	0.466	1.178	0.508	0.442	1.001	0.371	0.479	1.262	0.512
14376	0.489	1.329	0.481	0.480	1.246	0.273	0.511	1.464	0.224
14377	0.263	-0.120	0.262	0.234	-0.336	0.273	0.184	-0.625	0.501
14378	0.263	-0.120	0.185	0.102	-1.184	0.508	0.184	-0.625	0.409
14379	0.476	1.245	0.512	0.497	1.359	0.462	0.479	1.262	0.492
14380	0.355	0.470	0.508	0.149	-0.882	0.444	0.050	-1.485	0.492
14381	0.358	0.487	0.508	0.193	-0.600	0.512	0.000	?	0.097
14382	0.447	1.060	0.224	0.494	1.340	0.514	0.511	1.464	0.501
14383	0.505	1.430	0.224	0.462	1.133	0.478	0.505	1.430	0.501
14384	0.489	1.329	0.481	0.515	1.472	0.501	0.513	1.481	0.481
14385	0.500	1.397	0.262	0.459	1.114	0.514	0.489	1.329	0.097
14386	0.050	-1.485	0.501	0.275	-0.073	0.514	0.226	-0.356	0.467
14387	0.050	-1.485	0.481	0.275	-0.073	0.491	0.226	-0.356	0.262
14388	0.050	-1.485	0.185	0.275	-0.073	0.309	0.226	-0.356	0.450
14389	0.050	-1.485	0.097	0.401	0.737	0.309	0.050	-1.485	0.450
14390	0.495	1.363	0.262	0.500	1.378	0.491	0.505	1.430	0.450
14391	0.050	-1.485	0.224	0.398	0.718	0.512	0.142	-0.895	0.513
14392	0.142	-0.895	0.328	0.272	-0.091	0.512	0.142	-0.895	0.481

14393	0.050	-1.485	0.262	0.374	0.568	0.444	0.097	-1.181	0.296
14394	0.145	-0.878	0.142	0.275	-0.073	0.398	0.142	-0.895	0.512
14395	0.000	?	0.185	0.278	-0.054	0.371	0.261	-0.137	0.508
14396	0.503	1.413	0.450	0.512	1.453	0.501	0.482	1.279	0.512
14397	0.495	1.363	0.185	0.462	1.133	0.309	0.500	1.397	0.467
14398	0.097	-1.181	0.185	0.447	1.039	0.341	0.142	-0.895	0.050
14399	0.476	1.245	0.467	0.512	1.453	0.309	0.447	1.060	0.409
14400	0.500	1.397	0.296	0.515	1.472	0.341	0.455	1.110	0.142
14401	0.495	1.363	0.050	0.480	1.246	0.193	0.466	1.178	0.050
14402	0.463	1.161	0.450	0.503	1.396	0.193	0.358	0.487	0.050
14403	0.503	1.413	0.431	0.512	1.453	0.193	0.492	1.346	0.097
14404	0.503	1.413	0.409	0.512	1.453	0.501	0.511	1.464	0.097
14405	0.503	1.413	0.450	0.485	1.283	0.398	0.516	1.498	0.262
14406	0.097	-1.181	0.097	0.307	0.135	0.341	0.145	-0.878	0.508
14407	0.050	-1.485	0.224	0.310	0.153	0.478	0.226	-0.356	0.097
14408	0.497	1.380	0.328	0.497	1.359	0.501	0.516	1.498	0.431
14409	0.497	1.380	0.224	0.518	1.491	0.193	0.471	1.211	0.513
14410	0.497	1.380	0.224	0.518	1.491	0.235	0.471	1.211	0.513
14411	0.326	0.285	0.185	0.442	1.001	0.235	0.463	1.161	0.492
14412	0.495	1.363	0.385	0.398	0.718	0.508	0.429	0.942	0.481
14413	0.505	1.430	0.358	0.368	0.530	0.512	0.408	0.807	0.512
14414	0.432	0.958	0.431	0.506	1.415	0.478	0.461	1.144	0.501
14415	0.187	-0.608	0.431	0.310	0.153	0.491	0.184	-0.625	0.224
14416	0.329	0.301	0.224	0.339	0.342	0.508	0.097	-1.181	0.467
14417	0.050	-1.485	0.385	0.313	0.172	0.309	0.224	-0.373	0.467
14418	0.447	1.060	0.409	0.339	0.342	0.491	0.513	1.481	0.481
14419	0.050	-1.485	0.508	0.193	-0.600	0.422	0.297	0.099	0.508
14420	0.097	-1.181	0.185	0.196	-0.581	0.273	0.329	0.301	0.142
14421	0.187	-0.608	0.450	0.193	-0.600	0.273	0.184	-0.625	0.262
14422	0.500	1.397	0.262	0.497	1.359	0.508	0.518	1.514	0.501
14423	0.500	1.397	0.431	0.404	0.756	0.508	0.500	1.397	0.508
14424	0.000	?	0.431	0.345	0.379	0.462	0.224	-0.373	0.512
14425	0.500	1.397	0.296	0.482	1.265	0.491	0.508	1.447	0.224
14426	0.497	1.380	0.262	0.509	1.434	0.491	0.484	1.295	0.142
14427	0.050	-1.485	0.467	0.193	-0.600	0.491	0.295	0.082	0.492
14428	0.184	-0.625	0.296	0.237	-0.317	0.462	0.384	0.655	0.224
14429	0.184	-0.625	0.385	0.237	-0.317	0.149	0.384	0.655	0.508
14430	0.484	1.295	0.501	0.512	1.453	0.193	0.466	1.178	0.467
14431	0.474	1.228	0.262	0.520	1.509	0.514	0.508	1.447	0.224
14432	0.000	?	0.224	0.477	1.227	0.422	0.326	0.285	0.262
14433	0.503	1.413	0.492	0.494	1.340	0.514	0.468	1.194	0.142
14434	0.503	1.413	0.185	0.512	1.453	0.478	0.450	1.076	0.097
14435	0.050	-1.485	0.501	0.313	0.172	0.193	0.184	-0.625	0.050
14436	0.489	1.329	0.142	0.497	1.359	0.273	0.511	1.464	0.050
14437	0.050	-1.485	0.508	0.424	0.888	0.444	0.050	-1.485	0.296
14438	0.000	?	0.142	0.272	-0.091	0.501	0.387	0.672	0.142
14439	0.000	?	0.467	0.272	-0.091	0.478	0.387	0.672	0.501
14440	0.500	1.397	0.224	0.509	1.434	0.478	0.505	1.430	0.050
14441	0.500	1.397	0.142	0.509	1.434	0.309	0.505	1.430	0.185
14442	0.497	1.380	0.450	0.468	1.170	0.309	0.508	1.447	0.508
14443	0.497	1.380	0.431	0.488	1.302	0.309	0.382	0.638	0.262
14444	0.505	1.430	0.296	0.488	1.302	0.491	0.382	0.638	0.328
14445	0.505	1.430	0.481	0.488	1.302	0.501	0.382	0.638	0.512
14446	0.503	1.413	0.185	0.465	1.152	0.501	0.463	1.161	0.512
14447	0.361	0.504	0.142	0.149	-0.882	0.491	0.050	-1.485	0.512
14448	0.000	?	0.385	0.196	-0.581	0.491	0.329	0.301	0.513
14449	0.474	1.228	0.467	0.512	1.453	0.491	0.492	1.346	0.328
14450	0.097	-1.181	0.185	0.424	0.888	0.514	0.142	-0.895	0.142
14451	0.511	1.464	0.508	0.515	1.472	0.371	0.466	1.178	0.185
14452	0.511	1.464	0.050	0.480	1.246	0.422	0.408	0.807	0.142
14453	0.297	0.099	0.431	0.193	-0.600	0.462	0.097	-1.181	0.142

14454	0.224	-0.373	0.467	0.275	-0.073	0.309	0.050	-1.485	0.050
14455	0.497	1.380	0.450	0.450	1.057	0.508	0.487	1.312	0.358
14456	0.479	1.262	0.296	0.497	1.359	0.491	0.516	1.498	0.328
14457	0.500	1.397	0.492	0.477	1.227	0.398	0.495	1.363	0.513
14458	0.500	1.397	0.481	0.480	1.246	0.341	0.505	1.430	0.513
14459	0.097	-1.181	0.467	0.313	0.172	0.508	0.329	0.301	0.142
14460	0.097	-1.181	0.501	0.313	0.172	0.512	0.329	0.301	0.512
14461	0.097	-1.181	0.142	0.313	0.172	0.512	0.329	0.301	0.501
14462	0.503	1.413	0.492	0.488	1.302	0.508	0.495	1.363	0.501
14463	0.508	1.447	0.501	0.494	1.340	0.193	0.489	1.329	0.185
14464	0.511	1.464	0.467	0.494	1.340	0.501	0.500	1.397	0.508
14465	0.505	1.430	0.450	0.491	1.321	0.512	0.508	1.447	0.508
14466	0.505	1.430	0.501	0.491	1.321	0.512	0.505	1.430	0.508
14467	0.505	1.430	0.501	0.491	1.321	0.512	0.508	1.447	0.262
14468	0.495	1.363	0.142	0.512	1.453	0.508	0.500	1.397	0.097
14469	0.484	1.295	0.450	0.374	0.568	0.422	0.495	1.363	0.513
14470	0.326	0.285	0.185	0.421	0.869	0.444	0.261	-0.137	0.224
14471	0.513	1.481	0.467	0.462	1.133	0.422	0.495	1.363	0.492
14472	0.297	0.099	0.492	0.310	0.153	0.422	0.142	-0.895	0.409
14473	0.487	1.312	0.431	0.503	1.396	0.512	0.492	1.346	0.262
14474	0.482	1.279	0.328	0.485	1.283	0.512	0.497	1.380	0.296
14475	0.050	-1.485	0.508	0.401	0.737	0.501	0.050	-1.485	0.512
14476	0.187	-0.608	0.409	0.339	0.342	0.444	0.097	-1.181	0.142
14477	0.503	1.413	0.262	0.509	1.434	0.444	0.479	1.262	0.512
14478	0.503	1.413	0.262	0.512	1.453	0.478	0.466	1.178	0.185
14479	0.500	1.397	0.328	0.512	1.453	0.491	0.453	1.093	0.513
14480	0.500	1.397	0.328	0.506	1.415	0.444	0.453	1.093	0.224
14481	0.261	-0.137	0.328	0.196	-0.581	0.462	0.097	-1.181	0.431
14482	0.476	1.245	0.501	0.497	1.359	0.193	0.513	1.481	0.296
14483	0.487	1.312	0.185	0.509	1.434	0.235	0.508	1.447	0.296
14484	0.487	1.312	0.262	0.509	1.434	0.462	0.516	1.498	0.481
14485	0.482	1.279	0.450	0.506	1.415	0.462	0.450	1.076	0.492
14486	0.482	1.279	0.262	0.503	1.396	0.309	0.429	0.942	0.142
14487	0.508	1.447	0.328	0.418	0.850	0.508	0.476	1.245	0.501
14488	0.429	0.942	0.481	0.439	0.982	0.512	0.500	1.397	0.296
14489	0.466	1.178	0.142	0.418	0.850	0.501	0.500	1.397	0.185
14490	0.466	1.178	0.050	0.418	0.850	0.478	0.500	1.397	0.097
14491	0.505	1.430	0.224	0.506	1.415	0.309	0.479	1.262	0.508
14492	0.505	1.430	0.481	0.506	1.415	0.235	0.479	1.262	0.185
14493	0.495	1.363	0.492	0.500	1.378	0.508	0.484	1.295	0.481
14494	0.516	1.498	0.409	0.442	1.001	0.508	0.408	0.807	0.224
14495	0.513	1.481	0.142	0.442	1.001	0.501	0.408	0.807	0.492
14496	0.513	1.481	0.481	0.477	1.227	0.193	0.408	0.807	0.508
14497	0.516	1.498	0.450	0.488	1.302	0.444	0.408	0.807	0.508
14498	0.516	1.498	0.385	0.442	1.001	0.149	0.408	0.807	0.328
14499	0.516	1.498	0.513	0.459	1.114	0.508	0.408	0.807	0.467
14500	0.358	0.487	0.512	0.193	-0.600	0.508	0.224	-0.373	0.467
14501	0.329	0.301	0.296	0.237	-0.317	0.508	0.187	-0.608	0.492
14502	0.500	1.397	0.467	0.462	1.133	0.398	0.384	0.655	0.467
14503	0.489	1.329	0.296	0.465	1.152	0.444	0.516	1.498	0.224
14504	0.226	-0.356	0.296	0.310	0.153	0.053	0.226	-0.356	0.512
14505	0.426	0.925	0.262	0.503	1.396	0.512	0.500	1.397	0.501
14506	0.511	1.464	0.467	0.506	1.415	0.462	0.408	0.807	0.481
14507	0.482	1.279	0.501	0.491	1.321	0.514	0.492	1.346	0.501
14508	0.468	1.194	0.262	0.480	1.246	0.478	0.492	1.346	0.501
14509	0.329	0.301	0.224	0.307	0.135	0.341	0.000	?	0.501
14510	0.226	-0.356	0.142	0.237	-0.317	0.508	0.097	-1.181	0.224
14511	0.482	1.279	0.513	0.503	1.396	0.309	0.500	1.397	0.431
14512	0.482	1.279	0.185	0.503	1.396	0.341	0.500	1.397	0.185
14513	0.513	1.481	0.185	0.497	1.359	0.341	0.482	1.279	0.512
14514	0.300	0.116	0.508	0.193	-0.600	0.193	0.097	-1.181	0.492

14515	0.505	1.430	0.492	0.444	1.020	0.193	0.474	1.228	0.492
14516	0.184	-0.625	0.296	0.149	-0.882	0.309	0.266	-0.103	0.142
14517	0.508	1.447	0.097	0.509	1.434	0.478	0.450	1.076	0.467
14518	0.508	1.447	0.296	0.509	1.434	0.235	0.450	1.076	0.508
14519	0.508	1.447	0.224	0.509	1.434	0.491	0.450	1.076	0.185
14520	0.479	1.262	0.262	0.401	0.737	0.508	0.511	1.464	0.097
14521	0.492	1.346	0.409	0.442	1.001	0.508	0.474	1.228	0.097
14522	0.184	-0.625	0.501	0.053	-1.504	0.462	0.300	0.116	0.142
14523	0.479	1.262	0.328	0.512	1.453	0.514	0.513	1.481	0.142
14524	0.429	0.942	0.296	0.465	1.152	0.422	0.508	1.447	0.431
14525	0.468	1.194	0.262	0.515	1.472	0.491	0.511	1.464	0.142
14526	0.500	1.397	0.262	0.480	1.246	0.193	0.468	1.194	0.224
14527	0.187	-0.608	0.467	0.339	0.342	0.501	0.050	-1.485	0.512
14528	0.466	1.178	0.409	0.503	1.396	0.501	0.408	0.807	0.050
14529	0.226	-0.356	0.296	0.307	0.135	0.512	0.145	-0.878	0.508
14530	0.263	-0.120	0.296	0.339	0.342	0.491	0.050	-1.485	0.224
14531	0.297	0.099	0.296	0.339	0.342	0.371	0.050	-1.485	0.224
14532	0.405	0.790	0.097	0.193	-0.600	0.193	0.100	-1.164	0.142
14533	0.355	0.470	0.224	0.193	-0.600	0.491	0.100	-1.164	0.508
14534	0.050	-1.485	0.409	0.307	0.135	0.444	0.263	-0.120	0.508
14535	0.476	1.245	0.358	0.474	1.208	0.444	0.505	1.430	0.142
14536	0.329	0.301	0.385	0.237	-0.317	0.273	0.097	-1.181	0.358
14537	0.500	1.397	0.385	0.494	1.340	0.341	0.432	0.958	0.501
14538	0.497	1.380	0.328	0.503	1.396	0.422	0.513	1.481	0.501
14539	0.497	1.380	0.512	0.503	1.396	0.235	0.513	1.481	0.185
14540	0.500	1.397	0.262	0.462	1.133	0.444	0.495	1.363	0.512
14541	0.508	1.447	0.467	0.515	1.472	0.501	0.474	1.228	0.492
14542	0.489	1.329	0.262	0.424	0.888	0.235	0.505	1.430	0.185
14543	0.476	1.245	0.508	0.485	1.283	0.193	0.503	1.413	0.481
14544	0.187	-0.608	0.431	0.193	-0.600	0.149	0.224	-0.373	0.050
14545	0.513	1.481	0.501	0.500	1.378	0.398	0.463	1.161	0.050
14546	0.513	1.481	0.224	0.500	1.378	0.193	0.463	1.161	0.185
14547	0.513	1.481	0.262	0.512	1.453	0.273	0.476	1.245	0.492
14548	0.476	1.245	0.450	0.491	1.321	0.193	0.505	1.430	0.508
14549	0.261	-0.137	0.185	0.371	0.549	0.235	0.142	-0.895	0.097
14550	0.187	-0.608	0.224	0.193	-0.600	0.478	0.261	-0.137	0.097
14551	0.511	1.464	0.492	0.488	1.302	0.341	0.500	1.397	0.224
14552	0.503	1.413	0.492	0.444	1.020	0.193	0.508	1.447	0.481
14553	0.503	1.413	0.481	0.442	1.001	0.508	0.513	1.481	0.185
14554	0.224	-0.373	0.262	0.275	-0.073	0.102	0.224	-0.373	0.185
14555	0.226	-0.356	0.262	0.342	0.361	0.149	0.145	-0.878	0.450
14556	0.484	1.295	0.512	0.424	0.888	0.508	0.487	1.312	0.501
14557	0.263	-0.120	0.467	0.234	-0.336	0.508	0.224	-0.373	0.142
14558	0.503	1.413	0.296	0.442	1.001	0.512	0.503	1.413	0.142
14559	0.479	1.262	0.296	0.500	1.378	0.462	0.461	1.144	0.050
14560	0.184	-0.625	0.097	0.237	-0.317	0.149	0.224	-0.373	0.097
14561	0.097	-1.181	0.097	0.193	-0.600	0.193	0.263	-0.120	0.097
14562	0.261	-0.137	0.142	0.149	-0.882	0.149	0.142	-0.895	0.513
14563	0.226	-0.356	0.262	0.398	0.718	0.193	0.097	-1.181	0.492
14564	0.326	0.285	0.224	0.193	-0.600	0.193	0.050	-1.485	0.142
14565	0.266	-0.103	0.262	0.272	-0.091	0.309	0.050	-1.485	0.142
14566	0.142	-0.895	0.431	0.193	-0.600	0.478	0.297	0.099	0.142
14567	0.184	-0.625	0.431	0.234	-0.336	0.508	0.142	-0.895	0.481
14568	0.450	1.076	0.501	0.480	1.246	0.444	0.495	1.363	0.508
14569	0.184	-0.625	0.512	0.345	0.379	0.462	0.050	-1.485	0.142
14570	0.187	-0.608	0.467	0.196	-0.581	0.309	0.184	-0.625	0.097
14571	0.468	1.194	0.467	0.509	1.434	0.514	0.503	1.413	0.097
14572	0.297	0.099	0.467	0.102	-1.184	0.514	0.263	-0.120	0.185
14573	0.050	-1.485	0.224	0.152	-0.863	0.491	0.329	0.301	0.142
14574	0.450	1.076	0.328	0.503	1.396	0.193	0.508	1.447	0.481
14575	0.429	0.942	0.296	0.503	1.396	0.491	0.508	1.447	0.501

14576	0.408	0.807	0.262	0.509	1.434	0.273	0.508	1.447	0.050
14577	0.450	1.076	0.481	0.459	1.114	0.462	0.505	1.430	0.050
14578	0.097	-1.181	0.185	0.149	-0.882	0.462	0.326	0.285	0.262
14579	0.224	-0.373	0.431	0.193	-0.600	0.273	0.142	-0.895	0.481
14580	0.329	0.301	0.450	0.149	-0.882	0.193	0.184	-0.625	0.050
14581	0.224	-0.373	0.328	0.193	-0.600	0.422	0.142	-0.895	0.508
14582	0.224	-0.373	0.431	0.193	-0.600	0.193	0.142	-0.895	0.142
14583	0.187	-0.608	0.328	0.310	0.153	0.462	0.050	-1.485	0.512
14584	0.384	0.655	0.262	0.474	1.208	0.444	0.358	0.487	0.501
14585	0.358	0.487	0.508	0.506	1.415	0.102	0.329	0.301	0.467
14586	0.432	0.958	0.508	0.442	1.001	0.235	0.511	1.464	0.097
14587	0.432	0.958	0.492	0.459	1.114	0.462	0.511	1.464	0.097
14588	0.224	-0.373	0.492	0.310	0.153	0.273	0.145	-0.878	0.481
14589	0.384	0.655	0.450	0.512	1.453	0.501	0.508	1.447	0.224
14590	0.405	0.790	0.450	0.515	1.472	0.371	0.503	1.413	0.097
14591	0.511	1.464	0.467	0.491	1.321	0.371	0.492	1.346	0.481
14592	0.187	-0.608	0.481	0.193	-0.600	0.193	0.184	-0.625	0.481
14593	0.450	1.076	0.481	0.491	1.321	0.508	0.503	1.413	0.481
14594	0.261	-0.137	0.481	0.272	-0.091	0.193	0.000	?	0.224
14595	0.434	0.975	0.296	0.462	1.133	0.193	0.500	1.397	0.450
14596	0.434	0.975	0.450	0.462	1.133	0.478	0.500	1.397	0.512
14597	0.297	0.099	0.097	0.275	-0.073	0.462	0.263	-0.120	0.050
14598	0.261	-0.137	0.097	0.193	-0.600	0.235	0.100	-1.164	0.097
14599	0.468	1.194	0.296	0.418	0.850	0.508	0.508	1.447	0.185
14600	0.297	0.099	0.358	0.196	-0.581	0.102	0.226	-0.356	0.224
14601	0.387	0.672	0.501	0.459	1.114	0.235	0.492	1.346	0.050
14602	0.497	1.380	0.328	0.444	1.020	0.053	0.408	0.807	0.142
14603	0.261	-0.137	0.450	0.102	-1.184	0.491	0.261	-0.137	0.224
14604	0.224	-0.373	0.185	0.234	-0.336	0.193	0.295	0.082	0.296
14605	0.489	1.329	0.481	0.465	1.152	0.478	0.511	1.464	0.467
14606	0.184	-0.625	0.185	0.275	-0.073	0.309	0.142	-0.895	0.467
14607	0.505	1.430	0.185	0.500	1.378	0.512	0.505	1.430	0.431
14608	0.145	-0.878	0.224	0.374	0.568	0.491	0.184	-0.625	0.492
14609	0.508	1.447	0.142	0.377	0.587	0.444	0.508	1.447	0.508
14610	0.145	-0.878	0.185	0.196	-0.581	0.149	0.224	-0.373	0.508
14611	0.468	1.194	0.262	0.500	1.378	0.514	0.432	0.958	0.492
14612	0.226	-0.356	0.296	0.193	-0.600	0.508	0.297	0.099	0.142
14613	0.142	-0.895	0.296	0.193	-0.600	0.422	0.297	0.099	0.097
14614	0.453	1.093	0.296	0.477	1.227	0.462	0.476	1.245	0.097
14615	0.429	0.942	0.450	0.465	1.152	0.193	0.489	1.329	0.097
14616	0.297	0.099	0.450	0.237	-0.317	0.501	0.142	-0.895	0.513
14617	0.482	1.279	0.296	0.509	1.434	0.501	0.500	1.397	0.328
14618	0.184	-0.625	0.296	0.102	-1.184	0.478	0.297	0.099	0.481
14619	0.142	-0.895	0.224	0.234	-0.336	0.501	0.184	-0.625	0.501
14620	0.384	0.655	0.224	0.053	-1.504	0.462	0.097	-1.181	0.296
14621	0.468	1.194	0.296	0.485	1.283	0.462	0.503	1.413	0.481
14622	0.187	-0.608	0.328	0.193	-0.600	0.273	0.187	-0.608	0.224
14623	0.511	1.464	0.224	0.474	1.208	0.508	0.476	1.245	0.097
14624	0.050	-1.485	0.262	0.310	0.153	0.273	0.224	-0.373	0.467
14625	0.434	0.975	0.262	0.512	1.453	0.422	0.484	1.295	0.467
14626	0.468	1.194	0.224	0.485	1.283	0.478	0.503	1.413	0.467
14627	0.447	1.060	0.512	0.444	1.020	0.478	0.505	1.430	0.467
14628	0.297	0.099	0.467	0.149	-0.882	0.102	0.326	0.285	0.262
14629	0.495	1.363	0.450	0.509	1.434	0.422	0.463	1.161	0.508
14630	0.482	1.279	0.262	0.506	1.415	0.508	0.463	1.161	0.508
14631	0.466	1.178	0.328	0.424	0.888	0.309	0.484	1.295	0.508
14632	0.500	1.397	0.262	0.465	1.152	0.235	0.461	1.144	0.492
14633	0.142	-0.895	0.262	0.193	-0.600	0.309	0.226	-0.356	0.492
14634	0.492	1.346	0.097	0.509	1.434	0.235	0.508	1.447	0.296
14635	0.500	1.397	0.481	0.491	1.321	0.102	0.503	1.413	0.501
14636	0.463	1.161	0.450	0.480	1.246	0.508	0.476	1.245	0.185

14637	0.447	1.060	0.501	0.503	1.396	0.235	0.492	1.346	0.185
14638	0.505	1.430	0.467	0.459	1.114	0.102	0.492	1.346	0.262
14639	0.505	1.430	0.224	0.459	1.114	0.444	0.492	1.346	0.262
14640	0.142	-0.895	0.224	0.275	-0.073	0.273	0.224	-0.373	0.467
14641	0.447	1.060	0.224	0.503	1.396	0.193	0.429	0.942	0.142
14642	0.187	-0.608	0.467	0.272	-0.091	0.491	0.184	-0.625	0.512
14643	0.463	1.161	0.508	0.421	0.869	0.273	0.508	1.447	0.224
14644	0.492	1.346	0.328	0.477	1.227	0.273	0.487	1.312	0.431
14645	0.432	0.958	0.097	0.485	1.283	0.273	0.489	1.329	0.224
14646	0.329	0.301	0.296	0.102	-1.184	0.512	0.142	-0.895	0.224
14647	0.508	1.447	0.262	0.424	0.888	0.512	0.466	1.178	0.358
14648	0.405	0.790	0.467	0.509	1.434	0.053	0.500	1.397	0.262
14649	0.263	-0.120	0.185	0.313	0.172	0.235	0.184	-0.625	0.262
14650	0.263	-0.120	0.224	0.237	-0.317	0.512	0.097	-1.181	0.050
14651	0.329	0.301	0.224	0.310	0.153	0.501	0.097	-1.181	0.142
14652	0.329	0.301	0.262	0.237	-0.317	0.193	0.142	-0.895	0.142
14653	0.329	0.301	0.224	0.102	-1.184	0.462	0.142	-0.895	0.185
14654	0.497	1.380	0.467	0.506	1.415	0.422	0.426	0.925	0.492
14655	0.184	-0.625	0.185	0.234	-0.336	0.193	0.145	-0.878	0.492
14656	0.263	-0.120	0.513	0.102	-1.184	0.512	0.224	-0.373	0.050
14657	0.447	1.060	0.481	0.453	1.076	0.235	0.503	1.413	0.097
14658	0.261	-0.137	0.508	0.275	-0.073	0.235	0.000	?	0.262
14659	0.329	0.301	0.481	0.196	-0.581	0.102	0.050	-1.485	0.050
14660	0.479	1.262	0.467	0.491	1.321	0.491	0.505	1.430	0.296
14661	0.142	-0.895	0.142	0.272	-0.091	0.508	0.224	-0.373	0.262
14662	0.050	-1.485	0.508	0.275	-0.073	0.193	0.224	-0.373	0.296
14663	0.224	-0.373	0.508	0.278	-0.054	0.273	0.142	-0.895	0.142
14664	0.479	1.262	0.142	0.509	1.434	0.193	0.503	1.413	0.262
14665	0.492	1.346	0.185	0.509	1.434	0.462	0.503	1.413	0.508
14666	0.411	0.824	0.185	0.053	-1.504	0.309	0.142	-0.895	0.508
14667	0.145	-0.878	0.385	0.234	-0.336	0.309	0.358	0.487	0.409
14668	0.482	1.279	0.142	0.512	1.453	0.508	0.492	1.346	0.262
14669	0.445	1.043	0.185	0.503	1.396	0.501	0.497	1.380	0.142
14670	0.389	0.689	0.262	0.193	-0.600	0.273	0.184	-0.625	0.050
14671	0.516	1.498	0.409	0.465	1.152	0.309	0.511	1.464	0.142
14672	0.513	1.481	0.501	0.424	0.888	0.193	0.492	1.346	0.262
14673	0.295	0.082	0.409	0.193	-0.600	0.309	0.187	-0.608	0.492
14674	0.466	1.178	0.262	0.509	1.434	0.309	0.476	1.245	0.467
14675	0.295	0.082	0.467	0.234	-0.336	0.512	0.050	-1.485	0.481
14676	0.295	0.082	0.508	0.234	-0.336	0.491	0.050	-1.485	0.481
14677	0.261	-0.137	0.481	0.102	-1.184	0.193	0.184	-0.625	0.097
14678	0.468	1.194	0.224	0.494	1.340	0.193	0.489	1.329	0.097
14679	0.500	1.397	0.409	0.503	1.396	0.193	0.505	1.430	0.097
14680	0.263	-0.120	0.481	0.193	-0.600	0.149	0.097	-1.181	0.467
14681	0.224	-0.373	0.262	0.272	-0.091	0.478	0.097	-1.181	0.431
14682	0.142	-0.895	0.185	0.193	-0.600	0.462	0.226	-0.356	0.224
14683	0.508	1.447	0.262	0.462	1.133	0.235	0.476	1.245	0.262
14684	0.184	-0.625	0.262	0.310	0.153	0.309	0.184	-0.625	0.224
14685	0.184	-0.625	0.450	0.310	0.153	0.309	0.184	-0.625	0.185
14686	0.508	1.447	0.501	0.506	1.415	0.273	0.445	1.043	0.512
14687	0.492	1.346	0.501	0.497	1.359	0.273	0.500	1.397	0.142
14688	0.297	0.099	0.501	0.272	-0.091	0.514	0.142	-0.895	0.328
14689	0.097	-1.181	0.224	0.310	0.153	0.512	0.142	-0.895	0.296
14690	0.300	0.116	0.512	0.193	-0.600	0.235	0.050	-1.485	0.097
14691	0.224	-0.373	0.431	0.310	0.153	0.235	0.097	-1.181	0.224
14692	0.263	-0.120	0.224	0.310	0.153	0.235	0.097	-1.181	0.481
14693	0.413	0.841	0.185	0.506	1.415	0.512	0.508	1.447	0.224
14694	0.503	1.413	0.492	0.485	1.283	0.235	0.487	1.312	0.450
14695	0.329	0.301	0.097	0.196	-0.581	0.478	0.000	?	0.431
14696	0.297	0.099	0.097	0.193	-0.600	0.273	0.142	-0.895	0.481
14697	0.263	-0.120	0.409	0.193	-0.600	0.398	0.142	-0.895	0.492

14698	0.263	-0.120	0.385	0.149	-0.882	0.501	0.142	-0.895	0.431
14699	0.468	1.194	0.501	0.480	1.246	0.491	0.474	1.228	0.185
14700	0.411	0.824	0.358	0.459	1.114	0.235	0.500	1.397	0.385
14701	0.295	0.082	0.492	0.237	-0.317	0.273	0.145	-0.878	0.385
14702	0.297	0.099	0.481	0.310	0.153	0.501	0.097	-1.181	0.224
14703	0.297	0.099	0.481	0.310	0.153	0.149	0.097	-1.181	0.358
14704	0.097	-1.181	0.224	0.275	-0.073	0.309	0.184	-0.625	0.358
14705	0.226	-0.356	0.492	0.272	-0.091	0.512	0.145	-0.878	0.492
14706	0.408	0.807	0.501	0.512	1.453	0.512	0.474	1.228	0.224
14707	0.358	0.487	0.262	0.515	1.472	0.512	0.492	1.346	0.142
14708	0.384	0.655	0.481	0.237	-0.317	0.422	0.050	-1.485	0.262
14709	0.384	0.655	0.492	0.237	-0.317	0.235	0.050	-1.485	0.508
14710	0.332	0.318	0.501	0.234	-0.336	0.512	0.261	-0.137	0.385
14711	0.511	1.464	0.501	0.512	1.453	0.491	0.476	1.245	0.508
14712	0.261	-0.137	0.328	0.234	-0.336	0.193	0.050	-1.485	0.185
14713	0.463	1.161	0.431	0.480	1.246	0.235	0.503	1.413	0.508
14714	0.261	-0.137	0.513	0.272	-0.091	0.273	0.142	-0.895	0.492
14715	0.505	1.430	0.224	0.398	0.718	0.273	0.511	1.464	0.501
14716	0.429	0.942	0.450	0.500	1.378	0.398	0.497	1.380	0.185
14717	0.500	1.397	0.097	0.488	1.302	0.149	0.461	1.144	0.492
14718	0.229	-0.339	0.262	0.234	-0.336	0.193	0.097	-1.181	0.492
14719	0.266	-0.103	0.481	0.272	-0.091	0.193	0.097	-1.181	0.050
14720	0.450	1.076	0.508	0.503	1.396	0.491	0.474	1.228	0.185
14721	0.184	-0.625	0.508	0.149	-0.882	0.512	0.224	-0.373	0.328
14722	0.224	-0.373	0.513	0.310	0.153	0.478	0.097	-1.181	0.097
14723	0.495	1.363	0.508	0.515	1.472	0.508	0.474	1.228	0.501
14724	0.495	1.363	0.467	0.515	1.472	0.491	0.474	1.228	0.501
14725	0.484	1.295	0.450	0.515	1.472	0.501	0.474	1.228	0.481
14726	0.261	-0.137	0.450	0.427	0.907	0.501	0.000	?	0.481
14727	0.263	-0.120	0.467	0.237	-0.317	0.193	0.224	-0.373	0.142
14728	0.511	1.464	0.142	0.509	1.434	0.193	0.445	1.043	0.501
14729	0.468	1.194	0.481	0.494	1.340	0.309	0.505	1.430	0.512
14730	0.297	0.099	0.450	0.193	-0.600	0.341	0.050	-1.485	0.185
14731	0.297	0.099	0.142	0.234	-0.336	0.491	0.097	-1.181	0.050
14732	0.097	-1.181	0.224	0.275	-0.073	0.102	0.184	-0.625	0.512
14733	0.097	-1.181	0.450	0.275	-0.073	0.501	0.224	-0.373	0.358
14734	0.142	-0.895	0.262	0.398	0.718	0.501	0.050	-1.485	0.358
14735	0.261	-0.137	0.492	0.149	-0.882	0.235	0.145	-0.878	0.431
14736	0.224	-0.373	0.481	0.196	-0.581	0.501	0.226	-0.356	0.097
14737	0.261	-0.137	0.467	0.196	-0.581	0.193	0.297	0.099	0.481
14738	0.429	0.942	0.501	0.488	1.302	0.309	0.463	1.161	0.142
14739	0.434	0.975	0.224	0.512	1.453	0.462	0.461	1.144	0.508
14740	0.497	1.380	0.467	0.482	1.265	0.462	0.437	0.992	0.512
14741	0.516	1.498	0.262	0.509	1.434	0.444	0.484	1.295	0.508
14742	0.468	1.194	0.328	0.491	1.321	0.444	0.497	1.380	0.097
14743	0.468	1.194	0.328	0.500	1.378	0.235	0.497	1.380	0.492
14744	0.466	1.178	0.492	0.500	1.378	0.512	0.484	1.295	0.385
14745	0.224	-0.373	0.501	0.196	-0.581	0.512	0.142	-0.895	0.409
14746	0.332	0.318	0.492	0.193	-0.600	0.501	0.097	-1.181	0.513
14747	0.300	0.116	0.501	0.310	0.153	0.491	0.097	-1.181	0.508
14748	0.263	-0.120	0.512	0.345	0.379	0.478	0.097	-1.181	0.467
14749	0.482	1.279	0.358	0.485	1.283	0.309	0.508	1.447	0.512
14750	0.184	-0.625	0.296	0.102	-1.184	0.508	0.326	0.285	0.450
14751	0.429	0.942	0.431	0.500	1.378	0.309	0.476	1.245	0.409
14752	0.450	1.076	0.492	0.500	1.378	0.273	0.497	1.380	0.512
14753	0.329	0.301	0.512	0.237	-0.317	0.444	0.295	0.082	0.328
14754	0.429	0.942	0.501	0.503	1.396	0.444	0.484	1.295	0.508
14755	0.326	0.285	0.501	0.193	-0.600	0.491	0.224	-0.373	0.185
14756	0.261	-0.137	0.501	0.313	0.172	0.235	0.097	-1.181	0.185
14757	0.503	1.413	0.508	0.462	1.133	0.501	0.463	1.161	0.450
14758	0.503	1.413	0.512	0.462	1.133	0.273	0.463	1.161	0.501

14759	0.489	1.329	0.501	0.442	1.001	0.512	0.463	1.161	0.501
14760	0.489	1.329	0.467	0.442	1.001	0.193	0.463	1.161	0.492
14761	0.447	1.060	0.481	0.234	−0.336	0.193	0.263	−0.120	0.508
14762	0.447	1.060	0.501	0.515	1.472	0.193	0.505	1.430	0.467
14763	0.463	1.161	0.097	0.515	1.472	0.235	0.505	1.430	0.508
14764	0.479	1.262	0.262	0.503	1.396	0.149	0.505	1.430	0.508
14765	0.484	1.295	0.492	0.491	1.321	0.235	0.487	1.312	0.501
14766	0.484	1.295	0.481	0.480	1.246	0.102	0.487	1.312	0.050
14767	0.297	0.099	0.431	0.307	0.135	0.102	0.297	0.099	0.512
14768	0.450	1.076	0.450	0.509	1.434	0.309	0.505	1.430	0.508
14769	0.097	−1.181	0.224	0.313	0.172	0.508	0.184	−0.625	0.262
14770	0.097	−1.181	0.296	0.275	−0.073	0.508	0.184	−0.625	0.262
14771	0.295	0.082	0.501	0.444	1.020	0.309	0.261	−0.137	0.296
14772	0.361	0.504	0.142	0.444	1.020	0.273	0.261	−0.137	0.097
14773	0.505	1.430	0.142	0.488	1.302	0.273	0.461	1.144	0.358
14774	0.329	0.301	0.481	0.234	−0.336	0.193	0.142	−0.895	0.512
14775	0.447	1.060	0.358	0.497	1.359	0.193	0.508	1.447	0.224
14776	0.184	−0.625	0.097	0.275	−0.073	0.273	0.224	−0.373	0.512
14777	0.484	1.295	0.262	0.515	1.472	0.149	0.432	0.958	0.508
14778	0.184	−0.625	0.409	0.193	−0.600	0.149	0.224	−0.373	0.512
14779	0.184	−0.625	0.450	0.193	−0.600	0.149	0.224	−0.373	0.492
14780	0.224	−0.373	0.431	0.196	−0.581	0.235	0.355	0.470	0.097
14781	0.142	−0.895	0.224	0.237	−0.317	0.478	0.261	−0.137	0.508
14782	0.184	−0.625	0.185	0.152	−0.863	0.508	0.261	−0.137	0.262
14783	0.261	−0.137	0.492	0.237	−0.317	0.491	0.050	−1.485	0.142
14784	0.297	0.099	0.481	0.102	−1.184	0.235	0.142	−0.895	0.262
14785	0.297	0.099	0.508	0.102	−1.184	0.235	0.142	−0.895	0.501
14786	0.295	0.082	0.492	0.310	0.153	0.371	0.184	−0.625	0.512
14787	0.447	1.060	0.296	0.506	1.415	0.235	0.487	1.312	0.492
14788	0.447	1.060	0.097	0.506	1.415	0.235	0.487	1.312	0.492
14789	0.297	0.099	0.328	0.307	0.135	0.491	0.050	−1.485	0.508
14790	0.297	0.099	0.328	0.272	−0.091	0.514	0.000	?	0.185
14791	0.226	−0.356	0.501	0.275	−0.073	0.398	0.097	−1.181	0.097
14792	0.224	−0.373	0.296	0.193	−0.600	0.514	0.261	−0.137	0.467
14793	0.297	0.099	0.097	0.193	−0.600	0.235	0.050	−1.485	0.431
14794	0.329	0.301	0.508	0.275	−0.073	0.235	0.295	0.082	0.492
14795	0.224	−0.373	0.481	0.149	−0.882	0.235	0.263	−0.120	0.467
14796	0.263	−0.120	0.501	0.149	−0.882	0.512	0.297	0.099	0.467
14797	0.263	−0.120	0.450	0.149	−0.882	0.478	0.142	−0.895	0.508
14798	0.226	−0.356	0.185	0.237	−0.317	0.053	0.263	−0.120	0.512
14799	0.508	1.447	0.492	0.477	1.227	0.235	0.505	1.430	0.513
14800	0.466	1.178	0.501	0.512	1.453	0.149	0.500	1.397	0.450
14801	0.453	1.093	0.481	0.491	1.321	0.273	0.405	0.790	0.481
14802	0.261	−0.137	0.262	0.234	−0.336	0.462	0.261	−0.137	0.512
14803	0.329	0.301	0.185	0.237	−0.317	0.235	0.142	−0.895	0.512
14804	0.261	−0.137	0.296	0.371	0.549	0.193	0.050	−1.485	0.185
14805	0.263	−0.120	0.185	0.237	−0.317	0.371	0.142	−0.895	0.185
14806	0.097	−1.181	0.467	0.237	−0.317	0.341	0.263	−0.120	0.512
14807	0.479	1.262	0.512	0.497	1.359	0.309	0.489	1.329	0.501
14808	0.445	1.043	0.142	0.509	1.434	0.478	0.466	1.178	0.450
14809	0.495	1.363	0.512	0.398	0.718	0.149	0.479	1.262	0.492
14810	0.463	1.161	0.262	0.509	1.434	0.512	0.479	1.262	0.224
14811	0.226	−0.356	0.481	0.234	−0.336	0.514	0.097	−1.181	0.262
14812	0.226	−0.356	0.142	0.234	−0.336	0.478	0.097	−1.181	0.481
14813	0.226	−0.356	0.513	0.234	−0.336	0.491	0.097	−1.181	0.097
14814	0.471	1.211	0.492	0.506	1.415	0.508	0.466	1.178	0.097
14815	0.508	1.447	0.467	0.477	1.227	0.422	0.432	0.958	0.481
14816	0.329	0.301	0.512	0.053	−1.504	0.462	0.224	−0.373	0.097
14817	0.097	−1.181	0.262	0.237	−0.317	0.462	0.263	−0.120	0.328
14818	0.297	0.099	0.481	0.152	−0.863	0.193	0.224	−0.373	0.262
14819	0.261	−0.137	0.409	0.275	−0.073	0.235	0.184	−0.625	0.296

14820	0.468	1.194	0.224	0.459	1.114	0.235	0.503	1.413	0.450
14821	0.184	-0.625	0.450	0.234	-0.336	0.444	0.142	-0.895	0.328
14822	0.226	-0.356	0.185	0.193	-0.600	0.193	0.326	0.285	0.185
14823	0.226	-0.356	0.358	0.371	0.549	0.371	0.295	0.082	0.358
14824	0.263	-0.120	0.262	0.345	0.379	0.193	0.097	-1.181	0.512
14825	0.224	-0.373	0.262	0.313	0.172	0.444	0.224	-0.373	0.450
14826	0.468	1.194	0.481	0.480	1.246	0.501	0.476	1.245	0.481
14827	0.187	-0.608	0.501	0.149	-0.882	0.512	0.226	-0.356	0.224
14828	0.516	1.498	0.262	0.509	1.434	0.273	0.453	1.093	0.262
14829	0.482	1.279	0.262	0.512	1.453	0.491	0.434	0.975	0.385
14830	0.505	1.430	0.508	0.480	1.246	0.478	0.479	1.262	0.385
14831	0.476	1.245	0.431	0.491	1.321	0.478	0.489	1.329	0.492
14832	0.466	1.178	0.358	0.503	1.396	0.193	0.432	0.958	0.224
14833	0.145	-0.878	0.262	0.424	0.888	0.462	0.184	-0.625	0.097
14834	0.508	1.447	0.142	0.459	1.114	0.501	0.384	0.655	0.501
14835	0.508	1.447	0.431	0.459	1.114	0.235	0.384	0.655	0.508
14836	0.142	-0.895	0.467	0.193	-0.600	0.193	0.224	-0.373	0.512
14837	0.187	-0.608	0.467	0.237	-0.317	0.149	0.358	0.487	0.431
14838	0.187	-0.608	0.467	0.237	-0.317	0.235	0.358	0.487	0.296
14839	0.387	0.672	0.328	0.447	1.039	0.491	0.495	1.363	0.409
14840	0.145	-0.878	0.492	0.196	-0.581	0.491	0.224	-0.373	0.409
14841	0.184	-0.625	0.467	0.371	0.549	0.462	0.142	-0.895	0.467
14842	0.263	-0.120	0.481	0.193	-0.600	0.462	0.266	-0.103	0.385
14843	0.408	0.807	0.262	0.444	1.020	0.341	0.503	1.413	0.097
14844	0.497	1.380	0.481	0.500	1.378	0.462	0.384	0.655	0.431
14845	0.405	0.790	0.358	0.509	1.434	0.491	0.508	1.447	0.185
14846	0.263	-0.120	0.512	0.275	-0.073	0.235	0.187	-0.608	0.492
14847	0.468	1.194	0.501	0.491	1.321	0.371	0.505	1.430	0.512
14848	0.505	1.430	0.328	0.477	1.227	0.514	0.487	1.312	0.296
14849	0.479	1.262	0.431	0.480	1.246	0.235	0.492	1.346	0.481
14850	0.226	-0.356	0.450	0.196	-0.581	0.235	0.184	-0.625	0.328
14851	0.413	0.841	0.296	0.462	1.133	0.444	0.487	1.312	0.501
14852	0.484	1.295	0.481	0.500	1.378	0.193	0.487	1.312	0.262
14853	0.261	-0.137	0.142	0.237	-0.317	0.514	0.050	-1.485	0.450
14854	0.187	-0.608	0.185	0.196	-0.581	0.053	0.184	-0.625	0.492
14855	0.261	-0.137	0.185	0.149	-0.882	0.514	0.326	0.285	0.431
14856	0.261	-0.137	0.450	0.234	-0.336	0.514	0.097	-1.181	0.512
14857	0.450	1.076	0.262	0.488	1.302	0.512	0.497	1.380	0.492
14858	0.508	1.447	0.409	0.488	1.302	0.235	0.495	1.363	0.185
14859	0.508	1.447	0.481	0.459	1.114	0.514	0.476	1.245	0.481
14860	0.508	1.447	0.512	0.459	1.114	0.444	0.476	1.245	0.481
14861	0.229	-0.339	0.512	0.342	0.361	0.341	0.142	-0.895	0.097
14862	0.516	1.498	0.185	0.462	1.133	0.478	0.495	1.363	0.501
14863	0.429	0.942	0.224	0.488	1.302	0.478	0.508	1.447	0.262
14864	0.224	-0.373	0.185	0.237	-0.317	0.508	0.184	-0.625	0.328
14865	0.187	-0.608	0.224	0.368	0.530	0.512	0.050	-1.485	0.262
14866	0.489	1.329	0.358	0.518	1.491	0.512	0.511	1.464	0.262
14867	0.097	-1.181	0.385	0.234	-0.336	0.501	0.358	0.487	0.512
14868	0.097	-1.181	0.385	0.234	-0.336	0.508	0.358	0.487	0.467
14869	0.408	0.807	0.224	0.444	1.020	0.149	0.426	0.925	0.224
14870	0.387	0.672	0.501	0.196	-0.581	0.444	0.097	-1.181	0.224
14871	0.505	1.430	0.467	0.512	1.453	0.273	0.476	1.245	0.450
14872	0.358	0.487	0.224	0.053	-1.504	0.102	0.142	-0.895	0.450
14873	0.484	1.295	0.224	0.512	1.453	0.444	0.508	1.447	0.358
14874	0.474	1.228	0.142	0.512	1.453	0.491	0.511	1.464	0.142
14875	0.474	1.228	0.450	0.512	1.453	0.501	0.508	1.447	0.142
14876	0.224	-0.373	0.185	0.234	-0.336	0.422	0.097	-1.181	0.508
14877	0.497	1.380	0.512	0.509	1.434	0.508	0.487	1.312	0.513
14878	0.503	1.413	0.501	0.450	1.057	0.491	0.382	0.638	0.450
14879	0.263	-0.120	0.185	0.342	0.361	0.462	0.413	0.841	0.481
14880	0.474	1.228	0.431	0.480	1.246	0.422	0.513	1.481	0.224

14881	0.484	1.295	0.185	0.480	1.246	0.501	0.508	1.447	0.450
14882	0.505	1.430	0.385	0.509	1.434	0.341	0.466	1.178	0.512
14883	0.505	1.430	0.358	0.512	1.453	0.512	0.505	1.430	0.512
14884	0.326	0.285	0.501	0.512	1.453	0.512	0.453	1.093	0.224
14885	0.432	0.958	0.296	0.500	1.378	0.309	0.408	0.807	0.501
14886	0.511	1.464	0.262	0.509	1.434	0.309	0.508	1.447	0.296
14887	0.226	-0.356	0.501	0.149	-0.882	0.273	0.329	0.301	0.501
14888	0.447	1.060	0.492	0.447	1.039	0.309	0.505	1.430	0.481
14889	0.097	-1.181	0.467	0.275	-0.073	0.309	0.187	-0.608	0.224
14890	0.263	-0.120	0.450	0.105	-1.165	0.444	0.184	-0.625	0.501
14891	0.487	1.312	0.224	0.447	1.039	0.149	0.450	1.076	0.385
14892	0.516	1.498	0.492	0.494	1.340	0.501	0.500	1.397	0.097
14893	0.508	1.447	0.185	0.494	1.340	0.491	0.497	1.380	0.508
14894	0.511	1.464	0.185	0.418	0.850	0.462	0.487	1.312	0.262
14895	0.508	1.447	0.142	0.503	1.396	0.478	0.505	1.430	0.296
14896	0.466	1.178	0.501	0.488	1.302	0.235	0.466	1.178	0.296
14897	0.447	1.060	0.296	0.459	1.114	0.514	0.505	1.430	0.467
14898	0.447	1.060	0.262	0.421	0.869	0.309	0.505	1.430	0.185
14899	0.466	1.178	0.185	0.497	1.359	0.398	0.500	1.397	0.501
14900	0.142	-0.895	0.097	0.345	0.379	0.193	0.050	-1.485	0.481
14901	0.482	1.279	0.262	0.515	1.472	0.512	0.511	1.464	0.513
14902	0.450	1.076	0.508	0.515	1.472	0.512	0.508	1.447	0.513
14903	0.000	?	0.492	0.307	0.135	0.508	0.263	-0.120	0.142
14904	0.000	?	0.185	0.307	0.135	0.512	0.263	-0.120	0.097
14905	0.000	?	0.262	0.272	-0.091	0.512	0.297	0.099	0.185
14906	0.142	-0.895	0.450	0.310	0.153	0.273	0.097	-1.181	0.142
14907	0.224	-0.373	0.450	0.307	0.135	0.193	0.355	0.470	0.262
14908	0.447	1.060	0.385	0.439	0.982	0.462	0.511	1.464	0.508
14909	0.261	-0.137	0.508	0.149	-0.882	0.491	0.226	-0.356	0.508
14910	0.489	1.329	0.296	0.503	1.396	0.491	0.513	1.481	0.262
14911	0.476	1.245	0.481	0.491	1.321	0.398	0.508	1.447	0.450
14912	0.463	1.161	0.492	0.459	1.114	0.398	0.511	1.464	0.467
14913	0.503	1.413	0.050	0.488	1.302	0.508	0.489	1.329	0.262
14914	0.224	-0.373	0.142	0.234	-0.336	0.512	0.097	-1.181	0.262
14915	0.468	1.194	0.224	0.518	1.491	0.508	0.500	1.397	0.224
14916	0.263	-0.120	0.328	0.310	0.153	0.478	0.263	-0.120	0.501
14917	0.329	0.301	0.328	0.404	0.756	0.462	0.142	-0.895	0.224
14918	0.329	0.301	0.409	0.196	-0.581	0.462	0.263	-0.120	0.467
14919	0.484	1.295	0.185	0.515	1.472	0.501	0.497	1.380	0.481
14920	0.497	1.380	0.224	0.509	1.434	0.341	0.511	1.464	0.142
14921	0.492	1.346	0.224	0.512	1.453	0.309	0.492	1.346	0.450
14922	0.497	1.380	0.262	0.506	1.415	0.462	0.492	1.346	0.262
14923	0.508	1.447	0.142	0.509	1.434	0.491	0.508	1.447	0.185
14924	0.355	0.470	0.224	0.275	-0.073	0.514	0.184	-0.625	0.185
14925	0.295	0.082	0.224	0.196	-0.581	0.491	0.097	-1.181	0.481
14926	0.432	0.958	0.185	0.462	1.133	0.102	0.468	1.194	0.142
14927	0.489	1.329	0.296	0.497	1.359	0.149	0.426	0.925	0.097
14928	0.511	1.464	0.358	0.491	1.321	0.512	0.487	1.312	0.512
14929	0.505	1.430	0.328	0.395	0.700	0.309	0.461	1.144	0.481
14930	0.505	1.430	0.512	0.395	0.700	0.309	0.461	1.144	0.512
14931	0.503	1.413	0.296	0.512	1.453	0.462	0.505	1.430	0.492
14932	0.508	1.447	0.224	0.518	1.491	0.235	0.511	1.464	0.262
14933	0.513	1.481	0.050	0.512	1.453	0.371	0.511	1.464	0.467
14934	0.503	1.413	0.050	0.474	1.208	0.309	0.450	1.076	0.185
14935	0.463	1.161	0.358	0.462	1.133	0.102	0.482	1.279	0.185
14936	0.479	1.262	0.262	0.465	1.152	0.273	0.513	1.481	0.224
14937	0.500	1.397	0.409	0.497	1.359	0.478	0.511	1.464	0.512
14938	0.097	-1.181	0.262	0.345	0.379	0.149	0.184	-0.625	0.050
14939	0.263	-0.120	0.050	0.307	0.135	0.309	0.187	-0.608	0.142
14940	0.489	1.329	0.513	0.456	1.095	0.512	0.511	1.464	0.262
14941	0.479	1.262	0.512	0.494	1.340	0.444	0.505	1.430	0.224

14942	0.426	0.925	0.492	0.509	1.434	0.512	0.450	1.076	0.142
14943	0.447	1.060	0.358	0.491	1.321	0.512	0.489	1.329	0.450
14944	0.226	−0.356	0.450	0.102	−1.184	0.193	0.224	−0.373	0.492
14945	0.297	0.099	0.224	0.152	−0.863	0.235	0.261	−0.137	0.262
14946	0.503	1.413	0.224	0.518	1.491	0.309	0.482	1.279	0.224
14947	0.142	−0.895	0.508	0.310	0.153	0.309	0.097	−1.181	0.492
14948	0.142	−0.895	0.508	0.310	0.153	0.491	0.097	−1.181	0.481
14949	0.479	1.262	0.450	0.459	1.114	0.273	0.482	1.279	0.513
14950	0.361	0.504	0.492	0.234	−0.336	0.341	0.000	?	0.050
14951	0.097	−1.181	0.142	0.371	0.549	0.444	0.097	−1.181	0.481
14952	0.263	−0.120	0.142	0.310	0.153	0.512	0.329	0.301	0.481
14953	0.405	0.790	0.450	0.102	−1.184	0.462	0.263	−0.120	0.385
14954	0.447	1.060	0.185	0.272	−0.091	0.508	0.297	0.099	0.450
14955	0.432	0.958	0.328	0.482	1.265	0.309	0.445	1.043	0.185
14956	0.224	−0.373	0.185	0.149	−0.882	0.501	0.326	0.285	0.508
14957	0.184	−0.625	0.185	0.310	0.153	0.512	0.184	−0.625	0.508
14958	0.489	1.329	0.501	0.512	1.453	0.491	0.355	0.470	0.501
14959	0.476	1.245	0.224	0.444	1.020	0.235	0.513	1.481	0.224
14960	0.505	1.430	0.224	0.512	1.453	0.273	0.445	1.043	0.050
14961	0.495	1.363	0.262	0.506	1.415	0.273	0.476	1.245	0.262
14962	0.297	0.099	0.328	0.193	−0.600	0.193	0.224	−0.373	0.142
14963	0.097	−1.181	0.296	0.234	−0.336	0.508	0.261	−0.137	0.262
14964	0.329	0.301	0.501	0.307	0.135	0.422	0.384	0.655	0.224
14965	0.329	0.301	0.492	0.307	0.135	0.149	0.384	0.655	0.185
14966	0.497	1.380	0.431	0.497	1.359	0.491	0.489	1.329	0.296
14967	0.297	0.099	0.508	0.275	−0.073	0.371	0.226	−0.356	0.224
14968	0.097	−1.181	0.296	0.345	0.379	0.501	0.097	−1.181	0.385
14969	0.508	1.447	0.328	0.447	1.039	0.193	0.500	1.397	0.142
14970	0.482	1.279	0.450	0.509	1.434	0.193	0.503	1.413	0.513
14971	0.500	1.397	0.385	0.459	1.114	0.462	0.505	1.430	0.328
14972	0.453	1.093	0.262	0.509	1.434	0.478	0.429	0.942	0.328
14973	0.184	−0.625	0.508	0.310	0.153	0.491	0.297	0.099	0.296
14974	0.492	1.346	0.385	0.497	1.359	0.478	0.408	0.807	0.296
14975	0.505	1.430	0.512	0.506	1.415	0.422	0.408	0.807	0.358
14976	0.482	1.279	0.262	0.488	1.302	0.422	0.466	1.178	0.262
14977	0.261	−0.137	0.467	0.234	−0.336	0.422	0.389	0.689	0.467
14978	0.184	−0.625	0.224	0.275	−0.073	0.235	0.097	−1.181	0.262
14979	0.297	0.099	0.224	0.272	−0.091	0.462	0.432	0.958	0.296
14980	0.184	−0.625	0.224	0.193	−0.600	0.235	0.184	−0.625	0.481
14981	0.466	1.178	0.481	0.509	1.434	0.273	0.489	1.329	0.358
14982	0.513	1.481	0.450	0.421	0.869	0.193	0.511	1.464	0.501
14983	0.142	−0.895	0.224	0.149	−0.882	0.193	0.295	0.082	0.185
14984	0.511	1.464	0.513	0.494	1.340	0.444	0.484	1.295	0.512
14985	0.261	−0.137	0.513	0.374	0.568	0.508	0.332	0.318	0.142
14986	0.479	1.262	0.513	0.503	1.396	0.193	0.500	1.397	0.097
14987	0.145	−0.878	0.224	0.193	−0.600	0.193	0.263	−0.120	0.492
14988	0.000	?	0.142	0.196	−0.581	0.398	0.450	1.076	0.492
14989	0.508	1.447	0.492	0.459	1.114	0.478	0.492	1.346	0.501
14990	0.489	1.329	0.492	0.480	1.246	0.371	0.426	0.925	0.358
14991	0.466	1.178	0.262	0.488	1.302	0.149	0.505	1.430	0.185
14992	0.513	1.481	0.512	0.474	1.208	0.309	0.487	1.312	0.097
14993	0.263	−0.120	0.512	0.424	0.888	0.478	0.184	−0.625	0.431
14994	0.479	1.262	0.513	0.421	0.869	0.491	0.476	1.245	0.097
14995	0.405	0.790	0.097	0.424	0.888	0.491	0.482	1.279	0.262
14996	0.224	−0.373	0.512	0.234	−0.336	0.514	0.097	−1.181	0.185
14997	0.447	1.060	0.512	0.462	1.133	0.193	0.500	1.397	0.142
14998	0.184	−0.625	0.097	0.234	−0.336	0.491	0.263	−0.120	0.467
14999	0.358	0.487	0.142	0.272	−0.091	0.501	0.332	0.318	0.185
15000	0.261	−0.137	0.097	0.193	−0.600	0.444	0.261	−0.137	0.224
15001	0.261	−0.137	0.508	0.193	−0.600	0.235	0.261	−0.137	0.296
15002	0.476	1.245	0.262	0.439	0.982	0.508	0.511	1.464	0.142

15003	0.495	1.363	0.467	0.506	1.415	0.193	0.466	1.178	0.328
15004	0.261	−0.137	0.508	0.193	−0.600	0.422	0.224	−0.373	0.481
15005	0.261	−0.137	0.296	0.193	−0.600	0.508	0.224	−0.373	0.492
15006	0.508	1.447	0.508	0.395	0.700	0.193	0.450	1.076	0.501
15007	0.429	0.942	0.450	0.477	1.227	0.512	0.447	1.060	0.492
15008	0.361	0.504	0.185	0.371	0.549	0.478	0.361	0.504	0.185
15009	0.263	−0.120	0.501	0.149	−0.882	0.371	0.142	−0.895	0.185
15010	0.142	−0.895	0.512	0.307	0.135	0.444	0.145	−0.878	0.467
15011	0.432	0.958	0.501	0.471	1.189	0.309	0.505	1.430	0.296
15012	0.471	1.211	0.481	0.482	1.265	0.309	0.511	1.464	0.385
15013	0.471	1.211	0.185	0.488	1.302	0.309	0.447	1.060	0.512
15014	0.468	1.194	0.467	0.512	1.453	0.422	0.476	1.245	0.508
15015	0.329	0.301	0.097	0.193	−0.600	0.235	0.224	−0.373	0.508
15016	0.495	1.363	0.262	0.494	1.340	0.508	0.445	1.043	0.385
15017	0.474	1.228	0.467	0.503	1.396	0.508	0.511	1.464	0.385
15018	0.474	1.228	0.501	0.444	1.020	0.462	0.508	1.447	0.262
15019	0.261	−0.137	0.501	0.237	−0.317	0.462	0.226	−0.356	0.262
15020	0.479	1.262	0.481	0.503	1.396	0.309	0.505	1.430	0.262
15021	0.361	0.504	0.450	0.196	−0.581	0.235	0.295	0.082	0.450
15022	0.513	1.481	0.224	0.424	0.888	0.273	0.497	1.380	0.385
15023	0.500	1.397	0.224	0.509	1.434	0.273	0.479	1.262	0.142
15024	0.332	0.318	0.450	0.193	−0.600	0.235	0.224	−0.373	0.467
15025	0.426	0.925	0.501	0.518	1.491	0.422	0.492	1.346	0.450
15026	0.450	1.076	0.262	0.477	1.227	0.422	0.382	0.638	0.467
15027	0.300	0.116	0.296	0.371	0.549	0.309	0.097	−1.181	0.142
15028	0.482	1.279	0.328	0.442	1.001	0.508	0.503	1.413	0.262
15029	0.142	−0.895	0.224	0.307	0.135	0.501	0.263	−0.120	0.501
15030	0.184	−0.625	0.142	0.310	0.153	0.193	0.297	0.099	0.481
15031	0.184	−0.625	0.142	0.307	0.135	0.193	0.297	0.099	0.097
15032	0.450	1.076	0.467	0.424	0.888	0.193	0.463	1.161	0.328
15033	0.266	−0.103	0.508	0.234	−0.336	0.491	0.184	−0.625	0.492
15034	0.408	0.807	0.224	0.512	1.453	0.149	0.500	1.397	0.512
15035	0.479	1.262	0.224	0.509	1.434	0.501	0.479	1.262	0.501
15036	0.511	1.464	0.262	0.459	1.114	0.422	0.508	1.447	0.185
15037	0.511	1.464	0.142	0.459	1.114	0.273	0.508	1.447	0.450
15038	0.184	−0.625	0.185	0.310	0.153	0.512	0.142	−0.895	0.450
15039	0.224	−0.373	0.262	0.237	−0.317	0.193	0.097	−1.181	0.185
15040	0.184	−0.625	0.224	0.275	−0.073	0.193	0.184	−0.625	0.262
15041	0.224	−0.373	0.142	0.275	−0.073	0.193	0.142	−0.895	0.224
15042	0.358	0.487	0.512	0.237	−0.317	0.398	0.261	−0.137	0.501
15043	0.382	0.638	0.224	0.421	0.869	0.149	0.505	1.430	0.296
15044	0.382	0.638	0.450	0.421	0.869	0.341	0.505	1.430	0.508
15045	0.224	−0.373	0.224	0.307	0.135	0.491	0.261	−0.137	0.492
15046	0.503	1.413	0.224	0.506	1.415	0.508	0.447	1.060	0.224
15047	0.466	1.178	0.450	0.500	1.378	0.444	0.466	1.178	0.467
15048	0.226	−0.356	0.481	0.193	−0.600	0.444	0.263	−0.120	0.467
15049	0.226	−0.356	0.481	0.193	−0.600	0.053	0.263	−0.120	0.358
15050	0.142	−0.895	0.481	0.193	−0.600	0.512	0.226	−0.356	0.492
15051	0.453	1.093	0.296	0.488	1.302	0.273	0.497	1.380	0.492
15052	0.184	−0.625	0.296	0.149	−0.882	0.273	0.226	−0.356	0.513
15053	0.513	1.481	0.185	0.500	1.378	0.235	0.463	1.161	0.512
15054	0.500	1.397	0.296	0.418	0.850	0.501	0.479	1.262	0.431
15055	0.187	−0.608	0.431	0.272	−0.091	0.235	0.142	−0.895	0.467
15056	0.429	0.942	0.409	0.512	1.453	0.193	0.445	1.043	0.328
15057	0.184	−0.625	0.512	0.193	−0.600	0.341	0.261	−0.137	0.328
15058	0.387	0.672	0.450	0.193	−0.600	0.309	0.184	−0.625	0.501
15059	0.361	0.504	0.450	0.193	−0.600	0.341	0.184	−0.625	0.512
15060	0.503	1.413	0.450	0.398	0.718	0.491	0.482	1.279	0.513
15061	0.297	0.099	0.508	0.149	−0.882	0.444	0.142	−0.895	0.431
15062	0.263	−0.120	0.501	0.342	0.361	0.341	0.097	−1.181	0.508
15063	0.505	1.430	0.501	0.491	1.321	0.102	0.505	1.430	0.262

15064	0.495	1.363	0.185	0.512	1.453	0.444	0.476	1.245	0.262
15065	0.471	1.211	0.185	0.444	1.020	0.444	0.508	1.447	0.508
15066	0.453	1.093	0.328	0.447	1.039	0.273	0.487	1.312	0.492
15067	0.226	−0.356	0.358	0.053	−1.504	0.478	0.263	−0.120	0.050
15068	0.495	1.363	0.262	0.512	1.453	0.341	0.463	1.161	0.185
15069	0.184	−0.625	0.224	0.272	−0.091	0.444	0.187	−0.608	0.142
15070	0.184	−0.625	0.492	0.272	−0.091	0.501	0.187	−0.608	0.142
15071	0.142	−0.895	0.450	0.237	−0.317	0.149	0.224	−0.373	0.185
15072	0.505	1.430	0.409	0.497	1.359	0.309	0.511	1.464	0.185
15073	0.295	0.082	0.492	0.234	−0.336	0.193	0.050	−1.485	0.501
15074	0.261	−0.137	0.097	0.193	−0.600	0.422	0.142	−0.895	0.467
15075	0.184	−0.625	0.481	0.339	0.342	0.422	0.263	−0.120	0.224
15076	0.097	−1.181	0.513	0.307	0.135	0.501	0.226	−0.356	0.296
15077	0.266	−0.103	0.513	0.342	0.361	0.235	0.142	−0.895	0.262
15078	0.503	1.413	0.142	0.491	1.321	0.273	0.445	1.043	0.450
15079	0.492	1.346	0.097	0.444	1.020	0.341	0.484	1.295	0.262
15080	0.184	−0.625	0.224	0.345	0.379	0.371	0.261	−0.137	0.050
15081	0.261	−0.137	0.185	0.102	−1.184	0.341	0.229	−0.339	0.050
15082	0.450	1.076	0.262	0.444	1.020	0.235	0.492	1.346	0.296
15083	0.450	1.076	0.262	0.444	1.020	0.193	0.482	1.279	0.492
15084	0.384	0.655	0.481	0.275	−0.073	0.341	0.000	?	0.224
15085	0.508	1.447	0.481	0.477	1.227	0.193	0.508	1.447	0.450
15086	0.297	0.099	0.508	0.345	0.379	0.273	0.050	−1.485	0.097
15087	0.476	1.245	0.501	0.444	1.020	0.422	0.479	1.262	0.385
15088	0.487	1.312	0.296	0.500	1.378	0.491	0.479	1.262	0.513
15089	0.050	−1.485	0.296	0.149	−0.882	0.309	0.387	0.672	0.501
15090	0.142	−0.895	0.450	0.310	0.153	0.193	0.447	1.060	0.501
15091	0.224	−0.373	0.296	0.193	−0.600	0.273	0.184	−0.625	0.501
15092	0.326	0.285	0.450	0.421	0.869	0.273	0.511	1.464	0.328
15093	0.326	0.285	0.501	0.421	0.869	0.462	0.511	1.464	0.431
15094	0.408	0.807	0.224	0.500	1.378	0.273	0.505	1.430	0.262
15095	0.187	−0.608	0.142	0.234	−0.336	0.491	0.226	−0.356	0.224
15096	0.226	−0.356	0.508	0.272	−0.091	0.273	0.050	−1.485	0.508
15097	0.226	−0.356	0.513	0.345	0.379	0.398	0.261	−0.137	0.501
15098	0.261	−0.137	0.224	0.374	0.568	0.508	0.142	−0.895	0.492
15099	0.142	−0.895	0.492	0.342	0.361	0.444	0.261	−0.137	0.501
15100	0.226	−0.356	0.508	0.234	−0.336	0.501	0.224	−0.373	0.512
15101	0.226	−0.356	0.508	0.193	−0.600	0.398	0.184	−0.625	0.508
15102	0.184	−0.625	0.508	0.342	0.361	0.491	0.297	0.099	0.513
15103	0.297	0.099	0.492	0.193	−0.600	0.341	0.226	−0.356	0.385
15104	0.361	0.504	0.467	0.275	−0.073	0.235	0.387	0.672	0.501
15105	0.329	0.301	0.492	0.427	0.907	0.514	0.142	−0.895	0.358
15106	0.513	1.481	0.492	0.494	1.340	0.514	0.508	1.447	0.185
15107	0.297	0.099	0.467	0.313	0.172	0.491	0.326	0.285	0.328
15108	0.224	−0.373	0.492	0.196	−0.581	0.491	0.326	0.285	0.512
15109	0.050	−1.485	0.296	0.272	−0.091	0.309	0.297	0.099	0.358
15110	0.050	−1.485	0.296	0.272	−0.091	0.341	0.297	0.099	0.328
15111	0.355	0.470	0.481	0.468	1.170	0.508	0.361	0.504	0.481
15112	0.266	−0.103	0.224	0.275	−0.073	0.309	0.261	−0.137	0.512
15113	0.411	0.824	0.262	0.488	1.302	0.235	0.463	1.161	0.296
15114	0.266	−0.103	0.224	0.275	−0.073	0.235	0.261	−0.137	0.501
15115	0.050	−1.485	0.492	0.395	0.700	0.235	0.300	0.116	0.328
15116	0.511	1.464	0.508	0.512	1.453	0.501	0.484	1.295	0.508
15117	0.511	1.464	0.512	0.444	1.020	0.149	0.361	0.504	0.431
15118	0.492	1.346	0.296	0.497	1.359	0.149	0.497	1.380	0.431
15119	0.361	0.504	0.513	0.398	0.718	0.149	0.184	−0.625	0.296
15120	0.450	1.076	0.467	0.494	1.340	0.398	0.508	1.447	0.142
15121	0.224	−0.373	0.050	0.342	0.361	0.235	0.145	−0.878	0.050
15122	0.224	−0.373	0.501	0.234	−0.336	0.508	0.097	−1.181	0.262
15123	0.508	1.447	0.185	0.512	1.453	0.501	0.489	1.329	0.185
15124	0.508	1.447	0.224	0.512	1.453	0.478	0.489	1.329	0.185

15125	0.453	1.093	0.262	0.494	1.340	0.508	0.500	1.397	0.450
15126	0.495	1.363	0.492	0.491	1.321	0.053	0.355	0.470	0.492
15127	0.145	-0.878	0.481	0.307	0.135	0.102	0.184	-0.625	0.508
15128	0.142	-0.895	0.467	0.345	0.379	0.514	0.097	-1.181	0.492
15129	0.450	1.076	0.467	0.515	1.472	0.341	0.426	0.925	0.050
15130	0.184	-0.625	0.224	0.313	0.172	0.193	0.097	-1.181	0.185
15131	0.326	0.285	0.467	0.237	-0.317	0.491	0.261	-0.137	0.501
15132	0.184	-0.625	0.512	0.234	-0.336	0.512	0.184	-0.625	0.512
15133	0.184	-0.625	0.385	0.234	-0.336	0.462	0.142	-0.895	0.513
15134	0.503	1.413	0.492	0.500	1.378	0.309	0.463	1.161	0.431
15135	0.229	-0.339	0.431	0.149	-0.882	0.501	0.184	-0.625	0.050
15136	0.226	-0.356	0.450	0.149	-0.882	0.235	0.226	-0.356	0.097
15137	0.261	-0.137	0.262	0.152	-0.863	0.235	0.297	0.099	0.431
15138	0.329	0.301	0.409	0.401	0.737	0.235	0.142	-0.895	0.431
15139	0.295	0.082	0.185	0.234	-0.336	0.478	0.326	0.285	0.142
15140	0.500	1.397	0.142	0.506	1.415	0.512	0.482	1.279	0.492
15141	0.492	1.346	0.481	0.497	1.359	0.193	0.487	1.312	0.450
15142	0.434	0.975	0.185	0.474	1.208	0.512	0.495	1.363	0.450
15143	0.505	1.430	0.185	0.506	1.415	0.512	0.487	1.312	0.512
15144	0.297	0.099	0.481	0.053	-1.504	0.512	0.184	-0.625	0.431
15145	0.329	0.301	0.508	0.102	-1.184	0.193	0.184	-0.625	0.512
15146	0.447	1.060	0.492	0.509	1.434	0.149	0.463	1.161	0.508
15147	0.384	0.655	0.296	0.345	0.379	0.512	0.297	0.099	0.492
15148	0.261	-0.137	0.142	0.193	-0.600	0.491	0.384	0.655	0.508
15149	0.511	1.464	0.142	0.485	1.283	0.235	0.505	1.430	0.512
15150	0.382	0.638	0.142	0.512	1.453	0.444	0.505	1.430	0.142
15151	0.508	1.447	0.501	0.462	1.133	0.371	0.505	1.430	0.501
15152	0.261	-0.137	0.492	0.307	0.135	0.491	0.387	0.672	0.142
15153	0.468	1.194	0.481	0.506	1.415	0.514	0.384	0.655	0.142
15154	0.226	-0.356	0.467	0.234	-0.336	0.273	0.263	-0.120	0.050
15155	0.226	-0.356	0.142	0.234	-0.336	0.501	0.263	-0.120	0.501
15156	0.226	-0.356	0.142	0.234	-0.336	0.501	0.263	-0.120	0.512
15157	0.479	1.262	0.512	0.482	1.265	0.273	0.447	1.060	0.492
15158	0.445	1.043	0.512	0.512	1.453	0.341	0.384	0.655	0.050
15159	0.226	-0.356	0.501	0.196	-0.581	0.235	0.142	-0.895	0.467
15160	0.513	1.481	0.142	0.509	1.434	0.508	0.463	1.161	0.481
15161	0.513	1.481	0.296	0.509	1.434	0.341	0.445	1.043	0.050
15162	0.513	1.481	0.409	0.509	1.434	0.501	0.463	1.161	0.431
15163	0.226	-0.356	0.224	0.196	-0.581	0.462	0.142	-0.895	0.050
15164	0.142	-0.895	0.501	0.149	-0.882	0.149	0.266	-0.103	0.185
15165	0.489	1.329	0.262	0.512	1.453	0.501	0.497	1.380	0.185
15166	0.489	1.329	0.512	0.488	1.302	0.508	0.476	1.245	0.467
15167	0.263	-0.120	0.431	0.237	-0.317	0.444	0.097	-1.181	0.492
15168	0.000	?	0.492	0.447	1.039	0.478	0.326	0.285	0.467
15169	0.503	1.413	0.501	0.368	0.530	0.491	0.497	1.380	0.467
15170	0.503	1.413	0.450	0.494	1.340	0.491	0.513	1.481	0.185
15171	0.505	1.430	0.224	0.518	1.491	0.478	0.500	1.397	0.450
15172	0.097	-1.181	0.501	0.278	-0.054	0.309	0.184	-0.625	0.467
15173	0.500	1.397	0.385	0.506	1.415	0.444	0.450	1.076	0.508
15174	0.500	1.397	0.492	0.506	1.415	0.235	0.453	1.093	0.481
15175	0.097	-1.181	0.262	0.275	-0.073	0.398	0.187	-0.608	0.513
15176	0.142	-0.895	0.508	0.339	0.342	0.501	0.263	-0.120	0.513
15177	0.097	-1.181	0.501	0.237	-0.317	0.491	0.226	-0.356	0.185
15178	0.503	1.413	0.508	0.509	1.434	0.491	0.500	1.397	0.512
15179	0.263	-0.120	0.262	0.342	0.361	0.422	0.297	0.099	0.142
15180	0.463	1.161	0.467	0.497	1.359	0.491	0.505	1.430	0.185
15181	0.508	1.447	0.224	0.462	1.133	0.102	0.495	1.363	0.512
15182	0.297	0.099	0.224	0.152	-0.863	0.102	0.224	-0.373	0.185
15183	0.500	1.397	0.358	0.500	1.378	0.491	0.466	1.178	0.185
15184	0.450	1.076	0.450	0.506	1.415	0.398	0.466	1.178	0.450
15185	0.187	-0.608	0.508	0.447	1.039	0.235	0.358	0.487	0.501

15186	0.500	1.397	0.385	0.474	1.208	0.235	0.489	1.329	0.492
15187	0.511	1.464	0.296	0.485	1.283	0.193	0.489	1.329	0.185
15188	0.503	1.413	0.513	0.488	1.302	0.193	0.516	1.498	0.262
15189	0.484	1.295	0.142	0.480	1.246	0.273	0.511	1.464	0.385
15190	0.184	-0.625	0.512	0.307	0.135	0.273	0.437	0.992	0.358
15191	0.468	1.194	0.513	0.442	1.001	0.398	0.466	1.178	0.450
15192	0.097	-1.181	0.262	0.234	-0.336	0.501	0.332	0.318	0.481
15193	0.261	-0.137	0.328	0.401	0.737	0.102	0.332	0.318	0.481
15194	0.463	1.161	0.142	0.494	1.340	0.398	0.503	1.413	0.508
15195	0.497	1.380	0.508	0.485	1.283	0.371	0.511	1.464	0.185
15196	0.497	1.380	0.050	0.485	1.283	0.341	0.513	1.481	0.224
15197	0.479	1.262	0.050	0.421	0.869	0.102	0.426	0.925	0.467
15198	0.447	1.060	0.450	0.494	1.340	0.341	0.497	1.380	0.467
15199	0.226	-0.356	0.224	0.102	-1.184	0.309	0.261	-0.137	0.501
15200	0.226	-0.356	0.262	0.102	-1.184	0.235	0.261	-0.137	0.185
15201	0.447	1.060	0.185	0.491	1.321	0.444	0.500	1.397	0.224
15202	0.503	1.413	0.224	0.398	0.718	0.149	0.492	1.346	0.512
15203	0.263	-0.120	0.296	0.237	-0.317	0.422	0.050	-1.485	0.409
15204	0.297	0.099	0.262	0.237	-0.317	0.273	0.187	-0.608	0.501
15205	0.329	0.301	0.262	0.193	-0.600	0.341	0.142	-0.895	0.262
15206	0.226	-0.356	0.467	0.193	-0.600	0.478	0.142	-0.895	0.481
15207	0.142	-0.895	0.512	0.278	-0.054	0.478	0.184	-0.625	0.508
15208	0.142	-0.895	0.467	0.278	-0.054	0.478	0.184	-0.625	0.450
15209	0.461	1.144	0.481	0.398	0.718	0.478	0.503	1.413	0.501
15210	0.508	1.447	0.492	0.500	1.378	0.235	0.466	1.178	0.501
15211	0.224	-0.373	0.224	0.102	-1.184	0.273	0.224	-0.373	0.224
15212	0.224	-0.373	0.481	0.404	0.756	0.149	0.297	0.099	0.481
15213	0.261	-0.137	0.385	0.377	0.587	0.193	0.263	-0.120	0.481
15214	0.142	-0.895	0.513	0.345	0.379	0.444	0.447	1.060	0.513
15215	0.184	-0.625	0.512	0.102	-1.184	0.371	0.261	-0.137	0.050
15216	0.263	-0.120	0.224	0.339	0.342	0.512	0.050	-1.485	0.431
15217	0.226	-0.356	0.142	0.307	0.135	0.501	0.050	-1.485	0.431
15218	0.145	-0.878	0.450	0.234	-0.336	0.422	0.295	0.082	0.512
15219	0.508	1.447	0.409	0.442	1.001	0.444	0.489	1.329	0.296
15220	0.229	-0.339	0.508	0.149	-0.882	0.478	0.224	-0.373	0.501
15221	0.450	1.076	0.142	0.427	0.907	0.462	0.453	1.093	0.142
15222	0.226	-0.356	0.224	0.272	-0.091	0.478	0.097	-1.181	0.142
15223	0.226	-0.356	0.328	0.342	0.361	0.341	0.387	0.672	0.501
15224	0.450	1.076	0.185	0.477	1.227	0.193	0.513	1.481	0.467
15225	0.479	1.262	0.492	0.477	1.227	0.309	0.495	1.363	0.224
15226	0.479	1.262	0.185	0.477	1.227	0.422	0.495	1.363	0.224
15227	0.479	1.262	0.142	0.477	1.227	0.273	0.495	1.363	0.492
15228	0.300	0.116	0.358	0.237	-0.317	0.235	0.326	0.285	0.262
15229	0.297	0.099	0.262	0.278	-0.054	0.508	0.432	0.958	0.467
15230	0.184	-0.625	0.513	0.149	-0.882	0.491	0.261	-0.137	0.467
15231	0.297	0.099	0.467	0.193	-0.600	0.371	0.224	-0.373	0.262
15232	0.429	0.942	0.467	0.439	0.982	0.422	0.505	1.430	0.142
15233	0.408	0.807	0.097	0.371	0.549	0.193	0.505	1.430	0.142
15234	0.513	1.481	0.512	0.515	1.472	0.462	0.492	1.346	0.450
15235	0.445	1.043	0.512	0.500	1.378	0.512	0.497	1.380	0.296
15236	0.447	1.060	0.185	0.418	0.850	0.512	0.511	1.464	0.328
15237	0.447	1.060	0.467	0.439	0.982	0.193	0.508	1.447	0.501
15238	0.511	1.464	0.512	0.480	1.246	0.309	0.513	1.481	0.224
15239	0.500	1.397	0.185	0.462	1.133	0.273	0.387	0.672	0.097
15240	0.497	1.380	0.142	0.477	1.227	0.309	0.505	1.430	0.142
15241	0.187	-0.608	0.481	0.342	0.361	0.149	0.361	0.504	0.142
15242	0.184	-0.625	0.481	0.193	-0.600	0.149	0.187	-0.608	0.262
15243	0.329	0.301	0.450	0.313	0.172	0.478	0.329	0.301	0.142
15244	0.361	0.504	0.431	0.424	0.888	0.478	0.513	1.481	0.296
15245	0.263	-0.120	0.296	0.272	-0.091	0.491	0.358	0.487	0.512
15246	0.226	-0.356	0.501	0.234	-0.336	0.508	0.329	0.301	0.513

15247	0.487	1.312	0.501	0.506	1.415	0.235	0.479	1.262	0.492
15248	0.450	1.076	0.492	0.491	1.321	0.193	0.508	1.447	0.481
15249	0.411	0.824	0.513	0.368	0.530	0.501	0.297	0.099	0.512
15250	0.492	1.346	0.467	0.418	0.850	0.462	0.497	1.380	0.185
15251	0.097	−1.181	0.097	0.193	−0.600	0.462	0.326	0.285	0.501
15252	0.474	1.228	0.409	0.462	1.133	0.491	0.511	1.464	0.262
15253	0.511	1.464	0.431	0.512	1.453	0.341	0.426	0.925	0.492
15254	0.511	1.464	0.508	0.512	1.453	0.309	0.426	0.925	0.450
15255	0.142	−0.895	0.296	0.193	−0.600	0.508	0.295	0.082	0.224
15256	0.097	−1.181	0.296	0.313	0.172	0.491	0.142	−0.895	0.296
15257	0.226	−0.356	0.262	0.275	−0.073	0.273	0.050	−1.485	0.385
15258	0.187	−0.608	0.097	0.307	0.135	0.478	0.261	−0.137	0.512
15259	0.263	−0.120	0.224	0.152	−0.863	0.512	0.184	−0.625	0.450
15260	0.263	−0.120	0.224	0.152	−0.863	0.514	0.184	−0.625	0.501
15261	0.482	1.279	0.409	0.480	1.246	0.444	0.453	1.093	0.142
15262	0.479	1.262	0.097	0.474	1.208	0.512	0.489	1.329	0.296
15263	0.503	1.413	0.262	0.488	1.302	0.491	0.505	1.430	0.262
15264	0.497	1.380	0.142	0.503	1.396	0.508	0.492	1.346	0.262
15265	0.297	0.099	0.512	0.237	−0.317	0.478	0.050	−1.485	0.450
15266	0.297	0.099	0.142	0.196	−0.581	0.478	0.184	−0.625	0.358
15267	0.450	1.076	0.328	0.500	1.378	0.478	0.495	1.363	0.097
15268	0.297	0.099	0.508	0.465	1.152	0.273	0.503	1.413	0.501
15269	0.450	1.076	0.097	0.462	1.133	0.273	0.505	1.430	0.185
15270	0.503	1.413	0.512	0.482	1.265	0.341	0.426	0.925	0.385
15271	0.224	−0.373	0.508	0.339	0.342	0.193	0.050	−1.485	0.513
15272	0.145	−0.878	0.508	0.310	0.153	0.273	0.097	−1.181	0.513
15273	0.505	1.430	0.262	0.515	1.472	0.273	0.426	0.925	0.328
15274	0.521	1.531	0.512	0.491	1.321	0.501	0.429	0.942	0.512
15275	0.226	−0.356	0.512	0.272	−0.091	0.491	0.142	−0.895	0.492
15276	0.489	1.329	0.481	0.477	1.227	0.478	0.487	1.312	0.296
15277	0.505	1.430	0.431	0.506	1.415	0.193	0.447	1.060	0.501
15278	0.505	1.430	0.431	0.509	1.434	0.501	0.447	1.060	0.508
15279	0.508	1.447	0.450	0.442	1.001	0.512	0.505	1.430	0.328
15280	0.487	1.312	0.050	0.509	1.434	0.398	0.429	0.942	0.358
15281	0.466	1.178	0.142	0.491	1.321	0.514	0.505	1.430	0.512
15282	0.489	1.329	0.224	0.509	1.434	0.309	0.503	1.413	0.512
15283	0.492	1.346	0.501	0.477	1.227	0.273	0.484	1.295	0.467
15284	0.468	1.194	0.358	0.477	1.227	0.102	0.497	1.380	0.431
15285	0.492	1.346	0.296	0.480	1.246	0.491	0.505	1.430	0.224
15286	0.297	0.099	0.224	0.275	−0.073	0.478	0.142	−0.895	0.512
15287	0.297	0.099	0.492	0.275	−0.073	0.491	0.000	?	0.508
15288	0.479	1.262	0.328	0.342	0.361	0.491	0.495	1.363	0.513
15289	0.224	−0.373	0.492	0.193	−0.600	0.235	0.142	−0.895	0.501
15290	0.261	−0.137	0.512	0.275	−0.073	0.508	0.142	−0.895	0.409
15291	0.224	−0.373	0.224	0.278	−0.054	0.508	0.050	−1.485	0.296
15292	0.495	1.363	0.467	0.500	1.378	0.491	0.495	1.363	0.185
15293	0.508	1.447	0.262	0.491	1.321	0.501	0.508	1.447	0.492
15294	0.511	1.464	0.262	0.477	1.227	0.514	0.492	1.346	0.492
15295	0.300	0.116	0.481	0.193	−0.600	0.508	0.050	−1.485	0.097
15296	0.508	1.447	0.450	0.500	1.378	0.102	0.463	1.161	0.097
15297	0.466	1.178	0.262	0.512	1.453	0.512	0.474	1.228	0.097
15298	0.050	−1.485	0.296	0.401	0.737	0.235	0.050	−1.485	0.328
15299	0.503	1.413	0.385	0.509	1.434	0.235	0.426	0.925	0.262
15300	0.184	−0.625	0.481	0.310	0.153	0.478	0.050	−1.485	0.224
15301	0.226	−0.356	0.224	0.272	−0.091	0.235	0.184	−0.625	0.224
15302	0.263	−0.120	0.450	0.102	−1.184	0.193	0.184	−0.625	0.224
15303	0.495	1.363	0.296	0.485	1.283	0.508	0.463	1.161	0.262
15304	0.482	1.279	0.492	0.471	1.189	0.444	0.492	1.346	0.262
15305	0.466	1.178	0.224	0.488	1.302	0.462	0.463	1.161	0.512
15306	0.466	1.178	0.481	0.488	1.302	0.235	0.463	1.161	0.185
15307	0.224	−0.373	0.328	0.237	−0.317	0.149	0.184	−0.625	0.262

15308	0.474	1.228	0.296	0.509	1.434	0.341	0.445	1.043	0.431
15309	0.508	1.447	0.508	0.509	1.434	0.235	0.461	1.144	0.185
15310	0.384	0.655	0.296	0.494	1.340	0.508	0.500	1.397	0.512
15311	0.489	1.329	0.467	0.503	1.396	0.508	0.476	1.245	0.512
15312	0.432	0.958	0.296	0.515	1.472	0.491	0.505	1.430	0.481
15313	0.450	1.076	0.185	0.503	1.396	0.512	0.508	1.447	0.328
15314	0.261	−0.137	0.358	0.102	−1.184	0.273	0.184	−0.625	0.501
15315	0.408	0.807	0.409	0.515	1.472	0.371	0.500	1.397	0.512
15316	0.184	−0.625	0.481	0.234	−0.336	0.501	0.142	−0.895	0.508
15317	0.142	−0.895	0.450	0.234	−0.336	0.491	0.187	−0.608	0.513
15318	0.476	1.245	0.450	0.477	1.227	0.478	0.508	1.447	0.467
15319	0.187	−0.608	0.224	0.234	−0.336	0.235	0.184	−0.625	0.481
15320	0.187	−0.608	0.513	0.193	−0.600	0.149	0.184	−0.625	0.097
15321	0.476	1.245	0.513	0.503	1.396	0.514	0.450	1.076	0.142
15322	0.508	1.447	0.513	0.447	1.039	0.273	0.500	1.397	0.492
15323	0.497	1.380	0.358	0.471	1.189	0.501	0.489	1.329	0.142
15324	0.297	0.099	0.262	0.234	−0.336	0.309	0.187	−0.608	0.050
15325	0.145	−0.878	0.185	0.149	−0.882	0.514	0.263	−0.120	0.142
15326	0.142	−0.895	0.185	0.342	0.361	0.491	0.389	0.689	0.467
15327	0.142	−0.895	0.262	0.237	−0.317	0.508	0.361	0.504	0.050
15328	0.495	1.363	0.296	0.509	1.434	0.508	0.447	1.060	0.501
15329	0.489	1.329	0.296	0.506	1.415	0.478	0.479	1.262	0.508
15330	0.476	1.245	0.467	0.491	1.321	0.149	0.479	1.262	0.224
15331	0.466	1.178	0.508	0.512	1.453	0.398	0.503	1.413	0.512
15332	0.142	−0.895	0.224	0.278	−0.054	0.508	0.184	−0.625	0.142
15333	0.142	−0.895	0.224	0.374	0.568	0.491	0.226	−0.356	0.097
15334	0.505	1.430	0.296	0.503	1.396	0.398	0.461	1.144	0.481
15335	0.505	1.430	0.513	0.494	1.340	0.508	0.461	1.144	0.481
15336	0.497	1.380	0.185	0.482	1.265	0.508	0.495	1.363	0.492
15337	0.142	−0.895	0.262	0.240	−0.299	0.444	0.184	−0.625	0.501
15338	0.297	0.099	0.262	0.152	−0.863	0.273	0.224	−0.373	0.097
15339	0.408	0.807	0.450	0.515	1.472	0.422	0.516	1.498	0.508
15340	0.224	−0.373	0.262	0.272	−0.091	0.309	0.411	0.824	0.501
15341	0.500	1.397	0.508	0.500	1.378	0.235	0.497	1.380	0.097
15342	0.263	−0.120	0.262	0.310	0.153	0.341	0.263	−0.120	0.512
15343	0.508	1.447	0.296	0.512	1.453	0.512	0.479	1.262	0.097
15344	0.432	0.958	0.224	0.497	1.359	0.501	0.505	1.430	0.142
15345	0.487	1.312	0.224	0.506	1.415	0.309	0.455	1.110	0.492
15346	0.505	1.430	0.185	0.512	1.453	0.309	0.500	1.397	0.050
15347	0.453	1.093	0.262	0.480	1.246	0.491	0.497	1.380	0.501
15348	0.226	−0.356	0.492	0.149	−0.882	0.149	0.226	−0.356	0.050
15349	0.505	1.430	0.296	0.395	0.700	0.491	0.484	1.295	0.185
15350	0.387	0.672	0.358	0.509	1.434	0.501	0.482	1.279	0.050
15351	0.487	1.312	0.358	0.488	1.302	0.341	0.511	1.464	0.512
15352	0.263	−0.120	0.185	0.398	0.718	0.235	0.050	−1.485	0.508
15353	0.508	1.447	0.262	0.509	1.434	0.309	0.429	0.942	0.508
15354	0.500	1.397	0.492	0.509	1.434	0.508	0.429	0.942	0.481
15355	0.508	1.447	0.262	0.447	1.039	0.193	0.508	1.447	0.050
15356	0.263	−0.120	0.296	0.272	−0.091	0.273	0.295	0.082	0.508
15357	0.466	1.178	0.296	0.421	0.869	0.478	0.503	1.413	0.508
15358	0.229	−0.339	0.296	0.307	0.135	0.193	0.000	?	0.508
15359	0.226	−0.356	0.296	0.237	−0.317	0.309	0.142	−0.895	0.050
15360	0.361	0.504	0.501	0.342	0.361	0.235	0.142	−0.895	0.097
15361	0.447	1.060	0.450	0.509	1.434	0.309	0.500	1.397	0.097
15362	0.500	1.397	0.097	0.500	1.378	0.371	0.468	1.194	0.142
15363	0.382	0.638	0.328	0.313	0.172	0.273	0.224	−0.373	0.097
15364	0.297	0.099	0.097	0.310	0.153	0.102	0.224	−0.373	0.513
15365	0.511	1.464	0.142	0.488	1.302	0.444	0.492	1.346	0.508
15366	0.142	−0.895	0.185	0.149	−0.882	0.462	0.263	−0.120	0.050
15367	0.511	1.464	0.224	0.488	1.302	0.512	0.466	1.178	0.142
15368	0.508	1.447	0.328	0.503	1.396	0.512	0.463	1.161	0.097

15369	0.263	-0.120	0.481	0.342	0.361	0.462	0.261	-0.137	0.512
15370	0.329	0.301	0.142	0.234	-0.336	0.149	0.142	-0.895	0.142
15371	0.142	-0.895	0.450	0.310	0.153	0.491	0.142	-0.895	0.142
15372	0.503	1.413	0.224	0.503	1.396	0.235	0.450	1.076	0.097
15373	0.050	-1.485	0.296	0.193	-0.600	0.501	0.295	0.082	0.508
15374	0.050	-1.485	0.142	0.275	-0.073	0.501	0.326	0.285	0.508
15375	0.447	1.060	0.262	0.480	1.246	0.309	0.497	1.380	0.142
15376	0.224	-0.373	0.224	0.193	-0.600	0.235	0.224	-0.373	0.097
15377	0.261	-0.137	0.467	0.307	0.135	0.235	0.097	-1.181	0.185
15378	0.184	-0.625	0.358	0.234	-0.336	0.501	0.142	-0.895	0.097
15379	0.224	-0.373	0.512	0.307	0.135	0.478	0.142	-0.895	0.097
15380	0.297	0.099	0.512	0.374	0.568	0.444	0.263	-0.120	0.185
15381	0.261	-0.137	0.097	0.275	-0.073	0.341	0.142	-0.895	0.492
15382	0.261	-0.137	0.385	0.102	-1.184	0.341	0.300	0.116	0.050
15383	0.466	1.178	0.450	0.444	1.020	0.273	0.508	1.447	0.142
15384	0.513	1.481	0.097	0.462	1.133	0.235	0.513	1.481	0.142
15385	0.468	1.194	0.512	0.509	1.434	0.462	0.487	1.312	0.142
15386	0.479	1.262	0.512	0.515	1.472	0.235	0.479	1.262	0.097
15387	0.492	1.346	0.512	0.459	1.114	0.341	0.513	1.481	0.501
15388	0.226	-0.356	0.513	0.149	-0.882	0.508	0.187	-0.608	0.050
15389	0.476	1.245	0.467	0.491	1.321	0.273	0.497	1.380	0.097
15390	0.384	0.655	0.508	0.240	-0.299	0.508	0.263	-0.120	0.185
15391	0.508	1.447	0.492	0.503	1.396	0.398	0.489	1.329	0.097
15392	0.511	1.464	0.262	0.503	1.396	0.398	0.450	1.076	0.097
15393	0.000	?	0.450	0.307	0.135	0.273	0.224	-0.373	0.467
15394	0.226	-0.356	0.513	0.234	-0.336	0.371	0.297	0.099	0.512
15395	0.142	-0.895	0.450	0.237	-0.317	0.501	0.384	0.655	0.185
15396	0.450	1.076	0.431	0.500	1.378	0.053	0.511	1.464	0.097
15397	0.408	0.807	0.512	0.482	1.265	0.508	0.450	1.076	0.097
15398	0.505	1.430	0.508	0.444	1.020	0.462	0.503	1.413	0.097
15399	0.142	-0.895	0.508	0.345	0.379	0.193	0.142	-0.895	0.142
15400	0.226	-0.356	0.508	0.339	0.342	0.193	0.295	0.082	0.467
15401	0.329	0.301	0.501	0.275	-0.073	0.462	0.263	-0.120	0.142
15402	0.184	-0.625	0.185	0.237	-0.317	0.462	0.261	-0.137	0.512
15403	0.487	1.312	0.512	0.468	1.170	0.422	0.447	1.060	0.142
15404	0.184	-0.625	0.467	0.234	-0.336	0.514	0.361	0.504	0.097
15405	0.142	-0.895	0.224	0.342	0.361	0.149	0.097	-1.181	0.262
15406	0.358	0.487	0.224	0.509	1.434	0.491	0.505	1.430	0.097
15407	0.261	-0.137	0.492	0.272	-0.091	0.491	0.187	-0.608	0.050
15408	0.513	1.481	0.431	0.503	1.396	0.512	0.387	0.672	0.501
15409	0.463	1.161	0.501	0.398	0.718	0.462	0.511	1.464	0.467
15410	0.463	1.161	0.262	0.398	0.718	0.512	0.511	1.464	0.508
15411	0.097	-1.181	0.508	0.272	-0.091	0.273	0.326	0.285	0.262
15412	0.513	1.481	0.481	0.374	0.568	0.053	0.516	1.498	0.501
15413	0.511	1.464	0.224	0.497	1.359	0.341	0.489	1.329	0.512
15414	0.184	-0.625	0.296	0.053	-1.504	0.512	0.297	0.099	0.385
15415	0.463	1.161	0.492	0.506	1.415	0.341	0.500	1.397	0.385
15416	0.513	1.481	0.481	0.462	1.133	0.341	0.508	1.447	0.481
15417	0.187	-0.608	0.481	0.196	-0.581	0.235	0.329	0.301	0.481
15418	0.145	-0.878	0.224	0.193	-0.600	0.309	0.355	0.470	0.481
15419	0.482	1.279	0.501	0.462	1.133	0.273	0.513	1.481	0.512
15420	0.482	1.279	0.492	0.462	1.133	0.193	0.505	1.430	0.512
15421	0.450	1.076	0.358	0.418	0.850	0.309	0.466	1.178	0.508
15422	0.432	0.958	0.508	0.515	1.472	0.235	0.426	0.925	0.508
15423	0.297	0.099	0.508	0.149	-0.882	0.235	0.224	-0.373	0.142
15424	0.497	1.380	0.492	0.491	1.321	0.193	0.516	1.498	0.492
15425	0.497	1.380	0.185	0.491	1.321	0.478	0.513	1.481	0.467
15426	0.489	1.329	0.185	0.512	1.453	0.235	0.508	1.447	0.508
15427	0.511	1.464	0.142	0.459	1.114	0.371	0.497	1.380	0.508
15428	0.463	1.161	0.224	0.512	1.453	0.478	0.408	0.807	0.501
15429	0.097	-1.181	0.492	0.272	-0.091	0.341	0.295	0.082	0.467

15430	0.408	0.807	0.512	0.053	-1.504	0.341	0.187	-0.608	0.467
15431	0.434	0.975	0.512	0.342	0.361	0.193	0.492	1.346	0.481
15432	0.508	1.447	0.508	0.515	1.472	0.512	0.487	1.312	0.481
15433	0.297	0.099	0.262	0.342	0.361	0.235	0.097	-1.181	0.262
15434	0.297	0.099	0.501	0.342	0.361	0.462	0.097	-1.181	0.508
15435	0.263	-0.120	0.508	0.237	-0.317	0.444	0.097	-1.181	0.501
15436	0.097	-1.181	0.508	0.307	0.135	0.508	0.332	0.318	0.097
15437	0.224	-0.373	0.508	0.278	-0.054	0.514	0.261	-0.137	0.097
15438	0.224	-0.373	0.512	0.193	-0.600	0.512	0.224	-0.373	0.467
15439	0.408	0.807	0.508	0.310	0.153	0.501	0.224	-0.373	0.508
15440	0.097	-1.181	0.501	0.237	-0.317	0.422	0.224	-0.373	0.512
15441	0.261	-0.137	0.512	0.234	-0.336	0.309	0.261	-0.137	0.224
15442	0.145	-0.878	0.501	0.193	-0.600	0.193	0.261	-0.137	0.492
15443	0.508	1.447	0.450	0.477	1.227	0.491	0.511	1.464	0.431
15444	0.142	-0.895	0.501	0.234	-0.336	0.102	0.184	-0.625	0.224
15445	0.329	0.301	0.492	0.374	0.568	0.235	0.261	-0.137	0.185
15446	0.505	1.430	0.501	0.477	1.227	0.273	0.426	0.925	0.492
15447	0.097	-1.181	0.513	0.339	0.342	0.501	0.184	-0.625	0.501
15448	0.511	1.464	0.481	0.342	0.361	0.149	0.503	1.413	0.467
15449	0.505	1.430	0.467	0.193	-0.600	0.512	0.503	1.413	0.224
15450	0.500	1.397	0.508	0.512	1.453	0.478	0.479	1.262	0.481
15451	0.261	-0.137	0.050	0.237	-0.317	0.149	0.332	0.318	0.492
15452	0.508	1.447	0.512	0.459	1.114	0.462	0.497	1.380	0.501
15453	0.508	1.447	0.224	0.442	1.001	0.193	0.508	1.447	0.467
15454	0.479	1.262	0.224	0.509	1.434	0.193	0.505	1.430	0.512
15455	0.426	0.925	0.467	0.515	1.472	0.462	0.511	1.464	0.512
15456	0.432	0.958	0.358	0.509	1.434	0.398	0.463	1.161	0.296
15457	0.447	1.060	0.262	0.500	1.378	0.273	0.482	1.279	0.262
15458	0.050	-1.485	0.512	0.424	0.888	0.235	0.000	?	0.296
15459	0.145	-0.878	0.512	0.310	0.153	0.508	0.097	-1.181	0.262
15460	0.224	-0.373	0.513	0.193	-0.600	0.462	0.145	-0.878	0.513
15461	0.495	1.363	0.501	0.494	1.340	0.235	0.487	1.312	0.513
15462	0.358	0.487	0.512	0.102	-1.184	0.478	0.142	-0.895	0.492
15463	0.297	0.099	0.185	0.234	-0.336	0.273	0.050	-1.485	0.431
15464	0.224	-0.373	0.185	0.272	-0.091	0.462	0.142	-0.895	0.296
15465	0.489	1.329	0.501	0.497	1.359	0.235	0.463	1.161	0.450
15466	0.332	0.318	0.185	0.149	-0.882	0.491	0.050	-1.485	0.409
15467	0.492	1.346	0.142	0.512	1.453	0.149	0.492	1.346	0.409
15468	0.511	1.464	0.262	0.477	1.227	0.149	0.500	1.397	0.409
15469	0.226	-0.356	0.142	0.149	-0.882	0.508	0.226	-0.356	0.501
15470	0.466	1.178	0.097	0.459	1.114	0.193	0.505	1.430	0.431
15471	0.263	-0.120	0.512	0.193	-0.600	0.491	0.142	-0.895	0.501
15472	0.261	-0.137	0.467	0.193	-0.600	0.193	0.097	-1.181	0.513
15473	0.482	1.279	0.481	0.465	1.152	0.235	0.474	1.228	0.513
15474	0.450	1.076	0.501	0.401	0.737	0.102	0.474	1.228	0.409
15475	0.263	-0.120	0.512	0.275	-0.073	0.462	0.000	?	0.512
15476	0.297	0.099	0.481	0.237	-0.317	0.508	0.000	?	0.409
15477	0.387	0.672	0.481	0.503	1.396	0.508	0.487	1.312	0.501
15478	0.479	1.262	0.492	0.462	1.133	0.478	0.495	1.363	0.508
15479	0.224	-0.373	0.097	0.234	-0.336	0.273	0.097	-1.181	0.501
15480	0.447	1.060	0.142	0.480	1.246	0.478	0.500	1.397	0.508
15481	0.295	0.082	0.328	0.272	-0.091	0.478	0.000	?	0.512
15482	0.492	1.346	0.409	0.462	1.133	0.478	0.492	1.346	0.481
15483	0.226	-0.356	0.328	0.234	-0.336	0.235	0.097	-1.181	0.142
15484	0.484	1.295	0.481	0.488	1.302	0.193	0.505	1.430	0.492
15485	0.329	0.301	0.501	0.152	-0.863	0.309	0.097	-1.181	0.224
15486	0.297	0.099	0.467	0.152	-0.863	0.422	0.142	-0.895	0.142
15487	0.511	1.464	0.224	0.506	1.415	0.235	0.484	1.295	0.481
15488	0.300	0.116	0.185	0.193	-0.600	0.235	0.050	-1.485	0.262
15489	0.466	1.178	0.224	0.485	1.283	0.235	0.497	1.380	0.142
15490	0.297	0.099	0.513	0.196	-0.581	0.478	0.050	-1.485	0.467

15491	0.184	-0.625	0.431	0.234	-0.336	0.491	0.187	-0.608	0.467
15492	0.361	0.504	0.450	0.102	-1.184	0.309	0.050	-1.485	0.508
15493	0.408	0.807	0.409	0.462	1.133	0.193	0.503	1.413	0.492
15494	0.482	1.279	0.142	0.506	1.415	0.149	0.500	1.397	0.481
15495	0.453	1.093	0.508	0.506	1.415	0.422	0.500	1.397	0.296
15496	0.450	1.076	0.501	0.477	1.227	0.235	0.474	1.228	0.224
15497	0.226	-0.356	0.492	0.275	-0.073	0.193	0.050	-1.485	0.185
15498	0.511	1.464	0.492	0.482	1.265	0.193	0.497	1.380	0.501
15499	0.511	1.464	0.097	0.482	1.265	0.514	0.497	1.380	0.224
15500	0.511	1.464	0.492	0.482	1.265	0.273	0.497	1.380	0.296
15501	0.358	0.487	0.501	0.234	-0.336	0.478	0.050	-1.485	0.185
15502	0.261	-0.137	0.481	0.193	-0.600	0.273	0.097	-1.181	0.358
15503	0.184	-0.625	0.512	0.307	0.135	0.235	0.097	-1.181	0.385
15504	0.184	-0.625	0.501	0.427	0.907	0.149	0.142	-0.895	0.513
15505	0.263	-0.120	0.467	0.234	-0.336	0.235	0.097	-1.181	0.512
15506	0.297	0.099	0.492	0.237	-0.317	0.273	0.000	?	0.501
15507	0.297	0.099	0.508	0.237	-0.317	0.193	0.000	?	0.501
15508	0.463	1.161	0.508	0.477	1.227	0.478	0.508	1.447	0.409
15509	0.508	1.447	0.450	0.488	1.302	0.235	0.503	1.413	0.512
15510	0.224	-0.373	0.450	0.310	0.153	0.053	0.050	-1.485	0.512
15511	0.224	-0.373	0.508	0.196	-0.581	0.053	0.142	-0.895	0.512
15512	0.297	0.099	0.512	0.149	-0.882	0.273	0.097	-1.181	0.328
15513	0.516	1.498	0.492	0.418	0.850	0.193	0.503	1.413	0.224
15514	0.187	-0.608	0.501	0.237	-0.317	0.462	0.142	-0.895	0.508
15515	0.263	-0.120	0.097	0.193	-0.600	0.235	0.142	-0.895	0.481
15516	0.261	-0.137	0.513	0.193	-0.600	0.149	0.097	-1.181	0.328
15517	0.450	1.076	0.513	0.515	1.472	0.273	0.500	1.397	0.501
15518	0.266	-0.103	0.481	0.272	-0.091	0.273	0.000	?	0.492
15519	0.505	1.430	0.142	0.482	1.265	0.273	0.500	1.397	0.508
15520	0.263	-0.120	0.501	0.275	-0.073	0.512	0.142	-0.895	0.296
15521	0.295	0.082	0.481	0.234	-0.336	0.512	0.097	-1.181	0.296
15522	0.224	-0.373	0.481	0.152	-0.863	0.309	0.184	-0.625	0.296
15523	0.226	-0.356	0.492	0.234	-0.336	0.193	0.097	-1.181	0.508
15524	0.187	-0.608	0.501	0.275	-0.073	0.341	0.097	-1.181	0.508
15525	0.261	-0.137	0.501	0.196	-0.581	0.309	0.184	-0.625	0.492
15526	0.492	1.346	0.185	0.477	1.227	0.309	0.484	1.295	0.409
15527	0.297	0.099	0.185	0.237	-0.317	0.193	0.050	-1.485	0.431
15528	0.355	0.470	0.508	0.053	-1.504	0.193	0.142	-0.895	0.501
15529	0.355	0.470	0.508	0.053	-1.504	0.501	0.142	-0.895	0.467
15530	0.184	-0.625	0.508	0.275	-0.073	0.273	0.142	-0.895	0.467
15531	0.263	-0.120	0.467	0.196	-0.581	0.512	0.097	-1.181	0.508
15532	0.489	1.329	0.467	0.459	1.114	0.193	0.497	1.380	0.142
15533	0.263	-0.120	0.097	0.237	-0.317	0.149	0.050	-1.485	0.450
15534	0.300	0.116	0.501	0.149	-0.882	0.149	0.097	-1.181	0.501
15535	0.295	0.082	0.501	0.275	-0.073	0.193	0.187	-0.608	0.481
15536	0.295	0.082	0.513	0.275	-0.073	0.309	0.097	-1.181	0.513
15537	0.295	0.082	0.328	0.275	-0.073	0.491	0.097	-1.181	0.512
15538	0.500	1.397	0.492	0.512	1.453	0.193	0.461	1.144	0.501
15539	0.447	1.060	0.512	0.512	1.453	0.444	0.505	1.430	0.409
15540	0.097	-1.181	0.224	0.310	0.153	0.491	0.184	-0.625	0.450
15541	0.329	0.301	0.467	0.193	-0.600	0.235	0.000	?	0.450
15542	0.097	-1.181	0.492	0.345	0.379	0.514	0.097	-1.181	0.481
15543	0.145	-0.878	0.501	0.310	0.153	0.501	0.097	-1.181	0.501
15544	0.184	-0.625	0.508	0.307	0.135	0.102	0.097	-1.181	0.501
15545	0.229	-0.339	0.501	0.193	-0.600	0.053	0.142	-0.895	0.481
15546	0.329	0.301	0.501	0.193	-0.600	0.478	0.000	?	0.481
15547	0.484	1.295	0.431	0.503	1.396	0.478	0.463	1.161	0.501
15548	0.142	-0.895	0.185	0.275	-0.073	0.478	0.145	-0.878	0.185
15549	0.450	1.076	0.409	0.509	1.434	0.508	0.505	1.430	0.492
15550	0.224	-0.373	0.508	0.196	-0.581	0.501	0.142	-0.895	0.481
15551	0.295	0.082	0.142	0.149	-0.882	0.462	0.097	-1.181	0.467

15552	0.142	-0.895	0.508	0.149	-0.882	0.501	0.261	-0.137	0.328
15553	0.263	-0.120	0.508	0.196	-0.581	0.193	0.097	-1.181	0.501
15554	0.226	-0.356	0.512	0.310	0.153	0.501	0.050	-1.485	0.467
15555	0.463	1.161	0.512	0.491	1.321	0.491	0.495	1.363	0.481
15556	0.358	0.487	0.513	0.193	-0.600	0.491	0.000	?	0.508
15557	0.508	1.447	0.512	0.444	1.020	0.491	0.471	1.211	0.501
15558	0.511	1.464	0.409	0.485	1.283	0.462	0.511	1.464	0.501
15559	0.097	-1.181	0.512	0.237	-0.317	0.491	0.263	-0.120	0.296
15560	0.382	0.638	0.224	0.506	1.415	0.501	0.500	1.397	0.296
15561	0.447	1.060	0.224	0.497	1.359	0.514	0.508	1.447	0.431
15562	0.000	?	0.358	0.102	-1.184	0.491	0.382	0.638	0.492
15563	0.100	-1.164	0.296	0.053	-1.504	0.149	0.384	0.655	0.513
15564	0.511	1.464	0.262	0.477	1.227	0.508	0.482	1.279	0.501
15565	0.511	1.464	0.097	0.477	1.227	0.508	0.482	1.279	0.512
15566	0.511	1.464	0.501	0.477	1.227	0.309	0.482	1.279	0.262
15567	0.505	1.430	0.224	0.509	1.434	0.371	0.511	1.464	0.508
15568	0.468	1.194	0.450	0.500	1.378	0.514	0.508	1.447	0.512
15569	0.505	1.430	0.512	0.465	1.152	0.501	0.508	1.447	0.512
15570	0.492	1.346	0.224	0.503	1.396	0.491	0.505	1.430	0.224
15571	0.261	-0.137	0.501	0.196	-0.581	0.102	0.142	-0.895	0.512
15572	0.445	1.043	0.262	0.497	1.359	0.491	0.489	1.329	0.492
15573	0.513	1.481	0.481	0.485	1.283	0.501	0.466	1.178	0.224
15574	0.450	1.076	0.513	0.482	1.265	0.102	0.508	1.447	0.492
15575	0.432	0.958	0.508	0.482	1.265	0.341	0.508	1.447	0.508
15576	0.508	1.447	0.431	0.462	1.133	0.478	0.503	1.413	0.450
15577	0.497	1.380	0.185	0.494	1.340	0.508	0.471	1.211	0.508
15578	0.497	1.380	0.512	0.500	1.378	0.501	0.474	1.228	0.512
15579	0.497	1.380	0.296	0.512	1.453	0.102	0.487	1.312	0.508
15580	0.495	1.363	0.097	0.491	1.321	0.512	0.479	1.262	0.508
15581	0.184	-0.625	0.097	0.149	-0.882	0.512	0.263	-0.120	0.142
15582	0.505	1.430	0.508	0.512	1.453	0.478	0.508	1.447	0.513
15583	0.468	1.194	0.142	0.509	1.434	0.508	0.497	1.380	0.450
15584	0.226	-0.356	0.142	0.310	0.153	0.508	0.097	-1.181	0.328
15585	0.226	-0.356	0.501	0.371	0.549	0.501	0.097	-1.181	0.481
15586	0.487	1.312	0.097	0.515	1.472	0.235	0.463	1.161	0.501
15587	0.429	0.942	0.512	0.503	1.396	0.273	0.505	1.430	0.512
15588	0.497	1.380	0.508	0.488	1.302	0.235	0.511	1.464	0.431
15589	0.266	-0.103	0.512	0.102	-1.184	0.102	0.224	-0.373	0.513
15590	0.505	1.430	0.409	0.491	1.321	0.478	0.492	1.346	0.508
15591	0.479	1.262	0.262	0.500	1.378	0.501	0.434	0.975	0.492
15592	0.224	-0.373	0.431	0.102	-1.184	0.491	0.224	-0.373	0.512
15593	0.295	0.082	0.431	0.345	0.379	0.514	0.187	-0.608	0.185
15594	0.489	1.329	0.508	0.480	1.246	0.149	0.484	1.295	0.296
15595	0.474	1.228	0.224	0.506	1.415	0.508	0.500	1.397	0.097
15596	0.476	1.245	0.185	0.500	1.378	0.491	0.468	1.194	0.097
15597	0.226	-0.356	0.328	0.102	-1.184	0.491	0.226	-0.356	0.097
15598	0.495	1.363	0.508	0.515	1.472	0.491	0.476	1.245	0.385
15599	0.495	1.363	0.185	0.512	1.453	0.491	0.495	1.363	0.501
15600	0.358	0.487	0.512	0.482	1.265	0.444	0.505	1.430	0.385
15601	0.508	1.447	0.431	0.509	1.434	0.491	0.471	1.211	0.512
15602	0.503	1.413	0.492	0.509	1.434	0.462	0.508	1.447	0.385
15603	0.487	1.312	0.467	0.500	1.378	0.444	0.508	1.447	0.358
15604	0.184	-0.625	0.508	0.234	-0.336	0.491	0.295	0.082	0.385
15605	0.184	-0.625	0.185	0.272	-0.091	0.444	0.261	-0.137	0.358
15606	0.142	-0.895	0.185	0.234	-0.336	0.514	0.295	0.082	0.508
15607	0.226	-0.356	0.185	0.102	-1.184	0.501	0.263	-0.120	0.450
15608	0.492	1.346	0.492	0.474	1.208	0.444	0.511	1.464	0.508
15609	0.505	1.430	0.512	0.500	1.378	0.491	0.513	1.481	0.512
15610	0.500	1.397	0.508	0.497	1.359	0.512	0.489	1.329	0.185
15611	0.503	1.413	0.501	0.515	1.472	0.508	0.429	0.942	0.492
15612	0.263	-0.120	0.501	0.149	-0.882	0.398	0.297	0.099	0.185

15613	0.500	1.397	0.431	0.509	1.434	0.508	0.447	1.060	0.385
15614	0.508	1.447	0.513	0.488	1.302	0.149	0.405	0.790	0.385
15615	0.508	1.447	0.492	0.488	1.302	0.273	0.405	0.790	0.508
15616	0.508	1.447	0.513	0.488	1.302	0.508	0.405	0.790	0.097
15617	0.508	1.447	0.492	0.491	1.321	0.193	0.500	1.397	0.097
15618	0.000	?	0.512	0.000	?	0.193	0.429	0.942	0.450
15619	0.503	1.413	0.481	0.439	0.982	0.508	0.500	1.397	0.328
15620	0.500	1.397	0.501	0.485	1.283	0.514	0.511	1.464	0.501
15621	0.511	1.464	0.512	0.456	1.095	0.508	0.511	1.464	0.512
15622	0.500	1.397	0.492	0.442	1.001	0.512	0.408	0.807	0.492
15623	0.450	1.076	0.492	0.488	1.302	0.444	0.511	1.464	0.501
15624	0.500	1.397	0.492	0.442	1.001	0.371	0.408	0.807	0.262
15625	0.489	1.329	0.224	0.512	1.453	0.193	0.503	1.413	0.224
15626	0.500	1.397	0.185	0.497	1.359	0.193	0.513	1.481	0.492
15627	0.511	1.464	0.513	0.444	1.020	0.501	0.503	1.413	0.492
15628	0.476	1.245	0.481	0.491	1.321	0.422	0.508	1.447	0.513
15629	0.468	1.194	0.492	0.512	1.453	0.102	0.508	1.447	0.224
15630	0.503	1.413	0.097	0.509	1.434	0.193	0.482	1.279	0.185
15631	0.050	-1.485	0.185	0.401	0.737	0.371	0.142	-0.895	0.296
15632	0.505	1.430	0.409	0.506	1.415	0.478	0.492	1.346	0.467
15633	0.224	-0.373	0.467	0.149	-0.882	0.508	0.224	-0.373	0.296
15634	0.226	-0.356	0.481	0.275	-0.073	0.514	0.142	-0.895	0.467
15635	0.468	1.194	0.296	0.512	1.453	0.491	0.484	1.295	0.097
15636	0.363	0.520	0.492	0.193	-0.600	0.512	0.261	-0.137	0.262
15637	0.263	-0.120	0.050	0.193	-0.600	0.514	0.142	-0.895	0.492
15638	0.505	1.430	0.358	0.506	1.415	0.514	0.466	1.178	0.512
15639	0.505	1.430	0.512	0.512	1.453	0.501	0.466	1.178	0.224
15640	0.511	1.464	0.262	0.503	1.396	0.102	0.513	1.481	0.224
15641	0.492	1.346	0.492	0.515	1.472	0.193	0.492	1.346	0.224
15642	0.500	1.397	0.050	0.450	1.057	0.478	0.476	1.245	0.501
15643	0.000	?	0.358	0.377	0.587	0.462	0.295	0.082	0.409
15644	0.184	-0.625	0.358	0.196	-0.581	0.235	0.224	-0.373	0.492
15645	0.184	-0.625	0.050	0.196	-0.581	0.512	0.184	-0.625	0.492
15646	0.500	1.397	0.467	0.500	1.378	0.508	0.497	1.380	0.492
15647	0.184	-0.625	0.501	0.424	0.888	0.491	0.226	-0.356	0.501
15648	0.145	-0.878	0.512	0.102	-1.184	0.309	0.297	0.099	0.501
15649	0.263	-0.120	0.142	0.196	-0.581	0.273	0.184	-0.625	0.508
15650	0.142	-0.895	0.513	0.371	0.549	0.309	0.358	0.487	0.467
15651	0.097	-1.181	0.142	0.000	?	0.462	0.384	0.655	0.492
15652	0.505	1.430	0.467	0.477	1.227	0.491	0.511	1.464	0.513
15653	0.468	1.194	0.467	0.512	1.453	0.491	0.505	1.430	0.467
15654	0.482	1.279	0.431	0.509	1.434	0.501	0.495	1.363	0.501
15655	0.492	1.346	0.224	0.491	1.321	0.491	0.500	1.397	0.512
15656	0.513	1.481	0.385	0.515	1.472	0.478	0.413	0.841	0.492
15657	0.479	1.262	0.481	0.512	1.453	0.478	0.508	1.447	0.501
15658	0.479	1.262	0.262	0.512	1.453	0.508	0.508	1.447	0.481
15659	0.495	1.363	0.492	0.500	1.378	0.371	0.511	1.464	0.097
15660	0.097	-1.181	0.512	0.102	-1.184	0.508	0.326	0.285	0.224
15661	0.142	-0.895	0.512	0.193	-0.600	0.512	0.224	-0.373	0.492
15662	0.326	0.285	0.467	0.480	1.246	0.501	0.513	1.481	0.467
15663	0.405	0.790	0.142	0.465	1.152	0.491	0.479	1.262	0.467
15664	0.329	0.301	0.142	0.237	-0.317	0.491	0.332	0.318	0.142
15665	0.474	1.228	0.513	0.520	1.509	0.512	0.500	1.397	0.224
15666	0.503	1.413	0.513	0.503	1.396	0.508	0.495	1.363	0.467
15667	0.466	1.178	0.097	0.488	1.302	0.514	0.511	1.464	0.501
15668	0.224	-0.373	0.492	0.307	0.135	0.514	0.295	0.082	0.508
15669	0.184	-0.625	0.296	0.272	-0.091	0.478	0.295	0.082	0.358
15670	0.224	-0.373	0.262	0.307	0.135	0.501	0.295	0.082	0.508
15671	0.511	1.464	0.450	0.459	1.114	0.501	0.503	1.413	0.262
15672	0.429	0.942	0.512	0.491	1.321	0.508	0.505	1.430	0.467
15673	0.447	1.060	0.142	0.491	1.321	0.508	0.489	1.329	0.501

15674	0.411	0.824	0.185	0.500	1.378	0.508	0.411	0.824	0.185
15675	0.142	-0.895	0.185	0.000	?	0.273	0.432	0.958	0.513
15676	0.505	1.430	0.097	0.494	1.340	0.508	0.505	1.430	0.185
15677	0.497	1.380	0.501	0.480	1.246	0.501	0.471	1.211	0.328
15678	0.489	1.329	0.512	0.480	1.246	0.508	0.471	1.211	0.512
15679	0.484	1.295	0.142	0.515	1.472	0.053	0.505	1.430	0.224
15680	0.097	-1.181	0.467	0.368	0.530	0.501	0.142	-0.895	0.501
15681	0.492	1.346	0.492	0.509	1.434	0.508	0.445	1.043	0.467
15682	0.492	1.346	0.501	0.520	1.509	0.501	0.503	1.413	0.512
15683	0.476	1.245	0.097	0.497	1.359	0.508	0.479	1.262	0.262
15684	0.511	1.464	0.097	0.494	1.340	0.514	0.508	1.447	0.508
15685	0.492	1.346	0.097	0.491	1.321	0.514	0.516	1.498	0.097
15686	0.468	1.194	0.508	0.509	1.434	0.149	0.500	1.397	0.409
15687	0.492	1.346	0.508	0.509	1.434	0.149	0.408	0.807	0.385
15688	0.500	1.397	0.501	0.512	1.453	0.462	0.450	1.076	0.508
15689	0.500	1.397	0.492	0.512	1.453	0.478	0.450	1.076	0.050
15690	0.453	1.093	0.224	0.480	1.246	0.508	0.482	1.279	0.050
15691	0.453	1.093	0.501	0.503	1.396	0.508	0.505	1.430	0.508
15692	0.511	1.464	0.492	0.500	1.378	0.512	0.495	1.363	0.097
15693	0.505	1.430	0.512	0.512	1.453	0.235	0.479	1.262	0.501
15694	0.492	1.346	0.513	0.506	1.415	0.508	0.479	1.262	0.481
15695	0.495	1.363	0.481	0.506	1.415	0.514	0.500	1.397	0.481
15696	0.097	-1.181	0.512	0.272	-0.091	0.508	0.184	-0.625	0.508
15697	0.503	1.413	0.050	0.512	1.453	0.053	0.492	1.346	0.185
15698	0.503	1.413	0.185	0.506	1.415	0.508	0.479	1.262	0.185
15699	0.476	1.245	0.224	0.503	1.396	0.462	0.466	1.178	0.431
15700	0.142	-0.895	0.097	0.053	-1.504	0.149	0.329	0.301	0.450
15701	0.503	1.413	0.501	0.500	1.378	0.501	0.503	1.413	0.185
15702	0.474	1.228	0.501	0.509	1.434	0.501	0.468	1.194	0.431
15703	0.474	1.228	0.501	0.500	1.378	0.491	0.484	1.295	0.328
15704	0.497	1.380	0.224	0.509	1.434	0.491	0.505	1.430	0.328
15705	0.495	1.363	0.296	0.512	1.453	0.514	0.503	1.413	0.512
15706	0.495	1.363	0.262	0.512	1.453	0.512	0.503	1.413	0.492
15707	0.184	-0.625	0.513	0.149	-0.882	0.462	0.295	0.082	0.142
15708	0.184	-0.625	0.296	0.149	-0.882	0.273	0.295	0.082	0.328
15709	0.508	1.447	0.492	0.465	1.152	0.512	0.429	0.942	0.328
15710	0.508	1.447	0.328	0.480	1.246	0.491	0.489	1.329	0.224
15711	0.505	1.430	0.508	0.512	1.453	0.149	0.511	1.464	0.481
15712	0.466	1.178	0.097	0.506	1.415	0.512	0.495	1.363	0.450
15713	0.463	1.161	0.512	0.518	1.491	0.501	0.516	1.498	0.224
15714	0.097	-1.181	0.512	0.234	-0.336	0.501	0.263	-0.120	0.512
15715	0.495	1.363	0.450	0.509	1.434	0.371	0.513	1.481	0.513
15716	0.500	1.397	0.385	0.518	1.491	0.478	0.505	1.430	0.492
15717	0.511	1.464	0.501	0.512	1.453	0.398	0.508	1.447	0.358
15718	0.329	0.301	0.409	0.053	-1.504	0.508	0.224	-0.373	0.296
15719	0.492	1.346	0.481	0.506	1.415	0.491	0.511	1.464	0.328
15720	0.508	1.447	0.142	0.465	1.152	0.149	0.489	1.329	0.385
15721	0.224	-0.373	0.508	0.149	-0.882	0.193	0.226	-0.356	0.508
15722	0.466	1.178	0.481	0.500	1.378	0.149	0.484	1.295	0.409
15723	0.492	1.346	0.467	0.503	1.396	0.371	0.513	1.481	0.513
15724	0.497	1.380	0.467	0.494	1.340	0.341	0.450	1.076	0.185
15725	0.503	1.413	0.492	0.491	1.321	0.309	0.503	1.413	0.224
15726	0.495	1.363	0.492	0.515	1.472	0.514	0.508	1.447	0.512
15727	0.505	1.430	0.050	0.520	1.509	0.341	0.503	1.413	0.512
15728	0.432	0.958	0.467	0.465	1.152	0.508	0.500	1.397	0.481
15729	0.187	-0.608	0.450	0.272	-0.091	0.478	0.142	-0.895	0.450
15730	0.408	0.807	0.508	0.515	1.472	0.309	0.511	1.464	0.513
15731	0.508	1.447	0.508	0.491	1.321	0.422	0.445	1.043	0.481
15732	0.142	-0.895	0.508	0.149	-0.882	0.309	0.329	0.301	0.142
15733	0.497	1.380	0.512	0.509	1.434	0.514	0.489	1.329	0.328
15734	0.505	1.430	0.512	0.506	1.415	0.514	0.500	1.397	0.262

15735	0.508	1.447	0.512	0.506	1.415	0.501	0.508	1.447	0.224
15736	0.511	1.464	0.512	0.374	0.568	0.512	0.426	0.925	0.385
15737	0.511	1.464	0.450	0.471	1.189	0.273	0.516	1.498	0.508
15738	0.503	1.413	0.097	0.401	0.737	0.508	0.511	1.464	0.467
15739	0.408	0.807	0.512	0.506	1.415	0.309	0.497	1.380	0.185
15740	0.516	1.498	0.512	0.494	1.340	0.309	0.511	1.464	0.224
15741	0.224	-0.373	0.512	0.149	-0.882	0.235	0.184	-0.625	0.262
15742	0.226	-0.356	0.409	0.193	-0.600	0.508	0.297	0.099	0.450
15743	0.358	0.487	0.185	0.152	-0.863	0.309	0.097	-1.181	0.262
15744	0.295	0.082	0.467	0.371	0.549	0.309	0.097	-1.181	0.481
15745	0.261	-0.137	0.512	0.342	0.361	0.514	0.097	-1.181	0.050
15746	0.097	-1.181	0.492	0.310	0.153	0.102	0.384	0.655	0.492
15747	0.497	1.380	0.224	0.512	1.453	0.512	0.503	1.413	0.097
15748	0.224	-0.373	0.467	0.342	0.361	0.508	0.387	0.672	0.050
15749	0.447	1.060	0.501	0.512	1.453	0.491	0.513	1.481	0.450
15750	0.513	1.481	0.501	0.482	1.265	0.508	0.384	0.655	0.467
15751	0.224	-0.373	0.508	0.310	0.153	0.273	0.358	0.487	0.508
15752	0.500	1.397	0.513	0.430	0.926	0.341	0.384	0.655	0.467
15753	0.261	-0.137	0.185	0.310	0.153	0.512	0.358	0.487	0.467
15754	0.482	1.279	0.185	0.509	1.434	0.512	0.503	1.413	0.508
15755	0.508	1.447	0.185	0.512	1.453	0.462	0.453	1.093	0.508
15756	0.503	1.413	0.512	0.497	1.359	0.371	0.508	1.447	0.262
15757	0.432	0.958	0.513	0.503	1.396	0.341	0.511	1.464	0.512
15758	0.184	-0.625	0.508	0.275	-0.073	0.193	0.184	-0.625	0.512
15759	0.513	1.481	0.512	0.512	1.453	0.444	0.492	1.346	0.512
15760	0.295	0.082	0.513	0.310	0.153	0.193	0.187	-0.608	0.512
15761	0.097	-1.181	0.097	0.310	0.153	0.508	0.384	0.655	0.492
15762	0.097	-1.181	0.097	0.237	-0.317	0.444	0.384	0.655	0.492
15763	0.505	1.430	0.481	0.509	1.434	0.508	0.503	1.413	0.142
15764	0.142	-0.895	0.450	0.313	0.172	0.478	0.097	-1.181	0.492
15765	0.142	-0.895	0.481	0.313	0.172	0.501	0.097	-1.181	0.508
15766	0.495	1.363	0.481	0.512	1.453	0.235	0.453	1.093	0.512
15767	0.097	-1.181	0.185	0.102	-1.184	0.273	0.332	0.318	0.492
15768	0.511	1.464	0.512	0.506	1.415	0.235	0.500	1.397	0.492
15769	0.505	1.430	0.097	0.509	1.434	0.508	0.505	1.430	0.512
15770	0.508	1.447	0.142	0.491	1.321	0.444	0.487	1.312	0.501
15771	0.411	0.824	0.512	0.509	1.434	0.491	0.503	1.413	0.508
15772	0.000	?	0.467	0.275	-0.073	0.501	0.263	-0.120	0.508
15773	0.261	-0.137	0.492	0.342	0.361	0.501	0.226	-0.356	0.512
15774	0.429	0.942	0.185	0.512	1.453	0.512	0.492	1.346	0.185
15775	0.429	0.942	0.142	0.512	1.453	0.508	0.492	1.346	0.508
15776	0.497	1.380	0.142	0.462	1.133	0.491	0.518	1.514	0.431
15777	0.224	-0.373	0.481	0.371	0.549	0.508	0.226	-0.356	0.431
15778	0.184	-0.625	0.467	0.342	0.361	0.512	0.184	-0.625	0.513
15779	0.326	0.285	0.501	0.193	-0.600	0.491	0.297	0.099	0.358
15780	0.508	1.447	0.501	0.442	1.001	0.508	0.466	1.178	0.467
15781	0.187	-0.608	0.508	0.193	-0.600	0.508	0.295	0.082	0.409
15782	0.505	1.430	0.508	0.503	1.396	0.508	0.474	1.228	0.508
15783	0.429	0.942	0.513	0.450	1.057	0.512	0.097	-1.181	0.185
15784	0.489	1.329	0.513	0.512	1.453	0.512	0.261	-0.137	0.508
15785	0.468	1.194	0.224	0.477	1.227	0.512	0.487	1.312	0.492
15786	0.508	1.447	0.501	0.497	1.359	0.273	0.511	1.464	0.508
15787	0.187	-0.608	0.450	0.234	-0.336	0.309	0.224	-0.373	0.508
15788	0.187	-0.608	0.142	0.272	-0.091	0.512	0.224	-0.373	0.481
15789	0.187	-0.608	0.481	0.234	-0.336	0.514	0.224	-0.373	0.224
15790	0.492	1.346	0.142	0.506	1.415	0.512	0.500	1.397	0.224
15791	0.511	1.464	0.097	0.444	1.020	0.309	0.408	0.807	0.224
15792	0.500	1.397	0.467	0.488	1.302	0.371	0.495	1.363	0.512
15793	0.492	1.346	0.481	0.497	1.359	0.462	0.495	1.363	0.508
15794	0.492	1.346	0.050	0.497	1.359	0.512	0.495	1.363	0.508
15795	0.429	0.942	0.512	0.518	1.491	0.512	0.503	1.413	0.467

15796	0.500	1.397	0.492	0.515	1.472	0.193	0.505	1.430	0.508
15797	0.492	1.346	0.097	0.494	1.340	0.444	0.503	1.413	0.262
15798	0.513	1.481	0.296	0.500	1.378	0.235	0.463	1.161	0.224
15799	0.484	1.295	0.409	0.515	1.472	0.501	0.489	1.329	0.512
15800	0.503	1.413	0.328	0.491	1.321	0.462	0.513	1.481	0.508
15801	0.484	1.295	0.224	0.509	1.434	0.235	0.468	1.194	0.492
15802	0.500	1.397	0.512	0.512	1.453	0.501	0.503	1.413	0.501
15803	0.511	1.464	0.450	0.509	1.434	0.422	0.508	1.447	0.097
15804	0.489	1.329	0.467	0.512	1.453	0.273	0.487	1.312	0.508
15805	0.489	1.329	0.501	0.512	1.453	0.514	0.497	1.380	0.142
15806	0.489	1.329	0.142	0.512	1.453	0.309	0.476	1.245	0.097
15807	0.224	-0.373	0.492	0.272	-0.091	0.512	0.097	-1.181	0.492
15808	0.184	-0.625	0.431	0.313	0.172	0.478	0.226	-0.356	0.512
15809	0.505	1.430	0.431	0.512	1.453	0.422	0.492	1.346	0.492
15810	0.474	1.228	0.097	0.515	1.472	0.193	0.466	1.178	0.296
15811	0.484	1.295	0.513	0.515	1.472	0.478	0.466	1.178	0.296
15812	0.097	-1.181	0.050	0.313	0.172	0.462	0.142	-0.895	0.224
15813	0.184	-0.625	0.328	0.374	0.568	0.501	0.226	-0.356	0.501
15814	0.411	0.824	0.501	0.465	1.152	0.501	0.468	1.194	0.501
15815	0.471	1.211	0.513	0.515	1.472	0.422	0.503	1.413	0.481
15816	0.479	1.262	0.097	0.509	1.434	0.309	0.503	1.413	0.481
15817	0.297	0.099	0.050	0.193	-0.600	0.273	0.355	0.470	0.512
15818	0.487	1.312	0.512	0.442	1.001	0.514	0.511	1.464	0.501
15819	0.050	-1.485	0.512	0.234	-0.336	0.273	0.261	-0.137	0.450
15820	0.355	0.470	0.512	0.500	1.378	0.512	0.466	1.178	0.450
15821	0.500	1.397	0.450	0.465	1.152	0.514	0.503	1.413	0.296
15822	0.266	-0.103	0.467	0.234	-0.336	0.512	0.184	-0.625	0.481
15823	0.489	1.329	0.492	0.500	1.378	0.508	0.511	1.464	0.513
15824	0.050	-1.485	0.262	0.424	0.888	0.235	0.184	-0.625	0.185
15825	0.358	0.487	0.467	0.278	-0.054	0.235	0.329	0.301	0.512
15826	0.355	0.470	0.513	0.515	1.472	0.478	0.516	1.498	0.296
15827	0.050	-1.485	0.512	0.307	0.135	0.512	0.229	-0.339	0.224
15828	0.463	1.161	0.450	0.518	1.491	0.341	0.497	1.380	0.508
15829	0.503	1.413	0.450	0.474	1.208	0.491	0.468	1.194	0.512
15830	0.511	1.464	0.467	0.427	0.907	0.273	0.511	1.464	0.097
15831	0.142	-0.895	0.467	0.193	-0.600	0.273	0.263	-0.120	0.512
15832	0.516	1.498	0.296	0.482	1.265	0.508	0.505	1.430	0.431
15833	0.142	-0.895	0.262	0.462	1.133	0.478	0.097	-1.181	0.097
15834	0.468	1.194	0.224	0.503	1.396	0.309	0.408	0.807	0.431
15835	0.466	1.178	0.467	0.503	1.396	0.398	0.382	0.638	0.501
15836	0.429	0.942	0.512	0.424	0.888	0.371	0.508	1.447	0.097
15837	0.224	-0.373	0.481	0.307	0.135	0.273	0.050	-1.485	0.358
15838	0.384	0.655	0.450	0.272	-0.091	0.491	0.050	-1.485	0.296
15839	0.476	1.245	0.501	0.515	1.472	0.501	0.503	1.413	0.185
15840	0.263	-0.120	0.328	0.275	-0.073	0.193	0.097	-1.181	0.512
15841	0.492	1.346	0.262	0.512	1.453	0.491	0.497	1.380	0.431
15842	0.513	1.481	0.492	0.520	1.509	0.398	0.479	1.262	0.185
15843	0.511	1.464	0.512	0.506	1.415	0.514	0.479	1.262	0.431
15844	0.466	1.178	0.492	0.503	1.396	0.193	0.505	1.430	0.501
15845	0.142	-0.895	0.050	0.237	-0.317	0.193	0.187	-0.608	0.501
15846	0.142	-0.895	0.262	0.237	-0.317	0.149	0.187	-0.608	0.224
15847	0.516	1.498	0.431	0.477	1.227	0.501	0.434	0.975	0.501
15848	0.503	1.413	0.262	0.518	1.491	0.444	0.450	1.076	0.328
15849	0.097	-1.181	0.481	0.339	0.342	0.508	0.184	-0.625	0.296
15850	0.492	1.346	0.501	0.491	1.321	0.512	0.434	0.975	0.328
15851	0.300	0.116	0.513	0.275	-0.073	0.398	0.326	0.285	0.512
15852	0.263	-0.120	0.512	0.275	-0.073	0.273	0.326	0.285	0.512
15853	0.447	1.060	0.224	0.503	1.396	0.508	0.511	1.464	0.142
15854	0.511	1.464	0.481	0.480	1.246	0.514	0.495	1.363	0.262
15855	0.142	-0.895	0.467	0.307	0.135	0.491	0.142	-0.895	0.492
15856	0.184	-0.625	0.385	0.401	0.737	0.508	0.326	0.285	0.492

15857	0.184	-0.625	0.492	0.374	0.568	0.512	0.326	0.285	0.492
15858	0.097	-1.181	0.508	0.275	-0.073	0.514	0.224	-0.373	0.501
15859	0.503	1.413	0.050	0.485	1.283	0.371	0.482	1.279	0.501
15860	0.503	1.413	0.224	0.500	1.378	0.193	0.450	1.076	0.512
15861	0.142	-0.895	0.512	0.196	-0.581	0.149	0.226	-0.356	0.097
15862	0.468	1.194	0.513	0.488	1.302	0.273	0.508	1.447	0.467
15863	0.487	1.312	0.431	0.398	0.718	0.501	0.511	1.464	0.467
15864	0.497	1.380	0.512	0.518	1.491	0.462	0.487	1.312	0.467
15865	0.097	-1.181	0.492	0.193	-0.600	0.478	0.358	0.487	0.501
15866	0.097	-1.181	0.492	0.193	-0.600	0.149	0.295	0.082	0.492
15867	0.097	-1.181	0.328	0.149	-0.882	0.273	0.329	0.301	0.501
15868	0.500	1.397	0.296	0.497	1.359	0.235	0.384	0.655	0.501
15869	0.500	1.397	0.450	0.447	1.039	0.501	0.508	1.447	0.224
15870	0.495	1.363	0.467	0.506	1.415	0.193	0.411	0.824	0.224
15871	0.487	1.312	0.501	0.515	1.472	0.491	0.513	1.481	0.262
15872	0.000	?	0.508	0.401	0.737	0.193	0.184	-0.625	0.450
15873	0.224	-0.373	0.296	0.275	-0.073	0.491	0.226	-0.356	0.501
15874	0.500	1.397	0.513	0.506	1.415	0.341	0.511	1.464	0.512
15875	0.489	1.329	0.513	0.515	1.472	0.422	0.508	1.447	0.385
15876	0.508	1.447	0.508	0.494	1.340	0.508	0.479	1.262	0.385
15877	0.508	1.447	0.185	0.509	1.434	0.478	0.450	1.076	0.501
15878	0.474	1.228	0.508	0.509	1.434	0.508	0.513	1.481	0.467
15879	0.500	1.397	0.513	0.512	1.453	0.491	0.484	1.295	0.328
15880	0.050	-1.485	0.481	0.374	0.568	0.491	0.142	-0.895	0.328
15881	0.187	-0.608	0.481	0.196	-0.581	0.514	0.329	0.301	0.409
15882	0.226	-0.356	0.481	0.149	-0.882	0.512	0.263	-0.120	0.512
15883	0.097	-1.181	0.481	0.275	-0.073	0.273	0.226	-0.356	0.409
15884	0.500	1.397	0.508	0.503	1.396	0.512	0.384	0.655	0.358
15885	0.495	1.363	0.501	0.462	1.133	0.508	0.505	1.430	0.262
15886	0.500	1.397	0.224	0.471	1.189	0.508	0.466	1.178	0.512
15887	0.226	-0.356	0.262	0.149	-0.882	0.508	0.184	-0.625	0.358
15888	0.300	0.116	0.409	0.272	-0.091	0.501	0.226	-0.356	0.508
15889	0.266	-0.103	0.296	0.234	-0.336	0.501	0.263	-0.120	0.512
15890	0.508	1.447	0.185	0.503	1.396	0.341	0.447	1.060	0.492
15891	0.297	0.099	0.467	0.199	-0.562	0.491	0.261	-0.137	0.481
15892	0.487	1.312	0.296	0.485	1.283	0.462	0.482	1.279	0.296
15893	0.326	0.285	0.185	0.193	-0.600	0.491	0.050	-1.485	0.481
15894	0.508	1.447	0.501	0.494	1.340	0.491	0.495	1.363	0.513
15895	0.097	-1.181	0.262	0.339	0.342	0.514	0.097	-1.181	0.501
15896	0.000	?	0.501	0.418	0.850	0.491	0.050	-1.485	0.512
15897	0.500	1.397	0.501	0.515	1.472	0.491	0.447	1.060	0.431
15898	0.500	1.397	0.450	0.482	1.265	0.491	0.471	1.211	0.431
15899	0.453	1.093	0.097	0.509	1.434	0.478	0.503	1.413	0.262
15900	0.384	0.655	0.512	0.491	1.321	0.501	0.463	1.161	0.481
15901	0.497	1.380	0.508	0.494	1.340	0.273	0.463	1.161	0.467
15902	0.405	0.790	0.450	0.515	1.472	0.501	0.513	1.481	0.513
15903	0.482	1.279	0.501	0.518	1.491	0.512	0.508	1.447	0.409
15904	0.142	-0.895	0.224	0.272	-0.091	0.512	0.263	-0.120	0.508
15905	0.505	1.430	0.450	0.506	1.415	0.478	0.516	1.498	0.467
15906	0.474	1.228	0.262	0.512	1.453	0.149	0.505	1.430	0.185
15907	0.466	1.178	0.262	0.512	1.453	0.512	0.511	1.464	0.409
15908	0.466	1.178	0.512	0.512	1.453	0.491	0.511	1.464	0.450
15909	0.492	1.346	0.481	0.494	1.340	0.512	0.489	1.329	0.296
15910	0.492	1.346	0.296	0.494	1.340	0.149	0.489	1.329	0.512
15911	0.050	-1.485	0.296	0.342	0.361	0.514	0.142	-0.895	0.513
15912	0.463	1.161	0.512	0.488	1.302	0.508	0.489	1.329	0.501
15913	0.447	1.060	0.050	0.459	1.114	0.508	0.503	1.413	0.358
15914	0.505	1.430	0.050	0.485	1.283	0.514	0.511	1.464	0.501
15915	0.505	1.430	0.050	0.485	1.283	0.512	0.489	1.329	0.481
15916	0.503	1.413	0.501	0.515	1.472	0.462	0.489	1.329	0.450
15917	0.511	1.464	0.512	0.488	1.302	0.462	0.508	1.447	0.262

15918	0.511	1.464	0.409	0.485	1.283	0.462	0.503	1.413	0.492
15919	0.511	1.464	0.224	0.485	1.283	0.491	0.511	1.464	0.431
15920	0.511	1.464	0.262	0.474	1.208	0.512	0.511	1.464	0.508
15921	0.447	1.060	0.467	0.494	1.340	0.508	0.511	1.464	0.508
15922	0.097	-1.181	0.050	0.278	-0.054	0.444	0.184	-0.625	0.508
15923	0.508	1.447	0.142	0.503	1.396	0.508	0.508	1.447	0.481
15924	0.516	1.498	0.328	0.512	1.453	0.309	0.432	0.958	0.508
15925	0.516	1.498	0.224	0.512	1.453	0.235	0.432	0.958	0.492
15926	0.408	0.807	0.185	0.477	1.227	0.512	0.516	1.498	0.385
15927	0.184	-0.625	0.501	0.149	-0.882	0.501	0.361	0.504	0.224
15928	0.463	1.161	0.492	0.518	1.491	0.514	0.461	1.144	0.492
15929	0.513	1.481	0.508	0.491	1.321	0.422	0.411	0.824	0.097
15930	0.495	1.363	0.185	0.506	1.415	0.273	0.505	1.430	0.142
15931	0.224	-0.373	0.185	0.152	-0.863	0.501	0.184	-0.625	0.492
15932	0.463	1.161	0.481	0.512	1.453	0.309	0.503	1.413	0.185
15933	0.503	1.413	0.467	0.506	1.415	0.341	0.489	1.329	0.185
15934	0.500	1.397	0.481	0.506	1.415	0.501	0.503	1.413	0.512
15935	0.497	1.380	0.501	0.518	1.491	0.444	0.505	1.430	0.142
15936	0.511	1.464	0.296	0.506	1.415	0.508	0.479	1.262	0.513
15937	0.184	-0.625	0.492	0.468	1.170	0.273	0.224	-0.373	0.513
15938	0.184	-0.625	0.467	0.468	1.170	0.149	0.224	-0.373	0.512
15939	0.184	-0.625	0.508	0.468	1.170	0.193	0.224	-0.373	0.224
15940	0.500	1.397	0.508	0.491	1.321	0.491	0.521	1.531	0.467
15941	0.500	1.397	0.097	0.515	1.472	0.501	0.513	1.481	0.501
15942	0.511	1.464	0.050	0.506	1.415	0.512	0.511	1.464	0.450
15943	0.513	1.481	0.142	0.439	0.982	0.512	0.468	1.194	0.431
15944	0.516	1.498	0.409	0.506	1.415	0.501	0.511	1.464	0.185
15945	0.097	-1.181	0.296	0.313	0.172	0.508	0.261	-0.137	0.512
15946	0.097	-1.181	0.142	0.237	-0.317	0.501	0.226	-0.356	0.262
15947	0.482	1.279	0.142	0.515	1.472	0.501	0.511	1.464	0.262
15948	0.450	1.076	0.512	0.503	1.396	0.398	0.503	1.413	0.481
15949	0.479	1.262	0.492	0.512	1.453	0.512	0.492	1.346	0.467
15950	0.479	1.262	0.097	0.421	0.869	0.491	0.503	1.413	0.224
15951	0.184	-0.625	0.328	0.275	-0.073	0.273	0.097	-1.181	0.185
15952	0.511	1.464	0.328	0.494	1.340	0.478	0.508	1.447	0.481
15953	0.100	-1.164	0.328	0.310	0.153	0.273	0.142	-0.895	0.224
15954	0.142	-0.895	0.224	0.345	0.379	0.235	0.097	-1.181	0.224
15955	0.511	1.464	0.501	0.503	1.396	0.478	0.497	1.380	0.224
15956	0.466	1.178	0.224	0.447	1.039	0.514	0.513	1.481	0.508
15957	0.495	1.363	0.142	0.509	1.434	0.371	0.487	1.312	0.481
15958	0.184	-0.625	0.467	0.275	-0.073	0.478	0.300	0.116	0.142
15959	0.145	-0.878	0.501	0.149	-0.882	0.462	0.297	0.099	0.328
15960	0.145	-0.878	0.097	0.196	-0.581	0.341	0.224	-0.373	0.142
15961	0.484	1.295	0.142	0.491	1.321	0.102	0.503	1.413	0.512
15962	0.466	1.178	0.185	0.494	1.340	0.444	0.503	1.413	0.296
15963	0.505	1.430	0.481	0.515	1.472	0.235	0.479	1.262	0.224
15964	0.505	1.430	0.467	0.515	1.472	0.149	0.479	1.262	0.296
15965	0.508	1.447	0.185	0.503	1.396	0.478	0.508	1.447	0.142
15966	0.508	1.447	0.224	0.506	1.415	0.273	0.497	1.380	0.185
15967	0.511	1.464	0.224	0.500	1.378	0.512	0.450	1.076	0.467
15968	0.511	1.464	0.185	0.500	1.378	0.512	0.450	1.076	0.481
15969	0.224	-0.373	0.492	0.404	0.756	0.235	0.297	0.099	0.481
15970	0.500	1.397	0.185	0.509	1.434	0.512	0.479	1.262	0.328
15971	0.450	1.076	0.224	0.494	1.340	0.478	0.513	1.481	0.185
15972	0.142	-0.895	0.467	0.278	-0.054	0.478	0.184	-0.625	0.385
15973	0.476	1.245	0.431	0.477	1.227	0.235	0.518	1.514	0.409
15974	0.142	-0.895	0.512	0.278	-0.054	0.478	0.297	0.099	0.481
15975	0.097	-1.181	0.450	0.234	-0.336	0.341	0.229	-0.339	0.409
15976	0.468	1.194	0.142	0.480	1.246	0.235	0.508	1.447	0.050
15977	0.479	1.262	0.142	0.512	1.453	0.053	0.505	1.430	0.508
15978	0.050	-1.485	0.050	0.374	0.568	0.491	0.097	-1.181	0.409

15979	0.508	1.447	0.492	0.474	1.208	0.462	0.511	1.464	0.512
15980	0.492	1.346	0.262	0.462	1.133	0.309	0.432	0.958	0.467
15981	0.097	-1.181	0.492	0.339	0.342	0.422	0.097	-1.181	0.050
15982	0.295	0.082	0.508	0.102	-1.184	0.491	0.434	0.975	0.185
15983	0.405	0.790	0.501	0.447	1.039	0.508	0.503	1.413	0.185
15984	0.326	0.285	0.508	0.234	-0.336	0.501	0.097	-1.181	0.492
15985	0.224	-0.373	0.328	0.149	-0.882	0.512	0.361	0.504	0.328
15986	0.513	1.481	0.262	0.482	1.265	0.514	0.297	0.099	0.142
15987	0.453	1.093	0.508	0.275	-0.073	0.462	0.184	-0.625	0.328
15988	0.466	1.178	0.512	0.515	1.472	0.371	0.508	1.447	0.508
15989	0.503	1.413	0.508	0.512	1.453	0.501	0.429	0.942	0.512
15990	0.142	-0.895	0.501	0.237	-0.317	0.501	0.187	-0.608	0.328
15991	0.492	1.346	0.512	0.512	1.453	0.501	0.429	0.942	0.142
15992	0.432	0.958	0.513	0.480	1.246	0.512	0.503	1.413	0.142
15993	0.432	0.958	0.450	0.480	1.246	0.501	0.503	1.413	0.185
15994	0.100	-1.164	0.296	0.234	-0.336	0.514	0.224	-0.373	0.296
15995	0.500	1.397	0.508	0.480	1.246	0.514	0.503	1.413	0.385
15996	0.050	-1.485	0.481	0.342	0.361	0.193	0.332	0.318	0.224
15997	0.000	?	0.050	0.237	-0.317	0.102	0.295	0.082	0.296
15998	0.329	0.301	0.508	0.053	-1.504	0.193	0.329	0.301	0.492
15999	0.500	1.397	0.224	0.491	1.321	0.501	0.511	1.464	0.431
16000	0.516	1.498	0.185	0.459	1.114	0.512	0.511	1.464	0.185
16001	0.097	-1.181	0.097	0.310	0.153	0.514	0.142	-0.895	0.262
16002	0.050	-1.485	0.097	0.427	0.907	0.102	0.261	-0.137	0.185
16003	0.500	1.397	0.097	0.497	1.359	0.102	0.489	1.329	0.481
16004	0.500	1.397	0.431	0.515	1.472	0.371	0.492	1.346	0.508
16005	0.500	1.397	0.185	0.503	1.396	0.491	0.492	1.346	0.262
16006	0.447	1.060	0.385	0.515	1.472	0.422	0.500	1.397	0.296
16007	0.466	1.178	0.262	0.515	1.472	0.273	0.503	1.413	0.224
16008	0.489	1.329	0.481	0.462	1.133	0.478	0.513	1.481	0.185
16009	0.261	-0.137	0.185	0.371	0.549	0.512	0.097	-1.181	0.508
16010	0.461	1.144	0.467	0.500	1.378	0.444	0.463	1.161	0.296
16011	0.511	1.464	0.512	0.500	1.378	0.193	0.463	1.161	0.097
16012	0.511	1.464	0.224	0.500	1.378	0.102	0.463	1.161	0.467
16013	0.450	1.076	0.450	0.515	1.472	0.491	0.495	1.363	0.467
16014	0.447	1.060	0.492	0.503	1.396	0.309	0.487	1.312	0.512
16015	0.466	1.178	0.358	0.518	1.491	0.508	0.495	1.363	0.481
16016	0.466	1.178	0.262	0.518	1.491	0.508	0.495	1.363	0.262
16017	0.300	0.116	0.492	0.193	-0.600	0.478	0.226	-0.356	0.262
16018	0.263	-0.120	0.224	0.102	-1.184	0.508	0.226	-0.356	0.224
16019	0.224	-0.373	0.481	0.193	-0.600	0.235	0.266	-0.103	0.467
16020	0.468	1.194	0.481	0.497	1.359	0.462	0.450	1.076	0.185
16021	0.511	1.464	0.185	0.512	1.453	0.508	0.503	1.413	0.450
16022	0.484	1.295	0.328	0.515	1.472	0.462	0.511	1.464	0.431
16023	0.000	?	0.262	0.105	-1.165	0.462	0.384	0.655	0.431
16024	0.000	?	0.492	0.105	-1.165	0.508	0.384	0.655	0.431
16025	0.450	1.076	0.492	0.377	0.587	0.235	0.503	1.413	0.097
16026	0.500	1.397	0.481	0.497	1.359	0.193	0.468	1.194	0.262
16027	0.326	0.285	0.328	0.427	0.907	0.512	0.329	0.301	0.431
16028	0.261	-0.137	0.501	0.275	-0.073	0.501	0.332	0.318	0.409
16029	0.492	1.346	0.467	0.485	1.283	0.462	0.405	0.790	0.409
16030	0.511	1.464	0.296	0.509	1.434	0.501	0.511	1.464	0.512
16031	0.489	1.329	0.450	0.444	1.020	0.462	0.413	0.841	0.450
16032	0.050	-1.485	0.450	0.196	-0.581	0.462	0.358	0.487	0.450
16033	0.261	-0.137	0.224	0.102	-1.184	0.398	0.261	-0.137	0.508
16034	0.432	0.958	0.262	0.494	1.340	0.235	0.513	1.481	0.097
16035	0.263	-0.120	0.262	0.307	0.135	0.478	0.361	0.504	0.385
16036	0.476	1.245	0.050	0.512	1.453	0.309	0.500	1.397	0.450
16037	0.495	1.363	0.467	0.512	1.453	0.508	0.508	1.447	0.224
16038	0.513	1.481	0.431	0.474	1.208	0.501	0.487	1.312	0.431
16039	0.511	1.464	0.431	0.503	1.396	0.501	0.479	1.262	0.185

16040	0.229	-0.339	0.512	0.237	-0.317	0.273	0.295	0.082	0.224
16041	0.482	1.279	0.450	0.459	1.114	0.501	0.484	1.295	0.296
16042	0.468	1.194	0.508	0.506	1.415	0.478	0.505	1.430	0.296
16043	0.384	0.655	0.513	0.465	1.152	0.462	0.500	1.397	0.296
16044	0.487	1.312	0.512	0.462	1.133	0.273	0.511	1.464	0.512
16045	0.497	1.380	0.409	0.506	1.415	0.508	0.432	0.958	0.142
16046	0.050	-1.485	0.508	0.237	-0.317	0.514	0.432	0.958	0.358
16047	0.224	-0.373	0.142	0.196	-0.581	0.478	0.261	-0.137	0.296
16048	0.511	1.464	0.501	0.518	1.491	0.478	0.476	1.245	0.508
16049	0.511	1.464	0.492	0.500	1.378	0.478	0.461	1.144	0.262
16050	0.429	0.942	0.492	0.462	1.133	0.462	0.513	1.481	0.450
16051	0.508	1.447	0.501	0.497	1.359	0.478	0.405	0.790	0.513
16052	0.487	1.312	0.481	0.462	1.133	0.512	0.505	1.430	0.224
16053	0.489	1.329	0.467	0.468	1.170	0.235	0.466	1.178	0.431
16054	0.329	0.301	0.508	0.401	0.737	0.309	0.184	-0.625	0.492
16055	0.295	0.082	0.501	0.234	-0.336	0.512	0.413	0.841	0.185
16056	0.450	1.076	0.142	0.477	1.227	0.273	0.455	1.110	0.224
16057	0.000	?	0.262	0.310	0.153	0.235	0.295	0.082	0.508
16058	0.471	1.211	0.512	0.506	1.415	0.512	0.505	1.430	0.097
16059	0.503	1.413	0.512	0.506	1.415	0.193	0.511	1.464	0.508
16060	0.505	1.430	0.409	0.497	1.359	0.273	0.503	1.413	0.508
16061	0.300	0.116	0.050	0.272	-0.091	0.398	0.361	0.504	0.224
16062	0.503	1.413	0.508	0.503	1.396	0.273	0.503	1.413	0.328
16063	0.503	1.413	0.328	0.480	1.246	0.491	0.484	1.295	0.097
16064	0.508	1.447	0.492	0.459	1.114	0.491	0.450	1.076	0.512
16065	0.187	-0.608	0.513	0.272	-0.091	0.501	0.261	-0.137	0.431
16066	0.508	1.447	0.450	0.506	1.415	0.235	0.492	1.346	0.431
16067	0.503	1.413	0.262	0.512	1.453	0.491	0.432	0.958	0.262
16068	0.482	1.279	0.262	0.480	1.246	0.512	0.497	1.380	0.501
16069	0.482	1.279	0.492	0.480	1.246	0.491	0.497	1.380	0.481
16070	0.482	1.279	0.492	0.480	1.246	0.371	0.497	1.380	0.185
16071	0.476	1.245	0.508	0.465	1.152	0.193	0.484	1.295	0.501
16072	0.505	1.430	0.481	0.480	1.246	0.512	0.511	1.464	0.501
16073	0.495	1.363	0.508	0.515	1.472	0.273	0.487	1.312	0.262
16074	0.224	-0.373	0.467	0.234	-0.336	0.273	0.384	0.655	0.142
16075	0.261	-0.137	0.512	0.310	0.153	0.501	0.224	-0.373	0.262
16076	0.408	0.807	0.508	0.512	1.453	0.444	0.487	1.312	0.224
16077	0.295	0.082	0.501	0.275	-0.073	0.193	0.097	-1.181	0.512
16078	0.187	-0.608	0.097	0.237	-0.317	0.149	0.142	-0.895	0.097
16079	0.468	1.194	0.358	0.515	1.472	0.512	0.495	1.363	0.513
16080	0.295	0.082	0.358	0.196	-0.581	0.273	0.184	-0.625	0.492
16081	0.184	-0.625	0.481	0.272	-0.091	0.309	0.184	-0.625	0.492
16082	0.495	1.363	0.224	0.401	0.737	0.309	0.511	1.464	0.513
16083	0.266	-0.103	0.467	0.272	-0.091	0.371	0.142	-0.895	0.492
16084	0.505	1.430	0.512	0.491	1.321	0.501	0.513	1.481	0.431
16085	0.505	1.430	0.481	0.491	1.321	0.398	0.513	1.481	0.492
16086	0.450	1.076	0.142	0.503	1.396	0.193	0.508	1.447	0.224
16087	0.097	-1.181	0.262	0.237	-0.317	0.193	0.226	-0.356	0.512
16088	0.511	1.464	0.492	0.485	1.283	0.512	0.463	1.161	0.296
16089	0.503	1.413	0.492	0.512	1.453	0.341	0.500	1.397	0.512
16090	0.445	1.043	0.508	0.491	1.321	0.309	0.447	1.060	0.508
16091	0.503	1.413	0.508	0.371	0.549	0.309	0.432	0.958	0.512
16092	0.224	-0.373	0.385	0.193	-0.600	0.235	0.184	-0.625	0.512
16093	0.455	1.110	0.513	0.515	1.472	0.235	0.511	1.464	0.492
16094	0.263	-0.120	0.409	0.275	-0.073	0.501	0.261	-0.137	0.492
16095	0.263	-0.120	0.512	0.275	-0.073	0.491	0.261	-0.137	0.501
16096	0.503	1.413	0.185	0.503	1.396	0.478	0.482	1.279	0.492
16097	0.479	1.262	0.185	0.444	1.020	0.309	0.471	1.211	0.262
16098	0.297	0.099	0.358	0.193	-0.600	0.309	0.226	-0.356	0.185
16099	0.295	0.082	0.185	0.149	-0.882	0.508	0.184	-0.625	0.450
16100	0.511	1.464	0.450	0.509	1.434	0.508	0.479	1.262	0.512

16101	0.050	-1.485	0.142	0.272	-0.091	0.462	0.224	-0.373	0.513
16102	0.050	-1.485	0.142	0.310	0.153	0.508	0.224	-0.373	0.262
16103	0.050	-1.485	0.450	0.310	0.153	0.235	0.224	-0.373	0.512
16104	0.492	1.346	0.142	0.371	0.549	0.501	0.508	1.447	0.296
16105	0.508	1.447	0.450	0.503	1.396	0.491	0.482	1.279	0.512
16106	0.411	0.824	0.508	0.398	0.718	0.444	0.142	-0.895	0.481
16107	0.226	-0.356	0.467	0.196	-0.581	0.491	0.326	0.285	0.508
16108	0.261	-0.137	0.501	0.193	-0.600	0.398	0.142	-0.895	0.409
16109	0.471	1.211	0.508	0.515	1.472	0.309	0.513	1.481	0.185
16110	0.050	-1.485	0.358	0.345	0.379	0.235	0.297	0.099	0.492
16111	0.142	-0.895	0.501	0.313	0.172	0.422	0.224	-0.373	0.492
16112	0.329	0.301	0.431	0.313	0.172	0.398	0.297	0.099	0.492
16113	0.224	-0.373	0.224	0.240	-0.299	0.309	0.142	-0.895	0.512
16114	0.184	-0.625	0.492	0.234	-0.336	0.309	0.184	-0.625	0.467
16115	0.492	1.346	0.142	0.503	1.396	0.501	0.463	1.161	0.512
16116	0.489	1.329	0.481	0.491	1.321	0.444	0.476	1.245	0.467
16117	0.505	1.430	0.224	0.477	1.227	0.193	0.476	1.245	0.513
16118	0.187	-0.608	0.296	0.310	0.153	0.273	0.326	0.285	0.501
16119	0.184	-0.625	0.185	0.313	0.172	0.273	0.184	-0.625	0.328
16120	0.474	1.228	0.185	0.509	1.434	0.273	0.384	0.655	0.328
16121	0.461	1.144	0.481	0.509	1.434	0.371	0.408	0.807	0.097
16122	0.479	1.262	0.097	0.459	1.114	0.512	0.482	1.279	0.508
16123	0.495	1.363	0.492	0.509	1.434	0.235	0.408	0.807	0.296
16124	0.297	0.099	0.358	0.234	-0.336	0.193	0.050	-1.485	0.508
16125	0.489	1.329	0.358	0.500	1.378	0.422	0.505	1.430	0.481
16126	0.461	1.144	0.508	0.488	1.302	0.501	0.408	0.807	0.467
16127	0.500	1.397	0.050	0.444	1.020	0.273	0.513	1.481	0.224
16128	0.500	1.397	0.050	0.488	1.302	0.309	0.466	1.178	0.142
16129	0.097	-1.181	0.097	0.401	0.737	0.341	0.050	-1.485	0.508
16130	0.050	-1.485	0.262	0.310	0.153	0.491	0.187	-0.608	0.501
16131	0.142	-0.895	0.185	0.234	-0.336	0.462	0.184	-0.625	0.512
16132	0.411	0.824	0.512	0.424	0.888	0.273	0.492	1.346	0.513
16133	0.300	0.116	0.508	0.401	0.737	0.309	0.329	0.301	0.142
16134	0.145	-0.878	0.508	0.310	0.153	0.341	0.142	-0.895	0.508
16135	0.142	-0.895	0.050	0.310	0.153	0.235	0.332	0.318	0.508
16136	0.508	1.447	0.512	0.500	1.378	0.341	0.503	1.413	0.501
16137	0.497	1.380	0.050	0.442	1.001	0.149	0.513	1.481	0.224
16138	0.097	-1.181	0.501	0.193	-0.600	0.235	0.326	0.285	0.224
16139	0.329	0.301	0.513	0.272	-0.091	0.478	0.142	-0.895	0.481
16140	0.329	0.301	0.481	0.272	-0.091	0.508	0.142	-0.895	0.185
16141	0.329	0.301	0.097	0.272	-0.091	0.491	0.184	-0.625	0.409
16142	0.226	-0.356	0.512	0.374	0.568	0.512	0.295	0.082	0.296
16143	0.500	1.397	0.501	0.518	1.491	0.193	0.389	0.689	0.296
16144	0.224	-0.373	0.097	0.240	-0.299	0.193	0.224	-0.373	0.467
16145	0.145	-0.878	0.501	0.193	-0.600	0.273	0.297	0.099	0.097
16146	0.468	1.194	0.501	0.424	0.888	0.501	0.492	1.346	0.450
16147	0.500	1.397	0.296	0.503	1.396	0.273	0.432	0.958	0.501
16148	0.097	-1.181	0.512	0.275	-0.073	0.462	0.184	-0.625	0.512
16149	0.142	-0.895	0.492	0.310	0.153	0.514	0.266	-0.103	0.481
16150	0.184	-0.625	0.512	0.342	0.361	0.501	0.187	-0.608	0.512
16151	0.476	1.245	0.512	0.491	1.321	0.501	0.479	1.262	0.296
16152	0.468	1.194	0.296	0.468	1.170	0.273	0.503	1.413	0.512
16153	0.184	-0.625	0.142	0.275	-0.073	0.193	0.263	-0.120	0.512
16154	0.226	-0.356	0.185	0.310	0.153	0.501	0.295	0.082	0.450
16155	0.226	-0.356	0.508	0.342	0.361	0.501	0.224	-0.373	0.481
16156	0.184	-0.625	0.508	0.234	-0.336	0.501	0.184	-0.625	0.224
16157	0.489	1.329	0.508	0.339	0.342	0.508	0.508	1.447	0.513
16158	0.184	-0.625	0.481	0.149	-0.882	0.491	0.297	0.099	0.050
16159	0.224	-0.373	0.508	0.237	-0.317	0.491	0.097	-1.181	0.409
16160	0.466	1.178	0.450	0.477	1.227	0.514	0.466	1.178	0.185
16161	0.432	0.958	0.508	0.509	1.434	0.193	0.468	1.194	0.185

16162	0.505	1.430	0.409	0.494	1.340	0.508	0.505	1.430	0.481
16163	0.447	1.060	0.409	0.509	1.434	0.501	0.476	1.245	0.328
16164	0.142	-0.895	0.492	0.196	-0.581	0.273	0.263	-0.120	0.467
16165	0.142	-0.895	0.450	0.196	-0.581	0.501	0.263	-0.120	0.296
16166	0.050	-1.485	0.512	0.272	-0.091	0.193	0.224	-0.373	0.358
16167	0.495	1.363	0.296	0.494	1.340	0.149	0.463	1.161	0.224
16168	0.261	-0.137	0.513	0.272	-0.091	0.193	0.184	-0.625	0.467
16169	0.492	1.346	0.492	0.462	1.133	0.193	0.447	1.060	0.409
16170	0.508	1.447	0.224	0.512	1.453	0.193	0.432	0.958	0.328
16171	0.500	1.397	0.224	0.503	1.396	0.398	0.432	0.958	0.296
16172	0.505	1.430	0.501	0.503	1.396	0.273	0.432	0.958	0.328
16173	0.332	0.318	0.481	0.275	-0.073	0.235	0.097	-1.181	0.492
16174	0.263	-0.120	0.512	0.193	-0.600	0.273	0.261	-0.137	0.501
16175	0.508	1.447	0.097	0.497	1.359	0.514	0.426	0.925	0.467
16176	0.511	1.464	0.508	0.497	1.359	0.462	0.405	0.790	0.358
16177	0.508	1.447	0.501	0.497	1.359	0.508	0.405	0.790	0.508
16178	0.495	1.363	0.501	0.509	1.434	0.398	0.508	1.447	0.431
16179	0.511	1.464	0.142	0.491	1.321	0.102	0.447	1.060	0.481
16180	0.513	1.481	0.512	0.491	1.321	0.514	0.447	1.060	0.467
16181	0.453	1.093	0.185	0.518	1.491	0.478	0.511	1.464	0.508
16182	0.300	0.116	0.185	0.193	-0.600	0.341	0.097	-1.181	0.262
16183	0.508	1.447	0.492	0.515	1.472	0.309	0.382	0.638	0.467
16184	0.479	1.262	0.508	0.506	1.415	0.371	0.450	1.076	0.501
16185	0.050	-1.485	0.328	0.275	-0.073	0.273	0.224	-0.373	0.358
16186	0.497	1.380	0.142	0.497	1.359	0.501	0.434	0.975	0.481
16187	0.224	-0.373	0.508	0.193	-0.600	0.508	0.184	-0.625	0.512
16188	0.187	-0.608	0.296	0.149	-0.882	0.193	0.224	-0.373	0.224
16189	0.097	-1.181	0.185	0.196	-0.581	0.193	0.295	0.082	0.385
16190	0.097	-1.181	0.492	0.196	-0.581	0.273	0.295	0.082	0.512
16191	0.097	-1.181	0.296	0.196	-0.581	0.508	0.295	0.082	0.492
16192	0.432	0.958	0.296	0.398	0.718	0.462	0.513	1.481	0.431
16193	0.187	-0.608	0.262	0.272	-0.091	0.462	0.142	-0.895	0.409
16194	0.389	0.689	0.262	0.234	-0.336	0.273	0.358	0.487	0.185
16195	0.261	-0.137	0.508	0.275	-0.073	0.462	0.297	0.099	0.262
16196	0.479	1.262	0.508	0.515	1.472	0.444	0.505	1.430	0.431
16197	0.187	-0.608	0.512	0.465	1.152	0.102	0.261	-0.137	0.467
16198	0.471	1.211	0.512	0.506	1.415	0.444	0.455	1.110	0.492
16199	0.511	1.464	0.512	0.401	0.737	0.444	0.513	1.481	0.501
16200	0.224	-0.373	0.097	0.102	-1.184	0.193	0.226	-0.356	0.501
16201	0.450	1.076	0.358	0.515	1.472	0.149	0.429	0.942	0.513
16202	0.495	1.363	0.467	0.474	1.208	0.102	0.484	1.295	0.501
16203	0.361	0.504	0.508	0.345	0.379	0.371	0.184	-0.625	0.224
16204	0.263	-0.120	0.450	0.307	0.135	0.398	0.224	-0.373	0.224
16205	0.492	1.346	0.492	0.368	0.530	0.273	0.503	1.413	0.481
16206	0.226	-0.356	0.185	0.272	-0.091	0.398	0.097	-1.181	0.508
16207	0.476	1.245	0.224	0.503	1.396	0.508	0.508	1.447	0.492
16208	0.476	1.245	0.050	0.509	1.434	0.501	0.508	1.447	0.358
16209	0.187	-0.608	0.358	0.193	-0.600	0.491	0.226	-0.356	0.508
16210	0.329	0.301	0.512	0.193	-0.600	0.478	0.329	0.301	0.501
16211	0.261	-0.137	0.513	0.275	-0.073	0.491	0.097	-1.181	0.050
16212	0.489	1.329	0.508	0.506	1.415	0.501	0.508	1.447	0.328
16213	0.487	1.312	0.450	0.462	1.133	0.371	0.429	0.942	0.431
16214	0.476	1.245	0.512	0.462	1.133	0.478	0.429	0.942	0.467
16215	0.329	0.301	0.492	0.272	-0.091	0.149	0.263	-0.120	0.501
16216	0.492	1.346	0.481	0.465	1.152	0.444	0.505	1.430	0.296
16217	0.471	1.211	0.050	0.444	1.020	0.501	0.476	1.245	0.481
16218	0.295	0.082	0.296	0.102	-1.184	0.422	0.184	-0.625	0.512
16219	0.447	1.060	0.142	0.442	1.001	0.491	0.500	1.397	0.512
16220	0.447	1.060	0.481	0.442	1.001	0.444	0.500	1.397	0.224
16221	0.224	-0.373	0.492	0.196	-0.581	0.444	0.261	-0.137	0.513
16222	0.263	-0.120	0.185	0.149	-0.882	0.512	0.142	-0.895	0.224

16223	0.261	-0.137	0.508	0.102	-1.184	0.491	0.261	-0.137	0.050
16224	0.050	-1.485	0.185	0.371	0.549	0.341	0.224	-0.373	0.512
16225	0.463	1.161	0.328	0.404	0.756	0.193	0.511	1.464	0.513
16226	0.432	0.958	0.512	0.272	-0.091	0.491	0.097	-1.181	0.296
16227	0.429	0.942	0.512	0.398	0.718	0.478	0.508	1.447	0.224
16228	0.511	1.464	0.508	0.503	1.396	0.508	0.489	1.329	0.142
16229	0.450	1.076	0.467	0.500	1.378	0.235	0.489	1.329	0.467
16230	0.503	1.413	0.481	0.485	1.283	0.478	0.513	1.481	0.358
16231	0.513	1.481	0.296	0.474	1.208	0.398	0.487	1.312	0.224
16232	0.513	1.481	0.508	0.491	1.321	0.478	0.426	0.925	0.481
16233	0.411	0.824	0.508	0.500	1.378	0.501	0.487	1.312	0.385
16234	0.000	?	0.142	0.371	0.549	0.462	0.224	-0.373	0.385
16235	0.508	1.447	0.513	0.480	1.246	0.102	0.516	1.498	0.142
16236	0.142	-0.895	0.450	0.152	-0.863	0.193	0.295	0.082	0.492
16237	0.497	1.380	0.492	0.447	1.039	0.478	0.513	1.481	0.501
16238	0.492	1.346	0.450	0.509	1.434	0.491	0.505	1.430	0.508
16239	0.489	1.329	0.328	0.424	0.888	0.444	0.513	1.481	0.467
16240	0.500	1.397	0.512	0.494	1.340	0.462	0.513	1.481	0.450
16241	0.479	1.262	0.450	0.447	1.039	0.371	0.492	1.346	0.450
16242	0.463	1.161	0.492	0.447	1.039	0.512	0.492	1.346	0.328
16243	0.511	1.464	0.512	0.512	1.453	0.508	0.497	1.380	0.097
16244	0.500	1.397	0.097	0.488	1.302	0.501	0.489	1.329	0.431
16245	0.142	-0.895	0.142	0.348	0.398	0.491	0.261	-0.137	0.501
16246	0.266	-0.103	0.185	0.193	-0.600	0.149	0.184	-0.625	0.481
16247	0.511	1.464	0.050	0.491	1.321	0.398	0.450	1.076	0.492
16248	0.511	1.464	0.050	0.480	1.246	0.053	0.513	1.481	0.097
16249	0.411	0.824	0.508	0.506	1.415	0.478	0.518	1.514	0.142
16250	0.050	-1.485	0.481	0.234	-0.336	0.273	0.266	-0.103	0.296
16251	0.508	1.447	0.501	0.480	1.246	0.514	0.518	1.514	0.142
16252	0.332	0.318	0.508	0.401	0.737	0.491	0.297	0.099	0.501
16253	0.489	1.329	0.224	0.482	1.265	0.512	0.516	1.498	0.481
16254	0.511	1.464	0.512	0.506	1.415	0.193	0.479	1.262	0.508
16255	0.445	1.043	0.328	0.459	1.114	0.235	0.505	1.430	0.512
16256	0.263	-0.120	0.513	0.102	-1.184	0.508	0.408	0.807	0.508
16257	0.263	-0.120	0.328	0.193	-0.600	0.508	0.184	-0.625	0.481
16258	0.495	1.363	0.142	0.482	1.265	0.491	0.492	1.346	0.467
16259	0.495	1.363	0.512	0.494	1.340	0.491	0.492	1.346	0.262
16260	0.511	1.464	0.492	0.447	1.039	0.273	0.492	1.346	0.185
16261	0.489	1.329	0.224	0.459	1.114	0.508	0.511	1.464	0.142
16262	0.508	1.447	0.224	0.371	0.549	0.514	0.471	1.211	0.513
16263	0.466	1.178	0.224	0.515	1.472	0.508	0.518	1.514	0.481
16264	0.505	1.430	0.481	0.506	1.415	0.444	0.466	1.178	0.262
16265	0.503	1.413	0.508	0.497	1.359	0.371	0.513	1.481	0.492
16266	0.500	1.397	0.501	0.488	1.302	0.508	0.503	1.413	0.224
16267	0.097	-1.181	0.185	0.149	-0.882	0.341	0.329	0.301	0.508
16268	0.358	0.487	0.492	0.398	0.718	0.501	0.332	0.318	0.409
16269	0.355	0.470	0.050	0.053	-1.504	0.193	0.097	-1.181	0.467
16270	0.479	1.262	0.508	0.474	1.208	0.193	0.505	1.430	0.508
16271	0.226	-0.356	0.481	0.272	-0.091	0.491	0.295	0.082	0.467
16272	0.466	1.178	0.508	0.512	1.453	0.462	0.503	1.413	0.467
16273	0.513	1.481	0.508	0.494	1.340	0.514	0.482	1.279	0.508
16274	0.476	1.245	0.508	0.515	1.472	0.491	0.463	1.161	0.050
16275	0.145	-0.878	0.142	0.193	-0.600	0.501	0.224	-0.373	0.492
16276	0.263	-0.120	0.142	0.234	-0.336	0.512	0.142	-0.895	0.450
16277	0.492	1.346	0.508	0.509	1.434	0.501	0.505	1.430	0.224
16278	0.489	1.329	0.508	0.509	1.434	0.193	0.497	1.380	0.450
16279	0.497	1.380	0.508	0.494	1.340	0.512	0.511	1.464	0.508
16280	0.497	1.380	0.224	0.494	1.340	0.491	0.513	1.481	0.501
16281	0.382	0.638	0.185	0.272	-0.091	0.309	0.142	-0.895	0.508
16282	0.508	1.447	0.185	0.512	1.453	0.501	0.500	1.397	0.142
16283	0.411	0.824	0.492	0.509	1.434	0.193	0.500	1.397	0.385

16284	0.511	1.464	0.185	0.500	1.378	0.462	0.500	1.397	0.467
16285	0.184	-0.625	0.224	0.447	1.039	0.273	0.224	-0.373	0.467
16286	0.184	-0.625	0.512	0.377	0.587	0.341	0.224	-0.373	0.262
16287	0.355	0.470	0.431	0.509	1.434	0.193	0.479	1.262	0.481
16288	0.184	-0.625	0.512	0.345	0.379	0.193	0.184	-0.625	0.296
16289	0.453	1.093	0.512	0.494	1.340	0.398	0.405	0.790	0.224
16290	0.142	-0.895	0.492	0.193	-0.600	0.273	0.295	0.082	0.262
16291	0.142	-0.895	0.328	0.193	-0.600	0.508	0.295	0.082	0.262
16292	0.453	1.093	0.185	0.494	1.340	0.235	0.466	1.178	0.262
16293	0.142	-0.895	0.508	0.465	1.152	0.235	0.097	-1.181	0.481
16294	0.447	1.060	0.467	0.512	1.453	0.309	0.447	1.060	0.467
16295	0.503	1.413	0.467	0.488	1.302	0.422	0.503	1.413	0.409
16296	0.463	1.161	0.296	0.497	1.359	0.309	0.516	1.498	0.501
16297	0.497	1.380	0.050	0.512	1.453	0.309	0.479	1.262	0.185
16298	0.505	1.430	0.185	0.503	1.396	0.309	0.511	1.464	0.513
16299	0.358	0.487	0.467	0.193	-0.600	0.273	0.300	0.116	0.050
16300	0.497	1.380	0.512	0.518	1.491	0.235	0.513	1.481	0.512
16301	0.434	0.975	0.296	0.485	1.283	0.512	0.505	1.430	0.185
16302	0.224	-0.373	0.481	0.313	0.172	0.512	0.450	1.076	0.385
16303	0.489	1.329	0.513	0.497	1.359	0.462	0.482	1.279	0.508
16304	0.145	-0.878	0.501	0.193	-0.600	0.341	0.224	-0.373	0.501
16305	0.482	1.279	0.512	0.465	1.152	0.491	0.513	1.481	0.142
16306	0.226	-0.356	0.512	0.272	-0.091	0.193	0.050	-1.485	0.142
16307	0.295	0.082	0.097	0.345	0.379	0.512	0.413	0.841	0.142
16308	0.184	-0.625	0.501	0.196	-0.581	0.514	0.187	-0.608	0.513
16309	0.184	-0.625	0.492	0.196	-0.581	0.501	0.187	-0.608	0.512
16310	0.482	1.279	0.142	0.401	0.737	0.193	0.484	1.295	0.508
16311	0.097	-1.181	0.097	0.272	-0.091	0.512	0.329	0.301	0.097
16312	0.495	1.363	0.512	0.506	1.415	0.508	0.463	1.161	0.501
16313	0.355	0.470	0.508	0.234	-0.336	0.235	0.297	0.099	0.185
16314	0.355	0.470	0.508	0.234	-0.336	0.512	0.358	0.487	0.492
16315	0.000	?	0.508	0.307	0.135	0.512	0.224	-0.373	0.501
16316	0.500	1.397	0.492	0.421	0.869	0.309	0.468	1.194	0.501
16317	0.050	-1.485	0.224	0.307	0.135	0.514	0.411	0.824	0.501
16318	0.050	-1.485	0.097	0.310	0.153	0.422	0.332	0.318	0.501
16319	0.100	-1.164	0.262	0.310	0.153	0.478	0.297	0.099	0.224
16320	0.263	-0.120	0.492	0.272	-0.091	0.501	0.000	?	0.185
16321	0.187	-0.608	0.501	0.234	-0.336	0.309	0.332	0.318	0.513
16322	0.511	1.464	0.296	0.515	1.472	0.235	0.492	1.346	0.508
16323	0.505	1.430	0.185	0.515	1.472	0.273	0.503	1.413	0.492
16324	0.497	1.380	0.224	0.462	1.133	0.512	0.468	1.194	0.142
16325	0.050	-1.485	0.513	0.342	0.361	0.512	0.358	0.487	0.142
16326	0.500	1.397	0.097	0.491	1.321	0.491	0.518	1.514	0.142
16327	0.050	-1.485	0.185	0.193	-0.600	0.508	0.432	0.958	0.431
16328	0.492	1.346	0.185	0.512	1.453	0.508	0.482	1.279	0.224
16329	0.503	1.413	0.492	0.518	1.491	0.501	0.466	1.178	0.358
16330	0.474	1.228	0.185	0.503	1.396	0.501	0.508	1.447	0.431
16331	0.097	-1.181	0.508	0.196	-0.581	0.193	0.263	-0.120	0.501
16332	0.505	1.430	0.512	0.515	1.472	0.235	0.468	1.194	0.501
16333	0.505	1.430	0.501	0.506	1.415	0.462	0.495	1.363	0.501
16334	0.097	-1.181	0.513	0.237	-0.317	0.512	0.361	0.504	0.513
16335	0.495	1.363	0.467	0.509	1.434	0.478	0.484	1.295	0.262
16336	0.495	1.363	0.512	0.509	1.434	0.053	0.516	1.498	0.097
16337	0.295	0.082	0.512	0.313	0.172	0.491	0.226	-0.356	0.508
16338	0.503	1.413	0.224	0.515	1.472	0.508	0.384	0.655	0.450
16339	0.484	1.295	0.501	0.421	0.869	0.273	0.516	1.498	0.467
16340	0.505	1.430	0.508	0.477	1.227	0.149	0.495	1.363	0.296
16341	0.513	1.481	0.296	0.500	1.378	0.508	0.434	0.975	0.385
16342	0.300	0.116	0.512	0.310	0.153	0.462	0.411	0.824	0.142
16343	0.145	-0.878	0.431	0.237	-0.317	0.508	0.184	-0.625	0.492
16344	0.184	-0.625	0.050	0.275	-0.073	0.273	0.266	-0.103	0.492

16345	0.505	1.430	0.492	0.515	1.472	0.512	0.432	0.958	0.142
16346	0.497	1.380	0.512	0.520	1.509	0.462	0.463	1.161	0.508
16347	0.497	1.380	0.508	0.497	1.359	0.501	0.492	1.346	0.501
16348	0.482	1.279	0.512	0.509	1.434	0.193	0.497	1.380	0.450
16349	0.513	1.481	0.508	0.509	1.434	0.501	0.497	1.380	0.385
16350	0.450	1.076	0.224	0.497	1.359	0.422	0.508	1.447	0.385
16351	0.503	1.413	0.142	0.497	1.359	0.309	0.500	1.397	0.328
16352	0.411	0.824	0.142	0.193	-0.600	0.462	0.226	-0.356	0.508
16353	0.411	0.824	0.467	0.234	-0.336	0.478	0.226	-0.356	0.481
16354	0.489	1.329	0.512	0.459	1.114	0.273	0.479	1.262	0.224
16355	0.455	1.110	0.097	0.509	1.434	0.235	0.503	1.413	0.262
16356	0.511	1.464	0.508	0.480	1.246	0.371	0.495	1.363	0.501
16357	0.300	0.116	0.481	0.053	-1.504	0.508	0.358	0.487	0.467
16358	0.511	1.464	0.501	0.491	1.321	0.102	0.505	1.430	0.467
16359	0.489	1.329	0.142	0.509	1.434	0.149	0.500	1.397	0.467
16360	0.226	-0.356	0.513	0.275	-0.073	0.508	0.050	-1.485	0.508
16361	0.226	-0.356	0.450	0.149	-0.882	0.309	0.329	0.301	0.262
16362	0.503	1.413	0.358	0.509	1.434	0.309	0.434	0.975	0.296
16363	0.484	1.295	0.328	0.462	1.133	0.235	0.466	1.178	0.262
16364	0.508	1.447	0.512	0.503	1.396	0.235	0.500	1.397	0.296
16365	0.097	-1.181	0.467	0.275	-0.073	0.478	0.297	0.099	0.501
16366	0.503	1.413	0.481	0.515	1.472	0.478	0.476	1.245	0.508
16367	0.497	1.380	0.512	0.468	1.170	0.422	0.511	1.464	0.050
16368	0.505	1.430	0.185	0.500	1.378	0.462	0.508	1.447	0.262
16369	0.142	-0.895	0.450	0.196	-0.581	0.462	0.224	-0.373	0.328
16370	0.505	1.430	0.358	0.494	1.340	0.102	0.516	1.498	0.508
16371	0.184	-0.625	0.481	0.427	0.907	0.309	0.224	-0.373	0.328
16372	0.187	-0.608	0.512	0.310	0.153	0.478	0.050	-1.485	0.328
16373	0.484	1.295	0.097	0.468	1.170	0.371	0.511	1.464	0.328
16374	0.511	1.464	0.481	0.477	1.227	0.508	0.513	1.481	0.492
16375	0.329	0.301	0.512	0.275	-0.073	0.444	0.295	0.082	0.224
16376	0.142	-0.895	0.501	0.237	-0.317	0.273	0.224	-0.373	0.501
16377	0.000	?	0.501	0.374	0.568	0.273	0.142	-0.895	0.481
16378	0.500	1.397	0.262	0.509	1.434	0.193	0.466	1.178	0.508
16379	0.295	0.082	0.262	0.102	-1.184	0.273	0.355	0.470	0.513
16380	0.184	-0.625	0.262	0.149	-0.882	0.508	0.224	-0.373	0.508
16381	0.492	1.346	0.501	0.506	1.415	0.514	0.479	1.262	0.431
16382	0.295	0.082	0.492	0.310	0.153	0.491	0.387	0.672	0.431
16383	0.295	0.082	0.501	0.310	0.153	0.398	0.387	0.672	0.501
16384	0.261	-0.137	0.508	0.234	-0.336	0.512	0.142	-0.895	0.501
16385	0.261	-0.137	0.262	0.234	-0.336	0.512	0.495	1.363	0.185
16386	0.505	1.430	0.501	0.474	1.208	0.512	0.500	1.397	0.467
16387	0.505	1.430	0.262	0.474	1.208	0.273	0.505	1.430	0.501
16388	0.505	1.430	0.358	0.430	0.926	0.193	0.463	1.161	0.097
16389	0.511	1.464	0.296	0.462	1.133	0.273	0.458	1.127	0.431
16390	0.511	1.464	0.142	0.462	1.133	0.501	0.458	1.127	0.492
16391	0.097	-1.181	0.512	0.105	-1.165	0.501	0.326	0.285	0.481
16392	0.361	0.504	0.508	0.307	0.135	0.371	0.097	-1.181	0.512
16393	0.463	1.161	0.512	0.482	1.265	0.508	0.429	0.942	0.481
16394	0.508	1.447	0.296	0.374	0.568	0.309	0.497	1.380	0.097
16395	0.450	1.076	0.224	0.503	1.396	0.512	0.482	1.279	0.296
16396	0.495	1.363	0.262	0.444	1.020	0.371	0.495	1.363	0.262
16397	0.184	-0.625	0.142	0.272	-0.091	0.491	0.097	-1.181	0.450
16398	0.224	-0.373	0.450	0.275	-0.073	0.501	0.142	-0.895	0.467
16399	0.050	-1.485	0.492	0.193	-0.600	0.508	0.295	0.082	0.431
16400	0.358	0.487	0.450	0.272	-0.091	0.512	0.142	-0.895	0.450
16401	0.508	1.447	0.224	0.512	1.453	0.491	0.505	1.430	0.501
16402	0.508	1.447	0.431	0.515	1.472	0.235	0.476	1.245	0.467
16403	0.503	1.413	0.185	0.491	1.321	0.512	0.503	1.413	0.185
16404	0.453	1.093	0.450	0.401	0.737	0.514	0.511	1.464	0.385
16405	0.503	1.413	0.481	0.515	1.472	0.193	0.505	1.430	0.512

16406	0.484	1.295	0.358	0.515	1.472	0.512	0.479	1.262	0.185
16407	0.476	1.245	0.409	0.518	1.491	0.491	0.466	1.178	0.224
16408	0.050	-1.485	0.508	0.275	-0.073	0.501	0.263	-0.120	0.501
16409	0.297	0.099	0.501	0.193	-0.600	0.462	0.187	-0.608	0.512
16410	0.142	-0.895	0.501	0.275	-0.073	0.309	0.145	-0.878	0.508
16411	0.476	1.245	0.481	0.497	1.359	0.501	0.511	1.464	0.512
16412	0.492	1.346	0.501	0.500	1.378	0.422	0.484	1.295	0.262
16413	0.184	-0.625	0.467	0.371	0.549	0.444	0.266	-0.103	0.492
16414	0.500	1.397	0.296	0.509	1.434	0.273	0.492	1.346	0.296
16415	0.187	-0.608	0.512	0.310	0.153	0.501	0.226	-0.356	0.508
16416	0.329	0.301	0.431	0.518	1.491	0.149	0.508	1.447	0.512
16417	0.000	?	0.450	0.374	0.568	0.398	0.408	0.807	0.185
16418	0.505	1.430	0.097	0.491	1.321	0.422	0.466	1.178	0.501
16419	0.508	1.447	0.492	0.503	1.396	0.398	0.503	1.413	0.501
16420	0.505	1.430	0.185	0.506	1.415	0.398	0.463	1.161	0.501
16421	0.466	1.178	0.097	0.503	1.396	0.398	0.466	1.178	0.501
16422	0.484	1.295	0.185	0.488	1.302	0.501	0.511	1.464	0.185
16423	0.297	0.099	0.224	0.234	-0.336	0.514	0.050	-1.485	0.142
16424	0.511	1.464	0.501	0.509	1.434	0.501	0.492	1.346	0.185
16425	0.500	1.397	0.501	0.512	1.453	0.341	0.453	1.093	0.508
16426	0.142	-0.895	0.512	0.193	-0.600	0.444	0.226	-0.356	0.431
16427	0.513	1.481	0.508	0.506	1.415	0.235	0.450	1.076	0.512
16428	0.450	1.076	0.097	0.485	1.283	0.398	0.503	1.413	0.492
16429	0.495	1.363	0.358	0.497	1.359	0.235	0.500	1.397	0.097
16430	0.447	1.060	0.262	0.465	1.152	0.193	0.508	1.447	0.501
16431	0.329	0.301	0.467	0.310	0.153	0.462	0.145	-0.878	0.097
16432	0.508	1.447	0.385	0.503	1.396	0.444	0.387	0.672	0.050
16433	0.447	1.060	0.097	0.427	0.907	0.193	0.468	1.194	0.050
16434	0.489	1.329	0.224	0.442	1.001	0.193	0.468	1.194	0.142
16435	0.503	1.413	0.358	0.500	1.378	0.462	0.484	1.295	0.513
16436	0.100	-1.164	0.262	0.149	-0.882	0.478	0.295	0.082	0.501
16437	0.142	-0.895	0.185	0.401	0.737	0.514	0.224	-0.373	0.050
16438	0.184	-0.625	0.467	0.427	0.907	0.341	0.263	-0.120	0.097
16439	0.050	-1.485	0.508	0.401	0.737	0.462	0.263	-0.120	0.142
16440	0.050	-1.485	0.512	0.401	0.737	0.398	0.263	-0.120	0.512
16441	0.503	1.413	0.185	0.398	0.718	0.514	0.484	1.295	0.481
16442	0.476	1.245	0.481	0.497	1.359	0.508	0.468	1.194	0.512
16443	0.497	1.380	0.185	0.509	1.434	0.512	0.411	0.824	0.224
16444	0.503	1.413	0.501	0.497	1.359	0.512	0.500	1.397	0.501
16445	0.224	-0.373	0.501	0.342	0.361	0.512	0.184	-0.625	0.224
16446	0.511	1.464	0.409	0.444	1.020	0.193	0.513	1.481	0.501
16447	0.329	0.301	0.224	0.237	-0.317	0.235	0.050	-1.485	0.481
16448	0.516	1.498	0.142	0.404	0.756	0.462	0.508	1.447	0.142
16449	0.329	0.301	0.224	0.240	-0.299	0.514	0.184	-0.625	0.481
16450	0.142	-0.895	0.142	0.193	-0.600	0.491	0.382	0.638	0.513
16451	0.503	1.413	0.142	0.462	1.133	0.309	0.508	1.447	0.481
16452	0.492	1.346	0.508	0.447	1.039	0.235	0.505	1.430	0.513
16453	0.226	-0.356	0.501	0.196	-0.581	0.309	0.142	-0.895	0.481
16454	0.226	-0.356	0.450	0.196	-0.581	0.514	0.142	-0.895	0.481
16455	0.226	-0.356	0.512	0.196	-0.581	0.309	0.142	-0.895	0.481
16456	0.482	1.279	0.492	0.459	1.114	0.102	0.513	1.481	0.296
16457	0.503	1.413	0.467	0.477	1.227	0.512	0.508	1.447	0.431
16458	0.500	1.397	0.512	0.520	1.509	0.462	0.513	1.481	0.501
16459	0.184	-0.625	0.450	0.342	0.361	0.508	0.097	-1.181	0.501
16460	0.487	1.312	0.097	0.462	1.133	0.514	0.500	1.397	0.328
16461	0.050	-1.485	0.097	0.401	0.737	0.491	0.184	-0.625	0.450
16462	0.513	1.481	0.492	0.515	1.472	0.309	0.492	1.346	0.262
16463	0.484	1.295	0.501	0.509	1.434	0.309	0.500	1.397	0.358
16464	0.513	1.481	0.142	0.512	1.453	0.501	0.500	1.397	0.224
16465	0.513	1.481	0.224	0.512	1.453	0.512	0.500	1.397	0.262
16466	0.513	1.481	0.142	0.512	1.453	0.512	0.500	1.397	0.492

16467	0.142	-0.895	0.097	0.196	-0.581	0.102	0.224	-0.373	0.450
16468	0.145	-0.878	0.492	0.234	-0.336	0.309	0.187	-0.608	0.358
16469	0.505	1.430	0.185	0.462	1.133	0.273	0.513	1.481	0.492
16470	0.505	1.430	0.512	0.515	1.472	0.491	0.503	1.413	0.142
16471	0.508	1.447	0.142	0.485	1.283	0.491	0.489	1.329	0.185
16472	0.224	-0.373	0.481	0.310	0.153	0.149	0.142	-0.895	0.185
16473	0.187	-0.608	0.328	0.234	-0.336	0.514	0.142	-0.895	0.512
16474	0.187	-0.608	0.450	0.307	0.135	0.501	0.142	-0.895	0.296
16475	0.487	1.312	0.296	0.518	1.491	0.512	0.432	0.958	0.508
16476	0.184	-0.625	0.224	0.313	0.172	0.512	0.224	-0.373	0.224
16477	0.224	-0.373	0.481	0.105	-1.165	0.512	0.361	0.504	0.501
16478	0.513	1.481	0.262	0.509	1.434	0.341	0.426	0.925	0.296
16479	0.429	0.942	0.512	0.462	1.133	0.309	0.500	1.397	0.224
16480	0.503	1.413	0.512	0.500	1.378	0.512	0.508	1.447	0.050
16481	0.503	1.413	0.450	0.506	1.415	0.193	0.508	1.447	0.467
16482	0.489	1.329	0.481	0.488	1.302	0.371	0.513	1.481	0.481
16483	0.326	0.285	0.185	0.307	0.135	0.508	0.261	-0.137	0.481
16484	0.184	-0.625	0.513	0.310	0.153	0.501	0.097	-1.181	0.185
16485	0.508	1.447	0.296	0.506	1.415	0.501	0.503	1.413	0.492
16486	0.463	1.161	0.467	0.512	1.453	0.501	0.450	1.076	0.224
16487	0.463	1.161	0.512	0.512	1.453	0.501	0.466	1.178	0.431
16488	0.297	0.099	0.512	0.102	-1.184	0.193	0.297	0.099	0.431
16489	0.050	-1.485	0.508	0.307	0.135	0.273	0.382	0.638	0.328
16490	0.184	-0.625	0.508	0.278	-0.054	0.341	0.142	-0.895	0.185
16491	0.463	1.161	0.467	0.491	1.321	0.235	0.492	1.346	0.296
16492	0.513	1.481	0.501	0.494	1.340	0.478	0.495	1.363	0.262
16493	0.295	0.082	0.224	0.149	-0.882	0.478	0.142	-0.895	0.262
16494	0.482	1.279	0.467	0.509	1.434	0.273	0.508	1.447	0.262
16495	0.508	1.447	0.513	0.503	1.396	0.235	0.495	1.363	0.481
16496	0.492	1.346	0.508	0.518	1.491	0.193	0.453	1.093	0.512
16497	0.500	1.397	0.185	0.518	1.491	0.491	0.387	0.672	0.492
16498	0.500	1.397	0.467	0.518	1.491	0.273	0.387	0.672	0.450
16499	0.097	-1.181	0.358	0.342	0.361	0.273	0.329	0.301	0.492
16500	0.500	1.397	0.481	0.310	0.153	0.273	0.505	1.430	0.467
16501	0.492	1.346	0.385	0.520	1.509	0.491	0.482	1.279	0.385
16502	0.142	-0.895	0.385	0.193	-0.600	0.193	0.226	-0.356	0.450
16503	0.097	-1.181	0.385	0.374	0.568	0.514	0.263	-0.120	0.358
16504	0.500	1.397	0.296	0.509	1.434	0.508	0.503	1.413	0.328
16505	0.508	1.447	0.409	0.509	1.434	0.514	0.466	1.178	0.467
16506	0.508	1.447	0.224	0.509	1.434	0.491	0.466	1.178	0.262
16507	0.508	1.447	0.296	0.509	1.434	0.508	0.466	1.178	0.501
16508	0.484	1.295	0.262	0.503	1.396	0.444	0.513	1.481	0.328
16509	0.224	-0.373	0.262	0.193	-0.600	0.478	0.263	-0.120	0.328
16510	0.000	?	0.262	0.272	-0.091	0.309	0.297	0.099	0.262
16511	0.097	-1.181	0.501	0.342	0.361	0.444	0.263	-0.120	0.358
16512	0.263	-0.120	0.481	0.237	-0.317	0.501	0.295	0.082	0.501
16513	0.495	1.363	0.501	0.477	1.227	0.309	0.500	1.397	0.142
16514	0.503	1.413	0.431	0.477	1.227	0.501	0.508	1.447	0.467
16515	0.295	0.082	0.431	0.272	-0.091	0.508	0.050	-1.485	0.185
16516	0.184	-0.625	0.431	0.237	-0.317	0.371	0.263	-0.120	0.501
16517	0.226	-0.356	0.508	0.193	-0.600	0.478	0.329	0.301	0.224
16518	0.511	1.464	0.467	0.494	1.340	0.514	0.511	1.464	0.481
16519	0.097	-1.181	0.142	0.275	-0.073	0.512	0.332	0.318	0.224
16520	0.184	-0.625	0.142	0.275	-0.073	0.508	0.332	0.318	0.224
16521	0.184	-0.625	0.050	0.275	-0.073	0.512	0.332	0.318	0.431
16522	0.489	1.329	0.296	0.494	1.340	0.235	0.492	1.346	0.262
16523	0.184	-0.625	0.142	0.193	-0.600	0.309	0.226	-0.356	0.467
16524	0.508	1.447	0.431	0.509	1.434	0.309	0.497	1.380	0.467
16525	0.505	1.430	0.501	0.512	1.453	0.501	0.474	1.228	0.481
16526	0.500	1.397	0.481	0.515	1.472	0.491	0.503	1.413	0.467
16527	0.511	1.464	0.467	0.491	1.321	0.273	0.508	1.447	0.185

16528	0.461	1.144	0.328	0.503	1.396	0.478	0.508	1.447	0.450
16529	0.513	1.481	0.224	0.450	1.057	0.491	0.429	0.942	0.224
16530	0.513	1.481	0.262	0.485	1.283	0.512	0.429	0.942	0.512
16531	0.226	-0.356	0.481	0.310	0.153	0.273	0.000	?	0.431
16532	0.505	1.430	0.501	0.444	1.020	0.444	0.492	1.346	0.385
16533	0.505	1.430	0.492	0.497	1.359	0.444	0.497	1.380	0.492
16534	0.300	0.116	0.262	0.310	0.153	0.422	0.184	-0.625	0.431
16535	0.511	1.464	0.328	0.500	1.378	0.371	0.463	1.161	0.481
16536	0.429	0.942	0.409	0.509	1.434	0.491	0.495	1.363	0.481
16537	0.050	-1.485	0.328	0.374	0.568	0.444	0.097	-1.181	0.224
16538	0.497	1.380	0.450	0.482	1.265	0.512	0.429	0.942	0.492
16539	0.508	1.447	0.481	0.518	1.491	0.462	0.489	1.329	0.431
16540	0.503	1.413	0.224	0.515	1.472	0.235	0.476	1.245	0.481
16541	0.508	1.447	0.450	0.509	1.434	0.512	0.508	1.447	0.142
16542	0.503	1.413	0.513	0.515	1.472	0.193	0.476	1.245	0.508
16543	0.224	-0.373	0.492	0.240	-0.299	0.514	0.097	-1.181	0.224
16544	0.142	-0.895	0.097	0.313	0.172	0.462	0.295	0.082	0.508
16545	0.142	-0.895	0.508	0.313	0.172	0.309	0.261	-0.137	0.050
16546	0.466	1.178	0.185	0.503	1.396	0.462	0.445	1.043	0.481
16547	0.511	1.464	0.358	0.494	1.340	0.478	0.468	1.194	0.185
16548	0.097	-1.181	0.467	0.272	-0.091	0.478	0.432	0.958	0.097
16549	0.497	1.380	0.512	0.485	1.283	0.444	0.453	1.093	0.050
16550	0.479	1.262	0.512	0.494	1.340	0.149	0.500	1.397	0.185
16551	0.497	1.380	0.097	0.512	1.453	0.149	0.463	1.161	0.185
16552	0.142	-0.895	0.224	0.275	-0.073	0.149	0.187	-0.608	0.512
16553	0.516	1.498	0.512	0.442	1.001	0.508	0.387	0.672	0.508
16554	0.445	1.043	0.050	0.442	1.001	0.501	0.508	1.447	0.512
16555	0.358	0.487	0.512	0.427	0.907	0.478	0.184	-0.625	0.508
16556	0.329	0.301	0.492	0.374	0.568	0.501	0.224	-0.373	0.508
16557	0.513	1.481	0.512	0.488	1.302	0.193	0.492	1.346	0.508
16558	0.466	1.178	0.296	0.442	1.001	0.508	0.505	1.430	0.481
16559	0.479	1.262	0.450	0.509	1.434	0.273	0.503	1.413	0.467
16560	0.511	1.464	0.501	0.462	1.133	0.273	0.503	1.413	0.185
16561	0.184	-0.625	0.481	0.234	-0.336	0.309	0.263	-0.120	0.185
16562	0.453	1.093	0.481	0.518	1.491	0.273	0.489	1.329	0.142
16563	0.361	0.504	0.097	0.193	-0.600	0.422	0.295	0.082	0.050
16564	0.479	1.262	0.467	0.518	1.491	0.371	0.516	1.498	0.296
16565	0.511	1.464	0.296	0.465	1.152	0.491	0.518	1.514	0.431
16566	0.097	-1.181	0.296	0.313	0.172	0.273	0.184	-0.625	0.467
16567	0.484	1.295	0.512	0.459	1.114	0.273	0.500	1.397	0.431
16568	0.511	1.464	0.481	0.474	1.208	0.193	0.500	1.397	0.431
16569	0.500	1.397	0.512	0.474	1.208	0.273	0.500	1.397	0.385
16570	0.500	1.397	0.224	0.439	0.982	0.501	0.500	1.397	0.224
16571	0.261	-0.137	0.512	0.152	-0.863	0.508	0.187	-0.608	0.097
16572	0.261	-0.137	0.467	0.152	-0.863	0.462	0.145	-0.878	0.508
16573	0.261	-0.137	0.262	0.152	-0.863	0.193	0.187	-0.608	0.508
16574	0.500	1.397	0.097	0.509	1.434	0.501	0.505	1.430	0.508
16575	0.492	1.346	0.467	0.500	1.378	0.149	0.432	0.958	0.224
16576	0.500	1.397	0.050	0.477	1.227	0.398	0.505	1.430	0.328
16577	0.505	1.430	0.050	0.500	1.378	0.491	0.492	1.346	0.450
16578	0.263	-0.120	0.450	0.196	-0.581	0.193	0.097	-1.181	0.224
16579	0.500	1.397	0.185	0.509	1.434	0.462	0.492	1.346	0.467
16580	0.261	-0.137	0.513	0.272	-0.091	0.422	0.097	-1.181	0.513
16581	0.355	0.470	0.492	0.272	-0.091	0.478	0.050	-1.485	0.142
16582	0.297	0.099	0.512	0.307	0.135	0.508	0.050	-1.485	0.501
16583	0.142	-0.895	0.512	0.272	-0.091	0.512	0.142	-0.895	0.467
16584	0.505	1.430	0.512	0.424	0.888	0.491	0.484	1.295	0.481
16585	0.511	1.464	0.467	0.368	0.530	0.501	0.505	1.430	0.358
16586	0.513	1.481	0.450	0.494	1.340	0.273	0.495	1.363	0.508
16587	0.297	0.099	0.501	0.272	-0.091	0.462	0.000	?	0.328
16588	0.224	-0.373	0.512	0.272	-0.091	0.512	0.050	-1.485	0.142

16589	0.261	-0.137	0.512	0.193	-0.600	0.512	0.097	-1.181	0.501
16590	0.142	-0.895	0.328	0.275	-0.073	0.371	0.142	-0.895	0.450
16591	0.447	1.060	0.409	0.503	1.396	0.478	0.503	1.413	0.450
16592	0.492	1.346	0.385	0.509	1.434	0.193	0.474	1.228	0.296
16593	0.450	1.076	0.512	0.465	1.152	0.309	0.503	1.413	0.296
16594	0.226	-0.356	0.508	0.193	-0.600	0.193	0.226	-0.356	0.512
16595	0.432	0.958	0.513	0.500	1.378	0.235	0.492	1.346	0.296
16596	0.187	-0.608	0.050	0.149	-0.882	0.398	0.226	-0.356	0.492
16597	0.453	1.093	0.512	0.398	0.718	0.444	0.497	1.380	0.450
16598	0.482	1.279	0.185	0.494	1.340	0.501	0.476	1.245	0.501
16599	0.358	0.487	0.512	0.193	-0.600	0.444	0.142	-0.895	0.262
16600	0.408	0.807	0.508	0.462	1.133	0.309	0.482	1.279	0.358
16601	0.505	1.430	0.508	0.424	0.888	0.398	0.511	1.464	0.501
16602	0.497	1.380	0.512	0.480	1.246	0.193	0.479	1.262	0.467
16603	0.500	1.397	0.296	0.509	1.434	0.512	0.511	1.464	0.467
16604	0.476	1.245	0.358	0.515	1.472	0.444	0.479	1.262	0.097
16605	0.500	1.397	0.097	0.494	1.340	0.193	0.482	1.279	0.097
16606	0.463	1.161	0.050	0.506	1.415	0.273	0.487	1.312	0.512
16607	0.300	0.116	0.492	0.272	-0.091	0.508	0.297	0.099	0.224
16608	0.511	1.464	0.224	0.462	1.133	0.341	0.429	0.942	0.262
16609	0.432	0.958	0.492	0.512	1.453	0.102	0.497	1.380	0.513
16610	0.453	1.093	0.492	0.512	1.453	0.341	0.497	1.380	0.508
16611	0.097	-1.181	0.508	0.377	0.587	0.501	0.326	0.285	0.467
16612	0.487	1.312	0.508	0.480	1.246	0.444	0.450	1.076	0.142
16613	0.184	-0.625	0.508	0.196	-0.581	0.398	0.261	-0.137	0.467
16614	0.097	-1.181	0.512	0.310	0.153	0.309	0.361	0.504	0.467
16615	0.184	-0.625	0.431	0.196	-0.581	0.491	0.224	-0.373	0.262
16616	0.224	-0.373	0.296	0.240	-0.299	0.444	0.261	-0.137	0.296
16617	0.497	1.380	0.512	0.398	0.718	0.478	0.495	1.363	0.467
16618	0.500	1.397	0.328	0.447	1.039	0.491	0.447	1.060	0.409
16619	0.516	1.498	0.142	0.500	1.378	0.235	0.358	0.487	0.358
16620	0.505	1.430	0.262	0.442	1.001	0.273	0.492	1.346	0.481
16621	0.097	-1.181	0.513	0.310	0.153	0.309	0.142	-0.895	0.262
16622	0.358	0.487	0.481	0.404	0.756	0.149	0.184	-0.625	0.467
16623	0.263	-0.120	0.508	0.196	-0.581	0.398	0.184	-0.625	0.508
16624	0.463	1.161	0.508	0.509	1.434	0.398	0.511	1.464	0.481
16625	0.395	0.723	0.501	0.442	1.001	0.508	0.295	0.082	0.481
16626	0.097	-1.181	0.262	0.193	-0.600	0.422	0.508	1.447	0.481
16627	0.224	-0.373	0.492	0.275	-0.073	0.501	0.224	-0.373	0.492
16628	0.497	1.380	0.513	0.509	1.434	0.478	0.503	1.413	0.467
16629	0.358	0.487	0.513	0.342	0.361	0.501	0.297	0.099	0.450
16630	0.261	-0.137	0.512	0.105	-1.165	0.501	0.224	-0.373	0.513
16631	0.184	-0.625	0.512	0.342	0.361	0.491	0.050	-1.485	0.513
16632	0.463	1.161	0.224	0.506	1.415	0.444	0.468	1.194	0.512
16633	0.505	1.430	0.224	0.444	1.020	0.053	0.482	1.279	0.512
16634	0.508	1.447	0.508	0.401	0.737	0.273	0.479	1.262	0.513
16635	0.184	-0.625	0.467	0.310	0.153	0.444	0.184	-0.625	0.385
16636	0.482	1.279	0.185	0.485	1.283	0.309	0.487	1.312	0.501
16637	0.187	-0.608	0.501	0.444	1.020	0.478	0.224	-0.373	0.501
16638	0.497	1.380	0.481	0.480	1.246	0.309	0.429	0.942	0.328
16639	0.497	1.380	0.185	0.491	1.321	0.235	0.429	0.942	0.385
16640	0.413	0.841	0.328	0.237	-0.317	0.235	0.326	0.285	0.262
16641	0.224	-0.373	0.492	0.272	-0.091	0.422	0.184	-0.625	0.481
16642	0.142	-0.895	0.450	0.307	0.135	0.501	0.297	0.099	0.409
16643	0.224	-0.373	0.262	0.149	-0.882	0.273	0.266	-0.103	0.409
16644	0.142	-0.895	0.185	0.401	0.737	0.512	0.261	-0.137	0.492
16645	0.142	-0.895	0.467	0.401	0.737	0.235	0.261	-0.137	0.050
16646	0.513	1.481	0.224	0.512	1.453	0.462	0.482	1.279	0.142
16647	0.500	1.397	0.501	0.421	0.869	0.309	0.508	1.447	0.185
16648	0.447	1.060	0.409	0.500	1.378	0.478	0.489	1.329	0.097
16649	0.513	1.481	0.409	0.480	1.246	0.235	0.445	1.043	0.508

16650	0.495	1.363	0.050	0.500	1.378	0.149	0.495	1.363	0.142
16651	0.466	1.178	0.262	0.509	1.434	0.512	0.463	1.161	0.512
16652	0.511	1.464	0.431	0.494	1.340	0.193	0.389	0.689	0.512
16653	0.450	1.076	0.481	0.444	1.020	0.501	0.447	1.060	0.450
16654	0.097	-1.181	0.224	0.053	-1.504	0.501	0.361	0.504	0.358
16655	0.097	-1.181	0.501	0.275	-0.073	0.422	0.326	0.285	0.450
16656	0.492	1.346	0.467	0.442	1.001	0.462	0.466	1.178	0.450
16657	0.000	?	0.431	0.310	0.153	0.193	0.261	-0.137	0.385
16658	0.500	1.397	0.185	0.482	1.265	0.512	0.503	1.413	0.097
16659	0.142	-0.895	0.185	0.310	0.153	0.193	0.329	0.301	0.501
16660	0.226	-0.356	0.262	0.234	-0.336	0.478	0.329	0.301	0.097
16661	0.142	-0.895	0.142	0.234	-0.336	0.512	0.261	-0.137	0.097
16662	0.097	-1.181	0.501	0.424	0.888	0.512	0.361	0.504	0.262
16663	0.487	1.312	0.050	0.503	1.396	0.444	0.503	1.413	0.431
16664	0.184	-0.625	0.050	0.272	-0.091	0.512	0.142	-0.895	0.508
16665	0.511	1.464	0.501	0.512	1.453	0.462	0.463	1.161	0.492
16666	0.142	-0.895	0.512	0.237	-0.317	0.462	0.184	-0.625	0.512
16667	0.482	1.279	0.512	0.465	1.152	0.102	0.497	1.380	0.467
16668	0.332	0.318	0.050	0.310	0.153	0.501	0.224	-0.373	0.142
16669	0.450	1.076	0.450	0.480	1.246	0.398	0.482	1.279	0.501
16670	0.295	0.082	0.224	0.237	-0.317	0.514	0.224	-0.373	0.513
16671	0.224	-0.373	0.450	0.149	-0.882	0.235	0.224	-0.373	0.501
16672	0.484	1.295	0.431	0.518	1.491	0.491	0.426	0.925	0.508
16673	0.263	-0.120	0.431	0.193	-0.600	0.149	0.266	-0.103	0.481
16674	0.508	1.447	0.224	0.497	1.359	0.478	0.468	1.194	0.097
16675	0.508	1.447	0.467	0.497	1.359	0.309	0.468	1.194	0.185
16676	0.450	1.076	0.262	0.421	0.869	0.273	0.484	1.295	0.409
16677	0.479	1.262	0.296	0.465	1.152	0.273	0.471	1.211	0.501
16678	0.184	-0.625	0.296	0.196	-0.581	0.235	0.184	-0.625	0.358
16679	0.508	1.447	0.450	0.515	1.472	0.508	0.445	1.043	0.492
16680	0.297	0.099	0.512	0.193	-0.600	0.341	0.224	-0.373	0.409
16681	0.468	1.194	0.513	0.477	1.227	0.341	0.511	1.464	0.328
16682	0.513	1.481	0.224	0.512	1.453	0.235	0.432	0.958	0.185
16683	0.513	1.481	0.508	0.512	1.453	0.193	0.384	0.655	0.508
16684	0.511	1.464	0.492	0.444	1.020	0.193	0.492	1.346	0.508
16685	0.513	1.481	0.492	0.512	1.453	0.501	0.432	0.958	0.185
16686	0.466	1.178	0.513	0.459	1.114	0.508	0.476	1.245	0.185
16687	0.503	1.413	0.142	0.459	1.114	0.508	0.476	1.245	0.513
16688	0.224	-0.373	0.431	0.102	-1.184	0.462	0.224	-0.373	0.142
16689	0.466	1.178	0.262	0.497	1.359	0.444	0.487	1.312	0.508
16690	0.518	1.514	0.262	0.395	0.700	0.462	0.432	0.958	0.467
16691	0.508	1.447	0.050	0.515	1.472	0.508	0.479	1.262	0.467
16692	0.184	-0.625	0.142	0.237	-0.317	0.491	0.142	-0.895	0.262
16693	0.466	1.178	0.262	0.485	1.283	0.371	0.505	1.430	0.358
16694	0.361	0.504	0.450	0.149	-0.882	0.371	0.224	-0.373	0.467
16695	0.476	1.245	0.409	0.480	1.246	0.398	0.505	1.430	0.467
16696	0.387	0.672	0.481	0.310	0.153	0.235	0.000	?	0.050
16697	0.387	0.672	0.492	0.275	-0.073	0.193	0.000	?	0.492
16698	0.387	0.672	0.481	0.275	-0.073	0.508	0.000	?	0.513
16699	0.297	0.099	0.492	0.234	-0.336	0.341	0.050	-1.485	0.513
16700	0.411	0.824	0.224	0.512	1.453	0.508	0.474	1.228	0.185
16701	0.224	-0.373	0.262	0.342	0.361	0.462	0.184	-0.625	0.185
16702	0.297	0.099	0.450	0.342	0.361	0.309	0.097	-1.181	0.224
16703	0.261	-0.137	0.185	0.237	-0.317	0.149	0.050	-1.485	0.185
16704	0.261	-0.137	0.224	0.196	-0.581	0.193	0.187	-0.608	0.508
16705	0.261	-0.137	0.409	0.196	-0.581	0.501	0.187	-0.608	0.328
16706	0.497	1.380	0.431	0.500	1.378	0.478	0.511	1.464	0.262
16707	0.476	1.245	0.501	0.509	1.434	0.501	0.505	1.430	0.508
16708	0.497	1.380	0.431	0.506	1.415	0.193	0.508	1.447	0.450
16709	0.434	0.975	0.185	0.462	1.133	0.149	0.511	1.464	0.513
16710	0.434	0.975	0.492	0.444	1.020	0.491	0.511	1.464	0.296

16711	0.434	0.975	0.142	0.462	1.133	0.371	0.511	1.464	0.501
16712	0.505	1.430	0.481	0.503	1.396	0.491	0.479	1.262	0.097
16713	0.461	1.144	0.185	0.491	1.321	0.501	0.466	1.178	0.450
16714	0.142	-0.895	0.501	0.374	0.568	0.235	0.184	-0.625	0.467
16715	0.142	-0.895	0.262	0.374	0.568	0.478	0.184	-0.625	0.512
16716	0.050	-1.485	0.467	0.398	0.718	0.462	0.142	-0.895	0.185
16717	0.300	0.116	0.512	0.234	-0.336	0.491	0.050	-1.485	0.501
16718	0.142	-0.895	0.467	0.196	-0.581	0.341	0.297	0.099	0.262
16719	0.432	0.958	0.501	0.509	1.434	0.398	0.432	0.958	0.385
16720	0.503	1.413	0.185	0.342	0.361	0.193	0.461	1.144	0.224
16721	0.476	1.245	0.185	0.512	1.453	0.193	0.434	0.975	0.508
16722	0.463	1.161	0.512	0.459	1.114	0.491	0.432	0.958	0.492
16723	0.329	0.301	0.481	0.307	0.135	0.491	0.389	0.689	0.481
16724	0.224	-0.373	0.481	0.149	-0.882	0.491	0.226	-0.356	0.224
16725	0.261	-0.137	0.492	0.193	-0.600	0.309	0.097	-1.181	0.481
16726	0.484	1.295	0.501	0.506	1.415	0.422	0.505	1.430	0.513
16727	0.505	1.430	0.296	0.482	1.265	0.422	0.505	1.430	0.513
16728	0.495	1.363	0.450	0.503	1.396	0.309	0.508	1.447	0.467
16729	0.261	-0.137	0.185	0.193	-0.600	0.512	0.224	-0.373	0.328
16730	0.326	0.285	0.328	0.149	-0.882	0.512	0.326	0.285	0.097
16731	0.408	0.807	0.328	0.491	1.321	0.501	0.445	1.043	0.185
16732	0.329	0.301	0.481	0.371	0.549	0.149	0.224	-0.373	0.185
16733	0.453	1.093	0.224	0.491	1.321	0.508	0.468	1.194	0.185
16734	0.482	1.279	0.224	0.500	1.378	0.398	0.505	1.430	0.097
16735	0.226	-0.356	0.492	0.234	-0.336	0.512	0.142	-0.895	0.481
16736	0.453	1.093	0.501	0.477	1.227	0.512	0.497	1.380	0.450
16737	0.503	1.413	0.508	0.462	1.133	0.422	0.468	1.194	0.467
16738	0.484	1.295	0.512	0.488	1.302	0.422	0.482	1.279	0.513
16739	0.097	-1.181	0.328	0.345	0.379	0.508	0.355	0.470	0.512
16740	0.500	1.397	0.450	0.395	0.700	0.193	0.508	1.447	0.450
16741	0.187	-0.608	0.142	0.196	-0.581	0.235	0.329	0.301	0.224
16742	0.358	0.487	0.358	0.193	-0.600	0.478	0.142	-0.895	0.185
16743	0.468	1.194	0.358	0.491	1.321	0.462	0.500	1.397	0.467
16744	0.511	1.464	0.224	0.494	1.340	0.444	0.450	1.076	0.097
16745	0.511	1.464	0.185	0.494	1.340	0.235	0.450	1.076	0.185
16746	0.097	-1.181	0.328	0.313	0.172	0.512	0.295	0.082	0.262
16747	0.226	-0.356	0.508	0.424	0.888	0.478	0.295	0.082	0.467
16748	0.516	1.498	0.508	0.427	0.907	0.149	0.508	1.447	0.450
16749	0.050	-1.485	0.185	0.307	0.135	0.193	0.295	0.082	0.501
16750	0.503	1.413	0.431	0.512	1.453	0.444	0.492	1.346	0.142
16751	0.489	1.329	0.508	0.515	1.472	0.193	0.450	1.076	0.450
16752	0.511	1.464	0.508	0.497	1.359	0.149	0.497	1.380	0.224
16753	0.297	0.099	0.224	0.149	-0.882	0.478	0.261	-0.137	0.501
16754	0.447	1.060	0.262	0.509	1.434	0.102	0.358	0.487	0.185
16755	0.505	1.430	0.492	0.398	0.718	0.514	0.497	1.380	0.492
16756	0.482	1.279	0.262	0.518	1.491	0.444	0.468	1.194	0.142
16757	0.482	1.279	0.262	0.518	1.491	0.501	0.468	1.194	0.481
16758	0.097	-1.181	0.262	0.424	0.888	0.501	0.097	-1.181	0.512
16759	0.000	?	0.501	0.424	0.888	0.462	0.097	-1.181	0.512
16760	0.463	1.161	0.358	0.503	1.396	0.501	0.513	1.481	0.481
16761	0.297	0.099	0.358	0.196	-0.581	0.491	0.226	-0.356	0.142
16762	0.297	0.099	0.358	0.234	-0.336	0.512	0.266	-0.103	0.328
16763	0.511	1.464	0.296	0.474	1.208	0.514	0.508	1.447	0.450
16764	0.476	1.245	0.296	0.462	1.133	0.462	0.505	1.430	0.481
16765	0.513	1.481	0.508	0.447	1.039	0.508	0.471	1.211	0.467
16766	0.224	-0.373	0.262	0.237	-0.317	0.501	0.145	-0.878	0.431
16767	0.508	1.447	0.512	0.515	1.472	0.501	0.463	1.161	0.481
16768	0.461	1.144	0.501	0.474	1.208	0.512	0.468	1.194	0.224
16769	0.261	-0.137	0.508	0.152	-0.863	0.501	0.263	-0.120	0.512
16770	0.097	-1.181	0.328	0.196	-0.581	0.508	0.295	0.082	0.262
16771	0.461	1.144	0.508	0.450	1.057	0.193	0.466	1.178	0.097

16772	0.050	−1.485	0.492	0.196	−0.581	0.508	0.405	0.790	0.097
16773	0.050	−1.485	0.492	0.152	−0.863	0.235	0.355	0.470	0.481
16774	0.445	1.043	0.185	0.485	1.283	0.514	0.476	1.245	0.185
16775	0.184	−0.625	0.512	0.105	−1.165	0.512	0.263	−0.120	0.142
16776	0.513	1.481	0.513	0.509	1.434	0.512	0.461	1.144	0.512
16777	0.497	1.380	0.185	0.444	1.020	0.512	0.511	1.464	0.481
16778	0.508	1.447	0.262	0.497	1.359	0.193	0.482	1.279	0.501
16779	0.508	1.447	0.450	0.497	1.359	0.235	0.482	1.279	0.501
16780	0.508	1.447	0.358	0.459	1.114	0.273	0.482	1.279	0.050
16781	0.466	1.178	0.142	0.500	1.378	0.398	0.484	1.295	0.450
16782	0.445	1.043	0.097	0.491	1.321	0.514	0.466	1.178	0.296
16783	0.503	1.413	0.328	0.509	1.434	0.235	0.455	1.110	0.224
16784	0.503	1.413	0.431	0.512	1.453	0.462	0.516	1.498	0.224
16785	0.503	1.413	0.185	0.459	1.114	0.462	0.516	1.498	0.224
16786	0.329	0.301	0.431	0.512	1.453	0.514	0.511	1.464	0.050
16787	0.408	0.807	0.481	0.506	1.415	0.501	0.511	1.464	0.142
16788	0.384	0.655	0.262	0.506	1.415	0.501	0.511	1.464	0.450
16789	0.508	1.447	0.142	0.512	1.453	0.501	0.387	0.672	0.481
16790	0.497	1.380	0.224	0.506	1.415	0.341	0.503	1.413	0.185
16791	0.503	1.413	0.185	0.512	1.453	0.235	0.505	1.430	0.481
16792	0.050	−1.485	0.142	0.193	−0.600	0.462	0.329	0.301	0.431
16793	0.500	1.397	0.142	0.509	1.434	0.149	0.387	0.672	0.467
16794	0.184	−0.625	0.185	0.234	−0.336	0.309	0.266	−0.103	0.050
16795	0.500	1.397	0.262	0.515	1.472	0.371	0.487	1.312	0.050
16796	0.511	1.464	0.358	0.509	1.434	0.512	0.405	0.790	0.450
16797	0.511	1.464	0.224	0.509	1.434	0.512	0.405	0.790	0.185
16798	0.508	1.447	0.296	0.512	1.453	0.508	0.489	1.329	0.185
16799	0.295	0.082	0.467	0.193	−0.600	0.501	0.050	−1.485	0.409
16800	0.361	0.504	0.328	0.237	−0.317	0.478	0.142	−0.895	0.450
16801	0.097	−1.181	0.328	0.275	−0.073	0.235	0.187	−0.608	0.185
16802	0.050	−1.485	0.508	0.401	0.737	0.491	0.097	−1.181	0.097
16803	0.487	1.312	0.450	0.512	1.453	0.444	0.505	1.430	0.097
16804	0.224	−0.373	0.508	0.240	−0.299	0.512	0.142	−0.895	0.185
16805	0.489	1.329	0.501	0.471	1.189	0.501	0.511	1.464	0.097
16806	0.489	1.329	0.224	0.471	1.189	0.462	0.511	1.464	0.450
16807	0.503	1.413	0.142	0.515	1.472	0.341	0.447	1.060	0.097
16808	0.503	1.413	0.224	0.503	1.396	0.149	0.355	0.470	0.467
16809	0.503	1.413	0.224	0.503	1.396	0.514	0.447	1.060	0.501
16810	0.505	1.430	0.512	0.503	1.396	0.491	0.447	1.060	0.508
16811	0.432	0.958	0.185	0.339	0.342	0.149	0.387	0.672	0.512
16812	0.297	0.099	0.467	0.234	−0.336	0.478	0.097	−1.181	0.385
16813	0.505	1.430	0.142	0.459	1.114	0.491	0.500	1.397	0.467
16814	0.332	0.318	0.467	0.149	−0.882	0.102	0.097	−1.181	0.501
16815	0.142	−0.895	0.513	0.310	0.153	0.444	0.097	−1.181	0.185
16816	0.263	−0.120	0.409	0.377	0.587	0.462	0.261	−0.137	0.492
16817	0.513	1.481	0.358	0.515	1.472	0.501	0.426	0.925	0.431
16818	0.484	1.295	0.512	0.515	1.472	0.149	0.505	1.430	0.185
16819	0.508	1.447	0.296	0.506	1.415	0.193	0.484	1.295	0.224
16820	0.503	1.413	0.185	0.503	1.396	0.444	0.508	1.447	0.512
16821	0.503	1.413	0.501	0.474	1.208	0.193	0.461	1.144	0.409
16822	0.263	−0.120	0.262	0.234	−0.336	0.501	0.142	−0.895	0.224
16823	0.487	1.312	0.501	0.485	1.283	0.398	0.503	1.413	0.262
16824	0.516	1.498	0.262	0.444	1.020	0.398	0.508	1.447	0.097
16825	0.500	1.397	0.296	0.512	1.453	0.235	0.505	1.430	0.481
16826	0.500	1.397	0.450	0.500	1.378	0.193	0.513	1.481	0.185
16827	0.516	1.498	0.450	0.465	1.152	0.501	0.474	1.228	0.481
16828	0.224	−0.373	0.262	0.342	0.361	0.462	0.097	−1.181	0.501
16829	0.226	−0.356	0.513	0.149	−0.882	0.309	0.184	−0.625	0.097
16830	0.500	1.397	0.328	0.515	1.472	0.422	0.408	0.807	0.262
16831	0.463	1.161	0.262	0.491	1.321	0.501	0.497	1.380	0.097
16832	0.184	−0.625	0.328	0.152	−0.863	0.478	0.358	0.487	0.185

16833	0.500	1.397	0.224	0.482	1.265	0.235	0.487	1.312	0.142
16834	0.479	1.262	0.097	0.491	1.321	0.193	0.408	0.807	0.097
16835	0.184	-0.625	0.097	0.102	-1.184	0.341	0.329	0.301	0.224
16836	0.329	0.301	0.296	0.444	1.020	0.235	0.187	-0.608	0.185
16837	0.489	1.329	0.185	0.462	1.133	0.508	0.508	1.447	0.142
16838	0.445	1.043	0.296	0.503	1.396	0.341	0.505	1.430	0.512
16839	0.266	-0.103	0.262	0.149	-0.882	0.341	0.184	-0.625	0.185
16840	0.187	-0.608	0.185	0.193	-0.600	0.491	0.184	-0.625	0.185
16841	0.466	1.178	0.512	0.442	1.001	0.508	0.511	1.464	0.409
16842	0.224	-0.373	0.142	0.193	-0.600	0.491	0.145	-0.878	0.492
16843	0.495	1.363	0.512	0.497	1.359	0.193	0.508	1.447	0.492
16844	0.405	0.790	0.185	0.395	0.700	0.508	0.461	1.144	0.492
16845	0.405	0.790	0.512	0.395	0.700	0.273	0.461	1.144	0.097
16846	0.050	-1.485	0.097	0.234	-0.336	0.501	0.261	-0.137	0.296
16847	0.261	-0.137	0.512	0.196	-0.581	0.501	0.358	0.487	0.262
16848	0.426	0.925	0.450	0.506	1.415	0.512	0.466	1.178	0.224
16849	0.476	1.245	0.185	0.462	1.133	0.235	0.466	1.178	0.513
16850	0.224	-0.373	0.492	0.310	0.153	0.478	0.050	-1.485	0.296
16851	0.500	1.397	0.492	0.421	0.869	0.149	0.492	1.346	0.185
16852	0.468	1.194	0.358	0.503	1.396	0.371	0.511	1.464	0.492
16853	0.432	0.958	0.224	0.480	1.246	0.341	0.511	1.464	0.409
16854	0.184	-0.625	0.512	0.237	-0.317	0.462	0.184	-0.625	0.508
16855	0.184	-0.625	0.512	0.196	-0.581	0.501	0.184	-0.625	0.262
16856	0.263	-0.120	0.512	0.342	0.361	0.501	0.224	-0.373	0.513
16857	0.142	-0.895	0.185	0.240	-0.299	0.102	0.184	-0.625	0.512
16858	0.497	1.380	0.097	0.509	1.434	0.508	0.503	1.413	0.513
16859	0.050	-1.485	0.328	0.342	0.361	0.491	0.326	0.285	0.450
16860	0.050	-1.485	0.492	0.342	0.361	0.491	0.261	-0.137	0.224
16861	0.497	1.380	0.481	0.488	1.302	0.478	0.511	1.464	0.296
16862	0.505	1.430	0.467	0.506	1.415	0.053	0.455	1.110	0.481
16863	0.505	1.430	0.467	0.491	1.321	0.309	0.516	1.498	0.050
16864	0.050	-1.485	0.513	0.193	-0.600	0.273	0.297	0.099	0.512
16865	0.450	1.076	0.262	0.509	1.434	0.102	0.503	1.413	0.050
16866	0.224	-0.373	0.224	0.275	-0.073	0.341	0.097	-1.181	0.328
16867	0.453	1.093	0.492	0.506	1.415	0.309	0.445	1.043	0.481
16868	0.432	0.958	0.450	0.506	1.415	0.193	0.461	1.144	0.492
16869	0.426	0.925	0.262	0.520	1.509	0.478	0.511	1.464	0.050
16870	0.224	-0.373	0.409	0.234	-0.336	0.512	0.184	-0.625	0.467
16871	0.466	1.178	0.262	0.480	1.246	0.478	0.503	1.413	0.142
16872	0.263	-0.120	0.501	0.149	-0.882	0.462	0.263	-0.120	0.224
16873	0.300	0.116	0.224	0.377	0.587	0.422	0.382	0.638	0.328
16874	0.297	0.099	0.501	0.345	0.379	0.444	0.224	-0.373	0.142
16875	0.453	1.093	0.262	0.465	1.152	0.149	0.503	1.413	0.262
16876	0.508	1.447	0.481	0.506	1.415	0.422	0.489	1.329	0.262
16877	0.511	1.464	0.450	0.500	1.378	0.462	0.474	1.228	0.296
16878	0.224	-0.373	0.296	0.102	-1.184	0.341	0.224	-0.373	0.185
16879	0.508	1.447	0.224	0.509	1.434	0.149	0.474	1.228	0.142
16880	0.489	1.329	0.508	0.491	1.321	0.309	0.508	1.447	0.328
16881	0.489	1.329	0.185	0.491	1.321	0.491	0.508	1.447	0.050
16882	0.508	1.447	0.501	0.477	1.227	0.478	0.466	1.178	0.097
16883	0.145	-0.878	0.508	0.053	-1.504	0.501	0.329	0.301	0.262
16884	0.429	0.942	0.481	0.310	0.153	0.273	0.097	-1.181	0.467
16885	0.261	-0.137	0.481	0.272	-0.091	0.491	0.000	?	0.296
16886	0.261	-0.137	0.224	0.102	-1.184	0.273	0.187	-0.608	0.431
16887	0.050	-1.485	0.224	0.345	0.379	0.514	0.184	-0.625	0.328
16888	0.142	-0.895	0.142	0.307	0.135	0.193	0.184	-0.625	0.431
16889	0.261	-0.137	0.492	0.193	-0.600	0.501	0.097	-1.181	0.142
16890	0.447	1.060	0.142	0.477	1.227	0.193	0.479	1.262	0.467
16891	0.408	0.807	0.097	0.512	1.453	0.514	0.447	1.060	0.450
16892	0.476	1.245	0.328	0.477	1.227	0.444	0.466	1.178	0.224
16893	0.495	1.363	0.508	0.465	1.152	0.444	0.513	1.481	0.501

16894	0.476	1.245	0.481	0.424	0.888	0.491	0.508	1.447	0.501
16895	0.497	1.380	0.512	0.439	0.982	0.235	0.453	1.093	0.142
16896	0.224	-0.373	0.501	0.149	-0.882	0.235	0.226	-0.356	0.185
16897	0.261	-0.137	0.501	0.424	0.888	0.514	0.184	-0.625	0.481
16898	0.447	1.060	0.512	0.468	1.170	0.508	0.468	1.194	0.481
16899	0.184	-0.625	0.512	0.339	0.342	0.501	0.097	-1.181	0.467
16900	0.226	-0.356	0.481	0.149	-0.882	0.512	0.184	-0.625	0.185
16901	0.411	0.824	0.501	0.310	0.153	0.508	0.261	-0.137	0.296
16902	0.432	0.958	0.481	0.488	1.302	0.235	0.468	1.194	0.328
16903	0.503	1.413	0.481	0.471	1.189	0.462	0.450	1.076	0.492
16904	0.432	0.958	0.328	0.503	1.396	0.193	0.503	1.413	0.481
16905	0.184	-0.625	0.328	0.272	-0.091	0.102	0.142	-0.895	0.508
16906	0.487	1.312	0.185	0.494	1.340	0.102	0.447	1.060	0.508
16907	0.142	-0.895	0.508	0.275	-0.073	0.501	0.224	-0.373	0.450
16908	0.474	1.228	0.508	0.515	1.472	0.235	0.497	1.380	0.142
16909	0.187	-0.608	0.224	0.196	-0.581	0.193	0.184	-0.625	0.224
16910	0.503	1.413	0.508	0.494	1.340	0.398	0.489	1.329	0.431
16911	0.266	-0.103	0.501	0.193	-0.600	0.491	0.142	-0.895	0.513
16912	0.468	1.194	0.512	0.512	1.453	0.501	0.474	1.228	0.296
16913	0.511	1.464	0.185	0.447	1.039	0.422	0.503	1.413	0.481
16914	0.463	1.161	0.492	0.444	1.020	0.235	0.505	1.430	0.262
16915	0.500	1.397	0.492	0.497	1.359	0.508	0.476	1.245	0.513
16916	0.184	-0.625	0.450	0.237	-0.317	0.102	0.142	-0.895	0.185
16917	0.184	-0.625	0.328	0.237	-0.317	0.235	0.332	0.318	0.492
16918	0.511	1.464	0.097	0.515	1.472	0.235	0.450	1.076	0.185
16919	0.479	1.262	0.262	0.506	1.415	0.149	0.479	1.262	0.467
16920	0.479	1.262	0.512	0.500	1.378	0.309	0.463	1.161	0.501
16921	0.495	1.363	0.501	0.518	1.491	0.371	0.432	0.958	0.262
16922	0.500	1.397	0.481	0.509	1.434	0.501	0.484	1.295	0.431
16923	0.297	0.099	0.492	0.237	-0.317	0.462	0.224	-0.373	0.492
16924	0.450	1.076	0.296	0.468	1.170	0.273	0.508	1.447	0.262
16925	0.184	-0.625	0.142	0.193	-0.600	0.512	0.261	-0.137	0.467
16926	0.329	0.301	0.512	0.102	-1.184	0.491	0.097	-1.181	0.467
16927	0.329	0.301	0.513	0.102	-1.184	0.462	0.097	-1.181	0.492
16928	0.482	1.279	0.508	0.500	1.378	0.273	0.476	1.245	0.385
16929	0.226	-0.356	0.508	0.234	-0.336	0.235	0.184	-0.625	0.185
16930	0.224	-0.373	0.050	0.196	-0.581	0.444	0.142	-0.895	0.185
16931	0.495	1.363	0.508	0.398	0.718	0.193	0.500	1.397	0.262
16932	0.495	1.363	0.185	0.491	1.321	0.309	0.484	1.295	0.450
16933	0.500	1.397	0.481	0.503	1.396	0.273	0.503	1.413	0.224
16934	0.511	1.464	0.481	0.421	0.869	0.508	0.500	1.397	0.467
16935	0.329	0.301	0.328	0.237	-0.317	0.149	0.050	-1.485	0.492
16936	0.445	1.043	0.467	0.506	1.415	0.053	0.450	1.076	0.513
16937	0.142	-0.895	0.481	0.102	-1.184	0.053	0.300	0.116	0.512
16938	0.363	0.520	0.328	0.234	-0.336	0.273	0.224	-0.373	0.501
16939	0.363	0.520	0.501	0.234	-0.336	0.193	0.224	-0.373	0.512
16940	0.226	-0.356	0.501	0.149	-0.882	0.512	0.226	-0.356	0.508
16941	0.184	-0.625	0.501	0.310	0.153	0.371	0.050	-1.485	0.501
16942	0.332	0.318	0.508	0.368	0.530	0.514	0.142	-0.895	0.467
16943	0.508	1.447	0.142	0.497	1.359	0.398	0.445	1.043	0.512
16944	0.503	1.413	0.185	0.462	1.133	0.478	0.476	1.245	0.513
16945	0.184	-0.625	0.513	0.275	-0.073	0.508	0.184	-0.625	0.512
16946	0.434	0.975	0.501	0.509	1.434	0.508	0.479	1.262	0.512
16947	0.505	1.430	0.481	0.485	1.283	0.478	0.429	0.942	0.224
16948	0.503	1.413	0.501	0.468	1.170	0.512	0.466	1.178	0.224
16949	0.226	-0.356	0.050	0.275	-0.073	0.273	0.050	-1.485	0.142
16950	0.261	-0.137	0.050	0.234	-0.336	0.309	0.050	-1.485	0.492
16951	0.492	1.346	0.512	0.442	1.001	0.478	0.450	1.076	0.492
16952	0.261	-0.137	0.501	0.193	-0.600	0.193	0.184	-0.625	0.224
16953	0.261	-0.137	0.492	0.310	0.153	0.309	0.184	-0.625	0.508
16954	0.263	-0.120	0.512	0.275	-0.073	0.501	0.408	0.807	0.501

16955	0.503	1.413	0.142	0.500	1.378	0.273	0.447	1.060	0.492
16956	0.358	0.487	0.467	0.149	−0.882	0.398	0.187	−0.608	0.185
16957	0.358	0.487	0.467	0.053	−1.504	0.193	0.097	−1.181	0.431
16958	0.358	0.487	0.467	0.053	−1.504	0.102	0.097	−1.181	0.431
16959	0.295	0.082	0.467	0.272	−0.091	0.491	0.184	−0.625	0.481
16960	0.295	0.082	0.481	0.193	−0.600	0.273	0.097	−1.181	0.262
16961	0.505	1.430	0.385	0.509	1.434	0.491	0.447	1.060	0.262
16962	0.261	−0.137	0.097	0.374	0.568	0.422	0.097	−1.181	0.050
16963	0.500	1.397	0.501	0.520	1.509	0.235	0.463	1.161	0.467
16964	0.497	1.380	0.512	0.401	0.737	0.193	0.503	1.413	0.512
16965	0.511	1.464	0.385	0.477	1.227	0.371	0.497	1.380	0.492
16966	0.326	0.285	0.513	0.506	1.415	0.309	0.508	1.447	0.501
16967	0.500	1.397	0.492	0.509	1.434	0.309	0.384	0.655	0.097
16968	0.487	1.312	0.492	0.480	1.246	0.309	0.466	1.178	0.185
16969	0.489	1.329	0.296	0.512	1.453	0.235	0.497	1.380	0.450
16970	0.184	−0.625	0.296	0.272	−0.091	0.273	0.184	−0.625	0.481
16971	0.513	1.481	0.450	0.313	0.172	0.193	0.492	1.346	0.467
16972	0.508	1.447	0.262	0.474	1.208	0.235	0.434	0.975	0.467
16973	0.184	−0.625	0.467	0.196	−0.581	0.371	0.184	−0.625	0.328
16974	0.263	−0.120	0.512	0.307	0.135	0.491	0.224	−0.373	0.492
16975	0.450	1.076	0.431	0.497	1.359	0.193	0.516	1.498	0.097
16976	0.361	0.504	0.097	0.272	−0.091	0.193	0.408	0.807	0.481
16977	0.142	−0.895	0.224	0.404	0.756	0.491	0.224	−0.373	0.481
16978	0.097	−1.181	0.481	0.193	−0.600	0.478	0.263	−0.120	0.142
16979	0.332	0.318	0.262	0.102	−1.184	0.491	0.097	−1.181	0.512
16980	0.429	0.942	0.224	0.491	1.321	0.491	0.476	1.245	0.508
16981	0.187	−0.608	0.492	0.272	−0.091	0.235	0.184	−0.625	0.097
16982	0.429	0.942	0.512	0.491	1.321	0.149	0.476	1.245	0.492
16983	0.482	1.279	0.512	0.421	0.869	0.193	0.495	1.363	0.492
16984	0.261	−0.137	0.508	0.234	−0.336	0.149	0.097	−1.181	0.512
16985	0.142	−0.895	0.097	0.196	−0.581	0.501	0.261	−0.137	0.513
16986	0.224	−0.373	0.492	0.374	0.568	0.053	0.097	−1.181	0.262
16987	0.184	−0.625	0.492	0.310	0.153	0.422	0.184	−0.625	0.142
16988	0.142	−0.895	0.296	0.307	0.135	0.491	0.142	−0.895	0.501
16989	0.142	−0.895	0.508	0.310	0.153	0.102	0.097	−1.181	0.508
16990	0.184	−0.625	0.431	0.234	−0.336	0.512	0.224	−0.373	0.492
16991	0.263	−0.120	0.097	0.272	−0.091	0.309	0.000	?	0.508
16992	0.361	0.504	0.262	0.193	−0.600	0.398	0.184	−0.625	0.328
16993	0.224	−0.373	0.185	0.234	−0.336	0.398	0.142	−0.895	0.224
16994	0.295	0.082	0.296	0.374	0.568	0.491	0.000	?	0.508
16995	0.463	1.161	0.185	0.491	1.321	0.501	0.505	1.430	0.501
16996	0.329	0.301	0.185	0.193	−0.600	0.273	0.187	−0.608	0.481
16997	0.329	0.301	0.224	0.193	−0.600	0.309	0.187	−0.608	0.501
16998	0.511	1.464	0.358	0.485	1.283	0.478	0.405	0.790	0.185
16999	0.447	1.060	0.481	0.471	1.189	0.235	0.489	1.329	0.513
17000	0.505	1.430	0.481	0.485	1.283	0.422	0.489	1.329	0.513
17001	0.500	1.397	0.508	0.485	1.283	0.235	0.489	1.329	0.513
17002	0.226	−0.356	0.142	0.237	−0.317	0.235	0.097	−1.181	0.513
17003	0.142	−0.895	0.142	0.149	−0.882	0.501	0.297	0.099	0.513
17004	0.226	−0.356	0.385	0.193	−0.600	0.491	0.261	−0.137	0.097
17005	0.226	−0.356	0.512	0.149	−0.882	0.235	0.224	−0.373	0.328
17006	0.511	1.464	0.409	0.497	1.359	0.501	0.508	1.447	0.512
17007	0.187	−0.608	0.142	0.053	−1.504	0.193	0.297	0.099	0.481
17008	0.000	?	0.185	0.424	0.888	0.102	0.184	−0.625	0.050
17009	0.463	1.161	0.296	0.488	1.302	0.235	0.497	1.380	0.492
17010	0.142	−0.895	0.262	0.102	−1.184	0.273	0.413	0.841	0.501
17011	0.463	1.161	0.262	0.512	1.453	0.235	0.505	1.430	0.492
17012	0.000	?	0.185	0.310	0.153	0.235	0.263	−0.120	0.262
17013	0.503	1.413	0.431	0.398	0.718	0.102	0.513	1.481	0.262
17014	0.411	0.824	0.296	0.401	0.737	0.273	0.511	1.464	0.450
17015	0.361	0.504	0.481	0.494	1.340	0.371	0.508	1.447	0.185

17016	0.513	1.481	0.142	0.503	1.396	0.193	0.445	1.043	0.501
17017	0.297	0.099	0.262	0.272	-0.091	0.273	0.224	-0.373	0.467
17018	0.187	-0.608	0.481	0.307	0.135	0.193	0.300	0.116	0.492
17019	0.495	1.363	0.481	0.485	1.283	0.193	0.476	1.245	0.262
17020	0.263	-0.120	0.224	0.237	-0.317	0.444	0.050	-1.485	0.467
17021	0.503	1.413	0.492	0.424	0.888	0.341	0.511	1.464	0.185
17022	0.263	-0.120	0.358	0.237	-0.317	0.491	0.050	-1.485	0.142
17023	0.295	0.082	0.492	0.237	-0.317	0.341	0.329	0.301	0.450
17024	0.450	1.076	0.328	0.500	1.378	0.422	0.482	1.279	0.512
17025	0.450	1.076	0.450	0.488	1.302	0.341	0.492	1.346	0.513
17026	0.263	-0.120	0.262	0.237	-0.317	0.501	0.050	-1.485	0.481
17027	0.508	1.447	0.481	0.497	1.359	0.508	0.468	1.194	0.296
17028	0.329	0.301	0.296	0.196	-0.581	0.193	0.142	-0.895	0.481
17029	0.263	-0.120	0.450	0.102	-1.184	0.478	0.224	-0.373	0.513
17030	0.329	0.301	0.050	0.234	-0.336	0.478	0.329	0.301	0.224
17031	0.226	-0.356	0.050	0.272	-0.091	0.193	0.142	-0.895	0.512
17032	0.097	-1.181	0.050	0.234	-0.336	0.235	0.263	-0.120	0.513
17033	0.097	-1.181	0.050	0.234	-0.336	0.512	0.263	-0.120	0.296
17034	0.295	0.082	0.050	0.102	-1.184	0.508	0.300	0.116	0.262
17035	0.184	-0.625	0.142	0.278	-0.054	0.478	0.184	-0.625	0.050
17036	0.000	?	0.508	0.374	0.568	0.193	0.142	-0.895	0.185
17037	0.000	?	0.262	0.196	-0.581	0.273	0.326	0.285	0.050
17038	0.295	0.082	0.513	0.272	-0.091	0.149	0.050	-1.485	0.296
17039	0.261	-0.137	0.508	0.193	-0.600	0.514	0.097	-1.181	0.142
17040	0.184	-0.625	0.512	0.193	-0.600	0.512	0.261	-0.137	0.185
17041	0.500	1.397	0.481	0.447	1.039	0.501	0.468	1.194	0.508
17042	0.142	-0.895	0.185	0.345	0.379	0.491	0.295	0.082	0.508
17043	0.511	1.464	0.142	0.491	1.321	0.512	0.432	0.958	0.481
17044	0.184	-0.625	0.512	0.342	0.361	0.149	0.326	0.285	0.185
17045	0.503	1.413	0.097	0.424	0.888	0.149	0.434	0.975	0.185
17046	0.100	-1.164	0.050	0.342	0.361	0.444	0.142	-0.895	0.050
17047	0.508	1.447	0.501	0.503	1.396	0.514	0.463	1.161	0.431
17048	0.450	1.076	0.185	0.512	1.453	0.398	0.453	1.093	0.185
17049	0.184	-0.625	0.142	0.193	-0.600	0.508	0.226	-0.356	0.262
17050	0.489	1.329	0.296	0.477	1.227	0.053	0.503	1.413	0.358
17051	0.489	1.329	0.097	0.477	1.227	0.478	0.503	1.413	0.224
17052	0.358	0.487	0.512	0.196	-0.581	0.149	0.145	-0.878	0.224
17053	0.229	-0.339	0.492	0.234	-0.336	0.508	0.184	-0.625	0.224
17054	0.511	1.464	0.492	0.509	1.434	0.371	0.482	1.279	0.185
17055	0.511	1.464	0.492	0.503	1.396	0.491	0.482	1.279	0.501
17056	0.516	1.498	0.467	0.480	1.246	0.444	0.466	1.178	0.262
17057	0.184	-0.625	0.467	0.196	-0.581	0.273	0.184	-0.625	0.508
17058	0.097	-1.181	0.512	0.275	-0.073	0.193	0.295	0.082	0.185
17059	0.329	0.301	0.450	0.149	-0.882	0.514	0.329	0.301	0.185
17060	0.489	1.329	0.185	0.512	1.453	0.193	0.487	1.312	0.512
17061	0.479	1.262	0.262	0.512	1.453	0.512	0.474	1.228	0.512
17062	0.466	1.178	0.142	0.500	1.378	0.512	0.500	1.397	0.385
17063	0.466	1.178	0.481	0.494	1.340	0.512	0.505	1.430	0.450
17064	0.505	1.430	0.512	0.512	1.453	0.422	0.450	1.076	0.262
17065	0.263	-0.120	0.450	0.149	-0.882	0.193	0.145	-0.878	0.431
17066	0.224	-0.373	0.431	0.149	-0.882	0.149	0.224	-0.373	0.097
17067	0.487	1.312	0.492	0.447	1.039	0.193	0.432	0.958	0.508
17068	0.445	1.043	0.097	0.518	1.491	0.508	0.513	1.481	0.385
17069	0.263	-0.120	0.142	0.401	0.737	0.341	0.297	0.099	0.409
17070	0.405	0.790	0.224	0.509	1.434	0.235	0.479	1.262	0.185
17071	0.263	-0.120	0.431	0.053	-1.504	0.478	0.261	-0.137	0.409
17072	0.492	1.346	0.508	0.480	1.246	0.512	0.511	1.464	0.185
17073	0.226	-0.356	0.467	0.152	-0.863	0.512	0.184	-0.625	0.224
17074	0.492	1.346	0.508	0.506	1.415	0.508	0.495	1.363	0.224
17075	0.261	-0.137	0.262	0.371	0.549	0.478	0.187	-0.608	0.185
17076	0.479	1.262	0.513	0.491	1.321	0.478	0.466	1.178	0.262

17077	0.450	1.076	0.431	0.442	1.001	0.508	0.503	1.413	0.224
17078	0.297	0.099	0.467	0.272	-0.091	0.512	0.266	-0.103	0.385
17079	0.224	-0.373	0.492	0.193	-0.600	0.508	0.434	0.975	0.142
17080	0.503	1.413	0.431	0.512	1.453	0.508	0.489	1.329	0.513
17081	0.184	-0.625	0.508	0.193	-0.600	0.514	0.263	-0.120	0.492
17082	0.500	1.397	0.508	0.515	1.472	0.514	0.468	1.194	0.450
17083	0.505	1.430	0.481	0.515	1.472	0.235	0.468	1.194	0.431
17084	0.482	1.279	0.492	0.515	1.472	0.309	0.495	1.363	0.050
17085	0.482	1.279	0.262	0.427	0.907	0.235	0.384	0.655	0.512
17086	0.226	-0.356	0.262	0.193	-0.600	0.514	0.187	-0.608	0.296
17087	0.224	-0.373	0.262	0.149	-0.882	0.514	0.184	-0.625	0.296
17088	0.145	-0.878	0.512	0.193	-0.600	0.273	0.263	-0.120	0.467
17089	0.489	1.329	0.512	0.512	1.453	0.478	0.450	1.076	0.185
17090	0.145	-0.878	0.467	0.342	0.361	0.422	0.226	-0.356	0.296
17091	0.097	-1.181	0.224	0.234	-0.336	0.478	0.466	1.178	0.262
17092	0.326	0.285	0.512	0.480	1.246	0.273	0.492	1.346	0.296
17093	0.508	1.447	0.142	0.509	1.434	0.501	0.511	1.464	0.513
17094	0.482	1.279	0.097	0.509	1.434	0.501	0.511	1.464	0.467
17095	0.513	1.481	0.142	0.503	1.396	0.398	0.500	1.397	0.467
17096	0.497	1.380	0.142	0.480	1.246	0.235	0.511	1.464	0.467
17097	0.497	1.380	0.450	0.480	1.246	0.235	0.505	1.430	0.467
17098	0.508	1.447	0.508	0.509	1.434	0.341	0.497	1.380	0.492
17099	0.505	1.430	0.512	0.509	1.434	0.512	0.466	1.178	0.512
17100	0.479	1.262	0.481	0.503	1.396	0.478	0.511	1.464	0.512
17101	0.500	1.397	0.481	0.503	1.396	0.514	0.511	1.464	0.142
17102	0.479	1.262	0.385	0.509	1.434	0.444	0.511	1.464	0.262
17103	0.479	1.262	0.431	0.509	1.434	0.193	0.511	1.464	0.142
17104	0.329	0.301	0.431	0.234	-0.336	0.273	0.226	-0.356	0.512
17105	0.326	0.285	0.501	0.310	0.153	0.501	0.224	-0.373	0.501
17106	0.184	-0.625	0.512	0.234	-0.336	0.444	0.142	-0.895	0.508
17107	0.511	1.464	0.185	0.520	1.509	0.444	0.489	1.329	0.492
17108	0.511	1.464	0.142	0.520	1.509	0.444	0.489	1.329	0.508
17109	0.224	-0.373	0.450	0.278	-0.054	0.273	0.226	-0.356	0.224
17110	0.503	1.413	0.481	0.480	1.246	0.444	0.503	1.413	0.224
17111	0.497	1.380	0.513	0.424	0.888	0.341	0.497	1.380	0.185
17112	0.508	1.447	0.508	0.480	1.246	0.491	0.487	1.312	0.224
17113	0.187	-0.608	0.508	0.275	-0.073	0.491	0.184	-0.625	0.481
17114	0.492	1.346	0.409	0.497	1.359	0.102	0.426	0.925	0.467
17115	0.492	1.346	0.501	0.497	1.359	0.508	0.426	0.925	0.501
17116	0.450	1.076	0.262	0.401	0.737	0.508	0.484	1.295	0.185
17117	0.326	0.285	0.467	0.234	-0.336	0.149	0.266	-0.103	0.409
17118	0.097	-1.181	0.050	0.237	-0.317	0.512	0.263	-0.120	0.512
17119	0.263	-0.120	0.224	0.342	0.361	0.512	0.050	-1.485	0.467
17120	0.508	1.447	0.501	0.512	1.453	0.491	0.461	1.144	0.508
17121	0.492	1.346	0.481	0.480	1.246	0.491	0.516	1.498	0.501
17122	0.476	1.245	0.492	0.515	1.472	0.273	0.487	1.312	0.492
17123	0.492	1.346	0.142	0.444	1.020	0.273	0.492	1.346	0.492
17124	0.297	0.099	0.185	0.193	-0.600	0.512	0.097	-1.181	0.328
17125	0.142	-0.895	0.492	0.275	-0.073	0.398	0.187	-0.608	0.508
17126	0.500	1.397	0.224	0.500	1.378	0.512	0.453	1.093	0.224
17127	0.518	1.514	0.467	0.442	1.001	0.491	0.474	1.228	0.097
17128	0.513	1.481	0.513	0.444	1.020	0.149	0.461	1.144	0.097
17129	0.513	1.481	0.492	0.444	1.020	0.309	0.461	1.144	0.409
17130	0.050	-1.485	0.142	0.275	-0.073	0.478	0.329	0.301	0.409
17131	0.508	1.447	0.262	0.442	1.001	0.444	0.487	1.312	0.467
17132	0.187	-0.608	0.508	0.339	0.342	0.491	0.097	-1.181	0.358
17133	0.482	1.279	0.224	0.491	1.321	0.444	0.476	1.245	0.508
17134	0.482	1.279	0.508	0.491	1.321	0.371	0.476	1.245	0.097
17135	0.326	0.285	0.492	0.102	-1.184	0.422	0.145	-0.878	0.097
17136	0.466	1.178	0.492	0.509	1.434	0.422	0.513	1.481	0.097
17137	0.482	1.279	0.358	0.509	1.434	0.422	0.508	1.447	0.097

17138	0.332	0.318	0.097	0.149	-0.882	0.422	0.097	-1.181	0.513
17139	0.497	1.380	0.262	0.506	1.415	0.462	0.492	1.346	0.467
17140	0.497	1.380	0.262	0.506	1.415	0.235	0.492	1.346	0.513
17141	0.503	1.413	0.467	0.485	1.283	0.149	0.505	1.430	0.501
17142	0.511	1.464	0.467	0.485	1.283	0.508	0.505	1.430	0.501
17143	0.145	-0.878	0.513	0.275	-0.073	0.501	0.261	-0.137	0.512
17144	0.184	-0.625	0.501	0.275	-0.073	0.235	0.142	-0.895	0.481
17145	0.511	1.464	0.450	0.509	1.434	0.508	0.497	1.380	0.481
17146	0.503	1.413	0.450	0.398	0.718	0.462	0.500	1.397	0.481
17147	0.482	1.279	0.450	0.512	1.453	0.478	0.487	1.312	0.450
17148	0.500	1.397	0.050	0.488	1.302	0.193	0.503	1.413	0.328
17149	0.050	-1.485	0.262	0.152	-0.863	0.193	0.329	0.301	0.097
17150	0.050	-1.485	0.508	0.307	0.135	0.444	0.226	-0.356	0.508
17151	0.503	1.413	0.513	0.477	1.227	0.149	0.511	1.464	0.467
17152	0.500	1.397	0.224	0.442	1.001	0.501	0.495	1.363	0.481
17153	0.492	1.346	0.481	0.488	1.302	0.491	0.476	1.245	0.501
17154	0.511	1.464	0.409	0.442	1.001	0.444	0.495	1.363	0.501
17155	0.142	-0.895	0.262	0.374	0.568	0.193	0.187	-0.608	0.512
17156	0.468	1.194	0.492	0.421	0.869	0.309	0.508	1.447	0.512
17157	0.468	1.194	0.467	0.421	0.869	0.512	0.508	1.447	0.262
17158	0.468	1.194	0.467	0.421	0.869	0.273	0.508	1.447	0.481
17159	0.468	1.194	0.508	0.421	0.869	0.193	0.508	1.447	0.224
17160	0.482	1.279	0.050	0.462	1.133	0.514	0.508	1.447	0.224
17161	0.387	0.672	0.508	0.234	-0.336	0.478	0.097	-1.181	0.512
17162	0.097	-1.181	0.142	0.149	-0.882	0.478	0.332	0.318	0.328
17163	0.500	1.397	0.185	0.506	1.415	0.501	0.511	1.464	0.431
17164	0.500	1.397	0.508	0.509	1.434	0.235	0.476	1.245	0.409
17165	0.384	0.655	0.508	0.237	-0.317	0.508	0.050	-1.485	0.296
17166	0.513	1.481	0.097	0.506	1.415	0.491	0.492	1.346	0.513
17167	0.492	1.346	0.501	0.462	1.133	0.102	0.497	1.380	0.328
17168	0.489	1.329	0.262	0.482	1.265	0.462	0.495	1.363	0.492
17169	0.295	0.082	0.097	0.193	-0.600	0.508	0.263	-0.120	0.508
17170	0.295	0.082	0.262	0.193	-0.600	0.193	0.263	-0.120	0.450
17171	0.447	1.060	0.467	0.447	1.039	0.149	0.450	1.076	0.050
17172	0.263	-0.120	0.450	0.152	-0.863	0.309	0.184	-0.625	0.185
17173	0.463	1.161	0.508	0.497	1.359	0.102	0.497	1.380	0.450
17174	0.508	1.447	0.467	0.488	1.302	0.341	0.466	1.178	0.097
17175	0.426	0.925	0.142	0.442	1.001	0.235	0.492	1.346	0.481
17176	0.100	-1.164	0.492	0.193	-0.600	0.193	0.263	-0.120	0.481
17177	0.224	-0.373	0.501	0.307	0.135	0.149	0.000	?	0.409
17178	0.476	1.245	0.508	0.509	1.434	0.491	0.466	1.178	0.050
17179	0.261	-0.137	0.512	0.275	-0.073	0.508	0.184	-0.625	0.296
17180	0.226	-0.356	0.508	0.193	-0.600	0.501	0.142	-0.895	0.262
17181	0.484	1.295	0.142	0.515	1.472	0.309	0.455	1.110	0.262
17182	0.500	1.397	0.501	0.477	1.227	0.309	0.516	1.498	0.501
17183	0.500	1.397	0.097	0.477	1.227	0.371	0.516	1.498	0.512
17184	0.497	1.380	0.513	0.500	1.378	0.514	0.484	1.295	0.492
17185	0.097	-1.181	0.185	0.237	-0.317	0.371	0.297	0.099	0.467
17186	0.487	1.312	0.224	0.509	1.434	0.273	0.482	1.279	0.508
17187	0.492	1.346	0.450	0.491	1.321	0.309	0.505	1.430	0.508
17188	0.300	0.116	0.508	0.102	-1.184	0.235	0.224	-0.373	0.431
17189	0.503	1.413	0.501	0.462	1.133	0.341	0.508	1.447	0.050
17190	0.429	0.942	0.512	0.506	1.415	0.309	0.508	1.447	0.142
17191	0.097	-1.181	0.512	0.196	-0.581	0.235	0.295	0.082	0.492
17192	0.263	-0.120	0.296	0.149	-0.882	0.512	0.263	-0.120	0.492
17193	0.184	-0.625	0.481	0.307	0.135	0.478	0.050	-1.485	0.501
17194	0.295	0.082	0.142	0.102	-1.184	0.491	0.184	-0.625	0.450
17195	0.184	-0.625	0.501	0.339	0.342	0.235	0.050	-1.485	0.358
17196	0.187	-0.608	0.501	0.234	-0.336	0.102	0.297	0.099	0.492
17197	0.224	-0.373	0.513	0.196	-0.581	0.444	0.142	-0.895	0.512
17198	0.358	0.487	0.050	0.149	-0.882	0.508	0.187	-0.608	0.512

17199	0.479	1.262	0.513	0.488	1.302	0.444	0.503	1.413	0.450
17200	0.476	1.245	0.513	0.506	1.415	0.422	0.505	1.430	0.142
17201	0.503	1.413	0.050	0.497	1.359	0.193	0.479	1.262	0.431
17202	0.142	−0.895	0.501	0.310	0.153	0.508	0.184	−0.625	0.296
17203	0.142	−0.895	0.501	0.310	0.153	0.341	0.184	−0.625	0.142
17204	0.387	0.672	0.481	0.371	0.549	0.491	0.050	-1.485	0.224
17205	0.503	1.413	0.185	0.518	1.491	0.309	0.429	0.942	0.185
17206	0.411	0.824	0.501	0.371	0.549	0.462	0.184	−0.625	0.467
17207	0.142	−0.895	0.508	0.275	−0.073	0.102	0.261	−0.137	0.450
17208	0.187	−0.608	0.501	0.310	0.153	0.508	0.355	0.470	0.262
17209	0.297	0.099	0.501	0.237	−0.317	0.341	0.224	−0.373	0.262
17210	0.261	−0.137	0.050	0.345	0.379	0.309	0.226	−0.356	0.508
17211	0.261	−0.137	0.050	0.313	0.172	0.341	0.226	−0.356	0.296
17212	0.184	−0.625	0.512	0.237	−0.317	0.341	0.184	−0.625	0.385
17213	0.426	0.925	0.501	0.509	1.434	0.235	0.500	1.397	0.224
17214	0.295	0.082	0.050	0.482	1.265	0.273	0.263	−0.120	0.513
17215	0.484	1.295	0.513	0.491	1.321	0.508	0.508	1.447	0.513
17216	0.142	−0.895	0.513	0.234	−0.336	0.371	0.184	−0.625	0.431
17217	0.261	−0.137	0.512	0.102	−1.184	0.478	0.184	−0.625	0.481
17218	0.474	1.228	0.508	0.444	1.020	0.462	0.508	1.447	0.262
17219	0.476	1.245	0.492	0.506	1.415	0.508	0.511	1.464	0.409
17220	0.224	−0.373	0.512	0.450	1.057	0.508	0.384	0.655	0.409
17221	0.492	1.346	0.512	0.421	0.869	0.371	0.447	1.060	0.501
17222	0.358	0.487	0.513	0.196	−0.581	0.371	0.263	−0.120	0.481
17223	0.497	1.380	0.513	0.509	1.434	0.512	0.426	0.925	0.501
17224	0.329	0.301	0.508	0.342	0.361	0.235	0.097	−1.181	0.224
17225	0.447	1.060	0.512	0.494	1.340	0.235	0.508	1.447	0.512
17226	0.261	−0.137	0.467	0.310	0.153	0.371	0.382	0.638	0.224
17227	0.476	1.245	0.142	0.459	1.114	0.193	0.408	0.807	0.512
17228	0.295	0.082	0.185	0.105	−1.165	0.273	0.184	−0.625	0.328
17229	0.450	1.076	0.492	0.512	1.453	0.193	0.405	0.790	0.328
17230	0.050	−1.485	0.358	0.339	0.342	0.341	0.184	−0.625	0.467
17231	0.050	−1.485	0.508	0.310	0.153	0.501	0.224	−0.373	0.481
17232	0.050	−1.485	0.513	0.339	0.342	0.491	0.224	−0.373	0.450
17233	0.050	−1.485	0.450	0.339	0.342	0.491	0.184	−0.625	0.501
17234	0.050	−1.485	0.513	0.234	−0.336	0.508	0.263	−0.120	0.508
17235	0.142	−0.895	0.508	0.272	−0.091	0.512	0.224	−0.373	0.185
17236	0.511	1.464	0.328	0.503	1.396	0.501	0.384	0.655	0.501
17237	0.261	−0.137	0.328	0.374	0.568	0.501	0.142	−0.895	0.328
17238	0.508	1.447	0.328	0.477	1.227	0.508	0.513	1.481	0.097
17239	0.503	1.413	0.142	0.462	1.133	0.273	0.492	1.346	0.450
17240	0.513	1.481	0.358	0.512	1.453	0.398	0.450	1.076	0.492
17241	0.476	1.245	0.358	0.509	1.434	0.273	0.432	0.958	0.492
17242	0.187	−0.608	0.450	0.371	0.549	0.508	0.050	−1.485	0.512
17243	0.142	−0.895	0.431	0.371	0.549	0.422	0.000	?	0.224
17244	0.513	1.481	0.513	0.515	1.472	0.491	0.505	1.430	0.512
17245	0.097	−1.181	0.508	0.234	−0.336	0.501	0.300	0.116	0.512
17246	0.050	−1.485	0.508	0.234	−0.336	0.501	0.300	0.116	0.513
17247	0.500	1.397	0.142	0.371	0.549	0.273	0.463	1.161	0.512
17248	0.187	−0.608	0.185	0.196	−0.581	0.273	0.184	−0.625	0.508
17249	0.145	−0.878	0.508	0.275	−0.073	0.273	0.297	0.099	0.224
17250	0.297	0.099	0.508	0.193	−0.600	0.235	0.263	−0.120	0.492
17251	0.097	−1.181	0.185	0.342	0.361	0.422	0.295	0.082	0.431
17252	0.511	1.464	0.328	0.497	1.359	0.422	0.511	1.464	0.508
17253	0.487	1.312	0.185	0.491	1.321	0.422	0.463	1.161	0.467
17254	0.487	1.312	0.512	0.491	1.321	0.193	0.463	1.161	0.262
17255	0.487	1.312	0.050	0.509	1.434	0.508	0.463	1.161	0.358
17256	0.463	1.161	0.513	0.509	1.434	0.478	0.463	1.161	0.385
17257	0.461	1.144	0.409	0.497	1.359	0.491	0.497	1.380	0.508
17258	0.513	1.481	0.508	0.506	1.415	0.491	0.513	1.481	0.262
17259	0.450	1.076	0.142	0.509	1.434	0.444	0.513	1.481	0.492

17260	0.184	-0.625	0.481	0.272	-0.091	0.462	0.142	-0.895	0.492
17261	0.266	-0.103	0.492	0.398	0.718	0.462	0.263	-0.120	0.385
17262	0.142	-0.895	0.185	0.272	-0.091	0.501	0.142	-0.895	0.501
17263	0.476	1.245	0.296	0.512	1.453	0.444	0.513	1.481	0.512
17264	0.521	1.531	0.328	0.418	0.850	0.273	0.503	1.413	0.431
17265	0.453	1.093	0.262	0.491	1.321	0.341	0.503	1.413	0.431
17266	0.432	0.958	0.481	0.494	1.340	0.341	0.492	1.346	0.431
17267	0.484	1.295	0.450	0.500	1.378	0.491	0.508	1.447	0.431
17268	0.097	-1.181	0.467	0.278	-0.054	0.491	0.224	-0.373	0.481
17269	0.142	-0.895	0.508	0.278	-0.054	0.462	0.224	-0.373	0.501
17270	0.226	-0.356	0.050	0.272	-0.091	0.273	0.187	-0.608	0.467
17271	0.432	0.958	0.050	0.234	-0.336	0.501	0.224	-0.373	0.142
17272	0.516	1.498	0.262	0.421	0.869	0.371	0.479	1.262	0.142
17273	0.468	1.194	0.185	0.424	0.888	0.371	0.468	1.194	0.467
17274	0.505	1.430	0.508	0.424	0.888	0.371	0.500	1.397	0.097
17275	0.266	-0.103	0.050	0.193	-0.600	0.371	0.184	-0.625	0.467
17276	0.513	1.481	0.142	0.503	1.396	0.478	0.405	0.790	0.385
17277	0.429	0.942	0.050	0.477	1.227	0.512	0.513	1.481	0.512
17278	0.466	1.178	0.501	0.485	1.283	0.422	0.466	1.178	0.385
17279	0.492	1.346	0.501	0.491	1.321	0.501	0.500	1.397	0.450
17280	0.432	0.958	0.050	0.447	1.039	0.501	0.500	1.397	0.185
17281	0.508	1.447	0.508	0.456	1.095	0.508	0.489	1.329	0.513
17282	0.508	1.447	0.501	0.456	1.095	0.478	0.489	1.329	0.508
17283	0.482	1.279	0.262	0.500	1.378	0.478	0.329	0.301	0.481
17284	0.495	1.363	0.262	0.447	1.039	0.462	0.500	1.397	0.508
17285	0.261	-0.137	0.185	0.272	-0.091	0.491	0.224	-0.373	0.501
17286	0.263	-0.120	0.262	0.345	0.379	0.341	0.097	-1.181	0.328
17287	0.263	-0.120	0.185	0.345	0.379	0.309	0.097	-1.181	0.097
17288	0.513	1.481	0.508	0.488	1.302	0.341	0.405	0.790	0.450
17289	0.513	1.481	0.328	0.488	1.302	0.514	0.405	0.790	0.409
17290	0.468	1.194	0.501	0.465	1.152	0.478	0.463	1.161	0.142
17291	0.224	-0.373	0.097	0.275	-0.073	0.512	0.361	0.504	0.050
17292	0.513	1.481	0.328	0.497	1.359	0.508	0.503	1.413	0.142
17293	0.145	-0.878	0.185	0.371	0.549	0.501	0.097	-1.181	0.481
17294	0.097	-1.181	0.224	0.371	0.549	0.462	0.097	-1.181	0.185
17295	0.145	-0.878	0.492	0.371	0.549	0.273	0.097	-1.181	0.501
17296	0.145	-0.878	0.262	0.371	0.549	0.512	0.097	-1.181	0.467
17297	0.447	1.060	0.296	0.480	1.246	0.273	0.508	1.447	0.467
17298	0.508	1.447	0.385	0.509	1.434	0.235	0.461	1.144	0.142
17299	0.513	1.481	0.467	0.418	0.850	0.462	0.508	1.447	0.481
17300	0.479	1.262	0.185	0.503	1.396	0.512	0.505	1.430	0.512
17301	0.479	1.262	0.185	0.503	1.396	0.444	0.505	1.430	0.262
17302	0.382	0.638	0.185	0.503	1.396	0.309	0.513	1.481	0.097
17303	0.429	0.942	0.328	0.474	1.208	0.235	0.476	1.245	0.097
17304	0.429	0.942	0.328	0.474	1.208	0.462	0.476	1.245	0.328
17305	0.503	1.413	0.467	0.462	1.133	0.193	0.476	1.245	0.492
17306	0.513	1.481	0.508	0.491	1.321	0.514	0.447	1.060	0.508
17307	0.187	-0.608	0.513	0.342	0.361	0.491	0.329	0.301	0.481
17308	0.142	-0.895	0.512	0.307	0.135	0.512	0.097	-1.181	0.508
17309	0.450	1.076	0.385	0.342	0.361	0.341	0.508	1.447	0.262
17310	0.479	1.262	0.262	0.515	1.472	0.149	0.468	1.194	0.224
17311	0.511	1.464	0.481	0.482	1.265	0.491	0.476	1.245	0.142
17312	0.505	1.430	0.508	0.506	1.415	0.309	0.500	1.397	0.385
17313	0.505	1.430	0.185	0.503	1.396	0.514	0.500	1.397	0.450
17314	0.408	0.807	0.450	0.503	1.396	0.462	0.503	1.413	0.328
17315	0.497	1.380	0.328	0.462	1.133	0.478	0.511	1.464	0.224
17316	0.263	-0.120	0.296	0.275	-0.073	0.102	0.263	-0.120	0.262
17317	0.463	1.161	0.185	0.512	1.453	0.235	0.479	1.262	0.262
17318	0.050	-1.485	0.512	0.272	-0.091	0.102	0.224	-0.373	0.501
17319	0.224	-0.373	0.467	0.234	-0.336	0.102	0.226	-0.356	0.508
17320	0.500	1.397	0.512	0.459	1.114	0.462	0.505	1.430	0.142

17321	0.474	1.228	0.296	0.512	1.453	0.512	0.326	0.285	0.185
17322	0.492	1.346	0.296	0.447	1.039	0.514	0.429	0.942	0.492
17323	0.145	-0.878	0.296	0.310	0.153	0.478	0.408	0.807	0.512
17324	0.184	-0.625	0.481	0.237	-0.317	0.508	0.297	0.099	0.142
17325	0.489	1.329	0.492	0.459	1.114	0.512	0.511	1.464	0.142
17326	0.226	-0.356	0.185	0.196	-0.581	0.508	0.329	0.301	0.050
17327	0.463	1.161	0.142	0.518	1.491	0.444	0.487	1.312	0.097
17328	0.508	1.447	0.296	0.497	1.359	0.149	0.503	1.413	0.508
17329	0.497	1.380	0.492	0.518	1.491	0.508	0.450	1.076	0.501
17330	0.142	-0.895	0.492	0.342	0.361	0.444	0.050	-1.485	0.224
17331	0.266	-0.103	0.262	0.149	-0.882	0.398	0.184	-0.625	0.185
17332	0.505	1.430	0.481	0.488	1.302	0.444	0.447	1.060	0.142
17333	0.226	-0.356	0.481	0.310	0.153	0.508	0.097	-1.181	0.501
17334	0.513	1.481	0.467	0.512	1.453	0.235	0.479	1.262	0.328
17335	0.489	1.329	0.185	0.459	1.114	0.491	0.479	1.262	0.050
17336	0.495	1.363	0.501	0.477	1.227	0.512	0.408	0.807	0.050
17337	0.361	0.504	0.501	0.102	-1.184	0.512	0.050	-1.485	0.513
17338	0.097	-1.181	0.512	0.234	-0.336	0.512	0.300	0.116	0.142
17339	0.261	-0.137	0.224	0.102	-1.184	0.398	0.263	-0.120	0.097
17340	0.261	-0.137	0.262	0.102	-1.184	0.512	0.263	-0.120	0.185
17341	0.463	1.161	0.450	0.462	1.133	0.235	0.500	1.397	0.097
17342	0.466	1.178	0.512	0.509	1.434	0.273	0.511	1.464	0.050
17343	0.505	1.430	0.262	0.518	1.491	0.341	0.489	1.329	0.501
17344	0.500	1.397	0.262	0.480	1.246	0.341	0.461	1.144	0.385
17345	0.450	1.076	0.481	0.515	1.472	0.512	0.505	1.430	0.385
17346	0.450	1.076	0.296	0.518	1.491	0.491	0.508	1.447	0.385
17347	0.447	1.060	0.481	0.512	1.453	0.273	0.429	0.942	0.224
17348	0.050	-1.485	0.513	0.444	1.020	0.193	0.050	-1.485	0.501
17349	0.266	-0.103	0.328	0.149	-0.882	0.491	0.142	-0.895	0.450
17350	0.505	1.430	0.492	0.506	1.415	0.273	0.487	1.312	0.224
17351	0.513	1.481	0.262	0.442	1.001	0.193	0.484	1.295	0.512
17352	0.224	-0.373	0.508	0.401	0.737	0.053	0.000	?	0.262
17353	0.482	1.279	0.431	0.444	1.020	0.444	0.495	1.363	0.224
17354	0.408	0.807	0.224	0.509	1.434	0.444	0.447	1.060	0.262
17355	0.261	-0.137	0.224	0.237	-0.317	0.501	0.361	0.504	0.385
17356	0.489	1.329	0.262	0.488	1.302	0.478	0.487	1.312	0.512
17357	0.463	1.161	0.501	0.509	1.434	0.235	0.513	1.481	0.513
17358	0.463	1.161	0.328	0.509	1.434	0.508	0.513	1.481	0.512
17359	0.500	1.397	0.431	0.512	1.453	0.514	0.450	1.076	0.508
17360	0.050	-1.485	0.431	0.401	0.737	0.478	0.142	-0.895	0.142
17361	0.500	1.397	0.224	0.512	1.453	0.478	0.432	0.958	0.142
17362	0.142	-0.895	0.262	0.237	-0.317	0.501	0.297	0.099	0.097
17363	0.187	-0.608	0.142	0.275	-0.073	0.309	0.142	-0.895	0.512
17364	0.000	?	0.481	0.345	0.379	0.478	0.224	-0.373	0.467
17365	0.000	?	0.142	0.345	0.379	0.444	0.184	-0.625	0.185
17366	0.497	1.380	0.501	0.509	1.434	0.478	0.471	1.211	0.492
17367	0.497	1.380	0.467	0.497	1.359	0.371	0.445	1.043	0.492
17368	0.000	?	0.481	0.275	-0.073	0.341	0.263	-0.120	0.501
17369	0.097	-1.181	0.501	0.193	-0.600	0.512	0.266	-0.103	0.224
17370	0.503	1.413	0.501	0.488	1.302	0.512	0.505	1.430	0.508
17371	0.263	-0.120	0.431	0.275	-0.073	0.512	0.297	0.099	0.508
17372	0.097	-1.181	0.481	0.193	-0.600	0.508	0.387	0.672	0.501
17373	0.263	-0.120	0.185	0.053	-1.504	0.501	0.226	-0.356	0.097
17374	0.463	1.161	0.185	0.442	1.001	0.193	0.508	1.447	0.097
17375	0.453	1.093	0.431	0.444	1.020	0.501	0.516	1.498	0.501
17376	0.511	1.464	0.185	0.500	1.378	0.235	0.434	0.975	0.409
17377	0.466	1.178	0.512	0.480	1.246	0.309	0.476	1.245	0.513
17378	0.142	-0.895	0.512	0.234	-0.336	0.273	0.263	-0.120	0.224
17379	0.484	1.295	0.358	0.512	1.453	0.512	0.408	0.807	0.512
17380	0.492	1.346	0.492	0.518	1.491	0.512	0.408	0.807	0.492
17381	0.500	1.397	0.481	0.477	1.227	0.512	0.500	1.397	0.050

17382	0.500	1.397	0.185	0.480	1.246	0.398	0.482	1.279	0.512
17383	0.497	1.380	0.224	0.500	1.378	0.444	0.503	1.413	0.097
17384	0.142	−0.895	0.185	0.307	0.135	0.491	0.224	−0.373	0.431
17385	0.495	1.363	0.262	0.480	1.246	0.422	0.516	1.498	0.185
17386	0.100	−1.164	0.501	0.442	1.001	0.514	0.224	−0.373	0.185
17387	0.500	1.397	0.467	0.477	1.227	0.514	0.516	1.498	0.097
17388	0.184	−0.625	0.481	0.374	0.568	0.508	0.326	0.285	0.481
17389	0.224	−0.373	0.409	0.345	0.379	0.235	0.326	0.285	0.050
17390	0.445	1.043	0.358	0.518	1.491	0.501	0.468	1.194	0.050
17391	0.497	1.380	0.185	0.515	1.472	0.508	0.479	1.262	0.097
17392	0.492	1.346	0.358	0.518	1.491	0.512	0.453	1.093	0.142
17393	0.505	1.430	0.508	0.509	1.434	0.512	0.497	1.380	0.142
17394	0.503	1.413	0.358	0.503	1.396	0.398	0.505	1.430	0.467
17395	0.297	0.099	0.328	0.193	−0.600	0.398	0.184	−0.625	0.224
17396	0.476	1.245	0.431	0.503	1.396	0.501	0.497	1.380	0.409
17397	0.000	?	0.513	0.234	−0.336	0.501	0.329	0.301	0.467
17398	0.142	−0.895	0.296	0.313	0.172	0.371	0.097	−1.181	0.501
17399	0.000	?	0.328	0.275	−0.073	0.491	0.450	1.076	0.492
17400	0.492	1.346	0.185	0.515	1.472	0.422	0.492	1.346	0.492
17401	0.492	1.346	0.501	0.515	1.472	0.501	0.492	1.346	0.481
17402	0.503	1.413	0.296	0.512	1.453	0.512	0.513	1.481	0.431
17403	0.050	−1.485	0.328	0.404	0.756	0.508	0.224	−0.373	0.262
17404	0.503	1.413	0.492	0.444	1.020	0.444	0.513	1.481	0.262
17405	0.503	1.413	0.501	0.491	1.321	0.508	0.513	1.481	0.501
17406	0.050	−1.485	0.097	0.424	0.888	0.508	0.000	?	0.185
17407	0.492	1.346	0.296	0.512	1.453	0.508	0.516	1.498	0.512
17408	0.492	1.346	0.142	0.512	1.453	0.491	0.513	1.481	0.508
17409	0.476	1.245	0.296	0.509	1.434	0.398	0.508	1.447	0.050
17410	0.184	−0.625	0.296	0.234	−0.336	0.512	0.226	−0.356	0.492
17411	0.495	1.363	0.142	0.500	1.378	0.309	0.495	1.363	0.492
17412	0.497	1.380	0.142	0.512	1.453	0.309	0.429	0.942	0.224
17413	0.492	1.346	0.185	0.518	1.491	0.501	0.511	1.464	0.224
17414	0.492	1.346	0.262	0.518	1.491	0.193	0.466	1.178	0.224
17415	0.050	−1.485	0.097	0.401	0.737	0.462	0.263	−0.120	0.224
17416	0.050	−1.485	0.467	0.401	0.737	0.512	0.358	0.487	0.501
17417	0.500	1.397	0.050	0.500	1.378	0.514	0.384	0.655	0.501
17418	0.492	1.346	0.328	0.500	1.378	0.512	0.508	1.447	0.481
17419	0.050	−1.485	0.508	0.377	0.587	0.422	0.261	−0.137	0.492
17420	0.500	1.397	0.224	0.494	1.340	0.193	0.492	1.346	0.097
17421	0.503	1.413	0.467	0.424	0.888	0.193	0.489	1.329	0.142
17422	0.500	1.397	0.328	0.503	1.396	0.273	0.382	0.638	0.501
17423	0.500	1.397	0.409	0.515	1.472	0.273	0.503	1.413	0.492
17424	0.484	1.295	0.262	0.509	1.434	0.149	0.513	1.481	0.142
17425	0.508	1.447	0.328	0.442	1.001	0.149	0.429	0.942	0.050
17426	0.508	1.447	0.185	0.509	1.434	0.491	0.429	0.942	0.508
17427	0.508	1.447	0.508	0.509	1.434	0.491	0.429	0.942	0.512
17428	0.508	1.447	0.142	0.509	1.434	0.491	0.429	0.942	0.224
17429	0.503	1.413	0.296	0.494	1.340	0.478	0.482	1.279	0.224
17430	0.508	1.447	0.481	0.398	0.718	0.491	0.503	1.413	0.097
17431	0.461	1.144	0.385	0.515	1.472	0.053	0.463	1.161	0.512
17432	0.142	−0.895	0.224	0.310	0.153	0.341	0.142	−0.895	0.097
17433	0.184	−0.625	0.262	0.310	0.153	0.508	0.142	−0.895	0.097
17434	0.487	1.312	0.262	0.503	1.396	0.398	0.463	1.161	0.481
17435	0.361	0.504	0.450	0.193	−0.600	0.309	0.097	−1.181	0.512
17436	0.497	1.380	0.224	0.465	1.152	0.149	0.466	1.178	0.142
17437	0.511	1.464	0.492	0.509	1.434	0.273	0.384	0.655	0.142
17438	0.453	1.093	0.328	0.512	1.453	0.514	0.511	1.464	0.097
17439	0.511	1.464	0.185	0.512	1.453	0.309	0.384	0.655	0.097
17440	0.508	1.447	0.224	0.421	0.869	0.512	0.447	1.060	0.224
17441	0.329	0.301	0.185	0.193	−0.600	0.462	0.000	?	0.224
17442	0.329	0.301	0.501	0.193	−0.600	0.501	0.000	?	0.142

17443	0.332	0.318	0.508	0.272	-0.091	0.309	0.000	?	0.097
17444	0.142	-0.895	0.185	0.275	-0.073	0.501	0.184	-0.625	0.142
17445	0.361	0.504	0.508	0.149	-0.882	0.478	0.000	?	0.501
17446	0.361	0.504	0.508	0.149	-0.882	0.235	0.000	?	0.142
17447	0.450	1.076	0.508	0.491	1.321	0.149	0.503	1.413	0.508
17448	0.429	0.942	0.262	0.488	1.302	0.102	0.511	1.464	0.481
17449	0.511	1.464	0.224	0.488	1.302	0.149	0.482	1.279	0.185
17450	0.508	1.447	0.296	0.474	1.208	0.444	0.508	1.447	0.512
17451	0.508	1.447	0.328	0.485	1.283	0.462	0.503	1.413	0.050
17452	0.142	-0.895	0.224	0.053	-1.504	0.491	0.329	0.301	0.508
17453	0.184	-0.625	0.142	0.345	0.379	0.462	0.097	-1.181	0.097
17454	0.505	1.430	0.492	0.509	1.434	0.341	0.450	1.076	0.097
17455	0.505	1.430	0.481	0.398	0.718	0.398	0.411	0.824	0.097
17456	0.184	-0.625	0.262	0.310	0.153	0.149	0.142	-0.895	0.450
17457	0.332	0.318	0.224	0.149	-0.882	0.371	0.050	-1.485	0.328
17458	0.184	-0.625	0.224	0.272	-0.091	0.501	0.142	-0.895	0.142
17459	0.505	1.430	0.185	0.515	1.472	0.193	0.479	1.262	0.512
17460	0.050	-1.485	0.050	0.310	0.153	0.309	0.184	-0.625	0.185
17461	0.508	1.447	0.050	0.509	1.434	0.341	0.505	1.430	0.185
17462	0.408	0.807	0.513	0.459	1.114	0.371	0.463	1.161	0.185
17463	0.508	1.447	0.328	0.503	1.396	0.462	0.463	1.161	0.501
17464	0.142	-0.895	0.224	0.310	0.153	0.478	0.142	-0.895	0.097
17465	0.482	1.279	0.142	0.497	1.359	0.371	0.484	1.295	0.508
17466	0.489	1.329	0.501	0.474	1.208	0.341	0.513	1.481	0.050
17467	0.184	-0.625	0.185	0.237	-0.317	0.478	0.263	-0.120	0.142
17468	0.297	0.099	0.467	0.149	-0.882	0.514	0.097	-1.181	0.097
17469	0.329	0.301	0.224	0.102	-1.184	0.273	0.097	-1.181	0.097
17470	0.263	-0.120	0.501	0.149	-0.882	0.149	0.332	0.318	0.450
17471	0.482	1.279	0.385	0.442	1.001	0.235	0.489	1.329	0.450
17472	0.447	1.060	0.431	0.459	1.114	0.235	0.503	1.413	0.224
17473	0.461	1.144	0.185	0.488	1.302	0.273	0.482	1.279	0.492
17474	0.503	1.413	0.185	0.459	1.114	0.444	0.508	1.447	0.492
17475	0.050	-1.485	0.385	0.342	0.361	0.193	0.261	-0.137	0.492
17476	0.050	-1.485	0.508	0.398	0.718	0.478	0.226	-0.356	0.262
17477	0.266	-0.103	0.481	0.149	-0.882	0.235	0.142	-0.895	0.142
17478	0.184	-0.625	0.262	0.371	0.549	0.193	0.382	0.638	0.142
17479	0.497	1.380	0.501	0.500	1.378	0.193	0.453	1.093	0.097
17480	0.000	?	0.262	0.193	-0.600	0.193	0.326	0.285	0.142
17481	0.050	-1.485	0.262	0.310	0.153	0.444	0.224	-0.373	0.467
17482	0.142	-0.895	0.296	0.345	0.379	0.273	0.263	-0.120	0.467
17483	0.050	-1.485	0.513	0.371	0.549	0.341	0.261	-0.137	0.050
17484	0.492	1.346	0.481	0.465	1.152	0.193	0.500	1.397	0.142
17485	0.495	1.363	0.501	0.477	1.227	0.444	0.508	1.447	0.142
17486	0.000	?	0.467	0.368	0.530	0.309	0.145	-0.878	0.224
17487	0.050	-1.485	0.467	0.345	0.379	0.309	0.184	-0.625	0.050
17488	0.497	1.380	0.501	0.480	1.246	0.273	0.492	1.346	0.142
17489	0.492	1.346	0.501	0.518	1.491	0.102	0.511	1.464	0.492
17490	0.263	-0.120	0.508	0.275	-0.073	0.149	0.000	?	0.097
17491	0.261	-0.137	0.142	0.149	-0.882	0.514	0.142	-0.895	0.224
17492	0.187	-0.608	0.262	0.237	-0.317	0.501	0.142	-0.895	0.508
17493	0.263	-0.120	0.262	0.234	-0.336	0.501	0.050	-1.485	0.185
17494	0.184	-0.625	0.467	0.275	-0.073	0.508	0.097	-1.181	0.512
17495	0.505	1.430	0.185	0.444	1.020	0.193	0.500	1.397	0.142
17496	0.329	0.301	0.142	0.196	-0.581	0.235	0.000	?	0.512
17497	0.497	1.380	0.224	0.477	1.227	0.444	0.497	1.380	0.050
17498	0.097	-1.181	0.492	0.234	-0.336	0.491	0.226	-0.356	0.409
17499	0.326	0.285	0.492	0.196	-0.581	0.149	0.000	?	0.224
17500	0.187	-0.608	0.224	0.193	-0.600	0.462	0.187	-0.608	0.224
17501	0.226	-0.356	0.481	0.193	-0.600	0.149	0.142	-0.895	0.050
17502	0.495	1.363	0.467	0.442	1.001	0.193	0.492	1.346	0.512
17503	0.263	-0.120	0.142	0.272	-0.091	0.309	0.326	0.285	0.481

17504	0.295	0.082	0.512	0.345	0.379	0.462	0.050	-1.485	0.050
17505	0.384	0.655	0.512	0.193	-0.600	0.444	0.050	-1.485	0.467
17506	0.471	1.211	0.142	0.442	1.001	0.478	0.503	1.413	0.050
17507	0.184	-0.625	0.097	0.310	0.153	0.273	0.142	-0.895	0.050
17508	0.184	-0.625	0.513	0.310	0.153	0.273	0.097	-1.181	0.097
17509	0.184	-0.625	0.513	0.275	-0.073	0.273	0.187	-0.608	0.508
17510	0.332	0.318	0.097	0.102	-1.184	0.444	0.097	-1.181	0.512
17511	0.332	0.318	0.481	0.149	-0.882	0.422	0.050	-1.485	0.508
17512	0.466	1.178	0.262	0.512	1.453	0.273	0.495	1.363	0.492
17513	0.505	1.430	0.492	0.503	1.396	0.273	0.384	0.655	0.450
17514	0.511	1.464	0.224	0.503	1.396	0.149	0.384	0.655	0.467
17515	0.508	1.447	0.467	0.509	1.434	0.508	0.384	0.655	0.467
17516	0.384	0.655	0.097	0.193	-0.600	0.491	0.000	?	0.467
17517	0.263	-0.120	0.142	0.237	-0.317	0.149	0.224	-0.373	0.185
17518	0.479	1.262	0.508	0.442	1.001	0.501	0.497	1.380	0.224
17519	0.508	1.447	0.097	0.491	1.321	0.462	0.445	1.043	0.224
17520	0.184	-0.625	0.142	0.149	-0.882	0.422	0.226	-0.356	0.501
17521	0.450	1.076	0.185	0.459	1.114	0.149	0.505	1.430	0.142
17522	0.332	0.318	0.508	0.149	-0.882	0.462	0.263	-0.120	0.185
17523	0.297	0.099	0.481	0.193	-0.600	0.422	0.229	-0.339	0.224
17524	0.187	-0.608	0.431	0.310	0.153	0.501	0.263	-0.120	0.431
17525	0.508	1.447	0.262	0.462	1.133	0.309	0.382	0.638	0.431
17526	0.463	1.161	0.142	0.450	1.057	0.235	0.500	1.397	0.224
17527	0.513	1.481	0.224	0.474	1.208	0.512	0.505	1.430	0.431
17528	0.295	0.082	0.224	0.275	-0.073	0.512	0.000	?	0.512
17529	0.295	0.082	0.512	0.275	-0.073	0.371	0.000	?	0.142
17530	0.295	0.082	0.512	0.275	-0.073	0.371	0.000	?	0.450
17531	0.482	1.279	0.262	0.442	1.001	0.512	0.503	1.413	0.450
17532	0.489	1.329	0.508	0.424	0.888	0.102	0.500	1.397	0.142
17533	0.184	-0.625	0.481	0.272	-0.091	0.508	0.142	-0.895	0.224
17534	0.142	-0.895	0.097	0.272	-0.091	0.508	0.142	-0.895	0.508
17535	0.300	0.116	0.262	0.149	-0.882	0.149	0.097	-1.181	0.512
17536	0.497	1.380	0.142	0.506	1.415	0.422	0.505	1.430	0.296
17537	0.489	1.329	0.409	0.491	1.321	0.193	0.461	1.144	0.431
17538	0.263	-0.120	0.409	0.149	-0.882	0.444	0.184	-0.625	0.224
17539	0.487	1.312	0.224	0.494	1.340	0.149	0.484	1.295	0.467
17540	0.476	1.245	0.262	0.462	1.133	0.235	0.489	1.329	0.224
17541	0.466	1.178	0.508	0.424	0.888	0.235	0.492	1.346	0.467
17542	0.187	-0.608	0.508	0.149	-0.882	0.193	0.224	-0.373	0.262
17543	0.503	1.413	0.262	0.465	1.152	0.478	0.497	1.380	0.185
17544	0.503	1.413	0.513	0.424	0.888	0.309	0.497	1.380	0.224
17545	0.516	1.498	0.512	0.500	1.378	0.193	0.492	1.346	0.512
17546	0.224	-0.373	0.050	0.310	0.153	0.273	0.097	-1.181	0.262
17547	0.263	-0.120	0.050	0.234	-0.336	0.235	0.097	-1.181	0.142
17548	0.453	1.093	0.508	0.509	1.434	0.149	0.492	1.346	0.185
17549	0.508	1.447	0.296	0.506	1.415	0.273	0.408	0.807	0.508
17550	0.263	-0.120	0.450	0.368	0.530	0.462	0.000	?	0.467
17551	0.263	-0.120	0.296	0.368	0.530	0.235	0.000	?	0.508
17552	0.482	1.279	0.262	0.512	1.453	0.508	0.503	1.413	0.224
17553	0.297	0.099	0.296	0.102	-1.184	0.501	0.224	-0.373	0.296
17554	0.482	1.279	0.262	0.506	1.415	0.398	0.505	1.430	0.142
17555	0.516	1.498	0.142	0.506	1.415	0.491	0.484	1.295	0.142
17556	0.329	0.301	0.492	0.149	-0.882	0.491	0.050	-1.485	0.492
17557	0.492	1.346	0.501	0.424	0.888	0.501	0.500	1.397	0.492
17558	0.263	-0.120	0.467	0.193	-0.600	0.478	0.097	-1.181	0.224
17559	0.511	1.464	0.501	0.442	1.001	0.149	0.426	0.925	0.481
17560	0.432	0.958	0.296	0.149	-0.882	0.102	0.000	?	0.501
17561	0.226	-0.356	0.385	0.234	-0.336	0.514	0.187	-0.608	0.224
17562	0.226	-0.356	0.224	0.234	-0.336	0.193	0.187	-0.608	0.142
17563	0.261	-0.137	0.262	0.196	-0.581	0.462	0.097	-1.181	0.328
17564	0.497	1.380	0.262	0.477	1.227	0.444	0.476	1.245	0.512

17565	0.326	0.285	0.385	0.310	0.153	0.235	0.000	?	0.501
17566	0.432	0.958	0.296	0.193	−0.600	0.491	0.050	−1.485	0.097
17567	0.432	0.958	0.224	0.272	−0.091	0.491	0.050	−1.485	0.296
17568	0.224	−0.373	0.224	0.237	−0.317	0.273	0.097	−1.181	0.467
17569	0.261	−0.137	0.492	0.149	−0.882	0.273	0.142	−0.895	0.467
17570	0.142	−0.895	0.142	0.272	−0.091	0.273	0.142	−0.895	0.185
17571	0.484	1.295	0.450	0.456	1.095	0.273	0.463	1.161	0.481
17572	0.142	−0.895	0.185	0.237	−0.317	0.478	0.224	−0.373	0.508
17573	0.497	1.380	0.431	0.506	1.415	0.512	0.405	0.790	0.358
17574	0.463	1.161	0.508	0.503	1.396	0.508	0.463	1.161	0.358
17575	0.482	1.279	0.097	0.395	0.700	0.514	0.495	1.363	0.508
17576	0.500	1.397	0.492	0.485	1.283	0.053	0.489	1.329	0.185
17577	0.500	1.397	0.185	0.485	1.283	0.235	0.489	1.329	0.508
17578	0.429	0.942	0.501	0.503	1.396	0.508	0.474	1.228	0.262
17579	0.482	1.279	0.492	0.474	1.208	0.462	0.426	0.925	0.262
17580	0.184	−0.625	0.450	0.152	−0.863	0.235	0.263	−0.120	0.262
17581	0.184	−0.625	0.328	0.105	−1.165	0.341	0.263	−0.120	0.185
17582	0.429	0.942	0.142	0.515	1.472	0.478	0.495	1.363	0.328
17583	0.184	−0.625	0.508	0.193	−0.600	0.491	0.184	−0.625	0.467
17584	0.513	1.481	0.492	0.459	1.114	0.102	0.503	1.413	0.508
17585	0.513	1.481	0.409	0.442	1.001	0.102	0.500	1.397	0.481
17586	0.358	0.487	0.409	0.237	−0.317	0.341	0.050	−1.485	0.512
17587	0.492	1.346	0.501	0.491	1.321	0.444	0.484	1.295	0.185
17588	0.482	1.279	0.050	0.491	1.321	0.149	0.484	1.295	0.431
17589	0.184	−0.625	0.501	0.272	−0.091	0.193	0.226	−0.356	0.328
17590	0.224	−0.373	0.513	0.272	−0.091	0.444	0.226	−0.356	0.385
17591	0.184	−0.625	0.385	0.272	−0.091	0.398	0.226	−0.356	0.385
17592	0.261	−0.137	0.097	0.272	−0.091	0.309	0.226	−0.356	0.358
17593	0.497	1.380	0.224	0.480	1.246	0.235	0.495	1.363	0.262
17594	0.466	1.178	0.185	0.509	1.434	0.273	0.495	1.363	0.262
17595	0.482	1.279	0.185	0.506	1.415	0.149	0.479	1.262	0.481
17596	0.405	0.790	0.467	0.515	1.472	0.149	0.484	1.295	0.358
17597	0.355	0.470	0.512	0.053	−1.504	0.235	0.097	−1.181	0.501
17598	0.187	−0.608	0.450	0.237	−0.317	0.235	0.142	−0.895	0.385
17599	0.361	0.504	0.481	0.509	1.434	0.235	0.492	1.346	0.492
17600	0.508	1.447	0.142	0.465	1.152	0.193	0.484	1.295	0.508
17601	0.355	0.470	0.492	0.237	−0.317	0.341	0.142	−0.895	0.224
17602	0.326	0.285	0.224	0.345	0.379	0.508	0.050	−1.485	0.467
17603	0.432	0.958	0.224	0.480	1.246	0.341	0.503	1.413	0.224
17604	0.516	1.498	0.450	0.491	1.321	0.512	0.505	1.430	0.508
17605	0.297	0.099	0.508	0.102	−1.184	0.478	0.224	−0.373	0.409
17606	0.332	0.318	0.097	0.102	−1.184	0.235	0.224	−0.373	0.501
17607	0.187	−0.608	0.224	0.342	0.361	0.462	0.097	−1.181	0.185
17608	0.503	1.413	0.431	0.442	1.001	0.193	0.503	1.413	0.185
17609	0.297	0.099	0.224	0.149	−0.882	0.501	0.097	−1.181	0.508
17610	0.329	0.301	0.097	0.193	−0.600	0.193	0.097	−1.181	0.501
17611	0.492	1.346	0.385	0.439	0.982	0.235	0.479	1.262	0.492
17612	0.497	1.380	0.467	0.395	0.700	0.309	0.508	1.447	0.508
17613	0.097	−1.181	0.262	0.307	0.135	0.491	0.142	−0.895	0.501
17614	0.297	0.099	0.508	0.237	−0.317	0.053	0.000	?	0.358
17615	0.142	−0.895	0.142	0.275	−0.073	0.193	0.142	−0.895	0.467
17616	0.295	0.082	0.450	0.149	−0.882	0.398	0.097	−1.181	0.296
17617	0.295	0.082	0.450	0.149	−0.882	0.193	0.097	−1.181	0.385
17618	0.142	−0.895	0.185	0.234	−0.336	0.193	0.226	−0.356	0.513
17619	0.142	−0.895	0.501	0.234	−0.336	0.149	0.226	−0.356	0.185
17620	0.184	−0.625	0.224	0.237	−0.317	0.149	0.142	−0.895	0.224
17621	0.261	−0.137	0.224	0.193	−0.600	0.501	0.100	−1.164	0.185
17622	0.097	−1.181	0.142	0.342	0.361	0.273	0.142	−0.895	0.097
17623	0.466	1.178	0.328	0.509	1.434	0.478	0.495	1.363	0.409
17624	0.050	−1.485	0.328	0.342	0.361	0.341	0.142	−0.895	0.450
17625	0.326	0.285	0.262	0.518	1.491	0.309	0.503	1.413	0.467

17626	0.505	1.430	0.296	0.477	1.227	0.371	0.476	1.245	0.481
17627	0.226	-0.356	0.224	0.237	-0.317	0.273	0.184	-0.625	0.224
17628	0.466	1.178	0.492	0.462	1.133	0.508	0.505	1.430	0.512
17629	0.329	0.301	0.142	0.193	-0.600	0.444	0.050	-1.485	0.296
17630	0.408	0.807	0.185	0.503	1.396	0.149	0.500	1.397	0.296
17631	0.261	-0.137	0.508	0.196	-0.581	0.508	0.097	-1.181	0.492
17632	0.326	0.285	0.185	0.234	-0.336	0.508	0.097	-1.181	0.467
17633	0.184	-0.625	0.481	0.313	0.172	0.508	0.097	-1.181	0.296
17634	0.505	1.430	0.492	0.488	1.302	0.193	0.445	1.043	0.296
17635	0.142	-0.895	0.492	0.053	-1.504	0.273	0.329	0.301	0.501
17636	0.295	0.082	0.185	0.196	-0.581	0.102	0.145	-0.878	0.385
17637	0.482	1.279	0.512	0.398	0.718	0.193	0.503	1.413	0.185
17638	0.384	0.655	0.508	0.193	-0.600	0.235	0.000	?	0.508
17639	0.224	-0.373	0.512	0.193	-0.600	0.273	0.184	-0.625	0.492
17640	0.261	-0.137	0.142	0.149	-0.882	0.514	0.184	-0.625	0.328
17641	0.261	-0.137	0.481	0.149	-0.882	0.514	0.184	-0.625	0.492
17642	0.453	1.093	0.501	0.497	1.359	0.235	0.495	1.363	0.262
17643	0.224	-0.373	0.467	0.275	-0.073	0.273	0.097	-1.181	0.492
17644	0.487	1.312	0.142	0.480	1.246	0.273	0.503	1.413	0.450
17645	0.329	0.301	0.501	0.342	0.361	0.149	0.050	-1.485	0.328
17646	0.187	-0.608	0.328	0.307	0.135	0.398	0.142	-0.895	0.431
17647	0.224	-0.373	0.224	0.371	0.549	0.398	0.097	-1.181	0.358
17648	0.184	-0.625	0.431	0.275	-0.073	0.491	0.097	-1.181	0.296
17649	0.500	1.397	0.450	0.512	1.453	0.235	0.445	1.043	0.296
17650	0.505	1.430	0.492	0.444	1.020	0.235	0.445	1.043	0.385
17651	0.184	-0.625	0.262	0.149	-0.882	0.273	0.224	-0.373	0.431
17652	0.508	1.447	0.467	0.500	1.378	0.422	0.492	1.346	0.262
17653	0.508	1.447	0.358	0.500	1.378	0.235	0.492	1.346	0.224
17654	0.508	1.447	0.185	0.500	1.378	0.508	0.492	1.346	0.262
17655	0.261	-0.137	0.513	0.193	-0.600	0.235	0.261	-0.137	0.508
17656	0.226	-0.356	0.513	0.272	-0.091	0.491	0.142	-0.895	0.224
17657	0.300	0.116	0.142	0.102	-1.184	0.102	0.142	-0.895	0.328
17658	0.329	0.301	0.508	0.196	-0.581	0.512	0.000	?	0.450
17659	0.224	-0.373	0.512	0.234	-0.336	0.341	0.100	-1.164	0.296
17660	0.142	-0.895	0.512	0.278	-0.054	0.193	0.142	-0.895	0.501
17661	0.495	1.363	0.481	0.512	1.453	0.235	0.466	1.178	0.508
17662	0.482	1.279	0.262	0.512	1.453	0.478	0.466	1.178	0.508
17663	0.263	-0.120	0.508	0.237	-0.317	0.512	0.050	-1.485	0.142
17664	0.224	-0.373	0.296	0.272	-0.091	0.341	0.145	-0.878	0.385
17665	0.224	-0.373	0.512	0.272	-0.091	0.508	0.145	-0.878	0.501
17666	0.184	-0.625	0.409	0.152	-0.863	0.273	0.224	-0.373	0.513
17667	0.050	-1.485	0.450	0.401	0.737	0.235	0.050	-1.485	0.296
17668	0.050	-1.485	0.492	0.401	0.737	0.149	0.142	-0.895	0.481
17669	0.508	1.447	0.328	0.494	1.340	0.478	0.487	1.312	0.501
17670	0.329	0.301	0.481	0.234	-0.336	0.491	0.000	?	0.481
17671	0.224	-0.373	0.358	0.237	-0.317	0.444	0.097	-1.181	0.431
17672	0.142	-0.895	0.185	0.275	-0.073	0.501	0.224	-0.373	0.467
17673	0.503	1.413	0.513	0.421	0.869	0.501	0.497	1.380	0.328
17674	0.184	-0.625	0.501	0.234	-0.336	0.478	0.187	-0.608	0.224
17675	0.468	1.194	0.481	0.506	1.415	0.491	0.503	1.413	0.501
17676	0.224	-0.373	0.142	0.234	-0.336	0.478	0.142	-0.895	0.492
17677	0.503	1.413	0.481	0.491	1.321	0.273	0.500	1.397	0.513
17678	0.384	0.655	0.262	0.102	-1.184	0.053	0.050	-1.485	0.224
17679	0.426	0.925	0.512	0.515	1.472	0.053	0.411	0.824	0.512
17680	0.184	-0.625	0.512	0.342	0.361	0.478	0.226	-0.356	0.296
17681	0.184	-0.625	0.431	0.196	-0.581	0.235	0.224	-0.373	0.358
17682	0.387	0.672	0.481	0.237	-0.317	0.235	0.050	-1.485	0.450
17683	0.508	1.447	0.508	0.474	1.208	0.102	0.505	1.430	0.450
17684	0.484	1.295	0.492	0.509	1.434	0.462	0.474	1.228	0.185
17685	0.263	-0.120	0.185	0.342	0.361	0.462	0.050	-1.485	0.508
17686	0.503	1.413	0.481	0.503	1.396	0.102	0.461	1.144	0.508

17687	0.261	-0.137	0.142	0.272	-0.091	0.478	0.050	-1.485	0.508
17688	0.263	-0.120	0.185	0.237	-0.317	0.444	0.050	-1.485	0.501
17689	0.295	0.082	0.185	0.149	-0.882	0.273	0.097	-1.181	0.224
17690	0.516	1.498	0.224	0.477	1.227	0.491	0.500	1.397	0.501
17691	0.479	1.262	0.142	0.488	1.302	0.491	0.505	1.430	0.296
17692	0.505	1.430	0.481	0.442	1.001	0.309	0.500	1.397	0.450
17693	0.463	1.161	0.512	0.503	1.396	0.341	0.484	1.295	0.409
17694	0.466	1.178	0.185	0.500	1.378	0.491	0.447	1.060	0.501
17695	0.500	1.397	0.358	0.480	1.246	0.501	0.461	1.144	0.481
17696	0.500	1.397	0.508	0.491	1.321	0.235	0.461	1.144	0.262
17697	0.505	1.430	0.467	0.480	1.246	0.491	0.461	1.144	0.467
17698	0.142	-0.895	0.467	0.272	-0.091	0.309	0.184	-0.625	0.224
17699	0.261	-0.137	0.481	0.053	-1.504	0.512	0.224	-0.373	0.358
17700	0.261	-0.137	0.501	0.053	-1.504	0.149	0.224	-0.373	0.431
17701	0.468	1.194	0.328	0.477	1.227	0.444	0.492	1.346	0.467
17702	0.184	-0.625	0.097	0.237	-0.317	0.341	0.142	-0.895	0.492
17703	0.142	-0.895	0.328	0.234	-0.336	0.309	0.184	-0.625	0.450
17704	0.226	-0.356	0.512	0.102	-1.184	0.341	0.224	-0.373	0.431
17705	0.495	1.363	0.481	0.462	1.133	0.462	0.426	0.925	0.142
17706	0.495	1.363	0.481	0.462	1.133	0.341	0.426	0.925	0.467
17707	0.226	-0.356	0.296	0.102	-1.184	0.309	0.224	-0.373	0.467
17708	0.482	1.279	0.296	0.477	1.227	0.309	0.426	0.925	0.467
17709	0.468	1.194	0.501	0.442	1.001	0.514	0.503	1.413	0.501
17710	0.142	-0.895	0.262	0.275	-0.073	0.501	0.142	-0.895	0.467
17711	0.505	1.430	0.262	0.494	1.340	0.462	0.453	1.093	0.512
17712	0.505	1.430	0.450	0.494	1.340	0.341	0.453	1.093	0.097
17713	0.142	-0.895	0.097	0.307	0.135	0.235	0.142	-0.895	0.513
17714	0.097	-1.181	0.508	0.345	0.379	0.193	0.224	-0.373	0.296
17715	0.500	1.397	0.296	0.488	1.302	0.193	0.513	1.481	0.224
17716	0.500	1.397	0.185	0.500	1.378	0.462	0.518	1.514	0.296
17717	0.097	-1.181	0.501	0.237	-0.317	0.462	0.295	0.082	0.050
17718	0.479	1.262	0.185	0.491	1.321	0.193	0.429	0.942	0.328
17719	0.263	-0.120	0.185	0.310	0.153	0.491	0.224	-0.373	0.409
17720	0.492	1.346	0.481	0.509	1.434	0.491	0.466	1.178	0.450
17721	0.226	-0.356	0.262	0.149	-0.882	0.235	0.224	-0.373	0.185
17722	0.466	1.178	0.097	0.447	1.039	0.149	0.466	1.178	0.262
17723	0.097	-1.181	0.508	0.342	0.361	0.149	0.261	-0.137	0.492
17724	0.142	-0.895	0.467	0.307	0.135	0.444	0.184	-0.625	0.508
17725	0.000	?	0.224	0.345	0.379	0.398	0.224	-0.373	0.508
17726	0.503	1.413	0.224	0.462	1.133	0.309	0.508	1.447	0.508
17727	0.097	-1.181	0.224	0.345	0.379	0.444	0.263	-0.120	0.481
17728	0.142	-0.895	0.097	0.310	0.153	0.501	0.145	-0.878	0.328
17729	0.184	-0.625	0.481	0.310	0.153	0.501	0.187	-0.608	0.262
17730	0.505	1.430	0.385	0.515	1.472	0.102	0.508	1.447	0.328
17731	0.474	1.228	0.185	0.506	1.415	0.512	0.466	1.178	0.467
17732	0.432	0.958	0.385	0.459	1.114	0.478	0.505	1.430	0.508
17733	0.261	-0.137	0.508	0.345	0.379	0.273	0.224	-0.373	0.508
17734	0.145	-0.878	0.512	0.237	-0.317	0.341	0.295	0.082	0.481
17735	0.224	-0.373	0.328	0.193	-0.600	0.501	0.142	-0.895	0.481
17736	0.224	-0.373	0.224	0.193	-0.600	0.371	0.142	-0.895	0.450
17737	0.511	1.464	0.501	0.462	1.133	0.235	0.487	1.312	0.262
17738	0.511	1.464	0.450	0.462	1.133	0.193	0.487	1.312	0.262
17739	0.263	-0.120	0.328	0.193	-0.600	0.149	0.224	-0.373	0.481
17740	0.503	1.413	0.296	0.494	1.340	0.193	0.479	1.262	0.262
17741	0.479	1.262	0.467	0.494	1.340	0.273	0.503	1.413	0.481
17742	0.097	-1.181	0.296	0.234	-0.336	0.053	0.224	-0.373	0.296
17743	0.263	-0.120	0.262	0.149	-0.882	0.444	0.142	-0.895	0.328
17744	0.142	-0.895	0.492	0.149	-0.882	0.478	0.326	0.285	0.467
17745	0.411	0.824	0.450	0.442	1.001	0.508	0.505	1.430	0.185
17746	0.408	0.807	0.450	0.404	0.756	0.398	0.500	1.397	0.185
17747	0.226	-0.356	0.450	0.307	0.135	0.235	0.097	-1.181	0.409

17748	0.261	-0.137	0.467	0.444	1.020	0.512	0.300	0.116	0.358
17749	0.511	1.464	0.224	0.500	1.378	0.053	0.463	1.161	0.262
17750	0.511	1.464	0.431	0.500	1.378	0.193	0.463	1.161	0.431
17751	0.261	-0.137	0.431	0.102	-1.184	0.193	0.187	-0.608	0.492
17752	0.508	1.447	0.431	0.509	1.434	0.341	0.479	1.262	0.050
17753	0.516	1.498	0.467	0.480	1.246	0.309	0.505	1.430	0.050
17754	0.050	-1.485	0.224	0.275	-0.073	0.309	0.358	0.487	0.224
17755	0.050	-1.485	0.224	0.345	0.379	0.309	0.358	0.487	0.358
17756	0.511	1.464	0.224	0.503	1.396	0.508	0.500	1.397	0.467
17757	0.300	0.116	0.185	0.371	0.549	0.309	0.184	-0.625	0.262
17758	0.450	1.076	0.481	0.234	-0.336	0.514	0.508	1.447	0.224
17759	0.295	0.082	0.262	0.193	-0.600	0.309	0.261	-0.137	0.296
17760	0.261	-0.137	0.296	0.149	-0.882	0.491	0.261	-0.137	0.450
17761	0.297	0.099	0.296	0.196	-0.581	0.422	0.263	-0.120	0.492
17762	0.266	-0.103	0.481	0.275	-0.073	0.273	0.184	-0.625	0.097
17763	0.142	-0.895	0.142	0.053	-1.504	0.501	0.332	0.318	0.097
17764	0.492	1.346	0.185	0.439	0.982	0.235	0.466	1.178	0.501
17765	0.503	1.413	0.296	0.474	1.208	0.508	0.508	1.447	0.385
17766	0.466	1.178	0.409	0.509	1.434	0.508	0.482	1.279	0.224
17767	0.495	1.363	0.409	0.395	0.700	0.514	0.511	1.464	0.142
17768	0.295	0.082	0.385	0.234	-0.336	0.273	0.187	-0.608	0.512
17769	0.387	0.672	0.262	0.512	1.453	0.309	0.432	0.958	0.097
17770	0.224	-0.373	0.262	0.053	-1.504	0.478	0.329	0.301	0.185
17771	0.261	-0.137	0.185	0.193	-0.600	0.478	0.384	0.655	0.450
17772	0.261	-0.137	0.501	0.193	-0.600	0.512	0.384	0.655	0.467
17773	0.382	0.638	0.501	0.345	0.379	0.514	0.358	0.487	0.501
17774	0.295	0.082	0.501	0.313	0.172	0.422	0.000	?	0.501
17775	0.226	-0.356	0.224	0.310	0.153	0.309	0.261	-0.137	0.492
17776	0.226	-0.356	0.467	0.310	0.153	0.462	0.261	-0.137	0.224
17777	0.487	1.312	0.450	0.503	1.396	0.309	0.484	1.295	0.481
17778	0.142	-0.895	0.513	0.307	0.135	0.501	0.363	0.520	0.481
17779	0.453	1.093	0.185	0.509	1.434	0.422	0.503	1.413	0.481
17780	0.184	-0.625	0.492	0.310	0.153	0.309	0.387	0.672	0.467
17781	0.429	0.942	0.185	0.494	1.340	0.341	0.489	1.329	0.142
17782	0.508	1.447	0.185	0.424	0.888	0.341	0.508	1.447	0.142
17783	0.097	-1.181	0.185	0.275	-0.073	0.273	0.226	-0.356	0.185
17784	0.487	1.312	0.296	0.497	1.359	0.508	0.468	1.194	0.097
17785	0.187	-0.608	0.492	0.237	-0.317	0.491	0.224	-0.373	0.508
17786	0.500	1.397	0.358	0.503	1.396	0.478	0.508	1.447	0.050
17787	0.492	1.346	0.385	0.503	1.396	0.508	0.408	0.807	0.142
17788	0.447	1.060	0.409	0.512	1.453	0.235	0.500	1.397	0.097
17789	0.329	0.301	0.097	0.272	-0.091	0.512	0.184	-0.625	0.450
17790	0.142	-0.895	0.508	0.310	0.153	0.235	0.187	-0.608	0.142
17791	0.505	1.430	0.224	0.477	1.227	0.235	0.505	1.430	0.097
17792	0.492	1.346	0.508	0.477	1.227	0.398	0.497	1.380	0.185
17793	0.408	0.807	0.296	0.512	1.453	0.444	0.484	1.295	0.513
17794	0.408	0.807	0.328	0.512	1.453	0.149	0.511	1.464	0.512
17795	0.505	1.430	0.481	0.421	0.869	0.149	0.503	1.413	0.513
17796	0.050	-1.485	0.185	0.313	0.172	0.462	0.355	0.470	0.142
17797	0.497	1.380	0.492	0.459	1.114	0.149	0.466	1.178	0.142
17798	0.000	?	0.513	0.310	0.153	0.309	0.295	0.082	0.185
17799	0.508	1.447	0.467	0.500	1.378	0.478	0.382	0.638	0.492
17800	0.382	0.638	0.501	0.427	0.907	0.512	0.516	1.498	0.431
17801	0.097	-1.181	0.501	0.310	0.153	0.398	0.184	-0.625	0.467
17802	0.226	-0.356	0.328	0.342	0.361	0.501	0.226	-0.356	0.224
17803	0.187	-0.608	0.224	0.342	0.361	0.149	0.187	-0.608	0.481
17804	0.184	-0.625	0.431	0.272	-0.091	0.491	0.097	-1.181	0.450
17805	0.463	1.161	0.492	0.509	1.434	0.501	0.408	0.807	0.492
17806	0.511	1.464	0.467	0.494	1.340	0.273	0.453	1.093	0.142
17807	0.450	1.076	0.185	0.477	1.227	0.478	0.461	1.144	0.512
17808	0.476	1.245	0.481	0.503	1.396	0.309	0.479	1.262	0.262

17809	0.142	-0.895	0.501	0.237	-0.317	0.309	0.226	-0.356	0.262
17810	0.489	1.329	0.185	0.509	1.434	0.309	0.513	1.481	0.224
17811	0.226	-0.356	0.296	0.234	-0.336	0.341	0.297	0.099	0.185
17812	0.226	-0.356	0.481	0.234	-0.336	0.341	0.297	0.099	0.508
17813	0.453	1.093	0.224	0.398	0.718	0.398	0.492	1.346	0.185
17814	0.508	1.447	0.185	0.439	0.982	0.273	0.466	1.178	0.481
17815	0.097	-1.181	0.185	0.149	-0.882	0.235	0.297	0.099	0.431
17816	0.224	-0.373	0.450	0.152	-0.863	0.491	0.297	0.099	0.185
17817	0.429	0.942	0.296	0.462	1.133	0.309	0.500	1.397	0.492
17818	0.224	-0.373	0.467	0.149	-0.882	0.193	0.389	0.689	0.097
17819	0.097	-1.181	0.142	0.307	0.135	0.512	0.187	-0.608	0.450
17820	0.384	0.655	0.097	0.474	1.208	0.341	0.505	1.430	0.328
17821	0.466	1.178	0.508	0.509	1.434	0.508	0.487	1.312	0.050
17822	0.261	-0.137	0.142	0.401	0.737	0.501	0.326	0.285	0.385
17823	0.505	1.430	0.512	0.503	1.396	0.462	0.492	1.346	0.185
17824	0.142	-0.895	0.492	0.149	-0.882	0.235	0.261	-0.137	0.431
17825	0.447	1.060	0.431	0.491	1.321	0.462	0.492	1.346	0.501
17826	0.453	1.093	0.262	0.500	1.378	0.512	0.445	1.043	0.508
17827	0.187	-0.608	0.224	0.275	-0.073	0.398	0.329	0.301	0.431
17828	0.503	1.413	0.262	0.480	1.246	0.102	0.429	0.942	0.431
17829	0.226	-0.356	0.508	0.310	0.153	0.491	0.355	0.470	0.262
17830	0.224	-0.373	0.508	0.310	0.153	0.514	0.300	0.116	0.185
17831	0.142	-0.895	0.492	0.310	0.153	0.508	0.297	0.099	0.512
17832	0.326	0.285	0.185	0.342	0.361	0.193	0.387	0.672	0.431
17833	0.326	0.285	0.262	0.342	0.361	0.501	0.432	0.958	0.501
17834	0.263	-0.120	0.224	0.398	0.718	0.273	0.263	-0.120	0.224
17835	0.295	0.082	0.142	0.275	-0.073	0.422	0.224	-0.373	0.481
17836	0.224	-0.373	0.481	0.234	-0.336	0.508	0.266	-0.103	0.481
17837	0.492	1.346	0.501	0.485	1.283	0.501	0.505	1.430	0.185
17838	0.145	-0.878	0.431	0.307	0.135	0.478	0.224	-0.373	0.185
17839	0.187	-0.608	0.328	0.196	-0.581	0.193	0.326	0.285	0.467
17840	0.511	1.464	0.185	0.515	1.472	0.462	0.447	1.060	0.185
17841	0.184	-0.625	0.512	0.345	0.379	0.444	0.295	0.082	0.142
17842	0.482	1.279	0.492	0.512	1.453	0.053	0.503	1.413	0.142
17843	0.487	1.312	0.185	0.503	1.396	0.512	0.505	1.430	0.513
17844	0.487	1.312	0.224	0.468	1.170	0.512	0.508	1.447	0.050
17845	0.184	-0.625	0.050	0.237	-0.317	0.235	0.142	-0.895	0.508
17846	0.508	1.447	0.431	0.462	1.133	0.501	0.387	0.672	0.050
17847	0.503	1.413	0.431	0.509	1.434	0.478	0.500	1.397	0.224
17848	0.508	1.447	0.296	0.398	0.718	0.478	0.511	1.464	0.508
17849	0.142	-0.895	0.262	0.102	-1.184	0.512	0.295	0.082	0.185
17850	0.476	1.245	0.224	0.494	1.340	0.053	0.484	1.295	0.508
17851	0.497	1.380	0.296	0.515	1.472	0.514	0.505	1.430	0.512
17852	0.463	1.161	0.262	0.509	1.434	0.371	0.484	1.295	0.513
17853	0.142	-0.895	0.508	0.193	-0.600	0.422	0.434	0.975	0.097
17854	0.503	1.413	0.508	0.497	1.359	0.235	0.466	1.178	0.050
17855	0.332	0.318	0.358	0.272	-0.091	0.491	0.332	0.318	0.050
17856	0.226	-0.356	0.450	0.427	0.907	0.478	0.224	-0.373	0.431
17857	0.429	0.942	0.450	0.509	1.434	0.235	0.503	1.413	0.450
17858	0.458	1.127	0.224	0.500	1.378	0.478	0.489	1.329	0.501
17859	0.492	1.346	0.508	0.480	1.246	0.398	0.511	1.464	0.296
17860	0.261	-0.137	0.224	0.193	-0.600	0.478	0.224	-0.373	0.142
17861	0.461	1.144	0.450	0.459	1.114	0.514	0.511	1.464	0.185
17862	0.358	0.487	0.481	0.444	1.020	0.491	0.297	0.099	0.224
17863	0.184	-0.625	0.224	0.053	-1.504	0.491	0.355	0.470	0.450
17864	0.518	1.514	0.431	0.515	1.472	0.235	0.450	1.076	0.508
17865	0.518	1.514	0.262	0.515	1.472	0.508	0.450	1.076	0.512
17866	0.142	-0.895	0.481	0.234	-0.336	0.341	0.187	-0.608	0.142
17867	0.503	1.413	0.512	0.503	1.396	0.422	0.505	1.430	0.296
17868	0.508	1.447	0.185	0.477	1.227	0.422	0.505	1.430	0.467
17869	0.508	1.447	0.512	0.477	1.227	0.422	0.505	1.430	0.508

17870	0.479	1.262	0.492	0.512	1.453	0.444	0.500	1.397	0.142
17871	0.266	-0.103	0.385	0.053	-1.504	0.508	0.224	-0.373	0.328
17872	0.511	1.464	0.185	0.518	1.491	0.501	0.503	1.413	0.467
17873	0.295	0.082	0.262	0.371	0.549	0.309	0.297	0.099	0.481
17874	0.511	1.464	0.185	0.424	0.888	0.514	0.453	1.093	0.142
17875	0.411	0.824	0.467	0.237	-0.317	0.193	0.413	0.841	0.097
17876	0.447	1.060	0.050	0.485	1.283	0.341	0.500	1.397	0.097
17877	0.489	1.329	0.050	0.477	1.227	0.235	0.479	1.262	0.097
17878	0.332	0.318	0.262	0.234	-0.336	0.273	0.263	-0.120	0.512
17879	0.482	1.279	0.492	0.474	1.208	0.102	0.466	1.178	0.492
17880	0.358	0.487	0.481	0.404	0.756	0.501	0.224	-0.373	0.224
17881	0.184	-0.625	0.501	0.482	1.265	0.501	0.361	0.504	0.512
17882	0.516	1.498	0.501	0.515	1.472	0.193	0.434	0.975	0.512
17883	0.495	1.363	0.501	0.488	1.302	0.341	0.466	1.178	0.224
17884	0.476	1.245	0.501	0.488	1.302	0.444	0.489	1.329	0.501
17885	0.142	-0.895	0.501	0.237	-0.317	0.512	0.450	1.076	0.224
17886	0.476	1.245	0.224	0.509	1.434	0.512	0.432	0.958	0.512
17887	0.261	-0.137	0.185	0.345	0.379	0.491	0.145	-0.878	0.508
17888	0.508	1.447	0.409	0.427	0.907	0.512	0.466	1.178	0.262
17889	0.508	1.447	0.296	0.427	0.907	0.309	0.463	1.161	0.142
17890	0.432	0.958	0.481	0.430	0.926	0.341	0.466	1.178	0.328
17891	0.476	1.245	0.328	0.442	1.001	0.444	0.503	1.413	0.508
17892	0.505	1.430	0.409	0.497	1.359	0.478	0.468	1.194	0.492
17893	0.489	1.329	0.501	0.500	1.378	0.422	0.516	1.498	0.296
17894	0.184	-0.625	0.501	0.313	0.172	0.235	0.097	-1.181	0.431
17895	0.484	1.295	0.508	0.512	1.453	0.235	0.513	1.481	0.492
17896	0.142	-0.895	0.262	0.193	-0.600	0.512	0.297	0.099	0.142
17897	0.184	-0.625	0.262	0.342	0.361	0.149	0.226	-0.356	0.097
17898	0.184	-0.625	0.296	0.237	-0.317	0.149	0.297	0.099	0.328
17899	0.229	-0.339	0.492	0.272	-0.091	0.508	0.050	-1.485	0.185
17900	0.142	-0.895	0.431	0.102	-1.184	0.193	0.329	0.301	0.467
17901	0.482	1.279	0.492	0.503	1.396	0.512	0.411	0.824	0.358
17902	0.511	1.464	0.481	0.494	1.340	0.235	0.447	1.060	0.328
17903	0.184	-0.625	0.328	0.193	-0.600	0.149	0.184	-0.625	0.262
17904	0.358	0.487	0.481	0.342	0.361	0.478	0.263	-0.120	0.467
17905	0.505	1.430	0.481	0.453	1.076	0.273	0.487	1.312	0.512
17906	0.468	1.194	0.262	0.515	1.472	0.273	0.508	1.447	0.450
17907	0.468	1.194	0.328	0.515	1.472	0.508	0.508	1.447	0.358
17908	0.476	1.245	0.328	0.497	1.359	0.193	0.505	1.430	0.431
17909	0.500	1.397	0.481	0.515	1.472	0.235	0.482	1.279	0.431
17910	0.329	0.301	0.481	0.310	0.153	0.491	0.329	0.301	0.501
17911	0.097	-1.181	0.492	0.342	0.361	0.491	0.266	-0.103	0.050
17912	0.329	0.301	0.385	0.000	?	0.273	0.329	0.301	0.142
17913	0.505	1.430	0.262	0.442	1.001	0.273	0.466	1.178	0.508
17914	0.482	1.279	0.508	0.474	1.208	0.398	0.503	1.413	0.142
17915	0.487	1.312	0.262	0.424	0.888	0.053	0.508	1.447	0.328
17916	0.300	0.116	0.142	0.234	-0.336	0.478	0.484	1.295	0.512
17917	0.300	0.116	0.481	0.234	-0.336	0.309	0.484	1.295	0.508
17918	0.497	1.380	0.492	0.509	1.434	0.235	0.450	1.076	0.450
17919	0.263	-0.120	0.385	0.149	-0.882	0.235	0.263	-0.120	0.512
17920	0.263	-0.120	0.492	0.149	-0.882	0.478	0.263	-0.120	0.050
17921	0.447	1.060	0.481	0.503	1.396	0.444	0.479	1.262	0.097
17922	0.097	-1.181	0.450	0.193	-0.600	0.193	0.266	-0.103	0.097
17923	0.508	1.447	0.512	0.509	1.434	0.273	0.482	1.279	0.513
17924	0.297	0.099	0.262	0.234	-0.336	0.508	0.297	0.099	0.501
17925	0.184	-0.625	0.467	0.149	-0.882	0.501	0.329	0.301	0.501
17926	0.503	1.413	0.508	0.482	1.265	0.149	0.468	1.194	0.492
17927	0.184	-0.625	0.296	0.275	-0.073	0.149	0.184	-0.625	0.224
17928	0.184	-0.625	0.467	0.275	-0.073	0.512	0.184	-0.625	0.481
17929	0.484	1.295	0.328	0.512	1.453	0.341	0.408	0.807	0.481
17930	0.266	-0.103	0.481	0.193	-0.600	0.149	0.355	0.470	0.142

17931	0.097	-1.181	0.185	0.234	-0.336	0.512	0.261	-0.137	0.097
17932	0.503	1.413	0.431	0.494	1.340	0.501	0.434	0.975	0.097
17933	0.466	1.178	0.296	0.491	1.321	0.508	0.495	1.363	0.481
17934	0.226	-0.356	0.224	0.275	-0.073	0.508	0.050	-1.485	0.481
17935	0.226	-0.356	0.431	0.275	-0.073	0.512	0.050	-1.485	0.492
17936	0.224	-0.373	0.185	0.401	0.737	0.102	0.226	-0.356	0.142
17937	0.097	-1.181	0.296	0.053	-1.504	0.501	0.355	0.470	0.097
17938	0.476	1.245	0.492	0.477	1.227	0.501	0.466	1.178	0.467
17939	0.384	0.655	0.481	0.307	0.135	0.501	0.263	-0.120	0.142
17940	0.184	-0.625	0.512	0.234	-0.336	0.508	0.224	-0.373	0.450
17941	0.384	0.655	0.328	0.234	-0.336	0.273	0.297	0.099	0.513
17942	0.513	1.481	0.296	0.477	1.227	0.273	0.445	1.043	0.050
17943	0.513	1.481	0.501	0.444	1.020	0.193	0.487	1.312	0.508
17944	0.329	0.301	0.328	0.193	-0.600	0.273	0.097	-1.181	0.450
17945	0.226	-0.356	0.492	0.275	-0.073	0.422	0.097	-1.181	0.508
17946	0.503	1.413	0.328	0.506	1.415	0.309	0.492	1.346	0.481
17947	0.450	1.076	0.262	0.500	1.378	0.273	0.382	0.638	0.050
17948	0.329	0.301	0.262	0.149	-0.882	0.149	0.224	-0.373	0.492
17949	0.297	0.099	0.492	0.149	-0.882	0.478	0.142	-0.895	0.481
17950	0.463	1.161	0.224	0.512	1.453	0.273	0.505	1.430	0.097
17951	0.297	0.099	0.185	0.339	0.342	0.273	0.097	-1.181	0.450
17952	0.263	-0.120	0.224	0.149	-0.882	0.398	0.184	-0.625	0.142
17953	0.489	1.329	0.467	0.512	1.453	0.508	0.447	1.060	0.481
17954	0.450	1.076	0.142	0.506	1.415	0.501	0.466	1.178	0.224
17955	0.450	1.076	0.467	0.509	1.434	0.508	0.503	1.413	0.481
17956	0.450	1.076	0.185	0.509	1.434	0.235	0.503	1.413	0.185
17957	0.468	1.194	0.385	0.509	1.434	0.235	0.489	1.329	0.097
17958	0.226	-0.356	0.508	0.102	-1.184	0.273	0.224	-0.373	0.431
17959	0.429	0.942	0.142	0.503	1.396	0.422	0.476	1.245	0.185
17960	0.429	0.942	0.296	0.503	1.396	0.501	0.476	1.245	0.185
17961	0.429	0.942	0.492	0.503	1.396	0.462	0.476	1.245	0.450
17962	0.466	1.178	0.501	0.506	1.415	0.102	0.463	1.161	0.431
17963	0.224	-0.373	0.450	0.275	-0.073	0.508	0.142	-0.895	0.431
17964	0.224	-0.373	0.492	0.275	-0.073	0.512	0.142	-0.895	0.262
17965	0.224	-0.373	0.492	0.196	-0.581	0.444	0.184	-0.625	0.385
17966	0.184	-0.625	0.328	0.272	-0.091	0.235	0.097	-1.181	0.142
17967	0.476	1.245	0.492	0.424	0.888	0.501	0.503	1.413	0.492
17968	0.263	-0.120	0.142	0.310	0.153	0.149	0.050	-1.485	0.142
17969	0.295	0.082	0.513	0.275	-0.073	0.149	0.142	-0.895	0.142
17970	0.297	0.099	0.467	0.152	-0.863	0.149	0.097	-1.181	0.262
17971	0.482	1.279	0.296	0.482	1.265	0.149	0.445	1.043	0.385
17972	0.142	-0.895	0.431	0.275	-0.073	0.422	0.142	-0.895	0.262
17973	0.187	-0.608	0.224	0.272	-0.091	0.273	0.097	-1.181	0.262
17974	0.297	0.099	0.501	0.398	0.718	0.491	0.187	-0.608	0.050
17975	0.413	0.841	0.481	0.506	1.415	0.508	0.505	1.430	0.050
17976	0.413	0.841	0.142	0.500	1.378	0.273	0.505	1.430	0.467
17977	0.387	0.672	0.512	0.506	1.415	0.462	0.505	1.430	0.296
17978	0.263	-0.120	0.508	0.237	-0.317	0.235	0.142	-0.895	0.450
17979	0.263	-0.120	0.142	0.237	-0.317	0.478	0.142	-0.895	0.097
17980	0.184	-0.625	0.328	0.272	-0.091	0.102	0.184	-0.625	0.512
17981	0.500	1.397	0.512	0.421	0.869	0.193	0.492	1.346	0.467
17982	0.500	1.397	0.512	0.500	1.378	0.501	0.426	0.925	0.142
17983	0.497	1.380	0.262	0.462	1.133	0.235	0.463	1.161	0.224
17984	0.226	-0.356	0.224	0.102	-1.184	0.478	0.224	-0.373	0.467
17985	0.463	1.161	0.450	0.506	1.415	0.478	0.479	1.262	0.492
17986	0.453	1.093	0.481	0.509	1.434	0.478	0.445	1.043	0.467
17987	0.511	1.464	0.481	0.444	1.020	0.514	0.484	1.295	0.409
17988	0.184	-0.625	0.508	0.234	-0.336	0.514	0.142	-0.895	0.508
17989	0.487	1.312	0.492	0.500	1.378	0.102	0.508	1.447	0.185
17990	0.187	-0.608	0.328	0.149	-0.882	0.309	0.263	-0.120	0.492
17991	0.187	-0.608	0.481	0.149	-0.882	0.491	0.263	-0.120	0.224

17992	0.187	-0.608	0.492	0.149	-0.882	0.478	0.226	-0.356	0.501
17993	0.297	0.099	0.224	0.149	-0.882	0.398	0.184	-0.625	0.450
17994	0.489	1.329	0.450	0.424	0.888	0.273	0.500	1.397	0.050
17995	0.358	0.487	0.492	0.272	-0.091	0.514	0.184	-0.625	0.501
17996	0.387	0.672	0.501	0.488	1.302	0.508	0.482	1.279	0.185
17997	0.408	0.807	0.185	0.509	1.434	0.309	0.432	0.958	0.431
17998	0.097	-1.181	0.431	0.275	-0.073	0.053	0.184	-0.625	0.513
17999	0.508	1.447	0.501	0.462	1.133	0.491	0.487	1.312	0.358
18000	0.224	-0.373	0.142	0.234	-0.336	0.273	0.097	-1.181	0.467
18001	0.508	1.447	0.328	0.477	1.227	0.235	0.445	1.043	0.508
18002	0.297	0.099	0.512	0.102	-1.184	0.235	0.329	0.301	0.050
18003	0.329	0.301	0.296	0.196	-0.581	0.444	0.050	-1.485	0.185
18004	0.479	1.262	0.262	0.500	1.378	0.273	0.382	0.638	0.481
18005	0.184	-0.625	0.481	0.234	-0.336	0.478	0.184	-0.625	0.481
18006	0.495	1.363	0.409	0.482	1.265	0.341	0.426	0.925	0.358
18007	0.505	1.430	0.097	0.474	1.208	0.273	0.503	1.413	0.262
18008	0.466	1.178	0.501	0.477	1.227	0.462	0.503	1.413	0.512
18009	0.503	1.413	0.142	0.506	1.415	0.235	0.426	0.925	0.501
18010	0.503	1.413	0.501	0.506	1.415	0.501	0.426	0.925	0.501
18011	0.332	0.318	0.097	0.102	-1.184	0.508	0.263	-0.120	0.492
18012	0.224	-0.373	0.467	0.310	0.153	0.478	0.187	-0.608	0.513
18013	0.429	0.942	0.467	0.491	1.321	0.235	0.508	1.447	0.224
18014	0.492	1.346	0.481	0.480	1.246	0.309	0.432	0.958	0.492
18015	0.463	1.161	0.492	0.398	0.718	0.309	0.500	1.397	0.508
18016	0.184	-0.625	0.450	0.272	-0.091	0.508	0.229	-0.339	0.224
18017	0.479	1.262	0.296	0.515	1.472	0.508	0.482	1.279	0.492
18018	0.505	1.430	0.224	0.503	1.396	0.478	0.474	1.228	0.492
18019	0.184	-0.625	0.142	0.310	0.153	0.235	0.184	-0.625	0.501
18020	0.297	0.099	0.508	0.053	-1.504	0.149	0.187	-0.608	0.185
18021	0.482	1.279	0.385	0.488	1.302	0.149	0.463	1.161	0.501
18022	0.226	-0.356	0.262	0.272	-0.091	0.193	0.184	-0.625	0.508
18023	0.187	-0.608	0.224	0.237	-0.317	0.508	0.142	-0.895	0.224
18024	0.187	-0.608	0.467	0.237	-0.317	0.422	0.142	-0.895	0.185
18025	0.447	1.060	0.467	0.447	1.039	0.501	0.511	1.464	0.501
18026	0.297	0.099	0.501	0.275	-0.073	0.235	0.050	-1.485	0.262
18027	0.466	1.178	0.481	0.477	1.227	0.193	0.505	1.430	0.185
18028	0.142	-0.895	0.224	0.345	0.379	0.501	0.050	-1.485	0.431
18029	0.097	-1.181	0.492	0.275	-0.073	0.491	0.226	-0.356	0.513
18030	0.505	1.430	0.467	0.459	1.114	0.341	0.505	1.430	0.262
18031	0.142	-0.895	0.185	0.234	-0.336	0.235	0.184	-0.625	0.467
18032	0.513	1.481	0.050	0.497	1.359	0.235	0.503	1.413	0.142
18033	0.489	1.329	0.409	0.506	1.415	0.501	0.508	1.447	0.481
18034	0.429	0.942	0.328	0.477	1.227	0.508	0.511	1.464	0.185
18035	0.261	-0.137	0.467	0.237	-0.317	0.235	0.097	-1.181	0.450
18036	0.224	-0.373	0.467	0.310	0.153	0.462	0.050	-1.485	0.450
18037	0.263	-0.120	0.481	0.310	0.153	0.273	0.050	-1.485	0.501
18038	0.505	1.430	0.467	0.509	1.434	0.149	0.429	0.942	0.501
18039	0.505	1.430	0.262	0.509	1.434	0.193	0.447	1.060	0.501
18040	0.487	1.312	0.328	0.485	1.283	0.501	0.503	1.413	0.185
18041	0.184	-0.625	0.481	0.234	-0.336	0.462	0.295	0.082	0.142
18042	0.263	-0.120	0.142	0.149	-0.882	0.235	0.142	-0.895	0.328
18043	0.226	-0.356	0.185	0.149	-0.882	0.422	0.187	-0.608	0.481
18044	0.142	-0.895	0.185	0.193	-0.600	0.422	0.224	-0.373	0.512
18045	0.476	1.245	0.492	0.503	1.396	0.193	0.453	1.093	0.050
18046	0.500	1.397	0.512	0.418	0.850	0.478	0.503	1.413	0.097
18047	0.434	0.975	0.385	0.503	1.396	0.235	0.505	1.430	0.513
18048	0.332	0.318	0.513	0.237	-0.317	0.501	0.142	-0.895	0.492
18049	0.184	-0.625	0.296	0.196	-0.581	0.422	0.295	0.082	0.467
18050	0.505	1.430	0.185	0.503	1.396	0.398	0.466	1.178	0.501
18051	0.495	1.363	0.262	0.491	1.321	0.341	0.497	1.380	0.296
18052	0.184	-0.625	0.262	0.345	0.379	0.309	0.000	?	0.431

18053	0.224	−0.373	0.142	0.234	−0.336	0.444	0.145	−0.878	0.513
18054	0.050	−1.485	0.185	0.237	−0.317	0.508	0.326	0.285	0.142
18055	0.429	0.942	0.142	0.500	1.378	0.193	0.463	1.161	0.409
18056	0.429	0.942	0.467	0.509	1.434	0.478	0.476	1.245	0.467
18057	0.300	0.116	0.409	0.234	−0.336	0.514	0.000	?	0.385
18058	0.261	−0.137	0.262	0.459	1.114	0.149	0.142	−0.895	0.508
18059	0.224	−0.373	0.501	0.275	−0.073	0.273	0.097	−1.181	0.508
18060	0.297	0.099	0.224	0.149	−0.882	0.149	0.097	−1.181	0.508
18061	0.263	−0.120	0.492	0.193	−0.600	0.309	0.097	−1.181	0.508
18062	0.508	1.447	0.481	0.497	1.359	0.462	0.508	1.447	0.262
18063	0.511	1.464	0.185	0.459	1.114	0.193	0.487	1.312	0.296
18064	0.361	0.504	0.262	0.234	−0.336	0.193	0.224	−0.373	0.467
18065	0.450	1.076	0.501	0.418	0.850	0.235	0.508	1.447	0.224
18066	0.450	1.076	0.501	0.418	0.850	0.508	0.508	1.447	0.224
18067	0.226	−0.356	0.508	0.193	−0.600	0.478	0.224	−0.373	0.224
18068	0.508	1.447	0.185	0.474	1.208	0.478	0.497	1.380	0.501
18069	0.226	−0.356	0.492	0.234	−0.336	0.514	0.224	−0.373	0.467
18070	0.447	1.060	0.185	0.497	1.359	0.501	0.508	1.447	0.185
18071	0.484	1.295	0.358	0.418	0.850	0.501	0.500	1.397	0.508
18072	0.224	−0.373	0.481	0.398	0.718	0.501	0.263	−0.120	0.185
18073	0.432	0.958	0.296	0.342	0.361	0.309	0.145	−0.878	0.224
18074	0.261	−0.137	0.224	0.313	0.172	0.235	0.332	0.318	0.262
18075	0.479	1.262	0.481	0.444	1.020	0.462	0.508	1.447	0.262
18076	0.513	1.481	0.185	0.503	1.396	0.149	0.495	1.363	0.050
18077	0.184	−0.625	0.328	0.196	−0.581	0.508	0.297	0.099	0.050
18078	0.513	1.481	0.224	0.482	1.265	0.371	0.429	0.942	0.142
18079	0.495	1.363	0.513	0.512	1.453	0.371	0.489	1.329	0.513
18080	0.384	0.655	0.262	0.149	−0.882	0.501	0.142	−0.895	0.467
18081	0.184	−0.625	0.296	0.272	−0.091	0.478	0.097	−1.181	0.097
18082	0.261	−0.137	0.508	0.149	−0.882	0.462	0.329	0.301	0.450
18083	0.184	−0.625	0.481	0.310	0.153	0.398	0.184	−0.625	0.097
18084	0.463	1.161	0.467	0.459	1.114	0.309	0.466	1.178	0.431
18085	0.050	−1.485	0.467	0.193	−0.600	0.235	0.355	0.470	0.512
18086	0.050	−1.485	0.501	0.193	−0.600	0.422	0.326	0.285	0.224
18087	0.263	−0.120	0.501	0.234	−0.336	0.508	0.261	−0.137	0.262
18088	0.492	1.346	0.512	0.509	1.434	0.501	0.461	1.144	0.450
18089	0.476	1.245	0.481	0.474	1.208	0.478	0.511	1.464	0.450
18090	0.497	1.380	0.481	0.477	1.227	0.193	0.455	1.110	0.501
18091	0.500	1.397	0.492	0.509	1.434	0.514	0.355	0.470	0.262
18092	0.497	1.380	0.481	0.500	1.378	0.508	0.437	0.992	0.481
18093	0.497	1.380	0.262	0.500	1.378	0.235	0.437	0.992	0.185
18094	0.495	1.363	0.185	0.500	1.378	0.149	0.500	1.397	0.262
18095	0.224	−0.373	0.185	0.313	0.172	0.149	0.050	−1.485	0.513
18096	0.184	−0.625	0.224	0.237	−0.317	0.444	0.142	−0.895	0.358
18097	0.405	0.790	0.467	0.462	1.133	0.444	0.511	1.464	0.358
18098	0.297	0.099	0.224	0.149	−0.882	0.478	0.142	−0.895	0.467
18099	0.479	1.262	0.296	0.509	1.434	0.149	0.326	0.285	0.262
18100	0.329	0.301	0.296	0.368	0.530	0.193	0.000	?	0.185
18101	0.408	0.807	0.512	0.368	0.530	0.478	0.511	1.464	0.142
18102	0.500	1.397	0.262	0.497	1.359	0.273	0.508	1.447	0.467
18103	0.497	1.380	0.508	0.477	1.227	0.309	0.450	1.076	0.142
18104	0.508	1.447	0.467	0.459	1.114	0.501	0.511	1.464	0.142
18105	0.263	−0.120	0.450	0.401	0.737	0.398	0.050	−1.485	0.481
18106	0.263	−0.120	0.450	0.307	0.135	0.371	0.097	−1.181	0.450
18107	0.295	0.082	0.467	0.237	−0.317	0.444	0.097	−1.181	0.512
18108	0.492	1.346	0.385	0.421	0.869	0.462	0.508	1.447	0.513
18109	0.432	0.958	0.492	0.506	1.415	0.501	0.500	1.397	0.467
18110	0.495	1.363	0.512	0.500	1.378	0.478	0.492	1.346	0.512
18111	0.479	1.262	0.508	0.477	1.227	0.309	0.487	1.312	0.508
18112	0.332	0.318	0.513	0.196	−0.581	0.512	0.224	−0.373	0.501
18113	0.479	1.262	0.224	0.512	1.453	0.462	0.476	1.245	0.492

18114	0.476	1.245	0.450	0.506	1.415	0.193	0.476	1.245	0.481
18115	0.261	-0.137	0.481	0.237	-0.317	0.491	0.142	-0.895	0.492
18116	0.332	0.318	0.296	0.149	-0.882	0.341	0.097	-1.181	0.296
18117	0.332	0.318	0.501	0.149	-0.882	0.508	0.097	-1.181	0.142
18118	0.476	1.245	0.501	0.444	1.020	0.193	0.479	1.262	0.450
18119	0.476	1.245	0.467	0.444	1.020	0.508	0.479	1.262	0.097
18120	0.489	1.329	0.450	0.480	1.246	0.273	0.484	1.295	0.501
18121	0.387	0.672	0.467	0.149	-0.882	0.235	0.142	-0.895	0.142
18122	0.261	-0.137	0.492	0.193	-0.600	0.508	0.097	-1.181	0.050
18123	0.508	1.447	0.492	0.474	1.208	0.235	0.461	1.144	0.050
18124	0.263	-0.120	0.492	0.272	-0.091	0.273	0.000	?	0.467
18125	0.142	-0.895	0.450	0.313	0.172	0.478	0.142	-0.895	0.262
18126	0.484	1.295	0.450	0.506	1.415	0.512	0.445	1.043	0.508
18127	0.492	1.346	0.262	0.401	0.737	0.422	0.508	1.447	0.409
18128	0.387	0.672	0.481	0.371	0.549	0.235	0.050	-1.485	0.492
18129	0.492	1.346	0.501	0.444	1.020	0.102	0.500	1.397	0.492
18130	0.482	1.279	0.224	0.462	1.133	0.491	0.445	1.043	0.512
18131	0.450	1.076	0.358	0.497	1.359	0.235	0.503	1.413	0.512
18132	0.508	1.447	0.513	0.477	1.227	0.341	0.476	1.245	0.512
18133	0.261	-0.137	0.513	0.313	0.172	0.273	0.050	-1.485	0.481
18134	0.466	1.178	0.224	0.509	1.434	0.273	0.495	1.363	0.450
18135	0.508	1.447	0.262	0.462	1.133	0.235	0.479	1.262	0.492
18136	0.300	0.116	0.481	0.193	-0.600	0.444	0.097	-1.181	0.262
18137	0.466	1.178	0.296	0.485	1.283	0.273	0.447	1.060	0.512
18138	0.329	0.301	0.262	0.342	0.361	0.193	0.142	-0.895	0.512
18139	0.479	1.262	0.262	0.506	1.415	0.309	0.476	1.245	0.097
18140	0.184	-0.625	0.508	0.193	-0.600	0.273	0.224	-0.373	0.501
18141	0.432	0.958	0.224	0.506	1.415	0.501	0.474	1.228	0.501
18142	0.297	0.099	0.501	0.275	-0.073	0.273	0.184	-0.625	0.508
18143	0.224	-0.373	0.097	0.234	-0.336	0.478	0.097	-1.181	0.508
18144	0.429	0.942	0.097	0.506	1.415	0.341	0.426	0.925	0.508
18145	0.184	-0.625	0.224	0.234	-0.336	0.491	0.184	-0.625	0.431
18146	0.297	0.099	0.467	0.275	-0.073	0.444	0.184	-0.625	0.431
18147	0.492	1.346	0.431	0.474	1.208	0.273	0.447	1.060	0.431
18148	0.482	1.279	0.328	0.509	1.434	0.235	0.429	0.942	0.501
18149	0.513	1.481	0.512	0.424	0.888	0.422	0.426	0.925	0.501
18150	0.329	0.301	0.185	0.237	-0.317	0.512	0.000	?	0.097
18151	0.297	0.099	0.481	0.102	-1.184	0.491	0.263	-0.120	0.450
18152	0.495	1.363	0.224	0.488	1.302	0.491	0.384	0.655	0.481
18153	0.329	0.301	0.385	0.234	-0.336	0.462	0.142	-0.895	0.097
18154	0.495	1.363	0.501	0.342	0.361	0.444	0.484	1.295	0.185
18155	0.329	0.301	0.481	0.275	-0.073	0.508	0.142	-0.895	0.512
18156	0.263	-0.120	0.513	0.275	-0.073	0.193	0.142	-0.895	0.508
18157	0.261	-0.137	0.481	0.237	-0.317	0.491	0.261	-0.137	0.097
18158	0.492	1.346	0.512	0.444	1.020	0.508	0.384	0.655	0.050
18159	0.000	?	0.492	0.275	-0.073	0.193	0.261	-0.137	0.481
18160	0.224	-0.373	0.492	0.196	-0.581	0.512	0.261	-0.137	0.097
18161	0.184	-0.625	0.481	0.307	0.135	0.273	0.050	-1.485	0.224
18162	0.226	-0.356	0.501	0.275	-0.073	0.491	0.050	-1.485	0.224
18163	0.466	1.178	0.385	0.494	1.340	0.422	0.466	1.178	0.450
18164	0.142	-0.895	0.501	0.275	-0.073	0.235	0.297	0.099	0.097
18165	0.468	1.194	0.185	0.480	1.246	0.512	0.447	1.060	0.512
18166	0.187	-0.608	0.185	0.342	0.361	0.462	0.097	-1.181	0.185
18167	0.382	0.638	0.431	0.488	1.302	0.341	0.505	1.430	0.224
18168	0.503	1.413	0.185	0.444	1.020	0.149	0.463	1.161	0.185
18169	0.142	-0.895	0.224	0.275	-0.073	0.491	0.142	-0.895	0.512
18170	0.300	0.116	0.431	0.234	-0.336	0.491	0.224	-0.373	0.481
18171	0.495	1.363	0.050	0.421	0.869	0.309	0.466	1.178	0.262
18172	0.497	1.380	0.467	0.515	1.472	0.053	0.497	1.380	0.409
18173	0.450	1.076	0.431	0.491	1.321	0.512	0.468	1.194	0.224
18174	0.495	1.363	0.492	0.488	1.302	0.491	0.408	0.807	0.501

18175	0.489	1.329	0.262	0.462	1.133	0.491	0.513	1.481	0.097
18176	0.326	0.285	0.262	0.447	1.039	0.444	0.184	−0.625	0.097
18177	0.492	1.346	0.501	0.503	1.396	0.444	0.492	1.346	0.501
18178	0.142	−0.895	0.481	0.196	−0.581	0.193	0.226	−0.356	0.512
18179	0.508	1.447	0.262	0.491	1.321	0.444	0.497	1.380	0.501
18180	0.463	1.161	0.450	0.506	1.415	0.462	0.447	1.060	0.513
18181	0.300	0.116	0.512	0.193	−0.600	0.273	0.050	−1.485	0.512
18182	0.429	0.942	0.512	0.509	1.434	0.462	0.503	1.413	0.492
18183	0.224	−0.373	0.431	0.275	−0.073	0.444	0.184	−0.625	0.492
18184	0.503	1.413	0.467	0.491	1.321	0.444	0.426	0.925	0.481
18185	0.479	1.262	0.262	0.418	0.850	0.273	0.511	1.464	0.512
18186	0.142	−0.895	0.450	0.240	−0.299	0.491	0.358	0.487	0.296
18187	0.508	1.447	0.467	0.512	1.453	0.398	0.461	1.144	0.513
18188	0.505	1.430	0.508	0.465	1.152	0.273	0.505	1.430	0.224
18189	0.142	−0.895	0.224	0.342	0.361	0.102	0.050	−1.485	0.224
18190	0.329	0.301	0.262	0.152	−0.863	0.398	0.184	−0.625	0.431
18191	0.516	1.498	0.467	0.494	1.340	0.053	0.479	1.262	0.296
18192	0.516	1.498	0.328	0.494	1.340	0.102	0.479	1.262	0.224
18193	0.263	−0.120	0.262	0.310	0.153	0.514	0.361	0.504	0.501
18194	0.224	−0.373	0.450	0.053	−1.504	0.491	0.261	−0.137	0.185
18195	0.453	1.093	0.296	0.518	1.491	0.193	0.513	1.481	0.512
18196	0.476	1.245	0.296	0.485	1.283	0.444	0.503	1.413	0.508
18197	0.476	1.245	0.224	0.488	1.302	0.273	0.503	1.413	0.450
18198	0.508	1.447	0.185	0.442	1.001	0.444	0.484	1.295	0.185
18199	0.495	1.363	0.481	0.442	1.001	0.309	0.503	1.413	0.185
18200	0.329	0.301	0.296	0.193	−0.600	0.491	0.226	−0.356	0.481
18201	0.479	1.262	0.492	0.444	1.020	0.478	0.489	1.329	0.467
18202	0.495	1.363	0.450	0.465	1.152	0.462	0.503	1.413	0.185
18203	0.226	−0.356	0.512	0.272	−0.091	0.444	0.229	−0.339	0.501
18204	0.450	1.076	0.512	0.462	1.133	0.444	0.492	1.346	0.508
18205	0.489	1.329	0.508	0.444	1.020	0.193	0.487	1.312	0.501
18206	0.497	1.380	0.224	0.444	1.020	0.422	0.497	1.380	0.296
18207	0.184	−0.625	0.431	0.272	−0.091	0.273	0.184	−0.625	0.501
18208	0.429	0.942	0.328	0.491	1.321	0.478	0.495	1.363	0.224
18209	0.500	1.397	0.513	0.398	0.718	0.491	0.503	1.413	0.481
18210	0.142	−0.895	0.467	0.275	−0.073	0.273	0.224	−0.373	0.501
18211	0.326	0.285	0.142	0.102	−1.184	0.341	0.184	−0.625	0.508
18212	0.508	1.447	0.385	0.398	0.718	0.462	0.500	1.397	0.262
18213	0.297	0.099	0.185	0.053	−1.504	0.508	0.261	−0.137	0.262
18214	0.261	−0.137	0.296	0.102	−1.184	0.501	0.184	−0.625	0.431
18215	0.479	1.262	0.097	0.509	1.434	0.491	0.426	0.925	0.296
18216	0.408	0.807	0.328	0.491	1.321	0.193	0.508	1.447	0.142
18217	0.097	−1.181	0.508	0.196	−0.581	0.512	0.261	−0.137	0.467
18218	0.503	1.413	0.467	0.444	1.020	0.422	0.461	1.144	0.262
18219	0.142	−0.895	0.262	0.275	−0.073	0.341	0.142	−0.895	0.296
18220	0.500	1.397	0.328	0.439	0.982	0.398	0.479	1.262	0.142
18221	0.097	−1.181	0.328	0.310	0.153	0.235	0.184	−0.625	0.097
18222	0.463	1.161	0.050	0.491	1.321	0.508	0.445	1.043	0.513
18223	0.463	1.161	0.508	0.480	1.246	0.508	0.445	1.043	0.224
18224	0.476	1.245	0.296	0.465	1.152	0.508	0.500	1.397	0.492
18225	0.489	1.329	0.050	0.444	1.020	0.508	0.500	1.397	0.492
18226	0.450	1.076	0.409	0.442	1.001	0.341	0.495	1.363	0.501
18227	0.297	0.099	0.508	0.193	−0.600	0.371	0.187	−0.608	0.481
18228	0.224	−0.373	0.262	0.427	0.907	0.501	0.142	−0.895	0.481
18229	0.142	−0.895	0.224	0.272	−0.091	0.309	0.326	0.285	0.296
18230	0.508	1.447	0.262	0.477	1.227	0.273	0.476	1.245	0.512
18231	0.382	0.638	0.409	0.494	1.340	0.273	0.505	1.430	0.224
18232	0.261	−0.137	0.508	0.278	−0.054	0.462	0.050	−1.485	0.513
18233	0.224	−0.373	0.224	0.342	0.361	0.491	0.097	−1.181	0.512
18234	0.471	1.211	0.224	0.459	1.114	0.309	0.508	1.447	0.224
18235	0.466	1.178	0.224	0.506	1.415	0.491	0.489	1.329	0.262

18236	0.503	1.413	0.508	0.497	1.359	0.102	0.461	1.144	0.296
18237	0.482	1.279	0.431	0.485	1.283	0.193	0.503	1.413	0.409
18238	0.226	-0.356	0.050	0.193	-0.600	0.102	0.295	0.082	0.296
18239	0.489	1.329	0.097	0.509	1.434	0.102	0.434	0.975	0.185
18240	0.461	1.144	0.097	0.421	0.869	0.341	0.511	1.464	0.431
18241	0.184	-0.625	0.467	0.342	0.361	0.341	0.142	-0.895	0.467
18242	0.050	-1.485	0.262	0.000	?	0.309	0.405	0.790	0.262
18243	0.408	0.807	0.185	0.401	0.737	0.398	0.468	1.194	0.224
18244	0.332	0.318	0.481	0.237	-0.317	0.514	0.384	0.655	0.224
18245	0.468	1.194	0.050	0.506	1.415	0.193	0.505	1.430	0.328
18246	0.468	1.194	0.492	0.506	1.415	0.501	0.505	1.430	0.431
18247	0.482	1.279	0.262	0.506	1.415	0.341	0.505	1.430	0.224
18248	0.468	1.194	0.492	0.506	1.415	0.512	0.505	1.430	0.262
18249	0.261	-0.137	0.481	0.342	0.361	0.422	0.261	-0.137	0.467
18250	0.326	0.285	0.512	0.374	0.568	0.149	0.295	0.082	0.481
18251	0.479	1.262	0.492	0.500	1.378	0.149	0.463	1.161	0.224
18252	0.142	-0.895	0.224	0.307	0.135	0.501	0.226	-0.356	0.224
18253	0.184	-0.625	0.328	0.272	-0.091	0.514	0.226	-0.356	0.097
18254	0.187	-0.608	0.097	0.272	-0.091	0.398	0.226	-0.356	0.512
18255	0.495	1.363	0.409	0.465	1.152	0.102	0.505	1.430	0.512
18256	0.516	1.498	0.508	0.488	1.302	0.512	0.463	1.161	0.097
18257	0.387	0.672	0.501	0.310	0.153	0.235	0.187	-0.608	0.097
18258	0.513	1.481	0.467	0.488	1.302	0.273	0.505	1.430	0.097
18259	0.297	0.099	0.501	0.102	-1.184	0.508	0.184	-0.625	0.501
18260	0.187	-0.608	0.262	0.196	-0.581	0.501	0.224	-0.373	0.513
18261	0.261	-0.137	0.262	0.102	-1.184	0.102	0.263	-0.120	0.224
18262	0.261	-0.137	0.481	0.102	-1.184	0.309	0.263	-0.120	0.224
18263	0.142	-0.895	0.481	0.342	0.361	0.273	0.050	-1.485	0.224
18264	0.184	-0.625	0.508	0.342	0.361	0.508	0.050	-1.485	0.431
18265	0.142	-0.895	0.142	0.310	0.153	0.235	0.142	-0.895	0.262
18266	0.471	1.211	0.185	0.398	0.718	0.273	0.508	1.447	0.224
18267	0.408	0.807	0.481	0.515	1.472	0.501	0.461	1.144	0.512
18268	0.261	-0.137	0.142	0.193	-0.600	0.501	0.097	-1.181	0.262
18269	0.500	1.397	0.185	0.497	1.359	0.508	0.445	1.043	0.512
18270	0.224	-0.373	0.431	0.342	0.361	0.508	0.097	-1.181	0.185
18271	0.489	1.329	0.224	0.512	1.453	0.478	0.429	0.942	0.512
18272	0.479	1.262	0.224	0.421	0.869	0.501	0.508	1.447	0.492
18273	0.187	-0.608	0.512	0.149	-0.882	0.444	0.226	-0.356	0.492
18274	0.263	-0.120	0.296	0.149	-0.882	0.478	0.263	-0.120	0.481
18275	0.500	1.397	0.492	0.494	1.340	0.478	0.447	1.060	0.262
18276	0.503	1.413	0.224	0.512	1.453	0.508	0.447	1.060	0.224
18277	0.505	1.430	0.185	0.398	0.718	0.512	0.495	1.363	0.296
18278	0.184	-0.625	0.512	0.102	-1.184	0.341	0.263	-0.120	0.512
18279	0.489	1.329	0.512	0.503	1.396	0.341	0.482	1.279	0.409
18280	0.184	-0.625	0.262	0.234	-0.336	0.102	0.184	-0.625	0.492
18281	0.358	0.487	0.512	0.275	-0.073	0.273	0.261	-0.137	0.492
18282	0.482	1.279	0.512	0.506	1.415	0.371	0.516	1.498	0.492
18283	0.297	0.099	0.262	0.000	?	0.444	0.361	0.504	0.431
18284	0.224	-0.373	0.467	0.000	?	0.193	0.361	0.504	0.431
18285	0.476	1.245	0.481	0.497	1.359	0.193	0.468	1.194	0.481
18286	0.184	-0.625	0.501	0.105	-1.165	0.491	0.263	-0.120	0.467
18287	0.329	0.301	0.501	0.310	0.153	0.053	0.184	-0.625	0.512
18288	0.224	-0.373	0.481	0.275	-0.073	0.491	0.142	-0.895	0.262
18289	0.508	1.447	0.262	0.506	1.415	0.508	0.463	1.161	0.185
18290	0.261	-0.137	0.501	0.234	-0.336	0.508	0.142	-0.895	0.508
18291	0.295	0.082	0.431	0.272	-0.091	0.514	0.142	-0.895	0.328
18292	0.503	1.413	0.385	0.497	1.359	0.478	0.479	1.262	0.262
18293	0.476	1.245	0.358	0.497	1.359	0.491	0.447	1.060	0.262
18294	0.466	1.178	0.512	0.506	1.415	0.512	0.508	1.447	0.467
18295	0.466	1.178	0.512	0.506	1.415	0.444	0.511	1.464	0.097
18296	0.500	1.397	0.508	0.482	1.265	0.508	0.466	1.178	0.224

18297	0.500	1.397	0.501	0.500	1.378	0.514	0.511	1.464	0.481
18298	0.511	1.464	0.185	0.444	1.020	0.102	0.505	1.430	0.185
18299	0.479	1.262	0.481	0.482	1.265	0.508	0.503	1.413	0.467
18300	0.479	1.262	0.142	0.482	1.265	0.514	0.497	1.380	0.262
18301	0.492	1.346	0.185	0.503	1.396	0.149	0.476	1.245	0.185
18302	0.479	1.262	0.224	0.509	1.434	0.508	0.492	1.346	0.385
18303	0.263	−0.120	0.450	0.342	0.361	0.508	0.300	0.116	0.358
18304	0.184	−0.625	0.467	0.342	0.361	0.462	0.142	−0.895	0.224
18305	0.187	−0.608	0.467	0.102	−1.184	0.478	0.447	1.060	0.513
18306	0.224	−0.373	0.508	0.272	−0.091	0.514	0.097	−1.181	0.513
18307	0.461	1.144	0.296	0.374	0.568	0.508	0.497	1.380	0.050
18308	0.224	−0.373	0.508	0.444	1.020	0.508	0.145	−0.878	0.512
18309	0.297	0.099	0.508	0.193	−0.600	0.508	0.050	−1.485	0.431
18310	0.297	0.099	0.512	0.193	−0.600	0.462	0.050	−1.485	0.467
18311	0.513	1.481	0.501	0.491	1.321	0.462	0.463	1.161	0.467
18312	0.263	−0.120	0.185	0.053	−1.504	0.193	0.263	−0.120	0.501
18313	0.505	1.430	0.481	0.491	1.321	0.508	0.503	1.413	0.097
18314	0.466	1.178	0.262	0.512	1.453	0.501	0.408	0.807	0.450
18315	0.450	1.076	0.513	0.506	1.415	0.235	0.484	1.295	0.431
18316	0.447	1.060	0.512	0.509	1.434	0.149	0.492	1.346	0.185
18317	0.471	1.211	0.512	0.477	1.227	0.422	0.513	1.481	0.142
18318	0.387	0.672	0.224	0.491	1.321	0.422	0.503	1.413	0.467
18319	0.489	1.329	0.501	0.512	1.453	0.235	0.511	1.464	0.467
18320	0.508	1.447	0.358	0.444	1.020	0.235	0.479	1.262	0.467
18321	0.505	1.430	0.431	0.506	1.415	0.491	0.447	1.060	0.467
18322	0.513	1.481	0.481	0.506	1.415	0.273	0.492	1.346	0.385
18323	0.224	−0.373	0.481	0.102	−1.184	0.235	0.261	−0.137	0.467
18324	0.453	1.093	0.296	0.509	1.434	0.235	0.516	1.498	0.142
18325	0.484	1.295	0.481	0.515	1.472	0.508	0.513	1.481	0.296
18326	0.297	0.099	0.224	0.149	−0.882	0.235	0.097	−1.181	0.142
18327	0.497	1.380	0.224	0.509	1.434	0.478	0.500	1.397	0.492
18328	0.497	1.380	0.481	0.509	1.434	0.273	0.500	1.397	0.501
18329	0.466	1.178	0.481	0.465	1.152	0.273	0.500	1.397	0.492
18330	0.450	1.076	0.358	0.480	1.246	0.371	0.500	1.397	0.450
18331	0.466	1.178	0.467	0.509	1.434	0.514	0.503	1.413	0.467
18332	0.487	1.312	0.185	0.506	1.415	0.501	0.426	0.925	0.467
18333	0.487	1.312	0.185	0.506	1.415	0.341	0.426	0.925	0.467
18334	0.487	1.312	0.185	0.506	1.415	0.508	0.426	0.925	0.481
18335	0.447	1.060	0.450	0.459	1.114	0.371	0.503	1.413	0.501
18336	0.447	1.060	0.328	0.459	1.114	0.341	0.503	1.413	0.262
18337	0.263	−0.120	0.467	0.196	−0.581	0.235	0.097	−1.181	0.492
18338	0.482	1.279	0.467	0.503	1.396	0.053	0.445	1.043	0.224
18339	0.500	1.397	0.296	0.500	1.378	0.491	0.474	1.228	0.450
18340	0.224	−0.373	0.142	0.234	−0.336	0.491	0.097	−1.181	0.467
18341	0.361	0.504	0.262	0.149	−0.882	0.491	0.187	−0.608	0.467
18342	0.511	1.464	0.409	0.424	0.888	0.491	0.503	1.413	0.142
18343	0.511	1.464	0.513	0.424	0.888	0.462	0.500	1.397	0.481
18344	0.224	−0.373	0.050	0.240	−0.299	0.501	0.097	−1.181	0.142
18345	0.261	−0.137	0.185	0.234	−0.336	0.501	0.050	−1.485	0.467
18346	0.479	1.262	0.492	0.485	1.283	0.478	0.482	1.279	0.508
18347	0.295	0.082	0.492	0.275	−0.073	0.512	0.097	−1.181	0.512
18348	0.261	−0.137	0.142	0.234	−0.336	0.309	0.224	−0.373	0.185
18349	0.261	−0.137	0.513	0.234	−0.336	0.501	0.224	−0.373	0.508
18350	0.511	1.464	0.224	0.509	1.434	0.235	0.450	1.076	0.185
18351	0.224	−0.373	0.467	0.240	−0.299	0.235	0.097	−1.181	0.185
18352	0.500	1.397	0.508	0.477	1.227	0.444	0.505	1.430	0.481
18353	0.097	−1.181	0.296	0.275	−0.073	0.193	0.187	−0.608	0.481
18354	0.097	−1.181	0.358	0.275	−0.073	0.235	0.226	−0.356	0.142
18355	0.224	−0.373	0.358	0.377	0.587	0.491	0.184	−0.625	0.481
18356	0.463	1.161	0.296	0.515	1.472	0.371	0.508	1.447	0.142
18357	0.432	0.958	0.513	0.506	1.415	0.478	0.476	1.245	0.185

18358	0.329	0.301	0.501	0.345	0.379	0.478	0.263	-0.120	0.296
18359	0.505	1.430	0.185	0.509	1.434	0.508	0.408	0.807	0.492
18360	0.184	-0.625	0.142	0.374	0.568	0.462	0.226	-0.356	0.142
18361	0.479	1.262	0.513	0.339	0.342	0.462	0.497	1.380	0.501
18362	0.224	-0.373	0.467	0.237	-0.317	0.371	0.097	-1.181	0.501
18363	0.387	0.672	0.185	0.053	-1.504	0.501	0.097	-1.181	0.224
18364	0.500	1.397	0.481	0.485	1.283	0.149	0.505	1.430	0.328
18365	0.479	1.262	0.467	0.488	1.302	0.462	0.511	1.464	0.097
18366	0.513	1.481	0.224	0.488	1.302	0.341	0.500	1.397	0.481
18367	0.479	1.262	0.142	0.488	1.302	0.371	0.511	1.464	0.492
18368	0.511	1.464	0.501	0.456	1.095	0.273	0.513	1.481	0.224
18369	0.492	1.346	0.501	0.500	1.378	0.514	0.489	1.329	0.142
18370	0.492	1.346	0.224	0.500	1.378	0.149	0.489	1.329	0.481
18371	0.484	1.295	0.097	0.474	1.208	0.491	0.479	1.262	0.501
18372	0.497	1.380	0.358	0.512	1.453	0.512	0.513	1.481	0.097
18373	0.387	0.672	0.358	0.310	0.153	0.478	0.295	0.082	0.481
18374	0.497	1.380	0.481	0.500	1.378	0.193	0.508	1.447	0.097
18375	0.184	-0.625	0.431	0.237	-0.317	0.235	0.226	-0.356	0.481
18376	0.184	-0.625	0.450	0.237	-0.317	0.422	0.226	-0.356	0.492
18377	0.432	0.958	0.467	0.442	1.001	0.341	0.426	0.925	0.185
18378	0.184	-0.625	0.467	0.193	-0.600	0.235	0.300	0.116	0.142
18379	0.226	-0.356	0.467	0.234	-0.336	0.508	0.224	-0.373	0.142
18380	0.429	0.942	0.097	0.488	1.302	0.149	0.497	1.380	0.142
18381	0.050	-1.485	0.097	0.374	0.568	0.149	0.184	-0.625	0.467
18382	0.466	1.178	0.358	0.477	1.227	0.235	0.508	1.447	0.481
18383	0.432	0.958	0.450	0.477	1.227	0.273	0.505	1.430	0.185
18384	0.495	1.363	0.508	0.506	1.415	0.501	0.450	1.076	0.262
18385	0.263	-0.120	0.501	0.465	1.152	0.235	0.184	-0.625	0.450
18386	0.263	-0.120	0.501	0.465	1.152	0.512	0.184	-0.625	0.467
18387	0.505	1.430	0.224	0.374	0.568	0.514	0.476	1.245	0.224
18388	0.476	1.245	0.185	0.503	1.396	0.478	0.463	1.161	0.467
18389	0.261	-0.137	0.296	0.149	-0.882	0.462	0.184	-0.625	0.467
18390	0.450	1.076	0.296	0.465	1.152	0.444	0.503	1.413	0.185
18391	0.511	1.464	0.385	0.345	0.379	0.273	0.505	1.430	0.185
18392	0.511	1.464	0.501	0.374	0.568	0.478	0.500	1.397	0.492
18393	0.429	0.942	0.513	0.275	-0.073	0.149	0.295	0.082	0.492
18394	0.468	1.194	0.492	0.515	1.472	0.478	0.500	1.397	0.328
18395	0.261	-0.137	0.492	0.149	-0.882	0.422	0.224	-0.373	0.409
18396	0.453	1.093	0.142	0.488	1.302	0.193	0.474	1.228	0.050
18397	0.468	1.194	0.481	0.509	1.434	0.309	0.495	1.363	0.262
18398	0.503	1.413	0.097	0.477	1.227	0.273	0.505	1.430	0.328
18399	0.224	-0.373	0.185	0.193	-0.600	0.309	0.261	-0.137	0.481
18400	0.261	-0.137	0.512	0.234	-0.336	0.273	0.261	-0.137	0.467
18401	0.468	1.194	0.512	0.421	0.869	0.053	0.429	0.942	0.501
18402	0.326	0.285	0.512	0.345	0.379	0.478	0.295	0.082	0.513
18403	0.263	-0.120	0.492	0.237	-0.317	0.422	0.142	-0.895	0.513
18404	0.453	1.093	0.467	0.509	1.434	0.273	0.461	1.144	0.328
18405	0.297	0.099	0.508	0.149	-0.882	0.193	0.261	-0.137	0.296
18406	0.297	0.099	0.513	0.149	-0.882	0.102	0.295	0.082	0.097
18407	0.224	-0.373	0.513	0.237	-0.317	0.149	0.142	-0.895	0.450
18408	0.184	-0.625	0.481	0.275	-0.073	0.508	0.097	-1.181	0.513
18409	0.482	1.279	0.328	0.503	1.396	0.149	0.513	1.481	0.508
18410	0.295	0.082	0.501	0.234	-0.336	0.309	0.226	-0.356	0.508
18411	0.487	1.312	0.262	0.512	1.453	0.478	0.489	1.329	0.224
18412	0.447	1.060	0.501	0.515	1.472	0.508	0.492	1.346	0.185
18413	0.513	1.481	0.185	0.474	1.208	0.193	0.503	1.413	0.142
18414	0.511	1.464	0.262	0.462	1.133	0.273	0.482	1.279	0.050
18415	0.508	1.447	0.262	0.444	1.020	0.193	0.482	1.279	0.512
18416	0.226	-0.356	0.481	0.272	-0.091	0.462	0.297	0.099	0.431
18417	0.426	0.925	0.513	0.474	1.208	0.478	0.511	1.464	0.512
18418	0.332	0.318	0.328	0.149	-0.882	0.235	0.224	-0.373	0.501

18419	0.511	1.464	0.508	0.477	1.227	0.235	0.513	1.481	0.508
18420	0.466	1.178	0.358	0.418	0.850	0.235	0.511	1.464	0.185
18421	0.142	−0.895	0.501	0.193	−0.600	0.478	0.226	−0.356	0.467
18422	0.384	0.655	0.492	0.310	0.153	0.398	0.261	−0.137	0.185
18423	0.184	−0.625	0.508	0.275	−0.073	0.273	0.297	0.099	0.185
18424	0.297	0.099	0.508	0.313	0.172	0.273	0.408	0.807	0.467
18425	0.097	−1.181	0.481	0.272	−0.091	0.273	0.295	0.082	0.508
18426	0.329	0.301	0.492	0.053	−1.504	0.514	0.184	−0.625	0.508
18427	0.505	1.430	0.508	0.474	1.208	0.235	0.429	0.942	0.262
18428	0.468	1.194	0.508	0.421	0.869	0.273	0.468	1.194	0.262
18429	0.261	−0.137	0.508	0.275	−0.073	0.512	0.263	−0.120	0.501
18430	0.326	0.285	0.262	0.193	−0.600	0.235	0.226	−0.356	0.492
18431	0.329	0.301	0.508	0.102	−1.184	0.478	0.226	−0.356	0.431
18432	0.050	−1.485	0.185	0.152	−0.863	0.102	0.329	0.301	0.508
18433	0.503	1.413	0.512	0.512	1.453	0.444	0.426	0.925	0.508
18434	0.300	0.116	0.501	0.149	−0.882	0.508	0.224	−0.373	0.467
18435	0.050	−1.485	0.481	0.307	0.135	0.422	0.266	−0.103	0.185
18436	0.405	0.790	0.097	0.480	1.246	0.508	0.468	1.194	0.467
18437	0.508	1.447	0.492	0.503	1.396	0.102	0.479	1.262	0.262
18438	0.263	−0.120	0.142	0.193	−0.600	0.149	0.224	−0.373	0.481
18439	0.224	−0.373	0.501	0.272	−0.091	0.273	0.224	−0.373	0.142
18440	0.263	−0.120	0.097	0.193	−0.600	0.444	0.097	−1.181	0.097
18441	0.405	0.790	0.185	0.459	1.114	0.501	0.513	1.481	0.097
18442	0.508	1.447	0.481	0.480	1.246	0.462	0.508	1.447	0.512
18443	0.226	−0.356	0.185	0.234	−0.336	0.514	0.097	−1.181	0.467
18444	0.226	−0.356	0.501	0.234	−0.336	0.478	0.097	−1.181	0.097
18445	0.226	−0.356	0.501	0.234	−0.336	0.422	0.097	−1.181	0.450
18446	0.508	1.447	0.501	0.477	1.227	0.422	0.500	1.397	0.097
18447	0.429	0.942	0.501	0.395	0.700	0.512	0.508	1.447	0.431
18448	0.050	−1.485	0.508	0.275	−0.073	0.491	0.224	−0.373	0.481
18449	0.097	−1.181	0.512	0.275	−0.073	0.491	0.224	−0.373	0.492
18450	0.097	−1.181	0.512	0.275	−0.073	0.273	0.224	−0.373	0.481
18451	0.463	1.161	0.431	0.509	1.434	0.309	0.429	0.942	0.513
18452	0.263	−0.120	0.512	0.234	−0.336	0.462	0.261	−0.137	0.481
18453	0.184	−0.625	0.481	0.272	−0.091	0.193	0.226	−0.356	0.501
18454	0.484	1.295	0.185	0.509	1.434	0.273	0.513	1.481	0.501
18455	0.050	−1.485	0.409	0.234	−0.336	0.149	0.261	−0.137	0.512
18456	0.492	1.346	0.050	0.477	1.227	0.422	0.505	1.430	0.050
18457	0.261	−0.137	0.142	0.102	−1.184	0.235	0.184	−0.625	0.501
18458	0.489	1.329	0.050	0.444	1.020	0.371	0.508	1.447	0.224
18459	0.482	1.279	0.296	0.509	1.434	0.478	0.495	1.363	0.512
18460	0.508	1.447	0.142	0.424	0.888	0.273	0.489	1.329	0.513
18461	0.489	1.329	0.296	0.509	1.434	0.491	0.482	1.279	0.501
18462	0.224	−0.373	0.050	0.102	−1.184	0.193	0.263	−0.120	0.262
18463	0.329	0.301	0.512	0.149	−0.882	0.309	0.224	−0.373	0.492
18464	0.097	−1.181	0.513	0.272	−0.091	0.514	0.297	0.099	0.142
18465	0.413	0.841	0.481	0.442	1.001	0.508	0.503	1.413	0.450
18466	0.503	1.413	0.501	0.497	1.359	0.053	0.411	0.824	0.262
18467	0.495	1.363	0.481	0.462	1.133	0.462	0.489	1.329	0.097
18468	0.461	1.144	0.513	0.515	1.472	0.462	0.489	1.329	0.481
18469	0.495	1.363	0.492	0.480	1.246	0.273	0.492	1.346	0.224
18470	0.263	−0.120	0.492	0.424	0.888	0.422	0.434	0.975	0.513
18471	0.263	−0.120	0.262	0.424	0.888	0.501	0.434	0.975	0.467
18472	0.479	1.262	0.501	0.512	1.453	0.508	0.474	1.228	0.467
18473	0.484	1.295	0.501	0.488	1.302	0.508	0.466	1.178	0.508
18474	0.508	1.447	0.513	0.485	1.283	0.501	0.508	1.447	0.513
18475	0.142	−0.895	0.508	0.275	−0.073	0.193	0.263	−0.120	0.512
18476	0.184	−0.625	0.508	0.310	0.153	0.508	0.184	−0.625	0.513
18477	0.476	1.245	0.508	0.462	1.133	0.478	0.503	1.413	0.501
18478	0.142	−0.895	0.450	0.193	−0.600	0.149	0.329	0.301	0.481
18479	0.000	?	0.501	0.272	−0.091	0.193	0.261	−0.137	0.481

18480	0.184	-0.625	0.512	0.149	-0.882	0.512	0.263	-0.120	0.097
18481	0.429	0.942	0.224	0.421	0.869	0.512	0.463	1.161	0.512
18482	0.224	-0.373	0.512	0.234	-0.336	0.512	0.097	-1.181	0.262
18483	0.229	-0.339	0.508	0.371	0.549	0.508	0.224	-0.373	0.385
18484	0.513	1.481	0.513	0.477	1.227	0.193	0.479	1.262	0.262
18485	0.297	0.099	0.142	0.275	-0.073	0.444	0.184	-0.625	0.262
18486	0.489	1.329	0.262	0.494	1.340	0.273	0.463	1.161	0.358
18487	0.224	-0.373	0.481	0.196	-0.581	0.193	0.263	-0.120	0.296
18488	0.184	-0.625	0.224	0.310	0.153	0.193	0.184	-0.625	0.328
18489	0.505	1.430	0.512	0.512	1.453	0.341	0.387	0.672	0.492
18490	0.505	1.430	0.512	0.506	1.415	0.398	0.361	0.504	0.513
18491	0.263	-0.120	0.481	0.053	-1.504	0.512	0.224	-0.373	0.501
18492	0.513	1.481	0.501	0.465	1.152	0.462	0.511	1.464	0.492
18493	0.513	1.481	0.513	0.465	1.152	0.508	0.511	1.464	0.481
18494	0.261	-0.137	0.512	0.275	-0.073	0.512	0.050	-1.485	0.450
18495	0.466	1.178	0.481	0.421	0.869	0.512	0.508	1.447	0.508
18496	0.482	1.279	0.224	0.497	1.359	0.478	0.429	0.942	0.431
18497	0.503	1.413	0.512	0.503	1.396	0.422	0.463	1.161	0.262
18498	0.503	1.413	0.508	0.506	1.415	0.273	0.463	1.161	0.481
18499	0.479	1.262	0.262	0.500	1.378	0.491	0.497	1.380	0.481
18500	0.261	-0.137	0.512	0.193	-0.600	0.193	0.097	-1.181	0.501
18501	0.505	1.430	0.296	0.503	1.396	0.514	0.447	1.060	0.431
18502	0.429	0.942	0.501	0.480	1.246	0.462	0.429	0.942	0.450
18503	0.387	0.672	0.492	0.149	-0.882	0.478	0.184	-0.625	0.431
18504	0.363	0.520	0.492	0.193	-0.600	0.193	0.142	-0.895	0.508
18505	0.505	1.430	0.481	0.512	1.453	0.501	0.463	1.161	0.481
18506	0.505	1.430	0.185	0.512	1.453	0.273	0.463	1.161	0.513
18507	0.500	1.397	0.492	0.518	1.491	0.512	0.461	1.144	0.328
18508	0.503	1.413	0.492	0.512	1.453	0.501	0.466	1.178	0.467
18509	0.187	-0.608	0.512	0.193	-0.600	0.508	0.387	0.672	0.513
18510	0.482	1.279	0.185	0.447	1.039	0.149	0.466	1.178	0.508
18511	0.142	-0.895	0.501	0.272	-0.091	0.444	0.142	-0.895	0.185
18512	0.184	-0.625	0.501	0.193	-0.600	0.149	0.300	0.116	0.328
18513	0.226	-0.356	0.501	0.193	-0.600	0.149	0.145	-0.878	0.508
18514	0.450	1.076	0.492	0.342	0.361	0.508	0.489	1.329	0.262
18515	0.466	1.178	0.508	0.395	0.700	0.512	0.495	1.363	0.508
18516	0.466	1.178	0.508	0.512	1.453	0.273	0.487	1.312	0.467
18517	0.508	1.447	0.513	0.459	1.114	0.501	0.445	1.043	0.481
18518	0.297	0.099	0.512	0.506	1.415	0.309	0.463	1.161	0.492
18519	0.508	1.447	0.142	0.506	1.415	0.273	0.463	1.161	0.481
18520	0.508	1.447	0.508	0.506	1.415	0.053	0.463	1.161	0.492
18521	0.508	1.447	0.450	0.474	1.208	0.514	0.484	1.295	0.481
18522	0.497	1.380	0.450	0.421	0.869	0.149	0.503	1.413	0.328
18523	0.187	-0.608	0.508	0.275	-0.073	0.462	0.261	-0.137	0.467
18524	0.484	1.295	0.481	0.497	1.359	0.462	0.489	1.329	0.481
18525	0.261	-0.137	0.224	0.199	-0.562	0.273	0.224	-0.373	0.142
18526	0.513	1.481	0.185	0.520	1.509	0.149	0.450	1.076	0.481
18527	0.513	1.481	0.492	0.468	1.170	0.273	0.468	1.194	0.185
18528	0.513	1.481	0.508	0.482	1.265	0.462	0.468	1.194	0.508
18529	0.224	-0.373	0.097	0.193	-0.600	0.501	0.145	-0.878	0.492
18530	0.503	1.413	0.513	0.503	1.396	0.273	0.479	1.262	0.492
18531	0.358	0.487	0.508	0.272	-0.091	0.371	0.142	-0.895	0.492
18532	0.429	0.942	0.512	0.515	1.472	0.444	0.471	1.211	0.262
18533	0.482	1.279	0.097	0.497	1.359	0.444	0.503	1.413	0.513
18534	0.482	1.279	0.501	0.506	1.415	0.309	0.508	1.447	0.513
18535	0.300	0.116	0.431	0.149	-0.882	0.491	0.184	-0.625	0.492
18536	0.482	1.279	0.512	0.447	1.039	0.235	0.508	1.447	0.224
18537	0.226	-0.356	0.358	0.149	-0.882	0.444	0.184	-0.625	0.512
18538	0.226	-0.356	0.467	0.149	-0.882	0.491	0.184	-0.625	0.512
18539	0.482	1.279	0.262	0.515	1.472	0.193	0.479	1.262	0.512
18540	0.482	1.279	0.409	0.520	1.509	0.235	0.479	1.262	0.508

18541	0.358	0.487	0.409	0.272	-0.091	0.309	0.142	-0.895	0.512
18542	0.466	1.178	0.512	0.500	1.378	0.235	0.479	1.262	0.508
18543	0.184	-0.625	0.142	0.307	0.135	0.478	0.142	-0.895	0.492
18544	0.184	-0.625	0.512	0.272	-0.091	0.491	0.184	-0.625	0.501
18545	0.184	-0.625	0.512	0.053	-1.504	0.193	0.297	0.099	0.296
18546	0.447	1.060	0.512	0.518	1.491	0.149	0.489	1.329	0.512
18547	0.329	0.301	0.512	0.149	-0.882	0.444	0.142	-0.895	0.512
18548	0.468	1.194	0.050	0.465	1.152	0.462	0.495	1.363	0.512
18549	0.468	1.194	0.097	0.465	1.152	0.193	0.495	1.363	0.501
18550	0.297	0.099	0.262	0.275	-0.073	0.422	0.224	-0.373	0.492
18551	0.142	-0.895	0.501	0.152	-0.863	0.501	0.329	0.301	0.328
18552	0.263	-0.120	0.512	0.272	-0.091	0.193	0.097	-1.181	0.142
18553	0.411	0.824	0.185	0.462	1.133	0.341	0.474	1.228	0.385
18554	0.511	1.464	0.492	0.500	1.378	0.478	0.489	1.329	0.409
18555	0.050	-1.485	0.097	0.272	-0.091	0.462	0.224	-0.373	0.262
18556	0.184	-0.625	0.142	0.374	0.568	0.102	0.142	-0.895	0.262
18557	0.492	1.346	0.467	0.442	1.001	0.309	0.476	1.245	0.467
18558	0.492	1.346	0.508	0.442	1.001	0.235	0.500	1.397	0.501
18559	0.142	-0.895	0.481	0.310	0.153	0.193	0.097	-1.181	0.512
18560	0.508	1.447	0.512	0.491	1.321	0.462	0.479	1.262	0.262
18561	0.226	-0.356	0.501	0.237	-0.317	0.512	0.097	-1.181	0.481
18562	0.468	1.194	0.501	0.442	1.001	0.398	0.476	1.245	0.501
18563	0.513	1.481	0.501	0.482	1.265	0.444	0.489	1.329	0.481
18564	0.297	0.099	0.508	0.193	-0.600	0.422	0.184	-0.625	0.508
18565	0.363	0.520	0.508	0.234	-0.336	0.422	0.142	-0.895	0.467
18566	0.361	0.504	0.501	0.310	0.153	0.149	0.142	-0.895	0.097
18567	0.300	0.116	0.467	0.234	-0.336	0.149	0.142	-0.895	0.296
18568	0.511	1.464	0.481	0.480	1.246	0.235	0.468	1.194	0.512
18569	0.500	1.397	0.508	0.491	1.321	0.462	0.479	1.262	0.512
18570	0.184	-0.625	0.512	0.193	-0.600	0.501	0.187	-0.608	0.185
18571	0.145	-0.878	0.142	0.152	-0.863	0.501	0.261	-0.137	0.450
18572	0.508	1.447	0.142	0.444	1.020	0.462	0.447	1.060	0.508
18573	0.466	1.178	0.501	0.462	1.133	0.273	0.463	1.161	0.508
18574	0.184	-0.625	0.501	0.193	-0.600	0.235	0.224	-0.373	0.508
18575	0.479	1.262	0.492	0.418	0.850	0.273	0.466	1.178	0.358
18576	0.466	1.178	0.262	0.497	1.359	0.193	0.463	1.161	0.296
18577	0.224	-0.373	0.501	0.196	-0.581	0.398	0.184	-0.625	0.296
18578	0.142	-0.895	0.262	0.345	0.379	0.501	0.184	-0.625	0.481
18579	0.503	1.413	0.450	0.474	1.208	0.501	0.489	1.329	0.409
18580	0.503	1.413	0.512	0.459	1.114	0.462	0.489	1.329	0.262
18581	0.224	-0.373	0.450	0.102	-1.184	0.508	0.329	0.301	0.481
18582	0.097	-1.181	0.512	0.307	0.135	0.341	0.413	0.841	0.358
18583	0.358	0.487	0.450	0.234	-0.336	0.512	0.050	-1.485	0.224
18584	0.361	0.504	0.450	0.193	-0.600	0.309	0.263	-0.120	0.492
18585	0.476	1.245	0.296	0.459	1.114	0.341	0.332	0.318	0.512
18586	0.426	0.925	0.513	0.515	1.472	0.514	0.476	1.245	0.508
18587	0.450	1.076	0.262	0.398	0.718	0.444	0.466	1.178	0.512
18588	0.463	1.161	0.492	0.442	1.001	0.422	0.500	1.397	0.328
18589	0.463	1.161	0.492	0.421	0.869	0.273	0.508	1.447	0.328
18590	0.463	1.161	0.508	0.421	0.869	0.309	0.508	1.447	0.328
18591	0.097	-1.181	0.508	0.149	-0.882	0.371	0.329	0.301	0.512
18592	0.097	-1.181	0.508	0.149	-0.882	0.478	0.295	0.082	0.512
18593	0.361	0.504	0.508	0.234	-0.336	0.512	0.097	-1.181	0.492
18594	0.453	1.093	0.501	0.465	1.152	0.501	0.445	1.043	0.501
18595	0.505	1.430	0.513	0.500	1.378	0.501	0.508	1.447	0.501
18596	0.500	1.397	0.512	0.500	1.378	0.512	0.508	1.447	0.501
18597	0.497	1.380	0.097	0.462	1.133	0.193	0.503	1.413	0.508
18598	0.226	-0.356	0.501	0.275	-0.073	0.478	0.226	-0.356	0.262
18599	0.187	-0.608	0.501	0.237	-0.317	0.149	0.184	-0.625	0.262
18600	0.297	0.099	0.262	0.272	-0.091	0.512	0.142	-0.895	0.492
18601	0.297	0.099	0.385	0.193	-0.600	0.398	0.050	-1.485	0.512

18602	0.387	0.672	0.508	0.398	0.718	0.398	0.508	1.447	0.513
18603	0.495	1.363	0.513	0.497	1.359	0.341	0.432	0.958	0.142
18604	0.508	1.447	0.224	0.497	1.359	0.512	0.513	1.481	0.501
18605	0.497	1.380	0.512	0.462	1.133	0.491	0.497	1.380	0.142
18606	0.489	1.329	0.185	0.509	1.434	0.193	0.500	1.397	0.467
18607	0.142	-0.895	0.501	0.342	0.361	0.398	0.184	-0.625	0.512
18608	0.482	1.279	0.492	0.515	1.472	0.235	0.461	1.144	0.481
18609	0.097	-1.181	0.492	0.310	0.153	0.508	0.184	-0.625	0.501
18610	0.184	-0.625	0.385	0.345	0.379	0.444	0.184	-0.625	0.450
18611	0.505	1.430	0.501	0.512	1.453	0.512	0.466	1.178	0.481
18612	0.511	1.464	0.501	0.439	0.982	0.462	0.508	1.447	0.224
18613	0.511	1.464	0.513	0.418	0.850	0.462	0.508	1.447	0.513
18614	0.000	?	0.512	0.275	-0.073	0.444	0.263	-0.120	0.050
18615	0.000	?	0.508	0.310	0.153	0.491	0.263	-0.120	0.492
18616	0.492	1.346	0.512	0.374	0.568	0.512	0.505	1.430	0.512
18617	0.463	1.161	0.513	0.480	1.246	0.508	0.487	1.312	0.467
18618	0.505	1.430	0.224	0.515	1.472	0.235	0.429	0.942	0.513
18619	0.508	1.447	0.508	0.500	1.378	0.512	0.505	1.430	0.385
18620	0.508	1.447	0.501	0.500	1.378	0.341	0.505	1.430	0.512
18621	0.484	1.295	0.501	0.512	1.453	0.501	0.466	1.178	0.508
18622	0.329	0.301	0.262	0.193	-0.600	0.508	0.189	-0.592	0.492
18623	0.497	1.380	0.224	0.477	1.227	0.462	0.466	1.178	0.508
18624	0.261	-0.137	0.492	0.149	-0.882	0.193	0.266	-0.103	0.328
18625	0.503	1.413	0.501	0.509	1.434	0.444	0.476	1.245	0.492
18626	0.184	-0.625	0.224	0.398	0.718	0.273	0.142	-0.895	0.492
18627	0.261	-0.137	0.224	0.398	0.718	0.512	0.097	-1.181	0.050
18628	0.261	-0.137	0.185	0.342	0.361	0.341	0.097	-1.181	0.185
18629	0.482	1.279	0.185	0.515	1.472	0.273	0.508	1.447	0.358
18630	0.511	1.464	0.481	0.491	1.321	0.512	0.471	1.211	0.358
18631	0.329	0.301	0.431	0.193	-0.600	0.149	0.097	-1.181	0.358
18632	0.505	1.430	0.512	0.395	0.700	0.514	0.453	1.093	0.492
18633	0.358	0.487	0.512	0.234	-0.336	0.501	0.097	-1.181	0.328
18634	0.500	1.397	0.501	0.500	1.378	0.501	0.432	0.958	0.501
18635	0.492	1.346	0.512	0.447	1.039	0.512	0.476	1.245	0.501
18636	0.505	1.430	0.508	0.515	1.472	0.422	0.492	1.346	0.501
18637	0.505	1.430	0.492	0.462	1.133	0.444	0.474	1.228	0.513
18638	0.476	1.245	0.296	0.459	1.114	0.501	0.516	1.498	0.512
18639	0.487	1.312	0.097	0.442	1.001	0.512	0.476	1.245	0.512
18640	0.505	1.430	0.501	0.491	1.321	0.501	0.497	1.380	0.450
18641	0.505	1.430	0.224	0.509	1.434	0.508	0.497	1.380	0.508
18642	0.505	1.430	0.492	0.506	1.415	0.273	0.508	1.447	0.501
18643	0.263	-0.120	0.501	0.237	-0.317	0.491	0.050	-1.485	0.508
18644	0.508	1.447	0.501	0.506	1.415	0.309	0.497	1.380	0.492
18645	0.184	-0.625	0.492	0.348	0.398	0.273	0.224	-0.373	0.224
18646	0.511	1.464	0.501	0.494	1.340	0.309	0.516	1.498	0.492
18647	0.497	1.380	0.481	0.506	1.415	0.398	0.516	1.498	0.501
18648	0.476	1.245	0.050	0.462	1.133	0.102	0.500	1.397	0.501
18649	0.097	-1.181	0.513	0.196	-0.581	0.309	0.263	-0.120	0.262
18650	0.492	1.346	0.508	0.442	1.001	0.235	0.492	1.346	0.142
18651	0.142	-0.895	0.508	0.275	-0.073	0.512	0.142	-0.895	0.492
18652	0.500	1.397	0.431	0.515	1.472	0.501	0.445	1.043	0.508
18653	0.097	-1.181	0.512	0.339	0.342	0.462	0.261	-0.137	0.224
18654	0.184	-0.625	0.409	0.275	-0.073	0.508	0.097	-1.181	0.224
18655	0.479	1.262	0.467	0.515	1.472	0.508	0.482	1.279	0.224
18656	0.184	-0.625	0.328	0.149	-0.882	0.508	0.224	-0.373	0.358
18657	0.497	1.380	0.185	0.515	1.472	0.501	0.511	1.464	0.512
18658	0.492	1.346	0.224	0.506	1.415	0.514	0.466	1.178	0.467
18659	0.492	1.346	0.508	0.506	1.415	0.053	0.466	1.178	0.513
18660	0.503	1.413	0.513	0.506	1.415	0.491	0.508	1.447	0.513
18661	0.508	1.447	0.050	0.418	0.850	0.508	0.511	1.464	0.513
18662	0.511	1.464	0.097	0.439	0.982	0.508	0.508	1.447	0.513

18663	0.516	1.498	0.097	0.497	1.359	0.514	0.518	1.514	0.431
18664	0.429	0.942	0.142	0.512	1.453	0.512	0.500	1.397	0.501
18665	0.511	1.464	0.512	0.500	1.378	0.512	0.482	1.279	0.385
18666	0.474	1.228	0.512	0.515	1.472	0.501	0.479	1.262	0.185
18667	0.187	-0.608	0.512	0.275	-0.073	0.501	0.097	-1.181	0.385
18668	0.405	0.790	0.508	0.494	1.340	0.508	0.511	1.464	0.097
18669	0.050	-1.485	0.097	0.313	0.172	0.341	0.261	-0.137	0.467
18670	0.142	-0.895	0.142	0.272	-0.091	0.512	0.384	0.655	0.467
18671	0.050	-1.485	0.296	0.307	0.135	0.478	0.261	-0.137	0.512
18672	0.295	0.082	0.142	0.395	0.700	0.491	0.261	-0.137	0.481
18673	0.142	-0.895	0.512	0.102	-1.184	0.273	0.358	0.487	0.467
18674	0.297	0.099	0.508	0.310	0.153	0.309	0.295	0.082	0.508
18675	0.050	-1.485	0.512	0.237	-0.317	0.491	0.329	0.301	0.328
18676	0.500	1.397	0.512	0.520	1.509	0.235	0.489	1.329	0.512
18677	0.511	1.464	0.513	0.497	1.359	0.501	0.516	1.498	0.508
18678	0.482	1.279	0.450	0.459	1.114	0.512	0.503	1.413	0.513
18679	0.503	1.413	0.501	0.509	1.434	0.514	0.492	1.346	0.450
18680	0.479	1.262	0.481	0.503	1.396	0.478	0.479	1.262	0.512
18681	0.516	1.498	0.492	0.503	1.396	0.508	0.447	1.060	0.431
18682	0.492	1.346	0.508	0.506	1.415	0.512	0.511	1.464	0.508
18683	0.487	1.312	0.142	0.518	1.491	0.514	0.432	0.958	0.097
18684	0.263	-0.120	0.142	0.053	-1.504	0.371	0.263	-0.120	0.142
18685	0.503	1.413	0.142	0.491	1.321	0.508	0.479	1.262	0.142
18686	0.497	1.380	0.185	0.506	1.415	0.501	0.476	1.245	0.450
18687	0.508	1.447	0.358	0.506	1.415	0.273	0.503	1.413	0.508
18688	0.503	1.413	0.328	0.512	1.453	0.512	0.429	0.942	0.224
18689	0.503	1.413	0.262	0.509	1.434	0.193	0.450	1.076	0.224
18690	0.503	1.413	0.185	0.509	1.434	0.501	0.429	0.942	0.358
18691	0.445	1.043	0.328	0.500	1.378	0.501	0.505	1.430	0.185
18692	0.497	1.380	0.492	0.500	1.378	0.512	0.479	1.262	0.385
18693	0.511	1.464	0.467	0.509	1.434	0.501	0.505	1.430	0.450
18694	0.226	-0.356	0.501	0.345	0.379	0.102	0.329	0.301	0.481
18695	0.503	1.413	0.512	0.515	1.472	0.508	0.468	1.194	0.501
18696	0.500	1.397	0.512	0.482	1.265	0.508	0.511	1.464	0.450
18697	0.500	1.397	0.142	0.497	1.359	0.501	0.505	1.430	0.296
18698	0.145	-0.878	0.142	0.275	-0.073	0.193	0.184	-0.625	0.431
18699	0.263	-0.120	0.262	0.313	0.172	0.462	0.329	0.301	0.328
18700	0.479	1.262	0.467	0.488	1.302	0.462	0.505	1.430	0.385
18701	0.224	-0.373	0.097	0.237	-0.317	0.462	0.266	-0.103	0.508
18702	0.508	1.447	0.481	0.500	1.378	0.512	0.508	1.447	0.512
18703	0.508	1.447	0.508	0.509	1.434	0.422	0.466	1.178	0.512
18704	0.476	1.245	0.492	0.512	1.453	0.512	0.482	1.279	0.512
18705	0.500	1.397	0.492	0.480	1.246	0.444	0.492	1.346	0.501
18706	0.513	1.481	0.467	0.503	1.396	0.512	0.476	1.245	0.512
18707	0.518	1.514	0.450	0.518	1.491	0.102	0.489	1.329	0.358
18708	0.474	1.228	0.501	0.512	1.453	0.491	0.482	1.279	0.492
18709	0.224	-0.373	0.262	0.377	0.587	0.462	0.329	0.301	0.512
18710	0.508	1.447	0.142	0.506	1.415	0.462	0.466	1.178	0.409
18711	0.503	1.413	0.097	0.497	1.359	0.512	0.479	1.262	0.409
18712	0.263	-0.120	0.142	0.272	-0.091	0.501	0.142	-0.895	0.409
18713	0.516	1.498	0.508	0.515	1.472	0.341	0.482	1.279	0.409
18714	0.297	0.099	0.512	0.193	-0.600	0.309	0.184	-0.625	0.185
18715	0.500	1.397	0.512	0.500	1.378	0.478	0.505	1.430	0.142
18716	0.484	1.295	0.262	0.500	1.378	0.508	0.492	1.346	0.262
18717	0.487	1.312	0.501	0.515	1.472	0.309	0.495	1.363	0.450
18718	0.476	1.245	0.508	0.506	1.415	0.273	0.495	1.363	0.185
18719	0.184	-0.625	0.481	0.102	-1.184	0.512	0.263	-0.120	0.409
18720	0.492	1.346	0.508	0.503	1.396	0.508	0.511	1.464	0.481
18721	0.492	1.346	0.512	0.503	1.396	0.491	0.511	1.464	0.450
18722	0.511	1.464	0.050	0.503	1.396	0.273	0.489	1.329	0.467
18723	0.184	-0.625	0.501	0.193	-0.600	0.444	0.224	-0.373	0.467

18724	0.497	1.380	0.492	0.462	1.133	0.508	0.511	1.464	0.481
18725	0.497	1.380	0.050	0.462	1.133	0.462	0.511	1.464	0.097
18726	0.497	1.380	0.224	0.462	1.133	0.491	0.511	1.464	0.142
18727	0.489	1.329	0.224	0.512	1.453	0.491	0.503	1.413	0.142
18728	0.503	1.413	0.508	0.427	0.907	0.341	0.513	1.481	0.508
18729	0.503	1.413	0.050	0.515	1.472	0.193	0.503	1.413	0.097
18730	0.511	1.464	0.050	0.442	1.001	0.371	0.492	1.346	0.508
18731	0.513	1.481	0.501	0.515	1.472	0.398	0.495	1.363	0.508
18732	0.142	-0.895	0.513	0.102	-1.184	0.309	0.300	0.116	0.508
18733	0.505	1.430	0.050	0.488	1.302	0.501	0.508	1.447	0.508
18734	0.450	1.076	0.508	0.468	1.170	0.491	0.513	1.481	0.492
18735	0.447	1.060	0.328	0.459	1.114	0.491	0.511	1.464	0.508
18736	0.505	1.430	0.508	0.509	1.434	0.491	0.500	1.397	0.492
18737	0.479	1.262	0.050	0.500	1.378	0.341	0.492	1.346	0.409
18738	0.224	-0.373	0.512	0.342	0.361	0.341	0.326	0.285	0.224
18739	0.184	-0.625	0.224	0.307	0.135	0.235	0.142	-0.895	0.142
18740	0.495	1.363	0.450	0.480	1.246	0.501	0.382	0.638	0.296
18741	0.500	1.397	0.450	0.506	1.415	0.491	0.408	0.807	0.513
18742	0.000	?	0.512	0.310	0.153	0.235	0.263	-0.120	0.358
18743	0.097	-1.181	0.467	0.275	-0.073	0.512	0.263	-0.120	0.358
18744	0.516	1.498	0.481	0.509	1.434	0.102	0.463	1.161	0.185
18745	0.497	1.380	0.492	0.515	1.472	0.341	0.513	1.481	0.512
18746	0.503	1.413	0.185	0.497	1.359	0.462	0.521	1.531	0.501
18747	0.097	-1.181	0.185	0.275	-0.073	0.478	0.263	-0.120	0.513
18748	0.503	1.413	0.142	0.444	1.020	0.512	0.484	1.295	0.512
18749	0.432	0.958	0.185	0.506	1.415	0.478	0.508	1.447	0.450
18750	0.513	1.481	0.296	0.462	1.133	0.512	0.484	1.295	0.142
18751	0.361	0.504	0.508	0.491	1.321	0.512	0.511	1.464	0.481
18752	0.474	1.228	0.097	0.491	1.321	0.512	0.468	1.194	0.481
18753	0.266	-0.103	0.492	0.342	0.361	0.512	0.097	-1.181	0.358
18754	0.411	0.824	0.481	0.193	-0.600	0.512	0.300	0.116	0.296
18755	0.411	0.824	0.296	0.371	0.549	0.478	0.516	1.498	0.358
18756	0.513	1.481	0.481	0.398	0.718	0.444	0.516	1.498	0.409
18757	0.142	-0.895	0.328	0.310	0.153	0.512	0.184	-0.625	0.296
18758	0.503	1.413	0.328	0.506	1.415	0.462	0.455	1.110	0.358
18759	0.511	1.464	0.512	0.488	1.302	0.508	0.505	1.430	0.296
18760	0.511	1.464	0.508	0.488	1.302	0.235	0.505	1.430	0.513
18761	0.511	1.464	0.481	0.488	1.302	0.235	0.505	1.430	0.431
18762	0.050	-1.485	0.097	0.339	0.342	0.491	0.355	0.470	0.328
18763	0.097	-1.181	0.097	0.348	0.398	0.501	0.295	0.082	0.481
18764	0.263	-0.120	0.508	0.234	-0.336	0.444	0.297	0.099	0.050
18765	0.497	1.380	0.508	0.500	1.378	0.341	0.479	1.262	0.512
18766	0.513	1.481	0.513	0.497	1.359	0.478	0.411	0.824	0.296
18767	0.184	-0.625	0.513	0.234	-0.336	0.341	0.261	-0.137	0.508
18768	0.487	1.312	0.142	0.518	1.491	0.514	0.487	1.312	0.185
18769	0.097	-1.181	0.512	0.102	-1.184	0.491	0.358	0.487	0.501
18770	0.142	-0.895	0.296	0.345	0.379	0.514	0.226	-0.356	0.501
18771	0.468	1.194	0.262	0.459	1.114	0.398	0.495	1.363	0.467
18772	0.508	1.447	0.358	0.474	1.208	0.512	0.511	1.464	0.450
18773	0.479	1.262	0.513	0.509	1.434	0.512	0.508	1.447	0.513
18774	0.511	1.464	0.385	0.474	1.208	0.491	0.511	1.464	0.492
18775	0.500	1.397	0.142	0.500	1.378	0.309	0.326	0.285	0.262
18776	0.500	1.397	0.142	0.500	1.378	0.478	0.326	0.285	0.185
18777	0.500	1.397	0.513	0.500	1.378	0.462	0.326	0.285	0.296
18778	0.500	1.397	0.508	0.512	1.453	0.512	0.511	1.464	0.262
18779	0.500	1.397	0.050	0.512	1.453	0.501	0.511	1.464	0.185
18780	0.497	1.380	0.050	0.482	1.265	0.512	0.497	1.380	0.481
18781	0.461	1.144	0.508	0.444	1.020	0.501	0.511	1.464	0.296
18782	0.476	1.245	0.501	0.512	1.453	0.514	0.503	1.413	0.385
18783	0.503	1.413	0.501	0.462	1.133	0.512	0.503	1.413	0.481
18784	0.508	1.447	0.097	0.506	1.415	0.501	0.511	1.464	0.385

18785	0.142	-0.895	0.508	0.237	-0.317	0.102	0.261	-0.137	0.512
18786	0.142	-0.895	0.050	0.237	-0.317	0.491	0.261	-0.137	0.142
18787	0.492	1.346	0.512	0.494	1.340	0.491	0.492	1.346	0.142
18788	0.505	1.430	0.328	0.503	1.396	0.309	0.513	1.481	0.492
18789	0.489	1.329	0.328	0.442	1.001	0.398	0.511	1.464	0.512
18790	0.263	-0.120	0.358	0.342	0.361	0.512	0.142	-0.895	0.508
18791	0.497	1.380	0.224	0.482	1.265	0.512	0.500	1.397	0.296
18792	0.263	-0.120	0.501	0.342	0.361	0.273	0.142	-0.895	0.262
18793	0.445	1.043	0.501	0.518	1.491	0.501	0.463	1.161	0.481
18794	0.503	1.413	0.481	0.494	1.340	0.273	0.508	1.447	0.481
18795	0.445	1.043	0.050	0.515	1.472	0.514	0.482	1.279	0.262
18796	0.508	1.447	0.512	0.401	0.737	0.478	0.503	1.413	0.431
18797	0.445	1.043	0.512	0.512	1.453	0.462	0.447	1.060	0.431
18798	0.445	1.043	0.508	0.512	1.453	0.512	0.482	1.279	0.185
18799	0.300	0.116	0.508	0.000	?	0.508	0.224	-0.373	0.508
18800	0.508	1.447	0.492	0.488	1.302	0.508	0.511	1.464	0.296
18801	0.261	-0.137	0.501	0.307	0.135	0.491	0.050	-1.485	0.262
18802	0.492	1.346	0.512	0.480	1.246	0.501	0.489	1.329	0.224
18803	0.495	1.363	0.262	0.462	1.133	0.501	0.508	1.447	0.142
18804	0.500	1.397	0.481	0.515	1.472	0.478	0.463	1.161	0.481
18805	0.497	1.380	0.512	0.509	1.434	0.422	0.516	1.498	0.050
18806	0.500	1.397	0.262	0.520	1.509	0.102	0.387	0.672	0.296
18807	0.497	1.380	0.513	0.509	1.434	0.398	0.513	1.481	0.224
18808	0.492	1.346	0.508	0.515	1.472	0.398	0.508	1.447	0.450
18809	0.508	1.447	0.492	0.509	1.434	0.398	0.497	1.380	0.481
18810	0.516	1.498	0.501	0.500	1.378	0.149	0.513	1.481	0.328
18811	0.097	-1.181	0.467	0.105	-1.165	0.273	0.329	0.301	0.262
18812	0.503	1.413	0.512	0.494	1.340	0.512	0.492	1.346	0.467
18813	0.495	1.363	0.508	0.494	1.340	0.508	0.495	1.363	0.467
18814	0.261	-0.137	0.142	0.310	0.153	0.273	0.050	-1.485	0.481
18815	0.387	0.672	0.481	0.401	0.737	0.273	0.184	-0.625	0.450
18816	0.513	1.481	0.512	0.515	1.472	0.235	0.358	0.487	0.513
18817	0.516	1.498	0.508	0.515	1.472	0.273	0.358	0.487	0.296
18818	0.226	-0.356	0.501	0.275	-0.073	0.508	0.358	0.487	0.492
18819	0.508	1.447	0.508	0.494	1.340	0.478	0.492	1.346	0.142
18820	0.184	-0.625	0.513	0.275	-0.073	0.478	0.329	0.301	0.142
18821	0.492	1.346	0.492	0.515	1.472	0.478	0.503	1.413	0.296
18822	0.489	1.329	0.512	0.477	1.227	0.478	0.500	1.397	0.385
18823	0.489	1.329	0.512	0.462	1.133	0.478	0.500	1.397	0.481
18824	0.384	0.655	0.512	0.512	1.453	0.512	0.516	1.498	0.512
18825	0.503	1.413	0.467	0.506	1.415	0.398	0.516	1.498	0.512
18826	0.503	1.413	0.224	0.506	1.415	0.371	0.516	1.498	0.262
18827	0.511	1.464	0.508	0.491	1.321	0.273	0.458	1.127	0.385
18828	0.505	1.430	0.262	0.497	1.359	0.478	0.511	1.464	0.385
18829	0.508	1.447	0.296	0.509	1.434	0.235	0.497	1.380	0.385
18830	0.503	1.413	0.185	0.477	1.227	0.512	0.511	1.464	0.385
18831	0.500	1.397	0.097	0.427	0.907	0.512	0.492	1.346	0.513
18832	0.224	-0.373	0.513	0.102	-1.184	0.462	0.226	-0.356	0.296
18833	0.505	1.430	0.467	0.398	0.718	0.512	0.489	1.329	0.328
18834	0.497	1.380	0.467	0.398	0.718	0.508	0.500	1.397	0.513
18835	0.497	1.380	0.467	0.398	0.718	0.512	0.500	1.397	0.508
18836	0.263	-0.120	0.467	0.149	-0.882	0.193	0.261	-0.137	0.224
18837	0.224	-0.373	0.512	0.275	-0.073	0.478	0.142	-0.895	0.467
18838	0.489	1.329	0.512	0.515	1.472	0.512	0.489	1.329	0.328
18839	0.500	1.397	0.512	0.500	1.378	0.462	0.508	1.447	0.328
18840	0.224	-0.373	0.142	0.275	-0.073	0.512	0.224	-0.373	0.508
18841	0.224	-0.373	0.467	0.275	-0.073	0.491	0.224	-0.373	0.501
18842	0.184	-0.625	0.512	0.237	-0.317	0.514	0.224	-0.373	0.492
18843	0.184	-0.625	0.512	0.272	-0.091	0.478	0.355	0.470	0.467
18844	0.476	1.245	0.512	0.509	1.434	0.149	0.513	1.481	0.431
18845	0.426	0.925	0.501	0.477	1.227	0.235	0.466	1.178	0.508

18846	0.511	1.464	0.512	0.474	1.208	0.193	0.508	1.447	0.508
18847	0.511	1.464	0.512	0.474	1.208	0.508	0.508	1.447	0.512
18848	0.500	1.397	0.501	0.474	1.208	0.501	0.508	1.447	0.142
18849	0.511	1.464	0.508	0.474	1.208	0.309	0.508	1.447	0.508
18850	0.508	1.447	0.492	0.512	1.453	0.444	0.426	0.925	0.512
18851	0.495	1.363	0.492	0.398	0.718	0.341	0.497	1.380	0.481
18852	0.297	0.099	0.467	0.371	0.549	0.341	0.387	0.672	0.409
18853	0.097	-1.181	0.142	0.275	-0.073	0.273	0.184	-0.625	0.513
18854	0.505	1.430	0.185	0.480	1.246	0.512	0.387	0.672	0.481
18855	0.226	-0.356	0.512	0.234	-0.336	0.512	0.097	-1.181	0.142
18856	0.492	1.346	0.512	0.512	1.453	0.508	0.466	1.178	0.481
18857	0.503	1.413	0.501	0.512	1.453	0.508	0.466	1.178	0.492
18858	0.495	1.363	0.508	0.462	1.133	0.149	0.516	1.498	0.513
18859	0.484	1.295	0.224	0.512	1.453	0.193	0.482	1.279	0.467
18860	0.492	1.346	0.409	0.509	1.434	0.309	0.468	1.194	0.185
18861	0.476	1.245	0.142	0.512	1.453	0.053	0.505	1.430	0.224
18862	0.050	-1.485	0.501	0.196	-0.581	0.491	0.329	0.301	0.450
18863	0.505	1.430	0.185	0.482	1.265	0.508	0.513	1.481	0.224
18864	0.503	1.413	0.481	0.518	1.491	0.508	0.508	1.447	0.224
18865	0.497	1.380	0.481	0.462	1.133	0.508	0.516	1.498	0.328
18866	0.432	0.958	0.481	0.512	1.453	0.462	0.453	1.093	0.224
18867	0.513	1.481	0.508	0.488	1.302	0.422	0.518	1.514	0.501
18868	0.411	0.824	0.501	0.515	1.472	0.491	0.432	0.958	0.512
18869	0.463	1.161	0.481	0.477	1.227	0.462	0.511	1.464	0.512
18870	0.329	0.301	0.512	0.149	-0.882	0.491	0.100	-1.164	0.512
18871	0.184	-0.625	0.142	0.234	-0.336	0.508	0.142	-0.895	0.512
18872	0.226	-0.356	0.296	0.196	-0.581	0.149	0.142	-0.895	0.467
18873	0.503	1.413	0.508	0.509	1.434	0.149	0.450	1.076	0.467
18874	0.511	1.464	0.224	0.497	1.359	0.149	0.508	1.447	0.467
18875	0.050	-1.485	0.467	0.313	0.172	0.193	0.224	-0.373	0.358
18876	0.097	-1.181	0.512	0.444	1.020	0.309	0.224	-0.373	0.512
18877	0.000	?	0.467	0.345	0.379	0.444	0.358	0.487	0.512
18878	0.097	-1.181	0.467	0.342	0.361	0.149	0.184	-0.625	0.501
18879	0.145	-0.878	0.224	0.275	-0.073	0.273	0.384	0.655	0.501
18880	0.500	1.397	0.358	0.512	1.453	0.341	0.455	1.110	0.467
18881	0.500	1.397	0.492	0.515	1.472	0.508	0.482	1.279	0.097
18882	0.500	1.397	0.262	0.509	1.434	0.462	0.503	1.413	0.050
18883	0.503	1.413	0.508	0.509	1.434	0.444	0.453	1.093	0.501
18884	0.097	-1.181	0.224	0.149	-0.882	0.462	0.297	0.099	0.501
18885	0.142	-0.895	0.385	0.193	-0.600	0.501	0.434	0.975	0.513
18886	0.297	0.099	0.481	0.307	0.135	0.371	0.326	0.285	0.431
18887	0.142	-0.895	0.224	0.053	-1.504	0.273	0.382	0.638	0.492
18888	0.500	1.397	0.512	0.500	1.378	0.193	0.511	1.464	0.508
18889	0.508	1.447	0.508	0.509	1.434	0.478	0.505	1.430	0.512
18890	0.511	1.464	0.508	0.509	1.434	0.341	0.505	1.430	0.185
18891	0.511	1.464	0.328	0.509	1.434	0.491	0.505	1.430	0.224
18892	0.511	1.464	0.296	0.465	1.152	0.512	0.505	1.430	0.508
18893	0.447	1.060	0.467	0.424	0.888	0.512	0.508	1.447	0.481
18894	0.505	1.430	0.224	0.491	1.321	0.512	0.361	0.504	0.501
18895	0.479	1.262	0.450	0.462	1.133	0.501	0.489	1.329	0.513
18896	0.489	1.329	0.262	0.497	1.359	0.398	0.513	1.481	0.224
18897	0.497	1.380	0.512	0.506	1.415	0.444	0.411	0.824	0.501
18898	0.142	-0.895	0.512	0.152	-0.863	0.371	0.411	0.824	0.224
18899	0.142	-0.895	0.328	0.152	-0.863	0.341	0.411	0.824	0.501
18900	0.142	-0.895	0.481	0.152	-0.863	0.309	0.411	0.824	0.050
18901	0.187	-0.608	0.467	0.196	-0.581	0.309	0.184	-0.625	0.508
18902	0.361	0.504	0.492	0.310	0.153	0.512	0.142	-0.895	0.513
18903	0.329	0.301	0.492	0.447	1.039	0.508	0.261	-0.137	0.508
18904	0.261	-0.137	0.501	0.149	-0.882	0.512	0.455	1.110	0.492
18905	0.184	-0.625	0.508	0.275	-0.073	0.273	0.184	-0.625	0.501
18906	0.329	0.301	0.512	0.345	0.379	0.491	0.411	0.824	0.431

18907	0.492	1.346	0.296	0.506	1.415	0.508	0.474	1.228	0.508
18908	0.468	1.194	0.492	0.459	1.114	0.462	0.445	1.043	0.142
18909	0.500	1.397	0.513	0.442	1.001	0.501	0.463	1.161	0.296
18910	0.500	1.397	0.097	0.465	1.152	0.501	0.466	1.178	0.467
18911	0.511	1.464	0.296	0.500	1.378	0.341	0.482	1.279	0.097
18912	0.142	-0.895	0.262	0.371	0.549	0.514	0.097	-1.181	0.512
18913	0.142	-0.895	0.431	0.275	-0.073	0.462	0.145	-0.878	0.467
18914	0.261	-0.137	0.492	0.196	-0.581	0.193	0.142	-0.895	0.512
18915	0.466	1.178	0.492	0.480	1.246	0.149	0.513	1.481	0.512
18916	0.097	-1.181	0.481	0.342	0.361	0.235	0.097	-1.181	0.185
18917	0.476	1.245	0.481	0.488	1.302	0.501	0.505	1.430	0.185
18918	0.505	1.430	0.296	0.512	1.453	0.193	0.508	1.447	0.501
18919	0.489	1.329	0.296	0.512	1.453	0.341	0.508	1.447	0.142
18920	0.489	1.329	0.481	0.512	1.453	0.422	0.508	1.447	0.481
18921	0.466	1.178	0.296	0.506	1.415	0.444	0.492	1.346	0.224
18922	0.450	1.076	0.492	0.401	0.737	0.501	0.508	1.447	0.224
18923	0.503	1.413	0.431	0.447	1.039	0.309	0.492	1.346	0.481
18924	0.261	-0.137	0.508	0.368	0.530	0.491	0.411	0.824	0.358
18925	0.142	-0.895	0.142	0.342	0.361	0.149	0.224	-0.373	0.409
18926	0.097	-1.181	0.296	0.313	0.172	0.508	0.142	-0.895	0.431
18927	0.145	-0.878	0.296	0.313	0.172	0.235	0.295	0.082	0.492
18928	0.508	1.447	0.142	0.509	1.434	0.462	0.513	1.481	0.358
18929	0.503	1.413	0.431	0.509	1.434	0.371	0.358	0.487	0.224
18930	0.503	1.413	0.450	0.512	1.453	0.514	0.358	0.487	0.513
18931	0.261	-0.137	0.450	0.272	-0.091	0.514	0.187	-0.608	0.142
18932	0.500	1.397	0.328	0.491	1.321	0.512	0.505	1.430	0.492
18933	0.513	1.481	0.328	0.509	1.434	0.512	0.495	1.363	0.328
18934	0.482	1.279	0.492	0.462	1.133	0.491	0.508	1.447	0.467
18935	0.500	1.397	0.481	0.500	1.378	0.508	0.511	1.464	0.467
18936	0.505	1.430	0.224	0.497	1.359	0.102	0.453	1.093	0.185
18937	0.050	-1.485	0.224	0.342	0.361	0.193	0.145	-0.878	0.450
18938	0.503	1.413	0.508	0.515	1.472	0.273	0.484	1.295	0.481
18939	0.484	1.295	0.492	0.468	1.170	0.273	0.479	1.262	0.508
18940	0.050	-1.485	0.512	0.193	-0.600	0.149	0.358	0.487	0.508
18941	0.224	-0.373	0.512	0.149	-0.882	0.478	0.295	0.082	0.512
18942	0.224	-0.373	0.508	0.237	-0.317	0.371	0.358	0.487	0.508
18943	0.500	1.397	0.512	0.506	1.415	0.508	0.405	0.790	0.492
18944	0.050	-1.485	0.501	0.196	-0.581	0.491	0.295	0.082	0.224
18945	0.050	-1.485	0.224	0.345	0.379	0.193	0.358	0.487	0.513
18946	0.000	?	0.513	0.427	0.907	0.508	0.295	0.082	0.512
18947	0.497	1.380	0.501	0.442	1.001	0.422	0.511	1.464	0.142
18948	0.500	1.397	0.501	0.503	1.396	0.193	0.432	0.958	0.185
18949	0.050	-1.485	0.224	0.313	0.172	0.512	0.326	0.285	0.142
18950	0.505	1.430	0.385	0.491	1.321	0.508	0.479	1.262	0.467
18951	0.326	0.285	0.512	0.149	-0.882	0.462	0.050	-1.485	0.492
18952	0.497	1.380	0.492	0.515	1.472	0.309	0.516	1.498	0.512
18953	0.050	-1.485	0.508	0.234	-0.336	0.273	0.300	0.116	0.513
18954	0.505	1.430	0.512	0.468	1.170	0.512	0.503	1.413	0.501
18955	0.226	-0.356	0.508	0.371	0.549	0.512	0.187	-0.608	0.358
18956	0.450	1.076	0.508	0.512	1.453	0.273	0.503	1.413	0.358
18957	0.450	1.076	0.508	0.512	1.453	0.462	0.503	1.413	0.450
18958	0.511	1.464	0.508	0.512	1.453	0.462	0.461	1.144	0.142
18959	0.466	1.178	0.296	0.509	1.434	0.235	0.453	1.093	0.512
18960	0.479	1.262	0.409	0.497	1.359	0.491	0.511	1.464	0.508
18961	0.487	1.312	0.409	0.515	1.472	0.235	0.489	1.329	0.501
18962	0.184	-0.625	0.508	0.102	-1.184	0.235	0.261	-0.137	0.328
18963	0.184	-0.625	0.224	0.196	-0.581	0.193	0.187	-0.608	0.385
18964	0.142	-0.895	0.385	0.275	-0.073	0.053	0.295	0.082	0.385
18965	0.184	-0.625	0.492	0.272	-0.091	0.491	0.263	-0.120	0.328
18966	0.295	0.082	0.450	0.149	-0.882	0.235	0.184	-0.625	0.512
18967	0.503	1.413	0.467	0.477	1.227	0.193	0.479	1.262	0.513

18968	0.097	-1.181	0.513	0.368	0.530	0.193	0.297	0.099	0.513
18969	0.484	1.295	0.467	0.512	1.453	0.512	0.382	0.638	0.185
18970	0.474	1.228	0.450	0.497	1.359	0.512	0.479	1.262	0.185
18971	0.295	0.082	0.508	0.193	-0.600	0.462	0.387	0.672	0.508
18972	0.482	1.279	0.513	0.506	1.415	0.309	0.511	1.464	0.501
18973	0.326	0.285	0.262	0.418	0.850	0.491	0.142	-0.895	0.185
18974	0.326	0.285	0.328	0.193	-0.600	0.501	0.142	-0.895	0.142
18975	0.500	1.397	0.431	0.515	1.472	0.501	0.495	1.363	0.508
18976	0.500	1.397	0.492	0.512	1.453	0.102	0.513	1.481	0.431
18977	0.487	1.312	0.142	0.462	1.133	0.444	0.505	1.430	0.481
18978	0.100	-1.164	0.492	0.307	0.135	0.193	0.297	0.099	0.513
18979	0.100	-1.164	0.050	0.275	-0.073	0.478	0.261	-0.137	0.512
18980	0.500	1.397	0.492	0.515	1.472	0.053	0.476	1.245	0.513
18981	0.500	1.397	0.492	0.515	1.472	0.053	0.476	1.245	0.492
18982	0.000	?	0.508	0.275	-0.073	0.102	0.261	-0.137	0.185
18983	0.516	1.498	0.492	0.459	1.114	0.149	0.429	0.942	0.508
18984	0.516	1.498	0.409	0.459	1.114	0.512	0.429	0.942	0.513
18985	0.142	-0.895	0.185	0.237	-0.317	0.462	0.184	-0.625	0.508
18986	0.513	1.481	0.512	0.494	1.340	0.462	0.508	1.447	0.262
18987	0.000	?	0.512	0.234	-0.336	0.341	0.295	0.082	0.513
18988	0.300	0.116	0.513	0.234	-0.336	0.508	0.261	-0.137	0.512
18989	0.263	-0.120	0.467	0.193	-0.600	0.508	0.224	-0.373	0.512
18990	0.358	0.487	0.501	0.053	-1.504	0.508	0.142	-0.895	0.492
18991	0.513	1.481	0.512	0.488	1.302	0.508	0.476	1.245	0.508
18992	0.387	0.672	0.501	0.237	-0.317	0.514	0.050	-1.485	0.501
18993	0.142	-0.895	0.508	0.193	-0.600	0.273	0.297	0.099	0.501
18994	0.142	-0.895	0.492	0.193	-0.600	0.371	0.226	-0.356	0.501
18995	0.503	1.413	0.467	0.512	1.453	0.508	0.455	1.110	0.501
18996	0.497	1.380	0.508	0.512	1.453	0.501	0.487	1.312	0.185
18997	0.050	-1.485	0.508	0.462	1.133	0.341	0.332	0.318	0.513
18998	0.050	-1.485	0.501	0.310	0.153	0.462	0.263	-0.120	0.513
18999	0.497	1.380	0.296	0.491	1.321	0.273	0.468	1.194	0.328
19000	0.492	1.346	0.492	0.503	1.396	0.273	0.468	1.194	0.097
19001	0.503	1.413	0.512	0.503	1.396	0.501	0.476	1.245	0.512
19002	0.097	-1.181	0.508	0.105	-1.165	0.273	0.450	1.076	0.467
19003	0.497	1.380	0.224	0.444	1.020	0.491	0.513	1.481	0.431
19004	0.050	-1.485	0.224	0.196	-0.581	0.478	0.297	0.099	0.385
19005	0.511	1.464	0.513	0.477	1.227	0.462	0.495	1.363	0.385
19006	0.329	0.301	0.513	0.053	-1.504	0.512	0.145	-0.878	0.450
19007	0.329	0.301	0.262	0.053	-1.504	0.478	0.145	-0.878	0.492
19008	0.355	0.470	0.328	0.105	-1.165	0.501	0.297	0.099	0.508
19009	0.224	-0.373	0.512	0.149	-0.882	0.508	0.387	0.672	0.481
19010	0.495	1.363	0.185	0.509	1.434	0.309	0.482	1.279	0.050
19011	0.492	1.346	0.481	0.465	1.152	0.514	0.513	1.481	0.512
19012	0.474	1.228	0.358	0.462	1.133	0.478	0.511	1.464	0.501
19013	0.050	-1.485	0.492	0.345	0.379	0.478	0.261	-0.137	0.262
19014	0.511	1.464	0.512	0.503	1.396	0.491	0.384	0.655	0.481
19015	0.511	1.464	0.431	0.503	1.396	0.491	0.384	0.655	0.185
19016	0.508	1.447	0.513	0.503	1.396	0.501	0.384	0.655	0.501
19017	0.508	1.447	0.492	0.503	1.396	0.444	0.384	0.655	0.508
19018	0.489	1.329	0.512	0.512	1.453	0.444	0.513	1.481	0.450
19019	0.000	?	0.508	0.275	-0.073	0.444	0.297	0.099	0.513
19020	0.000	?	0.512	0.368	0.530	0.478	0.329	0.301	0.224
19021	0.503	1.413	0.512	0.506	1.415	0.478	0.505	1.430	0.050
19022	0.513	1.481	0.224	0.503	1.396	0.398	0.500	1.397	0.512
19023	0.261	-0.137	0.481	0.342	0.361	0.193	0.224	-0.373	0.512
19024	0.484	1.295	0.512	0.462	1.133	0.398	0.463	1.161	0.512
19025	0.000	?	0.508	0.275	-0.073	0.102	0.329	0.301	0.431
19026	0.000	?	0.185	0.275	-0.073	0.102	0.329	0.301	0.450
19027	0.500	1.397	0.450	0.506	1.415	0.422	0.511	1.464	0.512
19028	0.263	-0.120	0.492	0.272	-0.091	0.371	0.000	?	0.385

19029	0.513	1.481	0.431	0.485	1.283	0.398	0.500	1.397	0.481
19030	0.508	1.447	0.431	0.482	1.265	0.491	0.492	1.346	0.328
19031	0.495	1.363	0.224	0.462	1.133	0.491	0.463	1.161	0.501
19032	0.503	1.413	0.224	0.512	1.453	0.491	0.426	0.925	0.508
19033	0.468	1.194	0.431	0.477	1.227	0.491	0.505	1.430	0.508
19034	0.508	1.447	0.481	0.494	1.340	0.512	0.508	1.447	0.512
19035	0.503	1.413	0.185	0.512	1.453	0.512	0.511	1.464	0.262
19036	0.142	-0.895	0.142	0.313	0.172	0.512	0.142	-0.895	0.508
19037	0.482	1.279	0.508	0.512	1.453	0.053	0.500	1.397	0.431
19038	0.511	1.464	0.185	0.477	1.227	0.501	0.513	1.481	0.467
19039	0.513	1.481	0.224	0.474	1.208	0.501	0.484	1.295	0.328
19040	0.500	1.397	0.296	0.497	1.359	0.512	0.405	0.790	0.328
19041	0.500	1.397	0.328	0.494	1.340	0.512	0.508	1.447	0.512
19042	0.516	1.498	0.142	0.500	1.378	0.491	0.482	1.279	0.512
19043	0.495	1.363	0.142	0.447	1.039	0.398	0.142	-0.895	0.358
19044	0.500	1.397	0.224	0.450	1.057	0.462	0.484	1.295	0.385
19045	0.518	1.514	0.185	0.439	0.982	0.462	0.492	1.346	0.481
19046	0.505	1.430	0.142	0.480	1.246	0.462	0.513	1.481	0.185
19047	0.463	1.161	0.501	0.485	1.283	0.491	0.468	1.194	0.513
19048	0.000	?	0.492	0.401	0.737	0.501	0.184	-0.625	0.296
19049	0.224	-0.373	0.492	0.193	-0.600	0.508	0.224	-0.373	0.512
19050	0.513	1.481	0.467	0.398	0.718	0.508	0.450	1.076	0.467
19051	0.266	-0.103	0.501	0.102	-1.184	0.462	0.224	-0.373	0.431
19052	0.300	0.116	0.508	0.102	-1.184	0.341	0.224	-0.373	0.512
19053	0.187	-0.608	0.492	0.427	0.907	0.273	0.326	0.285	0.508
19054	0.097	-1.181	0.097	0.374	0.568	0.478	0.224	-0.373	0.508
19055	0.508	1.447	0.467	0.401	0.737	0.501	0.505	1.430	0.296
19056	0.468	1.194	0.492	0.485	1.283	0.444	0.508	1.447	0.409
19057	0.468	1.194	0.262	0.485	1.283	0.444	0.508	1.447	0.481
19058	0.468	1.194	0.385	0.485	1.283	0.193	0.508	1.447	0.481
19059	0.468	1.194	0.185	0.485	1.283	0.491	0.505	1.430	0.142
19060	0.505	1.430	0.481	0.512	1.453	0.273	0.468	1.194	0.512
19061	0.505	1.430	0.492	0.512	1.453	0.508	0.468	1.194	0.328
19062	0.505	1.430	0.431	0.512	1.453	0.309	0.468	1.194	0.385
19063	0.142	-0.895	0.512	0.053	-1.504	0.514	0.361	0.504	0.142
19064	0.471	1.211	0.097	0.503	1.396	0.478	0.513	1.481	0.512
19065	0.511	1.464	0.512	0.494	1.340	0.444	0.511	1.464	0.492
19066	0.511	1.464	0.481	0.506	1.415	0.462	0.500	1.397	0.492
19067	0.511	1.464	0.185	0.506	1.415	0.491	0.500	1.397	0.492
19068	0.492	1.346	0.501	0.494	1.340	0.398	0.463	1.161	0.185
19069	0.000	?	0.512	0.398	0.718	0.478	0.097	-1.181	0.185
19070	0.000	?	0.492	0.459	1.114	0.273	0.050	-1.485	0.467
19071	0.503	1.413	0.512	0.462	1.133	0.053	0.505	1.430	0.431
19072	0.503	1.413	0.467	0.462	1.133	0.514	0.505	1.430	0.185
19073	0.495	1.363	0.467	0.488	1.302	0.371	0.513	1.481	0.142
19074	0.500	1.397	0.450	0.500	1.378	0.462	0.429	0.942	0.409
19075	0.487	1.312	0.450	0.506	1.415	0.501	0.489	1.329	0.296
19076	0.489	1.329	0.512	0.506	1.415	0.462	0.503	1.413	0.262
19077	0.463	1.161	0.501	0.462	1.133	0.462	0.513	1.481	0.262
19078	0.142	-0.895	0.508	0.342	0.361	0.193	0.184	-0.625	0.296
19079	0.187	-0.608	0.262	0.272	-0.091	0.341	0.224	-0.373	0.262
19080	0.511	1.464	0.296	0.477	1.227	0.501	0.508	1.447	0.262
19081	0.516	1.498	0.450	0.500	1.378	0.273	0.476	1.245	0.328
19082	0.492	1.346	0.450	0.444	1.020	0.508	0.503	1.413	0.185
19083	0.508	1.447	0.501	0.442	1.001	0.235	0.508	1.447	0.185
19084	0.224	-0.373	0.385	0.196	-0.581	0.235	0.224	-0.373	0.481
19085	0.413	0.841	0.508	0.494	1.340	0.501	0.500	1.397	0.512
19086	0.142	-0.895	0.142	0.272	-0.091	0.235	0.224	-0.373	0.512
19087	0.497	1.380	0.508	0.509	1.434	0.508	0.495	1.363	0.501
19088	0.184	-0.625	0.508	0.313	0.172	0.491	0.050	-1.485	0.512
19089	0.484	1.295	0.512	0.515	1.472	0.501	0.505	1.430	0.512

19090	0.482	1.279	0.513	0.477	1.227	0.193	0.511	1.464	0.501
19091	0.482	1.279	0.450	0.444	1.020	0.102	0.505	1.430	0.224
19092	0.508	1.447	0.358	0.462	1.133	0.478	0.489	1.329	0.224
19093	0.497	1.380	0.409	0.485	1.283	0.193	0.503	1.413	0.467
19094	0.482	1.279	0.492	0.401	0.737	0.462	0.429	0.942	0.185
19095	0.513	1.481	0.262	0.474	1.208	0.398	0.500	1.397	0.450
19096	0.142	-0.895	0.481	0.272	-0.091	0.508	0.142	-0.895	0.185
19097	0.295	0.082	0.358	0.053	-1.504	0.501	0.297	0.099	0.512
19098	0.508	1.447	0.142	0.509	1.434	0.235	0.466	1.178	0.409
19099	0.224	-0.373	0.050	0.374	0.568	0.508	0.097	-1.181	0.512
19100	0.466	1.178	0.501	0.459	1.114	0.508	0.508	1.447	0.508
19101	0.511	1.464	0.185	0.500	1.378	0.491	0.463	1.161	0.185
19102	0.468	1.194	0.224	0.468	1.170	0.491	0.505	1.430	0.513
19103	0.468	1.194	0.185	0.468	1.170	0.422	0.505	1.430	0.467
19104	0.224	-0.373	0.512	0.193	-0.600	0.462	0.184	-0.625	0.296
19105	0.355	0.470	0.512	0.339	0.342	0.444	0.187	-0.608	0.409
19106	0.492	1.346	0.512	0.497	1.359	0.273	0.500	1.397	0.512
19107	0.263	-0.120	0.492	0.272	-0.091	0.422	0.142	-0.895	0.481
19108	0.505	1.430	0.142	0.506	1.415	0.512	0.482	1.279	0.492
19109	0.226	-0.356	0.185	0.234	-0.336	0.309	0.224	-0.373	0.508
19110	0.384	0.655	0.262	0.234	-0.336	0.102	0.224	-0.373	0.501
19111	0.482	1.279	0.097	0.497	1.359	0.193	0.482	1.279	0.492
19112	0.224	-0.373	0.358	0.234	-0.336	0.512	0.358	0.487	0.508
19113	0.505	1.430	0.508	0.506	1.415	0.501	0.411	0.824	0.501
19114	0.500	1.397	0.097	0.491	1.321	0.462	0.434	0.975	0.492
19115	0.505	1.430	0.385	0.503	1.396	0.491	0.492	1.346	0.492
19116	0.329	0.301	0.512	0.193	-0.600	0.371	0.355	0.470	0.262
19117	0.297	0.099	0.142	0.102	-1.184	0.371	0.224	-0.373	0.142
19118	0.466	1.178	0.513	0.477	1.227	0.371	0.513	1.481	0.508
19119	0.224	-0.373	0.481	0.193	-0.600	0.501	0.142	-0.895	0.508
19120	0.447	1.060	0.262	0.465	1.152	0.102	0.492	1.346	0.450
19121	0.261	-0.137	0.492	0.398	0.718	0.501	0.332	0.318	0.262
19122	0.500	1.397	0.185	0.512	1.453	0.501	0.466	1.178	0.512
19123	0.500	1.397	0.492	0.506	1.415	0.478	0.468	1.194	0.262
19124	0.329	0.301	0.508	0.237	-0.317	0.235	0.184	-0.625	0.492
19125	0.476	1.245	0.512	0.503	1.396	0.273	0.450	1.076	0.492
19126	0.463	1.161	0.512	0.503	1.396	0.273	0.479	1.262	0.508
19127	0.492	1.346	0.513	0.494	1.340	0.273	0.508	1.447	0.508
19128	0.492	1.346	0.513	0.488	1.302	0.491	0.500	1.397	0.224
19129	0.492	1.346	0.467	0.421	0.869	0.508	0.513	1.481	0.501
19130	0.497	1.380	0.050	0.462	1.133	0.508	0.511	1.464	0.185
19131	0.503	1.413	0.508	0.447	1.039	0.102	0.489	1.329	0.508
19132	0.297	0.099	0.097	0.275	-0.073	0.102	0.226	-0.356	0.492
19133	0.495	1.363	0.097	0.427	0.907	0.512	0.513	1.481	0.142
19134	0.513	1.481	0.097	0.515	1.472	0.512	0.505	1.430	0.501
19135	0.097	-1.181	0.512	0.310	0.153	0.149	0.142	-0.895	0.050
19136	0.300	0.116	0.512	0.102	-1.184	0.193	0.184	-0.625	0.296
19137	0.261	-0.137	0.492	0.196	-0.581	0.462	0.142	-0.895	0.296
19138	0.429	0.942	0.512	0.512	1.453	0.501	0.468	1.194	0.262
19139	0.492	1.346	0.481	0.497	1.359	0.491	0.495	1.363	0.513
19140	0.487	1.312	0.492	0.462	1.133	0.514	0.511	1.464	0.358
19141	0.476	1.245	0.508	0.497	1.359	0.478	0.513	1.481	0.185
19142	0.479	1.262	0.296	0.371	0.549	0.478	0.500	1.397	0.358
19143	0.300	0.116	0.142	0.374	0.568	0.501	0.358	0.487	0.450
19144	0.300	0.116	0.296	0.374	0.568	0.273	0.358	0.487	0.513
19145	0.482	1.279	0.296	0.503	1.396	0.491	0.455	1.110	0.262
19146	0.300	0.116	0.431	0.102	-1.184	0.514	0.142	-0.895	0.431
19147	0.495	1.363	0.492	0.503	1.396	0.491	0.516	1.498	0.385
19148	0.429	0.942	0.508	0.497	1.359	0.235	0.508	1.447	0.501
19149	0.505	1.430	0.296	0.477	1.227	0.512	0.500	1.397	0.224
19150	0.142	-0.895	0.508	0.237	-0.317	0.462	0.332	0.318	0.296

19151	0.297	0.099	0.262	0.275	-0.073	0.501	0.384	0.655	0.328
19152	0.295	0.082	0.501	0.275	-0.073	0.371	0.384	0.655	0.296
19153	0.142	-0.895	0.450	0.275	-0.073	0.514	0.332	0.318	0.262
19154	0.426	0.925	0.501	0.488	1.302	0.512	0.513	1.481	0.492
19155	0.447	1.060	0.450	0.506	1.415	0.512	0.513	1.481	0.358
19156	0.447	1.060	0.512	0.506	1.415	0.512	0.513	1.481	0.512
19157	0.329	0.301	0.501	0.102	-1.184	0.512	0.187	-0.608	0.501
19158	0.329	0.301	0.467	0.102	-1.184	0.102	0.187	-0.608	0.185
19159	0.489	1.329	0.481	0.512	1.453	0.371	0.505	1.430	0.513
19160	0.479	1.262	0.501	0.515	1.472	0.371	0.500	1.397	0.492
19161	0.229	-0.339	0.513	0.149	-0.882	0.512	0.184	-0.625	0.097
19162	0.224	-0.373	0.097	0.193	-0.600	0.235	0.142	-0.895	0.450
19163	0.508	1.447	0.296	0.459	1.114	0.514	0.500	1.397	0.262
19164	0.492	1.346	0.450	0.500	1.378	0.501	0.429	0.942	0.508
19165	0.513	1.481	0.185	0.494	1.340	0.462	0.484	1.295	0.492
19166	0.511	1.464	0.185	0.515	1.472	0.508	0.508	1.447	0.508
19167	0.505	1.430	0.467	0.477	1.227	0.491	0.508	1.447	0.508
19168	0.508	1.447	0.450	0.477	1.227	0.512	0.511	1.464	0.492
19169	0.500	1.397	0.224	0.497	1.359	0.398	0.492	1.346	0.262
19170	0.226	-0.356	0.492	0.272	-0.091	0.491	0.184	-0.625	0.481
19171	0.508	1.447	0.185	0.488	1.302	0.501	0.505	1.430	0.450
19172	0.492	1.346	0.467	0.515	1.472	0.235	0.513	1.481	0.097
19173	0.492	1.346	0.513	0.491	1.321	0.193	0.508	1.447	0.481
19174	0.226	-0.356	0.513	0.237	-0.317	0.501	0.263	-0.120	0.328
19175	0.384	0.655	0.050	0.515	1.472	0.462	0.505	1.430	0.328
19176	0.511	1.464	0.142	0.459	1.114	0.235	0.511	1.464	0.328
19177	0.492	1.346	0.492	0.497	1.359	0.444	0.511	1.464	0.508
19178	0.508	1.447	0.262	0.368	0.530	0.309	0.492	1.346	0.467
19179	0.503	1.413	0.262	0.515	1.472	0.491	0.508	1.447	0.513
19180	0.500	1.397	0.467	0.509	1.434	0.491	0.503	1.413	0.467
19181	0.500	1.397	0.467	0.509	1.434	0.462	0.503	1.413	0.508
19182	0.500	1.397	0.512	0.509	1.434	0.422	0.503	1.413	0.508
19183	0.500	1.397	0.262	0.509	1.434	0.235	0.503	1.413	0.481
19184	0.295	0.082	0.262	0.102	-1.184	0.309	0.184	-0.625	0.262
19185	0.405	0.790	0.262	0.374	0.568	0.501	0.513	1.481	0.185
19186	0.405	0.790	0.262	0.374	0.568	0.501	0.513	1.481	0.224
19187	0.503	1.413	0.142	0.518	1.491	0.512	0.334	0.335	0.224
19188	0.226	-0.356	0.501	0.234	-0.336	0.514	0.097	-1.181	0.492
19189	0.382	0.638	0.501	0.512	1.453	0.422	0.508	1.447	0.512
19190	0.492	1.346	0.481	0.500	1.378	0.501	0.474	1.228	0.467
19191	0.447	1.060	0.185	0.468	1.170	0.422	0.429	0.942	0.224
19192	0.474	1.228	0.224	0.506	1.415	0.512	0.384	0.655	0.467
19193	0.518	1.514	0.501	0.491	1.321	0.508	0.384	0.655	0.097
19194	0.471	1.211	0.185	0.509	1.434	0.508	0.453	1.093	0.512
19195	0.455	1.110	0.512	0.398	0.718	0.508	0.495	1.363	0.508
19196	0.503	1.413	0.467	0.482	1.265	0.508	0.511	1.464	0.467
19197	0.508	1.447	0.450	0.503	1.396	0.491	0.474	1.228	0.508
19198	0.263	-0.120	0.467	0.237	-0.317	0.273	0.050	-1.485	0.492
19199	0.329	0.301	0.512	0.196	-0.581	0.462	0.000	?	0.501
19200	0.432	0.958	0.512	0.500	1.378	0.444	0.508	1.447	0.492
19201	0.492	1.346	0.431	0.465	1.152	0.462	0.500	1.397	0.512
19202	0.145	-0.878	0.142	0.234	-0.336	0.149	0.261	-0.137	0.501
19203	0.492	1.346	0.492	0.442	1.001	0.102	0.476	1.245	0.508
19204	0.050	-1.485	0.431	0.310	0.153	0.478	0.184	-0.625	0.224
19205	0.484	1.295	0.492	0.494	1.340	0.478	0.497	1.380	0.262
19206	0.484	1.295	0.513	0.494	1.340	0.273	0.505	1.430	0.492
19207	0.513	1.481	0.050	0.459	1.114	0.371	0.453	1.093	0.358
19208	0.495	1.363	0.431	0.424	0.888	0.514	0.516	1.498	0.358
19209	0.411	0.824	0.431	0.234	-0.336	0.309	0.226	-0.356	0.501
19210	0.184	-0.625	0.481	0.307	0.135	0.422	0.050	-1.485	0.512
19211	0.508	1.447	0.508	0.494	1.340	0.341	0.513	1.481	0.450

19212	0.508	1.447	0.328	0.494	1.340	0.491	0.513	1.481	0.481
19213	0.508	1.447	0.224	0.506	1.415	0.512	0.513	1.481	0.262
19214	0.471	1.211	0.224	0.515	1.472	0.512	0.432	0.958	0.508
19215	0.505	1.430	0.501	0.424	0.888	0.309	0.450	1.076	0.508
19216	0.511	1.464	0.050	0.503	1.396	0.512	0.513	1.481	0.508
19217	0.500	1.397	0.501	0.427	0.907	0.478	0.387	0.672	0.142
19218	0.503	1.413	0.328	0.506	1.415	0.309	0.482	1.279	0.224
19219	0.492	1.346	0.262	0.503	1.396	0.508	0.326	0.285	0.508
19220	0.466	1.178	0.296	0.506	1.415	0.514	0.497	1.380	0.185
19221	0.508	1.447	0.185	0.515	1.472	0.512	0.505	1.430	0.185
19222	0.508	1.447	0.262	0.515	1.472	0.273	0.505	1.430	0.512
19223	0.495	1.363	0.512	0.485	1.283	0.491	0.513	1.481	0.492
19224	0.297	0.099	0.513	0.275	-0.073	0.508	0.263	-0.120	0.431
19225	0.495	1.363	0.512	0.462	1.133	0.501	0.505	1.430	0.296
19226	0.516	1.498	0.185	0.444	1.020	0.341	0.429	0.942	0.142
19227	0.505	1.430	0.224	0.462	1.133	0.462	0.463	1.161	0.097
19228	0.226	-0.356	0.224	0.149	-0.882	0.512	0.329	0.301	0.142
19229	0.226	-0.356	0.508	0.102	-1.184	0.501	0.329	0.301	0.328
19230	0.503	1.413	0.431	0.482	1.265	0.501	0.508	1.447	0.481
19231	0.503	1.413	0.142	0.482	1.265	0.193	0.508	1.447	0.481
19232	0.263	-0.120	0.492	0.275	-0.073	0.273	0.361	0.504	0.508
19233	0.329	0.301	0.492	0.374	0.568	0.508	0.387	0.672	0.262
19234	0.508	1.447	0.508	0.518	1.491	0.491	0.476	1.245	0.224
19235	0.184	-0.625	0.508	0.310	0.153	0.193	0.187	-0.608	0.501
19236	0.474	1.228	0.142	0.424	0.888	0.398	0.508	1.447	0.358
19237	0.355	0.470	0.142	0.339	0.342	0.512	0.295	0.082	0.224
19238	0.489	1.329	0.262	0.488	1.302	0.235	0.513	1.481	0.467
19239	0.505	1.430	0.513	0.512	1.453	0.102	0.463	1.161	0.513
19240	0.432	0.958	0.262	0.515	1.472	0.341	0.434	0.975	0.501
19241	0.513	1.481	0.224	0.310	0.153	0.341	0.516	1.498	0.492
19242	0.495	1.363	0.224	0.515	1.472	0.235	0.511	1.464	0.508
19243	0.500	1.397	0.185	0.480	1.246	0.149	0.508	1.447	0.481
19244	0.000	?	0.296	0.307	0.135	0.309	0.295	0.082	0.492
19245	0.497	1.380	0.358	0.509	1.434	0.341	0.411	0.824	0.262
19246	0.513	1.481	0.481	0.515	1.472	0.341	0.479	1.262	0.492
19247	0.513	1.481	0.501	0.515	1.472	0.512	0.479	1.262	0.501
19248	0.224	-0.373	0.097	0.275	-0.073	0.501	0.142	-0.895	0.508
19249	0.476	1.245	0.097	0.491	1.321	0.501	0.508	1.447	0.492
19250	0.516	1.498	0.185	0.506	1.415	0.514	0.326	0.285	0.224
19251	0.505	1.430	0.185	0.500	1.378	0.444	0.382	0.638	0.142
19252	0.187	-0.608	0.328	0.342	0.361	0.478	0.142	-0.895	0.296
19253	0.453	1.093	0.224	0.465	1.152	0.491	0.505	1.430	0.185
19254	0.492	1.346	0.262	0.515	1.472	0.371	0.487	1.312	0.492
19255	0.426	0.925	0.224	0.497	1.359	0.193	0.492	1.346	0.492
19256	0.426	0.925	0.142	0.497	1.359	0.491	0.492	1.346	0.431
19257	0.226	-0.356	0.492	0.193	-0.600	0.371	0.184	-0.625	0.467
19258	0.226	-0.356	0.481	0.272	-0.091	0.478	0.184	-0.625	0.296
19259	0.432	0.958	0.328	0.503	1.396	0.309	0.466	1.178	0.142
19260	0.482	1.279	0.512	0.494	1.340	0.478	0.429	0.942	0.185
19261	0.184	-0.625	0.512	0.196	-0.581	0.512	0.184	-0.625	0.492
19262	0.142	-0.895	0.512	0.398	0.718	0.398	0.145	-0.878	0.409
19263	0.505	1.430	0.512	0.512	1.453	0.444	0.408	0.807	0.262
19264	0.184	-0.625	0.142	0.240	-0.299	0.341	0.297	0.099	0.512
19265	0.226	-0.356	0.385	0.102	-1.184	0.462	0.261	-0.137	0.262
19266	0.300	0.116	0.142	0.342	0.361	0.512	0.261	-0.137	0.224
19267	0.329	0.301	0.512	0.342	0.361	0.149	0.300	0.116	0.262
19268	0.142	-0.895	0.508	0.237	-0.317	0.501	0.263	-0.120	0.185
19269	0.145	-0.878	0.328	0.149	-0.882	0.444	0.261	-0.137	0.097
19270	0.224	-0.373	0.262	0.307	0.135	0.462	0.332	0.318	0.512
19271	0.184	-0.625	0.467	0.342	0.361	0.512	0.184	-0.625	0.492
19272	0.142	-0.895	0.512	0.342	0.361	0.491	0.184	-0.625	0.513

19273	0.503	1.413	0.512	0.515	1.472	0.508	0.476	1.245	0.358
19274	0.495	1.363	0.050	0.500	1.378	0.422	0.508	1.447	0.142
19275	0.495	1.363	0.492	0.500	1.378	0.462	0.508	1.447	0.262
19276	0.468	1.194	0.501	0.515	1.472	0.508	0.500	1.397	0.513
19277	0.492	1.346	0.224	0.447	1.039	0.514	0.513	1.481	0.385
19278	0.503	1.413	0.262	0.477	1.227	0.512	0.513	1.481	0.224
19279	0.495	1.363	0.262	0.491	1.321	0.309	0.497	1.380	0.508
19280	0.097	-1.181	0.467	0.374	0.568	0.102	0.224	-0.373	0.508
19281	0.463	1.161	0.481	0.193	-0.600	0.462	0.224	-0.373	0.492
19282	0.492	1.346	0.328	0.494	1.340	0.462	0.466	1.178	0.492
19283	0.261	-0.137	0.513	0.374	0.568	0.514	0.187	-0.608	0.296
19284	0.387	0.672	0.513	0.482	1.265	0.273	0.450	1.076	0.328
19285	0.184	-0.625	0.050	0.310	0.153	0.444	0.184	-0.625	0.262
19286	0.479	1.262	0.050	0.482	1.265	0.235	0.508	1.447	0.481
19287	0.489	1.329	0.050	0.512	1.453	0.398	0.405	0.790	0.097
19288	0.426	0.925	0.262	0.395	0.700	0.462	0.513	1.481	0.142
19289	0.500	1.397	0.501	0.447	1.039	0.491	0.508	1.447	0.224
19290	0.100	-1.164	0.142	0.339	0.342	0.462	0.184	-0.625	0.185
19291	0.500	1.397	0.385	0.465	1.152	0.309	0.513	1.481	0.097
19292	0.482	1.279	0.467	0.515	1.472	0.501	0.471	1.211	0.097
19293	0.184	-0.625	0.385	0.152	-0.863	0.235	0.295	0.082	0.450
19294	0.495	1.363	0.450	0.503	1.396	0.501	0.405	0.790	0.385
19295	0.503	1.413	0.296	0.447	1.039	0.444	0.513	1.481	0.328
19296	0.489	1.329	0.501	0.465	1.152	0.235	0.482	1.279	0.296
19297	0.503	1.413	0.501	0.509	1.434	0.508	0.495	1.363	0.296
19298	0.468	1.194	0.513	0.488	1.302	0.273	0.503	1.413	0.142
19299	0.468	1.194	0.492	0.506	1.415	0.309	0.497	1.380	0.296
19300	0.453	1.093	0.492	0.427	0.907	0.273	0.492	1.346	0.224
19301	0.447	1.060	0.185	0.465	1.152	0.273	0.508	1.447	0.224
19302	0.503	1.413	0.501	0.512	1.453	0.478	0.503	1.413	0.185
19303	0.500	1.397	0.508	0.509	1.434	0.053	0.492	1.346	0.142
19304	0.505	1.430	0.508	0.509	1.434	0.309	0.489	1.329	0.467
19305	0.263	-0.120	0.501	0.310	0.153	0.193	0.263	-0.120	0.508
19306	0.297	0.099	0.097	0.102	-1.184	0.422	0.142	-0.895	0.142
19307	0.445	1.043	0.508	0.459	1.114	0.478	0.505	1.430	0.481
19308	0.445	1.043	0.224	0.459	1.114	0.273	0.505	1.430	0.450
19309	0.497	1.380	0.501	0.509	1.434	0.193	0.453	1.093	0.492
19310	0.387	0.672	0.467	0.272	-0.091	0.478	0.263	-0.120	0.492
19311	0.508	1.447	0.513	0.444	1.020	0.422	0.505	1.430	0.296
19312	0.142	-0.895	0.492	0.234	-0.336	0.193	0.263	-0.120	0.097
19313	0.497	1.380	0.358	0.401	0.737	0.193	0.489	1.329	0.185
19314	0.497	1.380	0.185	0.465	1.152	0.149	0.489	1.329	0.431
19315	0.500	1.397	0.481	0.494	1.340	0.149	0.508	1.447	0.492
19316	0.503	1.413	0.185	0.465	1.152	0.193	0.511	1.464	0.328
19317	0.000	?	0.185	0.310	0.153	0.514	0.226	-0.356	0.328
19318	0.455	1.110	0.185	0.497	1.359	0.053	0.497	1.380	0.492
19319	0.361	0.504	0.262	0.234	-0.336	0.512	0.184	-0.625	0.467
19320	0.405	0.790	0.185	0.503	1.396	0.508	0.505	1.430	0.512
19321	0.495	1.363	0.501	0.442	1.001	0.235	0.487	1.312	0.262
19322	0.263	-0.120	0.501	0.234	-0.336	0.491	0.142	-0.895	0.501
19323	0.482	1.279	0.501	0.506	1.415	0.501	0.503	1.413	0.508
19324	0.361	0.504	0.481	0.275	-0.073	0.235	0.050	-1.485	0.097
19325	0.145	-0.878	0.492	0.310	0.153	0.512	0.295	0.082	0.296
19326	0.000	?	0.467	0.272	-0.091	0.193	0.297	0.099	0.328
19327	0.050	-1.485	0.450	0.272	-0.091	0.491	0.261	-0.137	0.508
19328	0.505	1.430	0.409	0.480	1.246	0.508	0.516	1.498	0.508
19329	0.184	-0.625	0.358	0.053	-1.504	0.478	0.361	0.504	0.262
19330	0.224	-0.373	0.450	0.307	0.135	0.444	0.187	-0.608	0.492
19331	0.184	-0.625	0.262	0.193	-0.600	0.444	0.358	0.487	0.492
19332	0.508	1.447	0.185	0.424	0.888	0.273	0.447	1.060	0.296
19333	0.500	1.397	0.328	0.480	1.246	0.514	0.513	1.481	0.262

19334	0.000	?	0.467	0.275	-0.073	0.491	0.263	-0.120	0.296
19335	0.000	?	0.467	0.196	-0.581	0.422	0.432	0.958	0.185
19336	0.500	1.397	0.508	0.485	1.283	0.462	0.384	0.655	0.467
19337	0.487	1.312	0.501	0.418	0.850	0.149	0.505	1.430	0.328
19338	0.142	-0.895	0.501	0.193	-0.600	0.149	0.226	-0.356	0.224
19339	0.184	-0.625	0.467	0.193	-0.600	0.149	0.300	0.116	0.492
19340	0.266	-0.103	0.508	0.149	-0.882	0.478	0.326	0.285	0.224
19341	0.097	-1.181	0.481	0.149	-0.882	0.508	0.295	0.082	0.508
19342	0.361	0.504	0.097	0.193	-0.600	0.514	0.261	-0.137	0.142
19343	0.497	1.380	0.512	0.515	1.472	0.491	0.492	1.346	0.492
19344	0.100	-1.164	0.142	0.053	-1.504	0.508	0.358	0.487	0.492
19345	0.384	0.655	0.501	0.506	1.415	0.491	0.511	1.464	0.450
19346	0.500	1.397	0.501	0.512	1.453	0.512	0.505	1.430	0.467
19347	0.142	-0.895	0.262	0.234	-0.336	0.309	0.187	-0.608	0.467
19348	0.513	1.481	0.262	0.497	1.359	0.309	0.511	1.464	0.224
19349	0.479	1.262	0.481	0.506	1.415	0.235	0.489	1.329	0.467
19350	0.261	-0.137	0.262	0.237	-0.317	0.235	0.097	-1.181	0.467
19351	0.489	1.329	0.358	0.512	1.453	0.491	0.447	1.060	0.467
19352	0.184	-0.625	0.296	0.193	-0.600	0.478	0.261	-0.137	0.431
19353	0.484	1.295	0.185	0.494	1.340	0.491	0.505	1.430	0.142
19354	0.497	1.380	0.262	0.509	1.434	0.193	0.492	1.346	0.492
19355	0.508	1.447	0.512	0.477	1.227	0.512	0.511	1.464	0.185
19356	0.511	1.464	0.224	0.442	1.001	0.193	0.511	1.464	0.481
19357	0.503	1.413	0.185	0.442	1.001	0.508	0.495	1.363	0.501
19358	0.000	?	0.097	0.275	-0.073	0.462	0.261	-0.137	0.328
19359	0.505	1.430	0.508	0.515	1.472	0.514	0.482	1.279	0.492
19360	0.466	1.178	0.508	0.494	1.340	0.501	0.447	1.060	0.358
19361	0.050	-1.485	0.262	0.424	0.888	0.462	0.097	-1.181	0.224
19362	0.497	1.380	0.512	0.468	1.170	0.512	0.476	1.245	0.512
19363	0.097	-1.181	0.262	0.149	-0.882	0.512	0.329	0.301	0.262
19364	0.097	-1.181	0.262	0.149	-0.882	0.398	0.329	0.301	0.328
19365	0.097	-1.181	0.450	0.149	-0.882	0.512	0.329	0.301	0.296
19366	0.511	1.464	0.185	0.474	1.208	0.444	0.508	1.447	0.328
19367	0.516	1.498	0.481	0.503	1.396	0.235	0.466	1.178	0.296
19368	0.489	1.329	0.513	0.509	1.434	0.102	0.516	1.498	0.513
19369	0.511	1.464	0.508	0.491	1.321	0.501	0.463	1.161	0.467
19370	0.476	1.245	0.224	0.506	1.415	0.462	0.505	1.430	0.513
19371	0.484	1.295	0.185	0.488	1.302	0.491	0.511	1.464	0.513
19372	0.503	1.413	0.224	0.515	1.472	0.149	0.484	1.295	0.513
19373	0.297	0.099	0.508	0.310	0.153	0.514	0.263	-0.120	0.512
19374	0.142	-0.895	0.481	0.313	0.172	0.149	0.184	-0.625	0.481
19375	0.297	0.099	0.481	0.237	-0.317	0.501	0.226	-0.356	0.450
19376	0.297	0.099	0.262	0.237	-0.317	0.462	0.226	-0.356	0.224
19377	0.432	0.958	0.185	0.485	1.283	0.478	0.487	1.312	0.450
19378	0.489	1.329	0.501	0.474	1.208	0.398	0.513	1.481	0.097
19379	0.505	1.430	0.409	0.491	1.321	0.235	0.468	1.194	0.097
19380	0.297	0.099	0.467	0.193	-0.600	0.491	0.224	-0.373	0.296
19381	0.508	1.447	0.467	0.512	1.453	0.235	0.466	1.178	0.501
19382	0.261	-0.137	0.467	0.193	-0.600	0.235	0.097	-1.181	0.501
19383	0.492	1.346	0.501	0.509	1.434	0.514	0.513	1.481	0.512
19384	0.450	1.076	0.501	0.462	1.133	0.508	0.503	1.413	0.512
19385	0.492	1.346	0.501	0.515	1.472	0.508	0.466	1.178	0.501
19386	0.445	1.043	0.431	0.500	1.378	0.149	0.511	1.464	0.508
19387	0.505	1.430	0.328	0.462	1.133	0.193	0.492	1.346	0.512
19388	0.497	1.380	0.431	0.512	1.453	0.193	0.505	1.430	0.513
19389	0.463	1.161	0.431	0.509	1.434	0.193	0.495	1.363	0.328
19390	0.482	1.279	0.224	0.401	0.737	0.309	0.513	1.481	0.409
19391	0.503	1.413	0.501	0.512	1.453	0.478	0.497	1.380	0.262
19392	0.516	1.498	0.224	0.442	1.001	0.491	0.505	1.430	0.512
19393	0.100	-1.164	0.385	0.237	-0.317	0.478	0.224	-0.373	0.508
19394	0.295	0.082	0.512	0.105	-1.165	0.341	0.263	-0.120	0.097

19395	0.447	1.060	0.185	0.503	1.396	0.235	0.495	1.363	0.328
19396	0.187	−0.608	0.508	0.000	?	0.514	0.358	0.487	0.481
19397	0.187	−0.608	0.512	0.000	?	0.235	0.358	0.487	0.185
19398	0.474	1.228	0.501	0.465	1.152	0.149	0.497	1.380	0.224
19399	0.453	1.093	0.501	0.494	1.340	0.478	0.513	1.481	0.097
19400	0.226	−0.356	0.501	0.149	−0.882	0.491	0.453	1.093	0.224
19401	0.492	1.346	0.328	0.518	1.491	0.508	0.476	1.245	0.224
19402	0.187	−0.608	0.224	0.149	−0.882	0.501	0.261	−0.137	0.508
19403	0.466	1.178	0.481	0.506	1.415	0.478	0.505	1.430	0.185
19404	0.511	1.464	0.224	0.459	1.114	0.444	0.511	1.464	0.224
19405	0.511	1.464	0.262	0.477	1.227	0.512	0.511	1.464	0.328
19406	0.050	−1.485	0.508	0.398	0.718	0.309	0.142	−0.895	0.450
19407	0.142	−0.895	0.450	0.234	−0.336	0.501	0.226	−0.356	0.450
19408	0.495	1.363	0.296	0.491	1.321	0.462	0.505	1.430	0.185
19409	0.263	−0.120	0.512	0.234	−0.336	0.491	0.187	−0.608	0.492
19410	0.263	−0.120	0.262	0.234	−0.336	0.501	0.187	−0.608	0.142
19411	0.466	1.178	0.508	0.520	1.509	0.309	0.508	1.447	0.142
19412	0.466	1.178	0.508	0.515	1.472	0.398	0.489	1.329	0.450
19413	0.513	1.481	0.492	0.506	1.415	0.371	0.429	0.942	0.224
19414	0.263	−0.120	0.492	0.152	−0.863	0.371	0.297	0.099	0.492
19415	0.263	−0.120	0.508	0.196	−0.581	0.501	0.142	−0.895	0.492
19416	0.263	−0.120	0.358	0.196	−0.581	0.508	0.097	−1.181	0.431
19417	0.263	−0.120	0.358	0.196	−0.581	0.491	0.142	−0.895	0.097
19418	0.142	−0.895	0.358	0.307	0.135	0.398	0.326	0.285	0.097
19419	0.495	1.363	0.450	0.480	1.246	0.235	0.482	1.279	0.142
19420	0.495	1.363	0.185	0.491	1.321	0.193	0.482	1.279	0.431
19421	0.482	1.279	0.467	0.480	1.246	0.273	0.503	1.413	0.508
19422	0.184	−0.625	0.481	0.345	0.379	0.444	0.263	−0.120	0.467
19423	0.224	−0.373	0.481	0.234	−0.336	0.102	0.224	−0.373	0.262
19424	0.492	1.346	0.481	0.515	1.472	0.273	0.500	1.397	0.142
19425	0.000	?	0.512	0.234	−0.336	0.491	0.358	0.487	0.512
19426	0.184	−0.625	0.508	0.152	−0.863	0.309	0.226	−0.356	0.501
19427	0.508	1.447	0.185	0.480	1.246	0.235	0.466	1.178	0.512
19428	0.463	1.161	0.296	0.491	1.321	0.309	0.513	1.481	0.501
19429	0.445	1.043	0.050	0.509	1.434	0.273	0.500	1.397	0.501
19430	0.466	1.178	0.513	0.500	1.378	0.235	0.489	1.329	0.467
19431	0.505	1.430	0.513	0.480	1.246	0.422	0.503	1.413	0.450
19432	0.513	1.481	0.050	0.444	1.020	0.462	0.482	1.279	0.450
19433	0.434	0.975	0.467	0.512	1.453	0.462	0.492	1.346	0.508
19434	0.145	−0.878	0.328	0.310	0.153	0.193	0.261	−0.137	0.185
19435	0.495	1.363	0.501	0.500	1.378	0.341	0.500	1.397	0.508
19436	0.429	0.942	0.358	0.459	1.114	0.422	0.508	1.447	0.508
19437	0.495	1.363	0.358	0.491	1.321	0.462	0.513	1.481	0.185
19438	0.503	1.413	0.409	0.506	1.415	0.053	0.492	1.346	0.481
19439	0.000	?	0.050	0.313	0.172	0.273	0.224	−0.373	0.185
19440	0.000	?	0.224	0.401	0.737	0.462	0.142	−0.895	0.512
19441	0.050	−1.485	0.385	0.377	0.587	0.462	0.295	0.082	0.501
19442	0.000	?	0.050	0.374	0.568	0.514	0.184	−0.625	0.409
19443	0.429	0.942	0.467	0.497	1.359	0.514	0.492	1.346	0.481
19444	0.429	0.942	0.262	0.506	1.415	0.273	0.492	1.346	0.467
19445	0.479	1.262	0.262	0.488	1.302	0.273	0.432	0.958	0.512
19446	0.505	1.430	0.467	0.398	0.718	0.309	0.463	1.161	0.512
19447	0.326	0.285	0.512	0.237	−0.317	0.491	0.297	0.099	0.409
19448	0.226	−0.356	0.097	0.193	−0.600	0.273	0.142	−0.895	0.385
19449	0.226	−0.356	0.501	0.275	−0.073	0.341	0.184	−0.625	0.262
19450	0.505	1.430	0.097	0.447	1.039	0.273	0.495	1.363	0.450
19451	0.050	−1.485	0.513	0.105	−1.165	0.309	0.408	0.807	0.262
19452	0.000	?	0.513	0.275	−0.073	0.149	0.261	−0.137	0.142
19453	0.503	1.413	0.296	0.482	1.265	0.149	0.511	1.464	0.481
19454	0.326	0.285	0.296	0.307	0.135	0.501	0.263	−0.120	0.508
19455	0.261	−0.137	0.481	0.234	−0.336	0.514	0.224	−0.373	0.097

19456	0.297	0.099	0.142	0.307	0.135	0.273	0.263	-0.120	0.467
19457	0.187	-0.608	0.050	0.272	-0.091	0.273	0.187	-0.608	0.385
19458	0.263	-0.120	0.224	0.234	-0.336	0.102	0.097	-1.181	0.501
19459	0.505	1.430	0.492	0.421	0.869	0.235	0.511	1.464	0.508
19460	0.508	1.447	0.185	0.462	1.133	0.235	0.489	1.329	0.508
19461	0.513	1.481	0.501	0.462	1.133	0.193	0.518	1.514	0.431
19462	0.184	-0.625	0.501	0.193	-0.600	0.193	0.361	0.504	0.508
19463	0.224	-0.373	0.481	0.342	0.361	0.273	0.142	-0.895	0.142
19464	0.224	-0.373	0.224	0.430	0.926	0.273	0.261	-0.137	0.097
19465	0.505	1.430	0.224	0.465	1.152	0.491	0.511	1.464	0.512
19466	0.429	0.942	0.508	0.053	-1.504	0.478	0.387	0.672	0.501
19467	0.145	-0.878	0.224	0.275	-0.073	0.053	0.224	-0.373	0.185
19468	0.492	1.346	0.481	0.459	1.114	0.514	0.503	1.413	0.508
19469	0.492	1.346	0.512	0.459	1.114	0.514	0.503	1.413	0.508
19470	0.508	1.447	0.262	0.515	1.472	0.491	0.489	1.329	0.508
19471	0.508	1.447	0.501	0.515	1.472	0.491	0.489	1.329	0.508
19472	0.142	-0.895	0.492	0.275	-0.073	0.102	0.300	0.116	0.508
19473	0.142	-0.895	0.512	0.275	-0.073	0.149	0.332	0.318	0.409
19474	0.263	-0.120	0.508	0.310	0.153	0.273	0.261	-0.137	0.185
19475	0.513	1.481	0.224	0.494	1.340	0.462	0.479	1.262	0.142
19476	0.261	-0.137	0.492	0.275	-0.073	0.478	0.097	-1.181	0.431
19477	0.224	-0.373	0.262	0.339	0.342	0.053	0.142	-0.895	0.501
19478	0.224	-0.373	0.185	0.272	-0.091	0.309	0.226	-0.356	0.501
19479	0.184	-0.625	0.262	0.310	0.153	0.508	0.184	-0.625	0.185
19480	0.295	0.082	0.450	0.149	-0.882	0.514	0.097	-1.181	0.508
19481	0.358	0.487	0.512	0.149	-0.882	0.444	0.100	-1.164	0.097
19482	0.476	1.245	0.512	0.509	1.434	0.398	0.450	1.076	0.512
19483	0.492	1.346	0.513	0.515	1.472	0.501	0.382	0.638	0.224
19484	0.097	-1.181	0.513	0.272	-0.091	0.512	0.326	0.285	0.508
19485	0.097	-1.181	0.513	0.272	-0.091	0.273	0.295	0.082	0.508
19486	0.184	-0.625	0.512	0.102	-1.184	0.398	0.295	0.082	0.224
19487	0.187	-0.608	0.050	0.234	-0.336	0.149	0.142	-0.895	0.050
19488	0.329	0.301	0.492	0.234	-0.336	0.508	0.297	0.099	0.224
19489	0.224	-0.373	0.492	0.193	-0.600	0.508	0.224	-0.373	0.262
19490	0.261	-0.137	0.492	0.193	-0.600	0.149	0.224	-0.373	0.431
19491	0.224	-0.373	0.185	0.275	-0.073	0.422	0.187	-0.608	0.097
19492	0.142	-0.895	0.224	0.278	-0.054	0.422	0.142	-0.895	0.508
19493	0.489	1.329	0.296	0.488	1.302	0.273	0.461	1.144	0.508
19494	0.484	1.295	0.185	0.474	1.208	0.273	0.503	1.413	0.224
19495	0.326	0.285	0.185	0.053	-1.504	0.273	0.142	-0.895	0.358
19496	0.508	1.447	0.262	0.512	1.453	0.102	0.476	1.245	0.262
19497	0.508	1.447	0.224	0.509	1.434	0.478	0.447	1.060	0.492
19498	0.508	1.447	0.501	0.488	1.302	0.149	0.492	1.346	0.450
19499	0.508	1.447	0.492	0.488	1.302	0.102	0.492	1.346	0.358
19500	0.142	-0.895	0.508	0.102	-1.184	0.514	0.295	0.082	0.142
19501	0.387	0.672	0.508	0.149	-0.882	0.102	0.097	-1.181	0.431
19502	0.145	-0.878	0.508	0.272	-0.091	0.491	0.184	-0.625	0.142
19503	0.511	1.464	0.508	0.468	1.170	0.371	0.426	0.925	0.185
19504	0.497	1.380	0.224	0.477	1.227	0.491	0.497	1.380	0.097
19505	0.329	0.301	0.185	0.053	-1.504	0.491	0.329	0.301	0.409
19506	0.263	-0.120	0.185	0.313	0.172	0.512	0.329	0.301	0.224
19507	0.466	1.178	0.481	0.506	1.415	0.501	0.489	1.329	0.492
19508	0.503	1.413	0.481	0.515	1.472	0.501	0.468	1.194	0.481
19509	0.500	1.397	0.481	0.444	1.020	0.371	0.516	1.498	0.501
19510	0.050	-1.485	0.481	0.398	0.718	0.491	0.266	-0.103	0.224
19511	0.487	1.312	0.450	0.500	1.378	0.508	0.505	1.430	0.262
19512	0.497	1.380	0.262	0.512	1.453	0.508	0.503	1.413	0.409
19513	0.224	-0.373	0.262	0.275	-0.073	0.491	0.100	-1.164	0.262
19514	0.263	-0.120	0.296	0.401	0.737	0.371	0.297	0.099	0.481
19515	0.263	-0.120	0.450	0.149	-0.882	0.508	0.329	0.301	0.513
19516	0.463	1.161	0.450	0.512	1.453	0.193	0.508	1.447	0.142

19517	0.476	1.245	0.450	0.512	1.453	0.501	0.508	1.447	0.467
19518	0.329	0.301	0.450	0.149	-0.882	0.478	0.261	-0.137	0.467
19519	0.513	1.481	0.450	0.424	0.888	0.508	0.492	1.346	0.508
19520	0.513	1.481	0.481	0.424	0.888	0.514	0.492	1.346	0.481
19521	0.050	-1.485	0.224	0.275	-0.073	0.462	0.297	0.099	0.142
19522	0.050	-1.485	0.050	0.275	-0.073	0.193	0.263	-0.120	0.450
19523	0.050	-1.485	0.512	0.275	-0.073	0.491	0.297	0.099	0.097
19524	0.263	-0.120	0.508	0.102	-1.184	0.235	0.184	-0.625	0.050
19525	0.497	1.380	0.508	0.482	1.265	0.371	0.466	1.178	0.097
19526	0.142	-0.895	0.508	0.152	-0.863	0.309	0.329	0.301	0.513
19527	0.387	0.672	0.512	0.102	-1.184	0.371	0.226	-0.356	0.501
19528	0.468	1.194	0.467	0.518	1.491	0.273	0.489	1.329	0.492
19529	0.387	0.672	0.481	0.102	-1.184	0.478	0.226	-0.356	0.467
19530	0.450	1.076	0.512	0.491	1.321	0.501	0.505	1.430	0.467
19531	0.297	0.099	0.508	0.374	0.568	0.422	0.142	-0.895	0.467
19532	0.505	1.430	0.185	0.491	1.321	0.422	0.487	1.312	0.501
19533	0.505	1.430	0.492	0.491	1.321	0.422	0.487	1.312	0.050
19534	0.508	1.447	0.492	0.515	1.472	0.444	0.447	1.060	0.513
19535	0.489	1.329	0.185	0.500	1.378	0.514	0.463	1.161	0.508
19536	0.489	1.329	0.185	0.500	1.378	0.501	0.463	1.161	0.508
19537	0.187	-0.608	0.262	0.371	0.549	0.398	0.226	-0.356	0.097
19538	0.497	1.380	0.296	0.488	1.302	0.512	0.463	1.161	0.262
19539	0.503	1.413	0.431	0.506	1.415	0.193	0.463	1.161	0.328
19540	0.503	1.413	0.142	0.506	1.415	0.309	0.463	1.161	0.358
19541	0.495	1.363	0.262	0.497	1.359	0.398	0.426	0.925	0.358
19542	0.097	-1.181	0.508	0.374	0.568	0.491	0.142	-0.895	0.358
19543	0.508	1.447	0.142	0.512	1.453	0.491	0.492	1.346	0.296
19544	0.224	-0.373	0.142	0.193	-0.600	0.501	0.184	-0.625	0.492
19545	0.503	1.413	0.224	0.503	1.396	0.501	0.482	1.279	0.512
19546	0.461	1.144	0.492	0.477	1.227	0.514	0.500	1.397	0.512
19547	0.511	1.464	0.492	0.506	1.415	0.508	0.332	0.318	0.512
19548	0.484	1.295	0.262	0.512	1.453	0.491	0.497	1.380	0.512
19549	0.358	0.487	0.097	0.468	1.170	0.491	0.361	0.504	0.512
19550	0.184	-0.625	0.508	0.196	-0.581	0.273	0.226	-0.356	0.224
19551	0.482	1.279	0.501	0.488	1.302	0.512	0.513	1.481	0.142
19552	0.187	-0.608	0.492	0.234	-0.336	0.193	0.261	-0.137	0.296
19553	0.187	-0.608	0.328	0.371	0.549	0.478	0.329	0.301	0.328
19554	0.187	-0.608	0.328	0.313	0.172	0.478	0.295	0.082	0.492
19555	0.263	-0.120	0.501	0.374	0.568	0.341	0.326	0.285	0.492
19556	0.184	-0.625	0.501	0.275	-0.073	0.341	0.297	0.099	0.492
19557	0.497	1.380	0.467	0.471	1.189	0.501	0.513	1.481	0.492
19558	0.503	1.413	0.467	0.503	1.396	0.193	0.463	1.161	0.501
19559	0.505	1.430	0.296	0.424	0.888	0.309	0.511	1.464	0.385
19560	0.479	1.262	0.508	0.418	0.850	0.273	0.513	1.481	0.097
19561	0.489	1.329	0.450	0.418	0.850	0.235	0.513	1.481	0.050
19562	0.463	1.161	0.501	0.442	1.001	0.235	0.511	1.464	0.508
19563	0.453	1.093	0.431	0.512	1.453	0.501	0.484	1.295	0.501
19564	0.411	0.824	0.328	0.500	1.378	0.193	0.453	1.093	0.508
19565	0.363	0.520	0.492	0.398	0.718	0.193	0.224	-0.373	0.508
19566	0.453	1.093	0.142	0.512	1.453	0.102	0.450	1.076	0.508
19567	0.261	-0.137	0.512	0.193	-0.600	0.478	0.097	-1.181	0.358
19568	0.184	-0.625	0.450	0.310	0.153	0.478	0.097	-1.181	0.050
19569	0.332	0.318	0.492	0.398	0.718	0.102	0.295	0.082	0.224
19570	0.468	1.194	0.097	0.491	1.321	0.501	0.503	1.413	0.450
19571	0.468	1.194	0.467	0.491	1.321	0.235	0.503	1.413	0.492
19572	0.503	1.413	0.501	0.500	1.378	0.235	0.508	1.447	0.492
19573	0.497	1.380	0.481	0.500	1.378	0.508	0.508	1.447	0.508
19574	0.500	1.397	0.142	0.506	1.415	0.309	0.505	1.430	0.492
19575	0.466	1.178	0.185	0.500	1.378	0.508	0.511	1.464	0.492
19576	0.508	1.447	0.513	0.491	1.321	0.491	0.513	1.481	0.508
19577	0.479	1.262	0.224	0.488	1.302	0.508	0.516	1.498	0.512

19578	0.100	-1.164	0.185	0.275	-0.073	0.341	0.329	0.301	0.262
19579	0.503	1.413	0.467	0.515	1.472	0.341	0.411	0.824	0.501
19580	0.145	-0.878	0.358	0.196	-0.581	0.341	0.261	-0.137	0.262
19581	0.497	1.380	0.296	0.477	1.227	0.501	0.511	1.464	0.431
19582	0.497	1.380	0.185	0.477	1.227	0.501	0.508	1.447	0.409
19583	0.263	-0.120	0.296	0.345	0.379	0.444	0.097	-1.181	0.185
19584	0.263	-0.120	0.185	0.342	0.361	0.309	0.329	0.301	0.224
19585	0.484	1.295	0.142	0.494	1.340	0.235	0.479	1.262	0.224
19586	0.261	-0.137	0.501	0.193	-0.600	0.478	0.184	-0.625	0.097
19587	0.358	0.487	0.224	0.310	0.153	0.491	0.224	-0.373	0.501
19588	0.295	0.082	0.481	0.272	-0.091	0.491	0.097	-1.181	0.328
19589	0.187	-0.608	0.296	0.234	-0.336	0.512	0.226	-0.356	0.050
19590	0.263	-0.120	0.296	0.234	-0.336	0.508	0.226	-0.356	0.512
19591	0.500	1.397	0.450	0.500	1.378	0.462	0.511	1.464	0.185
19592	0.226	-0.356	0.296	0.193	-0.600	0.508	0.184	-0.625	0.185
19593	0.184	-0.625	0.481	0.193	-0.600	0.512	0.224	-0.373	0.262
19594	0.097	-1.181	0.385	0.102	-1.184	0.444	0.329	0.301	0.508
19595	0.500	1.397	0.296	0.480	1.246	0.149	0.450	1.076	0.508
19596	0.500	1.397	0.508	0.480	1.246	0.444	0.450	1.076	0.185
19597	0.266	-0.103	0.224	0.102	-1.184	0.444	0.184	-0.625	0.262
19598	0.508	1.447	0.501	0.497	1.359	0.235	0.489	1.329	0.513
19599	0.261	-0.137	0.385	0.237	-0.317	0.501	0.142	-0.895	0.513
19600	0.263	-0.120	0.262	0.237	-0.317	0.149	0.142	-0.895	0.501
19601	0.450	1.076	0.501	0.515	1.472	0.512	0.447	1.060	0.328
19602	0.184	-0.625	0.185	0.313	0.172	0.501	0.226	-0.356	0.328
19603	0.482	1.279	0.385	0.515	1.472	0.102	0.489	1.329	0.501
19604	0.508	1.447	0.481	0.488	1.302	0.501	0.489	1.329	0.492
19605	0.508	1.447	0.492	0.506	1.415	0.514	0.426	0.925	0.296
19606	0.226	-0.356	0.481	0.342	0.361	0.462	0.097	-1.181	0.508
19607	0.187	-0.608	0.513	0.342	0.361	0.462	0.097	-1.181	0.508
19608	0.226	-0.356	0.501	0.342	0.361	0.508	0.142	-0.895	0.142
19609	0.508	1.447	0.512	0.497	1.359	0.149	0.426	0.925	0.501
19610	0.482	1.279	0.358	0.506	1.415	0.102	0.503	1.413	0.467
19611	0.482	1.279	0.513	0.444	1.020	0.462	0.466	1.178	0.450
19612	0.261	-0.137	0.097	0.310	0.153	0.235	0.263	-0.120	0.481
19613	0.184	-0.625	0.512	0.240	-0.299	0.273	0.142	-0.895	0.097
19614	0.495	1.363	0.467	0.477	1.227	0.501	0.511	1.464	0.450
19615	0.408	0.807	0.467	0.497	1.359	0.508	0.495	1.363	0.358
19616	0.466	1.178	0.467	0.497	1.359	0.149	0.503	1.413	0.431
19617	0.463	1.161	0.481	0.518	1.491	0.508	0.495	1.363	0.512
19618	0.463	1.161	0.512	0.512	1.453	0.149	0.495	1.363	0.492
19619	0.500	1.397	0.450	0.459	1.114	0.512	0.463	1.161	0.142
19620	0.503	1.413	0.492	0.506	1.415	0.512	0.447	1.060	0.358
19621	0.503	1.413	0.050	0.509	1.434	0.512	0.447	1.060	0.450
19622	0.429	0.942	0.185	0.439	0.982	0.514	0.505	1.430	0.481
19623	0.329	0.301	0.481	0.149	-0.882	0.478	0.184	-0.625	0.328
19624	0.429	0.942	0.481	0.439	0.982	0.149	0.505	1.430	0.385
19625	0.429	0.942	0.512	0.439	0.982	0.371	0.505	1.430	0.431
19626	0.224	-0.373	0.512	0.234	-0.336	0.478	0.184	-0.625	0.224
19627	0.500	1.397	0.492	0.497	1.359	0.512	0.479	1.262	0.097
19628	0.224	-0.373	0.492	0.152	-0.863	0.235	0.187	-0.608	0.492
19629	0.382	0.638	0.492	0.512	1.453	0.514	0.511	1.464	0.501
19630	0.511	1.464	0.508	0.500	1.378	0.514	0.497	1.380	0.262
19631	0.184	-0.625	0.097	0.102	-1.184	0.514	0.413	0.841	0.262
19632	0.508	1.447	0.501	0.503	1.396	0.508	0.476	1.245	0.185
19633	0.503	1.413	0.097	0.512	1.453	0.491	0.468	1.194	0.296
19634	0.492	1.346	0.508	0.462	1.133	0.478	0.516	1.498	0.185
19635	0.492	1.346	0.508	0.462	1.133	0.235	0.516	1.498	0.512
19636	0.505	1.430	0.097	0.503	1.396	0.512	0.411	0.824	0.185
19637	0.329	0.301	0.097	0.149	-0.882	0.149	0.384	0.655	0.481
19638	0.224	-0.373	0.501	0.102	-1.184	0.422	0.261	-0.137	0.262

19639	0.482	1.279	0.224	0.465	1.152	0.508	0.450	1.076	0.262
19640	0.226	-0.356	0.262	0.237	-0.317	0.371	0.261	-0.137	0.501
19641	0.261	-0.137	0.097	0.275	-0.073	0.514	0.142	-0.895	0.328
19642	0.503	1.413	0.481	0.503	1.396	0.149	0.476	1.245	0.481
19643	0.450	1.076	0.050	0.503	1.396	0.501	0.503	1.413	0.512
19644	0.297	0.099	0.501	0.149	-0.882	0.149	0.097	-1.181	0.492
19645	0.511	1.464	0.185	0.512	1.453	0.508	0.466	1.178	0.185
19646	0.263	-0.120	0.296	0.149	-0.882	0.501	0.387	0.672	0.467
19647	0.508	1.447	0.296	0.515	1.472	0.273	0.503	1.413	0.358
19648	0.503	1.413	0.512	0.512	1.453	0.273	0.505	1.430	0.050
19649	0.487	1.312	0.508	0.512	1.453	0.273	0.508	1.447	0.492
19650	0.487	1.312	0.050	0.512	1.453	0.235	0.434	0.975	0.142
19651	0.508	1.447	0.050	0.474	1.208	0.444	0.508	1.447	0.508
19652	0.361	0.504	0.467	0.149	-0.882	0.193	0.142	-0.895	0.481
19653	0.358	0.487	0.492	0.368	0.530	0.309	0.100	-1.164	0.508
19654	0.355	0.470	0.262	0.474	1.208	0.371	0.511	1.464	0.492
19655	0.447	1.060	0.142	0.512	1.453	0.422	0.495	1.363	0.512
19656	0.187	-0.608	0.262	0.234	-0.336	0.273	0.184	-0.625	0.450
19657	0.463	1.161	0.481	0.515	1.472	0.501	0.516	1.498	0.409
19658	0.476	1.245	0.467	0.515	1.472	0.309	0.516	1.498	0.358
19659	0.476	1.245	0.224	0.515	1.472	0.478	0.516	1.498	0.481
19660	0.476	1.245	0.508	0.506	1.415	0.462	0.511	1.464	0.185
19661	0.508	1.447	0.050	0.488	1.302	0.341	0.511	1.464	0.508
19662	0.505	1.430	0.262	0.474	1.208	0.102	0.408	0.807	0.508
19663	0.187	-0.608	0.142	0.234	-0.336	0.508	0.184	-0.625	0.492
19664	0.297	0.099	0.142	0.515	1.472	0.341	0.142	-0.895	0.492
19665	0.050	-1.485	0.224	0.149	-0.882	0.341	0.432	0.958	0.467
19666	0.511	1.464	0.142	0.427	0.907	0.309	0.500	1.397	0.501
19667	0.511	1.464	0.097	0.515	1.472	0.462	0.500	1.397	0.492
19668	0.050	-1.485	0.097	0.374	0.568	0.341	0.184	-0.625	0.512
19669	0.466	1.178	0.501	0.515	1.472	0.478	0.505	1.430	0.142
19670	0.332	0.318	0.224	0.149	-0.882	0.514	0.097	-1.181	0.097
19671	0.503	1.413	0.224	0.506	1.415	0.444	0.511	1.464	0.450
19672	0.361	0.504	0.508	0.149	-0.882	0.193	0.226	-0.356	0.512
19673	0.355	0.470	0.450	0.503	1.396	0.309	0.508	1.447	0.409
19674	0.408	0.807	0.262	0.497	1.359	0.491	0.508	1.447	0.512
19675	0.050	-1.485	0.467	0.272	-0.091	0.309	0.226	-0.356	0.508
19676	0.224	-0.373	0.409	0.275	-0.073	0.501	0.050	-1.485	0.481
19677	0.389	0.689	0.296	0.275	-0.073	0.462	0.224	-0.373	0.508
19678	0.050	-1.485	0.142	0.234	-0.336	0.273	0.263	-0.120	0.513
19679	0.466	1.178	0.224	0.444	1.020	0.444	0.429	0.942	0.328
19680	0.266	-0.103	0.185	0.193	-0.600	0.508	0.097	-1.181	0.481
19681	0.261	-0.137	0.142	0.307	0.135	0.501	0.000	?	0.142
19682	0.468	1.194	0.492	0.371	0.549	0.462	0.508	1.447	0.450
19683	0.511	1.464	0.262	0.424	0.888	0.193	0.508	1.447	0.450
19684	0.097	-1.181	0.409	0.275	-0.073	0.512	0.224	-0.373	0.296
19685	0.497	1.380	0.508	0.506	1.415	0.273	0.355	0.470	0.185
19686	0.097	-1.181	0.262	0.313	0.172	0.371	0.261	-0.137	0.185
19687	0.511	1.464	0.328	0.477	1.227	0.235	0.492	1.346	0.185
19688	0.511	1.464	0.185	0.459	1.114	0.491	0.447	1.060	0.508
19689	0.295	0.082	0.492	0.345	0.379	0.512	0.358	0.487	0.185
19690	0.297	0.099	0.513	0.102	-1.184	0.512	0.142	-0.895	0.328
19691	0.482	1.279	0.512	0.503	1.396	0.491	0.429	0.942	0.224
19692	0.142	-0.895	0.431	0.342	0.361	0.491	0.142	-0.895	0.450
19693	0.050	-1.485	0.097	0.339	0.342	0.491	0.184	-0.625	0.185
19694	0.224	-0.373	0.512	0.307	0.135	0.491	0.097	-1.181	0.508
19695	0.489	1.329	0.508	0.465	1.152	0.398	0.408	0.807	0.142
19696	0.184	-0.625	0.097	0.342	0.361	0.491	0.224	-0.373	0.142
19697	0.497	1.380	0.050	0.480	1.246	0.508	0.487	1.312	0.185
19698	0.505	1.430	0.185	0.518	1.491	0.508	0.479	1.262	0.513
19699	0.482	1.279	0.358	0.447	1.039	0.273	0.503	1.413	0.501

19700	0.224	-0.373	0.185	0.193	-0.600	0.273	0.226	-0.356	0.296
19701	0.224	-0.373	0.508	0.307	0.135	0.193	0.261	-0.137	0.328
19702	0.511	1.464	0.142	0.497	1.359	0.193	0.408	0.807	0.501
19703	0.224	-0.373	0.508	0.310	0.153	0.193	0.261	-0.137	0.481
19704	0.474	1.228	0.501	0.503	1.396	0.235	0.482	1.279	0.185
19705	0.503	1.413	0.431	0.462	1.133	0.193	0.516	1.498	0.262
19706	0.263	-0.120	0.142	0.275	-0.073	0.491	0.142	-0.895	0.185
19707	0.503	1.413	0.481	0.447	1.039	0.501	0.463	1.161	0.512
19708	0.492	1.346	0.224	0.506	1.415	0.508	0.461	1.144	0.508
19709	0.511	1.464	0.097	0.503	1.396	0.508	0.500	1.397	0.358
19710	0.500	1.397	0.224	0.465	1.152	0.508	0.482	1.279	0.467
19711	0.226	-0.356	0.508	0.196	-0.581	0.508	0.142	-0.895	0.224
19712	0.489	1.329	0.508	0.512	1.453	0.273	0.445	1.043	0.097
19713	0.261	-0.137	0.224	0.272	-0.091	0.235	0.097	-1.181	0.262
19714	0.187	-0.608	0.142	0.371	0.549	0.341	0.050	-1.485	0.296
19715	0.261	-0.137	0.097	0.240	-0.299	0.235	0.097	-1.181	0.097
19716	0.445	1.043	0.431	0.488	1.302	0.478	0.513	1.481	0.296
19717	0.500	1.397	0.512	0.518	1.491	0.478	0.508	1.447	0.097
19718	0.505	1.430	0.185	0.512	1.453	0.478	0.489	1.329	0.097
19719	0.503	1.413	0.467	0.494	1.340	0.478	0.468	1.194	0.450
19720	0.503	1.413	0.142	0.494	1.340	0.371	0.468	1.194	0.262
19721	0.503	1.413	0.185	0.494	1.340	0.149	0.468	1.194	0.224
19722	0.503	1.413	0.501	0.494	1.340	0.235	0.503	1.413	0.328
19723	0.050	-1.485	0.142	0.398	0.718	0.273	0.050	-1.485	0.508
19724	0.492	1.346	0.467	0.488	1.302	0.478	0.505	1.430	0.185
19725	0.495	1.363	0.142	0.506	1.415	0.478	0.503	1.413	0.501
19726	0.495	1.363	0.262	0.506	1.415	0.478	0.503	1.413	0.431
19727	0.184	-0.625	0.224	0.272	-0.091	0.478	0.097	-1.181	0.142
19728	0.226	-0.356	0.481	0.275	-0.073	0.478	0.261	-0.137	0.097
19729	0.295	0.082	0.467	0.193	-0.600	0.491	0.334	0.335	0.358
19730	0.184	-0.625	0.508	0.196	-0.581	0.273	0.358	0.487	0.262
19731	0.184	-0.625	0.508	0.196	-0.581	0.371	0.358	0.487	0.296
19732	0.263	-0.120	0.097	0.234	-0.336	0.501	0.358	0.487	0.512
19733	0.226	-0.356	0.262	0.193	-0.600	0.514	0.297	0.099	0.185
19734	0.500	1.397	0.142	0.494	1.340	0.514	0.492	1.346	0.450
19735	0.492	1.346	0.142	0.503	1.396	0.491	0.516	1.498	0.450
19736	0.516	1.498	0.409	0.512	1.453	0.512	0.508	1.447	0.142
19737	0.516	1.498	0.328	0.512	1.453	0.501	0.508	1.447	0.224
19738	0.516	1.498	0.185	0.512	1.453	0.512	0.508	1.447	0.142
19739	0.516	1.498	0.512	0.512	1.453	0.341	0.508	1.447	0.508
19740	0.226	-0.356	0.050	0.275	-0.073	0.341	0.226	-0.356	0.467
19741	0.184	-0.625	0.142	0.234	-0.336	0.491	0.142	-0.895	0.481
19742	0.187	-0.608	0.185	0.345	0.379	0.149	0.295	0.082	0.501
19743	0.000	?	0.185	0.234	-0.336	0.512	0.332	0.318	0.224
19744	0.476	1.245	0.492	0.482	1.265	0.512	0.487	1.312	0.409
19745	0.476	1.245	0.501	0.482	1.265	0.193	0.487	1.312	0.512
19746	0.476	1.245	0.142	0.482	1.265	0.309	0.487	1.312	0.224
19747	0.476	1.245	0.512	0.482	1.265	0.102	0.487	1.312	0.262
19748	0.447	1.060	0.224	0.374	0.568	0.235	0.500	1.397	0.262
19749	0.261	-0.137	0.501	0.149	-0.882	0.508	0.389	0.689	0.328
19750	0.261	-0.137	0.467	0.234	-0.336	0.102	0.097	-1.181	0.185
19751	0.300	0.116	0.409	0.272	-0.091	0.398	0.050	-1.485	0.328
19752	0.447	1.060	0.328	0.480	1.246	0.501	0.505	1.430	0.185
19753	0.447	1.060	0.481	0.480	1.246	0.371	0.497	1.380	0.467
19754	0.447	1.060	0.142	0.480	1.246	0.235	0.505	1.430	0.409
19755	0.447	1.060	0.512	0.480	1.246	0.235	0.505	1.430	0.513
19756	0.447	1.060	0.431	0.480	1.246	0.478	0.505	1.430	0.328
19757	0.476	1.245	0.409	0.488	1.302	0.491	0.355	0.470	0.508
19758	0.226	-0.356	0.508	0.272	-0.091	0.193	0.050	-1.485	0.224
19759	0.050	-1.485	0.050	0.371	0.549	0.273	0.224	-0.373	0.409
19760	0.500	1.397	0.358	0.503	1.396	0.501	0.453	1.093	0.328

19761	0.497	1.380	0.492	0.518	1.491	0.512	0.492	1.346	0.508
19762	0.497	1.380	0.501	0.518	1.491	0.501	0.492	1.346	0.508
19763	0.497	1.380	0.508	0.494	1.340	0.235	0.505	1.430	0.224
19764	0.503	1.413	0.513	0.515	1.472	0.235	0.489	1.329	0.224
19765	0.468	1.194	0.508	0.503	1.396	0.478	0.487	1.312	0.262
19766	0.479	1.262	0.328	0.506	1.415	0.478	0.505	1.430	0.450
19767	0.500	1.397	0.224	0.342	0.361	0.309	0.511	1.464	0.481
19768	0.000	?	0.512	0.345	0.379	0.462	0.261	-0.137	0.185
19769	0.497	1.380	0.262	0.497	1.359	0.462	0.497	1.380	0.512
19770	0.184	-0.625	0.262	0.149	-0.882	0.341	0.263	-0.120	0.142
19771	0.487	1.312	0.501	0.512	1.453	0.501	0.432	0.958	0.142
19772	0.487	1.312	0.450	0.512	1.453	0.491	0.411	0.824	0.431
19773	0.184	-0.625	0.501	0.193	-0.600	0.478	0.184	-0.625	0.097
19774	0.184	-0.625	0.481	0.310	0.153	0.512	0.226	-0.356	0.508
19775	0.263	-0.120	0.142	0.102	-1.184	0.149	0.226	-0.356	0.142
19776	0.295	0.082	0.296	0.237	-0.317	0.512	0.097	-1.181	0.262
19777	0.429	0.942	0.501	0.509	1.434	0.149	0.492	1.346	0.262
19778	0.145	-0.878	0.467	0.102	-1.184	0.501	0.326	0.285	0.512
19779	0.263	-0.120	0.185	0.398	0.718	0.501	0.050	-1.485	0.185
19780	0.505	1.430	0.296	0.500	1.378	0.462	0.513	1.481	0.492
19781	0.142	-0.895	0.358	0.371	0.549	0.309	0.184	-0.625	0.492
19782	0.142	-0.895	0.512	0.237	-0.317	0.514	0.184	-0.625	0.142
19783	0.226	-0.356	0.501	0.234	-0.336	0.478	0.261	-0.137	0.097
19784	0.492	1.346	0.431	0.480	1.246	0.491	0.508	1.447	0.224
19785	0.492	1.346	0.431	0.494	1.340	0.235	0.508	1.447	0.224
19786	0.263	-0.120	0.431	0.196	-0.581	0.102	0.184	-0.625	0.512
19787	0.097	-1.181	0.431	0.272	-0.091	0.512	0.261	-0.137	0.224
19788	0.497	1.380	0.328	0.503	1.396	0.193	0.513	1.481	0.185
19789	0.495	1.363	0.508	0.509	1.434	0.462	0.513	1.481	0.467
19790	0.495	1.363	0.501	0.500	1.378	0.478	0.503	1.413	0.262
19791	0.329	0.301	0.142	0.234	-0.336	0.478	0.332	0.318	0.224
19792	0.329	0.301	0.142	0.234	-0.336	0.053	0.332	0.318	0.328
19793	0.497	1.380	0.097	0.477	1.227	0.273	0.497	1.380	0.142
19794	0.497	1.380	0.296	0.477	1.227	0.102	0.489	1.329	0.501
19795	0.000	?	0.358	0.307	0.135	0.273	0.300	0.116	0.512
19796	0.461	1.144	0.358	0.462	1.133	0.235	0.505	1.430	0.358
19797	0.461	1.144	0.358	0.462	1.133	0.508	0.505	1.430	0.481
19798	0.295	0.082	0.358	0.342	0.361	0.193	0.142	-0.895	0.358
19799	0.505	1.430	0.358	0.497	1.359	0.491	0.503	1.413	0.508
19800	0.447	1.060	0.501	0.491	1.321	0.235	0.468	1.194	0.492
19801	0.497	1.380	0.097	0.480	1.246	0.235	0.447	1.060	0.501
19802	0.429	0.942	0.296	0.518	1.491	0.422	0.482	1.279	0.185
19803	0.332	0.318	0.142	0.149	-0.882	0.235	0.097	-1.181	0.501
19804	0.495	1.363	0.481	0.515	1.472	0.478	0.445	1.043	0.142
19805	0.145	-0.878	0.431	0.149	-0.882	0.512	0.355	0.470	0.431
19806	0.500	1.397	0.262	0.503	1.396	0.235	0.432	0.958	0.481
19807	0.445	1.043	0.262	0.497	1.359	0.508	0.516	1.498	0.481
19808	0.484	1.295	0.481	0.462	1.133	0.273	0.492	1.346	0.467
19809	0.100	-1.164	0.467	0.310	0.153	0.478	0.142	-0.895	0.358
19810	0.461	1.144	0.492	0.512	1.453	0.341	0.358	0.487	0.385
19811	0.497	1.380	0.262	0.477	1.227	0.371	0.447	1.060	0.513
19812	0.479	1.262	0.328	0.494	1.340	0.341	0.479	1.262	0.512
19813	0.142	-0.895	0.513	0.237	-0.317	0.341	0.329	0.301	0.508
19814	0.184	-0.625	0.358	0.105	-1.165	0.491	0.384	0.655	0.481
19815	0.516	1.498	0.328	0.509	1.434	0.508	0.429	0.942	0.513
19816	0.224	-0.373	0.385	0.193	-0.600	0.508	0.226	-0.356	0.185
19817	0.187	-0.608	0.512	0.459	1.114	0.491	0.097	-1.181	0.224
19818	0.466	1.178	0.328	0.482	1.265	0.478	0.495	1.363	0.492
19819	0.358	0.487	0.050	0.480	1.246	0.512	0.503	1.413	0.328
19820	0.297	0.099	0.097	0.053	-1.504	0.512	0.263	-0.120	0.224
19821	0.187	-0.608	0.296	0.278	-0.054	0.235	0.261	-0.137	0.467

19822	0.295	0.082	0.481	0.102	-1.184	0.512	0.184	-0.625	0.492
19823	0.187	-0.608	0.262	0.278	-0.054	0.273	0.295	0.082	0.450
19824	0.142	-0.895	0.501	0.237	-0.317	0.501	0.184	-0.625	0.512
19825	0.497	1.380	0.512	0.506	1.415	0.508	0.508	1.447	0.224
19826	0.224	-0.373	0.431	0.196	-0.581	0.508	0.184	-0.625	0.142
19827	0.479	1.262	0.450	0.491	1.321	0.501	0.476	1.245	0.501
19828	0.300	0.116	0.492	0.234	-0.336	0.501	0.263	-0.120	0.262
19829	0.300	0.116	0.185	0.234	-0.336	0.501	0.263	-0.120	0.358
19830	0.447	1.060	0.185	0.427	0.907	0.514	0.505	1.430	0.142
19831	0.297	0.099	0.431	0.234	-0.336	0.501	0.332	0.318	0.185
19832	0.482	1.279	0.142	0.482	1.265	0.341	0.482	1.279	0.508
19833	0.384	0.655	0.097	0.509	1.434	0.371	0.508	1.447	0.501
19834	0.297	0.099	0.492	0.234	-0.336	0.491	0.000	?	0.512
19835	0.511	1.464	0.492	0.509	1.434	0.478	0.495	1.363	0.512
19836	0.224	-0.373	0.512	0.275	-0.073	0.508	0.184	-0.625	0.512
19837	0.503	1.413	0.501	0.474	1.208	0.398	0.466	1.178	0.512
19838	0.389	0.689	0.142	0.342	0.361	0.514	0.361	0.504	0.358
19839	0.266	-0.103	0.224	0.371	0.549	0.514	0.050	-1.485	0.492
19840	0.503	1.413	0.224	0.342	0.361	0.491	0.489	1.329	0.501
19841	0.184	-0.625	0.224	0.342	0.361	0.491	0.142	-0.895	0.296
19842	0.384	0.655	0.142	0.488	1.302	0.149	0.508	1.447	0.296
19843	0.484	1.295	0.142	0.512	1.453	0.501	0.482	1.279	0.296
19844	0.487	1.312	0.513	0.509	1.434	0.341	0.505	1.430	0.224
19845	0.484	1.295	0.512	0.488	1.302	0.508	0.489	1.329	0.185
19846	0.511	1.464	0.481	0.474	1.208	0.501	0.508	1.447	0.185
19847	0.505	1.430	0.296	0.512	1.453	0.273	0.450	1.076	0.185
19848	0.508	1.447	0.262	0.509	1.434	0.273	0.405	0.790	0.185
19849	0.361	0.504	0.224	0.234	-0.336	0.341	0.358	0.487	0.142
19850	0.511	1.464	0.224	0.509	1.434	0.341	0.476	1.245	0.512
19851	0.097	-1.181	0.262	0.275	-0.073	0.444	0.184	-0.625	0.185
19852	0.508	1.447	0.492	0.503	1.396	0.273	0.505	1.430	0.097
19853	0.466	1.178	0.097	0.506	1.415	0.102	0.505	1.430	0.262
19854	0.466	1.178	0.142	0.506	1.415	0.398	0.489	1.329	0.492
19855	0.466	1.178	0.501	0.497	1.359	0.501	0.489	1.329	0.492
19856	0.479	1.262	0.508	0.497	1.359	0.422	0.463	1.161	0.185
19857	0.508	1.447	0.508	0.503	1.396	0.478	0.495	1.363	0.185
19858	0.445	1.043	0.450	0.512	1.453	0.235	0.489	1.329	0.328
19859	0.492	1.346	0.262	0.506	1.415	0.149	0.511	1.464	0.508
19860	0.050	-1.485	0.431	0.345	0.379	0.371	0.142	-0.895	0.501
19861	0.187	-0.608	0.358	0.371	0.549	0.512	0.097	-1.181	0.358
19862	0.482	1.279	0.513	0.488	1.302	0.512	0.447	1.060	0.185
19863	0.474	1.228	0.481	0.480	1.246	0.341	0.516	1.498	0.450
19864	0.503	1.413	0.508	0.512	1.453	0.341	0.405	0.790	0.224
19865	0.503	1.413	0.431	0.398	0.718	0.514	0.511	1.464	0.513
19866	0.484	1.295	0.481	0.518	1.491	0.512	0.505	1.430	0.328
19867	0.484	1.295	0.262	0.518	1.491	0.193	0.479	1.262	0.409
19868	0.487	1.312	0.224	0.491	1.321	0.149	0.516	1.498	0.328
19869	0.503	1.413	0.385	0.491	1.321	0.235	0.521	1.531	0.142
19870	0.097	-1.181	0.501	0.149	-0.882	0.508	0.332	0.318	0.512
19871	0.492	1.346	0.450	0.503	1.396	0.491	0.479	1.262	0.296
19872	0.097	-1.181	0.142	0.342	0.361	0.235	0.142	-0.895	0.508
19873	0.500	1.397	0.262	0.512	1.453	0.501	0.447	1.060	0.358
19874	0.500	1.397	0.185	0.506	1.415	0.422	0.447	1.060	0.492
19875	0.097	-1.181	0.431	0.275	-0.073	0.273	0.297	0.099	0.328
19876	0.097	-1.181	0.358	0.275	-0.073	0.273	0.187	-0.608	0.481
19877	0.000	?	0.481	0.345	0.379	0.273	0.184	-0.625	0.501
19878	0.000	?	0.508	0.345	0.379	0.235	0.184	-0.625	0.512
19879	0.492	1.346	0.097	0.444	1.020	0.273	0.508	1.447	0.097
19880	0.224	-0.373	0.513	0.275	-0.073	0.371	0.184	-0.625	0.097
19881	0.261	-0.137	0.431	0.102	-1.184	0.371	0.329	0.301	0.481
19882	0.097	-1.181	0.501	0.395	0.700	0.444	0.224	-0.373	0.328

19883	0.476	1.245	0.185	0.503	1.396	0.235	0.450	1.076	0.262
19884	0.050	-1.485	0.224	0.421	0.869	0.235	0.184	-0.625	0.467
19885	0.497	1.380	0.328	0.474	1.208	0.444	0.505	1.430	0.467
19886	0.184	-0.625	0.328	0.234	-0.336	0.462	0.142	-0.895	0.481
19887	0.300	0.116	0.513	0.149	-0.882	0.309	0.142	-0.895	0.492
19888	0.295	0.082	0.512	0.374	0.568	0.508	0.184	-0.625	0.185
19889	0.503	1.413	0.512	0.509	1.434	0.512	0.518	1.514	0.224
19890	0.500	1.397	0.296	0.509	1.434	0.235	0.508	1.447	0.262
19891	0.050	-1.485	0.467	0.348	0.398	0.309	0.295	0.082	0.262
19892	0.050	-1.485	0.492	0.348	0.398	0.053	0.326	0.285	0.262
19893	0.466	1.178	0.185	0.512	1.453	0.273	0.497	1.380	0.296
19894	0.489	1.329	0.450	0.512	1.453	0.149	0.479	1.262	0.508
19895	0.261	-0.137	0.508	0.193	-0.600	0.501	0.184	-0.625	0.512
19896	0.142	-0.895	0.508	0.149	-0.882	0.309	0.261	-0.137	0.501
19897	0.261	-0.137	0.185	0.237	-0.317	0.501	0.184	-0.625	0.185
19898	0.482	1.279	0.501	0.503	1.396	0.398	0.513	1.481	0.262
19899	0.466	1.178	0.512	0.488	1.302	0.235	0.505	1.430	0.409
19900	0.224	-0.373	0.513	0.240	-0.299	0.235	0.358	0.487	0.142
19901	0.497	1.380	0.467	0.506	1.415	0.273	0.468	1.194	0.097
19902	0.050	-1.485	0.409	0.424	0.888	0.478	0.226	-0.356	0.481
19903	0.263	-0.120	0.409	0.272	-0.091	0.508	0.097	-1.181	0.358
19904	0.142	-0.895	0.224	0.278	-0.054	0.508	0.261	-0.137	0.224
19905	0.142	-0.895	0.450	0.278	-0.054	0.478	0.295	0.082	0.508
19906	0.226	-0.356	0.409	0.234	-0.336	0.273	0.097	-1.181	0.512
19907	0.142	-0.895	0.296	0.278	-0.054	0.501	0.295	0.082	0.450
19908	0.097	-1.181	0.450	0.371	0.549	0.501	0.097	-1.181	0.512
19909	0.097	-1.181	0.224	0.368	0.530	0.371	0.097	-1.181	0.050
19910	0.495	1.363	0.050	0.439	0.982	0.309	0.453	1.093	0.513
19911	0.226	-0.356	0.224	0.234	-0.336	0.273	0.263	-0.120	0.142
19912	0.226	-0.356	0.185	0.234	-0.336	0.341	0.226	-0.356	0.501
19913	0.511	1.464	0.409	0.444	1.020	0.309	0.326	0.285	0.512
19914	0.453	1.093	0.224	0.468	1.170	0.444	0.508	1.447	0.501
19915	0.263	-0.120	0.481	0.310	0.153	0.235	0.184	-0.625	0.501
19916	0.463	1.161	0.501	0.506	1.415	0.478	0.500	1.397	0.501
19917	0.408	0.807	0.142	0.509	1.434	0.514	0.429	0.942	0.185
19918	0.297	0.099	0.385	0.237	-0.317	0.512	0.142	-0.895	0.328
19919	0.142	-0.895	0.097	0.313	0.172	0.102	0.097	-1.181	0.185
19920	0.224	-0.373	0.501	0.053	-1.504	0.512	0.361	0.504	0.431
19921	0.184	-0.625	0.097	0.275	-0.073	0.149	0.261	-0.137	0.508
19922	0.142	-0.895	0.262	0.152	-0.863	0.193	0.297	0.099	0.328
19923	0.484	1.295	0.328	0.506	1.415	0.053	0.511	1.464	0.185
19924	0.263	-0.120	0.492	0.310	0.153	0.501	0.184	-0.625	0.224
19925	0.411	0.824	0.185	0.500	1.378	0.501	0.450	1.076	0.512
19926	0.503	1.413	0.185	0.395	0.700	0.309	0.450	1.076	0.512
19927	0.261	-0.137	0.481	0.237	-0.317	0.193	0.142	-0.895	0.467
19928	0.329	0.301	0.492	0.234	-0.336	0.273	0.224	-0.373	0.481
19929	0.184	-0.625	0.296	0.278	-0.054	0.478	0.142	-0.895	0.296
19930	0.492	1.346	0.358	0.471	1.189	0.501	0.508	1.447	0.481
19931	0.513	1.481	0.262	0.503	1.396	0.273	0.468	1.194	0.508
19932	0.511	1.464	0.224	0.503	1.396	0.512	0.482	1.279	0.481
19933	0.434	0.975	0.185	0.480	1.246	0.273	0.505	1.430	0.385
19934	0.100	-1.164	0.328	0.272	-0.091	0.273	0.226	-0.356	0.385
19935	0.513	1.481	0.508	0.500	1.378	0.491	0.411	0.824	0.385
19936	0.508	1.447	0.185	0.497	1.359	0.398	0.513	1.481	0.262
19937	0.097	-1.181	0.185	0.368	0.530	0.512	0.224	-0.373	0.512
19938	0.050	-1.485	0.501	0.310	0.153	0.273	0.261	-0.137	0.512
19939	0.184	-0.625	0.481	0.275	-0.073	0.398	0.261	-0.137	0.512
19940	0.361	0.504	0.224	0.342	0.361	0.371	0.326	0.285	0.431
19941	0.184	-0.625	0.409	0.307	0.135	0.501	0.184	-0.625	0.501
19942	0.508	1.447	0.481	0.444	1.020	0.341	0.329	0.301	0.492
19943	0.145	-0.878	0.185	0.237	-0.317	0.508	0.184	-0.625	0.142

19944	0.505	1.430	0.185	0.480	1.246	0.508	0.461	1.144	0.512
19945	0.500	1.397	0.185	0.518	1.491	0.398	0.408	0.807	0.501
19946	0.432	0.958	0.508	0.512	1.453	0.341	0.503	1.413	0.501
19947	0.142	-0.895	0.501	0.102	-1.184	0.341	0.329	0.301	0.142
19948	0.482	1.279	0.501	0.512	1.453	0.341	0.497	1.380	0.481
19949	0.226	-0.356	0.142	0.149	-0.882	0.512	0.224	-0.373	0.450
19950	0.097	-1.181	0.508	0.193	-0.600	0.371	0.408	0.807	0.513
19951	0.224	-0.373	0.050	0.053	-1.504	0.235	0.332	0.318	0.492
19952	0.508	1.447	0.467	0.497	1.359	0.491	0.508	1.447	0.492
19953	0.508	1.447	0.431	0.497	1.359	0.273	0.508	1.447	0.492
19954	0.226	-0.356	0.262	0.310	0.153	0.273	0.224	-0.373	0.050
19955	0.142	-0.895	0.262	0.193	-0.600	0.193	0.226	-0.356	0.513
19956	0.097	-1.181	0.467	0.272	-0.091	0.235	0.266	-0.103	0.512
19957	0.429	0.942	0.185	0.482	1.265	0.491	0.450	1.076	0.142
19958	0.513	1.481	0.050	0.506	1.415	0.501	0.476	1.245	0.508
19959	0.184	-0.625	0.262	0.275	-0.073	0.235	0.184	-0.625	0.142
19960	0.466	1.178	0.296	0.509	1.434	0.508	0.513	1.481	0.224
19961	0.145	-0.878	0.296	0.272	-0.091	0.398	0.142	-0.895	0.097
19962	0.187	-0.608	0.296	0.272	-0.091	0.501	0.142	-0.895	0.142
19963	0.497	1.380	0.296	0.488	1.302	0.462	0.432	0.958	0.508
19964	0.142	-0.895	0.224	0.398	0.718	0.444	0.097	-1.181	0.097
19965	0.468	1.194	0.431	0.509	1.434	0.341	0.511	1.464	0.501
19966	0.145	-0.878	0.142	0.272	-0.091	0.501	0.142	-0.895	0.513
19967	0.261	-0.137	0.508	0.398	0.718	0.341	0.263	-0.120	0.185
19968	0.224	-0.373	0.450	0.371	0.549	0.478	0.263	-0.120	0.508
19969	0.476	1.245	0.431	0.500	1.378	0.512	0.508	1.447	0.262
19970	0.468	1.194	0.262	0.339	0.342	0.512	0.184	-0.625	0.358
19971	0.500	1.397	0.501	0.509	1.434	0.371	0.497	1.380	0.224
19972	0.500	1.397	0.142	0.509	1.434	0.235	0.497	1.380	0.224
19973	0.097	-1.181	0.508	0.401	0.737	0.273	0.142	-0.895	0.296
19974	0.263	-0.120	0.097	0.339	0.342	0.514	0.097	-1.181	0.508
19975	0.142	-0.895	0.185	0.345	0.379	0.478	0.224	-0.373	0.142
19976	0.142	-0.895	0.385	0.345	0.379	0.512	0.224	-0.373	0.224
19977	0.411	0.824	0.185	0.515	1.472	0.508	0.511	1.464	0.492
19978	0.326	0.285	0.481	0.371	0.549	0.462	0.224	-0.373	0.492
19979	0.184	-0.625	0.481	0.237	-0.317	0.309	0.184	-0.625	0.050
19980	0.511	1.464	0.224	0.491	1.321	0.235	0.468	1.194	0.142
19981	0.050	-1.485	0.328	0.275	-0.073	0.501	0.263	-0.120	0.185
19982	0.142	-0.895	0.512	0.278	-0.054	0.149	0.224	-0.373	0.224
19983	0.187	-0.608	0.142	0.193	-0.600	0.193	0.326	0.285	0.097
19984	0.187	-0.608	0.512	0.237	-0.317	0.478	0.142	-0.895	0.328
19985	0.492	1.346	0.508	0.491	1.321	0.491	0.500	1.397	0.501
19986	0.500	1.397	0.508	0.506	1.415	0.512	0.513	1.481	0.262
19987	0.142	-0.895	0.481	0.237	-0.317	0.491	0.358	0.487	0.142
19988	0.511	1.464	0.508	0.506	1.415	0.193	0.479	1.262	0.501
19989	0.224	-0.373	0.508	0.406	0.775	0.273	0.355	0.470	0.467
19990	0.497	1.380	0.508	0.500	1.378	0.444	0.505	1.430	0.097
19991	0.466	1.178	0.481	0.465	1.152	0.273	0.489	1.329	0.501
19992	0.408	0.807	0.450	0.447	1.039	0.149	0.503	1.413	0.508
19993	0.329	0.301	0.512	0.342	0.361	0.102	0.187	-0.608	0.224
19994	0.474	1.228	0.508	0.506	1.415	0.149	0.495	1.363	0.224
19995	0.142	-0.895	0.467	0.345	0.379	0.478	0.142	-0.895	0.224
19996	0.500	1.397	0.050	0.477	1.227	0.478	0.434	0.975	0.512
19997	0.429	0.942	0.358	0.509	1.434	0.462	0.479	1.262	0.492
19998	0.408	0.807	0.358	0.509	1.434	0.491	0.479	1.262	0.513
19999	0.511	1.464	0.508	0.374	0.568	0.491	0.466	1.178	0.224
20000	0.050	-1.485	0.296	0.234	-0.336	0.491	0.358	0.487	0.508
20001	0.361	0.504	0.296	0.275	-0.073	0.235	0.384	0.655	0.296
20002	0.495	1.363	0.358	0.518	1.491	0.478	0.516	1.498	0.512
20003	0.508	1.447	0.358	0.480	1.246	0.478	0.505	1.430	0.492
20004	0.497	1.380	0.467	0.518	1.491	0.341	0.511	1.464	0.142

20005	0.503	1.413	0.358	0.512	1.453	0.341	0.482	1.279	0.142
20006	0.497	1.380	0.185	0.465	1.152	0.235	0.516	1.498	0.492
20007	0.329	0.301	0.185	0.307	0.135	0.371	0.184	-0.625	0.296
20008	0.224	-0.373	0.512	0.237	-0.317	0.193	0.224	-0.373	0.296
20009	0.508	1.447	0.508	0.503	1.396	0.193	0.495	1.363	0.262
20010	0.263	-0.120	0.481	0.152	-0.863	0.193	0.329	0.301	0.185
20011	0.261	-0.137	0.501	0.196	-0.581	0.193	0.224	-0.373	0.358
20012	0.503	1.413	0.262	0.482	1.265	0.193	0.463	1.161	0.358
20013	0.447	1.060	0.467	0.491	1.321	0.478	0.492	1.346	0.358
20014	0.500	1.397	0.481	0.518	1.491	0.273	0.450	1.076	0.142
20015	0.476	1.245	0.501	0.485	1.283	0.193	0.511	1.464	0.492
20016	0.142	-0.895	0.296	0.193	-0.600	0.193	0.224	-0.373	0.142
20017	0.300	0.116	0.481	0.275	-0.073	0.478	0.142	-0.895	0.481
20018	0.505	1.430	0.450	0.444	1.020	0.478	0.503	1.413	0.512
20019	0.471	1.211	0.142	0.272	-0.091	0.102	0.261	-0.137	0.501
20020	0.187	-0.608	0.467	0.152	-0.863	0.102	0.355	0.470	0.185
20021	0.300	0.116	0.142	0.102	-1.184	0.478	0.142	-0.895	0.481
20022	0.361	0.504	0.512	0.152	-0.863	0.501	0.184	-0.625	0.097
20023	0.511	1.464	0.512	0.474	1.208	0.478	0.503	1.413	0.450
20024	0.492	1.346	0.097	0.485	1.283	0.193	0.497	1.380	0.224
20025	0.429	0.942	0.501	0.462	1.133	0.273	0.505	1.430	0.262
20026	0.429	0.942	0.185	0.491	1.321	0.491	0.505	1.430	0.467
20027	0.429	0.942	0.185	0.491	1.321	0.273	0.505	1.430	0.358
20028	0.429	0.942	0.185	0.491	1.321	0.193	0.505	1.430	0.508
20029	0.329	0.301	0.508	0.234	-0.336	0.444	0.355	0.470	0.492
20030	0.497	1.380	0.185	0.482	1.265	0.512	0.492	1.346	0.142
20031	0.495	1.363	0.262	0.480	1.246	0.235	0.497	1.380	0.262
20032	0.142	-0.895	0.185	0.342	0.361	0.102	0.295	0.082	0.467
20033	0.142	-0.895	0.513	0.342	0.361	0.235	0.295	0.082	0.185
20034	0.097	-1.181	0.296	0.234	-0.336	0.309	0.295	0.082	0.467
20035	0.297	0.099	0.481	0.368	0.530	0.371	0.224	-0.373	0.450
20036	0.361	0.504	0.224	0.193	-0.600	0.149	0.184	-0.625	0.450
20037	0.361	0.504	0.097	0.193	-0.600	0.371	0.184	-0.625	0.508
20038	0.361	0.504	0.142	0.193	-0.600	0.371	0.184	-0.625	0.512
20039	0.361	0.504	0.501	0.193	-0.600	0.512	0.184	-0.625	0.512
20040	0.361	0.504	0.296	0.193	-0.600	0.235	0.142	-0.895	0.481
20041	0.503	1.413	0.142	0.480	1.246	0.512	0.511	1.464	0.492
20042	0.097	-1.181	0.262	0.272	-0.091	0.514	0.184	-0.625	0.492
20043	0.297	0.099	0.512	0.193	-0.600	0.512	0.097	-1.181	0.508
20044	0.142	-0.895	0.508	0.193	-0.600	0.273	0.263	-0.120	0.501
20045	0.474	1.228	0.492	0.480	1.246	0.273	0.489	1.329	0.492
20046	0.432	0.958	0.492	0.477	1.227	0.501	0.487	1.312	0.262
20047	0.261	-0.137	0.262	0.102	-1.184	0.053	0.184	-0.625	0.262
20048	0.261	-0.137	0.262	0.102	-1.184	0.341	0.184	-0.625	0.262
20049	0.482	1.279	0.262	0.480	1.246	0.478	0.326	0.285	0.450
20050	0.468	1.194	0.358	0.506	1.415	0.478	0.503	1.413	0.142
20051	0.489	1.329	0.185	0.482	1.265	0.512	0.497	1.380	0.142
20052	0.261	-0.137	0.501	0.193	-0.600	0.501	0.363	0.520	0.185
20053	0.329	0.301	0.050	0.275	-0.073	0.309	0.184	-0.625	0.142
20054	0.511	1.464	0.501	0.488	1.302	0.193	0.445	1.043	0.512
20055	0.361	0.504	0.328	0.272	-0.091	0.235	0.224	-0.373	0.224
20056	0.332	0.318	0.450	0.193	-0.600	0.235	0.000	?	0.296
20057	0.382	0.638	0.185	0.444	1.020	0.193	0.518	1.514	0.296
20058	0.513	1.481	0.142	0.515	1.472	0.235	0.329	0.301	0.467
20059	0.332	0.318	0.492	0.234	-0.336	0.478	0.000	?	0.501
20060	0.050	-1.485	0.328	0.102	-1.184	0.478	0.411	0.824	0.481
20061	0.097	-1.181	0.262	0.234	-0.336	0.512	0.326	0.285	0.467
20062	0.295	0.082	0.296	0.307	0.135	0.193	0.142	-0.895	0.185
20063	0.484	1.295	0.185	0.371	0.549	0.273	0.508	1.447	0.385
20064	0.266	-0.103	0.512	0.149	-0.882	0.309	0.295	0.082	0.508
20065	0.497	1.380	0.224	0.371	0.549	0.193	0.503	1.413	0.508

20066	0.000	?	0.262	0.371	0.549	0.235	0.361	0.504	0.409
20067	0.505	1.430	0.431	0.515	1.472	0.491	0.495	1.363	0.512
20068	0.000	?	0.431	0.237	-0.317	0.149	0.329	0.301	0.501
20069	0.432	0.958	0.513	0.515	1.472	0.193	0.482	1.279	0.296
20070	0.450	1.076	0.513	0.515	1.472	0.491	0.503	1.413	0.513
20071	0.495	1.363	0.508	0.509	1.434	0.341	0.503	1.413	0.328
20072	0.184	-0.625	0.508	0.275	-0.073	0.508	0.097	-1.181	0.467
20073	0.184	-0.625	0.481	0.275	-0.073	0.514	0.097	-1.181	0.467
20074	0.434	0.975	0.296	0.500	1.378	0.273	0.474	1.228	0.296
20075	0.142	-0.895	0.185	0.053	-1.504	0.508	0.332	0.318	0.492
20076	0.097	-1.181	0.492	0.342	0.361	0.371	0.261	-0.137	0.142
20077	0.495	1.363	0.501	0.480	1.246	0.512	0.468	1.194	0.185
20078	0.495	1.363	0.358	0.480	1.246	0.462	0.468	1.194	0.224
20079	0.513	1.481	0.501	0.512	1.453	0.491	0.482	1.279	0.501
20080	0.495	1.363	0.385	0.500	1.378	0.462	0.495	1.363	0.328
20081	0.142	-0.895	0.467	0.310	0.153	0.508	0.187	-0.608	0.142
20082	0.226	-0.356	0.501	0.196	-0.581	0.235	0.224	-0.373	0.224
20083	0.224	-0.373	0.431	0.237	-0.317	0.102	0.261	-0.137	0.450
20084	0.224	-0.373	0.262	0.237	-0.317	0.235	0.261	-0.137	0.050
20085	0.142	-0.895	0.328	0.196	-0.581	0.478	0.263	-0.120	0.508
20086	0.142	-0.895	0.431	0.237	-0.317	0.478	0.297	0.099	0.097
20087	0.511	1.464	0.467	0.477	1.227	0.053	0.503	1.413	0.296
20088	0.511	1.464	0.513	0.477	1.227	0.193	0.511	1.464	0.262
20089	0.479	1.262	0.512	0.509	1.434	0.149	0.500	1.397	0.481
20090	0.297	0.099	0.513	0.193	-0.600	0.512	0.187	-0.608	0.185
20091	0.263	-0.120	0.185	0.278	-0.054	0.491	0.261	-0.137	0.142
20092	0.226	-0.356	0.481	0.307	0.135	0.444	0.405	0.790	0.328
20093	0.226	-0.356	0.050	0.193	-0.600	0.491	0.145	-0.878	0.512
20094	0.263	-0.120	0.050	0.237	-0.317	0.273	0.097	-1.181	0.501
20095	0.492	1.346	0.508	0.488	1.302	0.491	0.474	1.228	0.508
20096	0.097	-1.181	0.481	0.149	-0.882	0.491	0.361	0.504	0.467
20097	0.145	-0.878	0.185	0.193	-0.600	0.512	0.226	-0.356	0.224
20098	0.500	1.397	0.481	0.497	1.359	0.102	0.513	1.481	0.224
20099	0.508	1.447	0.492	0.342	0.361	0.193	0.505	1.430	0.224
20100	0.508	1.447	0.328	0.509	1.434	0.273	0.445	1.043	0.512
20101	0.447	1.060	0.262	0.512	1.453	0.193	0.505	1.430	0.185
20102	0.261	-0.137	0.501	0.275	-0.073	0.491	0.050	-1.485	0.508
20103	0.429	0.942	0.492	0.506	1.415	0.501	0.508	1.447	0.050
20104	0.355	0.470	0.512	0.371	0.549	0.514	0.142	-0.895	0.512
20105	0.511	1.464	0.328	0.512	1.453	0.235	0.497	1.380	0.409
20106	0.476	1.245	0.224	0.462	1.133	0.422	0.508	1.447	0.513
20107	0.508	1.447	0.492	0.491	1.321	0.512	0.495	1.363	0.224
20108	0.432	0.958	0.185	0.465	1.152	0.273	0.495	1.363	0.142
20109	0.479	1.262	0.296	0.509	1.434	0.491	0.495	1.363	0.467
20110	0.263	-0.120	0.508	0.234	-0.336	0.512	0.184	-0.625	0.185
20111	0.224	-0.373	0.050	0.102	-1.184	0.512	0.326	0.285	0.097
20112	0.384	0.655	0.450	0.234	-0.336	0.273	0.184	-0.625	0.050
20113	0.503	1.413	0.262	0.480	1.246	0.508	0.432	0.958	0.185
20114	0.445	1.043	0.185	0.482	1.265	0.508	0.503	1.413	0.501
20115	0.142	-0.895	0.508	0.053	-1.504	0.508	0.329	0.301	0.328
20116	0.263	-0.120	0.262	0.193	-0.600	0.501	0.184	-0.625	0.097
20117	0.184	-0.625	0.262	0.152	-0.863	0.508	0.226	-0.356	0.481
20118	0.434	0.975	0.508	0.518	1.491	0.508	0.511	1.464	0.481
20119	0.358	0.487	0.467	0.497	1.359	0.273	0.508	1.447	0.501
20120	0.484	1.295	0.513	0.450	1.057	0.491	0.466	1.178	0.501
20121	0.503	1.413	0.262	0.488	1.302	0.501	0.482	1.279	0.481
20122	0.097	-1.181	0.224	0.272	-0.091	0.235	0.297	0.099	0.513
20123	0.508	1.447	0.262	0.488	1.302	0.478	0.482	1.279	0.501
20124	0.429	0.942	0.262	0.494	1.340	0.193	0.508	1.447	0.050
20125	0.497	1.380	0.296	0.509	1.434	0.341	0.482	1.279	0.142
20126	0.184	-0.625	0.224	0.102	-1.184	0.235	0.384	0.655	0.492

20127	0.226	−0.356	0.450	0.196	−0.581	0.235	0.384	0.655	0.501
20128	0.326	0.285	0.467	0.272	−0.091	0.508	0.384	0.655	0.512
20129	0.326	0.285	0.142	0.193	−0.600	0.235	0.263	−0.120	0.501
20130	0.511	1.464	0.450	0.491	1.321	0.501	0.513	1.481	0.508
20131	0.511	1.464	0.450	0.500	1.378	0.501	0.505	1.430	0.481
20132	0.516	1.498	0.508	0.512	1.453	0.371	0.503	1.413	0.467
20133	0.300	0.116	0.508	0.234	−0.336	0.491	0.432	0.958	0.450
20134	0.468	1.194	0.501	0.421	0.869	0.273	0.503	1.413	0.097
20135	0.487	1.312	0.508	0.512	1.453	0.491	0.492	1.346	0.185
20136	0.184	−0.625	0.450	0.272	−0.091	0.309	0.142	−0.895	0.492
20137	0.447	1.060	0.224	0.488	1.302	0.235	0.508	1.447	0.508
20138	0.500	1.397	0.224	0.509	1.434	0.309	0.505	1.430	0.492
20139	0.500	1.397	0.185	0.509	1.434	0.478	0.505	1.430	0.492
20140	0.184	−0.625	0.492	0.275	−0.073	0.273	0.142	−0.895	0.492
20141	0.492	1.346	0.358	0.515	1.472	0.149	0.484	1.295	0.142
20142	0.503	1.413	0.512	0.515	1.472	0.512	0.450	1.076	0.492
20143	0.516	1.498	0.185	0.509	1.434	0.462	0.513	1.481	0.185
20144	0.466	1.178	0.185	0.500	1.378	0.235	0.492	1.346	0.224
20145	0.408	0.807	0.142	0.509	1.434	0.193	0.484	1.295	0.467
20146	0.408	0.807	0.467	0.506	1.415	0.149	0.484	1.295	0.467
20147	0.226	−0.356	0.481	0.272	−0.091	0.309	0.050	−1.485	0.262
20148	0.447	1.060	0.185	0.494	1.340	0.341	0.503	1.413	0.501
20149	0.411	0.824	0.481	0.497	1.359	0.102	0.505	1.430	0.501
20150	0.297	0.099	0.224	0.234	−0.336	0.309	0.142	−0.895	0.142
20151	0.447	1.060	0.296	0.474	1.208	0.149	0.505	1.430	0.185
20152	0.226	−0.356	0.385	0.193	−0.600	0.235	0.145	−0.878	0.481
20153	0.050	−1.485	0.224	0.339	0.342	0.478	0.226	−0.356	0.508
20154	0.224	−0.373	0.512	0.234	−0.336	0.478	0.097	−1.181	0.501
20155	0.184	−0.625	0.431	0.234	−0.336	0.273	0.142	−0.895	0.296
20156	0.405	0.790	0.508	0.509	1.434	0.422	0.505	1.430	0.328
20157	0.229	−0.339	0.185	0.234	−0.336	0.491	0.097	−1.181	0.492
20158	0.482	1.279	0.431	0.497	1.359	0.193	0.497	1.380	0.296
20159	0.500	1.397	0.481	0.500	1.378	0.193	0.505	1.430	0.328
20160	0.142	−0.895	0.481	0.377	0.587	0.193	0.184	−0.625	0.467
20161	0.384	0.655	0.224	0.497	1.359	0.508	0.503	1.413	0.262
20162	0.097	−1.181	0.328	0.275	−0.073	0.398	0.263	−0.120	0.512
20163	0.503	1.413	0.296	0.488	1.302	0.422	0.361	0.504	0.224
20164	0.097	−1.181	0.481	0.310	0.153	0.309	0.224	−0.373	0.142
20165	0.261	−0.137	0.481	0.234	−0.336	0.341	0.226	−0.356	0.481
20166	0.326	0.285	0.467	0.310	0.153	0.462	0.297	0.099	0.142
20167	0.492	1.346	0.492	0.477	1.227	0.462	0.500	1.397	0.142
20168	0.187	−0.608	0.508	0.275	−0.073	0.478	0.142	−0.895	0.467
20169	0.187	−0.608	0.358	0.149	−0.882	0.235	0.224	−0.373	0.512
20170	0.482	1.279	0.508	0.509	1.434	0.193	0.492	1.346	0.501
20171	0.492	1.346	0.185	0.459	1.114	0.398	0.495	1.363	0.050
20172	0.297	0.099	0.501	0.237	−0.317	0.193	0.050	−1.485	0.050
20173	0.358	0.487	0.224	0.193	−0.600	0.193	0.145	−0.878	0.142
20174	0.263	−0.120	0.224	0.149	−0.882	0.309	0.187	−0.608	0.097
20175	0.224	−0.373	0.097	0.310	0.153	0.235	0.224	−0.373	0.185
20176	0.184	−0.625	0.262	0.339	0.342	0.398	0.100	−1.164	0.508
20177	0.326	0.285	0.431	0.102	−1.184	0.398	0.326	0.285	0.512
20178	0.505	1.430	0.431	0.307	0.135	0.398	0.492	1.346	0.262
20179	0.187	−0.608	0.385	0.149	−0.882	0.235	0.263	−0.120	0.512
20180	0.184	−0.625	0.492	0.234	−0.336	0.508	0.142	−0.895	0.512
20181	0.503	1.413	0.501	0.474	1.208	0.309	0.495	1.363	0.358
20182	0.476	1.245	0.501	0.480	1.246	0.491	0.463	1.161	0.358
20183	0.224	−0.373	0.501	0.275	−0.073	0.478	0.097	−1.181	0.358
20184	0.408	0.807	0.385	0.424	0.888	0.422	0.497	1.380	0.358
20185	0.476	1.245	0.431	0.491	1.321	0.235	0.511	1.464	0.512
20186	0.187	−0.608	0.508	0.193	−0.600	0.512	0.226	−0.356	0.142
20187	0.187	−0.608	0.501	0.193	−0.600	0.341	0.226	−0.356	0.142

20188	0.187	-0.608	0.185	0.193	-0.600	0.514	0.226	-0.356	0.097
20189	0.505	1.430	0.501	0.506	1.415	0.235	0.508	1.447	0.513
20190	0.505	1.430	0.501	0.395	0.700	0.193	0.489	1.329	0.262
20191	0.495	1.363	0.481	0.424	0.888	0.514	0.511	1.464	0.467
20192	0.142	-0.895	0.224	0.313	0.172	0.235	0.224	-0.373	0.097
20193	0.497	1.380	0.142	0.345	0.379	0.512	0.511	1.464	0.097
20194	0.050	-1.485	0.185	0.459	1.114	0.501	0.295	0.082	0.097
20195	0.466	1.178	0.492	0.465	1.152	0.273	0.508	1.447	0.431
20196	0.432	0.958	0.185	0.474	1.208	0.235	0.489	1.329	0.450
20197	0.261	-0.137	0.467	0.237	-0.317	0.444	0.142	-0.895	0.142
20198	0.263	-0.120	0.409	0.193	-0.600	0.235	0.142	-0.895	0.481
20199	0.468	1.194	0.513	0.401	0.737	0.444	0.487	1.312	0.409
20200	0.184	-0.625	0.467	0.196	-0.581	0.478	0.295	0.082	0.467
20201	0.050	-1.485	0.142	0.193	-0.600	0.478	0.295	0.082	0.513
20202	0.266	-0.103	0.492	0.310	0.153	0.491	0.261	-0.137	0.142
20203	0.297	0.099	0.512	0.237	-0.317	0.491	0.184	-0.625	0.501
20204	0.297	0.099	0.185	0.398	0.718	0.491	0.355	0.470	0.513
20205	0.297	0.099	0.501	0.398	0.718	0.508	0.355	0.470	0.513
20206	0.297	0.099	0.097	0.398	0.718	0.491	0.355	0.470	0.262
20207	0.224	-0.373	0.450	0.234	-0.336	0.491	0.142	-0.895	0.481
20208	0.426	0.925	0.513	0.512	1.453	0.478	0.487	1.312	0.492
20209	0.142	-0.895	0.512	0.310	0.153	0.491	0.142	-0.895	0.492
20210	0.511	1.464	0.501	0.491	1.321	0.491	0.487	1.312	0.142
20211	0.445	1.043	0.512	0.477	1.227	0.341	0.511	1.464	0.142
20212	0.429	0.942	0.450	0.421	0.869	0.273	0.500	1.397	0.050
20213	0.261	-0.137	0.185	0.234	-0.336	0.309	0.187	-0.608	0.409
20214	0.500	1.397	0.185	0.512	1.453	0.512	0.482	1.279	0.501
20215	0.142	-0.895	0.185	0.345	0.379	0.235	0.097	-1.181	0.467
20216	0.508	1.447	0.185	0.512	1.453	0.193	0.453	1.093	0.501
20217	0.097	-1.181	0.358	0.234	-0.336	0.309	0.224	-0.373	0.501
20218	0.184	-0.625	0.467	0.193	-0.600	0.309	0.263	-0.120	0.224
20219	0.384	0.655	0.142	0.515	1.472	0.478	0.466	1.178	0.512
20220	0.184	-0.625	0.409	0.234	-0.336	0.235	0.358	0.487	0.262
20221	0.482	1.279	0.409	0.515	1.472	0.193	0.508	1.447	0.409
20222	0.479	1.262	0.328	0.497	1.359	0.149	0.489	1.329	0.328
20223	0.226	-0.356	0.481	0.272	-0.091	0.491	0.142	-0.895	0.224
20224	0.326	0.285	0.262	0.237	-0.317	0.512	0.263	-0.120	0.262
20225	0.508	1.447	0.142	0.447	1.039	0.462	0.466	1.178	0.185
20226	0.142	-0.895	0.328	0.237	-0.317	0.491	0.187	-0.608	0.512
20227	0.508	1.447	0.481	0.447	1.039	0.193	0.466	1.178	0.512
20228	0.503	1.413	0.481	0.482	1.265	0.508	0.450	1.076	0.492
20229	0.503	1.413	0.185	0.482	1.265	0.512	0.450	1.076	0.467
20230	0.482	1.279	0.050	0.485	1.283	0.512	0.505	1.430	0.492
20231	0.500	1.397	0.508	0.491	1.321	0.273	0.513	1.481	0.450
20232	0.500	1.397	0.501	0.491	1.321	0.422	0.513	1.481	0.512
20233	0.500	1.397	0.450	0.509	1.434	0.491	0.482	1.279	0.296
20234	0.479	1.262	0.050	0.491	1.321	0.398	0.484	1.295	0.508
20235	0.450	1.076	0.185	0.491	1.321	0.501	0.484	1.295	0.481
20236	0.511	1.464	0.185	0.480	1.246	0.053	0.500	1.397	0.385
20237	0.508	1.447	0.492	0.491	1.321	0.512	0.492	1.346	0.385
20238	0.466	1.178	0.492	0.491	1.321	0.512	0.484	1.295	0.501
20239	0.050	-1.485	0.492	0.342	0.361	0.149	0.261	-0.137	0.492
20240	0.361	0.504	0.508	0.272	-0.091	0.491	0.261	-0.137	0.501
20241	0.361	0.504	0.296	0.307	0.135	0.371	0.261	-0.137	0.224
20242	0.511	1.464	0.185	0.509	1.434	0.341	0.445	1.043	0.224
20243	0.295	0.082	0.481	0.234	-0.336	0.193	0.142	-0.895	0.296
20244	0.297	0.099	0.450	0.196	-0.581	0.501	0.142	-0.895	0.508
20245	0.358	0.487	0.492	0.310	0.153	0.102	0.184	-0.625	0.296
20246	0.358	0.487	0.508	0.310	0.153	0.149	0.142	-0.895	0.512
20247	0.463	1.161	0.467	0.477	1.227	0.273	0.511	1.464	0.512
20248	0.358	0.487	0.508	0.234	-0.336	0.501	0.224	-0.373	0.512

20249	0.184	-0.625	0.501	0.196	-0.581	0.235	0.295	0.082	0.512
20250	0.184	-0.625	0.492	0.149	-0.882	0.462	0.297	0.099	0.185
20251	0.508	1.447	0.481	0.485	1.283	0.273	0.468	1.194	0.450
20252	0.505	1.430	0.508	0.509	1.434	0.193	0.505	1.430	0.512
20253	0.479	1.262	0.513	0.459	1.114	0.273	0.484	1.295	0.262
20254	0.497	1.380	0.513	0.488	1.302	0.398	0.463	1.161	0.431
20255	0.261	-0.137	0.185	0.193	-0.600	0.149	0.184	-0.625	0.097
20256	0.466	1.178	0.508	0.509	1.434	0.309	0.384	0.655	0.512
20257	0.476	1.245	0.262	0.509	1.434	0.309	0.505	1.430	0.450
20258	0.497	1.380	0.508	0.509	1.434	0.444	0.505	1.430	0.513
20259	0.300	0.116	0.097	0.275	-0.073	0.491	0.408	0.807	0.492
20260	0.484	1.295	0.185	0.421	0.869	0.512	0.511	1.464	0.385
20261	0.447	1.060	0.142	0.494	1.340	0.508	0.503	1.413	0.467
20262	0.142	-0.895	0.262	0.404	0.756	0.235	0.295	0.082	0.185
20263	0.463	1.161	0.385	0.506	1.415	0.053	0.511	1.464	0.224
20264	0.145	-0.878	0.328	0.053	-1.504	0.053	0.329	0.301	0.224
20265	0.508	1.447	0.492	0.506	1.415	0.514	0.466	1.178	0.224
20266	0.508	1.447	0.385	0.506	1.415	0.235	0.466	1.178	0.358
20267	0.097	-1.181	0.431	0.152	-0.863	0.491	0.297	0.099	0.492
20268	0.505	1.430	0.328	0.494	1.340	0.398	0.484	1.295	0.262
20269	0.187	-0.608	0.508	0.374	0.568	0.478	0.142	-0.895	0.142
20270	0.184	-0.625	0.431	0.342	0.361	0.273	0.184	-0.625	0.097
20271	0.187	-0.608	0.142	0.193	-0.600	0.398	0.224	-0.373	0.142
20272	0.503	1.413	0.492	0.503	1.396	0.235	0.500	1.397	0.508
20273	0.187	-0.608	0.097	0.102	-1.184	0.273	0.329	0.301	0.328
20274	0.261	-0.137	0.385	0.149	-0.882	0.512	0.142	-0.895	0.097
20275	0.184	-0.625	0.224	0.272	-0.091	0.193	0.224	-0.373	0.328
20276	0.511	1.464	0.224	0.503	1.396	0.193	0.445	1.043	0.467
20277	0.295	0.082	0.224	0.240	-0.299	0.235	0.050	-1.485	0.467
20278	0.482	1.279	0.097	0.456	1.095	0.193	0.503	1.413	0.142
20279	0.226	-0.356	0.508	0.272	-0.091	0.501	0.100	-1.164	0.142
20280	0.097	-1.181	0.492	0.196	-0.581	0.149	0.295	0.082	0.512
20281	0.142	-0.895	0.513	0.275	-0.073	0.193	0.261	-0.137	0.492
20282	0.497	1.380	0.513	0.404	0.756	0.512	0.476	1.245	0.501
20283	0.295	0.082	0.450	0.149	-0.882	0.512	0.184	-0.625	0.385
20284	0.142	-0.895	0.142	0.313	0.172	0.462	0.142	-0.895	0.328
20285	0.261	-0.137	0.296	0.310	0.153	0.462	0.329	0.301	0.358
20286	0.505	1.430	0.512	0.442	1.001	0.422	0.511	1.464	0.450
20287	0.505	1.430	0.328	0.485	1.283	0.508	0.500	1.397	0.467
20288	0.484	1.295	0.481	0.515	1.472	0.478	0.508	1.447	0.450
20289	0.492	1.346	0.328	0.506	1.415	0.341	0.468	1.194	0.450
20290	0.263	-0.120	0.097	0.234	-0.336	0.235	0.226	-0.356	0.050
20291	0.263	-0.120	0.296	0.053	-1.504	0.444	0.224	-0.373	0.185
20292	0.263	-0.120	0.185	0.053	-1.504	0.478	0.224	-0.373	0.450
20293	0.358	0.487	0.508	0.512	1.453	0.491	0.503	1.413	0.409
20294	0.187	-0.608	0.508	0.234	-0.336	0.462	0.184	-0.625	0.481
20295	0.500	1.397	0.450	0.491	1.321	0.512	0.500	1.397	0.512
20296	0.050	-1.485	0.262	0.401	0.737	0.462	0.050	-1.485	0.450
20297	0.503	1.413	0.508	0.480	1.246	0.444	0.508	1.447	0.501
20298	0.329	0.301	0.328	0.275	-0.073	0.512	0.408	0.807	0.508
20299	0.445	1.043	0.224	0.398	0.718	0.235	0.511	1.464	0.450
20300	0.184	-0.625	0.185	0.234	-0.336	0.309	0.224	-0.373	0.467
20301	0.142	-0.895	0.262	0.272	-0.091	0.444	0.145	-0.878	0.508
20302	0.484	1.295	0.185	0.512	1.453	0.491	0.468	1.194	0.450
20303	0.332	0.318	0.142	0.196	-0.581	0.491	0.184	-0.625	0.501
20304	0.261	-0.137	0.358	0.193	-0.600	0.491	0.097	-1.181	0.492
20305	0.300	0.116	0.481	0.234	-0.336	0.491	0.050	-1.485	0.512
20306	0.184	-0.625	0.481	0.193	-0.600	0.235	0.184	-0.625	0.185
20307	0.511	1.464	0.450	0.506	1.415	0.514	0.503	1.413	0.492
20308	0.224	-0.373	0.224	0.149	-0.882	0.309	0.329	0.301	0.262
20309	0.261	-0.137	0.142	0.193	-0.600	0.341	0.097	-1.181	0.097

20310	0.426	0.925	0.224	0.515	1.472	0.508	0.474	1.228	0.328
20311	0.426	0.925	0.262	0.515	1.472	0.491	0.474	1.228	0.431
20312	0.511	1.464	0.296	0.462	1.133	0.193	0.495	1.363	0.224
20313	0.511	1.464	0.296	0.462	1.133	0.512	0.495	1.363	0.224
20314	0.505	1.430	0.431	0.424	0.888	0.514	0.476	1.245	0.508
20315	0.503	1.413	0.262	0.503	1.396	0.053	0.513	1.481	0.481
20316	0.484	1.295	0.050	0.474	1.208	0.273	0.495	1.363	0.501
20317	0.297	0.099	0.296	0.342	0.361	0.501	0.050	−1.485	0.097
20318	0.184	−0.625	0.262	0.234	−0.336	0.398	0.142	−0.895	0.481
20319	0.495	1.363	0.224	0.444	1.020	0.478	0.487	1.312	0.508
20320	0.500	1.397	0.050	0.482	1.265	0.193	0.495	1.363	0.262
20321	0.358	0.487	0.492	0.488	1.302	0.309	0.505	1.430	0.512
20322	0.505	1.430	0.501	0.462	1.133	0.508	0.500	1.397	0.097
20323	0.384	0.655	0.142	0.515	1.472	0.309	0.505	1.430	0.097
20324	0.466	1.178	0.508	0.462	1.133	0.053	0.474	1.228	0.185
20325	0.503	1.413	0.142	0.444	1.020	0.512	0.461	1.144	0.296
20326	0.426	0.925	0.224	0.509	1.434	0.309	0.450	1.076	0.296
20327	0.261	−0.137	0.097	0.234	−0.336	0.478	0.097	−1.181	0.328
20328	0.326	0.285	0.450	0.313	0.172	0.053	0.184	−0.625	0.481
20329	0.432	0.958	0.409	0.447	1.039	0.341	0.484	1.295	0.481
20330	0.468	1.194	0.224	0.494	1.340	0.462	0.505	1.430	0.513
20331	0.511	1.464	0.467	0.491	1.321	0.193	0.487	1.312	0.492
20332	0.508	1.447	0.467	0.491	1.321	0.193	0.487	1.312	0.501
20333	0.511	1.464	0.481	0.491	1.321	0.478	0.487	1.312	0.513
20334	0.184	−0.625	0.512	0.237	−0.317	0.478	0.142	−0.895	0.481
20335	0.482	1.279	0.467	0.509	1.434	0.512	0.489	1.329	0.328
20336	0.050	−1.485	0.409	0.313	0.172	0.309	0.184	−0.625	0.501
20337	0.050	−1.485	0.513	0.342	0.361	0.273	0.226	−0.356	0.142
20338	0.508	1.447	0.492	0.503	1.396	0.273	0.463	1.161	0.224
20339	0.484	1.295	0.481	0.491	1.321	0.309	0.463	1.161	0.262
20340	0.187	−0.608	0.296	0.193	−0.600	0.309	0.261	−0.137	0.224
20341	0.479	1.262	0.262	0.512	1.453	0.273	0.497	1.380	0.467
20342	0.489	1.329	0.185	0.512	1.453	0.478	0.497	1.380	0.492
20343	0.329	0.301	0.508	0.053	−1.504	0.398	0.142	−0.895	0.513
20344	0.263	−0.120	0.492	0.272	−0.091	0.309	0.184	−0.625	0.358
20345	0.500	1.397	0.450	0.500	1.378	0.491	0.476	1.245	0.467
20346	0.495	1.363	0.262	0.401	0.737	0.491	0.505	1.430	0.185
20347	0.513	1.481	0.508	0.480	1.246	0.501	0.500	1.397	0.328
20348	0.329	0.301	0.097	0.193	−0.600	0.508	0.297	0.099	0.185
20349	0.224	−0.373	0.512	0.313	0.172	0.508	0.329	0.301	0.142
20350	0.497	1.380	0.358	0.512	1.453	0.508	0.495	1.363	0.262
20351	0.184	−0.625	0.142	0.310	0.153	0.422	0.297	0.099	0.262
20352	0.297	0.099	0.409	0.053	−1.504	0.341	0.332	0.318	0.185
20353	0.497	1.380	0.409	0.518	1.491	0.341	0.463	1.161	0.142
20354	0.050	−1.485	0.262	0.307	0.135	0.371	0.266	−0.103	0.450
20355	0.450	1.076	0.224	0.477	1.227	0.501	0.505	1.430	0.431
20356	0.261	−0.137	0.262	0.053	−1.504	0.273	0.224	−0.373	0.492
20357	0.184	−0.625	0.481	0.342	0.361	0.508	0.142	−0.895	0.262
20358	0.500	1.397	0.481	0.459	1.114	0.309	0.482	1.279	0.296
20359	0.261	−0.137	0.296	0.193	−0.600	0.341	0.142	−0.895	0.296
20360	0.261	−0.137	0.262	0.193	−0.600	0.309	0.142	−0.895	0.224
20361	0.505	1.430	0.501	0.474	1.208	0.478	0.463	1.161	0.224
20362	0.466	1.178	0.492	0.480	1.246	0.491	0.518	1.514	0.185
20363	0.508	1.447	0.142	0.512	1.453	0.235	0.497	1.380	0.450
20364	0.263	−0.120	0.467	0.307	0.135	0.422	0.000	?	0.262
20365	0.224	−0.373	0.467	0.272	−0.091	0.149	0.050	−1.485	0.097
20366	0.261	−0.137	0.492	0.272	−0.091	0.341	0.050	−1.485	0.142
20367	0.263	−0.120	0.142	0.307	0.135	0.478	0.142	−0.895	0.296
20368	0.297	0.099	0.296	0.307	0.135	0.235	0.097	−1.181	0.262
20369	0.226	−0.356	0.262	0.275	−0.073	0.478	0.184	−0.625	0.508
20370	0.450	1.076	0.142	0.477	1.227	0.501	0.503	1.413	0.467

20371	0.463	1.161	0.296	0.398	0.718	0.512	0.511	1.464	0.328
20372	0.142	-0.895	0.296	0.310	0.153	0.102	0.263	-0.120	0.512
20373	0.453	1.093	0.050	0.485	1.283	0.478	0.505	1.430	0.262
20374	0.453	1.093	0.185	0.485	1.283	0.462	0.505	1.430	0.296
20375	0.508	1.447	0.185	0.500	1.378	0.501	0.355	0.470	0.262
20376	0.508	1.447	0.185	0.506	1.415	0.273	0.355	0.470	0.262
20377	0.500	1.397	0.450	0.506	1.415	0.149	0.355	0.470	0.296
20378	0.508	1.447	0.450	0.506	1.415	0.235	0.355	0.470	0.481
20379	0.450	1.076	0.431	0.424	0.888	0.512	0.505	1.430	0.142
20380	0.226	-0.356	0.508	0.342	0.361	0.508	0.145	-0.878	0.501
20381	0.226	-0.356	0.358	0.342	0.361	0.512	0.145	-0.878	0.501
20382	0.184	-0.625	0.328	0.371	0.549	0.514	0.097	-1.181	0.512
20383	0.487	1.312	0.185	0.497	1.359	0.478	0.513	1.481	0.508
20384	0.358	0.487	0.296	0.272	-0.091	0.193	0.263	-0.120	0.512
20385	0.500	1.397	0.224	0.512	1.453	0.398	0.471	1.211	0.492
20386	0.187	-0.608	0.262	0.307	0.135	0.235	0.097	-1.181	0.512
20387	0.187	-0.608	0.262	0.342	0.361	0.501	0.097	-1.181	0.467
20388	0.145	-0.878	0.508	0.310	0.153	0.193	0.097	-1.181	0.492
20389	0.463	1.161	0.492	0.480	1.246	0.149	0.432	0.958	0.142
20390	0.476	1.245	0.296	0.491	1.321	0.193	0.450	1.076	0.185
20391	0.184	-0.625	0.097	0.237	-0.317	0.273	0.142	-0.895	0.450
20392	0.484	1.295	0.224	0.418	0.850	0.478	0.474	1.228	0.492
20393	0.224	-0.373	0.142	0.152	-0.863	0.491	0.408	0.807	0.481
20394	0.297	0.099	0.142	0.345	0.379	0.398	0.463	1.161	0.262
20395	0.384	0.655	0.224	0.480	1.246	0.501	0.516	1.498	0.492
20396	0.226	-0.356	0.224	0.234	-0.336	0.512	0.142	-0.895	0.296
20397	0.511	1.464	0.097	0.477	1.227	0.512	0.500	1.397	0.262
20398	0.426	0.925	0.358	0.503	1.396	0.309	0.505	1.430	0.508
20399	0.500	1.397	0.358	0.515	1.472	0.053	0.513	1.481	0.467
20400	0.184	-0.625	0.142	0.105	-1.165	0.508	0.263	-0.120	0.358
20401	0.432	0.958	0.185	0.477	1.227	0.491	0.479	1.262	0.481
20402	0.479	1.262	0.142	0.462	1.133	0.478	0.487	1.312	0.481
20403	0.479	1.262	0.142	0.500	1.378	0.273	0.487	1.312	0.358
20404	0.226	-0.356	0.185	0.275	-0.073	0.478	0.142	-0.895	0.050
20405	0.326	0.285	0.262	0.149	-0.882	0.491	0.142	-0.895	0.224
20406	0.300	0.116	0.467	0.234	-0.336	0.478	0.050	-1.485	0.501
20407	0.479	1.262	0.492	0.512	1.453	0.235	0.408	0.807	0.501
20408	0.484	1.295	0.185	0.512	1.453	0.235	0.500	1.397	0.224
20409	0.466	1.178	0.185	0.515	1.472	0.235	0.463	1.161	0.224
20410	0.492	1.346	0.185	0.515	1.472	0.512	0.500	1.397	0.450
20411	0.505	1.430	0.481	0.474	1.208	0.273	0.500	1.397	0.492
20412	0.358	0.487	0.262	0.193	-0.600	0.501	0.224	-0.373	0.185
20413	0.508	1.447	0.262	0.398	0.718	0.512	0.503	1.413	0.492
20414	0.184	-0.625	0.481	0.237	-0.317	0.444	0.263	-0.120	0.467
20415	0.495	1.363	0.262	0.503	1.396	0.514	0.405	0.790	0.481
20416	0.224	-0.373	0.508	0.196	-0.581	0.341	0.329	0.301	0.296
20417	0.229	-0.339	0.142	0.149	-0.882	0.508	0.224	-0.373	0.097
20418	0.097	-1.181	0.492	0.196	-0.581	0.462	0.261	-0.137	0.097
20419	0.263	-0.120	0.296	0.275	-0.073	0.235	0.184	-0.625	0.224
20420	0.432	0.958	0.513	0.480	1.246	0.444	0.511	1.464	0.262
20421	0.432	0.958	0.513	0.491	1.321	0.341	0.511	1.464	0.262
20422	0.382	0.638	0.512	0.401	0.737	0.462	0.492	1.346	0.358
20423	0.492	1.346	0.508	0.500	1.378	0.512	0.466	1.178	0.385
20424	0.497	1.380	0.097	0.515	1.472	0.512	0.492	1.346	0.097
20425	0.505	1.430	0.385	0.509	1.434	0.512	0.447	1.060	0.185
20426	0.503	1.413	0.431	0.307	0.135	0.514	0.511	1.464	0.296
20427	0.387	0.672	0.224	0.053	-1.504	0.501	0.295	0.082	0.467
20428	0.426	0.925	0.185	0.512	1.453	0.235	0.505	1.430	0.467
20429	0.508	1.447	0.501	0.491	1.321	0.235	0.476	1.245	0.467
20430	0.505	1.430	0.431	0.480	1.246	0.235	0.384	0.655	0.481
20431	0.187	-0.608	0.358	0.275	-0.073	0.235	0.384	0.655	0.224

20432	0.500	1.397	0.512	0.477	1.227	0.149	0.497	1.380	0.142
20433	0.500	1.397	0.512	0.491	1.321	0.309	0.484	1.295	0.142
20434	0.479	1.262	0.492	0.480	1.246	0.508	0.495	1.363	0.262
20435	0.224	-0.373	0.296	0.237	-0.317	0.341	0.226	-0.356	0.385
20436	0.142	-0.895	0.050	0.237	-0.317	0.102	0.226	-0.356	0.296
20437	0.184	-0.625	0.508	0.237	-0.317	0.341	0.300	0.116	0.296
20438	0.492	1.346	0.467	0.512	1.453	0.501	0.511	1.464	0.481
20439	0.184	-0.625	0.224	0.272	-0.091	0.193	0.300	0.116	0.501
20440	0.466	1.178	0.296	0.497	1.359	0.341	0.505	1.430	0.328
20441	0.411	0.824	0.492	0.515	1.472	0.149	0.505	1.430	0.296
20442	0.516	1.498	0.501	0.444	1.020	0.514	0.505	1.430	0.296
20443	0.471	1.211	0.467	0.515	1.472	0.514	0.505	1.430	0.224
20444	0.142	-0.895	0.142	0.345	0.379	0.273	0.184	-0.625	0.262
20445	0.489	1.329	0.296	0.509	1.434	0.371	0.447	1.060	0.262
20446	0.511	1.464	0.501	0.462	1.133	0.501	0.511	1.464	0.262
20447	0.184	-0.625	0.467	0.237	-0.317	0.444	0.263	-0.120	0.224
20448	0.500	1.397	0.185	0.444	1.020	0.501	0.429	0.942	0.224
20449	0.097	-1.181	0.501	0.345	0.379	0.371	0.097	-1.181	0.142
20450	0.447	1.060	0.508	0.462	1.133	0.273	0.508	1.447	0.185
20451	0.508	1.447	0.097	0.509	1.434	0.149	0.447	1.060	0.185
20452	0.503	1.413	0.450	0.512	1.453	0.512	0.513	1.481	0.097
20453	0.497	1.380	0.501	0.509	1.434	0.398	0.489	1.329	0.142
20454	0.513	1.481	0.050	0.509	1.434	0.398	0.382	0.638	0.097
20455	0.447	1.060	0.501	0.500	1.378	0.422	0.466	1.178	0.262
20456	0.184	-0.625	0.097	0.234	-0.336	0.444	0.187	-0.608	0.481
20457	0.184	-0.625	0.328	0.234	-0.336	0.193	0.226	-0.356	0.512
20458	0.184	-0.625	0.501	0.234	-0.336	0.512	0.226	-0.356	0.142
20459	0.184	-0.625	0.262	0.234	-0.336	0.508	0.226	-0.356	0.142
20460	0.358	0.487	0.262	0.149	-0.882	0.478	0.361	0.504	0.224
20461	0.466	1.178	0.501	0.310	0.153	0.501	0.495	1.363	0.481
20462	0.142	-0.895	0.501	0.509	1.434	0.462	0.263	-0.120	0.050
20463	0.413	0.841	0.262	0.339	0.342	0.478	0.142	-0.895	0.050
20464	0.411	0.824	0.431	0.102	-1.184	0.444	0.097	-1.181	0.492
20465	0.332	0.318	0.296	0.342	0.361	0.478	0.142	-0.895	0.050
20466	0.482	1.279	0.262	0.503	1.396	0.422	0.503	1.413	0.508
20467	0.261	-0.137	0.450	0.199	-0.562	0.491	0.329	0.301	0.097
20468	0.145	-0.878	0.142	0.339	0.342	0.508	0.097	-1.181	0.508
20469	0.329	0.301	0.142	0.149	-0.882	0.478	0.329	0.301	0.358
20470	0.487	1.312	0.501	0.518	1.491	0.193	0.463	1.161	0.508
20471	0.487	1.312	0.224	0.518	1.491	0.462	0.463	1.161	0.481
20472	0.184	-0.625	0.492	0.275	-0.073	0.235	0.142	-0.895	0.481
20473	0.050	-1.485	0.513	0.371	0.549	0.444	0.145	-0.878	0.512
20474	0.505	1.430	0.097	0.497	1.359	0.193	0.508	1.447	0.296
20475	0.500	1.397	0.467	0.444	1.020	0.273	0.484	1.295	0.492
20476	0.447	1.060	0.481	0.500	1.378	0.102	0.495	1.363	0.512
20477	0.050	-1.485	0.450	0.374	0.568	0.273	0.384	0.655	0.296
20478	0.187	-0.608	0.508	0.275	-0.073	0.193	0.329	0.301	0.328
20479	0.187	-0.608	0.508	0.149	-0.882	0.235	0.361	0.504	0.481
20480	0.489	1.329	0.508	0.509	1.434	0.514	0.450	1.076	0.508
20481	0.489	1.329	0.513	0.398	0.718	0.444	0.466	1.178	0.513
20482	0.489	1.329	0.296	0.398	0.718	0.398	0.447	1.060	0.431
20483	0.505	1.430	0.501	0.421	0.869	0.102	0.447	1.060	0.431
20484	0.295	0.082	0.481	0.444	1.020	0.508	0.050	-1.485	0.508
20485	0.187	-0.608	0.409	0.193	-0.600	0.478	0.184	-0.625	0.296
20486	0.487	1.312	0.142	0.509	1.434	0.149	0.450	1.076	0.262
20487	0.455	1.110	0.142	0.506	1.415	0.491	0.411	0.824	0.385
20488	0.497	1.380	0.142	0.482	1.265	0.398	0.482	1.279	0.501
20489	0.503	1.413	0.328	0.497	1.359	0.053	0.511	1.464	0.142
20490	0.468	1.194	0.512	0.459	1.114	0.149	0.453	1.093	0.224
20491	0.503	1.413	0.512	0.485	1.283	0.149	0.505	1.430	0.492
20492	0.497	1.380	0.501	0.447	1.039	0.309	0.511	1.464	0.409

20493	0.487	1.312	0.481	0.477	1.227	0.102	0.450	1.076	0.467
20494	0.476	1.245	0.512	0.421	0.869	0.478	0.466	1.178	0.328
20495	0.508	1.447	0.492	0.485	1.283	0.478	0.508	1.447	0.409
20496	0.513	1.481	0.492	0.503	1.396	0.478	0.447	1.060	0.492
20497	0.511	1.464	0.481	0.474	1.208	0.514	0.500	1.397	0.501
20498	0.184	−0.625	0.501	0.193	−0.600	0.491	0.487	1.312	0.296
20499	0.505	1.430	0.142	0.465	1.152	0.371	0.511	1.464	0.512
20500	0.263	−0.120	0.513	0.234	−0.336	0.512	0.187	−0.608	0.512
20501	0.503	1.413	0.481	0.447	1.039	0.398	0.487	1.312	0.296
20502	0.097	−1.181	0.262	0.193	−0.600	0.398	0.263	−0.120	0.481
20503	0.187	−0.608	0.262	0.275	−0.073	0.478	0.097	−1.181	0.508
20504	0.145	−0.878	0.224	0.102	−1.184	0.235	0.329	0.301	0.358
20505	0.261	−0.137	0.224	0.275	−0.073	0.235	0.432	0.958	0.481
20506	0.382	0.638	0.097	0.196	−0.581	0.235	0.229	−0.339	0.296
20507	0.326	0.285	0.508	0.234	−0.336	0.235	0.226	−0.356	0.224
20508	0.492	1.346	0.185	0.509	1.434	0.514	0.505	1.430	0.431
20509	0.382	0.638	0.481	0.444	1.020	0.478	0.476	1.245	0.296
20510	0.429	0.942	0.512	0.404	0.756	0.514	0.497	1.380	0.224
20511	0.329	0.301	0.224	0.102	−1.184	0.273	0.097	−1.181	0.501
20512	0.503	1.413	0.385	0.512	1.453	0.501	0.474	1.228	0.467
20513	0.432	0.958	0.501	0.477	1.227	0.371	0.500	1.397	0.224
20514	0.142	−0.895	0.450	0.149	−0.882	0.341	0.261	−0.137	0.450
20515	0.492	1.346	0.385	0.488	1.302	0.309	0.505	1.430	0.328
20516	0.097	−1.181	0.450	0.398	0.718	0.235	0.097	−1.181	0.450
20517	0.387	0.672	0.481	0.053	−1.504	0.235	0.097	−1.181	0.450
20518	0.224	−0.373	0.296	0.149	−0.882	0.149	0.184	−0.625	0.262
20519	0.224	−0.373	0.296	0.152	−0.863	0.235	0.297	0.099	0.224
20520	0.224	−0.373	0.262	0.310	0.153	0.193	0.297	0.099	0.512
20521	0.097	−1.181	0.450	0.102	−1.184	0.491	0.332	0.318	0.358
20522	0.505	1.430	0.358	0.477	1.227	0.478	0.476	1.245	0.501
20523	0.492	1.346	0.296	0.477	1.227	0.398	0.474	1.228	0.481
20524	0.508	1.447	0.450	0.474	1.208	0.149	0.516	1.498	0.224
20525	0.511	1.464	0.450	0.512	1.453	0.273	0.497	1.380	0.409
20526	0.450	1.076	0.097	0.488	1.302	0.102	0.495	1.363	0.492
20527	0.142	−0.895	0.481	0.371	0.549	0.053	0.000	?	0.512
20528	0.295	0.082	0.296	0.515	1.472	0.053	0.503	1.413	0.481
20529	0.508	1.447	0.508	0.398	0.718	0.341	0.476	1.245	0.481
20530	0.332	0.318	0.262	0.398	0.718	0.462	0.332	0.318	0.481
20531	0.482	1.279	0.224	0.474	1.208	0.193	0.495	1.363	0.409
20532	0.329	0.301	0.512	0.234	−0.336	0.444	0.145	−0.878	0.185
20533	0.100	−1.164	0.481	0.237	−0.317	0.309	0.224	−0.373	0.450
20534	0.300	0.116	0.358	0.234	−0.336	0.235	0.263	−0.120	0.513
20535	0.184	−0.625	0.185	0.237	−0.317	0.235	0.224	−0.373	0.481
20536	0.503	1.413	0.262	0.506	1.415	0.341	0.468	1.194	0.185
20537	0.500	1.397	0.481	0.482	1.265	0.501	0.495	1.363	0.142
20538	0.453	1.093	0.142	0.515	1.472	0.508	0.516	1.498	0.142
20539	0.261	−0.137	0.262	0.275	−0.073	0.309	0.361	0.504	0.142
20540	0.497	1.380	0.508	0.503	1.396	0.478	0.466	1.178	0.450
20541	0.000	?	0.467	0.374	0.568	0.193	0.184	−0.625	0.492
20542	0.329	0.301	0.492	0.339	0.342	0.193	0.332	0.318	0.358
20543	0.226	−0.356	0.185	0.307	0.135	0.193	0.189	−0.592	0.328
20544	0.187	−0.608	0.328	0.234	−0.336	0.273	0.142	−0.895	0.492
20545	0.263	−0.120	0.512	0.234	−0.336	0.309	0.261	−0.137	0.512
20546	0.184	−0.625	0.512	0.149	−0.882	0.462	0.263	−0.120	0.492
20547	0.142	−0.895	0.296	0.234	−0.336	0.235	0.184	−0.625	0.508
20548	0.358	0.487	0.385	0.193	−0.600	0.491	0.145	−0.878	0.296
20549	0.484	1.295	0.512	0.497	1.359	0.478	0.450	1.076	0.512
20550	0.479	1.262	0.512	0.477	1.227	0.501	0.426	0.925	0.224
20551	0.450	1.076	0.262	0.395	0.700	0.478	0.487	1.312	0.508
20552	0.224	−0.373	0.328	0.149	−0.882	0.462	0.261	−0.137	0.481
20553	0.145	−0.878	0.385	0.275	−0.073	0.508	0.295	0.082	0.050

20554	0.224	-0.373	0.409	0.102	-1.184	0.512	0.295	0.082	0.262
20555	0.263	-0.120	0.513	0.053	-1.504	0.478	0.224	-0.373	0.185
20556	0.297	0.099	0.508	0.053	-1.504	0.422	0.224	-0.373	0.296
20557	0.300	0.116	0.224	0.342	0.361	0.102	0.184	-0.625	0.142
20558	0.432	0.958	0.185	0.465	1.152	0.398	0.447	1.060	0.467
20559	0.263	-0.120	0.262	0.193	-0.600	0.235	0.263	-0.120	0.262
20560	0.050	-1.485	0.296	0.444	1.020	0.508	0.097	-1.181	0.481
20561	0.297	0.099	0.224	0.310	0.153	0.512	0.000	?	0.358
20562	0.263	-0.120	0.481	0.234	-0.336	0.501	0.142	-0.895	0.185
20563	0.226	-0.356	0.142	0.237	-0.317	0.149	0.297	0.099	0.296
20564	0.050	-1.485	0.512	0.342	0.361	0.491	0.261	-0.137	0.481
20565	0.492	1.346	0.467	0.497	1.359	0.273	0.503	1.413	0.492
20566	0.497	1.380	0.097	0.506	1.415	0.341	0.466	1.178	0.224
20567	0.145	-0.878	0.224	0.310	0.153	0.444	0.329	0.301	0.501
20568	0.497	1.380	0.481	0.477	1.227	0.478	0.513	1.481	0.508
20569	0.145	-0.878	0.224	0.193	-0.600	0.341	0.263	-0.120	0.224
20570	0.229	-0.339	0.450	0.193	-0.600	0.512	0.297	0.099	0.142
20571	0.097	-1.181	0.050	0.193	-0.600	0.512	0.261	-0.137	0.142
20572	0.000	?	0.512	0.275	-0.073	0.371	0.261	-0.137	0.512
20573	0.453	1.093	0.508	0.310	0.153	0.341	0.297	0.099	0.097
20574	0.413	0.841	0.492	0.462	1.133	0.149	0.482	1.279	0.296
20575	0.224	-0.373	0.185	0.234	-0.336	0.398	0.142	-0.895	0.450
20576	0.463	1.161	0.262	0.491	1.321	0.398	0.495	1.363	0.512
20577	0.463	1.161	0.492	0.480	1.246	0.371	0.495	1.363	0.185
20578	0.476	1.245	0.508	0.503	1.396	0.235	0.513	1.481	0.467
20579	0.508	1.447	0.492	0.480	1.246	0.491	0.508	1.447	0.262
20580	0.466	1.178	0.262	0.465	1.152	0.444	0.505	1.430	0.481
20581	0.413	0.841	0.508	0.512	1.453	0.309	0.492	1.346	0.262
20582	0.500	1.397	0.224	0.512	1.453	0.491	0.516	1.498	0.185
20583	0.489	1.329	0.513	0.477	1.227	0.478	0.463	1.161	0.467
20584	0.482	1.279	0.409	0.421	0.869	0.491	0.484	1.295	0.501
20585	0.297	0.099	0.508	0.102	-1.184	0.309	0.145	-0.878	0.467
20586	0.261	-0.137	0.224	0.404	0.756	0.235	0.000	?	0.142
20587	0.184	-0.625	0.185	0.237	-0.317	0.193	0.184	-0.625	0.142
20588	0.508	1.447	0.513	0.506	1.415	0.309	0.447	1.060	0.512
20589	0.495	1.363	0.185	0.509	1.434	0.398	0.487	1.312	0.097
20590	0.447	1.060	0.224	0.497	1.359	0.273	0.476	1.245	0.097
20591	0.261	-0.137	0.508	0.149	-0.882	0.444	0.263	-0.120	0.492
20592	0.505	1.430	0.450	0.491	1.321	0.309	0.487	1.312	0.501
20593	0.097	-1.181	0.492	0.272	-0.091	0.273	0.295	0.082	0.467
20594	0.000	?	0.492	0.342	0.361	0.193	0.261	-0.137	0.262
20595	0.500	1.397	0.224	0.447	1.039	0.102	0.508	1.447	0.185
20596	0.361	0.504	0.328	0.482	1.265	0.508	0.468	1.194	0.492
20597	0.142	-0.895	0.467	0.345	0.379	0.508	0.358	0.487	0.501
20598	0.413	0.841	0.262	0.515	1.472	0.491	0.479	1.262	0.185
20599	0.413	0.841	0.296	0.515	1.472	0.501	0.479	1.262	0.385
20600	0.261	-0.137	0.481	0.371	0.549	0.193	0.355	0.470	0.513
20601	0.226	-0.356	0.431	0.342	0.361	0.193	0.050	-1.485	0.481
20602	0.263	-0.120	0.512	0.149	-0.882	0.273	0.226	-0.356	0.262
20603	0.479	1.262	0.512	0.401	0.737	0.309	0.500	1.397	0.262
20604	0.479	1.262	0.431	0.401	0.737	0.053	0.500	1.397	0.097
20605	0.297	0.099	0.431	0.371	0.549	0.149	0.224	-0.373	0.481
20606	0.263	-0.120	0.185	0.234	-0.336	0.149	0.224	-0.373	0.508
20607	0.500	1.397	0.185	0.488	1.302	0.512	0.450	1.076	0.481
20608	0.489	1.329	0.467	0.450	1.057	0.462	0.489	1.329	0.296
20609	0.145	-0.878	0.467	0.307	0.135	0.371	0.187	-0.608	0.097
20610	0.468	1.194	0.431	0.494	1.340	0.193	0.492	1.346	0.097
20611	0.468	1.194	0.409	0.480	1.246	0.149	0.466	1.178	0.097
20612	0.492	1.346	0.185	0.488	1.302	0.273	0.479	1.262	0.185
20613	0.142	-0.895	0.142	0.310	0.153	0.371	0.295	0.082	0.512
20614	0.295	0.082	0.501	0.234	-0.336	0.309	0.097	-1.181	0.385

20615	0.261	-0.137	0.492	0.193	-0.600	0.273	0.097	-1.181	0.508
20616	0.142	-0.895	0.409	0.310	0.153	0.235	0.224	-0.373	0.492
20617	0.300	0.116	0.492	0.398	0.718	0.341	0.261	-0.137	0.097
20618	0.300	0.116	0.501	0.275	-0.073	0.235	0.261	-0.137	0.262
20619	0.000	?	0.481	0.444	1.020	0.341	0.361	0.504	0.501
20620	0.050	-1.485	0.185	0.310	0.153	0.341	0.382	0.638	0.296
20621	0.184	-0.625	0.481	0.275	-0.073	0.341	0.097	-1.181	0.467
20622	0.184	-0.625	0.512	0.193	-0.600	0.273	0.184	-0.625	0.328
20623	0.184	-0.625	0.481	0.102	-1.184	0.309	0.295	0.082	0.492
20624	0.450	1.076	0.185	0.509	1.434	0.309	0.461	1.144	0.450
20625	0.450	1.076	0.508	0.509	1.434	0.478	0.461	1.144	0.296
20626	0.429	0.942	0.508	0.491	1.321	0.462	0.461	1.144	0.142
20627	0.511	1.464	0.512	0.500	1.378	0.309	0.484	1.295	0.358
20628	0.361	0.504	0.097	0.193	-0.600	0.341	0.224	-0.373	0.467
20629	0.332	0.318	0.492	0.193	-0.600	0.235	0.142	-0.895	0.431
20630	0.184	-0.625	0.185	0.275	-0.073	0.462	0.142	-0.895	0.450
20631	0.297	0.099	0.262	0.310	0.153	0.341	0.263	-0.120	0.262
20632	0.226	-0.356	0.481	0.053	-1.504	0.341	0.387	0.672	0.492
20633	0.261	-0.137	0.492	0.149	-0.882	0.491	0.297	0.099	0.296
20634	0.261	-0.137	0.467	0.149	-0.882	0.273	0.297	0.099	0.513
20635	0.508	1.447	0.492	0.512	1.453	0.462	0.484	1.295	0.512
20636	0.487	1.312	0.142	0.465	1.152	0.309	0.505	1.430	0.501
20637	0.297	0.099	0.142	0.374	0.568	0.508	0.329	0.301	0.224
20638	0.097	-1.181	0.185	0.196	-0.581	0.102	0.295	0.082	0.262
20639	0.224	-0.373	0.142	0.149	-0.882	0.462	0.295	0.082	0.431
20640	0.142	-0.895	0.508	0.272	-0.091	0.422	0.224	-0.373	0.142
20641	0.142	-0.895	0.501	0.371	0.549	0.444	0.261	-0.137	0.097
20642	0.226	-0.356	0.492	0.310	0.153	0.514	0.261	-0.137	0.431
20643	0.226	-0.356	0.185	0.275	-0.073	0.149	0.261	-0.137	0.512
20644	0.097	-1.181	0.512	0.237	-0.317	0.508	0.226	-0.356	0.508
20645	0.355	0.470	0.328	0.345	0.379	0.462	0.229	-0.339	0.508
20646	0.358	0.487	0.224	0.234	-0.336	0.309	0.142	-0.895	0.050
20647	0.142	-0.895	0.508	0.342	0.361	0.053	0.187	-0.608	0.050
20648	0.184	-0.625	0.224	0.342	0.361	0.462	0.187	-0.608	0.492
20649	0.142	-0.895	0.431	0.342	0.361	0.491	0.097	-1.181	0.431
20650	0.142	-0.895	0.328	0.272	-0.091	0.508	0.142	-0.895	0.185
20651	0.187	-0.608	0.492	0.307	0.135	0.501	0.097	-1.181	0.512
20652	0.261	-0.137	0.328	0.310	0.153	0.462	0.261	-0.137	0.512
20653	0.468	1.194	0.467	0.477	1.227	0.508	0.476	1.245	0.501
20654	0.495	1.363	0.467	0.468	1.170	0.371	0.508	1.447	0.481
20655	0.184	-0.625	0.467	0.310	0.153	0.273	0.142	-0.895	0.467
20656	0.184	-0.625	0.467	0.342	0.361	0.398	0.142	-0.895	0.467
20657	0.184	-0.625	0.142	0.237	-0.317	0.398	0.224	-0.373	0.224
20658	0.482	1.279	0.501	0.462	1.133	0.193	0.474	1.228	0.142
20659	0.261	-0.137	0.492	0.339	0.342	0.273	0.050	-1.485	0.481
20660	0.261	-0.137	0.097	0.339	0.342	0.491	0.050	-1.485	0.481
20661	0.476	1.245	0.224	0.491	1.321	0.512	0.489	1.329	0.513
20662	0.261	-0.137	0.224	0.275	-0.073	0.508	0.050	-1.485	0.513
20663	0.508	1.447	0.501	0.465	1.152	0.053	0.511	1.464	0.328
20664	0.142	-0.895	0.513	0.307	0.135	0.273	0.097	-1.181	0.296
20665	0.489	1.329	0.142	0.506	1.415	0.422	0.505	1.430	0.512
20666	0.300	0.116	0.385	0.102	-1.184	0.491	0.358	0.487	0.501
20667	0.513	1.481	0.142	0.456	1.095	0.102	0.503	1.413	0.224
20668	0.508	1.447	0.262	0.424	0.888	0.501	0.476	1.245	0.467
20669	0.508	1.447	0.262	0.444	1.020	0.512	0.479	1.262	0.467
20670	0.497	1.380	0.296	0.518	1.491	0.235	0.511	1.464	0.512
20671	0.097	-1.181	0.492	0.149	-0.882	0.508	0.297	0.099	0.142
20672	0.384	0.655	0.501	0.512	1.453	0.478	0.495	1.363	0.450
20673	0.426	0.925	0.262	0.468	1.170	0.102	0.505	1.430	0.450
20674	0.226	-0.356	0.296	0.310	0.153	0.508	0.295	0.082	0.512
20675	0.187	-0.608	0.262	0.053	-1.504	0.149	0.326	0.285	0.296

20676	0.500	1.397	0.262	0.459	1.114	0.309	0.479	1.262	0.512
20677	0.429	0.942	0.262	0.494	1.340	0.462	0.497	1.380	0.512
20678	0.355	0.470	0.431	0.506	1.415	0.371	0.505	1.430	0.501
20679	0.500	1.397	0.501	0.506	1.415	0.193	0.434	0.975	0.296
20680	0.500	1.397	0.358	0.468	1.170	0.478	0.426	0.925	0.481
20681	0.489	1.329	0.224	0.503	1.396	0.478	0.505	1.430	0.142
20682	0.297	0.099	0.262	0.377	0.587	0.235	0.295	0.082	0.185
20683	0.050	-1.485	0.262	0.275	-0.073	0.512	0.263	-0.120	0.512
20684	0.505	1.430	0.224	0.398	0.718	0.398	0.382	0.638	0.513
20685	0.468	1.194	0.481	0.398	0.718	0.273	0.497	1.380	0.508
20686	0.224	-0.373	0.467	0.193	-0.600	0.514	0.142	-0.895	0.492
20687	0.295	0.082	0.262	0.272	-0.091	0.193	0.224	-0.373	0.097
20688	0.492	1.346	0.185	0.488	1.302	0.193	0.495	1.363	0.185
20689	0.497	1.380	0.501	0.515	1.472	0.512	0.405	0.790	0.050
20690	0.466	1.178	0.296	0.512	1.453	0.235	0.466	1.178	0.262
20691	0.142	-0.895	0.185	0.053	-1.504	0.478	0.326	0.285	0.501
20692	0.297	0.099	0.262	0.272	-0.091	0.512	0.408	0.807	0.512
20693	0.503	1.413	0.450	0.421	0.869	0.235	0.484	1.295	0.512
20694	0.468	1.194	0.450	0.485	1.283	0.512	0.495	1.363	0.385
20695	0.184	-0.625	0.262	0.102	-1.184	0.501	0.295	0.082	0.450
20696	0.492	1.346	0.508	0.500	1.378	0.422	0.513	1.481	0.508
20697	0.497	1.380	0.481	0.512	1.453	0.444	0.513	1.481	0.385
20698	0.097	-1.181	0.262	0.234	-0.336	0.398	0.295	0.082	0.097
20699	0.445	1.043	0.467	0.506	1.415	0.398	0.482	1.279	0.185
20700	0.500	1.397	0.262	0.477	1.227	0.444	0.503	1.413	0.467
20701	0.050	-1.485	0.296	0.102	-1.184	0.102	0.355	0.470	0.296
20702	0.495	1.363	0.508	0.506	1.415	0.501	0.482	1.279	0.508
20703	0.097	-1.181	0.224	0.149	-0.882	0.462	0.297	0.099	0.508
20704	0.329	0.301	0.185	0.313	0.172	0.491	0.224	-0.373	0.508
20705	0.500	1.397	0.501	0.468	1.170	0.341	0.429	0.942	0.512
20706	0.263	-0.120	0.142	0.374	0.568	0.341	0.297	0.099	0.262
20707	0.261	-0.137	0.142	0.193	-0.600	0.341	0.224	-0.373	0.492
20708	0.500	1.397	0.097	0.474	1.208	0.149	0.503	1.413	0.467
20709	0.500	1.397	0.262	0.474	1.208	0.491	0.468	1.194	0.097
20710	0.263	-0.120	0.262	0.234	-0.336	0.491	0.224	-0.373	0.097
20711	0.432	0.958	0.512	0.520	1.509	0.512	0.450	1.076	0.097
20712	0.295	0.082	0.050	0.401	0.737	0.512	0.332	0.318	0.492
20713	0.261	-0.137	0.431	0.272	-0.091	0.478	0.458	1.127	0.481
20714	0.450	1.076	0.501	0.518	1.491	0.501	0.450	1.076	0.328
20715	0.142	-0.895	0.262	0.196	-0.581	0.422	0.261	-0.137	0.224
20716	0.142	-0.895	0.508	0.193	-0.600	0.462	0.224	-0.373	0.358
20717	0.500	1.397	0.508	0.506	1.415	0.235	0.513	1.481	0.262
20718	0.226	-0.356	0.296	0.237	-0.317	0.491	0.355	0.470	0.142
20719	0.495	1.363	0.224	0.480	1.246	0.193	0.500	1.397	0.185
20720	0.513	1.481	0.492	0.515	1.472	0.501	0.482	1.279	0.467
20721	0.097	-1.181	0.492	0.237	-0.317	0.501	0.224	-0.373	0.492
20722	0.466	1.178	0.481	0.509	1.434	0.309	0.408	0.807	0.097
20723	0.482	1.279	0.508	0.500	1.378	0.102	0.489	1.329	0.185
20724	0.450	1.076	0.224	0.421	0.869	0.341	0.511	1.464	0.185
20725	0.505	1.430	0.358	0.444	1.020	0.193	0.476	1.245	0.296
20726	0.505	1.430	0.385	0.398	0.718	0.341	0.476	1.245	0.224
20727	0.505	1.430	0.512	0.398	0.718	0.462	0.476	1.245	0.508
20728	0.508	1.447	0.492	0.444	1.020	0.102	0.411	0.824	0.513
20729	0.297	0.099	0.492	0.102	-1.184	0.462	0.184	-0.625	0.050
20730	0.495	1.363	0.508	0.497	1.359	0.512	0.450	1.076	0.097
20731	0.479	1.262	0.492	0.465	1.152	0.309	0.511	1.464	0.142
20732	0.405	0.790	0.501	0.491	1.321	0.422	0.474	1.228	0.142
20733	0.142	-0.895	0.501	0.342	0.361	0.444	0.184	-0.625	0.262
20734	0.142	-0.895	0.358	0.345	0.379	0.444	0.142	-0.895	0.481
20735	0.142	-0.895	0.185	0.345	0.379	0.193	0.142	-0.895	0.450
20736	0.326	0.285	0.481	0.152	-0.863	0.508	0.142	-0.895	0.296

20737	0.516	1.498	0.467	0.488	1.302	0.422	0.453	1.093	0.142
20738	0.513	1.481	0.513	0.491	1.321	0.149	0.497	1.380	0.492
20739	0.500	1.397	0.431	0.512	1.453	0.193	0.358	0.487	0.050
20740	0.479	1.262	0.492	0.509	1.434	0.193	0.326	0.285	0.185
20741	0.503	1.413	0.224	0.480	1.246	0.508	0.495	1.363	0.224
20742	0.492	1.346	0.512	0.503	1.396	0.235	0.511	1.464	0.508
20743	0.492	1.346	0.262	0.424	0.888	0.341	0.484	1.295	0.508
20744	0.484	1.295	0.358	0.459	1.114	0.462	0.505	1.430	0.050
20745	0.000	?	0.262	0.234	-0.336	0.462	0.295	0.082	0.512
20746	0.500	1.397	0.262	0.488	1.302	0.273	0.508	1.447	0.501
20747	0.142	-0.895	0.467	0.193	-0.600	0.444	0.224	-0.373	0.142
20748	0.513	1.481	0.467	0.497	1.359	0.273	0.503	1.413	0.508
20749	0.479	1.262	0.501	0.497	1.359	0.501	0.476	1.245	0.185
20750	0.263	-0.120	0.185	0.307	0.135	0.309	0.050	-1.485	0.224
20751	0.266	-0.103	0.262	0.102	-1.184	0.149	0.261	-0.137	0.512
20752	0.224	-0.373	0.358	0.342	0.361	0.478	0.184	-0.625	0.224
20753	0.226	-0.356	0.492	0.196	-0.581	0.398	0.295	0.082	0.481
20754	0.097	-1.181	0.501	0.342	0.361	0.478	0.142	-0.895	0.296
20755	0.505	1.430	0.512	0.462	1.133	0.235	0.463	1.161	0.296
20756	0.184	-0.625	0.358	0.102	-1.184	0.235	0.261	-0.137	0.328
20757	0.482	1.279	0.328	0.459	1.114	0.462	0.476	1.245	0.097
20758	0.511	1.464	0.262	0.509	1.434	0.309	0.355	0.470	0.097
20759	0.226	-0.356	0.501	0.310	0.153	0.273	0.184	-0.625	0.492
20760	0.384	0.655	0.481	0.427	0.907	0.512	0.300	0.116	0.142
20761	0.497	1.380	0.512	0.444	1.020	0.462	0.476	1.245	0.385
20762	0.447	1.060	0.097	0.439	0.982	0.444	0.492	1.346	0.512
20763	0.382	0.638	0.224	0.196	-0.581	0.149	0.224	-0.373	0.262
20764	0.447	1.060	0.097	0.506	1.415	0.309	0.497	1.380	0.512
20765	0.484	1.295	0.097	0.421	0.869	0.512	0.511	1.464	0.512
20766	0.295	0.082	0.513	0.149	-0.882	0.501	0.224	-0.373	0.185
20767	0.297	0.099	0.224	0.193	-0.600	0.193	0.142	-0.895	0.142
20768	0.261	-0.137	0.296	0.193	-0.600	0.512	0.142	-0.895	0.385
20769	0.453	1.093	0.185	0.503	1.396	0.422	0.505	1.430	0.385
20770	0.358	0.487	0.450	0.234	-0.336	0.398	0.097	-1.181	0.492
20771	0.300	0.116	0.431	0.342	0.361	0.102	0.297	0.099	0.492
20772	0.453	1.093	0.328	0.465	1.152	0.102	0.450	1.076	0.142
20773	0.447	1.060	0.467	0.465	1.152	0.149	0.508	1.447	0.185
20774	0.097	-1.181	0.513	0.275	-0.073	0.514	0.184	-0.625	0.224
20775	0.482	1.279	0.508	0.447	1.039	0.422	0.463	1.161	0.409
20776	0.295	0.082	0.508	0.275	-0.073	0.462	0.261	-0.137	0.512
20777	0.508	1.447	0.224	0.494	1.340	0.341	0.479	1.262	0.512
20778	0.261	-0.137	0.224	0.310	0.153	0.273	0.261	-0.137	0.492
20779	0.224	-0.373	0.450	0.149	-0.882	0.273	0.184	-0.625	0.467
20780	0.513	1.481	0.508	0.480	1.246	0.193	0.468	1.194	0.501
20781	0.476	1.245	0.224	0.395	0.700	0.193	0.503	1.413	0.501
20782	0.358	0.487	0.328	0.480	1.246	0.501	0.461	1.144	0.050
20783	0.184	-0.625	0.224	0.234	-0.336	0.149	0.142	-0.895	0.224
20784	0.261	-0.137	0.224	0.234	-0.336	0.501	0.142	-0.895	0.501
20785	0.479	1.262	0.224	0.462	1.133	0.501	0.505	1.430	0.513
20786	0.145	-0.878	0.142	0.307	0.135	0.341	0.097	-1.181	0.431
20787	0.263	-0.120	0.481	0.272	-0.091	0.341	0.097	-1.181	0.512
20788	0.508	1.447	0.224	0.509	1.434	0.371	0.489	1.329	0.481
20789	0.466	1.178	0.224	0.459	1.114	0.273	0.503	1.413	0.185
20790	0.492	1.346	0.512	0.447	1.039	0.371	0.463	1.161	0.508
20791	0.184	-0.625	0.492	0.149	-0.882	0.235	0.261	-0.137	0.262
20792	0.329	0.301	0.481	0.313	0.172	0.508	0.184	-0.625	0.262
20793	0.505	1.430	0.492	0.509	1.434	0.501	0.495	1.363	0.185
20794	0.505	1.430	0.328	0.506	1.415	0.512	0.500	1.397	0.185
20795	0.297	0.099	0.097	0.193	-0.600	0.235	0.187	-0.608	0.512
20796	0.382	0.638	0.185	0.512	1.453	0.273	0.387	0.672	0.512
20797	0.508	1.447	0.467	0.424	0.888	0.508	0.518	1.514	0.513

20798	0.511	1.464	0.450	0.401	0.737	0.512	0.482	1.279	0.142
20799	0.261	-0.137	0.512	0.102	-1.184	0.462	0.261	-0.137	0.142
20800	0.332	0.318	0.501	0.102	-1.184	0.309	0.261	-0.137	0.097
20801	0.384	0.655	0.262	0.149	-0.882	0.462	0.097	-1.181	0.508
20802	0.405	0.790	0.501	0.512	1.453	0.193	0.479	1.262	0.467
20803	0.511	1.464	0.492	0.424	0.888	0.501	0.505	1.430	0.450
20804	0.500	1.397	0.501	0.465	1.152	0.514	0.484	1.295	0.050
20805	0.224	-0.373	0.296	0.345	0.379	0.478	0.295	0.082	0.185
20806	0.187	-0.608	0.224	0.275	-0.073	0.149	0.097	-1.181	0.512
20807	0.263	-0.120	0.262	0.272	-0.091	0.149	0.097	-1.181	0.409
20808	0.300	0.116	0.262	0.193	-0.600	0.508	0.097	-1.181	0.431
20809	0.226	-0.356	0.050	0.193	-0.600	0.341	0.184	-0.625	0.097
20810	0.476	1.245	0.224	0.500	1.378	0.273	0.508	1.447	0.224
20811	0.142	-0.895	0.467	0.152	-0.863	0.508	0.387	0.672	0.185
20812	0.511	1.464	0.296	0.497	1.359	0.501	0.503	1.413	0.328
20813	0.466	1.178	0.358	0.497	1.359	0.514	0.489	1.329	0.142
20814	0.097	-1.181	0.512	0.345	0.379	0.422	0.097	-1.181	0.431
20815	0.224	-0.373	0.481	0.345	0.379	0.273	0.263	-0.120	0.097
20816	0.479	1.262	0.512	0.371	0.549	0.273	0.497	1.380	0.262
20817	0.226	-0.356	0.097	0.272	-0.091	0.508	0.295	0.082	0.050
20818	0.447	1.060	0.512	0.371	0.549	0.309	0.468	1.194	0.501
20819	0.050	-1.485	0.513	0.234	-0.336	0.309	0.332	0.318	0.501
20820	0.503	1.413	0.512	0.506	1.415	0.422	0.497	1.380	0.142
20821	0.497	1.380	0.512	0.503	1.396	0.444	0.453	1.093	0.481
20822	0.489	1.329	0.050	0.518	1.491	0.512	0.295	0.082	0.450
20823	0.184	-0.625	0.501	0.234	-0.336	0.508	0.142	-0.895	0.481
20824	0.261	-0.137	0.097	0.275	-0.073	0.508	0.358	0.487	0.492
20825	0.489	1.329	0.508	0.509	1.434	0.508	0.463	1.161	0.224
20826	0.503	1.413	0.185	0.515	1.472	0.235	0.434	0.975	0.185
20827	0.492	1.346	0.142	0.462	1.133	0.398	0.455	1.110	0.512
20828	0.266	-0.103	0.328	0.307	0.135	0.444	0.263	-0.120	0.492
20829	0.511	1.464	0.224	0.456	1.095	0.444	0.497	1.380	0.185
20830	0.224	-0.373	0.185	0.196	-0.581	0.341	0.300	0.116	0.185
20831	0.505	1.430	0.097	0.506	1.415	0.341	0.508	1.447	0.097
20832	0.411	0.824	0.050	0.518	1.491	0.102	0.505	1.430	0.050
20833	0.505	1.430	0.431	0.482	1.265	0.193	0.497	1.380	0.224
20834	0.224	-0.373	0.467	0.149	-0.882	0.398	0.224	-0.373	0.097
20835	0.184	-0.625	0.508	0.149	-0.882	0.478	0.263	-0.120	0.512
20836	0.508	1.447	0.431	0.515	1.472	0.341	0.426	0.925	0.224
20837	0.184	-0.625	0.262	0.345	0.379	0.371	0.142	-0.895	0.224
20838	0.224	-0.373	0.328	0.272	-0.091	0.501	0.097	-1.181	0.508
20839	0.508	1.447	0.358	0.512	1.453	0.478	0.429	0.942	0.481
20840	0.453	1.093	0.431	0.503	1.396	0.235	0.508	1.447	0.492
20841	0.495	1.363	0.262	0.512	1.453	0.508	0.503	1.413	0.467
20842	0.492	1.346	0.050	0.427	0.907	0.512	0.500	1.397	0.185
20843	0.224	-0.373	0.050	0.272	-0.091	0.512	0.050	-1.485	0.385
20844	0.326	0.285	0.224	0.272	-0.091	0.102	0.050	-1.485	0.224
20845	0.466	1.178	0.185	0.506	1.415	0.478	0.489	1.329	0.481
20846	0.261	-0.137	0.328	0.313	0.172	0.444	0.434	0.975	0.508
20847	0.297	0.099	0.481	0.310	0.153	0.444	0.184	-0.625	0.467
20848	0.479	1.262	0.185	0.424	0.888	0.235	0.511	1.464	0.450
20849	0.432	0.958	0.097	0.442	1.001	0.512	0.511	1.464	0.097
20850	0.516	1.498	0.262	0.515	1.472	0.235	0.503	1.413	0.501
20851	0.508	1.447	0.501	0.506	1.415	0.102	0.479	1.262	0.492
20852	0.429	0.942	0.450	0.509	1.434	0.478	0.461	1.144	0.512
20853	0.429	0.942	0.328	0.509	1.434	0.491	0.461	1.144	0.185
20854	0.184	-0.625	0.492	0.234	-0.336	0.514	0.224	-0.373	0.050
20855	0.184	-0.625	0.467	0.398	0.718	0.501	0.142	-0.895	0.050
20856	0.468	1.194	0.467	0.444	1.020	0.309	0.476	1.245	0.097
20857	0.468	1.194	0.512	0.444	1.020	0.235	0.476	1.245	0.142
20858	0.426	0.925	0.224	0.345	0.379	0.309	0.511	1.464	0.097

20859	0.405	0.790	0.492	0.345	0.379	0.309	0.511	1.464	0.501
20860	0.184	-0.625	0.512	0.102	-1.184	0.491	0.329	0.301	0.097
20861	0.142	-0.895	0.481	0.193	-0.600	0.508	0.297	0.099	0.296
20862	0.497	1.380	0.142	0.398	0.718	0.508	0.505	1.430	0.501
20863	0.495	1.363	0.508	0.482	1.265	0.341	0.505	1.430	0.296
20864	0.411	0.824	0.097	0.345	0.379	0.508	0.224	-0.373	0.431
20865	0.495	1.363	0.097	0.374	0.568	0.341	0.466	1.178	0.481
20866	0.500	1.397	0.512	0.497	1.359	0.149	0.463	1.161	0.296
20867	0.474	1.228	0.185	0.482	1.265	0.462	0.513	1.481	0.224
20868	0.184	-0.625	0.224	0.237	-0.317	0.149	0.145	-0.878	0.185
20869	0.476	1.245	0.328	0.509	1.434	0.193	0.445	1.043	0.185
20870	0.508	1.447	0.501	0.506	1.415	0.309	0.450	1.076	0.185
20871	0.479	1.262	0.508	0.512	1.453	0.444	0.505	1.430	0.481
20872	0.187	-0.608	0.296	0.102	-1.184	0.193	0.295	0.082	0.481
20873	0.508	1.447	0.296	0.480	1.246	0.514	0.503	1.413	0.508
20874	0.511	1.464	0.142	0.439	0.982	0.512	0.508	1.447	0.508
20875	0.513	1.481	0.185	0.439	0.982	0.512	0.497	1.380	0.224
20876	0.097	-1.181	0.262	0.234	-0.336	0.514	0.295	0.082	0.481
20877	0.492	1.346	0.481	0.512	1.453	0.371	0.479	1.262	0.185
20878	0.187	-0.608	0.142	0.237	-0.317	0.508	0.142	-0.895	0.513
20879	0.261	-0.137	0.262	0.102	-1.184	0.501	0.187	-0.608	0.224
20880	0.482	1.279	0.481	0.477	1.227	0.371	0.508	1.447	0.262
20881	0.489	1.329	0.481	0.491	1.321	0.235	0.505	1.430	0.185
20882	0.468	1.194	0.492	0.515	1.472	0.235	0.503	1.413	0.262
20883	0.495	1.363	0.492	0.497	1.359	0.501	0.484	1.295	0.262
20884	0.145	-0.878	0.050	0.310	0.153	0.512	0.097	-1.181	0.262
20885	0.142	-0.895	0.097	0.234	-0.336	0.273	0.184	-0.625	0.328
20886	0.184	-0.625	0.262	0.307	0.135	0.235	0.050	-1.485	0.512
20887	0.142	-0.895	0.409	0.307	0.135	0.273	0.266	-0.103	0.501
20888	0.503	1.413	0.492	0.485	1.283	0.102	0.505	1.430	0.508
20889	0.503	1.413	0.450	0.506	1.415	0.193	0.505	1.430	0.508
20890	0.492	1.346	0.097	0.506	1.415	0.193	0.505	1.430	0.385
20891	0.184	-0.625	0.097	0.345	0.379	0.512	0.000	?	0.296
20892	0.513	1.481	0.296	0.503	1.396	0.514	0.382	0.638	0.409
20893	0.332	0.318	0.467	0.342	0.361	0.273	0.000	?	0.409
20894	0.224	-0.373	0.501	0.149	-0.882	0.371	0.455	1.110	0.450
20895	0.500	1.397	0.481	0.459	1.114	0.371	0.513	1.481	0.142
20896	0.224	-0.373	0.492	0.149	-0.882	0.309	0.387	0.672	0.262
20897	0.432	0.958	0.467	0.193	-0.600	0.371	0.097	-1.181	0.097
20898	0.326	0.285	0.224	0.313	0.172	0.478	0.184	-0.625	0.224
20899	0.492	1.346	0.481	0.439	0.982	0.462	0.463	1.161	0.142
20900	0.326	0.285	0.450	0.196	-0.581	0.193	0.295	0.082	0.492
20901	0.466	1.178	0.481	0.515	1.472	0.235	0.516	1.498	0.097
20902	0.466	1.178	0.512	0.515	1.472	0.235	0.516	1.498	0.050
20903	0.466	1.178	0.501	0.515	1.472	0.193	0.516	1.498	0.328
20904	0.466	1.178	0.328	0.515	1.472	0.053	0.516	1.498	0.501
20905	0.142	-0.895	0.328	0.374	0.568	0.491	0.263	-0.120	0.508
20906	0.492	1.346	0.492	0.512	1.453	0.491	0.489	1.329	0.050
20907	0.495	1.363	0.467	0.500	1.378	0.341	0.466	1.178	0.467
20908	0.097	-1.181	0.296	0.371	0.549	0.341	0.097	-1.181	0.492
20909	0.224	-0.373	0.142	0.237	-0.317	0.491	0.097	-1.181	0.492
20910	0.224	-0.373	0.492	0.237	-0.317	0.273	0.097	-1.181	0.508
20911	0.500	1.397	0.409	0.503	1.396	0.309	0.487	1.312	0.050
20912	0.516	1.498	0.467	0.512	1.453	0.235	0.476	1.245	0.512
20913	0.142	-0.895	0.224	0.275	-0.073	0.462	0.326	0.285	0.513
20914	0.384	0.655	0.450	0.234	-0.336	0.462	0.226	-0.356	0.501
20915	0.142	-0.895	0.262	0.275	-0.073	0.309	0.358	0.487	0.262
20916	0.263	-0.120	0.328	0.102	-1.184	0.501	0.263	-0.120	0.409
20917	0.263	-0.120	0.262	0.193	-0.600	0.478	0.142	-0.895	0.358
20918	0.300	0.116	0.492	0.193	-0.600	0.341	0.184	-0.625	0.142
20919	0.492	1.346	0.142	0.512	1.453	0.478	0.461	1.144	0.513

20920	0.503	1.413	0.142	0.515	1.472	0.193	0.487	1.312	0.185
20921	0.263	-0.120	0.501	0.272	-0.091	0.273	0.097	-1.181	0.142
20922	0.295	0.082	0.508	0.371	0.549	0.508	0.184	-0.625	0.409
20923	0.261	-0.137	0.142	0.371	0.549	0.341	0.184	-0.625	0.450
20924	0.261	-0.137	0.328	0.307	0.135	0.149	0.295	0.082	0.492
20925	0.261	-0.137	0.492	0.368	0.530	0.478	0.224	-0.373	0.142
20926	0.429	0.942	0.142	0.480	1.246	0.149	0.500	1.397	0.142
20927	0.505	1.430	0.481	0.462	1.133	0.149	0.513	1.481	0.358
20928	0.361	0.504	0.481	0.193	-0.600	0.149	0.050	-1.485	0.142
20929	0.226	-0.356	0.142	0.237	-0.317	0.341	0.097	-1.181	0.262
20930	0.263	-0.120	0.467	0.237	-0.317	0.341	0.142	-0.895	0.512
20931	0.263	-0.120	0.467	0.196	-0.581	0.508	0.142	-0.895	0.185
20932	0.226	-0.356	0.431	0.053	-1.504	0.371	0.263	-0.120	0.262
20933	0.476	1.245	0.328	0.488	1.302	0.478	0.482	1.279	0.512
20934	0.463	1.161	0.097	0.488	1.302	0.478	0.453	1.093	0.467
20935	0.261	-0.137	0.097	0.345	0.379	0.053	0.295	0.082	0.508
20936	0.184	-0.625	0.262	0.342	0.361	0.398	0.142	-0.895	0.467
20937	0.500	1.397	0.224	0.497	1.359	0.398	0.492	1.346	0.185
20938	0.297	0.099	0.385	0.272	-0.091	0.273	0.050	-1.485	0.097
20939	0.184	-0.625	0.224	0.307	0.135	0.235	0.184	-0.625	0.097
20940	0.266	-0.103	0.097	0.237	-0.317	0.501	0.224	-0.373	0.512
20941	0.447	1.060	0.097	0.462	1.133	0.501	0.503	1.413	0.513
20942	0.447	1.060	0.501	0.462	1.133	0.462	0.503	1.413	0.492
20943	0.263	-0.120	0.296	0.310	0.153	0.462	0.050	-1.485	0.224
20944	0.503	1.413	0.296	0.500	1.378	0.193	0.508	1.447	0.385
20945	0.482	1.279	0.224	0.482	1.265	0.193	0.497	1.380	0.185
20946	0.263	-0.120	0.185	0.345	0.379	0.053	0.142	-0.895	0.513
20947	0.463	1.161	0.185	0.480	1.246	0.491	0.508	1.447	0.512
20948	0.261	-0.137	0.481	0.193	-0.600	0.444	0.187	-0.608	0.492
20949	0.297	0.099	0.224	0.272	-0.091	0.444	0.224	-0.373	0.467
20950	0.505	1.430	0.224	0.506	1.415	0.508	0.508	1.447	0.450
20951	0.224	-0.373	0.385	0.342	0.361	0.491	0.000	?	0.224
20952	0.184	-0.625	0.481	0.149	-0.882	0.508	0.224	-0.373	0.508
20953	0.503	1.413	0.513	0.477	1.227	0.508	0.476	1.245	0.512
20954	0.145	-0.878	0.481	0.149	-0.882	0.149	0.295	0.082	0.431
20955	0.145	-0.878	0.262	0.149	-0.882	0.309	0.295	0.082	0.501
20956	0.097	-1.181	0.481	0.149	-0.882	0.512	0.329	0.301	0.450
20957	0.261	-0.137	0.262	0.342	0.361	0.491	0.100	-1.164	0.409
20958	0.261	-0.137	0.296	0.342	0.361	0.501	0.100	-1.164	0.409
20959	0.508	1.447	0.467	0.509	1.434	0.462	0.487	1.312	0.512
20960	0.050	-1.485	0.142	0.374	0.568	0.478	0.142	-0.895	0.492
20961	0.426	0.925	0.467	0.471	1.189	0.149	0.384	0.655	0.185
20962	0.500	1.397	0.512	0.477	1.227	0.491	0.508	1.447	0.142
20963	0.261	-0.137	0.296	0.053	-1.504	0.102	0.261	-0.137	0.296
20964	0.511	1.464	0.508	0.401	0.737	0.149	0.505	1.430	0.501
20965	0.511	1.464	0.513	0.401	0.737	0.149	0.505	1.430	0.501
20966	0.295	0.082	0.481	0.275	-0.073	0.149	0.184	-0.625	0.512
20967	0.226	-0.356	0.224	0.234	-0.336	0.444	0.142	-0.895	0.262
20968	0.495	1.363	0.097	0.500	1.378	0.444	0.384	0.655	0.512
20969	0.495	1.363	0.501	0.500	1.378	0.444	0.384	0.655	0.097
20970	0.479	1.262	0.481	0.459	1.114	0.273	0.489	1.329	0.097
20971	0.503	1.413	0.508	0.462	1.133	0.273	0.489	1.329	0.224
20972	0.226	-0.356	0.097	0.193	-0.600	0.149	0.142	-0.895	0.492
20973	0.358	0.487	0.296	0.193	-0.600	0.501	0.184	-0.625	0.328
20974	0.387	0.672	0.224	0.053	-1.504	0.462	0.224	-0.373	0.328
20975	0.511	1.464	0.185	0.488	1.302	0.478	0.405	0.790	0.513
20976	0.495	1.363	0.224	0.442	1.001	0.102	0.503	1.413	0.450
20977	0.495	1.363	0.262	0.442	1.001	0.102	0.503	1.413	0.185
20978	0.503	1.413	0.481	0.506	1.415	0.341	0.492	1.346	0.142
20979	0.489	1.329	0.501	0.491	1.321	0.491	0.466	1.178	0.508
20980	0.495	1.363	0.492	0.509	1.434	0.512	0.508	1.447	0.097

20981	0.495	1.363	0.296	0.509	1.434	0.508	0.508	1.447	0.481
20982	0.355	0.470	0.224	0.152	-0.863	0.398	0.050	-1.485	0.481
20983	0.184	-0.625	0.512	0.310	0.153	0.193	0.224	-0.373	0.185
20984	0.482	1.279	0.508	0.509	1.434	0.398	0.503	1.413	0.481
20985	0.463	1.161	0.512	0.491	1.321	0.273	0.511	1.464	0.481
20986	0.511	1.464	0.185	0.503	1.396	0.309	0.426	0.925	0.431
20987	0.429	0.942	0.185	0.468	1.170	0.444	0.513	1.481	0.262
20988	0.492	1.346	0.224	0.480	1.246	0.235	0.479	1.262	0.358
20989	0.224	-0.373	0.385	0.149	-0.882	0.102	0.187	-0.608	0.385
20990	0.511	1.464	0.501	0.494	1.340	0.309	0.505	1.430	0.142
20991	0.266	-0.103	0.385	0.102	-1.184	0.491	0.261	-0.137	0.296
20992	0.363	0.520	0.358	0.149	-0.882	0.514	0.261	-0.137	0.142
20993	0.261	-0.137	0.358	0.149	-0.882	0.235	0.184	-0.625	0.224
20994	0.261	-0.137	0.385	0.149	-0.882	0.478	0.184	-0.625	0.296
20995	0.463	1.161	0.185	0.442	1.001	0.478	0.508	1.447	0.296
20996	0.463	1.161	0.409	0.442	1.001	0.514	0.508	1.447	0.508
20997	0.497	1.380	0.492	0.442	1.001	0.514	0.511	1.464	0.097
20998	0.187	-0.608	0.328	0.275	-0.073	0.102	0.142	-0.895	0.185
20999	0.261	-0.137	0.296	0.278	-0.054	0.193	0.142	-0.895	0.185
21000	0.361	0.504	0.296	0.149	-0.882	0.462	0.097	-1.181	0.185
21001	0.484	1.295	0.467	0.503	1.396	0.508	0.505	1.430	0.185
21002	0.503	1.413	0.481	0.468	1.170	0.193	0.466	1.178	0.142
21003	0.511	1.464	0.492	0.474	1.208	0.341	0.447	1.060	0.508
21004	0.358	0.487	0.450	0.102	-1.184	0.309	0.050	-1.485	0.185
21005	0.326	0.285	0.328	0.102	-1.184	0.273	0.184	-0.625	0.185
21006	0.261	-0.137	0.328	0.342	0.361	0.102	0.000	?	0.501
21007	0.503	1.413	0.097	0.488	1.302	0.341	0.513	1.481	0.508
21008	0.482	1.279	0.431	0.509	1.434	0.478	0.405	0.790	0.512
21009	0.500	1.397	0.467	0.509	1.434	0.102	0.432	0.958	0.501
21010	0.097	-1.181	0.467	0.398	0.718	0.102	0.097	-1.181	0.142
21011	0.226	-0.356	0.296	0.193	-0.600	0.478	0.224	-0.373	0.481
21012	0.097	-1.181	0.296	0.398	0.718	0.501	0.184	-0.625	0.142
21013	0.000	?	0.501	0.272	-0.091	0.422	0.332	0.318	0.142
21014	0.097	-1.181	0.492	0.310	0.153	0.508	0.142	-0.895	0.501
21015	0.513	1.481	0.467	0.444	1.020	0.309	0.432	0.958	0.097
21016	0.226	-0.356	0.224	0.237	-0.317	0.273	0.097	-1.181	0.492
21017	0.297	0.099	0.097	0.102	-1.184	0.341	0.261	-0.137	0.492
21018	0.184	-0.625	0.328	0.307	0.135	0.491	0.050	-1.485	0.050
21019	0.450	1.076	0.224	0.494	1.340	0.508	0.500	1.397	0.431
21020	0.432	0.958	0.512	0.515	1.472	0.491	0.500	1.397	0.467
21021	0.329	0.301	0.508	0.234	-0.336	0.462	0.142	-0.895	0.481
21022	0.468	1.194	0.492	0.474	1.208	0.235	0.476	1.245	0.050
21023	0.511	1.464	0.097	0.477	1.227	0.501	0.450	1.076	0.185
21024	0.508	1.447	0.262	0.518	1.491	0.491	0.476	1.245	0.481
21025	0.508	1.447	0.224	0.518	1.491	0.501	0.487	1.312	0.512
21026	0.226	-0.356	0.501	0.102	-1.184	0.478	0.224	-0.373	0.481
21027	0.224	-0.373	0.142	0.193	-0.600	0.273	0.187	-0.608	0.142
21028	0.453	1.093	0.358	0.456	1.095	0.235	0.505	1.430	0.296
21029	0.505	1.430	0.185	0.503	1.396	0.235	0.489	1.329	0.328
21030	0.224	-0.373	0.224	0.193	-0.600	0.398	0.184	-0.625	0.142
21031	0.326	0.285	0.328	0.345	0.379	0.273	0.184	-0.625	0.097
21032	0.224	-0.373	0.467	0.313	0.172	0.508	0.097	-1.181	0.050
21033	0.224	-0.373	0.224	0.275	-0.073	0.235	0.050	-1.485	0.501
21034	0.224	-0.373	0.185	0.102	-1.184	0.273	0.224	-0.373	0.501
21035	0.142	-0.895	0.328	0.342	0.361	0.512	0.097	-1.181	0.501
21036	0.476	1.245	0.296	0.477	1.227	0.444	0.511	1.464	0.185
21037	0.226	-0.356	0.358	0.102	-1.184	0.501	0.226	-0.356	0.185
21038	0.226	-0.356	0.142	0.102	-1.184	0.422	0.226	-0.356	0.450
21039	0.505	1.430	0.262	0.480	1.246	0.398	0.505	1.430	0.512
21040	0.487	1.312	0.508	0.500	1.378	0.341	0.479	1.262	0.409
21041	0.476	1.245	0.467	0.421	0.869	0.398	0.492	1.346	0.358

21042	0.487	1.312	0.481	0.509	1.434	0.398	0.463	1.161	0.409
21043	0.329	0.301	0.296	0.313	0.172	0.422	0.184	-0.625	0.224
21044	0.097	-1.181	0.431	0.272	-0.091	0.478	0.387	0.672	0.501
21045	0.187	-0.608	0.409	0.342	0.361	0.398	0.226	-0.356	0.409
21046	0.463	1.161	0.224	0.494	1.340	0.462	0.476	1.245	0.512
21047	0.450	1.076	0.492	0.512	1.453	0.422	0.508	1.447	0.185
21048	0.513	1.481	0.492	0.494	1.340	0.273	0.468	1.194	0.185
21049	0.503	1.413	0.224	0.459	1.114	0.478	0.447	1.060	0.508
21050	0.261	-0.137	0.185	0.237	-0.317	0.309	0.097	-1.181	0.501
21051	0.505	1.430	0.224	0.503	1.396	0.508	0.500	1.397	0.224
21052	0.487	1.312	0.513	0.488	1.302	0.309	0.497	1.380	0.385
21053	0.495	1.363	0.142	0.503	1.396	0.193	0.516	1.498	0.508
21054	0.295	0.082	0.481	0.480	1.246	0.309	0.184	-0.625	0.185
21055	0.224	-0.373	0.508	0.272	-0.091	0.235	0.050	-1.485	0.185
21056	0.263	-0.120	0.185	0.234	-0.336	0.235	0.050	-1.485	0.501
21057	0.263	-0.120	0.185	0.234	-0.336	0.422	0.097	-1.181	0.481
21058	0.050	-1.485	0.224	0.401	0.737	0.273	0.142	-0.895	0.409
21059	0.224	-0.373	0.328	0.275	-0.073	0.398	0.097	-1.181	0.508
21060	0.466	1.178	0.501	0.509	1.434	0.462	0.492	1.346	0.097
21061	0.297	0.099	0.501	0.237	-0.317	0.422	0.097	-1.181	0.097
21062	0.358	0.487	0.050	0.275	-0.073	0.491	0.300	0.116	0.296
21063	0.511	1.464	0.185	0.512	1.453	0.193	0.503	1.413	0.512
21064	0.000	?	0.097	0.444	1.020	0.422	0.295	0.082	0.512
21065	0.474	1.228	0.262	0.503	1.396	0.193	0.429	0.942	0.467
21066	0.503	1.413	0.262	0.427	0.907	0.512	0.482	1.279	0.262
21067	0.097	-1.181	0.224	0.398	0.718	0.309	0.297	0.099	0.262
21068	0.000	?	0.262	0.342	0.361	0.371	0.224	-0.373	0.467
21069	0.000	?	0.296	0.401	0.737	0.235	0.184	-0.625	0.512
21070	0.000	?	0.492	0.401	0.737	0.273	0.184	-0.625	0.508
21071	0.000	?	0.492	0.424	0.888	0.149	0.184	-0.625	0.142
21072	0.513	1.481	0.262	0.477	1.227	0.193	0.476	1.245	0.142
21073	0.516	1.498	0.409	0.395	0.700	0.491	0.476	1.245	0.224
21074	0.505	1.430	0.050	0.465	1.152	0.309	0.503	1.413	0.262
21075	0.505	1.430	0.512	0.424	0.888	0.371	0.503	1.413	0.431
21076	0.050	-1.485	0.481	0.272	-0.091	0.193	0.224	-0.373	0.262
21077	0.495	1.363	0.224	0.477	1.227	0.508	0.482	1.279	0.513
21078	0.097	-1.181	0.358	0.307	0.135	0.478	0.184	-0.625	0.358
21079	0.503	1.413	0.224	0.509	1.434	0.235	0.513	1.481	0.185
21080	0.184	-0.625	0.097	0.310	0.153	0.478	0.226	-0.356	0.097
21081	0.142	-0.895	0.467	0.193	-0.600	0.462	0.261	-0.137	0.385
21082	0.326	0.285	0.185	0.310	0.153	0.512	0.184	-0.625	0.501
21083	0.224	-0.373	0.508	0.234	-0.336	0.444	0.261	-0.137	0.097
21084	0.184	-0.625	0.224	0.234	-0.336	0.309	0.261	-0.137	0.142
21085	0.097	-1.181	0.050	0.421	0.869	0.478	0.261	-0.137	0.262
21086	0.050	-1.485	0.097	0.272	-0.091	0.512	0.329	0.301	0.262
21087	0.426	0.925	0.508	0.398	0.718	0.501	0.511	1.464	0.142
21088	0.468	1.194	0.431	0.462	1.133	0.422	0.497	1.380	0.467
21089	0.511	1.464	0.224	0.424	0.888	0.422	0.500	1.397	0.262
21090	0.432	0.958	0.492	0.488	1.302	0.053	0.500	1.397	0.142
21091	0.261	-0.137	0.450	0.196	-0.581	0.341	0.382	0.638	0.097
21092	0.326	0.285	0.512	0.427	0.907	0.478	0.300	0.116	0.185
21093	0.361	0.504	0.450	0.193	-0.600	0.193	0.408	0.807	0.492
21094	0.426	0.925	0.142	0.515	1.472	0.235	0.411	0.824	0.262
21095	0.263	-0.120	0.508	0.310	0.153	0.341	0.450	1.076	0.185
21096	0.050	-1.485	0.142	0.371	0.549	0.478	0.142	-0.895	0.450
21097	0.050	-1.485	0.481	0.234	-0.336	0.422	0.261	-0.137	0.409
21098	0.224	-0.373	0.262	0.275	-0.073	0.102	0.100	-1.164	0.409
21099	0.184	-0.625	0.262	0.149	-0.882	0.102	0.226	-0.356	0.097
21100	0.332	0.318	0.328	0.193	-0.600	0.273	0.142	-0.895	0.513
21101	0.482	1.279	0.262	0.491	1.321	0.235	0.487	1.312	0.513
21102	0.184	-0.625	0.492	0.313	0.172	0.309	0.097	-1.181	0.185

21103	0.097	-1.181	0.142	0.374	0.568	0.422	0.050	-1.485	0.512
21104	0.261	-0.137	0.385	0.193	-0.600	0.273	0.326	0.285	0.512
21105	0.497	1.380	0.097	0.506	1.415	0.309	0.497	1.380	0.185
21106	0.450	1.076	0.492	0.477	1.227	0.478	0.508	1.447	0.262
21107	0.329	0.301	0.492	0.234	-0.336	0.491	0.050	-1.485	0.185
21108	0.489	1.329	0.224	0.477	1.227	0.508	0.466	1.178	0.409
21109	0.463	1.161	0.481	0.462	1.133	0.491	0.489	1.329	0.224
21110	0.466	1.178	0.296	0.515	1.472	0.309	0.484	1.295	0.501
21111	0.508	1.447	0.142	0.439	0.982	0.341	0.508	1.447	0.409
21112	0.226	-0.356	0.185	0.307	0.135	0.235	0.050	-1.485	0.142
21113	0.489	1.329	0.481	0.477	1.227	0.491	0.513	1.481	0.142
21114	0.505	1.430	0.492	0.509	1.434	0.501	0.513	1.481	0.142
21115	0.474	1.228	0.358	0.503	1.396	0.478	0.500	1.397	0.142
21116	0.142	-0.895	0.450	0.427	0.907	0.235	0.261	-0.137	0.501
21117	0.508	1.447	0.501	0.427	0.907	0.309	0.408	0.807	0.492
21118	0.100	-1.164	0.501	0.053	-1.504	0.235	0.358	0.487	0.142
21119	0.097	-1.181	0.512	0.339	0.342	0.508	0.142	-0.895	0.224
21120	0.508	1.447	0.185	0.480	1.246	0.444	0.511	1.464	0.262
21121	0.184	-0.625	0.142	0.193	-0.600	0.478	0.184	-0.625	0.142
21122	0.224	-0.373	0.262	0.234	-0.336	0.501	0.142	-0.895	0.142
21123	0.329	0.301	0.385	0.345	0.379	0.491	0.408	0.807	0.262
21124	0.503	1.413	0.185	0.482	1.265	0.149	0.445	1.043	0.224
21125	0.503	1.413	0.296	0.424	0.888	0.371	0.489	1.329	0.224
21126	0.295	0.082	0.431	0.102	-1.184	0.462	0.142	-0.895	0.512
21127	0.295	0.082	0.450	0.102	-1.184	0.444	0.142	-0.895	0.501
21128	0.142	-0.895	0.467	0.272	-0.091	0.491	0.358	0.487	0.097
21129	0.184	-0.625	0.492	0.234	-0.336	0.309	0.142	-0.895	0.501
21130	0.261	-0.137	0.467	0.307	0.135	0.235	0.263	-0.120	0.185
21131	0.482	1.279	0.508	0.424	0.888	0.235	0.508	1.447	0.512
21132	0.142	-0.895	0.501	0.275	-0.073	0.341	0.184	-0.625	0.508
21133	0.261	-0.137	0.185	0.310	0.153	0.501	0.263	-0.120	0.142
21134	0.479	1.262	0.185	0.477	1.227	0.102	0.508	1.447	0.358
21135	0.479	1.262	0.185	0.491	1.321	0.273	0.466	1.178	0.142
21136	0.492	1.346	0.185	0.509	1.434	0.422	0.505	1.430	0.262
21137	0.489	1.329	0.224	0.488	1.302	0.514	0.463	1.161	0.492
21138	0.050	-1.485	0.262	0.307	0.135	0.462	0.184	-0.625	0.185
21139	0.097	-1.181	0.097	0.339	0.342	0.501	0.097	-1.181	0.492
21140	0.263	-0.120	0.185	0.234	-0.336	0.053	0.097	-1.181	0.224
21141	0.408	0.807	0.097	0.488	1.302	0.491	0.508	1.447	0.185
21142	0.487	1.312	0.328	0.506	1.415	0.193	0.508	1.447	0.224
21143	0.447	1.060	0.185	0.480	1.246	0.235	0.489	1.329	0.512
21144	0.097	-1.181	0.467	0.240	-0.299	0.273	0.224	-0.373	0.492
21145	0.097	-1.181	0.467	0.307	0.135	0.508	0.384	0.655	0.262
21146	0.300	0.116	0.224	0.234	-0.336	0.371	0.184	-0.625	0.508
21147	0.471	1.211	0.358	0.500	1.378	0.371	0.508	1.447	0.450
21148	0.497	1.380	0.513	0.442	1.001	0.478	0.511	1.464	0.492
21149	0.479	1.262	0.512	0.474	1.208	0.514	0.492	1.346	0.512
21150	0.487	1.312	0.409	0.506	1.415	0.341	0.471	1.211	0.185
21151	0.463	1.161	0.508	0.488	1.302	0.102	0.447	1.060	0.385
21152	0.226	-0.356	0.296	0.149	-0.882	0.462	0.224	-0.373	0.185
21153	0.482	1.279	0.467	0.371	0.549	0.309	0.500	1.397	0.492
21154	0.453	1.093	0.262	0.462	1.133	0.478	0.513	1.481	0.328
21155	0.476	1.245	0.512	0.444	1.020	0.501	0.429	0.942	0.097
21156	0.508	1.447	0.224	0.491	1.321	0.309	0.497	1.380	0.224
21157	0.495	1.363	0.481	0.307	0.135	0.491	0.453	1.093	0.328
21158	0.329	0.301	0.097	0.237	-0.317	0.501	0.408	0.807	0.492
21159	0.329	0.301	0.262	0.237	-0.317	0.491	0.408	0.807	0.185
21160	0.497	1.380	0.450	0.345	0.379	0.102	0.511	1.464	0.358
21161	0.471	1.211	0.142	0.506	1.415	0.053	0.492	1.346	0.296
21162	0.297	0.099	0.224	0.102	-1.184	0.053	0.187	-0.608	0.508
21163	0.142	-0.895	0.481	0.272	-0.091	0.149	0.142	-0.895	0.512

21164	0.000	?	0.467	0.421	0.869	0.371	0.297	0.099	0.185
21165	0.489	1.329	0.450	0.509	1.434	0.341	0.503	1.413	0.508
21166	0.408	0.807	0.431	0.462	1.133	0.193	0.497	1.380	0.185
21167	0.463	1.161	0.185	0.497	1.359	0.235	0.508	1.447	0.224
21168	0.226	-0.356	0.492	0.053	-1.504	0.235	0.263	-0.120	0.328
21169	0.447	1.060	0.508	0.482	1.265	0.508	0.513	1.481	0.512
21170	0.263	-0.120	0.142	0.196	-0.581	0.149	0.097	-1.181	0.508
21171	0.329	0.301	0.512	0.234	-0.336	0.149	0.097	-1.181	0.328
21172	0.263	-0.120	0.142	0.275	-0.073	0.149	0.226	-0.356	0.385
21173	0.495	1.363	0.097	0.509	1.434	0.193	0.489	1.329	0.481
21174	0.142	-0.895	0.450	0.371	0.549	0.193	0.332	0.318	0.481
21175	0.142	-0.895	0.467	0.371	0.549	0.371	0.332	0.318	0.512
21176	0.505	1.430	0.501	0.474	1.208	0.491	0.505	1.430	0.185
21177	0.508	1.447	0.481	0.512	1.453	0.341	0.447	1.060	0.296
21178	0.142	-0.895	0.185	0.345	0.379	0.341	0.184	-0.625	0.142
21179	0.329	0.301	0.185	0.102	-1.184	0.491	0.142	-0.895	0.185
21180	0.489	1.329	0.512	0.465	1.152	0.444	0.503	1.413	0.328
21181	0.142	-0.895	0.492	0.310	0.153	0.478	0.097	-1.181	0.185
21182	0.479	1.262	0.501	0.477	1.227	0.398	0.479	1.262	0.142
21183	0.476	1.245	0.481	0.497	1.359	0.273	0.479	1.262	0.481
21184	0.142	-0.895	0.431	0.307	0.135	0.444	0.184	-0.625	0.481
21185	0.466	1.178	0.481	0.488	1.302	0.371	0.476	1.245	0.481
21186	0.468	1.194	0.492	0.500	1.378	0.102	0.476	1.245	0.512
21187	0.429	0.942	0.513	0.491	1.321	0.398	0.426	0.925	0.513
21188	0.332	0.318	0.358	0.102	-1.184	0.309	0.261	-0.137	0.431
21189	0.097	-1.181	0.512	0.053	-1.504	0.514	0.355	0.470	0.492
21190	0.097	-1.181	0.492	0.053	-1.504	0.491	0.382	0.638	0.142
21191	0.263	-0.120	0.142	0.149	-0.882	0.273	0.142	-0.895	0.142
21192	0.224	-0.373	0.512	0.368	0.530	0.478	0.297	0.099	0.142
21193	0.387	0.672	0.409	0.342	0.361	0.398	0.142	-0.895	0.142
21194	0.224	-0.373	0.296	0.193	-0.600	0.508	0.224	-0.373	0.262
21195	0.097	-1.181	0.450	0.234	-0.336	0.309	0.295	0.082	0.262
21196	0.097	-1.181	0.185	0.234	-0.336	0.273	0.295	0.082	0.224
21197	0.495	1.363	0.513	0.506	1.415	0.398	0.508	1.447	0.097
21198	0.295	0.082	0.097	0.149	-0.882	0.422	0.097	-1.181	0.185
21199	0.295	0.082	0.431	0.149	-0.882	0.508	0.187	-0.608	0.501
21200	0.224	-0.373	0.508	0.149	-0.882	0.371	0.184	-0.625	0.097
21201	0.184	-0.625	0.508	0.196	-0.581	0.398	0.184	-0.625	0.481
21202	0.184	-0.625	0.262	0.196	-0.581	0.341	0.184	-0.625	0.431
21203	0.000	?	0.185	0.368	0.530	0.235	0.142	-0.895	0.142
21204	0.484	1.295	0.450	0.488	1.302	0.273	0.500	1.397	0.508
21205	0.224	-0.373	0.142	0.345	0.379	0.273	0.187	-0.608	0.328
21206	0.224	-0.373	0.262	0.345	0.379	0.512	0.187	-0.608	0.431
21207	0.384	0.655	0.467	0.488	1.302	0.512	0.495	1.363	0.481
21208	0.484	1.295	0.296	0.442	1.001	0.512	0.508	1.447	0.450
21209	0.516	1.498	0.513	0.474	1.208	0.341	0.513	1.481	0.050
21210	0.479	1.262	0.296	0.401	0.737	0.273	0.505	1.430	0.492
21211	0.261	-0.137	0.492	0.275	-0.073	0.444	0.145	-0.878	0.409
21212	0.484	1.295	0.467	0.442	1.001	0.398	0.476	1.245	0.450
21213	0.261	-0.137	0.224	0.374	0.568	0.514	0.145	-0.878	0.296
21214	0.297	0.099	0.481	0.102	-1.184	0.053	0.142	-0.895	0.481
21215	0.468	1.194	0.142	0.398	0.718	0.102	0.492	1.346	0.262
21216	0.142	-0.895	0.467	0.313	0.172	0.149	0.097	-1.181	0.224
21217	0.463	1.161	0.481	0.515	1.472	0.491	0.492	1.346	0.492
21218	0.508	1.447	0.385	0.494	1.340	0.491	0.484	1.295	0.296
21219	0.297	0.099	0.296	0.275	-0.073	0.491	0.050	-1.485	0.262
21220	0.503	1.413	0.481	0.477	1.227	0.193	0.426	0.925	0.492
21221	0.511	1.464	0.142	0.395	0.700	0.193	0.463	1.161	0.481
21222	0.479	1.262	0.385	0.506	1.415	0.422	0.479	1.262	0.492
21223	0.224	-0.373	0.262	0.307	0.135	0.193	0.050	-1.485	0.492
21224	0.097	-1.181	0.481	0.275	-0.073	0.341	0.187	-0.608	0.224

21225	0.500	1.397	0.481	0.395	0.700	0.508	0.479	1.262	0.501
21226	0.474	1.228	0.513	0.430	0.926	0.444	0.513	1.481	0.501
21227	0.500	1.397	0.185	0.518	1.491	0.149	0.476	1.245	0.262
21228	0.097	-1.181	0.224	0.374	0.568	0.149	0.142	-0.895	0.513
21229	0.297	0.099	0.450	0.401	0.737	0.422	0.297	0.099	0.224
21230	0.224	-0.373	0.409	0.342	0.361	0.508	0.326	0.285	0.185
21231	0.184	-0.625	0.142	0.237	-0.317	0.501	0.142	-0.895	0.492
21232	0.224	-0.373	0.262	0.272	-0.091	0.462	0.097	-1.181	0.492
21233	0.263	-0.120	0.097	0.272	-0.091	0.371	0.050	-1.485	0.512
21234	0.479	1.262	0.142	0.518	1.491	0.371	0.495	1.363	0.513
21235	0.497	1.380	0.296	0.518	1.491	0.149	0.495	1.363	0.262
21236	0.489	1.329	0.224	0.518	1.491	0.501	0.495	1.363	0.097
21237	0.297	0.099	0.224	0.342	0.361	0.444	0.184	-0.625	0.385
21238	0.226	-0.356	0.097	0.272	-0.091	0.491	0.184	-0.625	0.501
21239	0.511	1.464	0.097	0.444	1.020	0.309	0.450	1.076	0.481
21240	0.497	1.380	0.508	0.401	0.737	0.309	0.516	1.498	0.385
21241	0.508	1.447	0.481	0.518	1.491	0.462	0.411	0.824	0.513
21242	0.184	-0.625	0.481	0.053	-1.504	0.508	0.358	0.487	0.492
21243	0.184	-0.625	0.296	0.102	-1.184	0.491	0.411	0.824	0.508
21244	0.224	-0.373	0.492	0.149	-0.882	0.341	0.224	-0.373	0.513
21245	0.384	0.655	0.431	0.491	1.321	0.309	0.497	1.380	0.296
21246	0.497	1.380	0.481	0.491	1.321	0.149	0.408	0.807	0.481
21247	0.384	0.655	0.262	0.488	1.302	0.398	0.508	1.447	0.431
21248	0.358	0.487	0.508	0.193	-0.600	0.508	0.184	-0.625	0.296
21249	0.358	0.487	0.492	0.193	-0.600	0.273	0.184	-0.625	0.481
21250	0.387	0.672	0.142	0.418	0.850	0.444	0.503	1.413	0.409
21251	0.187	-0.608	0.185	0.193	-0.600	0.512	0.503	1.413	0.097
21252	0.411	0.824	0.501	0.342	0.361	0.273	0.224	-0.373	0.508
21253	0.492	1.346	0.508	0.506	1.415	0.193	0.382	0.638	0.224
21254	0.326	0.285	0.512	0.442	1.001	0.193	0.503	1.413	0.501
21255	0.295	0.082	0.492	0.149	-0.882	0.422	0.184	-0.625	0.097
21256	0.295	0.082	0.501	0.149	-0.882	0.309	0.184	-0.625	0.508
21257	0.471	1.211	0.492	0.421	0.869	0.193	0.497	1.380	0.501
21258	0.479	1.262	0.508	0.506	1.415	0.149	0.482	1.279	0.501
21259	0.492	1.346	0.385	0.500	1.378	0.149	0.405	0.790	0.097
21260	0.447	1.060	0.409	0.459	1.114	0.273	0.508	1.447	0.142
21261	0.329	0.301	0.262	0.374	0.568	0.508	0.097	-1.181	0.501
21262	0.329	0.301	0.358	0.374	0.568	0.341	0.097	-1.181	0.262
21263	0.100	-1.164	0.467	0.152	-0.863	0.273	0.295	0.082	0.142
21264	0.429	0.942	0.262	0.497	1.359	0.273	0.513	1.481	0.467
21265	0.468	1.194	0.296	0.442	1.001	0.309	0.513	1.481	0.142
21266	0.468	1.194	0.492	0.491	1.321	0.508	0.471	1.211	0.501
21267	0.295	0.082	0.467	0.307	0.135	0.235	0.263	-0.120	0.224
21268	0.295	0.082	0.481	0.307	0.135	0.193	0.263	-0.120	0.492
21269	0.503	1.413	0.467	0.462	1.133	0.514	0.468	1.194	0.508
21270	0.492	1.346	0.185	0.512	1.453	0.491	0.511	1.464	0.185
21271	0.463	1.161	0.185	0.491	1.321	0.491	0.505	1.430	0.450
21272	0.226	-0.356	0.185	0.342	0.361	0.273	0.142	-0.895	0.262
21273	0.097	-1.181	0.296	0.307	0.135	0.512	0.142	-0.895	0.512
21274	0.334	0.335	0.431	0.149	-0.882	0.501	0.224	-0.373	0.508
21275	0.224	-0.373	0.224	0.401	0.737	0.193	0.261	-0.137	0.512
21276	0.513	1.481	0.481	0.509	1.434	0.501	0.426	0.925	0.050
21277	0.000	?	0.142	0.275	-0.073	0.501	0.263	-0.120	0.512
21278	0.497	1.380	0.224	0.444	1.020	0.149	0.516	1.498	0.296
21279	0.484	1.295	0.224	0.518	1.491	0.149	0.355	0.470	0.097
21280	0.097	-1.181	0.296	0.272	-0.091	0.149	0.184	-0.625	0.262
21281	0.261	-0.137	0.508	0.196	-0.581	0.478	0.097	-1.181	0.513
21282	0.224	-0.373	0.450	0.193	-0.600	0.193	0.387	0.672	0.513
21283	0.500	1.397	0.224	0.427	0.907	0.444	0.497	1.380	0.450
21284	0.142	-0.895	0.501	0.310	0.153	0.478	0.097	-1.181	0.224
21285	0.361	0.504	0.328	0.193	-0.600	0.273	0.142	-0.895	0.296

21286	0.187	-0.608	0.385	0.149	-0.882	0.235	0.261	-0.137	0.450
21287	0.226	-0.356	0.224	0.149	-0.882	0.235	0.261	-0.137	0.224
21288	0.326	0.285	0.513	0.272	-0.091	0.273	0.142	-0.895	0.224
21289	0.466	1.178	0.513	0.506	1.415	0.508	0.461	1.144	0.142
21290	0.224	-0.373	0.501	0.342	0.361	0.501	0.266	-0.103	0.328
21291	0.184	-0.625	0.508	0.275	-0.073	0.341	0.142	-0.895	0.385
21292	0.332	0.318	0.296	0.275	-0.073	0.149	0.097	-1.181	0.224
21293	0.297	0.099	0.142	0.307	0.135	0.235	0.184	-0.625	0.296
21294	0.355	0.470	0.097	0.506	1.415	0.273	0.487	1.312	0.185
21295	0.145	-0.878	0.185	0.234	-0.336	0.149	0.263	-0.120	0.224
21296	0.261	-0.137	0.185	0.196	-0.581	0.053	0.184	-0.625	0.296
21297	0.511	1.464	0.185	0.512	1.453	0.102	0.447	1.060	0.409
21298	0.466	1.178	0.262	0.488	1.302	0.053	0.408	0.807	0.508
21299	0.479	1.262	0.508	0.488	1.302	0.371	0.408	0.807	0.508
21300	0.300	0.116	0.450	0.272	-0.091	0.341	0.097	-1.181	0.097
21301	0.432	0.958	0.262	0.515	1.472	0.341	0.508	1.447	0.492
21302	0.411	0.824	0.450	0.503	1.396	0.462	0.508	1.447	0.501
21303	0.224	-0.373	0.224	0.193	-0.600	0.341	0.184	-0.625	0.467
21304	0.497	1.380	0.512	0.497	1.359	0.512	0.505	1.430	0.142
21305	0.497	1.380	0.492	0.497	1.359	0.512	0.505	1.430	0.467
21306	0.226	-0.356	0.050	0.149	-0.882	0.235	0.187	-0.608	0.508
21307	0.184	-0.625	0.142	0.149	-0.882	0.371	0.263	-0.120	0.142
21308	0.226	-0.356	0.512	0.149	-0.882	0.273	0.187	-0.608	0.262
21309	0.511	1.464	0.097	0.477	1.227	0.273	0.405	0.790	0.492
21310	0.142	-0.895	0.224	0.193	-0.600	0.462	0.224	-0.373	0.512
21311	0.479	1.262	0.185	0.442	1.001	0.273	0.500	1.397	0.409
21312	0.508	1.447	0.185	0.477	1.227	0.508	0.405	0.790	0.492
21313	0.184	-0.625	0.492	0.275	-0.073	0.235	0.145	-0.878	0.467
21314	0.184	-0.625	0.481	0.237	-0.317	0.341	0.145	-0.878	0.467
21315	0.224	-0.373	0.467	0.237	-0.317	0.273	0.145	-0.878	0.512
21316	0.329	0.301	0.492	0.275	-0.073	0.422	0.142	-0.895	0.512
21317	0.500	1.397	0.501	0.509	1.434	0.491	0.492	1.346	0.481
21318	0.500	1.397	0.262	0.500	1.378	0.149	0.489	1.329	0.185
21319	0.050	-1.485	0.262	0.342	0.361	0.514	0.142	-0.895	0.142
21320	0.187	-0.608	0.262	0.149	-0.882	0.501	0.224	-0.373	0.508
21321	0.097	-1.181	0.262	0.237	-0.317	0.309	0.261	-0.137	0.185
21322	0.263	-0.120	0.492	0.275	-0.073	0.422	0.142	-0.895	0.224
21323	0.261	-0.137	0.409	0.152	-0.863	0.235	0.142	-0.895	0.492
21324	0.226	-0.356	0.185	0.053	-1.504	0.512	0.261	-0.137	0.508
21325	0.263	-0.120	0.262	0.105	-1.165	0.309	0.224	-0.373	0.481
21326	0.295	0.082	0.492	0.053	-1.504	0.491	0.226	-0.356	0.501
21327	0.492	1.346	0.431	0.371	0.549	0.341	0.513	1.481	0.224
21328	0.492	1.346	0.467	0.342	0.361	0.193	0.503	1.413	0.185
21329	0.261	-0.137	0.481	0.342	0.361	0.193	0.097	-1.181	0.224
21330	0.405	0.790	0.262	0.459	1.114	0.309	0.505	1.430	0.142
21331	0.050	-1.485	0.296	0.339	0.342	0.478	0.184	-0.625	0.501
21332	0.503	1.413	0.508	0.512	1.453	0.309	0.513	1.481	0.224
21333	0.482	1.279	0.513	0.506	1.415	0.341	0.503	1.413	0.508
21334	0.224	-0.373	0.492	0.237	-0.317	0.235	0.142	-0.895	0.097
21335	0.361	0.504	0.492	0.374	0.568	0.478	0.358	0.487	0.097
21336	0.224	-0.373	0.224	0.272	-0.091	0.478	0.142	-0.895	0.296
21337	0.097	-1.181	0.185	0.272	-0.091	0.371	0.266	-0.103	0.142
21338	0.471	1.211	0.501	0.465	1.152	0.501	0.489	1.329	0.492
21339	0.184	-0.625	0.501	0.275	-0.073	0.235	0.184	-0.625	0.508
21340	0.505	1.430	0.501	0.509	1.434	0.193	0.484	1.295	0.097
21341	0.226	-0.356	0.513	0.237	-0.317	0.149	0.224	-0.373	0.431
21342	0.050	-1.485	0.467	0.345	0.379	0.491	0.184	-0.625	0.050
21343	0.097	-1.181	0.142	0.272	-0.091	0.478	0.224	-0.373	0.050
21344	0.508	1.447	0.492	0.421	0.869	0.102	0.511	1.464	0.328
21345	0.429	0.942	0.512	0.491	1.321	0.491	0.489	1.329	0.262
21346	0.224	-0.373	0.513	0.149	-0.882	0.508	0.261	-0.137	0.296

21347	0.492	1.346	0.409	0.512	1.453	0.508	0.508	1.447	0.224
21348	0.447	1.060	0.409	0.497	1.359	0.478	0.447	1.060	0.508
21349	0.508	1.447	0.224	0.307	0.135	0.422	0.492	1.346	0.508
21350	0.447	1.060	0.508	0.421	0.869	0.235	0.508	1.447	0.431
21351	0.142	-0.895	0.328	0.237	-0.317	0.309	0.184	-0.625	0.492
21352	0.500	1.397	0.224	0.515	1.472	0.149	0.382	0.638	0.142
21353	0.142	-0.895	0.142	0.310	0.153	0.149	0.184	-0.625	0.185
21354	0.479	1.262	0.142	0.485	1.283	0.193	0.489	1.329	0.224
21355	0.261	-0.137	0.224	0.345	0.379	0.193	0.329	0.301	0.296
21356	0.263	-0.120	0.450	0.196	-0.581	0.508	0.097	-1.181	0.296
21357	0.329	0.301	0.142	0.193	-0.600	0.501	0.224	-0.373	0.262
21358	0.263	-0.120	0.262	0.313	0.172	0.501	0.329	0.301	0.262
21359	0.495	1.363	0.450	0.480	1.246	0.478	0.489	1.329	0.492
21360	0.142	-0.895	0.467	0.310	0.153	0.462	0.184	-0.625	0.385
21361	0.387	0.672	0.492	0.345	0.379	0.508	0.358	0.487	0.296
21362	0.097	-1.181	0.185	0.234	-0.336	0.491	0.297	0.099	0.431
21363	0.492	1.346	0.508	0.480	1.246	0.309	0.503	1.413	0.050
21364	0.489	1.329	0.508	0.482	1.265	0.235	0.505	1.430	0.431
21365	0.226	-0.356	0.508	0.371	0.549	0.235	0.187	-0.608	0.508
21366	0.479	1.262	0.262	0.503	1.396	0.235	0.505	1.430	0.262
21367	0.300	0.116	0.185	0.234	-0.336	0.235	0.184	-0.625	0.097
21368	0.142	-0.895	0.185	0.196	-0.581	0.193	0.224	-0.373	0.492
21369	0.187	-0.608	0.508	0.149	-0.882	0.273	0.329	0.301	0.262
21370	0.476	1.245	0.501	0.488	1.302	0.422	0.511	1.464	0.097
21371	0.489	1.329	0.481	0.474	1.208	0.309	0.505	1.430	0.142
21372	0.361	0.504	0.492	0.102	-1.184	0.501	0.329	0.301	0.508
21373	0.447	1.060	0.224	0.488	1.302	0.273	0.382	0.638	0.492
21374	0.500	1.397	0.185	0.506	1.415	0.491	0.476	1.245	0.501
21375	0.500	1.397	0.450	0.506	1.415	0.508	0.476	1.245	0.481
21376	0.511	1.464	0.142	0.480	1.246	0.341	0.503	1.413	0.481
21377	0.184	-0.625	0.501	0.427	0.907	0.512	0.184	-0.625	0.185
21378	0.000	?	0.481	0.234	-0.336	0.478	0.297	0.099	0.142
21379	0.142	-0.895	0.328	0.307	0.135	0.491	0.142	-0.895	0.050
21380	0.263	-0.120	0.492	0.149	-0.882	0.501	0.187	-0.608	0.050
21381	0.384	0.655	0.481	0.149	-0.882	0.501	0.329	0.301	0.513
21382	0.184	-0.625	0.501	0.193	-0.600	0.341	0.184	-0.625	0.481
21383	0.295	0.082	0.501	0.193	-0.600	0.462	0.145	-0.878	0.142
21384	0.426	0.925	0.224	0.509	1.434	0.273	0.476	1.245	0.296
21385	0.447	1.060	0.409	0.503	1.396	0.491	0.476	1.245	0.409
21386	0.463	1.161	0.185	0.503	1.396	0.102	0.476	1.245	0.501
21387	0.487	1.312	0.328	0.477	1.227	0.462	0.513	1.481	0.492
21388	0.463	1.161	0.328	0.462	1.133	0.235	0.513	1.481	0.508
21389	0.505	1.430	0.512	0.506	1.415	0.149	0.432	0.958	0.097
21390	0.503	1.413	0.296	0.488	1.302	0.501	0.487	1.312	0.142
21391	0.184	-0.625	0.358	0.313	0.172	0.193	0.142	-0.895	0.508
21392	0.184	-0.625	0.513	0.234	-0.336	0.309	0.145	-0.878	0.431
21393	0.187	-0.608	0.296	0.237	-0.317	0.478	0.142	-0.895	0.513
21394	0.187	-0.608	0.185	0.237	-0.317	0.478	0.142	-0.895	0.512
21395	0.226	-0.356	0.185	0.237	-0.317	0.422	0.261	-0.137	0.050
21396	0.263	-0.120	0.501	0.193	-0.600	0.398	0.261	-0.137	0.262
21397	0.097	-1.181	0.501	0.275	-0.073	0.341	0.226	-0.356	0.508
21398	0.184	-0.625	0.431	0.424	0.888	0.501	0.097	-1.181	0.508
21399	0.097	-1.181	0.481	0.313	0.172	0.444	0.184	-0.625	0.508
21400	0.329	0.301	0.481	0.506	1.415	0.371	0.497	1.380	0.512
21401	0.184	-0.625	0.481	0.275	-0.073	0.491	0.097	-1.181	0.501
21402	0.466	1.178	0.262	0.488	1.302	0.273	0.479	1.262	0.050
21403	0.466	1.178	0.512	0.509	1.434	0.309	0.429	0.942	0.513
21404	0.224	-0.373	0.492	0.339	0.342	0.508	0.142	-0.895	0.508
21405	0.355	0.470	0.481	0.512	1.453	0.501	0.505	1.430	0.513
21406	0.513	1.481	0.097	0.480	1.246	0.422	0.326	0.285	0.513
21407	0.511	1.464	0.142	0.494	1.340	0.491	0.426	0.925	0.513

21408	0.408	0.807	0.296	0.497	1.359	0.371	0.474	1.228	0.185
21409	0.505	1.430	0.501	0.500	1.378	0.273	0.450	1.076	0.508
21410	0.297	0.099	0.409	0.342	0.361	0.491	0.050	-1.485	0.097
21411	0.463	1.161	0.409	0.465	1.152	0.462	0.495	1.363	0.185
21412	0.261	-0.137	0.296	0.275	-0.073	0.514	0.413	0.841	0.185
21413	0.508	1.447	0.492	0.491	1.321	0.501	0.445	1.043	0.501
21414	0.224	-0.373	0.481	0.102	-1.184	0.478	0.297	0.099	0.262
21415	0.482	1.279	0.328	0.462	1.133	0.508	0.476	1.245	0.097
21416	0.097	-1.181	0.512	0.234	-0.336	0.512	0.261	-0.137	0.467
21417	0.263	-0.120	0.262	0.149	-0.882	0.491	0.224	-0.373	0.508
21418	0.455	1.110	0.185	0.503	1.396	0.341	0.492	1.346	0.501
21419	0.145	-0.878	0.185	0.193	-0.600	0.514	0.297	0.099	0.262
21420	0.226	-0.356	0.508	0.310	0.153	0.508	0.263	-0.120	0.467
21421	0.482	1.279	0.409	0.477	1.227	0.273	0.484	1.295	0.481
21422	0.468	1.194	0.512	0.477	1.227	0.491	0.474	1.228	0.467
21423	0.455	1.110	0.512	0.427	0.907	0.273	0.484	1.295	0.185
21424	0.434	0.975	0.262	0.404	0.756	0.193	0.484	1.295	0.097
21425	0.184	-0.625	0.409	0.339	0.342	0.501	0.224	-0.373	0.097
21426	0.489	1.329	0.508	0.500	1.378	0.193	0.500	1.397	0.481
21427	0.505	1.430	0.409	0.442	1.001	0.422	0.497	1.380	0.481
21428	0.142	-0.895	0.185	0.374	0.568	0.398	0.261	-0.137	0.512
21429	0.503	1.413	0.409	0.491	1.321	0.462	0.516	1.498	0.501
21430	0.142	-0.895	0.224	0.272	-0.091	0.501	0.224	-0.373	0.097
21431	0.097	-1.181	0.508	0.310	0.153	0.501	0.187	-0.608	0.224
21432	0.450	1.076	0.508	0.512	1.453	0.341	0.487	1.312	0.492
21433	0.466	1.178	0.508	0.506	1.415	0.309	0.487	1.312	0.296
21434	0.495	1.363	0.262	0.424	0.888	0.512	0.508	1.447	0.450
21435	0.479	1.262	0.296	0.491	1.321	0.273	0.511	1.464	0.508
21436	0.184	-0.625	0.224	0.371	0.549	0.235	0.261	-0.137	0.224
21437	0.184	-0.625	0.508	0.275	-0.073	0.512	0.097	-1.181	0.467
21438	0.518	1.514	0.224	0.491	1.321	0.149	0.384	0.655	0.481
21439	0.489	1.329	0.501	0.465	1.152	0.512	0.492	1.346	0.512
21440	0.497	1.380	0.508	0.515	1.472	0.102	0.474	1.228	0.501
21441	0.479	1.262	0.224	0.500	1.378	0.501	0.384	0.655	0.501
21442	0.426	0.925	0.142	0.480	1.246	0.512	0.513	1.481	0.142
21443	0.474	1.228	0.358	0.509	1.434	0.149	0.492	1.346	0.185
21444	0.484	1.295	0.501	0.512	1.453	0.491	0.508	1.447	0.224
21445	0.513	1.481	0.224	0.497	1.359	0.309	0.516	1.498	0.492
21446	0.358	0.487	0.262	0.345	0.379	0.462	0.295	0.082	0.328
21447	0.513	1.481	0.142	0.512	1.453	0.512	0.479	1.262	0.492
21448	0.492	1.346	0.481	0.512	1.453	0.462	0.434	0.975	0.385
21449	0.142	-0.895	0.513	0.278	-0.054	0.398	0.295	0.082	0.224
21450	0.508	1.447	0.142	0.494	1.340	0.478	0.482	1.279	0.328
21451	0.411	0.824	0.481	0.272	-0.091	0.149	0.408	0.807	0.185
21452	0.295	0.082	0.508	0.196	-0.581	0.273	0.097	-1.181	0.501
21453	0.450	1.076	0.262	0.497	1.359	0.193	0.508	1.447	0.501
21454	0.184	-0.625	0.224	0.196	-0.581	0.491	0.224	-0.373	0.513
21455	0.500	1.397	0.224	0.424	0.888	0.491	0.503	1.413	0.512
21456	0.097	-1.181	0.501	0.401	0.737	0.491	0.097	-1.181	0.512
21457	0.429	0.942	0.328	0.468	1.170	0.235	0.505	1.430	0.512
21458	0.503	1.413	0.501	0.500	1.378	0.273	0.497	1.380	0.358
21459	0.503	1.413	0.185	0.500	1.378	0.512	0.497	1.380	0.512
21460	0.263	-0.120	0.185	0.345	0.379	0.273	0.097	-1.181	0.501
21461	0.184	-0.625	0.481	0.310	0.153	0.235	0.142	-0.895	0.492
21462	0.447	1.060	0.501	0.506	1.415	0.149	0.503	1.413	0.097
21463	0.142	-0.895	0.513	0.275	-0.073	0.235	0.263	-0.120	0.185
21464	0.266	-0.103	0.512	0.234	-0.336	0.341	0.142	-0.895	0.097
21465	0.468	1.194	0.481	0.503	1.396	0.273	0.463	1.161	0.513
21466	0.300	0.116	0.508	0.149	-0.882	0.235	0.142	-0.895	0.501
21467	0.513	1.481	0.097	0.509	1.434	0.149	0.495	1.363	0.492
21468	0.297	0.099	0.097	0.102	-1.184	0.273	0.226	-0.356	0.185

21469	0.487	1.312	0.501	0.506	1.415	0.273	0.495	1.363	0.492
21470	0.466	1.178	0.328	0.518	1.491	0.508	0.508	1.447	0.481
21471	0.226	-0.356	0.513	0.149	-0.882	0.508	0.187	-0.608	0.142
21472	0.476	1.245	0.431	0.491	1.321	0.508	0.447	1.060	0.492
21473	0.142	-0.895	0.508	0.307	0.135	0.235	0.263	-0.120	0.262
21474	0.468	1.194	0.296	0.459	1.114	0.514	0.505	1.430	0.050
21475	0.482	1.279	0.513	0.512	1.453	0.491	0.503	1.413	0.050
21476	0.384	0.655	0.508	0.462	1.133	0.462	0.505	1.430	0.481
21477	0.297	0.099	0.512	0.401	0.737	0.341	0.050	-1.485	0.450
21478	0.482	1.279	0.512	0.477	1.227	0.512	0.508	1.447	0.450
21479	0.142	-0.895	0.262	0.149	-0.882	0.501	0.300	0.116	0.467
21480	0.382	0.638	0.513	0.275	-0.073	0.309	0.097	-1.181	0.185
21481	0.263	-0.120	0.224	0.196	-0.581	0.102	0.263	-0.120	0.501
21482	0.479	1.262	0.224	0.494	1.340	0.512	0.511	1.464	0.512
21483	0.479	1.262	0.492	0.494	1.340	0.462	0.511	1.464	0.508
21484	0.513	1.481	0.224	0.488	1.302	0.501	0.445	1.043	0.185
21485	0.184	-0.625	0.467	0.234	-0.336	0.491	0.224	-0.373	0.501
21486	0.224	-0.373	0.513	0.275	-0.073	0.491	0.300	0.116	0.296
21487	0.450	1.076	0.508	0.520	1.509	0.491	0.445	1.043	0.492
21488	0.411	0.824	0.513	0.275	-0.073	0.422	0.226	-0.356	0.481
21489	0.142	-0.895	0.513	0.237	-0.317	0.444	0.226	-0.356	0.512
21490	0.263	-0.120	0.224	0.149	-0.882	0.444	0.142	-0.895	0.050
21491	0.097	-1.181	0.224	0.234	-0.336	0.149	0.329	0.301	0.097
21492	0.142	-0.895	0.185	0.342	0.361	0.273	0.384	0.655	0.097
21493	0.300	0.116	0.501	0.272	-0.091	0.501	0.224	-0.373	0.481
21494	0.226	-0.356	0.492	0.237	-0.317	0.235	0.295	0.082	0.185
21495	0.224	-0.373	0.358	0.149	-0.882	0.235	0.184	-0.625	0.358
21496	0.097	-1.181	0.224	0.275	-0.073	0.514	0.224	-0.373	0.501
21497	0.097	-1.181	0.224	0.275	-0.073	0.501	0.297	0.099	0.224
21498	0.500	1.397	0.185	0.509	1.434	0.478	0.413	0.841	0.296
21499	0.487	1.312	0.328	0.506	1.415	0.491	0.503	1.413	0.508
21500	0.487	1.312	0.512	0.506	1.415	0.149	0.503	1.413	0.501
21501	0.295	0.082	0.501	0.237	-0.317	0.235	0.097	-1.181	0.142
21502	0.487	1.312	0.501	0.506	1.415	0.149	0.495	1.363	0.097
21503	0.429	0.942	0.501	0.494	1.340	0.235	0.497	1.380	0.224
21504	0.476	1.245	0.512	0.465	1.152	0.508	0.463	1.161	0.501
21505	0.263	-0.120	0.513	0.345	0.379	0.102	0.142	-0.895	0.492
21506	0.505	1.430	0.501	0.515	1.472	0.491	0.463	1.161	0.142
21507	0.492	1.346	0.142	0.497	1.359	0.309	0.505	1.430	0.501
21508	0.145	-0.878	0.185	0.307	0.135	0.341	0.142	-0.895	0.481
21509	0.184	-0.625	0.185	0.102	-1.184	0.235	0.263	-0.120	0.050
21510	0.500	1.397	0.501	0.509	1.434	0.273	0.492	1.346	0.097
21511	0.505	1.430	0.513	0.462	1.133	0.478	0.513	1.481	0.224
21512	0.513	1.481	0.512	0.497	1.359	0.501	0.408	0.807	0.512
21513	0.489	1.329	0.450	0.491	1.321	0.235	0.487	1.312	0.097
21514	0.497	1.380	0.481	0.488	1.302	0.491	0.463	1.161	0.501
21515	0.489	1.329	0.185	0.488	1.302	0.193	0.463	1.161	0.142
21516	0.500	1.397	0.467	0.424	0.888	0.193	0.511	1.464	0.185
21517	0.387	0.672	0.501	0.447	1.039	0.235	0.508	1.447	0.513
21518	0.411	0.824	0.262	0.447	1.039	0.273	0.482	1.279	0.501
21519	0.261	-0.137	0.296	0.149	-0.882	0.309	0.184	-0.625	0.501
21520	0.358	0.487	0.467	0.272	-0.091	0.235	0.145	-0.878	0.492
21521	0.466	1.178	0.481	0.494	1.340	0.398	0.505	1.430	0.512
21522	0.263	-0.120	0.481	0.237	-0.317	0.398	0.184	-0.625	0.501
21523	0.297	0.099	0.142	0.234	-0.336	0.501	0.226	-0.356	0.262
21524	0.492	1.346	0.224	0.509	1.434	0.444	0.489	1.329	0.262
21525	0.463	1.161	0.492	0.500	1.378	0.102	0.505	1.430	0.513
21526	0.482	1.279	0.492	0.480	1.246	0.309	0.474	1.228	0.262
21527	0.468	1.194	0.492	0.494	1.340	0.273	0.495	1.363	0.492
21528	0.187	-0.608	0.512	0.149	-0.882	0.193	0.226	-0.356	0.512
21529	0.184	-0.625	0.512	0.237	-0.317	0.193	0.187	-0.608	0.492

21530	0.187	-0.608	0.185	0.149	-0.882	0.193	0.226	-0.356	0.142
21531	0.300	0.116	0.296	0.234	-0.336	0.193	0.142	-0.895	0.450
21532	0.429	0.942	0.512	0.509	1.434	0.508	0.508	1.447	0.492
21533	0.224	-0.373	0.501	0.102	-1.184	0.501	0.224	-0.373	0.508
21534	0.482	1.279	0.513	0.497	1.359	0.422	0.503	1.413	0.512
21535	0.142	-0.895	0.224	0.307	0.135	0.514	0.097	-1.181	0.224
21536	0.224	-0.373	0.501	0.339	0.342	0.309	0.097	-1.181	0.512
21537	0.229	-0.339	0.296	0.234	-0.336	0.514	0.295	0.082	0.224
21538	0.300	0.116	0.328	0.275	-0.073	0.422	0.142	-0.895	0.097
21539	0.508	1.447	0.512	0.477	1.227	0.309	0.484	1.295	0.501
21540	0.453	1.093	0.512	0.503	1.396	0.309	0.497	1.380	0.185
21541	0.226	-0.356	0.481	0.237	-0.317	0.512	0.097	-1.181	0.513
21542	0.500	1.397	0.450	0.491	1.321	0.398	0.429	0.942	0.481
21543	0.329	0.301	0.328	0.193	-0.600	0.102	0.050	-1.485	0.467
21544	0.384	0.655	0.328	0.193	-0.600	0.273	0.050	-1.485	0.481
21545	0.000	?	0.185	0.234	-0.336	0.235	0.329	0.301	0.512
21546	0.000	?	0.409	0.272	-0.091	0.444	0.263	-0.120	0.224
21547	0.000	?	0.185	0.310	0.153	0.508	0.297	0.099	0.224
21548	0.224	-0.373	0.467	0.234	-0.336	0.501	0.224	-0.373	0.224
21549	0.511	1.464	0.185	0.398	0.718	0.501	0.500	1.397	0.508
21550	0.511	1.464	0.467	0.398	0.718	0.444	0.500	1.397	0.467
21551	0.497	1.380	0.185	0.500	1.378	0.309	0.429	0.942	0.328
21552	0.513	1.481	0.512	0.439	0.982	0.341	0.492	1.346	0.328
21553	0.297	0.099	0.501	0.102	-1.184	0.235	0.145	-0.878	0.296
21554	0.145	-0.878	0.328	0.307	0.135	0.235	0.184	-0.625	0.097
21555	0.097	-1.181	0.467	0.275	-0.073	0.508	0.224	-0.373	0.512
21556	0.187	-0.608	0.501	0.193	-0.600	0.501	0.297	0.099	0.481
21557	0.187	-0.608	0.501	0.193	-0.600	0.235	0.297	0.099	0.501
21558	0.187	-0.608	0.508	0.193	-0.600	0.193	0.263	-0.120	0.481
21559	0.266	-0.103	0.508	0.193	-0.600	0.514	0.263	-0.120	0.262
21560	0.505	1.430	0.328	0.506	1.415	0.501	0.489	1.329	0.508
21561	0.447	1.060	0.512	0.500	1.378	0.478	0.384	0.655	0.513
21562	0.263	-0.120	0.492	0.424	0.888	0.478	0.295	0.082	0.512
21563	0.447	1.060	0.501	0.515	1.472	0.193	0.432	0.958	0.501
21564	0.224	-0.373	0.097	0.310	0.153	0.273	0.050	-1.485	0.409
21565	0.511	1.464	0.501	0.509	1.434	0.462	0.429	0.942	0.224
21566	0.495	1.363	0.185	0.418	0.850	0.491	0.500	1.397	0.513
21567	0.050	-1.485	0.296	0.313	0.172	0.508	0.261	-0.137	0.481
21568	0.142	-0.895	0.185	0.310	0.153	0.398	0.097	-1.181	0.467
21569	0.505	1.430	0.492	0.512	1.453	0.273	0.492	1.346	0.512
21570	0.097	-1.181	0.492	0.401	0.737	0.149	0.000	?	0.481
21571	0.226	-0.356	0.185	0.102	-1.184	0.501	0.263	-0.120	0.296
21572	0.184	-0.625	0.296	0.275	-0.073	0.478	0.097	-1.181	0.508
21573	0.184	-0.625	0.296	0.237	-0.317	0.478	0.142	-0.895	0.492
21574	0.492	1.346	0.142	0.444	1.020	0.501	0.500	1.397	0.185
21575	0.484	1.295	0.450	0.503	1.396	0.508	0.484	1.295	0.050
21576	0.466	1.178	0.512	0.497	1.359	0.371	0.497	1.380	0.508
21577	0.497	1.380	0.512	0.497	1.359	0.501	0.474	1.228	0.467
21578	0.503	1.413	0.328	0.442	1.001	0.491	0.474	1.228	0.513
21579	0.261	-0.137	0.262	0.313	0.172	0.462	0.184	-0.625	0.050
21580	0.263	-0.120	0.508	0.342	0.361	0.512	0.142	-0.895	0.142
21581	0.263	-0.120	0.358	0.237	-0.317	0.514	0.050	-1.485	0.492
21582	0.263	-0.120	0.450	0.237	-0.317	0.149	0.050	-1.485	0.492
21583	0.492	1.346	0.224	0.509	1.434	0.478	0.503	1.413	0.492
21584	0.408	0.807	0.224	0.503	1.396	0.235	0.474	1.228	0.385
21585	0.184	-0.625	0.450	0.234	-0.336	0.235	0.142	-0.895	0.409
21586	0.263	-0.120	0.050	0.193	-0.600	0.273	0.297	0.099	0.262
21587	0.487	1.312	0.224	0.512	1.453	0.235	0.405	0.790	0.142
21588	0.426	0.925	0.296	0.503	1.396	0.309	0.500	1.397	0.508
21589	0.468	1.194	0.492	0.477	1.227	0.478	0.489	1.329	0.512
21590	0.482	1.279	0.142	0.477	1.227	0.371	0.503	1.413	0.492

21591	0.263	-0.120	0.501	0.193	-0.600	0.309	0.097	-1.181	0.328
21592	0.297	0.099	0.508	0.275	-0.073	0.491	0.142	-0.895	0.492
21593	0.508	1.447	0.512	0.462	1.133	0.462	0.508	1.447	0.097
21594	0.513	1.481	0.328	0.491	1.321	0.398	0.429	0.942	0.512
21595	0.492	1.346	0.224	0.503	1.396	0.149	0.508	1.447	0.409
21596	0.489	1.329	0.431	0.398	0.718	0.444	0.505	1.430	0.508
21597	0.226	-0.356	0.224	0.275	-0.073	0.444	0.050	-1.485	0.513
21598	0.184	-0.625	0.224	0.149	-0.882	0.444	0.266	-0.103	0.385
21599	0.497	1.380	0.508	0.503	1.396	0.102	0.505	1.430	0.512
21600	0.497	1.380	0.142	0.480	1.246	0.273	0.505	1.430	0.097
21601	0.497	1.380	0.097	0.480	1.246	0.273	0.505	1.430	0.513
21602	0.508	1.447	0.513	0.497	1.359	0.491	0.511	1.464	0.481
21603	0.466	1.178	0.508	0.506	1.415	0.491	0.495	1.363	0.296
21604	0.142	-0.895	0.185	0.371	0.549	0.422	0.050	-1.485	0.431
21605	0.489	1.329	0.185	0.500	1.378	0.508	0.508	1.447	0.501
21606	0.508	1.447	0.262	0.491	1.321	0.235	0.503	1.413	0.358
21607	0.511	1.464	0.224	0.462	1.133	0.193	0.508	1.447	0.501
21608	0.408	0.807	0.512	0.506	1.415	0.462	0.511	1.464	0.501
21609	0.408	0.807	0.185	0.506	1.415	0.235	0.511	1.464	0.508
21610	0.224	-0.373	0.492	0.149	-0.882	0.491	0.184	-0.625	0.501
21611	0.513	1.481	0.492	0.480	1.246	0.508	0.497	1.380	0.501
21612	0.329	0.301	0.224	0.237	-0.317	0.235	0.000	?	0.224
21613	0.226	-0.356	0.224	0.234	-0.336	0.501	0.097	-1.181	0.501
21614	0.142	-0.895	0.501	0.275	-0.073	0.444	0.184	-0.625	0.450
21615	0.142	-0.895	0.512	0.234	-0.336	0.478	0.184	-0.625	0.512
21616	0.224	-0.373	0.508	0.307	0.135	0.514	0.000	?	0.224
21617	0.447	1.060	0.512	0.477	1.227	0.371	0.500	1.397	0.409
21618	0.142	-0.895	0.512	0.374	0.568	0.491	0.261	-0.137	0.097
21619	0.261	-0.137	0.512	0.313	0.172	0.235	0.097	-1.181	0.050
21620	0.450	1.076	0.513	0.488	1.302	0.273	0.461	1.144	0.224
21621	0.466	1.178	0.501	0.462	1.133	0.273	0.500	1.397	0.501
21622	0.492	1.346	0.512	0.401	0.737	0.512	0.503	1.413	0.431
21623	0.184	-0.625	0.512	0.149	-0.882	0.193	0.261	-0.137	0.097
21624	0.505	1.430	0.512	0.447	1.039	0.512	0.463	1.161	0.050
21625	0.505	1.430	0.501	0.447	1.039	0.422	0.476	1.245	0.097
21626	0.505	1.430	0.512	0.447	1.039	0.053	0.463	1.161	0.512
21627	0.266	-0.103	0.512	0.102	-1.184	0.193	0.184	-0.625	0.513
21628	0.187	-0.608	0.512	0.272	-0.091	0.371	0.100	-1.164	0.512
21629	0.187	-0.608	0.050	0.272	-0.091	0.398	0.100	-1.164	0.097
21630	0.508	1.447	0.513	0.488	1.302	0.508	0.474	1.228	0.097
21631	0.503	1.413	0.501	0.488	1.302	0.508	0.474	1.228	0.501
21632	0.479	1.262	0.512	0.424	0.888	0.508	0.505	1.430	0.097
21633	0.489	1.329	0.481	0.506	1.415	0.514	0.497	1.380	0.501
21634	0.224	-0.373	0.385	0.234	-0.336	0.273	0.097	-1.181	0.185
21635	0.184	-0.625	0.508	0.193	-0.600	0.491	0.224	-0.373	0.142
21636	0.453	1.093	0.512	0.462	1.133	0.462	0.495	1.363	0.501
21637	0.142	-0.895	0.512	0.234	-0.336	0.508	0.300	0.116	0.185
21638	0.500	1.397	0.513	0.497	1.359	0.341	0.453	1.093	0.097
21639	0.476	1.245	0.492	0.512	1.453	0.235	0.500	1.397	0.142
21640	0.326	0.285	0.508	0.237	-0.317	0.193	0.224	-0.373	0.481
21641	0.495	1.363	0.512	0.500	1.378	0.501	0.463	1.161	0.262
21642	0.482	1.279	0.508	0.442	1.001	0.398	0.474	1.228	0.501
21643	0.503	1.413	0.512	0.480	1.246	0.398	0.511	1.464	0.467
21644	0.503	1.413	0.492	0.518	1.491	0.102	0.492	1.346	0.450
21645	0.224	-0.373	0.501	0.374	0.568	0.491	0.000	?	0.097
21646	0.411	0.824	0.513	0.491	1.321	0.508	0.492	1.346	0.185
21647	0.184	-0.625	0.508	0.237	-0.317	0.235	0.142	-0.895	0.501
21648	0.326	0.285	0.513	0.278	-0.054	0.508	0.187	-0.608	0.185
21649	0.326	0.285	0.513	0.278	-0.054	0.273	0.226	-0.356	0.142
21650	0.508	1.447	0.501	0.506	1.415	0.309	0.495	1.363	0.501
21651	0.300	0.116	0.512	0.193	-0.600	0.309	0.329	0.301	0.142

21652	0.361	0.504	0.501	0.512	1.453	0.491	0.492	1.346	0.142
21653	0.516	1.498	0.450	0.421	0.869	0.514	0.384	0.655	0.481
21654	0.297	0.099	0.409	0.053	-1.504	0.501	0.224	-0.373	0.450
21655	0.184	-0.625	0.513	0.000	?	0.501	0.329	0.301	0.142
21656	0.184	-0.625	0.512	0.196	-0.581	0.149	0.184	-0.625	0.142
21657	0.500	1.397	0.512	0.371	0.549	0.501	0.495	1.363	0.142
21658	0.500	1.397	0.508	0.398	0.718	0.444	0.495	1.363	0.185
21659	0.429	0.942	0.508	0.509	1.434	0.444	0.513	1.481	0.450
21660	0.482	1.279	0.513	0.506	1.415	0.193	0.508	1.447	0.262
21661	0.482	1.279	0.501	0.506	1.415	0.462	0.508	1.447	0.501
21662	0.482	1.279	0.481	0.512	1.453	0.193	0.508	1.447	0.492
21663	0.263	-0.120	0.481	0.278	-0.054	0.508	0.358	0.487	0.142
21664	0.505	1.430	0.467	0.488	1.302	0.508	0.511	1.464	0.142
21665	0.492	1.346	0.467	0.459	1.114	0.512	0.495	1.363	0.431
21666	0.479	1.262	0.492	0.509	1.434	0.235	0.484	1.295	0.467
21667	0.097	-1.181	0.512	0.345	0.379	0.193	0.097	-1.181	0.450
21668	0.142	-0.895	0.501	0.234	-0.336	0.235	0.184	-0.625	0.467
21669	0.295	0.082	0.481	0.196	-0.581	0.478	0.097	-1.181	0.467
21670	0.503	1.413	0.224	0.500	1.378	0.235	0.503	1.413	0.508
21671	0.411	0.824	0.185	0.398	0.718	0.491	0.497	1.380	0.508
21672	0.411	0.824	0.224	0.398	0.718	0.462	0.489	1.329	0.467
21673	0.297	0.099	0.492	0.102	-1.184	0.235	0.184	-0.625	0.481
21674	0.492	1.346	0.142	0.491	1.321	0.341	0.484	1.295	0.481
21675	0.482	1.279	0.508	0.509	1.434	0.508	0.474	1.228	0.481
21676	0.326	0.285	0.513	0.240	-0.299	0.512	0.142	-0.895	0.501
21677	0.500	1.397	0.513	0.512	1.453	0.341	0.492	1.346	0.481
21678	0.261	-0.137	0.467	0.275	-0.073	0.273	0.263	-0.120	0.481
21679	0.184	-0.625	0.512	0.307	0.135	0.193	0.050	-1.485	0.492
21680	0.184	-0.625	0.513	0.307	0.135	0.512	0.050	-1.485	0.296
21681	0.505	1.430	0.409	0.494	1.340	0.491	0.474	1.228	0.481
21682	0.413	0.841	0.185	0.512	1.453	0.341	0.445	1.043	0.492
21683	0.513	1.481	0.185	0.500	1.378	0.508	0.445	1.043	0.450
21684	0.513	1.481	0.262	0.500	1.378	0.514	0.461	1.144	0.481
21685	0.263	-0.120	0.512	0.149	-0.882	0.235	0.184	-0.625	0.224
21686	0.408	0.807	0.450	0.503	1.396	0.235	0.492	1.346	0.512
21687	0.508	1.447	0.450	0.442	1.001	0.309	0.511	1.464	0.512
21688	0.405	0.790	0.492	0.442	1.001	0.514	0.505	1.430	0.492
21689	0.184	-0.625	0.492	0.193	-0.600	0.149	0.184	-0.625	0.501
21690	0.411	0.824	0.481	0.465	1.152	0.501	0.500	1.397	0.501
21691	0.226	-0.356	0.450	0.196	-0.581	0.273	0.297	0.099	0.508
21692	0.508	1.447	0.262	0.503	1.396	0.193	0.495	1.363	0.492
21693	0.508	1.447	0.508	0.503	1.396	0.371	0.484	1.295	0.508
21694	0.508	1.447	0.508	0.509	1.434	0.398	0.513	1.481	0.492
21695	0.263	-0.120	0.224	0.234	-0.336	0.478	0.050	-1.485	0.508
21696	0.297	0.099	0.501	0.193	-0.600	0.512	0.097	-1.181	0.513
21697	0.224	-0.373	0.492	0.234	-0.336	0.508	0.097	-1.181	0.512
21698	0.511	1.464	0.481	0.474	1.208	0.444	0.474	1.228	0.513
21699	0.226	-0.356	0.450	0.237	-0.317	0.235	0.184	-0.625	0.450
21700	0.497	1.380	0.481	0.488	1.302	0.235	0.358	0.487	0.467
21701	0.511	1.464	0.501	0.459	1.114	0.462	0.492	1.346	0.512
21702	0.226	-0.356	0.513	0.234	-0.336	0.371	0.226	-0.356	0.467
21703	0.142	-0.895	0.513	0.345	0.379	0.444	0.297	0.099	0.492
21704	0.361	0.504	0.513	0.503	1.396	0.491	0.503	1.413	0.512
21705	0.500	1.397	0.513	0.512	1.453	0.478	0.497	1.380	0.508
21706	0.226	-0.356	0.513	0.342	0.361	0.309	0.145	-0.878	0.492
21707	0.261	-0.137	0.512	0.275	-0.073	0.491	0.097	-1.181	0.431
21708	0.145	-0.878	0.512	0.193	-0.600	0.462	0.224	-0.373	0.431
21709	0.479	1.262	0.467	0.512	1.453	0.462	0.497	1.380	0.512
21710	0.508	1.447	0.185	0.494	1.340	0.444	0.484	1.295	0.512
21711	0.142	-0.895	0.492	0.342	0.361	0.149	0.142	-0.895	0.492
21712	0.482	1.279	0.512	0.509	1.434	0.478	0.500	1.397	0.512

21713	0.508	1.447	0.512	0.515	1.472	0.149	0.479	1.262	0.501
21714	0.263	-0.120	0.501	0.234	-0.336	0.273	0.050	-1.485	0.492
21715	0.226	-0.356	0.481	0.234	-0.336	0.501	0.100	-1.164	0.467
21716	0.224	-0.373	0.481	0.313	0.172	0.273	0.224	-0.373	0.467
21717	0.497	1.380	0.467	0.512	1.453	0.508	0.511	1.464	0.508
21718	0.329	0.301	0.328	0.152	-0.863	0.512	0.097	-1.181	0.512
21719	0.500	1.397	0.450	0.500	1.378	0.514	0.500	1.397	0.512
21720	0.184	-0.625	0.492	0.278	-0.054	0.444	0.142	-0.895	0.501
21721	0.184	-0.625	0.512	0.193	-0.600	0.508	0.187	-0.608	0.185
21722	0.474	1.228	0.512	0.371	0.549	0.102	0.511	1.464	0.224
21723	0.497	1.380	0.097	0.401	0.737	0.149	0.497	1.380	0.224
21724	0.508	1.447	0.501	0.477	1.227	0.235	0.505	1.430	0.492
21725	0.508	1.447	0.409	0.509	1.434	0.508	0.489	1.329	0.097
21726	0.482	1.279	0.431	0.509	1.434	0.508	0.508	1.447	0.513
21727	0.508	1.447	0.513	0.444	1.020	0.341	0.500	1.397	0.492
21728	0.097	-1.181	0.492	0.237	-0.317	0.422	0.263	-0.120	0.512
21729	0.097	-1.181	0.501	0.237	-0.317	0.422	0.263	-0.120	0.467
21730	0.495	1.363	0.185	0.462	1.133	0.273	0.518	1.514	0.492
21731	0.332	0.318	0.501	0.374	0.568	0.512	0.261	-0.137	0.492
21732	0.513	1.481	0.481	0.442	1.001	0.462	0.487	1.312	0.492
21733	0.432	0.958	0.481	0.491	1.321	0.491	0.505	1.430	0.328
21734	0.511	1.464	0.481	0.477	1.227	0.501	0.484	1.295	0.142
21735	0.295	0.082	0.481	0.307	0.135	0.273	0.142	-0.895	0.262
21736	0.505	1.430	0.450	0.488	1.302	0.491	0.450	1.076	0.508
21737	0.508	1.447	0.142	0.459	1.114	0.501	0.492	1.346	0.492
21738	0.505	1.430	0.492	0.465	1.152	0.501	0.505	1.430	0.492
21739	0.513	1.481	0.262	0.442	1.001	0.501	0.516	1.498	0.450
21740	0.263	-0.120	0.508	0.149	-0.882	0.273	0.224	-0.373	0.492
21741	0.508	1.447	0.508	0.474	1.208	0.193	0.511	1.464	0.492
21742	0.226	-0.356	0.508	0.149	-0.882	0.341	0.226	-0.356	0.501
21743	0.224	-0.373	0.508	0.272	-0.091	0.491	0.097	-1.181	0.262
21744	0.492	1.346	0.512	0.500	1.378	0.478	0.500	1.397	0.508
21745	0.224	-0.373	0.512	0.272	-0.091	0.508	0.184	-0.625	0.508
21746	0.463	1.161	0.492	0.509	1.434	0.491	0.505	1.430	0.185
21747	0.508	1.447	0.467	0.515	1.472	0.501	0.474	1.228	0.492
21748	0.513	1.481	0.501	0.512	1.453	0.235	0.468	1.194	0.409
21749	0.513	1.481	0.512	0.442	1.001	0.491	0.474	1.228	0.492
21750	0.511	1.464	0.512	0.509	1.434	0.462	0.508	1.447	0.512
21751	0.226	-0.356	0.512	0.102	-1.184	0.102	0.224	-0.373	0.501
21752	0.226	-0.356	0.508	0.149	-0.882	0.235	0.224	-0.373	0.492
21753	0.187	-0.608	0.492	0.234	-0.336	0.501	0.226	-0.356	0.492
21754	0.497	1.380	0.513	0.506	1.415	0.478	0.511	1.464	0.492
21755	0.495	1.363	0.513	0.512	1.453	0.491	0.463	1.161	0.492
21756	0.358	0.487	0.513	0.339	0.342	0.371	0.332	0.318	0.492
21757	0.226	-0.356	0.508	0.421	0.869	0.193	0.329	0.301	0.492
21758	0.226	-0.356	0.097	0.421	0.869	0.514	0.295	0.082	0.501
21759	0.187	-0.608	0.513	0.275	-0.073	0.512	0.097	-1.181	0.492
21760	0.329	0.301	0.481	0.509	1.434	0.514	0.505	1.430	0.467
21761	0.511	1.464	0.481	0.462	1.133	0.512	0.476	1.245	0.142
21762	0.503	1.413	0.262	0.491	1.321	0.512	0.492	1.346	0.512
21763	0.492	1.346	0.481	0.509	1.434	0.235	0.484	1.295	0.467
21764	0.000	?	0.481	0.275	-0.073	0.149	0.263	-0.120	0.467
21765	0.497	1.380	0.508	0.491	1.321	0.462	0.505	1.430	0.512
21766	0.513	1.481	0.296	0.497	1.359	0.501	0.505	1.430	0.501
21767	0.513	1.481	0.142	0.497	1.359	0.444	0.513	1.481	0.508
21768	0.505	1.430	0.358	0.497	1.359	0.341	0.497	1.380	0.513
21769	0.142	-0.895	0.224	0.272	-0.091	0.501	0.411	0.824	0.185
21770	0.184	-0.625	0.512	0.193	-0.600	0.149	0.224	-0.373	0.512
21771	0.184	-0.625	0.492	0.339	0.342	0.193	0.000	?	0.501
21772	0.495	1.363	0.142	0.497	1.359	0.422	0.508	1.447	0.431
21773	0.513	1.481	0.431	0.477	1.227	0.512	0.482	1.279	0.508

21774	0.508	1.447	0.431	0.503	1.396	0.462	0.466	1.178	0.142
21775	0.445	1.043	0.185	0.485	1.283	0.462	0.513	1.481	0.508
21776	0.479	1.262	0.492	0.497	1.359	0.235	0.479	1.262	0.467
21777	0.187	-0.608	0.512	0.237	-0.317	0.235	0.295	0.082	0.467
21778	0.471	1.211	0.481	0.485	1.283	0.235	0.503	1.413	0.481
21779	0.500	1.397	0.224	0.459	1.114	0.491	0.489	1.329	0.481
21780	0.263	-0.120	0.481	0.105	-1.165	0.309	0.184	-0.625	0.512
21781	0.297	0.099	0.501	0.234	-0.336	0.462	0.050	-1.485	0.262
21782	0.468	1.194	0.358	0.497	1.359	0.341	0.511	1.464	0.508
21783	0.482	1.279	0.481	0.477	1.227	0.491	0.466	1.178	0.512
21784	0.482	1.279	0.508	0.488	1.302	0.309	0.513	1.481	0.501
21785	0.145	-0.878	0.262	0.368	0.530	0.512	0.050	-1.485	0.501
21786	0.226	-0.356	0.358	0.193	-0.600	0.444	0.145	-0.878	0.501
21787	0.489	1.329	0.512	0.512	1.453	0.273	0.489	1.329	0.512
21788	0.489	1.329	0.512	0.512	1.453	0.501	0.489	1.329	0.296
21789	0.489	1.329	0.328	0.512	1.453	0.491	0.489	1.329	0.328
21790	0.508	1.447	0.142	0.512	1.453	0.501	0.384	0.655	0.262
21791	0.508	1.447	0.508	0.512	1.453	0.444	0.408	0.807	0.481
21792	0.184	-0.625	0.224	0.234	-0.336	0.444	0.263	-0.120	0.512
21793	0.297	0.099	0.512	0.149	-0.882	0.149	0.142	-0.895	0.512
21794	0.508	1.447	0.512	0.465	1.152	0.512	0.508	1.447	0.501
21795	0.500	1.397	0.513	0.503	1.396	0.501	0.513	1.481	0.512
21796	0.513	1.481	0.481	0.444	1.020	0.514	0.492	1.346	0.512
21797	0.224	-0.373	0.467	0.339	0.342	0.273	0.326	0.285	0.508
21798	0.495	1.363	0.385	0.497	1.359	0.462	0.495	1.363	0.501
21799	0.297	0.099	0.328	0.149	-0.882	0.341	0.097	-1.181	0.492
21800	0.332	0.318	0.481	0.193	-0.600	0.193	0.000	?	0.513
21801	0.508	1.447	0.185	0.418	0.850	0.341	0.508	1.447	0.492
21802	0.508	1.447	0.262	0.509	1.434	0.422	0.405	0.790	0.481
21803	0.479	1.262	0.185	0.459	1.114	0.501	0.505	1.430	0.501
21804	0.453	1.093	0.262	0.456	1.095	0.273	0.505	1.430	0.512
21805	0.329	0.301	0.467	0.234	-0.336	0.193	0.000	?	0.501
21806	0.329	0.301	0.513	0.234	-0.336	0.235	0.000	?	0.501
21807	0.184	-0.625	0.481	0.237	-0.317	0.309	0.384	0.655	0.431
21808	0.405	0.790	0.512	0.494	1.340	0.478	0.508	1.447	0.501
21809	0.184	-0.625	0.508	0.310	0.153	0.508	0.097	-1.181	0.224
21810	0.463	1.161	0.501	0.465	1.152	0.491	0.508	1.447	0.508
21811	0.184	-0.625	0.185	0.339	0.342	0.235	0.000	?	0.508
21812	0.468	1.194	0.508	0.491	1.321	0.273	0.474	1.228	0.508
21813	0.187	-0.608	0.481	0.310	0.153	0.478	0.295	0.082	0.097
21814	0.503	1.413	0.513	0.509	1.434	0.193	0.432	0.958	0.492
21815	0.492	1.346	0.512	0.444	1.020	0.422	0.503	1.413	0.492
21816	0.326	0.285	0.512	0.278	-0.054	0.309	0.358	0.487	0.501
21817	0.461	1.144	0.224	0.503	1.396	0.193	0.500	1.397	0.431
21818	0.497	1.380	0.512	0.494	1.340	0.512	0.497	1.380	0.296
21819	0.497	1.380	0.431	0.503	1.396	0.309	0.505	1.430	0.431
21820	0.511	1.464	0.508	0.442	1.001	0.235	0.492	1.346	0.142
21821	0.511	1.464	0.508	0.442	1.001	0.273	0.492	1.346	0.512
21822	0.326	0.285	0.508	0.149	-0.882	0.491	0.226	-0.356	0.481
21823	0.508	1.447	0.492	0.509	1.434	0.235	0.503	1.413	0.328
21824	0.489	1.329	0.513	0.497	1.359	0.491	0.447	1.060	0.481
21825	0.495	1.363	0.513	0.515	1.472	0.508	0.508	1.447	0.501
21826	0.097	-1.181	0.142	0.275	-0.073	0.491	0.224	-0.373	0.296
21827	0.495	1.363	0.513	0.468	1.170	0.235	0.413	0.841	0.501
21828	0.184	-0.625	0.262	0.345	0.379	0.149	0.097	-1.181	0.508
21829	0.297	0.099	0.492	0.193	-0.600	0.512	0.050	-1.485	0.492
21830	0.187	-0.608	0.501	0.342	0.361	0.149	0.226	-0.356	0.296
21831	0.492	1.346	0.481	0.424	0.888	0.235	0.503	1.413	0.431
21832	0.489	1.329	0.481	0.500	1.378	0.512	0.426	0.925	0.512
21833	0.184	-0.625	0.481	0.272	-0.091	0.341	0.097	-1.181	0.328
21834	0.297	0.099	0.501	0.193	-0.600	0.341	0.050	-1.485	0.512

21835	0.295	0.082	0.185	0.237	-0.317	0.501	0.000	?	0.450
21836	0.142	-0.895	0.467	0.310	0.153	0.508	0.097	-1.181	0.296
21837	0.450	1.076	0.508	0.477	1.227	0.235	0.508	1.447	0.358
21838	0.505	1.430	0.492	0.512	1.453	0.235	0.511	1.464	0.492
21839	0.505	1.430	0.467	0.494	1.340	0.149	0.508	1.447	0.492
21840	0.329	0.301	0.512	0.237	-0.317	0.149	0.097	-1.181	0.050
21841	0.261	-0.137	0.050	0.272	-0.091	0.491	0.097	-1.181	0.185
21842	0.500	1.397	0.512	0.477	1.227	0.193	0.500	1.397	0.501
21843	0.361	0.504	0.142	0.193	-0.600	0.512	0.097	-1.181	0.385
21844	0.450	1.076	0.185	0.424	0.888	0.508	0.500	1.397	0.467
21845	0.224	-0.373	0.501	0.310	0.153	0.273	0.184	-0.625	0.467
21846	0.224	-0.373	0.142	0.196	-0.581	0.273	0.142	-0.895	0.508
21847	0.447	1.060	0.492	0.512	1.453	0.478	0.500	1.397	0.492
21848	0.050	-1.485	0.492	0.307	0.135	0.512	0.184	-0.625	0.513
21849	0.224	-0.373	0.050	0.234	-0.336	0.398	0.097	-1.181	0.492
21850	0.297	0.099	0.492	0.272	-0.091	0.491	0.142	-0.895	0.050
21851	0.487	1.312	0.512	0.491	1.321	0.491	0.479	1.262	0.481
21852	0.142	-0.895	0.050	0.234	-0.336	0.491	0.261	-0.137	0.224
21853	0.495	1.363	0.481	0.485	1.283	0.491	0.503	1.413	0.097
21854	0.503	1.413	0.512	0.503	1.396	0.508	0.468	1.194	0.262
21855	0.513	1.481	0.185	0.488	1.302	0.491	0.445	1.043	0.262
21856	0.329	0.301	0.224	0.234	-0.336	0.501	0.097	-1.181	0.492
21857	0.226	-0.356	0.467	0.149	-0.882	0.501	0.184	-0.625	0.481
21858	0.429	0.942	0.513	0.506	1.415	0.398	0.505	1.430	0.501
21859	0.226	-0.356	0.513	0.149	-0.882	0.501	0.184	-0.625	0.481
21860	0.224	-0.373	0.501	0.237	-0.317	0.501	0.145	-0.878	0.358
21861	0.505	1.430	0.467	0.509	1.434	0.512	0.497	1.380	0.492
21862	0.142	-0.895	0.467	0.342	0.361	0.422	0.142	-0.895	0.328
21863	0.097	-1.181	0.467	0.339	0.342	0.478	0.142	-0.895	0.513
21864	0.508	1.447	0.467	0.500	1.378	0.478	0.484	1.295	0.492
21865	0.503	1.413	0.467	0.509	1.434	0.462	0.453	1.093	0.450
21866	0.184	-0.625	0.513	0.237	-0.317	0.478	0.142	-0.895	0.450
21867	0.184	-0.625	0.185	0.237	-0.317	0.193	0.142	-0.895	0.358
21868	0.261	-0.137	0.512	0.152	-0.863	0.501	0.142	-0.895	0.142
21869	0.226	-0.356	0.492	0.152	-0.863	0.501	0.184	-0.625	0.481
21870	0.505	1.430	0.501	0.491	1.321	0.512	0.450	1.076	0.492
21871	0.184	-0.625	0.481	0.196	-0.581	0.478	0.263	-0.120	0.508
21872	0.489	1.329	0.501	0.509	1.434	0.514	0.497	1.380	0.450
21873	0.489	1.329	0.450	0.503	1.396	0.501	0.489	1.329	0.262
21874	0.224	-0.373	0.142	0.275	-0.073	0.501	0.142	-0.895	0.513
21875	0.224	-0.373	0.431	0.275	-0.073	0.478	0.142	-0.895	0.492
21876	0.492	1.346	0.431	0.482	1.265	0.512	0.437	0.992	0.481
21877	0.505	1.430	0.501	0.512	1.453	0.478	0.466	1.178	0.501
21878	0.500	1.397	0.501	0.398	0.718	0.491	0.450	1.076	0.296
21879	0.513	1.481	0.512	0.488	1.302	0.422	0.471	1.211	0.508
21880	0.513	1.481	0.492	0.488	1.302	0.398	0.471	1.211	0.262
21881	0.513	1.481	0.492	0.488	1.302	0.508	0.513	1.481	0.501
21882	0.511	1.464	0.512	0.482	1.265	0.508	0.508	1.447	0.481
21883	0.500	1.397	0.262	0.503	1.396	0.491	0.468	1.194	0.467
21884	0.513	1.481	0.508	0.491	1.321	0.514	0.474	1.228	0.492
21885	0.513	1.481	0.185	0.503	1.396	0.478	0.474	1.228	0.467
21886	0.513	1.481	0.501	0.503	1.396	0.514	0.474	1.228	0.513
21887	0.503	1.413	0.513	0.398	0.718	0.508	0.495	1.363	0.224
21888	0.500	1.397	0.513	0.500	1.378	0.514	0.484	1.295	0.467
21889	0.500	1.397	0.513	0.500	1.378	0.508	0.484	1.295	0.508
21890	0.513	1.481	0.185	0.512	1.453	0.508	0.492	1.346	0.501
21891	0.050	-1.485	0.513	0.421	0.869	0.512	0.297	0.099	0.501
21892	0.513	1.481	0.492	0.485	1.283	0.491	0.479	1.262	0.512
21893	0.500	1.397	0.481	0.485	1.283	0.501	0.489	1.329	0.296
21894	0.511	1.464	0.142	0.459	1.114	0.462	0.447	1.060	0.296
21895	0.482	1.279	0.501	0.477	1.227	0.514	0.482	1.279	0.501

21896	0.384	0.655	0.185	0.193	-0.600	0.512	0.224	-0.373	0.185
21897	0.505	1.430	0.513	0.500	1.378	0.501	0.513	1.481	0.224
21898	0.511	1.464	0.501	0.500	1.378	0.444	0.513	1.481	0.467
21899	0.508	1.447	0.501	0.512	1.453	0.462	0.489	1.329	0.328
21900	0.511	1.464	0.512	0.480	1.246	0.508	0.497	1.380	0.501
21901	0.489	1.329	0.512	0.515	1.472	0.444	0.500	1.397	0.501
21902	0.503	1.413	0.050	0.500	1.378	0.514	0.505	1.430	0.224
21903	0.511	1.464	0.512	0.497	1.359	0.309	0.492	1.346	0.142
21904	0.511	1.464	0.508	0.482	1.265	0.341	0.505	1.430	0.508
21905	0.511	1.464	0.508	0.512	1.453	0.371	0.487	1.312	0.358
21906	0.489	1.329	0.501	0.480	1.246	0.508	0.505	1.430	0.358
21907	0.492	1.346	0.501	0.488	1.302	0.309	0.518	1.514	0.492
21908	0.513	1.481	0.512	0.424	0.888	0.514	0.508	1.447	0.512
21909	0.508	1.447	0.358	0.401	0.737	0.514	0.508	1.447	0.185
21910	0.511	1.464	0.467	0.503	1.396	0.491	0.447	1.060	0.097
21911	0.511	1.464	0.358	0.503	1.396	0.512	0.463	1.161	0.467
21912	0.497	1.380	0.481	0.491	1.321	0.491	0.505	1.430	0.501
21913	0.505	1.430	0.512	0.515	1.472	0.491	0.463	1.161	0.501
21914	0.492	1.346	0.385	0.482	1.265	0.508	0.495	1.363	0.492
21915	0.447	1.060	0.296	0.515	1.472	0.341	0.505	1.430	0.501
21916	0.408	0.807	0.512	0.512	1.453	0.235	0.505	1.430	0.508
21917	0.513	1.481	0.481	0.515	1.472	0.273	0.495	1.363	0.501
21918	0.503	1.413	0.512	0.506	1.415	0.462	0.432	0.958	0.512
21919	0.503	1.413	0.467	0.506	1.415	0.491	0.432	0.958	0.501
21920	0.505	1.430	0.467	0.512	1.453	0.491	0.513	1.481	0.492
21921	0.497	1.380	0.508	0.488	1.302	0.462	0.516	1.498	0.224
21922	0.516	1.498	0.431	0.503	1.396	0.462	0.489	1.329	0.467
21923	0.500	1.397	0.358	0.462	1.133	0.508	0.513	1.481	0.512
21924	0.484	1.295	0.385	0.515	1.472	0.491	0.500	1.397	0.481
21925	0.479	1.262	0.385	0.512	1.453	0.341	0.492	1.346	0.501
21926	0.468	1.194	0.385	0.506	1.415	0.514	0.466	1.178	0.492
21927	0.463	1.161	0.512	0.447	1.039	0.514	0.463	1.161	0.492
21928	0.492	1.346	0.512	0.462	1.133	0.273	0.508	1.447	0.262
21929	0.513	1.481	0.508	0.509	1.434	0.512	0.513	1.481	0.501
21930	0.500	1.397	0.492	0.447	1.039	0.508	0.516	1.498	0.501
21931	0.474	1.228	0.508	0.518	1.491	0.478	0.497	1.380	0.508
21932	0.226	-0.356	0.508	0.310	0.153	0.491	0.187	-0.608	0.512
21933	0.184	-0.625	0.296	0.342	0.361	0.508	0.226	-0.356	0.513
21934	0.226	-0.356	0.328	0.371	0.549	0.512	0.226	-0.356	0.508
21935	0.487	1.312	0.450	0.512	1.453	0.514	0.489	1.329	0.508
21936	0.142	-0.895	0.481	0.310	0.153	0.514	0.097	-1.181	0.508
21937	0.505	1.430	0.513	0.506	1.415	0.514	0.518	1.514	0.224
21938	0.511	1.464	0.501	0.506	1.415	0.514	0.497	1.380	0.508
21939	0.513	1.481	0.450	0.491	1.321	0.501	0.511	1.464	0.296
21940	0.466	1.178	0.450	0.515	1.472	0.501	0.463	1.161	0.508
21941	0.513	1.481	0.492	0.491	1.321	0.501	0.487	1.312	0.492
21942	0.516	1.498	0.501	0.491	1.321	0.508	0.487	1.312	0.450
21943	0.408	0.807	0.450	0.503	1.396	0.371	0.489	1.329	0.450
21944	0.184	-0.625	0.501	0.342	0.361	0.422	0.329	0.301	0.224
21945	0.184	-0.625	0.409	0.234	-0.336	0.478	0.145	-0.878	0.481
21946	0.261	-0.137	0.512	0.272	-0.091	0.501	0.261	-0.137	0.512
21947	0.511	1.464	0.224	0.459	1.114	0.478	0.505	1.430	0.501
21948	0.453	1.093	0.224	0.497	1.359	0.514	0.492	1.346	0.142
21949	0.453	1.093	0.185	0.497	1.359	0.478	0.492	1.346	0.501
21950	0.492	1.346	0.467	0.462	1.133	0.508	0.447	1.060	0.262
21951	0.484	1.295	0.296	0.471	1.189	0.235	0.492	1.346	0.501
21952	0.484	1.295	0.142	0.503	1.396	0.491	0.489	1.329	0.481
21953	0.453	1.093	0.501	0.482	1.265	0.422	0.497	1.380	0.467
21954	0.261	-0.137	0.508	0.342	0.361	0.491	0.261	-0.137	0.492
21955	0.503	1.413	0.450	0.512	1.453	0.371	0.505	1.430	0.492
21956	0.503	1.413	0.492	0.512	1.453	0.341	0.505	1.430	0.142

21957	0.226	-0.356	0.501	0.275	-0.073	0.478	0.184	-0.625	0.492
21958	0.503	1.413	0.513	0.515	1.472	0.514	0.489	1.329	0.508
21959	0.497	1.380	0.512	0.506	1.415	0.512	0.411	0.824	0.508
21960	0.476	1.245	0.508	0.474	1.208	0.514	0.495	1.363	0.481
21961	0.450	1.076	0.467	0.494	1.340	0.491	0.508	1.447	0.481
21962	0.482	1.279	0.296	0.506	1.415	0.422	0.505	1.430	0.512
21963	0.492	1.346	0.296	0.518	1.491	0.149	0.497	1.380	0.508
21964	0.511	1.464	0.296	0.509	1.434	0.512	0.489	1.329	0.501
21965	0.511	1.464	0.501	0.509	1.434	0.512	0.489	1.329	0.296
21966	0.511	1.464	0.501	0.509	1.434	0.512	0.489	1.329	0.508
21967	0.511	1.464	0.501	0.509	1.434	0.512	0.489	1.329	0.513
21968	0.511	1.464	0.501	0.497	1.359	0.512	0.489	1.329	0.224
21969	0.508	1.447	0.501	0.500	1.378	0.512	0.497	1.380	0.262
21970	0.508	1.447	0.328	0.500	1.378	0.149	0.489	1.329	0.501
21971	0.471	1.211	0.492	0.512	1.453	0.462	0.463	1.161	0.431
21972	0.187	-0.608	0.501	0.371	0.549	0.444	0.142	-0.895	0.501
21973	0.484	1.295	0.142	0.424	0.888	0.512	0.516	1.498	0.508
21974	0.505	1.430	0.142	0.477	1.227	0.422	0.466	1.178	0.508
21975	0.505	1.430	0.185	0.500	1.378	0.444	0.466	1.178	0.450
21976	0.495	1.363	0.508	0.482	1.265	0.444	0.511	1.464	0.512
21977	0.479	1.262	0.508	0.518	1.491	0.508	0.503	1.413	0.513
21978	0.479	1.262	0.508	0.480	1.246	0.508	0.508	1.447	0.501
21979	0.466	1.178	0.501	0.506	1.415	0.491	0.511	1.464	0.508
21980	0.329	0.301	0.501	0.237	-0.317	0.501	0.184	-0.625	0.409
21981	0.453	1.093	0.508	0.491	1.321	0.512	0.505	1.430	0.512
21982	0.487	1.312	0.508	0.418	0.850	0.422	0.508	1.447	0.481
21983	0.500	1.397	0.328	0.494	1.340	0.514	0.429	0.942	0.262
21984	0.503	1.413	0.224	0.371	0.549	0.462	0.511	1.464	0.450
21985	0.097	-1.181	0.450	0.345	0.379	0.501	0.142	-0.895	0.508
21986	0.492	1.346	0.481	0.482	1.265	0.512	0.505	1.430	0.492
21987	0.411	0.824	0.224	0.518	1.491	0.508	0.466	1.178	0.501
21988	0.432	0.958	0.050	0.515	1.472	0.508	0.466	1.178	0.501
21989	0.511	1.464	0.512	0.518	1.491	0.501	0.479	1.262	0.492
21990	0.495	1.363	0.512	0.491	1.321	0.501	0.482	1.279	0.492
21991	0.500	1.397	0.512	0.421	0.869	0.235	0.511	1.464	0.492
21992	0.184	-0.625	0.508	0.149	-0.882	0.491	0.261	-0.137	0.501
21993	0.492	1.346	0.508	0.512	1.453	0.508	0.508	1.447	0.492
21994	0.479	1.262	0.492	0.512	1.453	0.508	0.505	1.430	0.328
21995	0.479	1.262	0.050	0.512	1.453	0.193	0.497	1.380	0.481
21996	0.479	1.262	0.296	0.512	1.453	0.512	0.497	1.380	0.224
21997	0.479	1.262	0.262	0.512	1.453	0.512	0.497	1.380	0.481
21998	0.450	1.076	0.262	0.509	1.434	0.478	0.508	1.447	0.328
21999	0.142	-0.895	0.481	0.149	-0.882	0.309	0.300	0.116	0.224
22000	0.497	1.380	0.512	0.459	1.114	0.273	0.332	0.318	0.358
22001	0.261	-0.137	0.296	0.450	1.057	0.193	0.261	-0.137	0.328
22002	0.505	1.430	0.467	0.512	1.453	0.193	0.479	1.262	0.224
22003	0.503	1.413	0.492	0.418	0.850	0.462	0.516	1.498	0.224
22004	0.503	1.413	0.185	0.439	0.982	0.478	0.513	1.481	0.501
22005	0.503	1.413	0.328	0.439	0.982	0.102	0.500	1.397	0.450
22006	0.511	1.464	0.385	0.509	1.434	0.501	0.511	1.464	0.481
22007	0.511	1.464	0.481	0.509	1.434	0.508	0.511	1.464	0.513
22008	0.489	1.329	0.481	0.497	1.359	0.235	0.508	1.447	0.492
22009	0.463	1.161	0.185	0.503	1.396	0.491	0.500	1.397	0.513
22010	0.497	1.380	0.097	0.518	1.491	0.501	0.489	1.329	0.501
22011	0.508	1.447	0.501	0.424	0.888	0.462	0.508	1.447	0.501
22012	0.516	1.498	0.512	0.518	1.491	0.149	0.495	1.363	0.467
22013	0.511	1.464	0.512	0.465	1.152	0.462	0.479	1.262	0.296
22014	0.503	1.413	0.262	0.503	1.396	0.512	0.500	1.397	0.296
22015	0.487	1.312	0.492	0.512	1.453	0.422	0.511	1.464	0.328
22016	0.516	1.498	0.492	0.509	1.434	0.501	0.500	1.397	0.513
22017	0.516	1.498	0.262	0.509	1.434	0.508	0.500	1.397	0.512

22018	0.508	1.447	0.481	0.497	1.359	0.235	0.432	0.958	0.512
22019	0.503	1.413	0.513	0.497	1.359	0.235	0.500	1.397	0.512
22020	0.097	-1.181	0.481	0.237	-0.317	0.512	0.226	-0.356	0.512
22021	0.513	1.481	0.481	0.491	1.321	0.508	0.500	1.397	0.262
22022	0.476	1.245	0.262	0.509	1.434	0.193	0.508	1.447	0.467
22023	0.476	1.245	0.508	0.509	1.434	0.273	0.508	1.447	0.508
22024	0.263	-0.120	0.492	0.196	-0.581	0.491	0.097	-1.181	0.296
22025	0.479	1.262	0.467	0.506	1.415	0.235	0.492	1.346	0.328
22026	0.479	1.262	0.467	0.506	1.415	0.514	0.492	1.346	0.224
22027	0.508	1.447	0.467	0.471	1.189	0.514	0.503	1.413	0.508
22028	0.295	0.082	0.508	0.310	0.153	0.501	0.432	0.958	0.467
22029	0.142	-0.895	0.508	0.272	-0.091	0.501	0.300	0.116	0.508
22030	0.358	0.487	0.224	0.193	-0.600	0.508	0.432	0.958	0.512
22031	0.224	-0.373	0.296	0.193	-0.600	0.512	0.142	-0.895	0.512
22032	0.513	1.481	0.501	0.462	1.133	0.149	0.516	1.498	0.508
22033	0.492	1.346	0.328	0.477	1.227	0.478	0.479	1.262	0.481
22034	0.142	-0.895	0.492	0.102	-1.184	0.341	0.332	0.318	0.185
22035	0.432	0.958	0.262	0.506	1.415	0.309	0.482	1.279	0.097
22036	0.432	0.958	0.185	0.509	1.434	0.102	0.508	1.447	0.512
22037	0.184	-0.625	0.142	0.234	-0.336	0.102	0.300	0.116	0.513
22038	0.489	1.329	0.142	0.491	1.321	0.508	0.503	1.413	0.142
22039	0.511	1.464	0.142	0.500	1.378	0.512	0.505	1.430	0.097
22040	0.482	1.279	0.142	0.465	1.152	0.444	0.492	1.346	0.467
22041	0.224	-0.373	0.492	0.149	-0.882	0.514	0.295	0.082	0.467
22042	0.484	1.295	0.185	0.465	1.152	0.512	0.432	0.958	0.481
22043	0.503	1.413	0.492	0.518	1.491	0.512	0.508	1.447	0.513
22044	0.363	0.520	0.385	0.424	0.888	0.398	0.329	0.301	0.512
22045	0.476	1.245	0.467	0.500	1.378	0.491	0.511	1.464	0.492
22046	0.508	1.447	0.431	0.506	1.415	0.512	0.453	1.093	0.185
22047	0.261	-0.137	0.409	0.237	-0.317	0.514	0.297	0.099	0.185
22048	0.361	0.504	0.512	0.234	-0.336	0.514	0.355	0.470	0.385
22049	0.511	1.464	0.501	0.506	1.415	0.508	0.492	1.346	0.262
22050	0.511	1.464	0.501	0.503	1.396	0.398	0.492	1.346	0.409
22051	0.332	0.318	0.450	0.193	-0.600	0.514	0.050	-1.485	0.501
22052	0.142	-0.895	0.409	0.272	-0.091	0.491	0.184	-0.625	0.467
22053	0.503	1.413	0.492	0.491	1.321	0.491	0.497	1.380	0.450
22054	0.224	-0.373	0.431	0.234	-0.336	0.501	0.389	0.689	0.492
22055	0.511	1.464	0.492	0.515	1.472	0.398	0.468	1.194	0.142
22056	0.511	1.464	0.481	0.515	1.472	0.501	0.468	1.194	0.142
22057	0.511	1.464	0.450	0.503	1.396	0.514	0.500	1.397	0.467
22058	0.476	1.245	0.467	0.500	1.378	0.462	0.495	1.363	0.512
22059	0.466	1.178	0.050	0.506	1.415	0.478	0.508	1.447	0.512
22060	0.384	0.655	0.296	0.512	1.453	0.309	0.487	1.312	0.224
22061	0.326	0.285	0.492	0.149	-0.882	0.491	0.050	-1.485	0.296
22062	0.479	1.262	0.492	0.480	1.246	0.273	0.484	1.295	0.513
22063	0.184	-0.625	0.050	0.342	0.361	0.508	0.226	-0.356	0.492
22064	0.261	-0.137	0.467	0.307	0.135	0.514	0.097	-1.181	0.513
22065	0.184	-0.625	0.224	0.102	-1.184	0.478	0.261	-0.137	0.185
22066	0.261	-0.137	0.450	0.102	-1.184	0.462	0.261	-0.137	0.513
22067	0.466	1.178	0.467	0.509	1.434	0.491	0.489	1.329	0.431
22068	0.508	1.447	0.185	0.512	1.453	0.512	0.479	1.262	0.296
22069	0.476	1.245	0.508	0.444	1.020	0.193	0.500	1.397	0.467
22070	0.503	1.413	0.512	0.515	1.472	0.501	0.479	1.262	0.501
22071	0.497	1.380	0.508	0.515	1.472	0.478	0.355	0.470	0.450
22072	0.492	1.346	0.512	0.520	1.509	0.491	0.492	1.346	0.512
22073	0.187	-0.608	0.481	0.401	0.737	0.508	0.329	0.301	0.097
22074	0.503	1.413	0.481	0.491	1.321	0.444	0.513	1.481	0.501
22075	0.479	1.262	0.467	0.515	1.472	0.371	0.495	1.363	0.513
22076	0.511	1.464	0.492	0.512	1.453	0.273	0.447	1.060	0.508
22077	0.508	1.447	0.512	0.512	1.453	0.235	0.447	1.060	0.508
22078	0.500	1.397	0.513	0.512	1.453	0.235	0.358	0.487	0.508

22079	0.224	-0.373	0.508	0.401	0.737	0.149	0.142	-0.895	0.358
22080	0.500	1.397	0.481	0.515	1.472	0.491	0.482	1.279	0.512
22081	0.432	0.958	0.512	0.491	1.321	0.193	0.495	1.363	0.328
22082	0.503	1.413	0.508	0.488	1.302	0.514	0.505	1.430	0.224
22083	0.500	1.397	0.512	0.500	1.378	0.514	0.447	1.060	0.185
22084	0.000	?	0.512	0.401	0.737	0.309	0.261	-0.137	0.409
22085	0.508	1.447	0.501	0.497	1.359	0.371	0.511	1.464	0.097
22086	0.492	1.346	0.481	0.509	1.434	0.508	0.487	1.312	0.508
22087	0.505	1.430	0.508	0.462	1.133	0.512	0.482	1.279	0.328
22088	0.513	1.481	0.296	0.471	1.189	0.193	0.503	1.413	0.050
22089	0.142	-0.895	0.296	0.313	0.172	0.514	0.295	0.082	0.185
22090	0.508	1.447	0.508	0.485	1.283	0.491	0.513	1.481	0.185
22091	0.261	-0.137	0.501	0.275	-0.073	0.235	0.266	-0.103	0.185
22092	0.487	1.312	0.142	0.509	1.434	0.235	0.505	1.430	0.185
22093	0.495	1.363	0.501	0.512	1.453	0.512	0.484	1.295	0.492
22094	0.479	1.262	0.492	0.477	1.227	0.501	0.466	1.178	0.358
22095	0.476	1.245	0.492	0.468	1.170	0.501	0.495	1.363	0.508
22096	0.476	1.245	0.492	0.491	1.321	0.478	0.466	1.178	0.450
22097	0.500	1.397	0.512	0.515	1.472	0.462	0.513	1.481	0.481
22098	0.184	-0.625	0.508	0.196	-0.581	0.462	0.224	-0.373	0.508
22099	0.466	1.178	0.492	0.503	1.396	0.444	0.471	1.211	0.481
22100	0.511	1.464	0.492	0.480	1.246	0.512	0.511	1.464	0.481
22101	0.487	1.312	0.501	0.491	1.321	0.512	0.497	1.380	0.513
22102	0.463	1.161	0.508	0.509	1.434	0.512	0.497	1.380	0.513
22103	0.508	1.447	0.512	0.444	1.020	0.341	0.508	1.447	0.431
22104	0.050	-1.485	0.512	0.374	0.568	0.444	0.295	0.082	0.467
22105	0.000	?	0.481	0.272	-0.091	0.501	0.295	0.082	0.467
22106	0.513	1.481	0.450	0.234	-0.336	0.501	0.497	1.380	0.508
22107	0.142	-0.895	0.492	0.234	-0.336	0.512	0.184	-0.625	0.508
22108	0.187	-0.608	0.492	0.149	-0.882	0.491	0.224	-0.373	0.508
22109	0.500	1.397	0.185	0.491	1.321	0.462	0.466	1.178	0.508
22110	0.145	-0.878	0.512	0.196	-0.581	0.309	0.326	0.285	0.512
22111	0.487	1.312	0.185	0.509	1.434	0.512	0.503	1.413	0.142
22112	0.487	1.312	0.467	0.509	1.434	0.398	0.503	1.413	0.358
22113	0.000	?	0.450	0.307	0.135	0.478	0.224	-0.373	0.512
22114	0.050	-1.485	0.508	0.374	0.568	0.508	0.142	-0.895	0.512
22115	0.484	1.295	0.513	0.512	1.453	0.371	0.503	1.413	0.358
22116	0.503	1.413	0.512	0.512	1.453	0.422	0.355	0.470	0.481
22117	0.050	-1.485	0.358	0.196	-0.581	0.422	0.358	0.487	0.409
22118	0.476	1.245	0.492	0.512	1.453	0.501	0.489	1.329	0.467
22119	0.508	1.447	0.185	0.488	1.302	0.309	0.511	1.464	0.481
22120	0.184	-0.625	0.512	0.234	-0.336	0.508	0.184	-0.625	0.262
22121	0.224	-0.373	0.508	0.234	-0.336	0.398	0.097	-1.181	0.508
22122	0.463	1.161	0.513	0.512	1.453	0.491	0.463	1.161	0.492
22123	0.511	1.464	0.409	0.500	1.378	0.462	0.495	1.363	0.450
22124	0.511	1.464	0.296	0.500	1.378	0.478	0.495	1.363	0.431
22125	0.500	1.397	0.492	0.474	1.208	0.478	0.492	1.346	0.512
22126	0.463	1.161	0.492	0.459	1.114	0.235	0.503	1.413	0.512
22127	0.463	1.161	0.492	0.459	1.114	0.512	0.511	1.464	0.492
22128	0.463	1.161	0.513	0.439	0.982	0.462	0.503	1.413	0.492
22129	0.468	1.194	0.492	0.518	1.491	0.508	0.508	1.447	0.492
22130	0.468	1.194	0.513	0.518	1.491	0.273	0.497	1.380	0.512
22131	0.500	1.397	0.185	0.512	1.453	0.478	0.497	1.380	0.467
22132	0.184	-0.625	0.296	0.342	0.361	0.193	0.224	-0.373	0.501
22133	0.505	1.430	0.492	0.442	1.001	0.462	0.468	1.194	0.513
22134	0.495	1.363	0.296	0.503	1.396	0.512	0.511	1.464	0.409
22135	0.497	1.380	0.508	0.506	1.415	0.512	0.479	1.262	0.450
22136	0.476	1.245	0.328	0.518	1.491	0.501	0.500	1.397	0.492
22137	0.497	1.380	0.501	0.494	1.340	0.501	0.489	1.329	0.508
22138	0.450	1.076	0.501	0.459	1.114	0.422	0.489	1.329	0.508
22139	0.142	-0.895	0.501	0.310	0.153	0.501	0.266	-0.103	0.512

22140	0.434	0.975	0.501	0.512	1.453	0.501	0.500	1.397	0.224
22141	0.434	0.975	0.513	0.395	0.700	0.478	0.497	1.380	0.224
22142	0.492	1.346	0.097	0.477	1.227	0.514	0.508	1.447	0.481
22143	0.500	1.397	0.224	0.506	1.415	0.514	0.505	1.430	0.508
22144	0.513	1.481	0.262	0.374	0.568	0.422	0.513	1.481	0.296
22145	0.497	1.380	0.492	0.424	0.888	0.508	0.508	1.447	0.508
22146	0.497	1.380	0.142	0.424	0.888	0.491	0.508	1.447	0.481
22147	0.511	1.464	0.492	0.500	1.378	0.398	0.500	1.397	0.481
22148	0.266	-0.103	0.097	0.310	0.153	0.478	0.224	-0.373	0.492
22149	0.511	1.464	0.501	0.503	1.396	0.444	0.500	1.397	0.501
22150	0.187	-0.608	0.142	0.398	0.718	0.273	0.297	0.099	0.501
22151	0.505	1.430	0.492	0.491	1.321	0.193	0.508	1.447	0.492
22152	0.511	1.464	0.142	0.459	1.114	0.478	0.489	1.329	0.508
22153	0.511	1.464	0.224	0.477	1.227	0.512	0.455	1.110	0.501
22154	0.511	1.464	0.262	0.477	1.227	0.422	0.455	1.110	0.467
22155	0.184	-0.625	0.262	0.234	-0.336	0.508	0.226	-0.356	0.513
22156	0.511	1.464	0.296	0.512	1.453	0.508	0.479	1.262	0.481
22157	0.489	1.329	0.508	0.459	1.114	0.478	0.508	1.447	0.328
22158	0.484	1.295	0.492	0.506	1.415	0.501	0.495	1.363	0.450
22159	0.145	-0.878	0.224	0.275	-0.073	0.149	0.142	-0.895	0.512
22160	0.500	1.397	0.467	0.477	1.227	0.149	0.500	1.397	0.492
22161	0.187	-0.608	0.185	0.196	-0.581	0.149	0.261	-0.137	0.185
22162	0.511	1.464	0.492	0.462	1.133	0.149	0.500	1.397	0.508
22163	0.500	1.397	0.492	0.509	1.434	0.501	0.484	1.295	0.358
22164	0.500	1.397	0.224	0.509	1.434	0.491	0.471	1.211	0.508
22165	0.511	1.464	0.097	0.497	1.359	0.491	0.492	1.346	0.492
22166	0.511	1.464	0.492	0.497	1.359	0.478	0.492	1.346	0.481
22167	0.050	-1.485	0.501	0.421	0.869	0.508	0.142	-0.895	0.467
22168	0.500	1.397	0.508	0.506	1.415	0.508	0.489	1.329	0.431
22169	0.505	1.430	0.508	0.494	1.340	0.235	0.508	1.447	0.050
22170	0.505	1.430	0.481	0.471	1.189	0.193	0.508	1.447	0.358
22171	0.500	1.397	0.481	0.512	1.453	0.491	0.476	1.245	0.097
22172	0.500	1.397	0.450	0.512	1.453	0.501	0.476	1.245	0.492
22173	0.511	1.464	0.328	0.421	0.869	0.462	0.511	1.464	0.385
22174	0.361	0.504	0.224	0.503	1.396	0.491	0.511	1.464	0.492
22175	0.463	1.161	0.467	0.494	1.340	0.491	0.503	1.413	0.512
22176	0.355	0.470	0.508	0.401	0.737	0.491	0.303	0.133	0.328
22177	0.476	1.245	0.262	0.474	1.208	0.514	0.505	1.430	0.512
22178	0.508	1.447	0.328	0.439	0.982	0.501	0.511	1.464	0.512
22179	0.392	0.706	0.328	0.272	-0.091	0.444	0.224	-0.373	0.513
22180	0.300	0.116	0.262	0.193	-0.600	0.462	0.261	-0.137	0.508
22181	0.505	1.430	0.467	0.480	1.246	0.514	0.500	1.397	0.492
22182	0.479	1.262	0.296	0.515	1.472	0.422	0.429	0.942	0.513
22183	0.508	1.447	0.358	0.424	0.888	0.501	0.500	1.397	0.409
22184	0.466	1.178	0.512	0.515	1.472	0.149	0.511	1.464	0.262
22185	0.466	1.178	0.512	0.515	1.472	0.149	0.511	1.464	0.467
22186	0.503	1.413	0.512	0.477	1.227	0.193	0.445	1.043	0.296
22187	0.434	0.975	0.512	0.497	1.359	0.478	0.513	1.481	0.492
22188	0.361	0.504	0.512	0.149	-0.882	0.235	0.000	?	0.050
22189	0.387	0.672	0.513	0.149	-0.882	0.309	0.000	?	0.501
22190	0.387	0.672	0.050	0.149	-0.882	0.501	0.000	?	0.501
22191	0.387	0.672	0.185	0.149	-0.882	0.508	0.000	?	0.501
22192	0.503	1.413	0.492	0.506	1.415	0.444	0.513	1.481	0.501
22193	0.511	1.464	0.512	0.494	1.340	0.508	0.500	1.397	0.512
22194	0.505	1.430	0.512	0.491	1.321	0.508	0.508	1.447	0.262
22195	0.492	1.346	0.481	0.477	1.227	0.508	0.405	0.790	0.328
22196	0.505	1.430	0.492	0.506	1.415	0.491	0.508	1.447	0.142
22197	0.505	1.430	0.481	0.506	1.415	0.512	0.479	1.262	0.467
22198	0.295	0.082	0.097	0.237	-0.317	0.478	0.261	-0.137	0.224
22199	0.332	0.318	0.508	0.193	-0.600	0.273	0.453	1.093	0.512
22200	0.445	1.043	0.296	0.488	1.302	0.273	0.511	1.464	0.185

22201	0.476	1.245	0.097	0.500	1.378	0.273	0.492	1.346	0.467
22202	0.508	1.447	0.358	0.465	1.152	0.512	0.503	1.413	0.224
22203	0.500	1.397	0.262	0.494	1.340	0.512	0.497	1.380	0.467
22204	0.447	1.060	0.481	0.491	1.321	0.512	0.492	1.346	0.296
22205	0.447	1.060	0.224	0.491	1.321	0.501	0.492	1.346	0.262
22206	0.489	1.329	0.358	0.509	1.434	0.501	0.495	1.363	0.262
22207	0.492	1.346	0.508	0.503	1.396	0.193	0.503	1.413	0.185
22208	0.445	1.043	0.508	0.447	1.039	0.508	0.489	1.329	0.296
22209	0.505	1.430	0.508	0.462	1.133	0.422	0.326	0.285	0.467
22210	0.405	0.790	0.142	0.515	1.472	0.341	0.479	1.262	0.508
22211	0.000	?	0.385	0.427	0.907	0.309	0.224	-0.373	0.224
22212	0.511	1.464	0.508	0.494	1.340	0.149	0.482	1.279	0.296
22213	0.224	-0.373	0.508	0.149	-0.882	0.491	0.326	0.285	0.512
22214	0.224	-0.373	0.501	0.149	-0.882	0.478	0.224	-0.373	0.296
22215	0.184	-0.625	0.508	0.196	-0.581	0.514	0.358	0.487	0.501
22216	0.463	1.161	0.492	0.477	1.227	0.508	0.326	0.285	0.501
22217	0.295	0.082	0.512	0.234	-0.336	0.501	0.226	-0.356	0.262
22218	0.142	-0.895	0.513	0.310	0.153	0.501	0.226	-0.356	0.296
22219	0.495	1.363	0.142	0.500	1.378	0.508	0.500	1.397	0.513
22220	0.500	1.397	0.296	0.509	1.434	0.149	0.453	1.093	0.508
22221	0.450	1.076	0.481	0.444	1.020	0.273	0.479	1.262	0.492
22222	0.489	1.329	0.508	0.509	1.434	0.508	0.508	1.447	0.467
22223	0.500	1.397	0.513	0.500	1.378	0.508	0.495	1.363	0.508
22224	0.511	1.464	0.467	0.500	1.378	0.193	0.516	1.498	0.508
22225	0.503	1.413	0.450	0.500	1.378	0.398	0.503	1.413	0.508
22226	0.505	1.430	0.508	0.506	1.415	0.508	0.503	1.413	0.385
22227	0.463	1.161	0.508	0.474	1.208	0.508	0.466	1.178	0.328
22228	0.297	0.099	0.050	0.275	-0.073	0.508	0.297	0.099	0.513
22229	0.297	0.099	0.050	0.275	-0.073	0.491	0.297	0.099	0.481
22230	0.297	0.099	0.450	0.275	-0.073	0.491	0.332	0.318	0.328
22231	0.497	1.380	0.142	0.512	1.453	0.512	0.513	1.481	0.185
22232	0.497	1.380	0.296	0.512	1.453	0.371	0.513	1.481	0.224
22233	0.497	1.380	0.481	0.512	1.453	0.309	0.513	1.481	0.097
22234	0.497	1.380	0.512	0.500	1.378	0.309	0.513	1.481	0.501
22235	0.497	1.380	0.501	0.500	1.378	0.309	0.513	1.481	0.050
22236	0.332	0.318	0.501	0.193	-0.600	0.102	0.261	-0.137	0.512
22237	0.489	1.329	0.358	0.509	1.434	0.512	0.466	1.178	0.501
22238	0.497	1.380	0.262	0.424	0.888	0.512	0.505	1.430	0.501
22239	0.145	-0.878	0.492	0.342	0.361	0.235	0.295	0.082	0.512
22240	0.145	-0.878	0.328	0.307	0.135	0.512	0.326	0.285	0.481
22241	0.184	-0.625	0.224	0.149	-0.882	0.422	0.224	-0.373	0.481
22242	0.500	1.397	0.185	0.494	1.340	0.273	0.511	1.464	0.492
22243	0.503	1.413	0.262	0.480	1.246	0.235	0.466	1.178	0.142
22244	0.497	1.380	0.481	0.515	1.472	0.508	0.508	1.447	0.262
22245	0.492	1.346	0.508	0.512	1.453	0.478	0.513	1.481	0.409
22246	0.492	1.346	0.492	0.506	1.415	0.478	0.513	1.481	0.296
22247	0.497	1.380	0.492	0.503	1.396	0.235	0.511	1.464	0.296
22248	0.503	1.413	0.142	0.509	1.434	0.309	0.479	1.262	0.296
22249	0.329	0.301	0.508	0.149	-0.882	0.491	0.184	-0.625	0.492
22250	0.224	-0.373	0.492	0.272	-0.091	0.491	0.097	-1.181	0.508
22251	0.447	1.060	0.262	0.509	1.434	0.422	0.511	1.464	0.508
22252	0.476	1.245	0.508	0.506	1.415	0.235	0.513	1.481	0.508
22253	0.226	-0.356	0.492	0.196	-0.581	0.501	0.142	-0.895	0.492
22254	0.050	-1.485	0.501	0.398	0.718	0.514	0.097	-1.181	0.224
22255	0.505	1.430	0.508	0.506	1.415	0.273	0.471	1.211	0.508
22256	0.505	1.430	0.501	0.506	1.415	0.508	0.474	1.228	0.185
22257	0.505	1.430	0.185	0.506	1.415	0.478	0.487	1.312	0.224
22258	0.505	1.430	0.508	0.491	1.321	0.491	0.513	1.481	0.262
22259	0.505	1.430	0.262	0.491	1.321	0.444	0.513	1.481	0.296
22260	0.489	1.329	0.296	0.512	1.453	0.235	0.489	1.329	0.501
22261	0.000	?	0.513	0.371	0.549	0.491	0.187	-0.608	0.185

22262	0.050	-1.485	0.050	0.310	0.153	0.398	0.187	-0.608	0.224
22263	0.297	0.099	0.508	0.307	0.135	0.508	0.387	0.672	0.328
22264	0.261	-0.137	0.185	0.307	0.135	0.508	0.261	-0.137	0.512
22265	0.261	-0.137	0.296	0.102	-1.184	0.508	0.411	0.824	0.512
22266	0.482	1.279	0.512	0.512	1.453	0.444	0.495	1.363	0.501
22267	0.511	1.464	0.431	0.509	1.434	0.512	0.468	1.194	0.358
22268	0.297	0.099	0.512	0.237	-0.317	0.273	0.000	?	0.512
22269	0.463	1.161	0.501	0.509	1.434	0.273	0.453	1.093	0.492
22270	0.492	1.346	0.481	0.421	0.869	0.273	0.489	1.329	0.492
22271	0.187	-0.608	0.450	0.275	-0.073	0.478	0.142	-0.895	0.262
22272	0.329	0.301	0.450	0.237	-0.317	0.235	0.142	-0.895	0.508
22273	0.384	0.655	0.142	0.506	1.415	0.508	0.468	1.194	0.467
22274	0.482	1.279	0.512	0.477	1.227	0.235	0.511	1.464	0.512
22275	0.482	1.279	0.385	0.477	1.227	0.371	0.511	1.464	0.450
22276	0.187	-0.608	0.501	0.237	-0.317	0.309	0.224	-0.373	0.450
22277	0.097	-1.181	0.508	0.310	0.153	0.273	0.300	0.116	0.262
22278	0.497	1.380	0.508	0.497	1.359	0.273	0.513	1.481	0.097
22279	0.508	1.447	0.481	0.491	1.321	0.273	0.489	1.329	0.450
22280	0.505	1.430	0.262	0.421	0.869	0.512	0.513	1.481	0.508
22281	0.261	-0.137	0.358	0.234	-0.336	0.309	0.187	-0.608	0.508
22282	0.489	1.329	0.296	0.497	1.359	0.508	0.511	1.464	0.508
22283	0.489	1.329	0.224	0.512	1.453	0.501	0.429	0.942	0.513
22284	0.263	-0.120	0.142	0.275	-0.073	0.398	0.297	0.099	0.481
22285	0.479	1.262	0.501	0.512	1.453	0.508	0.463	1.161	0.513
22286	0.511	1.464	0.409	0.477	1.227	0.478	0.503	1.413	0.142
22287	0.479	1.262	0.185	0.488	1.302	0.491	0.455	1.110	0.142
22288	0.474	1.228	0.409	0.442	1.001	0.512	0.511	1.464	0.492
22289	0.263	-0.120	0.431	0.237	-0.317	0.501	0.097	-1.181	0.097
22290	0.503	1.413	0.450	0.488	1.302	0.512	0.503	1.413	0.296
22291	0.489	1.329	0.512	0.401	0.737	0.512	0.513	1.481	0.481
22292	0.466	1.178	0.296	0.500	1.378	0.491	0.505	1.430	0.467
22293	0.466	1.178	0.512	0.500	1.378	0.478	0.505	1.430	0.481
22294	0.466	1.178	0.296	0.500	1.378	0.444	0.505	1.430	0.492
22295	0.511	1.464	0.385	0.447	1.039	0.501	0.358	0.487	0.358
22296	0.508	1.447	0.262	0.512	1.453	0.478	0.505	1.430	0.262
22297	0.000	?	0.501	0.275	-0.073	0.478	0.329	0.301	0.508
22298	0.224	-0.373	0.262	0.272	-0.091	0.341	0.226	-0.356	0.097
22299	0.295	0.082	0.481	0.272	-0.091	0.235	0.184	-0.625	0.224
22300	0.503	1.413	0.358	0.477	1.227	0.514	0.405	0.790	0.508
22301	0.329	0.301	0.185	0.234	-0.336	0.514	0.097	-1.181	0.492
22302	0.492	1.346	0.508	0.509	1.434	0.309	0.503	1.413	0.512
22303	0.261	-0.137	0.185	0.234	-0.336	0.512	0.326	0.285	0.512
22304	0.184	-0.625	0.431	0.371	0.549	0.149	0.050	-1.485	0.358
22305	0.145	-0.878	0.492	0.307	0.135	0.478	0.184	-0.625	0.431
22306	0.145	-0.878	0.492	0.272	-0.091	0.491	0.184	-0.625	0.431
22307	0.145	-0.878	0.501	0.272	-0.091	0.193	0.184	-0.625	0.262
22308	0.145	-0.878	0.097	0.272	-0.091	0.491	0.184	-0.625	0.492
22309	0.487	1.312	0.467	0.515	1.472	0.514	0.497	1.380	0.512
22310	0.184	-0.625	0.328	0.310	0.153	0.491	0.358	0.487	0.467
22311	0.489	1.329	0.296	0.503	1.396	0.478	0.511	1.464	0.467
22312	0.384	0.655	0.450	0.500	1.378	0.508	0.450	1.076	0.467
22313	0.463	1.161	0.512	0.398	0.718	0.508	0.482	1.279	0.224
22314	0.429	0.942	0.224	0.506	1.415	0.462	0.505	1.430	0.508
22315	0.411	0.824	0.513	0.474	1.208	0.478	0.476	1.245	0.097
22316	0.516	1.498	0.431	0.488	1.302	0.512	0.476	1.245	0.385
22317	0.503	1.413	0.097	0.512	1.453	0.478	0.508	1.447	0.431
22318	0.503	1.413	0.185	0.500	1.378	0.514	0.508	1.447	0.097
22319	0.445	1.043	0.501	0.515	1.472	0.512	0.434	0.975	0.508
22320	0.405	0.790	0.467	0.515	1.472	0.508	0.471	1.211	0.358
22321	0.495	1.363	0.467	0.488	1.302	0.501	0.471	1.211	0.481
22322	0.432	0.958	0.501	0.474	1.208	0.514	0.505	1.430	0.512

22323	0.489	1.329	0.185	0.444	1.020	0.512	0.508	1.447	0.513
22324	0.479	1.262	0.185	0.497	1.359	0.478	0.508	1.447	0.467
22325	0.447	1.060	0.492	0.480	1.246	0.371	0.508	1.447	0.450
22326	0.463	1.161	0.481	0.477	1.227	0.398	0.513	1.481	0.512
22327	0.050	-1.485	0.185	0.339	0.342	0.149	0.142	-0.895	0.512
22328	0.297	0.099	0.097	0.237	-0.317	0.149	0.361	0.504	0.097
22329	0.487	1.312	0.450	0.515	1.472	0.508	0.508	1.447	0.508
22330	0.487	1.312	0.512	0.515	1.472	0.512	0.508	1.447	0.142
22331	0.050	-1.485	0.050	0.310	0.153	0.149	0.361	0.504	0.512
22332	0.461	1.144	0.481	0.512	1.453	0.491	0.484	1.295	0.513
22333	0.226	-0.356	0.492	0.149	-0.882	0.514	0.411	0.824	0.513
22334	0.453	1.093	0.185	0.477	1.227	0.514	0.466	1.178	0.467
22335	0.468	1.194	0.512	0.488	1.302	0.514	0.482	1.279	0.224
22336	0.184	-0.625	0.450	0.193	-0.600	0.422	0.266	-0.103	0.513
22337	0.497	1.380	0.492	0.494	1.340	0.501	0.505	1.430	0.224
22338	0.513	1.481	0.450	0.512	1.453	0.491	0.484	1.295	0.328
22339	0.497	1.380	0.481	0.494	1.340	0.508	0.447	1.060	0.328
22340	0.503	1.413	0.262	0.480	1.246	0.512	0.429	0.942	0.431
22341	0.479	1.262	0.513	0.506	1.415	0.508	0.511	1.464	0.224
22342	0.479	1.262	0.513	0.506	1.415	0.508	0.511	1.464	0.142
22343	0.468	1.194	0.481	0.462	1.133	0.514	0.484	1.295	0.512
22344	0.489	1.329	0.467	0.485	1.283	0.508	0.497	1.380	0.512
22345	0.505	1.430	0.467	0.512	1.453	0.508	0.492	1.346	0.512
22346	0.508	1.447	0.467	0.477	1.227	0.514	0.513	1.481	0.512
22347	0.505	1.430	0.492	0.512	1.453	0.514	0.468	1.194	0.508
22348	0.476	1.245	0.492	0.509	1.434	0.398	0.505	1.430	0.097
22349	0.508	1.447	0.501	0.506	1.415	0.501	0.511	1.464	0.097
22350	0.497	1.380	0.492	0.500	1.378	0.273	0.411	0.824	0.262
22351	0.513	1.481	0.481	0.512	1.453	0.512	0.450	1.076	0.508
22352	0.513	1.481	0.467	0.509	1.434	0.508	0.489	1.329	0.296
22353	0.503	1.413	0.501	0.477	1.227	0.462	0.508	1.447	0.501
22354	0.479	1.262	0.481	0.371	0.549	0.501	0.508	1.447	0.050
22355	0.511	1.464	0.431	0.395	0.700	0.501	0.508	1.447	0.501
22356	0.297	0.099	0.450	0.149	-0.882	0.309	0.226	-0.356	0.185
22357	0.297	0.099	0.508	0.149	-0.882	0.508	0.226	-0.356	0.142
22358	0.513	1.481	0.508	0.506	1.415	0.398	0.479	1.262	0.450
22359	0.508	1.447	0.262	0.509	1.434	0.508	0.505	1.430	0.262
22360	0.142	-0.895	0.262	0.149	-0.882	0.514	0.295	0.082	0.431
22361	0.503	1.413	0.450	0.494	1.340	0.478	0.511	1.464	0.512
22362	0.489	1.329	0.492	0.515	1.472	0.478	0.474	1.228	0.512
22363	0.489	1.329	0.262	0.515	1.472	0.193	0.474	1.228	0.492
22364	0.489	1.329	0.508	0.515	1.472	0.398	0.484	1.295	0.296
22365	0.503	1.413	0.508	0.421	0.869	0.398	0.505	1.430	0.508
22366	0.500	1.397	0.050	0.500	1.378	0.444	0.505	1.430	0.262
22367	0.495	1.363	0.501	0.494	1.340	0.444	0.495	1.363	0.185
22368	0.495	1.363	0.513	0.512	1.453	0.422	0.511	1.464	0.501
22369	0.503	1.413	0.185	0.515	1.472	0.444	0.505	1.430	0.508
22370	0.505	1.430	0.467	0.509	1.434	0.273	0.466	1.178	0.185
22371	0.508	1.447	0.224	0.500	1.378	0.273	0.516	1.498	0.481
22372	0.508	1.447	0.224	0.506	1.415	0.422	0.482	1.279	0.501
22373	0.482	1.279	0.501	0.512	1.453	0.273	0.332	0.318	0.328
22374	0.450	1.076	0.492	0.512	1.453	0.478	0.447	1.060	0.142
22375	0.495	1.363	0.097	0.518	1.491	0.235	0.513	1.481	0.467
22376	0.484	1.295	0.513	0.515	1.472	0.514	0.489	1.329	0.501
22377	0.184	-0.625	0.512	0.404	0.756	0.514	0.184	-0.625	0.450
22378	0.503	1.413	0.513	0.503	1.396	0.514	0.516	1.498	0.431
22379	0.187	-0.608	0.501	0.275	-0.073	0.514	0.358	0.487	0.296
22380	0.461	1.144	0.262	0.515	1.472	0.501	0.503	1.413	0.185
22381	0.445	1.043	0.262	0.509	1.434	0.235	0.487	1.312	0.467
22382	0.508	1.447	0.262	0.459	1.114	0.309	0.476	1.245	0.512
22383	0.508	1.447	0.262	0.500	1.378	0.273	0.466	1.178	0.224

22384	0.513	1.481	0.262	0.500	1.378	0.512	0.426	0.925	0.097
22385	0.358	0.487	0.512	0.307	0.135	0.193	0.050	-1.485	0.508
22386	0.489	1.329	0.262	0.509	1.434	0.508	0.358	0.487	0.431
22387	0.184	-0.625	0.224	0.404	0.756	0.273	0.097	-1.181	0.262
22388	0.513	1.481	0.185	0.506	1.415	0.478	0.492	1.346	0.508
22389	0.511	1.464	0.185	0.509	1.434	0.309	0.384	0.655	0.501
22390	0.518	1.514	0.450	0.482	1.265	0.514	0.492	1.346	0.296
22391	0.413	0.841	0.185	0.485	1.283	0.193	0.508	1.447	0.492
22392	0.300	0.116	0.481	0.193	-0.600	0.193	0.326	0.285	0.142
22393	0.489	1.329	0.224	0.398	0.718	0.149	0.516	1.498	0.481
22394	0.489	1.329	0.262	0.398	0.718	0.309	0.516	1.498	0.492
22395	0.489	1.329	0.142	0.442	1.001	0.149	0.516	1.498	0.512
22396	0.516	1.498	0.450	0.444	1.020	0.508	0.508	1.447	0.385
22397	0.495	1.363	0.142	0.421	0.869	0.508	0.492	1.346	0.450
22398	0.513	1.481	0.481	0.442	1.001	0.341	0.513	1.481	0.467
22399	0.184	-0.625	0.142	0.272	-0.091	0.235	0.408	0.807	0.328
22400	0.300	0.116	0.142	0.272	-0.091	0.462	0.261	-0.137	0.508
22401	0.492	1.346	0.481	0.424	0.888	0.341	0.463	1.161	0.508
22402	0.297	0.099	0.142	0.272	-0.091	0.235	0.295	0.082	0.508
22403	0.505	1.430	0.185	0.477	1.227	0.462	0.489	1.329	0.513
22404	0.332	0.318	0.467	0.234	-0.336	0.462	0.050	-1.485	0.512
22405	0.497	1.380	0.097	0.512	1.453	0.371	0.497	1.380	0.501
22406	0.497	1.380	0.501	0.512	1.453	0.193	0.497	1.380	0.450
22407	0.497	1.380	0.467	0.512	1.453	0.478	0.497	1.380	0.481
22408	0.497	1.380	0.467	0.512	1.453	0.508	0.497	1.380	0.512
22409	0.503	1.413	0.513	0.503	1.396	0.512	0.511	1.464	0.512
22410	0.100	-1.164	0.508	0.234	-0.336	0.512	0.261	-0.137	0.508
22411	0.226	-0.356	0.513	0.313	0.172	0.478	0.326	0.285	0.513
22412	0.261	-0.137	0.492	0.275	-0.073	0.478	0.142	-0.895	0.501
22413	0.495	1.363	0.296	0.515	1.472	0.422	0.463	1.161	0.501
22414	0.142	-0.895	0.467	0.193	-0.600	0.273	0.226	-0.356	0.501
22415	0.492	1.346	0.508	0.503	1.396	0.273	0.508	1.447	0.185
22416	0.097	-1.181	0.262	0.275	-0.073	0.512	0.187	-0.608	0.097
22417	0.503	1.413	0.512	0.477	1.227	0.462	0.466	1.178	0.467
22418	0.142	-0.895	0.409	0.307	0.135	0.149	0.224	-0.373	0.431
22419	0.495	1.363	0.512	0.515	1.472	0.309	0.463	1.161	0.097
22420	0.142	-0.895	0.467	0.196	-0.581	0.309	0.295	0.082	0.481
22421	0.224	-0.373	0.262	0.196	-0.581	0.273	0.261	-0.137	0.450
22422	0.261	-0.137	0.450	0.149	-0.882	0.462	0.261	-0.137	0.185
22423	0.263	-0.120	0.185	0.313	0.172	0.273	0.184	-0.625	0.501
22424	0.300	0.116	0.185	0.149	-0.882	0.491	0.295	0.082	0.409
22425	0.497	1.380	0.296	0.509	1.434	0.508	0.466	1.178	0.262
22426	0.492	1.346	0.501	0.506	1.415	0.478	0.503	1.413	0.501
22427	0.000	?	0.492	0.342	0.361	0.508	0.224	-0.373	0.296
22428	0.224	-0.373	0.492	0.234	-0.336	0.508	0.297	0.099	0.142
22429	0.463	1.161	0.431	0.459	1.114	0.508	0.511	1.464	0.097
22430	0.187	-0.608	0.492	0.342	0.361	0.501	0.000	?	0.467
22431	0.000	?	0.262	0.237	-0.317	0.371	0.297	0.099	0.467
22432	0.492	1.346	0.512	0.462	1.133	0.102	0.500	1.397	0.296
22433	0.492	1.346	0.097	0.462	1.133	0.491	0.500	1.397	0.450
22434	0.226	-0.356	0.508	0.371	0.549	0.235	0.263	-0.120	0.508
22435	0.097	-1.181	0.224	0.193	-0.600	0.235	0.297	0.099	0.481
22436	0.492	1.346	0.467	0.477	1.227	0.235	0.505	1.430	0.492
22437	0.503	1.413	0.431	0.506	1.415	0.508	0.500	1.397	0.501
22438	0.500	1.397	0.050	0.515	1.472	0.512	0.495	1.363	0.142
22439	0.500	1.397	0.450	0.515	1.472	0.508	0.468	1.194	0.142
22440	0.482	1.279	0.513	0.477	1.227	0.514	0.505	1.430	0.262
22441	0.482	1.279	0.508	0.477	1.227	0.508	0.505	1.430	0.262
22442	0.447	1.060	0.409	0.424	0.888	0.235	0.508	1.447	0.262
22443	0.329	0.301	0.409	0.275	-0.073	0.501	0.387	0.672	0.467
22444	0.224	-0.373	0.142	0.275	-0.073	0.371	0.332	0.318	0.050

22445	0.461	1.144	0.431	0.515	1.472	0.235	0.513	1.481	0.050
22446	0.508	1.447	0.358	0.459	1.114	0.309	0.474	1.228	0.262
22447	0.263	-0.120	0.508	0.149	-0.882	0.149	0.329	0.301	0.185
22448	0.332	0.318	0.508	0.310	0.153	0.514	0.184	-0.625	0.467
22449	0.329	0.301	0.142	0.310	0.153	0.149	0.224	-0.373	0.185
22450	0.261	-0.137	0.142	0.275	-0.073	0.422	0.097	-1.181	0.513
22451	0.471	1.211	0.328	0.459	1.114	0.235	0.503	1.413	0.050
22452	0.295	0.082	0.431	0.275	-0.073	0.491	0.050	-1.485	0.296
22453	0.355	0.470	0.142	0.497	1.359	0.491	0.503	1.413	0.185
22454	0.500	1.397	0.328	0.509	1.434	0.508	0.505	1.430	0.508
22455	0.500	1.397	0.501	0.480	1.246	0.149	0.503	1.413	0.185
22456	0.500	1.397	0.050	0.509	1.434	0.478	0.516	1.498	0.431
22457	0.505	1.430	0.481	0.506	1.415	0.508	0.484	1.295	0.224
22458	0.505	1.430	0.508	0.506	1.415	0.508	0.484	1.295	0.224
22459	0.505	1.430	0.185	0.500	1.378	0.309	0.492	1.346	0.501
22460	0.050	-1.485	0.185	0.368	0.530	0.491	0.142	-0.895	0.142
22461	0.184	-0.625	0.142	0.102	-1.184	0.501	0.263	-0.120	0.142
22462	0.492	1.346	0.358	0.491	1.321	0.398	0.413	0.841	0.508
22463	0.000	?	0.185	0.237	-0.317	0.514	0.297	0.099	0.262
22464	0.000	?	0.501	0.237	-0.317	0.512	0.297	0.099	0.512
22465	0.000	?	0.050	0.237	-0.317	0.149	0.297	0.099	0.512
22466	0.503	1.413	0.513	0.515	1.472	0.235	0.492	1.346	0.501
22467	0.508	1.447	0.508	0.506	1.415	0.512	0.508	1.447	0.467
22468	0.476	1.245	0.262	0.503	1.396	0.501	0.508	1.447	0.450
22469	0.492	1.346	0.142	0.518	1.491	0.501	0.508	1.447	0.501
22470	0.479	1.262	0.185	0.509	1.434	0.478	0.489	1.329	0.467
22471	0.100	-1.164	0.185	0.237	-0.317	0.508	0.224	-0.373	0.097
22472	0.500	1.397	0.185	0.500	1.378	0.309	0.511	1.464	0.481
22473	0.297	0.099	0.492	0.371	0.549	0.371	0.187	-0.608	0.481
22474	0.097	-1.181	0.492	0.234	-0.336	0.422	0.224	-0.373	0.142
22475	0.358	0.487	0.508	0.307	0.135	0.371	0.261	-0.137	0.481
22476	0.261	-0.137	0.513	0.149	-0.882	0.508	0.295	0.082	0.513
22477	0.479	1.262	0.467	0.506	1.415	0.341	0.503	1.413	0.501
22478	0.224	-0.373	0.467	0.149	-0.882	0.422	0.184	-0.625	0.431
22479	0.358	0.487	0.097	0.427	0.907	0.501	0.224	-0.373	0.185
22480	0.000	?	0.501	0.237	-0.317	0.491	0.329	0.301	0.508
22481	0.497	1.380	0.492	0.491	1.321	0.501	0.516	1.498	0.296
22482	0.497	1.380	0.501	0.491	1.321	0.478	0.516	1.498	0.296
22483	0.508	1.447	0.508	0.512	1.453	0.309	0.503	1.413	0.224
22484	0.142	-0.895	0.481	0.149	-0.882	0.102	0.358	0.487	0.450
22485	0.387	0.672	0.492	0.480	1.246	0.491	0.508	1.447	0.492
22486	0.503	1.413	0.431	0.509	1.434	0.102	0.492	1.346	0.501
22487	0.503	1.413	0.512	0.509	1.434	0.422	0.492	1.346	0.512
22488	0.000	?	0.508	0.313	0.172	0.341	0.261	-0.137	0.501
22489	0.495	1.363	0.512	0.485	1.283	0.273	0.511	1.464	0.142
22490	0.505	1.430	0.501	0.500	1.378	0.478	0.468	1.194	0.450
22491	0.489	1.329	0.513	0.395	0.700	0.478	0.511	1.464	0.262
22492	0.505	1.430	0.450	0.515	1.472	0.478	0.450	1.076	0.431
22493	0.508	1.447	0.431	0.512	1.453	0.478	0.450	1.076	0.385
22494	0.145	-0.878	0.185	0.149	-0.882	0.309	0.261	-0.137	0.501
22495	0.295	0.082	0.481	0.237	-0.317	0.501	0.097	-1.181	0.501
22496	0.482	1.279	0.512	0.509	1.434	0.508	0.453	1.093	0.385
22497	0.508	1.447	0.050	0.497	1.359	0.193	0.511	1.464	0.508
22498	0.513	1.481	0.050	0.497	1.359	0.444	0.511	1.464	0.492
22499	0.466	1.178	0.512	0.480	1.246	0.512	0.508	1.447	0.467
22500	0.447	1.060	0.492	0.509	1.434	0.501	0.505	1.430	0.358
22501	0.505	1.430	0.450	0.310	0.153	0.512	0.482	1.279	0.431
22502	0.505	1.430	0.328	0.374	0.568	0.444	0.511	1.464	0.142
22503	0.050	-1.485	0.409	0.418	0.850	0.371	0.142	-0.895	0.508
22504	0.050	-1.485	0.262	0.371	0.549	0.462	0.142	-0.895	0.467
22505	0.450	1.076	0.097	0.503	1.396	0.193	0.492	1.346	0.409

22506	0.142	−0.895	0.409	0.339	0.342	0.235	0.097	−1.181	0.409
22507	0.297	0.099	0.409	0.421	0.869	0.512	0.295	0.082	0.328
22508	0.484	1.295	0.481	0.497	1.359	0.371	0.484	1.295	0.296
22509	0.505	1.430	0.481	0.494	1.340	0.514	0.461	1.144	0.142
22510	0.500	1.397	0.512	0.497	1.359	0.102	0.479	1.262	0.501
22511	0.497	1.380	0.508	0.480	1.246	0.273	0.487	1.312	0.185
22512	0.361	0.504	0.262	0.310	0.153	0.501	0.361	0.504	0.050
22513	0.263	−0.120	0.050	0.102	−1.184	0.508	0.261	−0.137	0.262
22514	0.492	1.346	0.097	0.494	1.340	0.444	0.500	1.397	0.467
22515	0.326	0.285	0.431	0.105	−1.165	0.508	0.100	−1.164	0.185
22516	0.224	−0.373	0.492	0.430	0.926	0.512	0.224	−0.373	0.481
22517	0.184	−0.625	0.492	0.345	0.379	0.508	0.000	?	0.512
22518	0.261	−0.137	0.508	0.272	−0.091	0.508	0.000	?	0.185
22519	0.476	1.245	0.224	0.477	1.227	0.309	0.505	1.430	0.185
22520	0.508	1.447	0.185	0.477	1.227	0.462	0.495	1.363	0.185
22521	0.492	1.346	0.262	0.471	1.189	0.053	0.511	1.464	0.358
22522	0.492	1.346	0.431	0.471	1.189	0.514	0.511	1.464	0.185
22523	0.145	−0.878	0.296	0.307	0.135	0.501	0.358	0.487	0.508
22524	0.511	1.464	0.513	0.494	1.340	0.491	0.429	0.942	0.296
22525	0.495	1.363	0.481	0.503	1.396	0.491	0.429	0.942	0.450
22526	0.266	−0.103	0.492	0.193	−0.600	0.235	0.261	−0.137	0.481
22527	0.511	1.464	0.508	0.439	0.982	0.508	0.489	1.329	0.262
22528	0.492	1.346	0.224	0.509	1.434	0.235	0.511	1.464	0.328
22529	0.492	1.346	0.450	0.503	1.396	0.193	0.466	1.178	0.262
22530	0.500	1.397	0.262	0.515	1.472	0.193	0.463	1.161	0.185
22531	0.492	1.346	0.224	0.447	1.039	0.478	0.466	1.178	0.262
22532	0.184	−0.625	0.492	0.371	0.549	0.193	0.224	−0.373	0.492
22533	0.505	1.430	0.492	0.462	1.133	0.341	0.505	1.430	0.512
22534	0.263	−0.120	0.513	0.193	−0.600	0.508	0.097	−1.181	0.512
22535	0.300	0.116	0.224	0.237	−0.317	0.501	0.382	0.638	0.501
22536	0.500	1.397	0.358	0.515	1.472	0.501	0.432	0.958	0.508
22537	0.050	−1.485	0.481	0.371	0.549	0.478	0.097	−1.181	0.508
22538	0.505	1.430	0.492	0.509	1.434	0.235	0.511	1.464	0.142
22539	0.187	−0.608	0.512	0.102	−1.184	0.462	0.358	0.487	0.431
22540	0.297	0.099	0.512	0.272	−0.091	0.398	0.000	?	0.501
22541	0.511	1.464	0.142	0.497	1.359	0.398	0.476	1.245	0.501
22542	0.426	0.925	0.224	0.509	1.434	0.149	0.505	1.430	0.512
22543	0.508	1.447	0.328	0.447	1.039	0.478	0.508	1.447	0.508
22544	0.497	1.380	0.296	0.506	1.415	0.102	0.463	1.161	0.508
22545	0.479	1.262	0.050	0.515	1.472	0.422	0.445	1.043	0.450
22546	0.450	1.076	0.512	0.503	1.396	0.309	0.508	1.447	0.481
22547	0.450	1.076	0.385	0.503	1.396	0.444	0.508	1.447	0.481
22548	0.142	−0.895	0.224	0.310	0.153	0.273	0.097	−1.181	0.501
22549	0.503	1.413	0.385	0.462	1.133	0.235	0.508	1.447	0.508
22550	0.387	0.672	0.224	0.053	−1.504	0.501	0.142	−0.895	0.467
22551	0.500	1.397	0.185	0.515	1.472	0.462	0.508	1.447	0.513
22552	0.505	1.430	0.512	0.500	1.378	0.508	0.508	1.447	0.512
22553	0.505	1.430	0.185	0.491	1.321	0.444	0.508	1.447	0.224
22554	0.482	1.279	0.185	0.488	1.302	0.512	0.466	1.178	0.512
22555	0.263	−0.120	0.224	0.237	−0.317	0.462	0.224	−0.373	0.513
22556	0.358	0.487	0.481	0.506	1.415	0.371	0.511	1.464	0.328
22557	0.297	0.099	0.431	0.237	−0.317	0.512	0.224	−0.373	0.385
22558	0.226	−0.356	0.481	0.193	−0.600	0.309	0.326	0.285	0.262
22559	0.142	−0.895	0.224	0.193	−0.600	0.193	0.326	0.285	0.501
22560	0.495	1.363	0.512	0.480	1.246	0.444	0.432	0.958	0.508
22561	0.413	0.841	0.513	0.193	−0.600	0.512	0.224	−0.373	0.467
22562	0.184	−0.625	0.492	0.345	0.379	0.149	0.142	−0.895	0.328
22563	0.408	0.807	0.512	0.500	1.378	0.478	0.511	1.464	0.501
22564	0.432	0.958	0.467	0.494	1.340	0.508	0.511	1.464	0.296
22565	0.450	1.076	0.481	0.494	1.340	0.102	0.511	1.464	0.262
22566	0.516	1.498	0.358	0.482	1.265	0.235	0.511	1.464	0.512

22567	0.297	0.099	0.508	0.234	-0.336	0.478	0.000	?	0.512
22568	0.513	1.481	0.481	0.462	1.133	0.444	0.503	1.413	0.512
22569	0.000	?	0.185	0.401	0.737	0.491	0.097	-1.181	0.512
22570	0.000	?	0.358	0.401	0.737	0.462	0.097	-1.181	0.501
22571	0.297	0.099	0.409	0.152	-0.863	0.193	0.263	-0.120	0.501
22572	0.382	0.638	0.097	0.474	1.208	0.398	0.511	1.464	0.262
22573	0.261	-0.137	0.097	0.102	-1.184	0.501	0.300	0.116	0.409
22574	0.500	1.397	0.296	0.418	0.850	0.341	0.497	1.380	0.262
22575	0.263	-0.120	0.262	0.310	0.153	0.235	0.050	-1.485	0.508
22576	0.482	1.279	0.513	0.444	1.020	0.341	0.500	1.397	0.481
22577	0.355	0.470	0.512	0.272	-0.091	0.462	0.187	-0.608	0.481
22578	0.187	-0.608	0.142	0.234	-0.336	0.501	0.142	-0.895	0.492
22579	0.508	1.447	0.508	0.497	1.359	0.273	0.445	1.043	0.262
22580	0.184	-0.625	0.501	0.462	1.133	0.508	0.263	-0.120	0.296
22581	0.429	0.942	0.262	0.503	1.396	0.508	0.432	0.958	0.358
22582	0.489	1.329	0.501	0.447	1.039	0.235	0.513	1.481	0.508
22583	0.495	1.363	0.501	0.515	1.472	0.398	0.513	1.481	0.185
22584	0.500	1.397	0.501	0.468	1.170	0.235	0.492	1.346	0.296
22585	0.097	-1.181	0.467	0.368	0.530	0.508	0.295	0.082	0.501
22586	0.463	1.161	0.328	0.515	1.472	0.514	0.505	1.430	0.508
22587	0.326	0.285	0.185	0.307	0.135	0.501	0.266	-0.103	0.508
22588	0.295	0.082	0.501	0.193	-0.600	0.273	0.187	-0.608	0.262
22589	0.447	1.060	0.481	0.447	1.039	0.478	0.505	1.430	0.481
22590	0.513	1.481	0.508	0.512	1.453	0.478	0.505	1.430	0.185
22591	0.226	-0.356	0.262	0.149	-0.882	0.514	0.184	-0.625	0.142
22592	0.505	1.430	0.328	0.474	1.208	0.512	0.487	1.312	0.508
22593	0.432	0.958	0.262	0.506	1.415	0.512	0.500	1.397	0.508
22594	0.097	-1.181	0.328	0.102	-1.184	0.512	0.332	0.318	0.508
22595	0.184	-0.625	0.513	0.237	-0.317	0.444	0.145	-0.878	0.296
22596	0.497	1.380	0.513	0.485	1.283	0.444	0.466	1.178	0.142
22597	0.463	1.161	0.296	0.447	1.039	0.514	0.503	1.413	0.481
22598	0.463	1.161	0.467	0.497	1.359	0.514	0.450	1.076	0.467
22599	0.497	1.380	0.185	0.462	1.133	0.491	0.434	0.975	0.450
22600	0.187	-0.608	0.467	0.193	-0.600	0.512	0.297	0.099	0.492
22601	0.184	-0.625	0.296	0.398	0.718	0.512	0.184	-0.625	0.224
22602	0.487	1.312	0.224	0.500	1.378	0.514	0.463	1.161	0.262
22603	0.474	1.228	0.409	0.342	0.361	0.508	0.513	1.481	0.097
22604	0.184	-0.625	0.501	0.237	-0.317	0.512	0.226	-0.356	0.328
22605	0.097	-1.181	0.142	0.342	0.361	0.462	0.097	-1.181	0.142
22606	0.445	1.043	0.142	0.465	1.152	0.462	0.505	1.430	0.409
22607	0.500	1.397	0.513	0.503	1.396	0.309	0.432	0.958	0.467
22608	0.050	-1.485	0.512	0.275	-0.073	0.235	0.261	-0.137	0.224
22609	0.487	1.312	0.512	0.509	1.434	0.508	0.508	1.447	0.185
22610	0.497	1.380	0.508	0.509	1.434	0.491	0.500	1.397	0.296
22611	0.187	-0.608	0.224	0.234	-0.336	0.273	0.300	0.116	0.358
22612	0.508	1.447	0.508	0.401	0.737	0.478	0.487	1.312	0.296
22613	0.450	1.076	0.492	0.237	-0.317	0.491	0.145	-0.878	0.501
22614	0.492	1.346	0.513	0.506	1.415	0.371	0.479	1.262	0.185
22615	0.450	1.076	0.501	0.509	1.434	0.491	0.489	1.329	0.296
22616	0.482	1.279	0.513	0.494	1.340	0.491	0.508	1.447	0.142
22617	0.263	-0.120	0.492	0.272	-0.091	0.398	0.389	0.689	0.431
22618	0.513	1.481	0.492	0.485	1.283	0.478	0.450	1.076	0.501
22619	0.513	1.481	0.296	0.485	1.283	0.235	0.466	1.178	0.501
22620	0.484	1.295	0.481	0.515	1.472	0.235	0.326	0.285	0.185
22621	0.463	1.161	0.508	0.512	1.453	0.235	0.508	1.447	0.492
22622	0.463	1.161	0.358	0.512	1.453	0.514	0.508	1.447	0.492
22623	0.463	1.161	0.431	0.512	1.453	0.491	0.508	1.447	0.481
22624	0.489	1.329	0.185	0.442	1.001	0.149	0.513	1.481	0.508
22625	0.489	1.329	0.262	0.444	1.020	0.102	0.511	1.464	0.508
22626	0.505	1.430	0.224	0.512	1.453	0.512	0.500	1.397	0.185
22627	0.489	1.329	0.481	0.515	1.472	0.508	0.450	1.076	0.501

22628	0.479	1.262	0.508	0.491	1.321	0.508	0.482	1.279	0.142
22629	0.463	1.161	0.508	0.512	1.453	0.508	0.511	1.464	0.492
22630	0.500	1.397	0.262	0.512	1.453	0.149	0.511	1.464	0.481
22631	0.484	1.295	0.358	0.512	1.453	0.149	0.505	1.430	0.358
22632	0.432	0.958	0.501	0.509	1.434	0.273	0.505	1.430	0.142
22633	0.455	1.110	0.224	0.512	1.453	0.273	0.500	1.397	0.467
22634	0.500	1.397	0.467	0.465	1.152	0.273	0.505	1.430	0.358
22635	0.500	1.397	0.409	0.465	1.152	0.512	0.505	1.430	0.358
22636	0.261	-0.137	0.512	0.310	0.153	0.273	0.184	-0.625	0.492
22637	0.261	-0.137	0.513	0.237	-0.317	0.273	0.097	-1.181	0.431
22638	0.450	1.076	0.467	0.515	1.472	0.341	0.466	1.178	0.185
22639	0.495	1.363	0.481	0.500	1.378	0.193	0.429	0.942	0.481
22640	0.261	-0.137	0.296	0.272	-0.091	0.514	0.100	-1.164	0.501
22641	0.511	1.464	0.142	0.474	1.208	0.193	0.479	1.262	0.097
22642	0.511	1.464	0.142	0.488	1.302	0.501	0.447	1.060	0.492
22643	0.050	-1.485	0.450	0.371	0.549	0.444	0.184	-0.625	0.467
22644	0.495	1.363	0.296	0.488	1.302	0.422	0.505	1.430	0.467
22645	0.503	1.413	0.296	0.491	1.321	0.235	0.411	0.824	0.431
22646	0.184	-0.625	0.142	0.395	0.700	0.508	0.266	-0.103	0.185
22647	0.466	1.178	0.513	0.480	1.246	0.235	0.503	1.413	0.142
22648	0.000	?	0.224	0.237	-0.317	0.491	0.297	0.099	0.492
22649	0.229	-0.339	0.224	0.234	-0.336	0.235	0.142	-0.895	0.508
22650	0.229	-0.339	0.450	0.234	-0.336	0.235	0.097	-1.181	0.513
22651	0.503	1.413	0.450	0.515	1.472	0.501	0.463	1.161	0.050
22652	0.495	1.363	0.501	0.497	1.359	0.341	0.463	1.161	0.142
22653	0.097	-1.181	0.501	0.149	-0.882	0.422	0.295	0.082	0.097
22654	0.000	?	0.481	0.102	-1.184	0.508	0.450	1.076	0.142
22655	0.511	1.464	0.224	0.515	1.472	0.235	0.505	1.430	0.481
22656	0.511	1.464	0.481	0.503	1.396	0.398	0.476	1.245	0.481
22657	0.513	1.481	0.512	0.503	1.396	0.512	0.489	1.329	0.097
22658	0.497	1.380	0.513	0.506	1.415	0.422	0.500	1.397	0.481
22659	0.263	-0.120	0.512	0.149	-0.882	0.501	0.142	-0.895	0.142
22660	0.263	-0.120	0.512	0.149	-0.882	0.478	0.142	-0.895	0.431
22661	0.263	-0.120	0.512	0.275	-0.073	0.478	0.261	-0.137	0.142
22662	0.263	-0.120	0.501	0.275	-0.073	0.491	0.261	-0.137	0.142
22663	0.263	-0.120	0.508	0.275	-0.073	0.398	0.261	-0.137	0.185
22664	0.508	1.447	0.512	0.506	1.415	0.444	0.466	1.178	0.481
22665	0.261	-0.137	0.512	0.278	-0.054	0.512	0.050	-1.485	0.142
22666	0.224	-0.373	0.501	0.278	-0.054	0.149	0.050	-1.485	0.224
22667	0.187	-0.608	0.501	0.345	0.379	0.491	0.261	-0.137	0.467
22668	0.187	-0.608	0.512	0.193	-0.600	0.491	0.187	-0.608	0.450
22669	0.450	1.076	0.185	0.506	1.415	0.462	0.466	1.178	0.492
22670	0.187	-0.608	0.296	0.193	-0.600	0.514	0.187	-0.608	0.508
22671	0.479	1.262	0.224	0.503	1.396	0.235	0.511	1.464	0.224
22672	0.224	-0.373	0.142	0.447	1.039	0.501	0.050	-1.485	0.512
22673	0.263	-0.120	0.409	0.427	0.907	0.371	0.295	0.082	0.508
22674	0.142	-0.895	0.501	0.234	-0.336	0.371	0.184	-0.625	0.501
22675	0.447	1.060	0.492	0.509	1.434	0.102	0.500	1.397	0.501
22676	0.142	-0.895	0.097	0.234	-0.336	0.508	0.184	-0.625	0.431
22677	0.479	1.262	0.050	0.491	1.321	0.422	0.429	0.942	0.508
22678	0.142	-0.895	0.050	0.240	-0.299	0.422	0.224	-0.373	0.508
22679	0.142	-0.895	0.512	0.240	-0.299	0.508	0.224	-0.373	0.097
22680	0.482	1.279	0.512	0.497	1.359	0.478	0.497	1.380	0.050
22681	0.145	-0.878	0.512	0.342	0.361	0.444	0.142	-0.895	0.492
22682	0.187	-0.608	0.513	0.424	0.888	0.491	0.142	-0.895	0.492
22683	0.468	1.194	0.512	0.509	1.434	0.273	0.508	1.447	0.097
22684	0.097	-1.181	0.450	0.234	-0.336	0.512	0.261	-0.137	0.513
22685	0.497	1.380	0.450	0.398	0.718	0.341	0.511	1.464	0.262
22686	0.466	1.178	0.501	0.509	1.434	0.501	0.518	1.514	0.358
22687	0.463	1.161	0.501	0.424	0.888	0.491	0.497	1.380	0.508
22688	0.511	1.464	0.481	0.500	1.378	0.102	0.463	1.161	0.492

22689	0.508	1.447	0.512	0.480	1.246	0.501	0.450	1.076	0.481
22690	0.511	1.464	0.513	0.482	1.265	0.501	0.503	1.413	0.142
22691	0.484	1.295	0.508	0.494	1.340	0.512	0.463	1.161	0.467
22692	0.297	0.099	0.358	0.398	0.718	0.235	0.097	−1.181	0.185
22693	0.466	1.178	0.358	0.447	1.039	0.235	0.487	1.312	0.501
22694	0.508	1.447	0.450	0.518	1.491	0.273	0.484	1.295	0.431
22695	0.263	−0.120	0.512	0.149	−0.882	0.491	0.142	−0.895	0.481
22696	0.513	1.481	0.450	0.491	1.321	0.512	0.474	1.228	0.467
22697	0.405	0.790	0.097	0.491	1.321	0.478	0.516	1.498	0.508
22698	0.508	1.447	0.512	0.462	1.133	0.478	0.500	1.397	0.492
22699	0.466	1.178	0.513	0.518	1.491	0.102	0.429	0.942	0.296
22700	0.261	−0.137	0.513	0.237	−0.317	0.235	0.184	−0.625	0.050
22701	0.447	1.060	0.513	0.500	1.378	0.341	0.503	1.413	0.050
22702	0.184	−0.625	0.513	0.371	0.549	0.508	0.297	0.099	0.409
22703	0.184	−0.625	0.492	0.371	0.549	0.273	0.297	0.099	0.262
22704	0.295	0.082	0.409	0.102	−1.184	0.309	0.226	−0.356	0.296
22705	0.500	1.397	0.508	0.503	1.396	0.309	0.445	1.043	0.224
22706	0.489	1.329	0.358	0.421	0.869	0.508	0.495	1.363	0.508
22707	0.489	1.329	0.358	0.421	0.869	0.309	0.503	1.413	0.262
22708	0.429	0.942	0.262	0.503	1.396	0.462	0.513	1.481	0.262
22709	0.492	1.346	0.050	0.482	1.265	0.514	0.503	1.413	0.508
22710	0.261	−0.137	0.501	0.450	1.057	0.273	0.142	−0.895	0.508
22711	0.511	1.464	0.262	0.485	1.283	0.235	0.453	1.093	0.492
22712	0.097	−1.181	0.508	0.272	−0.091	0.235	0.261	−0.137	0.492
22713	0.513	1.481	0.492	0.512	1.453	0.371	0.429	0.942	0.450
22714	0.226	−0.356	0.431	0.339	0.342	0.193	0.387	0.672	0.262
22715	0.468	1.194	0.431	0.497	1.359	0.491	0.500	1.397	0.501
22716	0.432	0.958	0.513	0.497	1.359	0.309	0.503	1.413	0.450
22717	0.050	−1.485	0.501	0.102	−1.184	0.478	0.387	0.672	0.512
22718	0.445	1.043	0.512	0.497	1.359	0.444	0.508	1.447	0.501
22719	0.511	1.464	0.296	0.497	1.359	0.235	0.489	1.329	0.508
22720	0.497	1.380	0.481	0.512	1.453	0.235	0.468	1.194	0.501
22721	0.411	0.824	0.224	0.234	−0.336	0.149	0.358	0.487	0.512
22722	0.411	0.824	0.508	0.234	−0.336	0.371	0.434	0.975	0.513
22723	0.142	−0.895	0.508	0.275	−0.073	0.102	0.142	−0.895	0.431
22724	0.429	0.942	0.142	0.488	1.302	0.444	0.503	1.413	0.431
22725	0.358	0.487	0.097	0.515	1.472	0.501	0.463	1.161	0.409
22726	0.505	1.430	0.512	0.482	1.265	0.491	0.408	0.807	0.512
22727	0.505	1.430	0.185	0.482	1.265	0.514	0.408	0.807	0.481
22728	0.142	−0.895	0.492	0.102	−1.184	0.478	0.326	0.285	0.142
22729	0.142	−0.895	0.142	0.234	−0.336	0.478	0.297	0.099	0.142
22730	0.332	0.318	0.492	0.342	0.361	0.444	0.142	−0.895	0.185
22731	0.434	0.975	0.501	0.509	1.434	0.508	0.503	1.413	0.224
22732	0.142	−0.895	0.501	0.275	−0.073	0.508	0.184	−0.625	0.467
22733	0.329	0.301	0.501	0.313	0.172	0.491	0.050	−1.485	0.501
22734	0.000	?	0.185	0.310	0.153	0.491	0.263	−0.120	0.492
22735	0.500	1.397	0.450	0.512	1.453	0.501	0.471	1.211	0.224
22736	0.050	−1.485	0.508	0.313	0.172	0.512	0.184	−0.625	0.262
22737	0.479	1.262	0.431	0.462	1.133	0.508	0.482	1.279	0.224
22738	0.503	1.413	0.513	0.520	1.509	0.508	0.508	1.447	0.385
22739	0.187	−0.608	0.467	0.275	−0.073	0.508	0.184	−0.625	0.385
22740	0.184	−0.625	0.328	0.234	−0.336	0.501	0.184	−0.625	0.385
22741	0.142	−0.895	0.501	0.237	−0.317	0.512	0.184	−0.625	0.385
22742	0.358	0.487	0.431	0.371	0.549	0.508	0.358	0.487	0.508
22743	0.187	−0.608	0.431	0.196	−0.581	0.501	0.184	−0.625	0.512
22744	0.492	1.346	0.431	0.491	1.321	0.462	0.508	1.447	0.512
22745	0.050	−1.485	0.501	0.307	0.135	0.149	0.300	0.116	0.481
22746	0.516	1.498	0.501	0.480	1.246	0.422	0.447	1.060	0.508
22747	0.511	1.464	0.512	0.447	1.039	0.491	0.479	1.262	0.508
22748	0.263	−0.120	0.512	0.234	−0.336	0.193	0.261	−0.137	0.512
22749	0.142	−0.895	0.492	0.237	−0.317	0.422	0.329	0.301	0.508

22750	0.184	-0.625	0.492	0.149	-0.882	0.398	0.266	-0.103	0.481
22751	0.184	-0.625	0.142	0.371	0.549	0.501	0.184	-0.625	0.481
22752	0.184	-0.625	0.142	0.102	-1.184	0.462	0.261	-0.137	0.508
22753	0.492	1.346	0.142	0.439	0.982	0.462	0.489	1.329	0.512
22754	0.489	1.329	0.481	0.500	1.378	0.193	0.508	1.447	0.328
22755	0.508	1.447	0.262	0.488	1.302	0.478	0.508	1.447	0.467
22756	0.503	1.413	0.328	0.515	1.472	0.341	0.503	1.413	0.358
22757	0.463	1.161	0.224	0.480	1.246	0.193	0.513	1.481	0.492
22758	0.463	1.161	0.501	0.480	1.246	0.462	0.513	1.481	0.492
22759	0.097	-1.181	0.185	0.447	1.039	0.462	0.142	-0.895	0.508
22760	0.500	1.397	0.501	0.509	1.434	0.444	0.432	0.958	0.512
22761	0.484	1.295	0.501	0.512	1.453	0.444	0.503	1.413	0.512
22762	0.492	1.346	0.142	0.494	1.340	0.491	0.505	1.430	0.501
22763	0.500	1.397	0.185	0.491	1.321	0.462	0.513	1.481	0.328
22764	0.476	1.245	0.097	0.503	1.396	0.102	0.508	1.447	0.328
22765	0.487	1.312	0.513	0.515	1.472	0.149	0.508	1.447	0.481
22766	0.429	0.942	0.481	0.512	1.453	0.341	0.447	1.060	0.358
22767	0.508	1.447	0.467	0.503	1.396	0.491	0.479	1.262	0.512
22768	0.503	1.413	0.481	0.506	1.415	0.508	0.479	1.262	0.512
22769	0.513	1.481	0.450	0.503	1.396	0.508	0.503	1.413	0.501
22770	0.497	1.380	0.450	0.506	1.415	0.514	0.508	1.447	0.512
22771	0.516	1.498	0.224	0.506	1.415	0.235	0.471	1.211	0.513
22772	0.450	1.076	0.450	0.497	1.359	0.273	0.511	1.464	0.513
22773	0.429	0.942	0.501	0.465	1.152	0.341	0.513	1.481	0.512
22774	0.187	-0.608	0.501	0.149	-0.882	0.444	0.224	-0.373	0.142
22775	0.482	1.279	0.296	0.418	0.850	0.309	0.505	1.430	0.508
22776	0.508	1.447	0.097	0.488	1.302	0.512	0.516	1.498	0.358
22777	0.000	?	0.328	0.193	-0.600	0.512	0.332	0.318	0.513
22778	0.050	-1.485	0.508	0.421	0.869	0.512	0.387	0.672	0.508
22779	0.050	-1.485	0.512	0.401	0.737	0.444	0.261	-0.137	0.296
22780	0.505	1.430	0.501	0.500	1.378	0.102	0.503	1.413	0.328
22781	0.487	1.312	0.501	0.465	1.152	0.491	0.508	1.447	0.481
22782	0.450	1.076	0.501	0.462	1.133	0.102	0.466	1.178	0.385
22783	0.329	0.301	0.358	0.193	-0.600	0.235	0.329	0.301	0.512
22784	0.413	0.841	0.492	0.480	1.246	0.444	0.497	1.380	0.409
22785	0.261	-0.137	0.409	0.342	0.361	0.444	0.295	0.082	0.481
22786	0.097	-1.181	0.513	0.196	-0.581	0.462	0.263	-0.120	0.431
22787	0.408	0.807	0.512	0.462	1.133	0.273	0.508	1.447	0.431
22788	0.408	0.807	0.501	0.462	1.133	0.193	0.508	1.447	0.431
22789	0.482	1.279	0.050	0.444	1.020	0.444	0.505	1.430	0.296
22790	0.482	1.279	0.512	0.444	1.020	0.501	0.505	1.430	0.501
22791	0.513	1.481	0.508	0.494	1.340	0.508	0.495	1.363	0.481
22792	0.505	1.430	0.409	0.459	1.114	0.501	0.497	1.380	0.450
22793	0.263	-0.120	0.224	0.102	-1.184	0.273	0.263	-0.120	0.481
22794	0.050	-1.485	0.481	0.152	-0.863	0.193	0.405	0.790	0.450
22795	0.097	-1.181	0.050	0.339	0.342	0.309	0.261	-0.137	0.513
22796	0.429	0.942	0.224	0.488	1.302	0.273	0.511	1.464	0.481
22797	0.484	1.295	0.296	0.512	1.453	0.309	0.482	1.279	0.508
22798	0.484	1.295	0.467	0.512	1.453	0.341	0.482	1.279	0.508
22799	0.497	1.380	0.508	0.512	1.453	0.508	0.495	1.363	0.508
22800	0.226	-0.356	0.328	0.234	-0.336	0.273	0.261	-0.137	0.501
22801	0.184	-0.625	0.050	0.272	-0.091	0.193	0.297	0.099	0.501
22802	0.263	-0.120	0.467	0.339	0.342	0.309	0.358	0.487	0.481
22803	0.429	0.942	0.481	0.442	1.001	0.235	0.505	1.430	0.501
22804	0.297	0.099	0.296	0.310	0.153	0.341	0.187	-0.608	0.492
22805	0.503	1.413	0.262	0.509	1.434	0.422	0.297	0.099	0.492
22806	0.476	1.245	0.050	0.509	1.434	0.235	0.500	1.397	0.296
22807	0.489	1.329	0.050	0.509	1.434	0.149	0.508	1.447	0.296
22808	0.503	1.413	0.501	0.442	1.001	0.235	0.505	1.430	0.296
22809	0.226	-0.356	0.481	0.102	-1.184	0.512	0.263	-0.120	0.481
22810	0.450	1.076	0.501	0.494	1.340	0.512	0.479	1.262	0.097

22811	0.266	−0.103	0.501	0.102	−1.184	0.501	0.184	−0.625	0.262
22812	0.229	−0.339	0.262	0.234	−0.336	0.309	0.142	−0.895	0.142
22813	0.492	1.346	0.431	0.439	0.982	0.422	0.503	1.413	0.481
22814	0.492	1.346	0.296	0.439	0.982	0.508	0.503	1.413	0.296
22815	0.511	1.464	0.508	0.459	1.114	0.501	0.503	1.413	0.513
22816	0.226	−0.356	0.262	0.275	−0.073	0.341	0.297	0.099	0.513
22817	0.358	0.487	0.450	0.196	−0.581	0.422	0.142	−0.895	0.224
22818	0.479	1.262	0.097	0.447	1.039	0.341	0.484	1.295	0.262
22819	0.489	1.329	0.097	0.497	1.359	0.491	0.466	1.178	0.296
22820	0.516	1.498	0.508	0.512	1.453	0.422	0.453	1.093	0.481
22821	0.513	1.481	0.492	0.497	1.359	0.514	0.489	1.329	0.508
22822	0.142	−0.895	0.492	0.275	−0.073	0.491	0.226	−0.356	0.508
22823	0.224	−0.373	0.224	0.193	−0.600	0.444	0.261	−0.137	0.501
22824	0.332	0.318	0.481	0.307	0.135	0.371	0.097	−1.181	0.508
22825	0.000	?	0.224	0.275	−0.073	0.508	0.329	0.301	0.508
22826	0.297	0.099	0.224	0.307	0.135	0.053	0.142	−0.895	0.185
22827	0.050	−1.485	0.185	0.339	0.342	0.053	0.413	0.841	0.450
22828	0.503	1.413	0.224	0.506	1.415	0.309	0.468	1.194	0.508
22829	0.384	0.655	0.262	0.275	−0.073	0.309	0.224	−0.373	0.450
22830	0.224	−0.373	0.508	0.196	−0.581	0.501	0.184	−0.625	0.262
22831	0.384	0.655	0.262	0.310	0.153	0.501	0.300	0.116	0.050
22832	0.224	−0.373	0.358	0.234	−0.336	0.309	0.358	0.487	0.050
22833	0.184	−0.625	0.512	0.345	0.379	0.478	0.297	0.099	0.492
22834	0.511	1.464	0.512	0.424	0.888	0.508	0.492	1.346	0.492
22835	0.184	−0.625	0.508	0.234	−0.336	0.371	0.187	−0.608	0.501
22836	0.184	−0.625	0.467	0.149	−0.882	0.478	0.300	0.116	0.501
22837	0.224	−0.373	0.185	0.237	−0.317	0.478	0.142	−0.895	0.508
22838	0.476	1.245	0.142	0.512	1.453	0.478	0.429	0.942	0.385
22839	0.426	0.925	0.097	0.512	1.453	0.462	0.505	1.430	0.450
22840	0.484	1.295	0.262	0.503	1.396	0.341	0.500	1.397	0.467
22841	0.226	−0.356	0.501	0.313	0.172	0.235	0.184	−0.625	0.501
22842	0.513	1.481	0.501	0.421	0.869	0.514	0.492	1.346	0.501
22843	0.503	1.413	0.450	0.509	1.434	0.491	0.492	1.346	0.492
22844	0.489	1.329	0.492	0.497	1.359	0.422	0.479	1.262	0.296
22845	0.500	1.397	0.508	0.342	0.361	0.309	0.511	1.464	0.431
22846	0.463	1.161	0.508	0.424	0.888	0.273	0.503	1.413	0.492
22847	0.000	?	0.501	0.342	0.361	0.273	0.187	−0.608	0.508
22848	0.474	1.228	0.508	0.491	1.321	0.193	0.505	1.430	0.097
22849	0.361	0.504	0.508	0.424	0.888	0.444	0.142	−0.895	0.501
22850	0.511	1.464	0.409	0.509	1.434	0.444	0.492	1.346	0.296
22851	0.484	1.295	0.097	0.491	1.321	0.235	0.484	1.295	0.328
22852	0.184	−0.625	0.097	0.444	1.020	0.501	0.361	0.504	0.185
22853	0.358	0.487	0.508	0.374	0.568	0.273	0.142	−0.895	0.185
22854	0.408	0.807	0.492	0.512	1.453	0.508	0.468	1.194	0.431
22855	0.097	−1.181	0.431	0.053	−1.504	0.149	0.358	0.487	0.501
22856	0.097	−1.181	0.512	0.053	−1.504	0.193	0.358	0.487	0.142
22857	0.295	0.082	0.185	0.313	0.172	0.149	0.000	?	0.142
22858	0.261	−0.137	0.508	0.313	0.172	0.508	0.000	?	0.492
22859	0.513	1.481	0.050	0.503	1.396	0.273	0.492	1.346	0.501
22860	0.513	1.481	0.513	0.503	1.396	0.235	0.429	0.942	0.185
22861	0.142	−0.895	0.512	0.313	0.172	0.478	0.184	−0.625	0.296
22862	0.505	1.430	0.501	0.480	1.246	0.514	0.482	1.279	0.142
22863	0.492	1.346	0.508	0.506	1.415	0.514	0.503	1.413	0.142
22864	0.263	−0.120	0.508	0.374	0.568	0.462	0.097	−1.181	0.431
22865	0.497	1.380	0.185	0.477	1.227	0.398	0.489	1.329	0.481
22866	0.497	1.380	0.431	0.477	1.227	0.508	0.466	1.178	0.450
22867	0.497	1.380	0.409	0.477	1.227	0.512	0.466	1.178	0.450
22868	0.466	1.178	0.513	0.465	1.152	0.371	0.429	0.942	0.409
22869	0.332	0.318	0.492	0.342	0.361	0.508	0.184	−0.625	0.512
22870	0.187	−0.608	0.185	0.234	−0.336	0.512	0.142	−0.895	0.142
22871	0.503	1.413	0.296	0.509	1.434	0.514	0.489	1.329	0.467

22872	0.482	1.279	0.492	0.491	1.321	0.514	0.503	1.413	0.097
22873	0.508	1.447	0.513	0.427	0.907	0.398	0.508	1.447	0.512
22874	0.263	-0.120	0.224	0.310	0.153	0.341	0.050	-1.485	0.097
22875	0.329	0.301	0.501	0.272	-0.091	0.501	0.142	-0.895	0.050
22876	0.261	-0.137	0.467	0.272	-0.091	0.512	0.097	-1.181	0.492
22877	0.329	0.301	0.508	0.193	-0.600	0.235	0.142	-0.895	0.492
22878	0.511	1.464	0.224	0.442	1.001	0.491	0.476	1.245	0.481
22879	0.511	1.464	0.185	0.442	1.001	0.235	0.476	1.245	0.185
22880	0.295	0.082	0.296	0.234	-0.336	0.371	0.097	-1.181	0.467
22881	0.468	1.194	0.512	0.506	1.415	0.371	0.476	1.245	0.142
22882	0.184	-0.625	0.450	0.275	-0.073	0.491	0.142	-0.895	0.185
22883	0.466	1.178	0.450	0.506	1.415	0.514	0.429	0.942	0.328
22884	0.295	0.082	0.513	0.149	-0.882	0.512	0.142	-0.895	0.431
22885	0.226	-0.356	0.185	0.193	-0.600	0.235	0.145	-0.878	0.224
22886	0.408	0.807	0.450	0.152	-0.863	0.309	0.184	-0.625	0.481
22887	0.508	1.447	0.501	0.509	1.434	0.422	0.482	1.279	0.358
22888	0.142	-0.895	0.467	0.275	-0.073	0.309	0.142	-0.895	0.262
22889	0.145	-0.878	0.185	0.237	-0.317	0.512	0.224	-0.373	0.481
22890	0.503	1.413	0.142	0.482	1.265	0.514	0.468	1.194	0.492
22891	0.500	1.397	0.512	0.520	1.509	0.444	0.453	1.093	0.481
22892	0.500	1.397	0.142	0.520	1.509	0.422	0.497	1.380	0.501
22893	0.497	1.380	0.142	0.471	1.189	0.508	0.511	1.464	0.262
22894	0.229	-0.339	0.262	0.398	0.718	0.462	0.224	-0.373	0.097
22895	0.508	1.447	0.508	0.509	1.434	0.193	0.511	1.464	0.328
22896	0.492	1.346	0.481	0.515	1.472	0.422	0.505	1.430	0.296
22897	0.511	1.464	0.513	0.371	0.549	0.422	0.492	1.346	0.481
22898	0.495	1.363	0.262	0.509	1.434	0.512	0.503	1.413	0.481
22899	0.505	1.430	0.224	0.515	1.472	0.193	0.434	0.975	0.508
22900	0.492	1.346	0.508	0.512	1.453	0.149	0.505	1.430	0.513
22901	0.492	1.346	0.501	0.512	1.453	0.398	0.505	1.430	0.508
22902	0.000	?	0.224	0.401	0.737	0.444	0.097	-1.181	0.508
22903	0.297	0.099	0.296	0.345	0.379	0.149	0.050	-1.485	0.501
22904	0.476	1.245	0.296	0.500	1.378	0.149	0.489	1.329	0.385
22905	0.503	1.413	0.512	0.512	1.453	0.422	0.489	1.329	0.385
22906	0.355	0.470	0.467	0.234	-0.336	0.422	0.097	-1.181	0.492
22907	0.434	0.975	0.262	0.488	1.302	0.478	0.516	1.498	0.358
22908	0.184	-0.625	0.431	0.237	-0.317	0.478	0.263	-0.120	0.358
22909	0.263	-0.120	0.508	0.371	0.549	0.501	0.361	0.504	0.508
22910	0.226	-0.356	0.358	0.371	0.549	0.102	0.000	?	0.385
22911	0.479	1.262	0.501	0.485	1.283	0.478	0.505	1.430	0.467
22912	0.505	1.430	0.512	0.518	1.491	0.491	0.489	1.329	0.508
22913	0.508	1.447	0.224	0.512	1.453	0.444	0.482	1.279	0.328
22914	0.263	-0.120	0.501	0.234	-0.336	0.371	0.142	-0.895	0.508
22915	0.358	0.487	0.501	0.310	0.153	0.462	0.000	?	0.296
22916	0.495	1.363	0.450	0.424	0.888	0.491	0.468	1.194	0.450
22917	0.224	-0.373	0.431	0.310	0.153	0.491	0.184	-0.625	0.385
22918	0.463	1.161	0.358	0.515	1.472	0.512	0.503	1.413	0.508
22919	0.408	0.807	0.512	0.515	1.472	0.491	0.434	0.975	0.431
22920	0.513	1.481	0.512	0.442	1.001	0.491	0.484	1.295	0.431
22921	0.516	1.498	0.512	0.421	0.869	0.512	0.471	1.211	0.358
22922	0.468	1.194	0.512	0.506	1.415	0.462	0.513	1.481	0.512
22923	0.482	1.279	0.224	0.462	1.133	0.478	0.492	1.346	0.512
22924	0.300	0.116	0.492	0.193	-0.600	0.273	0.295	0.082	0.481
22925	0.142	-0.895	0.224	0.424	0.888	0.102	0.050	-1.485	0.501
22926	0.142	-0.895	0.262	0.424	0.888	0.193	0.050	-1.485	0.142
22927	0.447	1.060	0.224	0.518	1.491	0.309	0.411	0.824	0.262
22928	0.300	0.116	0.224	0.193	-0.600	0.501	0.263	-0.120	0.481
22929	0.300	0.116	0.508	0.149	-0.882	0.508	0.297	0.099	0.481
22930	0.142	-0.895	0.097	0.398	0.718	0.462	0.224	-0.373	0.358
22931	0.511	1.464	0.450	0.444	1.020	0.398	0.503	1.413	0.512
22932	0.224	-0.373	0.508	0.149	-0.882	0.309	0.261	-0.137	0.450

22933	0.224	−0.373	0.467	0.149	−0.882	0.309	0.261	−0.137	0.508
22934	0.450	1.076	0.224	0.427	0.907	0.512	0.508	1.447	0.296
22935	0.450	1.076	0.224	0.427	0.907	0.512	0.508	1.447	0.262
22936	0.500	1.397	0.224	0.480	1.246	0.512	0.489	1.329	0.142
22937	0.500	1.397	0.467	0.480	1.246	0.512	0.489	1.329	0.142
22938	0.479	1.262	0.409	0.503	1.396	0.478	0.445	1.043	0.512
22939	0.224	−0.373	0.262	0.102	−1.184	0.512	0.261	−0.137	0.501
22940	0.479	1.262	0.481	0.474	1.208	0.501	0.503	1.413	0.501
22941	0.508	1.447	0.512	0.488	1.302	0.514	0.447	1.060	0.501
22942	0.500	1.397	0.481	0.447	1.039	0.508	0.511	1.464	0.385
22943	0.500	1.397	0.262	0.371	0.549	0.512	0.503	1.413	0.513
22944	0.500	1.397	0.385	0.465	1.152	0.491	0.513	1.481	0.450
22945	0.500	1.397	0.481	0.494	1.340	0.491	0.505	1.430	0.513
22946	0.503	1.413	0.262	0.488	1.302	0.512	0.505	1.430	0.296
22947	0.511	1.464	0.513	0.509	1.434	0.193	0.505	1.430	0.142
22948	0.511	1.464	0.492	0.488	1.302	0.193	0.434	0.975	0.501
22949	0.511	1.464	0.185	0.488	1.302	0.491	0.434	0.975	0.358
22950	0.500	1.397	0.467	0.509	1.434	0.462	0.411	0.824	0.385
22951	0.503	1.413	0.050	0.459	1.114	0.422	0.516	1.498	0.224
22952	0.513	1.481	0.050	0.477	1.227	0.102	0.474	1.228	0.481
22953	0.184	−0.625	0.512	0.272	−0.091	0.512	0.142	−0.895	0.501
22954	0.300	0.116	0.185	0.102	−1.184	0.512	0.142	−0.895	0.481
22955	0.226	−0.356	0.492	0.196	−0.581	0.462	0.184	−0.625	0.328
22956	0.142	−0.895	0.328	0.307	0.135	0.462	0.224	−0.373	0.097
22957	0.408	0.807	0.296	0.503	1.396	0.462	0.463	1.161	0.409
22958	0.500	1.397	0.492	0.506	1.415	0.512	0.500	1.397	0.467
22959	0.492	1.346	0.481	0.459	1.114	0.501	0.489	1.329	0.050
22960	0.100	−1.164	0.508	0.401	0.737	0.512	0.224	−0.373	0.224
22961	0.050	−1.485	0.508	0.313	0.172	0.193	0.261	−0.137	0.224
22962	0.050	−1.485	0.450	0.313	0.172	0.193	0.224	−0.373	0.492
22963	0.503	1.413	0.492	0.515	1.472	0.371	0.382	0.638	0.501
22964	0.503	1.413	0.492	0.515	1.472	0.273	0.382	0.638	0.185
22965	0.503	1.413	0.450	0.515	1.472	0.508	0.382	0.638	0.501
22966	0.503	1.413	0.501	0.515	1.472	0.444	0.382	0.638	0.467
22967	0.503	1.413	0.224	0.480	1.246	0.462	0.505	1.430	0.431
22968	0.450	1.076	0.492	0.509	1.434	0.491	0.511	1.464	0.385
22969	0.447	1.060	0.328	0.500	1.378	0.508	0.516	1.498	0.142
22970	0.500	1.397	0.262	0.512	1.453	0.508	0.484	1.295	0.431
22971	0.497	1.380	0.185	0.509	1.434	0.462	0.503	1.413	0.450
22972	0.476	1.245	0.512	0.515	1.472	0.462	0.500	1.397	0.450
22973	0.508	1.447	0.097	0.491	1.321	0.371	0.508	1.447	0.512
22974	0.511	1.464	0.185	0.491	1.321	0.149	0.505	1.430	0.224
22975	0.503	1.413	0.501	0.512	1.453	0.514	0.479	1.262	0.467
22976	0.358	0.487	0.501	0.193	−0.600	0.193	0.000	?	0.450
22977	0.358	0.487	0.501	0.193	−0.600	0.478	0.000	?	0.450
22978	0.447	1.060	0.262	0.491	1.321	0.512	0.482	1.279	0.450
22979	0.511	1.464	0.450	0.465	1.152	0.341	0.511	1.464	0.097
22980	0.445	1.043	0.450	0.418	0.850	0.235	0.513	1.481	0.492
22981	0.097	−1.181	0.508	0.102	−1.184	0.444	0.329	0.301	0.262
22982	0.508	1.447	0.508	0.520	1.509	0.273	0.468	1.194	0.262
22983	0.511	1.464	0.513	0.518	1.491	0.422	0.361	0.504	0.050
22984	0.511	1.464	0.450	0.459	1.114	0.309	0.492	1.346	0.431
22985	0.511	1.464	0.481	0.459	1.114	0.462	0.492	1.346	0.512
22986	0.511	1.464	0.142	0.459	1.114	0.508	0.505	1.430	0.328
22987	0.492	1.346	0.142	0.509	1.434	0.508	0.505	1.430	0.492
22988	0.408	0.807	0.467	0.497	1.359	0.501	0.505	1.430	0.501
22989	0.503	1.413	0.185	0.509	1.434	0.235	0.500	1.397	0.513
22990	0.358	0.487	0.050	0.196	−0.581	0.341	0.000	?	0.185
22991	0.358	0.487	0.050	0.196	−0.581	0.508	0.000	?	0.050
22992	0.263	−0.120	0.142	0.371	0.549	0.193	0.326	0.285	0.262
22993	0.050	−1.485	0.142	0.275	−0.073	0.514	0.326	0.285	0.508

22994	0.497	1.380	0.224	0.506	1.415	0.512	0.479	1.262	0.512
22995	0.263	-0.120	0.224	0.450	1.057	0.491	0.358	0.487	0.262
22996	0.505	1.430	0.224	0.465	1.152	0.193	0.503	1.413	0.492
22997	0.492	1.346	0.450	0.491	1.321	0.491	0.503	1.413	0.385
22998	0.432	0.958	0.450	0.506	1.415	0.512	0.497	1.380	0.508
22999	0.432	0.958	0.296	0.506	1.415	0.512	0.508	1.447	0.050
23000	0.511	1.464	0.185	0.462	1.133	0.512	0.513	1.481	0.513
23001	0.503	1.413	0.385	0.465	1.152	0.501	0.511	1.464	0.512
23002	0.513	1.481	0.224	0.371	0.549	0.501	0.513	1.481	0.097
23003	0.297	0.099	0.501	0.149	-0.882	0.491	0.142	-0.895	0.492
23004	0.482	1.279	0.450	0.512	1.453	0.491	0.505	1.430	0.450
23005	0.224	-0.373	0.431	0.193	-0.600	0.508	0.361	0.504	0.142
23006	0.503	1.413	0.358	0.480	1.246	0.508	0.513	1.481	0.481
23007	0.505	1.430	0.224	0.518	1.491	0.309	0.508	1.447	0.224
23008	0.142	-0.895	0.409	0.342	0.361	0.309	0.297	0.099	0.224
23009	0.097	-1.181	0.050	0.234	-0.336	0.309	0.326	0.285	0.431
23010	0.511	1.464	0.508	0.444	1.020	0.508	0.474	1.228	0.185
23011	0.184	-0.625	0.467	0.275	-0.073	0.341	0.384	0.655	0.185
23012	0.489	1.329	0.508	0.424	0.888	0.341	0.511	1.464	0.512
23013	0.142	-0.895	0.142	0.310	0.153	0.309	0.408	0.807	0.224
23014	0.487	1.312	0.508	0.465	1.152	0.514	0.479	1.262	0.296
23015	0.495	1.363	0.512	0.512	1.453	0.235	0.429	0.942	0.512
23016	0.495	1.363	0.508	0.512	1.453	0.444	0.429	0.942	0.481
23017	0.495	1.363	0.296	0.503	1.396	0.444	0.429	0.942	0.481
23018	0.187	-0.608	0.185	0.234	-0.336	0.193	0.300	0.116	0.481
23019	0.447	1.060	0.296	0.339	0.342	0.398	0.505	1.430	0.508
23020	0.511	1.464	0.501	0.506	1.415	0.273	0.482	1.279	0.385
23021	0.000	?	0.450	0.193	-0.600	0.422	0.447	1.060	0.385
23022	0.429	0.942	0.481	0.512	1.453	0.462	0.482	1.279	0.513
23023	0.508	1.447	0.450	0.512	1.453	0.501	0.450	1.076	0.508
23024	0.461	1.144	0.492	0.488	1.302	0.462	0.513	1.481	0.296
23025	0.332	0.318	0.450	0.193	-0.600	0.462	0.000	?	0.481
23026	0.497	1.380	0.262	0.491	1.321	0.462	0.479	1.262	0.142
23027	0.429	0.942	0.467	0.512	1.453	0.193	0.508	1.447	0.185
23028	0.429	0.942	0.450	0.512	1.453	0.478	0.508	1.447	0.142
23029	0.429	0.942	0.185	0.512	1.453	0.508	0.508	1.447	0.501
23030	0.500	1.397	0.185	0.500	1.378	0.444	0.495	1.363	0.142
23031	0.500	1.397	0.296	0.500	1.378	0.371	0.495	1.363	0.431
23032	0.503	1.413	0.296	0.488	1.302	0.398	0.482	1.279	0.501
23033	0.503	1.413	0.481	0.488	1.302	0.193	0.503	1.413	0.501
23034	0.484	1.295	0.481	0.506	1.415	0.462	0.492	1.346	0.501
23035	0.484	1.295	0.513	0.506	1.415	0.478	0.492	1.346	0.097
23036	0.142	-0.895	0.481	0.307	0.135	0.478	0.300	0.116	0.409
23037	0.142	-0.895	0.508	0.307	0.135	0.478	0.300	0.116	0.409
23038	0.142	-0.895	0.508	0.307	0.135	0.491	0.300	0.116	0.508
23039	0.476	1.245	0.224	0.506	1.415	0.501	0.484	1.295	0.512
23040	0.263	-0.120	0.385	0.345	0.379	0.501	0.097	-1.181	0.481
23041	0.332	0.318	0.262	0.342	0.361	0.478	0.263	-0.120	0.450
23042	0.224	-0.373	0.481	0.310	0.153	0.478	0.145	-0.878	0.481
23043	0.500	1.397	0.262	0.512	1.453	0.478	0.482	1.279	0.262
23044	0.184	-0.625	0.508	0.240	-0.299	0.462	0.297	0.099	0.185
23045	0.503	1.413	0.467	0.447	1.039	0.193	0.508	1.447	0.512
23046	0.503	1.413	0.501	0.447	1.039	0.478	0.508	1.447	0.224
23047	0.142	-0.895	0.431	0.193	-0.600	0.508	0.224	-0.373	0.097
23048	0.187	-0.608	0.142	0.398	0.718	0.478	0.263	-0.120	0.097
23049	0.097	-1.181	0.508	0.272	-0.091	0.235	0.295	0.082	0.185
23050	0.511	1.464	0.097	0.421	0.869	0.491	0.474	1.228	0.142
23051	0.476	1.245	0.050	0.462	1.133	0.235	0.511	1.464	0.385
23052	0.466	1.178	0.224	0.500	1.378	0.273	0.508	1.447	0.262
23053	0.482	1.279	0.513	0.462	1.133	0.235	0.500	1.397	0.385
23054	0.450	1.076	0.501	0.462	1.133	0.341	0.508	1.447	0.512

23055	0.450	1.076	0.450	0.462	1.133	0.444	0.508	1.447	0.501
23056	0.224	-0.373	0.501	0.193	-0.600	0.514	0.184	-0.625	0.050
23057	0.447	1.060	0.467	0.480	1.246	0.235	0.445	1.043	0.224
23058	0.495	1.363	0.409	0.509	1.434	0.371	0.508	1.447	0.185
23059	0.497	1.380	0.513	0.442	1.001	0.491	0.450	1.076	0.296
23060	0.297	0.099	0.492	0.368	0.530	0.422	0.261	-0.137	0.492
23061	0.097	-1.181	0.501	0.398	0.718	0.149	0.050	-1.485	0.513
23062	0.329	0.301	0.328	0.193	-0.600	0.235	0.050	-1.485	0.512
23063	0.511	1.464	0.467	0.462	1.133	0.341	0.489	1.329	0.185
23064	0.518	1.514	0.467	0.477	1.227	0.341	0.492	1.346	0.409
23065	0.503	1.413	0.185	0.474	1.208	0.444	0.495	1.363	0.142
23066	0.503	1.413	0.481	0.474	1.208	0.462	0.495	1.363	0.385
23067	0.503	1.413	0.450	0.488	1.302	0.501	0.500	1.397	0.328
23068	0.355	0.470	0.185	0.497	1.359	0.501	0.384	0.655	0.385
23069	0.492	1.346	0.185	0.503	1.396	0.235	0.447	1.060	0.512
23070	0.411	0.824	0.431	0.477	1.227	0.444	0.466	1.178	0.142
23071	0.516	1.498	0.467	0.477	1.227	0.149	0.503	1.413	0.467
23072	0.513	1.481	0.467	0.477	1.227	0.491	0.503	1.413	0.512
23073	0.497	1.380	0.508	0.462	1.133	0.193	0.492	1.346	0.481
23074	0.050	-1.485	0.513	0.193	-0.600	0.508	0.295	0.082	0.142
23075	0.516	1.498	0.185	0.477	1.227	0.371	0.429	0.942	0.409
23076	0.505	1.430	0.185	0.509	1.434	0.371	0.492	1.346	0.450
23077	0.413	0.841	0.224	0.482	1.265	0.462	0.505	1.430	0.501
23078	0.224	-0.373	0.385	0.237	-0.317	0.512	0.097	-1.181	0.481
23079	0.476	1.245	0.501	0.491	1.321	0.512	0.508	1.447	0.431
23080	0.000	?	0.224	0.234	-0.336	0.273	0.300	0.116	0.467
23081	0.050	-1.485	0.050	0.275	-0.073	0.422	0.329	0.301	0.450
23082	0.224	-0.373	0.501	0.234	-0.336	0.193	0.184	-0.625	0.185
23083	0.300	0.116	0.512	0.339	0.342	0.273	0.184	-0.625	0.508
23084	0.471	1.211	0.481	0.444	1.020	0.149	0.432	0.958	0.513
23085	0.497	1.380	0.185	0.518	1.491	0.273	0.500	1.397	0.224
23086	0.326	0.285	0.142	0.234	-0.336	0.309	0.142	-0.895	0.262
23087	0.050	-1.485	0.492	0.371	0.549	0.508	0.142	-0.895	0.050
23088	0.463	1.161	0.512	0.491	1.321	0.149	0.487	1.312	0.097
23089	0.476	1.245	0.185	0.418	0.850	0.273	0.505	1.430	0.501
23090	0.295	0.082	0.185	0.152	-0.863	0.514	0.184	-0.625	0.481
23091	0.263	-0.120	0.450	0.234	-0.336	0.501	0.297	0.099	0.492
23092	0.050	-1.485	0.481	0.342	0.361	0.491	0.142	-0.895	0.508
23093	0.050	-1.485	0.501	0.342	0.361	0.501	0.142	-0.895	0.492
23094	0.500	1.397	0.142	0.447	1.039	0.273	0.429	0.942	0.492
23095	0.479	1.262	0.296	0.465	1.152	0.309	0.476	1.245	0.097
23096	0.500	1.397	0.262	0.497	1.359	0.149	0.447	1.060	0.358
23097	0.500	1.397	0.492	0.497	1.359	0.514	0.447	1.060	0.185
23098	0.263	-0.120	0.512	0.234	-0.336	0.508	0.413	0.841	0.508
23099	0.429	0.942	0.224	0.447	1.039	0.398	0.511	1.464	0.224
23100	0.297	0.099	0.513	0.149	-0.882	0.462	0.142	-0.895	0.501
23101	0.508	1.447	0.050	0.494	1.340	0.371	0.466	1.178	0.512
23102	0.263	-0.120	0.050	0.193	-0.600	0.371	0.097	-1.181	0.501
23103	0.450	1.076	0.097	0.506	1.415	0.491	0.505	1.430	0.501
23104	0.097	-1.181	0.512	0.371	0.549	0.512	0.097	-1.181	0.224
23105	0.097	-1.181	0.481	0.374	0.568	0.512	0.050	-1.485	0.512
23106	0.511	1.464	0.501	0.459	1.114	0.512	0.489	1.329	0.262
23107	0.495	1.363	0.481	0.509	1.434	0.193	0.484	1.295	0.296
23108	0.495	1.363	0.512	0.509	1.434	0.193	0.474	1.228	0.142
23109	0.224	-0.373	0.513	0.275	-0.073	0.422	0.184	-0.625	0.467
23110	0.482	1.279	0.513	0.427	0.907	0.478	0.468	1.194	0.481
23111	0.224	-0.373	0.513	0.193	-0.600	0.478	0.142	-0.895	0.512
23112	0.224	-0.373	0.513	0.275	-0.073	0.371	0.184	-0.625	0.513
23113	0.184	-0.625	0.481	0.149	-0.882	0.102	0.329	0.301	0.481
23114	0.224	-0.373	0.481	0.272	-0.091	0.422	0.434	0.975	0.492
23115	0.261	-0.137	0.481	0.313	0.172	0.235	0.226	-0.356	0.467

23116	0.508	1.447	0.481	0.506	1.415	0.491	0.479	1.262	0.431
23117	0.263	−0.120	0.508	0.152	−0.863	0.444	0.355	0.470	0.512
23118	0.358	0.487	0.508	0.278	−0.054	0.478	0.263	−0.120	0.224
23119	0.503	1.413	0.508	0.518	1.491	0.501	0.476	1.245	0.501
23120	0.503	1.413	0.508	0.506	1.415	0.491	0.487	1.312	0.501
23121	0.500	1.397	0.512	0.497	1.359	0.273	0.476	1.245	0.224
23122	0.466	1.178	0.512	0.503	1.396	0.508	0.497	1.380	0.501
23123	0.184	−0.625	0.296	0.272	−0.091	0.491	0.261	−0.137	0.501
23124	0.142	−0.895	0.501	0.313	0.172	0.491	0.097	−1.181	0.262
23125	0.097	−1.181	0.097	0.149	−0.882	0.501	0.332	0.318	0.262
23126	0.261	−0.137	0.050	0.000	?	0.235	0.300	0.116	0.501
23127	0.500	1.397	0.050	0.512	1.453	0.371	0.482	1.279	0.492
23128	0.500	1.397	0.262	0.506	1.415	0.508	0.482	1.279	0.501
23129	0.453	1.093	0.185	0.398	0.718	0.514	0.503	1.413	0.492
23130	0.497	1.380	0.512	0.462	1.133	0.235	0.511	1.464	0.512
23131	0.513	1.481	0.358	0.374	0.568	0.462	0.503	1.413	0.450
23132	0.513	1.481	0.296	0.374	0.568	0.514	0.503	1.413	0.224
23133	0.503	1.413	0.431	0.494	1.340	0.422	0.500	1.397	0.224
23134	0.505	1.430	0.262	0.512	1.453	0.102	0.382	0.638	0.185
23135	0.505	1.430	0.508	0.512	1.453	0.102	0.382	0.638	0.385
23136	0.405	0.790	0.501	0.509	1.434	0.235	0.489	1.329	0.409
23137	0.097	−1.181	0.512	0.196	−0.581	0.371	0.358	0.487	0.296
23138	0.097	−1.181	0.262	0.196	−0.581	0.501	0.358	0.487	0.296
23139	0.505	1.430	0.409	0.421	0.869	0.462	0.505	1.430	0.431
23140	0.489	1.329	0.513	0.477	1.227	0.462	0.382	0.638	0.512
23141	0.432	0.958	0.296	0.482	1.265	0.398	0.463	1.161	0.481
23142	0.511	1.464	0.328	0.368	0.530	0.235	0.503	1.413	0.050
23143	0.187	−0.608	0.492	0.102	−1.184	0.491	0.329	0.301	0.328
23144	0.511	1.464	0.224	0.418	0.850	0.514	0.503	1.413	0.501
23145	0.050	−1.485	0.385	0.237	−0.317	0.491	0.295	0.082	0.501
23146	0.508	1.447	0.481	0.491	1.321	0.149	0.453	1.093	0.097
23147	0.508	1.447	0.467	0.444	1.020	0.341	0.384	0.655	0.050
23148	0.497	1.380	0.467	0.480	1.246	0.501	0.508	1.447	0.492
23149	0.508	1.447	0.431	0.494	1.340	0.341	0.426	0.925	0.508
23150	0.508	1.447	0.431	0.485	1.283	0.371	0.426	0.925	0.512
23151	0.187	−0.608	0.262	0.275	−0.073	0.235	0.358	0.487	0.185
23152	0.432	0.958	0.512	0.503	1.396	0.462	0.511	1.464	0.296
23153	0.411	0.824	0.328	0.488	1.302	0.478	0.511	1.464	0.185
23154	0.516	1.498	0.224	0.491	1.321	0.491	0.476	1.245	0.492
23155	0.489	1.329	0.224	0.506	1.415	0.053	0.500	1.397	0.431
23156	0.184	−0.625	0.296	0.234	−0.336	0.273	0.142	−0.895	0.185
23157	0.297	0.099	0.296	0.374	0.568	0.491	0.261	−0.137	0.481
23158	0.492	1.346	0.385	0.506	1.415	0.462	0.476	1.245	0.185
23159	0.511	1.464	0.481	0.518	1.491	0.273	0.482	1.279	0.224
23160	0.229	−0.339	0.296	0.234	−0.336	0.053	0.355	0.470	0.050
23161	0.500	1.397	0.296	0.468	1.170	0.053	0.508	1.447	0.508
23162	0.471	1.211	0.409	0.518	1.491	0.462	0.445	1.043	0.501
23163	0.508	1.447	0.409	0.421	0.869	0.491	0.508	1.447	0.492
23164	0.226	−0.356	0.224	0.102	−1.184	0.235	0.297	0.099	0.501
23165	0.187	−0.608	0.431	0.102	−1.184	0.508	0.263	−0.120	0.409
23166	0.297	0.099	0.501	0.234	−0.336	0.462	0.142	−0.895	0.385
23167	0.000	?	0.501	0.374	0.568	0.149	0.142	−0.895	0.097
23168	0.513	1.481	0.262	0.500	1.378	0.371	0.505	1.430	0.431
23169	0.450	1.076	0.296	0.465	1.152	0.508	0.495	1.363	0.467
23170	0.450	1.076	0.358	0.465	1.152	0.273	0.495	1.363	0.467
23171	0.513	1.481	0.501	0.398	0.718	0.398	0.495	1.363	0.467
23172	0.184	−0.625	0.385	0.237	−0.317	0.462	0.387	0.672	0.501
23173	0.450	1.076	0.097	0.488	1.302	0.501	0.511	1.464	0.492
23174	0.497	1.380	0.185	0.509	1.434	0.501	0.450	1.076	0.492
23175	0.466	1.178	0.296	0.488	1.302	0.341	0.511	1.464	0.492
23176	0.184	−0.625	0.142	0.152	−0.863	0.478	0.297	0.099	0.492

23177	0.142	-0.895	0.142	0.342	0.361	0.501	0.142	-0.895	0.492
23178	0.500	1.397	0.224	0.503	1.396	0.514	0.503	1.413	0.492
23179	0.142	-0.895	0.450	0.345	0.379	0.514	0.358	0.487	0.492
23180	0.142	-0.895	0.492	0.374	0.568	0.371	0.384	0.655	0.492
23181	0.263	-0.120	0.501	0.237	-0.317	0.514	0.226	-0.356	0.501
23182	0.505	1.430	0.508	0.462	1.133	0.193	0.474	1.228	0.508
23183	0.482	1.279	0.262	0.485	1.283	0.149	0.495	1.363	0.185
23184	0.511	1.464	0.296	0.488	1.302	0.341	0.476	1.245	0.450
23185	0.261	-0.137	0.508	0.053	-1.504	0.053	0.332	0.318	0.385
23186	0.224	-0.373	0.513	0.275	-0.073	0.508	0.097	-1.181	0.296
23187	0.508	1.447	0.513	0.494	1.340	0.193	0.408	0.807	0.224
23188	0.500	1.397	0.512	0.465	1.152	0.491	0.468	1.194	0.262
23189	0.224	-0.373	0.508	0.272	-0.091	0.508	0.050	-1.485	0.262
23190	0.295	0.082	0.501	0.053	-1.504	0.508	0.224	-0.373	0.512
23191	0.295	0.082	0.501	0.053	-1.504	0.273	0.224	-0.373	0.097
23192	0.516	1.498	0.296	0.456	1.095	0.309	0.489	1.329	0.185
23193	0.468	1.194	0.512	0.485	1.283	0.235	0.500	1.397	0.467
23194	0.261	-0.137	0.358	0.237	-0.317	0.508	0.184	-0.625	0.296
23195	0.429	0.942	0.467	0.503	1.396	0.512	0.500	1.397	0.501
23196	0.508	1.447	0.481	0.459	1.114	0.149	0.466	1.178	0.508
23197	0.358	0.487	0.508	0.149	-0.882	0.478	0.000	?	0.385
23198	0.495	1.363	0.262	0.377	0.587	0.501	0.426	0.925	0.185
23199	0.505	1.430	0.492	0.509	1.434	0.478	0.382	0.638	0.481
23200	0.226	-0.356	0.467	0.275	-0.073	0.235	0.142	-0.895	0.450
23201	0.495	1.363	0.385	0.404	0.756	0.508	0.434	0.975	0.358
23202	0.503	1.413	0.328	0.456	1.095	0.478	0.447	1.060	0.358
23203	0.453	1.093	0.492	0.503	1.396	0.235	0.447	1.060	0.501
23204	0.429	0.942	0.492	0.500	1.378	0.491	0.513	1.481	0.409
23205	0.361	0.504	0.492	0.342	0.361	0.501	0.224	-0.373	0.328
23206	0.511	1.464	0.492	0.482	1.265	0.309	0.471	1.211	0.501
23207	0.511	1.464	0.512	0.500	1.378	0.491	0.445	1.043	0.492
23208	0.508	1.447	0.512	0.512	1.453	0.422	0.447	1.060	0.501
23209	0.508	1.447	0.224	0.512	1.453	0.444	0.447	1.060	0.467
23210	0.226	-0.356	0.501	0.374	0.568	0.478	0.097	-1.181	0.467
23211	0.492	1.346	0.262	0.512	1.453	0.341	0.489	1.329	0.185
23212	0.226	-0.356	0.492	0.193	-0.600	0.193	0.261	-0.137	0.508
23213	0.261	-0.137	0.508	0.149	-0.882	0.491	0.261	-0.137	0.142
23214	0.224	-0.373	0.508	0.345	0.379	0.273	0.050	-1.485	0.185
23215	0.226	-0.356	0.296	0.053	-1.504	0.235	0.432	0.958	0.185
23216	0.508	1.447	0.512	0.512	1.453	0.491	0.513	1.481	0.185
23217	0.097	-1.181	0.385	0.193	-0.600	0.491	0.326	0.285	0.097
23218	0.455	1.110	0.097	0.491	1.321	0.491	0.495	1.363	0.512
23219	0.503	1.413	0.512	0.509	1.434	0.491	0.497	1.380	0.501
23220	0.463	1.161	0.512	0.506	1.415	0.512	0.511	1.464	0.185
23221	0.224	-0.373	0.385	0.275	-0.073	0.512	0.184	-0.625	0.296
23222	0.224	-0.373	0.262	0.310	0.153	0.512	0.050	-1.485	0.492
23223	0.224	-0.373	0.185	0.234	-0.336	0.512	0.263	-0.120	0.492
23224	0.468	1.194	0.508	0.506	1.415	0.422	0.503	1.413	0.262
23225	0.405	0.790	0.467	0.509	1.434	0.193	0.511	1.464	0.508
23226	0.261	-0.137	0.185	0.149	-0.882	0.422	0.261	-0.137	0.501
23227	0.479	1.262	0.262	0.474	1.208	0.235	0.489	1.329	0.508
23228	0.513	1.481	0.431	0.503	1.396	0.102	0.384	0.655	0.050
23229	0.476	1.245	0.296	0.480	1.246	0.235	0.505	1.430	0.328
23230	0.263	-0.120	0.185	0.237	-0.317	0.491	0.050	-1.485	0.224
23231	0.384	0.655	0.262	0.509	1.434	0.341	0.505	1.430	0.492
23232	0.482	1.279	0.501	0.474	1.208	0.398	0.508	1.447	0.142
23233	0.263	-0.120	0.512	0.237	-0.317	0.508	0.097	-1.181	0.185
23234	0.511	1.464	0.512	0.488	1.302	0.514	0.489	1.329	0.050
23235	0.489	1.329	0.185	0.500	1.378	0.508	0.447	1.060	0.296
23236	0.184	-0.625	0.142	0.307	0.135	0.193	0.142	-0.895	0.185
23237	0.466	1.178	0.142	0.491	1.321	0.501	0.476	1.245	0.185

23238	0.050	-1.485	0.492	0.427	0.907	0.501	0.224	-0.373	0.185
23239	0.050	-1.485	0.142	0.447	1.039	0.444	0.224	-0.373	0.481
23240	0.500	1.397	0.262	0.480	1.246	0.478	0.432	0.958	0.508
23241	0.000	?	0.262	0.342	0.361	0.508	0.184	-0.625	0.512
23242	0.187	-0.608	0.467	0.193	-0.600	0.422	0.184	-0.625	0.512
23243	0.484	1.295	0.296	0.491	1.321	0.478	0.511	1.464	0.224
23244	0.332	0.318	0.224	0.275	-0.073	0.149	0.224	-0.373	0.262
23245	0.300	0.116	0.492	0.234	-0.336	0.235	0.295	0.082	0.512
23246	0.497	1.380	0.501	0.494	1.340	0.462	0.513	1.481	0.501
23247	0.484	1.295	0.508	0.497	1.359	0.193	0.479	1.262	0.501
23248	0.511	1.464	0.262	0.485	1.283	0.371	0.474	1.228	0.512
23249	0.511	1.464	0.262	0.485	1.283	0.371	0.474	1.228	0.508
23250	0.445	1.043	0.492	0.512	1.453	0.273	0.511	1.464	0.492
23251	0.489	1.329	0.512	0.509	1.434	0.273	0.382	0.638	0.492
23252	0.489	1.329	0.467	0.509	1.434	0.309	0.382	0.638	0.185
23253	0.450	1.076	0.358	0.509	1.434	0.193	0.505	1.430	0.431
23254	0.505	1.430	0.508	0.424	0.888	0.309	0.497	1.380	0.185
23255	0.226	-0.356	0.097	0.193	-0.600	0.491	0.295	0.082	0.512
23256	0.495	1.363	0.185	0.418	0.850	0.491	0.476	1.245	0.450
23257	0.329	0.301	0.262	0.234	-0.336	0.193	0.142	-0.895	0.501
23258	0.261	-0.137	0.508	0.102	-1.184	0.149	0.187	-0.608	0.142
23259	0.187	-0.608	0.431	0.234	-0.336	0.273	0.142	-0.895	0.508
23260	0.508	1.447	0.431	0.488	1.302	0.193	0.500	1.397	0.501
23261	0.097	-1.181	0.142	0.345	0.379	0.491	0.142	-0.895	0.512
23262	0.184	-0.625	0.467	0.401	0.737	0.508	0.434	0.975	0.508
23263	0.503	1.413	0.467	0.503	1.396	0.508	0.500	1.397	0.512
23264	0.503	1.413	0.296	0.509	1.434	0.341	0.500	1.397	0.512
23265	0.503	1.413	0.467	0.503	1.396	0.193	0.500	1.397	0.512
23266	0.263	-0.120	0.508	0.196	-0.581	0.371	0.097	-1.181	0.512
23267	0.447	1.060	0.296	0.503	1.396	0.341	0.408	0.807	0.508
23268	0.447	1.060	0.501	0.503	1.396	0.508	0.408	0.807	0.508
23269	0.497	1.380	0.142	0.450	1.057	0.514	0.505	1.430	0.050
23270	0.497	1.380	0.501	0.482	1.265	0.491	0.516	1.498	0.481
23271	0.500	1.397	0.262	0.515	1.472	0.273	0.484	1.295	0.328
23272	0.453	1.093	0.501	0.418	0.850	0.398	0.447	1.060	0.467
23273	0.479	1.262	0.501	0.480	1.246	0.478	0.479	1.262	0.508
23274	0.142	-0.895	0.512	0.149	-0.882	0.478	0.263	-0.120	0.512
23275	0.142	-0.895	0.508	0.237	-0.317	0.102	0.184	-0.625	0.296
23276	0.463	1.161	0.224	0.465	1.152	0.273	0.508	1.447	0.481
23277	0.184	-0.625	0.224	0.193	-0.600	0.235	0.224	-0.373	0.508
23278	0.050	-1.485	0.492	0.371	0.549	0.508	0.100	-1.164	0.262
23279	0.050	-1.485	0.492	0.371	0.549	0.508	0.100	-1.164	0.431
23280	0.142	-0.895	0.492	0.275	-0.073	0.512	0.184	-0.625	0.508
23281	0.142	-0.895	0.513	0.278	-0.054	0.508	0.142	-0.895	0.185
23282	0.224	-0.373	0.508	0.310	0.153	0.422	0.387	0.672	0.262
23283	0.224	-0.373	0.492	0.196	-0.581	0.512	0.263	-0.120	0.262
23284	0.224	-0.373	0.512	0.310	0.153	0.235	0.387	0.672	0.481
23285	0.447	1.060	0.492	0.491	1.321	0.491	0.513	1.481	0.467
23286	0.450	1.076	0.185	0.491	1.321	0.508	0.492	1.346	0.328
23287	0.297	0.099	0.262	0.193	-0.600	0.149	0.050	-1.485	0.358
23288	0.184	-0.625	0.262	0.149	-0.882	0.193	0.226	-0.356	0.467
23289	0.384	0.655	0.508	0.272	-0.091	0.398	0.184	-0.625	0.358
23290	0.226	-0.356	0.512	0.193	-0.600	0.193	0.295	0.082	0.224
23291	0.503	1.413	0.296	0.491	1.321	0.508	0.492	1.346	0.358
23292	0.453	1.093	0.358	0.509	1.434	0.444	0.511	1.464	0.467
23293	0.432	0.958	0.262	0.509	1.434	0.501	0.508	1.447	0.467
23294	0.361	0.504	0.262	0.345	0.379	0.491	0.184	-0.625	0.467
23295	0.224	-0.373	0.185	0.193	-0.600	0.501	0.413	0.841	0.142
23296	0.411	0.824	0.224	0.374	0.568	0.501	0.142	-0.895	0.296
23297	0.050	-1.485	0.492	0.342	0.361	0.235	0.387	0.672	0.185
23298	0.500	1.397	0.467	0.509	1.434	0.273	0.326	0.285	0.431

23299	0.463	1.161	0.508	0.515	1.472	0.193	0.382	0.638	0.185
23300	0.508	1.447	0.296	0.488	1.302	0.491	0.508	1.447	0.050
23301	0.142	-0.895	0.224	0.272	-0.091	0.235	0.142	-0.895	0.431
23302	0.505	1.430	0.296	0.401	0.737	0.491	0.466	1.178	0.385
23303	0.508	1.447	0.185	0.474	1.208	0.508	0.513	1.481	0.050
23304	0.505	1.430	0.431	0.474	1.208	0.422	0.482	1.279	0.450
23305	0.297	0.099	0.142	0.102	-1.184	0.501	0.142	-0.895	0.501
23306	0.187	-0.608	0.142	0.272	-0.091	0.235	0.413	0.841	0.508
23307	0.297	0.099	0.296	0.237	-0.317	0.478	0.000	?	0.142
23308	0.497	1.380	0.142	0.515	1.472	0.341	0.447	1.060	0.142
23309	0.445	1.043	0.467	0.515	1.472	0.309	0.497	1.380	0.501
23310	0.482	1.279	0.142	0.509	1.434	0.273	0.474	1.228	0.481
23311	0.445	1.043	0.142	0.509	1.434	0.478	0.434	0.975	0.467
23312	0.489	1.329	0.358	0.421	0.869	0.501	0.468	1.194	0.185
23313	0.450	1.076	0.296	0.515	1.472	0.508	0.447	1.060	0.512
23314	0.263	-0.120	0.481	0.234	-0.336	0.478	0.187	-0.608	0.328
23315	0.466	1.178	0.328	0.491	1.321	0.491	0.500	1.397	0.296
23316	0.450	1.076	0.296	0.512	1.453	0.501	0.505	1.430	0.262
23317	0.184	-0.625	0.481	0.149	-0.882	0.514	0.229	-0.339	0.492
23318	0.184	-0.625	0.050	0.193	-0.600	0.478	0.266	-0.103	0.501
23319	0.300	0.116	0.224	0.395	0.700	0.508	0.050	-1.485	0.508
23320	0.300	0.116	0.358	0.193	-0.600	0.149	0.097	-1.181	0.262
23321	0.482	1.279	0.296	0.509	1.434	0.462	0.492	1.346	0.513
23322	0.476	1.245	0.513	0.450	1.057	0.462	0.476	1.245	0.513
23323	0.511	1.464	0.450	0.500	1.378	0.193	0.487	1.312	0.224
23324	0.479	1.262	0.450	0.491	1.321	0.478	0.503	1.413	0.513
23325	0.497	1.380	0.513	0.503	1.396	0.462	0.492	1.346	0.512
23326	0.497	1.380	0.224	0.503	1.396	0.309	0.492	1.346	0.050
23327	0.224	-0.373	0.224	0.234	-0.336	0.309	0.097	-1.181	0.512
23328	0.384	0.655	0.358	0.272	-0.091	0.508	0.358	0.487	0.328
23329	0.261	-0.137	0.450	0.196	-0.581	0.512	0.184	-0.625	0.512
23330	0.479	1.262	0.450	0.485	1.283	0.514	0.505	1.430	0.050
23331	0.261	-0.137	0.262	0.237	-0.317	0.508	0.224	-0.373	0.431
23332	0.508	1.447	0.450	0.491	1.321	0.398	0.503	1.413	0.467
23333	0.466	1.178	0.262	0.509	1.434	0.273	0.511	1.464	0.450
23334	0.500	1.397	0.358	0.424	0.888	0.193	0.500	1.397	0.513
23335	0.426	0.925	0.492	0.500	1.378	0.149	0.500	1.397	0.224
23336	0.142	-0.895	0.224	0.237	-0.317	0.341	0.224	-0.373	0.262
23337	0.505	1.430	0.097	0.477	1.227	0.478	0.508	1.447	0.431
23338	0.097	-1.181	0.142	0.339	0.342	0.444	0.261	-0.137	0.450
23339	0.050	-1.485	0.262	0.307	0.135	0.273	0.297	0.099	0.450
23340	0.226	-0.356	0.224	0.275	-0.073	0.309	0.142	-0.895	0.481
23341	0.508	1.447	0.450	0.477	1.227	0.512	0.463	1.161	0.512
23342	0.497	1.380	0.097	0.503	1.396	0.508	0.479	1.262	0.512
23343	0.447	1.060	0.409	0.512	1.453	0.514	0.513	1.481	0.450
23344	0.497	1.380	0.501	0.480	1.246	0.371	0.513	1.481	0.501
23345	0.466	1.178	0.512	0.491	1.321	0.149	0.474	1.228	0.224
23346	0.413	0.841	0.097	0.503	1.396	0.501	0.492	1.346	0.050
23347	0.511	1.464	0.492	0.515	1.472	0.422	0.466	1.178	0.328
23348	0.487	1.312	0.097	0.477	1.227	0.273	0.429	0.942	0.512
23349	0.500	1.397	0.501	0.509	1.434	0.309	0.508	1.447	0.508
23350	0.329	0.301	0.224	0.149	-0.882	0.508	0.224	-0.373	0.050
23351	0.463	1.161	0.492	0.462	1.133	0.501	0.500	1.397	0.050
23352	0.468	1.194	0.467	0.462	1.133	0.508	0.497	1.380	0.185
23353	0.187	-0.608	0.328	0.196	-0.581	0.309	0.224	-0.373	0.185
23354	0.484	1.295	0.296	0.480	1.246	0.309	0.503	1.413	0.142
23355	0.447	1.060	0.501	0.465	1.152	0.149	0.495	1.363	0.185
23356	0.187	-0.608	0.262	0.307	0.135	0.514	0.263	-0.120	0.501
23357	0.187	-0.608	0.224	0.307	0.135	0.508	0.263	-0.120	0.501
23358	0.429	0.942	0.296	0.506	1.415	0.235	0.495	1.363	0.450
23359	0.466	1.178	0.296	0.512	1.453	0.512	0.487	1.312	0.385

23360	0.466	1.178	0.450	0.512	1.453	0.341	0.497	1.380	0.328
23361	0.505	1.430	0.513	0.509	1.434	0.273	0.489	1.329	0.262
23362	0.513	1.481	0.050	0.398	0.718	0.371	0.503	1.413	0.492
23363	0.000	?	0.508	0.275	−0.073	0.512	0.450	1.076	0.262
23364	0.184	−0.625	0.296	0.193	−0.600	0.462	0.224	−0.373	0.296
23365	0.184	−0.625	0.481	0.149	−0.882	0.508	0.224	−0.373	0.097
23366	0.224	−0.373	0.513	0.339	0.342	0.478	0.184	−0.625	0.358
23367	0.384	0.655	0.512	0.482	1.265	0.102	0.387	0.672	0.492
23368	0.505	1.430	0.508	0.442	1.001	0.102	0.411	0.824	0.185
23369	0.226	−0.356	0.508	0.275	−0.073	0.398	0.297	0.099	0.185
23370	0.050	−1.485	0.431	0.313	0.172	0.501	0.295	0.082	0.185
23371	0.492	1.346	0.409	0.512	1.453	0.512	0.432	0.958	0.224
23372	0.503	1.413	0.262	0.509	1.434	0.512	0.511	1.464	0.501
23373	0.482	1.279	0.262	0.518	1.491	0.512	0.482	1.279	0.224
23374	0.187	−0.608	0.481	0.371	0.549	0.398	0.050	−1.485	0.142
23375	0.142	−0.895	0.481	0.149	−0.882	0.478	0.326	0.285	0.450
23376	0.489	1.329	0.481	0.503	1.396	0.478	0.503	1.413	0.358
23377	0.505	1.430	0.481	0.421	0.869	0.491	0.500	1.397	0.097
23378	0.187	−0.608	0.097	0.275	−0.073	0.478	0.097	−1.181	0.097
23379	0.187	−0.608	0.501	0.310	0.153	0.501	0.050	−1.485	0.296
23380	0.445	1.043	0.508	0.509	1.434	0.501	0.495	1.363	0.450
23381	0.479	1.262	0.492	0.503	1.396	0.491	0.508	1.447	0.385
23382	0.500	1.397	0.508	0.509	1.434	0.512	0.511	1.464	0.512
23383	0.142	−0.895	0.050	0.313	0.172	0.478	0.184	−0.625	0.431
23384	0.297	0.099	0.508	0.307	0.135	0.478	0.300	0.116	0.224
23385	0.261	−0.137	0.508	0.152	−0.863	0.341	0.184	−0.625	0.328
23386	0.484	1.295	0.358	0.520	1.509	0.462	0.492	1.346	0.050
23387	0.513	1.481	0.185	0.512	1.453	0.149	0.429	0.942	0.481
23388	0.226	−0.356	0.097	0.234	−0.336	0.309	0.184	−0.625	0.481
23389	0.516	1.498	0.512	0.515	1.472	0.371	0.484	1.295	0.097
23390	0.050	−1.485	0.512	0.342	0.361	0.102	0.184	−0.625	0.508
23391	0.050	−1.485	0.142	0.275	−0.073	0.273	0.224	−0.373	0.431
23392	0.097	−1.181	0.481	0.371	0.549	0.501	0.050	−1.485	0.262
23393	0.511	1.464	0.467	0.512	1.453	0.309	0.505	1.430	0.492
23394	0.476	1.245	0.508	0.462	1.133	0.273	0.500	1.397	0.224
23395	0.497	1.380	0.467	0.506	1.415	0.491	0.489	1.329	0.224
23396	0.482	1.279	0.262	0.474	1.208	0.371	0.497	1.380	0.328
23397	0.000	?	0.328	0.102	−1.184	0.478	0.408	0.807	0.262
23398	0.000	?	0.185	0.102	−1.184	0.508	0.382	0.638	0.224
23399	0.000	?	0.142	0.401	0.737	0.514	0.097	−1.181	0.481
23400	0.500	1.397	0.097	0.503	1.396	0.149	0.432	0.958	0.431
23401	0.508	1.447	0.185	0.509	1.434	0.501	0.468	1.194	0.492
23402	0.508	1.447	0.185	0.509	1.434	0.512	0.468	1.194	0.481
23403	0.508	1.447	0.262	0.509	1.434	0.273	0.468	1.194	0.501
23404	0.513	1.481	0.508	0.404	0.756	0.273	0.503	1.413	0.328
23405	0.479	1.262	0.501	0.474	1.208	0.491	0.497	1.380	0.508
23406	0.479	1.262	0.262	0.474	1.208	0.309	0.497	1.380	0.328
23407	0.479	1.262	0.358	0.485	1.283	0.149	0.497	1.380	0.492
23408	0.479	1.262	0.224	0.474	1.208	0.371	0.497	1.380	0.328
23409	0.503	1.413	0.185	0.500	1.378	0.478	0.497	1.380	0.508
23410	0.503	1.413	0.492	0.500	1.378	0.491	0.497	1.380	0.508
23411	0.497	1.380	0.492	0.494	1.340	0.478	0.492	1.346	0.185
23412	0.497	1.380	0.224	0.518	1.491	0.478	0.492	1.346	0.142
23413	0.511	1.464	0.492	0.480	1.246	0.341	0.495	1.363	0.501
23414	0.511	1.464	0.512	0.480	1.246	0.462	0.500	1.397	0.142
23415	0.295	0.082	0.492	0.345	0.379	0.102	0.187	−0.608	0.142
23416	0.497	1.380	0.492	0.462	1.133	0.149	0.447	1.060	0.050
23417	0.097	−1.181	0.262	0.149	−0.882	0.149	0.382	0.638	0.224
23418	0.050	−1.485	0.296	0.307	0.135	0.235	0.300	0.116	0.492
23419	0.050	−1.485	0.512	0.374	0.568	0.149	0.224	−0.373	0.224
23420	0.263	−0.120	0.358	0.102	−1.184	0.501	0.261	−0.137	0.481

23421	0.184	-0.625	0.262	0.272	-0.091	0.491	0.261	-0.137	0.050
23422	0.508	1.447	0.431	0.500	1.378	0.053	0.508	1.447	0.467
23423	0.358	0.487	0.431	0.310	0.153	0.193	0.097	-1.181	0.508
23424	0.297	0.099	0.142	0.272	-0.091	0.491	0.184	-0.625	0.512
23425	0.432	0.958	0.185	0.497	1.359	0.491	0.468	1.194	0.467
23426	0.261	-0.137	0.328	0.371	0.549	0.235	0.297	0.099	0.450
23427	0.505	1.430	0.328	0.471	1.189	0.512	0.505	1.430	0.501
23428	0.492	1.346	0.512	0.509	1.434	0.501	0.508	1.447	0.501
23429	0.503	1.413	0.431	0.515	1.472	0.508	0.382	0.638	0.224
23430	0.261	-0.137	0.501	0.152	-0.863	0.273	0.184	-0.625	0.296
23431	0.408	0.807	0.501	0.497	1.359	0.273	0.476	1.245	0.513
23432	0.513	1.481	0.224	0.512	1.453	0.309	0.447	1.060	0.481
23433	0.297	0.099	0.224	0.272	-0.091	0.478	0.355	0.470	0.513
23434	0.329	0.301	0.431	0.272	-0.091	0.341	0.361	0.504	0.513
23435	0.492	1.346	0.450	0.491	1.321	0.273	0.497	1.380	0.224
23436	0.224	-0.373	0.097	0.307	0.135	0.309	0.411	0.824	0.450
23437	0.384	0.655	0.501	0.149	-0.882	0.193	0.329	0.301	0.385
23438	0.484	1.295	0.501	0.371	0.549	0.235	0.503	1.413	0.358
23439	0.471	1.211	0.501	0.480	1.246	0.235	0.492	1.346	0.450
23440	0.471	1.211	0.142	0.491	1.321	0.273	0.500	1.397	0.508
23441	0.432	0.958	0.508	0.477	1.227	0.501	0.466	1.178	0.097
23442	0.432	0.958	0.508	0.477	1.227	0.478	0.466	1.178	0.328
23443	0.263	-0.120	0.508	0.342	0.361	0.462	0.187	-0.608	0.409
23444	0.516	1.498	0.512	0.465	1.152	0.478	0.505	1.430	0.492
23445	0.329	0.301	0.481	0.102	-1.184	0.235	0.142	-0.895	0.508
23446	0.224	-0.373	0.142	0.152	-0.863	0.149	0.187	-0.608	0.224
23447	0.224	-0.373	0.328	0.152	-0.863	0.398	0.187	-0.608	0.224
23448	0.297	0.099	0.296	0.237	-0.317	0.508	0.187	-0.608	0.467
23449	0.297	0.099	0.142	0.149	-0.882	0.501	0.097	-1.181	0.481
23450	0.387	0.672	0.467	0.500	1.378	0.508	0.511	1.464	0.262
23451	0.482	1.279	0.450	0.491	1.321	0.512	0.505	1.430	0.512
23452	0.297	0.099	0.512	0.053	-1.504	0.444	0.187	-0.608	0.513
23453	0.300	0.116	0.508	0.193	-0.600	0.444	0.297	0.099	0.492
23454	0.411	0.824	0.508	0.494	1.340	0.273	0.495	1.363	0.501
23455	0.411	0.824	0.508	0.494	1.340	0.478	0.495	1.363	0.185
23456	0.226	-0.356	0.296	0.237	-0.317	0.309	0.261	-0.137	0.185
23457	0.432	0.958	0.262	0.506	1.415	0.491	0.508	1.447	0.050
23458	0.495	1.363	0.262	0.503	1.396	0.491	0.500	1.397	0.262
23459	0.503	1.413	0.185	0.509	1.434	0.462	0.505	1.430	0.262
23460	0.263	-0.120	0.508	0.275	-0.073	0.193	0.050	-1.485	0.050
23461	0.297	0.099	0.185	0.272	-0.091	0.462	0.329	0.301	0.185
23462	0.363	0.520	0.185	0.310	0.153	0.478	0.224	-0.373	0.050
23463	0.503	1.413	0.385	0.477	1.227	0.514	0.489	1.329	0.513
23464	0.384	0.655	0.481	0.342	0.361	0.508	0.142	-0.895	0.501
23465	0.097	-1.181	0.358	0.275	-0.073	0.508	0.184	-0.625	0.358
23466	0.187	-0.608	0.262	0.310	0.153	0.512	0.050	-1.485	0.358
23467	0.297	0.099	0.328	0.196	-0.581	0.514	0.297	0.099	0.262
23468	0.142	-0.895	0.450	0.237	-0.317	0.514	0.184	-0.625	0.262
23469	0.142	-0.895	0.467	0.237	-0.317	0.444	0.184	-0.625	0.481
23470	0.226	-0.356	0.262	0.275	-0.073	0.501	0.184	-0.625	0.501
23471	0.447	1.060	0.512	0.503	1.396	0.273	0.482	1.279	0.328
23472	0.484	1.295	0.142	0.477	1.227	0.462	0.508	1.447	0.492
23473	0.495	1.363	0.508	0.459	1.114	0.273	0.513	1.481	0.328
23474	0.505	1.430	0.296	0.477	1.227	0.508	0.505	1.430	0.481
23475	0.263	-0.120	0.185	0.234	-0.336	0.514	0.226	-0.356	0.481
23476	0.295	0.082	0.450	0.149	-0.882	0.508	0.266	-0.103	0.224
23477	0.500	1.397	0.467	0.398	0.718	0.309	0.508	1.447	0.097
23478	0.508	1.447	0.481	0.512	1.453	0.491	0.495	1.363	0.512
23479	0.508	1.447	0.224	0.503	1.396	0.422	0.495	1.363	0.358
23480	0.508	1.447	0.185	0.506	1.415	0.235	0.508	1.447	0.296
23481	0.505	1.430	0.512	0.518	1.491	0.491	0.511	1.464	0.513

23482	0.492	1.346	0.512	0.444	1.020	0.512	0.492	1.346	0.508
23483	0.492	1.346	0.501	0.444	1.020	0.309	0.492	1.346	0.358
23484	0.297	0.099	0.508	0.272	-0.091	0.102	0.187	-0.608	0.185
23485	0.511	1.464	0.508	0.477	1.227	0.501	0.429	0.942	0.050
23486	0.358	0.487	0.142	0.307	0.135	0.512	0.187	-0.608	0.050
23487	0.466	1.178	0.142	0.494	1.340	0.273	0.511	1.464	0.508
23488	0.479	1.262	0.501	0.488	1.302	0.149	0.450	1.076	0.501
23489	0.508	1.447	0.224	0.462	1.133	0.444	0.505	1.430	0.450
23490	0.261	-0.137	0.513	0.193	-0.600	0.235	0.145	-0.878	0.450
23491	0.492	1.346	0.097	0.459	1.114	0.235	0.500	1.397	0.142
23492	0.468	1.194	0.097	0.477	1.227	0.193	0.503	1.413	0.224
23493	0.384	0.655	0.501	0.512	1.453	0.508	0.513	1.481	0.296
23494	0.329	0.301	0.512	0.509	1.434	0.512	0.503	1.413	0.512
23495	0.489	1.329	0.050	0.509	1.434	0.512	0.511	1.464	0.512
23496	0.489	1.329	0.501	0.509	1.434	0.273	0.511	1.464	0.328
23497	0.489	1.329	0.328	0.512	1.453	0.235	0.511	1.464	0.508
23498	0.489	1.329	0.142	0.512	1.453	0.235	0.511	1.464	0.508
23499	0.505	1.430	0.508	0.444	1.020	0.444	0.511	1.464	0.467
23500	0.511	1.464	0.262	0.500	1.378	0.235	0.503	1.413	0.185
23501	0.224	-0.373	0.296	0.278	-0.054	0.273	0.050	-1.485	0.508
23502	0.500	1.397	0.185	0.462	1.133	0.273	0.484	1.295	0.508
23503	0.263	-0.120	0.467	0.275	-0.073	0.398	0.326	0.285	0.512
23504	0.489	1.329	0.328	0.506	1.415	0.341	0.463	1.161	0.508
23505	0.516	1.498	0.501	0.515	1.472	0.309	0.505	1.430	0.481
23506	0.516	1.498	0.185	0.506	1.415	0.514	0.511	1.464	0.224
23507	0.300	0.116	0.513	0.307	0.135	0.512	0.297	0.099	0.224
23508	0.511	1.464	0.097	0.488	1.302	0.512	0.479	1.262	0.296
23509	0.384	0.655	0.513	0.427	0.907	0.193	0.511	1.464	0.142
23510	0.097	-1.181	0.513	0.234	-0.336	0.149	0.261	-0.137	0.508
23511	0.513	1.481	0.481	0.491	1.321	0.501	0.432	0.958	0.501
23512	0.513	1.481	0.185	0.518	1.491	0.508	0.505	1.430	0.467
23513	0.387	0.672	0.185	0.307	0.135	0.491	0.184	-0.625	0.450
23514	0.263	-0.120	0.492	0.000	?	0.491	0.263	-0.120	0.450
23515	0.187	-0.608	0.142	0.102	-1.184	0.102	0.263	-0.120	0.501
23516	0.508	1.447	0.508	0.500	1.378	0.235	0.482	1.279	0.262
23517	0.466	1.178	0.262	0.515	1.472	0.053	0.471	1.211	0.328
23518	0.184	-0.625	0.262	0.275	-0.073	0.508	0.332	0.318	0.328
23519	0.266	-0.103	0.142	0.149	-0.882	0.508	0.358	0.487	0.385
23520	0.429	0.942	0.492	0.444	1.020	0.501	0.471	1.211	0.501
23521	0.297	0.099	0.185	0.237	-0.317	0.149	0.361	0.504	0.142
23522	0.184	-0.625	0.512	0.237	-0.317	0.514	0.224	-0.373	0.142
23523	0.266	-0.103	0.512	0.193	-0.600	0.512	0.358	0.487	0.481
23524	0.503	1.413	0.224	0.506	1.415	0.273	0.461	1.144	0.501
23525	0.513	1.481	0.358	0.509	1.434	0.491	0.461	1.144	0.409
23526	0.513	1.481	0.512	0.509	1.434	0.462	0.461	1.144	0.262
23527	0.184	-0.625	0.185	0.275	-0.073	0.193	0.142	-0.895	0.142
23528	0.145	-0.878	0.185	0.234	-0.336	0.508	0.295	0.082	0.508
23529	0.145	-0.878	0.481	0.237	-0.317	0.371	0.184	-0.625	0.501
23530	0.497	1.380	0.501	0.444	1.020	0.491	0.432	0.958	0.097
23531	0.145	-0.878	0.224	0.237	-0.317	0.371	0.184	-0.625	0.508
23532	0.263	-0.120	0.385	0.275	-0.073	0.514	0.050	-1.485	0.328
23533	0.263	-0.120	0.185	0.275	-0.073	0.444	0.000	?	0.467
23534	0.461	1.144	0.185	0.398	0.718	0.512	0.429	0.942	0.262
23535	0.295	0.082	0.185	0.342	0.361	0.462	0.384	0.655	0.409
23536	0.229	-0.339	0.185	0.307	0.135	0.149	0.050	-1.485	0.481
23537	0.489	1.329	0.492	0.515	1.472	0.102	0.450	1.076	0.501
23538	0.500	1.397	0.050	0.515	1.472	0.462	0.497	1.380	0.501
23539	0.505	1.430	0.492	0.512	1.453	0.508	0.503	1.413	0.142
23540	0.261	-0.137	0.262	0.196	-0.581	0.508	0.145	-0.878	0.185
23541	0.261	-0.137	0.185	0.152	-0.863	0.501	0.145	-0.878	0.501
23542	0.492	1.346	0.508	0.503	1.396	0.462	0.503	1.413	0.501

23543	0.511	1.464	0.508	0.506	1.415	0.512	0.476	1.245	0.385
23544	0.466	1.178	0.492	0.494	1.340	0.512	0.471	1.211	0.328
23545	0.355	0.470	0.185	0.000	?	0.508	0.187	-0.608	0.431
23546	0.508	1.447	0.481	0.488	1.302	0.193	0.511	1.464	0.296
23547	0.097	-1.181	0.296	0.102	-1.184	0.273	0.332	0.318	0.328
23548	0.187	-0.608	0.050	0.237	-0.317	0.309	0.297	0.099	0.385
23549	0.263	-0.120	0.050	0.053	-1.504	0.501	0.263	-0.120	0.296
23550	0.500	1.397	0.513	0.515	1.472	0.462	0.492	1.346	0.467
23551	0.429	0.942	0.296	0.512	1.453	0.193	0.500	1.397	0.262
23552	0.429	0.942	0.450	0.503	1.396	0.102	0.508	1.447	0.224
23553	0.145	-0.878	0.262	0.149	-0.882	0.273	0.261	-0.137	0.501
23554	0.463	1.161	0.262	0.512	1.453	0.273	0.508	1.447	0.501
23555	0.463	1.161	0.513	0.512	1.453	0.235	0.508	1.447	0.142
23556	0.297	0.099	0.481	0.275	-0.073	0.193	0.224	-0.373	0.501
23557	0.468	1.194	0.185	0.485	1.283	0.508	0.513	1.481	0.097
23558	0.505	1.430	0.501	0.468	1.170	0.514	0.511	1.464	0.358
23559	0.300	0.116	0.481	0.193	-0.600	0.491	0.050	-1.485	0.512
23560	0.500	1.397	0.385	0.509	1.434	0.235	0.511	1.464	0.050
23561	0.142	-0.895	0.224	0.371	0.549	0.193	0.332	0.318	0.050
23562	0.500	1.397	0.467	0.488	1.302	0.273	0.508	1.447	0.142
23563	0.261	-0.137	0.185	0.371	0.549	0.341	0.050	-1.485	0.512
23564	0.505	1.430	0.492	0.512	1.453	0.508	0.432	0.958	0.262
23565	0.503	1.413	0.492	0.447	1.039	0.193	0.466	1.178	0.501
23566	0.511	1.464	0.467	0.512	1.453	0.193	0.447	1.060	0.262
23567	0.508	1.447	0.185	0.465	1.152	0.273	0.511	1.464	0.431
23568	0.229	-0.339	0.142	0.149	-0.882	0.273	0.224	-0.373	0.262
23569	0.229	-0.339	0.358	0.102	-1.184	0.398	0.261	-0.137	0.481
23570	0.497	1.380	0.050	0.462	1.133	0.371	0.429	0.942	0.481
23571	0.489	1.329	0.492	0.509	1.434	0.371	0.453	1.093	0.467
23572	0.489	1.329	0.262	0.512	1.453	0.235	0.453	1.093	0.097
23573	0.511	1.464	0.224	0.500	1.378	0.235	0.484	1.295	0.385
23574	0.505	1.430	0.450	0.459	1.114	0.371	0.508	1.447	0.512
23575	0.492	1.346	0.185	0.515	1.472	0.422	0.513	1.481	0.185
23576	0.511	1.464	0.508	0.509	1.434	0.371	0.447	1.060	0.481
23577	0.497	1.380	0.508	0.512	1.453	0.512	0.500	1.397	0.409
23578	0.184	-0.625	0.142	0.193	-0.600	0.102	0.226	-0.356	0.409
23579	0.261	-0.137	0.296	0.275	-0.073	0.235	0.050	-1.485	0.385
23580	0.263	-0.120	0.185	0.310	0.153	0.371	0.329	0.301	0.508
23581	0.503	1.413	0.328	0.503	1.396	0.444	0.513	1.481	0.185
23582	0.511	1.464	0.328	0.465	1.152	0.491	0.503	1.413	0.492
23583	0.297	0.099	0.328	0.193	-0.600	0.491	0.050	-1.485	0.492
23584	0.358	0.487	0.492	0.102	-1.184	0.478	0.050	-1.485	0.262
23585	0.261	-0.137	0.328	0.272	-0.091	0.478	0.184	-0.625	0.328
23586	0.261	-0.137	0.328	0.272	-0.091	0.193	0.184	-0.625	0.467
23587	0.187	-0.608	0.467	0.237	-0.317	0.193	0.142	-0.895	0.262
23588	0.226	-0.356	0.097	0.193	-0.600	0.235	0.184	-0.625	0.224
23589	0.487	1.312	0.328	0.509	1.434	0.501	0.497	1.380	0.501
23590	0.468	1.194	0.296	0.515	1.472	0.501	0.505	1.430	0.409
23591	0.500	1.397	0.481	0.491	1.321	0.273	0.447	1.060	0.224
23592	0.297	0.099	0.431	0.234	-0.336	0.514	0.387	0.672	0.328
23593	0.224	-0.373	0.492	0.196	-0.581	0.371	0.332	0.318	0.185
23594	0.295	0.082	0.492	0.275	-0.073	0.341	0.261	-0.137	0.185
23595	0.187	-0.608	0.512	0.339	0.342	0.462	0.000	?	0.185
23596	0.429	0.942	0.467	0.509	1.434	0.341	0.492	1.346	0.185
23597	0.142	-0.895	0.467	0.199	-0.562	0.235	0.261	-0.137	0.481
23598	0.142	-0.895	0.512	0.193	-0.600	0.273	0.295	0.082	0.328
23599	0.297	0.099	0.224	0.272	-0.091	0.371	0.097	-1.181	0.467
23600	0.142	-0.895	0.185	0.278	-0.054	0.193	0.355	0.470	0.224
23601	0.474	1.228	0.262	0.398	0.718	0.444	0.492	1.346	0.224
23602	0.142	-0.895	0.450	0.374	0.568	0.149	0.187	-0.608	0.492
23603	0.142	-0.895	0.262	0.374	0.568	0.478	0.187	-0.608	0.492

23604	0.361	0.504	0.431	0.237	-0.317	0.501	0.184	-0.625	0.512
23605	0.297	0.099	0.431	0.237	-0.317	0.512	0.224	-0.373	0.142
23606	0.487	1.312	0.296	0.371	0.549	0.235	0.503	1.413	0.450
23607	0.326	0.285	0.050	0.427	0.907	0.478	0.224	-0.373	0.185
23608	0.295	0.082	0.050	0.377	0.587	0.149	0.142	-0.895	0.097
23609	0.479	1.262	0.492	0.515	1.472	0.444	0.445	1.043	0.185
23610	0.050	-1.485	0.450	0.102	-1.184	0.102	0.358	0.487	0.385
23611	0.224	-0.373	0.097	0.237	-0.317	0.478	0.097	-1.181	0.185
23612	0.355	0.470	0.450	0.374	0.568	0.478	0.097	-1.181	0.512
23613	0.295	0.082	0.450	0.442	1.001	0.341	0.297	0.099	0.224
23614	0.513	1.481	0.450	0.488	1.302	0.273	0.450	1.076	0.431
23615	0.450	1.076	0.296	0.494	1.340	0.501	0.384	0.655	0.492
23616	0.450	1.076	0.501	0.482	1.265	0.235	0.505	1.430	0.224
23617	0.516	1.498	0.512	0.480	1.246	0.235	0.429	0.942	0.431
23618	0.224	-0.373	0.512	0.193	-0.600	0.235	0.224	-0.373	0.501
23619	0.226	-0.356	0.385	0.193	-0.600	0.273	0.326	0.285	0.450
23620	0.361	0.504	0.296	0.234	-0.336	0.273	0.050	-1.485	0.142
23621	0.450	1.076	0.224	0.503	1.396	0.508	0.474	1.228	0.450
23622	0.450	1.076	0.262	0.503	1.396	0.371	0.474	1.228	0.142
23623	0.263	-0.120	0.262	0.272	-0.091	0.398	0.097	-1.181	0.508
23624	0.447	1.060	0.385	0.518	1.491	0.193	0.511	1.464	0.512
23625	0.261	-0.137	0.409	0.374	0.568	0.422	0.434	0.975	0.512
23626	0.361	0.504	0.224	0.342	0.361	0.462	0.261	-0.137	0.358
23627	0.487	1.312	0.224	0.459	1.114	0.501	0.492	1.346	0.224
23628	0.224	-0.373	0.224	0.345	0.379	0.478	0.224	-0.373	0.481
23629	0.097	-1.181	0.358	0.234	-0.336	0.478	0.224	-0.373	0.358
23630	0.142	-0.895	0.185	0.272	-0.091	0.514	0.326	0.285	0.481
23631	0.261	-0.137	0.431	0.371	0.549	0.514	0.266	-0.103	0.142
23632	0.226	-0.356	0.262	0.196	-0.581	0.273	0.224	-0.373	0.224
23633	0.453	1.093	0.513	0.439	0.982	0.309	0.482	1.279	0.512
23634	0.097	-1.181	0.481	0.152	-0.863	0.462	0.432	0.958	0.409
23635	0.411	0.824	0.481	0.480	1.246	0.514	0.492	1.346	0.501
23636	0.495	1.363	0.262	0.500	1.378	0.512	0.482	1.279	0.501
23637	0.505	1.430	0.508	0.512	1.453	0.501	0.503	1.413	0.296
23638	0.097	-1.181	0.481	0.234	-0.336	0.273	0.332	0.318	0.185
23639	0.484	1.295	0.508	0.482	1.265	0.491	0.505	1.430	0.262
23640	0.097	-1.181	0.467	0.149	-0.882	0.501	0.332	0.318	0.492
23641	0.497	1.380	0.513	0.439	0.982	0.273	0.495	1.363	0.492
23642	0.226	-0.356	0.513	0.102	-1.184	0.514	0.329	0.301	0.142
23643	0.492	1.346	0.513	0.477	1.227	0.491	0.503	1.413	0.481
23644	0.466	1.178	0.508	0.477	1.227	0.371	0.503	1.413	0.142
23645	0.326	0.285	0.467	0.342	0.361	0.341	0.184	-0.625	0.142
23646	0.295	0.082	0.492	0.275	-0.073	0.462	0.142	-0.895	0.492
23647	0.503	1.413	0.450	0.503	1.396	0.501	0.500	1.397	0.296
23648	0.261	-0.137	0.492	0.234	-0.336	0.478	0.145	-0.878	0.467
23649	0.224	-0.373	0.492	0.234	-0.336	0.341	0.145	-0.878	0.142
23650	0.297	0.099	0.185	0.237	-0.317	0.273	0.000	?	0.501
23651	0.297	0.099	0.142	0.275	-0.073	0.512	0.050	-1.485	0.508
23652	0.450	1.076	0.224	0.272	-0.091	0.512	0.229	-0.339	0.492
23653	0.511	1.464	0.508	0.506	1.415	0.193	0.487	1.312	0.409
23654	0.050	-1.485	0.142	0.374	0.568	0.501	0.224	-0.373	0.450
23655	0.497	1.380	0.328	0.401	0.737	0.514	0.484	1.295	0.508
23656	0.300	0.116	0.508	0.193	-0.600	0.508	0.050	-1.485	0.508
23657	0.479	1.262	0.492	0.421	0.869	0.501	0.463	1.161	0.409
23658	0.503	1.413	0.262	0.465	1.152	0.149	0.505	1.430	0.097
23659	0.500	1.397	0.328	0.503	1.396	0.149	0.513	1.481	0.142
23660	0.500	1.397	0.467	0.482	1.265	0.309	0.461	1.144	0.224
23661	0.500	1.397	0.492	0.482	1.265	0.235	0.445	1.043	0.467
23662	0.426	0.925	0.512	0.512	1.453	0.193	0.497	1.380	0.262
23663	0.405	0.790	0.501	0.512	1.453	0.371	0.497	1.380	0.467
23664	0.263	-0.120	0.224	0.272	-0.091	0.193	0.224	-0.373	0.450

23665	0.263	-0.120	0.358	0.307	0.135	0.273	0.295	0.082	0.262
23666	0.484	1.295	0.450	0.459	1.114	0.501	0.513	1.481	0.296
23667	0.476	1.245	0.224	0.515	1.472	0.508	0.476	1.245	0.296
23668	0.476	1.245	0.224	0.512	1.453	0.371	0.511	1.464	0.467
23669	0.476	1.245	0.262	0.500	1.378	0.273	0.511	1.464	0.508
23670	0.097	-1.181	0.513	0.275	-0.073	0.235	0.224	-0.373	0.262
23671	0.492	1.346	0.431	0.491	1.321	0.273	0.453	1.093	0.481
23672	0.497	1.380	0.409	0.500	1.378	0.478	0.384	0.655	0.501
23673	0.000	?	0.481	0.275	-0.073	0.508	0.355	0.470	0.501
23674	0.484	1.295	0.481	0.520	1.509	0.235	0.453	1.093	0.296
23675	0.503	1.413	0.431	0.488	1.302	0.508	0.508	1.447	0.492
23676	0.050	-1.485	0.262	0.368	0.530	0.491	0.097	-1.181	0.467
23677	0.000	?	0.481	0.345	0.379	0.462	0.326	0.285	0.385
23678	0.500	1.397	0.328	0.462	1.133	0.462	0.408	0.807	0.050
23679	0.505	1.430	0.328	0.503	1.396	0.273	0.495	1.363	0.224
23680	0.355	0.470	0.185	0.477	1.227	0.149	0.511	1.464	0.097
23681	0.184	-0.625	0.097	0.339	0.342	0.491	0.226	-0.356	0.185
23682	0.097	-1.181	0.185	0.272	-0.091	0.309	0.226	-0.356	0.185
23683	0.500	1.397	0.262	0.518	1.491	0.273	0.468	1.194	0.512
23684	0.500	1.397	0.481	0.515	1.472	0.512	0.482	1.279	0.512
23685	0.142	-0.895	0.467	0.196	-0.581	0.512	0.261	-0.137	0.296
23686	0.476	1.245	0.481	0.497	1.359	0.102	0.511	1.464	0.296
23687	0.463	1.161	0.481	0.512	1.453	0.193	0.508	1.447	0.050
23688	0.505	1.430	0.431	0.509	1.434	0.149	0.489	1.329	0.185
23689	0.466	1.178	0.431	0.494	1.340	0.149	0.497	1.380	0.185
23690	0.261	-0.137	0.481	0.149	-0.882	0.462	0.184	-0.625	0.431
23691	0.332	0.318	0.385	0.149	-0.882	0.462	0.184	-0.625	0.185
23692	0.184	-0.625	0.296	0.310	0.153	0.512	0.050	-1.485	0.513
23693	0.142	-0.895	0.385	0.237	-0.317	0.512	0.263	-0.120	0.481
23694	0.097	-1.181	0.409	0.196	-0.581	0.193	0.263	-0.120	0.409
23695	0.184	-0.625	0.385	0.368	0.530	0.371	0.050	-1.485	0.328
23696	0.184	-0.625	0.501	0.196	-0.581	0.235	0.184	-0.625	0.358
23697	0.261	-0.137	0.328	0.272	-0.091	0.501	0.050	-1.485	0.450
23698	0.508	1.447	0.492	0.494	1.340	0.491	0.511	1.464	0.328
23699	0.500	1.397	0.508	0.503	1.396	0.309	0.497	1.380	0.328
23700	0.261	-0.137	0.513	0.374	0.568	0.514	0.361	0.504	0.328
23701	0.358	0.487	0.481	0.272	-0.091	0.514	0.361	0.504	0.328
23702	0.224	-0.373	0.262	0.234	-0.336	0.514	0.263	-0.120	0.328
23703	0.187	-0.608	0.142	0.275	-0.073	0.235	0.263	-0.120	0.508
23704	0.492	1.346	0.467	0.480	1.246	0.491	0.476	1.245	0.492
23705	0.492	1.346	0.185	0.512	1.453	0.514	0.500	1.397	0.501
23706	0.224	-0.373	0.224	0.237	-0.317	0.512	0.326	0.285	0.501
23707	0.489	1.329	0.097	0.509	1.434	0.478	0.495	1.363	0.501
23708	0.513	1.481	0.185	0.491	1.321	0.501	0.329	0.301	0.501
23709	0.495	1.363	0.508	0.462	1.133	0.235	0.482	1.279	0.097
23710	0.495	1.363	0.508	0.462	1.133	0.309	0.482	1.279	0.185
23711	0.263	-0.120	0.358	0.275	-0.073	0.309	0.226	-0.356	0.185
23712	0.297	0.099	0.224	0.152	-0.863	0.398	0.097	-1.181	0.481
23713	0.508	1.447	0.501	0.488	1.302	0.444	0.513	1.481	0.358
23714	0.361	0.504	0.224	0.310	0.153	0.501	0.361	0.504	0.262
23715	0.261	-0.137	0.142	0.272	-0.091	0.462	0.297	0.099	0.450
23716	0.429	0.942	0.142	0.512	1.453	0.478	0.513	1.481	0.492
23717	0.429	0.942	0.450	0.512	1.453	0.478	0.505	1.430	0.185
23718	0.142	-0.895	0.501	0.102	-1.184	0.501	0.358	0.487	0.050
23719	0.187	-0.608	0.185	0.193	-0.600	0.341	0.184	-0.625	0.492
23720	0.329	0.301	0.492	0.149	-0.882	0.309	0.050	-1.485	0.508
23721	0.329	0.301	0.501	0.149	-0.882	0.309	0.050	-1.485	0.409
23722	0.513	1.481	0.262	0.468	1.170	0.309	0.505	1.430	0.481
23723	0.434	0.975	0.224	0.462	1.133	0.514	0.500	1.397	0.296
23724	0.500	1.397	0.185	0.512	1.453	0.273	0.447	1.060	0.142
23725	0.500	1.397	0.262	0.512	1.453	0.371	0.447	1.060	0.512

23726	0.224	-0.373	0.185	0.193	-0.600	0.462	0.142	-0.895	0.185
23727	0.229	-0.339	0.142	0.371	0.549	0.478	0.224	-0.373	0.185
23728	0.000	?	0.501	0.237	-0.317	0.235	0.297	0.099	0.097
23729	0.429	0.942	0.481	0.500	1.378	0.309	0.505	1.430	0.492
23730	0.453	1.093	0.358	0.488	1.302	0.053	0.503	1.413	0.508
23731	0.097	-1.181	0.481	0.307	0.135	0.478	0.332	0.318	0.512
23732	0.495	1.363	0.512	0.512	1.453	0.514	0.508	1.447	0.501
23733	0.495	1.363	0.512	0.512	1.453	0.235	0.508	1.447	0.501
23734	0.495	1.363	0.224	0.512	1.453	0.491	0.466	1.178	0.508
23735	0.142	-0.895	0.512	0.234	-0.336	0.309	0.187	-0.608	0.224
23736	0.503	1.413	0.467	0.491	1.321	0.501	0.505	1.430	0.492
23737	0.503	1.413	0.450	0.515	1.472	0.149	0.505	1.430	0.185
23738	0.503	1.413	0.185	0.515	1.472	0.444	0.508	1.447	0.050
23739	0.508	1.447	0.296	0.477	1.227	0.508	0.508	1.447	0.097
23740	0.479	1.262	0.142	0.500	1.378	0.462	0.476	1.245	0.224
23741	0.142	-0.895	0.508	0.234	-0.336	0.462	0.229	-0.339	0.142
23742	0.329	0.301	0.296	0.313	0.172	0.235	0.224	-0.373	0.142
23743	0.295	0.082	0.224	0.310	0.153	0.193	0.297	0.099	0.508
23744	0.142	-0.895	0.262	0.398	0.718	0.501	0.142	-0.895	0.431
23745	0.463	1.161	0.328	0.442	1.001	0.501	0.505	1.430	0.481
23746	0.447	1.060	0.328	0.497	1.359	0.508	0.497	1.380	0.481
23747	0.505	1.430	0.262	0.459	1.114	0.491	0.471	1.211	0.492
23748	0.503	1.413	0.296	0.477	1.227	0.512	0.447	1.060	0.467
23749	0.503	1.413	0.296	0.477	1.227	0.102	0.447	1.060	0.492
23750	0.505	1.430	0.508	0.497	1.359	0.193	0.500	1.397	0.097
23751	0.295	0.082	0.431	0.342	0.361	0.508	0.261	-0.137	0.097
23752	0.261	-0.137	0.142	0.310	0.153	0.444	0.326	0.285	0.185
23753	0.261	-0.137	0.328	0.310	0.153	0.422	0.326	0.285	0.512
23754	0.184	-0.625	0.185	0.313	0.172	0.341	0.384	0.655	0.513
23755	0.505	1.430	0.185	0.512	1.453	0.309	0.497	1.380	0.467
23756	0.184	-0.625	0.224	0.272	-0.091	0.444	0.142	-0.895	0.512
23757	0.184	-0.625	0.467	0.371	0.549	0.462	0.142	-0.895	0.512
23758	0.384	0.655	0.512	0.471	1.189	0.444	0.479	1.262	0.508
23759	0.482	1.279	0.492	0.477	1.227	0.491	0.503	1.413	0.492
23760	0.358	0.487	0.492	0.237	-0.317	0.273	0.411	0.824	0.492
23761	0.261	-0.137	0.224	0.307	0.135	0.235	0.261	-0.137	0.050
23762	0.329	0.301	0.481	0.053	-1.504	0.478	0.142	-0.895	0.224
23763	0.450	1.076	0.481	0.474	1.208	0.235	0.511	1.464	0.492
23764	0.463	1.161	0.492	0.509	1.434	0.273	0.503	1.413	0.142
23765	0.263	-0.120	0.513	0.237	-0.317	0.235	0.097	-1.181	0.142
23766	0.505	1.430	0.492	0.491	1.321	0.398	0.508	1.447	0.142
23767	0.142	-0.895	0.097	0.310	0.153	0.273	0.329	0.301	0.142
23768	0.508	1.447	0.467	0.500	1.378	0.478	0.461	1.144	0.450
23769	0.297	0.099	0.512	0.149	-0.882	0.398	0.261	-0.137	0.450
23770	0.187	-0.608	0.262	0.447	1.039	0.501	0.408	0.807	0.097
23771	0.450	1.076	0.512	0.509	1.434	0.273	0.484	1.295	0.050
23772	0.466	1.178	0.262	0.462	1.133	0.491	0.497	1.380	0.492
23773	0.479	1.262	0.513	0.462	1.133	0.491	0.497	1.380	0.358
23774	0.224	-0.373	0.512	0.237	-0.317	0.512	0.145	-0.878	0.296
23775	0.184	-0.625	0.467	0.193	-0.600	0.398	0.184	-0.625	0.296
23776	0.518	1.514	0.467	0.500	1.378	0.398	0.503	1.413	0.512
23777	0.518	1.514	0.467	0.500	1.378	0.478	0.503	1.413	0.481
23778	0.503	1.413	0.492	0.515	1.472	0.235	0.384	0.655	0.328
23779	0.508	1.447	0.385	0.497	1.359	0.462	0.329	0.301	0.508
23780	0.503	1.413	0.467	0.515	1.472	0.309	0.426	0.925	0.512
23781	0.142	-0.895	0.481	0.102	-1.184	0.309	0.297	0.099	0.142
23782	0.145	-0.878	0.431	0.193	-0.600	0.273	0.326	0.285	0.142
23783	0.500	1.397	0.224	0.512	1.453	0.508	0.384	0.655	0.142
23784	0.224	-0.373	0.513	0.447	1.039	0.193	0.297	0.099	0.385
23785	0.513	1.481	0.385	0.430	0.926	0.514	0.463	1.161	0.142
23786	0.097	-1.181	0.224	0.345	0.379	0.514	0.261	-0.137	0.142

23787	0.097	-1.181	0.501	0.310	0.153	0.149	0.224	-0.373	0.512
23788	0.500	1.397	0.296	0.447	1.039	0.149	0.497	1.380	0.450
23789	0.508	1.447	0.296	0.465	1.152	0.462	0.500	1.397	0.224
23790	0.050	-1.485	0.450	0.444	1.020	0.235	0.142	-0.895	0.467
23791	0.497	1.380	0.508	0.488	1.302	0.193	0.500	1.397	0.481
23792	0.332	0.318	0.512	0.275	-0.073	0.371	0.097	-1.181	0.467
23793	0.142	-0.895	0.501	0.237	-0.317	0.478	0.355	0.470	0.097
23794	0.500	1.397	0.142	0.477	1.227	0.371	0.511	1.464	0.431
23795	0.263	-0.120	0.481	0.237	-0.317	0.371	0.050	-1.485	0.508
23796	0.297	0.099	0.450	0.275	-0.073	0.309	0.050	-1.485	0.508
23797	0.184	-0.625	0.185	0.234	-0.336	0.309	0.142	-0.895	0.142
23798	0.463	1.161	0.431	0.398	0.718	0.309	0.505	1.430	0.385
23799	0.329	0.301	0.508	0.275	-0.073	0.309	0.263	-0.120	0.296
23800	0.500	1.397	0.409	0.477	1.227	0.512	0.503	1.413	0.450
23801	0.184	-0.625	0.185	0.398	0.718	0.193	0.263	-0.120	0.296
23802	0.511	1.464	0.296	0.500	1.378	0.512	0.432	0.958	0.142
23803	0.097	-1.181	0.328	0.272	-0.091	0.149	0.263	-0.120	0.296
23804	0.516	1.498	0.450	0.494	1.340	0.341	0.482	1.279	0.328
23805	0.516	1.498	0.512	0.494	1.340	0.501	0.482	1.279	0.328
23806	0.479	1.262	0.501	0.515	1.472	0.501	0.466	1.178	0.328
23807	0.184	-0.625	0.142	0.401	0.737	0.478	0.097	-1.181	0.296
23808	0.184	-0.625	0.296	0.401	0.737	0.235	0.384	0.655	0.185
23809	0.489	1.329	0.481	0.482	1.265	0.501	0.513	1.481	0.492
23810	0.142	-0.895	0.262	0.234	-0.336	0.193	0.187	-0.608	0.431
23811	0.503	1.413	0.481	0.459	1.114	0.371	0.479	1.262	0.358
23812	0.261	-0.137	0.224	0.310	0.153	0.309	0.411	0.824	0.224
23813	0.261	-0.137	0.097	0.310	0.153	0.514	0.411	0.824	0.262
23814	0.142	-0.895	0.508	0.275	-0.073	0.102	0.384	0.655	0.262
23815	0.484	1.295	0.492	0.509	1.434	0.501	0.505	1.430	0.142
23816	0.184	-0.625	0.508	0.193	-0.600	0.273	0.187	-0.608	0.481
23817	0.508	1.447	0.185	0.515	1.472	0.273	0.487	1.312	0.508
23818	0.508	1.447	0.512	0.515	1.472	0.501	0.487	1.312	0.492
23819	0.224	-0.373	0.224	0.152	-0.863	0.512	0.261	-0.137	0.431
23820	0.358	0.487	0.142	0.149	-0.882	0.235	0.332	0.318	0.328
23821	0.511	1.464	0.142	0.459	1.114	0.501	0.466	1.178	0.450
23822	0.187	-0.608	0.142	0.234	-0.336	0.508	0.263	-0.120	0.450
23823	0.187	-0.608	0.142	0.193	-0.600	0.508	0.226	-0.356	0.501
23824	0.482	1.279	0.501	0.368	0.530	0.235	0.505	1.430	0.450
23825	0.503	1.413	0.142	0.477	1.227	0.491	0.413	0.841	0.512
23826	0.224	-0.373	0.142	0.371	0.549	0.273	0.226	-0.356	0.185
23827	0.389	0.689	0.501	0.371	0.549	0.501	0.332	0.318	0.513
23828	0.184	-0.625	0.385	0.307	0.135	0.462	0.184	-0.625	0.512
23829	0.184	-0.625	0.262	0.307	0.135	0.341	0.184	-0.625	0.142
23830	0.184	-0.625	0.224	0.307	0.135	0.273	0.184	-0.625	0.467
23831	0.184	-0.625	0.513	0.307	0.135	0.341	0.184	-0.625	0.185
23832	0.487	1.312	0.142	0.515	1.472	0.514	0.479	1.262	0.492
23833	0.050	-1.485	0.224	0.196	-0.581	0.273	0.329	0.301	0.501
23834	0.495	1.363	0.467	0.506	1.415	0.478	0.471	1.211	0.508
23835	0.261	-0.137	0.481	0.152	-0.863	0.235	0.224	-0.373	0.508
23836	0.184	-0.625	0.501	0.339	0.342	0.273	0.224	-0.373	0.508
23837	0.505	1.430	0.467	0.497	1.359	0.478	0.497	1.380	0.501
23838	0.505	1.430	0.142	0.497	1.359	0.309	0.492	1.346	0.501
23839	0.489	1.329	0.409	0.477	1.227	0.478	0.511	1.464	0.501
23840	0.187	-0.608	0.296	0.237	-0.317	0.508	0.142	-0.895	0.450
23841	0.482	1.279	0.512	0.494	1.340	0.193	0.445	1.043	0.185
23842	0.297	0.099	0.224	0.196	-0.581	0.193	0.184	-0.625	0.296
23843	0.050	-1.485	0.450	0.374	0.568	0.193	0.097	-1.181	0.492
23844	0.050	-1.485	0.467	0.307	0.135	0.478	0.184	-0.625	0.467
23845	0.516	1.498	0.385	0.515	1.472	0.478	0.384	0.655	0.224
23846	0.295	0.082	0.358	0.102	-1.184	0.193	0.184	-0.625	0.385
23847	0.450	1.076	0.358	0.497	1.359	0.444	0.505	1.430	0.097

23848	0.261	-0.137	0.450	0.278	-0.054	0.193	0.000	?	0.097
23849	0.261	-0.137	0.450	0.278	-0.054	0.193	0.226	-0.356	0.512
23850	0.511	1.464	0.142	0.506	1.415	0.422	0.429	0.942	0.481
23851	0.482	1.279	0.501	0.509	1.434	0.149	0.487	1.312	0.467
23852	0.184	-0.625	0.481	0.234	-0.336	0.508	0.226	-0.356	0.492
23853	0.500	1.397	0.262	0.506	1.415	0.193	0.432	0.958	0.224
23854	0.479	1.262	0.262	0.515	1.472	0.478	0.505	1.430	0.492
23855	0.384	0.655	0.185	0.503	1.396	0.398	0.450	1.076	0.501
23856	0.224	-0.373	0.450	0.234	-0.336	0.422	0.142	-0.895	0.262
23857	0.461	1.144	0.512	0.491	1.321	0.512	0.453	1.093	0.508
23858	0.184	-0.625	0.185	0.275	-0.073	0.508	0.142	-0.895	0.450
23859	0.487	1.312	0.185	0.500	1.378	0.398	0.505	1.430	0.513
23860	0.492	1.346	0.185	0.459	1.114	0.398	0.505	1.430	0.142
23861	0.466	1.178	0.050	0.342	0.361	0.462	0.508	1.447	0.185
23862	0.184	-0.625	0.450	0.272	-0.091	0.273	0.361	0.504	0.358
23863	0.142	-0.895	0.481	0.342	0.361	0.309	0.229	-0.339	0.450
23864	0.358	0.487	0.467	0.509	1.434	0.149	0.482	1.279	0.467
23865	0.050	-1.485	0.508	0.275	-0.073	0.462	0.361	0.504	0.481
23866	0.500	1.397	0.492	0.480	1.246	0.193	0.482	1.279	0.142
23867	0.261	-0.137	0.224	0.237	-0.317	0.462	0.142	-0.895	0.142
23868	0.226	-0.356	0.296	0.272	-0.091	0.462	0.224	-0.373	0.512
23869	0.453	1.093	0.296	0.480	1.246	0.102	0.511	1.464	0.142
23870	0.184	-0.625	0.262	0.313	0.172	0.193	0.411	0.824	0.467
23871	0.511	1.464	0.501	0.477	1.227	0.193	0.500	1.397	0.097
23872	0.513	1.481	0.185	0.506	1.415	0.514	0.500	1.397	0.142
23873	0.142	-0.895	0.513	0.193	-0.600	0.514	0.297	0.099	0.513
23874	0.297	0.099	0.409	0.193	-0.600	0.309	0.184	-0.625	0.512
23875	0.184	-0.625	0.328	0.193	-0.600	0.508	0.261	-0.137	0.501
23876	0.326	0.285	0.224	0.474	1.208	0.514	0.489	1.329	0.050
23877	0.326	0.285	0.492	0.474	1.208	0.512	0.489	1.329	0.501
23878	0.329	0.301	0.358	0.196	-0.581	0.235	0.142	-0.895	0.142
23879	0.495	1.363	0.358	0.442	1.001	0.501	0.474	1.228	0.050
23880	0.329	0.301	0.492	0.196	-0.581	0.514	0.142	-0.895	0.050
23881	0.329	0.301	0.224	0.196	-0.581	0.508	0.142	-0.895	0.185
23882	0.471	1.211	0.450	0.424	0.888	0.273	0.484	1.295	0.185
23883	0.097	-1.181	0.328	0.149	-0.882	0.193	0.300	0.116	0.450
23884	0.329	0.301	0.262	0.509	1.434	0.273	0.468	1.194	0.185
23885	0.300	0.116	0.492	0.193	-0.600	0.273	0.142	-0.895	0.185
23886	0.482	1.279	0.467	0.477	1.227	0.273	0.503	1.413	0.492
23887	0.434	0.975	0.508	0.404	0.756	0.478	0.508	1.447	0.142
23888	0.489	1.329	0.481	0.421	0.869	0.478	0.489	1.329	0.142
23889	0.489	1.329	0.481	0.509	1.434	0.341	0.405	0.790	0.224
23890	0.511	1.464	0.262	0.506	1.415	0.341	0.445	1.043	0.467
23891	0.466	1.178	0.142	0.395	0.700	0.309	0.503	1.413	0.185
23892	0.466	1.178	0.501	0.395	0.700	0.341	0.503	1.413	0.142
23893	0.508	1.447	0.224	0.462	1.133	0.273	0.408	0.807	0.492
23894	0.226	-0.356	0.467	0.275	-0.073	0.478	0.097	-1.181	0.508
23895	0.184	-0.625	0.450	0.310	0.153	0.193	0.142	-0.895	0.512
23896	0.261	-0.137	0.185	0.149	-0.882	0.422	0.224	-0.373	0.508
23897	0.450	1.076	0.185	0.462	1.133	0.462	0.466	1.178	0.262
23898	0.263	-0.120	0.185	0.193	-0.600	0.462	0.261	-0.137	0.512
23899	0.426	0.925	0.185	0.465	1.152	0.235	0.463	1.161	0.050
23900	0.426	0.925	0.512	0.465	1.152	0.491	0.447	1.060	0.508
23901	0.295	0.082	0.508	0.102	-1.184	0.514	0.261	-0.137	0.097
23902	0.050	-1.485	0.262	0.196	-0.581	0.508	0.297	0.099	0.501
23903	0.050	-1.485	0.224	0.196	-0.581	0.512	0.297	0.099	0.501
23904	0.489	1.329	0.262	0.509	1.434	0.491	0.466	1.178	0.050
23905	0.450	1.076	0.262	0.515	1.472	0.491	0.511	1.464	0.512
23906	0.097	-1.181	0.224	0.310	0.153	0.491	0.263	-0.120	0.512
23907	0.453	1.093	0.512	0.509	1.434	0.444	0.479	1.262	0.512
23908	0.450	1.076	0.050	0.518	1.491	0.514	0.500	1.397	0.492

23909	0.450	1.076	0.224	0.518	1.491	0.508	0.500	1.397	0.097
23910	0.300	0.116	0.467	0.234	-0.336	0.512	0.295	0.082	0.508
23911	0.503	1.413	0.467	0.500	1.378	0.508	0.492	1.346	0.492
23912	0.513	1.481	0.224	0.515	1.472	0.508	0.466	1.178	0.185
23913	0.511	1.464	0.262	0.506	1.415	0.273	0.389	0.689	0.185
23914	0.387	0.672	0.262	0.275	-0.073	0.341	0.050	-1.485	0.050
23915	0.300	0.116	0.512	0.193	-0.600	0.235	0.224	-0.373	0.492
23916	0.224	-0.373	0.467	0.275	-0.073	0.514	0.097	-1.181	0.358
23917	0.261	-0.137	0.262	0.275	-0.073	0.235	0.184	-0.625	0.142
23918	0.261	-0.137	0.508	0.275	-0.073	0.149	0.184	-0.625	0.185
23919	0.382	0.638	0.450	0.474	1.208	0.491	0.513	1.481	0.142
23920	0.408	0.807	0.224	0.474	1.208	0.478	0.513	1.481	0.185
23921	0.224	-0.373	0.409	0.345	0.379	0.102	0.295	0.082	0.508
23922	0.224	-0.373	0.481	0.345	0.379	0.193	0.295	0.082	0.358
23923	0.224	-0.373	0.492	0.307	0.135	0.398	0.050	-1.485	0.467
23924	0.361	0.504	0.409	0.342	0.361	0.444	0.187	-0.608	0.385
23925	0.187	-0.608	0.328	0.275	-0.073	0.371	0.184	-0.625	0.492
23926	0.434	0.975	0.224	0.482	1.265	0.501	0.429	0.942	0.224
23927	0.263	-0.120	0.431	0.196	-0.581	0.193	0.184	-0.625	0.097
23928	0.511	1.464	0.508	0.421	0.869	0.102	0.511	1.464	0.050
23929	0.482	1.279	0.358	0.465	1.152	0.341	0.479	1.262	0.224
23930	0.482	1.279	0.358	0.459	1.114	0.273	0.405	0.790	0.512
23931	0.261	-0.137	0.508	0.234	-0.336	0.273	0.329	0.301	0.296
23932	0.500	1.397	0.224	0.491	1.321	0.235	0.355	0.470	0.450
23933	0.476	1.245	0.224	0.515	1.472	0.462	0.447	1.060	0.501
23934	0.505	1.430	0.224	0.512	1.453	0.478	0.326	0.285	0.296
23935	0.463	1.161	0.508	0.515	1.472	0.508	0.326	0.285	0.224
23936	0.508	1.447	0.185	0.494	1.340	0.398	0.326	0.285	0.492
23937	0.508	1.447	0.467	0.494	1.340	0.398	0.326	0.285	0.262
23938	0.508	1.447	0.501	0.494	1.340	0.491	0.326	0.285	0.185
23939	0.508	1.447	0.262	0.444	1.020	0.273	0.508	1.447	0.492
23940	0.463	1.161	0.492	0.509	1.434	0.512	0.492	1.346	0.262
23941	0.492	1.346	0.385	0.509	1.434	0.053	0.500	1.397	0.467
23942	0.447	1.060	0.450	0.509	1.434	0.235	0.500	1.397	0.224
23943	0.492	1.346	0.492	0.506	1.415	0.371	0.500	1.397	0.050
23944	0.492	1.346	0.185	0.506	1.415	0.235	0.500	1.397	0.501
23945	0.184	-0.625	0.185	0.272	-0.091	0.235	0.097	-1.181	0.492
23946	0.142	-0.895	0.142	0.345	0.379	0.235	0.184	-0.625	0.409
23947	0.226	-0.356	0.492	0.234	-0.336	0.478	0.187	-0.608	0.492
23948	0.508	1.447	0.224	0.515	1.472	0.508	0.484	1.295	0.501
23949	0.142	-0.895	0.185	0.234	-0.336	0.508	0.361	0.504	0.097
23950	0.329	0.301	0.185	0.152	-0.863	0.512	0.263	-0.120	0.262
23951	0.505	1.430	0.224	0.485	1.283	0.501	0.450	1.076	0.501
23952	0.489	1.329	0.508	0.471	1.189	0.422	0.492	1.346	0.050
23953	0.263	-0.120	0.142	0.102	-1.184	0.512	0.184	-0.625	0.467
23954	0.332	0.318	0.501	0.193	-0.600	0.193	0.050	-1.485	0.467
23955	0.466	1.178	0.501	0.401	0.737	0.309	0.492	1.346	0.142
23956	0.495	1.363	0.501	0.447	1.039	0.193	0.505	1.430	0.262
23957	0.511	1.464	0.224	0.374	0.568	0.444	0.408	0.807	0.262
23958	0.505	1.430	0.450	0.503	1.396	0.508	0.482	1.279	0.262
23959	0.224	-0.373	0.450	0.193	-0.600	0.491	0.295	0.082	0.512
23960	0.358	0.487	0.296	0.102	-1.184	0.501	0.142	-0.895	0.513
23961	0.455	1.110	0.262	0.342	0.361	0.478	0.511	1.464	0.097
23962	0.226	-0.356	0.142	0.272	-0.091	0.491	0.184	-0.625	0.142
23963	0.226	-0.356	0.142	0.272	-0.091	0.491	0.184	-0.625	0.097
23964	0.263	-0.120	0.512	0.234	-0.336	0.478	0.097	-1.181	0.097
23965	0.516	1.498	0.492	0.468	1.170	0.273	0.495	1.363	0.050
[truncated: 600,273 more chars]
